# Systematic analysis of hepatotoxicity: combining literature mining and AI language models

**DOI:** 10.3389/frai.2025.1561292

**Published:** 2025-07-21

**Authors:** Chris Bauer, Long Tran Duc Dang, Twan van den Beucken, Johannes Schuchhardt, Ralf Herwig

**Affiliations:** ^1^MicroDiscovery GmbH, Berlin, Germany; ^2^Department of Toxicogenomics, Maastricht University, Maastricht, Netherlands; ^3^Department of Computational Molecular Biology, Max Planck Institute for Molecular Genetics, Berlin, Germany

**Keywords:** toxicology, hepatotoxicity, text mining, artificial intelligence (AI), large language model (LLM)

## Abstract

**Background:**

The body of toxicological knowledge and literature is expanding at an accelerating pace. This rapid growth presents significant challenges for researchers, who must stay abreast with latest studies while also synthesizing the vast amount of published information.

**Goal:**

Our goal is to automatically identify potential hepatoxicants from over 50,000 compounds using the wealth of scientific publications and knowledge.

**Methods:**

We employ and compare three distinct methods for automatic information extraction from unstructured text: (1) text mining (2) word embeddings and (3) large language models. These approaches are combined to calculate a hepatotoxicity score for over 50,000 compounds. We assess the performance of the different methods with a use case on Drug-Induced Liver Injury (DILI).

**Results:**

We evaluated hepatotoxicity for over 50,000 compounds and calculated a hepatotoxicity score for each compound. Our results indicate that text mining is effective for this purpose, achieving an Area Under the Curve (AUC) of 0.8 in DILI validation. Large language models performed even better, with an AUC of 0.85, thanks to their ability to interpret the semantic context accurately. Combining these methods further improved performance, yielding an AUC of 0.87 in DILI validation. All findings are available for download to support further research on toxicity assessment.

**Conclusions:**

We demonstrated that automated text mining is able to successfully assess the toxicity of compounds. A text mining approach seems to be superior to word embeddings. However, the application of a large language model with prompt engineering showed the best performance.

## 1 Introduction

Drug toxicity refers to the adverse effects or harmful reactions that occur in the body as a result of exposure to a pharmaceutical drug or substance. While drugs are designed to elicit therapeutic effects to treat or manage various medical conditions, they may also have unintended and undesirable consequences that can be harmful to the patient. Toxicity can be rather systemic, involving widespread impact throughout the entire organism (e.g. cytotoxicity or genotoxicity) or localized to a specific organ or tissue in the body. (e.g. hepatotoxicity or cardiotoxicity).

There is a vast amount of literature published in the area of toxicology (e.g. searching pubmed for ‘hepatotoxicity OR cardiotoxicity OR cytotoxicity OR genotoxicity' results in almost 350k publications between 2003 and 2024 - status 2024-08). It is practically impossible for an individual to keep up with the vast amount of information available, even for a specific topic. Scientific literature, including research papers, articles, and conference proceedings, often present information in a narrative, textual format. Although these texts are human readable they are not per se machine-interpretable.

Different workflows and tools have been published trying to assess toxicity based on literature data. Limtox (Canada et al., 2017) tries to characterize adverse hepatobiliary effects induced by chemical compounds. AOP-helpFinder is a tool that helps in the construction of adverse outcome pathways (AOPs) by examining large collections of scientific literature based on concept tagging and integrative systems biology (Carvaillo et al., 2019; Jornod et al., 2022; Jaylet et al., 2023). In a recent investigation, we assessed the relation between a selected list of anti-cancer drugs and different tumor types based on the available literature (Bauer et al., 2021).

These workflows and tools require a transformation of text into machine-interpretable data. There are at least three conceptually different approaches to make unstructured text accessible to machine learning algorithms.

1. *Concept taggers* aim to recognize (bio)concepts (e.g. genes, compounds, diseases, etc.) in unstructured text. They are conceptually relatively easy and transparent. They have a comparably long history and were constantly enhanced over the last decades. Concept taggers are typically working locally considering only one word, sentence or paragraph. There is no real semantic text interpretation. Two widely used concept taggers are: TaggerOne (Leaman and Lu, 2016) which uses semi-Markov models to detect and normalize bioconcepts such as chemicals and diseases and GNormPlus (Wei et al., 2015) aiming to tag and normalize genes and proteins in scientific literature. The tool PubTator (Wei et al., 2019) integrates different taggers and provides bioconcept annotations in full text biomedical articles for genes/proteins, genetic variants, diseases, chemicals, species and cell lines. One of the most advanced tagger is BERN2 (Sung et al., 2022; Lee et al., 2020), employing a neural network with transformer architecture (which is also used for large language models) for concept tagging and normalization. To assess the relation between two entities (e.g. a compound and a toxicity type), further association statistics (e.g. based on a Fisher test) can be used.

2. *Word embeddings* are conceptually different and have been proposed a decade ago. They are designed to transform the text into machine interpretable numeric vectors. They are working also locally in a text assessing the proximity of words. They allow to directly assess the relation between words (e.g. a compound and a toxicity type) by comparing the word vectors. The two most common implementations are Global Vectors for Word Representation (Glove) (Pennington et al., 2014) and Word2Vec (Mikolov et al., 2013). Word2Vec uses a relatively simple classical two-layer neural network to assess the relation between co-occuring words. Although the neural network is a black box, the algorithm and the results are relatively transparent.

3. *Large Language Models (LLMs)* are transformer-based neural networks which semantically encode passages. They are relatively new and conceptually complicated and aim to build up a semantic text understanding. They are computationally highly demanding since they require a large pre-training, but can be employed in a wide range of tasks given their remarkable reasoning capabilities. Due to the complexity, the algorithm is a complete black box and the results are not transparent. A common task for LLMs is Q&A (Question and Answer—chat bot), but the use cases are very diverse, some of which are knowledge-intensive Q&A, constructive feedback, etc. Prompting techniques have shown to significantly improve accuracy on several Q&A benchmarks, even surpassing smaller expert models (Nori et al., 2023; Singhal et al., 2023), which depend on large fine-tuning datasets. The top performing LLMs have hundreds of billions of parameters, making them highly demanding for large scale analysis. While smaller models lack core knowledge, they can compete with larger models in terms of language skills in multi-turn dialogues (Bai et al., 2024; Chiang et al., 2024). LLMs can be augmented with relevant context for knowledge-intensive tasks, maintaining much faster inference speed compared to larger models.

In this manuscript, we aim to automatically assess hepatotoxicity of more than 50,000 drugs using biomedical publications and common knowledge as represented indirectly in the LLM. We apply three different text mining methods, firstly a supervised approach with text mining strategies, secondly an unsupervised approach based on the calculation of word embeddings and thirdly a semantic large language model.

To assess the toxicity of the compounds we used more than 16 million publications (with at least one compound or hepatotoxicity term) and computed hepatoxicity scores for each of the different approaches as well as a final consensus score for all compounds. All results are available for download in Supplementary Table 1. To validate and compare the approaches we used a drug list ranked by the risk for inducing liver injury (DILI) (Chen et al., 2016).

## 2 Materials and methods

An overview on the entire workflow is given in Supplementary Figure 1.

### 2.1 Selection of compounds

We included all MeSH terms (Medical Subject Headings) for compounds identified by PubTator's Named-Entity Recognition (NER) in the available literature (all MeSH terms identified by PubTator—see file “chemical2pubtatorcentral”). We removed very rare terms found in less than 5 publications. Finally, we included more than 50,000 MeSH terms.

### 2.2 Confidence scoring

If there is only limited information available for a compound, the output of the different mining approaches can be unreliable. We calculated a confidence score for each compound representing the automatic accessibility of the compound. The confidence score provides a measure how well a compound is known or described by the literature. The main motivation here is to detect and filter noisy and unreliable compounds in order to increase reliability of the results.

**Figure 1:**
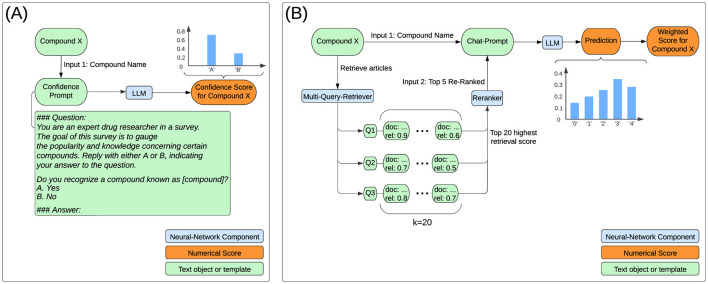
Flowchart for computing confidence and hepatotoxicity scores. **(A)** The confidence score for each compound represents the generation probability of the token ‘A' in response to the confidence prompt. **(B)** The LLM outputs a probability distribution across risk classes, which is used to calculate the toxicity score by applying predefined class weights: 0, 0.25, 0.5, 0.75, and 1 for classes 0–4, respectively. The retriever stores articles (documents) and their relevance scores as key-value pairs, combining scores for duplicate documents. Initially, 20 articles are retrieved per query (k = 20). These articles are reranked using a Cross-Encoder to enhance relevance to the specific task. Finally, the top 5 reranked abstracts are selected to generate the response.

For the confidence assessement we employ a large language model. We used 8-bit quantized Llama-3-8B-Instruct to compute confidence scores between 0 and 1, reflecting the LLMs knowledge on each compound. We generated a single token with the Q&A prompt shown in Figure 1A.

The log-probability of generating the single token ‘A' was converted to a standard probability and used as the confidence score.

### 2.3 Text mining

The text mining approach was based on PubTator (export in 2023-08).

We extracted all relevant publications for hepatotoxicity and all compounds using the provided NER files from PubTator (e.g. disease2pubtatorcentral and chemical2pubtatorcentral). The association of a publication with hepatotoxicity was done based on textual occurrences of any of the following terms in the PubTator data: “hepatotoxicity”, “hepatotoxic”, “liver toxicity”, or “hepatic toxicity”. The most frequent MESH terms annotated by PubTator are: “Chemical and Drug Induced Liver Injury” (MESH: D056486) in more than 95% of the publications; “Kidney Diseases” (MESH: D007674) in 1% of the publications and “Drug-Related Side Effects and Adverse Reactions” (MESH: D064420) in 0.2% of the publications. For the association between a compound and a publication we only used the MESH term of the compound. This resulted in more than 16 million publications. In order to calculate a toxicity score for one compound with respect to hepatotoxicity, we computed the co-occurrence by calculating an odds-ratio and an over-representation p-value using the hypergeometric distribution.

As an example, N-acetyl-4-benzoquinoneimine (MESH:C028473), a toxic metabolite of acetaminophen, was found in 1,651 publications. 1,232 of these 1,651 publications mentioned also hepatotoxicity (total number of hepatotoxicity related publications: 77,046). 419 publications for N-acetyl-4-benzoquinoneimine are other publications (total number of other publications: 16,440,105). This gives an odds-ratio of:


(1) $$\begin{array}{l} OR=\frac{1232/77046}{419/16440105}=627.4096 \end{array}$$

The -*log*_10_ *p*-value using the hypergeometric distribution for this example is 5699. A final toxicity score is calculated by multiplying -*log*_10_ *p*-value and the odds-ratio.

The score is normalized (divided by the highest value) in order to obtain a value between 0 and 1. The normalization is needed to better compare the three approaches.

### 2.4 Word embeddings

We trained a Word2Vec skip-gram neural network on the same 16 million PubMed abstracts as above with training parameters shown in Table 1. Specifically, the training script is based on gensim 4.3.2 and python 3.10.13. We only selected publications mentioning both a drug and a disease according to PubTator central annotations. Furthermore, we split the abstracts into 50 million sentences total, which were consequently preprocessed to remove noisy tokens and apply lower casing. To further improve the relevance of extracted information, sentences that mention only a single entity of type drug, disease or gene were removed. Since Word2Vec is based on words, it is necessary to consider synonyms and multi-word descriptions of compounds. As an example the compound: “Carbon Tetrachloride” would be treated as two word without a proper normalization. To this end, BERN2 was employed, an efficient biomedical text mining tool for multi concept recognition and normalization (Sung et al., 2022). For calculating the word embeddings the compounds were replaced by the corresponding MeSH terms.

**Table 1:** Hyperparameters for training Word2Vec skip-gram neural network.

**Epoch(s) trained**	**Number of negative samples**	**Minimum word count**	**Initial learning rate**	**Window size**	**Embedding dimension**
10	5	5	0.025	5	1000

In summary, we generated normalized word embeddings for about 50,000 compounds, 15,000 diseases and 35,000 genes. Since the neural network inherently learns from co-occurrence patterns, we measure cosine similarity as a form of association strength. We computed cosine similarity relationships across hepatotoxicity and the 50,000 compounds (for further investigating of the arithmetic properties we also used 14 other toxicity types (Tran et al., 2023)). Identical to correlation metrics, cosine similarity ranges from -1 to 1, where 0 conveys no association and positive values indicate positive association. However, given Word2Vec's design, negative similarity values shouldn't be interpreted as antonyms, but rather as unrelated.

### 2.5 Large language model

We employed the two pre-trained large language models Llama-3.1-8B-Instruct and Llama-3.1-70B-Instruct using the Fireworks API.^1^

The training of the models most probably included also openly available full text publications (Meta did not explicitely publish the sources).

For inferencing, we designed a chat-prompt comprising the following elements:

1. Instruction set defining the model behavior.
2. Multiple choice task with definitions for each risk category.
3. Context information for the example drug based on two-stage retrieval.
  a. Retrieve: Encode articles and multiple queries, then compute cosine similarities for article-query pairs to calculate cumulative relevance score.
  b. Rerank: Compute new relevance scores with cross-encoder model to rerank results.
4. Specific task to be performed with inputs for drug name and context placeholders.

**Figure 2:**
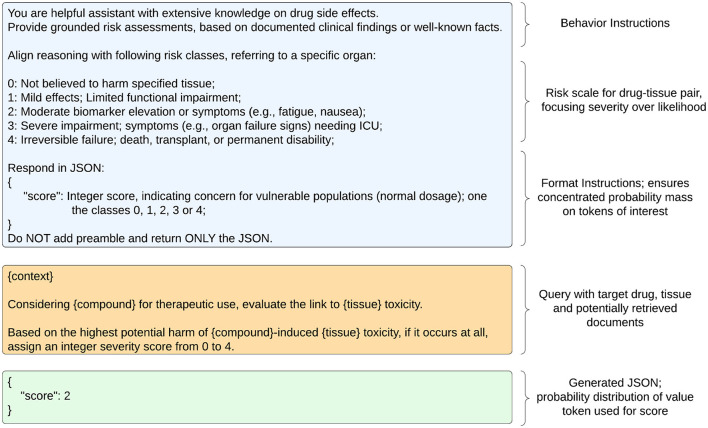
Chat-history and input prompt for LLM. **Left** side shows system (blue), user (orange) and assistant (green) messages. **Right** side describes employed strategies.

The user message of the chat-prompt instructs the LLM to generate a hepatotoxicity score, including probabilities for all multiple task options (Logprobs), by leveraging provided context. Figure 1B conveys the overall computation processes for a single compound. The multiple choice task is similar to the class descriptions from LiverTox, which groups compounds into five likelihood classes (from A to E) or associated cases into five severity classes (1-5) in terms of their potential to cause hepatotoxicity (Hoofnagle, 2013). LiverTox is an open-access website that provides up-to-date, unbiased and easily accessed information on the hepatotoxicity of drugs including clinical course, diagnosis, patterns, outcomes, severity scales, and causality grading systems for drug-induced liver injury. In our experiments, the LLM sees a risk scale from 0 to 4 that focuses on severity over likelihood with rather simple descriptions of harm. The prompt format of user-assistant interactions is shown in Figure 2.

#### 2.5.1 Context information

Providing the LLM with relevant contextual information, a technique known as Retrieval-Augmented Generation (RAG), has been shown to significantly enhance performance (Petroni et al., 2020). We used an advanced retrieval pipeline to include title and abstracts of the five most relevant publications retrieved for each prompt. The pipeline consists of two main stages which are processed at inference. Firstly, we encode all PubMed publications which mention both the compound and DILI in their full text by using bge-base-en-v1.5 to embed respective titles and abstracts. In total, roughly 230K publications were encoded this way. We selected 20 publications based on cosine similarity by combining the results for three retrieval questions:

Q1 Does [compound] cause liver damage?
Q2 Does [compound] induce hepatotoxicity?
Q3 Does [compound] have hepatic effects?

This retrieval stage results in 20 publications with the highest cumulative relevance scores.

Secondly, a cross-encoder, bge-reranker-base, re-ranked previous results with respect to a more specialized query: “*Does [compound] cause fatigue, jaundice, nausea or other liver toxicity symptoms?”* Each of the top five re-ranked publications was truncated at 100 words before incorporating into the chat-prompt for more consistent behavior.

#### 2.5.2 Calculation of the final toxicity score

The formatted task description invokes the generation of the hepatotoxicity score, i.e. the probabilities across all multiple choice options (Logprobs). Logprobs are the generation probabilities for each multiple choice label and can be used to quantify model confidence.

To derive a toxicity score, we compute the expected value, i.e. utilize a weighted sum approach, where each class weight is multiplied by the corresponding probability from the distribution of the generated choice token. Since we are using numerical labels, a mapping to predefined weights is not necessary. The scaling function applied to each risk label *k*, shown in Equation 2, simply normalizes the score to the range [0, 1].


(2) $$\begin{array}{l} f\left(k\right)=0.25\cdot k,\;k\in \left\{0,1,2,3,4\right\}. \end{array}$$

While this formulation reflects linearly scaled risk classes, the final score does not account for the LLM's inherent bias toward each option. The outcome represents a continuous toxicity score. E.g. the hepatotoxicity score for cysteine was computed as


(3) $$\begin{array}{l} 0\cdot 0+0.74\cdot 0.25+0.26\cdot 0.5+0\cdot 0.75+0\cdot 1\approx 0.315 \end{array}$$

where each choice label has generation probabilities


(4) $$\begin{array}{l} P\left(X=x\right)=\{\begin{array}{ll} 0.74 & \text{if }x=1,\\ 0.26 & \text{if }x=2,\\ 0 & \text{if }x\in \left\{0,3,4\right\}. \end{array} \end{array}$$

## 3 Results

### 3.1 Confidence score

For each compound we calculated a knowlegde based confidence score using an LLM as described in the methods section. The top results with the highest knowlegde confidence are acetaminophen, caffein and magnesium (see Table 2). Most of the high confidence compounds also show a high number of publications while the number of publications for compounds with low confidence scores are often limited. The Spearman correlation coefficient between the confidence score and the number of publications is 0.86. However, there are exceptions, e.g. the compound “trimethoprim, sulfadoxine drug combination” (MESH: C000301) has a high confidence score but a rather low number of publications. The confidence score reflects the LLM knowlegde but not necessarily the frequency of scientific publications.

**Table 2:** Table with the top 10 compounds with highest or lowest confidence score.

**MESH**	**Name**	**Confidence**	**N pub all**
D000082	Acetaminophen	1.00	80029.00
D002110	Caffeine	1.00	76229.00
D008274	Magnesium	1.00	186908.00
D011054	Poliovirus vaccine, inactivated	1.00	2112.00
D011055	Poliovirus vaccine, oral	1.00	3004.00
D014807	Vitamin D	1.00	119538.00
D022542	Measles-Mumps-Rubella Vaccine	1.00	1886.00
C000006	Insulin, neutral	0.99	267.00
C000179	N-acetylaspartate	0.99	11165.00
C000301	Trimethoprim, sulfadoxine drug combination	0.99	58.00
C588086	1,10,10-trimethyl-2-(3,4,5-trimethoxyphenyl)-1,2,2a,4,10,10a-hexahydro-3H-cyclobuta(4,5)pyrano(3,2-c)quinolin-3-one	0.05	7.00
C577626	24,25,26,27-tetranor-apotirucalla-(apoeupha)-1-senecioyloxy-3,7-dihydroxy-14,20,22-trien-21,23-epoxy	0.05	13.00
C514449	22-O-acetyl-21-O-(4'-O-angeloyl)-beta-d-fucopyranosyl theasapogenol B	0.05	8.00
C502982	9-(benzoyloxy)-2-(3-furanyl)dodecahydro-6a,10b-dimethyl-4,10-dioxo-2H-naphtho(2,1-c)pyran-7-carboxylic acid methyl ester	0.05	70.00
C068231	Trisphenylcarbamoylcellulose	0.05	15.00
C045331	Bromoacetylalprenololmenthane	0.05	72.00
C477956	3-O-rhamnopyranosyl-1-2-xylopyranosyl oleanolic acid 28-O-rhamnopyranosyl-1-4-glucopyranosyl-1-6-glucopyranosyl ester	0.04	15.00
C505284	1,1,2,2-tetrahydroheptadecafluorodecanol-3,3,4,4,5,5,6,6,7,7,8,8,9,9,10,10,10-heptadecafluoro-1-decanol	0.03	7.00
C486444	4-zido-5-isobutyrylamino-2,3-didehydro-2,3,4,5-tetradeoxyglycerogalacto-2-nonulopyranosic acid	0.03	26.00
C085636	GpenGRGDSPCA	0.03	7.00

Column “N Pub All” gives the number of publications found using the literature mining approach (in general without focus on hepatotoxicity).

For the primary analyses in the paper, we focus on the compounds with high confidence (confidence score ≥0.9).

### 3.2 Text mining

Using the pre-processed PubTator databases we assessed the relation between all compounds and the hepatotoxicity as described in the Methods section. The top hepatotoxic compounds include: silymarin (MESH: D012838), carbon tetrachloride (MESH: D002251), acetaminophen (MESH: D000082), thioacetamide (MESH: D013853), bilirubin (MESH: D001663) and malondialdehyde (MESH: D008315) (see Table 3). The compound ‘N-acetyl-4-benzoquinoneimine' (MESH: C028473) showing the highest hepatotoxicity score of 1 is not listed since the confidence score is only 0.79.

**Table 3:** Table with the top 15 compounds with the highest hepatotoxicity score for the literature mining (column “Score LM”).

**MESH**	**Name**	**N Pub Hepa**	**Odds ratio**	***P*-value (-log10)**	**Score LM**	**Confidence**
D012838	Silymarin	2153	78.25	6956.04	0.77	0.99
D002251	Carbon tetrachloride	7341	51.38	21289.53	0.77	0.99
D000082	Acetaminophen	11896	37.26	31458.93	0.75	1.00
D013853	Thioacetamide	1480	65.74	4558.98	0.74	0.97
D001663	Bilirubin	9275	22.55	20141.80	0.72	0.99
D008315	Malondialdehyde	6289	17.74	12167.67	0.71	0.98
D000111	Acetylcysteine	4389	18.78	8671.83	0.70	0.99
D011794	Quercetin	4352	18.69	8578.48	0.70	0.99
D005688	Galactosamine	1396	41.29	3731.41	0.70	0.96
C029684	Thiobarbituric acid	2392	27.50	5528.12	0.70	0.93
D005978	Glutathione	13705	14.80	24891.57	0.70	0.99
D001647	Bile acids and salts	5081	15.49	9170.80	0.69	0.99
D003474	Curcumin	4268	17.14	8074.41	0.69	0.99
D005609	Free radicals	6565	13.22	10964.70	0.69	0.99
D059808	Polyphenols	5198	13.81	8846.15	0.69	0.99

We include only compounds with confidence score ≥0.9.

### 3.3 Word embeddings

We computed cosine similarities between all compounds and hepatotoxicity. The top compounds with the highest score for hepatotoxicity (closest to the term “hepatotoxicity”) include carbon tetrachloride (MESH: D002251), galanin (MESH: D019004), thioacetamide (MESH: D013853), acetaminophen (MESH: D000082) and silymarin (MESH: D012838). Carbon tetrachloride, thioacetamide, acetaminophen and silymarin are also the top 4 hits of the literature mining results. See Table 4 for more results.

**Table 4:** Table with the top 15 compounds with the highest hepatotoxicity score (cosinde similarity) for the Word2Vec approach (column “Score W2V”).

**MESH**	**Name**	**Score W2V**	**Confidence**
D002251	Carbon tetrachloride	0.49	0.99
D019004	Galanin	0.42	0.98
D013853	Thioacetamide	0.42	0.97
C002614	Liv 52	0.41	0.97
C038266	Silybin dihemisuccinate	0.41	0.92
D000082	Acetaminophen	0.40	1.00
C034499	Allyl formate	0.40	0.94
C588038	Lonicerae flos	0.40	0.91
C056076	Acetaminophen, butalbital, caffeine drug combination	0.39	0.99
C493903	Cep290 protein, human	0.38	0.92
C461320	Shosaiko-to	0.38	0.93
C006463	Allyl alcohol	0.37	0.99
D011619	Psychotropic drugs	0.36	0.99
D012838	Silymarin	0.35	0.99
C043819	Somatostatin, cyclic hexapeptide(Phe-Phe-Trp-Lys-Thr-Phe)-	0.34	0.99

We include only compounds with confidence score ≥0.9.

**Figure 3:**
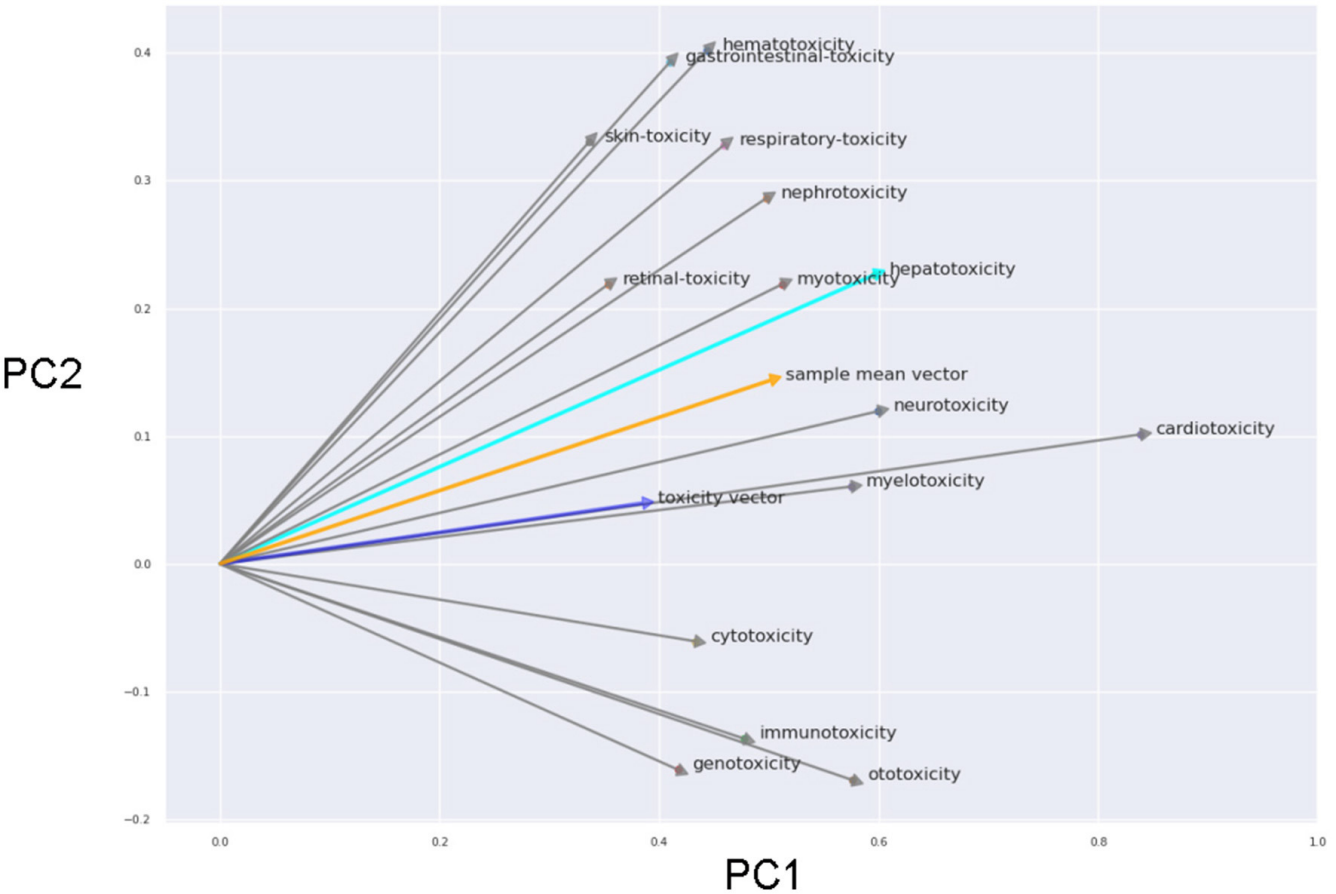
First and second principle components of the PCA (prinicpal component analysis) using the word embeddings including general word “toxicity”, all single toxicity types and the corresponding organs/tissues. The arrow connect the terms for the toxicity types with the corresponding organ/tissue and reflects the toxicity vector (in 2D projection). The blue arrow is the projected vector of the general “toxicity” term. The orange arrow is the mean vector of all black arrows.

To further emphasize the encoding's inherent arithmetic potential, we explored its capabilities through arithmetic operations. We performed a PCA of the word embeddings using general word ‘toxicity', single toxicity types (beside hepatotoxicity we also used 14 other toxicity types) and the corresponding organs/tissues (see Figure 3).

Remarkably, the vectors for the different toxicity types were rather similar even in the 2D projection. The averaged toxicity vector and the vector of the general toxicity term were highly simliar. This emphasizes the arithmetic properties of the word vectors (e.g. “heart” + “toxicity” ~ “cardiotoxicity”).

### 3.4 Large language model

Openly available pre-trained LLM models Llama-3-8B and Llama-3-70B were instructed to generate predictions for all compounds as described in the methods. Among the compounds with the highest toxicity scores are: nimesulide (MESH: C012655), bromfenac (MESH: C053083), agatoxins (MESH: D060848) and crizotinib (MESH: D000077547) with very high scores for both models. polonium-210 (MESH: C000615141) had a score of 1 from the LLM70B but only 0.79 for LLM8B. Table 5 shows most top scoring compounds.

**Table 5:** Table with the top compounds with the highest hepatotoxicity score derived from the top 10 of the two large language models (columns “Score LLM8B” and “Score LLM70B”).

**MESH**	**Name**	**Score LLM8B**	**Score LLM70B**	**Confidence**
C012655	Nimesulide	1.00	0.99	0.95
C053083	Bromfenac	1.00	1.00	0.94
C075975	Oil of chenopodium	0.99	0.96	0.95
C011007	Methyl ethyl ketone peroxide	0.99	1.00	0.91
D060848	Agatoxins	0.99	1.00	0.93
C093607	Microcystin LL	0.98	0.98	0.98
D000077547	Crizotinib	0.98	1.00	0.99
C023470	Perhexiline maleate	0.98	1.00	0.92
C033158	Sulfuric acid	0.98	0.85	0.99
D012966	Sodium Cyanide	0.98	0.85	0.98
C000615141	Polonium-210	0.79	1.00	0.94
C029227	Aflatoxin D1	0.96	1.00	0.91
C072611	Aflatoxin B1-DNA adduct	0.82	1.00	0.97
C522419	Catumaxomab	0.93	1.00	0.93
D000077867	Tolcapone	0.96	1.00	0.97
D000348	Aflatoxins	0.98	1.00	0.99

We include only compounds with confidence score ≥0.9.

### 3.5 Method comparison

**Figure 4:**
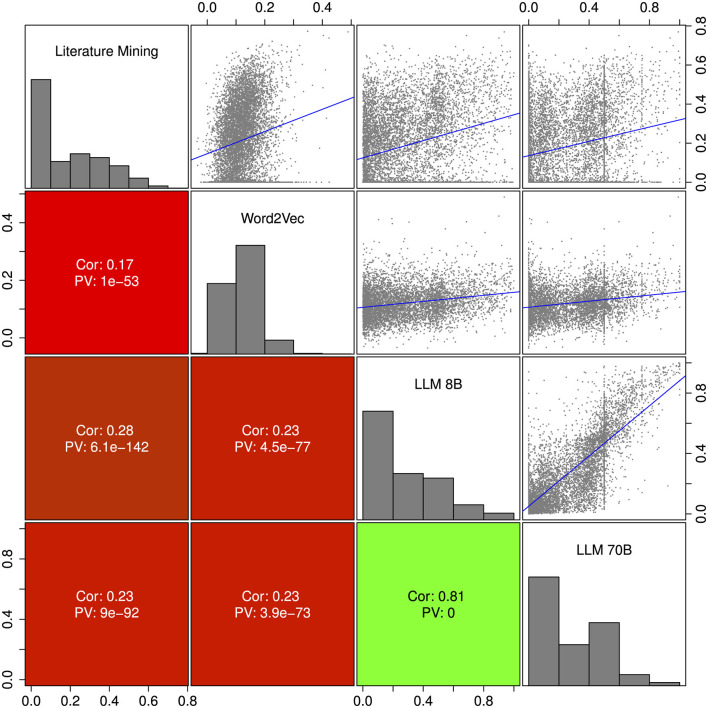
Pairwise corralation and scatter plot of the three different approaches: text mining, Word2Vec and large language model. **Upper** diagonal shows scatterplots including a linear regression line in blue. The vertical lines for the LLM 70B result from a very sharp distribution of the probability scores (see Methods section). The **lower** diagonal shows Pearson correlation coefficients and correlation *p*-values. On the diagnoal we show histograms of the scores. We include only compounds with confidence score ≥0.9.

The toxicity scores from the three different approaches, literature mining, Word2Vec and large language models, were compared by pairwise correlation and scatter plots in Figure 4. The average Spearman correlation coefficient ranged from 0.17 for the comparison of literature mining and Word2Vec to 0.28 for the comparison of literature mining versus LLM 8B. The two LLMs were similar with a Pearson correlation coefficient of 0.81. In general the correlations between the methods were relatively low which may suggest that the three methods were rather complementary.

**Figure 5:**
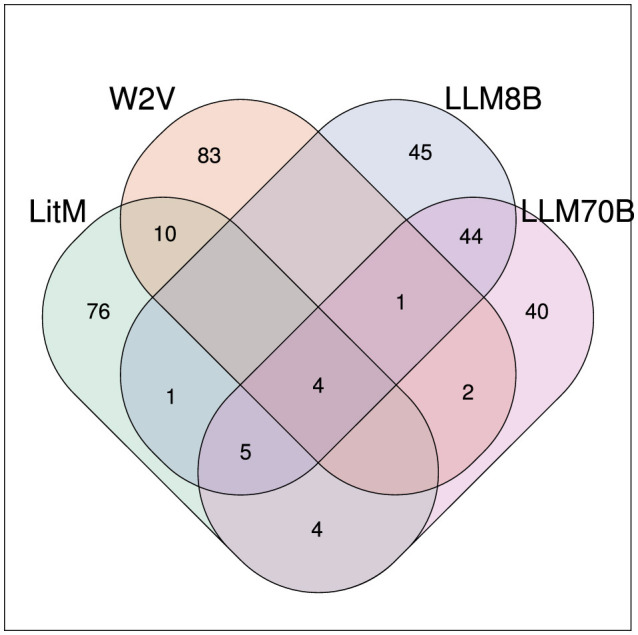
Venn diagram showing the overlap of the top 100 compounds (with the highest hepatotoxicity score) for the different methods.

Among the top 100 results with the highest hepatotoxicity score 4 compounds were found with all methods: carbon tetrachloride (MESH: D002251), galactosamine (MESH: D005688), isoniazid (MESH: D007538) and thioacetamide (MESH: D013853) (see Figure 5).

### 3.6 Hepatotoxicity combined score

We observed only a limited correlation between the different methods. The reason for this limited correlation may be that the three methods rather complement each other and include different aspects of toxicity. Thus, in order to calculate a final toxicity score we combined all three approaches using a weighted mean (weights: 2 for LLM8B, LLM70B and literature mining and only 1 for Word2Vec since the performance of Word2Vec seemed to be lower). The table with the 15 most hepatotoxic compounds is shown in Table 6. The full table is available for download in Supplementary Table 1.

**Table 6:** Table with the top results with the highest final hepatotoxicity score derived from the all methods.

**MESH**	**Name**	**Final**	**LM**	**W2V**	**LLM 8B**	**LLM 70B**	**Confidence**
D002251	Carbon tetrachloride	0.84	0.77	0.49	0.94	0.99	0.99
C029227	Aflatoxin D1	0.80	0.45		0.96	1.00	0.91
D013853	Thioacetamide	0.80	0.74	0.42	0.90	0.94	0.97
D000077288	Troglitazone	0.79	0.67	0.23	0.98	0.99	0.97
D005688	Galactosamine	0.78	0.70	0.32	0.92	0.96	0.96
D000348	Aflatoxins	0.78	0.64	0.19	0.98	1.00	0.99
D000077867	Tolcapone	0.76	0.61	0.22	0.96	1.00	0.97
C012655	Nimesulide	0.76	0.56	0.20	1.00	0.99	0.95
D007538	Isoniazid	0.75	0.67	0.28	0.96	0.87	0.99
C053083	Bromfenac	0.75	0.54	0.17	1.00	1.00	0.94
D000431	Ethanol	0.75	0.64	0.20	0.88	1.00	0.99
D052998	Microcystins	0.74	0.67	0.18	0.93	0.91	0.99
D004221	Disulfiram	0.74	0.54	0.27	0.98	0.94	0.99
D000438	Alcohols	0.74	0.63	0.13	0.93	0.97	0.99
D003606	Dacarbazine	0.74	0.51	0.21	0.96	1.00	0.98

We include only compounds with confidence score ≥0.9.

### 3.7 Validation

**Figure 6:**
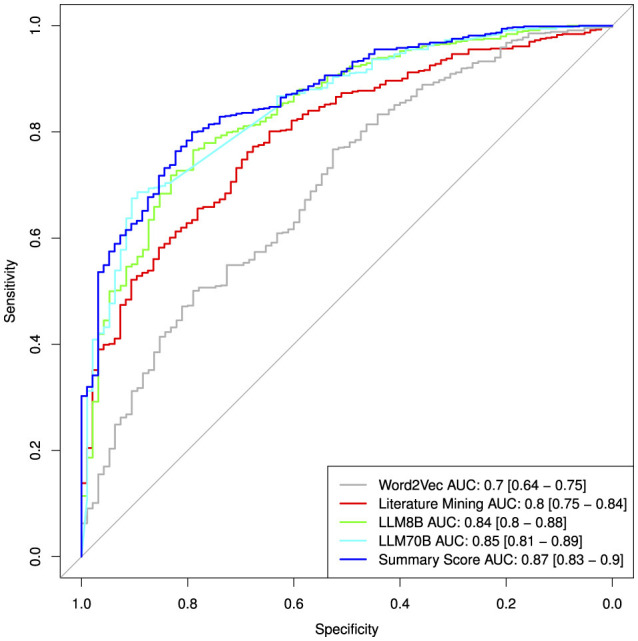
ROC curves for the prediction of DILI class comparing the different approaches.

To validate and compare the three different approaches for assessing hepatotoxicity, we used as ground truth a published drug list that ranks compounds for their risk to induce liver injury (DILI) (Chen et al., 2016). To this end we calculated single value ROC curves for the prediction of the highest DILI severity class (class 8) vs. the rest (see Figure 6). More detailed performance measures are displayed in Table 7. As before, we focussed on the compounds with high confidence values (confidence score ≥0.9). The Word2Vec approach showed the lowest performance with an AUC of 0.7 [CI: 0.64–0.75]. The literature mining approach was significantly better (Delong **p**-value: 0.00028) with an AUC of 0.8 [CI: 0.75–0.84]. The more complex 8B large language model was marginally better (Delong *p*-value: 0.057) with an AUC of 0.84 [CI: 0.8–0.88]. The best performance was obtained for the most complex model, the LLM 70B with an AUC of 0.85 [CI: 0.81–0.89] (Delong *p*-value compared to LLM8B: 0.5; Delong *p*-value compared to literature mining 0.044). The combined score showed an AUC of 0.87 [CI: 0.83–0.9] which is marginal better compared to the LLM70B (Delong *p*-value: 0.07) and significantly better than the literature mining (Delong *p*-value: 0.0008).

**Table 7:** Classification performances from single value ROC curves for the prediction of the highest DILI severity class (class 8) vs. the rest.

**Method**	**Threshold**	**tp**	**tn**	**fp**	**fn**	**ppv**	**npv**	**Sensitivity**	**Specificity**	**BACC**	**AUC**	**Lower CI AUC**	**Upper CI AUC**
Word2Vec	0.15	524	50	45	159	0.92	0.24	0.77	0.53	0.65	0.70	0.64	0.75
Literature mining	0.46	529	66	30	165	0.95	0.29	0.76	0.69	0.72	0.80	0.75	0.84
LLM8B	0.51	530	75	20	162	0.96	0.32	0.77	0.79	0.78	0.84	0.80	0.88
LLM70B	0.48	475	85	10	217	0.98	0.28	0.69	0.89	0.79	0.85	0.81	0.89
LLM70B no RAG	0.47	388	88	7	303	0.98	0.23	0.56	0.93	0.74	0.82	0.78	0.86
Summary score	0.44	555	76	20	139	0.97	0.35	0.80	0.79	0.80	0.87	0.83	0.90

### 3.8 Influence of the RAG component

**Figure 7:**
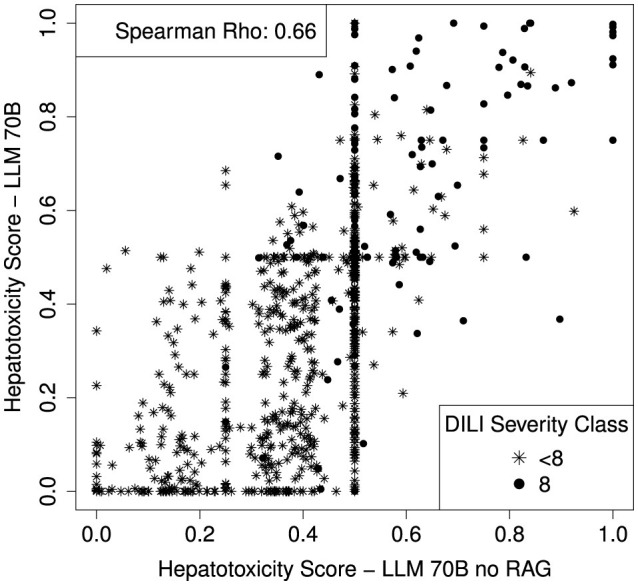
Scatterplot comparing the hepatotoxicity scores of the LLM with and without the RAG component (with and without additional context to the query). Spearman correlation coefficient is shown on top left side of the plot.

In order to investigate the benefit of the RAG component (additional context provided to the LLM query), we run the LLM70B without providing additional context. Without the RAG component, the LLM70B resulted in an AUC of 0.82 [CI: 0.78–0.86]. The RAG component does not increase the validation performance significantly (Delong *p*-value: 0.15) but the missing context leads to a high increase of indecisive compounds. 36.6% of the DILI compounds have a hepatotoxicity score of 0.5 when no additional context is provided (a score of 0.5 can be interpreted as if the LLM is indecisive). When providing additional context only 14.2% of the DILI compounds have a hepatotoxicity score of 0.5. A scatterplot comparing the hepatotoxicity scores with and without the additional context is shown in Figure 7.

## 4 Discussion

We performed a comprehensive assessment of hepatotoxicity with more than 50,000 compounds based on published biomedical literature and common knowledge. We calculated combined hepatotoxicity score for all compounds with four conceptually different methods which can be used as a repository for further analysis. The validation using DILI underlines the usefulness of the approaches.

Especially the combined hepatotoxicity score seems to be capable to predict DILI for compounds not included in the DILI set with high accuracy. The main results are available for download and further investigations.

For the association between a publication and hepatotoxicity (or a publication and a compound) we used NER provided by PubTator. The overall hepatotoxicity score (Supplementary Table 1) aligns with published studies or assessments of hepatotoxins, for example, with the US DILIN prospective study (Chalasani et al., 2015): here, 1257 patients were investigated longitudinally for DILI in the presence of drug usage. Resulting compounds identified in that study mostly affecting DILI in the patients were reflected by our DILI ranking, for example azithromycin (rank 251 in our combined score list, score = 0.61), nitrofurantoin (rank 76, 0.69), minocycline (rank 143, 0.65) among others. However, it should be emphasized that our associations are related to NER and disease terms, and there are many hepatic endpoints that might not be associated with a disease term. For example, the dataset in CAMDA 2022 (https://bipress.boku.ac.at/camda2022/) showed that it was complex and non-trivial to accurately identify DILI-(un)related publications. An interesting, more general approach here would be to incorporate the concept of *key characteristics* for the toxicity under study, allowing for further chemically and biologically motivated associations. In the context of DILI such key characteristics could be *Causes apoptosis/necrosis of liver cells* or *disrupts transport function* as have been recently demonstrated (Rusyn et al., 2021). Strong hepatotoxins reported in this study based on 12 key characteristics are also highly ranked in our assessment such as acetaminophen (rank 165, 0.64) or amoxicillin (2,554, 0.42), however, there are also discordant cases in particular with respect to chemicals or environmental contaminants which are not targeted by our approach.

Textmining based on PubTator performs much better than word embeddings based on the same set of publications (DeLong *p*-value for comparing ROC curves 0.00028). A reason might be that the text mining approach is superwised and uses only pre-defined information on compounds and toxicity types. In contrast, word embeddings are unsuperwised and contain more noise. However, for compounds with a very high association to hepatotoxicity, the correspondance between both methods increases strongly (14% of the top 100 of both methods are overlapping).

Large language models (both 8B and 70B) seem to be superior compared to literature mining. One reason for this is certaintly that literature mining is restricted to PubMed while LLMs have a broader set of input information. In addition, LLMs are capable of considering the semantic relation while the literature mining approach only uses co-occurrence. The missing directionality for literature mining results in higher scores also for hepatoprotective drugs. E.g. drugs like acetylcysteine (MESH: D000111) are hepatoprotective with DILI severity class = 0. Due to the missing directionality the hepatotoxicity score for acetylcysteine is high in the literature mining approach (hepatotoxicity scores: literature mining = 0.7; Word2Vec = 0.26; LLM8B = 0.14; LLM70B = 0). The same can also be true for the word embeddings. E.g. the compound shosaiko-to (MESH: C461320) is a chinese herbal supplement, believed to enhance liver health and is among the top 10 hepatotoxic compounds from Word2Vec (hepatotoxicity scores: literature mining = 0; Word2Vec = 0.38; LLM8B = 0.54; LLM70B = 0.5).

Interestingly the performance of the 70B LLM is comparable to the performance of the 8B LLM. The main reason for this is most probable the multi-step workflow. We first retrieve and re-rank drug related context information and provide this context to the LLM prompt. So the highly relevant context information is the same for the 8B LLM and the 70B LLM which may by the reason for the similar performance.

The performance of Word2Vec for the toxicity assessment is weaker compared to the other methods but the arithmetic properties are interesting and helpful. An experiment using word vectors showed that the word embedding neural network organizes the vectors in a way that allows for arithmetic operations. Interestingly, the direction vectors connecting the position vectors of toxicity type and body part highly correlates with the direction vector of the term “toxicity” (even in this 2D projection). An advantage of this arithmetic is, that it could also be applied to less common toxicities, e.g. to get the vector of “pancreatic toxicity”.

LLM selection significantly influences the classifier's performance. An absence of refined reasoning capabilities can hinder the effectiveness of prompting strategies. We noticed that our employed LLM is influenced by the provided context. Although the context does not incease the validation performance significantly (Delong *p*-value: 0.15) without the additional context the LLM seem to be more indecisive. Therefore we highly believe that the RAG component is well suited to increase the power of the predictions. However, the provided context may distract the LLM from the acutal task. For some compounds we saw a high mismatch between the DILI rank and the prediction. E.g. balsalazide (MESH: C038637) with the highest DILI severity score and a LLM confidence score of 0.93 has a toxicity score of 0.15 with the LLM70B (so it should be well known and highly toxic). None of the publications in the context is mentioning a direct relation between the compound and hepatotoxicity. For a more comprehensive assessment a PDF file is available in the supplement showing context for querying the DILI compounds (Supplementary material 1).

Opting for a larger model enables better reasoning and evaluation of the context but comes with more demanding system prerequisites. If the system's capacity is large enough, such a choice can be advantageous, given that only a single token is generated per inference.

The purpose of our study was to compare different approaches for automated information retrieval and its application to characterize hepatotoxins at a general level. In particular, LLMs performed well and might have the potential to be utilized for more complex tasks such as risk or hazard assessment (Ma and Wolfinger, 2024). However, this task would require gathering domain-specific texts, including scientific literature, regulatory documents, safety data sheets and further chemical databases to fine-tune the LLM on these specific tasks. Data could include chemical properties of compounds such as molecular structure, reactivity and persistence in the environment among others, knowledge on exposure pathways, toxicological information, regulatory standards, risk assessment frameworks, for example dose-response assessments, hazard identification and many other factors.

### 4.1 Limitations

The results for the text mining and for word embeddings are “undirected” and reflect only co-occurrence. This means top results are not necessarily most toxic compounds but e.g. also compounds used to treat toxicities. Examples are acetylcysteine (MESH: D000111) or vitamin E (MESH: D014810) found with higher scores in the text mining and Word2Vec.

All approaches are based on the published literature or common knowledge. We can only unravel hidden information or summarize and reorganize already existing information. But using the existing literature, we can not assess or predict toxicity of novel compounds directly.

In order to predict DILI for completely unknown compounds we would need additional layers of information such as physico-chemical properties of compounds, information on modes of action or molecular targets. These could be used to predict toxicity for the unknown compounds with the help of known compounds with similar properties.

Finally, validating LLMs poses challenges due to their extensive training process. In addition, validation information may have been included in the training process (directly or indirectly). This means that the LLM could have seen the FDA DILI rank labels during training and identified the dataset patterns during inference. This eventually leads to artificially high performance.

## Data Availability

The original contributions presented in the study are included in the article/Supplementary material, further inquiries can be directed to the corresponding author.

## Footnotes

^1^https://api.fireworks.ai/inference/v1/chat/completions.

## References

[B1] Bai G. Liu J. Bu X. He Y. Liu J. Zhou Z.. (2024). Mt-bench-101: A fine-grained benchmark for evaluating large language models in multi-turn dialogues. arXiv [preprint] arXiv.2402.14762. 10.48550/arXiv.2402.14762

[B2] Bauer C. Herwig R. Lienhard M. Prasse P. Scheffer T. Schuchhardt J. (2021). Large-scale literature mining to assess the relation between anti-cancer drugs and cancer types. J. Transl. Med. 19:274. 10.1186/s12967-021-02941-z 34174885 PMC8236166

[B3] Canada A. Capella-Gutierrez S. Rabal O. Oyarzabal J. Valencia A. Krallinger M. (2017). LimTox: a web tool for applied text mining of adverse event and toxicity associations of compounds, drugs and genes. Nucleic Acids Res. 45, W484–W489. 10.1093/nar/gkx462 28531339 PMC5570141

[B4] Carvaillo J. C. Barouki R. Coumoul X. Audouze K. (2019). Linking bisphenol s to adverse outcome pathways using a combined text mining and systems biology approach. Environ. Health Perspect. 127:47005. 10.1289/EHP4200 30994381 PMC6785233

[B5] Chalasani N. Bonkovsky H. Fontana R. Lee W. Stolz A. Talwalkar J.. (2015). Features and outcomes of 899 patients with drug-induced liver injury: the DILIN prospective study. Gastroenterology 148, 1340–1352.e7. 10.1053/j.gastro.2015.03.006 25754159 PMC4446235

[B6] Chen M. Suzuki A. Thakkar S. Yu K. Hu C. Tong W. (2016). DILIrank: the largest reference drug list ranked by the risk for developing drug-induced liver injury in humans. Drug Discov. Today 21, 648–653. 10.1016/j.drudis.2016.02.015 26948801

[B7] Chiang W.-L. Zheng L. Sheng Y. Angelopoulos A. N. Li T. Li D.. (2024). Chatbot Arena: An Open Platform for Evaluating LLMs by Human Preference. Ithaca, NY: Cornell University. 10.48550/arXiv.2403.04132

[B8] Hoofnagle J. H. (2013). “Chapter 40 - livertox: A website on drug-induced liver injury,” in Drug-Induced Liver Disease (Third Edition), eds. Kaplowitz N. DeLeve L. D. (Boston: Academic Press), 725–732.

[B9] Jaylet T. Coustillet T. Jornod F. Margaritte-Jeannin P. Audouze K. (2023). AOP-helpFinder 2.0: Integration of an event-event searches module. Environ. Int. 177:108017. 10.1016/j.envint.2023.108017 37295163

[B10] Jornod F. Jaylet T. Blaha L. Sarigiannis D. Tamisier L. Audouze K. (2022). AOP-helpFinder webserver: a tool for comprehensive analysis of the literature to support adverse outcome pathways development. Bioinformatics 38, 1173–1175. 10.1093/bioinformatics/btab750 34718414 PMC8796376

[B11] Leaman R. Lu Z. (2016). TaggerOne: joint named entity recognition and normalization with semi-Markov Models. Bioinformatics 32, 2839–2846. 10.1093/bioinformatics/btw343 27283952 PMC5018376

[B12] Lee J. Yoon W. Kim S. Kim D. Kim S. So C. H.. (2020). BioBERT: a pre-trained biomedical language representation model for biomedical text mining. Bioinformatics 36, 1234–1240. 10.1093/bioinformatics/btz682 31501885 PMC7703786

[B13] Ma C. Wolfinger R. (2024). Toward an explainable large language model for the automatic identification of the drug-induced liver injury literature. Chem. Res. Toxicol. 37, 1524–1534. 10.1021/acs.chemrestox.4c00134 39190012

[B14] Mikolov T. Sutskever I. Chen K. Corrado G. Dean J. (2013). Distributed representations of words and phrases and their compositionality. arXiv [preprint] arXiv.1310.4546. 10.48550/arXiv.1310.4546

[B15] Nori H. Lee Y. T. Zhang S. Carignan D. Edgar R. Fusi N.. (2023). Can Generalist Foundation Models Outcompete Special-Purpose Tuning? Case Study in Medicine. Ithaca, NY: Cornell University. 10.48550/arXiv.2311.16452

[B16] Pennington J. Socher R. Manning C. D. (2014). “Glove: Global vectors for word representation,” in Proceedings of the 2014 Conference on Empirical Methods in Natural Language Processing (EMNLP) (Doha: Association for Computational Linguistics), 1532–1543.

[B17] Petroni F. Lewis P. Piktus A. Rocktäschel T. Wu Y. Miller A. H.. (2020). “How context affects language models' factual predictions,” in Automated Knowledge Base Construction. Ithaca, NY: Cornell University. 10.48550/arXiv.2005.04611

[B18] Rusyn I. Arzuaga X. Cattley R. Corton J. Ferguson S. Godoy P.. (2021). Key characteristics of human hepatotoxicants as a basis for identification and characterization of the causes of liver toxicity. Hepatology 74, 3486–3496. 10.1002/hep.31999 34105804 PMC8901129

[B19] Singhal K. Azizi S. Tu T. Mahdavi S. S. Wei J. Chung H. W.. (2023). Large language models encode clinical knowledge. Nature 620, 172–180. 10.1038/s41586-023-06291-2 37438534 PMC10396962

[B20] Sung M. Jeong M. Choi Y. Kim D. Lee J. Kang J. (2022). BERN2: an advanced neural biomedical named entity recognition and normalization tool. Bioinformatics 38, 4837–4839. 10.1093/bioinformatics/btac598 36053172 PMC9563680

[B21] Tran T. T. V. Surya Wibowo A. Tayara H. Chong K. T. (2023). Artificial intelligence in drug toxicity prediction: recent advances, challenges, and future perspectives. J. Chem. Inf. Model. 63, 2628–2643. 10.1021/acs.jcim.3c00200 37125780

[B22] Wei C. H. Allot A. Leaman R. Lu Z. (2019). PubTator central: automated concept annotation for biomedical full text articles. Nucleic Acids Res. 47, W587–W593. 10.1093/nar/gkz389 31114887 PMC6602571

[B23] Wei C. H. Kao H. Y. Lu Z. (2015). GNormPlus: an integrative approach for tagging genes, gene families, and protein domains. Biomed Res. Int. 2015:918710. 10.1155/2015/918710 26380306 PMC4561873

