# Supplementary material for: Systematic analysis of hepatotoxicity: combining literature mining and AI language models
Source: Front Artif Intell. 2025 Jul 21;8:1561292. doi: 10.3389/frai.2025.1561292 (PMC12338115; doi:10.3389/frai.2025.1561292)
Supplement: Supplementary file 2 [file Data_Sheet_1.pdf]

MESH:C004343 - mebanazine

Summary:

LLM Prediction Score	0.000 (normalized)
LLM Confidence Score	0.830
Golden Answer (Severity Class)	1.0 (normalized)
Prediction Error	1.000

Retrieved Context:

Title: Mechanisms of drug toxicity and relevance to pharmaceutical development.
Toxicity has been estimated to be responsible for the attrition of approximately one-third of drug candidates and is a major contributor to the high cost of drug development, particularly when not recognized until late in clinical trials or post-marketing. The causes of drug toxicity can be classified in several ways and include mechanism-based (on-target) toxicity, immune hypersensitivity, off-target toxicity, and bioactivation/covalent modification. In addition, idiosyncratic responses are rare but can be one of the most problematic issues; several hypotheses for these have been advanced. Although covalent binding of drugs to proteins was described almost 40 years ago, the significance to... (truncated)

MESH:C447622 - fiduxosin

Summary:

LLM Prediction Score	0.005 (normalized)
LLM Confidence Score	0.580
Golden Answer (Severity Class)	1.0 (normalized)
Prediction Error	0.995

Retrieved Context:

Title: Inhibition of APE1/Ref-1 for Neovascular Eye Diseases: From Biology to Therapy.
Proliferative diabetic retinopathy (PDR), neovascular age-related macular degeneration (nvAMD), retinopathy of prematurity (ROP) and other eye diseases are characterized by retinal and/or choroidal neovascularization, ultimately causing vision loss in millions of people worldwide. nvAMD and PDR are associated with aging and the number of those affected is expected to increase as the global median age and life expectancy continue to rise. With this increase in prevalence, the development of novel, orally bioavailable therapies for neovascular eye diseases that target multiple pathways is critical, since current anti-vascular endothelial growth factor (VEGF) treatments, delivered by intravitreal injection, are accompanied with tachyphylaxis, a... (truncated)

MESH:C086167 - tasosartan

Summary:

LLM Prediction Score	0.048 (normalized)
LLM Confidence Score	0.700
Golden Answer (Severity Class)	1.0 (normalized)
Prediction Error	0.952

Retrieved Context:

Title: Therapeutic Effect of Losartan, an Angiotensin II Type 1 Receptor Antagonist, on CCl_4-Induced Skeletal Muscle Injury.
TGF-β1 is known to inhibit muscle regeneration after muscle injury. However, it is unknown if high systemic levels of TGF-β can affect the muscle regeneration process. In the present study, we demonstrated the effect of a CCl_4 intra-peritoneal injection and losartan (an angiotensin II type 1 receptor antagonist) on skeletal muscle (gastrocnemius muscle) injury and regeneration. Male C57BL/6 mice were grouped randomly as follows: control (n = 7), CCl_4-treatment group (n = 7), and CCl_4 + losartan treatment group (n = 7). After CCl_4 treatment for a 16-week period, the animals were sacrificed and analyzed. The expression of dystrophin significantly... (truncated)

Title: CSH guidelines for the diagnosis and treatment of drug-induced liver injury.
Drug-induced liver injury (DILI) is an important clinical problem, which has received more attention in recent decades. It can be induced by small chemical molecules, biological agents, traditional Chinese medicines (TCM), natural medicines (NM), health products (HP), and dietary supplements (DS). Idiosyncratic DILI is far more common than intrinsic DILI clinically and can be classified into hepatocellular injury, cholestatic injury, hepatocellular-cholestatic mixed injury, and vascular injury based on the types of injured target cells. The CSH guidelines summarized the epidemiology, pathogenesis, pathology, and clinical manifestation and gives 16 evidence-based recommendations on diagnosis, differential diagnosis, treatment, and prevention of DILI.

Title: In Vitro Rescue of the Bile Acid Transport Function of ABCB11 Variants by CFTR Potentiators.
ABCB11 is responsible for biliary bile acid secretion at the canalicular membrane of hepatocytes. Variations in the <i>ABCB11</i> gene cause a spectrum of rare liver diseases. The most severe form is progressive familial intrahepatic cholestasis type 2 (PFIC2). Current medical treatments have limited efficacy. Here, we report the in vitro study of Abcb11 missense variants identified in PFIC2 patients and their functional rescue using cystic fibrosis transmembrane conductance regulator potentiators. Three ABCB11 disease-causing variations identified in PFIC2 patients (i.e., A257V, T463I and G562D) were reproduced in a plasmid encoding an Abcb11-green fluorescent protein. After transfection, the expression and localization of... (truncated)

Title: Upcycling the anthracyclines: New mechanisms of action, toxicology, and pharmacology.
The anthracyclines are a family of natural products isolated from soil bacteria with over 2000 chemical representatives. Since their discovery seventy years ago by Waksman and co-workers, anthracyclines have become one of the best-characterized anticancer chemotherapies in clinical use. The anthracyclines exhibit broad-spectrum antineoplastic activity for the treatment of a variety of solid and liquid tumors, however, their clinical use is limited by their dose-limiting cardiotoxicity. In this review article, we discuss the toxicity of the anthracyclines on several organ systems, including new insights into doxorubicin-induced cardiotoxicity. In addition, we discuss new medicinal chemistry developments in the biosynthesis of new... (truncated)

Title: Pyrido[2,3-<i>d</i>]pyrimidin-7(8<i>H</i>)-ones: Synthesis and Biomedical Applications.
Pyrido[2,3-<i>d</i>]pyrimidines (1) are a type of privileged heterocyclic scaffolds capable of providing ligands for several receptors in the body. Among such structures, our group and others have been particularly interested in pyrido[2,3-<i>d</i>]pyrimidine-7(8<i>H</i>)-ones (2) due to the similitude with nitrogen bases present in DNA and RNA. Currently there are more than 20,000 structures 2 described which correspond to around 2900 references (half of them being patents). Furthermore, the number of references containing compounds of general structure 2 have increased almost exponentially in the last 10 years. The present review covers the synthetic methods used for the synthesis of pyrido[2,3-<i>d</i>]pyrimidine-7(8<i>H</i>)-ones (2), both... (truncated)

MESH:D013948 - moxisylyte

Summary:

LLM Prediction Score	0.071 (normalized)
LLM Confidence Score	0.790
Golden Answer (Severity Class)	1.0 (normalized)
Prediction Error	0.929

Retrieved Context:

Title: Alterations of Gut Microbiome and Serum Metabolome in Coronary Artery Disease Patients Complicated With Non-alcoholic Fatty Liver Disease Are Associated With Adverse Cardiovascular Outcomes.
Rationale: Patients suffering from coronary artery disease (CAD) complicated with nonalcoholic fatty liver disease (NAFLD) present worse cardiovascular outcomes than CAD patients without NAFLD. The progression of CAD is recently reported to be associated with gut microbiota and microbe-derived metabolites. However, it remains unclear how the complication of NAFLD will affect gut microbiota and microbe-derived metabolites in CAD patients, and whether or not this interplay is related to the worse cardiovascular outcomes in CAD-NAFLD patients. Methods: We performed 16S rRNA sequencing and serum metabolomic analysis in 27 CAD patients with NAFLD, 81 CAD patients without NAFLD, and 24 matched healthy... (truncated)

Title: Translating clinical findings into knowledge in drug safety evaluation--drug induced liver injury prediction system (DILIps).
Drug-induced liver injury (DILI) is a significant concern in drug development due to the poor concordance between preclinical and clinical findings of liver toxicity. We hypothesized that the DILI types (hepatotoxic side effects) seen in the clinic can be translated into the development of predictive in silico models for use in the drug discovery phase. We identified 13 hepatotoxic side effects with high accuracy for classifying marketed drugs for their DILI potential. We then developed in silico predictive models for each of these 13 side effects, which were further combined to construct a DILI prediction system (DILIps). The DILIps yielded... (truncated)

Title: Association of CYP1A1 and CYP1B1 inhibition in in vitro assays with drug-induced liver injury.
Drug-induced liver injury (DILI) is one of the major causes for the discontinuation of drug development and withdrawal of drugs from the market. Since it is known that reactive metabolite formation and being substrates or inhibitors of cytochrome P450s (P450s) are associated with DILI, we systematically investigated the association between human P450 inhibition and DILI. The inhibitory activity of 266 DILI-positive drugs (DILI drugs) and 92 DILI-negative drugs (no-DILI drugs), which were selected from Liver Toxicity Knowledge Base (US Food and Drug Administration), against 8 human P450 forms was assessed using recombinant enzymes and luminescent substrates, and the threshold values... (truncated)

Title: Effect of common medications on the expression of SARS-CoV-2 entry receptors in liver tissue.
Besides lung drastic involvement, SARS-CoV-2 severely affected other systems including liver. Emerging epidemiological studies brought the attentions towards liver injury and impairment as a potential outcome of COVID19. Angiotensin-converting enzyme 2 (ACE2) and Transmembrane serine protease (TMPRSS2) are the main cell entry receptors of SARS-CoV-2. We have tested the ability of medications to regulate expression of SARS-CoV-2 receptors. Understanding that may reflect how such medications may affect the level of infectivity and permissibility of the liver following COVID-19. Using transcriptomic datasets, Toxicogenomic Project-Genomics Assisted Toxicity Evaluation System (Open TG-GATEs) and GSE30351, we have tested the ability of ninety common medications... (truncated)

Title: Mechanisms of drug toxicity and relevance to pharmaceutical development.
Toxicity has been estimated to be responsible for the attrition of approximately one-third of drug candidates and is a major contributor to the high cost of drug development, particularly when not recognized until late in clinical trials or post-marketing. The causes of drug toxicity can be classified in several ways and include mechanism-based (on-target) toxicity, immune hypersensitivity, off-target toxicity, and bioactivation/covalent modification. In addition, idiosyncratic responses are rare but can be one of the most problematic issues; several hypotheses for these have been advanced. Although covalent binding of drugs to proteins was described almost 40 years ago, the significance to... (truncated)

MESH:C037665 - tilbroquinol

Summary:

LLM Prediction Score	0.102 (normalized)
LLM Confidence Score	0.620
Golden Answer (Severity Class)	1.0 (normalized)
Prediction Error	0.898

Retrieved Context:

Title: An underestimated sexually transmitted infection: amoebiasis.
<i>Entamoeba histolytica</i> is a cosmopolitan pathogenic parasite. It is spread via the feco-oral route and, to a lesser extent, via sexual intercourse. We report a case of hepatic and intestinal amoebiasis in a 67-year-old man who had never travelled to an endemic area. Abdominal CT investigations detected two liver abscesses and chronic colitis. Positive amoebic serology and a positive PCR test for <i>E. histolytica</i> in the hepatic liquid and faeces confirmed the diagnosis. Curative metronidazole and tiliquinol-tilbroquinol were administered successfully. The patient had been contaminated through heterosexual intercourse with his healthy French female partner who was a carrier of the... (truncated)

MESH:D011464 - epoprostenol

Summary:

LLM Prediction Score	0.000 (normalized)
LLM Confidence Score	0.980
Golden Answer (Severity Class)	0.875 (normalized)
Prediction Error	0.875

Retrieved Context:

Title: Prostanoids and phosphodiesterase inhibitors in experimental pulmonary hypertension.
Pulmonary arterial hypertension (PAH) is a progressive disease with a poor prognosis, characterized by intimal lesions, medial hypertrophy, and adventitial thickening of precapillary pulmonary arteries. Several approved therapies are currently available for the treatment of PAH, of which intravenous epoprostenol is the best explored over the past decade. Newly available oral endothelin receptor antagonists, although clinically efficacious, bear the risk of liver toxicity in a significant portion of patients. Substances that stimulate the formation of the second messengers cyclic adenosine monophosphate (cAMP) or guanosine monophosphate (cGMP) have proved useful in the treatment of various forms of pre-capillary pulmonary hypertension. These... (truncated)

Title: Epoprostenol-associated ascites in pulmonary arterial hypertension.
The development of ascites in pulmonary arterial hypertension (PAH) in the absence of pre-existing hepatic dysfunction is usually associated with decompensated right heart failure or cardiac cirrhosis. Ascites in PAH has rarely been associated with intravenous epoprostenol, a synthetic form of the prostaglandin PGI₂.

Title: Portopulmonary hypertension.
It has been widely accepted that development of porto-pulmonary hypertension (POPH) is independent of the cause of portal hypertension. The degree of hepatic damage and liver function do not correlate with predisposition to POPH or its severity. However, portal hypertension has been confirmed as a prerequisite for developing pulmonary hypertension. Transthoracic echocardiography is the best screening test for the presence of POPH, but a diagnosis of POPH can be established only by right heart catheterization. Randomized controlled trials comparing the efficacy and safety of different pharmacologic strategies are lacking in patients with POPH. The general management includes diuretics and oxygen... (truncated)

Title: Hospitalization and survival in patients using epoprostenol for injection in the PROSPECT observational study.
Few studies have prospectively reported outcomes in patients with pulmonary arterial hypertension (PAH) treated with epoprostenol in the modern-day era of oral therapy and combination treatments. The Registry to Prospectively Describe Use of Epoprostenol for Injection (Veletri, prolonged room temperature stable-epoprostenol [RTS-Epo]) in Patients with Pulmonary Arterial Hypertension (PROSPECT) was established to prospectively describe the course of PAH in patients prescribed RTS-Epo.

Title: Nonselective inhibition of prostaglandin-endoperoxide synthases by naproxen ameliorates acute or chronic liver injury in animals.
The rising prevalence of hepatic injury due to toxins, metabolites, viruses, etc., necessitates development of further mechanisms for protecting the liver and for treating acute or chronic liver diseases. To examine whether inhibition of inflammation is directed by cyclo-oxygenase pathways, we performed animal studies with naproxen, which inhibits prostaglandin-endoperoxide synthases 1 and 2 and is in extensive clinical use. We administered carbon tetrachloride to induce acute liver injury and ligated the common bile duct to induce chronic liver injury in adult rats. These experimental manipulations produced abnormalities in liver tests, tissue necrosis, compensatory hepatocyte or biliary proliferation, and onset of... (truncated)

MESH:D000077612 - anidulafungin

Summary:

LLM Prediction Score	0.000 (normalized)
LLM Confidence Score	0.940
Golden Answer (Severity Class)	0.875 (normalized)
Prediction Error	0.875

Retrieved Context:

Title: [Anidulafungin].
Anidulafungin is a new echinocandin antifungal agent recently approved in Spain by the Spanish Drug Agency. As other echinocandins, it inhibits a selective target, 1,3- beta-D-glucan synthesis, a major structural component of the fungal cell wall which is not present in mammalian cells, this avoiding toxicity problems. It has fungicidal activity against many Candida spp., including fluconazole-resistant, and fungistatic activity against other yeast and moulds such as Aspergillus spp. Clinical trials have shown non-inferiority of anidulafungin to fluconazole for invasive, including candidemia, and non-invasive Candida infections. It is well-tolerated, and no drug-related serious adverse events have been reported. Anidulafungin, which... (truncated)

Title: Switching to anidulafungin from caspofungin in cancer patients in the setting of liver dysfunction is associated with improvement of liver function tests.
Anidulafungin does not undergo hepatic metabolism like the other echinocandins. Therefore, there is a perception that anidulafungin may be less hepatotoxic or less likely to exacerbate existing liver damage. This has not been substantiated in the literature.

Title: Echinocandins: A ray of hope in antifungal drug therapy.
Invasive fungal infections are on the rise. Amphotericin B and azole antifungals have been the mainstay of antifungal therapy so far. The high incidence of infusion related toxicity and nephrotoxicity with amphotericin B and the emergence of fluconazole resistant strains of Candida glabrata egged on the search for alternatives. Echinocandins are a new class of antifungal drugs that act by inhibition of beta (1, 3)-D- glucan synthase, a key enzyme necessary for integrity of the fungal cell wall.Caspofungin was the first drug in this class to be approved. It is indicated for esophageal candidiasis, candidemia, invasive candidiasis, empirical therapy in... (truncated)

Title: Preclinical Evaluation of the Stability, Safety, and Efficacy of CD101, a Novel Echinocandin.
Fungal infections pose a significant public health burden with high morbidity and mortality. CD101 is a novel echinocandin under development for the treatment and prevention of systemic Candida infections. Preclinical studies were conducted to evaluate the metabolic stability, plasma protein binding, pharmacokinetics, toxicity, and efficacy of CD101 at various dose levels. CD101 was stable to biotransformation in rat, monkey, and human liver microsomes and rat, monkey, dog, and human hepatocytes. In vitro studies suggest minimal interaction with recombinant cytochrome P450 enzymes (50% inhibitory concentrations [IC₅₀s] of >10 μM). Similar to anidulafungin, CD101 bound avidly (>98%) to human, mouse, rat, and... (truncated)

Title: Safety of anidulafungin in solid organ transplant recipients.
The aim of this study was the evaluation of the safety of anidulafungin in adult solid organ transplantation (SOT) recipients. During the study period (14 months), we included all consecutive SOT recipients from 14 centers who received anidulafungin for at least 48 hours for the treatment of invasive fungal infections (IFIs) or as prophylaxis. Relevant clinical and analytical information on clinical charts was reviewed. Clinical side effects, liver function tests, and serum creatinine levels were assessed at least weekly. The need for the modification of immunosuppressive drugs was also recorded by the investigators. All patients were followed for at least... (truncated)

MESH:C038637 - balsalazide

Summary:

LLM Prediction Score	0.148 (normalized)
LLM Confidence Score	0.930
Golden Answer (Severity Class)	1.0 (normalized)
Prediction Error	0.852

Retrieved Context:

Title: Safety, efficacy, and pharmacokinetics of balsalazide in pediatric patients with mild-to-moderate active ulcerative colitis: results of a randomized, double-blind study.
: A multicenter, double-blind study was conducted to evaluate the safety, efficacy, and pharmacokinetics of balsalazide in pediatric patients with mild-to-moderate ulcerative colitis (UC).

Title: Risk-stratified monitoring for thiopurine toxicity in immune-mediated inflammatory diseases: prognostic model development, validation, and, health economic evaluation.
Patients established on thiopurines (e.g., azathioprine) are recommended to undergo three-monthly blood tests for the early detection of blood, liver, or kidney toxicity. These side-effects are uncommon during long-term treatment. We developed a prognostic model that could be used to inform risk-stratified decisions on frequency of monitoring blood-tests during long-term thiopurine treatment, and, performed health-economic evaluation of alternate monitoring intervals.

Title: Current and emerging drugs for the treatment of inflammatory bowel disease.
During the last decade a large number of biological agents against tumor necrosis factor-α (TNF-α), as well as many biochemical substances and molecules specifically for the medical treatment of patients with inflammatory bowel disease (IBD), have been developed. This enormous progress was a consequence of the significant advances in biotechnology along with the increased knowledge of the underlying pathophysiological mechanisms involved in the pathogenesis of IBD. However, conventional therapies remain the cornerstone of treatment for most patients. During recent years conventional and biologic IBD therapies have been optimized. Newer mesalazine formulations with a reduced pill size and only one dose... (truncated)

Title: Inflammatory bowel disease type influences development of elevated liver enzymes.
Up to a third of patients with inflammatory bowel disease (IBD) have elevated liver enzymes (ELE). We evaluated the incidence, predictors, and outcomes associated with ELE in a diverse and vulnerable IBD cohort.

Title: Preclinical Pharmacokinetics and Acute Toxicity in Rats of 5-{[(2E)-3-Bromo-3-carboxyprop-2-enoyl]amino}-2-hydroxybenzoic Acid: A Novel 5-Aminosalicylic Acid Derivative with Potent Anti-Inflammatory Activity.
Compound 5-{[(2E)-3-bromo-3-carboxyprop-2-enoyl]amino}-2-hydroxybenzoic acid (C1), a new 5-aminosalicylic acid (5-ASA) derivative, has proven to be an antioxidant in vitro and an anti-inflammatory agent in mice. The in vivo inhibition of myeloperoxidase was comparable to that of indomethacin. The aim of this study was to take another step in the preclinical evaluation of C1 by examining acute toxicity with the up-and-down OECD method and pharmacokinetic profiles by administration of the compound to Wistar rats through intravenous (i.v.), oral (p.o.), and intraperitoneal (i.p.) routes. According to the Globally Harmonized System, C1 belongs to categories 4 and 5 for the i.p. and p.o. routes,... (truncated)

MESH:D000077334 - zolpidem

Summary:

LLM Prediction Score	0.050 (normalized)
LLM Confidence Score	0.990
Golden Answer (Severity Class)	0.875 (normalized)
Prediction Error	0.825

Retrieved Context:

Title: Evaluation of the potential for drug-induced liver injury based on in vitro covalent binding to human liver proteins.
Prediction of idiosyncratic drug-induced liver injury (DILI) is difficult, and the underlying mechanisms are not fully understood. However, many drugs causing DILI are considered to form reactive metabolites and covalently bind to cellular macromolecules in the liver. The objective of this study was to clarify whether the risk of idiosyncratic DILI can be estimated by comparing in vitro covalent binding (CB) levels among 12 positive compounds (acetaminophen, alpidem, bromfenac, carbamazepine, diclofenac, flutamide, imipramine, nefazodone, tacrine, ticlopidine, tienilic acid, and troglitazone) for DILI and 12 negative compounds (acetylsalicylic acid, caffeine, dexamethasone, losartan, ibuprofen, paroxetine, pioglitazone, rosiglitazone, sertraline, theophylline, venlafaxine, and zolpidem).... (truncated)

Title: Zolpidem high-dose abuse: what about the liver? Results from a series of 107 patients.
Objectives: Z-Drugs (ZDs) have been developed to limit benzodiazepines (BZDs) abuse for sleep disorders. Data on the liver toxicity of zolpidem (ZLM) are lacking or anecdotal. The authors evaluated the presence of drug-induced liver injury (DILI) among a cohort of high-dose ZLM abusers. Methods: Retrospective study analyzing clinical records of 1112 consecutive patients admitted for BZDs detoxification from 2003 to 2018. Inclusion criteria: age >18 y.o.; ZLM abuse/dependence; high-dose ZDs abuse. Exclusion criteria: missing lab data; lack of informed consent. Main outcome was the presence of DILI measured as elevation of ALT/AST levels >250 U/l. Results: A total of 107... (truncated)

Title: Severe chronic abuse of zolpidem for over 10 years: a case report and review of similar cases.
Insomnia is a major health issue, and zolpidem is an effective treatment for insomnia. However, high doses of zolpidem can cause dependence, abuse, and withdrawal symptoms, questioning its advantages.

Title: The implementation of per-se limits for driving under the influence of benzodiazepines and related drugs: No increased risk for arrest during therapeutic use in Norway.
Objective: To investigate whether the use of recommended therapeutic doses of medicinal drugs has led to suspicion of driving under the influence of drugs (DUID) after implementation of legislative limits for illicit and medicinal drugs in 2012.Methods: Data from suspected drug-impaired drivers apprehended by the police from 2013 to 2015 were selected from the Norwegian Forensic Toxicology Database. The blood samples had been analyzed for benzodiazepines (BZDs), z-hypnotics, opioids, stimulants, certain hallucinogens, and alcohol. Drivers who tested positive for one BZD or a z-hypnotic only, were included in the study. Drug concentrations measured in their blood samples were compared to... (truncated)

Title: Anxioselective anxiolytics: on a quest for the Holy Grail.
The discovery of benzodiazepine receptors provided the impetus to discover and develop anxioselective anxiolytics ('Valium without the side effects'). The market potential for an anxioselective based on the γ-aminobutyric acid A (GABA(A)) receptor resulted in clinical trials of multiple compounds. In contrast to the anxioselective profile displayed in preclinical models, compounds such as bretazenil, TPA023, and MRK 409 produced benzodiazepine-like side effects (sedation, dizziness) in Phase I studies, whereas alpidem and ocinaplon exhibited many of the characteristics of an anxioselective in the clinic. Alpidem was briefly marketed for the treatment of anxiety, but was withdrawn because of liver toxicity. Reversible... (truncated)

MESH:D017294 - ondansetron

Summary:

LLM Prediction Score	0.074 (normalized)
LLM Confidence Score	0.990
Golden Answer (Severity Class)	0.875 (normalized)
Prediction Error	0.801

Retrieved Context:

Title: Ondansetron--the first of a new class of antiemetic agents.
The chemistry, pharmacokinetics, adverse effects, stability, compatibility, and dosage of ondansetron hydrochloride are described, and clinical studies of the use of ondansetron for the prophylaxis of nausea and vomiting induced by antineoplastic therapy are reviewed. Ondansetron hydrochloride is a specific antagonist of serotonin type 3 (5-HT3) receptors, both in the chemoreceptor trigger zone and in the GI tract. Peak plasma concentrations of ondansetron occur approximately one hour after an oral dose and 6 to 20 minutes after an i.v. dose. The mean elimination half-life is approximately 3.5 hours in healthy volunteers, but it is extended in elderly patients (mean of... (truncated)

Title: Effect of ondansetron in preventing postoperative nausea and vomiting under different conditions of general anesthesia: a preliminary, randomized, controlled study.
Two hundred and forty patients were randomly allocated into six groups: Group I, anesthesia was maintained with sevoflurane; Group II, anesthesia was maintained with sevoflurane and 8 mg of ondansetron; Group III, anesthesia was maintained with propofol; Group IV, anesthesia was maintained with propofol and 8 mg of ondansetron; Group V, anesthesia was maintained with sevoflurane and propofol; Group VI, anesthesia was maintained with sevoflurane combined with propofol and 8 mg of ondansetron.

Title: Early Postoperative Ondansetron Exposure is Associated with Reduced 90-Day Mortality in Patients Undergoing Cardiac Surgery.
Ondansetron is a widely used anti-emetic for the prevention and treatment of nausea and vomiting for patients in critical care. Recent retrospective cohort studies suggest the potential beneficial effects of ondansetron in critically ill patients. In this study, we investigate the impact of ondansetron use on patient outcomes after cardiac surgery.

Title: Controlling emesis related to cancer therapy.
Combinations of dopamine antagonists or high-dose metoclopramide with steroids can provide complete control of chemotherapy-related nausea and vomiting in up to 60-70% of patients undergoing high-dose cisplatin-based chemotherapy. High-dose metoclopramide probably acts as a 5-HT3 receptor antagonist, but because of its dopamine-receptor antagonism it is the cause of extrapyramidal side-effects. These compounds, and the agents used in combination with them, tend to cause sedation, an undesirable effect in the outpatient setting. Specific 5-HT3 receptor antagonists (ondansetron, granisetron, tropisetron) give a similar control of chemotherapy related nausea and vomiting, with minimum side-effects. These drugs can cause headaches and constipation and some... (truncated)

Title: A Comparison of Fosaprepitant and Ondansetron for Preventing Postoperative Nausea and Vomiting in Moderate to High Risk Patients: A Retrospective Database Analysis.
Postoperative nausea and vomiting (PONV) occur in 30-50% of patients undergoing general anesthesia and in 70-80% of high PONV risk patients. In this study, we investigated the efficacy of fosaprepitant, a neurokinin-1 (NK1) receptor antagonist, compared to ondansetron, a selective 5-hydroxytryptamine type 3 (5-HT3) receptor antagonist, in moderate to high PONV risk patients from our previous randomized controlled trials. Patients (171 patients from 4 pooled studies) with the Apfel simplified score ≥ 2 and undergoing general anesthesia were randomly allocated to receive intravenous fosaprepitant 150 mg (NK1 group, <i>n</i> = 82) and intravenous ondansetron 4 mg (ONS group, <i>n</i> = 89) before... (truncated)

MESH:D008704 - methazolamide

Summary:

LLM Prediction Score	0.089 (normalized)
LLM Confidence Score	0.880
Golden Answer (Severity Class)	0.875 (normalized)
Prediction Error	0.786

Retrieved Context:

Title: Genotyping for severe drug hypersensitivity.
Over the past decade, there have been significant advances in our understanding of the immunopathogenesis and pharmacogenomics of severe immunologically-mediated adverse drug reactions. Such T-cell-mediated adverse drug reactions such as Stevens-Johnson syndrome/toxic epidermal necrolysis (SJS/TEN), drug-induced liver disease (DILI) and other drug hypersensitivity syndromes have more recently been shown to be mediated through interactions with various class I and II HLA alleles. Key examples have included the associations of HLA-B*15:02 and carbamazepine induced SJS/TEN in Southeast Asian populations and HLA-B*57:01 and abacavir hypersensitivity. HLA-B*57:01 screening to prevent abacavir hypersensitivity exemplifies a successful translational roadmap from pharmacogenomic discovery through to widespread... (truncated)

Title: Fever, rash, and systemic symptoms: understanding the role of virus and HLA in severe cutaneous drug allergy.
Drug hypersensitivity syndromes such as abacavir hypersensitivity and the severe cutaneous adverse drug reactions have been associated with significant short- and long-term morbidity and mortality. More recently, these immunologically mediated and previously unpredictable diseases have been shown to be associated with primarily class I but also class II HLA alleles. The case of the association of HLA-B*57:01 and abacavir hypersensitivity has created a translational roadmap for how this knowledge can be used in the clinic to prevent severe reactions. Although many hurdles exist to the widespread translation of such HLA screening approaches, our understanding of how drugs interact with the... (truncated)

Title: Disruption of thioredoxin reductase 1 protects mice from acute acetaminophen-induced hepatotoxicity through enhanced NRF2 activity.
The critical importance of glutathione in mitigating the deleterious effects of electrophile generating drugs such as acetaminophen (APAP) is well established. However, the role of other antioxidant systems, such as that provided by thioredoxin, has not been extensively studied. Selenoprotein thioredoxin reductase 1 (Txnrd1) is important for attenuating activation of the apoptosis signaling-regulating kinase 1 (ASK1) and the c-Jun N-terminal kinase (JNK) pathway caused by high doses of APAP. Therefore, a detailed investigation of the role of Txnrd1 in APAP-induced hepatotoxicity was conducted. Liver-specific Txnrd1 knockout mice (Txnrd1(ΔLiv)) were generated and treated with a hepatotoxic dose (400 mg/kg) of APAP... (truncated)

Title: Association of CYP1A1 and CYP1B1 inhibition in in vitro assays with drug-induced liver injury.
Drug-induced liver injury (DILI) is one of the major causes for the discontinuation of drug development and withdrawal of drugs from the market. Since it is known that reactive metabolite formation and being substrates or inhibitors of cytochrome P450s (P450s) are associated with DILI, we systematically investigated the association between human P450 inhibition and DILI. The inhibitory activity of 266 DILI-positive drugs (DILI drugs) and 92 DILI-negative drugs (no-DILI drugs), which were selected from Liver Toxicity Knowledge Base (US Food and Drug Administration), against 8 human P450 forms was assessed using recombinant enzymes and luminescent substrates, and the threshold values... (truncated)

Title: HLA Association with Drug-Induced Adverse Reactions.
Adverse drug reactions (ADRs) remain a common and major problem in healthcare. Severe cutaneous adverse drug reactions (SCARs), such as Stevens-Johnson syndrome (SJS)/toxic epidermal necrolysis (TEN) with mortality rate ranges from 10% to more than 30%, can be life threatening. A number of recent studies demonstrated that ADRs possess strong genetic predisposition. ADRs induced by several drugs have been shown to have significant associations with specific alleles of human leukocyte antigen (HLA) genes. For example, hypersensitivity to abacavir, a drug used for treating of human immunodeficiency virus (HIV) infection, has been proposed to be associated with allele 57:01 of <i>HLA-B</i>... (truncated)

MESH:C025293 - alaproclate

Summary:

LLM Prediction Score	0.238 (normalized)
LLM Confidence Score	0.620
Golden Answer (Severity Class)	1.0 (normalized)
Prediction Error	0.762

Retrieved Context:

Title: Association of CYP1A1 and CYP1B1 inhibition in in vitro assays with drug-induced liver injury.
Drug-induced liver injury (DILI) is one of the major causes for the discontinuation of drug development and withdrawal of drugs from the market. Since it is known that reactive metabolite formation and being substrates or inhibitors of cytochrome P450s (P450s) are associated with DILI, we systematically investigated the association between human P450 inhibition and DILI. The inhibitory activity of 266 DILI-positive drugs (DILI drugs) and 92 DILI-negative drugs (no-DILI drugs), which were selected from Liver Toxicity Knowledge Base (US Food and Drug Administration), against 8 human P450 forms was assessed using recombinant enzymes and luminescent substrates, and the threshold values... (truncated)

Title: An ensemble learning approach for modeling the systems biology of drug-induced injury.
Drug-induced liver injury (DILI) is an adverse reaction caused by the intake of drugs of common use that produces liver damage. The impact of DILI is estimated to affect around 20 in 100,000 inhabitants worldwide each year. Despite being one of the main causes of liver failure, the pathophysiology and mechanisms of DILI are poorly understood. In the present study, we developed an ensemble learning approach based on different features (CMap gene expression, chemical structures, drug targets) to predict drugs that might cause DILI and gain a better understanding of the mechanisms linked to the adverse reaction.

Title: Novel Approaches for the Treatment of Alzheimer's and Parkinson's Disease.
Neurodegenerative disorders affect around one billion people worldwide. They can arise from a combination of genomic, epigenomic, metabolic, and environmental factors. Aging is the leading risk factor for most chronic illnesses of old age, including Alzheimer's and Parkinson's diseases. A progressive neurodegenerative process and neuroinflammation occur, and no current therapies can prevent, slow, or halt disease progression. To date, no novel disease-modifying therapies have been shown to provide significant benefit for patients who suffer from these devastating disorders. Therefore, early diagnosis and the discovery of new targets and novel therapies are of upmost importance. Neurodegenerative diseases, like in other age-related... (truncated)

MESH:C077073 - nitisinone

Summary:

LLM Prediction Score	0.114 (normalized)
LLM Confidence Score	0.950
Golden Answer (Severity Class)	0.875 (normalized)
Prediction Error	0.761

Retrieved Context:

Title: Diagnostic and Therapeutic Challenges of Hereditary Tyrosinemia Type 1 in Lebanon: A 12-Year Retrospective Review.
Background: Hereditary tyrosinemia type 1 is a rare genetic disorder leading to liver cirrhosis and hepatocellular carcinoma. Few decades ago, dietary measures and ultimately liver transplant constituted the only treatment modalities. Nowadays, early diagnosis and therapy with nitisinone can reverse the clinical picture. In developing countries, diagnostic and therapeutic challenges may affect the outcome of this disease. The choice of the treatment modality may depend on the economic status of each country. Few reports on the long-term outcome of hereditary tyrosinemia type 1 are available from developing and Arab countries. Methods: A retrospective study of charts of Lebanese patients diagnosed... (truncated)

Title: Clinical experience with hepatorenal tyrosinemia from a single Egyptian center.
Although very recently, in Egypt, sick newborn screening has included screening for hepatorenal tyrosinemia, yet, it is not yet included in nationwide neonatal screening and hence diagnosis may be delayed. The aim of this study was to analyze data of all cases presenting with hepatorenal tyrosinemia to the Pediatric Hepatology Unit, Cairo University, Egypt from 2006 to 2019. Data were retrieved from patients' files including age of onset of symptoms, clinical signs, blood counts, liver functions, serum phosphorous, alpha-fetoprotein, succinylacetone and abdominal ultrasound. During this period, 76 patients were diagnosed with hepatorenal tyrosinemia if succinylacetone in dry blood spot was... (truncated)

Title: Oxidative Stress, Glutathione Metabolism, and Liver Regeneration Pathways Are Activated in Hereditary Tyrosinemia Type 1 Mice upon Short-Term Nitisinone Discontinuation.
Hereditary tyrosinemia type 1 (HT1) is an inherited condition in which the body is unable to break down the amino acid tyrosine due to mutations in the fumarylacetoacetate hydrolase (FAH) gene, coding for the final enzyme of the tyrosine degradation pathway. As a consequence, HT1 patients accumulate toxic tyrosine derivatives causing severe liver damage. Since its introduction, the drug nitisinone (NTBC) has offered a life-saving treatment that inhibits the upstream enzyme 4-hydroxyphenylpyruvate dioxygenase (HPD), thereby preventing production of downstream toxic metabolites. However, HT1 patients under NTBC therapy remain unable to degrade tyrosine. To control the disease and side-effects of the... (truncated)

Title: Hereditary Tyrosinemia Type 1 Mice under Continuous Nitisinone Treatment Display Remnants of an Uncorrected Liver Disease Phenotype.
Hereditary tyrosinemia type 1 (HT1) is a genetic disorder of the tyrosine degradation pathway (TIMD) with unmet therapeutic needs. HT1 patients are unable to fully break down the amino acid tyrosine due to a deficient fumarylacetoacetate hydrolase (FAH) enzyme and, therefore, accumulate toxic tyrosine intermediates. If left untreated, they experience hepatic failure with comorbidities involving the renal and neurological system and the development of hepatocellular carcinoma (HCC). Nitisinone (NTBC), a potent inhibitor of the 4-hydroxyphenylpyruvate dioxygenase (HPD) enzyme, rescues HT1 patients from severe illness and death. However, despite its demonstrated benefits, HT1 patients under continuous NTBC therapy are at risk... (truncated)

Title: Mildly elevated succinylacetone and normal liver function in compound heterozygotes with pathogenic and pseudodeficient <i>FAH</i> alleles.
Label="BACKGROUND" NlmCategory="BACKGROUND">A high level of succinylacetone (SA) in blood is a sensitive, specific marker for the screening and diagnosis of hepatorenal tyrosinemia (HT1, MIM 276700). HT1 is caused by mutations in the <i>FAH</i> gene, resulting in deficiency of fumarylacetoacetate hydrolase. HT1 newborns are usually clinically asymptomatic, but have coagulation abnormalities revealing liver dysfunction. Treatment with nitisinone (NTBC) plus dietary restriction of tyrosine and phenylalanine prevents the complications of HT1.

MESH:D008694 - methamphetamine

Summary:

LLM Prediction Score	0.760 (normalized)
LLM Confidence Score	0.990
Golden Answer (Severity Class)	0.0 (normalized)
Prediction Error	0.760

Retrieved Context:

Title: Methamphetamine causes acute hyperthermia-dependent liver damage.
Methamphetamine-induced neurotoxicity has been correlated with damage to the liver but this damage has not been extensively characterized. Moreover, the mechanism by which the drug contributes to liver damage is unknown. This study characterizes the hepatocellular toxicity of methamphetamine and examines if hyperthermia contributes to this liver damage. Livers from methamphetamine-treated rats were examined using electron microscopy and hematoxylin and eosin staining. Methamphetamine increased glycogen stores, mitochondrial aggregation, microvesicular lipid, and hydropic change. These changes were diffuse throughout the hepatic lobule, as evidenced by a lack of hematoxylin and eosin staining. To confirm if these changes were indicative of damage,... (truncated)

Title: Induction of mitochondrial permeability transition (MPT) pore opening and ROS formation as a mechanism for methamphetamine-induced mitochondrial toxicity.
During the past 10 years, the use of methamphetamine (METH) has significantly increased in Iran and around the world. The widespread use of 3,4-methylenedioxymethamphetamine as a recreational drug has been responsible for the incidence of several cases of liver failure in young people. This issue made researchers focus on METH toxicity due to the lack of effective treatment and human health risk assessment. There are several reports showing that its long-term use increases the risk for dopamine depletion, but the toxicity mechanisms of METH in liver are not well understood. Therefore, we aimed to investigate the mitochondrial toxicity mechanisms of... (truncated)

Title: Antibiotics Attenuate Methamphetamine-Induced Hepatotoxicity by Regulating Oxidative Stress and TLR4/MyD88/Traf6 Axis.
Methamphetamine (METH) is a major psychostimulant drug of abuse worldwide, and its neurotoxicity has been studied extensively. In addition to neurotoxicity, METH can also induce hepatotoxicity. The underlying mechanism of intestinal microorganisms in METH-induced hepatotoxicity remains unclear. In this study, mice have received antibiotics intragastrically or PBS once each day for 1 week, followed by METH or saline. The antibiotics attenuated METH-induced hepatotoxicity as evidenced by histopathological observation and biochemical analysis; furthermore, they alleviated METH-induced oxidative stress. The effect of antibiotics on METH-induced hepatotoxicity was investigated using RNA-sequencing (RNA-seq). The RNA-seq results demonstrated that antibiotics could regulate 580 differentially expressed... (truncated)

Title: Chlorogenic and caftaric acids in liver toxicity and oxidative stress induced by methamphetamine.
Methamphetamine intoxication can cause acute hepatic failure. Chlorogenic and caftaric acids are the major dietary polyphenols present in various foods. The aim of this study was to evaluate the protective role of chlorogenic and caftaric acids in liver toxicity and oxidative stress induced by methamphetamine in rats. Thirty-two male albino rats were divided into 4 equal groups. Group 1, which was control group, was injected (i.p) with saline (1 mL/kg) twice a day over seven-day period. Groups 2, 3, and 4 were injected (i.p) with methamphetamine (10 mg/kg) twice a day over seven-day period, where groups 3 and 4 were... (truncated)

Title: Peripheral ammonia as a mediator of methamphetamine neurotoxicity.
Ammonia is metabolized by the liver and has established neurological effects. The current study examined the possibility that ammonia contributes to the neurotoxic effects of methamphetamine (METH). The results show that a binge dosing regimen of METH to the rat increased plasma and brain ammonia concentrations that were paralleled by evidence of hepatotoxicity. The role of peripheral ammonia in the neurotoxic effects of METH was further substantiated by the demonstration that the enhancement of peripheral ammonia excretion blocked the increases in brain and plasma ammonia and attenuated the long-term depletions of dopamine and serotonin typically produced by METH. Conversely, the... (truncated)

MESH:D000077287 - levetiracetam

Summary:

LLM Prediction Score	0.265 (normalized)
LLM Confidence Score	0.990
Golden Answer (Severity Class)	1.0 (normalized)
Prediction Error	0.735

Retrieved Context:

Title: Analysis of the clinical characteristics of the liver injury induced by levetiracetam.
Levetiracetam (LEV) has a low risk of hepatotoxicity due to low liver metabolism. Knowledge regarding the association between LEV exposure and liver injury is based mainly on case reports. The purpose of this study is to summarize the clinical features of LEV-induced liver injury.

Title: A Rare Case of Drug-Induced Liver Injury Caused by Levetiracetam.
Levetiracetam (LEV) is one of the newest antiepileptic drugs available on the market and is frequently used in neurosurgical patients requiring antiepileptic assistance. LEV is mainly excreted by the kidney with minimal hepatic metabolism, so it is considered to have a low liver toxicity. Drug-induced liver injury (DILI) associated with LEV administration is extremely rare, with only eight reported cases. In this report, we describe the case of a 44-year-old man who was admitted because of generalized convulsion, and LEV administration at a dose of 3000 mg/day was started following a diagnosis of status epilepticus. Laboratory values before LEV administration... (truncated)

Title: Levetiracetam-induced transaminitis in a young male with traumatic brain injury.
Levetiracetam is a commonly prescribed antiepileptic drug for seizure prophylaxis in patients with traumatic brain injury (TBI). Levetiracetam metabolism has been reported to be non-dependent on hepatic cytochrome P450 (CYP450) isoenzyme system. Furthermore, levetiracetam and its metabolites are reported to be eliminated from systemic circulation via renal excretion. Therefore, due to its well-known renal clearance mechanism with no dosage adjustments recommended for hepatic impairment, levetiracetam is often chosen as the drug of choice in patients with suspected or ongoing hepatic dysfunction. Furthermore, monitoring of liver enzymes is often not considered to be critical in levetiracetam therapy. However, hepatotoxicity is still... (truncated)

Title: Levetiracetam Liver Injury: A Benign Antiepileptic Agent?
Levetiracetam is a commonly prescribed antiepileptic agent and has rarely been linked to hepatotoxicity. This case describes a patient with drug-induced autoimmune hepatitis secondary to levetiracetam.

Title: Antiepileptic Drugs and Liver Disease.
Acute, symptomatic seizures or epilepsy may complicate the course of hepatic disease. Choosing the most appropriate antiepileptic drug in this setting represents a difficult challenge, as most medications are metabolized by the liver. This article focuses on the acute and chronic treatment of seizures in patients with advanced liver disease and reviews the hepatotoxic potential of specific antiepileptic drugs. Newer antiepileptic drugs without, or with minimal, hepatic metabolism, such as levetiracetam, lacosamide, topiramate, gabapentin, and pregabalin should be used as first-line therapy. Medications undergoing extensive hepatic metabolism, such as valproic acid, phenytoin, and felbamate should be used as drugs of... (truncated)

MESH:D009526 - nialamide

Summary:

LLM Prediction Score	0.268 (normalized)
LLM Confidence Score	0.860
Golden Answer (Severity Class)	1.0 (normalized)
Prediction Error	0.732

Retrieved Context:

Title: Microbiota-Host-Irinotecan Axis: A New Insight Toward Irinotecan Chemotherapy.
Irinotecan (CPT11) and its active metabolite ethyl-10-hydroxy-camptothecin (SN38) are broad-spectrum cytotoxic anticancer agents. Both cause cell death in rapidly dividing cells (e.g., cancer cells, epithelial cells, hematopoietic cells) and commensal bacteria. Therefore, CPT11 can induce a series of toxic side-effects, of which the most conspicuous is gastrointestinal toxicity (nausea, vomiting, diarrhea). Studies have shown that the gut microbiota modulates the host response to chemotherapeutic drugs. Targeting the gut microbiota influences the efficacy and toxicity of CPT11 chemotherapy through three key mechanisms: microbial ecocline, catalysis of microbial enzymes, and immunoregulation. This review summarizes and explores how the gut microbiota participates in... (truncated)

Title: Mechanisms of drug toxicity and relevance to pharmaceutical development.
Toxicity has been estimated to be responsible for the attrition of approximately one-third of drug candidates and is a major contributor to the high cost of drug development, particularly when not recognized until late in clinical trials or post-marketing. The causes of drug toxicity can be classified in several ways and include mechanism-based (on-target) toxicity, immune hypersensitivity, off-target toxicity, and bioactivation/covalent modification. In addition, idiosyncratic responses are rare but can be one of the most problematic issues; several hypotheses for these have been advanced. Although covalent binding of drugs to proteins was described almost 40 years ago, the significance to... (truncated)

Title: Association of CYP1A1 and CYP1B1 inhibition in in vitro assays with drug-induced liver injury.
Drug-induced liver injury (DILI) is one of the major causes for the discontinuation of drug development and withdrawal of drugs from the market. Since it is known that reactive metabolite formation and being substrates or inhibitors of cytochrome P450s (P450s) are associated with DILI, we systematically investigated the association between human P450 inhibition and DILI. The inhibitory activity of 266 DILI-positive drugs (DILI drugs) and 92 DILI-negative drugs (no-DILI drugs), which were selected from Liver Toxicity Knowledge Base (US Food and Drug Administration), against 8 human P450 forms was assessed using recombinant enzymes and luminescent substrates, and the threshold values... (truncated)

Title: Interactions between Food and Drugs, and Nutritional Status in Renal Patients: A Narrative Review.
Drugs and food interact mutually: drugs may affect the nutritional status of the body, acting on senses, appetite, resting energy expenditure, and food intake; conversely, food or one of its components may affect bioavailability and half-life, circulating plasma concentrations of drugs resulting in an increased risk of toxicity and its adverse effects, or therapeutic failure. Therefore, the knowledge of these possible interactions is fundamental for the implementation of a nutritional treatment in the presence of a pharmacological therapy. This is the case of chronic kidney disease (CKD), for which the medication burden could be a problem, and nutritional therapy plays... (truncated)

Title: Therapeutic targeting of CPT-11 induced diarrhea: a case for prophylaxis.
CPT-11 (irinotecan), a DNA topoisomerase I inhibitor is one of the main treatments for colorectal cancer. The main dose limiting toxicities are neutropenia and late onset diarrhea. Though neutropenia is manageable, CPT-11 induced diarrhea is frequently severe, resulting in hospitalizations, dose reductions or omissions leading to ineffective treatment administration. Many potential agents have been tested in preclinical and clinical studies to prevent or ameliorate CPT-11 induced late onset diarrhea. It is predicted that prophylaxis of CPT-11 induced diarrhea will reduce sub-therapeutic dosing as well as hospitalizations and will eventually lead to dose escalations resulting in better response rates. This article... (truncated)

MESH:C467894 - ambrisentan

Summary:

LLM Prediction Score	0.147 (normalized)
LLM Confidence Score	0.960
Golden Answer (Severity Class)	0.875 (normalized)
Prediction Error	0.728

Retrieved Context:

Title: Safety and Efficacy of Ambrisentan-Phosphodiesterase Type 5 (PDE5) Inhibitor Combination Therapy for Japanese Pulmonary Arterial Hypertension Patients in Real-World Clinical Practice.
<i>Background:</i> This retrospective study was conducted to evaluate the safety and efficacy of ambrisentan combination therapy with phosphodiesterase type 5 (PDE5) inhibitors in Japanese patients with pulmonary arterial hypertension (PAH). <i>Methods and Results:</i> PAH patients who received ambrisentan for the first time in combination with a PDE5 inhibitor between January 2013 and the end of August 2015 were included in this study. Adverse drug reaction (ADR) safety analysis, as well as the efficacy analysis focusing on changes in clinical parameters, were investigated for overall cases and cases stratified by patient background. Forty-eight consecutive patients (n=21, 43.8% with idiopathic PAH; male/female, 18/30; average... (truncated)

Title: Hepatotoxicity by bosentan in a patient with portopulmonary hypertension: a case-report and review of the literature.
Bosentan is an endothelin receptor antagonist approved for treatment of pulmonary arterial hypertension. Mild liver reactions occur in about 10% of treated patients but severe hepatotoxicity is rare. We present clinical data and treatment outcome of a severe drug induced liver injury due to bosentan in a patient with non-cirrhotic portopulmonary hypertension. After 18 months of uncomplicated therapy with bosentan 125 mg b.i.d., the patient developed a severe mixed hepatic injury. Serum levels of bilirubin were 316 µmol/l (ref. value <20 micromol/l), AST 14 µkat/l (ref. value < 0.9 µkat/l), ALT 10 µkat/l (ref. value < 0.9 µkat/l), ALP 8... (truncated)

Title: Evaluation of the endothelin receptor antagonists ambrisentan, bosentan, macitentan, and sitaxsentan as hepatobiliary transporter inhibitors and substrates in sandwich-cultured human hepatocytes.
Inhibition of the transporter-mediated hepatobiliary elimination of bile salts is a putative mechanism for liver toxicity observed with some endothelin receptor antagonists (ERAs).

Title: Multiple compound-related adverse properties contribute to liver injury caused by endothelin receptor antagonists.
Drug-induced liver injury has been observed in patients treated with the endothelin receptor antagonists sitaxentan and bosentan, but not following treatment with ambrisentan. The aim of our studies was to assess the possible role of multiple contributory mechanisms in this clinically relevant toxicity. Inhibition of the bile salt export pump (BSEP) and multidrug resistance-associated protein 2 was quantified using membrane vesicle assays. Inhibition of mitochondrial respiration in human liver-derived HuH-7 cells was determined using a Seahorse XF(e96) analyzer. Cytochrome P450 (P450)-independent and P450-mediated cell toxicity was assessed using transfected SV40-T-antigen-immortalized human liver epithelial (THLE) cell lines. Exposure-adjusted assay ratios were... (truncated)

Title: A review of pulmonary arterial hypertension: role of ambrisentan.
Pulmonary arterial hypertension (PAH) is a rare fatal disease. Current disease-specific therapeutic interventions in PAH target 1 of 3 established pathways in disease pathobiology: prostacyclin, nitric oxide, and endothelin-1. Endothelin receptor antagonists (ERAs) act on the endothelin pathway by blocking binding of endothelin-1 to its receptors (endothelin type-A [ET(A)] and/or type-B [ET(B)]) on the surface of endothelial and smooth muscle cells. Ambrisentan is an oral, once-daily, ET(A)-selective ERA in development for the treatment of PAH. In Phase 3 clinical trials in patients with PAH, ambrisentan (2.5-10 mg orally once-daily) improved exercise capacity, Borg dyspnea index, time to clinical worsening, WHO... (truncated)

MESH:D004977 - ethambutol

Summary:

LLM Prediction Score	0.277 (normalized)
LLM Confidence Score	0.990
Golden Answer (Severity Class)	1.0 (normalized)
Prediction Error	0.723

Retrieved Context:

Title: Isoniazid-rifampicin-induced submassive hepatic necrosis.
A 58-year-old woman with tuberculosis received antituberculous drugs which included isoniazid, rifampicin, and ethambutol. Nausea and anorexia were initial symptoms while jaundice and abdominal pain were late manifestations. She became comatose and died 7 weeks after therapy. Autopsy revealed submassive necrosis of the liver and active advanced pulmonary tuberculosis. It is, thus, necessary for the physician to be alert for this serious complication in prescribing a combination of these antituberculous drugs.

Title: Study on hepatotoxicity and other side-effects of antituberculosis drugs.
A prospective study of different side-effects and toxicity of different antituberculosis drugs was made on 125 cases of pulmonary tuberculosis, divided into 3 groups according to the regime of treatment. Group A consisted of 50 patients, taking streptomycin, ethambutol and isoniazid. Group B of 50 patients received streptomycin plus ethambutol plus isoniazid and rifampicin and 25 patients comprising group C received streptomycin plus isoniazid plus ethambutol and pyrazinamide. The group B showed hepatotoxicity in 30% cases, out of which clinical jaundice with abnormal liver function tests being 26% and rest 4% cases were of anicteric hepatitis, while group A showed... (truncated)

Title: Hepatotoxicity caused by the combined action of isoniazid and rifampicin.
A 35 year old black Somalian woman with miliary tuberculosis developed hepatotoxicity after a few days of treatment with isoniazid, rifampicin, pyrazinamide, and ethambutol. After withdrawal of all drugs the liver profile returned to normal and remained so after challenge with isoniazid. Hepatotoxicity recurred when rifampicin was added, but it was well tolerated when reintroduced without isoniazid.

Title: Hepatotoxicity to different antituberculosis drug combinations.
Hepatotoxicity to different combinations of anti-tuberculosis drugs containing, Rifampicin (R), Streptomycin (S), Isoniazid (H), Pyrazinamide (Z) and Myambutol (E) is described in 47 patients who completed 6 to 9 months therapy. Seven cases (15%) showed signs of toxicity and in 4 patients (8.5%) the drugs had to be withdrawn. Two patients developed hepatitis, one with jaundice and the other with fever and deranged liver functions, while others 2 developed severe hypersensitivity reactions. Burning palms, difficulty in micturition, itching and giddiness were complained of by one patient each, which settled in due course without recourse to withdrawal of drugs.

Title: [Incidence of hepatotoxic side effects during antituberculous therapy (INH, RMP, EMB) in relation to the acetylator phenotype (author's transl)].
In 95 patients with active tuberculosis, we investigated in a prospective study the influence of the acetylator phenotype on the hepatotoxic side effects of the antituberculous regimen isoniazid (INH) 10 mg/kg, rifampicin (RMP) 10 mg/kg, and ethambutol (EMB) 25 mg/kg. Besides a much higher incidence of isoniazid hepatitis (SGOT, SGPT greater than 200 U/l) in 12.6% of patients treated--as compared to the incidence reported in large chemoprophylaxis trials with isoniazid monotherapy in the range of 0.5%-1% (IUAT 1969, U.S.P.H.S. 1971)--we observed a significant, higher risk of isoniazid-induced hepatotoxicity in slow acetylators (p less than 0.01): in 26 of 56 slow... (truncated)

MESH:D020123 - sirolimus

Summary:

LLM Prediction Score	0.290 (normalized)
LLM Confidence Score	0.990
Golden Answer (Severity Class)	1.0 (normalized)
Prediction Error	0.710

Retrieved Context:

Title: Sirolimus therapy following early cyclosporine withdrawal in transplant patients: mechanisms of action and clinical results.
Cyclosporine (CsA), a member of the family of calcineurin inhibitors, is a cornerstone of the immunosuppressive treatments used after organ transplantation. However, it exhibits significant toxicity, including nephrotoxicity and increased cardiovascular risk factors. CsA withdrawal has been used as a strategy to improve renal allograft function and other CsA-related toxicities. In order to maintain adequate immunosuppression levels, sirolimus may be used in association with CsA withdrawal. Sirolimus is a member of the mammalian target of rapamycin (mTOR) family. It presents a good immunosuppressive efficacy associated with antiproliferative actions. Early withdrawal of CsA with sirolimus is associated with a significant improvement... (truncated)

Title: Sirolimus-associated hepatotoxicity in liver transplantation.
Sirolimus is an immunosuppressant that exerts anti-rejection activity by inhibiting T-cell activity and is used to treat chronic rejection and calcineurin-related nephrotoxicity. Unlike tacrolimus and cyclosporine, it has no effect on calcineurin activity in liver transplant recipients.

Title: Sirolimus <i>vs</i> tacrolimus: Which one is the best therapeutic option for patients undergoing liver transplantation for hepatocellular carcinoma?
Liver transplantation (LT) withstands as the most preferred therapeutic option for patients afflicted with hepatocellular carcinoma (HCC) and cirrhosis. To improve prognosis post-transplant, as well as to prevent the occurrence of rejection, a life-long immunosuppression strategy is implemented. The following letter to the editor highlights and provides novel evidence from recently published literature on topics discussed within the review article titled "Trends of rapamycin in survival benefits of liver transplantation for hepatocellular carcinoma" in <i>World J Gastrointest Surg</i> 2021; 13: 953-966. In the recent manuscript, the authors compared immunosuppressive drugs such as the newer option first-generation mammalian target of rapamycin... (truncated)

Title: Lack of hepatotoxicity upon sirolimus addition to a calcineurin inhibitor-based regimen in hepatitis virus-positive renal transplant recipients.
We retrospectively analyzed the impact of sirolimus addition (SRL) with a 25% dosing reduction in calcineurin inhibitors on liver function among patients with or without hepatitis B virus (HBV) or hepatitis C virus (HCV) infection.

Title: Comparison of long-term impact of immunosuppressants at therapeutic doses on hepatic function and histological changes in unilateral nephrectomized rats.
Cyclosporine, tacrolimus and sirolimus are commonly used in renal transplant recipients to prevent rejection. Various adverse effects of these agents on the multiple organ system have been reported clinically. However, animal studies are necessary to determine and compare these effects on individual organ given the presence of multiple confounding factors and multi-pharmacy in clinical settings. In a physiologically and clinically relevant rat model of unilateral nephrectomy, the long-term impacts of commonly used immunosuppressants at doses equivalent to the therapeutic levels used for post-renal transplant patients on hepatic function and histological changes of the liver were examined. Cyclosporine induced significant hepatocellular... (truncated)

MESH:D002434 - cefadroxil

Summary:

LLM Prediction Score	0.191 (normalized)
LLM Confidence Score	0.970
Golden Answer (Severity Class)	0.875 (normalized)
Prediction Error	0.684

Retrieved Context:

Title: Identification and Characterization of Cefazolin-Induced Liver Injury.
Cephalosporin antibiotics are popular because they have a broad spectrum of activity and are generally well tolerated; however, cephalosporin-induced liver injury is considered rare. We describe a new syndrome associated with a single intravenous dose of cefazolin and the clinical features of cephalosporin-induced liver injury.

Title: The role of intestinal microflora in anti-inflammatory effect of baicalin in mice.
Baicalin, a main constituent of the rhizome of Scutellaria baicalensis, is metabolized to baicalein and oroxylin A in the intestine before its absorption. To understand the role of intestinal microflora in the pharmacological activities of baicalin, we investigated its anti-inflammatory effect in mice treated with and without antibiotics. Orally administered baicalin showed the anti-inflammatory effect in mice than intraperitoneally treated one, apart from intraperitoneally administered its metabolites, baicalein and oroxylin A, which potently inhibited LPS-induced inflammation. Of these metabolites, oroxylin A showed more potent anti-inflammatory effect. However, treatment with the mixture of cefadroxil, oxytetracycline and erythromycin (COE) significantly attenuated the... (truncated)

Title: Differential Effects of 1α,25-Dihydroxyvitamin D₃ on the Expressions and Functions of Hepatic CYP and UGT Enzymes and Its Pharmacokinetic Consequences In Vivo.
The compound 1α,25-Dihydroxyvitamin D₃ (1,25(OH)₂D₃) is the active form of vitamin D₃ and a representative ligand of the vitamin D receptor (VDR). Previous studies have described the impacts of 1,25(OH)₂D₃ on a small number of cytochrome P450 (CYP) and uridine diphosphate-glucuronyltransferase (UGT) enzymes, but comparatively little is known about interactions between several important CYP and UGT isoforms and 1,25(OH)₂D₃ in vitro and/or in vivo. Thus, we investigated the effects of 1,25(OH)₂D₃ on the gene and protein expressions and functional activities of selected CYPs and UGTs and their impacts on drug pharmacokinetics in rats. The mRNA/protein expressions of Cyp2b1 and Cyp2c11... (truncated)

Title: Application of International Consensus Meeting Criteria for classifying drug-induced liver disorders.
To report a patient with 2 consecutive reversible drug-induced liver disorders and the application of International Consensus Meeting Criteria for the screening and diagnosis of drug-induced liver disorders.

Title: Modulation of gut microbiota mediates berberine-induced expansion of immuno-suppressive cells to against alcoholic liver disease.
Berberine is an isoquinoline alkaloid compound derived from many herbs, which has been used extensively to improve liver function. But action mechanism of its hepatoprotection in alcoholic liver disease (ALD) is far from being clear.

MESH:C013756 - nitrefazole

Summary:

LLM Prediction Score	0.337 (normalized)
LLM Confidence Score	0.780
Golden Answer (Severity Class)	1.0 (normalized)
Prediction Error	0.663

Retrieved Context:

Title: [Tolerance of nitrefazole in alcoholics with liver disease. A 4-week placebo-controlled double-blind study].
Liver damage is one of the most common organ manifestations of chronic alcoholism. The recovery process following abstinence should not be impaired by therapy with alcohol sensitizing drugs. In a double-blind multicentre-study (controlled against placebo) the liver tolerance of Nitrefazole which is indicated as an alcohol sensitizing agent for therapy of alcoholics, was tested during the first four weeks of a planned longterm therapy. A total of 62 patients with alcoholic liver disease--demonstrated clinically and in the laboratory--were tested. The patients received 800 mg of Nitrefazole (4 capsules a 200 mg), respectively 4 placebo capsules of identical appearance, once a... (truncated)

Title: Design strategies and application progress of therapeutic exosomes.
Exosomes have great potential to be drug delivery vehicles due to their natural material transportation properties, intrinsic long-term circulatory capability, and excellent biocompatibility, which are suitable for delivering a variety of chemicals, proteins, nucleic acids, and gene therapeutic agents. However, an effective method of loading specific protein agents into exosomes for absorption by target cells is still lacking. The application potential of exosome is still limited. In this review, we discussed the methods for loading specific treating molecules (proteins, nucleic acids and small chemicals) into exosomes, the design strategies for cell and tissue targeting, and the factors for exosome formation.... (truncated)

MESH:D000086 - acetazolamide

Summary:

LLM Prediction Score	0.342 (normalized)
LLM Confidence Score	0.990
Golden Answer (Severity Class)	1.0 (normalized)
Prediction Error	0.658

Retrieved Context:

Title: Idiosyncratic toxicity associated with potentiated sulfonamides in the dog.
Idiosyncratic toxicity to potentiated sulfonamides occurs in both humans and dogs, with considerable clinical similarities. The syndrome in dogs can consist of fever, arthropathy, blood dyscrasias (neutropenia, thrombocytopenia, or hemolytic anemia), hepatopathy consisting of cholestasis or necrosis, skin eruptions, uveitis, or keratoconjunctivitis sicca. Other manifestations seen less commonly include protein-losing nephropathy, meningitis, pancreatitis, pneumonitis, or facial nerve palsy. The pathogenesis of these reactions is not completely understood, but may be due to a T-cell-mediated response to proteins haptenated by oxidative sulfonamide metabolites. Our laboratory is working on tests to characterize dogs with possible idiosyncratic sulfonamide reactions, to include ELISA for... (truncated)

Title: New Insights Into Diuretic Use to Treat Congestion in the ICU: Beyond Furosemide.
Diuretics are commonly used in critically ill patients with acute kidney injury (AKI) and fluid overload in intensive care units (ICU), furosemide being the diuretic of choice in more than 90% of the cases. Current evidence shows that other diuretics with distinct mechanisms of action could be used with good results in patients with selected profiles. From acetazolamide to tolvaptan, we will discuss recent studies and highlight how specific diuretic mechanisms could help to manage different ICU problems, such as loop diuretic resistance, hypernatremia, hyponatremia, or metabolic alkalosis. The current review tries to shed some light on the potential use... (truncated)

Title: A prospective study on urine alkalization with an oral regimen consisting of sodium bicarbonate and acetazolamide in patients receiving high-dose methotrexate.
Intravenous (IV) sodium bicarbonate is typically used in alkalization regimens for the safe use of the chemotherapeutic agent high-dose methotrexate (HDMTX). Urine parameters including urine output and pH are important in order to minimize the risk of kidney injury, which increases adverse effects and hospital length of stay following HDMTX. IV sodium bicarbonate has been on shortage, and there are limited literature describing the safety of alternative regimens.

Title: Nano-hydroxyapatite improves intestinal absorption of acetazolamide (BCS Class IV drug)-but how?
We earlier reported that coating poorly water-soluble drugs with nano-hydroxyapatite (nano-HAP) improves bioavailability after oral administration. In the present study, we coated BCS Class IV drug acetazolamide (AZ) with nano-HAP (AZ/HAP formulation), and investigated its bioavailability and nano-HAP's role in promoting it. We tested AZ bioavailability after a single oral dose of the AZ/HAP formulation in rats, followed by a series of in vitro, ex vivo and in vivo testing. The binding state of AZ and nano-HAP was analyzed by gel filtration chromatography. AZ permeability was studied using a Caco-2 cell monolayer assay kit, to test for tight junction penetration,... (truncated)

Title: Mortality and drug exposure in a 5-year cohort of patients with chronic liver disease.
Chronic liver diseases are common in the general population. Drug treatment in this group may be challenging, as many drugs are hepatically metabolised and hepatotoxic.

MESH:D005472 - fluorouracil

Summary:

LLM Prediction Score	0.649 (normalized)
LLM Confidence Score	0.990
Golden Answer (Severity Class)	0.0 (normalized)
Prediction Error	0.649

Retrieved Context:

Title: Effectiveness and low toxicity of hepatic artery infusion with fluorouracil and mitomycin for metastatic colorectal cancer confined to the liver. The Swiss Group for Clinical and Epidemiological Cancer Research (SAKK).
The usefulness of hepatic artery infusion (HAI) with floxuridine is limited by the severe biliary and hepatic toxicity of floxuridine. This prompted the SAKK to evaluate the effectiveness, toxicity and feasibility of HAI with fluorouracil (FU) and mitomycin (MMC) administered by an external portable pump. Of 28 patients treated, partial responses were obtained in 14 (50%, 95% confidence interval: 30% to 70%) and stabilization in 11 (39%, 21% to 60%), for a median duration of 12.6+ months. Median survival was 19.5+ months. Grade I-II toxicity (WHO) consisted of nausea (46%), leucopenia (32%) thrombocytopenia (21%) and abdominal discomfort (25%). Two patients... (truncated)

Title: Lethal hepatotoxicity following 5-fluorouracil/cisplatin chemotherapy: a relevant case report.
Some articles have reported severe toxicities induced by cisplatin/5-fluorouracil regimens, nevertheless, severe and lethal liver toxicity has not been previously reported. In this article, we report the case of a 72-year-old woman, who developed fulminant hepatitis, hypoglycemia and hypotension with atrial fibrillation not responding to treatment. After ruling out all other possible causes of hepatitis, the toxicity was more likely attributed to 5-fluorouracil. Genotyping was performed and the patient was found to be a homozygote carrier of the T variant of the MTHFR gene. The patient died two days later. Several factors, including genetic factors, could explain this severe toxicity.... (truncated)

Title: A Rodent Model of Human-Dose-Equivalent 5-Fluorouracil: Toxicity in the Liver, Kidneys, and Lungs.
5-Fluorouracil (5-FU) is a chemotherapy drug widely used to treat a range of cancer types, despite the recurrence of adverse reactions. Therefore, information on its side effects when administered at a clinically recommended dose is relevant. On this basis, we examined the effects of the 5-FU clinical treatment on the integrity of the liver, kidneys, and lungs of rats. For this purpose, 14 male Wistar rats were divided into treated and control groups and 5-FU was administered at 15 mg/kg (4 consecutive days), 6 mg/kg (4 alternate days), and 15 mg/kg on the 14th day. On the 15th day, blood,... (truncated)

Title: Liver toxicity in colorectal cancer patients treated with first-line FOLFIRI-containing regimen: a single institution experience.
Chemotherapy-induced toxic liver injury is a relevant issue in the clinical management of patients affected with metastatic colorectal cancer (mCRC). This retrospective study evaluated patterns of liver toxicity in patients treated with FOLinic acid, Fluorouracil, IRInotecan (FOLFIRI)-based regimens.

Title: Hepatic toxicity associated with fluorouracil plus levamisole adjuvant therapy.
To determine the frequency and nature of hepatic toxicity associated with fluorouracil (5-FU) plus levamisole adjuvant therapy.

MESH:D008784 - methysergide

Summary:

LLM Prediction Score	0.644 (normalized)
LLM Confidence Score	0.960
Golden Answer (Severity Class)	0.0 (normalized)
Prediction Error	0.644

Retrieved Context:

Title: The Traditional Uses, Phytochemistry, Pharmacology, Toxicology, and Clinical Uses of <i>Metagentiana Rhodantha</i> (Franch.) T.N.Ho and S.W.Liu, an Ethnomedicine in Southwest China.
Background: <i>Metagentiana rhodantha</i> (Franch.) T.N.Ho and S.W.Liu (MR) belongs to Gentianales<i>,</i> and it is often called Hong-hua-long-dan in Chinese. Traditionally, it has been used to cure acute icteric hepatitis, sore throat, dysentery, acute gastritis, carbuncle, and furuncle based on traditional Chinese medicine (TCM) concepts. Aim of Study: This review manages to provide a critical and comprehensive analysis on the traditional uses, phytochemistry, pharmacology, toxicology, and clinical uses of MR and to evaluate the therapeutic potential of this plant. Methods: Relevant data mainly literatures on MR were selected from available database. All the papers reviewed provided evidence that the source herbs... (truncated)

Title: Methamphetamine-induced toxicity: an updated review on issues related to hyperthermia.
Reports of methamphetamine-related emergency room visits suggest that elevated body temperature is a universal presenting symptom, with lethal overdoses generally associated with extreme hyperthermia. This review summarizes the available information on methamphetamine toxicity as it pertains to elevations in body temperature. First, a brief overview of thermoregulatory mechanisms is presented. Next, central and peripheral targets that have been considered for potential involvement in methamphetamine hyperthermia are discussed. Finally, future areas of investigation are proposed, as further studies are needed to provide greater insight into the mechanisms that mediate the alterations in body temperature elicited by methamphetamine.

Title: Relationships Between Pharmacovigilance, Molecular, Structural, and Pathway Data: Revealing Mechanisms for Immune-Mediated Drug-Induced Liver Injury.
Immune-mediated drug-induced liver injury (IMDILI) can be devastating, irreversible, and fatal in the absence of successful transplantation surgery. We present a novel approach that combines the methods of pharmacoepidemiology with in silico molecular modeling to identify specific features in toxic ligands that are associated with clinical features of IMDILI. Specifically, from pharmacovigilance data multivariate logistic regression identified 18 drugs associated with IMDILI ($P < 0.00015$). Eleven of these drugs, along with their known and proposed metabolites, constituted a training set used to develop a four-point pharmacophore model (sensitivity 75%; specificity 85%). Subsequently, this information was combined with information from immune-pathway... (truncated)

Title: Serotonergic Regulation of Hepatic Energy Metabolism.
The liver is a vital organ that regulates systemic energy metabolism and many physiological functions. Nonalcoholic fatty liver disease (NAFLD) is the commonest cause of chronic liver disease and end-stage liver failure. NAFLD is primarily caused by metabolic disruption of lipid and glucose homeostasis. Serotonin (5-hydroxytryptamine [5-HT]) is a biogenic amine with several functions in both the central and peripheral systems. 5-HT functions as a neurotransmitter in the brain and a hormone in peripheral tissues to regulate systemic energy homeostasis. Several recent studies have proposed various roles of 5-HT in hepatic metabolism and inflammation using tissue-specific knockout mice and 5-HT-receptor... (truncated)

Title: New Approaches to Shifting the Migraine Treatment Paradigm.
The standard of care paradigm for migraine treatment has been based almost exclusively on approaches that grew out of the happenstance use of market pharmaceuticals. Only methysergide, which has long since been removed from use for safety concerns, the ergotamine family of drugs, and the triptans were explicitly developed with migraine and other vascular headaches in mind. While the forward and innovative thinking to utilize the broad array of agents to treat migraine served millions well, their therapeutic efficacy was often low, and adverse event profiles were troublesome in the least. Advances in biochemical and molecular biology and the application... (truncated)

MESH:C476331 - pralnacasan

Summary:

LLM Prediction Score	0.357 (normalized)
LLM Confidence Score	0.720
Golden Answer (Severity Class)	1.0 (normalized)
Prediction Error	0.643

Retrieved Context:

Title: Novel Antioxidant, Deethylated Ethoxyquin, Protects against Carbon Tetrachloride Induced Hepatotoxicity in Rats by Inhibiting NLRP3 Inflammasome Activation and Apoptosis.
Inflammation and an increase in antioxidant responses mediated by oxidative stress play an important role in the pathogenesis of acute liver injury (ALI). We utilized in silico prediction of biological activity spectra for substances (PASS) analysis to estimate the potential biological activity profile of deethylated ethoxyquin (DEQ) and hypothesized that DEQ exhibits antioxidant and anti-inflammatory effects in a rat model of carbon tetrachloride (CCl₄)-induced ALI. Our results demonstrate that DEQ improved liver function which was indicated by the reduction of histopathological liver changes. Treatment with DEQ reduced CCl₄-induced elevation of gene expression, and the activity of antioxidant enzymes (AEs), as... (truncated)

Title: Death Receptor-Mediated Cell Death and Proinflammatory Signaling in Nonalcoholic Steatohepatitis.
Nonalcoholic fatty liver disease (NAFLD) is becoming public health problem worldwide. A subset of patients develop an inflammatory disease, nonalcoholic steatohepatitis (NASH), characterized by steatosis, hepatocellular death, macrophage and neutrophil accumulation and varying stages of fibrosis. Hepatocyte cell death triggers the cellular inflammatory response and, therefore, reducing cell death may be salutary in the steatohepatitis disease process. Recently, a better understanding of hepatocyte apoptosis in NASH has been obtained and new information regarding other cell death modes, such as necroptosis and pyroptosis, has been reported. Hepatocyte lipotoxicity is often triggered by death receptors. In addition to causing apoptosis, death receptors... (truncated)

Title: NLRP3: a new therapeutic target in alcoholic liver disease.
The liver is in charge of a wide range of critical physiological processes and it plays an important role in activating the innate immune system which elicits the inflammatory events. Chronic ethanol exposure disrupts hepatic inflammatory mechanism and leads to the release of proinflammatory mediators such as chemokines, cytokines and activation of inflammasomes. The mechanism of liver fibrosis/cirrhosis involve activation of NLRP3 inflammasome, leading to the destruction of hepatocytes and subsequent metabolic dysregulation in humans. In addition, increasing evidence suggests that alcohol intake significantly modifies liver epigenetics, promoting the development of alcoholic liver disease (ALD). Epigenetic changes including histone modification,... (truncated)

Title: Intervention with a caspase-1 inhibitor reduces obesity-associated hyperinsulinemia, non-alcoholic steatohepatitis and hepatic fibrosis in LDLR-/-.Leiden mice.
Non-alcoholic steatohepatitis (NASH) is a serious liver condition, closely associated with obesity and insulin resistance. Recent studies have suggested an important role for inflammasome/caspase-1 in the development of NASH, but the potential therapeutic value of caspase-1 inhibition remains unclear. Therefore, we aimed to investigate the effects of caspase-1 inhibition in the ongoing disease process, to mimic the clinical setting.

Title: Focus on the Role of NLRP3 Inflammasome in Diseases.
Inflammation is a protective reaction activated in response to detrimental stimuli, such as dead cells, irritants or pathogens, by the evolutionarily conserved immune system and is regulated by the host. The inflammasomes are recognized as innate immune system sensors and receptors that manage the activation of caspase-1 and stimulate inflammation response. They have been associated with several inflammatory disorders. The NLRP3 inflammasome is the most well characterized. It is so called because NLRP3 belongs to the family of nucleotide-binding and oligomerization domain-like receptors (NLRs). Recent evidence has greatly improved our understanding of the mechanisms by which the NLRP3 inflammasome is... (truncated)

MESH:D015773 - enalaprilat

Summary:

LLM Prediction Score	0.232 (normalized)
LLM Confidence Score	0.950
Golden Answer (Severity Class)	0.875 (normalized)
Prediction Error	0.643

Retrieved Context:

Title: Evaluation of angiotensin converting enzyme (ACE) in the pharmacokinetics and pharmacodynamics of ACE inhibitors.
The increasing number of angiotensin converting enzyme (ACE) inhibitors means that compounds with different enzyme kinetics, pharmacokinetics, bioavailability, and pharmacodynamics will appear. They will, however, all inhibit ACE, and their hypotensive effect will be a consequence of this action. Enalapril (MK-421) is an esterified prodrug, which in man is converted by the liver to the bioactive potent ACE inhibitor enalaprilate (enalaprilic acid, MK-422). This probably accounts for the slower plasma appearance of MK-422 and the longer duration of action of enalapril. The clinical significance of deesterification by the liver needs further study but minor abnormalities of liver function, such as... (truncated)

Title: Drug interaction study of flavonoids toward OATP1B1 and their 3D structure activity relationship analysis for predicting hepatoprotective effects.
Organic anion transporting polypeptide 1B1 (OATP1B1), a liver-specific uptake transporter, was associated with drug induced liver injury (DILI). Screening and identifying potent OATP1B1 inhibitors with little toxicity is of great value in reducing OATP1B1-mediated DILI. Flavonoids are a group of polyphenols ubiquitously present in vegetables, fruits and herbal products, some of them were reported to produce transporter-mediated DDI. Our objective was to investigate potential inhibitors of OATP1B1 from 99 flavonoids, and to assess the hepatoprotective effects on bosentan induced liver injury. Eight flavonoids, including biochanin A, hispidulin, isoliquiritigenin, isosinensetin, kaempferol, licochalcone A, luteolin and sinensetin exhibited significant inhibition (>50 %)... (truncated)

Title: TJ-M2010-5, A self-developed MyD88 inhibitor, attenuates liver fibrosis by inhibiting the NF-κB pathway.
Liver fibrosis is the result of most chronic inflammatory liver damage and seriously endangers human health. However, no drugs have been approved to treat this disease. Previous studies showed that the Toll-like receptors (TLRs)/myeloid differentiation factor-88 (MyD88)/nuclear factor-κB (NF-κB) pathway plays a key role in liver fibrosis. TJ-M2010-5 is a self-developed small molecule MyD88 inhibitor, which has been proven to have a good protective effect in a variety of inflammatory disease models. In the present study, to investigate the anti-fibrotic effect of TJ-M2010-5, mice were injected with carbon tetrachloride (CCl₄) in vivo and LX2 cells (a human hepatic stellate cell... (truncated)

Title: Evaluating the Sensitivity and Specificity of Promising Circulating Biomarkers to Diagnose Liver Injury in Humans.
Early diagnosis of drug-induced liver injury (DILI) continues to be a major hurdle during drug development and postmarketing. The objective of this study was to evaluate the diagnostic performance of promising biomarkers of liver injury-glutamate dehydrogenase (GLDH), cytokeratin-18 (K18), caspase-cleaved K18 (ccK18), osteopontin (OPN), macrophage colony-stimulating factor (MCSF), MCSF receptor (MCSFR), and microRNA-122 (miR-122) in comparison to the traditional biomarker alanine aminotransferase (ALT). Biomarkers were evaluated individually and as a multivariate model in a cohort of acetaminophen overdose (n = 175) subjects and were further tested in cohorts of healthy adults (n = 135), patients with liver damage from various... (truncated)

Title: Effect of trichloroacetaldehyde on the activation of CD4⁺T cells in occupational medicamentosa-like dermatitis: An in vivo and in vitro study.
Occupational medicamentosa-like dermatitis induced by trichloroethylene (OMLDT) is a hypersensitivity disease with autoimmune liver injury, which has increasingly become a serious occupational health problem in China. However, the pathogenesis of OMLDT remained undefined. In this study, 30 TCE-induced OMLDT patients, 58 exposure controls, and 40 non-exposure controls were recruited. We showed that the ratio of activated CD4⁺ T cells (downregulation of CD62 L) was dramatically increased in OMLDT patients compared to exposure and non-exposure control, suggesting that CD4⁺ T cells activation was a key cellular event in the development of OMLDT. In parallel, the expression of cytokine including IL-2, IFN-γ, TNF-α... (truncated)

MESH:D000077563 - norethindrone acetate

Summary:

LLM Prediction Score	0.638 (normalized)
LLM Confidence Score	0.960
Golden Answer (Severity Class)	0.0 (normalized)
Prediction Error	0.638

Retrieved Context:

Title: [Experience in the treatment of metastatic breast carcinoma with norethisterone acetate in a clinical study].
Twenty-nine patients with progressive advanced carcinoma of the breast were treated with 40 mg/day norethisterone acetate orally in this clinical trial. 27 of 29 patients were evaluable for response to the drug. One patient achieved a partial response (duration 2 months), 6 had a stable disease (median duration 7.6 months) and 20 developed a progressive disease on treatment. There was a high rate of liver toxicity in 23 patients [grade 3 and 4 according to WHO (1)]. The literature on norethisterone acetate is reviewed and compared with present results. The role of progestogenic agents in treatment of advanced mammary carcinoma... (truncated)

Title: Uses of progesterone in clinical practice.
Progesterone is the natural progestagen produced by the corpus luteum during the luteal phase. It is absorbed when administered orally, but is greater than 90% metabolized during the first hepatic pass. This greatly limits the efficacy of once-daily administration and also results in unphysiologically high levels of progesterone metabolites, particularly those reduced at the 5-a position. These metabolites can cause dizziness and drowsiness to the point of preventing the operation of a motor vehicle. Synthetic progestins, such as medroxyprogesterone acetate and norethindrone acetate (NETA), have been specifically designed to resist enzymatic degradation and remain active after oral administration. However, these... (truncated)

Title: Thromboembolic safety of norethisterone vs levonorgestrel in combined oral contraceptive users: a pooled analysis of 4 large prospective cohort studies.
Norethisterone (acetate) and levonorgestrel are marketed globally as components of combined oral contraceptives. Although guidelines recommend both as first-line combined oral contraceptives, no direct, comparative safety studies are available.

Title: Relugolix/Estradiol/Norethisterone (Norethindrone) Acetate: A Review in Symptomatic Uterine Fibroids.
An oral fixed-dose combination of relugolix/estradiol/norethisterone (also known as norethindrone) acetate (Ryeqo®; Myfembree®) has been approved for the management of heavy menstrual bleeding associated with uterine fibroids in the USA and management of moderate to severe symptoms of uterine fibroids in the EU. Relugolix is a gonadotropin releasing hormone (GnRH) receptor antagonist that decreases serum estradiol and progesterone concentrations to postmenopausal levels. The addition of estradiol/norethisterone acetate to relugolix ameliorates relugolix-induced bone loss and hot flush. In the two phase 3 LIBERTY trials, relugolix + estradiol/norethisterone substantially decreased menstrual bleeding and improved a range of other uterine fibroid symptoms in... (truncated)

Title: A Randomized Open-Label Study of Relugolix Alone or Relugolix Combination Therapy in Premenopausal Women.
Relugolix is a gonadotropin-releasing hormone receptor antagonist. Relugolix 40-mg monotherapy is associated with vasomotor symptoms and long-term bone mineral density loss due to hypoestrogenism. This study assessed whether the addition of estradiol (E2) 1 mg and norethindrone acetate (NETA) 0.5 mg to relugolix 40 mg (relugolix combination therapy) provides systemic E2 concentrations in the 20-50 pg/mL range to minimize these undesirable effects.

MESH:C100190 - mepazine

Summary:

LLM Prediction Score	0.363 (normalized)
LLM Confidence Score	0.840
Golden Answer (Severity Class)	1.0 (normalized)
Prediction Error	0.637

Retrieved Context:

Title: Identification of New Toxicity Mechanisms in Drug-Induced Liver Injury through Systems Pharmacology.
Among adverse drug reactions, drug-induced liver injury presents particular challenges because of its complexity, and the underlying mechanisms are still not completely characterized. Our knowledge of the topic is limited and based on the assumption that a drug acts on one molecular target. We have leveraged drug polypharmacology, i.e., the ability of a drug to bind multiple targets and thus perturb several biological processes, to develop a systems pharmacology platform that integrates all drug-target interactions. Our analysis sheds light on the molecular mechanisms of drugs involved in drug-induced liver injury and provides new hypotheses to study this phenomenon.

Title: A human immune dysregulation syndrome characterized by severe hyperinflammation with a homozygous nonsense Roquin-1 mutation.
Hyperinflammatory syndromes are life-threatening disorders caused by overzealous immune cell activation and cytokine release, often resulting from defects in negative feedback mechanisms. In the quintessential hyperinflammatory syndrome familial hemophagocytic lymphohistiocytosis (HLH), inborn errors of cytotoxicity result in effector cell accumulation, immune dysregulation and, if untreated, tissue damage and death. Here, we describe a human case with a homozygous nonsense R688* RC3H1 mutation suffering from hyperinflammation, presenting as relapsing HLH. RC3H1 encodes Roquin-1, a posttranscriptional repressor of immune-regulatory proteins such as ICOS, OX40 and TNF. Comparing the R688* variant with the murine M199R variant reveals a phenotypic resemblance, both in immune... (truncated)

Title: Roquin--a multifunctional regulator of immune homeostasis.
Roquin-1 (Rc3h1) is an E3 ubiquitin ligase originally discovered in a mutational screen for genetic factors contributory to systemic lupus erythematosus-like symptoms in mice. A single base-pair mutation in the Rc3h1 gene resulted in the manifestation of autoantibody production and sustained immunological inflammation characterized by excessive T follicular helper cell activation and formation of germinal centers. Subsequent studies have uncovered a multifactorial process by which Roquin-1 contributes to the maintenance of immune homeostasis. Through its interactions with partner proteins, Roquin-1 targets mRNAs for decay with inducible costimulator being a primary target. In this review, we discuss newly discovered functions of... (truncated)

MESH:C058702 - benziodarone

Summary:

LLM Prediction Score	0.364 (normalized)
LLM Confidence Score	0.820
Golden Answer (Severity Class)	1.0 (normalized)
Prediction Error	0.636

Retrieved Context:

Title: Investigating bile acid-mediated cholestatic drug-induced liver injury using a mechanistic model of multidrug resistance protein 3 (MDR3) inhibition.
Inhibition of the canalicular phospholipid floppase multidrug resistance protein 3 (MDR3) has been implicated in cholestatic drug-induced liver injury (DILI), which is clinically characterized by disrupted bile flow and damage to the biliary epithelium. Reduction in phospholipid excretion, as a consequence of MDR3 inhibition, decreases the formation of mixed micelles consisting of bile acids and phospholipids in the bile duct, resulting in a surplus of free bile acids that can damage the bile duct epithelial cells, i.e., cholangiocytes. Cholangiocytes may compensate for biliary increases in bile acid monomers <i>via</i> the cholehepatic shunt pathway or bicarbonate secretion, thereby influencing viability or... (truncated)

Title: Attenuation of LPS-Induced Lung Injury by Benziodarone via Reactive Oxygen Species Reduction.
As overproduction of reactive oxygen species (ROS) causes various diseases, antioxidants that scavenge ROS, or inhibitors that suppress excessive ROS generation, can be used as therapeutic agents. From a library of approved drugs, we screened compounds that reduced superoxide anions produced by pyocyanin-stimulated leukemia cells and identified benzbromarone. Further investigation of several of its analogues showed that benziodarone possessed the highest activity in reducing superoxide anions without causing cytotoxicity. In contrast, in a cell-free assay, benziodarone induced only a minimal decrease in superoxide anion levels generated by xanthine oxidase. These results suggest that benziodarone is an inhibitor of NADPH oxidases... (truncated)

Title: Cheminformatics analysis of assertions mined from literature that describe drug-induced liver injury in different species.
Drug-induced liver injury is one of the main causes of drug attrition. The ability to predict the liver effects of drug candidates from their chemical structures is critical to help guide experimental drug discovery projects toward safer medicines. In this study, we have compiled a data set of 951 compounds reported to produce a wide range of effects in the liver in different species, comprising humans, rodents, and nonrodents. The liver effects for this data set were obtained as assertional metadata, generated from MEDLINE abstracts using a unique combination of lexical and linguistic methods and ontological rules. We have analyzed... (truncated)

Title: Classification of Cholestatic and Necrotic Hepatotoxicants Using Transcriptomics on Human Precision-Cut Liver Slices.
Human toxicity screening is an important stage in the development of safe drug candidates. Hepatotoxicity is one of the major reasons for the withdrawal of drugs from the market because the liver is the major organ involved in drug metabolism, and it can generate toxic metabolites. There is a need to screen molecules for drug-induced hepatotoxicity in humans at an earlier stage. Transcriptomics is a technique widely used to screen molecules for toxicity and to unravel toxicity mechanisms. To date, the majority of such studies were performed using animals or animal cells, with concomitant difficulty in interpretation due to species... (truncated)

Title: Association of CYP1A1 and CYP1B1 inhibition in in vitro assays with drug-induced liver injury.
Drug-induced liver injury (DILI) is one of the major causes for the discontinuation of drug development and withdrawal of drugs from the market. Since it is known that reactive metabolite formation and being substrates or inhibitors of cytochrome P450s (P450s) are associated with DILI, we systematically investigated the association between human P450 inhibition and DILI. The inhibitory activity of 266 DILI-positive drugs (DILI drugs) and 92 DILI-negative drugs (no-DILI drugs), which were selected from Liver Toxicity Knowledge Base (US Food and Drug Administration), against 8 human P450 forms was assessed using recombinant enzymes and luminescent substrates, and the threshold values... (truncated)

MESH:C040550 - tolrestat

Summary:

LLM Prediction Score	0.368 (normalized)
LLM Confidence Score	0.770
Golden Answer (Severity Class)	1.0 (normalized)
Prediction Error	0.632

Retrieved Context:

Title: Novel role for epalrestat: protecting against NLRP3 inflammasome-driven NASH by targeting aldose reductase.
Nonalcoholic steatohepatitis (NASH) is a progressive and inflammatory subtype of nonalcoholic fatty liver disease (NAFLD) characterized by hepatocellular injury, inflammation, and fibrosis in various stages. More than 20% of patients with NASH will progress to cirrhosis. Currently, there is a lack of clinically effective drugs for treating NASH, as improving liver histology in NASH is difficult to achieve and maintain through weight loss alone. Hence, the present study aimed to investigate potential therapeutic drugs for NASH.

Title: Mechanisms of drug toxicity and relevance to pharmaceutical development.
Toxicity has been estimated to be responsible for the attrition of approximately one-third of drug candidates and is a major contributor to the high cost of drug development, particularly when not recognized until late in clinical trials or post-marketing. The causes of drug toxicity can be classified in several ways and include mechanism-based (on-target) toxicity, immune hypersensitivity, off-target toxicity, and bioactivation/covalent modification. In addition, idiosyncratic responses are rare but can be one of the most problematic issues; several hypotheses for these have been advanced. Although covalent binding of drugs to proteins was described almost 40 years ago, the significance to... (truncated)

Title: A compound attributes-based predictive model for drug induced liver injury in humans.
Drug induced liver injury (DILI) is one of the key safety concerns in drug development. To assess the likelihood of drug candidates with potential adverse reactions of liver, we propose a compound attributes-based approach to predicting hepatobiliary disorders that are routinely reported to US Food and Drug Administration (FDA) Adverse Event Reporting System (FAERS). Specifically, we developed a support vector machine (SVM) model with recursive feature extraction, based on physicochemical and structural properties of compounds as model input. Cross validation demonstrates that the predictive model has a robust performance with averaged 70% of both sensitivity and specificity over 500 trials.... (truncated)

Title: Comprehensive Evaluation of Organotypic and Microphysiological Liver Models for Prediction of Drug-Induced Liver Injury.
Drug-induced liver injury (DILI) is a major concern for the pharmaceutical industry and constitutes one of the most important reasons for the termination of promising drug development projects. Reliable prediction of DILI liability in preclinical stages is difficult, as current experimental model systems do not accurately reflect the molecular phenotype and functionality of the human liver. As a result, multiple drugs that passed preclinical safety evaluations failed due to liver toxicity in clinical trials or postmarketing stages in recent years. To improve the selection of molecules that are taken forward into the clinics, the development of more predictive <i>in vitro</i>... (truncated)

Title: Association of CYP1A1 and CYP1B1 inhibition in in vitro assays with drug-induced liver injury.
Drug-induced liver injury (DILI) is one of the major causes for the discontinuation of drug development and withdrawal of drugs from the market. Since it is known that reactive metabolite formation and being substrates or inhibitors of cytochrome P450s (P450s) are associated with DILI, we systematically investigated the association between human P450 inhibition and DILI. The inhibitory activity of 266 DILI-positive drugs (DILI drugs) and 92 DILI-negative drugs (no-DILI drugs), which were selected from Liver Toxicity Knowledge Base (US Food and Drug Administration), against 8 human P450 forms was assessed using recombinant enzymes and luminescent substrates, and the threshold values... (truncated)

MESH:C011301 - metaxalone

Summary:

LLM Prediction Score	0.000 (normalized)
LLM Confidence Score	0.970
Golden Answer (Severity Class)	0.625 (normalized)
Prediction Error	0.625

Retrieved Context:

Title: Characterization of hospitalized patients who received naloxone while receiving opioids with or without gabapentinoids.
Gabapentin and pregabalin (gabapentinoids) can be given with opioids for opioid-sparing and adjuvant analgesic effects. In the context of certain comorbidities and high dosages, coadministration of these agents can lead to respiratory depression or oversedation, necessitating naloxone administration.

Title: Forensic Aspects of a Fatal Intoxication Involving Acetaminophen, Citalopram and Trazodone: A Case Report.
We report the case of a young man, a former heroin addict, found dead at home by the Police Forces in an advanced state of decomposition. Numerous blisters and unpacked tablets of medications were found all over the bed and on the floor of the room. Multiple injuries to the face, left arm and neck of the deceased were noted. The latter damages were attributed to post-mortem dog bites, since no indications of a possible defense against the animal were observed. The autopsy findings were unremarkable. Toxicological investigations performed on peripheral blood and urine by gas chromatography-mass spectrometry (GC-MS) technique... (truncated)

Title: Population-Based Signals of Antidepressant Drug Interactions Associated With Unintentional Traumatic Injury.
Antidepressants are very widely used and associated with traumatic injury, yet little is known about their potential for harmful drug interactions. We aimed to identify potential drug interaction signals by assessing concomitant medications (precipitant drugs) taken with individual antidepressants (object drugs) that were associated with unintentional traumatic injury. We conducted pharmacoepidemiologic screening of 2000-2015 Optum Clinformatics data, identifying drug interaction signals by performing self-controlled case series studies for antidepressant + precipitant pairs and injury. We included persons aged 16-90 years codispensed an antidepressant and ≥ 1 precipitant drug(s), with an injury during antidepressant therapy. We classified antidepressant person-days as either... (truncated)

Title: The Pharmacological Treatment of Chronic Pain: From Guidelines to Daily Clinical Practice.
In agreement with the International Association for the Study of Pain, chronic pain is an unpleasant sensory and emotional experience associated with actual or potential tissue damage. To date, there are several types of pain: nociceptive, neuropathic, and nociplastic. In the present narrative review, we evaluated the characteristics of the drugs used for each type of pain, according to guidelines, and their effects in people with comorbidity to reduce the development of severe adverse events.

Title: Resting state connectivity correlates with drug and placebo response in fibromyalgia patients.
Fibromyalgia is a chronic pain syndrome characterized by widespread pain, fatigue, and memory and mood disturbances. Despite advances in our understanding of the underlying pathophysiology, treatment is often challenging. New research indicates that changes in functional connectivity between brain regions, as can be measured by magnetic resonance imaging (fcMRI) of the resting state, may underlie the pathogenesis of this and other chronic pain states. As such, this parameter may be able to be used to monitor changes in brain function associated with pharmacological treatment, and might also be able to predict treatment response. We performed a resting state fcMRI trial... (truncated)

MESH:C044815 - etonogestrel

Summary:

LLM Prediction Score	0.000 (normalized)
LLM Confidence Score	0.980
Golden Answer (Severity Class)	0.625 (normalized)
Prediction Error	0.625

Retrieved Context:

Title: Advances in contraception: vaginal contraceptive rings.
The vaginal contraceptive ring is very effective and user dependent. In this article, we will discuss the different types of vaginal contraceptive rings, namely, the etonogestrel/ethinyl estradiol (ENG/EE) ring (NuvaRing, Merck, Rahway, NJ, USA) and the segesterone acetate (SA)/EE (Annovera, Mayne Pharma, Raleigh, NC, USA) ring. The details of dosing and administration, indications, advantages, disadvantages, and cost-effectiveness are presented. This literature review was conducted using PubMed and Google Scholar. The search terms included 'vaginal contraceptive ring', 'etonogestrel/ethinyl estradiol ring', and 'segesterone acetate/ethinyl estradiol ring'. The search was then sorted by year from 2000 until present, and the most recent articles... (truncated)

Title: Decreased efficacy of an etonogestrel implant in a woman on antiepileptic medications: a case report.
Many antiepileptic drugs decrease the efficacy of combined hormonal contraceptives due to their inducing effect on cytochrome P450 liver metabolism. Less is known about the pharmacokinetics and outcomes of concomitant use of the etonogestrel implant and hepatic enzyme-inducing medications.

Title: Retrospective Analysis of the Effectiveness and Reversibility of Long-Acting Contraception Etonogestrel (Implanon®) inCommon Marmosets (<i>Callithrix jacchus</i>).
Contraception is an important population control method for the colony management of primates housed in captivity. Etonogestrel (ENG) implants (i.e., Implanon®) are a widely used progestin-based contraceptive in common marmosets (<i>Callithrix jacchus</i>) with the theoretical advantages of being reversible and long-acting. However, no dose and efficacy data are available yet. Therefore, data from 52 adult female marmosets contracepted with ENG (one-fourth or one-third of an implant) housed at the Biomedical Primate Research Centre (BPRC, Rijswijk, The Netherlands) over the past 18 years were analyzed. Using an electronic database, a retrospective longitudinal cohort study was conducted to calculate the reproductive data... (truncated)

Title: Esters of levonorgestrel and etonogestrel intended as single, subcutaneous-injection, long-lasting contraceptives.
An effort with the goal of discovering single-dose, long-lasting (>6 months) injectable contraceptives began using levonorgestrel (LNG)-17-β esters linked to a sulfonamide function purposed as human carbonic anhydrase II (hCA 2) ligands. One single analog from this first series showed noticeably superior anti-ovulatory activity in murine models, and a subsequent structure-activity relationship (SAR, the relationship between a compound's molecular structure and its biological activity) study based on this compound identified a LNG-phenoxyacetic acid ester analog exhibiting longer anti-ovulatory properties using the murine model at 2 and 4 mg dose than medroxyprogesterone acetate (MPA). The same ester function linked to etonogestrel... (truncated)

Title: Relationship Between Etonogestrel Concentrations and Bleeding Patterns in Contraceptive Implant Users.
To estimate whether serum etonogestrel concentrations influence bleeding patterns and related side effects in contraceptive implant users.

MESH:D000078764 - milnacipran

Summary:

LLM Prediction Score	0.250 (normalized)
LLM Confidence Score	0.970
Golden Answer (Severity Class)	0.875 (normalized)
Prediction Error	0.625

Retrieved Context:

Title: Antidepressants and Hepatotoxicity: A Cohort Study among 5 Million Individuals Registered in the French National Health Insurance Database.
Hepatotoxicity may be a concern when prescribing antidepressants. Nevertheless, this risk remains poorly understood for serotonin and noradrenaline reuptake inhibitors (SNRIs: venlafaxine, milnacipran, duloxetine) and 'other antidepressants' (mianserin, mirtazapine, tianeptine and agomelatine), particularly in comparison with selective serotonin reuptake inhibitors (SSRIs: fluoxetine, citalopram, paroxetine, sertraline, fluvoxamine, escitalopram), which are by far the most commonly prescribed antidepressants.

Title: Duloxetine: a review of its safety and efficacy in the management of fibromyalgia syndrome.
Fibromyalgia (FM) is a chronic disorder characterized by widespread pain and other associated symptoms including fatigue, insomnia, cognitive/memory problems, and even psychological distress. Duloxetine is one of three FDA approved medications (the other two being milnacipran and pregabalin) for the treatment of FM. It has been demonstrated that FM patients possess low central nervous system levels of serotonin and norepinephrine. Duloxetine, which is classified pharmacologically as a serotonin-norepinephrine reuptake inhibitor (SNRI), may be beneficial for FM patients by increasing these levels. This review will touch briefly upon the pathophysiology of FM, diagnostic tools, currently available therapeutic options (both pharmacologic and... (truncated)

Title: Efficacy and tolerability of milnacipran: an overview.
The relative benefits and risks of milnacipran, a novel antidepressant which selectively inhibits the reuptake of serotonin and noradrenaline, have been evaluated in comparative trials against tricyclic antidepressants (TCAs) or selective serotonin reuptake inhibitors (SSRIs). A total of 2462 patients with major depressive disorders have been investigated. At the optimal dose (50 mg twice a day), the efficacy of milnacipran was equivalent to that of the TCAs, with response rates of approximately 65% in both cases. Milnacipran was consistently effective against all of the principal elements of depression (anxiety, cognitive function, sleep and psychomotor retardation), and did not produce sedation... (truncated)

Title: Levomilnacipran for the treatment of major depressive disorder: a review.
Levomilnacipran (LVM, Fetzima(®)) was recently approved by the US Food and Drug Administration for the treatment of major depressive disorder. It is a unique dual neurotransmitter reuptake inhibitor. In contrast with other selective serotonin norepinephrine reuptake inhibitors, including duloxetine, venlafaxine, and desvenlafaxine, it has greater selectivity for inhibiting norepinephrine reuptake than serotonin reuptake. Our review focuses on the efficacy, safety, and tolerability data for five double-blind, placebo-controlled, short-term studies and two long-term studies. In the short-term studies, LVM was found to be more effective than placebo in reducing depression (Montgomery-Åsberg Depression Rating Scale) scores as well as improving functional impairment... (truncated)

Title: Gut microbe-derived milnacipran enhances tolerance to gut ischemia/reperfusion injury.
There are significant differences in the susceptibility of populations to intestinal ischemia/reperfusion (I/R), but the underlying mechanisms remain elusive. Here, we show that mice exhibit significant differences in susceptibility to I/R-induced enterogenic sepsis. Notably, the milnacipran (MC) content in the enterogenic-sepsis-tolerant mice is significantly higher. We also reveal that the pre-operative fecal MC content in cardiopulmonary bypass patients, including those with intestinal I/R injury, is associated with susceptibility to post-operative gastrointestinal injury. We reveal that MC attenuates mouse I/R injury in wild-type mice but not in intestinal epithelial aryl hydrocarbon receptor (AHR) gene conditional knockout mice (AHR^{flox/flox}) or IL-22 gene... (truncated)

MESH:C451734 - etravirine

Summary:

LLM Prediction Score	0.389 (normalized)
LLM Confidence Score	0.980
Golden Answer (Severity Class)	1.0 (normalized)
Prediction Error	0.611

Retrieved Context:

Title: [Safety and tolerability of etravirine].
Etravirine (ETR) is the first representative of a new generation of non-nucleoside reverse transcriptase inhibitors (NNRTI) and is indicated in patients with HIV infection and virological failure. The recommended dose is 200 mg (two tablets) every 12 hours after a meal. ETR has good tolerability and the tablets can be dissolved in water, which can aid swallowing in some patients. This drug has a plasma half-life of 30-40 hours and consequently is a candidate for once-daily regimens. The most frequent adverse effect is rash (affecting 19% of patients), which is usually mild (grades 1 or 2) and does not lead... (truncated)

Title: [Role of etravirine in combination antiretroviral therapy].
Etravirine (ETR) is a new antiretroviral drug of the non-nucleoside reverse transcriptase inhibitor (NNRTI) family that has recently been approved by the regulatory agencies for the treatment of patients with prior experience with antiretrovirals, evidence of active viral replication, and who harbor multidrug resistant HIV-1 strains. In this context, in Europe, the use of this drug has been authorized combined with boosted protease inhibitors and nucleoside reverse transcriptase inhibitors. This approval was based on the results of the randomized double-blind DUET studies, in which the ETR arm was statistically superior to the placebo arms in terms of virological efficacy, immunological... (truncated)

Title: Liver toxicity in HIV-infected patients receiving novel second-generation nonnucleoside reverse transcriptase inhibitors etravirine and rilpivirine.
There are few data on the hepatic safety profile of the novel second-generation nonnucleoside reverse transcriptase inhibitors etravirine and rilpivirine. Previous extensive studies including other drugs of the same class, nevirapine and efavirenz, have shown an incidence of liver toxicity of 3-20%, higher in the case of nevirapine. The pathogenic mechanisms involved are related to hypersensitivity, as described with nevirapine, impaired metabolism and therefore increased drug levels, and direct toxic effects with production of toxic metabolites. Hepatitis C coinfection seems to be the most important factor for toxicity, especially in the case of advanced liver fibrosis. Etravirine showed a similar... (truncated)

Title: [Etravirine in highly treatment-experienced patients].
Etravirine (ETR) has demonstrated efficacy in patients with multiple prior treatments with prior virological failure and resistance mutations to various families of antiretroviral drugs. Most of the evidence concerning this drug has been drawn from the DUET studies, consisting of two multicenter, randomized, double-blind clinical trials with identical designs that included 1,200 patients. These trials showed that ETR obtained a superior virological and immunological response to placebo, reducing the incidence of hospital admissions and progression to AIDS/death. The most frequent adverse effect was rash, which was generally mild to moderate and required treatment discontinuation in only 2%. There were no... (truncated)

Title: Liver toxicity and risk of discontinuation in HIV/hepatitis C virus-coinfected patients receiving an etravirine-containing antiretroviral regimen: influence of liver fibrosis.
The aim of the study was to establish the risk of liver toxicity in HIV/hepatitis C virus (HCV)-coinfected patients receiving etravirine, according to the degree of liver fibrosis.

MESH:D000643 - ammonium chloride

Summary:

LLM Prediction Score	0.596 (normalized)
LLM Confidence Score	0.990
Golden Answer (Severity Class)	0.0 (normalized)
Prediction Error	0.596

Retrieved Context:

Title: <i>Commiphora molmol</i> Modulates Glutamate-Nitric Oxide-cGMP and Nrf2/ARE/HO-1 Pathways and Attenuates Oxidative Stress and Hematological Alterations in Hyperammonemic Rats.
Hyperammonemia is a serious complication of liver disease and may lead to encephalopathy and death. This study investigated the effects of <i>Commiphora molmol</i> resin on oxidative stress, inflammation, and hematological alterations in ammonium chloride- (NH₄Cl-) induced hyperammonemic rats, with an emphasis on the glutamate-NO-cGMP and Nrf2/ARE/HO-1 signaling pathways. Rats received NH₄Cl and <i>C. molmol</i> for 8 weeks. NH₄Cl-induced rats showed significant increase in blood ammonia, liver function markers, and tumor necrosis factor-alpha (TNF-<i>α</i>). Concurrent supplementation of <i>C. molmol</i> significantly decreased circulating ammonia, liver function markers, and TNF-<i>α</i> in hyperammonemic rats. <i>C. molmol</i> suppressed lipid peroxidation and nitric oxide and enhanced... (truncated)

Title: Peripheral ammonia as a mediator of methamphetamine neurotoxicity.
Ammonia is metabolized by the liver and has established neurological effects. The current study examined the possibility that ammonia contributes to the neurotoxic effects of methamphetamine (METH). The results show that a binge dosing regimen of METH to the rat increased plasma and brain ammonia concentrations that were paralleled by evidence of hepatotoxicity. The role of peripheral ammonia in the neurotoxic effects of METH was further substantiated by the demonstration that the enhancement of peripheral ammonia excretion blocked the increases in brain and plasma ammonia and attenuated the long-term depletions of dopamine and serotonin typically produced by METH. Conversely, the... (truncated)

Title: Hepatic injury is associated with cell cycle arrest and apoptosis with alteration of cyclin A and D1 in ammonium chloride-induced hyperammonemic rats.
Hyperammonemia is considered to be central to the pathophysiology of hepatic encephalopathy in patients exhibiting hepatic failure (HF). It has previously been determined that hyperammonemia is a serious metabolic disorder commonly observed in patients with HF. However, it is unclear whether hyperammonemia has a direct adverse effect on hepatic cells or serves as a cause and effect of HF. The present study investigated whether hepatic injury is caused by hyperammonemia, and aimed to provide an insight into the causes and mechanisms of HF. Hyperammonemic rats were established via intragastric administration of ammonium chloride solution. Hepatic tissues were assessed using biochemistry,... (truncated)

Title: Ammonia-induced mitochondrial impairment is intensified by manganese co-exposure: relevance to the management of subclinical hepatic encephalopathy and cirrhosis-associated brain injury.
Hepatic encephalopathy (HE) is a neuropsychiatric syndrome ensuing from liver failure. The liver is the major site of ammonia detoxification in the human body. Hence, acute and chronic liver dysfunction can lead to hyperammonemia. Manganese (Mn) is a trace element incorporated in several physiological processes in the human body. Mn is excreted through bile. It has been found that cirrhosis is associated with hyperammonemia as well as body Mn accumulation. The brain is the primary target organ for both ammonia and Mn toxicity. On the other hand, brain mitochondria impairment is involved in the mechanism of Mn and ammonia neurotoxicity.

Title: [The (15N) ammonium test in clinical research].
By use of the [15N]-ammoniumtest the liver function is investigated under influence of hormonal contraceptives in women and in liver diseases in children. With the described noninvasive nonradioactive isotope test the ammonia detoxification capability and the urea synthesis capacity of the liver is determined by measuring of the 15N-exceretion in ammonia and urea in the urine after oral administering of [15N]-ammonium chloride. The [15N]-ammonium test shows a significant influence of the hormonal contraceptives on the liver function and gives diagnostic evidence for liver diseases in children.

MESH:C004624 - clomacran

Summary:

LLM Prediction Score	0.408 (normalized)
LLM Confidence Score	0.630
Golden Answer (Severity Class)	1.0 (normalized)
Prediction Error	0.592

Retrieved Context:

DB does not contain articles for query 'clomacran liver toxicity'. Provide an educated estimation as an expert.

MESH:D014147 - tramadol

Summary:

LLM Prediction Score	0.286 (normalized)
LLM Confidence Score	0.990
Golden Answer (Severity Class)	0.875 (normalized)
Prediction Error	0.589

Retrieved Context:

Title: [Polypharmacy and pain treatment].
Pain is a frequent symptom in clinical practice. Elderly and chronically ill patients are particularly affected. On account of the high prevalence of polypharmacy among these patients, pharmacological pain therapy becomes a challenge for physicians. Drug side effects and drug-drug interactions have to be taken into account so as to minimize the health risk for these patients. Especially the group of NSAID has a high risk of adverse drug reactions and drug interactions. The gastrointestinal, the cardiovascular, the renal and the coagulation system are particularly affected. Except for the toxic effect on the liver (in a high dose) Paracetamol (acetaminophen)... (truncated)

Title: Significant Efficacy of Tramadol/Acetaminophen in Elderly Patients with Chronic Low Back Pain Uncontrolled by NSAIDs: An Observational Study.
Chronic low back pain (LBP) is a common condition and is generally treated using non-steroidal anti-inflammatory drug (NSAID); however, chronic NSAID use can decrease renal function. Tramadol, a weak opioid agonist, may improve chronic LBP and disability, while avoiding adverse effects such as gastrointestinal and renal toxicity. However, few studies have evaluated the short-term efficacy of opioids in Asian patients with chronic LBP. In this study, 24 patients with chronic LBP unresponsive to NSAIDs (10 men, 14 women; mean age, 65.1 ± 12.1 years) were prescribed tramadol/acetaminophen (37.5 mg/325 mg; four tablets daily) for 1 month. Then, the following parameters... (truncated)

Title: Analgesics in patients with hepatic impairment: pharmacology and clinical implications.
The physiological changes that accompany hepatic impairment alter drug disposition. Porto-systemic shunting might decrease the first-pass metabolism of a drug and lead to increased oral bioavailability of highly extracted drugs. Distribution can also be altered as a result of impaired production of drug-binding proteins or changes in body composition. Furthermore, the activity and capacity of hepatic drug metabolizing enzymes might be affected to various degrees in patients with chronic liver disease. These changes would result in increased concentrations and reduced plasma clearance of drugs, which is often difficult to predict. The pharmacology of analgesics is also altered in liver disease.... (truncated)

Title: Biochemical and neurotransmitters changes associated with tramadol in streptozotocin-induced diabetes in rats.
The incidence of diabetes is increasing worldwide. Chronic neuropathic pain occurs in approximately 25% of diabetic patients. Tramadol, an atypical analgesic with a unique dual mechanism of action, is used in the management of painful diabetic neuropathy. It acts on monoamine transporters to inhibit the reuptake of norepinephrine (NE), serotonin (5-HT), and dopamine (DA). The purpose of this study was to evaluate the effects of diabetes on the brain neurotransmitter alterations induced by tramadol in rats, and to study the hepatic and renal toxicities of the drug. Eighty Sprague-Dawley rats were divided randomly into two sets: the normal set and... (truncated)

Title: Pharmacological aspects of successful long-term analgesia.
Persistent pain represents a major quality-of-life burden for patients and a challenge for their physician. Chronic pain often arises from multiple tissue sources and involves multiple chemical mediators and pain transmission pathways. Successful long-term pain management requires analgesic regimens that can treat pains of multiple origin and type. Safety and tolerability are also a high priority when prescribing chronic therapy. Recent publications and regulatory developments affecting anti-inflammatory drugs have limited the options available for the management of chronic pain. Major concerns in long-term use of anti-inflammatory drugs include renal toxicity, gastrointestinal ulceration and bleeding and cardiovascular events, which can be... (truncated)

MESH:D017308 - etodolac

Summary:

LLM Prediction Score	0.412 (normalized)
LLM Confidence Score	0.950
Golden Answer (Severity Class)	1.0 (normalized)
Prediction Error	0.588

Retrieved Context:

Title: Glucuronidation of drugs and drug-induced toxicity in humanized UDP-glucuronosyltransferase 1 mice.
UDP-glucuronosyltransferases (UGTs) are phase II drug-metabolizing enzymes that catalyze glucuronidation of various drugs. Although experimental rodents are used in preclinical studies to predict glucuronidation and toxicity of drugs in humans, species differences in glucuronidation and drug-induced toxicity have been reported. Humanized UGT1 mice in which the original Ugt1 locus was disrupted and replaced with the human UGT1 locus (hUGT1 mice) were recently developed. In this study, acyl-glucuronidations of etodolac, diclofenac, and ibuprofen in liver microsomes of hUGT1 mice were examined and compared with those of humans and regular mice. The kinetics of etodolac, diclofenac, and ibuprofen acyl-glucuronidation in hUGT1 mice... (truncated)

Title: Pharmacokinetic profile of etodolac in special populations.
The pharmacokinetics of etodolac in healthy normal volunteers has been extensively studied and is well described. Etodolac is characterised by a high oral bioavailability, low clearance, a small volume of distribution, and a 7-hour half-life. It is essentially completely metabolised, therefore little is excreted unchanged. Etodolac is highly protein bound. To investigate the effect of disease states or concomitant drug administration on a patient's response to etodolac, additional pharmacokinetic studies were carried out in special populations. Since etodolac has a well-defined pharmacokinetic-pharmacodynamic relationship, measurement of pharmacokinetic parameters is clinically relevant. Data from studies to date show that disease states, underlying... (truncated)

Title: Liver injury from nonsteroidal anti-inflammatory drugs in the United States.
Nonsteroidal anti-inflammatory drugs (NSAIDs) are commonly used and have been associated with hepatotoxicity. Studies of liver injury from NSAIDs have been retrospective and prospective data are lacking. The aim was to report the features and outcomes of the subjects with severe drug-induced liver injury from NSAIDS.

Title: Acarbose Use and Liver Injury in Diabetic Patients With Severe Renal Insufficiency and Hepatic Diseases: A Propensity Score-Matched Cohort Study.
Background: Acarbose has been deemed contraindicated in diabetic patients with chronic kidney disease (CKD) or end-stage renal disease (ESRD), but such use is not uncommon. We tested whether this concept hold true in this population with different background hepatic diseases. Methods: All incident diabetic patients (<i>n</i> = 2,036,531) with stage 5 CKD/ESRD were enrolled from Taiwan between 2017 and 2013 and divided into those without chronic liver disease (CLD), with CLD but without cirrhosis, and those with cirrhosis. Among each group, acarbose users, defined as cumulative use >30 days within the preceding year, were propensity-score matched 1:2 to non-users. Our... (truncated)

Title: Comparison of Clinical Effectiveness and Safety of Newer Nonsteroidal Anti-inflammatory Drugs in Patients of Osteoarthritis of Knee Joint: A Randomized, Prospective, Open-label Parallel-group Study.
Osteoarthritis (OA) is a chronic progressive degenerative disease of weight-bearing joints and the leading cause of disability in elderly. Current medical management of OA is mostly palliative with nonsteroidal anti-inflammatory drugs (NSAIDs) being the mainstay of therapy. Reports of gastrointestinal adverse effects with traditional NSAIDs and cardiovascular adverse effects associated with selective cyclooxygenase-2 (COX-2) inhibitors have prompted the hunt for a better NSAID with no or minimal adverse effects. This study compares the clinical effectiveness and safety of newer NSAIDS etodolac and lornoxicam to diclofenac which has been a standard therapy in patients of OA of knee joint.

MESH:C430592 - ixabepilone

Summary:

LLM Prediction Score	0.289 (normalized)
LLM Confidence Score	0.890
Golden Answer (Severity Class)	0.875 (normalized)
Prediction Error	0.586

Retrieved Context:

Title: A phase II trial of trastuzumab plus weekly ixabepilone and carboplatin in patients with HER2-positive metastatic breast cancer: an Eastern Cooperative Oncology Group Trial.
The epothilone B analogue, ixabepilone, binds to b-tubulin, is effective for taxane-refractory metastatic breast cancer (MBC), and may be given every 3 weeks or weekly. We evaluated the efficacy of weekly ixabepilone (I) plus trastuzumab (T) and carboplatin (C) as first line therapy in HER2 + MBC. Patients with HER2+ (3+ by IHC or FISH amplified) MBC received I (15 mg/m2 IV) and C (area under the curve, AUC = 2 IV) on days 1, 8, and 15 of a 28-day cycle for a maximum of 6 cycles, plus weekly T (4 mg/kg loading dose then 2 mg/kg IV) during... (truncated)

Title: Novel microtubule-targeting agents - the epothilones.
Epothilones are a new class of antimicrotubule agents currently in clinical trials. Their chemical structures are distinct from taxanes and are more amenable to synthetic modification. Six epothilones have been studied in preclinical and clinical trials: patupilone (epothilone B), ixabepilone (BMS247550), BMS 310705, sagopilone (ZK-EPO), KOS-862 (epothilone D), and KOS-1584. In vitro data have shown increased potency in taxane-sensitive and taxane-resistant cancer cell lines. This enhanced cytotoxic effect has been attributed to epothilone being a poor substrate for p-glycoprotein drug resistance protein and having high affinity to the various beta tubulin isoforms. Phase I clinical data have shown different dose-limiting... (truncated)

Title: Phase II trial of ixabepilone administered daily for five days in children and young adults with refractory solid tumors: a report from the children's oncology group.
Ixabepilone is a microtubule-stabilizing agent with activity in adult solid tumors and in pediatric tumor xenograft models that are resistant to paclitaxel. The maximum tolerated dose on the daily-for-5-days i.v. schedule was 6 mg/m(2)/dose in adults and 8 mg/m(2)/dose in children, and the primary dose-limiting toxicity (DLT) was neutropenia. This study aimed to determine the response rate to ixabepilone in six solid tumor strata in children and young adults.

Title: PhytoNanotechnology: Enhancing Delivery of Plant Based Anti-cancer Drugs.
Natural resources continue to be an invaluable source of new, novel chemical entities of therapeutic utility due to the vast structural diversity observed in them. The quest for new and better drugs has witnessed an upsurge in exploring and harnessing nature especially for discovery of antimicrobial, antidiabetic, and anticancer agents. Nature has historically provide us with potent anticancer agents which include vinca alkaloids [vincristine (VCR), vinblastine, vindesine, vinorelbine], taxanes [paclitaxel (PTX), docetaxel], podophyllotoxin and its derivatives [etoposide (ETP), teniposide], camptothecin (CPT) and its derivatives (topotecan, irinotecan), anthracyclines (doxorubicin, daunorubicin, epirubicin, idarubicin), and others. In fact, half of all the anti-cancer... (truncated)

Title: Ixabepilone: a new chemotherapeutic option for refractory metastatic breast cancer.
Taxane therapy is commonly used in the treatment of metastatic breast cancer. However, most patients will eventually become refractory to these agents. Ixabepilone is a newly approved chemotherapeutic agent for the treatment of metastatic breast cancer. Although it targets microtubules similarly to docetaxel and paclitaxel, ixabepilone has activity in patients that are refractory to taxanes. This review summarizes the pharmacology of ixapebilone and clinical trials with the drug both as a single agent and in combination. Data were obtained using searches of PubMed and abstracts of the annual meetings of the American Society of Clinical Oncology and the San Antonio... (truncated)

MESH:D014223 - triamterene

Summary:

LLM Prediction Score	0.044 (normalized)
LLM Confidence Score	0.960
Golden Answer (Severity Class)	0.625 (normalized)
Prediction Error	0.581

Retrieved Context:

Title: [Pharmacokinetics of triamterene in healthy subjects and patients with liver and kidney function disorders].
The knowledge about the pharmacokinetics of triamterene (TA) was limited until recently. The metabolic pathway of TA is the formation of p-hydroxytriamterene (OH-TA), which is subsequently conjugated with active sulfate to form p-hydroxytriamterene sulfuric acid ester (OH-TA-ester). The phase-II-metabolite is surprisingly pharmacologically active. TA and its metabolites were measured concomitantly by a specific and sensitive tlc-method. The i.v. kinetics of TA were determined after application of a newly developed lactic acid solution of the drug. Comparing these data with results after oral application of TA the bioavailability of TA was 52% and the extent of absorption 83%. The bioavailability of... (truncated)

Title: Pharmacokinetics of triamterene.
The knowledge about the pharmacokinetics of triamterene (TA) was limited until recently. The metabolic pathway of TA is the formation of p-hydroxytriamterene (OH-TA), which is subsequently conjugated with active sulfate to form p-hydroxytriamterene sulfuric acid ester (OH-TA-ester). The phase-II-metabolite is surprisingly pharmacologically active. TA and its metabolites were measured by a specific and sensitive tlc-method concomitantly. The i.v. kinetics of TA were determined after application of a newly developed lactic acid solution of the drug. Comparing these data with results after oral application of TA the bioavailability of TA was 52% and the extent of absorption 83%. The bioavailability of... (truncated)

Title: Nilvadipine. A review of its pharmacodynamic and pharmacokinetic properties, therapeutic use in hypertension and potential in cerebrovascular disease and angina.
Nilvadipine, a calcium antagonist of the dihydropyridine class, selectively blocks calcium channels in vascular smooth muscle. Compared with nifedipine, the prototype of the dihydropyridines, nilvadipine has a longer duration of action. The antihypertensive efficacy of nilvadipine appears to be comparable with that of nicardipine and nitrendipine, enalapril and captopril and hydrochlorothiazide/triamterene, although further clinical experience is required to establish the claimed advantages nilvadipine may have over the other dihydropyridine derivatives currently used to treat hypertension. Preliminary studies suggest that nilvadipine may also be useful in the treatment of patients with stable exertional or variant angina. Studies conducted in Japan indicate... (truncated)

Title: Drug-induced interstitial nephritis as a result of sodium valproate and triamterene.
we presented a case of drug-induced interstitial nephritis in a 43-year-old woman, having the history of renal calculi, as a result of sodium valproate and triamterene. Renal biopsy was used to confirm the diagnosis, however, due to poor prognosis along with discontinuation of drugs, she was treated with corticosteroids.

Title: Hulk-Like Urine: A Case of Green Urine Caused by Flupirtine Intoxication.
Acute intoxications are common causes of admission to the Emergency Department (ED). Flupirtine is a non-opioid analgesic, originally used for acute and chronic pain. Because of several reports of severe liver toxicity, its use was limited to acute pain in 2013 by the European Medicines Agency. Although withdrawn from the European market in March 2018, there are still flupirtine tablets in many households, and most people are unaware of the hazards they might be facing. A 58-year-old man was admitted to the ED after a suicide attempt with 1 g of flupirtine. He was lethargic and confused but presented no... (truncated)

MESH:D000077610 - bexarotene

Summary:

LLM Prediction Score	0.441 (normalized)
LLM Confidence Score	0.950
Golden Answer (Severity Class)	1.0 (normalized)
Prediction Error	0.559

Retrieved Context:

Title: OAB-14, a bexarotene derivative, improves Alzheimer's disease-related pathologies and cognitive impairments by increasing β-amyloid clearance in APP/PS1 mice.
The pathogenesis of Alzheimer's disease (AD) is complex, though the clinical failures of anti-AD candidates targeting Aβ production (such as β- and γ-secretase inhibitors) make people suspect the Aβ hypothesis, in which the neurotoxicity of Aβ is undoubtedly involved. According to studies, >95% of AD patients with sporadic AD are primarily associated with abnormal Aβ clearance. Therefore, drugs that increase Aβ clearance are becoming new prospects for the treatment of AD. Here, the novel small molecule OAB-14, designed using bexarotene as the lead compound, significantly alleviated cognitive impairments in amyloid precursor protein (APP)/presenilin 1 (PS1) transgenic mice after administration for... (truncated)

Title: Rescuing effects of RXR agonist bexarotene on aging-related synapse loss depend on neuronal LRP1.
Apolipoprotein E (apoE) plays a critical role in maintaining synaptic integrity by transporting cholesterol to neurons through the low-density lipoprotein receptor related protein-1 (LRP1). Bexarotene, a retinoid X receptor (RXR) agonist, has been reported to have potential beneficial effects on cognition by increasing brain apoE levels and lipidation. To investigate the effects of bexarotene on aging-related synapse loss and the contribution of neuronal LRP1 to the pathway, forebrain neuron-specific LRP1 knockout (nLrp1(-/-)) and littermate control mice were administered with bexarotene-formulated diet (100mg/kg/day) or control diet at the age of 20-24 months for 8 weeks. Upon bexarotene treatment, levels of brain... (truncated)

Title: Phase I/II study of the oral retinoid X receptor agonist bexarotene in Japanese patients with cutaneous T-cell lymphomas.
Safety, tolerability, pharmacokinetics and efficacy of bexarotene, a novel retinoid X receptor (RXR)-selective retinoid, were evaluated in Japanese patients with stage IIB-IVB and relapsed/refractory stage IB-IIA cutaneous T-cell lymphomas (CTCL). This study was conducted as a multicenter, open-label, historically controlled, single-arm phase I/II study. Bexarotene was p.o. administrated once daily at a dose of 300 mg/m² for 24 weeks in 13 patients, following an evaluation of safety and tolerability for 4 weeks at a dose of 150 mg/m² in three patients. Eight of 13 patients (61.5%) with an initial dose of 300 mg/m² met the response criteria using the modified... (truncated)

Title: Chemotherapy induced liver abnormalities: an imaging perspective.
Treating patients undergoing chemotherapy who display findings of liver toxicity, requires a solid understanding of these medications. It is important for any clinician to have an index of suspicion for liver toxicity and be able to recognize it, even on imaging. Cancer chemotherapy has evolved, and newer medications that target cell biology have a different pattern of liver toxicity and may differ from the more traditional cytotoxic agents. There are several hepatic conditions that can result and keen clinical as well as radiographic recognition are paramount. Conditions such as sinusoidal obstructive syndrome, steatosis, and pseudocirrhosis are more commonly associated with... (truncated)

Title: Drug screening identifies tazarotene and bexarotene as therapeutic agents in multiple sulfatase deficiency.
Multiple sulfatase deficiency (MSD, MIM #272200) results from pathogenic variants in the SUMF1 gene that impair proper function of the formylglycine-generating enzyme (FGE). FGE is essential for the posttranslational activation of cellular sulfatases. MSD patients display reduced or absent sulfatase activities and, as a result, clinical signs of single sulfatase disorders in a unique combination. Up to date therapeutic options for MSD are limited and mostly palliative. We performed a screen of FDA-approved drugs using immortalized MSD patient fibroblasts. Recovery of arylsulfatase A activity served as the primary readout. Subsequent analysis confirmed that treatment of primary MSD fibroblasts with tazarotene... (truncated)

MESH:D008787 - metoclopramide

Summary:

LLM Prediction Score	0.071 (normalized)
LLM Confidence Score	0.990
Golden Answer (Severity Class)	0.625 (normalized)
Prediction Error	0.554

Retrieved Context:

Title: Ondansetron--the first of a new class of antiemetic agents.
The chemistry, pharmacokinetics, adverse effects, stability, compatibility, and dosage of ondansetron hydrochloride are described, and clinical studies of the use of ondansetron for the prophylaxis of nausea and vomiting induced by antineoplastic therapy are reviewed. Ondansetron hydrochloride is a specific antagonist of serotonin type 3 (5-HT3) receptors, both in the chemoreceptor trigger zone and in the GI tract. Peak plasma concentrations of ondansetron occur approximately one hour after an oral dose and 6 to 20 minutes after an i.v. dose. The mean elimination half-life is approximately 3.5 hours in healthy volunteers, but it is extended in elderly patients (mean of... (truncated)

Title: Case report: metoclopramide induced acute dystonic reaction in adolescent CYP2D6 poor metabolizers.
Metoclopramide is indicated for the management of gastroesophageal reflux, gastric stasis, nausea, and vomiting. Metoclopramide-induced acute dystonic reactions (MIADRs), along with repetitive involuntary protrusion of the tongue, are well-known phenomena in children and young adults that may appear after the first dose. The drug is primarily metabolized via oxidation by the cytochrome P450 enzyme CYP2D6 and to a lesser extent by CYP3A4 and CYP1A2. A recommendation to decrease metoclopramide dosing in patients with severely limited to no CYP2D6 activity (i.e., poor metabolizers, PMs) is included in the drug label. It is important to note, however, that a requirement or recommendation... (truncated)

Title: Pharmacokinetics of high-dose metoclopramide in cancer patients.
The introduction of new cytotoxic drug regimens has been associated with an increase in the incidence and severity of adverse effects. This in turn has highlighted the need for more effective adjuvant therapy. The use of metoclopramide for the prophylaxis of nausea and vomiting, in high intravenous doses (50 to 1000 mg), has become established since 1981. As a lipid-soluble drug, metoclopramide has a large volume of distribution. The reported mean values after high doses range between 2.8 and 4.6 L/kg. The mean values for total body clearance and terminal half-life range from 0.31 to 0.69 L/kg/h and from 4.5... (truncated)

Title: Controlling emesis related to cancer therapy.
Combinations of dopamine antagonists or high-dose metoclopramide with steroids can provide complete control of chemotherapy-related nausea and vomiting in up to 60-70% of patients undergoing high-dose cisplatin-based chemotherapy. High-dose metoclopramide probably acts as a 5-HT3 receptor antagonist, but because of its dopamine-receptor antagonism it is the cause of extrapyramidal side-effects. These compounds, and the agents used in combination with them, tend to cause sedation, an undesirable effect in the outpatient setting. Specific 5-HT3 receptor antagonists (ondansetron, granisetron, tropisetron) give a similar control of chemotherapy related nausea and vomiting, with minimum side-effects. These drugs can cause headaches and constipation and some... (truncated)

Title: A case of probable esomeprazole-induced transient liver injury in a pregnant woman with hyperemesis.
We report a case of 22-year-old primigravida presented to Women's Hospital - Hamad Medical Corporation emergency with severe epigastric pain, nausea, and vomiting. On admission, she was dehydrated with remarkably worsening symptoms. Laboratory findings revealed significantly elevated liver enzymes with unknown etiology. Her past medical history showed an admission for nausea and vomiting 3 weeks previously and she was discharged on antiemetics, and esomeprazole for the first time. Due to the predominantly elevated liver enzymes, the clinical pharmacist discussed the possibility of esomeprazole-induced adverse effects and suggested to suspend esomeprazole based on the evidence from literature review. The liver enzymes... (truncated)

MESH:D002701 - chloramphenicol

Summary:

LLM Prediction Score	0.548 (normalized)
LLM Confidence Score	0.990
Golden Answer (Severity Class)	0.0 (normalized)
Prediction Error	0.548

Retrieved Context:

Title: Liver.
The liver's unique metabolism and relationship to the gastrointestinal tract make it an important target of the toxicity of drugs and xenobiotics. The developmental changes that occur in the liver's metabolic activity from birth to adolescence contribute to the varied sensitivity to toxins seen in the pediatric population. Hepatic drug metabolism, often with an imbalance between the generation of toxic metabolites and detoxification processes, can influence the degree of hepatotoxicity. The decreased capacity of the neonatal liver to metabolize, detoxify, and excrete xenobiotics explains the prolonged action of drugs such as phenobarbital, theophyline, and phenytoin. The reduced capacity of glucuronide... (truncated)

Title: [A case of severe methacycline damage to the liver and bone marrow].
A case of a male patient with bronchopneumonia incorrectly treated for a long time with methacycline (rondomycin), an oxytetracycline drug, is reported. methacycline was applied in a dose of 8 capsules daily (2 capsules 4 times) in the course of 2 1/2 months, the total dose amounting to about 150 g. The patient developed severe toxic hepatitis as a result of this incorrect treatment. The hepatitis was manifested by jaundice and cytolysis. The bone marrow was also affected--hypoplasia marked by combined depression of leuko-, erythro- and thrombopoiesis and peripheral pancytopenia. In addition chloramphenicol treatment was applied which increased the toxic... (truncated)

Title: Effect of phenobarbital on chloramphonicol-induced toxicity in rat liver and small intestine.
The aim of the present study is to determine the effect of Chloramphenicol (CAP) on rat liver and small intestine. Effect of phenobarbital (PB) on CAP toxicity was also investigated.

Title: [Clinical and laboratory studies with typhoid fever 178 patients].
One hundred and seventy-eight cases of typhoid patients were studied on clinical and bacteriological aspects. The main clinical findings were as follows: (1) Most of the cases had sustained fever (66.3%). (2) Gastroenterial symptoms developed as the disesase progressed. (3) Rose spots were found in 32.6% of them. (4) Liver and spleen were enlarged in 69.5% of the cases. (5) Blood eosinophil disappeared in most of the patients and leukopenia was noted in 94.3%. (6) There were toxic hepatitis (47.1%), toxic myocarditis (22.4%) and intestinal hemorrhage (19.7%) as complications. In the drug sensitivity test, the number of ampicillin-resistant and chloramphenicol-resistant... (truncated)

Title: Tetracyclines, chloramphenicol, erythromycin, and clindamycin.
Tetracyclines are active in vitro against most urinary tract pathogens, Chlamydia, Mycoplasma pneumoniae, Brucella, rickettsiae, and Nocardia. Chloramphenicol is used primarily for anaerobic infections, Haemophilus influenzae meningitis, and infections due to Salmonella typhi. Erythromycin is active in vitro against M. pneumoniae, Streptococcus pneumoniae, and group A beta-hemolytic streptococci. Erythromycin may be used as prophylactic therapy for subacute bacterial endocarditis and for recurrence of acute rheumatic fever in patients who are allergic to penicillin. Clindamycin should be used only for the treatment of anaerobic infections. Tetracycline may cause gastrointestinal upset; phototoxic dermatitis; hepatitis, especially in pregnant females; discoloration of teeth and... (truncated)

MESH:D000077404 - cidofovir

Summary:

LLM Prediction Score	0.079 (normalized)
LLM Confidence Score	0.990
Golden Answer (Severity Class)	0.625 (normalized)
Prediction Error	0.546

Retrieved Context:

Title: [Adverse ocular drug reactions of systemic antimicrobial agents].
Antimicrobial therapy can cause adverse ocular drug reactions. They are most often noticed by changes of the eyes' anterior segments or by pain and visual disturbances. It is important that physicians but also patients are watchful for the symptoms and know about their potential dangerous consequences because the chance for reversibility may depend on their early detection. During therapy with voriconazol about one third of patients complain of visual disturbances soon after the first doses but symptoms generally resolve after a short period of time without sequelae. Telithromycin may impair accommodation due to its anticholinergic activity. Neuropathies of the optic... (truncated)

Title: A Case of Adenovirus Viremia in a Pediatric Liver Transplant Recipient With Neutropenia and Lymphopenia: Who and When Should We Treat?
Human adenovirus (HAdV) is one of the most feared infections among immunocompromised patients. In particular, in liver transplant patients, HAdV has been implicated in acute liver failure with resultant mortality. The development of current molecular techniques and surveillance testing protocols have provided tools for early detection of HAdV infection, prior to or at the early onset of HAdV disease. Although reduction in immune suppression is the mainstay of therapy, many researchers have also advocated for early administration of antiviral therapy. In multiple reports, cidofovir treatment has been associated with declines in HAdV viral loads or clinical improvement in solid organ... (truncated)

Title: In vitro activity of potential anti-poxvirus agents.
The potential use of variola or another orthopoxvirus such as monkeypox as a weapon of bioterrorism has stimulated efforts to develop new drugs for treatment of smallpox or other poxvirus infections. At the present time only cidofovir is approved for use in the emergency treatment of smallpox outbreaks. Although cidofovir is very active against the orthopoxviruses in vitro and in animal model infections, it is not active when given orally and must be administered with precaution so as to avoid renal toxicity. In an attempt to identify alternative treatment modalities for these infections we have determined the anti-poxvirus activity in... (truncated)

Title: NPP-669, a Novel Broad-Spectrum Antiviral Therapeutic with Excellent Cellular Uptake, Antiviral Potency, Oral Bioavailability, Preclinical Efficacy, and a Promising Safety Margin.
DNA viruses are responsible for many diseases in humans. Current treatments are often limited by toxicity, as in the case of cidofovir (CDV, Vistide), a compound used against cytomegalovirus (CMV) and adenovirus (AdV) infections. CDV is a polar molecule with poor bioavailability, and its overall clinical utility is limited by the high occurrence of acute nephrotoxicity. To circumvent these disadvantages, we designed nine CDV prodrug analogues. The prodrugs modulate the polarity of CDV with a long sulfonyl alkyl chain attached to one of the phosphono oxygens. We added capping groups to the end of the alkyl chain to minimize β-oxidation... (truncated)

Title: New drug on the horizon for treating adenovirus.
Human adenoviruses can cause serious disseminated infections including death in immunosuppressed patients, especially pediatric allogeneic hematopoietic stem cell transplant (allo-HSCT) patients. There are no drugs approved to treat such infections. Cidofovir is used intravenously in many transplant clinics, probably with some effect, but controlled trials have not been completed. Cidofovir is an acyclic nucleoside phosphonate analog of cytidine monophosphate. Following conversion to its diphosphate form within cells, cidofovir is a preferred substrate for the adenovirus DNA polymerase, leading to viral DNA chain termination. Problems with cidofovir include poor cellular uptake and nephrotoxicity. Brincidofovir, a lipid-linked derivative of cidofovir which is... (truncated)

MESH:D007190 - indapamide

Summary:

LLM Prediction Score	0.082 (normalized)
LLM Confidence Score	0.940
Golden Answer (Severity Class)	0.625 (normalized)
Prediction Error	0.543

Retrieved Context:

Title: Indapamide-Induced Rhabdomyolysis: An Evaluation of Case Reports in VigiBase Using the Bradford Hill Criteria.
Indapamide can cause hypokalaemia and hyponatraemia. Rhabdomyolysis associated with these electrolyte abnormalities has been reported.

Title: Serum binding of indapamide in health and disease: primary role of alpha 1-acid glycoprotein.
The serum concentrations of alpha-1-acid glycoprotein (AAG), albumin (HSA), and non-esterified fatty acids (NEFA), and the serum binding of indapamide were measured in four groups of individuals: control (healthy) subjects (N = 24), patients with inflammatory syndrome (N = 28), with hepatic (N = 20) and renal (N = 27) insufficiency. Indapamide serum binding was increased in patients with inflammatory syndrome (82.2 +/- 3.4%, P less than .001), decreased in patients with hepatic insufficiency (72.3 +/- 5.9%, P less than .001) and unchanged in patients with renal insufficiency (77.7 +/- 2.8%) as compared with controls (78.2 +/- 3.1%). A multivariate... (truncated)

Title: Therapeutic Drug Monitoring in Arterial Hypertension.
(1) Background: This study was planned to assess the concentration of antihypertensive drugs (AHD) in the blood serum in patients with controlled and uncontrolled arterial hypertension (AH). (2) Methods: We assessed 46 patients with AH. Based on the results of 24 h blood pressure monitoring (ABPM), the patients were randomized into two groups. The first group consisted of the patients with controlled AH; the second group consisted of the patients with uncontrolled AH. Venous blood was taken in both groups of patients in the morning before and 2 h after taking drugs to assess the concentration of lisinopril, amlodipine, valsartan,... (truncated)

Title: The Efficacy and Tolerability of a Fixed Combination of Perindopril and Indapamide in the Treatment of Unregulated Essential Hypertension - a Postmarketing Study.
The objective of this non-interventional post-marketing clinical trial was to analyze the antihypertensive effect and safety of a fixed combination of perindopril and indapamide in the treatment of unregulated essential hypertension.

Title: An integrated safety analysis of combined acetaminophen and ibuprofen (Maxigesic ® /Combogesic®) in adults.
Acetaminophen (APAP) and ibuprofen (IBP) are two analgesic compounds with a long history of use. Both are considered safe at recommended over-the-counter daily doses. Chronic use, high doses, or concomitant medication can produce safety risks for both drugs. APAP is associated with increased risk of hepatic injury, while IBP can produce gastric bleeding and thromboembolic events. Using a combination of APAP and IBP provides superior analgesia without transgressing daily dose limits of each individual drug.

MESH:D008721 - methocarbamol

Summary:

LLM Prediction Score	0.084 (normalized)
LLM Confidence Score	0.970
Golden Answer (Severity Class)	0.625 (normalized)
Prediction Error	0.541

Retrieved Context:

Title: Hulk-Like Urine: A Case of Green Urine Caused by Flupirtine Intoxication.
Acute intoxications are common causes of admission to the Emergency Department (ED). Flupirtine is a non-opioid analgesic, originally used for acute and chronic pain. Because of several reports of severe liver toxicity, its use was limited to acute pain in 2013 by the European Medicines Agency. Although withdrawn from the European market in March 2018, there are still flupirtine tablets in many households, and most people are unaware of the hazards they might be facing. A 58-year-old man was admitted to the ED after a suicide attempt with 1 g of flupirtine. He was lethargic and confused but presented no... (truncated)

Title: Acetaminophen toxicity with concomitant use of carbamazepine.
Acetaminophen is a widely used analgesic that can cause acute liver failure when consumed above a maximum daily dose. Certain patients may be at increased risk of hepatocellular damage even at conventional therapeutic doses. We report a case of a 34-year-old man on carbamazepine for complex partial seizures who developed acute liver and renal failure on less than 2.5 grams a day of acetaminophen. This raises caution that patients on carbamazepine should avoid chronic use of acetaminophen, and if required use at lower doses with vigilant monitoring for signs of liver damage.

Title: A scoping review to identify and map the multidimensional domains of pain in adults with advanced liver disease.
Background: Pain is a significant problem in adults living with advanced liver disease, having limited guidance available for its clinical management. While pain is considered a multidimensional experience, there have been limited reviews of the pain literature in advanced liver disease conducted with a multidimensional framework. Aims: The goal of this scoping review was to identify and map the multidimensional domains of pain in adults with advanced liver disease using the biopsychosocial model. Methods: We used Arksey and O'Malley's scoping framework. A search was conducted in MEDLINE, Embase, AMED, and CINAHL databases and the gray literature using specific eligibility criteria... (truncated)

Title: Utility of Lymphocyte Transformation Test for Assisting Updated Roussel Uclaf Causality Assessment Method in Drug-Induced Liver Injury: A Case-Control Study.
Background: The Roussel Uclaf Causality Assessment Method (RUCAM) is a validated tool for assessing causality in cases of suspected drug-induced liver injury (DILI). However, RUCAM cannot discriminate between concomitant hepatotoxic drugs with the same temporal sequence. Objective: To analyse the utility of the lymphocyte transformation test (LTT) for assisting updated RUCAM in 45 patients and 40 controls with a clinical diagnosis of DILI. Methods: Suspected DILI cases were detected through the Prospective Pharmacovigilance Program from Laboratory Signals in Hospital (PPLSH) or by consultations. The controls completed the drug therapy with no adverse reactions during the study period. A receiver operating... (truncated)

Title: Mining hidden knowledge for drug safety assessment: topic modeling of LiverTox as a case study.
Given the significant impact on public health and drug development, drug safety has been a focal point and research emphasis across multiple disciplines in addition to scientific investigation, including consumer advocates, drug developers and regulators. Such a concern and effort has led numerous databases with drug safety information available in the public domain and the majority of them contain substantial textual data. Text mining offers an opportunity to leverage the hidden knowledge within these textual data for the enhanced understanding of drug safety and thus improving public health.

MESH:D000077551 - micafungin

Summary:

LLM Prediction Score	0.337 (normalized)
LLM Confidence Score	0.980
Golden Answer (Severity Class)	0.875 (normalized)
Prediction Error	0.538

Retrieved Context:

Title: [Liver toxicity of micafungin. Is this drug safe?].
Micafungin was commercialized in Japan in 2002 and has been used in more than 750,000 patients. As a member of the candin family, the drug's clinical and analytical tolerability is very good, both in adults and in children, including neonates. In this latter population, micafungin is the most frequently used candin. The most common adverse effects are nausea and elevated transaminase levels. Preclinical studies showed the development of benign liver tumors in rats treated with extremely high doses of the drug for prolonged periods. These data were not reproduced in other species and no cases have been reported in humans.

Title: Drug-Induced Liver Injury (DILI) With Micafungin: The Importance of Causality Assessment.
Background: Micafungin is increasingly used in the treatment and prevention of candidiasis in hospitalized patients. Limited data are available from which to assess the risk of drug-induced liver injury (DILI) with micafungin. No studies, to date, have applied a standardized causality assessment method to the study of micafungin-associated DILI. Objective: This study aimed to identify the frequency and clinical pattern of DILI in micafungin-treated patients as determined using 2 standardized causality assessment algorithms. Methods: A retrospective analysis was conducted of micafungin-treated patients at a single center between May 15, 2017, and May 15, 2018. DILI was defined on the basis... (truncated)

Title: Caspofungin versus micafungin in the incidence of hepatotoxicity in patients with normal to moderate liver failure.
One of the major adverse events of caspofungin and micafungin is hepatotoxicity, however, there are few reports compared the incidence of hepatotoxicity between caspofungin and micafungin. Herein, the primary objective of this study was to compare the incidence of hepatotoxicity between caspofungin and micafungin treatments for patients with fungal or suspected fungal infection.

Title: Short-term risk of liver and renal injury in hospitalized patients using micafungin: a multicentre cohort study.
Although echinocandins are generally well tolerated, there is little information on the frequency with which renal and hepatic adverse effects occur during use of micafungin or other parenteral antifungal (PAF) agents in clinical practice.

Title: Micafungin in the treatment of invasive candidiasis and invasive aspergillosis.
Micafungin is an echinocandin antifungal agent available for clinical use in Japan, Europe, and the United States. Through inhibition of β-1,3-glucan production, an essential component of the fungal cell wall, micafungin exhibits potent antifungal activity against key pathogenic fungi, including Candida and Aspergillus species, while contributing minimal toxicity to mammalian cells. This activity is maintained against polyene and azole-resistant isolates. Pharmacokinetic and pharmacodynamic studies have demonstrated linear kinetics both in adults and children with concentration-dependent activity observed both in vitro and in vivo. Dosage escalation studies have also demonstrated that doses much higher than those currently recommended may be administered... (truncated)

MESH:D004329 - droperidol

Summary:

LLM Prediction Score	0.536 (normalized)
LLM Confidence Score	0.980
Golden Answer (Severity Class)	0.0 (normalized)
Prediction Error	0.536

Retrieved Context:

Title: [Psychiatric drugs as risk factor in fatal heat stroke].
Two men aged 33 and 31 years suffered a fatal heat stroke on a warm summer day. One of them used pimozide and clomipramine, the other zuclopenthixol, dexetimide, droperidol, promethazine and propranolol as psychiatric medication. Both of them had a body temperature > 42.3 degrees C, without perspiring. At first only a comatose situation with practically normal laboratory values existed; this was rapidly followed by massive liver damage, disseminated intravascular coagulation, anaemia, thrombopenia and acute renal failure. In spite of adequate and rapid treatment these complications were fatal. Both patients used medication with an antidopaminergic and anticholinergic (side) effect. The... (truncated)

Title: [Effects of general anaesthesia with halothane and neuroleptic anaesthesia on free plasma amino acids (author's transl)].
Changes of plasma amino acids in animal studies were observed after administration of different narcotic drugs. In a randomised study the effects of neuroleptic anaesthesia and general anaesthesia with halothane on free plasma amino acids in respect to liver toxicity were investigated in patients during microsurgery of the middle ear. 24 hours postoperatively in both groups a significant increase of branched chain amino acids and of phenylalanine as metabolic sign of stress was found. Typical changes of amino acids as shown in hepatic failure could not be seen in both groups. The study showed that none of the narcotics used... (truncated)

Title: The effect of transdermal scopolamine for the prevention of postoperative nausea and vomiting.
Postoperative nausea and vomiting (PONV) is one of the most common and undesirable complaints recorded in as many as 70-80% of high-risk surgical patients. The current prophylactic therapy recommendations for PONV management stated in the Society of Ambulatory Anesthesia (SAMBA) guidelines should start with monotherapy and patients at moderate to high risk, a combination of antiemetic medication should be considered. Consequently, if rescue medication is required, the antiemetic drug chosen should be from a different therapeutic class and administration mode than the drug used for prophylaxis. The guidelines restrict the use of dexamethasone, transdermal scopolamine, aprepitant, and palonosetron as rescue... (truncated)

Title: [Clinical experience with low- and minimal-flow isoflurane in extensive resections of the liver and liver transplantation].
A comparative study was performed of general balanced anesthesia on the basis of neuroleptic analgesia (NLA) and inhalation low- and minimal-flow anesthesia with isoflurane in anesthesiological management of extensive operations on the liver. A total of 75 anesthesias were conducted. Because of low hepatotoxicity, absence of its metabolites' pharmacological activity, fast elimination from the body unchanged, isoflurane is effective in long and traumatic operations on the liver. Low-flow isoflurane significantly lowers pharmacological load with opiates, myorelaxants which is essential in patients with hepatic diseases. This leads to more rapid recovery of adequate spontaneous respiration and activation of the patient in... (truncated)

Title: Gastrointestinal bleeding and massive liver damage in neuroleptic malignant syndrome.
Neuroleptic malignant syndrome (NMS) is a rare side effect of antipsychotic therapy characterized by fever, muscular rigidity, altered mental status, increased level of serum creatinine phosphokinase, and increased number of white blood cells. The mortality rate of patients with NMS remains elevated.

MESH:D004809 - ephedrine

Summary:

LLM Prediction Score	0.533 (normalized)
LLM Confidence Score	0.990
Golden Answer (Severity Class)	0.0 (normalized)
Prediction Error	0.533

Retrieved Context:

Title: Ephedrine causes liver toxicity in SD rats via oxidative stress and inflammatory responses.
Ephedrine abuse has spread in many parts of the world and severely threatens human health. The mechanism of ephedrine-induced toxicity still remains unclear. This study was performed to investigate the effects of ephedrine treatment on the liver and explore the underlying mechanisms. Sprague Dawley rats were divided into saline and ephedrine groups. Rats were treated with ephedrine at 20 mg/kg or 40 mg/kg (<i>n</i> = 10) by oral gavage daily for 7 days. Pathological changes were examined by hematoxylin and eosin staining and terminal deoxynucleotidyl transferase deoxyuridine triphosphate nick end labeling assay. Enzyme-linked immunosorbent assays were used to measure the... (truncated)

Title: Ephedrine-induced mitophagy via oxidative stress in human hepatic stellate cells.
The herb Ephedra sinica (also known as Chinese ephedra or Ma Huang), used in traditional Chinese medicine, contains alkaloids identical to ephedrine and pseudoephedrine as its principal active constituents. Recent studies have reported that ephedrine has various side effects in the cardiovascular and nervous systems. In addition, herbal Ephedra, a plant containing many pharmacologically active alkaloids, principally ephedrine, has been reported to cause acute hepatitis. Many studies reported clinical cases, however, the cellular mechanism of liver toxicity by ephedrine remains unknown. In this study, we investigated hepatotoxicity and key regulation of mitophagy in ephedrine-treated LX-2 cells. Ephedrine triggered mitochondrial oxidative... (truncated)

Title: Acute hepatitis associated with the use of a Chinese herbal product, ma-huang.
Herbal medicines are widely perceived by the public as being healthful and innocuous. A number of herbal medicines have now been linked with hepatotoxicity. We report a case of acute hepatitis associated with the use of ma-huang, a herbal product derived from plants of the Ephedra species, which is advertised as being useful for causing weight loss and enhancing energy levels. Given the lack of reports in the literature of hepatotoxicity with ma-huang and ephedrine, we speculate that the ma-huang product our patient took contained some other ingredient or contaminant or was misidentified. Our report and others in the literature,... (truncated)

Title: Effect of extractions from <i>Ephedra sinica</i> Stapf on hyperlipidemia in mice.
The aim of the present study was to investigate the hypolipidemic and antioxidant potential of ephedra extractions in diet-induced hyperlipidemic mice. Mice were fed a diet high in fat to establish the hyperlipidemic model. A total of 48 mice were randomly divided into six groups, which included the normal control, model control, positive control, ephedra alkaloid, ephedra polysaccharide and ephedra non-alkaloid groups. Intragastric administration of the respective treatments was provided continuously for four weeks and the body weight was recorded weekly. The total levels of cholesterol (TC), triglycerides (TG), high-density lipoprotein cholesterol (HDL-C) and malondialdehyde (MDA), and the activity levels... (truncated)

Title: Sympathomimetic amine compounds and hepatotoxicity: Not all are alike-Key distinctions noted in a short review.
Sympathomimetic amine compounds are often pooled together and incorrectly assumed to be interchangeable with respect to potential adverse effects. A brief and specific review of sympathomimetic compounds and one instance (i.e., hepatotoxicity) where these compounds have been improperly grouped together is covered. A review of the proposed mechanisms through which known hepatotoxic sympathomimetic agents (e.g., 3,4-methylenedioxymethamphetamine or MDMA, methamphetamine and amphetamine) cause liver injury, along with a corresponding review of in vitro data, interventional data, animal model studies and observational data allow for a comparison/contrast of different agents and reveals a lack of potential toxicity for some agents (e.g., pseudoephedrine,... (truncated)

MESH:D010076 - oxazepam

Summary:

LLM Prediction Score	0.093 (normalized)
LLM Confidence Score	0.990
Golden Answer (Severity Class)	0.625 (normalized)
Prediction Error	0.532

Retrieved Context:

Title: Glucuronidation of drugs by hepatic microsomes derived from healthy and cirrhotic human livers.
Pharmacokinetic studies demonstrated that the decrease in drug biotransformation in hepatic failure depends on the metabolic pathways involved. To test whether glucuronidation reactions supported by UDP-glucuronosyltransferases are differentially affected in such conditions, we investigated the in vitro glucuronidation of four selected drugs and xenobiotics (zidovudine, oxazepam, lamotrigine, and umbelliferone) by using microsomes from human healthy and unhealthy (cirrhosis, hepatitis) livers as enzyme sources. Theses substances are glucuronidated by several UDP-glucuronosyltransferase isoforms. Lidocaine N-deethylation activity measured concomitantly was used as a positive control, because the inhibition of this reaction in patients with hepatic diseases is well documented. The metabolic clearances of... (truncated)

Title: [Voluntary drug poisoning: epidemiology, performance and limits of the emergency laboratory].
The aim of this study is to determine the efficiency ot toxicologic screening (detection of barbiturates, benzodiazepines, tricyclic antidepressants, salicylates, phenothiazines, meprobamate and ethanol assay), during acute drug poisoning. In 1988, 898 patients are enclose in this study. Screenings are negative in 17% of cases; benzodiazepines, alcohol and antidepressants are often found. The recovery is very good for barbiturates and salicylates but it's not perfect for benzodiazepines, particularly flunitrazepam triazolam, loflazepate, oxazepam, and non tricyclic antidepressants. This failure probably depends on these emergency methods.

Title: Metabolism and elimination of oxazepam in F344 rats.
The antianxiety agent, oxazepam, is a mouse liver carcinogen as determined by a National Toxicology Program bioassay. An equivalent study in the F344 rat is currently in progress. In an effort to gain insight into whether the mouse or rat will be a better model for human risk assessment, extensive comparative metabolism studies have been conducted in both rodent species and compared with the human literature. In this study, male rats were treated with 25, 250, or 500 mg/kg of radiolabeled oxazepam. In addition, sex comparisons were made at 500 mg/kg after 0 and 14 days of 2500 ppm oxazepam... (truncated)

Title: Normal disposition of oxazepam in acute viral hepatitis and cirrhosis.
Oxazepam (Serax) is a tranquilizer-sedative of the benzodiazepine group that is predominantly metabolized to a pharmacologically inactive glucuronide and subsequently excreted by way of the kidneys. We administered this drug as a single oral dose to seven patients with acute viral hepatitis, to six with cirrhosis, and to age-matched control subjects. Elimination half-life (T1/2) and the apparent oral plasma clearance for the drug in patients with hepatitis and cirrhosis were comparable to values obtained in age-matched controls (P greater than 0.05). In addition, the apparent volume of distribution of oxazepam, its plasma binding, blood/plasma ratio, and the rate of urinary... (truncated)

Title: Feline hepatic biotransformation of diazepam: Differences between cats and dogs.
In contrast to humans and dogs, diazepam has been reported to induce severe hepatic side effects in cats, particularly after repeated dosing. With the aim to elucidate the mechanisms underlying this apparent sensitivity of cats to drug-induced liver injury, in a series of in vitro experiments, the feline-specific biotransformation of diazepam was studied with liver microsomes obtained from cats and dogs and the possible inhibition of the bile salt export pump (Bsep) was measured in isolated membrane vesicles overexpressing feline and canine Bsep. In line with previous in vivo studies, the phase I metabolites nordiazepam, temazepam and oxazepam were measurable... (truncated)

MESH:D002997 - clomipramine

Summary:

LLM Prediction Score	0.477 (normalized)
LLM Confidence Score	0.990
Golden Answer (Severity Class)	1.0 (normalized)
Prediction Error	0.523

Retrieved Context:

Title: Cross hepatotoxicity between tricyclic antidepressants.
Cross hepatotoxicity between drugs in very uncommon. We report the case of a patient in whom acute hepatitis was induced by a tricyclic antidepressant, amineptine, and recurred early after administration of another tricyclic antidepressant, clomipramine. This observation suggests that the tricyclic ring is involved in the mechanism of the deleterious effect of both drugs to the liver.

Title: Treatment of Cataplexy with Clomipramine.
A new antodepressant drug, clomipramine hydrochloride, closely related to imipramine hydrochloride, was used to treat four patients suffering from cataplexy, sleep paralysis, and hypnagogic hallucinations. Attacks of cataplexy were associated with rapid-eye-movement (REM) electroencephalographic patterns. Cloripramine, in doses of 25 to 75 mg/day, completely stopped all attacks of cataplexy, sleep paralysis, and hypnagogic hallucinations within 48 hours of initial therapy. The patients have been free of symptoms for periods of 10 to 21 months. Side effects included impotence in the male patients, but no hematologic, cardiovascular, hepatic, or renal toxic effects were observed. Available evidence suggests that such drugs inhibit... (truncated)

Title: A case of photosensitivity and contact allergy to systemic tricyclic drugs, with unusual features.
A 43-year-old farmer on tricyclic antidepressive drugs developed a severe photodermatitis with associated liver involvement. The lesions spread to covered areas of the skin, suggesting photoallergy clinically. Patch and photopatch testing revealed photoallergy and contact allergy to clomipramine and contact allergy also to carbamazepine. In addition, the patient had positive patch test reactions to chlorpromazine, balsam of Peru and fragrance-mix, as well as a positive photopatch test to fentichlor. UVA and UVB erythema thresholds were normal. In this patient, an initial episode of photosensitization, probably elicited by clomipramine, was accompanied by contact allergy to this drug and to carbamazepine. The... (truncated)

Title: Safety of Cyproheptadine, an Orexigenic Drug. Analysis of the French National Pharmacovigilance Data-Base and Systematic Review.
Objectives: Cyproheptadine is a first-generation H1-antihistamine drug first that was distributed in the 1960s. While its orexigenic effect was observed early, cyproheptadine is not yet authorized for this indication in all countries today. There is an increasing medical interest and demand for the orexigenic effect of cyproheptadine, especially in children with poor appetite. As cyproheptadine might be evaluated in future clinical trials, we wanted to assess its safety profile. Methods: Using the French national pharmacovigilance database, we retrospectively analyzed all pediatric and adult reports of adverse effects of cyproheptadine recorded since its first distribution in France. Next, we performed a... (truncated)

Title: Imipramine Accelerates Nonalcoholic Fatty Liver Disease, Renal Impairment, Diabetic Retinopathy, Insulin Resistance, and Urinary Chromium Loss in Obese Mice.
Imipramine is a tricyclic antidepressant that has been approved for treating depression and anxiety in patients and animals and that has relatively mild side effects. However, the mechanisms of imipramine-associated disruption to metabolism and negative hepatic, renal, and retinal effects are not well defined. In this study, we evaluated C57BL6/J mice subjected to a high-fat diet (HFD) to study imipramine's influences on obesity, fatty liver scores, glucose homeostasis, hepatic damage, distribution of chromium, and retinal/renal impairments. Obese mice receiving imipramine treatment had higher body, epididymal fat pad, and liver weights; higher serum triglyceride, aspartate and alanine aminotransferase, creatinine, blood urea... (truncated)

MESH:D015725 - fluconazole

Summary:

LLM Prediction Score	0.482 (normalized)
LLM Confidence Score	0.990
Golden Answer (Severity Class)	1.0 (normalized)
Prediction Error	0.518

Retrieved Context:

Title: The possible efficacy of artichoke in fluconazole related hepatotoxicity.
Although fluconazole related hepatotoxicity (FRH) is rare, mortal acute hepatic necrosis and jaundice were reported in immunocompromised states such as acquired immunodeficiency syndrome (AIDS) and bone marrow transplant (BMT). We present a case of a patient with multiple sclerosis who developed hepatotoxicity with the use of a single 150 mg fluconazole tablet for fungal vaginitis, 10 days after methylprednisolone pulse treatment. Our patient's alanine aminotransferase (ALT) and aspartate aminotransferase (AST) levels were decreased, 1200 U/L and 800 U/L, respectively, and bilirubin levels were consistent at 37 mg/dL. Artichoke which has anticholestatic and antioxidant properties was used by our patient. She... (truncated)

Title: Worsening of liver function with fluconazole and review of azole antifungal hepatotoxicity.
To report a case of fluconazole worsening of liver dysfunction.

Title: Comparative Hepatotoxicity of Fluconazole, Ketoconazole, Itraconazole, Terbinafine, and Griseofulvin in Rats.
Oral ketoconazole was recently the subject of regulatory safety warnings because of its association with increased risk of inducing hepatic injury. However, the relative hepatotoxicity of antifungal agents has not been clearly established. The aim of this study was to compare the hepatotoxicity induced by five commonly prescribed oral antifungal agents. Rats were treated with therapeutic oral doses of griseofulvin, fluconazole, itraconazole, ketoconazole, and terbinafine. After 14 days, only ketoconazole had significantly higher ALT levels (<i>p</i> = 0.0017) and AST levels (<i>p</i> = 0.0008) than the control group. After 28 days, ALT levels were highest in the rats treated with... (truncated)

Title: Evaluation of hepatic injury arising during fluconazole therapy.
Previous reports have described hepatotoxicity associated with ketoconazole therapy. There is also concern that therapy with fluconazole might cause the same side effect as ketoconazole. We describe two patients who developed unexplained liver test abnormalities after beginning fluconazole therapy. To determine whether fluconazole might have been responsible, liver biopsies were performed. Specimens from both patients demonstrated an absence of hepatocyte necrosis, which, if present, would have necessitated discontinuation of fluconazole therapy. A critical review of other case reports of fluconazole-associated hepatitis also failed to produce a consistent picture. Our experience indicates that a liver biopsy may be useful in selected... (truncated)

Title: Hepatotoxicity Due to Azole Antimycotic Agents in a HLA B*35:02-Positive Patient.
We will present a 42-year-old woman with acute myeloid leukemia and pulmonary aspergillosis. She was treated with several antifungal agents, including three triazoles. Voriconazole, posaconazole, and isavuconazole all led to hepatocellular liver injury. Voriconazole administration led to a peak alanine aminotransferase (ALT) value of 1,793 U/L (normal range, 9-59 U/L). After posaconazole and isavuconazole treatment, ALT rose over 500 U/L. The typical course of events, exclusion of differential diagnoses, and normalization of the liver function tests (LFTs) after stopping the triazoles were highly suspicious for a drug-induced liver injury (DILI). Interestingly, our patient carries a rare HLA B allele (HLA... (truncated)

MESH:D001374 - azacitidine

Summary:

LLM Prediction Score	0.483 (normalized)
LLM Confidence Score	0.980
Golden Answer (Severity Class)	1.0 (normalized)
Prediction Error	0.517

Retrieved Context:

Title: Phase I study of 5-azacytidine (NSC-102816) .
Thirty patients with various solid tumors were treated with 5-azacytidine. Total doses ranged from 1.0 to 24.0 mg/kg and were given over a minimal period of 8 days. The major toxic effect was hematologic with significant leukopenia and thrombocytopenia usually occurring 20-30 days after the start of therapy, especially at higher dose levels. The marrow depression lasted 1-5 weeks and was fully reversible. Nausea and mild diarrhea were common following injection of the drug. Serum glutamic oxaloacetic transaminase levels rose in several patients. No other evidence of hepatic toxicity was seen. Objective remissions were noted in seven of 11 patients... (truncated)

Title: 5-Azacytidine. A new anticancer drug with effectiveness in acute myelogenous leukemia.
Clinical studies involving 5-azacytidine, a ring analogue of cytidine, began in Europe in 1967 and the United States in 1970, and we review available preclinical and clinical studies here. The drug possesses cytotoxic, antimicrobial, antineoplastic, abortive, and mutagenic activity in various biological systems. 5-Azacytidine is thought to exert its antineoplastic effect through interference with nucleic acid metabolism. The dose-limiting toxicities are nausea, vomiting, and leukopenia, while the incidence of thrombocytopenia is low. Hepatic toxicity ranges from abnormal findings in liver function tests to hepatic coma. Clinical results in solid tumors are not encouraging, but 5-azacytidine shows consistent antitumor activity in... (truncated)

Title: 5-azacytidine in refractory acute leukemia.
11 patients (aged 23--75 years) with refractory acute leukemia were treated at the Maine Medical Center with 5-Azacytidine, 150 mg/m2/day, by continuous infusion for 5 days every 2 weeks. Prior therapy included anthracycline/cytosine arabinoside protocols. Of the 8 patients with refractory de novo acute myelogenous leukemia, 6 achieved remission at an overall response rate of 75% (3 complete remission and 3 partial remission). An average of 1.67 courses was necessary to achieve a response. Remissions were not seen in blastic chronic myelogenous leukemia nor in acute leukemia secondary to cytotoxic drugs. Toxicity included myelosuppression, moderate nausea and vomiting, abnormal liver... (truncated)

Title: A Phase II Multicenter Study of the Addition of Azacitidine to Reduced-Intensity Conditioning Allogeneic Transplant for High-Risk Myelodysplasia (and Older Patients with Acute Myeloid Leukemia): Results of CALGB 100801 (Alliance).
Relapse remains the major cause of death in older patients transplanted for acute myeloid leukemia (AML) in first complete remission or for patients with advanced myelodysplastic syndrome (MDS) at any age. Conventional myeloablative conditioning followed by allogeneic blood or marrow transplantation is associated with significantly less relapse compared with reduced-intensity conditioning when performed in younger patients with AML or MDS, but the toxicity of this approach in older patients is prohibitive. We hypothesized that pharmacokinetic targeting to optimize busulfan (BU) exposure, combined with the administration of azacitidine (AZA) post-transplant would mitigate the risk of relapse while reducing nonrelapse mortality and... (truncated)

Title: Anti-oxidative and anti-inflammatory benefits of the ribonucleoside analogue 5-azacitidine in mice with acetaminophen-induced toxic hepatitis.
Toxic hepatitis induced by overdose of acetaminophen (APAP) is one of the major life-threatening problems, oxidative stress and inflammatory injury are the essential underlying mechanisms. 5-Azacytidine (5-AZA) is a ribonucleoside analogue which has been approved for the treatment of patients with acute myeloid leukemia and myelodyplastic syndrome, but recent studies also found that 5-AZA might have anti-oxidative and anti-inflammatory benefits in non-tumor disorders. In the present study, the potential effects of 5-AZA on APAP-induced toxic hepatitis were investigated in a mouse model in vivo. The results indicated that treatment with 5-AZA suppressed the elevation of alanine aminotransferase (ALT) and aspartate... (truncated)

MESH:D009254 - nafcillin

Summary:

LLM Prediction Score	0.892 (normalized)
LLM Confidence Score	0.960
Golden Answer (Severity Class)	0.375 (normalized)
Prediction Error	0.517

Retrieved Context:

Title: Nafcillin-Induced Allergic Eosinophilic Cholestatic Hepatitis.
A 71-year-old female with no history of liver disease or antibiotic allergy developed jaundice with elevated liver enzymes and eosinophilia following treatment with nafcillin for septic arthritis. Further workup demonstrated hepatocellular dysfunction and liver biopsy showed expansion of portal tracts by lymphocytes and eosinophils consistent with a hypersensitivity reaction. Nafcillin and related antibiotics were withdrawn, and her symptoms resolved 3 months later. We searched PubMed using terms of "nafcillin cholestasis" and "nafcillin hepatitis", and a review of the literature showed other reports of nafcillin-induced hepatitis and cholestasis. Avoidance and on occasion the guarded use of glucocorticoids can lead to recovery... (truncated)

Title: A Patient with Nafcillin-Associated Drug-Induced Liver Failure.
Nafcillin-induced acute liver injury is a rare and potentially fatal complication that has been known since the 1960s but inadequately studied. At this time, the only proven treatment is early discontinuation of the drug. Because of the high prevalence of nafcillin class antibiotic use in the United States, it is important for clinicians to have a high clinical suspicion for this diagnosis. We present a case of liver failure attributable to nafcillin use in a 68-year-old male with a history methicillin-sensitive <i>Staphylococcus</i> and L3/L4 osteomyelitis. After starting long-term antibiotic therapy, he presented with painless jaundice which necessitated discontinuation of the... (truncated)

Title: A fatal case of nafcillin-induced hepatotoxicity: a case report and the literature review.
Background. Drug-induced hepatotoxicity (DIH) is quite common, and there are several recommendations for its treatment based upon its etiology. DIH may range from mild and subclinical to fulminant liver failure and death. Even though there is extensive list of drugs causing DIH, antibiotics, as a class of drugs, are the most common cause of DIH. Here, we present a fatal case of nafcillin-induced hepatotoxicity confirmed by liver biopsy, with total bilirubin peaking to 21.8 mg/dl and subsequent further extensive evaluation for hepatic injury turning out to be negative.

Title: Nafcillin-associated hepatotoxicity. Report of a case and review of the literature.
Nafcillin is a semisynthetic penicillin that is generally well tolerated with few side effects. Hepatic complications are rare but have a potential for serious liver dysfunction. This unusual complication causes a predominantly cholestatic injury, which can persist for prolonged periods even after discontinuing the medication. The pathophysiology may include direct cytotoxicity or an immune-mediate hypersensitivity. Treatment is generally supportive, except for severely symptomatic patients who may require steroids. We report a case of nafcillin-associated hepatotoxicity and review the literature of this disorder.

Title: Hepatic and renal dysfunction following nafcillin administration.
To review four cases of combined hepatic and renal toxicity that may be associated with the administration of nafcillin in adults. This type of adverse event with the use of nafcillin has not been previously documented in the literature.

MESH:D000077525 - cefdinir

Summary:

LLM Prediction Score	0.360 (normalized)
LLM Confidence Score	0.980
Golden Answer (Severity Class)	0.875 (normalized)
Prediction Error	0.515

Retrieved Context:

Title: Uncommon Cause of Acute Drug-induced Liver Injury Following Mammoplasty.
Cephalexin is a well tolerated antimicrobial and hepatic injury is an infrequent occurrence with its use. We here describe a 21-year-old female who presented with jaundice and elevated liver enzymes after 4 weeks completion of 10 day course of cephalexin, prescribed prophylactically after mammoplasty. Extensive work up including all causes of hepatitis was within normal limits and she improved with conservative management. This case highlights the need to suspect drug induced liver injury in cases of jaundice and cephalexin use.

Title: Cephalexin induced cholestatic jaundice.
Cephalexin is a very commonly prescribed orally administered antibiotic which has many potential side effects. Amongst these cholestatic jaundice has been infrequently reported as an adverse reaction. We present a case of a 57-year-old male who exhibited features of cholestatic jaundice including elevated liver function tests (LFTs) after taking cephalexin and showed improvement on removal of the offending agent. During this time he was symptomatically treated with cholestyramine. Complete resolution of LFTs was seen in four weeks. Cephalexin induced cholestasis is rare and hence requires a high degree of clinical suspicion for prompt diagnosis and treatment.

Title: Hepatic safety of antibiotics used in primary care.
Antibiotics used by general practitioners frequently appear in adverse-event reports of drug-induced hepatotoxicity. Most cases are idiosyncratic (the adverse reaction cannot be predicted from the drug's pharmacological profile or from pre-clinical toxicology tests) and occur via an immunological reaction or in response to the presence of hepatotoxic metabolites. With the exception of trovafloxacin and telithromycin (now severely restricted), hepatotoxicity crude incidence remains globally low but variable. Thus, amoxicillin/clavulanate and co-trimoxazole, as well as flucloxacillin, cause hepatotoxic reactions at rates that make them visible in general practice (cases are often isolated, may have a delayed onset, sometimes appear only after cessation... (truncated)

Title: Cephalosporin's induced hepatic enzyme derangement - An educational report.
No abstract available.

Title: Differential Effects of 1α,25-Dihydroxyvitamin D₃ on the Expressions and Functions of Hepatic CYP and UGT Enzymes and Its Pharmacokinetic Consequences In Vivo.
The compound 1α,25-Dihydroxyvitamin D₃ (1,25(OH)₂D₃) is the active form of vitamin D₃ and a representative ligand of the vitamin D receptor (VDR). Previous studies have described the impacts of 1,25(OH)₂D₃ on a small number of cytochrome P450 (CYP) and uridine diphosphate-glucuronyltransferase (UGT) enzymes, but comparatively little is known about interactions between several important CYP and UGT isoforms and 1,25(OH)₂D₃ in vitro and/or in vivo. Thus, we investigated the effects of 1,25(OH)₂D₃ on the gene and protein expressions and functional activities of selected CYPs and UGTs and their impacts on drug pharmacokinetics in rats. The mRNA/protein expressions of Cyp2b1 and Cyp2c11... (truncated)

MESH:D016685 - mitomycin

Summary:

LLM Prediction Score	0.515 (normalized)
LLM Confidence Score	0.990
Golden Answer (Severity Class)	0.0 (normalized)
Prediction Error	0.515

Retrieved Context:

Title: Phase II study of intra-arterial fluorouracil and mitomycin-C for liver metastases of colorectal cancer.
Effectiveness, toxicity and complications of 5-fluorouracil (FU) and mitomycin-C (MMC) treatment were analyzed in 30 patients with metastatic colorectal cancer confined to the liver. The treatment schedule was FU 2.0-2.5 g/day for 5 days followed by MMC 10 mg/m2 every 2 h on day 6 to a maximum total dose of 60 mg. Treatment courses were repeated every 6 weeks and were given on an outpatient basis via external pump and arterial port systems. In 30 fully evaluable patients, one complete response, 17 partial responses (overall response rate 60%), and stabilization of disease in 8 patients (26%) were obtained for... (truncated)

Title: Effectiveness and low toxicity of hepatic artery infusion with fluorouracil and mitomycin for metastatic colorectal cancer confined to the liver. The Swiss Group for Clinical and Epidemiological Cancer Research (SAKK).
The usefulness of hepatic artery infusion (HAI) with floxuridine is limited by the severe biliary and hepatic toxicity of floxuridine. This prompted the SAKK to evaluate the effectiveness, toxicity and feasibility of HAI with fluorouracil (FU) and mitomycin (MMC) administered by an external portable pump. Of 28 patients treated, partial responses were obtained in 14 (50%, 95% confidence interval: 30% to 70%) and stabilization in 11 (39%, 21% to 60%), for a median duration of 12.6+ months. Median survival was 19.5+ months. Grade I-II toxicity (WHO) consisted of nausea (46%), leucopenia (32%) thrombocytopenia (21%) and abdominal discomfort (25%). Two patients... (truncated)

Title: [Arterial infusion of combination chemotherapy consisting of adriamycin and mitomycin C for liver metastases of breast cancer].
Forty-nine patients with liver metastases of breast cancer were treated with arterial infusion involving simultaneous use of adriamycin: 30-50 mg and mitomycin C: 10-20 mg through the hepatic artery by Seldinger catheter. Of 45 evaluable patients, there were 5 complete and 12 partial responses (CR + PR: 38%), 21 no change and 7 cases of progressive disease. The median duration of response was 5 months. The median survival time was 7.5 months in all cases, 8.7 months for responders and 6.4 months for non-responders. Leukopenia less than 4 X 10(3)/mm3 was observed in 73% of cases, thrombocytopenia less than 100... (truncated)

Title: Therapy of hepatocellular cancer with combined intrahepatic arterial chemotherapy and whole liver irradiation.
Hepatocellular cancer provides an ideal model for studying combined modality chemotherapy and radiation interactions. We have treated 20 evaluable patients. All patients received intrahepatic arterial (IA) 5 FU (10 mg/kg/d continuous infusion times 5 minus 9 d) + Adriamycin (3-5 mg/m(2)/d bolus times 5 minus 7 d), and 1,500 and 2,100 rads whole liver radiation (300 rads/day). Additionally, 3 patients have received IA Mitomycin C (8 mg/m(2)). After this "induction" therapy patients usually received IV Adriamycin + 5FU +/- Mitomycin monthly. Objective regressions occurred in 9/20 (45%) and another 9/20 (45%) and stable disease. Median duration of response is 5+... (truncated)

Title: [Side effects and anti-cancer effects following intraperitoneal (ip) administration of mitomycin C (MMC)].
Nineteen patients with gynecological cancers were initially treated intraperitoneally with MMC at a dose of 20 to 52 mg/body and then intravenously with CAP regimen. The following side effects were observed in patients intraperitoneally administered doses of MMC greater than 25 mg/m2; leucocytopenia 7/12, thrombocytopenia 5/12, liver damage 8/12, renal damage 2/12, ileus 3/12 and severe anorexia 2/12. Sixteen patients were evaluated for their prognoses more than 1 year after treatment. Seven of them are well with no sign of recurrence, but 9 suffered recurrence with or without peritonitis carcinomatosa (PC). The incidence of PC among these 9 cases was... (truncated)

MESH:D000077407 - cilostazol

Summary:

LLM Prediction Score	0.111 (normalized)
LLM Confidence Score	0.980
Golden Answer (Severity Class)	0.625 (normalized)
Prediction Error	0.514

Retrieved Context:

Title: Cilostazol attenuates murine hepatic ischemia and reperfusion injury via heme oxygenase-dependent activation of mitochondrial biogenesis.
Hepatic ischemia-reperfusion (I/R) can cause hepatocellular injury associated with the inflammatory response and mitochondrial dysfunction. We studied the protective effects of the phosphodiesterase inhibitor cilostazol in hepatic I/R and the roles of mitochondria and the Nrf2/heme oxygenase-1 (HO-1) system. Wild-type, Hmox1(-/-), or Nrf2(-/-) mice were subjected to hepatic I/R in the absence or presence of cilostazol followed by measurements of liver injury. Primary hepatocytes were subjected to cilostazol with the HO-1 inhibitor ZnPP, or Nrf2-specific siRNA, followed by assessment of mitochondrial biogenesis. Preconditioning with cilostazol prior to hepatic I/R protected against hepatocellular injury and mitochondrial dysfunction. Cilostazol reduced the serum... (truncated)

Title: Detection of Synergistic Interaction on an Additive Scale Between Two Drugs on Abnormal Elevation of Serum Alanine Aminotransferase Using Machine-Learning Algorithms.
Drug-induced liver injury (DILI) is a common adverse drug reaction, with abnormal elevation of serum alanine aminotransferase (ALT). Several clinical studies have investigated whether a combination of two drugs alters the reporting frequency of DILI using traditional statistical methods such as multiple logistic regression (MLR), but this model may over-fit the data. This study aimed to detect a synergistic interaction between two drugs on the risk of abnormal elevation of serum ALT in Japanese adult patients using three machine-learning algorithms: MLR, logistic least absolute shrinkage and selection operator (LASSO) regression, and extreme gradient boosting (XGBoost) algorithms. A total of 58,413... (truncated)

Title: Pharmacological Prevention of Ectopic Erythrophagocytosis by Cilostazol Mitigates Ferroptosis in NASH.
Hepatic iron overload (HIO) is a hallmark of nonalcoholic fatty liver disease (NAFLD) with a poor prognosis. Recently, the role of hepatic erythrophagocytosis in NAFLD is emerging as a cause of HIO. We undertook various assays using human NAFLD patient pathology samples and an in vivo nonalcoholic steatohepatitis (NASH) mouse model named STAM^{TM}. To make the in vitro conditions comparable to those of the in vivo NASH model, red blood cells (RBCs) and platelets were suspended and subjected to metabolic and inflammatory stresses. An insert-coculture system, in which activated THP-1 cells and RBCs are separated from HepG2 cells by a... (truncated)

Title: Effect of hepatic impairment on the pharmacokinetics of a single dose of cilostazol.
The pharmacokinetic profiles of cilostazol and its metabolites following a single oral dose of cilostazol 100 mg were compared between individuals with impaired and normal liver function.

Title: Clopidogrel-induced neutropenia after coronary stenting: is cilostazol a good alternative?
Dual antiplatelet therapy with aspirin plus thienopyridines has become the standard treatment of patients undergoing coronary stenting. Clopidogrel has mostly replaced the use of ticlopidine due to its more favourable adverse event profile. However, also the use of clopidogrel is not without side effects. Clopidogrel major adverse events are represented by marrow suppression, manifesting with aplastic anaemia, thrombocytopenia and neutropenia. When clopidogrel toxicity occurs, there are few and unsubstantiated alternative treatments and thus, in these cases, medical decisions may be very difficult. We report a case of clopidogrel-induced bone marrow toxicity manifesting with severe neutropenia in a patient treated with... (truncated)

MESH:D013012 - sorbitol

Summary:

LLM Prediction Score	0.514 (normalized)
LLM Confidence Score	0.990
Golden Answer (Severity Class)	0.0 (normalized)
Prediction Error	0.514

Retrieved Context:

Title: [Severe toxic liver injury after overdosage of parenteral administered carbohydrates: a case report (author's transl)].
A 31-year old female patient with anorexia nervosa developed a severe toxic liver injury after parenteral hyperalimentation. Over a period of five days she received a total amount of carbohydrates of 0.47-1.07 g/kg/hr consisting of glucose, fructose and the polyalcohols sorbitol and xylitol. A steep rise in SGOT, SGPT, and GLDH were noted as well as prolongation of the prothrombin time and decrease of the clotting factors; uric acid and lactate increased, serum phosphate decreased. After termination of parenteral hyperalimentation a laparoscopy and liver biopsy were performed. The liver biopsy revealed by light- and electronmicroscopy signs of a severe toxic... (truncated)

Title: Re-evaluation of sorbitan monostearate (E 491), sorbitan tristearate (E 492), sorbitan monolaurate (E 493), sorbitan monooleate (E 494) and sorbitan monopalmitate (E 495) when used as food additives.
The Panel on Food Additives and Nutrient Sources added to Food (ANS) provides a scientific opinion re-evaluating the safety of sorbitan monostearate (E 491), sorbitan tristearate (E 492), sorbitan monolaurate (E 493), sorbitan monooleate (E 494) and sorbitan monopalmitate (E 495) when used as food additives. The Scientific Committee on Food (SCF) allocated an acceptable daily intake (ADI) of 25 mg/kg body weight (bw) per day for E 491, E 492 and E 495 singly or in combination; and a separate group ADI for E 493 and E 494 singly or in combination of 5 mg/kg bw per day calculated as sorbitan monolaurate in 1974. The Panel noted that after oral administration sorbitan monostearate... (truncated)

Title: The physiological role of glucokinase binding and translocation in hepatocytes.
The compartmentation of glucokinase in the hepatocyte is regulated by the extracellular glucose concentration and by substrates that alter the concentration of fructose 1-phosphate in the hepatocyte. At low glucose concentrations, that mimic the fasted state, glucokinase is sequestered in an inactive state bound to the 68 kDa regulatory protein in the nucleus. In these conditions the rate of glucose phosphorylation is less than 15% of the total glucokinase activity. An increase in extracellular glucose concentration, within the range occurring in the portal vein in the absorptive state, or low concentrations of fructose or sorbitol (precursors of fructose 1-phosphate), cause... (truncated)

Title: Daily Fructose Traces Intake and Liver Injury in Children with Hereditary Fructose Intolerance.
Hereditary fructose intolerance (HFI) is a rare genetic disorder of fructose metabolism due to aldolase B enzyme deficiency. Treatment consists of fructose, sorbitol, and sucrose (FSS)-free diet. We explore possible correlations between daily fructose traces intake and liver injury biomarkers on a long-term period, in a cohort of young patients affected by HFI.

Title: [Repeated perioperative administration of fructose and sorbitol in a female patient with hereditary fructose intolerance [HFI)].
The present paper reports on an adult female patient whose hereditary fructose intolerance (HFI) was at first not diagnosed and who, within the space of 2 years after repeated elective surgery and the perioperative administration of fructose and sorbitol, developed "hepatic and renal failure of unclear origin." At a later stage we were able to establish the diagnosis of HFI by means of a fructose tolerance test in both she and her brother, for whom intolerance to fruit and desserts had been known since early childhood. In addition, literature references to fatalities following the parenteral application of fructose and sorbitol... (truncated)

MESH:D064704 - levofloxacin

Summary:

LLM Prediction Score	0.486 (normalized)
LLM Confidence Score	0.990
Golden Answer (Severity Class)	1.0 (normalized)
Prediction Error	0.514

Retrieved Context:

Title: [Levofloxacin adverse effects, data from clinical trials and pharmacovigilance].
With 5388 patients in the marketing application dossiers and post-marketing experience of more than 130 million prescriptions of levofloxacin worldwide, the tolerability profile of this anti-infective is now well defined. During clinical trials, 12 per cent of patients treated with levofloxacin experienced an adverse event considered to be related to the study drug compared with 13 per cent of the patients with a comparator. Nausea and diarrhoea were the most frequent adverse effects. During clinical trials, the frequency of tendinitis, psychotic episodes and seizures was less than 0.1 per cent. Following recent concerns with some fluoroquinolones, specific attention was paid... (truncated)

Title: A Case of Levofloxacin-Induced Hepatotoxicity.
BACKGROUND Levofloxacin covers a broad spectrum of pathogens and is readily prescribed by clinicians. Hepatotoxicity is a known but unusual complication of levofloxacin use. Here, we present a case of severe transaminitis caused by levofloxacin. CASE REPORT A young man in his thirties with a history of asthma, chronic alcoholism, methamphetamine intravenous drug abuse (IVDA), and non-compliant insulin-dependent diabetes mellitus (IDDM) presented to an emergency department with suicidal ideation. Vital signs were stable and the patient was noted to have cellulitis of the right forearm, for which cultures were drawn, and he received IV clindamycin. He was admitted to behavioral... (truncated)

Title: Comparison of side effects of levofloxacin versus other fluoroquinolones.
The side-effect profile of levofloxacin was compared with that of other fluoroquinolones based on European and international data from approximately 130 million prescriptions. Levofloxacin was found to be very safe with a low rate of hepatic abnormalities (1/650,000). In contrast, 140 trovafloxacin-treated patients developed hepatic problems, 14 of which were severe, and 8 required transplantation. The main CNS problems associated with fluoroquinolones include dizziness, convulsions, psychosis, and insomnia. Levofloxacin, ofloxacin, and moxifloxacin reportedly have the lowest potential of inducing central nervous system (CNS) adverse events among the fluoroquinolones currently available. Cardiovascular problems were seen in 1/15 million levofloxacin prescriptions compared... (truncated)

Title: Levofloxacin-induced hepatotoxicity and death.
Drug-induced hepatotoxicity is a major cause of hepatocellular injury in patients admitting to emergency services with acute liver failure. Hepatic necrosis may be at varying degrees from mild elevations in transaminases to fulminant hepatitis, and even death. The case of a 53-year-old female patient with toxic hepatitis due to levofloxacin and multiple organ failure secondary to toxic hepatitis is presented. Patient suffered itching, redness, and rash after receiving a single dose of 750 mg of levofloxacin tablets for pulmonary infection 10 days ago. Skin lesions had regressed within 3 days, but desquamation formed all over the body. After the fifth... (truncated)

Title: Hepatotoxicity Secondary to Levofloxacin Use.
Levofloxacin is a broad-spectrum antibiotic that is used in the treatment of many infections. A rare adverse drug reaction following the use of levofloxacin is drug-induced liver injury. The exact mechanism behind fluoroquinolone-induced liver injury is unknown, but many severe, sometimes fatal hepatotoxicity cases are reported. Current recommendations advise clinicians to discontinue levofloxacin immediately if the patient develops signs and symptoms of hepatitis. This case report presents a 79-year-old male who was prescribed levofloxacin 500 mg by mouth daily for seven days. The patient had a past medical history of dementia, seizures, cerebral vascular accident, pulmonary fibrosis, and chronic kidney... (truncated)

MESH:D013999 - timolol

Summary:

LLM Prediction Score	0.112 (normalized)
LLM Confidence Score	0.990
Golden Answer (Severity Class)	0.625 (normalized)
Prediction Error	0.513

Retrieved Context:

Title: A Severe Case of Drug-Induced Liver Injury after Gemcitabine Administration: A Highly Probable Causality Grading as Assessed by the Updated RUCAM Diagnostic Scoring System.
Gemcitabine is an antineoplastic drug used in several forms of advanced pancreatic, lung, breast, ovarian, and bladder cancer. Common side effects include bone marrow suppression, fatigue, diarrhea, nausea, gastrointestinal upset, rash, alopecia, and stomatitis. Transient serum enzyme elevations could be observed during therapy, but clinically significant acute liver injury has been rarely associated with its use. Few cases of acute liver injury have been reported in the literature. We reported the clinical case of a 73--year-old man who developed clinically significant acute hepatic injury after using gemcitabine. Possible causes, clinical presentation, and treatments are discussed. According to the updated RUCAM... (truncated)

Title: Non-selective beta-blockers and the incidence of hepatocellular carcinoma in patients with cirrhosis: a meta-analysis.
Background: Hepatocellular carcinoma (HCC) is a serious complication of cirrhosis. Currently, non-selective beta-blockers (NSBBs) are commonly used to treat portal hypertension in patients with cirrhosis. The latest research shows that NSBBs can induce apoptosis and S-phase arrest in liver cancer cells and inhibit the development of hepatic vascular endothelial cells, which may be effective in preventing HCC in cirrhosis patients. Aim: To determine the relationship between different NSBBs and HCC incidence in patients with cirrhosis. Methods: We searched the Cochrane database, MEDLINE, EMBASE, PubMed, and Web of Science. Cohort studies, case‒control studies, and randomized controlled trials were included if they... (truncated)

Title: Comprehensive Evaluation of Organotypic and Microphysiological Liver Models for Prediction of Drug-Induced Liver Injury.
Drug-induced liver injury (DILI) is a major concern for the pharmaceutical industry and constitutes one of the most important reasons for the termination of promising drug development projects. Reliable prediction of DILI liability in preclinical stages is difficult, as current experimental model systems do not accurately reflect the molecular phenotype and functionality of the human liver. As a result, multiple drugs that passed preclinical safety evaluations failed due to liver toxicity in clinical trials or postmarketing stages in recent years. To improve the selection of molecules that are taken forward into the clinics, the development of more predictive <i>in vitro</i>... (truncated)

Title: Intestinal cytochromes P450 regulating the intestinal microbiota and its probiotic profile.
Cytochromes P450 (CYPs) enzymes metabolize a large variety of xenobiotic substances. In this vein, a plethora of studies were conducted to investigate their role, as cytochromes are located in both liver and intestinal tissues. The P450 profile of the human intestine has not been fully characterized. Human intestine serves primarily as an absorptive organ for nutrients, although it has also the ability to metabolize drugs. CYPs are responsible for the majority of phase I drug metabolism reactions. CYP3A represents the major intestinal CYP (80%) followed by CYP2C9. CYP1A is expressed at high level in the duodenum, together with less abundant... (truncated)

Title: Generating hepatic cell lineages from pluripotent stem cells for drug toxicity screening.
Hepatotoxicity is an enormous and increasing problem for the pharmaceutical industry. Early detection of problems during the drug discovery pathway is advantageous to minimize costs and improve patient safety. However, current cellular models are sub-optimal. This review addresses the potential use of pluripotent stem cells in the generation of hepatic cell lineages. It begins by highlighting the scale of the problem faced by the pharmaceutical industry, the precise nature of drug-induced liver injury and where in the drug discovery pathway the need for additional cell models arises. Current research is discussed, mainly for generating hepatocyte-like cells rather than other liver... (truncated)

MESH:D003506 - cyclofenil

Summary:

LLM Prediction Score	0.488 (normalized)
LLM Confidence Score	0.740
Golden Answer (Severity Class)	1.0 (normalized)
Prediction Error	0.512

Retrieved Context:

Title: Hepatic reactions to cyclofenil.
Thirty patients with hepatic reactions to cyclofenil, a non-steroidal drug with a stimulating effect on ovulation, are reviewed. The liver damage was probably related to metabolic idiosyncrasy, and was reversible in all patients.

Title: Cyclofenil versus placebo in progressive systemic sclerosis. A one-year double-blind crossover study of 27 patients.
Cyclofenil was evaluated versus placebo in the treatment of progressive systemic sclerosis (PSS, scleroderma) in a 2 x 6-month double-blind crossover study. The mean duration of disease was six years. Of 38 patients entering the study, 27 completed both periods. Reasons for drop-outs were very high liver transaminases in three cases, cardiac death in two, and drug allergy, alcoholic problems, suspected congestive heart failure, reactivation of tuberculosis, arteriosclerotic heart disease, and lethal progression of PSS in one case each. No fatality was attributed to cyclofenil. Liver enzyme abnormalities were seen in 13 of 35 active drug periods and in 5... (truncated)

Title: Acute hepatitis induced by cyclofenil: a case report.
The case of a 47-year old woman suffering from acute hepatitis caused by cyclofenil, a drug proposed for the treatment of anovulation and scleroderma, is presented. Hepatitis developed seven weeks after the beginning of administration of the drug and its course was reversible after withdrawal. The case is documented on the basis of liver histology and the exclusion of other causes of acute hepatitis.

Title: Association of CYP1A1 and CYP1B1 inhibition in in vitro assays with drug-induced liver injury.
Drug-induced liver injury (DILI) is one of the major causes for the discontinuation of drug development and withdrawal of drugs from the market. Since it is known that reactive metabolite formation and being substrates or inhibitors of cytochrome P450s (P450s) are associated with DILI, we systematically investigated the association between human P450 inhibition and DILI. The inhibitory activity of 266 DILI-positive drugs (DILI drugs) and 92 DILI-negative drugs (no-DILI drugs), which were selected from Liver Toxicity Knowledge Base (US Food and Drug Administration), against 8 human P450 forms was assessed using recombinant enzymes and luminescent substrates, and the threshold values... (truncated)

Title: Mechanisms of drug toxicity and relevance to pharmaceutical development.
Toxicity has been estimated to be responsible for the attrition of approximately one-third of drug candidates and is a major contributor to the high cost of drug development, particularly when not recognized until late in clinical trials or post-marketing. The causes of drug toxicity can be classified in several ways and include mechanism-based (on-target) toxicity, immune hypersensitivity, off-target toxicity, and bioactivation/covalent modification. In addition, idiosyncratic responses are rare but can be one of the most problematic issues; several hypotheses for these have been advanced. Although covalent binding of drugs to proteins was described almost 40 years ago, the significance to... (truncated)

MESH:D010396 - penicillamine

Summary:

LLM Prediction Score	0.762 (normalized)
LLM Confidence Score	0.990
Golden Answer (Severity Class)	0.25 (normalized)
Prediction Error	0.512

Retrieved Context:

Title: Fatal aplastic anaemia and liver toxicity caused by D-penicillamine treatment of rheumatoid arthritis.
A 65 year old woman with rheumatoid arthritis developed marrow aplasia and jaundice owing to D-penicillamine treatment. Recovery of bone marrow was ineffective, and the patient finally died despite intensive therapeutic measures. The rare coexistence of myelotoxicity and hepatotoxicity is presented and discussed.

Title: Cholestatic jaundice associated with D-penicillamine therapy.
Cholestatic jaundice is a rare complication of penicillamine therapy. We report here a 35-year-old patient who developed fever, a rash and cholestatic jaundice 16 days after commencing treatment with penicillamine for cystinuria. The jaundice subsided slowly after discontinuation of the drug and with prolonged therapy with prednisone. The literature on penicillamine-induced liver injury is reviewed.

Title: Cholestatic jaundice caused by D-penicillamine.
D-penicillamine is not generally considered to cause hepatic damage. Cholestatic jaundice developed in a patient with rheumatoid arthritis 4 weeks after penicillamine was added to his regimen, and he died in acute renal failure. The probability that penicillamine caused the cholestasis is discussed.

Title: Penicillamine-induced cupriuria in normal subjects and in patients with active liver disease.
No abstract available.

Title: Hepatotoxicity associated with use of D-penicillamine in rheumatoid arthritis.
Two patients with rheumatoid arthritis developed evidence of hepatotoxicity while receiving D-penicillamine. Both recovered after withdrawal of the drug. These cases and a review of the literature suggested that hepatotoxicity, though rare, should be added to the list of adverse reactions to D-penicillamine.

MESH:D010074 - oxandrolone

Summary:

LLM Prediction Score	0.488 (normalized)
LLM Confidence Score	0.980
Golden Answer (Severity Class)	1.0 (normalized)
Prediction Error	0.512

Retrieved Context:

Title: Liver abnormalities in Turner syndrome.
We evaluated whether hepatic abnormalities represent a specific feature in girls with Turner syndrome (TS) or whether they are related to an increased susceptibility to hormonal therapies and/or other factors. Alanine aminotransferase, aspartate aminotransferase and gamma-glutamyl transferase were monitored in 70 patients with TS for a mean period of 7.6+/-4.2 years. An increase in serum liver enzymes was observed in 14 out of 70 girls (20%) at a mean age of 12.7 years; it was present at entry before hormonal therapy in 3 girls and developed thereafter during the follow up in the other 11. The increase in serum liver... (truncated)

Title: Incidence of oxandrolone induced hepatic transaminitis in patients with burn injury.
The benefits of oxandrolone in burn patients has led to its accepted use in the burn care community, however details regarding the most common adverse effect, transaminitis, remains unclear. The purpose of this study was to determine the incidence of transaminitis in patients with burn injury and identify risk factors associated with the development of transaminitis. This single-center, retrospective risk factor analysis compared burn patients on oxandrolone with and without the development of transaminitis, defined as any aspartate aminotransferase or alanine aminotransferase value >100mg/dL. Patient demographics, past medical history, lab values, and burn characteristics were recorded. Overall 28 out of... (truncated)

Title: Drug, Herb, and Dietary Supplement Hepatotoxicity.
The past decade has witnessed drugs, herbs, and dietary supplements share the common feature of potential liver injury in a few susceptible individuals.[...].

Title: Incidence of hepatic dysfunction is equivalent in burn patients receiving oxandrolone and controls.
Oxandrolone has been shown to improve lean muscle mass in patients with burns. Hepatic dysfunction is a known side effect of treatment with oxandrolone. The purpose of this study was to examine the incidence of hepatic dysfunction in our series of burn patients receiving oxandrolone. Fourteen patients who received oxandrolone (5 mg, n = 8; 10 mg, n = 6) were identified from our prospectively collected burn database. The records of 61 control patients also were reviewed. Demographics such as age, comorbidities, and burn size were recorded. The incidence of hepatic dysfunction was determined by the presence of abnormal liver... (truncated)

Title: Oxandrolone enhances hepatic ketogenesis in adult men.
Immediate administration of oxandrolone markedly increases hepatic lipase activity and reduces levels of plasma high-density lipoprotein.

MESH:D005422 - flavoxate

Summary:

LLM Prediction Score	0.511 (normalized)
LLM Confidence Score	0.870
Golden Answer (Severity Class)	0.0 (normalized)
Prediction Error	0.511

Retrieved Context:

Title: Drug-induced acute cholestatic liver damage in a patient with mutation of UGT1A1.
A 54-year-old woman presented with a 3-week history of fatigue and with jaundice that began 2 days before admission. She had been undergoing treatment with flavoxate for urinary incontinence (for 2 months before admission) and with tibolone for climacteric syndrome (for 6 months before admission). Laboratory tests revealed elevated concentrations of aminotransferases, bilirubin, gamma-glutamyltransferase and alkaline phosphatase. Liver biopsy revealed histological evidence of subacute, drug-induced liver damage.

Title: The evolution of strategies to minimise the risk of human drug-induced liver injury (DILI) in drug discovery and development.
Early identification of toxicity associated with new chemical entities (NCEs) is critical in preventing late-stage drug development attrition. Liver injury remains a leading cause of drug failures in clinical trials and post-approval withdrawals reflecting the poor translation between traditional preclinical animal models and human clinical outcomes. For this reason, preclinical strategies have evolved over recent years to incorporate more sophisticated human in vitro cell-based models with multi-parametric endpoints. This review aims to highlight the evolution of the strategies adopted to improve human hepatotoxicity prediction in drug discovery and compares/contrasts these with recent activities in our lab. The key role of... (truncated)

Title: Worldwide Use of RUCAM for Causality Assessment in 81,856 Idiosyncratic DILI and 14,029 HILI Cases Published 1993-Mid 2020: A Comprehensive Analysis.
Background: A large number of idiosyncratic drug induced liver injury (iDILI) and herb induced liver injury(HILI) cases of variable quality has been published but some are a matter of concern if the cases were not evaluated for causality using a robust causality assessment method (CAM) such as RUCAM (Roussel Uclaf Causality Assessment Method) as diagnostiinjuryc algorithm. The purpose of this analysis was to evaluate the worldwide use of RUCAM in iDILI and HILI cases. Methods: The PubMed database (1993-30 June 2020) was searched for articles by using the following key terms: Roussel Uclaf Causality Assessment Method; RUCAM; Idiosyncratic drug induced... (truncated)

Title: The Diagnosis of Drug-induced Liver Injury: Current Diagnostic Ability and Future Challenges of the Digestive Disease Week-Japan 2004 Scale 15 Years after Its Proposal.
Objective This study examined whether or not the Digestive Disease Week-Japan (DDW-J) 2004 scale proposed over 15 years ago can be applied to current cases of drug-induced liver injury (DILI). Methods The new patients group included 125 patients from 2012 to 2019 and was divided into 2 subgroups: 96 patients in the new DILI group and 29 patients in the new non-DILI group. Similarly, the old patients group included 105 patients from 1997 to 2002 and was divided into 2 subgroups: 59 patients in the old DILI group and 46 patients in the old non-DILI group. Patients were assessed by... (truncated)

Title: State of the Art and Uses for the Biopharmaceutics Drug Disposition Classification System (BDDCS): New Additions, Revisions, and Citation References.
The Biopharmaceutics Drug Disposition Classification system (BDDCS) is a four-class approach based on water solubility and extent of metabolism/permeability rate. Based on the BDDCS class to which a drug is assigned, it is possible to predict the role of metabolic enzymes and transporters on the drug disposition of a new molecular entity (NME) prior to its administration to animals or humans. Here, we report a total of 1475 drugs and active metabolites to which the BDDCS is applied. Of these, 379 are new entries, and 1096 are revisions of former classification studies with the addition of references for the approved... (truncated)

MESH:C529000 - golimumab

Summary:

LLM Prediction Score	0.490 (normalized)
LLM Confidence Score	0.930
Golden Answer (Severity Class)	1.0 (normalized)
Prediction Error	0.510

Retrieved Context:

Title: Hepatitis B virus (HBV) reactivation in patients receiving tumor necrosis factor (TNF)-targeted therapy: analysis of 257 cases.
The emergence of tumor necrosis factor-α (TNF-α)-targeted therapies as a key therapeutic option for patients with rheumatic, digestive, and dermatologic autoimmune diseases has been associated with increasing reports of liver damage in patients with hepatitis B virus (HBV) infection. We studied the current evidence on the use of anti-TNF agents in patients with HBV through a systematic analysis of cases reported in the MEDLINE and EMBASE databases using the MeSH term "hepatitis B virus" combined with the terms "infliximab," "etanercept," "adalimumab," "certolizumab," "golimumab," and "anti-TNF agents," and summarize the results here. We analyzed 257 patients with positive HBV markers who... (truncated)

Title: Description of the efficacy and safety of three new biologics in the treatment of rheumatoid arthritis.
English articles on abatacept, golimumab, and tocilizumab in rheumatoid arthritis published between 2002 and 2009 were reviewed systematically. All randomized clinical trials, open-label extensions, meta-analyses, and reviews were examined. There were thirteen articles on abatacept, four on golimumab, and seven on tocilizumab. All three drugs were effective in methotrexate-naïve, methotrexate-incomplete responders, and tumor-necrosis-factor-failure rheumatoid arthritis patients. Of the three, only abatacept has been tested in a head-to-head trial with infliximab, in which it was found to be equivalent to infliximab. Golimumab resulted in a more modest improvement than the others in methotrexate-naïve patients, although no direct comparisons among the three... (truncated)

Title: Hepatotoxicity Associated with the Use of Anti-TNF-α Agents.
Medications to inhibit the actions of tumour necrosis factor alpha have revolutionized the treatment of several pro-inflammatory autoimmune conditions. Despite their many benefits, several serious side effects exist and adverse reactions do occur from these medications. While many of the medications' potential adverse effects were anticipated and recognized in clinical trials prior to drug approval, several more rare adverse reactions were recorded in the literature as the popularity, availability and distribution of these medications grew. Of these potential adverse reactions, liver injury, although uncommon, has been observed in some patients. As case reports accrued over time and ultimately case series... (truncated)

Title: Use of tumor necrosis factor alpha inhibitors in hepatitis B surface antigen-positive patients: a literature review and potential mechanisms of action.
As a class, tumor necrosis factor (TNF)-alpha inhibitors have provided clinicians significant control over chronic inflammatory diseases. With their widespread use has come the emergence of new side effects such as the reactivation of latent infections. One such infection that may reactivate is the hepatitis B virus (HBV). It is currently unknown if HBV reactivation is a class effect or attributable to a particular TNF-alpha inhibitor. To answer this question, a comprehensive literature review to identify trends in related cases was performed. A systemic literature review was performed using the PubMed and Medline databases (1996 to January 2010) searching for... (truncated)

Title: Anti-Tumor Necrosis Factor Therapy in Intestinal Behçet's Disease.
Intestinal Behçet's disease is a rare, immune-mediated chronic intestinal inflammatory disease; therefore, clinical trials to optimize the management and treatment of patients are scarce. Moreover, intestinal Behçet's disease is difficult to treat and often requires surgery because of the failure of conventional medical treatment. Administration of anti-tumor necrosis factor-α, a potential therapeutic strategy, is currently under active clinical investigation, and evidence of its effectiveness for both intestinal Behçet's disease and inflammatory bowel diseases has been accumulating. Here, we review updated data on current experiences and outcomes after the administration of anti-tumor necrosis factor-α for the treatment of intestinal Behçet's disease.... (truncated)

MESH:C107201 - tipranavir

Summary:

LLM Prediction Score	0.491 (normalized)
LLM Confidence Score	0.970
Golden Answer (Severity Class)	1.0 (normalized)
Prediction Error	0.509

Retrieved Context:

Title: Review of tipranavir in the treatment of drug-resistant HIV.
Highly active antiretroviral therapy (HAART) has dramatically improved the prognosis of patients with HIV. Low adherence and toxicity among HIV-positive patients starting HAART, however, can lead to discontinuation of therapy and limit long-term treatment success. Moreover, increasing prevalence of primary resistance (>10%) as well as the accumulation of mutations resulting from continued selection pressure exerted by ongoing antiretroviral treatment in patients failing virologically, mean that new compounds are needed that retain antiretroviral activity against resistant strains. Tipranavir (Aptivus((R))) is a novel protease inhibitor (NPPI), which is characterized by a unique genetic resistance profile that allows it to remain active against... (truncated)

Title: Tipranavir: a novel protease inhibitor for HIV therapy.
Tipranavir (TPV) is a nonpeptidic protease inhibitor with potent in vitro activity against most HIV-1 strains resistant to other protease inhibitors. In vitro data have shown that resistance to TPV develops slowly. When coadministered with ritonavir (RTV) as a booster, TPV has shown potent antiviral activity in multiple antiretroviral-experienced patients. In the RESIST-1 and RESIST-2 studies, the efficacy and safety of TPV/RTV (500/200 mg twice daily) in highly treatment-experienced HIV-1-positive patients was assessed. Analysis at 48 weeks showed that TPV/RTV-containing regimens significantly improved immune and virological responses compared with a RTV-boosted comparator protease inhibitor plus optimized background regimen. TPV is... (truncated)

Title: MRP2 Inhibition by HIV Protease Inhibitors in Rat and Human Hepatocytes: A Quantitative Confocal Microscopy Study.
Hepatic drug transporters play a pivotal role in the excretion of drugs from the body, in drug-drug interactions, as well as in drug-induced liver toxicity. Hepatocytes cultured in sandwich configuration are an advantageous model to investigate the interactions of drug candidates with apical efflux transporters in a biorelevant manner. However, the commonly used "offline" assays (i.e., that rely on measuring intracellular accumulated amounts after cell lysis) are time- and resource-consuming, and the data output is often highly variable. In the present study, we used confocal microscopy to investigate the inhibitory effect of all marketed HIV protease inhibitors (10 <i>μ</i>M) on... (truncated)

Title: Tipranavir: a review of its use in the management of HIV infection.
Tipranavir (Aptivus) is a selective nonpeptidic HIV-1 protease inhibitor (PI) that is used in the treatment of treatment-experienced adults with HIV-1 infection. Tipranavir is administered orally twice daily and must be given in combination with low-dose ritonavir, which is used to boost its bioavailability. The durable efficacy of tipranavir, in combination with low-dose ritonavir (tipranavir/ritonavir 500 mg/200 mg twice daily), has been demonstrated in well designed trials in treatment-experienced adults infected with multidrug-resistant strains of HIV-1. In treatment-experienced adults with HIV-1 infection receiving an optimized background regimen, viral suppression was greater and immunological responses were better with regimens containing tipranavir/ritonavir... (truncated)

Title: Role of tipranavir in treatment of patients with multidrug-resistant HIV.
The worldwide emergence of multidrug-resistant human immunodeficiency virus (HIV)-1 strains has the driven the development of new antiretroviral (ARV) agents. Over the past 5 years, HIV-entry and integrase inhibitor ARVs, as well as improved non-nucleoside reverse transcriptase inhibitors (NRTIs) and protease inhibitors (PIs), have become available for treatment. It is important to assess how these new ARVs might be most judiciously used, paying close attention to viral susceptibility patterns, pharmacodynamic parameters, and the likelihood that patients will adhere to their therapy. Herein we review published material in Medline, EMBASE, and ISI for each antiretroviral agent/classes currently approved and summarize the... (truncated)

MESH:C057619 - glimepiride

Summary:

LLM Prediction Score	0.367 (normalized)
LLM Confidence Score	0.960
Golden Answer (Severity Class)	0.875 (normalized)
Prediction Error	0.508

Retrieved Context:

Title: Cholestatic liver injury after glimepiride therapy.
Drug induced hepatotoxicity has been reported infrequently with sulfonylureas. For glimepiride, a second-generation sulfonylurea there is no report of hepatotoxicity in English literature. A patient with non-insulin-dependent diabetes mellitus who developed cholestatic liver injury soon after initiation of glimepiride therapy is presented. Complete work-up disclosed no other cause for hepatotoxicity including negative serological results for viral hepatitis. Liver biopsy was consistent with drug-induced cholestasis. The patient recovered 50 days after stopping glimepiride with no further recurrences.

Title: Comparison of Tofogliflozin and Glimepiride Effects on Nonalcoholic Fatty Liver Disease in Participants With Type 2 Diabetes: A Randomized, 48-Week, Open-Label, Active-Controlled Trial.
Nonalcoholic fatty liver disease (NAFLD) is a liver phenotype of type 2 diabetes and obesity. Currently, the efficacy of sodium-glucose cotransporter 2 (SGLT2) inhibitors and sulfonylureas in liver pathology and hepatic gene expression profiles for type 2 diabetes with NAFLD are unknown.

Title: In vitro platforms for evaluating liver toxicity.
The liver is a heterogeneous organ with many vital functions, including metabolism of pharmaceutical drugs and is highly susceptible to injury from these substances. The etiology of drug-induced liver disease is still debated although generally regarded as a continuum between an activated immune response and hepatocyte metabolic dysfunction, most often resulting from an intermediate reactive metabolite. This debate stems from the fact that current animal and in vitro models provide limited physiologically relevant information, and their shortcomings have resulted in "silent" hepatotoxic drugs being introduced into clinical trials, garnering huge financial losses for drug companies through withdrawals and late stage... (truncated)

Title: Comparative study of Dapagliflozin versus Glimepiride effect on insulin regulated aminopeptidase (IRAP) and interleukin-34 (IL-34) in patient with type 2 diabetes mellitus.
Type 2 diabetes mellitus (T2DM) is one of the most common diseases, that managed by several medications such as Glimepiride and Dapagliflozin. This study aims to compare the effects of Dapagliflozin versus Glimepiride on glycemic control, insulin resistance, and biomarkers as (extracellular domain of insulin regulated aminopeptidase) IRAPe, (interleukin-34) IL-34, and (N-terminal pro b-type natriuretic peptide) NT-proBNP. This study included 60 type 2 diabetic patients, who are randomized to receive either Glimepiride 4 mg/day (group 1) or Dapagliflozin 10 mg/day (group 2). Blood samples were collected at baseline and after 3 months of treatment for biochemical analysis. Additionally, HOMA-IR is... (truncated)

Title: Dapagliflozin plus saxagliptin add-on to metformin reduces liver fat and adipose tissue volume in patients with type 2 diabetes.
To assess the effects of dapagliflozin plus saxagliptin plus metformin versus glimepiride plus metformin on liver fat (proton density fat fraction) and visceral and subcutaneous adipose tissue volumes over 52 weeks of treatment.

MESH:D000077603 - nandrolone decanoate

Summary:

LLM Prediction Score	0.370 (normalized)
LLM Confidence Score	0.980
Golden Answer (Severity Class)	0.875 (normalized)
Prediction Error	0.505

Retrieved Context:

Title: Nandrolone Decanoate: Use, Abuse and Side Effects.
<i>Background and Objectives:</i> Androgens play a significant role in the development of male reproductive organs. The clinical use of synthetic testosterone derivatives, such as nandrolone, is focused on maximizing the anabolic effects and minimizing the androgenic ones. Class II anabolic androgenic steroids (AAS), including nandrolone, are rapidly becoming a widespread group of drugs used both clinically and illicitly. The illicit use of AAS is diffused among adolescent and bodybuilders because of their anabolic proprieties and their capacity to increase tolerance to exercise. This systematic review aims to focus on side effects related to illicit AAS abuse, evaluating the scientific literature... (truncated)

Title: Androgen trial in renal anaemia.
A double blind cross-over trial of Nandrolone decanoate (Decadurabolin) was carried out in 27 patients with anaemia due to end stage renal disease, stabilised on regular haemodialysis. Sixteen patients completed the study, the other patients being excluded from the final analysis for a variety of reasons including side effects related to the androgen. There was no sustained significant rise in haemoglobin concentration or in red cell mass. Erythropoietin levels did not alter, they were within or below the normal range, but were lower than would be expected for the degree of anaemia. A majority of patients reported increased well-being including... (truncated)

Title: Effects of nandrolone decanoate on telomere length and clinical outcome in patients with telomeropathies: a prospective trial.
Androgens have been reported to elongate telomeres in retrospective and prospective trials with patients with telomeropathies, mainly with bone marrow failure. In our single-arm prospective clinical trial (clinicaltrials gov. Identifier: NCT02055456), 17 patients with short telomeres and/or germline pathogenic variants in telomere biology genes associated with at least one cytopenia and/or radiologic diagnosis of interstitial lung disease were treated with 5 mg/kg of intramuscular nandrolone decanoate every 15 days for 2 years. Ten of 13 evaluable patients (77%) showed telomere elongation at 12 months by flow-fluorescence in situ hybridization (average increase, 0.87 kb; 95% confidence interval: 0.20-1.55 kb; P=0.01). At... (truncated)

Title: Influence of nandrolone decanoate administration on serum lipids and liver enzymes in rats.
Anabolic-androgenic steroids have been associated with several side effects range. This experimental study was conducted to evaluate the effects of nandrolone decanoate (ND, an anabolic steroid) on lipid profile and liver enzymes in rats in Iran.

Title: Lipid Profile Changes Induced by Chronic Administration of Anabolic Androgenic Steroids and Taurine in Rats.
<i>Background and Objectives</i>: Anabolic androgenic steroids (AAS), used as a therapy in various diseases and abused in sports, are atherogenic in supraphysiological administration, altering the plasma lipid profile. Taurine, a conditionally-essential amino acid often used in dietary supplements, was acknowledged to delay the onset and progression of atherogenesis, and to mitigate hyperlipidemia. The aim of the present study was to verify if taurine could prevent the alterations induced by concomitant chronic administration of high doses of AAS nandrolone decanoate (DECA) in rats. <i>Materials and Methods</i>: Thirty-two male Wistar rats, assigned to 4 equal groups, were treated for 12 weeks either... (truncated)

MESH:D015735 - mifepristone

Summary:

LLM Prediction Score	0.502 (normalized)
LLM Confidence Score	0.990
Golden Answer (Severity Class)	0.0 (normalized)
Prediction Error	0.502

Retrieved Context:

Title: Mifepristone: An Uncommon Cause of Drug-Induced Liver Injury.
Mifepristone is frequently used in large doses for management of Cushing's syndrome. This is a case of a 35-year-old woman with Cushing's syndrome, who presented with abdominal pain and jaundice. A month prior to admission, she had been started on a daily dose of 1,200 mg mifepristone. After evaluating for various other causes of liver injury, biopsy revealed cholestatic pattern of liver disease, likely associated with drug-induced hepatotoxicity. Mifepristone was discontinued and her symptoms resolved. We believe this is one of the first few reported cases of drug-induced liver injury (DILI) associated with mifepristone use.

Title: Progesterone antagonists and progesterone receptor modulators in the treatment of breast cancer.
Progesterone antagonists (PAs) (antiprogestins) or progesterone receptor modulators (PRMs) form an interesting category of new hormonal agents in the treatment of breast cancer. In vitro, antiproliferative effects of different PAs are mainly observed in estrogen-stimulated growth of PR-positive tumor cell lines. Both progestin agonist/antagonist actions on mammary tumor cells are dependent on the type of cell line, culture medium and concentrations of the PAs used, and type of biologic response measured. In various experimental animal tumor models, different PAs showed a greater antitumor activity than tamoxifen or high-dose progestins. Most interestingly, combination treatment of different PAs (mifepristone, ORG 31710, onapristone)... (truncated)

Title: Correction to: Mifepristone: An Uncommon Cause of Drug-Induced Liver Injury.
[This corrects the article DOI: 10.14740/gr1188.].

Title: Thirty-day rat toxicity study reveals reversible liver toxicity of mifepristone (RU486) and metapristone.
Mifepristone (RU486) is an oral first-line contraceptive used by hundreds of millions of women, and recently it was tested for anticancer activity in both genders worldwide. We are developing metapristone (the N-monodemethyl RU486) as a potential metastasis chemopreventive. The present acute and 30-d subacute toxicity study aimed at examining and compared in parallel the potential toxicity of the two drugs.

Title: Effects of Mifepristone on Nonalcoholic Fatty Liver Disease in a Patient with a Cortisol-Secreting Adrenal Adenoma.
Cushing syndrome (CS), a complex, multisystemic condition resulting from prolonged exposure to cortisol, is frequently associated with nonalcoholic fatty liver disease (NAFLD). In patients with adrenal adenoma(s) and NAFLD, it is essential to rule out coexisting endocrine disorders like CS, so that the underlying condition can be properly addressed. We report a case of a 49-year-old woman with a history of hypertension, prediabetes, dyslipidemia, biopsy-confirmed steatohepatitis, and benign adrenal adenoma, who was referred for endocrine work-up for persistent weight gain. Overt Cushing features were absent. Biochemical evaluation revealed nonsuppressed cortisol on multiple 1-mg dexamethasone suppression tests, suppressed adrenocorticotropic hormone, and... (truncated)

MESH:D014750 - vincristine

Summary:

LLM Prediction Score	0.499 (normalized)
LLM Confidence Score	0.990
Golden Answer (Severity Class)	1.0 (normalized)
Prediction Error	0.501

Retrieved Context:

Title: [Complications of antitumor and antileukemic chemotherapy. 1].
The recent development of chemotherapy in the treatment of cancer and leukemia requires that all practitioners involved have a thorough knowledge of the sometimes life-threatening side-effects of chemotherapeutic agents. All these agents, whether used alone or in a combination, carry a risk because of their lack of specificity which make active on normal cells, especially those with a rapid turn-over such as the hematopoietic cells or the cells of the digestive tract. Prior to the prescription of a chemotherapeutic regimen, the acceptable risk must always be clearly defined, according to the seriousness of the disease and to the patient's age,... (truncated)

Title: Fatal hepatitis following irradiation and vincristine.
The increasing use of combination therapy with irradiation and cytostatics in malignant disease increases the risk of toxicity. An account is given of a patient with liver toxicity terminating fatally, in whom increased radiosensitivity may have been induced by vincristine.

Title: [Post-anesthetic hepatitis. The role of halothane and antimitotic combinations].
A 12 year old boy with Burkitt's lymphoma developed severe hepatitis with hepatomegaly, subclinical jaundice, and a small rise in body temperature, associated with an important rise in SGPT and fall in prothrombin titres, 6 days after anticancer chemotherapy and 24 hours after halothane anaesthesia. Hepatitis A and B serology remained negative. This hepatic failure explained perhaps the unusually severe vincristine toxicity which gave rise to a polyneuritis with important sequelae. The association of halothane hepatitis with antimitotic drugs appeared particularly dangerous, and halothane should probably be avoided in all patients been given or about to be given anticancer chemotherapy.

Title: Hepatotoxicity following vincristine therapy.
A patient is reported with small cell lung cancer treated with combination chemotherapy (cyclophosphamide, vincristine and etoposide [VP-16-213] who developed transient liver function abnormalities secondary to vincristine therapy. Serum transaminase (SGOT and SGPT) levels rose by 2 to 6 times, lactic dehydrogenase (LDH) 1.5 to 2 times, and alkaline phosphatase and gamma-glutamyl transpeptidase (GGTP) 1.5 to 2 times normal. Enzyme abnormalities were observed by the 6th day following drug administration and returned to normal between 16 and 48 days, except for the GGTP elevations which persisted longer. Vincristine has been suspected to cause liver damage and to enhance radiation-induced hepatic... (truncated)

Title: Age is a risk factor for chemotherapy-induced hepatopathy with vincristine, dactinomycin, and cyclophosphamide.
To evaluate the spectrum of and determine the risk factors for the development of liver toxicity (hepatopathy) after therapy with vincristine, dactinomycin, and cyclophosphamide (VAC) for rhabdomyosarcoma in children and adolescents.

MESH:D012110 - reserpine

Summary:

LLM Prediction Score	0.501 (normalized)
LLM Confidence Score	0.980
Golden Answer (Severity Class)	0.0 (normalized)
Prediction Error	0.501

Retrieved Context:

Title: Aspects of tolerability of centrally acting antihypertensive drugs.
Traditional centrally acting antihypertensives have been associated with a high incidence of adverse effects and are no longer recommended as first-line therapy. The newer imidazoline receptor agonists must overcome this reputation if they are to gain recognition as potential first-line agents for hypertension. Methyldopa, a centrally acting alpha(2)-agonist, is characterized by a number of serious adverse reactions that limit its use. Although unpredictable idiosyncratic or hypersensitivity reactions are uncommon, these include hepatitis, myocarditis, and hemolytic anaemia. Less serious problems such as abnormal liver function tests, positive Coombs test, drug-induced fever, and pancreatitis also occur. Central side effects include drowsiness, fatigue,... (truncated)

Title: An Antioxidant Extract of the Insectivorous Plant Drosera burmannii Vahl. Alleviates Iron-Induced Oxidative Stress and Hepatic Injury in Mice.
Free iron typically leads to the formation of excess free radicals, and additional iron deposition in the liver contributes to the oxidative pathologic processes of liver disease. Many pharmacological properties of the insectivorous plant Drosera burmannii Vahl. have been reported in previous studies; however, there is no evidence of its antioxidant or hepatoprotective potential against iron overload. The antioxidant activity of 70% methanolic extract of D. burmannii (DBME) was evaluated. DBME showed excellent DPPH, hydroxyl, hypochlorous, superoxide, singlet oxygen, nitric oxide, peroxynitrite radical and hydrogen peroxide scavenging activity. A substantial iron chelation (IC50 = 40.90 ± 0.31 μg/ml) and supercoiled... (truncated)

Title: [Liver damage caused by adelphan].
Hepatic damage due to Adelphan or to one of its two components (Rauwolfia alkaloids [Reserpin] and 1,4-dihydrazinophthalazine [Nepresol]) has not thus far been described. A patient is presented who repeatedly reacted to Adelphan with fever, chills and hepatic damage. Adelphan should therefore be added to the list of drugs which may cause hepatic damage.

Title: Ayurvedic medicine- Not always a safe bet.
Ayurvedic medicine, a traditional system of medicine practiced in the Indian subcontinent is considered to be devoid of adverse effects. We report three cases which highlight the possibility of adverse events with the use of ayurvedic products. A 35 years old woman with hepatitis took ayurvedic powders and had her liver injury worsen, possibly due to alkaloids, and developed nephrotic syndrome, possibly due to gold salt. A 57 years old hypertensive man was taking ayurvedic medicine containing reserpine which had long been withdrawn from the allopathic system of medicine due to wide range of side effects. A 47 years old... (truncated)

Title: Wild Edible Fruit of Prunus nepalensis Ser. (Steud), a Potential Source of Antioxidants, Ameliorates Iron Overload-Induced Hepatotoxicity and Liver Fibrosis in Mice.
The antioxidant and restoration potentials of hepatic injury by Prunus nepalensis Ser. (Steud), a wild fruit plant from the Northeastern region of India, were investigated. The fruit extract (PNME) exhibited excellent antioxidant and reducing properties and also scavenged the 2,2-diphenyl-1-picrylhydrazyl (DPPH) radical (IC50 = 30.92 ± 0.40 μg/ml). PNME demonstrated promising scavenging potency, as assessed by the scavenging of different reactive oxygen and nitrogen species. Moreover, the extract revealed an exceptional iron chelation capacity with an IC50 of 25.64 ± 0.60 μg/ml. The extract induced significant improvement of hepatic injury and liver fibrosis against iron overload induced hepatotoxicity in mice... (truncated)

MESH:D000069285 - infliximab

Summary:

LLM Prediction Score	0.500 (normalized)
LLM Confidence Score	0.990
Golden Answer (Severity Class)	1.0 (normalized)
Prediction Error	0.500

Retrieved Context:

Title: Toxic hepatitis induced by infliximab in a patient with rheumatoid arthritis with no relapse after switching to etanercept.
We present a case of toxic hepatitis related to infliximab treatment in a 38-year-old woman with rheumatoid arthritis (RA). The patient had previously been treated with different disease-modifying drugs (DMARDs) alone or in combination but had never revealed signs of liver dysfunction. Due to high disease activity, treatment with infliximab (3 mg/kg i.v.) was initiated in combination with methotrexate (MTX) (25 mg/week) and folic acid (5 mg/week). The patient stopped MTX and folic acid on her own initiative after 3 weeks due to improvement of joint symptoms. After seven infusions, progressive elevations of the transaminases up to five times the... (truncated)

Title: Infliximab (IFX)-Biosimilar Induced Drug-Induced Liver Injury (DILI): A Case Report.
Infliximab (IFX) is a chimeric human-murine monoclonal antibody that prevents tumor necrosis factor alpha (TNF-α) activation by binding to both soluble and transmembrane forms of TNF-α. Antagonists of TNF (anti-TNF agents) can cause drug-induced liver injury (DILI). We present a non-anti-TNF naïve patient suffering from severe Crohn's disease who developed DILI with a hepatocellular pattern, without jaundice, after two infusions of an IFX biosimilar.

Title: Infliximab-related hepatitis: discussion of a case and review of the literature.
Despite its rarity, infliximab-related hepatitis constitutes a cutting edge and challenging problem. In December 2004, a drug warning was issued by the Food and Drug Administration to alert healthcare professionals to the risk of hepatotoxicity in course of infliximab therapy. Subsequently, several reports of probable infliximab hepatitis have been published and interest is growing in trying to elucidate the impact of these events on clinical practice. After discussing our case report, the main characteristics of infliximab-mediated liver injury are analyzed, coupled with a review of the medical literature. Infliximab seems to provoke both immunomediated and a direct liver injury, but... (truncated)

Title: Successful treatment with etanercept in a patient with hepatotoxicity closely related to infliximab.
To date, hepatotoxicity with anti-TNF therapy has been associated with concomitant liver-toxicity drugs, infection or malignant diseases. We report the case of one patient with spondyloarthropathty who presented severe liver dysfunction related to infliximab. After the second infusion serum controls showed an slightly increase of transaminases. Before the administration of fifth infusion, infliximab therapy was stopped due to severe liver damage (AST 327 mU/ml, ALT 656 mU/mL, GGT 140 mU/mL, alkaline phosphate 227 mU/mL). Ten weeks after infliximab discontinuation serum concentrations of liver blood tests were normal but ankylosing spondylitis symptoms had relapsed. Therefore, he was treated with etanercept with... (truncated)

Title: Drug induced autoimmune hepatitis and TNF-α blocking agents: is there a real relationship?
Hepatotoxicity is an expected side effect of tumour necrosis factor-α (anti-TNF-α) blocking agents including, infliximab, etanercept and adalimumab. Although mild to moderate elevations of liver enzymes have been recognised after the use of these agents, severe hepatitis is rarely reported. Reactivation of viral hepatitis and drug induced liver injury is two main causes of liver dysfunction in these patients. A broad spectrum, ranging from minor immunological alterations to systemic autoimmune disease, has been reported during treatment with anti-TNF-α. Therefore, in recent studies TNF-α blocking agents have been considered a potential cause of drug induced autoimmune hepatitis. Taking into account the... (truncated)

MESH:C106538 - abacavir

Summary:

LLM Prediction Score	0.500 (normalized)
LLM Confidence Score	0.990
Golden Answer (Severity Class)	1.0 (normalized)
Prediction Error	0.500

Retrieved Context:

Title: Abacavir-induced liver toxicity.
Abacavir-induced liver toxicity is a rare event almost exclusively occurring in HLA B*5701-positive patients. Herein, we report one case of abnormal liver function tests occurring in a young HLA B*5701-negative woman on a stable nevirapine-based regimen with no history of liver problems or alcohol abuse after switching to abacavir from tenofovir. We also investigated the reasons for abacavir discontinuation in a cohort of patients treated with abacavir-lamivudine-nevirapine.

Title: Abacavir-induced liver toxicity in an HIV-infected patient.
No abstract available.

Title: Abacavir-induced fulminant hepatic failure in a HIV/HCV co-infected patient.
Abacavir hypersensitivity is a rare, yet significant adverse reaction that results in a spectrum of physical and laboratory abnormalities, and has been postulated to stem from a variety of aetiological factors. The major histocompatibility complex haplotype human leucocyte antigen (HLA)-B5701 is a significant risk factor in development of hypersensitivity reactions, yet only 55% of HLA-B5701+ individuals develop such reactions, suggesting a multifactorial aetiology. Nevertheless, prospective screening and avoidance of abacavir in these patients has limited adverse events. Within this spectrum of adverse events, abacavir-induced liver toxicity is exceedingly rare and reported events have ranged from mild elevations of aminotransferases to... (truncated)

Title: Abacavir/Dolutegravir/Lamivudine (Triumeq)-Induced Liver Toxicity in a Human Immunodeficiency Virus-Infected Patient.
Drug-induced liver injury related to Triumeq (abacavir/lamivudine/dolutegravir) has not been reported in clinical trials. We report a case of hepatotoxicity related to Triumeq exposure in a human immunodeficiency virus-infected patient. Clinicians should remain aware of the risk for acute and late-onset hepatitis with these agents. Close monitoring is recommended.

Title: Hepatotoxicity of antiretroviral therapy.
Hepatotoxicity is a serious complication in patients taking HAART. Coinfection with hepatitis viruses increases the risk of liver toxicity while taking antiretroviral therapy. Baseline transaminases should be checked before beginning antiretorviral therapy and all patients should be screened for pre-existing liver disease, most notably hepatitis B and C infections. Regular monitoring of transaminases is mandatory when commencing antiretroviral therapy. In patients with normal liver function, transaminases may be checked monthly after commencing HAART for the first 3 months. If stable this can be broadened to 3 month intervals. In patients with pre-existing liver disease monitoring should be performed more frequently... (truncated)

MESH:D013806 - theophylline

Summary:

LLM Prediction Score	0.500 (normalized)
LLM Confidence Score	0.990
Golden Answer (Severity Class)	0.0 (normalized)
Prediction Error	0.500

Retrieved Context:

Title: Theophylline.
Theophylline (3-methyxanthine) has been used to treat airway diseases for over 70 years. It was originally used as a bronchodilator but the relatively high doses required are associated with frequent side effects, so its use declined as inhaled β_2-agonists became more widely used. More recently it has been shown to have anti-inflammatory effects in asthma and COPD at lower concentrations. The molecular mechanism of bronchodilatation is inhibition of phosphodiesterase(PDE)3 and PDE4, but the anti-inflammatory effect may be due to histone deacetylase (HDAC) activation, resulting in switching off of activated inflammatory genes. Through this mechanism theophylline also reverses corticosteroid resistance and... (truncated)

Title: Markedly prolonged theophylline half-life in liver failure.
Theophylline toxicity developed within 48 hours of admission in a 63-year-old alcoholic male who presented with shortness of breath, edema, and lethargy. Using liquid chromatography, a serum theophylline half-life of 134 hours was observed following discontinuation of the drug. This report emphasizes the effect of hepatic disease on theophylline clearance and the need for frequent monitoring of plasma theophylline concentrations in cirrhotic patients.

Title: A prospective clinical study of theophylline safety in 3810 elderly with asthma or COPD.
A large-scale prospective study was conducted in 3810 Japanese elderly (> or =65 years old) patients with asthma or chronic obstructive pulmonary disease (COPD) who had been treated with sustained-release theophylline tablets (THEODUR) at a dose of 400 mg/day for 1-6 months, in principle. Among 3798 protocol-complying patients (mean age: 73.8 +/- 0.10 years, 1997 with COPD), 261 theophylline-related adverse events were observed in 179 (4.71%) patients. The 5 most frequently observed adverse events were "nausea" (40 episodes, 1.05%), "loss of appetite" (22 episodes, 0.56%), "hyperuricemia" (16 episodes, 0.42%), "palpitation" (15 episodes, 0.39%), and "increased alkaline phosphatase" (11 episodes, 0.28%).... (truncated)

Title: [Psychiatric symptoms due to theophylline overdose; the necessity of blood level monitoring].
Two patients presented with severe side effects of theophylline. Due to the theophylline intoxication patient A developed ischaemic hepatitis, which made haemodialysis treatment necessary to accelerate the elimination of the drug from the body. The intoxication caused immobilisation and total dependence with respect to the daily activities of patient B. In neither case was the association between mood disorders--like depression, agitation and anxiety--and an overdose of theophylline recognised. Administration of theophylline should be monitored with regular measurements of the serum theophylline concentration, because of its narrow therapeutic margin and the serious toxic side effects.

Title: Evaluation of the potential for drug-induced liver injury based on in vitro covalent binding to human liver proteins.
Prediction of idiosyncratic drug-induced liver injury (DILI) is difficult, and the underlying mechanisms are not fully understood. However, many drugs causing DILI are considered to form reactive metabolites and covalently bind to cellular macromolecules in the liver. The objective of this study was to clarify whether the risk of idiosyncratic DILI can be estimated by comparing in vitro covalent binding (CB) levels among 12 positive compounds (acetaminophen, alpidem, bromfenac, carbamazepine, diclofenac, flutamide, imipramine, nefazodone, tacrine, ticlopidine, tienilic acid, and troglitazone) for DILI and 12 negative compounds (acetylsalicylic acid, caffeine, dexamethasone, losartan, ibuprofen, paroxetine, pioglitazone, rosiglitazone, sertraline, theophylline, venlafaxine, and zolpidem).... (truncated)

MESH:D000074323 - alemtuzumab

Summary:

LLM Prediction Score	0.500 (normalized)
LLM Confidence Score	0.980
Golden Answer (Severity Class)	0.0 (normalized)
Prediction Error	0.500

Retrieved Context:

Title: Acute severe hepatitis with alemtuzumab and rechallenge after a year.
Alemtuzumab is a monoclonal antibody used as a disease modifying agent in relapsing and remitting multiple sclerosis. It has not previously been associated with drug induced liver injury. Here we present a case of a 49 year old female developing drug induced liver injury secondary to alemtuzumab, confirmed upon rechallenge. Our patient developed severe hepatitis within two days of starting alemtuzumab, both initially and upon rechallenge. The alanine aminotransferase peaked at 577 units per litre and 426 units per litre after initial dose of alemtuzumab and rechallenge respectively. The patient's liver function tests improved significantly between doses of alemtuzumab and... (truncated)

Title: The Disease-Modifying Therapies of Relapsing-Remitting Multiple Sclerosis and Liver Injury: A Narrative Review.
In this narrative review, we analyze pre-registration and post-marketing data concerning hepatotoxicity of all disease-modifying therapies (DMTs) available for the treatment of relapsing-remitting multiple sclerosis, including beta interferon, glatiramer acetate, fingolimod, teriflunomide, dimethyl fumarate, cladribine, natalizumab, alemtuzumab, and ocrelizumab. We review the proposed causal mechanisms described in the literature and we also address issues like use of DMTs in patients with viral hepatitis or liver cirrhosis. Most data emerged in the post-marketing phase by reports to national pharmacovigilance agencies and published case reports or case series. Serious liver adverse events are rare, but exact incidence is largely unknown, as are... (truncated)

Title: The use of immune modulating drugs for the treatment of multiple sclerosis.
This review discusses the mechanisms of action of 4 immune modulating drugs currently used in the treatment of multiple sclerosis (MS), including Alemtuzumab, a humanized monoclonal antibody that functions by targeting CD52, an antigen primarily expressed on T and B lymphocytes and monocytes/macrophages, resulting in their depletion and subsequent repopulation; Dimethyl fumarate that switches cytokine production toward a T helper 2 profile and enhances cytosolic levels of nuclear factor erythroid 2-related factor 2, which has immune regulatory and cytoprotective effects on oligodendrocytes, neurons, and glial cells; Fingolimod functions by blocking the release of activated lymphocytes from lymph nodes by targeting... (truncated)

Title: Phase I-II study of clofarabine-melphalan-alemtuzumab conditioning for allogeneic hematopoietic cell transplantation.
We conducted a phase I-II study of transplantation conditioning with clofarabine-melphalan-alemtuzumab for patients with advanced hematologic malignancies. Ten patients were accrued to the phase I portion, which utilized an accelerated titration design. No dose-limiting toxicity was observed, and clofarabine 40 mg/m(2) × 5, melphalan 140 mg/m(2) × 1, and alemtuzumab 20 mg × 5 was adopted for the phase II study, which accrued 72 patients. Median age was 54 years. There were 44 patients with acute myelogenous leukemia or myelodysplastic syndromes, 27 with non-Hodgkin lymphoma, and nine patients with other hematologic malignancies. The largest subgroup of 35 patients had American... (truncated)

Title: Hepatitis C virus reactivation in cancer patients in the era of targeted therapies.
The purpose of this review is to summarize the evidence of hepatitis C reactivation in cancer patients in the era of targeted therapies. Targeted therapies are novel therapeutics frequently used in cancer patients. During treatment with targeted therapies, viral replication is one of the major problems that can occur. The PubMed database, ASCO, and ASCO Gastrointestinal Cancer Symposium abstracts were searched up until September 15, 2013 using the following search keywords: "targeted therapies, rituximab, alemtuzumab, brentuximab, hepatitis, hepatitis C reactivation, tyrosine kinase inhibitors, imatinib, mammalian target of rapamycin (mTOR) inhibitors, everolimus, anti-HER therapies, trastuzumab, pertuzumab, lapatinib, anti-epidermal growth factor receptor... (truncated)

MESH:D000069454 - darunavir

Summary:

LLM Prediction Score	0.500 (normalized)
LLM Confidence Score	0.990
Golden Answer (Severity Class)	1.0 (normalized)
Prediction Error	0.500

Retrieved Context:

Title: [Safety and tolerability of etravirine].
Etravirine (ETR) is the first representative of a new generation of non-nucleoside reverse transcriptase inhibitors (NNRTI) and is indicated in patients with HIV infection and virological failure. The recommended dose is 200 mg (two tablets) every 12 hours after a meal. ETR has good tolerability and the tablets can be dissolved in water, which can aid swallowing in some patients. This drug has a plasma half-life of 30-40 hours and consequently is a candidate for once-daily regimens. The most frequent adverse effect is rash (affecting 19% of patients), which is usually mild (grades 1 or 2) and does not lead... (truncated)

Title: [Safety and tolerability of darunavir].
Darunavir, previously known as TMC-114, is a new protease inhibitor (PI) with a high affinity for the HIV-1 protease and strong ability to inhibit its action, even in mutated forms. Consequently, this drug is considered to have great intrinsic potency and a high genetic barrier. At the time of writing, data on the tolerability and safety of darunavir come mainly from studies of late rescue therapy (POWER, DUET), which have included more than 1,600 patients. Recent data, relating to shorter time periods, are also available from studies in early treatment-experienced patients (TITAN) and in treatment-naïve patients (ARTEMIS), increasing experience to... (truncated)

Title: The Effect of Rifampicin on Darunavir, Ritonavir, and Dolutegravir Exposure within Peripheral Blood Mononuclear Cells: a Dose Escalation Study.
Ritonavir-boosted darunavir (DRV/r) and dolutegravir (DTG) are affected by induction of metabolizing enzymes and efflux transporters caused by rifampicin (RIF). This complicates the treatment of people living with HIV (PLWH) diagnosed with tuberculosis. Recent data showed that doubling DRV/r dose did not compensate for this effect, and hepatic safety was unsatisfactory. We aimed to evaluate the pharmacokinetics of DRV, ritonavir (RTV), and DTG in the presence and absence of RIF in peripheral blood mononuclear cells (PBMCs). PLWH were enrolled in a dose-escalation crossover study with 6 treatment periods of 7 days. Participants started with DRV/r 800/100 mg once daily (QD), RIF and... (truncated)

Title: Low risk of liver toxicity using the most recently approved antiretroviral agents but still increased in HIV-hepatitis C virus coinfected patients.
Liver enzyme elevations (LEE) were investigated in 2717 episodes of initiation of antiretroviral therapy since January 2010 in 1982 HIV patients. Serum hepatitis C virus (HCV)-RNA was positive in 24%. Any grade of LEE was recognized in 9% of episodes, being 6% in HCV-negative and 17% in HCV-positive patients ($P < 0.001$). Grades 3-4 LEE only occurred in 0.4% of patients. Overall, LEE were more frequent with ritonavir-boosted darunavir and atazanavir than with raltegravir and etravirine.

Title: [Darunavir in HIV/HVC/HVB coinfection].
Darunavir/ritonavir is indicated in combination with other antiretroviral drugs for the treatment of HIV-1 infection in pre-treated adult patients. In hepatitis B or C co-infected patients, the virological response rate to darunavir/ritonavir appeared to be unaffected and, except for increased liver enzymes, the incidence of adverse events was not higher than in patients without co-infection. Drug-induced hepatitis has been reported in 0.5% of patients receiving combination therapy with darunavir/ritonavir. Patients with pre-existing liver dysfunction, including chronic active hepatitis B or C, have an increased risk for liver function abnormalities including severe hepatic adverse events. Therefore AST/ALT monitoring should be considered... (truncated)

MESH:C101425 - posaconazole

Summary:

LLM Prediction Score	0.500 (normalized)
LLM Confidence Score	0.980
Golden Answer (Severity Class)	1.0 (normalized)
Prediction Error	0.500

Retrieved Context:

Title: Posaconazole: An Update of Its Clinical Use.
Posaconazole (PCZ) is a relatively new addition to the azole antifungals. It has fungicidal activities against <i>Aspergillus fumigatus</i>, <i>Blastomyces dermatitidis</i>, selected <i>Candida</i> species, <i>Crytopcoccus neoformans</i>, and <i>Trichosporon</i>. PCZ also has fungistatic activities against <i>Candida</i>, <i>Coccidioides</i>, selected <i>Fusarium</i> spp., <i>Histoplasma</i>, <i>Scedosporium</i> and <i>Zygomycetes.</i> In addition, combining the drug with caspofungin or amphotericin B results in a synergistic interaction against <i>A. fumigatus</i>, <i>C. glabrata</i> and <i>C. neoformans</i>. The absorption of PCZ suspension is enhanced when given with food, nutritional supplements, and carbonated beverages. Oral administration of PCZ in divided doses also increases its bioavailability. PCZ has a large volume of distribution and... (truncated)

Title: Posaconazole (Noxafil): a new triazole antifungal agent.
Posaconazole is the newest triazole antifungal agent. It is structurally related to itraconazole and has activity against Candida species, Aspergillus species, Cryptococcus neoformans, the zygomycetes, and other filamentous fungi. Randomized, double-blind trials have shown posaconazole to be at least as efficacious as fluconazole for the prevention of invasive fungal infections in immunocompromised patients. It has also shown promising results in the treatment of various fungal infections refractory to other antifungal therapy. The dose of posaconazole is 200 mg orally three times daily for the prevention of invasive fungal infections and 800 mg daily in two to four divided doses for... (truncated)

Title: Posaconazole prophylaxis during induction therapy of patients with acute lymphoblastic leukaemia.
Novel treatment schedules of induction therapy for acute lympoblastic leukaemia (ALL) use combinations of immunosuppressive and cytotoxic drugs that are associated with neutropenia and acquisition of invasive fungal infections. It has been described that posaconazole, a triazole antifungal drug, is active against a variety of Candida and Aspergillus species in vitro. Moreover, large clinical trials using posaconazole in severely immunosuppressed patients provided data on efficacy against Aspergillus in vivo. As patients with ALL are also affected by difficult-to-treat Aspergillus infections, we conducted a pilot study to prove the safety of posaconazole in patients undergoing intensified induction phase treatment. We report... (truncated)

Title: Posaconazole achieves prompt recovery of voriconazole-induced liver injury in a case of invasive aspergillosis.
Azole antifungals have frequently been linked to the presence of hepatotoxicity, but there is scarce information on cross-toxicity between these drugs or on the possibility of using some of them when this type of toxicity occurs. We report the case of a 64-year-old man with invasive aspergillosis (IA) leading to spondylodiscitis with neurological involvement. Early management included intravenous (iv) voriconazole, which had to be interrupted after 1 week due to liver damage. Therapeutic drug monitoring (TDM) of voriconazole showed that the plasma concentration was within the therapeutic range. However, it was replaced by a combination therapy of oral posaconazole plus... (truncated)

Title: Hepatotoxicity Due to Azole Antimycotic Agents in a HLA B*35:02-Positive Patient.
We will present a 42-year-old woman with acute myeloid leukemia and pulmonary aspergillosis. She was treated with several antifungal agents, including three triazoles. Voriconazole, posaconazole, and isavuconazole all led to hepatocellular liver injury. Voriconazole administration led to a peak alanine aminotransferase (ALT) value of 1,793 U/L (normal range, 9-59 U/L). After posaconazole and isavuconazole treatment, ALT rose over 500 U/L. The typical course of events, exclusion of differential diagnoses, and normalization of the liver function tests (LFTs) after stopping the triazoles were highly suspicious for a drug-induced liver injury (DILI). Interestingly, our patient carries a rare HLA B allele (HLA... (truncated)

MESH:C029892 - cupric chloride

Summary:

LLM Prediction Score	0.500 (normalized)
LLM Confidence Score	0.980
Golden Answer (Severity Class)	0.0 (normalized)
Prediction Error	0.500

Retrieved Context:

Title: In vitro cytotoxicity assay with selected chemicals using human cells to predict target-organ toxicity of liver and kidney.
In order to elucidate the feasibility of predicting liver and kidney target-organ toxicity using in vitro cytotoxicity assay, cytotoxicity of selected chemicals, acetaminophen (AAP), mitomycin (MMC), cupric chloride (CuCl2), phenacetin, cadmium chloride (CdCl2) and aristolochic acid (AA), was studied using human hepatoma (Bel-7402) cells and human renal tubular epithelial (HK-2) cells. Cell viability and mitochondrial permeability transition (MPT) were assessed by the neutral red (NR) assay and laser scanning confocal microscope, respectively. The results of the NR assay indicated that cytotoxicity of hepatoxicants, AAP, MMC and CuCl2 in liver cells was higher than that in kidney cells. Cytotoxicitiy of nephrotoxicant,... (truncated)

Title: Subchronic toxicity of copper oxide nanoparticles and its attenuation with the help of a combination of bioprotectors.
In the copper metallurgy workplace air is polluted with condensation aerosols, which a significant fraction of is presented by copper oxide particles<100 nm. In the scientific literature, there is a lack of their in vivo toxicity characterization and virtually no attempts of enhancing organism's resistance to their impact. A stable suspension of copper oxide particles with mean (±SD) diameter 20±10 nm was prepared by laser ablation of pure copper in water. It was being injected intraperitoneally to rats at a dose of 10 mg/kg (0.5 mg per mL of deionized water) three times a week up to 19 injections. In... (truncated)

Title: Comparative toxicity and biodistribution of copper nanoparticles and cupric ions in rats.
Despite widespread use and prospective biomedical applications of copper nanoparticles (Cu NPs), their biosafety issues and kinetics remain unclear. Thus, the aim of this study was to compare the detailed in vivo toxicity of Cu NPs and cupric ions (CuCl2; Cu ions) after a single oral dose. We determined the physicochemical characteristics of Cu NPs, including morphology, hydrodynamic size, zeta potential, and dissolution in gastric (pH 1.5), vehicle (pH 6.5), and intestinal (pH 7.8) conditions. We also evaluated the kinetics of Cu following a single equivalent dose (500 mg/kg) of Cu NPs and Cu ions. Cu NPs had highest dissolution... (truncated)

Title: Mitigating the Growth, Biochemical Changes, Genotoxic and Pathological Effects of Copper Toxicity in Broiler Chickens by Supplementing Vitamins C and E.
This experiment was carried out to explore the efficiency of an individual or combined doses of vitamin C (Vit. C) and vitamin E (Vit. E) in alleviating biochemical, genotoxicity, and pathological changes in the liver induced by copper sulfate (CuSO₄) toxicity in broiler chickens. Two hundred and fifty-one-day-old broiler chicks were haphazardly allotted into five groups (five replicates/group, ten chicks/replicate). The birds were fed five experimental diets; (1) basal diet with no additives (CON), (2) basal diets supplemented with 300 mg CuSO₄/kg diet (CuSO₄), (3) basal diets supplemented with 300 mg CuSO₄/kg diet + 250 mg Vit. C /kg diet,... (truncated)

Title: Micronuclei in bone marrow and liver in relation to hepatic metabolism and antioxidant response due to coexposure to chloroform, dichloromethane, and toluene in the rat model.
Genotoxicity in cells may occur in different ways, direct interaction, production of electrophilic metabolites, and secondary genotoxicity via oxidative stress. Chloroform, dichloromethane, and toluene are primarily metabolized in liver by CYP2E1, producing reactive electrophilic metabolites, and may also produce oxidative stress via the uncoupled CYP2E1 catalytic cycle. Additionally, GSTT1 also participates in dichloromethane activation. Despite the oxidative metabolism of these compounds and the production of oxidative adducts, their genotoxicity in the bone marrow micronucleus test is unclear. The objective of this work was to analyze whether the oxidative metabolism induced by the coexposure to these compounds would account for increased... (truncated)

MESH:C087123 - romidepsin

Summary:

LLM Prediction Score	0.500 (normalized)
LLM Confidence Score	0.970
Golden Answer (Severity Class)	0.0 (normalized)
Prediction Error	0.500

Retrieved Context:

Title: Romidepsin: a new therapy for cutaneous T-cell lymphoma and a potential therapy for solid tumors.
Romidepsin is a histone deacetylase inhibitor (HDI), approved by the US FDA for the treatment of cutaneous T-cell lymphoma (CTCL). Although various mechanisms have been proposed for the activity of HDIs, including induction of genes controlling cell cycle, acetylation of cytoplasmic proteins and direct induction of apoptosis, the mechanism underlying activity of romidepsin and other HDIs in CTCL is not known. Romidepsin induces long-lasting responses. The side-effect profile is similar to that of other HDIs, causing fatigue, nausea and thrombocytopenia. Management of the CTCL population requires vigilence to prevent infection with skin contaminants, and monitoring of potassium and magnesium, electrolytes... (truncated)

Title: Pharmacokinetics and Immunological Effects of Romidepsin in Rhesus Macaques.
HIV/SIV persistence in latent reservoirs requires lifelong antiretroviral treatment and calls for effective cure strategies. Romidepsin (RMD), a histone deacetylase inhibitor, was reported to reactivate HIV/SIV from reservoirs in virus-suppressed individuals. We characterized in detail the pharmacokinetics and safety profile of RMD in three SIV-naïve rhesus macaques which received two rounds of treatment. In plasma, RMD mean terminal half-life was 15.3 h. In comparison, RMD mean terminal half-life was much longer in tissues: 110 h in the lymph nodes (LNs) and 28 h in gastrointestinal tract. RMD administration was accompanied by transient liver and systemic toxicity. Isoflurane anesthesia induced near-immediate transient lymphopenia, which... (truncated)

Title: Romidepsin and lenalidomide-based regimens have efficacy in relapsed/refractory lymphoma: Combined analysis of two phase I studies with expansion cohorts.
Romidepsin (histone deacetylase inhibitor), lenalidomide (immunomodulatory agent), and carfilzomib (proteasome inhibitor), have efficacy and lack cumulative toxicity in relapsed/refractory lymphoma. We performed two investigator initiated sequential phase I studies to evaluate the maximum tolerated dose (MTD) of romidepsin and lenalidomide (regimen A) and romidepsin, lenalidomide, and carfilzomib (regimen B) in relapsed/refractory lymphoma. Cohorts in T-cell lymphoma (TCL), B-cell lymphoma (BCL) were enrolled at the MTD. Forty-nine patients were treated in study A (27 TCL, 17 BCL, 5 Hodgkin lymphoma (HL)) and 27 (16 TCL, 11 BCL) in study B. The MTD of regimen A was romidepsin 14 mg/m² IV on days... (truncated)

Title: Protein-driven mechanism of multiorgan damage in COVID-19.
We propose a new plausible mechanism by mean of which SARS-CoV-2 produces extrapulmonary damages in severe COVID-19 patients. The mechanism consist on the existence of vulnerable proteins (VPs), which are (i) mainly expressed outside the lungs; (ii) their perturbations is known to produce human diseases; and (iii) can be perturbed directly or indirectly by SARS-CoV-2 proteins. These VPs are perturbed by other proteins, which are: (i) mainly expressed in the lungs, (ii) are targeted directly by SARS-CoV-2 proteins, (iii) can navigate outside the lungs as cargo of extracellular vesicles (EVs); and (iv) can activate VPs via subdiffusive processes inside the... (truncated)

Title: Romidepsin (FK228) in a Mouse Model of Lipopolysaccharide-Induced Acute Kidney Injury is Associated with Down-Regulation of the CYP2E1 Gene.
BACKGROUND Romidepsin (FK228) or depsipeptide, is a selective inhibitor of histone deacetylase 1 (HDAC1) and HDAC2. This study aimed to investigate the effects and molecular mechanisms of romidepsin (FK228) in a mouse model of acute kidney injury (AKI) induced by lipopolysaccharide (LPS). MATERIAL AND METHODS The mouse model of AKI was developed by intraperitoneal injection of LPS. The mice were also treated intraperitoneally with romidepsin (FK228) six hours following injection of LPS. Markers of renal injury were measured, including blood urea nitrogen (BUN), serum creatinine (SCR), and serum cystatin C (Cys C) were measured. Histology and transmission electron microscopy were... (truncated)

MESH:D019386 - alendronate

Summary:

LLM Prediction Score	0.500 (normalized)
LLM Confidence Score	0.980
Golden Answer (Severity Class)	0.0 (normalized)
Prediction Error	0.500

Retrieved Context:

Title: Extrahepatic complications of chronic cholestasis: current diagnosis and treatment.
Pruritus, fatigue and osteoporosis are the main symptoms of the extra hepatic manifestations of chronic cholestasis that affect patients' quality of life. Pruritus affects more often female patients, varies as intensity during a day and for longer period of time, typically can be localized on the palms of hands and soles of feet or can be generalized. Pruritus can be treated with anions resines exchange--cholestiramine, the pregnanne X receptor agonist Rifampicine, Naltrexone. Liver transplantation can be considered if severe pruritus remains refractory to all medical treatments. Fatigue is the most disabling complain in chronic colestasis. No specific therapies are available... (truncated)

Title: Evaluation and Management of Chronic Cholestatic Liver Diseases.
Cholestasis is defined as stagnation or a marked reduction in bile secretion and flow. Cholestatic jaundice can thus be classified as intrahepatic or extrahepatic cholestatic, depending on the level of obstruction to bile flow. It is important to recognize the complications of cholestatic in patients with chronic cholestatic liver disease. The two most common complications of cholestasis are pruritus and fatigue, with the former being the most responsive to treatment. Cholestyramine is the first-line treatment for cholestatic pruritus. Rifampicin and oral opioid antagonist naltrexone are extremely effective second-line treatments. To date, there are no specific treatments for chronic cholestatic fatigue... (truncated)

Title: Hepatotoxicity induced by alendronate therapy.
Here we describe a 47-year-old postmenopausal woman who had been taking alendronate 70 mg/week for osteoporosis. After two months of alendronate therapy, she developed hepatotoxicity, and no other etiological factors for this besides the alendronate were apparent. After the alendronate therapy was discontinued, the patient's hepatic enzyme levels slowly returned to normal. Hepatotoxicity due to alendronate therapy is a rare but possible adverse effect.

Title: [Treatment for hepatic osteodystrophy].
Chronic liver diseases, including liver cirrhosis, are caused by various pathogenesis, such as viral hepatitis, primary biliary cirrhosis, autoimmune hepatitis and steatohepatitis. There have not been enough clinical evidence about the treatment of hepatic osteodystrophy at the present time. Several reports suggested that bisphosphonates, such as alendronate, are effective for an increase in bone mineral density in patients with chronic liver disease. Vitamin D treatment might be useful for the frequent prevalence of vitamin D deficiency in the pathogenesis of hepatic oseodystrophy. The use of estrogens will be limited for the risk of liver dysfunction and hepatocellular carcinoma.

Title: Hepatitis after alendronate.
A 77 year-old woman developed severe hepatitis. No cause was found apart from alendronate medication. The hepatitis resolved after alendronate was stopped.

MESH:D017964 - itraconazole

Summary:

LLM Prediction Score	0.500 (normalized)
LLM Confidence Score	0.990
Golden Answer (Severity Class)	1.0 (normalized)
Prediction Error	0.500

Retrieved Context:

Title: Hepatotoxicity related to itraconazole: report of three cases.
The antimycotic synthetic azole compounds are known to lead to toxic liver injury. The occurrence of acute hepatitis is best known for ketoconazole. With itraconazole, hepatotoxic reactions have only very rarely been reported, and histologic data are lacking. We report on three patients who developed acute liver damage during therapy with itraconazole, and in whom liver biopsy specimens were obtained.

Title: Hepatic failure related to itraconazole use successfully treated by corticosteroids.
Itraconazole is believed to carry a low risk of hepatic toxicity owing to its low affinity for the human P-450 enzyme. Therefore, hepatic failure caused by itraconazole is exceedingly rare.

Title: Comparative Hepatotoxicity of Fluconazole, Ketoconazole, Itraconazole, Terbinafine, and Griseofulvin in Rats.
Oral ketoconazole was recently the subject of regulatory safety warnings because of its association with increased risk of inducing hepatic injury. However, the relative hepatotoxicity of antifungal agents has not been clearly established. The aim of this study was to compare the hepatotoxicity induced by five commonly prescribed oral antifungal agents. Rats were treated with therapeutic oral doses of griseofulvin, fluconazole, itraconazole, ketoconazole, and terbinafine. After 14 days, only ketoconazole had significantly higher ALT levels (<i>p</i> = 0.0017) and AST levels (<i>p</i> = 0.0008) than the control group. After 28 days, ALT levels were highest in the rats treated with... (truncated)

Title: Itraconazole and fluconazole and certain rare, serious adverse events.
To determine rates of drug-induced, rare, serious adverse events affecting the liver, kidneys, skin, or blood, occurring within 45 days of completing a prescription or refill for itraconazole or fluconazole.

Title: [Liver damage during administration of itraconazole (Trisporal)].
Three case histories are described of patients, two women aged 62 and 57 and a man aged 75 years, who developed symptomatic hepatic injury five to six weeks after starting itraconazole treatment. In two of them the biochemical pattern of liver injury was cholestatic. Other causes of hepatic injury were excluded. Monitoring serum liver enzymes is advisable in patients treated with itraconazole for one month or longer.

MESH:D064730 - dexrazoxane

Summary:

LLM Prediction Score	0.000 (normalized)
LLM Confidence Score	0.960
Golden Answer (Severity Class)	0.5 (normalized)
Prediction Error	0.500

Retrieved Context:

Title: The Role of Flavonoids as a Cardioprotective Strategy against Doxorubicin-Induced Cardiotoxicity: A Review.
Doxorubicin is a widely used and promising anticancer drug; however, a severe dose-dependent cardiotoxicity hampers its therapeutic value. Doxorubicin may cause acute and chronic issues, depending on the duration of toxicity. In clinical practice, the accumulative toxic dose is up to 400 mg/m² and increasing the dose will increase the probability of cardiac toxicity. Several molecular mechanisms underlying the pathogenesis of doxorubicin cardiotoxicity have been proposed, including oxidative stress, topoisomerase beta II inhibition, mitochondrial dysfunction, Ca²⁺ homeostasis dysregulation, intracellular iron accumulation, ensuing cell death (apoptosis and necrosis), autophagy, and myofibrillar disarray and loss. Natural products including flavonoids have been widely... (truncated)

Title: Treatment of Drug-Induced Liver Injury.
Current pharmacotherapy options of drug-induced liver injury (DILI) remain under discussion and are now evaluated in this analysis. Needless to say, the use of the offending drug must be stopped as soon as DILI is suspected. Normal dosed drugs may cause idiosyncratic DILI, and drugs taken in overdose commonly lead to intrinsic DILI. Empirically used but not substantiated regarding efficiency by randomized controlled trials (RCTs) is the intravenous antidote treatment with N-acetylcysteine (NAC) in patients with intrinsic DILI by N-acetyl-p-aminophenol (APAP) overdose. Good data recommending pharmacotherapy in idiosyncratic DILI caused by hundreds of different drugs are lacking. Indeed, a recent... (truncated)

Title: Triptolide impairs thioredoxin system by suppressing Notch1-mediated PTEN/Akt/Txnip signaling in hepatocytes.
Triptolide (TP) is the main ingredient of Chinese herb Tripterygium wilfordii Hook f. (TWHF). Despite of its multifunction in pharmaceutics, accumulating evidences showed that TP caused obvious hepatotoxicity in clinic. The current study investigated the role of Notch1 signaling in TP-induced hepatotoxicity. Our data indicated that TP inhibited the protein expression of Notch1 and its active form Notch intracellular domain (NICD) leading to increased PTEN (phosphatase and tensin homolog deleted on chromosome ten) expression. Moreover, PTEN triggered Txnip (thioredoxin-interacting protein) activation by inhibiting Akt phosphorylation, which resulted in reduction of Trx (thioredoxin). In conclusion, TP caused liver injury through initiating... (truncated)

Title: Dexrazoxane improves cardiac autonomic function in epirubicin-treated breast cancer patients with type 2 diabetes.
The study objective was to investigate the protective effects of dexrazoxane (DRZ) on the cardiac autonomic nervous system (ANS) activity in anthracycline-treated breast cancer patients with diabetes.

Title: Deleterious effects of reactive metabolites.
A number of drugs have been withdrawn from the market or severely restricted in their use because of unexpected toxicities that become apparent only after the launch of new drug entities. Circumstantial evidence suggests that, in most cases, reactive metabolites are responsible for these unexpected toxicities. In this review, a general overview of the types of reactive metabolites and the consequences of their formation are presented. The current approaches to evaluate bioactivation potential of new compounds with particular emphasis on the advantages and limitation of these procedures will be discussed. Reasonable reasons for the excellent safety record of certain drugs... (truncated)

MESH:D007649 - ketamine

Summary:

LLM Prediction Score	0.500 (normalized)
LLM Confidence Score	0.990
Golden Answer (Severity Class)	0.0 (normalized)
Prediction Error	0.500

Retrieved Context:

Title: Cholestasis and biliary dilatation associated with chronic ketamine abuse: a case series.
Ketamine is a dissociative anaesthetic agent that is still widely used in veterinary and human medicine. It is increasingly being used as a recreational hallucinogenic drug. Chronic ketamine abuse is known to account for lower urinary tract symptoms and urinary bladder dysfunction. There is now emerging evidence that ketamine misuse is also associated with abnormal liver function tests and biliary tract abnormality. We report three cases of chronic ketamine misuse in three young men who all presented with obstructive jaundice and biliary tract abnormality. We also describe the clinical features, radiological findings and potential underlying mechanisms for this new entity.

Title: Dilated common bile duct and deranged liver function tests associated with ketamine use in two HIV-positive MSM.
We report here the first two cases of hepatobiliary pathology in HIV-positive men following recreational use of ketamine: >1 g/day over a 12-month period while on ritonavir-based antiretroviral therapy. Presentation in each case was acute with nausea, vomiting and epigastric pain. Alanine aminotransferase was raised at 3.2× and 10.1 × upper limit of normal and alkaline phosphatase was raised at 1.7× and 2.5 × ULN for cases 1 and 2, respectively. Magnetic resonance cholangiopancreatography showed dilatation of the common bile duct; case 1, 18 mm and case 2, 14 mm with no ductal obstruction on endoscopic retrograde cholangiopancreatography. The symptoms... (truncated)

Title: Drug-induced liver injury following a repeated course of ketamine treatment for chronic pain in CRPS type 1 patients: a report of 3 cases.
Studies on the efficacy of ketamine in the treatment of chronic pain indicate that prolonged or repetitive infusions are required to ensure prolonged pain relief. Few studies address ketamine-induced toxicity. Here we present data on the occurrence of ketamine-induced liver injury during repeated administrations of S(+)-ketamine for treatment of chronic pain in patients with complex regional pain syndrome type 1 as part of a larger study exploring possible time frames for ketamine re-administration. Six patients were scheduled to receive 2 continuous intravenous 100-hour S(+)-ketamine infusions (infusion rate 10-20mg/h) separated by 16 days. Three of these patients developed hepatotoxicity. Patient A,... (truncated)

Title: Ketamine hydrochloride as sole anesthetic for open liver biopsy.
We evaluated the use of ketamine as sole anesthetic agent for open liver biopsy, with particular reference to its effect on liver function and hepatotoxicity and its effect on cardiovascular stability and respiration. From 386 patients who underwent liver biopsy at Jordan University Hospital, 12 had open liver biopsy because of contra-indications for closed needle biopsy. The surgical procedure consisted of a small right paramedian incision allowing inspection of the liver surface and a wedge and needle biopsy. Ketamine HCl was used in a dose of 2 mg/kg I.V. with supplemental doses as necessary. No significant fluctuations in cardio-respiratory vital... (truncated)

Title: Persistent Ketamine-Induced Cholangiopathy: An Approach to Management.
A 32-year-old man presented with profound jaundice, rigors and decreased appetite. Initial liver function tests (LFTs) were deranged in a cholestatic pattern with imaging demonstrating a dilated biliary system, with no filling defects. It has been observed that LFTs typically improve upon ketamine cessation, but this case demonstrated escalating hyperbilirubinaemia, despite ketamine cessation. Recurrent cholangitis and biliary duct stricturing were demonstrated on magnetic resonance cholangiopancreatography (MRCP). This prompted investigation of other biliary pathology and consideration for intervention.

MESH:D000077337 - vorinostat

Summary:

LLM Prediction Score	0.500 (normalized)
LLM Confidence Score	0.980
Golden Answer (Severity Class)	0.0 (normalized)
Prediction Error	0.500

Retrieved Context:

Title: Vorinostat and hydroxychloroquine improve immunity and inhibit autophagy in metastatic colorectal cancer.
Hydroxychloroquine (HCQ) enhances the anti-cancer activity of the histone deacetylase inhibitor, vorinostat (VOR), in pre-clinical models and early phase clinical studies of metastatic colorectal cancer (mCRC). Mechanisms could include autophagy inhibition, accumulation of ubiquitinated proteins, and subsequent tumor cell apoptosis. There is growing evidence that autophagy inhibition could lead to improved anti-cancer immunity. To date, effects of autophagy on immunity have not been reported in cancer patients. To address this, we expanded an ongoing clinical study to include patients with advanced, refractory mCRC to evaluate further the clinical efficacy and immune effects of VOR plus HCQ. Refractory mCRC patients received... (truncated)

Title: Update on the treatment of cutaneous T-cell lymphoma (CTCL): Focus on vorinostat.
Epigenetic regulation of gene transcription by small molecule inhibitors of histone deacetylases (HDAC) is a novel cancer therapy. Vorinostat (Zolinza()) is the first FDA approved HDAC-inhibitor for treatment of patients with cutaneous T cell lymphoma (CTCL) who have progressive, persistent or recurrent disease on or following two systemic therapies. Vorinostat was active against solid tumors and hematologic malignancies as intravenous and oral preparations in Phase I development. In two Phase II trials, vorinostat was safe and effective at an oral dose of 400 mg/day with an overall response rate of 24%-30% in refractory advanced patients with CTCL including large cell... (truncated)

Title: A phase I study of vorinostat in combination with idarubicin in relapsed or refractory leukaemia.
Histone deacetylase inhibitors (HDACi) affect chromatin remodelling and modulate the expression of aberrantly silenced genes. HDACi have single-agent clinical activity in haematological malignancies and have synergistic anti-leukaemia activity when combined with anthracyclines in vitro. We conducted a two-arm, parallel Phase I trial to investigate two schedules of escalating doses of vorinostat (Schedule A: thrice daily (TID) for 14 d; B: TID for 3 d) in combination with a fixed dose of idarubicin in patients with refractory leukaemia. Of the 41 patients enrolled, 90% had acute myeloid leukaemia, with a median of 3 prior therapies. Seven responses (17%) were documented (two... (truncated)

Title: Amelioration of high-fat diet (HFD) + CCl4 induced NASH/NAFLD in CF-1 mice by activation of SIRT-1 using cinnamoyl sulfonamide hydroxamate derivatives: in-silico molecular modelling and in-vivo prediction.
Non-alcoholic fatty liver disease (NAFLD) is one of the major hepatic metabolic disorders that occurs because of the accumulation of lipids in hepatocytes in the form of free fatty acids (FFA) and triglycerides (TG) which become non-alcoholic steatohepatitis (NASH). NOTCH-1 receptors act as novel targets for the development of NAFLD/NASH, where overexpression of NOTCH-1 receptor alters the lipid metabolism in hepatocytes leading to NAFLD. SIRT-1 deacetylates the NOTCH-1 receptor and inhibits NAFLD. Hence, computer-aided drug design (CADD) was used to check the SIRT-1 activation ability of cinnamic sulfonyl hydroxamate derivatives (NMJ 1-8), resveratrol, and vorinostat. SIRT-1 (PDB ID: 5BTR) was... (truncated)

Title: Effectiveness of an <i>O</i>-Alkyl Hydroxamate in Dogs with Naturally Acquired Canine Leishmaniosis: An Exploratory Clinical Trial.
Canine leishmaniosis is a challenge in veterinary medicine and no drug to date has achieved parasite clearance in dogs. Histone deacetylase inhibitors are a drug class widely used in cancer chemotherapy. We have successfully used <i>O</i>-alkyl hydroxamates (vorinostat derivatives) in the treatment of a laboratory model of visceral leishmaniasis without showing toxicity. In order to test the effectiveness of a particular compound, MTC-305, a parallel-group, randomized, single-centre, exploratory study was designed in naturally infected dogs. In this clinical trial, 18 dogs were allocated into 3 groups and were treated with either meglumine antimoniate (104 mg Sb^V/kg), MTC-305 (3.75 mg/kg) or... (truncated)

MESH:D000077866 - clofarabine

Summary:

LLM Prediction Score	0.500 (normalized)
LLM Confidence Score	0.960
Golden Answer (Severity Class)	1.0 (normalized)
Prediction Error	0.500

Retrieved Context:

Title: A phase I trial of high-dose clofarabine, etoposide, and cyclophosphamide and autologous peripheral blood stem cell transplantation in patients with primary refractory and relapsed and refractory non-Hodgkin lymphoma.
Clofarabine has significant single-agent activity in patients with indolent and aggressive non-Hodgkin lymphoma and synergizes with DNA-damaging drugs. Treatment, however, may be associated with severe and prolonged myelosuppression. We conducted a phase I trial to determine the maximum tolerated dose (MTD) of clofarabine in combination with high-dose etoposide and cyclophosphamide followed by autologous peripheral blood stem cell transplantation in patients with refractory non-Hodgkin lymphoma (NHL). Patients received clofarabine at 30-70 mg/m(2)/day on days -6 to -2 in successive cohorts, in combination with etoposide 60 mg/kg (day -8), and cyclophosphamide 100 mg/kg (day -6), followed by filgrastim-mobilized PBSC on day 0.... (truncated)

Title: Impact of pharmacokinetics on the toxicity and efficacy of clofarabine in patients with relapsed or refractory acute myeloid leukemia.
Common side effects of clofarabine (CFB) are liver toxicity, particularly a transient elevation of transaminases and skin toxicity. We studied the correlation of pharmacokinetic (PK) parameters with these toxicities and the efficacy of CFB in patients with relapsed or refractory acute myeloid leukemia. Clofarabine PK parameters showed large inter-individual variability. A higher CFB area under the curve was significantly associated with higher transaminase levels ($p = .011$ for aspartate aminotransferase (AST), adjusted for age, sex, cumulated CFB dosage, baseline AST, and glomerular filtration rate (GFR)). No significant association could be found between maximum concentration and the liver toxicity parameters. The... (truncated)

Title: Fatal skin and liver toxicity in a patient treated with clofarabine.
No abstract available.

Title: Clofarabine, cyclophosphamide and etoposide as single-course re-induction therapy for children with refractory/multiple relapsed acute lymphoblastic leukaemia.
The safety and efficacy of the combination clofarabine/cyclophosphamide/etoposide were evaluated in children with advanced acute lymphoblastic leukaemia (ALL). The study enrolled 25 paediatric patients (median age 12.5 years) with either refractory ($n = 17$; 68%) or multiple relapsed ($n = 8$; 32%) ALL to receive clofarabine 40 mg/m(2), cyclophosphamide 400 mg/m(2) and etoposide 150 mg/m(2), daily for 5 consecutive days. No patient died from treatment-related complications. The most common adverse events were febrile neutropenia, mucositis and reversible liver toxicity; no case of liver veno-occlusive disease was reported. The overall remission rate was 56%: 13 patients (52%) achieved complete remission (CR)... (truncated)

Title: Cytarabine and clofarabine after high-dose cytarabine in relapsed or refractory AML patients.
Clofarabine has been shown to be effective in AML patients, either as single agent or, mainly, in association with intermediate dose cytarabine. Based on these reports, we conducted a preliminary study combining clofarabine and intermediate dose cytarabine in AML patients who relapsed or failed to respond to at least two induction therapies. We treated 47 patients affected by relapsed/refractory AML with a regimen including clofarabine at 22.5 mg/m(2) daily on days 1-5, followed after 3 hr by cytarabine at 1 g/m(2) daily on days 1-5. Ten patients received a further consolidation cycle with clofarabine at 22.5 mg/m(2) and cytarabine at... (truncated)

MESH:C032587 - olsalazine

Summary:

LLM Prediction Score	0.500 (normalized)
LLM Confidence Score	0.910
Golden Answer (Severity Class)	1.0 (normalized)
Prediction Error	0.500

Retrieved Context:

Title: Comparative tolerability of treatments for inflammatory bowel disease.
Despite limited understanding of therapeutic aetiopathogenesis of ulcerative colitis and Crohn's disease, there is a strong evidence base for the efficacy of pharmacological and biological therapies. It is equally important to recognise toxicity of the medical armamentarium for inflammatory bowel disease (IBD). Sulfasalazine consists of sulfapyridine linked to 5-aminosalicylic acid (5-ASA) via an azo bond. Common adverse effects related to sulfapyridine 'intolerance' include headache, nausea, anorexia, and malaise. Other allergic or toxic adverse effects include fever, rash, haemolytic anaemia, hepatitis, pancreatitis, paradoxical worsening of colitis, and reversible sperm abnormalities. The newer 5-ASA agents were developed to deliver the active ingredient... (truncated)

Title: Sulfasalazine desensitization in children and adolescents with chronic inflammatory bowel disease.
Sulfasalazine is an important therapeutic agent in the management of chronic inflammatory bowel disease (CIBD). Unfortunately, adverse reactions to this drug have been reported in 5-55% of treated patients. These include dose-related side effects like nausea, malaise, and headache or hypersensitivity reactions such as rash, fever, hives, arthralgia, hepatitis, etc. Studies in adults with successful reintroduction of sulfasalazine after a desensitization program have been reported; however, with regard to children, no such data are available. Fourteen children and adolescents (5-16 yr old) diagnosed to have CIBD manifested hypersensitivity to sulfasalazine within 2 months of onset of treatment. All had pancolitis--secondary... (truncated)

Title: The Effect of Pregnancy and Inflammatory Bowel Disease on the Pharmacokinetics of Drugs Related to Inflammatory Bowel Disease-A Systematic Literature Review.
Due to ethical and practical reasons, a knowledge gap exists on the pharmacokinetics (PK) of inflammatory bowel disease (IBD)-related drugs in pregnant women with IBD. Before evidence-based dosing can be proposed, insight into the PK has to be gained to optimize drug therapy for both mother and fetus. This systematic review aimed to describe the effect of pregnancy and IBD on the PK of drugs used for IBD. One aminosalicylate study, two thiopurine studies and twelve studies with biologicals were included. Most drugs within these groups presented data over multiple moments before, during and after pregnancy, except for mesalazine, ustekinumab... (truncated)

Title: Risk-stratified monitoring for thiopurine toxicity in immune-mediated inflammatory diseases: prognostic model development, validation, and, health economic evaluation.
Patients established on thiopurines (e.g., azathioprine) are recommended to undergo three-monthly blood tests for the early detection of blood, liver, or kidney toxicity. These side-effects are uncommon during long-term treatment. We developed a prognostic model that could be used to inform risk-stratified decisions on frequency of monitoring blood-tests during long-term thiopurine treatment, and, performed health-economic evaluation of alternate monitoring intervals.

Title: The role of mesalamine in the treatment of ulcerative colitis.
Ulcerative colitis (UC) is a chronic inflammatory condition of unclear etiology affecting the large bowel, most commonly the rectum and extending proximally in a continuous fashion. The overall principle in the pathophysiolgy of ulcerative colitis is the dysregulation of the normal immune system against an antigenic trigger leading to a prolonged mucosal inflammatory response. The diagnosing of UC is made by combining the clinical picture, tissue biopsy with the endoscopic appearance of mucosal ulceration, friable, edematous, erythematous granular appearing mucus. The approach to therapy of UC has been dependent on severity of symptoms with frontline therapy being salicylate based sulfasalazine.... (truncated)

MESH:D000068538 - dutasteride

Summary:

LLM Prediction Score	0.500 (normalized)
LLM Confidence Score	0.990
Golden Answer (Severity Class)	0.0 (normalized)
Prediction Error	0.500

Retrieved Context:

Title: Detection of Anti-mitochondrial Antibodies Accompanied by Drug-induced Hepatic Injury due to Atorvastatin.
A 44-year-old Japanese woman was admitted to our hospital with fatigue and an altered liver function. She had been receiving atorvastatin treatment for 10 months. Although no jaundice was seen, the patient's serum alkaline phosphatase and γ-glutamyl transpeptidase levels were markedly elevated. Based on the results of a drug-induced lymphocyte-stimulation test, her liver disease was diagnosed as atorvastatin-induced hepatic injury. Subsequently, anti-mitochondrial antibodies (AMAs) were detected in her serum; however, a liver biopsy specimen did not show the characteristic features of primary biliary cholangitis. We herein report the detection of AMAs accompanied by drug-induced hepatic injury caused by atorvastatin.

Title: Effects of testosterone administration (and its 5-alpha-reduction) on parenchymal organ volumes in healthy young men: findings from a dose-response trial.
Animal data shows that testosterone administration increases the volume of some parenchymal organs. However, the effects of exogenous testosterone on solid abdominal organs in humans remain unknown. The present study evaluated the effects of testosterone administration on the volume of liver, spleen and kidneys in a dose-response trial. Young healthy men aged 18-50 years participating in the 5α-Reductase (5aR) Trial. All participants received monthly injections of 7.5 mg leuprolide acetate to suppress endogenous testosterone secretion and weekly injections of 50, 125, 300 or 600 mg of testosterone enanthate, and were randomized to receive either 2.5 mg dutasteride (5 α-reductase inhibitor) or placebo daily for... (truncated)

Title: Sex hormone influence on hepatitis in young male A/JCr mice infected with Helicobacter hepaticus.
Hepatitis B virus (HBV), the leading cause of human hepatocellular carcinoma, is especially virulent in males infected at an early age. Likewise, the murine liver carcinogen Helicobacter hepaticus is most pathogenic in male mice infected before puberty. We used this model to investigate the influence of male sex hormone signaling on infectious hepatitis. Male A/JCr mice were infected with H. hepaticus or vehicle at 4 weeks and randomized into surgical and pharmacologic treatment groups. Interruption of androgen pathways was confirmed by hormone measurements, histopathology, and liver gene and Cyp4a protein expression. Castrated males and those receiving the competitive androgen receptor... (truncated)

Title: Dual-5α-Reductase Inhibition Promotes Hepatic Lipid Accumulation in Man.
5α-Reductase 1 and 2 (SRD5A1, SRD5A2) inactivate cortisol to 5α-dihydrocortisol in addition to their role in the generation of DHT. Dutasteride (dual SRD5A1 and SRD5A2 inhibitor) and finasteride (selective SRD5A2 inhibitor) are commonly prescribed, but their potential metabolic effects have only recently been identified.

Title: AKR1D1 regulates glucocorticoid availability and glucocorticoid receptor activation in human hepatoma cells.
Steroid hormones, including glucocorticoids and androgens, have potent actions to regulate many cellular processes within the liver. The steroid A-ring reductase, 5β-reductase (AKR1D1), is predominantly expressed in the liver, where it inactivates steroid hormones and, in addition, plays a crucial role in bile acid synthesis. However, the precise functional role of AKR1D1 to regulate steroid hormone action in vitro has not been demonstrated. We have therefore hypothesised that genetic manipulation of AKR1D1 has the potential to regulate glucocorticoid availability and action in human hepatocytes. In both liver (HepG2) and non-liver cell (HEK293) lines, AKR1D1 over-expression increased glucocorticoid clearance with a... (truncated)

MESH:D000069465 - febuxostat

Summary:

LLM Prediction Score	0.500 (normalized)
LLM Confidence Score	0.960
Golden Answer (Severity Class)	1.0 (normalized)
Prediction Error	0.500

Retrieved Context:

Title: Two cases report of febuxostat-induced acute liver injury: acute heart failure as a probable risk factor?
Drug induced liver injury, as a sub-type of hepatotoxicity, is rare but practical problem, producing challenges for clinicians. Within the recent two months, two patients with heart failure develop febuxostat-induced acute liver injury during hospital stay. To the best of our knowledge, very few cases of febuxostat-induced hepatotoxicity have been reported up to now. In this paper, two unusual cases of febuxostat-induced acute liver injury are herein described. The medical history, drug treatment, clinical symptoms, liver function tests, diagnosis and prognosis are fully given in this paper. It should be noticed that, two liver injury happen in patients of heart... (truncated)

Title: Febuxostat attenuates aluminum chloride-induced hepatorenal injury in rats with the impact of Nrf2, Crat, Car3, and MNK-mediated apoptosis.
Aluminum (Al) is a ubiquitous xenobiotic with known toxicity for both humans and animals. Our study was conducted to investigate the protective role of febuxostat (Feb) against aluminum chloride (AlCl3)-induced hepatorenal injury in rats. Hepatorenal injury was induced by oral administration of AlCl3 (40 mg/kg b.w.), for 2 months. Twenty-four male Sprague-Dawley rats were randomly allocated into four groups (six rats/group). The first group received the vehicle thought the experiment. The second group was considered as a control positive group. The third and fourth groups received oral treatment of Feb (10 mg/kg.b.w.) and (15 mg/kg.b.w.), respectively with AlCl3, concurrently for 2 months. Twenty-four hours, after... (truncated)

Title: Febuxostat in the management of hyperuricemia and chronic gout: a review.
Febuxostat is a novel, potent, non-purine selective xanthine oxidase inhibitor, which in clinical trials demonstrated superior ability to lower and maintain serum urate levels below 6 mg/dL compared with conventionally used doses of allopurinol. Febuxostat was well tolerated in long term treatment in patients with hyperuricemia including those experiencing hypersensitity/intolerance to allopurinol. Dose adjustment appears unnecessary in patients with mild to moderate renal or liver insufficiency or advanced age. The most common adverse reactions reported were abnormal liver function tests, headache, and gastrointestinal symptoms, which were usually mild and transient. However, whether hepatotoxicity becomes a limitation in the use of... (truncated)

Title: Combined Use of Febuxostat and Colchicine Does Not Increase Acute Hepatotoxicity in Patients with Gout: A Retrospective Study.
Colchicine has been effectively used to prevent acute flares in patients with gout, but drug-related adverse events have frequently occurred. We investigated whether colchicine therapy with febuxostat is associated with hepatotoxicity in gout patients. Gout patients treated with (<i>n</i> = 121) or without (<i>n</i> = 57) colchicine were enrolled upon initiating febuxostat as a urate-lowering treatment, and clinical and laboratory data at diagnosis were compared. Logistic regression analysis was performed to evaluate the risk factors related to hepatotoxicity. Median age of the with-colchicine and without-colchicine groups was 51.0 (37.0-62.0) and 56.0 (43.5-68.5) years, respectively. During the three months of febuxostat... (truncated)

Title: Febuxostat-induced agranulocytosis in an end-stage renal disease patient: A case report.
Febuxostat, a nonpurine xanthine oxidase inhibitor, is approved as the first-line urate-lowering therapy in gout patients with normal renal function or mild to moderate renal impairment. The most common adverse effects of febuxostat are liver function test abnormalities, diarrhea, and skin rash. However, there is insufficient data in patients with severe renal impairment and end-stage renal disease (ESRD). We report the first case, to our knowledge, in which agranulocytosis developed after febuxostat treatment in an ESRD patient.

MESH:D020909 - acarbose

Summary:

LLM Prediction Score	0.500 (normalized)
LLM Confidence Score	0.990
Golden Answer (Severity Class)	1.0 (normalized)
Prediction Error	0.500

Retrieved Context:

Title: Hepatotoxicity of commonly used drugs: nonsteroidal anti-inflammatory drugs, antihypertensives, antidiabetic agents, anticonvulsants, lipid-lowering agents, psychotropic drugs.
Hepatotoxic adverse drug reactions have contributed to the decline of many promising therapies, even among mainstream medication classes (bromfenac and troglitazone are recent examples). The spectrum of nonsteroidal anti-inflammatory drug-related liver toxicity continues to expand, with reports in children, interactive toxicity in persons with hepatitis C, and recognition of the toxicity of both the preferential and selective cyclooxygenase-2 inhibitors. Of the antihypertensive agents, methyldopa is now rarely prescribed and adverse effects are reported infrequently, whereas cases of liver injury associated with the angiotensin receptor and converting enzyme inhibitors are increasingly reported. Of the antidiabetic agents, acarbose, gliclazide, metformin, and human... (truncated)

Title: Acarbose presents in vitro and in vivo antileishmanial activity against Leishmania infantum and is a promising therapeutic candidate against visceral leishmaniasis.
Treatment against visceral leishmaniasis (VL) is mainly hampered by drug toxicity, long treatment regimens and/or high costs. Thus, the identification of novel and low-cost antileishmanial agents is urgent. Acarbose (ACA) is a specific inhibitor of glucosidase-like proteins, which has been used for treating diabetes. In the present study, we show that this molecule also presents in vitro and in vivo specific antileishmanial activity against Leishmania infantum. Results showed an in vitro direct action against L. infantum promastigotes and amastigotes, and low toxicity to mammalian cells. In addition, in vivo experiments performed using free ACA or incorporated in a Pluronic® F127-based... (truncated)

Title: [Acarbose-induced acute hepatitis. Report of two events in the same patient].
A 57-year-old woman with non-insulin-dependent diabetes mellitus and inadequate glycemic control was prescribed acarbose (100 mg 3 times daily). Two months later she presented with acute hepatitis (ALT 2,300 IU/l). Other causes of liver damage were excluded. Three months after acarbose had been discontinued, all results of laboratory tests returned to normal values. Three years later the patient was given acarbose again. Acarbose (100 mg three times daily) had been added to glibenclamide (15 mg daily) 2 weeks before she presented with acute hepatitis (ALT 2,778 IU/l). Acarbose was stopped and the results of liver tests returned to normal within... (truncated)

Title: Hepatotoxicity associated with acarbose therapy.
To report a case of acarbose-induced hepatotoxicity and compare other reported cases from the literature.

Title: Acarbose Use and Liver Injury in Diabetic Patients With Severe Renal Insufficiency and Hepatic Diseases: A Propensity Score-Matched Cohort Study.
Background: Acarbose has been deemed contraindicated in diabetic patients with chronic kidney disease (CKD) or end-stage renal disease (ESRD), but such use is not uncommon. We tested whether this concept hold true in this population with different background hepatic diseases. Methods: All incident diabetic patients (<i>n</i> = 2,036,531) with stage 5 CKD/ESRD were enrolled from Taiwan between 2017 and 2013 and divided into those without chronic liver disease (CLD), with CLD but without cirrhosis, and those with cirrhosis. Among each group, acarbose users, defined as cumulative use >30 days within the preceding year, were propensity-score matched 1:2 to non-users. Our... (truncated)

MESH:D006918 - hydroxyurea

Summary:

LLM Prediction Score	0.500 (normalized)
LLM Confidence Score	0.990
Golden Answer (Severity Class)	1.0 (normalized)
Prediction Error	0.500

Retrieved Context:

Title: Hydroxyurea induced acute elevations in liver function tests.
Hydroxyurea (HU) is a ribonucleotide reductase inhibitor used to treat myeloproliferative diseases including polycythemia vera (PV) and essential thrombocythemia (ET). We describe an 82-year-old male who was started on HU 500 mg three times weekly for the treatment of PV. Eight days after initiation of HU he experienced anorexia, nausea, vomiting, fever, fatigue, dizziness, and shaking chills. Discontinuation of the HU resulted in resolution of his symptoms within 2 days, and HU was re-started. Ten days after re-starting HU, the patient re-presented with nausea and anorexia. Lab tests revealed elevations in liver enzyme function tests, which resolved promptly after cessation... (truncated)

Title: Sequential oral hydroxyurea and intravenous cytosine arabinoside in refractory childhood acute leukemia: a pediatric oncology group phase 1 study.
At concentrations >0.1 mM, hydroxyurea (HU) enhances the accumulation of cytosine arabinoside (ara-C) in leukemia cells in vitro. This study of children with refractory acute leukemia was designed to take advantage of this biochemical modulation. A fixed dose of HU and an escalating dose of ara-C were used. Oral HU (1200 mg/m2) was followed 2 hours later by ara-C (250-3100 mg/m2) intravenously in 15 minutes. The combination was given on days 1, 2, 3 and 8, 9, 10. Thirty-three children [26 acute lymphocytic leukemia (ALL), 7 acute nonlymphocytic leukemia] were treated; 29 received at least 1 full course. All patients... (truncated)

Title: Choline antagonism of methotrexate liver toxicity in the rat.
Because of the frequent reports of hepatic toxicity associated with long-term administration of methotrexate, a rat model was developed utilizing daily methotrexate administration. This model revealed an incidence of fatty metamorphosis of over 80 percent, atrophy and necrosis of 30 percent, and fibrosis of 10 percent. Fatty liver changes did not differ substantially from control animals in those animals receiving long-term thydroxyurea, an agent which, like methotrexate, inhibits DNA synthesis but unlike methotrexate, does not impair methylation reactions. Because choline has a lipotropic effect and because its synthesis requires methylation, an attempt was made to block the liver toxicity of... (truncated)

Title: Hydroxyurea in the treatment of HIV infection: clinical efficacy and safety concerns.
Data from basic science and clinical studies suggest that hydroxyurea (hydroxycarbamide)-based regimens are effective treatment options for patients with HIV at various stages of disease. In vitro studies of HIV-infected lymphocytes have shown that hydroxyurea: (i) inhibits viral DNA synthesis; (ii) synergistically interacts with nucleoside reverse transcriptase inhibitors (NRTI); and (iii) increases the antiviral activity of didanosine. Clinical studies have confirmed that hydroxyurea in combination with didanosine produces potent and sustained viral suppression in patients with HIV infection. However, some concerns have been recently raised on the use of hydroxyurea in association with NRTI. Hydroxyurea can cause myelosuppression, skin toxicities,... (truncated)

Title: High hepatotoxicity rate seen among HAART patients.
Evidence continues to mount showing that HIV patients receiving antiretroviral treatment are at high risk for hepatotoxicity. The National Institutes of Health presented findings on liver toxicity among 10,011 HIV patients at the first International AIDS Society conference. The retrospective analysis shows that liver toxicity is associated with all classes of antiretroviral medications in use, and not just with regimens containing nevirapine or hydroxyurea, the two drugs about which the FDA recently issued hepatotoxicity warnings.

MESH:D000077341 - lapatinib

Summary:

LLM Prediction Score	0.500 (normalized)
LLM Confidence Score	0.980
Golden Answer (Severity Class)	1.0 (normalized)
Prediction Error	0.500

Retrieved Context:

Title: Does the use of lapatinib increase the risk of fatigue and hepatic toxicities in patients with solid tumors? A critical literature review and meta-analysis.
A systematic review and meta-analysis of fatigue and hepatic adverse events associated with lapatinib use in solid tumor patients were performed.

Title: Lapatinib-induced hepatitis: a case report.
Lapatinib is an inhibitor of the tyrosine kinases of human epidermal growth factor receptor type 2 (HER2) and epidermal growth factor receptor type 1, with clinical activity in HER2-positive metastatic breast cancer. We present here a 60 year-old patient with metastatic breast cancer who presented with jaundice and increased serum aminotransferase levels and who had been treated with lapatinib for the previous 14 days. Laboratory tests excluded other causes of acute liver injury. Liver biopsy revealed lesions compatible with drug-induced hepatotoxicity. Bilirubin and liver enzymes returned to normal within three months of lapatinib discontinuation. Lapatinib should be included among the... (truncated)

Title: Hepatotoxicity of tyrosine kinase inhibitors: clinical and regulatory perspectives.
The introduction of small-molecule tyrosine kinase inhibitors (TKIs) in clinical oncology has transformed the treatment of certain forms of cancers. As of 31 March 2013, 18 such agents have been approved by the US Food and Drug Administration (FDA), 15 of these also by the European Medicines Agency (EMA), and a large number of others are in development or under regulatory review. Unexpectedly, however, their use has been found to be associated with serious toxic effects on a number of vital organs including the liver. Drug-induced hepatotoxicity has resulted in withdrawal from the market of many widely used drugs and... (truncated)

Title: Human metabolism of lapatinib, a dual kinase inhibitor: implications for hepatotoxicity.
Lapatinib (Tykerb, Tyverb) is an important orally active dual tyrosine kinase inhibitor efficacious in combination therapy for patients with progressive human epidermal receptor 2-overexpressing metastatic breast cancer. However, clinically significant liver injury, which may be associated with lapatinib metabolic activation, has been reported. We describe the metabolism and excretion of [(14)C]lapatinib in six healthy human volunteers after a single oral dose of 250 mg and the potential relationships between metabolism and clinical hepatotoxicity. Overall, elimination showed high intersubject variability, with fecal elimination being the predominant pathway, representing a median of 92% of the dose with lapatinib as the largest component... (truncated)

Title: Hepatotoxicity of molecular targeted therapy.
A constant increase in occurrence of neoplasms is observed; hence new methods of therapy are being intensively researched. One of the methods of antineoplastic treatment is molecular targeted therapy, which aims to influence individual processes occurring in cells. Using this type of medications is associated with unwanted effects resulting from the treatment. Liver damage is a major adverse effect diagnosed during targeted therapy. Drug-induced liver damage can occur as necrosis of hepatocytes, cholestatic liver damage and cirrhosis. Hepatotoxicity is evaluated on the basis of International Consensus Criteria. Susceptibility of the liver to injury is connected not only with toxicity of... (truncated)

MESH:C084615 - alclofenac

Summary:

LLM Prediction Score	0.500 (normalized)
LLM Confidence Score	0.740
Golden Answer (Severity Class)	1.0 (normalized)
Prediction Error	0.500

Retrieved Context:

Title: Modeling the Bioactivation and Subsequent Reactivity of Drugs.
Electrophilically reactive drug metabolites are implicated in many adverse drug reactions. In this mechanism-termed bioactivation-metabolic enzymes convert drugs into reactive metabolites that often conjugate to nucleophilic sites within biological macromolecules like proteins. Toxic metabolite-product adducts induce severe immune responses that can cause sometimes fatal disorders, most commonly in the form of liver injury, blood dyscrasia, or the dermatologic conditions toxic epidermal necrolysis and Stevens-Johnson syndrome. This study models four of the most common metabolic transformations that result in bioactivation: quinone formation, epoxidation, thiophene sulfur-oxidation, and nitroaromatic reduction, by synthesizing models of metabolism and reactivity. First, the metabolism models predict the... (truncated)

Title: Association of CYP1A1 and CYP1B1 inhibition in in vitro assays with drug-induced liver injury.
Drug-induced liver injury (DILI) is one of the major causes for the discontinuation of drug development and withdrawal of drugs from the market. Since it is known that reactive metabolite formation and being substrates or inhibitors of cytochrome P450s (P450s) are associated with DILI, we systematically investigated the association between human P450 inhibition and DILI. The inhibitory activity of 266 DILI-positive drugs (DILI drugs) and 92 DILI-negative drugs (no-DILI drugs), which were selected from Liver Toxicity Knowledge Base (US Food and Drug Administration), against 8 human P450 forms was assessed using recombinant enzymes and luminescent substrates, and the threshold values... (truncated)

Title: Data-driven identification of structural alerts for mitigating the risk of drug-induced human liver injuries.
The use of structural alerts to de-prioritize compounds with undesirable features as drug candidates has been gaining in popularity. Hundreds of molecular structural moieties have been proposed as structural alerts. An emerging issue is that strict application of these alerts will result in a significant reduction of the chemistry space for new drug discovery, as more than half of the oral drugs on the market match at least one of the alerts. To mitigate this issue, we propose to apply a rigorous statistical analysis to derive/validate structural alerts before use.

Title: A Review on Current Repurposing Drugs for the Treatment of COVID-19: Reality and Challenges.
The coronavirus disease 2019 (COVID-19) caused by the novel severe acute respiratory syndrome coronavirus 2 (SARS-CoV-2) has become a global pandemic with a high growth rate of confirmed cases. Therefore, therapeutic options are desperately urgent to fight with this damning virus. As it may take years to develop a specific therapy of COVID-19, it is urgent to emphasize the repurposing of drugs used for other conditions. This study reviewed the most common drugs for COVID-19 based on available online literature representing the latest in vitro clinical trial database, rational of use, adverse effects, potential toxicities, and US National Institute of... (truncated)

Title: Structural and Chemical Biology of the Interaction of Cyclooxygenase with Substrates and Non-Steroidal Anti-Inflammatory Drugs.
Cyclooxgenases are key enzymes of lipid signaling. They carry out the first step in the production of prostaglandins, important mediators of inflammation, pain, cardiovascular disease, and cancer, and they are the molecular targets for nonsteroidal anti-inflammatory drugs, which are among the oldest and most chemically diverse set of drugs known. Homodimeric proteins that behave as allosterically modulated, functional heterodimers, the cyclooxygenases exhibit complex kinetic behavior, requiring peroxide-dependent activation and undergoing suicide inactivation. Due to their important physiological and pathophysiological roles and keen interest on the part of the pharmaceutical industry, the cyclooxygenases have been the focus of a vast array... (truncated)

MESH:D004061 - diflunisal

Summary:

LLM Prediction Score	0.500 (normalized)
LLM Confidence Score	0.960
Golden Answer (Severity Class)	1.0 (normalized)
Prediction Error	0.500

Retrieved Context:

Title: The problems and pitfalls of NSAID therapy in the elderly (Part I).
Nonsteroidal anti-inflammatory drugs (NSAIDs) are the most commonly prescribed drugs worldwide when grouped by generic categories and account for 3 to 9% of total prescription numbers in various countries. While NSAIDs are responsible for approximately 25% of all reported adverse drug reactions, aging may substantially increase the risk of NSAID-induced reactions. Several factors may contribute to NSAID-related toxicity in the elderly. The increase in morbidity associated with aging may result in consumption of a wide range of potent drugs, while inappropriate drug therapy and aberrant compliance are also capable of contributing to adverse drug reactions in geriatric patients. Age-related alterations... (truncated)

Title: Translating clinical findings into knowledge in drug safety evaluation--drug induced liver injury prediction system (DILIps).
Drug-induced liver injury (DILI) is a significant concern in drug development due to the poor concordance between preclinical and clinical findings of liver toxicity. We hypothesized that the DILI types (hepatotoxic side effects) seen in the clinic can be translated into the development of predictive in silico models for use in the drug discovery phase. We identified 13 hepatotoxic side effects with high accuracy for classifying marketed drugs for their DILI potential. We then developed in silico predictive models for each of these 13 side effects, which were further combined to construct a DILI prediction system (DILIps). The DILIps yielded... (truncated)

Title: Diflunisal Targeted Delivery Systems: A Review.
Diflunisal is a well-known drug for the treatment of rheumatoid arthritis, osteoarthritis, primary dysmenorrhea, and colon cancer. This molecule belongs to the group of nonsteroidal anti-inflammatory drugs (NSAID) and thus possesses serious side effects such as cardiovascular diseases risk development, renal injury, and hepatic reactions. The last clinical data demonstrated that diflunisal is one of the recognized drugs for the treatment of cardiac amyloidosis and possesses a survival benefit similar to that of clinically approved tafamidis. Diflunisal stabilizes the transthyretin (TTR) tetramer and prevents the misfolding of monomers and dimers from forming amyloid deposits in the heart. To avoid serious... (truncated)

Title: Prediction of liver injury induced by chemicals in human with a multiparametric assay on isolated mouse liver mitochondria.
Drug-induced liver injury (DILI) in humans is difficult to predict using classical in vitro cytotoxicity screening and regulatory animal studies. This explains why numerous compounds are stopped during clinical trials or withdrawn from the market due to hepatotoxicity. Thus, it is important to improve early prediction of DILI in human. In this study, we hypothesized that this goal could be achieved by investigating drug-induced mitochondrial dysfunction as this toxic effect is a major mechanism of DILI. To this end, we developed a high-throughput screening platform using isolated mouse liver mitochondria. Our broad spectrum multiparametric assay was designed to detect the... (truncated)

Title: Gender-specific changes in energy metabolism and protein degradation as major pathways affected in livers of mice treated with ibuprofen.
Ibuprofen, an inhibitor of prostanoid biosynthesis, is a common pharmacological agent used for the management of pain, inflammation and fever. However, the chronic use of ibuprofen at high doses is associated with increased risk for cardiovascular, renal, gastrointestinal and liver injuries. The underlying mechanisms of ibuprofen-mediated effects on liver remain unclear. To determine the mechanisms and signaling pathways affected by ibuprofen (100 mg/kg/day for seven days), we performed proteomic profiling of male mice liver with quantitative liquid chromatography tandem mass spectrometry (LC-MS/MS) using ten-plex tandem mass tag (TMT) labeling. More than 300 proteins were significantly altered between the control and... (truncated)

MESH:C073716 - niperotidine

Summary:

LLM Prediction Score	0.500 (normalized)
LLM Confidence Score	0.430
Golden Answer (Severity Class)	1.0 (normalized)
Prediction Error	0.500

Retrieved Context:

Title: Acute liver injury related to the use of niperotidine.
H2-receptor antagonists are widely used for the therapy of peptic disease, since they ensure a protracted and intense inhibition of gastric acidity. Niperotidine (piperonyl-ranitidine) is a new H2 blocking agent recently proposed for clinical use.

Title: Mechanisms of drug toxicity and relevance to pharmaceutical development.
Toxicity has been estimated to be responsible for the attrition of approximately one-third of drug candidates and is a major contributor to the high cost of drug development, particularly when not recognized until late in clinical trials or post-marketing. The causes of drug toxicity can be classified in several ways and include mechanism-based (on-target) toxicity, immune hypersensitivity, off-target toxicity, and bioactivation/covalent modification. In addition, idiosyncratic responses are rare but can be one of the most problematic issues; several hypotheses for these have been advanced. Although covalent binding of drugs to proteins was described almost 40 years ago, the significance to... (truncated)

Title: Data-driven identification of structural alerts for mitigating the risk of drug-induced human liver injuries.
The use of structural alerts to de-prioritize compounds with undesirable features as drug candidates has been gaining in popularity. Hundreds of molecular structural moieties have been proposed as structural alerts. An emerging issue is that strict application of these alerts will result in a significant reduction of the chemistry space for new drug discovery, as more than half of the oral drugs on the market match at least one of the alerts. To mitigate this issue, we propose to apply a rigorous statistical analysis to derive/validate structural alerts before use.

MESH:C021277 - nilutamide

Summary:

LLM Prediction Score	0.500 (normalized)
LLM Confidence Score	0.930
Golden Answer (Severity Class)	1.0 (normalized)
Prediction Error	0.500

Retrieved Context:

Title: Simultaneous liver and lung toxicity related to the nonsteroidal antiandrogen nilutamide (Anandron): a case report.
We report the case of a 69-year-old patient treated for stage C adenocarcinoma of the prostate with the combination of the luteinizing hormone-releasing hormone agonist [D-Trp6, des-Gly-NH2(10)] LHRH ethylamide and the antiandrogen nilutamide (Anandron) who developed simultaneous liver and lung toxicity. Investigation revealed an abnormal chest radiograph accompanied by altered respiratory function tests, suggesting the diagnosis of interstitial lung disease. This diagnosis was confirmed by an open chest biopsy. At the same time, liver function tests yielded abnormal results. All toxic manifestations disappeared after cessation of treatment with Anandron.

Title: Mitochondrial permeability transition as a source of superoxide anion induced by the nitroaromatic drug nimesulide in vitro.
Nimesulide, a widely used nonsteroidal anti-inflammatory drug containing a nitroaromatic moiety, has been associated with rare but serious hepatic adverse effects. The mechanisms underlying this idiosyncratic hepatotoxicity are unknown; however, both mitochondrial injury and oxidative stress have been implicated in contributing to liver injury in susceptible patients. The aim of this study was, first, to explore whether membrane permeability transition (MPT) could contribute to nimesulide's mitochondrial toxicity and, second, whether metabolism-derived reactive oxygen species (ROS) were responsible for MPT. We found that isolated mouse liver mitochondria readily underwent Ca2+-dependent, cyclosporin A-sensitive MPT upon exposure to nimesulide (at >or=3 microM). Net... (truncated)

Title: Antiandrogens: a summary review of pharmacodynamic properties and tolerability in prostate cancer therapy.
This article provides a summary of the pharmacodynamic properties of major antiandrogens as well as an extensive review of their tolerability. Presently there are two classes of androgen receptor antagonists: the so-called pure, non-steroidal antiandrogens which include flutamide, nilutamide and the more recent bicalutamide and the steroidal antiandrogens cyproterone acetate, megestrol acetate and WIN 49596. Although non steroidal and steroidal compounds have been found to be equally effective in the treatment of prostate cancer presently no studies comparing the use of steroidal or non steroidal antiandrogens with chemical or surgical castration have evaluated quality of life per se. The only... (truncated)

Title: Novel hormonal therapies in the management of advanced prostate cancer: extrapolating Asian findings to Southeast Asia.
There is a paucity of information on the use of novel hormonal agents in Southeast Asian patients. We reviewed the clinical roles of novel hormonal therapy (NHT), namely abiraterone acetate (AA), enzalutamide, apalutamide and darolutamide, in the management of advanced prostate cancer, and data on its use in Asian patients, in order to extrapolate these findings to the Southeast Asian patient population. There are some differences in the molecular features between the NHTs, which influenced their respective permeabilities through the blood-brain barrier. The Asian sub-analyses of the landmark studies of each NHT were limited. The primary endpoints of the Asian... (truncated)

Title: The Importance of Patient-Specific Factors for Hepatic Drug Response and Toxicity.
Responses to drugs and pharmacological treatments differ considerably between individuals. Importantly, only 50%-75% of patients have been shown to react adequately to pharmacological interventions, whereas the others experience either a lack of efficacy or suffer from adverse events. The liver is of central importance in the metabolism of most drugs. Because of this exposed status, hepatotoxicity is amongst the most common adverse drug reactions and hepatic liabilities are the most prevalent reason for the termination of development programs of novel drug candidates. In recent years, more and more factors were unveiled that shape hepatic drug responses and thus underlie the... (truncated)

MESH:C031942 - argatroban

Summary:

LLM Prediction Score	0.500 (normalized)
LLM Confidence Score	0.970
Golden Answer (Severity Class)	0.0 (normalized)
Prediction Error	0.500

Retrieved Context:

Title: [New antitoagulants].
In contrast to older anticoagulant agents vitamin K antagonists and heparins, the new ones are directed towards a single target in general. The main characteristics of the new agents are: their site of action in the coagulation cascade and their mechanism of action which is indirect, antithrombin dependent, most often such as Fondaparinux and Idraparinux or direct such as Dabigatran, Rivaroxaban; the specificity of the new molecules, since they must not interact with other enzymes: trypsin, kallikrein, t-PA, etc...; their mode of administration parenteral and/or oral; their pharmacokinetics and their clearance frequently by the kidney (Hirudin, fondaparinux) or through hepatic... (truncated)

Title: Acute coagulation disorder in a critically ill patient - A case report.
A 79-year-old critically ill woman presented with remarkable prolongation of activated partial thromboplastin time and thrombin time combined with high levels of anti-factor IIa activity 26 days after coronary artery bypass grafting. Coagulation disorder was associated with severe bleeding. Cause of coagulopathy was accidental administration of argatroban in an unknown dosage. Clearance of argatroban was significantly prolonged because of a liver function disorder related to septic multiorgan failure. Argatroban reversal was performed with prothrombin complex concentrate.

Title: Argatroban dose reductions for suspected heparin-induced thrombocytopenia complicated by child-pugh class C liver disease.
To report our experience of reduced-dose argatroban in a patient with suspected heparin-induced thrombocytopenia (HIT) and Child-Pugh class C liver disease and review the relevant literature to summarize current recommendations on argatroban use in patients with severe liver disease.

Title: The pharmacokinetics and pharmacodynamics of argatroban: effects of age, gender, and hepatic or renal dysfunction.
To determine the pharmacokinetics and pharmacodynamics of argatroban in healthy volunteers and patients with hepatic or renal dysfunction.

Title: Update on argatroban for the prophylaxis and treatment of heparin-induced thrombocytopenia type II.
Heparin-induced thrombocytopenia (HIT) is a rare but potentially severe complication of heparin therapy that is strongly associated with venous and arterial thrombosis (HIT and thrombosis syndrome, HITTS), which requires urgent detection and treatment with a nonheparin anticoagulant. Argatroban, a synthetic direct thrombin inhibitor, is indicated for the treatment and prophylaxis of thrombosis in patients with HIT, including those undergoing percutaneous coronary intervention. Argatroban has a relatively short elimination half-life of approximately 45 minutes, which is predominantly performed via hepatic metabolism. It is derived from L-arginine that selectively and reversibly inhibits thrombin, both clot-bound and free, at the catalytic site. Argatroban... (truncated)

MESH:D006118 - griseofulvin

Summary:

LLM Prediction Score	0.500 (normalized)
LLM Confidence Score	0.990
Golden Answer (Severity Class)	1.0 (normalized)
Prediction Error	0.500

Retrieved Context:

Title: Toxic effects of griseofulvin: disease models, mechanisms, and risk assessment.
Griseofulvin (GF) has been in use for more than 30 years as a pharmaceutical drug in humans for the treatment of dermatomycoses. Animal studies give clear evidence that it causes a variety of acute and chronic toxic effects, including liver and thyroid cancer in rodents, abnormal germ cell maturation, teratogenicity, and embroyotoxicity in various species. No sufficient data from human studies are available at present to exclude a risk in humans: therefore, attempts were made to elucidate the mechanisms responsible for the toxic effects of GF and to address the question whether such effects might occur in humans undergoing GF... (truncated)

Title: Griseofulvin: An Updated Overview of Old and Current Knowledge.
Griseofulvin is an antifungal polyketide metabolite produced mainly by ascomycetes. Since it was commercially introduced in 1959, griseofulvin has been used in treating dermatophyte infections. This fungistatic has gained increasing interest for multifunctional applications in the last decades due to its potential to disrupt mitosis and cell division in human cancer cells and arrest hepatitis C virus replication. In addition to these inhibitory effects, we and others found griseofulvin may enhance ACE2 function, contribute to vascular vasodilation, and improve capillary blood flow. Furthermore, molecular docking analysis revealed that griseofulvin and its derivatives have good binding potential with SARS-CoV-2 main protease,... (truncated)

Title: Comparative Hepatotoxicity of Fluconazole, Ketoconazole, Itraconazole, Terbinafine, and Griseofulvin in Rats.
Oral ketoconazole was recently the subject of regulatory safety warnings because of its association with increased risk of inducing hepatic injury. However, the relative hepatotoxicity of antifungal agents has not been clearly established. The aim of this study was to compare the hepatotoxicity induced by five commonly prescribed oral antifungal agents. Rats were treated with therapeutic oral doses of griseofulvin, fluconazole, itraconazole, ketoconazole, and terbinafine. After 14 days, only ketoconazole had significantly higher ALT levels (<i>p</i> = 0.0017) and AST levels (<i>p</i> = 0.0008) than the control group. After 28 days, ALT levels were highest in the rats treated with... (truncated)

Title: Pharmacokinetic optimisation of oral antifungal therapy.
The range of oral antifungal therapy has been expanded recently by the introduction of itraconazole, and terbinafine. These agents have a broader spectrum of activity than griseofulvin and flucytosine, and induce less liver toxicity than ketoconazole. Treatment with these agents may be optimised by application of pharmacokinetic principles. Griseofulvin, ketoconazole and itraconazole should be administered with food to ensure adequate absorption. Maximal absorption of griseofulvin is achieved by administration of the drug as a solid solution in polyethylene glycol. Absorption of azole antifungal agents is impaired by high gastric pH, which is observed in some patients with acquired immunodeficiency syndrome.... (truncated)

Title: A metabolomic perspective of griseofulvin-induced liver injury in mice.
Griseofulvin (GSF) causes hepatic porphyria in mice, which mimics the liver injury associated with erythropoietic protoporphyria (EPP) in humans. The current study investigated the biochemical basis of GSF-induced liver injury in mice using a metabolimic approach. GSF treatment in mice resulted in significant accumulations of protoporphyrin IX (PPIX), N-methyl PPIX, bile acids, and glutathione (GSH) in the liver. Metabolomic analysis also revealed bioactivation pathways of GSF that contributed to the formation of GSF-PPIX, GSF-GSH and GSF-proline adducts. GSF-PPIX is the precursor of N-methyl PPIX. A six-fold increase of N-methyl PPIX was observed in the liver of mice after GSF treatment.... (truncated)

MESH:D019772 - topotecan

Summary:

LLM Prediction Score	0.500 (normalized)
LLM Confidence Score	0.980
Golden Answer (Severity Class)	0.0 (normalized)
Prediction Error	0.500

Retrieved Context:

Title: Topotecan combined with Ifosfamide, Etoposide, and L-asparaginase (TIEL) regimen improves outcomes in aggressive T-cell lymphoma.
This study evaluated the efficacy and safety of a new regimen consisting of Topotecan, Ifosfamide, Etoposide, and L-asparaginase (TIEL) in treating aggressive T-cell lymphoma. Twenty-four patients were included in the research, eighteen males and six females. Half of the patients were in stages III and IV, and nearly half of them experienced failure of at least one regimen. Eleven were diagnosed as peripheral T-cell lymphoma (PTCL), five extranodal NK/T-cell lymphoma, non-specific, four angioimmunoblastic, and four anaplastic large-cell lymphoma (2 ALK positive). Patients were given 98 cycles of TIEL altogether. The responsive rate to TIEL was 76.9 % among 13 cases... (truncated)

Title: Activity of topotecan 21-day infusion in patients with previously treated large cell lymphoma: long-term follow-up of an Eastern Cooperative Oncology Group study (E5493).
The purpose of this study was to determine the activity of topotecan given by 21-day continuous infusion in patients previously treated with one prior therapy for a diffuse large-cell lymphoma or immunoblastic lymphoma. Patients with appropriate histology and measurable disease who had been treated with one prior chemotherapy regimen were eligible for study. Slides of tumor biopsies were submitted for central review of pathology. Patients were required to have an Eastern Cooperative Oncology Group (ECOG) performance status of 0, 1 or 2 and adequate bone marrow function. Patients were treated with continuous infusion topotecan, 0.4 mg/m(2)/day × 21 days. Therapy... (truncated)

Title: Lesser Toxicities of Belotecan in Patients with Small Cell Lung Cancer: A Retrospective Single-Center Study of Camptothecin Analogs.
<i>Purpose</i>. Topotecan and belotecan are camptothecin derivatives that are used to treat small cell lung cancer (SCLC). This study compared the toxicities and efficacies of belotecan and topotecan monotherapies in patients with SCLC. <i>Methods</i>. We retrospectively reviewed data from 94 patients with SCLC (with or without prior chemotherapy) who were treated using belotecan monotherapy (<i>n</i> = 59, 188 cycles) or topotecan monotherapy (<i>n</i> = 35, 65 cycles) between September 2003 and December 2011. <i>Results</i>. Thrombocytopenia occurred during 42% and 61.5% of the belotecan and topotecan cycles, respectively (<i>p</i> = 0.007). Significant differences between belotecan and topotecan were also observed for... (truncated)

Title: Topotecan induces hepatocellular injury via ASCT2 mediated oxidative stress.
Topotecan is an anti-cancer chemotherapy drug with common side effects, including hepatotoxicity. In this study, we aim to investigate the mechanisms of topotecan-induced hepatocellular injury beyond conventional DNA damage.

Title: Toxicity of weekly oral topotecan in relation to dosage for gynecologic malignancies: a phase I study.
The aim of this study was to determine the dose of weekly oral topotecan that allows safe administration and to evaluate the pharmacokinetics of this dose in patients with recurrent gynecologic malignancies. The first cohort of patients received oral topotecan 6 mg/week administered orally on days 1, 8, and 15 of a 28-day regimen. A standard 3+3 dose-escalating phase design was used for dose levels II-V (8, 10, 12 and 14 mg/week). Toxicity was scored according to the Common Terminology Criteria for Adverse Events. Cumulative toxicity was summarized in the 6-12 mg/week combined cohort and 14 mg/week cohort separately. Pharmacokinetic... (truncated)

MESH:D001589 - benzphetamine

Summary:

LLM Prediction Score	0.500 (normalized)
LLM Confidence Score	0.930
Golden Answer (Severity Class)	0.0 (normalized)
Prediction Error	0.500

Retrieved Context:

Title: Cocaine-Induced Time-Dependent Alterations in Cytochrome P450 and Liver Function.
Cytochrome P450 is responsible for the metabolism of endogenous substrates, drugs and substances of abuse. The brain and nervous system regulate liver cytochrome P450 via neuroendocrine mechanisms, as shown in rodents. Cocaine exerts its addictive effects through the dopaminergic system, the functioning of which undergoes changes during its continuous use. Therefore, it can be hypothesized that the regulation of cytochrome P450 by cocaine may also alter during the addiction process, cessation and relapse. We analyzed preclinical studies on the mechanisms of the pharmacological action of cocaine, the role of the brain's dopaminergic system in the neuroendocrine regulation of cytochrome P450... (truncated)

Title: Association of CYP1A1 and CYP1B1 inhibition in in vitro assays with drug-induced liver injury.
Drug-induced liver injury (DILI) is one of the major causes for the discontinuation of drug development and withdrawal of drugs from the market. Since it is known that reactive metabolite formation and being substrates or inhibitors of cytochrome P450s (P450s) are associated with DILI, we systematically investigated the association between human P450 inhibition and DILI. The inhibitory activity of 266 DILI-positive drugs (DILI drugs) and 92 DILI-negative drugs (no-DILI drugs), which were selected from Liver Toxicity Knowledge Base (US Food and Drug Administration), against 8 human P450 forms was assessed using recombinant enzymes and luminescent substrates, and the threshold values... (truncated)

Title: Phytotoxicological study of selected poisonous plants from Azad Jammu & Kashmir.
Poisonous plants cause tremendous economic losses to the livestock industry. These economic losses are deterioration in their health, decreased productivity, deformed offspring, and reduced longevity. The current study is the first comprehensive report on poisonous plants of Azad Jammu and Kashmir which systematically documents the phytotoxicological effect and mode of action in livestock. The information was gathered from 271 informants including 167 men and 104 women through semi-structured interviews and literature search through available databases. The data collected through interviews was analyzed with quantitative tools viz. the factor informant consensus and fidelity level. A total of 38 species of flowering... (truncated)

Title: The Evaluation of Drug Delivery Nanocarrier Development and Pharmacological Briefing for Metabolic-Associated Fatty Liver Disease (MAFLD): An Update.
Current research indicates that the next silent epidemic will be linked to chronic liver diseases, specifically non-alcoholic fatty liver disease (NAFLD), which was renamed as metabolic-associated fatty liver disease (MAFLD) in 2020. Globally, MAFLD mortality is on the rise. The etiology of MAFLD is multifactorial and still incompletely understood, but includes the accumulation of intrahepatic lipids, alterations in energy metabolism, insulin resistance, and inflammatory processes. The available MAFLD treatment, therefore, relies on improving the patient's lifestyle and multidisciplinary pharmacotherapeutic options, whereas the option of surgery is useless without managing the comorbidities of the MAFLD. Nanotechnology is an emerging approach addressing... (truncated)

Title: Lung injury induced by pyrrolizidine alkaloids depends on metabolism by hepatic cytochrome P450s and blood transport of reactive metabolites.
Pyrrolizidine alkaloids (PAs) are common phytotoxins with both hepatotoxicity and pneumotoxicity. Hepatic cytochrome P450 enzymes are known to bioactivate PAs into reactive metabolites, which can interact with proteins to form pyrrole-protein adducts and cause intrahepatic cytotoxicity. However, the metabolic and initiation biochemical mechanisms underlying PA-induced pneumotoxicity remain unclear. To investigate the in vivo metabolism basis for PA-induced lung injury, this study used mice with conditional deletion of the cytochrome P450 reductase (Cpr) gene and resultant tissue-selective ablation of microsomal P450 enzyme activities. After oral exposure to monocrotaline (MCT), a pneumotoxic PA widely used to establish animal lung injury models, liver-specific... (truncated)

MESH:D000077589 - sulfathiazole

Summary:

LLM Prediction Score	0.500 (normalized)
LLM Confidence Score	0.970
Golden Answer (Severity Class)	1.0 (normalized)
Prediction Error	0.500

Retrieved Context:

Title: Dual mechanisms suppress meloxicam bioactivation relative to sudoxicam.
Thiazoles are biologically active aromatic heterocyclic rings occurring frequently in natural products and drugs. These molecules undergo typically harmless elimination; however, a hepatotoxic response can occur due to multistep bioactivation of the thiazole to generate a reactive thioamide. A basis for those differences in outcomes remains unknown. A textbook example is the high hepatotoxicity observed for sudoxicam in contrast to the relative safe use and marketability of meloxicam, which differs in structure from sudoxicam by the addition of a single methyl group. Both drugs undergo bioactivation, but meloxicam exhibits an additional detoxification pathway due to hydroxylation of the methyl group.... (truncated)

Title: Association of CYP1A1 and CYP1B1 inhibition in in vitro assays with drug-induced liver injury.
Drug-induced liver injury (DILI) is one of the major causes for the discontinuation of drug development and withdrawal of drugs from the market. Since it is known that reactive metabolite formation and being substrates or inhibitors of cytochrome P450s (P450s) are associated with DILI, we systematically investigated the association between human P450 inhibition and DILI. The inhibitory activity of 266 DILI-positive drugs (DILI drugs) and 92 DILI-negative drugs (no-DILI drugs), which were selected from Liver Toxicity Knowledge Base (US Food and Drug Administration), against 8 human P450 forms was assessed using recombinant enzymes and luminescent substrates, and the threshold values... (truncated)

Title: Systemic quinolones and risk of acute liver failure I: Analysis of data from the US FDA adverse event reporting system.
Quinolones are a potent and globally popular group of antibiotics that are used to treat a wide range of infections. Some case reports have raised concern about their possible association with acute hepatic failure (AHF). Data from the US FDA Adverse Event Reporting System were evaluated for signals of AHF in association with systemically administered quinolone antibiotics.

Title: Lapatinib promotes the incidence of hepatotoxicity by increasing chemotherapeutic agent accumulation in hepatocytes.
Lapatinib has been used in combination with capecitabine or paclitaxel to treat patients with progressive HER2-overexpressing metastatic breast cancer (MBC). Unfortunately, an increased incidence of hepatotoxicity had been reported in the combinational therapy. The aim of this study was to investigate the potential mechanisms of this combinational therapy. We found that the patients receiving lapatinib and paclitaxel treatment showed a higher incidence of hepatobiliary system disorders than those receiving paclitaxel alone. Lapatinib was shown to increase the accumulation of doxorubicin in ABCB1-overexpressing hepatocellular cancer cells and normal liver tissues without altering the protein level of ABCB1. Pharmacokinetic studies revealed that... (truncated)

Title: An Automated Solid-Phase Extraction-UPLC-MS/MS Method for Simultaneous Determination of Sulfonamide Antimicrobials in Environmental Water.
The large-scale use of sulfonamide antimicrobials in human and veterinary medicine has seriously endangered the ecological environment and human health. The objective of this study was to develop and validate a simple and robust method for the simultaneous determination of seventeen sulfonamides in water using ultra-high performance liquid chromatography-tandem mass spectrometry coupled with fully automated solid-phase extraction. Seventeen isotope-labeled internal standards for sulfonamides were used to correct matrix effects. Several parameters affecting extraction efficiency were systematically optimized, and the enrichment factors were up to 982-1033 and only requiring about 60 min per six samples. Under the optimized conditions, this method... (truncated)

MESH:D002753 - chlorzoxazone

Summary:

LLM Prediction Score	0.500 (normalized)
LLM Confidence Score	0.930
Golden Answer (Severity Class)	1.0 (normalized)
Prediction Error	0.500

Retrieved Context:

Title: [Severe hepatocellular damage after administration of paracetamol and chlorzoxazone in therapeutic dosage].
A 64 year old patient developed severe hepatocellular damage with jaundice and coagulopathy during ingestion of a combination of paracetamol and chlorzoxazone in therapeutic dosage. The risk factors for the development of liver cell necrosis following ingestion of paracetamol in therapeutic dosage are discussed. In particular in patients with risk factors (e.g. alcoholics and patients with heart failure) paracetamol-induced liver failure has to be considered in the presence of high transaminases, even when paracetamol was ingested in therapeutic dosage. Chlorzoxazone itself rarely can induce an idiosyncratic hepatocellular damage.

Title: Study of cytochrome P450 2E1 and its allele variants in liver injury of nondiabetic, nonalcoholic steatohepatitis obese women.
CYP2E1 enzyme is related to nonalcoholic steatohepatitis (NASH) due to its ability for reactive oxygen species production, which can be influenced by polymorphisms in the gene. The aim of this study was to investigate hepatic levels, activity, and polymorphisms of the CYP2E1 gene to correlate it with clinical and histological features in 48 female obese NASH patients. Subjects were divided into three groups: (i) normal; (ii) steatosis; and (iii) steatohepatitis. CYP2E1 protein level was assayed in microsomes from liver biopsies, and in vivo chlorzoxazone hydroxylation was determined by HPLC. Genomic DNA was isolated for genotype analysis through PCR. The results... (truncated)

Title: Cellular imaging predictions of clinical drug-induced liver injury.
Drug-induced liver injury (DILI) is the most common adverse event causing drug nonapprovals and drug withdrawals. Using drugs as test agents and measuring a panel of cellular phenotypes that are directly linked to key mechanisms of hepatotoxicity, we have developed an in vitro testing strategy that is predictive of many clinical outcomes of DILI. Mitochondrial damage, oxidative stress, and intracellular glutathione, all measured by high content cellular imaging in primary human hepatocyte cultures, are the three most important features contributing to the hepatotoxicity prediction. When applied to over 300 drugs and chemicals including many that caused rare and idiosyncratic liver... (truncated)

Title: Silymarin Protects against Acute Liver Injury Induced by Acetaminophen by Downregulating the Expression and Activity of the CYP2E1 Enzyme.
Previous studies have shown that silymarin protects against various types of drug-induced liver injury, but whether the protective mechanism of silymarin against acetaminophen-induced liver injury is related to the CYP2E1 enzyme remains unclear. In this study, we investigated the effect of silymarin on the activity and expression of CYP2E1 in vitro and in vivo. The results of in vitro studies showed that silymarin not only inhibited the activity of CYP2E1 in human and rat liver microsomes but also reduced the expression of CYP2E1 in HepG2 cells. In vivo studies showed that silymarin pretreatment significantly reduced the conversion of chlorzoxazone to... (truncated)

Title: Dietary restriction of energy and sugar results in a reduction in human cytochrome P450 2E1 activity.
Dietary habits are often considered as a pathogenic factor for fatty liver. The impact of dietary intake and steatosis on drug metabolism remains poorly investigated. Our aim was to assess the effect of dietary intake on in vivo cytochrome P450 (CYP) activities in eleven patients with abnormal liver function tests potentially due to fatty liver and associated with a high-sugar diet. Liver function tests, liver volume, aminopyrine breath test (ABT) and chlorzoxazone (CZ) pharmacokinetics (area under the curve, AUC) which are known to reflect CYP2E1 activity were evaluated before and after 2 months restriction of dietary sugar intake. Features at... (truncated)

MESH:C056498 - aceclofenac

Summary:

LLM Prediction Score	0.500 (normalized)
LLM Confidence Score	0.970
Golden Answer (Severity Class)	0.0 (normalized)
Prediction Error	0.500

Retrieved Context:

Title: [NSAID-induced hepatotoxicity: aceclofenac and diclofenac].
Diclofenac (Voltaren, Geigy) and aceclofenac (Falcol, Bayer) belong to a chemical subgroup of nonsteroidal antiinflammatory drugs (NSAID) that are arylalkanoic groups of phenylacetic acid. Hepatotoxicity has been reported with the majority of NSAID although its incidence is not known. We describe two patients who presented hepatic injury after diclofenac and aceclofenac therapy. Recognition of cholestasis associated with NSAID may prevent invasive investigations in jaundiced patients. Clinically liver injury associated with NSAID is not prevalent but we think it is interesting to report those patients for recognition of the role of certain risk factors and to suggest close monitoring.

Title: Aceclofenac-induced hepatotoxicity: An ameliorative effect of <i>Terminalia bellirica</i> fruit and ellagic acid.
Label="BACKGROUND" NlmCategory="BACKGROUND">Aceclofenac (ACF), a widely used nonsteroidal anti-inflammatory drug, has been associated with a number of severe cases of clinical hepatotoxicity. <i>Terminalia bellirica,</i> an evergreen tree, is known to have several ethnomedicinal uses including antioxidant and hepatoprotective effects. Hence <i>T. bellirica</i> fruit extracts and its phytoconstituent ellagic acid (EA) are expected to provide protection against oxidative stress and liver damage produced by long-term use of ACF.

Title: Incidence of spontaneous notifications of adverse reactions with aceclofenac, meloxicam, and rofecoxib during the first year after marketing in the United Kingdom.
The objective was to compare the incidence of adverse reactions reported with three nonsteroidal anti-inflammatory drugs with different cyclo-oxygenase (COX)-2 selectivity. All spontaneous adverse reaction notifications in the pharmacovigilance database of the World Health Organisation Collaborating Centre for International Drug Monitoring with aceclofenac, meloxicam, and rofecoxib that were recorded during the first year of marketing were included. The incidence rate (adverse reactions/10(6) defined daily dose) and 95% confidence interval for total adverse reactions was 8.7 (6.1-12.0) for aceclofenac, 24.8 (23.1-26.6) for meloxicam, and 52.6 (49.9-55.4) for rofecoxib. Aceclofenac had a lower incidence of gastrointestinal bleeding, abdominal pain, and arterial hypertension... (truncated)

Title: [Hepatic tolerance of aceclofenac].
NSAID's are largely used drugs. Among the reported side effects of this type of drugs is liver damage. 73 patients suffering from diverse rheumatological pathologies (arthrosis 46.6%, rotators 9.68%, rheumatoid arthritis 21.92%, lumbalgia 5.48%, other diagnosis 6.85%) were evaluated. Aceclofenac (AC) 100 mg. dose every 12 hours was administered. A Transaminase value determination was taken both at the beginning and end of medication for each patient. Average age of patients was 52.71 years, 78% being female. Treatment was administered as follows, 42.47% of the patients received a 60 days treatment, 28.8% a 45 days treatment, 23.3% a 30 days treatment... (truncated)

Title: A Review of Aceclofenac: Analgesic and Anti-Inflammatory Effects on Musculoskeletal Disorders.
Aceclofenac is an oral non-steroidal anti-inflammatory drug (NSAID) with anti-inflammatory and analgesic properties. Although there are some differences in the authorized indications between countries, aceclofenac is mainly recommended for the treatment of inflammatory and painful processes, such as low back pain (LBP), scapulohumeral periarthritis, extraarticular rheumatism, odontalgia, and osteoarthritis (OA), rheumatoid arthritis (RA), and ankylosing spondylitis (AS). The analgesic properties and tolerability profile of aceclofenac in musculoskeletal disorders are reviewed, focusing on relevant and recent studies. The efficacy and safety comparison of aceclofenac with other analgesics and anti-inflammatory agents in OA, AS, RA, and LBP is described. Relevant studies were... (truncated)

MESH:D013739 - testosterone

Summary:

LLM Prediction Score	0.500 (normalized)
LLM Confidence Score	0.990
Golden Answer (Severity Class)	1.0 (normalized)
Prediction Error	0.500

Retrieved Context:

Title: Low Serum Total Testosterone Is Associated with Non-Alcoholic Fatty Liver Disease in Men but Not in Women with Type 2 Diabetes Mellitus.
There were 1155 patients with T2DM included in the analysis. Serum levels of total testosterone and the precursors of androgens, including androstenedione, DHEA, and DHEAS, were quantified using liquid chromatography-tandem mass spectrometry assays.

Title: Anabolic Steroid-Induced Cholestatic Liver Injury: A Case Report.
Owing to performance-enhancing and cosmetic effects, illicit use of anabolic-androgenic steroids (AAS) has been well-described and can be associated with significant complications. We report a 27-year-old Caucasian male who self-medicated with AAS in the form of intramuscular injections and oral testosterone for a one-year duration. He complained of persistent jaundice and moderate generalized itching for one month. On admission, his total bilirubin level was 11.4 mg/dl (normal: 0-1.2 mg/dl), and liver enzymes were slightly elevated. On follow-up, the patient stated complete resolution of symptoms and near-normalization of lab results after one month of conservative management.

Title: Androgenic/Anabolic steroid-induced toxic hepatitis.
Athletes and bodybuilders often misuse androgenic/anabolic steroids. When used in therapeutic doses, these drugs produce clinical jaundice in just a small number of recipients. We present a 26-year-old male bodybuilder who self-administered high doses of androgenic/anabolic steroids that induced liver damage. One month before admission to the hospital, he used testosterone enanthate (500 mg intramuscularly, twice weekly), stanozolol (40 mg/d), and methylandrostenediol (30 mg/d by mouth, for 5 weeks). On admission, his bilirubin level was 470 micromol/L (direct, 360 micromol/L), his aspartate aminotransferase (AST) level was 5,870 IU/L, his alanine aminotransferase (ALT) level was 10,580 IU/L, his alkaline phosphatase (ALP)... (truncated)

Title: Selective Androgen Receptor Modulator Induced Hepatotoxicity.
Selective androgen receptor modulators (SARMs) have been developed as an alternate to traditional anabolic steroids due to their favorable effects on the bones and muscles without androgenic side effects. They are very popular among athletes and bodybuilders and are available online or over the counter. The FDA has warned of their side effects including liver injury. Here we present the case of a 29-year-old patient who presented with jaundice, fatigue, and elevated liver function tests after starting SARM supplements. His symptoms improved and eventually resolved with stopping the supplements. The purpose of this case report is to raise awareness and... (truncated)

Title: Anabolic androgenic steroid-induced liver injury: An update.
Anabolic androgenic steroids (AASs) are a group of molecules including endogenous testosterone and synthetic derivatives that have both androgenic and anabolic effects. These properties make them therapeutically beneficial in medical conditions such as hypogonadism. However, they are commonly bought illegally and misused for their anabolic, skeletal muscle building, and performance-enhancing effects. Supraphysiologic and long-term use of AASs affects all organs, leading to cardiovascular, neurological, endocrine, gastrointestinal, renal, and hematologic disorders. Hepatotoxicity is one of the major concerns regarding AASs treatment and abuse. Testosterone and its derivatives have been most often shown to induce a specific form of cholestasis, peliosis hepatis,... (truncated)

MESH:D008713 - methimazole

Summary:

LLM Prediction Score	0.500 (normalized)
LLM Confidence Score	0.970
Golden Answer (Severity Class)	1.0 (normalized)
Prediction Error	0.500

Retrieved Context:

Title: [Methimazole-induced cholestatic jaundice in a hyperthyroid patient].
Hyperthyroidism is one of the most frequent endocrine disorders and its current treatment is based on drugs, surgery and radioactive iodine. Methimazole is the antithyroid drug of choice because of its potency and infrequent side effects, usually mild. This medication is rarely associated with liver toxicity, usually manifested as cholestatic jaundice. Here we report the case of a 33-year-old woman treated at the University Hospital Fundación Santa Fe de Bogota, with hepatotoxicity induced by a methimazole-based treatment for Graves' disease. The pruritus and jaundice appeared after three weeks of therapy, viral hepatitis markers were negative, hepatobiliary ultrasonography was normal, and... (truncated)

Title: [Methimazole and propylthiouracil induced acute toxic hepatitis].
Hyperthyroidism is one of the most common endocrinology disorders. Treatment can be either pharmacological, surgical or using radioactive iodine. In Europe methimazole is the antithyroid drug of choice because it can be administered in a single daily dose and has a lower risk of adverse reactions. Around 5% of patients taking thionamides can present any of their side effects, which are usually mild. Liver toxicity due to thionamides is very rare, and severe due to propylthiouracil. We present a clinical case of a cholestatic jaundice and acute toxic hepatitis due to methimazole and a cross-reaction with propylthiouracil. Based on this... (truncated)

Title: Methimazole-induced cholestatic hepatitis: two cases report and literature review.
Methimazole is commonly prescribed for patients who are thyrotoxic. Cholestatic hepatitis is a rare but serious adverse event which may be associated with interventional therapy. In this case report, we present two Chinese women with cholestatic jaundice due to methimazole treatment. Both patients had a history of hyperthyroidism; initial laboratory studies of liver function were normal and cholestatic hepatitis occurred after treatment with methimazole. Concomitant liver disease, such as viral hepatitis (A, B, C, D, E), autoimmune hepatitis, primary biliary cirrhosis and calculus of bile duct, were excluded. Liver enzyme levels in both patients returned to normal after stopping methimazole... (truncated)

Title: Hepatotoxicity induced by methimazole in a previously healthy patient.
We report a case of hepatotoxicity induced by methimazole treatment in a patient affected by hyperthyroidism. A 54-year-old man, presented to our observation for palpitations, excessive sweating, weakness, heat intolerance and weight loss. On physical examination, his blood pressure was 140/90 mmHg and heart beat was 100/min regular. He had mild tremors and left exophthalmos. Laboratory test revealed a significant increase in serum thyroid hormone levels with a decrease in thyroid stimulating hormone levels. A diagnosis of hyperthyroidism was made and he began treatment with methimazole (30 mg/day). Fourteen days later, he returned for the development of scleral icterus, followed... (truncated)

Title: Delayed cholestatic hepatitis due to methimazole.
We report on a 71-year-old man who presented to the medical department of Our Lady of Maryknoll Hospital with progressive cholestatic hepatitis. Tests for hepatitis viral markers gave negative results and ultrasonography revealed no dilated bile ducts. Endoscopic retrograde cholangiopancreatography showed a normal biliary tree. The patient had completed a 5-month course of methimazole to treat thyrotoxicosis a few weeks before the onset of the jaundice. Methimazole was suspected to be the cause of the cholestatic hepatitis; this diagnosis was supported by the results from a liver biopsy. The presentation of the patient was unusual by virtue of the delayed... (truncated)

MESH:D000069549 - ustekinumab

Summary:

LLM Prediction Score	0.500 (normalized)
LLM Confidence Score	0.970
Golden Answer (Severity Class)	0.0 (normalized)
Prediction Error	0.500

Retrieved Context:

Title: Liver Injury in Psoriasis Patients Receiving Ustekinumab: A Retrospective Study of 44 Patients Treated in the Clinical Practice Setting.
The therapy of patients with psoriasis and liver disease can be a challenge due to the increased risk of adverse effects from traditional systemic treatments; in addition, although the anti-tumor necrosis factor agents are considered safer, they have also been associated with drug-induced liver injury and reactivation of viral hepatitis. Ustekinumab has a different mechanism of action and the little that is known of its effects on the liver comes from pivotal studies. The objectives of this study were to estimate the incidence of drug-induced liver injury in patients treated with ustekinumab in daily clinical practice and to analyze liver... (truncated)

Title: A Case of Severe Transaminase Elevation Following a Single Ustekinumab Dose with Remission After Drug Withdrawal.
Ustekinumab is a fully human monoclonal antibody which binds Interleukin (IL)-12/23. It is indicated for the treatment of moderate-severe psoriasis and active psoriatic arthritis. Few data are available about the possibility of an interaction between ustekinumab and the liver.

Title: Review article: safety of new biologic agents for inflammatory bowel disease in the liver.
New biologic agents (vedolizumab, ustekinumab and tofacitinib) represent an effective treatment for inflammatory bowel diseases and have been recently approved. However, with a rapidly evolving complement of advanced targeted therapies, new concerns about their potentially undesirable effects on liver function emerge. In particular, little is known about safety data in patients with hepatitis B virus, hepatitis C virus chronic infections, cirrhosis and in transplanted patients who are accumulating. In addition, these new agents have also been associated with drug-induced liver injury. Limited data on the efficacy of vedolizumab in patients with primary sclerosing cholangitis are also available. This article reviews... (truncated)

Title: The impact of the interleukin 12/23 inhibitor ustekinumab on the risk of infections in patients with psoriatic arthritis.
Introduction: Psoriatic arthritis (PsA) is characterized by chronic inflammation mediated by pro-inflammatory cytokines, with clinical features resulting from dysfunctional integrated signaling pathways affecting different constituents of the immune system. Increased understanding of the processes responsible for enthesitis, synovial inflammation, joint erosion, and new bone formation during PsA has led to development of biologic therapies targeting these cytokines. There is an increased risk of opportunistic infections in patients with PsA, and this risk is increased further with targeted biologic therapy.Areas covered: This paper reviews the role of the interleukin (IL)-12, IL-23 and IL-17 axis in the pathogenesis of PsA. The data... (truncated)

Title: Liver-side of inflammatory bowel diseases: Hepatobiliary and drug-induced disorders.
Hepatobiliary disorders are among the most common extraintestinal manifestations in inflammatory bowel diseases (IBD), both in Crohn's disease and ulcerative colitis (UC), and therefore represent a diagnostic challenge. Immune-mediated conditions include primary sclerosing cholangitis (PSC) as the main form, variant forms of PSC (namely small-duct PSC, PSC-autoimmune hepatitis overlap syndrome and IgG4-related sclerosing cholangitis) and granulomatous hepatitis. PSC is by far the most common, presenting in up to 8% of IBD patients, more frequently in UC. Several genetic foci have been identified, but environmental factors are preponderant on disease pathogenesis. The course of the two diseases is typically independent. PSC... (truncated)

MESH:D000093542 - gemcitabine

Summary:

LLM Prediction Score	0.500 (normalized)
LLM Confidence Score	0.990
Golden Answer (Severity Class)	1.0 (normalized)
Prediction Error	0.500

Retrieved Context:

Title: Gemcitabine-induced liver fibrosis in a patient with pancreatic cancer.
Gemcitabine is the only cytotoxic agent approved by FDA for the treatment of pancreatic carcinoma. Gemcitabine has a relatively safe profile. Major side effects include bone marrow suppression and flu-like syndrome. Transient abnormalities of liver transaminase enzymes are seen in two third of patients: elevations of alkaline phosphatase and bilirubin are less common, but severe hepatic toxicity is uncommon. Four case reports regarding severe hepatic toxicity of gemcitabine leading to rapid deterioration in patients' health status and death have been reported. We report the fifth case in which liver functions were within normal limits but liver toxicity was preceded by... (truncated)

Title: A Severe Case of Drug-Induced Liver Injury after Gemcitabine Administration: A Highly Probable Causality Grading as Assessed by the Updated RUCAM Diagnostic Scoring System.
Gemcitabine is an antineoplastic drug used in several forms of advanced pancreatic, lung, breast, ovarian, and bladder cancer. Common side effects include bone marrow suppression, fatigue, diarrhea, nausea, gastrointestinal upset, rash, alopecia, and stomatitis. Transient serum enzyme elevations could be observed during therapy, but clinically significant acute liver injury has been rarely associated with its use. Few cases of acute liver injury have been reported in the literature. We reported the clinical case of a 73--year-old man who developed clinically significant acute hepatic injury after using gemcitabine. Possible causes, clinical presentation, and treatments are discussed. According to the updated RUCAM... (truncated)

Title: Phase II studies of gemcitabine for non-small cell lung cancer in Japan.
To determine the activity and toxicity of gemcitabine (2',2'-difluorodeoxycytidine), three phase II single-agent studies have been conducted in patients with non-small cell lung cancer in Japan. In an early phase II study, 17 previously treated and 47 untreated patients were treated with gemcitabine. Gemcitabine was given intravenously at a dose of 800 mg/m2 or 1,000 mg/m2 once a week for 3 weeks followed by a week of rest, repeating every 4 weeks. Although none of the patients with prior therapy responded, eight (17%) of 47 previously untreated patients showed a partial response. Toxicities of grade 3 or greater included leukopenia... (truncated)

Title: [Phase II studies of gemcitabine for non-small cell lung cancer in Japan].
To determine the activity and toxicity of gemcitabine in non-small cell lung cancer, three phase II studies of single agent gemcitabine have been conducted between 1990 and 1994. In an early phase II study, gemcitabine was administered of 800 mg/m2 on day 1, 8, 15 every four weeks (step I), and 1,000 mg/m2 (step II). Response was observed in 3 of 13 patients with previously untreated non-small cell lung cancer, although there was no responders in the previously treated patients. Late phase II studies were performed at 20 (group A) and 24 (group B) Japanese institutions to confirm the efficacy... (truncated)

Title: Safety profile of gemcitabine.
This paper reviews the toxicity profile of gemcitabine in a large group of patients (up to 790) from pivotal phase II studies, in which the drug was given intravenously as a 30 min infusion, in a schedule once a week for 3 weeks followed by a week of rest. The safety profile of gemcitabine is unusually mild for such an active agent in solid tumours. Haematological toxicity is mild and short-lived with modest WHO grades 3 and 4 for haemoglobin (6.4% and 0.9% of patients), leukocytes (8.1% and 0.5%), neutrophils (18.7% and 5.7%) and platelets (6.4% and 0.9%). The incidence... (truncated)

MESH:D009638 - norepinephrine

Summary:

LLM Prediction Score	0.500 (normalized)
LLM Confidence Score	0.990
Golden Answer (Severity Class)	0.0 (normalized)
Prediction Error	0.500

Retrieved Context:

Title: Sympathetic nervous system catecholamines and neuropeptide Y neurotransmitters are upregulated in human NAFLD and modulate the fibrogenic function of hepatic stellate cells.
Sympathetic nervous system (SNS) signalling regulates murine hepatic fibrogenesis through effects on hepatic stellate cells (HSC), and obesity-related hypertension with SNS activation accelerates progression of non-alcoholic fatty liver disease (NAFLD), the commonest cause of chronic liver disease. NAFLD may lead to cirrhosis. The effects of the SNS neurotransmitters norepinephrine (NE), epinephrine (EPI) and neuropeptide Y (NPY) on human primary HSC (hHSC) function and in NAFLD pathogenesis are poorly understood.

Title: The Role of Catecholamines in Pathophysiological Liver Processes.
Over the last few years, the number of research publications about the role of catecholamines (epinephrine, norepinephrine, and dopamine) in the development of liver diseases such as liver fibrosis, fatty liver diseases, or liver cancers is constantly increasing. However, the mechanisms involved in these effects are not well understood. In this review, we first recapitulate the way the liver is in contact with catecholamines and consider liver implications in their metabolism. A focus on the expression of the adrenergic and dopaminergic receptors by the liver cells is also discussed. Involvement of catecholamines in physiological (glucose metabolism, lipids metabolism, and liver... (truncated)

Title: Sympathomimetic amine compounds and hepatotoxicity: Not all are alike-Key distinctions noted in a short review.
Sympathomimetic amine compounds are often pooled together and incorrectly assumed to be interchangeable with respect to potential adverse effects. A brief and specific review of sympathomimetic compounds and one instance (i.e., hepatotoxicity) where these compounds have been improperly grouped together is covered. A review of the proposed mechanisms through which known hepatotoxic sympathomimetic agents (e.g., 3,4-methylenedioxymethamphetamine or MDMA, methamphetamine and amphetamine) cause liver injury, along with a corresponding review of in vitro data, interventional data, animal model studies and observational data allow for a comparison/contrast of different agents and reveals a lack of potential toxicity for some agents (e.g., pseudoephedrine,... (truncated)

Title: Neuroimmunomodulation of adrenoblockers during liver cirrhosis: modulation of hepatic stellate cell activity.
The sympathetic nervous system and the immune system are responsible for producing neurotransmitters and cytokines that interact by binding to receptors; due to this, there is communication between these systems. Liver immune cells and nerve fibres are systematically distributed in the liver, and the partial overlap of both patterns may favour interactions between certain elements. Dendritic cells are attached to fibroblasts, and nerve fibres are connected via the dendritic cell-fibroblast complex. Receptors for most neuroactive substances, such as catecholamines, have been discovered on dendritic cells. The sympathetic nervous system regulates hepatic fibrosis through sympathetic fibres and adrenaline from the adrenal... (truncated)

Title: Beta-adrenergic receptor 1 selective antagonism inhibits norepinephrine-mediated TNF-alpha downregulation in experimental liver cirrhosis.
Bacterial translocation is a frequent event in cirrhosis leading to an increased inflammatory response. Splanchnic adrenergic system hyperactivation has been related with increased bacterial translocation. We aim at evaluating the interacting mechanism between hepatic norepinephrine and inflammation during liver damage in the presence of bacterial-DNA.

MESH:D013419 - sulfamethizole

Summary:

LLM Prediction Score	0.500 (normalized)
LLM Confidence Score	0.900
Golden Answer (Severity Class)	0.0 (normalized)
Prediction Error	0.500

Retrieved Context:

Title: Drug-induced liver disease in Denmark. An analysis of 572 cases of hepatotoxicity reported to the Danish Board of Adverse Reactions to Drugs.
During the decade 1968-1978 the Danish Board of Adverse Reactions to Drugs received 572 (6% of the total number) reports on hepatotoxicity. Halothane amounted to one fourth of the reported cases. Among the 94 psychotropic-induced adverse drug reactions 54 cases were attributed to chlorpromazine, 10 to tricyclic antidepressants, and only 2 to benzodiazepines. Considering the drug consumption data, the combination trimethoprim-sulfamethoxazole is nearly five times more frequently associated with hepatotoxicity than administration of sulfamethizole. Almost two thirds of the hepatotoxic reactions were classified as cytotoxic. Halothane, oxyphenisatin, rifampicin, alfa-methyldopa, papaverine, phenytoin, and ajmaline were almost exclusively related to cytotoxic reactions.... (truncated)

Title: Multiple attacks of jaundice associated with repeated sulfonamide treatment.
Four women who were treated with sulfonamides because of recurrent urinary tract infections experienced adverse liver reactions with jaundice during their third, fourth and fifth course of treatment, respectively. In spite of this, sulfonamide treatment was reinitiated some years later. Adverse liver reactions with jaundice recurred on all occasions. The clinical picture of the liver reactions was indistinguishable from that of viral hepatitis and a hepatitis-like reaction was also seen histologically. Signs of fibrosis appeared histologically after a third attack of jaundice associated with sulfonamides in one patient, but otherwise no persisting abnormalities were noted.

Title: Hepatic safety of antibiotics used in primary care.
Antibiotics used by general practitioners frequently appear in adverse-event reports of drug-induced hepatotoxicity. Most cases are idiosyncratic (the adverse reaction cannot be predicted from the drug's pharmacological profile or from pre-clinical toxicology tests) and occur via an immunological reaction or in response to the presence of hepatotoxic metabolites. With the exception of trovafloxacin and telithromycin (now severely restricted), hepatotoxicity crude incidence remains globally low but variable. Thus, amoxicillin/clavulanate and co-trimoxazole, as well as flucloxacillin, cause hepatotoxic reactions at rates that make them visible in general practice (cases are often isolated, may have a delayed onset, sometimes appear only after cessation... (truncated)

Title: Association of CYP1A1 and CYP1B1 inhibition in in vitro assays with drug-induced liver injury.
Drug-induced liver injury (DILI) is one of the major causes for the discontinuation of drug development and withdrawal of drugs from the market. Since it is known that reactive metabolite formation and being substrates or inhibitors of cytochrome P450s (P450s) are associated with DILI, we systematically investigated the association between human P450 inhibition and DILI. The inhibitory activity of 266 DILI-positive drugs (DILI drugs) and 92 DILI-negative drugs (no-DILI drugs), which were selected from Liver Toxicity Knowledge Base (US Food and Drug Administration), against 8 human P450 forms was assessed using recombinant enzymes and luminescent substrates, and the threshold values... (truncated)

Title: An Automated Solid-Phase Extraction-UPLC-MS/MS Method for Simultaneous Determination of Sulfonamide Antimicrobials in Environmental Water.
The large-scale use of sulfonamide antimicrobials in human and veterinary medicine has seriously endangered the ecological environment and human health. The objective of this study was to develop and validate a simple and robust method for the simultaneous determination of seventeen sulfonamides in water using ultra-high performance liquid chromatography-tandem mass spectrometry coupled with fully automated solid-phase extraction. Seventeen isotope-labeled internal standards for sulfonamides were used to correct matrix effects. Several parameters affecting extraction efficiency were systematically optimized, and the enrichment factors were up to 982-1033 and only requiring about 60 min per six samples. Under the optimized conditions, this method... (truncated)

MESH:D000068258 - bevacizumab

Summary:

LLM Prediction Score	0.500 (normalized)
LLM Confidence Score	0.980
Golden Answer (Severity Class)	0.0 (normalized)
Prediction Error	0.500

Retrieved Context:

Title: Sclerosing cholangitis in the era of target chemotherapy: a possible anti-VEGF effect.
Preoperative systemic chemotherapy is generally applied in patients who undergo hepatic resection for colorectal metastases. Although the tumour response rate has been improved recently with the development of new molecular targeted therapies the related hepatic injury is ill defined. Bevacizumab is a monoclonal antibody to vascular endothelial growth factor. It can achieve high response rates and is accepted as a first line treatment in the metastatic colorectal disease. However, the data about its hepatotoxicity profile is still limited. We describe a case of secondary sclerosing cholangitis in a patient with liver metastases treated by Bevacizumab in the neoadjuvant setting and... (truncated)

Title: Hepatotoxicity following systemic therapy for colorectal liver metastases and the impact of chemotherapy-associated liver injury on outcomes after curative liver resection.
Patients with colorectal liver metastases (CLM) have remarkably benefited from the advances in medical multimodal treatment and surgical techniques over the last two decades leading to significant improvements in long-term survival. More patients are currently undergoing liver resection following neoadjuvant chemotherapy, which has been increasingly established within the framework of curative-indented treatment strategies. However, the use of several cytotoxic agents has been linked to specific liver injuries that not only impair the ability of liver tissue to regenerate but also decrease long-term survival. One of the most common agents included in modern chemotherapy regimens is oxaliplatin, which is considered to... (truncated)

Title: Sinusoidal obstruction syndrome and nodular regenerative hyperplasia are frequent oxaliplatin-associated liver lesions and partially prevented by bevacizumab in patients with hepatic colorectal metastasis.
Because of its efficacy, oxaliplatin (OX) is increasingly used as a chemotherapeutic agent in the treatment of colorectal liver metastases (CRLM). Oxaliplatin-associated liver toxicity has been reported and can affect clinical practice, but studies on its prevalence and a full pathological description are lacking. The aims of this study were to fill this gap by providing, from a pathologist's perspective, a detailed assessment of the spectrum of hepatic lesions associated with OX, to suggest a scoring system to quantify them, and to investigate the protective effect of bevacizumab against OX-associated damage.

Title: Bevacizumab exacerbates sinusoidal obstruction syndrome (SOS) in the animal model and increases MMP 9 production.
Thanks to modern multimodal treatment the ouctome of patients with colorectal cancer has experienced significant improvements. As a downside, agent specific side effects have been observed such as sinusoidal obstruction syndrome (SOS) after oxaliplatin chemotherapy (OX). Bevacizumab targeting VEGF is nowadays comprehensively used in combination protocols with OX but its impact on hepatotoxicity is thus far elusive and focus of the present study.

Title: Histopathologic evaluation of liver metastases from colorectal cancer in patients treated with FOLFOXIRI plus bevacizumab.
The FOLFOXIRI regimen produces a high rate of radiological and histopathological responses. Bevacizumab added to chemotherapy showed an improvement in pathological response and necrosis of colorectal liver metastases (CLMs). FOLFOXIRI plus bevacizumab produced promising early clinical results and is under investigation in several randomised trials, although no data are currently available on its effects on response of CLMs and on liver toxicities.

MESH:C426686 - ximelagatran

Summary:

LLM Prediction Score	0.500 (normalized)
LLM Confidence Score	0.910
Golden Answer (Severity Class)	1.0 (normalized)
Prediction Error	0.500

Retrieved Context:

Title: Ximelagatran: direct thrombin inhibitor.
Warfarin sodium is an effective oral anticoagulant drug. However, warfarin has a narrow therapeutic window with significant risks of hemorrhage at therapeutic concentrations. Dosing is difficult and requires frequent monitoring. New oral anticoagulant agents are required to improve current anticoagulant therapy. Furthermore, while warfarin is effective in venous disease, it does not provide more than 60% risk reduction compared with placebo in venous thrombosis prophylaxis and considerably lower risk reduction in terms of arterial thrombosis. Ximelagatran is an oral pro-drug of melagatran, a synthetic small peptidomimetic with direct thrombin inhibitory actions and anticoagulant activity. As an oral agent, ximelagatran has... (truncated)

Title: Safety assessment of new antithrombotic agents: lessons from the EXTEND study on ximelagatran.
Ximelagatran, the first oral direct thrombin inhibitor, was shown to be an effective antithrombotic agent but was associated with potential liver toxicity after prolonged administration.

Title: Drug-induced liver injury in humans: the case of ximelagatran.
Ximelagatran was the first orally available direct thrombin inhibitor under clinical development that also reached the market. Ximelagatran was tested in an extensive clinical programme. Short-term use (<12 days) in humans including the phase III clinical trials did not indicate any hepatotoxic potential. Increased hepatic enzyme levels were first observed at a higher frequency when evaluating the long-term (>35 days) use of ximelagatran (incidence of >3x upper limit of normal (ULN) plasma ALT was 7.9%). The frequency of elevated total bilirubin levels was similar in the ximelagatran and the comparator groups. However, the combination of ALT > 3x ULN and... (truncated)

Title: Idiosyncratic drug reactions: past, present, and future.
Although the major working hypothesis for the mechanism of idiosyncratic drug reactions (IDRs), the hapten hypothesis, has not changed since 1987, several hypotheses have been added, for example, the danger hypothesis and the pharmaceutical interaction hypothesis. Genetic studies have found that several IDRs are linked to specific HLA genes, providing additional evidence that they are immune-mediated. Evidence that most IDRs are caused by reactive metabolites has led pharmaceutical companies to avoid drug candidates that form significant amounts of reactive metabolites; however, at least one IDR, ximelagatran-induced liver toxicity, does not appear to be caused by a reactive metabolite. It is... (truncated)

Title: Ximelagatran in prevention of cardiovascular events.
Ximelagatran (Exanta (R) is the first oral anticoagulant in a new class of drugs called direct thrombin inhibitors. Two studies suggest that ximelagatran is at least as effective as warfarin in preventing stroke in high risk patients with atrial fibrillation. Ximelagatran may also reduce the rate of major cardiovascular events after a myocardial infarction, compared to placebo. Ximelagatran does not require dose adjustments or routine blood monitoring. As with warfarin, bleeding risks increase with higher doses of ximelagatran. There is, however, no specific antidote to help manage bleeding. The safety of ximelagatran will not be fully known without further evaluation... (truncated)

MESH:C100286 - methsuximide

Summary:

LLM Prediction Score	0.495 (normalized)
LLM Confidence Score	0.860
Golden Answer (Severity Class)	0.0 (normalized)
Prediction Error	0.495

Retrieved Context:

Title: Synthesis, Molecular Docking, and Preclinical Evaluation of a New Succinimide Derivative for Cardioprotective, Hepatoprotective and Lipid-Lowering Effects.
Cardiac and hepatotoxicities are major concerns in the development of new drugs. Better alternatives to other treatments are being sought to protect these vital organs from the toxicities of these pharmaceuticals. In this regard, a preclinical study is designed to investigate the histopathological effects of a new succinimide derivative (Comp-1) on myocardial and liver tissues, and the biochemical effects on selected cardiac biomarkers, hepatic enzymes, and lipid profiles. For this, an initially lethal/toxic dose was determined, followed by a grouping of selected albino rats into five groups (each group had n = 6). The control group received daily oral saline... (truncated)

Title: Safety of anticonvulsants in hepatic porphyrias.
Because acute attacks of porphyria may be precipitated by anticonvulsants, a therapeutic dilemma arises when seizures complicate hepatic porphyria. The list of unsafe agents includes barbiturates, primidone, phenytoin, mephenytoin, ethotoin, ethosuximide, methsuximide, phensuximide, and trimethadione. Agents are considered unsafe if they induce experimental porphyria in animals, and short trials in patients are unreliable for screening. Using drug incubation in chick-embryo hepatocyte culture, we found that porphyrin was increased by carbamazepine, clonazepam, and valproate. These agents should probably be avoided or used with caution in porphyric patients. Alternative approaches to acute porphyric attacks with seizures are discussed.

Title: Clinical characteristics of antiepileptic-induced liver injury in patients from the DILIN prospective study.
Antiepileptic drugs (AEDs) are a common cause of drug-induced liver injury (DILI). Over the last few decades, several newer AEDs were approved for marketing in the United States, and they are increasingly prescribed for indications other than seizures. Contemporaneous data related to trends and characteristics of AED-related liver injury are sparse.

Title: Pharmacokinetic characteristics of antiepileptic drugs (AEDs).
Antiepileptic drugs (AEDs) are routinely prescribed for the management of a variety of neurologic and psychiatric conditions, including epilepsy and epilepsy syndromes. Physiologic changes due to aging, pregnancy, nutritional status, drug interactions, and diseases (ie, those involving liver and kidney function) can affect pharmacokinetics of AEDs. This review discusses foundational pharmacokinetic characteristics of AEDs currently available in the United States, including clobazam but excluding the other benzodiazepines. Commonalities of pharmacokinetic properties of AEDs are discussed in detail. Important differences among AEDs and clinically relevant pharmacokinetic interactions in absorption, distribution, metabolism, and/or elimination associated with AEDs are highlighted. In general, newer... (truncated)

Title: State of the Art and Uses for the Biopharmaceutics Drug Disposition Classification System (BDDCS): New Additions, Revisions, and Citation References.
The Biopharmaceutics Drug Disposition Classification system (BDDCS) is a four-class approach based on water solubility and extent of metabolism/permeability rate. Based on the BDDCS class to which a drug is assigned, it is possible to predict the role of metabolic enzymes and transporters on the drug disposition of a new molecular entity (NME) prior to its administration to animals or humans. Here, we report a total of 1475 drugs and active metabolites to which the BDDCS is applied. Of these, 379 are new entries, and 1096 are revisions of former classification studies with the addition of references for the approved... (truncated)

MESH:D011530 - protriptyline

Summary:

LLM Prediction Score	0.492 (normalized)
LLM Confidence Score	0.880
Golden Answer (Severity Class)	0.0 (normalized)
Prediction Error	0.492

Retrieved Context:

Title: The Importance of Patient-Specific Factors for Hepatic Drug Response and Toxicity.
Responses to drugs and pharmacological treatments differ considerably between individuals. Importantly, only 50%-75% of patients have been shown to react adequately to pharmacological interventions, whereas the others experience either a lack of efficacy or suffer from adverse events. The liver is of central importance in the metabolism of most drugs. Because of this exposed status, hepatotoxicity is amongst the most common adverse drug reactions and hepatic liabilities are the most prevalent reason for the termination of development programs of novel drug candidates. In recent years, more and more factors were unveiled that shape hepatic drug responses and thus underlie the... (truncated)

Title: Tricyclic antidepressants induce liver inflammation by targeting NLRP3 inflammasome activation.
Idiosyncratic drug-induced liver injury (IDILI) is common in hepatology practices and, in some cases, lethal. Increasing evidence show that tricyclic antidepressants (TCAs) can induce IDILI in clinical applications but the underlying mechanisms are still poorly understood.

Title: Narcolepsy: current treatment options and future approaches.
The management of narcolepsy is presently at a turning point. Three main avenues are considered in this review: 1) Two tendencies characterize the conventional treatment of narcolepsy. Modafinil has replaced methylphenidate and amphetamine as the first-line treatment of excessive daytime sleepiness (EDS) and sleep attacks, based on randomized, double blind, placebo-controlled clinical trials of modafinil, but on no direct comparison of modafinil versus traditional stimulants. For cataplexy, sleep paralysis, and hypnagogic hallucinations, new antidepressants tend to replace tricyclic antidepressants and selective serotonin reuptake inhibitors (SSRIs) in spite of a lack of randomized, double blind, placebo-controlled clinical trials of these compounds;... (truncated)

Title: Inhibition of acid sphingomyelinase by tricyclic antidepressants and analogons.
Amitriptyline, a tricyclic antidepressant, has been used in the clinic to treat a number of disorders, in particular major depression and neuropathic pain. In the 1970s the ability of tricyclic antidepressants to inhibit acid sphingomyelinase (ASM) was discovered. The enzyme ASM catalyzes the hydrolysis of sphingomyelin to ceramide. ASM and ceramide were shown to play a crucial role in a wide range of diseases, including cancer, cystic fibrosis, diabetes, Alzheimer's disease, and major depression, as well as viral (e.g., measles virus) and bacterial (e.g., Staphylococcus aureus, Pseudomonas aeruginosa) infections. Ceramide molecules may act in these diseases by the alteration of... (truncated)

Title: Review of Pharmacotherapy for Tinnitus.
Various medications are currently used in the treatment of tinnitus, including anesthetics, antiarrhythmics, anticonvulsants, antidepressants, antihistamines, antipsychotics, anxiolytics, calcium channel blockers, cholinergic antagonists, NMDA antagonists, muscle relaxants, vasodilators, and vitamins. To date, however, no medications have been specifically approved to treat tinnitus by the US Food and Drug Administration (FDA). In addition, medicines used to treat other diseases, as well as foods and other ingested materials, can result in unwanted tinnitus. These include alcohol, antineoplastic chemotherapeutic agents and heavy metals, antimetabolites, antitumor agents, antibiotics, caffeine, cocaine, marijuana, nonnarcotic analgesics and antipyretics, ototoxic antibiotics and diuretics, oral contraceptives, quinine and chloroquine,... (truncated)

MESH:D003613 - danazol

Summary:

LLM Prediction Score	0.511 (normalized)
LLM Confidence Score	0.970
Golden Answer (Severity Class)	1.0 (normalized)
Prediction Error	0.489

Retrieved Context:

Title: Long-term therapy of hereditary angioedema with danazol.
We treated 69 patients who had hereditary angioedema with danazol to alleviate attacks of mucocutaneous angioedema involving the skin, oropharynx, and gastrointestinal tract, and we documented the continued efficacy of danazol for long-term treatment (1 to 6 years) of hereditary angioedema. Significant dose-related, adverse reactions occurred, including weight gain, myalgias, headaches, microscopic hematuria, abnormal liver function tests, anxiety, altered libido, alopecia, dizziness, and nausea. Alterations in menstrual function were consistently observed. About 10% of patients noted masculinizing side effects, such as acne, hirsutism, and voice deepening. We recommend downward titration of danazol dosage to achieve control of attacks and minimize... (truncated)

Title: Danazol induces apoptosis and cytotoxicity of leukemic cells alone and in combination with purine nucleoside analogs in chronic lymphocytic leukemia.
Recently, great progress has been achieved in the treatment of chronic lymphocytic leukemia (CLL). However, some patients, particularly older patients with comorbidities or with relapsed/refractory leukemia, still have limited therapeutic options. There is an urgent need to discover less toxic and more effective drugs for CLL patients. Applying new modalities or substances that are widely used for the treatment of other diseases has been reported to improve results in CLL treatment. This study aimed to assess the non-chemotherapeutic drug danazol for its potential to destroy leukemic cells. Leukemic cells, obtained from the peripheral blood and bone marrow of 23 CLL... (truncated)

Title: Morphologic evaluation of the liver in hereditary angioedema patients on long-term treatment with androgen derivatives.
17 alpha-Alkylated androgens are highly effective in preventing attacks in HAE patients. These drugs, however, seem to be implicated in the development of cholestatic jaundice, peliosis hepatis, and liver tumors. In order to assess the risk-benefit balance of the long-term therapy with androgen derivatives, a follow-up investigation was performed in 13 HAE patients. The results of this study indicate that long-term treatment (15 to 47 mo) with low doses of danazol or stanozolol does not induce significant hepatic damage detectable by laboratory tests or liver biopsy. However, the limited number of patients, although in a rather long period of observation,... (truncated)

Title: Refractory bleeding from intestinal angiodysplasias successfully treated with danazol in three patients with von Willebrand disease.
von Willebrand disease (VWD) is associated with development of gastrointestinal (GI) vascular malformations that lead to chronic GI bleeding. Conventional management, including von Willebrand factor concentrate replacement and endoscopic ablation or bowel resection, does not consistently reduce hemorrhage. We describe three patients with VWD for whom conventional management failed to control GI bleeding. We retrospectively reviewed medical records of patients with VWD and GI bleeding. After patients began treatment with danazol, we observed long-term reductions in GI bleeding and packed red blood cell transfusion requirements. One patient had severe liver toxicity and was found to have concomitant primary biliary cirrhosis.... (truncated)

Title: Effect of stanozolol on factors VIII and IX and serum aminotransferases in haemophilia.
The treatment of haemophilia has been dramatically improved since the introduction of factor VIII and IX concentrates, however these concentrates have brought new problems such as hepatitis and A.I.D.S. An oral agent which could raise endogenous levels of factor VIII and IX would be of great benefit. Danazol, an anabolic steroid, has recently been shown to increase levels of factors VIII and IX in haemophilia. We therefore studied the effect of stanozolol, a closely related anabolic steroid, in 15 patients with haemophilia A or Christmas disease over a 2-4 week period. There was no consistent change in factor VIIIc or... (truncated)

MESH:D013629 - tamoxifen

Summary:

LLM Prediction Score	0.511 (normalized)
LLM Confidence Score	0.990
Golden Answer (Severity Class)	1.0 (normalized)
Prediction Error	0.489

Retrieved Context:

Title: Tamoxifen in liver disease: potential exacerbation of hepatic dysfunction.
Tamoxifen, a non-steroidal anti-estrogen, has been used successfully for a decade as post-operative adjuvant therapy for breast cancer. Tamoxifen is generally well tolerated with few side effects, especially at the typical dose of 10 mg twice daily. However, hepatic effects have been reported after tamoxifen administration and are usually found to be cholestatic in nature. Although previous reports concentrate on tamoxifen as a probable cause of drug-induced hepatotoxicity, very little attention has been focused on the use of tamoxifen in patients with pre-existing liver dysfunction and the possible need for dose adjustment. We present the case of a 48-year-old woman... (truncated)

Title: Hepatic toxicity caused by adjuvant CMF/CNF in breast cancer patients and reversal by tamoxifen.
The purpose of the study was to determine the effect of adjuvant chemotherapy on liver enzymes in breast cancer patients. Furthermore, the effect of tamoxifen on liver enzymes was analyzed. Liver function tests from 194 breast cancer patients who received adjuvant chemotherapy with or without tamoxifen (TAM) were reviewed. Statistically very significant increases were seen in alkaline phosphatase, aspartate acetyl transferase, and gamma glutamyl transferase levels in these patients receiving adjuvant chemotherapy. No statistical changes were noticed in bilirubin levels. If tamoxifen was given together with adjuvant chemotherapy, no changes in liver function tests were detected. Hepatic toxicity was induced... (truncated)

Title: Mitochondria: the gateway for tamoxifen-induced liver injury.
Tamoxifen (TAM) is routinely used in the treatment of breast carcinoma. TAM-induced liver injury remains a major concern, as TAM causes hepatic steatosis in a significant number of patients, which can progress toward steatohepatitis. Liver toxicity is generally believed to involve mitochondrial dysfunction and TAM exerts multiple deleterious effects on mitochondria, which may account for the hepatotoxicity observed in patients treated with TAM. Endoxifen (EDX), a key active metabolite of TAM that is being investigated as an alternative to TAM in breast cancer therapy, slightly affects mitochondria in comparison with TAM and this demonstration well correlates with the absence of... (truncated)

Title: Zinc abrogates anticancer drug tamoxifen-induced hepatotoxicity by suppressing redox imbalance, NO/iNOS/NF-κB signaling, and caspase-3-dependent apoptosis in female rats.
Tamoxifen (TAM) is used in breast cancer chemotherapy since its approval by the Food and Drug Administration in 1977. However, TAM therapy is accompanied with hepatotoxicity - a source of worry to clinicians. Oxidative stress and inflammation are the major implicated mechanisms contributing to TAM hepatotoxicity. In this study, we explored whether zinc (Zn) supplementation could prevent TAM-induced hepatotoxicity in female Wistar rats. Rats were subjected to oral pretreatment of Zn (100 mg/kg body weight (b.w.)/day) for 14 days against hepatic toxicity induced by single intraperitoneal administration of TAM (50 mg/kg b.w.) on day 13. TAM markedly elevated serum liver... (truncated)

Title: Tamoxifen induced hepatotoxicity in breast cancer patients with pre-existing liver steatosis: the role of glucose intolerance.
Tamoxifen induced hepatotoxicity has not been investigated in breast cancer patients with pre-existing liver steatosis. The aim of our study was to investigate the most common predisposing factors for non-alcoholic fatty liver disease in breast cancer patients with liver steatosis, treated with adjuvant tamoxifen therapy, in order to evaluate their role in the appearance of tamoxifen induced hepatotoxicity.

MESH:C033343 - ethanolamine oleate

Summary:

LLM Prediction Score	0.488 (normalized)
LLM Confidence Score	0.930
Golden Answer (Severity Class)	0.0 (normalized)
Prediction Error	0.488

Retrieved Context:

Title: [Death after delayed recovery and respiratory failure following injection sclerotherapy in a small infant under general anesthesia].
A 12 month old boy weighing 6.4kg with esophageal varices caused by congenital biliary hypoplasia was scheduled for emergency sclerotherapy under general anesthesia. Anesthesia was induced with thiamylal sodium 3mg.kg-1 i.v. and then maintained with nitrous oxide, oxygen and a low concentration of enflurane, paralysed with pancuronium bromide. As soon as a small dose of sclerosant (5% ethanolamine oleate) was injected, transient moderate bradycardia and hypotension occurred. As his spontaneous breathing was very weak and the movements of extremities convulsive and his consciousness drowsy, prophylactic respiratory care was carried out. He had pneumonia and manifestation of DIC 4 days after... (truncated)

Title: [Deterioration of liver function following endoscopic injection sclerotherapy of esophageal varices--significance of the new liver function test "intravariceal injection (I.I.) index"].
Changes of liver functions associated with endoscopic injection sclerotherapy (EIS) were investigated in 143 patients with remarkable esophageal varices. The index used for evaluating hepatic reserve was the ratio between the increase of total bilirubin and the increase of LDH after EIS [I.I. index = (delta T.Bil./delta LDH) x 100] in cases treated by intravariceal injection of 5% ethanolamine oleate. I.I. index value caused by hemolysis was stable and always below 0.2, while the elevation of I.I. index above 0.2 was regarded as the reflection of the deterioration of liver function. After the entire sessions of EIS, the changes of... (truncated)

Title: Sclerotherapy for simple cysts with use of ethanolamine oleate: preliminary experience.
We evaluated the efficacy of ethanolamine oleate (EO) as a sclerosing agent for a symptomatic hepatic or renal cyst. Seven patients with symptomatic hepatic (n = 3) or renal cysts (n = 4) were treated by sclerotherapy with EO. The cyst size in the greater diameter ranged from 6 to 13 cm. The cyst was punctured under ultrasound guidance, and after all of the cyst's content was aspirated, an iodized contrast agent was injected to check the absence of communication between the cyst and biliary tree, urinary tract, or vessels. Then, the solution of ethanolamine oleate-iopamidol mixture (EOI) of 10%... (truncated)

Title: KASL clinical practice guidelines for liver cirrhosis: Varices, hepatic encephalopathy, and related complications.
No abstract available.

Title: Friend or Foe? Spontaneous Portosystemic Shunts in Cirrhosis-Current Understanding and Future Prospects.
Portal hypertension (PHT) in cirrhosis results from increased resistance to splanchnic blood flow secondary to parenchymal and vascular changes within the liver. In an attempt to counteract the increased portal pressure, two mechanisms simultaneously occur: splanchnic vasodilatation and formation of spontaneous portosystemic shunts (SPSS). Long considered to be a compensatory mechanism to decompress the portal venous system, it is now well established that SPSS are not only inefficient in decreasing the portal pressure but also contribute to reduced hepatocyte perfusion and increased splanchnic blood flow and resistance, associated with worsening PHT. Recent studies have described a high prevalence of SPSS... (truncated)

MESH:D016567 - nizatidine

Summary:

LLM Prediction Score	0.138 (normalized)
LLM Confidence Score	0.960
Golden Answer (Severity Class)	0.625 (normalized)
Prediction Error	0.487

Retrieved Context:

Title: Effects of ranitidine and nizatidine on the risk of gastrointestinal cancer.
Label="Purpose" NlmCategory="UNASSIGNED">Gastrointestinal (GI) cancer occurs in digestive organs such as the stomach, colon, liver, esophagus, and pancreas. About 83,034 cases occurred in Korea alone in 2020. Dietary factors, alcohol consumption, <i>Helicobacter pylori</i> (<i>H. pylori</i>), and lifestyle factors increase the incidence of diseases such as gastritis, peptic ulcer, pancreatitis, and gastroesophageal reflux disease (GERD), which can develop into GI cancer. However, in 2019, the US Food and Drug Administration announced that the drugs ranitidine and nizatidine, which are used for digestive disorders, contain carcinogens. In this study, we investigated the effects of ranitidine and nizatidine on the development of GI cancer.

Title: Safety of nizatidine in clinical trials conducted in the USA and Europe.
The safety of nizatidine as treatment for active duodenal or gastric ulcer disease or as maintenance therapy following ulcer healing was assessed in 3800 nizatidine-treated individuals in clinical trials conducted in the USA and Europe. Safety parameters included physical examinations, electrocardiograms, eye examinations, serum chemistries and testosterone, hematology, and urinalyses. Adverse events were recorded without judgment of causality. Early discontinuations and adverse events, including complications of active duodenal ulcer disease, occurred more frequently in placebo-treated patients than in those given nizatidine. No differences were observed between the nizatidine and ranitidine treatment groups in regard to adverse event incidence or severity.... (truncated)

Title: A human liver cell-based system modeling a clinical prognostic liver signature for therapeutic discovery.
Chronic liver disease and hepatocellular carcinoma (HCC) are life-threatening diseases with limited treatment options. The lack of clinically relevant/tractable experimental models hampers therapeutic discovery. Here, we develop a simple and robust human liver cell-based system modeling a clinical prognostic liver signature (PLS) predicting long-term liver disease progression toward HCC. Using the PLS as a readout, followed by validation in nonalcoholic steatohepatitis/fibrosis/HCC animal models and patient-derived liver spheroids, we identify nizatidine, a histamine receptor H2 (HRH2) blocker, for treatment of advanced liver disease and HCC chemoprevention. Moreover, perturbation studies combined with single cell RNA-Seq analyses of patient liver tissues uncover hepatocytes and... (truncated)

Title: The safety of drugs used in acid-related disorders and functional gastrointestinal disorders.
Medicines are frequently used in the management of acid-related disorders and functional gastrointestinal disorders. With the exception of complicated peptic ulcer disease, these disorders are not associated with appreciable mortality. Drug treatments have consequently been held to the highest standards of safety. Some medicines have been withdrawn or restricted based on assessments and perceptions of risk. However, the risk of serious toxicity is low for most of the agents discussed in this article. Assessments are made of the safety and adverse-event profiles of certain drug classes and, where appropriate, individual medicines. For conditions with a low risk of mortality or... (truncated)

Title: Alcohol and medication interactions.
Many medications can interact with alcohol, thereby altering the metabolism or effects of alcohol and/or the medication. Some of these interactions can occur even at moderate drinking levels and result in adverse health effects for the drinker. Two types of alcohol-medication interactions exist: (1) pharmacokinetic interactions, in which alcohol interferes with the metabolism of the medication, and (2) pharmacodynamic interactions, in which alcohol enhances the effects of the medication, particularly in the central nervous system (e.g., sedation). Pharmacokinetic interactions generally occur in the liver, where both alcohol and many medications are metabolized, frequently by the same enzymes. Numerous classes of... (truncated)

MESH:D004319 - doxylamine

Summary:

LLM Prediction Score	0.487 (normalized)
LLM Confidence Score	0.980
Golden Answer (Severity Class)	0.0 (normalized)
Prediction Error	0.487

Retrieved Context:

Title: When Overdose of Doxylamine Leads to Severe Rhabdomyolysis and Renal Failure That Requires Hemodialysis: A Case Report and Literature Review.
A 52-year-old male with acute onset right-sided weakness, numbness, and buttock pain after consuming 30 tablets of doxylamine antihistamine the night prior. Laboratory tests showed elevated creatinine kinase, blood urea nitrogen, creatinine, troponins, liver transaminases, and phosphate. The patient was admitted to the medical intensive care unit for severe rhabdomyolysis, acute liver failure, and acute kidney injury secondary to doxylamine intoxication. Studies describe symptoms of severe doxylamine intoxication, such as impaired consciousness (coma), grand mal seizures, and cardiopulmonary arrest. Circulating myoglobin causes oxidative injury to the kidney through the formation of F2-isoprostanes leading to renal vasoconstriction. One study explained drug-induced... (truncated)

Title: [Hyperemesis gravidarum: a rare but potentially severe complication of the first trimester of pregnancy].
Although nausea and vomiting are common symptoms in early pregnancy, hyperemesis gravidarum (HG) is a rare complication of the first trimester of pregnancy. This condition is defined as intractable vomiting occurring before 20 weeks of gestation, with fluid and electrolyte disturbance, significant weight loss, and ketonuria, leading to hospitalization in the absence of other cause than pregnancy. Some biological disturbances found in HG, such as hyperthyroidism and hepatic cytolysis, which are correlated with the importance of vomiting, are without severe clinical consequences, but may represent diagnostic pitfalls. The aetiology is unknown, but human chorionic gonadotropin hormones likely play the first... (truncated)

Title: Jaundice Caused by Hyperemesis Gravidarum.
Background: Hyperemesis gravidarum is characterized by intractable vomiting and associated with weight loss exceeding 5% of prepregnancy body weight, dehydration, and ketosis. Hyperemesis gravidarum occurs during the first trimester and typically resolves by 16 to 20 weeks of gestation. Approximately half of all hospitalized females with hyperemesis gravidarum have a mild elevation in liver enzymes; however, jaundice and hepatic synthetic dysfunction are uncommon. Case Report: A 22-year-old gravida 1 para 0 in her ninth week with a singleton gestation was hospitalized with persistent nausea, vomiting, weight loss of 11% of her prepregnancy body weight, dehydration, hypokalemia, and jaundice. Liver function... (truncated)

Title: Gastrointestinal diseases during pregnancy: what does the gastroenterologist need to know?
Pregnancy is characterized by numerous physiological changes that may lead to a diversity of symptoms and frequently to gastrointestinal complaints, such as heartburn, nausea and vomiting, or constipation. Chronic gastrointestinal diseases require treatment maintenance during this period, raising the challenging question whether outcomes beneficial to the mother may be harmful for the fetus. In addition, certain diseases, such as acute fatty liver of pregnancy, only develop during pregnancy and may require urgent procedures, such as fetus delivery. Even though they are not present in our day-to-day practice, knowledge of pregnancy-related diseases is fundamental and collaboration between gastroenterologists and obstetricians is... (truncated)

Title: Nausea and vomiting of pregnancy.
Nausea and vomiting are common experiences in pregnancy, affecting 70% to 80% of all pregnant women. Various metabolic and neuromuscular factors have been implicated in the pathogenesis of nausea and vomiting of pregnancy (NVP) and hyperemesis gravidarum (HG), an entity distinct from NVP. However, their exact cause is unknown. Consequently, treatment of NVP and HG can be difficult, as neither the optimal targets for treatment nor the full effects of potential treatments on the developing fetus are known. This article reviews the epidemiology, pathology, diagnosis, outcomes, and treatment of NVP and HG.

MESH:C075773 - 4-phenylbutyric acid

Summary:

LLM Prediction Score	0.013 (normalized)
LLM Confidence Score	0.880
Golden Answer (Severity Class)	0.5 (normalized)
Prediction Error	0.487

Retrieved Context:

Title: 4-Phenylbutyric acid improves free fatty acid-induced hepatic insulin resistance in vivo.
Plasma free fatty acids (FFAs) are elevated in obesity and can induce insulin resistance via endoplasmic reticulum (ER) stress. However, it is unknown whether hepatic insulin resistance caused by the elevation of plasma FFAs is alleviated by chemical chaperones. Rats received one of the following i.v. treatments for 48 h: saline, intralipid plus heparin (IH), IH plus the chemical chaperone 4-phenylbutyric acid (PBA), or PBA alone and a hyperinsulinemic-euglycemic clamp was performed during the last 2 h. PBA co-infusion normalized IH-induced peripheral insulin resistance, similar to our previous findings with an antioxidant and an IκBα kinase β (IKKβ) inhibitor. Different... (truncated)

Title: Effects of 4-phenylbutyrate therapy in a preterm infant with cholestasis and liver fibrosis.
The bile salt export pump is expressed at the canalicular membrane of hepatocytes and mediates biliary excretion of bile salts. 4-Phenylbutyrate (4 PB), a drug used to treat ornithine transcarbamylase deficiency, has been found to increase the hepatocanalicular expression of bile salt export pump. The beneficial effects of 4-phenylbutyrate therapy have been reported for patients with progressive familial intrahepatic cholestasis, an inherited autosomal recessive liver disease. This is the first study to show the therapeutic effect of 4 PB in a preterm infant with cholestasis and liver fibrosis. The preterm infant had severe cholestasis with jaundice and failure to thrive... (truncated)

Title: 4-Phenylbutyrate protects against rifampin-induced liver injury via regulating MRP2 ubiquitination through inhibiting endoplasmic reticulum stress.
Rifampin (RFP), a first-line anti-tuberculosis drug, often induces cholestatic liver injury and hyperbilirubinemia which limits its clinical use. Multidrug resistance-associated protein 2 (MRP2) localizes to the hepatocyte apical membrane and plays a pivotal role in the biliary excretion of bilirubin glucuronides. RFP is discovered to reduce MRP2 expression in liver cells. 4-Phenylbutyrate (4-PBA), a drug used to treat ornithine transcarbamylase deficiency (DILI), is reported to alleviate RFP-induced liver cell injury. However, the underlying mechanism still remains unclear. In the current study, we discovered that RFP induced HepG2 cell viability reduction, apoptosis and MRP2 ubiquitination degradation. Administration of 4-PBA alleviated the... (truncated)

Title: Sodium 4-phenylbutyrate prevents murine dietary steatohepatitis caused by trans-fatty acid plus fructose.
Excess consumption of trans-fatty acid could increase the risk of non-alcoholic steatohepatitis (NASH); however, treatment targeting trans-fatty acid-induced NASH has not been examined. Here we focused on the influence of trans-fatty acid intake on endoplasmic reticulum (ER) stress in hepatocytes, so we investigated the effect of the chemical chaperone 4-phenylbutyric acid (PBA), on trans-fatty acid-caused steatohepatitis using diabetic KK-A(y) mice. Elaidic acid (EA, trans-fatty acid) alone did not cause definitive liver injury. In contrast, EA plus low-dose fructose induced extensive apoptosis in hepatocytes with severe fat accumulation. EA plus fructose significantly increased ER stress markers such as glucose-regulated protein 78... (truncated)

Title: 4-phenylbutyric acid attenuates endoplasmic reticulum stress-mediated apoptosis and protects the hepatocytes from intermittent hypoxia-induced injury.
To investigate the effect of 4-phenylbutyric acid (4-PBA) on intermittent hypoxia (IH)-induced liver cell injury and to clarify the underlying mechanisms.

MESH:D005279 - fenoprofen

Summary:

LLM Prediction Score	0.514 (normalized)
LLM Confidence Score	0.920
Golden Answer (Severity Class)	1.0 (normalized)
Prediction Error	0.486

Retrieved Context:

Title: [Hepatitis secondary to current non-steroidal anti-inflammatory agents].
The authors report 83 cases of acute hepatitis secondary to non steroid anti-inflammatory drugs (NSAID), published in the literature. The NSAID in question are: niflumic acid, tolfenamic acid, diclofenac, fenoprofen, ibuprofen, indomethacin, naproxen, piroxicam, pirprofen and sulindac. Six deaths are directly ascribed to NSAID: although rare, these forms of hepatitis deserve therefore to be reported. They are usually mixed immuno-allergic forms of hepatitis (cytolytic as well as cholestatic). They often affect elderly women taking multiple medications. Monitoring of the liver function tests is necessary, during prolonged treatment with NSAID, especially during the first six months.

Title: Risk factors of drug interaction between warfarin and nonsteroidal anti-inflammatory drugs in practical setting.
Nonsteroidal anti-inflammatory drugs (NSAIDs) are known to interact with the oral anticoagulant warfarin and can cause a serious bleeding complication. In this study, we evaluated the risk factors for international normalized ratio (INR) increase, which is a surrogate marker of bleeding, after addition of an NSAID in a total of 98 patients who used warfarin. Patient age, sex, body mass index, maintenance warfarin dose, baseline INR, coadministered medications, underlying diseases, and liver and kidney functions were evaluated for possible risk factors with INR increase > or =15.0% as the primary end-point. Of the 98 patients, 39 (39.8%) showed an INR... (truncated)

Title: Alcohol and medication interactions.
Many medications can interact with alcohol, thereby altering the metabolism or effects of alcohol and/or the medication. Some of these interactions can occur even at moderate drinking levels and result in adverse health effects for the drinker. Two types of alcohol-medication interactions exist: (1) pharmacokinetic interactions, in which alcohol interferes with the metabolism of the medication, and (2) pharmacodynamic interactions, in which alcohol enhances the effects of the medication, particularly in the central nervous system (e.g., sedation). Pharmacokinetic interactions generally occur in the liver, where both alcohol and many medications are metabolized, frequently by the same enzymes. Numerous classes of... (truncated)

Title: TJ-M2010-5, A self-developed MyD88 inhibitor, attenuates liver fibrosis by inhibiting the NF-κB pathway.
Liver fibrosis is the result of most chronic inflammatory liver damage and seriously endangers human health. However, no drugs have been approved to treat this disease. Previous studies showed that the Toll-like receptors (TLRs)/myeloid differentiation factor-88 (MyD88)/nuclear factor-κB (NF-κB) pathway plays a key role in liver fibrosis. TJ-M2010-5 is a self-developed small molecule MyD88 inhibitor, which has been proven to have a good protective effect in a variety of inflammatory disease models. In the present study, to investigate the anti-fibrotic effect of TJ-M2010-5, mice were injected with carbon tetrachloride (CCl₄) in vivo and LX2 cells (a human hepatic stellate cell... (truncated)

Title: Risk factors for idiosyncratic drug-induced liver injury.
Idiosyncratic drug-induced liver injury (DILI) is a rare disorder that is not related directly to dosage and little is known about individuals who are at increased risk. There are no suitable preclinical models for the study of idiosyncratic DILI and its pathogenesis is poorly understood. It is likely to arise from complex interactions among genetic, nongenetic host susceptibility, and environmental factors. Nongenetic risk factors include age, sex, and other diseases (eg, chronic liver disease or human immunodeficiency virus infection). Compound-specific risk factors include daily dose, metabolism characteristics, and propensity for drug interactions. Alcohol consumption has been proposed as a risk... (truncated)

MESH:D008528 - mefenamic acid

Summary:

LLM Prediction Score	0.514 (normalized)
LLM Confidence Score	0.980
Golden Answer (Severity Class)	1.0 (normalized)
Prediction Error	0.486

Retrieved Context:

Title: Characterization of the acyl-adenylate linked metabolite of mefenamic Acid.
Mefenamic acid, (MFA), a carboxylic acid-containing nonsteroidal anti-inflammatory drug (NSAID), is metabolized into the chemically reactive conjugates MFA-1-O-acyl-glucuronide (MFA-1-O-G) and MFA-S-acyl-CoA (MFA-CoA), which are both implicated in the formation of MFA-S-acyl-glutathione (MFA-GSH) conjugates, protein-adduct formation, and thus the potential toxicity of the drug. However, current studies suggest that an additional acyl-linked metabolite may be implicated in the formation of MFA-GSH. In the present study, we investigated the ability of MFA to become bioactivated into the acyl-linked metabolite, mefenamyl-adenylate (MFA-AMP). In vitro incubations in rat hepatocytes with MFA (100 μM), followed by LC-MS/MS analyses of extracts, led to the detection of... (truncated)

Title: Mefenamic Acid-Upregulated Nrf2/SQSTM1 Protects Hepatocytes against Oxidative Stress-Induced Cell Damage.
Mefenamic acid (MFA) is a commonly prescribed non-steroidal anti-inflammatory drug (NSAID) with anti-inflammatory and analgesic properties. MFA is known to have potent antioxidant properties and a neuroprotective effect against oxidative stress. However, its impact on the liver is unclear. This study aimed to elucidate the antioxidative effects of MFA and their underlying mechanisms. We observed that MFA treatment upregulated the nuclear factor erythroid 2-related factor 2 (Nrf2) pathway. Treatment with various anthranilic acid derivative-class NSAIDs, including MFA, increased the expression of sequestosome 1 (SQSTM1) in HepG2 cells. MFA disrupted the interaction between Kelch-like ECH-associated protein 1 (Keap1) and Nrf2, activating... (truncated)

Title: Cytochrome P450-mediated bioactivation of mefenamic acid to quinoneimine intermediates and inactivation by human glutathione S-transferases.
Mefenamic acid (MFA) has been associated with rare but severe cases of hepatotoxicity, nephrotoxicity, gastrointestinal toxicity, and hypersensitivity reactions that are believed to result from the formation of reactive metabolites. Although formation of protein-reactive acylating metabolites by phase II metabolism has been well-studied and proposed to be the cause of these toxic side effects, the oxidative bioactivation of MFA has not yet been competely characterized. In the present study, the oxidative bioactivation of MFA was studied using human liver microsomes (HLM) and recombinant human P450 enzymes. In addition to the major metabolite 3'-OH-methyl-MFA, resulting from the benzylic hydroxylation by CYP2C9,... (truncated)

Title: Idiosyncratic NSAID drug induced oxidative stress.
Many idiosyncratic non-steroidal anti-inflammatory drugs (NSAIDs) cause GI, liver and bone marrow toxicity in some patients which results in GI bleeding/ulceration/fulminant hepatic failure/hepatitis or agranulocytosis/aplastic anemia. The toxic mechanisms proposed have been reviewed. Evidence is presented showing that idiosyncratic NSAID drugs form prooxidant radicals when metabolised by peroxidases known to be present in these tissues. Thus GSH, NADH and/or ascorbate were cooxidised by catalytic amounts of NSAIDs and hydrogen peroxide in the presence of peroxidase. During GSH and NADH cooxidation, oxygen uptake and activation occurred. Furthermore the formation of NSAID oxidation products was prevented during the cooxidation indicating that the... (truncated)

Title: The problems and pitfalls of NSAID therapy in the elderly (Part I).
Nonsteroidal anti-inflammatory drugs (NSAIDs) are the most commonly prescribed drugs worldwide when grouped by generic categories and account for 3 to 9% of total prescription numbers in various countries. While NSAIDs are responsible for approximately 25% of all reported adverse drug reactions, aging may substantially increase the risk of NSAID-induced reactions. Several factors may contribute to NSAID-related toxicity in the elderly. The increase in morbidity associated with aging may result in consumption of a wide range of potent drugs, while inappropriate drug therapy and aberrant compliance are also capable of contributing to adverse drug reactions in geriatric patients.
Age-related alterations... (truncated)

MESH:D019782 - riluzole

Summary:

LLM Prediction Score	0.514 (normalized)
LLM Confidence Score	0.990
Golden Answer (Severity Class)	1.0 (normalized)
Prediction Error	0.486

Retrieved Context:

Title: The tolerability of riluzole in the treatment of patients with amyotrophic lateral sclerosis.
Riluzole is the only disease-modifying drug approved for the treatment of amyotrophic lateral sclerosis (ALS), in which it has been demonstrated to extend survival. The overall tolerability of riluzole is good and the drug can be used in all patients with ALS except those with elevated transaminase levels or active liver disease. The most frequently encountered adverse events (AEs) that appear to be attributed to riluzole are asthenia and nausea, observed in 18 and 15% of patients taking riluzole in the randomised clinical trial programme, respectively. These same AEs, albeit at a lower frequency, are also reported in Phase IV... (truncated)

Title: [Liver Injury Risk Factors in Amyotrophic Lateral Sclerosis Patients Treated with Riluzole].
Riluzole, a drug used in the management of amyotrophic lateral sclerosis (ALS), is associated with a high incidence of liver failure. It is imperative to determine risk factors and severity of liver injury in patients taking riluzole to devise an appropriate treatment regimen. We, therefore, studied risk factors for liver injury in ALS patients who were prescribed riluzole at Kitasato University East Hospital from 1999 to 2015. Of the 222 patients enrolled in this study, 113 and 109 patients were diagnosed with mild to moderate (grade 1 or 2) and without (grade 0) liver injury, respectively. Prediction of risk factors... (truncated)

Title: Assessing Effects of BHV-0223 40 mg Zydis Sublingual Formulation and Riluzole 50 mg Oral Tablet on Liver Function Test Parameters Utilizing DILIsym.
For patients with amyotrophic lateral sclerosis who take oral riluzole tablets, approximately 50% experience alanine transaminase (ALT) levels above upper limit of normal (ULN), 8% above 3× ULN, and 2% above 5× ULN. BHV-0223 is a novel 40 mg rapidly sublingually disintegrating (Zydis) formulation of riluzole, bioequivalent to conventional riluzole 50 mg oral tablets, that averts the need for swallowing tablets and mitigates first-pass hepatic metabolism, thereby potentially reducing risk of liver toxicity. DILIsym is a validated multiscale computational model that supports evaluation of liver toxicity risks. DILIsym was used to compare the hepatotoxicity potential of oral riluzole tablets (50... (truncated)

Title: Combined Treatment with Herbal Medicine and Drug Ameliorates Inflammation and Metabolic Abnormalities in the Liver of an Amyotrophic Lateral Sclerosis Mouse Model.
To date, no effective drugs exist for amyotrophic lateral sclerosis (ALS), although riluzole (RZ) and edaravone have been approved for treatment. We previously reported that Bojungikgi-tang (BJIGT) improved motor activity through anti-inflammatory effects in the muscle and spinal cord of hSOD1^{G93A} mice. Therefore, whether combined treatment with BJIGT and RZ synergistically affects liver function in hSOD1^{G93A} mice was investigated. Two-month-old male hSOD1^{G93A} mice were treated with BJIGT (1 mg/g) and RZ (8 μg/g) administered orally for 5 weeks. Drug metabolism and liver function tests of serum and liver homogenates were conducted. mRNA expression levels of cytochrome P450 (CYP) isozymes, inflammatory... (truncated)

Title: Riluzole: A neuroprotective drug with potential as a novel anti-cancer agent (Review).
Riluzole, a glutamate release inhibitor, has been in use for the treatment of amyotrophic lateral sclerosis for over two decades since its approval by the Food and Drug Administration. Recently, riluzole has been evaluated in cancer cells and indicated to block cell proliferation and/or induce cell death. Riluzole has been proven effective as an anti‑neoplastic drug in cancers of various tissue origins, including the skin, breast, pancreas, colon, liver, bone, brain, lung and nasopharynx. While cancer cells expressing glutamate receptors frequently respond to riluzole treatment, numerous types of cancer cell lacking glutamate receptors unexpectedly responded to riluzole treatment as well.... (truncated)

MESH:D000077545 - eplerenone

Summary:

LLM Prediction Score	0.015 (normalized)
LLM Confidence Score	0.970
Golden Answer (Severity Class)	0.5 (normalized)
Prediction Error	0.485

Retrieved Context:

Title: Reversible Fulminant Hepatitis Secondary to Cocaine in the Setting of β-Blocker Use.
<i>Background</i>. Fulminant hepatitis is acute hepatic injury with severe decline in hepatic function manifested by encephalopathy, hypercoagulable state, jaundice, renal failure, hypoglycemia, or a constellation of these symptoms in patients without preexisting liver disease. Etiologies include viral infections, hepatotoxic drugs, autoimmune diseases, vaso-occlusive diseases, sepsis, and malignant infiltration. <i>Case Report.</i> A 56-year-old man presented with acute heart failure in the setting of cocaine use. The patient subsequently developed fulminant hepatic failure manifested by acute hypoglycemia, elevated liver enzyme, and worsening liver function, which resolved over 1 week with supportive care. The patient was on β-blocker, which was stopped during the... (truncated)

Title: Safety and Antihypertensive Effect of Selara® (Eplerenone): Results from a Postmarketing Surveillance in Japan.
Prospective postmarketing surveillance of Selara (eplerenone), a selective mineralocorticoid receptor antagonist, was performed to confirm its safety and efficacy for hypertension treatment in Japan. The change in blood pressure after initiation of eplerenone treatment was also examined. Patients with essential hypertension who were eplerenone-naïve were recruited regardless of the use of other antihypertensive drugs. For examination of changes in blood pressure, patients were excluded if eplerenone was contraindicated or used off-label. Patients received 50-100 mg of eplerenone once daily and were observed for 12 weeks. No treatments including antihypertensive drugs were restricted during the surveillance period. Across Japan, 3,166 patients... (truncated)

Title: Eplerenone Reverses Cardiac Fibrosis via the Suppression of Tregs by Inhibition of Kv1.3 Channel.
Background: Fibroblast proliferation is a critical feature during heart failure development. Previous studies reported regulatory T-lymphocytes (Tregs)' protective role against myocardial fibrosis. However, notably, Tregs also secrete fibrogenic cytokine TGF-β when activated. This study aimed to clarify the intriguing link between Tregs and fibrosis, the role of Tregs Kv1.3 potassium channel (regulating T-lymphocytes activation) in the fibrosis process, and how selective aldosterone receptor antagonist Eplerenone affects Tregs and fibrosis through its action on Kv1.3 channel. Methods and Results: After co-incubation with Tregs, cardiac fibroblast proliferation (CCK-8 assay) and levels of collagen I, III, and Matrix metalloproteinase2 (ELISA) significantly elevated. Cell... (truncated)

Title: The effect of eplerenone on adenosine formation in humans in vivo: a double-blinded randomised controlled study.
It has been suggested that mineralocorticoid receptor antagonists have direct cardioprotective properties, because these drugs reduce mortality in patients with heart failure. In murine models of myocardial infarction, mineralocorticoid receptor antagonists reduce infarct size. Using gene deletion and pharmacological approaches, it has been shown that extracellular formation of the endogenous nucleoside adenosine is crucial for this protective effect. We now aim to translate this finding to humans, by investigating the effects of the selective mineralocorticoid receptor antagonist eplerenone on the vasodilator effect of the adenosine uptake inhibitor dipyridamole, which is a well-validated surrogate marker for extracellular adenosine formation.

Title: Phase 1 Studies to Define the Safety, Tolerability, and Pharmacokinetic and Pharmacodynamic Profiles of the Nonsteroidal Mineralocorticoid Receptor Antagonist Apararenone in Healthy Volunteers.
Apararenone is a long-acting, nonsteroidal mineralocorticoid receptor antagonist (MRA). The safety, tolerability, and pharmacokinetic (PK) and pharmacodynamic (PD) profiles of single- and multiple-dose apararenone were assessed in 3 phase 1 randomized, double-blind studies in 223 healthy adults. Study 1 assessed the PK, safety/tolerability, and PD of single-dose apararenone (3.75-640 mg) and multiple-dose apararenone (10-40 mg/day on days 1-14, 320 mg loading dose on day 1 + 10 mg/day on days 2-14, or 40-320 mg loading dose on day 1 + 2.5-20 mg/day on days 2-14) in Caucasian and Black men and women. Study 2 assessed the PK and safety of... (truncated)

MESH:D010431 - pentoxifylline

Summary:

LLM Prediction Score	0.140 (normalized)
LLM Confidence Score	0.990
Golden Answer (Severity Class)	0.625 (normalized)
Prediction Error	0.485

Retrieved Context:

Title: [Cholestatic hepatitis. Presumptive role of ticlopidine].
A seventy-one-year-old woman presented with jaundice (total bilirubinemia 91 microM, conjugated bilirubinemia 76 microM) and cytolysis (ALAT greater than 6 N) after ten days of pentoxifylline-ticlopidine combination therapy. Blood count was normal excepting transient anemia. Protein electrophoresis was normal. Jaundice resolved 10 days after both drugs had been discontinued. Viral serology (B, A, nonA-nonB, mononucleosis, cytomegalovirus, herpes simplex virus) was negative. Ultrasonography and cholecystography were normal. Responsibility of a drug is therefore likely and we are inclined to incriminate ticlopidine as two similar cases have previously been observed.

Title: Pentoxifylline in the management of metabolic syndrome and chronic hepatitis C.
Metabolic syndrome (MS) and chronic hepatitis C (CHC) are prevalent diseases with many serious and fatal outcomes. Many of these outcomes are attributed to increased level of TNF-α which causes insulin resistance (IR), liver damage, increased incidence and mortality of hepatorenal syndrome (HRS), liver fibrosis and nonalcoholic steatohepatitis (NASH). So, an approach that depends on reducing the TNF-α levels is considered a reasonable method to help treat these conditions. Putting together the available data in the previous literature about pentoxifylline (PTX) would highly suggest that this drug is perfect for managing these conditions. Through its inhibitory effect on the production... (truncated)

Title: Effects of Pentoxifylline on Non-Alcoholic Steatohepatitis: A Randomized, Double-Blind, Placebo-Controlled Trial in Iran.
Non-alcoholic steatohepatitis (NASH) is a progressive form of nonalcoholic fatty liver disease. Several studies suggest that pentoxifylline (PTX) can improve the disease outcome.

Title: Pentoxifylline decreases oxidized lipid products in nonalcoholic steatohepatitis: new evidence on the potential therapeutic mechanism.
Pentoxifylline (PTX) improved the histological features of nonalcoholic steatohepatitis (NASH) in a recent randomized placebo-controlled trial. However, the underlying mechanism responsible for the beneficial effects of PTX in NASH remains unidentified. A key role of lipid oxidation in the pathogenesis and progression of NASH has been established. PTX is known to decrease free-radical-mediated oxidative stress and inhibit lipid oxidation. The primary aim of this study was to evaluate the effects of PTX on levels of lipid oxidation products in patients with NASH. Levels of multiple structurally specific oxidized fatty acids including hydroxy-octadecadienoic acids (HODEs), oxo-octadecadienoic acids (oxoODEs), and hydroxy-eicosatetraenoic acids... (truncated)

Title: Pentoxifylline Protects the Rat Liver Against Fibrosis and Apoptosis Induced by Acute Administration of 3,4-Methylenedioxymethamphetamine (MDMA or Ecstasy).
3,4-Methylenedioxymethamphetamine (MDMA) is one of the most popular drugs of abuse in the world with hallucinogenic properties that has been shown to induce apoptosis in liver cells. The present study aimed to investigate the effects of pentoxifylline (PTX) on liver damage induced by acute administration of MDMA in Wistar rat.

MESH:C023754 - tizanidine

Summary:

LLM Prediction Score	0.521 (normalized)
LLM Confidence Score	0.990
Golden Answer (Severity Class)	1.0 (normalized)
Prediction Error	0.479

Retrieved Context:

Title: A case of tizanidine-induced hepatic injury.
The case history is presented of a woman who developed serious liver injury while taking 36 mg tizanidine daily. Other causes of hepatic injury were excluded. Symptoms resolved after discontinuation of tizanidine, and the liver enzyme levels were nearly normal 6 weeks after discontinuation of the drug. Rechallenge with 4 mg tizanidine caused a relapse. The temporal relationship between the symptoms and liver enzyme elevations, the absence of other potential causes, and the reaction to rechallenge, strongly implicate tizanidine as the cause of hepatic injury. As we are not aware of similar case histories, this seems to be the first... (truncated)

Title: Managing Multiple Myeloma in the Face of Drug-Induced Adverse Drug Reaction.
Drug-induced liver injury has been reported to cause up to 10% of adverse drug reactions in the United States. Risk factors for druginduced liver injury include female gender, older age, interacting medications and drugs that are metabolized by the liver. This case report describes a patient who was newly initiated on tizanidine, an alpha2 adrenergic agonist used for muscle spasm and musculoskeletal pain, and bortezomib, a proteasome inhibitor used for multiple myeloma. Both medications are metabolized by cytochrome P450 isoenzyme 1A2. The medications were suspected of causing acute hepatitis based on the timing of their initiation and evidence to suggest... (truncated)

Title: Spasticity and drug therapy.
An overview is presented of pathophysiology, classification and measurement of spasticity and of its treatment, especially with dantrolene and baclofen. In spasticity, the balance between excitatory and inhibitory neurotransmitters in the central nervous system is impaired by mechanisms that are for the greater part unknown. Spasticity includes various disorders of motor control, and classification is needed for a meaningful evaluation of antispastic therapy. Cerebral palsy is a specific disorder, sometimes also called spasticity. Measurement of spasticity is complicated and should include signs characteristic of spasticity and parameters for clinical improvement. Dantrolene and baclofen have established their place in the treatment... (truncated)

Title: [Extreme sinus bradycardia (30/min) with acute right heart failure under tizanidine (Sirdalud). Possible pharmacological interaction with rofecoxib (Vioxx)].
The case of a 59-year-old healthy woman is described, who developed an extreme sinus bradycardia (30/min) with chest pain and acute right heart failure associated with gastrointestinal symptoms and elevation of the liver enzymes while simultaneously taking tizanidine (Sirdalud), diclofenac (Voltaren), and rofecoxib (Vioxx). The symptomatology resolved promptly after stopping the medication.

Title: Coadministration of tizanidine and ciprofloxacin: a retrospective analysis of the WHO pharmacovigilance database.
Tizanidine, an alpha-adrenergic substance with antinociceptive and antihypertensive effects, is extensively metabolized via cytochrome P450 (CYP) 1A2. Therefore, coadministration with potent CYP1A2 inhibitors, such as ciprofloxacin, is contraindicated. However, both drugs are broadly utilized in various countries. Their concomitant use bears an inherent high risk for clinically significant symptoms, especially in multimorbid patients experiencing polypharmacy. This study aims to investigate the impact of coadministration of tizanidine and ciprofloxacin using real-world pharmacovigilance data and to raise awareness of this potentially underestimated safety issue.

MESH:D010868 - pimozide

Summary:

LLM Prediction Score	0.477 (normalized)
LLM Confidence Score	0.950
Golden Answer (Severity Class)	0.0 (normalized)
Prediction Error	0.477

Retrieved Context:

Title: Pimozide in chronic schizophrenic outpatients.
In a double blind placebo controlled clinical evaluation of maintenance therapy in chronic schizophrenic female outpatients, thiordazine in single daily doses not exceeding 375 mg./day for 6 months was shown to be effective maintenance treatment compared with PL, thereby establishing the sensitivity of the experiment. Pimozide was also shown to be effective in a single oral dose not exceeding 16 mg./day and comparable overall to the standard drug. The experimental design was based on the anticipated retrogression of PL treated subjects during the 6-month study period, which was reflected in 5 of 9 (56%) "treatment failures" in the PL group... (truncated)

Title: [Psychiatric drugs as risk factor in fatal heat stroke].
Two men aged 33 and 31 years suffered a fatal heat stroke on a warm summer day. One of them used pimozide and clomipramine, the other zuclopenthixol, dexetimide, droperidol, promethazine and propranolol as psychiatric medication. Both of them had a body temperature > 42.3 degrees C, without perspiring. At first only a comatose situation with practically normal laboratory values existed; this was rapidly followed by massive liver damage, disseminated intravascular coagulation, anaemia, thrombopenia and acute renal failure. In spite of adequate and rapid treatment these complications were fatal. Both patients used medication with an antidopaminergic and anticholinergic (side) effect. The... (truncated)

Title: The Importance of Patient-Specific Factors for Hepatic Drug Response and Toxicity.
Responses to drugs and pharmacological treatments differ considerably between individuals. Importantly, only 50%-75% of patients have been shown to react adequately to pharmacological interventions, whereas the others experience either a lack of efficacy or suffer from adverse events. The liver is of central importance in the metabolism of most drugs. Because of this exposed status, hepatotoxicity is amongst the most common adverse drug reactions and hepatic liabilities are the most prevalent reason for the termination of development programs of novel drug candidates. In recent years, more and more factors were unveiled that shape hepatic drug responses and thus underlie the... (truncated)

Title: Capitalizing on the autophagic response for treatment of liver disease caused by alpha-1-antitrypsin deficiency and other genetic diseases.
Alpha-1-antitrypsin deficiency (ATD) is one of the most common genetic causes of liver disease and is a prototype of liver diseases caused by the pathologic accumulation of aggregated mutant alpha-1-antitrypsin Z (ATZ) within liver cells. In the case of ATD-associated liver disease, the resulting "gain-of-function" toxicity can lead to serious clinical manifestations, including cirrhosis and hepatocellular carcinoma. Currently, the only definitive therapy for ATD-associated liver disease is liver transplantation, but recent efforts have demonstrated the exciting potential for novel therapies that target disposal of the mutant protein aggregates by harnessing a cellular homeostasis mechanism called autophagy. In this review, we... (truncated)

Title: Novel Score-based Decision Approach in Chronic Myeloid Leukemia Patients After Acute Toxic Imatinib-induced Liver Injury.
The tyrosine kinase inhibitor (TKI) imatinib in rare cases can cause acute toxic hepatitis, hepatic failure, and death. Currently, the choice of further chronic myeloid leukemia (CML) therapy in patients after acute hepatotoxicity is still a difficult question, which requires a complex individual approach based on the clinical guidelines of adverse event management. Data about the further follow-up strategy approach in patients with CML after acute toxic imatinib-induced liver injury are of concern, and at times controversial. In addition, one of the questions is about the necessity and safety of the imatinib therapy resumption after acute hepatotoxicity. In some publications, imatinib... (truncated)

MESH:C104457 - nelarabine

Summary:

LLM Prediction Score	0.023 (normalized)
LLM Confidence Score	0.970
Golden Answer (Severity Class)	0.5 (normalized)
Prediction Error	0.477

Retrieved Context:

Title: Neurotoxicity Associated with Treatment of Acute Lymphoblastic Leukemia Chemotherapy and Immunotherapy.
Immunotherapy is a milestone in the treatment of poor-prognosis pediatric acute lymphoblastic leukemia (ALL) and is expected to improve treatment outcomes and reduce doses of conventional chemotherapy without compromising the effectiveness of the therapy. However, both chemotherapy and immunotherapy cause side effects, including neurological ones. Acute neurological complications occur in 3.6-11% of children treated for ALL. The most neurotoxical chemotherapeutics are L-asparaginase (L-ASP), methotrexate (MTX), vincristine (VCR), and nelarabine (Ara-G). Neurotoxicity associated with methotrexate (MTX-NT) occurs in 3-7% of children treated for ALL and is characterized by seizures, stroke-like symptoms, speech disturbances, and encephalopathy. Recent studies indicate that specific polymorphisms... (truncated)

Title: Relapsed/Refractory ETP-ALL Successfully Treated With Venetoclax and Nelarabine as a Bridge to Allogeneic Stem Cell Transplant.
No abstract available.

Title: Novel therapies for relapsed acute lymphoblastic leukemia.
The outcome of salvage therapy for relapsed acute lymphoblastic leukemia (ALL) remains poor. Salvage therapy mimics regimens with activity in newly diagnosed ALL. Novel strategies under investigation as monotherapy or in combination with chemotherapy improve the treatment of relapsed disease. For some ALL subsets, specific therapies are indicated. The addition of targeted therapy in Philadelphia chromo some-positive ALL has improved responses in relapsed patients without resistance to available tyrosine kinase inhibitors. Nelarabine demonstrates activity as monotherapy in T-cell ALL and is approved by the US Food and Drug Administration. Clofarabine, a second-generation purine analogue approved in pediatric leukemia, has shown... (truncated)

Title: Clinical Utility of Pegaspargase in Children, Adolescents and Young Adult Patients with Acute Lymphoblastic Leukemia: A Review.
Acute lymphoblastic leukemia (ALL) is a heterogenous hematological malignancy representing 25% of all cancers in children less than 15 years of age. Significant improvements in survival and cure rates have been made over the past four decades in pediatric ALL treatment. Asparaginases, derived from <i>Escherichia coli</i> and <i>Erwinia chrysanthemi</i>, have become a critical component of ALL therapy since the 1960s. Asparaginases cause depletion of serum asparagine, leading to deprivation of this critical amino acid for protein synthesis, and hence limit survival of lymphoblasts. Pegaspargase, a conjugate of monomethoxypolyethylene glycol (mPEG) and L-asparaginase, has become an integral component of pediatric upfront... (truncated)

Title: Adverse Reactions to Drugs of Special Interest in a Pediatric Oncohematology Service.
Introduction: Drugs used in oncological diseases are frequently related to adverse drug reactions (ADR). Few studies have analyzed the toxicity of cancer treatments in children in real practice. Methods: An observational, longitudinal and prospective study has been carried out in an Oncohematology Service of a tertiary hospital. During 2017, patients exposed to one or more drugs of a previously agreed list were identified and followed-up for at least 6 months each. Characteristics of ADR, incidence, causality and possible preventability, have been evaluated. Results: 72 patients have been treated with at least one study drug, and 159 ADR episodes involving at least... (truncated)

MESH:D000078304 - tigecycline

Summary:

LLM Prediction Score	0.523 (normalized)
LLM Confidence Score	0.990
Golden Answer (Severity Class)	1.0 (normalized)
Prediction Error	0.477

Retrieved Context:

Title: Influence of Antibiotics on Functionality and Viability of Liver Cells In Vitro.
(1) Antibiotics are an important weapon in the fight against serious bacterial infections and are considered a common cause of drug-induced liver injury (DILI). The hepatotoxicity of many drugs, including antibiotics, is poorly analyzed in human in vitro models. (2) A standardized assay with a human hepatoma cell line was used to test the hepatotoxicity of various concentrations (Cmax, 5× Cmax, and 10× Cmax) of antibiotics. In an ICU, the most frequently prescribed antibiotics, ampicillin, cefepime, cefuroxime, levofloxacin, linezolid, meropenem, rifampicin, tigecycline, and vancomycin, were incubated with HepG2/C3A cells for 6 days. Cell viability (XTT assay, LDH release, and vitality),... (truncated)

Title: Risk Factors for Tigecycline-Associated Hepatotoxicity in Patients in the Intensive Care Units of 2 Tertiary Hospitals: A Retrospective Study.
Tigecycline is a broad-spectrum antibacterial agent. As the incidence of multidrug-resistant bacterial infections has increased in intensive care units (ICUs) over the past decades, tigecycline is often used in ICUs. Information about tigecycline-associated hepatotoxicity in ICU patients is limited. To investigate the potential risk factors for tigecycline-associated hepatotoxicity in ICU patients, 148 patients from 2 centers who had received tigecycline for at least 4 days were retrospectively analyzed. Hepatotoxicity was classified according to the National Cancer Institute Common Terminology Criteria for Adverse Events (5.0) grading system. As a result, 33.8% of patients experienced hepatotoxicity events in the ICU. The multivariate... (truncated)

Title: Real-World Data of Tigecycline-Associated Drug-Induced Liver Injury Among Patients in China: A 3-year Retrospective Study as Assessed by the Updated RUCAM.
Background: Tigecycline, a glycylcycline antibiotic, is increasingly used clinically for the treatment of severe infections caused by multidrug-resistant bacteria, but it is also associated with hepatotoxicity. However, the incidence and risk factors of tigecycline-associated drug-induced liver injury (DILI) are unclear. We conducted this study to investigate the incidence, characteristics and risk factors of tigecycline-associated DILI in the real-world clinic setting. Patients and Methods: A retrospective analysis was conducted in inpatients who received tigecycline treatment from January 2018 to January 2020. Based on the biochemical criteria of DILI and the causality assessment by Roussel Uclaf Causality Assessment Method (RUCAM) using cases... (truncated)

Title: Tigecycline Tango: A Case of Antibiotic-Induced Pancreatitis.
Acute pancreatitis is a frequent cause of hospitalization, with the most common triggers being alcohol consumption and gallstones. Although the incidence of drug-induced pancreatitis remains low, it is steadily increasing due to the advent of newly discovered broad-spectrum antibiotics targeting multi-drug resistant organisms. Tigecycline, a broad-spectrum intravenous antibiotic derived from the tetracycline class, was approved by the FDA in 2005 for the treatment of complicated skin and skin structure infections, complicated intra-abdominal infections, and community-acquired pneumonia. It has activity against vancomycin-resistant Enterococcus, Methicillin-resistant <i>Staphylococcus aureus</i>, multi-drug-resistant <i>Acinetobacter baumannii</i>, multi-drug-resistant <i>Stenotrophomonas maltophilia</i>, and Extended Spectrum Beta-lactamase (ESBL) producing Enterobacter species. However, it was later... (truncated)

Title: A case report of drug-induced liver injury after tigecycline administration: histopathological evidence and a probable causality grading as assessed by the updated RUCAM diagnostic scale.
There have been no reports of tigecycline-associated drug-related liver injury (DILI) identified by histopathological assistance and causal assessment method. We reported the histopathological manifestations for the first time and described tigecycline-associated liver injury's pattern, severity, duration, and outcome.

MESH:D017291 - clarithromycin

Summary:

LLM Prediction Score	0.523 (normalized)
LLM Confidence Score	0.990
Golden Answer (Severity Class)	1.0 (normalized)
Prediction Error	0.477

Retrieved Context:

Title: Pyrimethamine-clarithromycin combination for therapy of acute Toxoplasma encephalitis in patients with AIDS.
Clarithromycin, a new macrolide, is effective in treating experimental Toxoplasma gondii infection. A pyrimethamine-clarithromycin combination was evaluated for the treatment of acute Toxoplasma encephalitis in 13 AIDS patients. The scheduled regimen was 2 g of clarithromycin per day and 75 mg of pyrimethamine per day for 6 weeks. The protocol was completed in eight patients and stopped in five patients (because of voluntary withdrawal by two patients, deterioration of neurological condition and thrombocytopenia in two patients, and suspicion of liver toxicity in one patient). The clinical and computed tomography scan responses at week 6 of treatment were 80 and 50%,... (truncated)

Title: Fatal fulminant hepatitis following administration of clarithromycin in a patient chronically treated with antipsycotic drugs.
Clarithromycin is a widely used antibiotic, especially prescribed for the treatment of respiratory tract infections, The drug is generally well tolerated. It is described as a very rare cause of fulminant liver failure.

Title: Risk of acute liver injury associated with the use of moxifloxacin and other oral antimicrobials: a retrospective, population-based cohort study.
To estimate the incidence and relative risk of a hospitalization or emergency visit for noninfectious liver injury in users of eight oral antimicrobials-amoxicillin, amoxicillin-clavulanic acid, clarithromycin, cefuroxime, doxycycline, levofloxacin, moxifloxacin, telithromycin-compared with nonusers of these antimicrobials.

Title: Clarithromycin-induced acute liver injury in a patient with positive <i>Helicobacter pylori</i>: a case report and review of the literature.
Label="Introduction and importance" NlmCategory="UNASSIGNED">While 14-day triple therapy with clarithromycin is a common approach for eradicating <i>Helicobacter pylori</i> infection, it is essential to note that this treatment does not come without potential side effects.

Title: Hepatic safety of antibiotics used in primary care.
Antibiotics used by general practitioners frequently appear in adverse-event reports of drug-induced hepatotoxicity. Most cases are idiosyncratic (the adverse reaction cannot be predicted from the drug's pharmacological profile or from pre-clinical toxicology tests) and occur via an immunological reaction or in response to the presence of hepatotoxic metabolites. With the exception of trovafloxacin and telithromycin (now severely restricted), hepatotoxicity crude incidence remains globally low but variable. Thus, amoxicillin/clavulanate and co-trimoxazole, as well as flucloxacillin, cause hepatotoxic reactions at rates that make them visible in general practice (cases are often isolated, may have a delayed onset, sometimes appear only after cessation... (truncated)

MESH:D000077924 - palonosetron

Summary:

LLM Prediction Score	0.024 (normalized)
LLM Confidence Score	0.990
Golden Answer (Severity Class)	0.5 (normalized)
Prediction Error	0.476

Retrieved Context:

Title: Palonosetron versus older 5-HT3 receptor antagonists for nausea prevention in patients receiving chemotherapy: a multistudy analysis.
No clinical standard currently exists for the optimal management of nausea induced by emetogenic chemotherapy, 7particularly delayed nausea.

Title: The effect of transdermal scopolamine for the prevention of postoperative nausea and vomiting.
Postoperative nausea and vomiting (PONV) is one of the most common and undesirable complaints recorded in as many as 70-80% of high-risk surgical patients. The current prophylactic therapy recommendations for PONV management stated in the Society of Ambulatory Anesthesia (SAMBA) guidelines should start with monotherapy and patients at moderate to high risk, a combination of antiemetic medication should be considered. Consequently, if rescue medication is required, the antiemetic drug chosen should be from a different therapeutic class and administration mode than the drug used for prophylaxis. The guidelines restrict the use of dexamethasone, transdermal scopolamine, aprepitant, and palonosetron as rescue... (truncated)

Title: Practical Guidance for the Management of Adverse Events in Patients with KRASG12C-Mutated Non-Small Cell Lung Cancer Receiving Adagrasib.
Adagrasib (MRTX849) is a KRASG12C inhibitor with favorable properties, including long half-life (23 h), dose-dependent pharmacokinetics, and central nervous system (CNS) penetration. As of September 1, 2022, a total of 853 patients with KRASG12C-mutated solid tumors, including patients with CNS metastases, had received adagrasib (monotherapy or in combination). Adagrasib-related treatment-related adverse events (TRAEs) are generally mild to moderate in severity, start early in treatment, resolve quickly with appropriate intervention, and result in a low rate of treatment discontinuation. Common TRAEs seen in clinical trials included gastrointestinal-related toxicities (diarrhea, nausea, and vomiting); hepatic toxicities (increased alanine aminotransferase/aspartate aminotransferase) and fatigue, which... (truncated)

Title: Phase II study of dacarbazine given with modern prophylactic anti-emetics and growth factor support to patients with metastatic, resistant soft tissue, and bone sarcoma.
Historically, administration of dacarbazine to sarcoma patients was limited by frequent treat-ment-related nausea/vomiting and neutropenia. These toxicities are now largely preventable with contemporary antiemetics and growth factor support. In this single-arm, phase II study, dacarbazine 850 mg/m² was given on day 1 of each 3-week cycle until disease progression or intolerance with prophylactic serotonin-3 receptor, neurokinin-1 antagonists, corticosteroids, and pegfilgrastim. Coprimary endpoints included clinical benefit rate (CBR), and any grade of nausea/vomiting and/or grade 3-4 neutropenia. With a sample size of 80 patients, >24 patients with clinical benefit would indicate that the CBR exceeds the historical (<20%) [Power 0.80; alpha... (truncated)

Title: Toxicity profile of antibody-drug conjugates in breast cancer: practical considerations.
Antibody-drug conjugates (ADCs) represent a novel and evolving class of antineoplastic agents, constituted by monoclonal antibody linked to biologically active drugs, delivering cytotoxic compounds at the tumor site, reducing the likelihood of systemic exposure and toxicity. They are generally well tolerated, nevertheless some predictable adverse reactions need careful monitoring and timely approach. These include neutropenia, nausea and vomiting, alopecia, diarrhea, left ventricular dysfunction, ILD/pneumonitis. The mechanisms leading to drug-associated toxicities are summarized, and prophylaxis protocols and appropriate management strategies are proposed, based on current literature. This review aims to collect the most updated evidence on toxicities potentially occurring during breast... (truncated)

MESH:D000077602 - tolvaptan

Summary:

LLM Prediction Score	0.524 (normalized)
LLM Confidence Score	0.980
Golden Answer (Severity Class)	1.0 (normalized)
Prediction Error	0.476

Retrieved Context:

Title: Tolvaptan-induced Liver Injury: Who is at Risk? A Case Report and Literature Review.
Hyponatremia is a common clinical condition encountered in the hospital setting. Syndrome of inappropriate antidiuretic hormone (SIADH) is an important and one of the most common causes of hyponatremia. SIADH accounts for approximately one-third of all cases of hyponatremia. Tolvaptan is a vasopressin receptor antagonist used to treat SIADH. Hepatoxicity is a rare yet dangerous side effect from Tolvaptan use. We present a case of cholestatic liver injury in an elderly female who presented with hyponatremia. She received two doses of tolvaptan 15mg and developed worsening in her total bilirubin (T Bili) and alkaline phosphatase (Alk Phos) levels. Tolvaptan is... (truncated)

Title: A cross-sectional survey of hospitalization and blood tests implementation status in patients who received tolvaptan under 75 years of age using a Japanese claims database.
Hypernatremia and liver injury are typical adverse effects of tolvaptan. Therefore, hospitalization and frequent monitoring of serum sodium concentration and liver function are necessary for tolvaptan initiation. We performed a cross-sectional survey to evaluate these situations.

Title: Application of a Mechanistic Model to Evaluate Putative Mechanisms of Tolvaptan Drug-Induced Liver Injury and Identify Patient Susceptibility Factors.
Tolvaptan is a selective vasopressin V2 receptor antagonist, approved in several countries for the treatment of hyponatremia and autosomal dominant polycystic kidney disease (ADPKD). No liver injury has been observed with tolvaptan treatment in healthy subjects and in non-ADPKD indications, but ADPKD clinical trials showed evidence of drug-induced liver injury (DILI). Although all DILI events resolved, additional monitoring in tolvaptan-treated ADPKD patients is required. In vitro assays identified alterations in bile acid disposition and inhibition of mitochondrial respiration as potential mechanisms underlying tolvaptan hepatotoxicity. This report details the application of DILIsym software to determine whether these mechanisms could account for... (truncated)

Title: Safety Profile of Tolvaptan in the Treatment of Autosomal Dominant Polycystic Kidney Disease.
Autosomal dominant polycystic kidney disease constitutes the most prevalent hereditary kidney disease, associated with high rates of morbidity leading eventually to end-stage renal disease. Tolvaptan is a selective vasopressin antagonist and has emerged as a promising therapeutic option for patients with autosomal dominant polycystic kidney disease. The present review summarized current evidence regarding the safety profile of tolvaptan in patients with the disease. Consistent with its pharmacological action, aquaretic adverse events represent the most common side effects of tolvaptan, consisting of polyuria, pollakiuria and polydipsia. Gradual dose titration based on urinary osmolality, as well as dietary interventions aiming to reduce... (truncated)

Title: Shedding Light on Drug-Induced Liver Injury: Activation of T Cells From Drug Naive Human Donors With Tolvaptan and a Hydroxybutyric Acid Metabolite.
Exposure to tolvaptan is associated with a significant risk of liver injury in a small fraction of patients with autosomal dominant polycystic kidney disease. The observed delayed onset of liver injury of between 3 and 18 months after commencing tolvaptan treatment, along with rapid recurrence of symptoms following re-challenge is indicative of an adaptive immune attack. This study set out to assess the intrinsic immunogenicity of tolvaptan and pathways of drug-specific T-cell activation using in vitro cell culture platforms. Tolvaptan (n = 7), as well as oxybutyric (DM-4103, n = 1) and hydroxybutyric acid (DM-4107, n = 18) metabolite-specific T-cell... (truncated)

MESH:D000241 - adenosine

Summary:

LLM Prediction Score	0.476 (normalized)
LLM Confidence Score	0.990
Golden Answer (Severity Class)	0.0 (normalized)
Prediction Error	0.476

Retrieved Context:

Title: Adenosine: tipping the balance towards hepatic steatosis and fibrosis.
Fatty liver is commonly associated with alcohol ingestion and abuse. While the molecular pathogenesis of these fatty changes is well understood, the histochemical and pharmacological mechanisms by which ethanol stimulates these molecular changes remain unknown. During ethanol metabolism, adenosine is generated by the enzyme ecto-5'-nucleotidase, and adenosine production and adenosine receptor activation are known to play critical roles in the development of hepatic fibrosis. We therefore investigated whether adenosine and its receptors play a role in the development of alcohol-induced fatty liver. WT mice fed ethanol on the Lieber-DeCarli diet developed hepatic steatosis, including increased hepatic triglyceride content, while mice... (truncated)

Title: Altered responsiveness to extracellular ATP enhances acetaminophen hepatotoxicity.
Adenosine triphosphate (ATP) is secreted from hepatocytes under physiological conditions and plays an important role in liver biology through the activation of P2 receptors. Conversely, higher extracellular ATP concentrations, as observed during necrosis, trigger inflammatory responses that contribute to the progression of liver injury. Impaired calcium (Ca^{2+}) homeostasis is a hallmark of acetaminophen (APAP)-induced hepatotoxicity, and since ATP induces mobilization of the intracellular Ca^{2+} stocks, we evaluated if the release of ATP during APAP-induced necrosis could directly contribute to hepatocyte death.

Title: Ischemia/Reperfusion Injury of Fatty Liver Is Protected by A2AR and Exacerbated by A1R Stimulation through Opposite Effects on ASK1 Activation.
Hepatic ischemia/reperfusion injury (IRI) is aggravated by steatosis and is a main risk factor in fatty liver transplantation. Adenosine receptors (ARs) are emerging as therapeutic targets in liver diseases. By using cellular and in vivo systems of hepatic steatosis and IRI, here we evaluated the effects of pharmacological A2AR and A1R activation. The A2AR agonist CGS21680 protected the primary steatotic murine hepatocyte from IR damage and the activation of ASK1 and JNK. Such an effect was attributed to a phosphatidylinositol-3-kinase (PI3K)/Akt-dependent inhibition of ASK1. By contrast, the A1R agonist CCPA enhanced IR damage, intracellular steatosis and oxidative species (OS) production,... (truncated)

Title: Adenosine inhibits chemotaxis and induces hepatocyte-specific genes in bone marrow mesenchymal stem cells.
Bone marrow-derived mesenchymal stem cells (MSCs) have therapeutic potential in liver injury, but the signals responsible for MSC localization to sites of injury and initiation of differentiation are not known. Adenosine concentration is increased at sites of cellular injury and inflammation, and adenosine is known to signal a variety of cellular changes. We hypothesized that local elevations in the concentration of adenosine at sites of tissue injury regulate MSC homing and differentiation. Here we demonstrate that adenosine does not induce MSC chemotaxis but dramatically inhibits MSC chemotaxis in response to the chemoattractant hepatocyte growth factor (HGF). Inhibition of HGF-induced chemotaxis... (truncated)

Title: Caffeine in liver diseases: Pharmacology and toxicology.
We have previously shown that adenosine A1AR antagonists, adenosine A2aAR antagonists, and caffeine have significant inhibitory effects on the activation and proliferation of hepatic stellate cells in alcoholic liver fibrosis. Many recent studies have found that moderate coffee consumption is beneficial for various liver diseases. The main active ingredient of coffee is caffeine, which is a natural non-selective adenosine receptor antagonist. Moreover, numerous preclinical epidemiological studies and clinical trials have examined the association between frequent coffee consumption and the risk of developing different liver diseases. In this review, we summarize and analyze the prophylactic and therapeutic effects of caffeine on... (truncated)

MESH:D000078262 - rifaximin

Summary:

LLM Prediction Score	0.399 (normalized)
LLM Confidence Score	0.980
Golden Answer (Severity Class)	0.875 (normalized)
Prediction Error	0.476

Retrieved Context:

Title: Chronic exposure to rifaximin causes hepatic steatosis in pregnane X receptor-humanized mice.
Rifaximin, a nonsystemic antibiotic that exhibits low gastrointestinal absorption, is a potent agonist of human pregnane X receptor (PXR), which contributes to its therapeutic efficacy in inflammatory bowel disease. To investigate the effects of long-term administration of rifaximin on the liver, PXR-humanized mice were administered rifaximin for 6 months; wild-type and Pxr-null mice were treated in parallel as controls. Histological analysis revealed time-dependent intense hepatocellular fatty degeneration and increased hepatic triglycerides in PXR-humanized mice and not in wild-type and Pxr-null mice. After long-term treatment, PXR target genes were induced in small intestine and liver, with significant up-regulation in the expression... (truncated)

Title: Rifaximin-α for liver fibrosis in patients with alcohol-related liver disease (GALA-RIF): a randomised, double-blind, placebo-controlled, phase 2 trial.
Alcohol is the leading cause of liver-related mortality worldwide. The gut-liver axis is considered a key driver in alcohol-related liver disease. Rifaximin-α improves gut-barrier function and reduces systemic inflammation in patients with cirrhosis. We aimed to compare the efficacy and safety of rifaximin-α with placebo in patients with alcohol-related liver disease.

Title: Rifaximin Prevents T-Lymphocytes and Macrophages Infiltration in Cerebellum and Restores Motor Incoordination in Rats with Mild Liver Damage.
In patients with liver cirrhosis, minimal hepatic encephalopathy (MHE) is triggered by a shift in peripheral inflammation, promoting lymphocyte infiltration into the brain. Rifaximin improves neurological function in MHE by normalizing peripheral inflammation. Patients who died with steatohepatitis showed T-lymphocyte infiltration and neuroinflammation in the cerebellum, suggesting that MHE may already occur in these patients. The aims of this work were to assess, in a rat model of mild liver damage similar to steatohepatitis, whether: (1) the rats show impaired motor coordination in the early phases of liver damage; (2) this is associated with changes in the immune system and... (truncated)

Title: Rifaximin Ameliorates Non-alcoholic Steatohepatitis in Mice Through Regulating gut Microbiome-Related Bile Acids.
Non-alcoholic steatohepatitis (NASH) is the progressive stage of non-alcoholic fatty liver disease (NAFLD). The non-absorbable antibiotic rifaximin has been used for treatment of irritable bowel syndrome, traveling diarrhea, and hepatic encephalopathy, but the efficacy of rifaximin in NASH patients remains controversial. This study investigated the effects and underlying mechanisms of rifaximin treatment in mice with methionine and choline deficient (MCD) diet-induced NASH. We found that rifaximin greatly ameliorated hepatic steatosis, lobular inflammation, and fibrogenesis in MCD-fed mice. Bacterial 16S rRNA sequencing revealed that the gut microbiome was significantly altered in MCD-fed mice. Rifaximin treatment enriched 13 amplicon sequence variants (ASVs)... (truncated)

Title: Rifaximin Alfa and Liver Diseases: More Than a Treatment for Encephalopathy, a Disease Modifier.
RFX, a rifamycin-based antibacterial agent obtained by the culture of the actinomycete Streptomyces mediterranei, has a broad antibacterial spectrum covering gram- positive, gram-negative, aerobic, and anaerobic bacteria. RFX is an antibiotic that elicits its effect by inhibiting bacterial RNA synthesis. When administered orally, its intestinal absorption is extremely low (<0.4%), restricting antibacterial activity mainly in the intestinal tract, with few systemic side effects. RFX has been recommended by the American Association for the Study of Liver Diseases and the European Association for the Study of the Liver guidelines for the treatment of HE. RFX may contribute to restore hepatic function... (truncated)

MESH:D000077432 - tapentadol

Summary:

LLM Prediction Score	0.473 (normalized)
LLM Confidence Score	0.980
Golden Answer (Severity Class)	0.0 (normalized)
Prediction Error	0.473

Retrieved Context:

Title: Acute administration of tramadol and tapentadol at effective analgesic and maximum tolerated doses causes hepato- and nephrotoxic effects in Wistar rats.
Tramadol and tapentadol are two atypical synthetic opioid analgesics, with monoamine reuptake inhibition properties. Mainly aimed at the treatment of moderate to severe pain, these drugs are extensively prescribed for multiple clinical applications. Along with the increase in their use, there has been an increment in their abuse, and consequently in the reported number of adverse reactions and intoxications. However, little is known about their mechanisms of toxicity. In this study, we have analyzed the in vivo toxicological effects in liver and kidney resulting from an acute exposure of a rodent animal model to both opioids. Male Wistar rats were... (truncated)

Title: Repeated Administration of Clinical Doses of Tramadol and Tapentadol Causes Hepato- and Nephrotoxic Effects in Wistar Rats.
Tramadol and tapentadol are fully synthetic and extensively used analgesic opioids, presenting enhanced therapeutic and safety profiles as compared with their peers. However, reports of adverse reactions, intoxications and fatalities have been increasing. Information regarding the molecular, biochemical, and histological alterations underlying their toxicological potential is missing, particularly for tapentadol, owing to its more recent market authorization. Considering the paramount importance of liver and kidney for the metabolism and excretion of both opioids, these organs are especially susceptible to toxicological damage. In the present study, we aimed to characterize the putative hepatic and renal deleterious effects of repeated exposure to... (truncated)

Title: Safe Use of Opioids in Chronic Kidney Disease and Hemodialysis Patients: Tips and Tricks for Non-Pain Specialists.
In patients suffering from moderate-to-severe chronic kidney disease (CKD) or end-stage renal disease (ESRD), subjected to hemodialysis (HD), pain is very common, but often underestimated. Opioids are still the mainstay of severe chronic pain management; however, their prescription in CKD and HD patients is still significantly low and pain is often under-treated. Altered pharmacokinetics and the lack of clinical trials on the use of opioids in patients with renal impairment increase physicians' concerns in this specific population. This narrative review focused on the correct and safe use of opioids in patients with CKD and HD. Morphine and codeine are not... (truncated)

Title: Pharmacologic Therapy for Acute Pain.
Pharmacologic management of acute pain should be tailored for each patient, including a review of treatment expectations and a plan for the time course of prescriptions. Acetaminophen and nonsteroidal anti-inflammatory drugs (NSAIDs) are first-line treatment options for most patients with acute mild to moderate pain. Topical NSAIDs are recommended for non-low back, musculoskeletal injuries. Acetaminophen is well tolerated; however, lower doses should be used in patients with advanced hepatic disease, malnutrition, or severe alcohol use disorder. Nonselective NSAIDs are effective but should be used with caution in patients with a history of gastrointestinal bleeding, cardiovascular disease, or chronic renal disease.... (truncated)

Title: IMI2-PainCare-BioPain-RCT1: study protocol for a randomized, double-blind, placebo-controlled, crossover, multi-center trial in healthy subjects to investigate the effects of lacosamide, pregabalin, and tapentadol on biomarkers of pain processing observed by peripheral nerve excitability testing (NET).
Few new drugs have been developed for chronic pain. Drug development is challenged by uncertainty about whether the drug engages the human target sufficiently to have a meaningful pharmacodynamic effect. IMI2-PainCare-BioPain-RCT1 is one of four similarly designed studies that aim to link different functional biomarkers of drug effects on the nociceptive system that could serve to accelerate the future development of analgesics. This study focusses on biomarkers derived from nerve excitability testing (NET) using threshold tracking of the peripheral nervous system.

MESH:C052932 - exifone

Summary:

LLM Prediction Score	0.527 (normalized)
LLM Confidence Score	0.520
Golden Answer (Severity Class)	1.0 (normalized)
Prediction Error	0.473

Retrieved Context:

Title: [Hepatitis probably caused by exifone (Adlone)].
Exifone, a drug recently proposed for the treatment of cognitive deficiencies of old age, has been marketed in France beginning in April 1988. This report concerns 2 patients who developed jaundice after taking this drug for 2 and 5 months, respectively. Serum aminotransferase was markedly increased. There was no hepatic failure. In both cases, histologic examination of a liver sample showed centrilobular hepatocyte necrosis and cholestasis. Necrotic cells were infiltrated with numerous red blood cells and scarce inflammatory cells. These lesions were associated with alterations of the walls of centrilobular veins. Discontinuation of exifone was followed by the prompt disappearance... (truncated)

Title: Mechanisms of drug toxicity and relevance to pharmaceutical development.
Toxicity has been estimated to be responsible for the attrition of approximately one-third of drug candidates and is a major contributor to the high cost of drug development, particularly when not recognized until late in clinical trials or post-marketing. The causes of drug toxicity can be classified in several ways and include mechanism-based (on-target) toxicity, immune hypersensitivity, off-target toxicity, and bioactivation/covalent modification. In addition, idiosyncratic responses are rare but can be one of the most problematic issues; several hypotheses for these have been advanced. Although covalent binding of drugs to proteins was described almost 40 years ago, the significance to... (truncated)

Title: Association of CYP1A1 and CYP1B1 inhibition in in vitro assays with drug-induced liver injury.
Drug-induced liver injury (DILI) is one of the major causes for the discontinuation of drug development and withdrawal of drugs from the market. Since it is known that reactive metabolite formation and being substrates or inhibitors of cytochrome P450s (P450s) are associated with DILI, we systematically investigated the association between human P450 inhibition and DILI. The inhibitory activity of 266 DILI-positive drugs (DILI drugs) and 92 DILI-negative drugs (no-DILI drugs), which were selected from Liver Toxicity Knowledge Base (US Food and Drug Administration), against 8 human P450 forms was assessed using recombinant enzymes and luminescent substrates, and the threshold values... (truncated)

Title: Vitamin C Inhibits Blood-Stage <i>Plasmodium</i> Parasites via Oxidative Stress.
During the <i>Plasmodium</i> erythrocytic cycle, glucose is taken up by glucose transporters (GLUTs) in red blood cells (RBCs) and supplied to parasites via the <i>Plasmodium</i> hexose transporter. Here, we demonstrate that the glucose uptake pathway in infected RBCs (iRBCs) can be hijacked by vitamin C (Vc). GLUTs preferentially transport the oxidized form of Vc, which is subsequently reduced in the cytosol. Vc, which is expected to burden the intracellular reducing capacity, inhibits <i>Plasmodium berghei</i> and <i>Plasmodium falciparum</i> growth. Vc uptake is drastically increased in iRBCs, with a large proportion entering parasites. Increased absorption of Vc causes accumulation of reactive oxygen... (truncated)

Title: Plants of the Genus <i>Terminalia</i>: An Insight on Its Biological Potentials, Pre-Clinical and Clinical Studies.
The evaluation and confirmation of healing properties of several plant species of genus <i>Terminalia</i> based on their traditional uses and the clinical claims are of utmost importance. Genus <i>Terminalia</i> has received more attention to assess and validate the therapeutic potential and clinical approval due to its immense folk medicinal and traditional applications. Various species of <i>Terminalia</i> genus are used in the form of herbal medicine and formulations, in treatment of diseases, including headache, fever, pneumonia, flu, geriatric, cancer, to improve memory, abdominal and back pain, cough and cold, conjunctivitis, diarrhea, heart disorder, leprosy, sexually transmitted diseases, and urinary tract disorders.... (truncated)

MESH:D004155 - diphenhydramine

Summary:

LLM Prediction Score	0.471 (normalized)
LLM Confidence Score	0.990
Golden Answer (Severity Class)	0.0 (normalized)
Prediction Error	0.471

Retrieved Context:

Title: Diphenhydramine as a Cause of Drug-Induced Liver Injury.
Drug-induced liver injury (DILI) is the most common cause of acute liver failure in the Unites States and accounts for 10% of acute hepatitis cases. We report the only known case of diphenhydramine-induced acute liver injury in the absence of concomitant medications. A 28-year-old man with history of 13/14-chromosomal translocation presented with fevers, vomiting, and jaundice. Aspartate-aminotransferase and alanine-aminotransferase levels peaked above 20,000 IU/L and 5,000 IU/L, respectively. He developed coagulopathy but without altered mental status. Patient reported taking up to 400 mg diphenhydramine nightly, without concomitant acetaminophen, for insomnia. He denied taking other medications, supplements, antibiotics, and herbals. A... (truncated)

Title: Diphenhydramine overdose detected early by integration of toxidrome and electrocardiography and treated with venoarterial extracorporeal membrane oxygenation: a case report.
Drug overdose can lead to a range of symptoms, including potentially life-threatening cardiac arrhythmias. However, identifying the specific causative drug upon admission can be challenging in many cases. The toxidrome approach is a method that utilizes toxidromes, which are collections of findings obtained from physical examination and ancillary tests, that may be caused by a specific toxin. In this particular case, a man presented with an unknown drug overdose that caused symptoms indicative of anticholinergic effects and abnormal electrocardiogram (ECG) findings. The ECG revealed an R wave in lead aVR, S waves in leads I and aVL, and wide QRS... (truncated)

Title: Focus on Over-the-Counter Drugs' Misuse: A Systematic Review on Antihistamines, Cough Medicines, and Decongestants.
Background: Over the past 20 years or so, the drug misuse scenario has seen the emergence of both prescription-only and over-the-counter (OTC) medications being reported as ingested for recreational purposes. OTC drugs such as antihistamines, cough/cold medications, and decongestants are reportedly the most popular in being diverted and misused. Objective: While the current related knowledge is limited, the aim here was to examine the published clinical data on OTC misuse, focusing on antihistamines (e.g., diphenhydramine, promethazine, chlorpheniramine, and dimenhydrinate), dextromethorphan (DXM)- and codeine-based cough medicines, and the nasal decongestant pseudoephedrine. Methods: A systematic literature review was carried out with the... (truncated)

Title: Jaundice and rash associated with the use of phenobarbital and hydrochlorothiazide.
Rash, lymphadenopaty, splenomegaly, periorbital edema, and hepatitis occurred in an 18-year-old woman who was taking phenobarbital and hydrochlorothiazide. Tests for fluorescent antinuclear antibody and hepatitis-associated antigen and antibody were negative. Liver biopsy was not characteristic of viral hepatitis. Clinical recovery occurred within two weeks. Treatment consisted of withdrawal of the above drugs plus the administration of methylprednisolone and diphenhydramine.

Title: A case of diphenhydramine intoxication showing prolonged false positive tricyclic antidepressant in the urine assay.
The urine immunochromatographic assay is a useful screening tool for patients suspected of acute drug intoxication in emergency conditions. Diphenhydramine intoxication shows symptoms similar to those of tricyclic antidepressant (TCA) intoxication.

MESH:D016912 - levonorgestrel

Summary:

LLM Prediction Score	0.470 (normalized)
LLM Confidence Score	0.990
Golden Answer (Severity Class)	0.0 (normalized)
Prediction Error	0.470

Retrieved Context:

Title: Hepatotoxicity induced by a second-generation combined oral contraceptive: case report and review of the literature.
Case: Second-generation combined oral contraceptives (COCs) are widely used and are believed to be safe for birth control and in the treatment of gynaecological diseases. No randomised controlled study has shown elevations in alanine transaminase (ALT) levels in relation to the use of a second-generation COC. We report a case of drug-induced liver injury (DILI) in a young, moderately obese woman, due to the use of a second-generation COC containing 30 µg ethinylestradiol and 150 µg levonorgestrel. COC use had been initiated 2 years prior to admission to our department. The diagnosis was based on elevated levels of ALT during COC use... (truncated)

Title: Influence of acetaminophen-induced hepatic necrosis on the pharmacokinetics of levonorgestrel.
The pharmacokinetics of levonorgestrel in control mice and in mice with induced hepatic necrosis were investigated. Hepatic necrosis was induced by fasting mice for 18 hr and then giving the acetaminophen in a dose of 750 mg/kg i.p. Measurement by radioimmunoassay of plasma levonorgestrel has been used to compare the pharmacokinetic parameters of the drug after oral and intravenous administration in both control and liver-affected animals. The pharmacokinetic parameters of levonorgestrel in control mice showed some similarity to those observed in human subjects, save the systemic bioavailability which was about 67% in mice compared to 100% in humans. The animals... (truncated)

Title: Effects of oral contraception on liver function tests and serum proteins in women with active schistosomiasis.
Thirty-eight women with urinary or intestinal schistosomiasis but without clinical or laboratory evidence of hepatic involvement and 30 healthy control women were treated with an oral contraceptive containing 0.05mg ethinyl estradiol and 0.05mg levonorgestrel for six consecutive months. Liver function tests (serum bilirubin, SGOT, SGPT, serum alkaline phosphatase) and serum proteins (total, albumin, globulins, ceruloplasmin, haptoglobin and alpha-1 antitrypsin) were measured before beginning the treatment and after three and six months of use. Both group experienced significant increases in SGOT, SGPT and serum alkaline phosphatase during the first three months of treatment with tendencies to decrease during the subsequent 3... (truncated)

Title: Levonorgestrel-Releasing Intrauterine Device-Related Acute Liver Injury.
Oral contraceptives have long been associated with liver injury. However, very little attention is paid to the metabolic side effects of hormone-releasing intrauterine devices (IUDs). These devices are generally considered safe and commonly used. We report for the first time acute liver injury associated with a levonorgestrel-releasing IUD. Our patient did not have any comorbidities that could have caused or exacerbated liver injury. A detailed workup and liver biopsy remained negative for any other potential cause of liver injury. The patient's symptoms resolved with removal of the device. She remained symptom free on subsequent outpatient follow-ups.

Title: Effects of oral contraception on liver function tests and serum proteins in women with past viral hepatitis.
Forty-three women who had viral hepatitis one or more years ago and 35 healthy women who were age and parity matched were given an oral contraceptive containing 0.05mg ethinyl estradiol and 0.5mg levonorgestrel for six consecutive months. Liver function tests (serum bilirubin, SGOT, SGPT and serum alkaline phosphatase) and serum proteins (total, albumin, globulins, ceruloplasmin, haptoglobin and alpha-1 antitrypsin) were measured before beginning treatment and after three and six months of use. Past hepatitis women experienced increased unconjugated bilirubin, SGOT, SGPT and alkaline phosphatase levels throughout the six months while the control women showed less pronounced changes during the first... (truncated)

MESH:D006886 - hydroxychloroquine

Summary:

LLM Prediction Score	0.407 (normalized)
LLM Confidence Score	0.990
Golden Answer (Severity Class)	0.875 (normalized)
Prediction Error	0.468

Retrieved Context:

Title: Severe acute hepatitis related to hydroxychloroquine in a woman with mixed connective tissue disease.
Antimalarial drugs are used for the control of mild manifestations of autoimmune diseases due to their low toxicity. Hydroxychloroquine (HCQ), a alpha-hydroxylated derivative of chloroquine, is usually preferred because of its higher tolerability. Mild and unspecific gastrointestinal symptoms are the main secondary effects related to HCQ use. Less than 1% of subjects show liver enzyme increase, although the percentage can be as high as 50% in subjects with chronic liver disease. A woman with mixed connective tissue disease who developed a reversible acute hepatitis shortly after the initiation of low-dose HCQ is presented. Two previous cases of patients with acute... (truncated)

Title: Hydroxychloroquine Improves Obesity-Associated Insulin Resistance and Hepatic Steatosis by Regulating Lipid Metabolism.
The burden of obesity and associated cardiometabolic diseases has been considered as an important risk factor for lupus patients. Therefore, whether obesity is involved in the over-activation of autoimmune response has attracted more and more attention. Hydroxychloroquine is a synthetic antimalarial drug and has been the clinical treatment of rheumatic diseases irreplaceable first-line drugs. Hydroxychloroquine has been suggested to have beneficial effects on lipids and insulin sensitivity, which may contribute in lowering high cardiovascular risk in SLE patients. However, its mechanism on insulin sensitivity and lipid disorders is far from being completely understood. In the present study, the therapeutic effects... (truncated)

Title: Acute chloroquine and hydroxychloroquine toxicity: A review for emergency clinicians.
Acute chloroquine and hydroxychloroquine toxicity is characterized by a combination of direct cardiovascular effects and electrolyte derangements with resultant dysrhythmias and is associated with significant morbidity and mortality.

Title: Hydroxychloroquine-induced toxic hepatitis in a patient with systemic lupus erythematosus: a case report.
Increased serum level of liver enzymes is a common finding in patients with systemic lupus erythematosus (SLE). Hepatotoxic drugs, viral hepatitis and fatty liver are thought to be the main causes of hepatic lesion in these patients. Our aim was to determine the cause of strikingly elevated liver enzymes in a case with systemic lupus presenting with acute abdomen. Liver enzyme abnormality was defined as a 10-fold or greater increase in aspartate aminotransferase and alanine aminotransferase. Acute toxic hepatitis was diagnosed, which rapidly returned to normal after cessation of the suspected causative medication, hydroxychloroquine, and subsequent administration of mycophenolate mofetil.... (truncated)

Title: Hydroxychloroquine attenuates autoimmune hepatitis by suppressing the interaction of GRK2 with PI3K in T lymphocytes.
Hydroxychloroquine (HCQ) is derivative of the heterocyclic aromatic compound quinoline, which has been used for the treatment of autoimmune diseases. The central purpose of this study was to investigate therapeutic effects and inflammatory immunological molecular mechanism of HCQ in experimental autoimmune hepatitis (AIH). Treatment with HCQ ameliorated hepatic pathologic damage, inflammatory infiltration, while promoted regulatory T cell (T_{reg}) and down-regulated CD8⁺T cell differentiation in AIH mice induced by S-100 antigen. <i>In vitro</i>, HCQ also suppressed pro-inflammatory cytokine (IFN-γ, TNF-α, and IL-12) secretion, promoted anti-inflammatory cytokine (TGF-β₁) secretion. HCQ mainly impaired T cell lipid metabolism but not glycolysis to promote T_{reg}... (truncated)

MESH:D011324 - primidone

Summary:

LLM Prediction Score	0.466 (normalized)
LLM Confidence Score	0.980
Golden Answer (Severity Class)	0.0 (normalized)
Prediction Error	0.466

Retrieved Context:

Title: Phenytoin-induced hypersensitivity reactions.
A case of phenytoin-induced hepatitis with mononucleosis is reported, and syndromes associated with phenytoin hypersensitivity reactions are discussed. A 23-year-old black woman with a two-month history of seizure disorder was admitted to a hospital with nausea, vomiting, fever, lymphadenopathy, diffuse maculopapular rash, left-upper-quadrant tenderness, and hepatomegaly. She was receiving phenytoin sodium 300 mg/day; carbamazepine 200 mg four times daily had been discontinued four days before admission because of leukopenia. Phenytoin was discontinued after admission; however, phenytoin 1 g i.v. was given for a tonic-clonic seizure two days after admission, after which swelling of the face and legs and pruritus developed.... (truncated)

Title: Does cytochrome P450 liver isoenzyme induction increase the risk of liver toxicity after paracetamol overdose?
Paracetamol (acetaminophen, N-acetyl-p-aminophenol, 4-hydroxyacetanilide) is the most common cause of acute liver failure in developed countries. There are a number of factors which potentially impact on the risk of an individual developing hepatotoxicity following an acute paracetamol overdose. These include the dose of paracetamol ingested, time to presentation, decreased liver glutathione, and induction of cytochrome P450 (CYP) isoenzymes responsible for the metabolism of paracetamol to its toxic metabolite N-acetyl-p-benzoquinoneimine (NAPQI). In this paper, we review the currently published literature to determine whether induction of relevant CYP isoenzymes is a risk factor for hepatotoxicity in patients with acute paracetamol overdose. Animal... (truncated)

Title: Use of antiepileptic drugs in hepatic and renal disease.
The use of antiepileptic drugs in patients with renal or hepatic disease is common in clinical practice. Since the liver and kidney are the main organs involved in the elimination of most drugs, their dysfunction can have important effects on the disposition of antiepileptic drugs. Renal or hepatic disease can prolong the elimination of the parent drug or an active metabolite leading to accumulation and clinical toxicity. It can also affect the protein binding, distribution, and metabolism of a drug. The protein binding of anionic acidic drugs, such as phenytoin and valproate, can be reduced significantly by renal failure, causing... (truncated)

Title: Essential tremor leading to toxic liver damage successfully treated with deep brain stimulation.
We present a patient with severe essential tremor (ET), who underwent thalamic deep brain stimulation (DBS). After previous medical treatment with Propranolol and Primidone failed, the patient resorted to alcohol, which greatly alleviated the symptoms. The downside of this situation, however, was that it led to alcoholism with severely disturbed liver enzymes and hepatic steatosis.

Title: Ursodeoxycholic Acid Can Improve Liver Transaminase Quantities in Children with Anticonvulsant Drugs Hepatotoxicity: a Pilot Study.
The present study has been directed to investigate Ursodeoxycholic Acid (UDCA) effect in children, to reduce the high Liver transaminases induced by Anticonvulsant drugs (drug induced hepatitis). This idea has been driven from Cytoprotective and antioxidant properties of UDCA to be used in drug induced inflammation in Liver. Twenty two epileptic patients aged between 4 mo - 3 yr whom were under anticonvulsant therapy with drugs such as valperoic acid, primidone, levetiracetam, Phenobarbital or any combination of them and had shown Liver transaminases rise , after rule out of Viral-Autoimmune, Metabolic and Anatomic causes, have been prescribed UDCA in dose... (truncated)

MESH:C000123 - bendazac

Summary:

LLM Prediction Score	0.536 (normalized)
LLM Confidence Score	0.820
Golden Answer (Severity Class)	1.0 (normalized)
Prediction Error	0.464

Retrieved Context:

Title: [Bendazac hepatotoxicity: analysis of 16 cases].
Hepatic lesions induced by non-steroid antiinflammatory drugs are a common cause of disease, particularly among elderly patients. Although its clinical expression is polymorphous, occasional increases in serum transaminase values predominate. Sixteen cases of liver involvement by bendazac were studied. This NSAID is used in Spain for the treatment of cataracts, from a total of 112 patients observing such therapy. The clinical spectrum of liver disease induced by bendazac is discussed and emphasis is placed on the necessity to judiciously select the indications for NSAIDs.

Title: Perturbation of bile acid homeostasis is an early pathogenesis event of drug induced liver injury in rats.
Drug-induced liver injury (DILI) is a significant consideration for drug development. Current preclinical DILI assessment relying on histopathology and clinical chemistry has limitations in sensitivity and discordance with human. To gain insights on DILI pathogenesis and identify potential biomarkers for improved DILI detection, we performed untargeted metabolomic analyses on rats treated with thirteen known hepatotoxins causing various types of DILI: necrosis (acetaminophen, bendazac, cyclosporine A, carbon tetrachloride, ethionine), cholestasis (methapyrilene and naphthylisothiocyanate), steatosis (tetracycline and ticlopidine), and idiosyncratic (carbamazepine, chlorzoxasone, flutamide, and nimesulide) at two doses and two time points. Statistical analysis and pathway mapping of the nearly 1900 metabolites... (truncated)

Title: Oxidative Stress and Liver Morphology in Experimental Cyclosporine A-Induced Hepatotoxicity.
Cyclosporine A is an immunosuppressive drug used after organ's transplantation. The adverse effects on such organs as kidney or liver may limit its use. Oxidative stress is proposed as one of the mechanisms of organs injury. The study was designed to elucidate CsA-induced changes in liver function, morphology, oxidative stress parameters, and mitochondria in rat's hepatocytes. Male Wistar rats were used: group A (control) receiving physiological saline, group B cyclosporine A in a dose of 15 mg/kg/day subcutaneously, and group C the CsA-vehicle (olive oil). On the 28th day rats were anesthetized. The following biochemical changes were observed in CsA-treated... (truncated)

Title: Comparison of Bile Acids and Acetaminophen Protein Adducts in Children and Adolescents with Acetaminophen Toxicity.
Metabolomics approaches have enabled the study of new mechanisms of liver injury in experimental models of drug toxicity. Disruption of bile acid homeostasis is a known mechanism of drug induced liver injury. The relationship of individual bile acids to indicators of oxidative drug metabolism (acetaminophen protein adducts) and liver injury was examined in children with acetaminophen overdose, hospitalized children with low dose exposure to acetaminophen, and children with no recent exposure to acetaminophen. Nine bile acids were quantified through targeted metabolomic analysis in the serum samples of the three groups. Bile acids were compared to serum levels of acetaminophen protein... (truncated)

Title: Mechanisms of drug toxicity and relevance to pharmaceutical development.
Toxicity has been estimated to be responsible for the attrition of approximately one-third of drug candidates and is a major contributor to the high cost of drug development, particularly when not recognized until late in clinical trials or post-marketing. The causes of drug toxicity can be classified in several ways and include mechanism-based (on-target) toxicity, immune hypersensitivity, off-target toxicity, and bioactivation/covalent modification. In addition, idiosyncratic responses are rare but can be one of the most problematic issues; several hypotheses for these have been advanced. Although covalent binding of drugs to proteins was described almost 40 years ago, the significance to... (truncated)

MESH:D001241 - aspirin

Summary:

LLM Prediction Score	0.460 (normalized)
LLM Confidence Score	0.990
Golden Answer (Severity Class)	0.0 (normalized)
Prediction Error	0.460

Retrieved Context:

Title: Effects of non-narcotic analgesics on the liver.
Serious hepatotoxicity is uncommon with the proper therapeutic use of non-narcotic analgesics but experience with new non-steroidal anti-inflammatory drugs (NSAIDs) is limited. Drugs such as ibufenac, fenclofenac and benoxaprofen were withdrawn from the market because of hepatotoxicity, and liver damage has been reported on occasion with virtually all non-narcotic analgesics. However, a clear pattern of toxicity with characteristic clinical, biochemical and histopathological abnormalities has emerged with relatively few. With the exception of acute hepatic necrosis following overdosage of paracetamol, little is known of the mechanisms of liver injury induced by non-narcotic analgesics. Involvement of the liver in a generalised drug... (truncated)

Title: Effects of aspirin and acetaminophen on the liver.
The mechanism for aspirin-caused liver injury is not clear. Aspirin produces hepatotoxic reactions as a cumulative phenomenon, requiring days or weeks to develop. Patients with active rheumatic or collagen disease, as well as children, are especially susceptible. Blood levels of salicylate higher than 25 mg/dL are particularly likely to lead to hepatic injury. Levels lower than 15 mg/dL rarely do. The mechanism for acetaminophen liver damage is quite clear. It produces hepatic injury as a result of a large single overdose, usually suicidal in intent. Patients with acetaminophen blood levels higher than 300 mg/dL at four hours after intake are... (truncated)

Title: Aspirin hepatotoxicity.
A case of aspirin hepatotoxicity in a 46-year-old male with rheumatoid arthritis is discussed, and this adverse reaction is reviewed. The patient was started on 900 mg aspirin four times daily; five days later the dose was increased to 1200 mg four times daily. After six days' therapy of 4.8 g aspirin daily, the serum salicylate level rose to 25 mg/100 ml and liver enzymes became elevated. Aspirin was discontinued and ibuprofen, 600 mg four times daily, begun. Eight days after cessation of aspirin therapy, the patient's liver enzyme values returned to normal. Previous case reports and studies of aspirin-induced... (truncated)

Title: Hepatotoxicity of non-narcotic analgesics.
The central role of the liver in drug metabolism sets the stage for drug-related hepatotoxicity. The incidence of hepatotoxicity associated with non-narcotic analgesics is low, but their widespread use both prescription and over-the-counter-makes analgesic-associated hepatotoxicity a clinically and economically important problem. Hepatotoxicity is considered a class characteristic of nonsteroidal anti-inflammatory drugs (NSAIDs), despite the fact that they are a widely diverse group of chemicals. In fact, there are many differences in the incidence, histologic pattern, and mechanisms of hepatotoxicity between, as well as within, chemical classes. Most NSAID reactions are hepatocellular and occur because of individual patient susceptibility (idiosyncrasy). Aspirin,... (truncated)

Title: Daily Aspirin Use Associated With Reduced Risk For Fibrosis Progression In Patients With Nonalcoholic Fatty Liver Disease.
There are few data from prospective studies on the effects of aspirin on fibrosis in patients with nonalcoholic fatty liver disease (NAFLD).

MESH:D002216 - captopril

Summary:

LLM Prediction Score	0.415 (normalized)
LLM Confidence Score	0.990
Golden Answer (Severity Class)	0.875 (normalized)
Prediction Error	0.460

Retrieved Context:

Title: Cholestatic jaundice associated with captopril therapy.
Captopril has attained widespread use as an effective agent in the treatment of heart failure and hypertension. Dermatological, renal and haematological toxicity associated with its use has been widely described and is usually well recognized. There have been comparatively few reports implicating it as causing hepatic drug reactions. Most descriptions have emphasized strongly cholestatic features, although a mixed hepatocellular cholestatic picture and predominant hepatocellular reactions have been reported. Between November 1972 and June 1990 only five cases of possible Captopril-associated hepatic dysfunction were reported to the Australian Adverse Drug Reaction Advisory Committee. Cases reported suggest equal sex distribution, latent period... (truncated)

Title: Captopril-associated "pseudocholangitis'. A case report and review of the literature.
Captopril, a competitive inhibitor of angiotensin-converting enzyme, is widely used in the treatment of hypertension and heart failure. Captopril is known to be associated with dermatologic, hematologic, and pulmonary adverse effects. However, hepatotoxicity is extremely rare. A patient with severe cholestatic jaundice induced by captopril is presented. On admission to the hospital, the patient was diagnosed and treated as having cholangitis. Review of the literature showed similar occurrences in other patients. Patients treated with captopril who develop "atypical cholangitis" should be suspected of having captopril-associated liver damage.

Title: Ramipril-associated cholestasis in the setting of recurrent drug-induced liver injury.
Aim: Angiotensin-converting enzyme inhibitors (ACEIs) are commonly used to treat hypertension. Although generally well tolerated, the adverse effects of ACEIs include hypotension, cough, acute kidney injury and hyperkalemia. Rare reports of ACEI-induced hepatotoxicity have been described, most notably a cholestatic pattern of injury related to captopril. A 67-year-old male presented to the emergency department with a three-week history of jaundice, pruritis and weakness. Eight weeks before, he began taking ramipril and clopidogrel. His past medical history was significant for previous acute cholestatic liver injury approximately 20 years earlier, which was attributed to methimazole. Abnormal blood work demonstrated aspartate aminotransferase (AST)... (truncated)

Title: [Long-term treatment of severe cardiac failure with captopril (author's transl)].
Nine patients with primary, non-obstructive myocardiopathy were treated during 6 months with captopril in daily doses of 75 to 225 mg. Early signs of haemodynamic improvement included significant increase in cardiac output (p Less Than 0.01) and stroke volume (p Less Than 0.01) and significant decrease in peripheral resistance (p Less Than 0.001) and pulmonary wedge pressure (p Less Than 0.001). These effects were sustained throughout treatment and were accompanied with noticeable improvement in the patients' functional status. Echocardiographic studies showed a reduction in the telesystolic (p Less Than 0.01) and telediastolic (p Less Than 0.05) diameters of the left... (truncated)

Title: Antioxidant effects of captopril against lead acetate-induced hepatic and splenic tissue toxicity in Swiss albino mice.
Considering that lead caused a lot of health problems in the world, the present study was carried out to investigate the protective effect of captopril as antioxidants to reduce liver and spleen toxicity induced by lead. Animals were divided into 3 groups, the 1st group served as control group, the 2nd group received 20 mg/kg of lead acetate and the 3rd group received 50 mg/kg of captopril one hour prior to lead administration for 5 days. Results showed that lead intake caused severe alterations in the liver and spleen manifested by hepatocytes degeneration, leukocytic infiltration, fibrosis in liver and moderate... (truncated)

MESH:D016049 - didanosine

Summary:

LLM Prediction Score	0.541 (normalized)
LLM Confidence Score	0.970
Golden Answer (Severity Class)	1.0 (normalized)
Prediction Error	0.459

Retrieved Context:

Title: Antiviral hepatitis and antiretroviral drug interactions.
More and more HIV-infected patients are treated for viral hepatitis, increasing interactions. HEPATITIS C: The concomitant use of didanosine and ribavirin increases the risk of mitochondrial toxicity, responsible for pancreatitis and/or lactic acidosis. Lactic acidosis is characterized by a high mortality rate. Thus, didanosine, but also stavudine, should not be co-administered with ribavirin. Cases of hepatic decompensation have been reported in cirrhotics concomitantly receiving ribavirin and didanosine. Thus, this co-admininistration should be contraindicated in patients with advanced liver fibrosis. Anemia is a frequent side effect of ribavirin. In patients with zidovudine-related anemia, this drug should be discontinued before prescribing ribavirin.... (truncated)

Title: Randomized study of two doses of didanosine in children infected with human immunodeficiency virus.
2'3'-Dideoxyinosine (didanosine) is a nucleoside analog active in vitro against human immunodeficiency virus. Few data are available regarding its use for the treatment of children. In a single-center, randomized, open-label trial, we compared two dosages of didanosine (120 vs 270 mg/m2 per day) for at least 6 months in 34 children infected with human immunodeficiency virus who had become resistant to or were intolerant of zidovudine. Serum levels of didanosine 1 hour after administration were significantly different in the two groups and remained stable with time. There was a significant reduction in human immunodeficiency virus-p24 antigenemia and quantitative cellular viremia... (truncated)

Title: [Antiretroviral agents in HIV-infected patients with cirrhosis].
Since highly active antiretroviral therapies became available, the future of HIV-infected patients has been transformed. However, 20 to 25% of HIV patients are co-infected with hepatitis B or C viruses, and the course of these diseases has worsened, since these patients have an enhanced sensitivity to the hepatic toxicity of antiretrovirals. The relation between high antiretroviral concentrations and toxicity has been clearly demonstrated with certain protease inhibitors and non-nucleoside reverse transcriptase inhibitors (NNRTI) that have a predominantly hepatic metabolism (CYP4503A4). The nucleoside reverse transcriptase inhibitors (NRTI) are not predominantly metabolized by the liver, but may nevertheless be toxic for the... (truncated)

Title: Rapid improvement in fasting lipids and hepatic toxicity after switching from didanosine/lamivudine to tenofovir/emtricitabine in patients with toxicity attributable to didanosine.
No abstract available.

Title: Liver Fibrosis during Antiretroviral Treatment in HIV-Infected Individuals. Truth or Tale?
After the introduction of antiretroviral treatment (ART) back in 1996, the lifespan of people living with HIV (PLWH) has been substantially increased, while the major causes of morbidity and mortality have switched from opportunistic infections and AIDS-related neoplasms to cardiovascular and liver diseases. HIV itself may lead to liver damage and subsequent liver fibrosis (LF) through multiple pathways. Apart from HIV, viral hepatitis, alcoholic and especially non-alcoholic liver diseases have been implicated in liver involvement among PLWH. Another well known cause of hepatotoxicity is ART, raising clinically significant concerns about LF in long-term treatment. In this review we present the... (truncated)

MESH:D001539 - bendroflumethiazide

Summary:

LLM Prediction Score	0.170 (normalized)
LLM Confidence Score	0.870
Golden Answer (Severity Class)	0.625 (normalized)
Prediction Error	0.455

Retrieved Context:

Title: Screening for biomarkers of liver injury induced by Polygonum multiflorum: a targeted metabolomic study.
Heshouwu (HSW), the dry roots of Polygonum multiflorum, a classical traditional Chinese medicine is used as a tonic for a wide range of conditions, particularly those associated with aging. However, it tends to be taken overdose or long term in these years, which has resulted in liver damage reported in many countries. In this study, the indicative roles of nine bile acids (BAs) were evaluated to offer potential biomarkers for HSW induced liver injury. Nine BAs including cholic acid (CA) and chenodeoxycholic acid (CDCA), taurocholic acid (TCA), glycocholic acid (GCA), glycochenodeoxycholic acid (GCDCA), deoxycholic acid (DCA), glycodeoxycholic acid (GDCA), ursodeoxycholic... (truncated)

Title: Liver steatosis and fibrosis in people with human immunodeficiency virus in West Africa and the relationship with hepatitis B virus coinfection.
There is a heavy burden of liver disease in West Africa. While the role of hepatitis B virus (HBV) infection is well recognized, less is known about the contributing role of liver steatosis and how the two interact in the context of human immunodeficiency virus (HIV) infection. Adults with HIV in Ghana underwent FibroScan measurements to determine prevalence of liver steatosis (expressed as controlled attenuation parameter [CAP]) and fibrosis (expressed as liver stiffness [LS]). We explored contributing factors in linear regression models, including demographics, lifestyle characteristics, medical history, HIV and HBV status, and measurements of metabolic syndrome. Among 329 adults... (truncated)

Title: Association of CYP1A1 and CYP1B1 inhibition in in vitro assays with drug-induced liver injury.
Drug-induced liver injury (DILI) is one of the major causes for the discontinuation of drug development and withdrawal of drugs from the market. Since it is known that reactive metabolite formation and being substrates or inhibitors of cytochrome P450s (P450s) are associated with DILI, we systematically investigated the association between human P450 inhibition and DILI. The inhibitory activity of 266 DILI-positive drugs (DILI drugs) and 92 DILI-negative drugs (no-DILI drugs), which were selected from Liver Toxicity Knowledge Base (US Food and Drug Administration), against 8 human P450 forms was assessed using recombinant enzymes and luminescent substrates, and the threshold values... (truncated)

Title: State of the Art and Uses for the Biopharmaceutics Drug Disposition Classification System (BDDCS): New Additions, Revisions, and Citation References.
The Biopharmaceutics Drug Disposition Classification system (BDDCS) is a four-class approach based on water solubility and extent of metabolism/permeability rate. Based on the BDDCS class to which a drug is assigned, it is possible to predict the role of metabolic enzymes and transporters on the drug disposition of a new molecular entity (NME) prior to its administration to animals or humans. Here, we report a total of 1475 drugs and active metabolites to which the BDDCS is applied. Of these, 379 are new entries, and 1096 are revisions of former classification studies with the addition of references for the approved... (truncated)

Title: Quantitative NTCP pharmacophore and lack of association between DILI and NTCP Inhibition.
The human sodium taurocholate cotransporting polypeptide (NTCP) is a hepatic bile acid transporter. Inhibition of NTCP uptake may potentially also prevent hepatitis B virus (HBV) infection. The first objective was to develop a quantitative pharmacophore for NTCP inhibition. Recent studies showed that hepatotoxic drugs could inhibit bile acid uptake into hepatocytes, without inhibiting canalicular efflux, and cause bile acid elevation in plasma. Hence, a second objective was to examine whether NTCP inhibition is associated with drug induced liver injury (DILI). Twenty-seven drugs from our previous study were used as the training set to develop a quantitative pharmacophore. From secondary screening... (truncated)

MESH:D000077143 - docetaxel

Summary:

LLM Prediction Score	0.826 (normalized)
LLM Confidence Score	0.990
Golden Answer (Severity Class)	0.375 (normalized)
Prediction Error	0.451

Retrieved Context:

Title: Weekly docetaxel versus CMF as adjuvant chemotherapy for elderly breast cancer patients: safety data from the multicentre phase 3 randomised ELDA trial.
Within an ongoing multicentre phase 3 randomised trial (ELDA, cancertrials.gov ID: NCT00331097), early breast cancer patients, 65-79 years old, with average to high risk of recurrence, are randomly assigned to receive CMF (cyclophosphamide 600 mg/m2, methotrexate 40 mg/m2, fluorouracil 600 mg/m2, days 1-8) or docetaxel (35 mg/m2 days 1-8-15), every 4 weeks. Here we report an unplanned safety analysis prompted by an amendment introducing creatinine clearance as a tool to adjust methotrexate dose. Before such change, 101 patients with a median age of 70 were randomly assigned CMF (53 patients) or docetaxel (48 patients). At least one grades 3-4 toxic... (truncated)

Title: Phase I/II study of S-1 combined with weekly docetaxel in patients with metastatic gastric carcinoma.
We designed a phase I/II trial of S-1 combined with weekly docetaxel to determine the maximum tolerated dose (MTD) and recommended dose (RD) and to evaluate the efficacy and toxicity in metastatic gastric carcinoma (MGC). Patients with measurable disease received S-1 orally b.i.d. on days 1-14 and docetaxel intravenously on days 1 and 8 every 3 weeks. In phase I (n=30), each cohort received escalating doses of S-1 (30-45 mg m(-2) b.i.d.) and docetaxel (25-40 mg m(-2)); MTD was 45 mg m(-2) b.i.d. S-1/35 mg m(-2) docetaxel and RD was 40 mg m(-2) b.i.d. S-1/35 mg m(-2) docetaxel. Dose-limiting toxicities... (truncated)

Title: Optimal use of docetaxel (Taxotere): maximizing its potential.
The safety of docetaxel (Taxotere) has been evaluated in the safety overview population consisting of 1070 patients recruited to phase II trials. These patients received a total of 4989 cycles of therapy (median four cycles per patient). Since docetaxel is known to be metabolized in the liver, hepatic impairment was predicted to be a risk factor for increased toxicity and was studied prospectively, comparing the 42 patients in the overview population with moderate hepatic impairment with the 1028 patients with liver function within normal limits. Hepatic dysfunction was associated with an increase in the percentage of cycles of therapy during... (truncated)

Title: Multiple effects of magnesium isoglycyrrhizinate on the disposition of docetaxel in docetaxel-induced liver injury.
1. Magnesium isoglycyrrhizinate (MgIg) has been extensively used in treating liver injury which is the common adverse reaction of docetaxel (DOC). Due to the narrow therapeutic window, small changes in pharmacokinetic profiles can alter the toxicity and therapeutic efficacy of DOC significantly. The study aimed to explore the effects of MgIg on the disposition of DOC and the potential mechanism in DOC-induced liver injury. 2. Pharmacokinetics and tissues distribution behaviors showed that there was no significant difference between DOC group (DOCG) and MgIg + DOC group (MDOCG). The mRNA and protein levels of cytochrome P450 3A1 (CYP3A1) in liver, intestine, and kidney were significantly... (truncated)

Title: [Lethal course after chemotherapy with docetaxel. Acute liver failure with accompanying erythema multiforme major].
We report on the lethal course of a patient receiving low-dose, weekly docetaxel who developed acute liver failure accompanied by a Stevens-Johnson syndrome. After receiving the fifth application of his chemotherapy, the patient was admitted to hospital because of neutropenia and severe erythema. The course worsened towards an acute liver failure and an erythema multiforme major. Despite an interdisciplinary approach, the further course could not be influenced and the patient died 6 weeks after admission due the toxicity of docetaxel. This case report underlines the spectrum of toxicity of docetaxel even in the low-dose weekly schedule.

MESH:D008777 - methyltestosterone

Summary:

LLM Prediction Score	0.699 (normalized)
LLM Confidence Score	0.990
Golden Answer (Severity Class)	0.25 (normalized)
Prediction Error	0.449

Retrieved Context:

Title: Liver damage from long-term methyltestosterone.
Of 60 patients (42 female transsexuals and 18 impotent males) receiving long-term therapy with methyltestosterone 50 mg three times a day, 19 had abnormal liver-function tests and 33 out of 52 had abnormal liver scans, particularly those who had been treated for more than a year. Liver biopsy specimens showed accumulation of hepatocytes in the liver cords and within the walls of centrilobular veins, and early peliosis hepatis. One patient had a hepatic adenoma. Of the androgens, only 17alpha-alkylated steroids seem to be implicated in the development of cholestatic jaundice, peliosis hepatis, and liver tumours.

Title: Methyltestosterone-induced cholestasis. The importance of disproportionately low serum alkaline phosphatase level.
We describe a 64-year-old man who developed cholestatic jaundice after receiving 20 to 40 mg of methyltestosterone daily for 6 months for impotence but failed to mention it as part of his drug history. He underwent endoscopic retrograde and papillotomy before a positive history for methyltestosterone ingestion could be obtained. Since methyltestosterone is most often used for sexual impotence, the patient may be quite reluctant to mention this hormone as part of his medication. A normal or mildly elevated alkaline phosphatase level, disproportionate to the level of hyperbilirubinemia seen in this patient and in all previous reports, appears to be... (truncated)

Title: Endocrine characterization of the designer steroid methyl-1-testosterone: investigations on tissue-specific anabolic-androgenic potency, side effects, and metabolism.
Various products containing rarely characterized anabolic steroids are nowadays marketed as dietary supplements. Herein, the designer steroid methyl-1-testosterone (M1T) (17β-hydroxy-17α-methyl-5α-androst-1-en-3-one) was identified, and its biological activity, potential adverse effects, and metabolism were investigated. The affinity of M1T toward the androgen receptor (AR) was tested in vitro using a yeast AR transactivation assay. Its tissue-specific androgenic and anabolic potency and potential adverse effects were studied in a Hershberger assay (sc or oral), and tissue weights and selected molecular markers were investigated. Determination of M1T and its metabolites was performed by gas chromatography mass spectrometry. In the yeast AR transactivation assay, M1T... (truncated)

Title: Anabolic androgenic steroid-induced liver injury: An update.
Anabolic androgenic steroids (AASs) are a group of molecules including endogenous testosterone and synthetic derivatives that have both androgenic and anabolic effects. These properties make them therapeutically beneficial in medical conditions such as hypogonadism. However, they are commonly bought illegally and misused for their anabolic, skeletal muscle building, and performance-enhancing effects. Supraphysiologic and long-term use of AASs affects all organs, leading to cardiovascular, neurological, endocrine, gastrointestinal, renal, and hematologic disorders. Hepatotoxicity is one of the major concerns regarding AASs treatment and abuse. Testosterone and its derivatives have been most often shown to induce a specific form of cholestasis, peliosis hepatis,... (truncated)

Title: Safety of estrogen/androgen regimens.
A persistent view of testosterone as the "male hormone" deprives many clinically androgen deficient women of effective treatment, although data from the 1960s to the present have indicted the importance of androgens to libido and feelings of well-being in women, providing relief from vasomotor symptoms that are unresponsive to estrogen alone. The safety of androgen replacement therapy is reviewed in this article. The risk of androgen toxicity is influenced by dosage and route of administration. Most products developed for use in men produce androgen levels that are too high for safety in women. Low-dose androgen replacement therapy as used in... (truncated)

MESH:D017257 - ramipril

Summary:

LLM Prediction Score	0.427 (normalized)
LLM Confidence Score	0.980
Golden Answer (Severity Class)	0.875 (normalized)
Prediction Error	0.448

Retrieved Context:

Title: Ramipril-associated cholestasis in the setting of recurrent drug-induced liver injury.
Aim: Angiotensin-converting enzyme inhibitors (ACEIs) are commonly used to treat hypertension. Although generally well tolerated, the adverse effects of ACEIs include hypotension, cough, acute kidney injury and hyperkalemia. Rare reports of ACEI-induced hepatotoxicity have been described, most notably a cholestatic pattern of injury related to captopril. A 67-year-old male presented to the emergency department with a three-week history of jaundice, pruritis and weakness. Eight weeks before, he began taking ramipril and clopidogrel. His past medical history was significant for previous acute cholestatic liver injury approximately 20 years earlier, which was attributed to methimazole. Abnormal blood work demonstrated aspartate aminotransferase (AST)... (truncated)

Title: Ramipril-induced liver injury: case report and review of the literature.
Ramipril, an inhibitor of the angiotensin-converting enzyme (ACEI), is a drug commonly used in the therapy of hypertension. ACEI-induced hepatotoxicity is rare, and most of the reported cases are associated with captopril. Here, we present the first case of ramipril-induced liver injury proven by positive rechallenge and a review of the literature including the data from the US Food and Drug Administration adverse event reporting system (FAERS).

Title: A logistic regression model based on inpatient health records to predict drug-induced liver injury caused by ramipril-An angiotensin-converting enzyme inhibitor.
Drug-induced liver injury (DILI) is a rare side effect of angiotensin-converting enzyme inhibitors (ACEIs). Ramipril is a widely used ACE compound because of its effectiveness in the treatment of hypertension and heart failure, as well as its low risk of adverse effects. However, the clinical features of ramipril, and the risk of DILI, have not been adequately studied. A retrospective cohort study was performed based on data from 3909 inpatients to compare the risk of DILI conferred by ramipril and other ACEIs. A logistic regression model was then constructed and validated against data from 1686 patients using ramipril, of which... (truncated)

Title: Ramipril-associated hepatotoxicity.
Angiotensin-converting enzyme inhibitors are prescribed for many cardiovascular and renal diseases. Adverse hepatic events, especially cholestasis, have rarely been reported with captopril, enalapril, lisinopril, and fosinopril. To date, hepatic injury associated with ramipril has not been reported.

Title: Effect of Candesartan and Ramipril on Liver Fibrosis in Patients with Chronic Hepatitis C Viral Infection: A Randomized Controlled Prospective Study.
Objective: This study aimed at evaluating the effects of candesartan and ramipril on liver fibrosis in patients with chronic hepatitis C. Methods: This randomized controlled prospective study involved 64 patients with chronic hepatitis C and liver fibrosis. Participants were randomized into 3 groups: group I (control group; n = 21), members of which received traditional therapy only; group 2 (ramipril group; n = 21), members of which received traditional therapy plus 1.25 mg/d oral ramipril; and group 3 (candesartan group; n = 22), members of which received traditional therapy plus 8 mg/d oral candesartan. Patients were assessed at baseline and 6 months after intervention through measuring... (truncated)

MESH:D000068878 - trastuzumab

Summary:

LLM Prediction Score	0.445 (normalized)
LLM Confidence Score	0.990
Golden Answer (Severity Class)	0.0 (normalized)
Prediction Error	0.445

Retrieved Context:

Title: Safety and Efficacy of Trastuzumab Emtansine in Advanced Human Epidermal Growth Factor Receptor 2-Positive Breast Cancer: a Meta-analysis.
Advanced or metastatic breast cancer is an incurable disease with high mortality rate worldwide and about 20% of breast cancers overexpress and amplify the human epidermal growth factor receptor 2 (HER2). Achievements in targeted therapy have benefited people during the past decades. Trastuzumab emtansine (T-DM1), a novel antibody-drug conjugate playing a powerful role in anti-tumor activity, not only blocks the HER2 signaling pathways, but also disturbs the microtubule dynamics. To access the efficacy and safety of T-DM1, we analyzed 9 clinical trials on T-DM1. Results showed that fatigue (0.604, 95% CI 0.551, 0.654), nausea (0.450, 95% CI 0.365, 0.537), increased... (truncated)

Title: Dose-reduced trastuzumab emtansine: active and safe in acute hepatic dysfunction.
Breast cancer is the most common cancer in women worldwide. The majority of deaths attributed to breast cancer are a result of metastatic disease, and 30% of early breast cancers (EBC) will develop distant disease. The 5-year survival of patients with metastatic disease is estimated at 23%. Breast cancer subtypes continue to be stratified histologically on oestrogen, progesterone and human epidermal growth factor-2 (HER2) receptor expression. HER2-positive breast cancers represent 25% of all breast cancer diagnoses. The therapies available for metastatic breast cancer (MBC) are expanding, in particular within the field of HER2-positive disease, with the approval of trastuzumab, pertuzumab,... (truncated)

Title: Metastatic human epidermal growth factor 2 (HER2/neu) amplified breast cancer with acute fulminant hepatitis responding to trastuzumab, pertuzumab and carboplatin.
A 30-year-old woman presented to an outside hospital with pain in the right upper abdomen. Imaging revealed over 100 liver lesions, the largest measuring 74 mm×71 mm, and multiple lytic bone lesions. An outpatient liver biopsy showed a poorly differentiated adenocarcinoma favouring a breast primary. The tumour was oestrogen and progesterone receptor negative, but human epidermal growth factor 2 (HER2/neu) amplified. In her second clinic visit she had decompensated liver failure manifested by new-onset ascites and jaundice. Initially, the chemotherapy plan was for docetaxel, pertuzumab and trastuzumab, but given her severe liver dysfunction we used a combination of carboplatin, pertuzumab... (truncated)

Title: Hepatotoxicity induced by trastuzumab used for breast cancer adjuvant therapy: a case report.
Trastuzumab is generally considered a highly safe drug, but there have been cases of infusion reaction and cardiotoxicity. This report will present a rare case of hepatotoxicity induced by trastuzumab used for adjuvant therapy of human epidermal growth factor receptor type 2-positive breast cancer.

Title: Trastuzumab-induced hepatotoxicity.
To report a case of probable trastuzumab-induced hepatotoxicity.

MESH:C413685 - entecavir

Summary:

LLM Prediction Score	0.430 (normalized)
LLM Confidence Score	0.990
Golden Answer (Severity Class)	0.875 (normalized)
Prediction Error	0.445

Retrieved Context:

Title: Adverse effects of oral antiviral therapy in chronic hepatitis B.
Oral nucleoside/nucleotide analogues (NAs) are currently the backbone of chronic hepatitis B (CHB) infection treatment. They are generally well-tolerated by patients and safe to use. To date, a significant number of patients have been treated with NAs. Safety data has accumulated over the years. The aim of this article is to review and update the adverse effects of oral NAs. NAs can cause class adverse effects (<i>i.e</i>., myopathy, neuropathy, lactic acidosis) and dissimilar adverse effects. All NAs carry a "Black Box" warning because of the potential risk for mitochondrial dysfunction. However, these adverse effects are rarely reported. The majority of... (truncated)

Title: Entecavir: new drug. Chronic hepatitis B: a last resort.
(1) For patients with HBeAg-positive chronic hepatitis B, peginterferon alfa-2a is the standard treatment used to prevent clinical complications and death. Lamivudine and adefovir dipivoxil, both taken orally, are second-line options. They can also be used as first-line treatments in patients with HBeAg-negative chronic hepatitis B if the adverse effects of prolonged peginterferon therapy are likely to pose a major problem. (2) Entecavir, a nucleoside analogue, is now marketed for oral treatment of chronic hepatitis B in adults. (3) Entecavir has not been compared with adefovir in clinical trials. Its evaluation is based mainly on three 48-week trials versus lamivudine.... (truncated)

Title: [A case of drug-induced liver injury caused by entecavir for treatment of hepatitis B virus reactivation during RCHOP in a patient with non-Hodgkin lymphoma].
A 49-year-old-man, a healthy carrier of hepatitis B virus (HBV), received chemotherapy with a rituximab/cyclo-phosphamide/doxorubicin/vincristine/prednisolone (R-CHOP) regimen for non-Hodgkin's lymphoma. At the first course of chemotherapy, not only the liver function but the HBV DNA level was elevated. These symptoms were diagnosed as hepatic injury induced by HBV reactivation, and, therefore, entecavir (ETV) was started. As a result, although the treatment with ETV decreased the HBV DNA level, liver function values were remarkably elevated again (over 3 times the levels before beginning ETV). ETV was discontinued because of suspicion regarding the onset of hepatic injury it caused. After switching... (truncated)

Title: Entecavir is a potent anti-HBV drug superior to lamivudine: experience from clinical trials in China.
Infection with the hepatitis B virus (HBV) can result in chronic hepatitis B (CHB) in many patients. Patients with CHB require regular screening and monitoring to facilitate disease surveillance and to determine if/when treatment is indicated. The current goal of CHB treatment is sustained viral suppression with the aim of reducing or preventing hepatic injury and disease progression. Effective anti-HBV therapy is now available that can suppress, but not eradicate, HBV replication. Among the currently licensed and approved anti-HBV nucleos(t)ides, entecavir demonstrates a potent anti-HBV activity and a low rate of emergence of drug resistance, with good safety and tolerability... (truncated)

Title: [Impending liver failure after chemoimmunotherapy-induced reactivation of hepatitis B - successful treatment with entecavir].
An 83-year old woman had been treated with bendamustin and rituximab for prolymphocytic leukemia. Two weeks after cycle 6 of chemotherapy, signs and symptoms of a severe hepatitis occurred.

MESH:D000069583 - pregabalin

Summary:

LLM Prediction Score	0.443 (normalized)
LLM Confidence Score	0.990
Golden Answer (Severity Class)	0.0 (normalized)
Prediction Error	0.443

Retrieved Context:

Title: Antiepileptic Drugs and Liver Disease.
Acute, symptomatic seizures or epilepsy may complicate the course of hepatic disease. Choosing the most appropriate antiepileptic drug in this setting represents a difficult challenge, as most medications are metabolized by the liver. This article focuses on the acute and chronic treatment of seizures in patients with advanced liver disease and reviews the hepatotoxic potential of specific antiepileptic drugs. Newer antiepileptic drugs without, or with minimal, hepatic metabolism, such as levetiracetam, lacosamide, topiramate, gabapentin, and pregabalin should be used as first-line therapy. Medications undergoing extensive hepatic metabolism, such as valproic acid, phenytoin, and felbamate should be used as drugs of... (truncated)

Title: [Pregabalin as a rare cause of liver disease].
In this report we describe a patient who developed liver failure due to new administration of pregabaline.

Title: Use of antiepileptic drugs in hepatic and renal disease.
The use of antiepileptic drugs in patients with renal or hepatic disease is common in clinical practice. Since the liver and kidney are the main organs involved in the elimination of most drugs, their dysfunction can have important effects on the disposition of antiepileptic drugs. Renal or hepatic disease can prolong the elimination of the parent drug or an active metabolite leading to accumulation and clinical toxicity. It can also affect the protein binding, distribution, and metabolism of a drug. The protein binding of anionic acidic drugs, such as phenytoin and valproate, can be reduced significantly by renal failure, causing... (truncated)

Title: Pregabalin-induced hepatotoxicity.
To report a case of acute elevation of hepatic enzyme levels as a probable adverse reaction associated with pregabalin.

Title: Acetaminophen: A Liver Killer or Thriller.
Acetaminophen, or paracetamol, ranks among the most extensively utilized analgesic and antipyretic medications globally. The administration of acetaminophen to individuals with underlying liver disease has long sparked concerns regarding the potential risk of hepatotoxicity. However, the available literature and recommendations consider it a safe option in all forms of liver diseases and is deemed safe when used at recommended doses. This article aims to offer a concise review of the pharmacokinetics, toxicity profile, and the intricate considerations surrounding the safety of acetaminophen in patients with liver disease. By delving into the liver-acetaminophen interactions, we seek to provide a nuanced perspective... (truncated)

MESH:D019438 - ritonavir

Summary:

LLM Prediction Score	0.560 (normalized)
LLM Confidence Score	0.980
Golden Answer (Severity Class)	1.0 (normalized)
Prediction Error	0.440

Retrieved Context:

Title: Acute hepatitis and bleeding possibly induced by zidovudine and ritonavir in an infant with HIV infection.
Acute hepatitis led to abnormal coagulopathy, bleeding, and death in a nonhemophiliac infant infected with the human immunodeficiency virus, possibly due to zidovudine or ritonavir or both. Acute hepatitis during ritonavir treatment and episodes of spontaneous bleeding have been reported in patients with hemophilia. Zidovudine is associated with elevated liver enzymes, elevated bilirubin, and hepatomegaly leading to abnormal coagulopathy, bleeding, and death in adults. A temporal relationship between the start of combination antiretroviral therapy and onset of hepatosplenomegaly and rise in liver enzymes suggests that zidovudine or ritonavir, or both, are the likely cause of this adverse event. Ritonavir is... (truncated)

Title: Low rate of adverse hepatic events associated with fosamprenavir/ritonavir-based antiretroviral regimens.
To appraise the incidence of liver toxicity in a population of patients receiving fosamprenavir/ritonavir (FPV/r) with a high frequency of viral hepatitis co-infection.

Title: Liver toxicity associated with antiretroviral therapy including efavirenz or ritonavir-boosted protease inhibitors in a cohort of HIV/hepatitis C virus co-infected patients.
To compare the frequency of grade 3 or 4 transaminase elevations (TEs) in HIV/hepatitis C virus (HCV) co-infected patients who started a three-antiretroviral drug regimen including efavirenz or a ritonavir-boosted protease inhibitor (PI/r) and the influence of pre-existing significant hepatic fibrosis or cirrhosis.

Title: [Antiretroviral agents in HIV-infected patients with cirrhosis].
Since highly active antiretroviral therapies became available, the future of HIV-infected patients has been transformed. However, 20 to 25% of HIV patients are co-infected with hepatitis B or C viruses, and the course of these diseases has worsened, since these patients have an enhanced sensitivity to the hepatic toxicity of antiretrovirals. The relation between high antiretroviral concentrations and toxicity has been clearly demonstrated with certain protease inhibitors and non-nucleoside reverse transcriptase inhibitors (NNRTI) that have a predominantly hepatic metabolism (CYP4503A4). The nucleoside reverse transcriptase inhibitors (NRTI) are not predominantly metabolized by the liver, but may nevertheless be toxic for the... (truncated)

Title: Liver toxicity of antiretroviral combinations including atazanavir/ritonavir in patients co-infected with HIV and hepatitis viruses: impact of pre-existing liver fibrosis.
To appraise the rate of grade 3-4 transaminase elevations (TEs) and grade 4 total bilirubin elevation (TBE) in patients co-infected with human immunodeficiency virus (HIV) and hepatitis C or hepatitis B virus (HCV or HBV, respectively) who receive atazanavir/ritonavir. Moreover, the relationship between these events and the degree of prior liver fibrosis was evaluated.

MESH:D014801 - vitamin a

Summary:

LLM Prediction Score	0.815 (normalized)
LLM Confidence Score	0.990
Golden Answer (Severity Class)	0.375 (normalized)
Prediction Error	0.440

Retrieved Context:

Title: Vitamin A toxicity: when one a day doesn't keep the doctor away.
Vitamin A toxicity has been reported to cause severe liver disease and, occasionally, liver failure. Herein we present the case of a 60-year-old male with symptoms of muscle soreness, alopecia, nail dystrophy, and ascites. He continued to deteriorate with the development of refractory ascites, renal insufficiency, encephalopathy, and failure to thrive. A liver biopsy demonstrated presence of Ito cells and vacuolated Kupffer cells without the presence of cirrhosis. His clinical history revealed ingestion of large doses of vitamin A. His worsening clinical situation ruled out the possibility of a transjugular intrahepatic portosystemic shunt. The patient underwent orthotopic liver transplantation with... (truncated)

Title: Vitamin A-induced cholestatic hepatitis: a case report.
We report a case of intrahepatic cholestasis due to chronic vitamin A supplementation. A 70-year-old woman was admitted to the hospital for jaundice and reduced nutritional and general status with a 2-month history of increasing cholestasis. Some years previously she had suffered from breast and ovarian cancer with subsequent surgery and chemotherapy. Chemotherapy was terminated one month before elevated serum transaminase activities and cholestatic serum markers were noted. Following the chemotherapy, supportive care included weekly vitamin A injections (100,000 IU per injection). Liver biopsy showed an acute toxic liver injury with focal parenchymal necrosis, sinusoidal lesions, inflammatory infiltrate (round cells,... (truncated)

Title: Liver toxicity from vitamin A.
Hepatic toxicity secondary to hypervitaminosis A is extremely rare. We report the case of a 27-year-old Caucasian female who presented for an investigation of abdominal pain, cholestatic liver function tests, and abnormal computerized tomography findings. She had been prescribed isotretinoin for her acne and had subsequently purchased vitamin A online, which she consumed daily for over 18 months.

Title: Short-term vitamin A supplementation at therapeutic doses induces a pro-oxidative state in the hepatic environment and facilitates calcium-ion-induced oxidative stress in rat liver mitochondria independently from permeability transition pore formation : detrimental effects of vitamin A supplementation on rat liver redox and bioenergetic states homeostasis.
There is a growing body of evidence showing that vitamin A induces toxic effects in several experimental models and in human beings. In the present work, we have investigated the effects of short-term vitamin A supplementation on the adult rat liver redox status. We have found that vitamin A at therapeutic doses induces a hepatic oxidative insult. Furthermore, we have observed increased antioxidant enzyme activity in the liver of vitamin-A-treated rats. Additionally, some mitochondrial dysfunction was found since superoxide anion production was increased in vitamin-A-treated rat liver submitochondrial particles, which may be the result of impaired mitochondrial electron transfer chain... (truncated)

Title: Evaluation of vitamin A toxicity.
Toxicity has been associated with abuse of vitamin A supplements and with diets extremely high in preformed vitamin A. Consumption of 25,000-50,000 IU/d for periods of several months or more can produce multiple adverse effects. The lowest reported intakes causing toxicity have occurred in persons with liver function compromised by drugs, viral hepatitis, or protein-energy malnutrition. Certain drugs or other chemicals may markedly potentiate vitamin A toxicity in animals. Especially vulnerable groups include children, with adverse effects occurring with intakes as low as 1,500 IU.kg-1.d-1, and pregnant women, with birth defects being associated with maternal intakes as low as approximately... (truncated)

MESH:D005424 - flecainide

Summary:

LLM Prediction Score	0.436 (normalized)
LLM Confidence Score	0.970
Golden Answer (Severity Class)	0.875 (normalized)
Prediction Error	0.439

Retrieved Context:

Title: [Flecainide-induced hepatitis].
Flecainide was given to a patient in a dose of 150 mg twice daily to convert a newly developed atrial fibrillation; concomitant therapy was unchanged. After the fourth dose the patient complained of upper abdominal pain and nausea. GOT and GPT, normal at admission to the hospital, became markedly elevated and reached a maximum of 960 IU/l (GOT) and 993 IU/l (GPT) one day later, although the enzymes which indicate cholestasis remained at a normal level or did not increase. On the assumption of a drug-induced allergic reaction, flecainide was withdrawn, after which liver enzymes rapidly returned to control values.... (truncated)

Title: Highlights of the 22nd French pharmacovigilance meeting.
(1) The 22nd French pharmacovigilance meeting, held in July 2001, presented data on adverse effects notified by health professionals to regional pharmacovigilance centres in France. (2) Non specific "immunostimulants" are not harmless placebos, as might be concluded from the relative lack of data. There have been 315 notifications of severe adverse effects, some with positive rechallenge, reporting cutaneous, gastrointestinal, respiratory, haematological and other disorders. Attributability was considered "likely" in 68% of cases. Three deaths occurred. (3) Other well known adverse effects continue to occur: convulsions with camphor, visual hallucinations with oxybutynin, headache with antimigraine drugs, liver damage with dextropropoxyphene, neuropsychological... (truncated)

Title: Narrow therapeutic index drugs: a clinical pharmacological consideration to flecainide.
The therapeutic index (TI) is the range of doses at which a medication is effective without unacceptable adverse events. Drugs with a narrow TI (NTIDs) have a narrow window between their effective doses and those at which they produce adverse toxic effects. Generic drugs may be substituted for brand-name drugs provided that they meet the recommended bioequivalence (BE) limits. However, an appropriate range of BE for NTIDs is essential to define due to the potential for ineffectiveness or adverse events. Flecainide is an antiarrhythmic agent that has the potential to be considered an NTID. This review aims to evaluate the... (truncated)

Title: Acute lung affection in an endurance-trained man under amiodarone medication.
Patients undergoing treatment with amiodarone can develop severe pulmonary side effects. This effect, which is often highly underestimated, can lead to dyspnea, pneumonitis, and further fibrosis. A recent change in the labeling of amdiodarone by the American Food and Drug Administration (FDA) supports this suspicion. Tracing the symptoms back to the causing agent can be difficult, as shown in our report. The subject of this case report is an endurance-trained 65 year old male marathon runner who appeared with atrial fibrillation during a routine check up in autumn 2003. After medical cardioversion with flecainide a complaint free interval of 8... (truncated)

Title: Flecainide-induced pneumonitis: a case report.
We report a case of acute respiratory distress associated with a histological pattern of acute fibrinous and organizing pneumonia, and discuss the possible responsibility of flecainide therapy.

MESH:D000628 - aminophylline

Summary:

LLM Prediction Score	0.437 (normalized)
LLM Confidence Score	0.980
Golden Answer (Severity Class)	0.0 (normalized)
Prediction Error	0.437

Retrieved Context:

Title: Theophylline prescribing, serum concentrations, and toxicity.
Slow-release theophylline preparations have been prescribed increasingly at the Brompton Hospital, and a serum theophylline assay is requested about once for every ten prescriptions. In a period of eighteen months 1913 such assays were performed, and their influence on management was assessed by retrospective analysis in two groups--113 outpatients on stable dosage with no recent exacerbations of disease; and 28 inpatients and outpatients with concentrations exceeding 25 mg/l. In those with stable treatment, there was only a loose relation between dose and serum theophylline level. High serum levels were associated with abnormal liver function, diuretic use, and duplicate prescribing. When... (truncated)

Title: Metabolic basis for high paracetamol dosage without hepatic injury: a case study.
1. Studies of paracetamol metabolism were performed in a 58-year-old female with rheumatoid arthritis who had consumed 15-20 g paracetamol daily for 5 years without developing liver damage and data were compared with results in seven normal volunteers. 2. After a test dose of 2 g paracetamol, the formation of paracetamol sulphate and glucuronide conjugates detected in plasma from the patient was delayed by around 2 h relative to values in normal volunteers and the proportion of sulphate conjugates excreted in urine was 1.5 to 2 times those in normal volunteers (52% vs 26-35% of dose, respectively). The fractional metabolite... (truncated)

Title: Theophylline.
Theophylline (3-methyxanthine) has been used to treat airway diseases for over 70 years. It was originally used as a bronchodilator but the relatively high doses required are associated with frequent side effects, so its use declined as inhaled β_2-agonists became more widely used. More recently it has been shown to have anti-inflammatory effects in asthma and COPD at lower concentrations. The molecular mechanism of bronchodilatation is inhibition of phosphodiesterase(PDE)3 and PDE4, but the anti-inflammatory effect may be due to histone deacetylase (HDAC) activation, resulting in switching off of activated inflammatory genes. Through this mechanism theophylline also reverses corticosteroid resistance and... (truncated)

Title: Hepatic function after porto-systemic shunt.
Advanced hepatic injury can be identified by the appearance of jaundice, coagulopathy, or encephalopathy but these conditions are late, irreversible findings and represent the end stage of a long insidious process. Currently available methods for assessing "liver function" (SGOT, SGPT, GGT, LDH, etc.) do not actually measure liver function. In this study we prospectively evaluated "true" liver function in patients undergoing porto-systemic shunt. Effective hepatic blood flow [low dose galactose clearance (EHBF)], hepatocyte transport (theophylline levels at 24 hr), and hepatic conjugation ability [acetaminophen metabolism to its glucuronide and sulfate conjugates ((S + G)/A) and acetaminophen remaining at 24... (truncated)

Title: Pharmacokinetics of theophylline in hepatic disease.
The disposition of theophylline was examined in eight male cirrhotic (six proven by biopsy) patients without heart failure. An oral dose of 100 mg of theophylline per square meter of surface area was administered, and samples of serum and saliva were collected from 0 to 60 hours and were assayed by high-pressure liquid chromatographic techniques. Controls were 57 young normal subjects and 25 age-matched patients. The body clearance of theophylline in cirrhotic patients was low, averaging 18.8 +/- 11.3 ml/kg/hr (+/- SD) vs 53.7 +/- 19.3 and 63.0 +/- 28.5 ml/kg/hr in the control patients and the normal subjects, respectively.... (truncated)

MESH:C101207 - darifenacin

Summary:

LLM Prediction Score	0.437 (normalized)
LLM Confidence Score	0.970
Golden Answer (Severity Class)	0.0 (normalized)
Prediction Error	0.437

Retrieved Context:

Title: Treatment of overactive bladder in the aging population: focus on darifenacin.
Anticholinergics are commonly used in primary and secondary care settings for the treatment of overactive bladder syndrome. The number of anticholinergic drugs available on the market is increasing and various studies, both observational and randomized controlled trials, have evaluated effectiveness of the different preparations available. When anticholinergic therapy is prescribed, there is still uncertainty about which anticholinergic drugs are most effective, at which dose, and by which route of administration. There is also uncertainty about the role of anticholinergic drugs in different patient groups, particularly in the elderly. The rationale for using anticholinergic drugs in the treatment of overactive bladder... (truncated)

Title: Effects of modulating M3 muscarinic receptor activity on azoxymethane-induced liver injury in mice.
Previously, we reported that azoxymethane (AOM)-induced liver injury is robustly exacerbated in M3 muscarinic receptor (M3R)-deficient mice. We used the same mouse model to test the hypothesis that selective pharmacological modulation of M3R activity regulates the liver injury response. Initial experiments confirmed that giving a selective M3R antagonist, darifenacin, to AOM-treated mice mimicked M3R gene ablation. Compared to vehicle controls, mice treated with the M3R antagonist had reduced survival and increased liver nodularity and fibrosis. We next assessed AOM-induced liver injury in mice treated with a selective M3R agonist, pilocarpine. After pilocarpine treatment, stimulation of post-M3R signaling in the liver... (truncated)

Title: Targeting acetylcholine signaling modulates persistent drug tolerance in EGFR-mutant lung cancer and impedes tumor relapse.
Although first-line epidermal growth factor receptor (EGFR) tyrosine kinase inhibitor (TKI) therapy is effective for treating EGFR-mutant non-small cell lung cancer (NSCLC), it is now understood that drug-tolerant persister (DTP) cells escaping from initial treatment eventually drives drug resistance. Here, through integration of metabolomics and transcriptomics, we found that the neurotransmitter acetylcholine (ACh) was specifically accumulated in DTP cells, and demonstrated that treatment with EGFR-TKI heightened the expression of the rate-limiting enzyme choline acetyltransferase (ChAT) in ACh biosynthesis via YAP mediation. Genetic and pharmacological manipulation of ACh biosynthesis or ACh signaling could predictably regulate the extent of DTP formation in... (truncated)

Title: Pharmacotherapy of overactive bladder in adults: a review of efficacy, tolerability, and quality of life.
Overactive bladder (OAB) is a prevalent condition that has a significant impact on quality of life. The usual treatment approach is both behavioral and pharmacological. The first-line pharmacological treatment commonly utilizes anticholinergic agents, which may be limited by their tolerability, efficacy, and long-term compliance. Developments in elucidating the pathophysiology of OAB and alternative targets for pharmacological therapy have led to newer agents being developed to manage this condition. These agents include mirabegron and botulinum toxin, which have alternate mechanism of action and avoid the anticholinergic side effects.

Title: Evaluation of Clinically Relevant Drug-Drug Interactions and Population Pharmacokinetics of Darolutamide in Patients with Nonmetastatic Castration-Resistant Prostate Cancer: Results of Pre-Specified and Post Hoc Analyses of the Phase III ARAMIS Trial.
Darolutamide, an androgen receptor antagonist with a distinct molecular structure, significantly prolonged metastasis-free survival versus placebo in the phase III ARAMIS study in men with nonmetastatic castration-resistant prostate cancer (nmCRPC). In this population, polypharmacy for age-related comorbidities is common and may increase drug-drug interaction (DDI) risks. Preclinical/phase I study data suggest darolutamide has a low DDI potential-other than breast cancer resistance protein/organic anion transporter protein substrates (e.g., statins), no clinically relevant effect on comedications is expected.

MESH:D010098 - oxycodone

Summary:

LLM Prediction Score	0.435 (normalized)
LLM Confidence Score	0.990
Golden Answer (Severity Class)	0.0 (normalized)
Prediction Error	0.435

Retrieved Context:

Title: Cholestatic hepatitis as a possible new side-effect of oxycodone: a case report.
Oxycodone is a widely-used semisynthetic opioid analgesic that has been used for over eighty years. Oxycodone is known to cause side effects such as nausea, pruritus, dizziness, constipation and somnolence. As far as we are aware cholestatic hepatitis as a result of oxycodone use has not been reported so far in the world literature.

Title: [Oxycodone hepatotoxicity in a patient with non-alcoholic fatty liver disease without fibrosis].
No abstract available.

Title: Limited Knowledge of Acetaminophen in Patients with Liver Disease.
<i>Background and Aims:</i> Unintentional acetaminophen overdose remains the leading cause of acute liver failure in the United States. Patients with underlying liver disease are at higher risk of poor outcomes from acetaminophen overdose. Limited knowledge of acetaminophen may be a preventable contributor to elevated rates of overdose and thus acute liver failure. The purpose of this study is to assess knowledge of acetaminophen dosing and presence of acetaminophen in common combination products in patients with liver disease. <i>Methods:</i> We performed a cross-sectional study of patients with liver disease at the Pfleger Liver Institute at the University of California, Los Angeles... (truncated)

Title: Acute Hepatocellular Drug-Induced Liver Injury From Bupropion and Doxycycline.
The management and diagnosis of drug-induced liver injury (DILI) is often challenging, particularly when patients are taking multiple medications. We present a 29-year-old African American man who presented with jaundice and malaise after starting bupropion and doxycycline 2 weeks prior. He was found to have acute hepatocellular drug-induced liver injury with autoimmune features, and made a complete recovery with prednisone. Although bupropion and doxycycline are both known to cause liver toxicity, a closer inspection of the signature of liver injury and a review of prior related DILI cases assigns causality more to bupropion than doxycycline.

Title: Analgesics in patients with hepatic impairment: pharmacology and clinical implications.
The physiological changes that accompany hepatic impairment alter drug disposition. Porto-systemic shunting might decrease the first-pass metabolism of a drug and lead to increased oral bioavailability of highly extracted drugs. Distribution can also be altered as a result of impaired production of drug-binding proteins or changes in body composition. Furthermore, the activity and capacity of hepatic drug metabolizing enzymes might be affected to various degrees in patients with chronic liver disease. These changes would result in increased concentrations and reduced plasma clearance of drugs, which is often difficult to predict. The pharmacology of analgesics is also altered in liver disease.... (truncated)

MESH:C005419 - oxybutynin

Summary:

LLM Prediction Score	0.434 (normalized)
LLM Confidence Score	0.990
Golden Answer (Severity Class)	0.0 (normalized)
Prediction Error	0.434

Retrieved Context:

Title: Highlights of the 22nd French pharmacovigilance meeting.
(1) The 22nd French pharmacovigilance meeting, held in July 2001, presented data on adverse effects notified by health professionals to regional pharmacovigilance centres in France. (2) Non specific "immunostimulants" are not harmless placebos, as might be concluded from the relative lack of data. There have been 315 notifications of severe adverse effects, some with positive rechallenge, reporting cutaneous, gastrointestinal, respiratory, haematological and other disorders. Attributability was considered "likely" in 68% of cases. Three deaths occurred. (3) Other well known adverse effects continue to occur: convulsions with camphor, visual hallucinations with oxybutynin, headache with antimigraine drugs, liver damage with dextropropoxyphene, neuropsychological... (truncated)

Title: Managing the adverse events of intravesical bacillus Calmette-Guérin therapy.
This paper provides recommendations on the management of complications arising from intravesical treatment with bacillus Calmette-Guérin (BCG) for nonmuscle-invasive bladder tumors. There is minimal recommendations currently available as randomized trials on the side effects of intravesical BCG are lacking and severe complications are usually described in case reports only. All physicians giving intravesical BCG should be aware of the possible complications that could arise and how to treat these. The incidence of bladder irritation, general malaise, and fever is very high, while severe complications remain rare. Approximately 8% of patients have to stop treatment because of these complications. BCG infections... (truncated)

Title: A case of probable oxybutynin-induced increase in liver enzymes.
We describe the case of a 49-year-old male who presented to the emergency department with right-sided weakness and inability to speak. He was diagnosed with stroke and was admitted to Qatar Rehabilitation Institute after he was treated for the acute phase at Hamad General Hospital. As part of his management, he was started on oxybutynin 5 mg orally twice daily for the treatment of overactive bladder. Within a week, his liver enzymes started to increase. After a thorough medication review, oxybutynin was suspended as it was the only suspected medication to be responsible of this elevation in liver enzymes. When... (truncated)

Title: Utility of spherical human liver microtissues for prediction of clinical drug-induced liver injury.
Drug-induced liver injury (DILI) continues to be a major source of clinical attrition, precautionary warnings, and post-market withdrawal of drugs. Accordingly, there is a need for more predictive tools to assess hepatotoxicity risk in drug discovery. Three-dimensional (3D) spheroid hepatic cultures have emerged as promising tools to assess mechanisms of hepatotoxicity, as they demonstrate enhanced liver phenotype, metabolic activity, and stability in culture not attainable with conventional two-dimensional hepatic models. Increased sensitivity of these models to drug-induced cytotoxicity has been demonstrated with relatively small panels of hepatotoxicants. However, a comprehensive evaluation of these models is lacking. Here, the predictive value... (truncated)

Title: Drug Induced Liver Injury Attributed to a Curcumin Supplement.
More severe reactions, higher acute liver failure rates, and higher recurrence rates on re-challenge occur with supplement-related Drug Induced Liver Injury (DILI) (Medina-Caliz et al., 2018). We report a case of curcumin-induced hepatocellular DILI in a 78-year old female admitted with jaundice, with a one-month latency. Extensive evaluation for alternative etiologies of hepatotoxicity was unremarkable. The Roussel Uclaf Causality Assessment Method (RUCAM) score of 6 for the supplement indicated a probable association (score >8: highly probable association). Peak levels of aspartate aminotransferase (AST) and alanine aminotransferase (ALT) were >20 times upper limit of normal. A 48% decrease in AST and... (truncated)

MESH:C004649 - carbinoxamine

Summary:

LLM Prediction Score	0.434 (normalized)
LLM Confidence Score	0.940
Golden Answer (Severity Class)	0.0 (normalized)
Prediction Error	0.434

Retrieved Context:

Title: A case of cholestatic hepatitis associated with histologic features of acute cholangitis.
This report describes a case showing histologic features of acute cholangitis with an over-the-counter drug. A 48-year-old woman was diagnosed with general malaise and progressive jaundice. A thorough review of her medical history revealed that the patient had taken an over-the-counter drug, Pabron Gold(®), which she had used previously, that may have caused liver injury. Laboratory investigations revealed jaundice and liver dysfunction. Endoscopic retrograde cholangiography detected no extrahepatic biliary duct dilatation or stones. Liver biopsy indicated acute cholangitis involving neutrophils and eosinophils. Electron microscopy revealed fragmented nuclei, indicating that the degenerative bile duct-related epithelial cells were in an apoptotic process.

Title: Delayed increase in serum acetaminophen concentration after ingestion of a combination medications: a case report.
Acetaminophen is absorbed rapidly after oral intake, and serum concentration peaks within 4 hours. The Rumack-Matthew (RM) nomogram is widely used to identify the potential risk of liver dysfunction. However, the RM nomogram was intended for use only when a single agent was ingested. We report the case of a patient with overdose ingestion of an over-the-counter combination cold medication that contained acetaminophen, where the patient's serum concentration increased over time. Over-the-counter combination cold medications are designed to relieve cold symptoms. However, the possibility that other agents that were present in the drug may change gastrointestinal kinetics should also be... (truncated)

Title: Prevalence and factors associated with potentially inappropriate medication use in older medicare beneficiaries with cancer.
To assess the factors related to potentially inappropriate medication (PIM) use in elderly patients with cancer, as well as to compare the PIM prevalence in older adults with and without cancer.

Title: Antipsychotic Drugs Efficacy in Dextromethorphan-Induced Psychosis.
Psychosis is known as a broad term of symptoms that cause serious disorganization of behavior, thinking, and perception of reality. One of the medicines that recently gained much attention in terms of its psychotic potential is dextromethorphan (DXM). DXM, a widely used antitussive drug, is a commonly abused drug because of its euphoric, hallucinogenic, and dissociative properties. To date, DXM is a legally marketed cough suppressant that is neither a controlled substance nor a regulated chemical under the Controlled Substances Act. The management of DXM-related psychosis is dependent on the type of psychotic symptoms. Atypical neuroleptics (i.e., olanzapine, risperidone, quetiapine)... (truncated)

Title: Increased risk of death with codeine use in the elderly over 85 years old and patients with respiratory disease: A case-control study using retrospective insurance claims database.
To investigate the risk of mortality associated with exposure to codeine, considering various risk groups, using population-based national insurance claims data.National sample cohort data from the National Health Insurance Service of South Korea (2002-2013) was used in this case-control study. Cases were defined as patients with a death record between January 1, 2002 and December 31, 2013. Each case was matched to 10 controls based on age, sex, baseline comorbidities, and year of death. Definition of exposure was codeine prescription in 30 days prior to death and sensitivity analyses were performed for 15 and 60-day exposures. Adjusted odds ratios (aORs)... (truncated)

MESH:D000077210 - sunitinib

Summary:

LLM Prediction Score	0.566 (normalized)
LLM Confidence Score	0.990
Golden Answer (Severity Class)	1.0 (normalized)
Prediction Error	0.434

Retrieved Context:

Title: Severe tyrosine-kinase inhibitor induced liver injury in metastatic renal cell carcinoma patients: two case reports assessed for causality using the updated RUCAM and review of the literature.
Sunitinib and pazopanib are both oral small molecule multityrosine kinase inhibitors (MTKI) used in the treatment of renal cell carcinoma (RCC). Hepatotoxicity or "liver injury" is the most important adverse effect of pazopanib administration, but little is known about the underlying mechanism. Liver injury may also occur in patients treated with sunitinib, but severe toxicity is extremely rare. Herein we report two new cases of severe liver injury induced by MTKI. Both cases are unique and exceptional. We assessed both cases for drug-induced liver injury (DILI) using the updated score Roussel Uclaf causality assessment method (RUCAM). The literature on potential... (truncated)

Title: Sunitinib induces hepatocyte mitochondrial damage and apoptosis in mice.
Reports concerning hepatic mitochondrial toxicity of sunitinib are conflicting. We therefore decided to conduct a toxicological study in mice. After having determined the highest dose that did not affect nutrient ingestion and body weight, we treated mice orally with sunitinib (7.5 mg/kg/day) for 2 weeks. At the end of treatment, peak sunitinib plasma concentrations were comparable to those achieved in humans and liver concentrations were approximately 25-fold higher than in plasma. Sunitinib did not affect body weight, but increased plasma ALT activity 6-fold. The activity of enzyme complexes of the electron transport chain (ETC) was decreased numerically in freshly isolated... (truncated)

Title: Sunitinib induced hepatotoxicity in L02 cells <i>via</i> ROS-MAPKs signaling pathway.
Sunitinib is a multi-targeted tyrosine kinase inhibitor with remarkable anticancer activity, while hepatotoxicity is a potentially fatal adverse effect of its administration. The aim of this study was to elucidate the mechanism of hepatotoxicity induced by Sunitinib and the protective effect of glycyrrhetinic acid (GA). Sunitinib significantly reduced the survival of human normal hepatocytes (L02 cells), induced the increase of alanine aminotransferase (ALT), aspartate aminotransferase (AST) and lactate dehydrogenase (LDH). Chloroquine (CQ) and Z-VAD-FMK were applied to clarify the cell death patterns induced by Sunitinib. Sunitinib significantly induced L02 cells death by triggering apoptosis and autophagy acted as a self-defense... (truncated)

Title: Sunitinib-Induced Acute Liver Failure.
Drug-induced liver injury is an uncommon but life-threatening entity. Sunitinib is a tyrosine kinase inhibitor used for advanced and imatinib-refractory gastrointestinal stromal tumors. It causes transient elevation in liver enzymes. The incidence of fatal acute liver failure is rare. Five cases of sunitinib-induced acute liver injury have been reported in the literature thus far. We present a case of fatal acute liver failure and cardiomyopathy within 2 weeks of sunitinib therapy initiation for advanced pancreatic neuroendocrine carcinoma. We believe our case is unique due to the rarity of its presentation. It highlights hepatotoxicity as a potentially fatal side effect of... (truncated)

Title: Hepatotoxicity of tyrosine kinase inhibitors: clinical and regulatory perspectives.
The introduction of small-molecule tyrosine kinase inhibitors (TKIs) in clinical oncology has transformed the treatment of certain forms of cancers. As of 31 March 2013, 18 such agents have been approved by the US Food and Drug Administration (FDA), 15 of these also by the European Medicines Agency (EMA), and a large number of others are in development or under regulatory review. Unexpectedly, however, their use has been found to be associated with serious toxic effects on a number of vital organs including the liver. Drug-induced hepatotoxicity has resulted in withdrawal from the market of many widely used drugs and... (truncated)

MESH:D053626 - atovaquone

Summary:

LLM Prediction Score	0.568 (normalized)
LLM Confidence Score	0.980
Golden Answer (Severity Class)	1.0 (normalized)
Prediction Error	0.432

Retrieved Context:

Title: The first report of atovaquone/proguanil-induced vanishing bile duct syndrome: Case report and mini-review.
The combination of Atovaquone and Proguanil (Malarone™) has been widely used for treatment and prevention of Plasmodium falciparum malaria. Transient elevation of liver enzymes is a recognized side effect of the medication. The association of Vanishing bile duct syndrome (VBDS) with the use of Atovaqoune/Proguanil was not previously reported. We describe a case of a 62-year-old male with no history of liver disease who presented with painless jaundice after receiving malaria prophylaxis with Atovaquone-proguanil for 25 days. The patient developed severe hepatitis with Vanishing bile duct syndrome. This case highlights a serious side effect of a usually well-tolerated medication.

Title: Atovaquone/proguanil-induced autoimmune-like hepatitis.
We report a novel association between the commonly used antimalarial medication atovaquone/proguanil and drug-induced autoimmune-like hepatitis. The patient developed severe liver disease fulfilling biochemical, immunologic, and histologic criteria for the diagnosis of autoimmune hepatitis after the inadvertent rechallenge with the offending drug, which had caused self-limited hepatitic symptoms a year previously. Over a period of 18 months, the patient underwent two follow-up liver biopsies showing progressive resolution of the liver inflammation and achieved complete biochemical and immunologic remission on steroids. This remission persisted for 20 months following treatment withdrawal. <i>Conclusion</i>: This well documented case raises awareness of the potential hepatotoxicity... (truncated)

Title: Tolerability of Atovaquone-Proguanil Application in Common Buzzard Nestlings.
Differences in drug tolerability among vertebrate groups and species can create substantial challenges for wildlife and ex situ conservation programmes. Knowledge of tolerance in the use of new drugs is, therefore, important to avoid severe toxicity in species, which are both commonly admitted in veterinary clinics and are of conservation concern. Antimalarial drugs have been developed for use in human medicine, but treatment with different agents has also long been used in avian medicine, as haemosporidian infections play a major role in many avian species. This study investigates the effects of the application of atovaquone-proguanil (Malarone®, GlaxoSmithKline) in common buzzards... (truncated)

Title: Adverse events associated with trimethoprim-sulfamethoxazole and atovaquone during the treatment of AIDS-related Pneumocystis carinii pneumonia.
Atovaquone was compared to trimethoprim-sulfamethoxazole (TMP-SMZ) for the relationship of time receiving therapy, plasma drug concentrations, and incidence of adverse reactions in patients with AIDS-associated Pneumocystis carinii pneumonia. Treatment-limiting adverse events occurred in 9% of atovaquone-treated patients and 24% of TMP-SMZ-treated patients. Adverse events usually did not occur before day 7 for either treatment. Only the incidence of rash increased with increasing plasma concentrations of atovaquone. The incidence of anemia, neutropenia, and azotemia increased with increasing trimethoprim plasma concentration, while other adverse events (gastrointestinal disorders, rash, fever, and liver function abnormalities) were independent of plasma drug concentration.

Title: Atovaquone ameliorate gastrointestinal toxoplasmosis complications in a pregnancy model.
Toxoplasma is an important source of foodborne hospitalization with no safe and effective therapy against chronic or congenital Toxopalsmosis. Atovaquone is a drug of choice but not approved for use in congenital Toxoplasmosis. We hypothesized atovaquone to be safe and effective against feto-maternal Toxoplasmosis.

MESH:D020927 - dexmedetomidine

Summary:

LLM Prediction Score	0.068 (normalized)
LLM Confidence Score	0.990
Golden Answer (Severity Class)	0.5 (normalized)
Prediction Error	0.432

Retrieved Context:

Title: Effects of dexmedetomidine on perioperative stress response, inflammation and immune function in patients with different degrees of liver cirrhosis.
Effects of dexmedetomidine (DEX) on perioperative stress response, inflammation and immune function in patients with different degrees of liver cirrhosis were investigated. A total of 94 patients with liver cirrhosis who were admitted to the Affiliated Hospital of Shandong University of Traditional Chinese Medicine from December 2016 to November 2017 were included, and randomly divided into control and observation group (n=47). Patients in control group were given remifentanil for anesthesia, while patients in observation group were treated with remifentanil and for DEX anesthesia. Venous blood was collected immediately before induction of anesthesia (T1), 10 min (T2) after the beginning of... (truncated)

Title: The MAP2K4/JNK/c-Jun Signaling Pathway Plays A Key Role In Dexmedetomidine Protection Against Acetaminophen-Induced Liver Toxicity.
Label="PURPOSE" NlmCategory="OBJECTIVE">Dexmedetomidine [DEX; (S)-4-[1-(2,3-dimethylphenyl)ethyl]-3H-imidazole] is a selective α₂-adrenergic receptor (α₂-AR) agonist that attenuates the liver damage associated with local or systemic inflammation. However, it remains unclear whether DEX has protective effects against acetaminophen (Paracetamol, PARA)-induced liver toxicity (PILT).

Title: The pharmacokinetics of dexmedetomidine in patients with obstructive jaundice: A clinical trial.
Dexmedetomidine, a highly selective central α2-agonist, undergoes mainly biotransformation in the liver. The pharmacokinetics of dexmedetomidine were significantly affected by hepatic insufficiency. The clearance of dexmedetomidine in patients with severe hepatic failure decreased by 50% compared with controls. We tested the hypothesis that the pharmacokinetics of dexmedetomidine would be affected by obstructive jaundice. The prospective registration number of clinical trial is ChiCTR-IPR-15007572.

Title: Dexmedetomidine alleviates hepatic injury via the inhibition of oxidative stress and activation of the Nrf2/HO-1 signaling pathway.
Dexmedetomidine (Dex), frequently used as an effective sedative, was reported to play a critical role in the protection of multiple organs. However, its underlying mechanism of a putative protective effect on ischemia/reperfusion (I/R)-induced liver injury is still unclear. A hepatocyte injury model was established by treating WRL-68 cells with oxygen and glucose deprivation/reoxygenation (OGD/R). Enzyme Linked Immunosorbent Assay (ELISA) kits were used to determine the level of inflammatory factors (IL-6, IL-1β, and TNF-α), and oxidative stress indicators (ROS, MDA, GSH-Px, and SOD). MTT assay and flow cytometry analysis were used to determine the influence of Dex on cell viability and... (truncated)

Title: Protective effect of dexmedetomidine against organ dysfunction in a two-hit model of hemorrhage/resuscitation and endotoxemia in rats.
Dexmedetomidine (DEX), a selective agonist of α2-adrenergic receptors, has anti-inflammation properties and potential beneficial effects against trauma, shock, or infection. Therefore, this study aimed to investigate whether DEX might protect against multiple-organ dysfunction in a two-hit model of hemorrhage/resuscitation (HS) and subsequent endotoxemia. Eighty Wistar rats were randomized into four groups: NS (normal saline), HS/L (HS plus lipopolysaccharide), HS/L+D (HS/L plus dexmedetomidine), and HS/L+D+Y (HS/L+D plus yohimbine). Six hours after resuscitation, blood gas (PaO2) and serum alanine aminotransferase (ALT), aspartate aminotransferase (AST), blood urine nitrogen (BUN), creatinine (Cr), TNF-α, IL-β, IL-6, IL-8, IL-10, and nitric oxide (NO) were measured. The... (truncated)

MESH:C081222 - sargramostim

Summary:

LLM Prediction Score	0.432 (normalized)
LLM Confidence Score	0.970
Golden Answer (Severity Class)	0.0 (normalized)
Prediction Error	0.432

Retrieved Context:

Title: Single Institution Experience of Ipilimumab 3 mg/kg with Sargramostim (GM-CSF) in Metastatic Melanoma.
Ipilimumab, 10 mg/kg with sargramostim (GM-CSF; GM), improved overall survival (OS) and safety of patients with advanced melanoma over ipilimumab in a randomized phase II trial. The FDA-approved dose of ipilimumab of 3 mg/kg has not been assessed with GM (IPI-GM). Consecutive patients treated with IPI-GM at a single institution were reviewed. Treatment included ipilimumab every 3 weeks × 4 and GM, 250-μg s.c. injection days 1 to 14 of each ipilimumab cycle. Efficacy, clinical characteristics, toxicities, and blinded radiology review of tumor burden were evaluated. Thirty-two patients were identified with 25 (78%) having immune-related response criteria (irRC) measurable disease... (truncated)

Title: A comparison of immune reconstitution and graft-versus-host disease following myeloablative conditioning versus reduced toxicity conditioning and umbilical cord blood transplantation in paediatric recipients.
Immune reconstitution appears to be delayed following myeloablative conditioning (MAC) and umbilical cord blood transplantation (UCBT) in paediatric recipients. Although reduced toxicity conditioning (RTC) versus MAC prior to allogeneic stem cell transplantation is associated with decreased transplant-related mortality, the effects of RTC versus MAC prior to UCBT on immune reconstitution and risk of graft-versus-host disease (GVHD) are unknown. In 88 consecutive paediatric recipients of UCBT, we assessed immune cell recovery and immunoglobulin reconstitution at days +100, 180 and 365 and analysed risk factors associated with acute and chronic GVHD. Immune cell subset recovery, immunoglobulin reconstitution, and the incidence of opportunistic... (truncated)

Title: Managing immune checkpoint-blocking antibody side effects.
Immune checkpoint-blocking antibodies that enhance the immune system's ability to fight cancer are becoming important components of treatment for patients with a variety of malignancies. Cytotoxic T-lymphocyte-associated antigen 4 (CTLA-4) was the first immune checkpoint to be clinically targeted, and ipilimumab, an inhibitor of CTLA-4, was approved by the U.S. Food and Drug Administration (FDA) for patients with advanced melanoma. The programmed cell death-1 (PD-1) receptor and one of its ligands, PD-L1, more recently have shown great promise as therapeutic targets in a variety of malignancies. Nivolumab and pembrolizumab recently have been FDA- approved for patients with melanoma and additional... (truncated)

Title: Phase II study of combination human recombinant GM-CSF with intermediate-dose cytarabine and mitoxantrone chemotherapy in patients with high-risk myelodysplastic syndromes (RAEB, RAEBT, and CMML): an Eastern Cooperative Oncology Group Study.
A Phase II study of GM-CSF with intermediate-dose cytarabine and mitoxantrone was conducted in patients with high-risk myelodysplastic syndrome. It was designed to evaluate if priming with growth factor could increase the efficiency of chemotherapy. In this older population only two of 10 patients achieved a bone marrow CR, including one patient whose leukemic blasts had an "S" phase increase of 2.55x at 48 hr. Unexpected hepatotoxicity was noted. This regimen cannot be recommended for this elderly population of patients.

MESH:D010894 - piroxicam

Summary:

LLM Prediction Score	0.805 (normalized)
LLM Confidence Score	0.980
Golden Answer (Severity Class)	0.375 (normalized)
Prediction Error	0.430

Retrieved Context:

Title: Severe cholestatic jaundice associated with piroxicam.
A 62-year-old man with rheumatoid arthritis developed jaundice while taking piroxicam. A full evaluation including ultrasound, computerized tomography, endoscopic cholangiography, and liver biopsy confirmed the diagnosis of intrahepatic cholestasis. The patient's jaundice and all other liver function abnormalities normalized 4 months after he discontinued taking piroxicam. This is the first case report in the United States of severe liver toxicity associated with piroxicam. The six cases in the English-language literature are reviewed, featuring the presentation, patterns of liver injury, and outcome in each. Piroxicam should be considered as a potential cause of cholestatic jaundice when other more common etiologies have... (truncated)

Title: Fatal submassive necrosis of the liver associated with piroxicam.
A 64-yr-old woman developed acute hepatitis after 3 wk of treatment with 40 mg/day of piroxicam (Feldene). Jaundice was preceded by a skin rash associated with eosinophilia. Despite withdrawal of the drug, she developed severe hepatocellular failure and died 53 days after the onset. Hepatitis was attributed to piroxicam because of the absence of other etiological factors.

Title: Mechanisms Involved in Toxicity of Liver Caused by Piroxicam in Mice and Protective Effects of Leaf Extract of Hibiscus rosa-sinensis L.
Piroxicam is one of the important therapeutic nonsteroidal anti-inflammatory class of drugs used mainly to suppress pain and inflammation in arthritis and other musculoskeletal disorders. Besides being anti-inflammatory, these drugs are analgesic and antipyretic often used for the relief of nonspecific fever condition. Recently, piroxicam has also gained attention as an effective therapy for tumors, colorectal, and invasive bladder cancers. The objective of the current study is to evaluate the protective effects of the alcoholic leaf extract of Hibiscus rosa-sinensis (AEH), Malvaceae, against piroxicam-induced toxicity in mice. Sixty adult Swiss albino mice (Mus musculus) were divided into four groups (n... (truncated)

Title: Nonsteroidal anti-inflammatory drug-induced liver injury: a case-control study in primary care.
Several nonsteroidal anti-inflammatory drugs (NSAIDs) have been withdrawn from the market because of hepatic adverse drug reactions (ADRs). Moreover, some cases of liver diseases have been reported in patients taking NSAIDs (arylcarboxylic NSAIDs, piroxicam, sulindac, nimesulide, etc.). Pharmacoepidemiological studies have shown a risk of hepatic ADRs with NSAIDs used in association with other hepatotoxic drugs. In contrast, other studies performed in hospitalized patients did not found any association. The aim of this study was to assess the hepatic risk associated with the use of NSAID in the setting of primary care. The study design was a case-control study where cases... (truncated)

Title: [Hepatitis due to nonsteroidal anti-inflammatory agents].
The extended prescription of non-steroidal anti-inflammatory drugs in medical practice involve numerous adverse effects. Among them, hepatic injuries, rather uncommon, are very diverse with regard to clinical type and evolution scheme, according to the derivatives used. Salicylates, when taken at high doses, increase serum transaminases, mostly without overt clinical symptoms. Phenylbutazone is obviously hepatotoxic: it induces cytolytic hepatitis, in some cases with fatal issue. Among the indole derivatives, indometacine was involved, especially in children; mixed hepatitis have been noted during sulindac therapy, mostly with favourable outcome. In the group of propionic acid derivatives, ibuprofen, pirprofen and naproxen have been implicated... (truncated)

MESH:D013866 - thioguanine

Summary:

LLM Prediction Score	0.677 (normalized)
LLM Confidence Score	0.970
Golden Answer (Severity Class)	0.25 (normalized)
Prediction Error	0.427

Retrieved Context:

Title: Splitting a therapeutic dose of thioguanine may avoid liver toxicity and be an efficacious treatment for severe inflammatory bowel disease: a 2-center observational cohort study.
Thioguanine (TG) is a treatment for inflammatory bowel disease, but association with nodular regenerative hyperplasia has restricted its use. We conjectured that splitting a therapeutic daily dose of TG would be efficacious and should avoid liver toxicity.

Title: Limited relevance and progression of histological alterations in the liver during thioguanine therapy in inflammatory bowel disease patients.
Background: Thioguanine is associated with liver toxicity, especially nodular regenerative hyperplasia (NRH). We assessed if liver histology alters during long-term maintenance treatment with thioguanine in patients with inflammatory bowel disease (IBD). Methods: Liver specimens of thioguanine treated IBD patients with at least two liver biopsies were revised by two independent liver pathologists, blinded to clinical characteristics. Alterations in histopathological findings between first and sequential liver specimen were evaluated and associated clinical data, including laboratory parameters and abdominal imaging reports, were collected. Results: Twenty-five IBD patients underwent sequential liver biopsies prior to, at time of, or after cessation of thioguanine treatment.... (truncated)

Title: 6-thioguanine as a cause of toxic veno-occlusive disease of the liver.
Lesions of hepatic veno-occlusive disease were found in the needle biopsy specimen of one patient suffering from chronic granulocytic leukaemia and in the liver at necropsy of a second patient suffering from acute myeloid leukaemia. The treatment included administration of 6-thioguanine which was the only relevant compound used in the first patient and which was combined with cytosine arabinoside in the second patient.

Title: Peliosis hepatis induced by 6-thioguanine administration.
A patient with acute myeloblastic leukaemia developed jaundice revealing peliosis hepatis after receiving 6-thioguanine for two months. Peliosis hepatis was severe and was associated with mild lesions of centrilobular veins. Withdrawal of 6-thioguanine was followed by a progressive improvement of liver dysfunction. This report shows that 6-thioguanine, a thiopurine already reported to be responsible for veno-occlusive disease of the liver, can induce peliosis hepatis. This suggests that some liver vascular disorders caused by thiopurines (6-thioguanine, azathioprine and 6-mercaptopurine), particularly peliosis hepatis, veno-occlusive disease, sinusoidal dilatation and perisinusoidal fibrosis, might be related syndromes caused by similar lesions at different sites.

Title: Is there a role for thioguanine therapy in IBD in 2017 and beyond?
Conventional thiopurines are effective for the maintenance of remission of Crohn's disease and ulcerative colitis, however, up to half of patients are intolerant or unresponsive to these medications. Thioguanine is an alternative thiopurine that has shown efficacy in inflammatory bowel disease, and is particularly useful to circumvent certain side effects associated with conventional thiopurines, for example, pancreatitis. Its association with nodular regenerative hyperplasia of the liver has hindered its widespread use. Areas covered: We aim to outline the rational use of thioguanine, including safety monitoring, with particular regard to hepatotoxicity. A literature search was performed: PubMed was searched for full... (truncated)

MESH:C401859 - temsirolimus

Summary:

LLM Prediction Score	0.427 (normalized)
LLM Confidence Score	0.970
Golden Answer (Severity Class)	0.0 (normalized)
Prediction Error	0.427

Retrieved Context:

Title: The mTOR Inhibitor Temsirolimus Added to Rituximab Combined With Dexamethasone, Cytarabine, and Cisplatinum (R-DHAP) for the Treatment of Patients With Relapsed or Refractory DLBCL - Results From the Phase-II STORM Trial.
There is a high need for novel treatment options in relapsed and refractory diffuse large B-cell lymphoma. Single agent mammalian target of rapamycin (mTOR) inhibitor treatment has shown promising efficacy in this entity. Here, we report on the results of the mTOR-inhibitor temsirolimus combined to standard rituximab-DHAP salvage regimen in a prospective, multicenter, phase II, open-label study. The STORM regimen consisted of rituximab 375 mg/m² (day 2) and DHAP (dexamethasone 40 mg day 3-6, cisplatinum 100 mg/m² day 3, cytarabine 2 × 2 g/m² day 4) with temsirolimus added on day 1 and 8 of a 21-day cycle, with 2 to 4 cycles... (truncated)

Title: Temsirolimus in the treatment of relapsed or refractory mantle cell lymphoma.
Mantle cell lymphoma (MCL) is a rare and aggressive subtype of lymphoma associated with a poor prognosis. Chemotherapy is the mainstay of frontline treatment for patients with this disease. Despite high response rates to combination chemotherapy regimens, the majority of patients relapse within a few years of treatment. Therefore, finding efficacious treatments for relapsed or refractory disease has become a growing area of clinical research. The mammalian target of rapamycin (mTOR) is responsible for integrating cell signals from growth factors, hormones, and nutrients and communicating energy status. Scientific research on aberrant molecular pathways in cancer has revealed that several proteins... (truncated)

Title: A phase I study of temsirolimus and thoracic radiation in non--small-cell lung cancer.
The addition of targeted agents to thoracic radiation has not improved outcomes in patients with locally advanced non-small-cell lung cancer (NSCLC). To improve cure rates in locally advanced NSCLC, effective targeted therapies need to be identified that can be given safely with radiation therapy. Temsirolimus is an inhibitor of the mammalian target of rapamycin (mTOR) pathway and has single-agent activity in lung cancer. Inhibition of the mTOR pathway has been found to augment the cytotoxic effect of radiation in preclinical studies. There is scant clinical experience with mTOR inhibitors and radiation.

Title: Treatment Options in Metastatic Renal Cell Carcinoma: Focus on mTOR Inhibitors.
THE AGENTS CURRENTLY APPROVED FOR USE IN METASTATIC RENAL CELL CARCINOMA (MRCC) CAN BE DIVIDED BROADLY INTO TWO CATEGORIES: (1) vascular endothelial growth factor receptor (VEGFR)-directed therapies or (2) inhibitors of the mammalian target of rapamycin (mTOR). The latter category includes everolimus and temsirolimus, both approved for distinct indications in mRCC. Everolimus gained its approval on the basis of phase III data showing a benefit in progression-free survival relative to placebo in patients previously treated with sunitinib and/or sorafenib. In contrast, temsirolimus was approved on the basis of a phase III trial in treatment-naïve patients with poor-risk mRCC, demonstrating an... (truncated)

Title: Phase I/II study of temsirolimus for patients with unresectable Hepatocellular Carcinoma (HCC)- a correlative study to explore potential biomarkers for response.
The oncogenic PI3K/Akt/mTOR pathway is frequently activated in HCC. Data on the mTOR inhibitor, temsirolimus, is limited in HCC patients with concomitant chronic liver disease. The objectives of this study were: (1) In phase I, to determine DLTs and MTD of temsirolimus in HCC patients with chronic liver disease; (2) In phase II, to assess activity of temsirolimus in HCC, and (3) to explore potential biomarkers for response.

MESH:D004112 - dimercaprol

Summary:

LLM Prediction Score	0.427 (normalized)
LLM Confidence Score	0.990
Golden Answer (Severity Class)	0.0 (normalized)
Prediction Error	0.427

Retrieved Context:

Title: Intravenous self-administration of elemental mercury: efficacy of dimercaprol therapy.
Deliberate parenteral self-injection of mercury is extremely rare, and is associated with a high degree of mortality and morbidity. Because mercury depresses cellular enzymatic mechanisms by combining with sulfhydryl groups, soluble mercuric salts are toxic to all cells. Embolization of mercury in the lungs has been reported with varying degrees of changes in pulmonary function. Mercury causes urticaria progressing to weeping dermatitis, leukopenia, anemia, diarrhea, salivation, liver damage, and renal damage progressing to acute renal failure with anuria. Dimercaprol is an effective antidote in acute heavy metal intoxication because its two sulfhydryl groups successfully compete with tissue enzyme sulfhydryl groups... (truncated)

Title: Acute arsenic poisoning treated by intravenous dimercaptosuccinic acid (DMSA) and combined extrarenal epuration techniques.
Arsenic poisoning was diagnosed in a 26-year-old man who had been criminally intoxicated over the last two weeks preceding admission by the surreptitious oral administration of probably 10 g of arsenic trioxide (As2O3). The patient developed severe manifestations of toxic hepatitis and pancreatitis, and thereafter neurological disorders, respiratory distress, acute renal failure, and cardiovascular disturbances. In addition to supportive therapy, extrarenal elimination techniques and chelating agents were used. Dimercaprol (BAL) and dimercaptosuccinic acid (DMSA or succimer) were used simultaneously as arsenic chelating agents for two days, and thereafter DMSA was used alone. DMSA was administered by intravenous (20 mg/kg/d for... (truncated)

Title: Acute mercuric chloride poisoning at a potentially lethal dose ended with survival: symptoms, concentration in cerebrospinal fluid, treatment.
This study aims to present a case of acute mercuric chloride poisoning at a potentially lethal dose treated with the antidote - 2,3-dimercapto- 1-propanesulfonic acid (DMPS) and continuous renal replacement therapy (CRRT) combined with CytoSorb. A 21-year-old woman was admitted to a hospital with abdominal pain, vomiting, and suspected gastrointestinal bleeding after taking 5000 mg of mercuric chloride for suicidal purposes. Due to the patient deteriorating general condition and multiple organ damage, on the third day she was transported to the Clinic of Anaesthesiology and Intensive Care (CAaIC), Łódź, Poland. Laboratory tests confirmed features of acute kidney injury and high... (truncated)

Title: Severe Abdominal Pain Caused by Lead Toxicity without Response to Oral Chelators: A Case Report.
A 19-year-old woman was referred to the Emergency Surgery Department with severe abdominal pain, icterus, and anemia. The patient's clinical and paraclinical findings in addition to her occupational and social history, convinced us to assay blood lead level (BLL), which was 41/5 µg/dL. Therefore toxicology consult was performed to treat lead toxicity. Recheck of the BLL showed the level as 53/7 µg/dL. So oral chelator with succimer was started. Despite consumption of oral chelator, there was no response and the pain continued. Because our repeated evaluations were negative, we decided to re-treat lead poisoning by intravenous and intramuscular chelators. Dimercaprol... (truncated)

Title: Cause of death in Wilson disease.
Before 1948, all patients with Wilson disease died shortly after diagnosis. In 1948, BAL (dimercaprol) was introduced as a possible effective treatment, to be followed by penicillamine (1955), zinc salts (1961), trientine (1969), liver transplantation (1982), and tetrathiomolybdate (1984). Despite this wide range of therapeutic options, patients still die. This article examines the cause of death in 67 patients (33 men, 34 women) out of a series of 300 seen between 1948 and 2000. Patients were classified according to their presentation as neurological, 32 patients, hepatic 11, mixed hepatic/neurological 10, hemolytic, 6, and "sibling biopsy " 8. Diagnostic failure was... (truncated)

MESH:D014747 - vinblastine

Summary:

LLM Prediction Score	0.425 (normalized)
LLM Confidence Score	0.990
Golden Answer (Severity Class)	0.0 (normalized)
Prediction Error	0.425

Retrieved Context:

Title: Phase II study of recombinant interferon alpha-2a and vinblastine in advanced renal cell carcinoma.
A total of 66 patients with advanced renal cell cancer received a combination of recombinant interferon alpha-2a (18 times 10(6) units subcutaneously 3 times weekly) and vinblastine (0.1 mg. per kg. intravenously every 3 weeks). Four patients were ineligible and 6 were inevaluable for response but evaluable for toxicity. There were no complete and 9 partial responses among the 56 evaluable patients, for a response rate of 16 per cent. Median duration of response was 26 weeks, with a range of 8 to 50 weeks. Responses were observed predominantly in patients with lung and soft tissue metastases. Patients who had... (truncated)

Title: Hyponatremia and other toxic effects during a phase I trial of recombinant human gamma interferon and vinblastine.
Recombinant human gamma interferon (Biogen) and vinblastine were administered in a phase I study. Side effects included fever and chills, nausea and vomiting, acute symptomatic hyponatremia, reversible myelosuppression, hepatitis, transient hypotension, congestive heart failure, renal insufficiency, and nonselective proteinuria. In most patients, additional host factors contributed to these toxic effects. Side effects occurred despite dose reduction; therefore, protocol accrual was prematurely closed. No correlation between serum concentrations and toxicity was noted. Median serum vinblastine concentration was 1.04 ng/ml; median serum interferon concentration was 17.3 IU/ml.

Title: [Hodgkin lymphoma with hepatic involvement treated with dexametasone, gemcitabine and cisplatin as a bridge to standard therapy: report of one case].
The initial presentation of Hodgkin lymphoma with liver involvement is rare. In these patients, the standard first-line therapy with ABVD (Adriamycin, Bleomycine, Vinblastine, Dacarbazine) imply an additional risk for liver toxicity. We report a 64-year-old woman who presented with jaundice, choluria, malaise and weight loss. In the initial evaluation she had jaundice and palpable groin lymph nodes. An obstructive biliary disease was ruled out with magnetic resonance imaging studies. A lymph node biopsy showed a Hodgkins lymphoma, Mixed-cellularity subtype. Considering the liver dysfunction, an alternative scheme of chemotherapy with dexamethasone, gemcitabine and cisplatin (GDP) was administered. After 4 cycles, a... (truncated)

Title: Vinblastine pharmacokinetics in mouse, dog, and human in the context of a physiologically based model incorporating tissue-specific drug binding, transport, and metabolism.
Vinblastine (VBL) is a vinca alkaloid-class cytotoxic chemotherapeutic that causes microtubule disruption and is typically used to treat hematologic malignancies. VBL is characterized by a narrow therapeutic index, with key dose-limiting toxicities being myelosuppression and neurotoxicity. Pharmacokinetics (PK) of VBL is primarily driven by ABCB1-mediated efflux and CYP3A4 metabolism, creating potential for drug-drug interaction. To characterize sources of variability in VBL PK, we developed a physiologically based pharmacokinetic (PBPK) model in Mdr1a/b(-/-) knockout and wild-type mice by incorporating key drivers of PK, including ABCB1 efflux, CYP3A4 metabolism, and tissue-specific tubulin binding, and scaled this model to accurately simulate VBL PK... (truncated)

Title: Recombinant interferon-alpha 2a and vinblastine in advanced renal cell cancer: a clinical phase I-II study.
Twenty patients with metastatic renal cell cancer were treated with a combination of recombinant interferon-alpha 2a (Roferon-A), 18 MU intramuscularly three times weekly and vinblastine 0.1 mg/kg intravenously once every 3 weeks. Three patients experienced a complete response (CR) (15%) and three a partial response (PR) (15%). The response duration was 3, 13, and 15 months in the CR group, and PRs lasted 11, 13, and 14 months. Constitutional symptoms like fever, anorexia, and fatigue were the most common side effects. One patient had reversible hepatitis, which was probably unrelated to antineoplastic therapy. Dose modifications had to be made in... (truncated)

MESH:D011319 - primaquine

Summary:

LLM Prediction Score	0.424 (normalized)
LLM Confidence Score	0.980
Golden Answer (Severity Class)	0.0 (normalized)
Prediction Error	0.424

Retrieved Context:

Title: Evaluation of antimalarial activity and toxicity of a new primaquine prodrug.
Plasmodium vivax is the most prevalent of the five species causing malaria in humans. The current available treatment for P. vivax malaria is limited and unsatisfactory due to at least two drawbacks: the undesirable side effects of primaquine (PQ) and drug resistance to chloroquine. Phenylalanine-alanine-PQ (Phe-Ala-PQ) is a PQ prodrug with a more favorable pharmacokinetic profile compared to PQ. The toxicity of this prodrug was evaluated in in vitro assays using a human hepatoma cell line (HepG2), a monkey kidney cell line (BGM), and human red blood cells deficient in the enzyme glucose-6-phosphate-dehydrogenase (G6PD). In addition, in vivo toxicity assays... (truncated)

Title: High-Dose Primaquine Induces Proximal Tubular Degeneration and Ventricular Cardiomyopathy Linked to Host Cells Mitochondrial Dysregulation.
Primaquine (PQ) is the only antimalarial medication used to eradicate many species of <i>Plasmodium</i> gametocytes and prevent relapse in vivax and ovale malarias. PQ metabolites induce oxidative stress and impair parasitic mitochondria, leading to protozoal growth retardation and death. Collateral damage is also presented in mammalian host cells, particularly erythrocytes, resulting in hemolysis and tissue destruction. However, the underlying mechanisms of these complications, particularly the mitochondria-mediated cell death of the host, are poorly understood. In the present study, toxicopathological studies were conducted on a rat model to determine the effect of PQ on affected tissues and mitochondrial toxicity. The results... (truncated)

Title: Differential kinetic profiles and metabolism of primaquine enantiomers by human hepatocytes.
The clinical utility of primaquine (PQ), used as a racemic mixture of two enantiomers, is limited due to metabolism-linked hemolytic toxicity in individuals with genetic deficiency in glucose-6-phosphate dehydrogenase. The current study investigated differential metabolism of PQ enantiomers in light of the suggestions that toxicity and efficacy might be largely enantioselective.

Title: [A case of malarial hepatitis by Plasmodium vivax].
Malarial infection is one of the most important tropical diseases, but also increasing in the temperate regions. Severe malaria with organ dysfunction is commonly associated with Plasmodium falciparum, but rarely with Plasmodium vivax. Malarial hepatitis is also unusual in P. falciparum and very rare in P. vivax. Only 3 cases of malarial hepatitis caused by P. vivax have been reported in the world. Because the presence of hepatitis in malaria indicates a more severe illness with higher incidence of other complications and poor prognosis, malarial patients should be meticulously monitored for hepatic dysfunction with or without jaundice. We report here... (truncated)

Title: Origins and implications of neglect of G6PD deficiency and primaquine toxicity in Plasmodium vivax malaria.
Most of the tens of millions of clinical attacks caused by Plasmodium vivax each year likely originate from dormant liver forms called hypnozoites. We do not systematically attack that reservoir because the only drug available, primaquine, is poorly suited to doing so. Primaquine was licenced for anti-relapse therapy in 1952 and became available despite threatening patients having an inborn deficiency of glucose-6-phosphate dehydrogenase (G6PD) with acute haemolytic anaemia. The standard method for screening G6PD deficiency, the fluorescent spot test, has proved impractical where most malaria patients live. The blind administration of daily primaquine is dangerous, but so too are the... (truncated)

MESH:C053541 - bicalutamide

Summary:

LLM Prediction Score	0.579 (normalized)
LLM Confidence Score	0.990
Golden Answer (Severity Class)	1.0 (normalized)
Prediction Error	0.421

Retrieved Context:

Title: Atypical onset of bicalutamide-induced liver injury.
Anti-androgen therapy is the leading treatment for advanced prostate cancer and is commonly used for neoadjuvant or adjuvant treatment. Bicalutamide is a non-steroidal anti-androgen, used during the initiation of androgen deprivation therapy along with a luteinizing hormone-releasing hormone agonist to reduce the symptoms of tumor-related flares in patients with advanced prostate cancer. As side effects, bicalutamide can cause fatigue, gynecomastia, and decreased libido through competitive androgen receptor blockade. Additionally, although not as common, drug-induced liver injury has also been reported. Herein, we report a case of hepatotoxicity secondary to bicalutamide use. Typically, bicalutamide-induced hepatotoxicity develops after a few days; however,... (truncated)

Title: Bicalutamide-induced hepatotoxicity: A rare adverse effect.
Male, 81 FINAL DIAGNOSIS: Prostate cancer Symptoms: Anorexia • dark urine • joundice • letargy

Title: Identification of the Additional Mitochondrial Liabilities of 2-Hydroxyflutamide When Compared With its Parent Compound, Flutamide in HepG2 Cells.
The androgen receptor antagonist, flutamide, is strongly associated with idiosyncratic drug-induced liver injury (DILI). Following administration, flutamide undergoes extensive first-pass metabolism to its primary metabolite, 2-hydroxyflutamide. Flutamide is a known mitochondrial toxicant; however there has been limited investigation into the potential mitochondrial toxicity of 2-hydroxyflutamide and its contribution to flutamide-induced liver injury. In this study we have used the acute glucose or galactose-conditioning of HepG2 cells to compare the mitochondrial toxicity of flutamide, 2-hydroxyflutamide and the structurally-related, non-hepatotoxic androgen receptor antagonist, bicalutamide. Compound-induced changes in mitochondrial oxygen consumption rate were assessed using Seahorse technology. Permeabilization of cells and delivery of... (truncated)

Title: Drug-related hepatotoxicity and hepatic failure following combined androgen blockade.
Androgen deprivation therapy has been the mainstay of treatment for metastatic prostate cancer and of less advanced cancers as neoadjuvant and adjuvant treatment. Abnormal liver function test, in particular elevated transaminases, is an adverse reaction more frequently noticed during androgen deprivation therapy with antiandrogen. We report the case of a patient with acute hepatic failure associated with the use of bicalutamide.

Title: Mechanism of non-steroidal anti-androgen-induced liver injury: Reactive metabolites of flutamide and bicalutamide activate inflammasomes.
Flutamide is a non-steroidal anti-androgen agent, which is mainly used for the treatment of prostate cancer. Flutamide is known to cause severe adverse events, which includes idiosyncratic liver injury. However, details of the mechanism of these adverse reactions have not been elucidated. We investigated whether flutamide induces the release of damage-associated molecular patterns (DAMPs) that activate inflammasomes. We also tested bicalutamide, enzalutamide, apalutamide, and darolutamide for their ability to activate inflammasomes in differentiated THP-1 cells. The supernatant from the incubation of flutamide and bicalutamide with human hepatocarcinoma functional liver cell-4 (FLC-4) cells increased caspase-1 activity and production of IL-1ß by... (truncated)

MESH:D003913 - dextroamphetamine

Summary:

LLM Prediction Score	0.421 (normalized)
LLM Confidence Score	0.990
Golden Answer (Severity Class)	0.0 (normalized)
Prediction Error	0.421

Retrieved Context:

Title: Adderall induced acute liver injury: a rare case and review of the literature.
Adderall (dextroamphetamine/amphetamine) is a widely prescribed medicine for the treatment of attention-deficit/hyperactivity disorder (ADHD) and is considered safe with due precautions. Use of prescribed Adderall without intention to overdose as a cause of acute liver injury is extremely rare, and to our knowledge no cases have been reported in the English literature. Amphetamine is an ingredient of recreational drugs such as Ecstacy and is known to cause hepatotoxicity. We describe here the case of a 55-year-old woman who developed acute liver failure during the treatment of ADHD with Adderall. She presented to the emergency room with worsening abdominal pain, malaise,... (truncated)

Title: Stimulant medication therapy in the treatment of children with attention deficit hyperactivity disorder.
Despite the tremendous research advances that have increased our knowledge regarding the pharmacodynamics, clinical pharmacology, pharmacokinetics, and adverse effects of stimulant medications in the treatment of children with ADHD, our knowledge is yet incomplete. Perhaps the most central unresolved issue concerns our understanding of the pathogenesis, pathophysiology, and diagnosis of ADHD. This review has touched briefly on the controversy and confusion surrounding this issue. Although our understanding of the use of stimulant medications in this disorder is similarly incomplete, a review of the literature does allow certain conclusions to be made that are helpful to the practitioner. 1. Stimulant medications... (truncated)

Title: An insight into the hepatocellular death induced by amphetamines, individually and in combination: the involvement of necrosis and apoptosis.
The liver is a vulnerable target for amphetamine toxicity, but the mechanisms involved in the drug's hepatotoxicity remain poorly understood. The purpose of the current research was to characterize the mode of death elicited by four amphetamines and to evaluate whether their combination triggered similar mechanisms in immortalized human HepG2 cells. The obtained data revealed a time- and temperature-dependent mortality of HepG2 cells exposed to 3,4-methylenedioxymethamphetamine (MDMA, ecstasy; 1.3 mM), methamphetamine (3 mM), 4-methylthioamphetamine (0.5 mM) and D-amphetamine (1.7 mM), alone or combined (1.6 mM mixture). At physiological temperature (37 °C), 24-h exposures caused HepG2 death preferentially by apoptosis, while... (truncated)

Title: Open-label pilot study of lisdexamfetamine for cocaine use disorder.
<i>Background</i>: Cocaine use disorder (CUD) is a substantial public health problem with no FDA-approved medication treatments. Psychostimulants have shown promise as pharmacotherapy for CUD. Lisdexamfetamine, a novel prodrug psychostimulant, is roughly 40-50% as potent as dextroamphetamine.<i>Objectives</i>: To evaluate the safety, tolerability, and optimal dosing of lisdexamfetamine for treating CUD.<i>Methods</i>: Open-label, 8-week trial of 17 CUD adults. Participants were titrated to the maximum tolerated dose of 140 mg over 2-week period and maintained for 4 weeks, followed by a two-week taper period. The primary outcome measures were the maximum daily dose achieved during the study period and tolerability as measured by... (truncated)

Title: MDMA toxicity and pathological consequences: a review about experimental data and autopsy findings.
Studies conducted in humans or in animals explored the presence, nature and potential causes of 3,4-methylenedioxymethamphetamine (MDMA) toxicity. According to literature, there are four principal types of such serious toxicity: hepatic, cardiovascular, cerebral and hyperpyrexic. The molecular mechanisms involved in the genesis of these toxic effects are not yet fully clarified, but the oxidative stress, excitotoxicity, and mitochondrial dysfunction appear to be causal events that converge to mediate MDMA-induced toxicity. Studies conducted on animals demonstrated that the acute administration of MDMA elicits cardiovascular responses that are similar to those elicited by d-amphetamine, and that these responses appear to involve catecholaminergic... (truncated)

MESH:D004053 - diethylpropion

Summary:

LLM Prediction Score	0.419 (normalized)
LLM Confidence Score	0.960
Golden Answer (Severity Class)	0.0 (normalized)
Prediction Error	0.419

Retrieved Context:

Title: Emerging drugs of abuse: current perspectives on substituted cathinones.
Substituted cathinones are synthetic analogs of cathinone that can be considered as derivatives of phenethylamines with a beta-keto group on the side chain. They appeared in the recreational drug market in the mid-2000s and now represent a large class of new popular drugs of abuse. Initially considered as legal highs, their legal status is variable by country and is rapidly changing, with government institutions encouraging their control. Some cathinones (such as diethylpropion or pyrovalerone) have been used in a medical setting and bupropion is actually indicated for smoking cessation. Substituted cathinones are widely available from internet websites, retail shops, and... (truncated)

Title: Fatalities Involving Khat in Jazan, Saudi Arabia, 2018 to 2021.
Interpreting fatalities involving khat is challenging due to a lack of data on cathinone and cathine reference concentrations in postmortem tissues. This study investigated the autopsy findings and toxicological results of fatalities involving khat in Saudi Arabia's Jazan region from 1 January 2018 to 31 December 2021. All confirmed cathine and cathinone results in postmortem blood, urine, brain, liver, kidney, and stomach samples were recorded and analyzed. Autopsy findings and the manner and cause of death of the deceased were assessed. Saudi Arabia's Forensic Medicine Center investigated 651 fatality cases over four years. Thirty postmortem samples were positive for khat's... (truncated)

Title: 18KHT01, a Potent Anti-Obesity Polyherbal Formulation.
Obesity is a life-threatening metabolic disorder necessitating urgent development of safe and effective therapy. Currently, limited such therapeutic measures are available for obesity. The present study was designed to develop a novel, safe and effective herbal therapy for the management of obesity. A polyherbal formulation (18KHT01) was developed by homogeneously mixing a specific proportion of crude <i>Quercus acutissima</i> (acorn jelly powder), <i>Camellia sinensis</i> (dry leaf buds), and <i>Geranium thunbergii</i> (dry aerial part) along with <i>Citrus limon</i> (fruit juice). Synergistic antioxidant, antiadipogenic, and anti-obesity activities were evaluated by <i>in vitro</i> as well as <i>in vivo</i> studies. <i>In vitro</i> experiments revealed strong... (truncated)

Title: The Evaluation of Drug Delivery Nanocarrier Development and Pharmacological Briefing for Metabolic-Associated Fatty Liver Disease (MAFLD): An Update.
Current research indicates that the next silent epidemic will be linked to chronic liver diseases, specifically non-alcoholic fatty liver disease (NAFLD), which was renamed as metabolic-associated fatty liver disease (MAFLD) in 2020. Globally, MAFLD mortality is on the rise. The etiology of MAFLD is multifactorial and still incompletely understood, but includes the accumulation of intrahepatic lipids, alterations in energy metabolism, insulin resistance, and inflammatory processes. The available MAFLD treatment, therefore, relies on improving the patient's lifestyle and multidisciplinary pharmacotherapeutic options, whereas the option of surgery is useless without managing the comorbidities of the MAFLD. Nanotechnology is an emerging approach addressing... (truncated)

Title: Non-paracetamol drug-induced fulminant hepatic failure among adults in Scotland.
This study details 30 cases of non-paracetamol drug-induced fulminant hepatic failure (NPDI-FHF) that have presented to the Scottish Liver Transplant Unit since 1992. Using the patients' case notes and a previously constructed database, the demographics of NPDI-FHF in Scotland were studied. The clinical and biochemical features, and the outcome of each individual case were also investigated.

MESH:C024352 - fludarabine

Summary:

LLM Prediction Score	0.419 (normalized)
LLM Confidence Score	0.980
Golden Answer (Severity Class)	0.0 (normalized)
Prediction Error	0.419

Retrieved Context:

Title: Hepatic injury after nonmyeloablative conditioning followed by allogeneic hematopoietic cell transplantation: a study of 193 patients.
Liver injury is a frequent, serious complication of allogeneic hematopoietic cell transplantation (HCT) following myeloablative preparative regimens. We sought to determine the frequency and severity of hepatic injury after nonmyeloablative conditioning and its relationship to outcomes. One hundred ninety-three consecutive patients who received 2 Gy total body irradiation with or without fludarabine were evaluated for end points related to liver injury. Patients with diseases treatable by HCT who were ineligible for conventional myeloablative allogeneic HCT because of advanced age and/or comorbid conditions were included. Fifty-one patients (26%) developed hyperbilirubinemia of 68.4 microM (4 mg/dL) or greater, most commonly resulting from... (truncated)

Title: A fludarabine-based dose-reduced conditioning regimen followed by allogeneic stem cell transplantation from related or unrelated donors in patients with myelodysplastic syndrome.
We investigated the feasibility and efficacy of a fludarabine-based dose-reduced conditioning regimen followed by stem cell transplantation from related (n = 5) or unrelated HLA-matched donors (n = 7) in 12 patients with high risk MDS, who were not eligible for a standard myeloablative conditioning regimen. The conditioning regimen consisted of fludarabine 30 mg/m(2) daily for 6 days, busulfan 4 mg/kg daily for 2 days and anti-thymocyte globulin (ATG, rabbit) 10 mg/kg daily for 4 days in 11 patients, while one patient received fludarabine, ATG, cyclophosphamide and thiotepa. Graft-versus-host disease prophylaxis consisted of cyclosporine and a short course of methotrexate.... (truncated)

Title: [Long-term results of treatment of chronic B-cell lymphoid leukocytes in persons who participated in the elimination of the effects of the nuclear accident at the Chernobyl nuclear station].
Clinical-and-hematological characteristics are presented of B-cell chronic lymphoid leukosis in those persons who took part in the elimination of the effects of the Chornobyl accident in the remote period. Results are highlighted of treatment of 16 patients with making use of different chemical drug preparations. Employment of fludarabin and cyclophosphan combined in treatment of the medical condition in question has been shown to promote long-term complete and partial remissions in a major proportion of patients, which effect was not achievable with standard means of remediation. A side effect of fludarabin was leukocytopenia that in the presence of changed immunity threatened... (truncated)

Title: [Clinical study on fludarabine combined with cytarabine regimen in the treatment of patients with refractory and relapsed acute myeloid leukemia].
The aim of study was to evaluate the clinical efficacy and toxicity of fludarabine combined with cytarabine (FA) regimen in the treatment of patients with refractory and/or relapsed acute myeloid leukemia (AML). Nineteen cases with refractory/relapsed AML were treated with FA regimen in which fludarabine phosphate 25 mg/(m(2) x d), d1-5; cytarabine (Ara-C) 2 g/(m(2) x d), d1-5. Another 20 cases were treated with salvage chemotherapy (MAE regimen: mitoxantrone, Ara-C and etoposide or DAE regimen: daunorubicin, Ara-C and etoposide). All patients received at least 2 cycles chemotherapy. The results showed that 9 patients (47%) in FA regimen group achieved complete... (truncated)

Title: Fludarabine, cyclophosphamide and mitoxantrone for untreated follicular lymphoma: a report from the non-Hodgkin's lymphoma co-operative study group.
The aim of the study was to determine the safety and efficacy of the combination of fludarabine (FLU), cyclophosphamide (CY) and mitoxantrone (FLU/CY/MITO) in untreated follicular lymphomas (FL), Sixty patients with newly diagnosed stage II bulky to IV FL, median age 59 years (range 36-70), received FLU/CY/MITO regimen (FLU 25 mg/m2 days 1-3, CY 300 mg/m2 days 1-3, Mito 10 mg/m2 day 1). Patients received antibiotic oral prophylaxis during all treatments, and growth factors (G-CSF) when grade III granulocytopenia (WHO) occurred. The overall response rate was 87%: 46 patients achieved complete response (CR) (77%), 6 a partial response (10%) and... (truncated)

MESH:D014325 - tromethamine

Summary:

LLM Prediction Score	0.332 (normalized)
LLM Confidence Score	0.940
Golden Answer (Severity Class)	0.75 (normalized)
Prediction Error	0.418

Retrieved Context:

Title: THAM for control of ICP.
Our goal was to perform a systematic review of the literature on the use of tromethamine (THAM) and its effects on intracranial pressure (ICP) in patients with neurological illness. All articles from MEDLINE, BIOSIS, EMBASE, Global Health, HealthStar, Scopus, Cochrane Library, the International Clinical Trials Registry Platform (inception to February 2014), reference lists of relevant articles, and gray literature were searched. Two reviewers independently identified all manuscripts pertaining to the administration of THAM in human patients that recorded effects on ICP. Secondary outcomes of effect on cerebral perfusion pressure, mean arterial pressure, patient outcome, and adverse effects were recorded. Two... (truncated)

Title: Pomegranate action in curbing the incidence of liver injury triggered by Diethylnitrosamine by declining oxidative stress via Nrf2 and NFκB regulation.
Unearthing and employment of healthy substitutes is now in demand to tackle a number of diseases due to the excessive repercussions of synthetic drugs. In this frame of reference pomegranate juice (PGJ) is a boon comprising of anthocyanins and hydrolysable tannins, known for its anti-oxidant and anti-inflammatory properties. Despite various documented roles of PGJ, there are no studies on antifibrotic potential in NDEA-induced mammalian liver fibrotic model. Hepatic fibrosis in rats was induced by the intra-peritoneal injection of NDEA (10 mlkg⁻¹b.wt. of 1% NDEA) in two weeks. Biochemical, histopathological and ultra-structural studies were carried out on control, fibrotic and treated rats.... (truncated)

Title: High risk of drug toxicity in social isolation stress due to liver dysfunction: Role of oxidative stress and inflammation.
Previous studies have shown that social isolation stress (SIS) could associate with several systemic diseases; however, the role of SIS on liver dysfunction has yet to be established. This study aimed to investigate the effect of SIS on liver function and possible drug toxicity through liver inflammation and oxidative stress.

Title: Neutropenia, thrombocytopenia and hepatic injury associated with dexketoprofen trometamol therapy in a previously healthy 35-year-old woman.
This case report describes a previously healthy 35-year-old woman, with an episode of fever, neutropenia, thrombocytopenia and elevation of biochemical markers of liver injury, 10 days after beginning drug therapy with dexketoprofen trometamol. Infectious and autoimmune causes of neutropenia, and viral or autoimmune hepatitis were excluded. The resolution following withdrawal of dexketoprofen trometamol confirms the possibility of an adverse drug reaction.

Title: An in vitro liver model--assessing oxidative stress and genotoxicity following exposure of hepatocytes to a panel of engineered nanomaterials.
Following exposure via inhalation, intratracheal instillation or ingestion some nanomaterials (NM) have been shown to translocate to the liver. Since oxidative stress has been implicated as a possible mechanism for NM toxicity this study aimed to investigate the effects of various materials (five titanium dioxide (TiO_2), two zinc oxide (ZnO), two multi-walled carbon nanotubes (MWCNT) and one silver (Ag) NM) on oxidative responses of C3A cell line as a model for potential detrimental properties of nanomaterials on the liver.

MESH:D010303 - paromomycin

Summary:

LLM Prediction Score	0.418 (normalized)
LLM Confidence Score	0.980
Golden Answer (Severity Class)	0.0 (normalized)
Prediction Error	0.418

Retrieved Context:

Title: Effect of topical Nanoliposomes of Paromomycin on Rats Liver and Kidney.
Hepatotoxicity due to drugs is the most common cause of deaths. Nephrotoxicity of the drugs is usually associated with the drugs accumulation in renal tissue. Paromomycin sulfate (PMS) is an anti-leishmania drug. Although the topical approach for the treatment of leishmania is attractive, its use might cause nephrotoxicity and hepatotoxicity.

Title: Flavonoids as a Natural Treatment Against <i>Entamoeba histolytica</i>.
Over the past 20 years, gastrointestinal infections in developing countries have been a serious health problem and are the second leading cause of morbidity among all age groups. Among pathogenic protozoans that cause diarrheal disease, the parasite <i>Entamoeba histolytica</i> produces amebic colitis as well as the most frequent extra-intestinal lesion, an amebic liver abscess (ALA). Usually, intestinal amebiasis and ALA are treated with synthetic chemical compounds (iodoquinol, paromomycin, diloxanide furoate, and nitroimidazoles). Metronidazole is the most common treatment for amebiasis. Although the efficacy of nitroimidazoles in killing amebas is known, the potential resistance of <i>E. histolytica</i> to this treatment is... (truncated)

Title: Therapeutic Modalities in Post Kala-azar Dermal Leishmaniasis: A Systematic Review of the Effectiveness and Safety of the Treatment Options.
Post-kala-azar dermal Leishmaniasis (PKDL) is one of the important neglected tropical diseases, which has a tremendous epidemiological significance, being the reservoir of kala-azar. Relapse and resistance to treatment along with the lack of a drug of choice and consensus treatment guideline pose a significant problem in the management of PKDL. The aim of this article was to review the available therapeutic options for PKDL, with special emphasis on their pharmaco-dynamics, pharmaco-kinetics, effectiveness, safety, tolerability, and cost factor. A comprehensive English language literature search was done for therapeutic options in PKDL across multiple databases (PubMed, EMBASE, MEDLINE, and Cochrane) for keywords... (truncated)

Title: Modified solid lipid nanoparticles encapsulated with Amphotericin B and Paromomycin: an effective oral combination against experimental murine visceral leishmaniasis.
The development of an effective oral therapeutics is an immediate need for the control and elimination of visceral leishmaniasis (VL). We exemplify the preparation and optimization of 2-hydroxypropyl-β-cyclodextrin (HPCD) modified solid lipid nanoparticles (SLNs) based oral combinational cargo system of Amphotericin B (AmB) and Paromomycin (PM) against murine VL. The emulsion solvent evaporation method was employed to prepare HPCD modified dual drug-loaded solid lipid nanoparticles (m-DDSLNs). The optimized formulations have a mean particle size of 141 ± 3.2 nm, a polydispersity index of 0.248 ± 0.11 and entrapment efficiency for AmB and PM was found to be 96% and 90%... (truncated)

Title: Is paromomycin an effective and safe treatment against cutaneous leishmaniasis? A meta-analysis of 14 randomized controlled trials.
High cost, poor compliance, and systemic toxicity have limited the use of pentavalent antimony compounds (SbV), the treatment of choice for cutaneous leishmaniasis (CL). Paromomycin (PR) has been developed as an alternative to SbV, but existing data are conflicting.

MESH:D007213 - indomethacin

Summary:

LLM Prediction Score	0.585 (normalized)
LLM Confidence Score	0.980
Golden Answer (Severity Class)	1.0 (normalized)
Prediction Error	0.415

Retrieved Context:

Title: Idiosyncratic NSAID drug induced oxidative stress.
Many idiosyncratic non-steroidal anti-inflammatory drugs (NSAIDs) cause GI, liver and bone marrow toxicity in some patients which results in GI bleeding/ulceration/fulminant hepatic failure/hepatitis or agranulocytosis/aplastic anemia. The toxic mechanisms proposed have been reviewed. Evidence is presented showing that idiosyncratic NSAID drugs form prooxidant radicals when metabolised by peroxidases known to be present in these tissues. Thus GSH, NADH and/or ascorbate were cooxidised by catalytic amounts of NSAIDs and hydrogen peroxide in the presence of peroxidase. During GSH and NADH cooxidation, oxygen uptake and activation occurred. Furthermore the formation of NSAID oxidation products was prevented during the cooxidation indicating that the... (truncated)

Title: Comparative effect of indomethacin (IndoM) on the enzymes of carbohydrate metabolism, brush border membrane and oxidative stress in the kidney, small intestine and liver of rats.
Indomethacin (IndoM) has prominent anti-inflammatory and analgesic-antipyretic properties. However, high incidence and severity of side-effects on the structure and functions of the kidney, liver and intestine limits its clinical use. The present study tested the hypothesis that IndoM causes multi-organ toxicity by inducing oxidative stress that alters the structure of various cellular membranes, metabolism and hence functions. The effect of IndoM was determined on the enzymes of carbohydrate metabolism, brush border membrane (BBM) and oxidative stress in the rat kideny, liver and intestine to understand the mechanism of IndoM induced toxicity. Adult male Wister rats were given IndoM (20 mg/kg)... (truncated)

Title: A case of indometacin-induced acute hepatitis developing into chronic autoimmune hepatitis.
A 47-year-old woman who presented with hip pain as a result of osteoarthritis was treated with indometacin. Ten days after beginning treatment she developed jaundice and ascites. All medications were discontinued, but her symptoms continued to worsen.

Title: [Hepatic lesions induced by drugs. Report of 26 cases].
Two thousand six hundred and seventy one liver biopsies were reviewed from 1972 to 1985 at the Hospital A. Posadas. There were 26 patients with drug-induced liver injury; those who fulfilled the following criteria were included: contact with a drug known to produce hepatotoxicity; clinic, biologic and histologic picture corresponding to the drug studied, complete recovery after the drug was stopped and no other hepatic toxics. Fourteen patients showed estrogen-induced intrahepatic cholestasis, 5 had hepatitis-like lesions due to: alphamethyldopa (3), ketoconazole (1) and indomethacin (1). Carbon tetrachloride caused fatty degeneration in two patients and phenylbutazone a granulomatous hepatitis in one... (truncated)

Title: Hepatotoxic effects of anti-rheumatic drugs in cultured rat hepatocytes.
Drug-induced liver injury has been suggested as a possible mechanism for the liver damage in rheumatic diseases. To evaluate the role of direct toxic action of drugs on hepatocytes, the effects of salicylate, chloroquine, prednisolone and indomethacin on LDH leakage from cultured rat hepatocytes were studied. Exposure for 24 h to the first two drugs induced liver damage, as reflected by LDH release, at concentrations 2-10 times as high as the therapeutic plasma levels in humans. Indomethacin and prednisolone at concentrations approx 50-100-fold higher than the therapeutic plasma levels, were not toxic to cultured hepatocytes. The data suggest that direct... (truncated)

MESH:D017374 - paroxetine

Summary:

LLM Prediction Score	0.587 (normalized)
LLM Confidence Score	0.990
Golden Answer (Severity Class)	1.0 (normalized)
Prediction Error	0.413

Retrieved Context:

Title: Severe hepatotoxicity with jaundice associated with paroxetine.
Hepatotoxicity due to paroxetine, a selective serotonin reuptake inhibitor, is very rare, and to the best of our knowledge, only five cases of liver injury in association with paroxetine have previously been reported in the medical literature. We describe the clinical, biochemical, and pathological findings in a patient with paroxetine hepatotoxicity, which was reversed after withdrawal of the drug. The present case and the others previously reported suggest that hepatotoxicity should be taken into account as a rare complication, sometimes severe, that may occur with paroxetine.

Title: Atrium and paroxetine-related severe hepatitis.
Two cases of severe hepatitis in young women using Atrium and paroxetine are presented. Both patients presented jaundice, marked increase in aminotransferases activities, and pronounced prolongation in prothrombin time. In both cases, liver biopsy specimen examination revealed lesions compatible with drug-related injury. Other causes of hepatic injury were reasonably ruled out by complete careful screening. Outcome was marked by rapid complete recovery in one case and by slow recovery in the other. We suggest that simultaneous treatment with Atrium and paroxetine could increase each of these drugs' hepatotoxicity.

Title: [Severe hepatitis attributed to paroxetine (Seroxat)].
A 52-year-old man suffering from chronic hepatitis B related liver cirrhosis was treated for depression with paroxetine (Serotax) 5 mg and subsequently 15 mg once daily. A severe hepatitis with liver failure (jaundice, hypoalbuminaemia, edemas, and ascites) developed. After the drug was withdrawn the patient recovered completely. The adverse reaction may be explained by a change in the pharmacokinetics of the drug caused by the liver cirrhosis. Caution is required in prescribing long-term paroxetine therapy for patients with documented liver cirrhosis. The liver function should be monitored and administration of the drug should be discontinued when functional disturbances are noticed.

Title: Liver injury associated with antidepressants.
Antidepressants are commonly prescribed and used in the management of depression, anxiety disorders, and other psychiatric illnesses. Antidepressants used in therapeutic dosing ranges are associated with causing several adverse drug reactions including hepatotoxicity. Paroxetine, fluoxetine, fluvoxamine, citalopram, mirtazapine and venlafaxine are associated with reversible liver injury upon discontinuation of the agent. Patient cases of hepatotoxicity involving the use of nefazodone, trazodone, duloxetine, bupropion, and sertraline are linked to causing death in its users. Due to the idiosyncratic nature of hepatotoxicity, monitoring of liver function tests and immediate discontinuation upon abnormal lab findings or signs and symptoms of liver dysfunction are... (truncated)

Title: The role of NLRP3 inflammasome in psychotropic drug-induced hepatotoxicity.
Increased medical application of psychotropic drugs raised attention concerning their toxicological effects. In fact, more than 160 psychotropic drugs including antidepressants and antipsychotics, have been shown to cause liver side effects, but the underlying mechanisms are still poorly understood. Here, we discovered that fluoxetine, a common antidepressant, was specifically sensed by NLRP3 inflammasome, whose subsequent activation resulted in the maturation of caspase-1 and IL-1β, as well as gasdermin D (GSDMD) cleavage, which could be completely abrogated by a selective NLRP3 inhibitor MCC950 or Nlrp3 knockout (Nlrp3^{-/-}). Mechanistically, mitochondrial damage and the subsequent mitochondrial reactive oxygen species (mtROS) accumulation were crucial... (truncated)

MESH:D000525 - alprazolam

Summary:

LLM Prediction Score	0.466 (normalized)
LLM Confidence Score	0.990
Golden Answer (Severity Class)	0.875 (normalized)
Prediction Error	0.409

Retrieved Context:

Title: Alprazolam in panic disorder and agoraphobia: results from a multicenter trial. II. Patient acceptance, side effects, and safety.
In a multicenter placebo-controlled study, the safety, side effects, and patient acceptance of alprazolam for the treatment of panic disorder and agoraphobia were examined. A total of 525 patients meeting DSM-III criteria for agoraphobia with panic attacks or panic disorder were randomly assigned to receive alprazolam or placebo, which they took for eight weeks. The mean daily dose at the end of the study was 5.7 mg of alprazolam or 7.5 capsules of placebo daily. Potentially serious reactions to alprazolam occurred in ten of 263 subjects who received the drug. These included acute intoxication (three), hepatitis (two), mania (two), amnesia... (truncated)

Title: Characterization of Potential Intoxications with Medicines in a Regional Setting.
The Portuguese Poison Information Center (from Portuguese-CIAV) is a call center that offers medical assistance in case of possible intoxication with any kind of product, including medicines. This center´s main goal is to inform and guide the general public and health professionals. This work aimed to analyze and compare data corresponding to the telephone calls from the Algarve region (South of Portugal), received by CIAV during 2019 and 2020, regarding potential intoxications with medicines. To this end, data provided by CIAV on possible cases of medication intoxication in the Algarve region were collected, including the number of calls received, the... (truncated)

Title: In vitro platforms for evaluating liver toxicity.
The liver is a heterogeneous organ with many vital functions, including metabolism of pharmaceutical drugs and is highly susceptible to injury from these substances. The etiology of drug-induced liver disease is still debated although generally regarded as a continuum between an activated immune response and hepatocyte metabolic dysfunction, most often resulting from an intermediate reactive metabolite. This debate stems from the fact that current animal and in vitro models provide limited physiologically relevant information, and their shortcomings have resulted in "silent" hepatotoxic drugs being introduced into clinical trials, garnering huge financial losses for drug companies through withdrawals and late stage... (truncated)

Title: Separate and combined impact of acute naltrexone and alprazolam on subjective and physiological effects of oral d-amphetamine in stimulant users.
Opioid antagonists (e.g., naltrexone) and positive modulators of γ-aminobutyric-acidA (GABAA) receptors (e.g., alprazolam) modestly attenuate the abuse-related effects of stimulants like amphetamine. The use of higher doses to achieve greater efficacy is precluded by side effects. Combining naltrexone and alprazolam might safely maximize efficacy while avoiding the untoward effects of the constituent compounds.

Title: Comprehensive review of hepatotoxicity associated with traditional Indian Ayurvedic herbs.
With growing antipathy toward conventional prescription drugs due to the fear of adverse events, the general and patient populations have been increasingly using complementary and alternative medications (CAMs) for managing acute and chronic diseases. The general misconception is that natural herbal-based preparations are devoid of toxicity, and hence short- and long-term use remain justified among people as well as the CAM practitioners who prescribe these medicines. In this regard, Ayurvedic herbal medications have become one of the most utilized in the East, specifically the Indian sub-continent, with increasing use in the West. Recent well-performed observational studies have confirmed the hepatotoxic... (truncated)

MESH:C062735 - zafirlukast

Summary:

LLM Prediction Score	0.591 (normalized)
LLM Confidence Score	0.970
Golden Answer (Severity Class)	1.0 (normalized)
Prediction Error	0.409

Retrieved Context:

Title: Zafirlukast-induced acute hepatitis.
Zafirlukast, a competitive cysteinyl leukotriene receptor antagonist, is a new class of asthma medications. It has shown an adverse event profile similar to that of placebo. Herein, we present a 69-year-old female patient who suffered from general malaise, poor appetite, nausea and jaundice after 3 months of zafirlukast therapy for asthma. She had no past history of liver disease, nor history of alcoholism, herb medication, blood transfusion, acupuncture, tattoo or recent traveling history. Liver biochemistries revealed elevated serum alanine aminotransferase and aspartase aminotransferase levels up to 481 U/L and 212 U/L, respectively. Moreover, peak serum total bilirubin level was elevated... (truncated)

Title: Zafirlukast ameliorates Docetaxel-induced activation of NOD-like receptor protein 3 (NLRP3) inflammasome, mediated by sirtuin1 (SIRT1) in hepatocytes.
Docetaxel-associated liver injury has become a serious public health problem, resulting in therapy discontinuation, liver failure, and death. Zafirlukast is a typical leukotriene receptor antagonist used for prophylaxis and chronic treatment of asthma. In this study, we investigate whether treatment with Zafirlukast could alleviate Docetaxel-induced cytotoxicity in hepatocytes. Our results indicate that Zafirlukast mitigated Docetaxel-induced toxicity in LO-2 hepatocytes. Firstly, Zafirlukast reduced the production of 8-hydroxy-2p-deoxyguanosine (8-OHdG) and increased the levels of reduced glutathione (GSH) against Docetaxel. Secondly, Zafirlukast elevated the levels of mitochondrial membrane potential (ΔΨm) and adenosine triphosphate (ATP). Thirdly, Zafirlukast prevented Docetaxel-induced release of lactate dehydrogenase (LDH)... (truncated)

Title: The use of 5-lipoxygenase inhibitors and leukotriene receptor antagonists in the treatment of chronic asthma.
With the elucidation of asthma as a chronic inflammatory disease, therapeutic approaches have shifted from treatment of symptoms with bronchodilators to treatment of the underlying disease with antiinflammatory agents. Along with concerns about corticosteroid side effects on the part of both physicians and patients, this shift has motivated researchers to develop and test new agents with antiinflammatory capabilities. The leukotrienes are endogenous mediators with three inflammatory effects: they increase vascular permeability, recruit other inflammatory leukocytes, and induce bronchoconstriction. A number of antileukotriene agents are in various stages of development. Zileuton, a leukotriene synthesis inhibitor, has been shown to improve airway... (truncated)

Title: The Effect of Montelukast on Liver Damage in an Experimental Obstructive Jaundice Model.
Montelukast is a cysteinyl-leukotriene type 1 (CysLT1) selective receptor antagonist. In recent years, investigations have shown that montelukast possesses secondary anti-inflammatory activities and also antioxidant effects. For this reason, we aimed to determine the possible effects of montelukast on liver damage in experimental obstructive jaundice.

Title: Severe liver injury after treatment with the leukotriene receptor antagonist zafirlukast.
In registration trials, zafirlukast, an asthma medication, caused asymptomatic elevated aminotransferase levels in up to 5% of participants. Until now, however, no cases of severe hepatitis attributed to zafirlukast have been reported.

MESH:C098320 - efavirenz

Summary:

LLM Prediction Score	0.466 (normalized)
LLM Confidence Score	0.990
Golden Answer (Severity Class)	0.875 (normalized)
Prediction Error	0.409

Retrieved Context:

Title: Liver toxicity induced by non-nucleoside reverse transcriptase inhibitors.
Liver toxicity is one of the most relevant adverse effects of antiretroviral therapy. Within the non-nucleoside reverse transcriptase inhibitors (NNRTIs), efavirenz can be considered a safer drug for the liver than nevirapine. In fact, the frequency of severe increased liver enzymes in patients on efavirenz ranges from 1 to 8%, whereas in patients treated with nevirapine, it ranges from 4 to 18%. Likewise, nevirapine is more commonly associated than efavirenz with early acute hepatitis, which is produced by a hypersensitivity mechanism and has a defined risk profile that often makes it avoidable. Despite the fact that most cases of NNRTI-induced... (truncated)

Title: Acute Liver Toxicity due to Efavirenz/Emtricitabine/Tenofovir.
The fixed-dose combination of Efavirenz/Emtricitabine/Tenofovir is a first-line agent for the treatment of HIV; however few cases have reported hepatotoxicity associated with the drug. We report a case of Efavirenz/Emtricitabine/Tenofovir-associated hepatotoxicity presenting mainly with hepatocellular injury characterized by extremely elevated aminotransferase levels, which resolved without acute liver failure or need for liver transplant referral.

Title: Liver toxicity in HIV-infected patients receiving novel second-generation nonnucleoside reverse transcriptase inhibitors etravirine and rilpivirine.
There are few data on the hepatic safety profile of the novel second-generation nonnucleoside reverse transcriptase inhibitors etravirine and rilpivirine. Previous extensive studies including other drugs of the same class, nevirapine and efavirenz, have shown an incidence of liver toxicity of 3-20%, higher in the case of nevirapine. The pathogenic mechanisms involved are related to hypersensitivity, as described with nevirapine, impaired metabolism and therefore increased drug levels, and direct toxic effects with production of toxic metabolites. Hepatitis C coinfection seems to be the most important factor for toxicity, especially in the case of advanced liver fibrosis. Etravirine showed a similar... (truncated)

Title: ER stress in human hepatic cells treated with Efavirenz: mitochondria again.
ER stress is associated with a growing number of liver diseases, including drug-induced hepatotoxicity. The non-nucleoside analogue reverse transcriptase inhibitor Efavirenz, a cornerstone of the multidrug strategy employed to treat HIV1 infection, has been related to the development of various adverse events, including metabolic disturbances and hepatic toxicity, the mechanisms of which remain elusive. Recent evidence has pinpointed a specific mitochondrial effect of Efavirenz in human hepatic cells. This study assesses the induction of ER stress by Efavirenz in the same model and the implication of mitochondria in this process.

Title: Incidence of liver injury after beginning antiretroviral therapy with efavirenz or nevirapine.
To compare the incidence and define the risk factors associated with liver toxicity in patients beginning treatment with nevirapine (NVP) and efavirenz (EFZ).

MESH:D058766 - levoleucovorin

Summary:

LLM Prediction Score	0.407 (normalized)
LLM Confidence Score	0.970
Golden Answer (Severity Class)	0.0 (normalized)
Prediction Error	0.407

Retrieved Context:

Title: Partial response after transcatheter arterial infusion chemotherapy in a patient with systemic chemotherapy-resistant unresectable colon cancer and hepatic metastasis: (case report).
We report here a case of partial response to hepatic arterial infusion chemotherapy in a patient who developed serious hepatic failure due to unresectable colorectal cancer and hepatic metastasis and showed resistance to systemic chemotherapy with molecular targeted drugs, mFOLFOX6, and FOLFIRI. The patient was a 60-year-old woman who underwent sigmoidectomy for sigmoid colon cancer, lateral posterior hepatic segmentectomy for metastatic liver cancer, and postoperative radiation therapy for metastatic lung cancer. As first-line systemic chemotherapy, mFOLFOX6 (oxaliplatin, 5-fluorouracil, and leucovorin), bevacizumab + FOLFIRI (irinotecan, 5-fluorouracil, leucovorin), and anti-epidermal growth factor receptor antibody + irinotecan were administered, in that order. However,... (truncated)

Title: Substitution of l-leucovorin for d,l-leucovorin in the rescue from high-dose methotrexate treatment in patients with osteosarcoma.
Studies in which high-dose methotrexate (HDMTX) is used for the treatment of osteosarcoma have utilized commercial formulations of d,l-leucovorin (leucovorin calcium) for rescue from potential methotrexate (MTX) toxicity. These formulations are racemic mixtures containing equal amounts of d and l isomers of leucovorin. All of the available data indicate that the l isomer is the pharmacologically active diastereomer. A clinical study was conducted to determine if l-leucovorin was as safe and efficacious as d,l-leucovorin in the rescue of patients with osteosarcoma who were treated with HDMTX (12.5 g/m2 over 6 h). Because d,l-leucovorin consists of equal proportions of d and... (truncated)

Title: Toxicity during l-LV/5FU adjuvant chemotherapy as a modified RPMI regimen for patients with colorectal cancer.
l-leucovorin (LV)/5-fluorouracil (5FU) may play an important role, as an adjuvant chemotherapy, in improving the survival of patients with stage III colorectal cancer. However, severe toxicity of the chemotherapeutic agent could be fatal. Adverse effects, including bone marrow suppression, liver damage, renal damage, and glucose tolerance, were evaluated daily during 3 courses of l-LV/5FU-modified RPMI regimen adjuvant chemotherapy for 22 patients with stage III colorectal cancer. Decrease in the serum levels of neutrophils and platelets occurred in the 1st course, which became more obvious after three or four administrations of l-LV/5FU in the 1st course. Furthermore, serum levels of leukocytes,... (truncated)

Title: Pseudocirrhosis in Gastric Cancer with Diffuse Liver Metastases after a Dramatic Response to Chemotherapy.
We present the first reported case of pseudocirrhosis arising after a dramatic response to chemotherapy in metastatic gastric cancer. A 74-year-old man was diagnosed with gastric adenocarcinoma having multiple liver metastases. His general condition was poor, with an Eastern Cooperative Oncology Group performance status of 3, inadequate oral intake, and jaundice (total bilirubin 2.8 mg/dl). Chemotherapy with oxaliplatin, l-leucovorin, and 5-fluorouracil (modified FOLFOX-6) was initiated. After four treatment cycles, he experienced a marked regression of liver metastases; however, he developed massive ascites with a lobular liver surface and segmental atrophy, which were consistent with pseudocirrhosis. Chemotherapy was continued along with... (truncated)

Title: A phase II study of levofolinic acid and 5-fluorouracil plus cisplatin in patients with advanced head and neck squamous cell carcinoma.
Forty patients with advanced squamous cell carcinoma of the head and neck (SCHNC) were treated with a combination of levofolinic acid 100 mg/m2+5-fluorouracil 375 mg/m2 in a 4-hour infusion plus cisplatin 20 mg/m2 in a 2-hour infusion for 5 consecutive days, repeated every 21-28 days. In the group of 20 previously untreated patients, a 90% overall response rate (ORR) with a 30% complete response rate (CRR) was obtained. In the group of 20 pretreated patients with recurrent and/or metastatic SCHNC, a 55% ORR with 15% CRR was achieved. This treatment was given on an outpatient basis and was generally very... (truncated)

MESH:D008939 - mitotane

Summary:

LLM Prediction Score	0.407 (normalized)
LLM Confidence Score	0.980
Golden Answer (Severity Class)	0.0 (normalized)
Prediction Error	0.407

Retrieved Context:

Title: EDP-mitotane in children: reassuring evidence of reversible side-effects and neurotoxicity.
Adrenocortical carcinoma affects one in 5 million children each year. Since prognosis for children older than 4 years is limited, clinicians often choose aggressive treatment with etoposide, doxorubicin, cisplatin (EDP) and mitotane after resection. However, little is known about the impact of EDP-mitotane in children. We provide an overview of case-reports and case series listing side-effects and neurotoxicity of EDP-mitotane in children. Fourteen studies were identified describing a range of gastro-intestinal, endocrine, developmental and neuropsychological side-effects. Neurotoxicity included motor- and speech delay, decreased concentration and lower school performance. These side-effects appear to be reversible after mitotane discontinuation. We have added... (truncated)

Title: Role of Mitotane in Adrenocortical Carcinoma - Review and State of the art.
Adrenocortical carcinoma (ACC) is a rare and aggressive endocrine tumour deriving from the adrenal cortex. A correct therapeutic strategy requires a multidisciplinary approach between endocrinologist, surgeon and oncologist. Surgery is the mainstay treatment in ACC while mitotane, deriving from the insecticide dichloro-diphenyl-trichloro-ethane, is the main base of the medical treatment of ACC in consideration of its adrenocytolitic activity. However, the use of mitotane as adjuvant therapy is still controversial, also in consideration of the retrospective nature of several studies. A prospective randomised trial (ADIUVO), recruiting patients with low-intermediate risk of recurrence, is evaluating the utility of adjuvant treatment with mitotane... (truncated)

Title: Current approaches to the pharmacological management of Cushing's disease.
If treatment of Cushing's disease (CD) by surgery is not successful, medical therapy is often required. Long-term use of metyrapone is limited by hirsutism and hypertension and escape because of increased ACTH levels. Although ketoconazole can normalize cortisol levels in 50%, liver toxicity limits its use. Mitotane, an adrenolytic agent, has had minimal use for benign disease. Etomidate is useful when rapid reduction in cortisol levels is needed. Cabergoline can normalize cortisol levels in CD in about one-third of patients and is well tolerated. Pasireotide can normalize cortisol levels in CD in about 25% but causes worsening of glucose tolerance... (truncated)

Title: Steroid biosynthesis inhibitors in the therapy of hypercortisolism: theory and practice.
Cushing's syndrome is a rare disease with significant morbidity and mortality. Surgical intervention represents the most effective treatment option in both adrenocorticotropin-dependent and -independent forms of hypercortisolism. It is not uncommon, however, that surgery fails to cure or control the disease. Pharmacotherapy with drugs inhibiting steroid biosynthesis can be effectively used in these cases in order to alleviate symptoms or even to induce chemical adrenalectomy. A few drugs inhibiting single or multiple steps in adrenal steroid biosynthesis can be used in clinical practice. Drugs predominantly inhibiting single enzymatic steps include the 11beta-hydroxylase inhibitor metyrapone and the 3beta-hydroxysteroid dehydrogenase inhibitor trilostane,... (truncated)

Title: Metabolic and Endocrine Toxicities of Mitotane: A Systematic Review.
Despite the pivotal role of mitotane in adrenocortical carcinoma (ACC) management, data on the endocrine toxicities of this treatment are lacking. The aim of this systematic review is to collect the available evidence on the side effects of mitotane on the endocrine and metabolic systems in both children and adults affected by adrenal carcinoma. Sixteen articles on 493 patients were included. Among the adrenal insufficiency, which is an expected side effect of mitotane, 24.5% of patients increased glucocorticoid replacement therapy. Mineralocorticoid insufficiency usually occurred late in treatment in 36.8% of patients. Thyroid dysfunction is characterized by a decrease in FT4,... (truncated)

MESH:D014439 - tyramine

Summary:

LLM Prediction Score	0.407 (normalized)
LLM Confidence Score	0.990
Golden Answer (Severity Class)	0.0 (normalized)
Prediction Error	0.407

Retrieved Context:

Title: Antidepressant drug therapy: associated risks.
Aspects of risks associated with treatment with three classes of antidepressants: tricyclic (TCA), second generation ("new") antidepressants and monoamine oxidase inhibitor (MAOI), are discussed. Moclobemide, a benzamide derivative, is a new MAOI antidepressant with reversible and preferential inhibition of the A-form of monoamine oxidase. Moclobemide is free of liver toxicity and the risk of a pressor response with tyramine-containing food is so low that strict diet restrictions are unnecessary. That MAOIs have a low incidence of side effects, particularly so called anticholinergic side effects is also true for moclobemide. A serious risk with antidepressant drugs is that the patient will... (truncated)

Title: A neglected modality in psychiatric treatment--the monoamine oxidase inhibitors.
The monoamine oxidase inhibitors are at present being used relatively infrequently in my opinion because of reports of severe and dangerous side effects such as toxic hepatocellular damage and hypertensive crises and also on account of several studies which have not given a very encouraging picture regarding the efficacy of this group of drugs. The purpose of this article is to demonstrate that this group of antidepressant drugs is very useful when the proper indications for their employment are observed and are relatively safe provided that appropriate precautions such as the avoidance of cheese and other foods high in tyramine... (truncated)

Title: Study on the hypochlolesterolemic and antioxidative effects of tyramine derivatives from the root bark of Lycium chenese Miller.
The aim of the present study was to investigate the hypocholesterolemic effect and potential of tyramine derivatives from Lycii Cortex Radicis (LCR), the root bark of lycium (Lycium chenese Miller) in reducing lipid peroxidation. The activities of enzymes, hepatic 3-hydroxy 3-methylglutaryl (HMG) CoA reductase and acyl-CoA:cholesterol acyltransferase (ACAT) and LDL oxidation were measured in vitro and animal experiments were also performed by feeding LCR extracts to rats. The test compounds employed for in vitro study were trans-N-p-coumaroyltyramine (CT) and trans-N-feruloyltyramine (FT), LCR components, N-(p-coumaroyl)serotonin (CS) and N-feruloylserotonin (FS) from safflower seeds, ferulic acid (FA) and 10-gingerol. It was observed that... (truncated)

Title: Natural Sympathomimetic Drugs: From Pharmacology to Toxicology.
Sympathomimetic agents are a group of chemical compounds that are able to activate the sympathetic nervous system either directly via adrenergic receptors or indirectly by increasing endogenous catecholamine levels or mimicking their intracellular signaling pathways. Compounds from this group, both used therapeutically or abused, comprise endogenous catecholamines (such as adrenaline and noradrenaline), synthetic amines (e.g., isoproterenol and dobutamine), trace amines (e.g., tyramine, tryptamine, histamine and octopamine), illicit drugs (e.g., ephedrine, cathinone, and cocaine), or even caffeine and synephrine. In addition to the effects triggered by stimulation of the sympathetic system, the discovery of trace amine associated receptors (TAARs) in humans... (truncated)

Title: Fermented Soybean Paste Attenuates Biogenic Amine-Induced Liver Damage in Obese Mice.
Biogenic amines are cellular components produced by the decarboxylation of amino acids; however, excessive biogenic amine production causes adverse health problems. The relationship between hepatic damage and biogenic amine levels in nonalcoholic fatty liver disease (NAFLD) remains unclear. In this study, mice were fed a high-fat diet (HFD) for 10 weeks to induce obesity, presenting early-stage of NAFLD. We administered histamine (20 mg/kg) + tyramine (100 mg/kg) via oral gavage for 6 days to mice with HFD-induced early-stage NAFLD. The results showed that combined histamine and tyramine administration increased cleaved PARP-1 and IL-1β in the liver, as well as MAO-A,... (truncated)

MESH:D003630 - daunorubicin

Summary:

LLM Prediction Score	0.406 (normalized)
LLM Confidence Score	0.990
Golden Answer (Severity Class)	0.0 (normalized)
Prediction Error	0.406

Retrieved Context:

Title: A phase II study of idarubicin (4-demethoxydaunorubicin) in advanced myeloma.
Idarubicin (IDA) is an anthracycline analog which differs from the parent compound by the substitution of a C4 methoxyl group with an hydrogen atom in the aglycone moiety. This drug has shown greater potency and activity in experimental and human leukemias and lymphomas by intravenous and oral routes of administration together with less cardiotoxicity than doxorubicin (DX) and daunorubicin (DNR). We have treated 15 patients with advanced multiple myeloma (MM) refractory or relapsed to standard chemotherapy regimens. The treatment schedule consisted of idarubicin 40 mg/m2 orally on day 1 every 3 weeks for 6-8 months. We obtained 8/14 partial response,... (truncated)

Title: Nanomicelle formulation modifies the pharmacokinetic profiles and cardiac toxicity of daunorubicin.
Treatment with daunorubicin (DNR) in acute myeloid leukemia is moderately effective and associated with significant side effects, including cardiac toxicity. We recently developed a nanomicellar formulation of DNR that specifically targets acute myeloid leukemia stem cells.

Title: Phase I clinical trial of liposomal daunorubicin in hepatocellular carcinoma complicating liver cirrhosis.
Chemotherapy has been proposed for patients with hepatocellular carcinoma (HCC) associated with well-compensated cirrhosis who are unsuitable for locoregional treatments. Anthracyclines are the most active agents against HCC, although their toxicity is often unpredictable; thus, there is a need for new active drugs with a safe toxicity profile. The liposomal formulation of the anthracycline daunorubicin has low systemic toxicity and is taken up strongly by the liver. We started a phase I study with liposomal daunorubicin (starting dose 80 mg/m2 every 21 days) in patients with hepatocellular carcinoma and Child-Pugh stage A or B liver cirrhosis. At the starting dose,... (truncated)

Title: Successful Treatment of Pediatric Acute Myeloid Leukemia Presenting with Hyperbilirubinemia Secondary to Myeloid Sarcoma: A Case Report.
Myeloid sarcoma (MS), a tumor consisting of myeloid blasts with or without maturation, occurs at anatomical sites other than the bone marrow. MS of the gastrointestinal tract presenting with jaundice in children is rare. We report the case of a 4-year-old boy with a 6-week history of symptoms of obstructive jaundice due to a peripancreatic mass compressing the common bile duct. Six weeks later, blasts were found in a peripheral smear prior to surgical biopsy; bone marrow evaluation and flow cytometry results led to a diagnosis of acute myeloid leukemia (AML) with MS. No further invasive testing or temporary drainage... (truncated)

Title: Daunorubicin, cytosine arabinoside, 6-mercaptopurine riboside, and prednisolone (DCMP) combination chemotherapy for acute myelogenous leukemia in adults.
Thirty-seven adults with acute myelogenous leukemia (AML) were treated with a combination of daunorubicin, cytosine arabinoside, 6-mercaptopurine riboside, and prednisolone (DCMP) for remission induction. Twenty-three of 37 patients (62.2%) achieved complete remission, three, partial remission and 11, failure. Patients with prior therapy responded as well as patients without it. The median survival time of the patients who received DCMP for their initial remission induction therapy was 10.3 months and that of the patients who obtained complete remission was 17 months. Complete remission occurred in 21 out of 28 patients (75%) less than 40 years old but in only two out... (truncated)

MESH:D010830 - physostigmine

Summary:

LLM Prediction Score	0.405 (normalized)
LLM Confidence Score	0.990
Golden Answer (Severity Class)	0.0 (normalized)
Prediction Error	0.405

Retrieved Context:

Title: The pharmacotherapy of Alzheimer's disease based on the cholinergic hypothesis: an update.
Alzheimer's disease (AD) is a neurodegenerative disorder with impairment of cognitive function and personality. The synaptic loss, neuronal atrophy and degeneration of cholinergic nuclei in the basal forebrain may be associated with a reduction in oxidative metabolism of glucose, a fall in acetyl CoA and ATP. Current pharmacological strategies, aimed at increasing cholinergic activity include acetylcholinesterase (AChE) inhibitors, cholinergic agonists, acetylcholine (ACh) releasers and stimulants of nerve growth factors (NGF). AChE inhibitors, physostigmine and Tacrine can slow the decline of cognitive function and memory in some patients with mild or moderate AD, if given for at least 3-6 months in... (truncated)

Title: Brain selective inhibition of acetylcholinesterase: a novel approach to therapy for Alzheimer's disease.
It could be argued that clinical experience with cholinergic drugs in the therapy of AD has not yet shown relevant symptomatic improvements. The main reasons for this might be attributed to peripheral cholinergic effects and the liver toxicity of some of these drugs, which limit their use and prevent confirmation of the cholinergic hypothesis (Gray et al., 1989). The main disadvantages of the cholinesterase inhibitors used in clinical trials are the short duration of action in the case of physostigmine and the potential for liver toxicity seen with the aminoacridine derivatives. The results presented with SDZ ENA 713 indicate that... (truncated)

Title: Investigation into the role of the cholinergic system in radiation-induced damage in the rat liver and ileum.
It has been previously shown that acetylcholine (ACh) may affect pro-inflammatory and anti-inflammatory cytokines. The role of the cholinergic system in radiation-induced inflammatory responses and tissue damage remains unclear. Therefore, the present study was designed to determine the radio-protective properties of the cholinergic system in the ileum and the liver of rats. Rats were exposed to 8-Gy single-fraction whole-abdominal irradiation and were then decapitated at either 36 h or 10 d post-irradiation. The rats were treated either with intraperitoneal physiological saline (1 ml/kg), physostigmine (80 µg/kg) or atropine (50 µg/kg) twice daily for 36 h or 10 d. Cardiac blood... (truncated)

Title: Delayed resuscitation with physostigmine increases end organ damage in alcohol intoxicated rats.
Previous studies from our laboratory have identified a role for blunted central sympathetic activation in the acute alcohol intoxication (AAI)-induced impairment of the counterregulatory response to hemorrhagic shock (HS). Immediate fluid resuscitation (FR) with acetylcholinesterase inhibitors restores the neuroendocrine and pressor responses to FR in AAI + HS. We hypothesized this intervention would remain beneficial after delay and that restoration of mean arterial blood pressure (MABP) during FR would attenuate organ damage. Male Sprague-Dawley rats received a primed constant alcohol infusion (2.5 g · kg + 0.3 g · kg · h for 15 h) or isocaloric dextrose (DEX) before... (truncated)

Title: Atropa belladonna intoxication: a case report.
Atropa belladonna is a poisonous plant also called deadly nightshade. Its roots, leaves and fruits contain alkaloids: atropine, hyocyamine and scopolamine. The risk of poisoning in children is important because of possible confusion with other berries. Atropa belladonna acute intoxication is a severe condition, it's should be considered in the presence of anti-cholinergic toxidrome, the differential diagnosis include other plants or psychoactive drugs containing atropine. The treatment is mainly symptomatic including gastrointestinal decontamination with activated charcoal. In severe cases, physostigmine can be used as an antidote. We report the case of 11 year old girl with Atropa belladonna poisoning which... (truncated)

MESH:D002746 - chlorpromazine

Summary:

LLM Prediction Score	0.654 (normalized)
LLM Confidence Score	0.990
Golden Answer (Severity Class)	0.25 (normalized)
Prediction Error	0.404

Retrieved Context:

Title: Immune responses to chlorpromazine in rats. Detection and relation to hepatotoxicity.
It has frequently been suggested that the jaundice which occurs in a small percentage of human patients following treatment with chlorpromazine is due to a hypersensitivity reaction. It has, however, proved impossible to obtain an animal model for this condition. We now show that oral administration of chlorpromazine at 25 mg/kg per day to Wistar albino rats results in formation of both humoral and secretory antibodies to chlorpromazine. We also demonstrate that the severity of the hepatic changes observed in chlorpromazine-fed animals (periportal glycogen loss and centrilobular fatty change) is enhanced by preimmunization of the rats via the gut-associated lymphoid... (truncated)

Title: Drug induced hepatitis.
During the last decade an increased incidence of the adverse reactions to drugs with liver involvement was found. The liver lesions produced by drugs constituted a constant concern. We followed the course of liver disease produced by tuberculostatics, cytostatics, antidepressive, antibiotics, narcotics, antirheumatics, by biochemical, immunological and morphological investigations (needle biopsy of the liver with optical and electronic microscopy). The pattern of the damage to the liver ranges from minimal functional changes to severe aspects with cytolysis and cholestasis. Hypersensitivity to drugs (cutaneous eruption, rash, fever, eosinophilia) were observed only in three cases. The cholestatic clinical form was the most... (truncated)

Title: Jaundice during chlorpromazine (thorazine) therapy; a histologic study of the hepatic lesions in five patients.
No abstract available.

Title: Chlorpromazine-induced cholestatic liver disease with ductopenia.
We describe a 30-year-old pregnant woman in whom cholestatic liver disease developed 16 resp. 18 days after the medication of chlorprothixeni hydrochloridum and chlorpromazine treatment in the 33rd week of pregnancy. Clinically, the course was characterized by severe jaundice lasting 10 months, fever, pruritus, high serum alkaline phosphatase level, transient aminotransferase elevation, and hypercholesterolemia. The pregnancy was terminated in the 35th week by cesarean section with the birth of a premature female newborn without any signs of liver damage. The histological examination of the mother's liver revealed ductopenia, defined by the absence of interlobular bile ducts in at least 50%... (truncated)

Title: [Cytolytic hepatitis during treatment with phenothiazines: apropos of a case].
In contrast to the well known chlorpromazine-induced cholestatic hepatitis, we report the case of a schizophrenic patient who presents a cytolytic hepatitis, without any prior hepatic disease. Mr G. was first hospitalized for depressive symptomatology. A pseudo-nevrotic schizophrenia was diagnosed. Pretherapeutic clinical and biological data were normal. A treatment with chlorpromazine 400 mg/day was given. At day 8, the patient was still anxious and began to be agitated. An increase to 500 mg/day of chlorpromazine posology and an addition of haloperidol 200 mg/day was implemented. At day 10, the following clinical symptoms appeared: 38.6 degrees C fever; headache; myalgia; epigastralgia... (truncated)

MESH:D005578 - fosfomycin

Summary:

LLM Prediction Score	0.347 (normalized)
LLM Confidence Score	0.980
Golden Answer (Severity Class)	0.75 (normalized)
Prediction Error	0.403

Retrieved Context:

Title: Acute, recurrent fosfomycin-induced liver toxicity in an adult patient with cystic fibrosis.
We report a very unusual adverse effect--fosfomycin-induced repeat liver toxicity--in a female adult with cystic fibrosis (CF).

Title: Fosfomycin: the characteristics, activity, and use in critical care.
Fosfomycin (C₃H₇O₄P) is a phosphonic acid derivative representing an epoxide class of antibiotics. The drug is a re-emerging bactericidal antibiotic with a wide range of actions against several Gram-positive and Gram-negative bacteria. Among the existing antibacterial agents, fosfomycin has the lowest molecular weight (138 Da), which is not structurally associated with other classes of antibiotics. In intensive care unit (ICU) patients, severe soft tissue infections (STIs) may lead to serious life-threatening problems, and therefore, appropriate antibiotic therapy and often intensive care management (ICM) coupled with surgical intervention are necessary. Fosfomycin is an antibiotic primarily utilized for the treatment of STIs... (truncated)

Title: Fosfomycin-induced agranulocytosis: a case report and review of the literature.
The intravenous form of fosfomycin, a bactericide antibiotic used to treat multiresistant bacterial infections is little prescribed. The most common reported adverse effects are hypokaliemia and hypernatremia. We describe a case of agranulocytosis, a rarely described side effect that may be fatal.

Title: [Efficacy of combination therapy against MRSA in Ibaraki Prefecture].
Clinical efficacies of fosfomycin (FOM) or arbekacin (ABK) plus beta-lactam combination therapies against methicillin-resistant Staphylococcus aureus (MRSA) infections were examined in 15 major hospitals in Ibaraki Prefecture. The subjects were 54 inpatients from January 1991 to April 1993, and most of them showed moderate to severe infections with underlying diseases. MRSA alone was isolated from 23 patients and the other 31 patients had polymicobes including MRSA. Pseudomonas aeruginosa was the most frequent among the co-isolated strains. The number of patients treated with FOM and cefmetazole (CMZ) was 22 (Group C) and that with FOM and flomoxef (FMOX) was 25 (Group... (truncated)

Title: Efficacy of Single Dose of Fosfomycin Versus a Five-Day Course of Ciprofloxacin in Patients With Uncomplicated Urinary Tract Infection.
Treatment has become more challenging due to an aging population, polypharmacy and high prevalence of comorbid illness, antimicrobial antibiotic allergy or sensitivity, an increase in the number of individuals with underlying immunological or structural abnormalities, as well as the frequency of multidrug-resistant infections. Many multidrug-resistant bacteria are still susceptible to nitrofurantoin and fosfomycin, two ancient medicines. Their high urine concentrations and low toxicity give them an advantage over newer medications. This study aimed to compare the efficacy of a single dose of fosfomycin versus a five-day course of ciprofloxacin in patients with uncomplicated urinary tract infections. Methodology and data collection... (truncated)

MESH:D012642 - selegiline

Summary:

LLM Prediction Score	0.403 (normalized)
LLM Confidence Score	0.990
Golden Answer (Severity Class)	0.0 (normalized)
Prediction Error	0.403

Retrieved Context:

Title: New drugs for Alzheimer's disease.
Alzheimer's disease is characterized by degeneration of various structures in the brain, with development of amyloid plaques and neurofibrillary tangles. Deficiencies of acetylcholine and other neurotransmitters also occur. Pharmacologic treatment of the disease generally seeks to correct the histopathology, the biochemical derangements or their effects. The only drugs labeled to date for the treatment of cognitive symptoms in patients with Alzheimer's disease are two cholinesterase inhibitors that prevent the breakdown of acetylcholine in the synapse. Both medications are associated with modest improvements in cognitive function. However, all benefit is lost when these drugs are discontinued; the disease then progresses to... (truncated)

Title: The combined use of human neural and liver cell lines and mouse hepatocytes improves the predictability of the neurotoxicity of selected drugs.
The cytotoxicity of amitriptyline (0-100microM), selegiline (0-4.5microM), carbamazepine (0-420microM) and paracetamol (0-10mM) was studied in metabolically competent mouse hepatocytes, metabolically incompetent human hepatoblastoma (HepG2) cells, and in neuroblastoma (SH-SY5Y) and astrocytoma (U-373 MG) cells, by using luminescence-based ATP measurement as an endpoint of cell toxicity. The aim was to evaluate the potential of the selected cell cultures to recognize metabolism-induced toxicity of the test compounds, and to predict further hepatic and neural toxicity. In SH-SY5Y cells amitriptyline was severely toxic, while selegiline and paracetamol failed to show any toxic effect, and carbamazepine was only slightly toxic at the highest concentration.... (truncated)

Title: Long-term efficacy and safety of deprenyl (selegiline) in advanced Parkinson's disease.
Twenty-two patients with advanced Parkinson's disease whose symptom fluctuations had initially responded to deprenyl supplementation were followed for 19 to 27 months on that drug. The improvement disappeared in most cases after a mean of 7-8 months but persists at latest follow-up in 5 patients. Transient or continuing abnormalities in liver function tests occurred in 9 patients.

Title: Marked effect of liver and kidney function on the pharmacokinetics of selegiline.
The pharmacokinetics of selegiline was investigated in an open study with 4 parallel groups of 10 subjects in each. Patients with liver disease, those receiving a drug that induced hepatic enzyme activity, and those with impaired kidney function were compared with control subjects.

Title: Selegiline: a molecule with innovative potential.
Monoamine oxidase B (MAO-B) inhibitors have an established role in the treatment of Parkinson's disease as monotherapy or adjuvant to levodopa. Two major recognitions were required for their introduction into this therapeutic field. The first was the elucidation of the novel pharmacological properties of selegiline as a selective MAO-B inhibitor by Knoll and Magyar and the original idea of Riederer and Youdim, supported by Birkmayer, to explore its effect in parkinsonian patients with on-off phases. In the 1960s, MAO inhibitors were mainly studied as potential antidepressants, but Birkmayer found that combined use of levodopa and various MAO inhibitors improved akinesia... (truncated)

MESH:D010423 - pentazocine

Summary:

LLM Prediction Score	0.393 (normalized)
LLM Confidence Score	0.960
Golden Answer (Severity Class)	0.0 (normalized)
Prediction Error	0.393

Retrieved Context:

Title: Can Lemborexant for Insomnia Prevent Delirium in High-Risk Patients with Pancreato-Biliary Disease after Endoscopic Procedures under Deep Sedation?
Background and aim: Pancreato-biliary patients who undergo endoscopic procedures have high potential risk of delirium. Although benzodiazepine has traditionally been used to treat insomnia, this drug might increase delirium. Lemborexant may be useful for patients with insomnia, without worsening delirium, although there is no evidence for high-risk patients with pancreato-biliary disease. The aim of this pilot study was to evaluate the safety and efficacy of lemborexant for insomnia and the frequency of delirium after endoscopic procedures under deep sedation in patients with pancreato-biliary disease. Method: This retrospective study included consecutive patients who were administered lemborexant after endoscopic procedures for pancreato-biliary... (truncated)

Title: Clinical and socio-demographic determinants of pentazocine misuse among patients with sickle cell disease, Benin City, Nigeria: a case-control study.
Opioids are a mainstay in sickle cell disease (SCD) pain care. Opioids are known to cause physical and/or psychological dependence. Increasingly, a significant number of Nigerian SCD patients ("Pentaholics") are observed to abuse pentazocine. This trend is associated with new patterns of medical complications. This study aimed to describe the local spectrum of pentazocine abuse complications and identify possible clinical and socio-demographic determinants.

Title: Cotton fever and pregnancy. A confusing clinical problem.
We discuss the case of a 24-year-old black woman at 33--34 weeks gestation, who after intravenous injection of Talwin presented with the following symptom complex: pyrexia, nausea, vomiting, shaking, chills, headache, myalgias, polyarthralgias, severe abdominal pain and "contractions." This symptomatology presents a complex diagnostic problem. Systematic laboratory evaluation eliminated more common etiologies, i.e., sub-acute bacterial endocarditis, HAA + hepatitis, placental abruption, chorioamnionitis, and urinary tract infection. The Talwin had been filtered through cotton ball. History plus exclusion of other etiologies led to the diagnosis of "cotton fever." The available literature is reviewed, and the importance of recognizing this entity when... (truncated)

Title: Use of Herbal Medicine by Pregnant Women: What Physicians Need to Know.
About 80% of the consumers worldwide use herbal medicine (HMs) or other natural products. The percentage may vary significantly (7%-55%) among pregnant women, depending upon social status, ethnicity, and cultural traditions. This manuscript discusses the most common HMs used by pregnant women, and the potential interactions of HMs with conventional drugs in some medical conditions that occur during pregnancy (e.g., hypertension, asthma, epilepsy). It also includes an examination of the characteristics of pregnant HM consumers, the primary conditions for which HMs are taken, and a discussion related to the potential toxicity of HMs taken during pregnancy. Many cultures have used... (truncated)

Title: Analysis of Mitochondrial Function in Cell Membranes as Indicator of Tissue Vulnerability to Drugs in Humans.
Drug side effects are one of the main reasons for treatment withdrawal during clinical trials. Reactive oxygen species formation is involved in many of the drug side effects, mainly by interacting with the components of the cellular respiration. Thus, the early detection of these effects in the drug discovery process is a key aspect for the optimization of pharmacological research. To this end, the superoxide formation of a series of drugs and compounds with antidepressant, antipsychotic, anticholinergic, narcotic, and analgesic properties was evaluated in isolated bovine heart membranes and on cell membrane microarrays from a collection of human tissues, together... (truncated)

MESH:D000661 - amphetamine

Summary:

LLM Prediction Score	0.392 (normalized)
LLM Confidence Score	0.990
Golden Answer (Severity Class)	0.0 (normalized)
Prediction Error	0.392

Retrieved Context:

Title: Adderall induced acute liver injury: a rare case and review of the literature.
Adderall (dextroamphetamine/amphetamine) is a widely prescribed medicine for the treatment of attention-deficit/hyperactivity disorder (ADHD) and is considered safe with due precautions. Use of prescribed Adderall without intention to overdose as a cause of acute liver injury is extremely rare, and to our knowledge no cases have been reported in the English literature. Amphetamine is an ingredient of recreational drugs such as Ecstacy and is known to cause hepatotoxicity. We describe here the case of a 55-year-old woman who developed acute liver failure during the treatment of ADHD with Adderall. She presented to the emergency room with worsening abdominal pain, malaise,... (truncated)

Title: Mechanisms underlying the hepatotoxic effects of ecstasy.
3,4-Methylenedioxymethamphetamine (MDMA or ecstasy) is a worldwide illegally used amphetamine-derived designer drug known to be hepatotoxic to humans. Jaundice, hepatomegaly, centrilobular necrosis, hepatitis and fibrosis represent some of the adverse effects caused by MDMA in the liver. Although there is irrefutable evidence of MDMA-induced hepatocellular damage, the mechanisms responsible for that toxicity remain to be thoroughly clarified. One well thought-of mechanism imply MDMA metabolism in the liver into reactive metabolites as responsible for the MDMA-elicited hepatotoxicity. However, other factors, including MDMA-induced hyperthermia, the increase in neurotransmitters efflux, the oxidation of biogenic amines, polydrug abuse pattern, and environmental features accompanying illicit... (truncated)

Title: An insight into the hepatocellular death induced by amphetamines, individually and in combination: the involvement of necrosis and apoptosis.
The liver is a vulnerable target for amphetamine toxicity, but the mechanisms involved in the drug's hepatotoxicity remain poorly understood. The purpose of the current research was to characterize the mode of death elicited by four amphetamines and to evaluate whether their combination triggered similar mechanisms in immortalized human HepG2 cells. The obtained data revealed a time- and temperature-dependent mortality of HepG2 cells exposed to 3,4-methylenedioxymethamphetamine (MDMA, ecstasy; 1.3 mM), methamphetamine (3 mM), 4-methylthioamphetamine (0.5 mM) and D-amphetamine (1.7 mM), alone or combined (1.6 mM mixture). At physiological temperature (37 °C), 24-h exposures caused HepG2 death preferentially by apoptosis, while... (truncated)

Title: Amphetamine- and methamphetamine-induced hyperthermia: Implications of the effects produced in brain vasculature and peripheral organs to forebrain neurotoxicity.
The adverse effects of amphetamine- (AMPH) and methamphetamine- (METH) induced hyperthermia on vasculature, peripheral organs and peripheral immune system are discussed. Hyperthermia alone does not produce amphetamine-like neurotoxicity but AMPH and METH exposures that do not produce hyperthermia (≥40°C) are minimally neurotoxic. Hyperthermia likely enhances AMPH and METH neurotoxicity directly through disruption of protein function, ion channels and enhanced ROS production. Forebrain neurotoxicity can also be indirectly influenced through the effects of AMPH- and METH- induced hyperthermia on vasculature. The hyperthermia and the hypertension produced by high doses amphetamines are a primary cause of transient breakdowns in the blood-brain barrier... (truncated)

Title: 4-Fluoromethamphetamine (4-FMA) induces in vitro hepatotoxicity mediated by CYP2E1, CYP2D6, and CYP3A4 metabolism.
4-Fluoromethamphetamine (4-FMA) is an amphetamine-like psychoactive substance with recognized entactogenic and stimulant effects, but hitherto unclear toxicological mechanisms. Taking into consideration that the vast majority of 4-FMA users consume this substance through oral route, the liver is expected to be highly exposed. The aim of this work was to determine the hepatotoxic potential of 4-FMA using in vitro hepatocellular models: primary rat hepatocytes (PRH), human hepatoma cell lines HepaRG and HepG2, and resorting to concentrations ranging from 37 μM to 30 mM, during a 24-h exposure. EC₅₀ values, estimated from the MTT viability assay data, were 2.21 mM, 5.59 mM...

MESH:D009020 - morphine

Summary:

LLM Prediction Score	0.389 (normalized)
LLM Confidence Score	0.990
Golden Answer (Severity Class)	0.0 (normalized)
Prediction Error	0.389

Retrieved Context:

Title: Prospective observational pharmacogenetic study of side effects induced by intravenous morphine for postoperative analgesia.
Nausea and vomiting are probably the most unpleasant side effects that occur when morphine used. A number of studies have investigated the effect on pain relief of single nucleotide polymorphisms (SNPs) in genes involved in morphine's metabolism, distribution, binding, and cellular action. The mechanism through which morphine causes nausea and vomiting has not been elucidated clearly. We examined all the reported SNPs which are associated with the complications of morphine, including SNPs in genes for phase I and phase II metabolic enzymes, ABC binding cassette drug transporters, κ and δ opioid receptors, and ion channels implicated in the postreceptor action... (truncated)

Title: Protective effect of crocin on liver toxicity induced by morphine.
Crocin, a bioactive molecule of saffron can be purely isolated from the saffron extract. It has different pharmacological effects such as antioxidant and anticancer activities. Morphine is an opioid analgesic drug. It is mainly metabolized in liver and causes devastating effects. It can increase the generation of free radicals. This study was designed to evaluate the protective role of crocin against morphine-induced toxicity in the mouse liver. In this study, various doses of crocin (12.5, 25 and 50 mg/kg) and crocin plus morphine were administered interaperitoneally once daily to 48 male mice for 20 consecutive days. These mice were randomly... (truncated)

Title: [Conventional techniques for analgesia: opioids and non-opioids. Indications, adverse effects and monitoring].
Morphine dosage must be carefully adapted in patients with renal failure or severe liver failure. The i.v. route is used for morphine titration in the post anaesthesia care unit (PACU), or for analgesia in children. Systematic (not on demand) intramuscular or subcutaneous morphine must be administered at intervals not longer than 4 hours. Dosage is best determined after i.v. titration in the PACU. Codeine, administered orally, is metabolised into morphine. Codeine has almost no effect in 7% of Caucasians and at least 15% of Asians. Nalbuphine, which has a sedative effect and a short half-life, is mainly used in children.... (truncated)

Title: Interactive toxicity of cocaine with phenobarbital, morphine and ethanol in organ cultured human and rat liver slices.
No abstract available.

Title: Methadone toxicity causing death in ten subjects starting on a methadone maintenance program.
Methadone maintenance therapy is designed to reduce the need for addicts to use heroin or other illegal opiates. Death in patients starting on such a program has not previously been documented. We report the death of 10 persons who died within days of starting a methadone maintenance program administered by general practitioners. Their bodies were subject to a full autopsy by forensic pathologists, with a full toxicological examination. The mean starting dose had been 53 mg, which had been increased to a mean of 57 mg by the final dose. Death occurred after a mean of 3 days. The mean... (truncated)

MESH:D020682 - cefixime

Summary:

LLM Prediction Score	0.111 (normalized)
LLM Confidence Score	0.980
Golden Answer (Severity Class)	0.5 (normalized)
Prediction Error	0.389

Retrieved Context:

Title: Comparative evaluation of the clinical and microbiological efficacy of co-amoxiclav vs cefixime or ciprofloxacin in bacterial exacerbation of chronic bronchitis.
In an open randomized study 218 outpatients (159 males and 59 females) ranging between 18 and 85 years of age (mean 61.9) suffering from bacterial exacerbation of chronic bronchitis have been randomly treated: 79 with co-amoxiclav (amoxicillin 875 mg+clavulanic acid 125 mg) twice daily, 69 with cefixime (400 mg) once daily, and 70 with ciprofloxacin (500 mg) twice daily for an average period of 10 days. Before treatment start, 234 bacterial strains (105 Gram-positive and 129 Gram-negative) were isolated as the cause of exacerbation; the leading pathogens were Streptococcus pneumoniae and Haemophilus spp. Eradication rates at the end of treatment... (truncated)

Title: Studies on the therapeutic effect of propolis along with standard antibacterial drug in Salmonella enterica serovar Typhimurium infected BALB/c mice.
Label="BACKGROUND" NlmCategory="BACKGROUND">Antibiotic resistance is an emerging public health problem. Centers for Disease Control and Prevention (CDC) has described antibiotic resistance as one of the world's most pressing health problems in 21st century. WHO rated antibiotic resistance as "one of the three greatest threats to human health". One important strategy employed to overcome this resistance is the use of combination of drugs. Many plants, natural extracts have been shown to exhibit synergistic response with standard drugs against microorganisms. The present study focused on the antibacterial potential of propolis in combination with the standard antibiotic Cefixime against the typhoid causing bacteria i.e.... (truncated)

Title: Azithromycin and cefixime combination versus azithromycin alone for the out-patient treatment of clinically suspected or confirmed uncomplicated typhoid fever in South Asia: a randomised controlled trial protocol.
Background: Typhoid and paratyphoid fever (enteric fever) is a common cause of non-specific febrile infection in adults and children presenting to health care facilities in low resource settings such as the South Asia. A 7-day course of a single oral antimicrobial such as ciprofloxacin, cefixime or azithromycin is commonly used for its treatment. Increasing antimicrobial resistance threatens the effectiveness of these treatment choices. We hypothesize that combined treatment with azithromycin (active mainly intracellularly) and cefixime (active mainly extracellularly) will be a better option for the treatment of typhoid fever in South Asia. Methods: This is a phase IV, international multi-centre,... (truncated)

Title: [Clinical studies on cefixime in pediatrics].
A clinical study of cefixime (CFIX), a new oral cephalosporin, was carried out to evaluate its therapeutic effectiveness on bacterial infections in children. CFIX was orally administered to 13 patients including 6 with upper respiratory tract infection (RTI), 3 with pneumonia, and 1 each with bronchitis, otitis media, skin abscess, and urinary tract infection (UTI). The daily dosage per kg bodyweight ranged from 5.1 to 17.4 mg (average: 8.7 mg), and was given in 2 or 3 divided doses per day for 3 to 10 days (average: 5.8 days). The clinical response was excellent in 4 (30.8%), good in 7... (truncated)

Title: [Clinical studies of cefixime in pediatric field].
Pharmacokinetic and clinical studies of cefixime (CFIX) in children were done and the following results were obtained. Serum and urinary concentrations of CFIX were determined in 6 children aged 5 to 14 years given single doses of 1.5 or 6.0 mg/kg. Mean serum concentrations peaked at 4 hours after the administration of either 1.5 or 6.0 mg/kg, and respective peak values were 0.71 and 4.46 micrograms/ml. Biological half-lives for the low and the high doses were 5.28 and 4.45 hours, respectively. The 12-hours urinary recovery ranged from 7.0 to 13.8% after administration of 1.5 mg/kg, and the 8-hours urinary recovery... (truncated)

MESH:D015767 - mefloquine

Summary:

LLM Prediction Score	0.487 (normalized)
LLM Confidence Score	0.990
Golden Answer (Severity Class)	0.875 (normalized)
Prediction Error	0.388

Retrieved Context:

Title: Mefloquine use, psychosis, and violence: a retinoid toxicity hypothesis.
Mefloquine use has been linked to severe gastrointestinal and neuropsychiatric adverse effects, including cognitive disturbances, anxiety, depression, psychosis, and violence. The adverse effects of the drug are thought to result from the secondary consequences of hepatocellular injury; in fact, mefloquine is known to cause a transient, anicteric chemical hepatitis. However, the mechanism of mefloquine-associated liver damage and the associated neuropsychiatric and behavioral effects of the drug are not well understood. Mefloquine and other 8-amino-quinolines are the only antimalarial drugs that target the liver-stage malaria parasites, which selectively absorb vitamin A from the host. Vitamin A is also stored mainly in... (truncated)

Title: Antimalarial drug toxicity: a review.
Malaria, caused mostly by Plasmodium falciparum and P. vivax, remains one of the most important infectious diseases in the world. Antimalarial drug toxicity is one side of the risk-benefit equation and is viewed differently depending upon whether the clinical indication for drug administration is malaria treatment or prophylaxis. Drug toxicity must be acceptable to patients and cause less harm than the disease itself. Research that leads to drug registration tends to omit two important groups who are particularly vulnerable to malaria--very young children and pregnant women. Prescribing in pregnancy is a particular problem for clinicians because the risk-benefit ratio is... (truncated)

Title: Mefloquine-induced acute hepatitis.
Mefloquine is an effective drug for prophylaxis and treatment of malaria caused by Plasmodium falciparum. It is generally well tolerated with few side effects. Minimal elevation of liver function tests has been reported after exposure to mefloquine, especially in susceptible individuals with prior abnormal liver function tests. Our patient, who had had elevated liver function tests attributed to heart failure, experienced an acute elevation of liver transaminases 6 weeks after exposure to mefloquine 250 mg/week. Cessation of the drug caused test results to return to normal. Mefloquine should be prescribed cautiously in patients with liver disease.

Title: Investigation of the mechanism of action of mefloquine and derivatives against the parasite Echinococcus multilocularis.
Alveolar echinococcosis (AE) is caused by infection with the fox tapeworm E. multilocularis. The disease affects humans, dogs, captive monkeys, and other mammals, and it is caused by the metacestode stage of the parasite growing invasively in the liver. The current drug treatment is based on non-parasiticidal benzimidazoles. Thus, they are only limitedly curative and can cause severe side effects. Therefore, novel and improved treatment options for AE are needed. Mefloquine (MEF), an antimalarial agent, was previously shown to be effective against E. multilocularis in vitro and in experimentally infected mice. However, MEF is not parasiticidal and needs improvement for... (truncated)

Title: Antimalarial drugs and the prevalence of mental and neurological manifestations: A systematic review and meta-analysis.
Background: Antimalarial drugs affect the central nervous system, but it is difficult to differentiate the effect of these drugs from that of the malaria illness. We conducted a systematic review to determine the association between anti-malarial drugs and mental and neurological impairment in humans. Methods: We systematically searched online databases, including Medline/PubMed, PsychoInfo, and Embase, for articles published up to 14th July 2016. Pooled prevalence, heterogeneity and factors associated with prevalence of mental and neurological manifestations were determined using meta-analytic techniques. Results: Of the 2,349 records identified in the initial search, 51 human studies met the eligibility criteria. The median... (truncated)

MESH:C119467 - lanthanum carbonate

Summary:

LLM Prediction Score	0.387 (normalized)
LLM Confidence Score	0.900
Golden Answer (Severity Class)	0.0 (normalized)
Prediction Error	0.387

Retrieved Context:

Title: [Efficacy and safety of lanthanum carbonate in chronic kidney disease patients with hyperphosphataemia].
Hyperphosphataemia is a frequent complication in patients with chronic kidney disease and is associated with increased cardiovascular morbidity. Lanthanum carbonate is a calcium-free phosphate binder indicated in patients with chronic kidney disease. Its digestive absorption is minimal (<0,002%). This minimal quantity is rapidly excreted by the hepatobiliary system, but there is an initial accumulation in liver and bone, which reaches a plateau within a few weeks. Long-term follow-up until six years did not show any bone or liver toxicity. Efficacy and safety of lanthanum carbonate have been assessed in randomized trials. The most common side effects reported were gastrointestinal and... (truncated)

Title: Lanthanum associated abnormal liver function tests in two patients on dialysis: a case report.
Lanthanum (La) is a phosphate binder used in patients on dialysis in the UK. As it has only recently been in use, there are no long-term data about safety of this rare metal in human subjects with renal failure on renal replacement therapy. La has not been previously reported to cause any adverse reactions apart from nausea, sickness, dialysis graft occlusion and abdominal pain. We report here La induced abnormal liver function tests in a male and a female patient of 70 and 44 years old each, on peritoneal dialysis (PD) and haemodialysis (HD) respectively, the first report of such... (truncated)

Title: New strategies in treatment of mineral and bone disorders and associated cardiovascular disease in patients with chronic kidney disease.
Mineral and bone disorders in chronic kidney disease (CKD) patients along with the use of calcium-based phosphate binders may result in vascular calcification (VC) development and associated increase in cardiovascular diseases (CVD) mortality. A few treatment modalities to control hyperphosphatemia, VC and CVD over the years have failed. Recently appeared calcium-aluminum free phosphate binders (sevelamer hydrochloride and lanthanum carbonate) have reduced hypercalcemic adverse events compared to calcium-based binders, although beneficial effects on CVD outcome to justify further widespread utilization of these agents in CKD patients are not reported so far. At present long-term safety of lanthanum administration has been challenged... (truncated)

Title: Comment on: 'Lanthanum carbonate possibly responsible for acute liver failure in a patient with Child-Pugh stage A liver cirrhosis'.
No abstract available.

Title: Role of Oxidative Stress in La₂O₃ Nanoparticle-Induced Cytotoxicity and Apoptosis in CHANG and HuH-7 Cells.
Label="INTRODUCTION" NlmCategory="BACKGROUND">Nanoparticles are extensively applied in pharmaceutical, agriculture, food processing industries, and in many other fields. In the current experiment, we have determined the mechanism of toxicity of lanthanum oxide nanoparticles (La₂O₃ NPs) on human liver cell lines.

MESH:C100146 - trimethobenzamide

Summary:

LLM Prediction Score	0.244 (normalized)
LLM Confidence Score	0.930
Golden Answer (Severity Class)	0.625 (normalized)
Prediction Error	0.381

Retrieved Context:

Title: Antioxidant and prophylactic effects of Delonix elata L., stem bark extracts, and flavonoid isolated quercetin against carbon tetrachloride-induced hepatotoxicity in rats.
Delonix elata L. (Ceasalpinaceae), is widely used by the traditional medical practitioners of Karnataka, India, to cure jaundice, and bronchial and rheumatic problems. The objective of this study was to screen the in vitro antioxidant and hepatoprotective activity of the stem bark extracts against CCl4-induced liver damage in rats. Among different stem bark extracts tested, the ethanol extract (DSE) has shown significant in vitro antioxidant property in radicals scavenging, metal chelating, and lipid peroxidation inhibition assays. HPLC analysis of the DSE revealed the presence of known antioxidant molecules, namely, gallic acid, ellagic acid, coumaric acid, quercetin, and rutin. Bioassay-guided fractionation... (truncated)

Title: Sumatriptan (subcutaneous route of administration) for acute migraine attacks in adults.
Migraine is a highly disabling condition for the individual and also has wide-reaching implications for society, healthcare services, and the economy. Sumatriptan is an abortive medication for migraine attacks, belonging to the triptan family. Subcutaneous administration may be preferable to oral for individuals experiencing nausea and/or vomiting

Title: Drug and herbal/dietary supplements-induced liver injury: A tertiary care center experience.
Drug-induced liver injury (DILI) and herbal/dietary supplements (HDS) related liver injury present unique diagnostic challenges. Collaboration between the clinician and the pathologist is required for an accurate diagnosis and management.

Title: Toxin Induced Parkinsonism and Hospitalization Related Adverse Outcome Mitigation for Parkinson's Disease: A Comprehensive Review.
Patients with Parkinson's disease admitted to the hospital have unique presentations. This unique subset of patients requires a multidisciplinary approach with a knowledge-based care team that can demonstrate awareness of complications specific to Parkinson's disease to reduce critical care admissions, morbidity, and mortality. Early recognition of toxic exposures, medication withdrawals, or medication-induced symptoms can reduce morbidity and mortality. This review can assist in the critical assessment of new or exacerbating Parkinson's disease symptoms.

Title: Current Treatments and New, Tentative Therapies for Parkinson's Disease.
Parkinson's disease (PD) is a neurodegenerative pathology, the origin of which is associated with the death of neuronal cells involved in the production of dopamine. The prevalence of PD has increased exponentially. The aim of this review was to describe the novel treatments for PD that are currently under investigation and study and the possible therapeutic targets. The pathophysiology of this disease is based on the formation of alpha-synuclein folds that generate Lewy bodies, which are cytotoxic and reduce dopamine levels. Most pharmacological treatments for PD target alpha-synuclein to reduce the symptoms. These include treatments aimed at reducing the accumulation... (truncated)

MESH:D006854 - hydrocortisone

Summary:

LLM Prediction Score	0.379 (normalized)
LLM Confidence Score	0.990
Golden Answer (Severity Class)	0.0 (normalized)
Prediction Error	0.379

Retrieved Context:

Title: Glucocorticoid Replacement for Adrenal Insufficiency and the Development of Non-Alcoholic Fatty Liver Disease.
Glucocorticoid excess is a known risk factor for non-alcoholic fatty liver disease (NAFLD). Our objective was to analyse the impact of glucocorticoid replacement therapy on the development of NAFLD and NAFLD-related fibrosis and, therefore, on cardiovascular as well as hepatic morbidity in patients with adrenal insufficiency. Two hundred and fifteen individuals with primary (<i>n</i> = 111) or secondary (<i>n</i> = 104) adrenal insufficiency were investigated for hepatic steatosis and fibrosis using the fatty liver index (FLI), NAFLD fibrosis score (NAFLD-FS), Fibrosis-4 Index (FiB-4) plus sonographic transient elastography. Results were correlated with glucocorticoid doses and cardiometabolic risk parameters. The median dose... (truncated)

Title: Immuno-inflammatory <i>in vitro</i> hepatotoxicity models to assess side effects of biologicals exemplified by aldesleukin.
Label="Introduction" NlmCategory="UNASSIGNED">Hepatotoxicity induced by immunotherapeutics is an appearing cause for immune-mediated drug-induced liver injury. Such immuno-toxic mechanisms are difficult to assess using current preclinical models and the incidence is too low to detect in clinical trials. As hepatotoxicity is a frequent reason for post-authorisation drug withdrawal, there is an urgent need for immuno-inflammatory <i>in vitro</i> models to assess the hepatotoxic potential of immuno-modulatory drug candidates. We developed several immuno-inflammatory hepatotoxicity test systems based on recombinant human interleukin-2 (aldesleukin).

Title: [Glucocorticoid deficiency of the adrenal cortex in chronic liver diseases].
The study of blood levels of hydrocortisone and ACTH in patients with chronic hepatic lesions.

Title: Comparison of hydrocortisone and prednisone in the glucocorticoid replacement therapy post-adrenalectomy of Cushing's Syndrome.
Cushing's syndrome requires glucocorticoid replacement following adrenalectomy. Based on a simplified glucocorticoid therapy scheme and the peri-operative observation, we investigated its efficacy and safety up to 6 months post-adrenalectomy in this cohort study. We found the adrenocorticotropic hormone (ACTH) levels were normal post-adrenalectomy, and sufficient to stimulate the recovery of the dystrophic adrenal cortex, thus exogenous supplemental ACTH might not be necessary. Patients were grouped by oral reception of either hydrocortisone or prednisone since day 2 post-adrenalectomy. Both groups had similar baseline responses to adrenalectomy, regarding the correction of hypertension (10/15 vs.12/19), hyperglycemia (6/11 vs. 7/10), and hypokalemia (12/12 vs.... (truncated)

Title: A double-blinded, randomized trial of hydrocortisone in acute hepatic failure. The Acute Hepatic Failure Study Group.
The Acute Hepatic Failure Study Group (AHFSG) has conducted a double-blinded, randomized evaluation of hydrocortisone in patients with acute hepatic failure. From July 1975 through August 1978, a 38-month period, 18 medical centers in the United States and one in Canada participated in this trial. A total of 64 patients were accessed and found eligible to participate in the study; two of them were subsequently eliminated from our analysis. Eighteen patients received placebo; 23 received 400 mg hydrocortisone per day, and 21 patients were administered 800 mg hydrocortisone per day. We did not observe any therapeutic effect of hydrocortisone, and... (truncated)

MESH:D004317 - doxorubicin

Summary:

LLM Prediction Score	0.753 (normalized)
LLM Confidence Score	0.990
Golden Answer (Severity Class)	0.375 (normalized)
Prediction Error	0.378

Retrieved Context:

Title: Hepatic injury during doxorubicin therapy.
Six patients with acute lymphoblastic leukemia manifested liver dysfunction related to doxorubicin hydrochloride therapy. Other causes, eg, infection, hepatitis, posttransfusion reaction, and leukemic infiltration were ruled out. There was close correlation between the administration of doxorubicin and the appearance of hepatic dysfunction. Doxorubicin may produce an idiosyncratic reaction and must be considered a drug with potential liver toxicity.

Title: Different effects of resveratrol on dose-related Doxorubicin-induced heart and liver toxicity.
The aim of the study was to evaluate the effect of resveratrol in doxorubicin-induced cardiac and hepatic toxicity. Doxorubicin was administered once a week throughout the period of 7 weeks with 1.0 or 2.0 mg/kg body weight or concomitantly with resveratrol (20 mg/kg of feed). Heart and liver toxicity was histologically and biochemically evaluated. Resveratrol protected from the heart lipid peroxidation caused by 1 mg doxorubicin and it sharply diminished superoxide dismutase activity. An insignificant effect of resveratrol on the lipid peroxidation level and the superoxide dismutase activity was observed in the hearts of rats administered a higher dose of... (truncated)

Title: Metabolic Effects of Doxorubicin on the Rat Liver Assessed With Hyperpolarized MRI and Metabolomics.
Doxorubicin (DOX) is a successful chemotherapeutic widely used for the treatment of a range of cancers. However, DOX can have serious side-effects, with cardiotoxicity and hepatotoxicity being the most common events. Oxidative stress and changes in metabolism and bioenergetics are thought to be at the core of these toxicities. We have previously shown in a clinically-relevant rat model that a low DOX dose of 2 mg kg⁻¹ week⁻¹ for 6 weeks does not lead to cardiac functional decline or changes in cardiac carbohydrate metabolism, assessed with hyperpolarized [1-¹³C]pyruvate magnetic resonance spectroscopy (MRS). We now set out to assess whether there... (truncated)

Title: Slow infusion rate of doxorubicin induces higher pro-inflammatory cytokine production.
Different infusion rates of doxorubicin (DOX) have been used for treating human malignancies. Organ toxicity after DOX infusion is a major issue in treatment disruption. However, whether different DOX infusion rates induce different toxicity is still unknown. In this study, we examined the toxicity effects of different DOX infusion rates in the early phase of organ toxicity. Thirty-six rats were randomly divided into 5-, 15-, and 30-min infusion rate groups. A single dose of DOX (8.3 mg/kg, I.V.) was administered at different infusion rates. Blood samples were collected from the femoral artery at 1, 3, 6, 9, 12, 18, 24,... (truncated)

Title: Acute liver failure due to hepatitis B virus reactivation induced by doxorubicin and cyclophosphamide chemotherapy for adjuvant treatment of breast cancer: A case report.
Acute liver failure developed in a 48-year-old woman within days after she received adjuvant chemotherapy for breast cancer. On arrival at ED, she had severe encephalopathy and jaundice. Serum analyses demonstrated coagulopathy and markedly increased transaminases. She was admitted to the ICU for supportive treatment but died several days later.

MESH:D009288 - naproxen

Summary:

LLM Prediction Score	0.750 (normalized)
LLM Confidence Score	0.990
Golden Answer (Severity Class)	0.375 (normalized)
Prediction Error	0.375

Retrieved Context:

Title: [Fulminating hepatitis after treatment with naproxen and/or disulfiram?].
A 49 year old female was started on disulfiram. Six weeks later she was given naproxen because of epicondylitis. After 5 days' treatment with naproxen she complained of nausea, anorexia and jaundice. At admission, bilirubin was 452 mumol/l, aspartate aminotransferase (ASAT) 1925 U/l, alanine aminotransferase (ALAT) 2815 U/l and prothrombin time measured as Normotest was 27%. The patient developed a fulminant hepatitis and died in hepatic coma almost four weeks after the introduction of naproxen. Postmortem examination disclosed a small liver (1,100 g) and histological examination showed massive necrosis and collapse of the lobules. The naproxen was the most probable... (truncated)

Title: Jaundice associated with naproxen.
Hepatic injury in association with naproxen therapy is described and documented by liver biopsy in one patient. Histological findings were consistent with a drug-induced hepatitis, and cessation of therapy led to reversal of the clinical and biochemical changes. Circumstantial evidence is in favour of a hypersensitivity response to the drug rather than direct hepatotoxicity. Increased awareness of clinicians of this probably rare side effect of naproxen may help prompt identification of similar cases.

Title: Diclofenac induced hepatitis. 3 cases with features of autoimmune chronic active hepatitis.
Diclofenac is a frequently prescribed nonsteroidal antiinflammatory drug (NSAID). Significant hepatotoxicity related to diclofenac may be more common than previously recognized, as three patients with diclofenac-associated hepatitis were seen by one clinician in a single year. All patients were ANA positive during the hepatitis and had histologic features of chronic active hepatitis. Two had been inappropriately treated with corticosteroids. The third patient presented more acutely with jaundice and symptoms of hepatitis. Two of the patients developed the same hepatic reaction when rechallenged with diclofenac. The third patient was changed to tiaprofenic acid, a NSAID of the same family, and redeveloped... (truncated)

Title: Fatal acute hepatitis after sequential treatment with levofloxacin, doxycycline, and naproxen in a patient presenting with acute Mycoplasma pneumoniae infection.
The diagnosis of drug-induced liver injury relies on comprehensive clinical assessments due to the absence of an established biomarker or pathognomonic features of liver histology. However, prompt recognition of a culprit drug as the cause of liver injury is the most important aspect in the management of hepatotoxicity.

Title: [Severe toxic liver failure after acute poisoning with paracetamol, ferrous sulphate and naproxen].
We present the case of 20-year-old woman intoxicated with mixed drugs, composed of paracetamol (acetaminophen), ferrous sulphate, naproxen and benzodiazepines. Acute toxic liver damage with clinical symptoms of coma resolved at the patient. Lack of the past history doesn't let to specific therapy and systemic complications. In this data we confirm, that past history, clinical symptoms and laboratory results are needed in designing a treatment strategy.

MESH:D018170 - sumatriptan

Summary:

LLM Prediction Score	0.000 (normalized)
LLM Confidence Score	0.990
Golden Answer (Severity Class)	0.375 (normalized)
Prediction Error	0.375

Retrieved Context:

Title: Sumatriptan (intranasal route of administration) for acute migraine attacks in adults.
Migraine is a highly disabling condition for the individual and also has wide-reaching implications for society, healthcare services, and the economy. Sumatriptan is an abortive medication for migraine attacks, belonging to the triptan family. Intranasal administration may be preferable to oral for individuals experiencing nausea and/or vomiting, although it is primarily absorbed in the gut, not the nasal mucosa.

Title: Sumatriptan (subcutaneous route of administration) for acute migraine attacks in adults.
Migraine is a highly disabling condition for the individual and also has wide-reaching implications for society, healthcare services, and the economy. Sumatriptan is an abortive medication for migraine attacks, belonging to the triptan family. Subcutaneous administration may be preferable to oral for individuals experiencing nausea and/or vomiting

Title: The Effect of Sumatriptan, Theophylline, Pregabalin and Caffeine on Prevention of Headache Caused By Spinal Anaesthesia (PDPH): A Systematic Review.
Spinal anaesthesia (SA) is a common method during surgery due to easy administration, rapid effects, relaxes muscles and controls pain. But, post-dural puncture headache (PDPH) is a common problem after SA that occurs in 6%-36% of SA. We assessed the effect of four common treatment drugs sumatriptan, theophylline, pregabalin and oral caffeine on prevention of PDPH. In this systematic review, all randomized clinical trials (RCTs) during January 2015 and December 2021 were searched from PubMed, Google Scholar, Web of Science, Cochrane review and Clinical Key with a specific search strategy. The article qualities were assessed by two independent authors and... (truncated)

Title: New Trends in Migraine Pharmacology: Targeting Calcitonin Gene-Related Peptide (CGRP) With Monoclonal Antibodies.
Migraine is a common neurologic disorder characterized by attacks consisting of unilateral, throbbing headache accompanied by photophobia, phonophobia, and nausea which remarkably reduces the patients' quality of life. Not migraine-specific non-steroidal anti-inflammatory drugs (NSAIDs) are effective in patients affected by mild episodic migraine whilst in moderate or severe episodic migraine and in chronic migraineurs triptans and preventative therapies are needed. Since these treatments are endowed with serious side effects and have limited effectiveness new pharmacological approaches have been investigated. The demonstrated pivotal role of calcitonin gene-related peptide (CGRP) has fostered the development of CGRP antagonists, unfortunately endowed with liver toxicity,... (truncated)

Title: Hepatobiliary Events in Migraine Therapy with Herbs-The Case of Petadolex, A Petasites Hybridus Extract.
Petadolex®, a defined butterbur extract has clinically proven efficacy against migraine attacks. However, spontaneous reports indicate cases of herbal induced liver injury (HILI). While most HILI patients presented mild serum biochemistry changes (<3 ULN, dose range 50 to 225 mg/day; treatment duration 4-730 days) nine developed severe HILI (average time-to-onset 103 days, ALT-range 3-153; AST 2-104-fold ULN). HILI cases resolved after medication withdrawal though two patients required liver transplantation. Liver biopsies revealed an inconsistent injury pattern, i.e. necrosis, macrovesicular steatosis, inflammation, cholestasis, and bile duct proliferation. Causality assessment rated 3 cases likely, 13 possible, 8 unlikely and 24 as unclassifiable/unclassified.... (truncated)

MESH:D010624 - phenelzine

Summary:

LLM Prediction Score	0.750 (normalized)
LLM Confidence Score	0.980
Golden Answer (Severity Class)	0.375 (normalized)
Prediction Error	0.375

Retrieved Context:

Title: Clinical consequences of polymorphic acetylation of basic drugs.
The clinical consequences (therapeutic and toxic) of drug acetylation polymorphism are reviewed for procainamide, hydralazine, phenelzine, isoniazid, and salicylazosulfapyridine. Genetic slow acetylators are more likely than rapid acetylators to experience the following adverse drug reactions: (1) earlier development of procainamide-induced antinuclear antibody; (2) earlier and more frequent development of procainamide-induced systemic lupus erythematosus (SLE); (3) hydralazine-induced SLE; (4) spontaneous SLE; (5) drowsiness and nausea from phenelzine; (6) cyanosis, hemolysis, and transient reticulocytosis from salicylazosulfapyridine; and (7) polyneuropathy after isoniazid therapy. The incidence of isoniazid hepatitis may, however, be more common in rapid than than in slow acetylators. Genetic slow acetylators... (truncated)

Title: A fatal case of neuroleptic malignant syndrome.
A fatal case of Neuroleptic Malignant Syndrome (NMS) affecting a middle-aged woman is presented. Most of the signs and symptoms described for NMS were present and death occurred three hours after the onset of hyperpyrexia. Laboratory and postmortem findings were non-specific. The uses and risks of Haloperidol and Phenelzine on a patient with severe liver impairment are considered. Finally, the medico-legal implications in the context of sudden unexpected death are mentioned.

Title: Severe liver injury due to phenelzine with unique hepatic deposition of extracellular material.
Severe acute and chronic hepatic damage occurred in a white man who had taken phenelzine sulfate (Nardil) 45 mg daily for 70 days. Liver biopsy showed a mixed hepatitic and cholestatic pattern with extracellular deposition of a unique homogeneous collagenous substance. Portal cirrhosis developed and has persisted. The patient was found to have a "rapid acetylator phenotype" and high rate of metabolism of antipyrine. These innate factors may have predisposed to hepatic injury due to phenelzine.

Title: Transdermal selegiline for the treatment of major depressive disorder.
Non-selective inhibition of monoamine oxidase (MAO) enzymes (ie, isoforms A and B) in the brain are associated with clinically significant antidepressant effects. In the US, the selegiline transdermal system (STS; EMSAM) is the first antidepressant transdermal delivery system to receive Food and Drug Administration (FDA) approved labeling for the treatment of major depressive disorder (MDD). Currently, the use of orally administered MAO inhibitor antidepressants (eg, phenelzine, tranylcypromine) is limited by the risk of tyramine-provoked events (eg, acute hypertension and headache, also known as the "cheese reaction") when combined with dietary tyramine. The selegiline transdermal system is the only MAOI available... (truncated)

Title: Antidepressants- and antipsychotics-induced hepatotoxicity.
Drug-induced liver injury (DILI) is a serious health burden. It has diverse clinical presentations that can escalate to acute liver failure. The worldwide increase in the use of psychotropic drugs, their long-term use on a daily basis, common comorbidities of psychiatric and metabolic disorders, and polypharmacy in psychiatric patients increase the incidence of psychotropics-induced DILI. During the last 2 decades, hepatotoxicity of various antidepressants (ADs) and antipsychotics (APs) received much attention. Comprehensive review and discussion of accumulated literature data concerning this issue are performed in this study, as hepatotoxic effects of most commonly prescribed ADs and APs are classified, described,... (truncated)

MESH:D008559 - memantine

Summary:

LLM Prediction Score	0.500 (normalized)
LLM Confidence Score	0.990
Golden Answer (Severity Class)	0.875 (normalized)
Prediction Error	0.375

Retrieved Context:

Title: Treatment of cognitive impairment in Alzheimer's disease.
In Alzheimer's disease, cognition now responds to several drugs. Anticholinesterases target the acetylcholine deficit. In mild-to-moderate Alzheimer's disease, they all provide significant benefit versus placebo on the Alzheimer's Disease Assessment ScheduleCognitive Section (ADAS-Cog), Side effects, in 5% to 15% of cases, include nausea, vomiting, diarrhea, anorexia, and dizziness. Tacrine, the leading anticholinesterase, caused frequent hepatic enzyme elevation and was withdrawn; once-daily donepezil spares the liver and improves global measures of change in severe dementia; rivasiigmine is indicated in comorbid vascular disease; while galaniamine modulates the cerebral nicotinic acetylcholine receptors that potentiate the response to acetylcholine. Alternative agents include the N-methyl-D-aspartate... (truncated)

Title: Memantine-Induced Liver Injury With Probable Causality as Assessed Using the Roussel Uclaf Causality Assessment Method (RUCAM).
Presentations of drug-induced liver injury (DILI) are highly variable. Although biochemical evidence of cholestasis is common, the extent of aminotransferase elevations and patterns of liver injury vary. Patients may be asymptomatic, and many cases may never be diagnosed. We describe a case of memantine-induced hepatotoxicity in an elderly patient with Alzheimer's dementia, with probable causality for drug-induced liver injury, as assessed using the Roussel Uclaf Causality Assessment Method (RUCAM) score.

Title: Activation of N-methyl-D-aspartate receptor regulates insulin sensitivity and lipid metabolism.
Label="RATIONALE">Although significant progress has been made in understanding the mechanisms of steatosis and insulin resistance, the physiological functions of regulators in these processes remain largely elusive. Evidence has suggested that the glutamate/N-methyl-D-aspartic acid receptor (NMDAR) axis contributes to acute lung injury, pulmonary arterial hypertension, and diabetes, but the specific metabolic contribution of the glutamate/NMDAR axis is not clear. Here we provide data at the animal, cellular, and molecular levels to support the role of the glutamate/NMDAR axis as a therapeutic target for metabolic syndrome in obesity. Methods: We examined the glutamate level in the obese mouse induced by a high-fat... (truncated)

Title: Potential Pharmacokinetic Drug⁻Drug Interaction Between Harmine, a Cholinesterase Inhibitor, and Memantine, a Non-Competitive <i>N</i>-Methyl-d-Aspartate Receptor Antagonist.
Harmine (HAR) is a beta-carboline alkaloid widely distributed in nature. It exhibits psychopharmacological effects of improving learning and memory. However, excessive dose of HAR can cause central tremor toxicity, which may be related to the glutamate system. Memantine (MEM) is a non-competitive <i>N</i>-methyl-d-aspartate receptor antagonist. It can be used for the treatment of Alzheimer's disease and also can block the neurotoxicity caused by glutamate. Therefore, combination of HAR and MEM would be meaningful and the pharmacokinetics investigation of HAR and MEM in combination is necessary. A ultra-performance liquid chromatography tandem mass spectrometry (UPLC-MS/MS) method was established and validated for the... (truncated)

Title: Memantine-induced hepatitis with cholestasis in a very elderly patient.
No abstract available.

MESH:C079703 - rufinamide

Summary:

LLM Prediction Score	0.000 (normalized)
LLM Confidence Score	0.960
Golden Answer (Severity Class)	0.375 (normalized)
Prediction Error	0.375

Retrieved Context:

Title: Role of rufinamide in the management of Lennox-Gastaut syndrome (childhood epileptic encephalopathy).
Rufinamide, a triazole derivative that is structurally distinct from currently marketed antiepileptic drugs (AEDs), is in development for the adjunctive treatment of Lennox-Gastaut syndrome (LGS) in children and adults. Rufinamide is well absorbed after oral administration, demonstrates low protein binding, and is metabolized by enzymatic hydrolysis without involvement of cytochrome P450 enzymes, conferring a low drug interaction potential. In a randomized, double-blind trial involving 138 adult and pediatric patients with LGS, compared with placebo, rufinamide 45 mg/kg/day resulted in significantly superior reductions in drop attacks (median change -42.5% vs +1.4% with placebo) and total seizures (-32.1% vs -11.7% with placebo),... (truncated)

Title: Use of antiepileptic drugs in hepatic and renal disease.
The use of antiepileptic drugs in patients with renal or hepatic disease is common in clinical practice. Since the liver and kidney are the main organs involved in the elimination of most drugs, their dysfunction can have important effects on the disposition of antiepileptic drugs. Renal or hepatic disease can prolong the elimination of the parent drug or an active metabolite leading to accumulation and clinical toxicity. It can also affect the protein binding, distribution, and metabolism of a drug. The protein binding of anionic acidic drugs, such as phenytoin and valproate, can be reduced significantly by renal failure, causing... (truncated)

Title: An Updated Overview on Therapeutic Drug Monitoring of Recent Antiepileptic Drugs.
Given the distinctive characteristics of both epilepsy and antiepileptic drugs (AEDs), therapeutic drug monitoring (TDM) can make a significant contribution to the field of epilepsy. The measurement and interpretation of serum drug concentrations can be of benefit in the treatment of uncontrollable seizures and in cases of clinical toxicity; it can aid in the individualization of therapy and in adjusting for variable or nonlinear pharmacokinetics; and can be useful in special populations such as pregnancy. This review examines the potential for TDM of newer AEDs such as eslicarbazepine acetate, felbamate, gabapentin, lacosamide, lamotrigine, levetiracetam, perampanel, pregabalin, rufinamide, retigabine, stiripentol, tiagabine,... (truncated)

Title: Pharmacological diversity amongst approved and emerging antiseizure medications for the treatment of developmental and epileptic encephalopathies.
Developmental and epileptic encephalopathies (DEEs) are rare neurodevelopmental disorders characterised by early-onset and often intractable seizures and developmental delay/regression, and include Dravet syndrome and Lennox-Gastaut syndrome (LGS). Rufinamide, fenfluramine, stiripentol, cannabidiol and ganaxolone are antiseizure medications (ASMs) with diverse mechanisms of action that have been approved for treating specific DEEs. Rufinamide is thought to suppress neuronal hyperexcitability by preventing the functional recycling of voltage-gated sodium channels from the inactivated to resting state. It is licensed for adjunctive treatment of seizures associated with LGS. Fenfluramine increases extracellular serotonin levels and may reduce seizures via activation of specific serotonin receptors and positive... (truncated)

Title: Therapeutic Drug Monitoring of the Newer Anti-Epilepsy Medications.
In the past twenty years, 14 new antiepileptic drugs have been approved for use in the United States and/or Europe. These drugs are eslicarbazepine acetate, felbamate, gabapentin, lacosamide, lamotrigine, levetiracetam, oxcarbazepine, pregabalin, rufinamide, stiripentol, tiagabine, topiramate, vigabatrin and zonisamide. In general, the clinical utility of therapeutic drug monitoring has not been established in clinical trials for these new anticonvulsants, and clear guidelines for drug monitoring have yet to be defined. The antiepileptic drugs with the strongest justifications for drug monitoring are lamotrigine, oxcarbazepine, stiripentol, and zonisamide. Stiripentol and tiagabine are strongly protein bound and are candidates for free drug monitoring.... (truncated)

MESH:D017829 - granisetron

Summary:

LLM Prediction Score	0.000 (normalized)
LLM Confidence Score	0.990
Golden Answer (Severity Class)	0.375 (normalized)
Prediction Error	0.375

Retrieved Context:

Title: Controlling emesis related to cancer therapy.
Combinations of dopamine antagonists or high-dose metoclopramide with steroids can provide complete control of chemotherapy-related nausea and vomiting in up to 60-70% of patients undergoing high-dose cisplatin-based chemotherapy. High-dose metoclopramide probably acts as a 5-HT3 receptor antagonist, but because of its dopamine-receptor antagonism it is the cause of extrapyramidal side-effects. These compounds, and the agents used in combination with them, tend to cause sedation, an undesirable effect in the outpatient setting. Specific 5-HT3 receptor antagonists (ondansetron, granisetron, tropisetron) give a similar control of chemotherapy related nausea and vomiting, with minimum side-effects. These drugs can cause headaches and constipation and some... (truncated)

Title: Intestinal Microbiota Mediates the Susceptibility to Polymicrobial Sepsis-Induced Liver Injury by Granisetron Generation in Mice.
Sepsis-induced liver injury is recognized as a key problem in intensive care units. The gut microbiota has been touted as an important mediator of liver disease development; however, the precise roles of gut microbiota in regulating sepsis-induced liver injury are unknown. Here, we aimed to investigate the role of the gut microbiota in sepsis-induced liver injury and the underlying mechanism. Cecal ligation and puncture (CLP) was used to induce polymicrobial sepsis and related liver injury. Fecal microbiota transplantation (FMT) was used to validate the roles of gut microbiota in these pathologies. Metabolomics analysis was performed to characterize the metabolic profile... (truncated)

Title: Effective Dose of Ramosetron for Prophylaxis of Postoperative Nausea and Vomiting in High-Risk Patients.
Postoperative nausea and vomiting (PONV) are common adverse events with an incidence of up to 80% in high-risk patients. Ramosetron, a selective 5-HT3 receptor antagonist, is widely used to prevent PONV. The purpose of this study was to evaluate the effective dose of ramosetron for the prevention of PONV in high-risk patients.

Title: Effect of total intravenous anaesthesia and prophylactic ramosetron on postoperative nausea and vomiting after thyroidectomy: A prospective, randomized controlled study.
To investigate the effect of combined prophylactic ramosetron and total intravenous anaesthesia (TIVA) on postoperative nausea and vomiting (PONV), compared with sevoflurane anaesthesia without prophylactic antiemetics, in female patients undergoing thyroidectomy.

Title: Palonosetron versus older 5-HT3 receptor antagonists for nausea prevention in patients receiving chemotherapy: a multistudy analysis.
No clinical standard currently exists for the optimal management of nausea induced by emetogenic chemotherapy, 7particularly delayed nausea.

MESH:D015784 - betaxolol

Summary:

LLM Prediction Score	0.000 (normalized)
LLM Confidence Score	0.960
Golden Answer (Severity Class)	0.375 (normalized)
Prediction Error	0.375

Retrieved Context:

Title: Drug-induced liver injury after switching from tamoxifen to anastrozole in a patient with a history of breast cancer being treated for hypertension and diabetes.
Anastrozole is a selective non-steroidal aromatase inhibitor that blocks the conversion of androgens to estrogens in peripheral tissues. It is used as adjuvant therapy for early-stage hormone-sensitive breast cancer in postmenopausal women. Significant side effects of anastrozole include osteoporosis and increased levels of cholesterol. To date, seven case reports on anastrozole hepatotoxicity have been published. We report the case of an 81-year-old woman with a history of breast cancer, arterial hypertension, type 2 diabetes mellitus, hyperlipidemia, and chronic renal insufficiency. Four days after switching hormone therapy from tamoxifen to anastrozole, icterus developed along with a significant increase in liver enzymes... (truncated)

Title: Phase I Clinical Study of the Dietary Supplement, Agaricus blazei Murill, in Cancer Patients in Remission.
Although many cancer patients use complementary and alternative medicine, including Agaricus blazei Murill (ABM), safety is not yet well understood. Cancer survivors took 1.8, 3.6, or 5.4 g ABM granulated powder (Kyowa Wellness Co., Ltd., Tokyo, Japan) per day orally for 6 months. Adverse events were defined by subjective/objective symptoms and laboratory data according to the National Cancer Institute Common Terminology Criteria for Adverse Events version 3.0 (NCI-CTCAE v3.0). Seventy-eight patients were assessed for safety of ABM (30/24/24 subjects at 1/2/3 packs per day, resp.). Adverse events were observed in 9 patients (12%). Most were digestive in nature such as... (truncated)

Title: Fatal rhabdomyolysis in a patient with liver cirrhosis after switching from simvastatin to fluvastatin.
HMG-CoA reductase inhibitors (statins) are widely used to treat hypercholesterolemia. Among the adverse effects associated with these drugs are statin-associated myopathies, ranging from asymptomatic elevation of serum creatine kinase to fatal rhabdomyolysis. Fluvastatin-induced fatal rhabdomyolysis has not been previously reported. We describe here a patient with liver cirrhosis who experienced fluvastatin-induced fatal rhabdomyolysis. This patient had been treated with simvastatin (20 mg/day) for coronary artery disease and was switched to fluvastatin (20 mg/day) 10 days before admission. He was also taking aspirin, betaxolol, candesartan, lactulose, and entecavir. Rhabdomyolysis was complicated and continued to progress. He was treated with massive hydration,... (truncated)

Title: Comedication with interacting drugs predisposes amiodarone users in cardiac and surgical intensive care units to acute liver injury: A retrospective analysis.
Risk factors and underlying mechanisms for liver injury associated with amiodarone remain elusive. This study aimed to investigate the drug-related covariates for acute liver injury by amiodarone-an intriguing compound of high lipophilicity, with a long half-life and notable efficacy.The medical, pharmacy, and laboratory records of new amiodarone users admitted to the cardiac or surgical intensive care units of a medical center were examined retrospectively. A Cox regression model with time-varying dose-related variables of amiodarone was utilized to estimate the hazard ratio (HR) of amiodarone-associated liver injury while adjusting for concomitant therapy and relevant covariates.Of the 131 eligible patients among 6,572... (truncated)

Title: Corrigendum to "Drug-induced liver injury after switching from tamoxifen to anastrozole in a patient with a history of breast cancer being treated for hypertension and diabetes".
[This corrects the article DOI: 10.1177/2040622320964152.].

MESH:D015649 - pentostatin

Summary:

LLM Prediction Score	0.000 (normalized)
LLM Confidence Score	0.940
Golden Answer (Severity Class)	0.375 (normalized)
Prediction Error	0.375

Retrieved Context:

Title: Mangiferin, a natural xanthone, protects murine liver in Pb(II) induced hepatic damage and cell death via MAP kinase, NF-κB and mitochondria dependent pathways.
One of the most well-known naturally occurring environmental heavy metals, lead (Pb) has been reported to cause liver injury and cellular apoptosis by disturbing the prooxidant-antioxidant balance via oxidative stress. Several studies, on the other hand, reported that mangiferin, a naturally occurring xanthone, has been used for a broad range of therapeutic purposes. In the present study, we, therefore, investigated the molecular mechanisms of the protective action of mangiferin against lead-induced hepatic pathophysiology. Lead [Pb(II)] in the form of $Pb(NO_3)_2$ (at a dose of 5 mg/kg body weight, 6 days, orally) induced oxidative stress, hepatic dysfunction and cell death in... (truncated)

Title: Obeticholic acid attenuates human immunodeficiency virus/alcohol metabolism-induced pro-fibrotic activation in liver cells.
The morbidity and mortality of human immunodeficiency virus (HIV)-infection is often associated with liver disease, which progresses slowly into severe liver dysfunction. There are multiple insults which exacerbate HIV-related liver injury, including HIV-associated dysregulation of lipid metabolism and fat turnover, co-infections with hepatotropic viruses and alcohol abuse. As we reported before, exposure of hepatocytes to HIV and alcohol metabolites causes high oxidative stress, impairs proteasomal and lysosomal functions leading to accumulation of HIV in these cells, which end-ups with apoptotic cell death and finally promotes development of liver fibrosis.

Title: Interaction of <i>Garcinia cambogia</i> (Gaertn.) Desr. and Drugs as a Possible Mechanism of Liver Injury: The Case of Montelukast.
Overweight and obesity prevalence has increased worldwide. Apart from conventional approaches, people also resort to botanical supplements for reducing body weight, although several adverse events have been associated with these products. In this context, the present study aimed at evaluating the toxicity of <i>Garcinia cambogia</i>-based products and shedding light on the mechanisms involved. The suspected hepatotoxic reactions related to <i>G. cambogia</i>-containing products collected within the Italian Phytovigilance System (IPS) were examined. Then, an in vitro study was performed to evaluate the possible mechanisms responsible for the liver toxicity, focusing on the modulation of oxidative stress and Nrf2 expression. From March... (truncated)

Title: The Modulatory Role of CYP3A4 in Dictamnine-Induced Hepatotoxicity.
Dictamni Cortex (DC) has been reported to be associated with acute hepatitis in clinic and may lead to a selective sub-chronic hepatotoxicity in rats. Nevertheless, the potent toxic ingredient and the underlying mechanism remain unknown. Dictamnine (DTN), the main alkaloid from DC, possesses a furan ring which was suspected of being responsible for hepatotoxicity via metabolic activation primarily by CYP3A4. Herein, the present study aimed to evaluate the role of CYP3A4 in DTN-induced liver injury. The <i>in vitro</i> results showed that the EC₅₀ values in primary human hepatocytes (PHH), L02, HepG2 and NIH3T3 cells were correlated with the CYP3A4 expression... (truncated)

Title: Hepatic NF-κB-Inducing Kinase and Inhibitor of NF-κB Kinase Subunit α Promote Liver Oxidative Stress, Ferroptosis, and Liver Injury.
Drug-induced hepatotoxicity limits development of new effective medications. Drugs and numerous endogenous/exogenous agents are metabolized/detoxified by hepatocytes, during which reactive oxygen species (ROS) are generated as a by-product. ROS has broad adverse effects on liver function and integrity, including damaging hepatocyte proteins, lipids, and DNA and promoting liver inflammation and fibrosis. ROS in concert with iron overload drives ferroptosis. Hepatic nuclear factor kappa B (NF-κB)-inducing kinase (NIK) is aberrantly activated in a broad spectrum of liver disease. NIK phosphorylates and activates inhibitor of NF-κB kinase subunit alpha (IKKα), and the hepatic NIK/IKKα cascade suppresses liver regeneration. However, the NIK/IKKα pathway... (truncated)

MESH:C083544 - lepirudin

Summary:

LLM Prediction Score	0.000 (normalized)
LLM Confidence Score	0.960
Golden Answer (Severity Class)	0.375 (normalized)
Prediction Error	0.375

Retrieved Context:

Title: Heparin protects against septic mortality via apoE-antagonism.

Apolipoprotein E (apoE), a component of plasma lipoproteins, increases septic mortality in a rodent model of sepsis, presumably by enhancing lipid antigen presentation to antigen-presenting cells via the low-density lipoprotein receptor (LDLR). Downstream, this culminates in natural killer T (NKT) cell activation and cytokine secretion. To determine whether apoE antagonism would protect against septic mortality in mice, apoE-LDLR binding was antagonized using heparin, which can inhibit apoE's LDLR-binding site.

MESH:D017298 - bisoprolol

Summary:

LLM Prediction Score	0.000 (normalized)
LLM Confidence Score	0.950
Golden Answer (Severity Class)	0.375 (normalized)
Prediction Error	0.375

Retrieved Context:

Title: Ramipril-associated cholestasis in the setting of recurrent drug-induced liver injury.
Aim: Angiotensin-converting enzyme inhibitors (ACEIs) are commonly used to treat hypertension. Although generally well tolerated, the adverse effects of ACEIs include hypotension, cough, acute kidney injury and hyperkalemia. Rare reports of ACEI-induced hepatotoxicity have been described, most notably a cholestatic pattern of injury related to captopril. A 67-year-old male presented to the emergency department with a three-week history of jaundice, pruritis and weakness. Eight weeks before, he began taking ramipril and clopidogrel. His past medical history was significant for previous acute cholestatic liver injury approximately 20 years earlier, which was attributed to methimazole. Abnormal blood work demonstrated aspartate aminotransferase (AST)... (truncated)

Title: [HEPATOCELLULAR TOXICITY IN THE BACKGROUND OF AMIODARON-INDUCED THYROID DYSFUNCTION IN PATIENTS WITH ATRIAL FIBRILLATION].
The purpose of the work was to establish the frequency and conditions in which structural and functional changes of the liver might occur in case of long-term amiodarone use, depending on thyroid dysfunction. The study included 80 patients with cardiosclerosis with atrial fibrillation (AF). The patients were assigned to: group I (n=60) - received amiodarone at a maintenance dose for one year (on background of basic therapy); control group (CG) - patients (n=20) who received on the background of basic therapy digoxin and bisoprolol. Biochemical tests were conducted: fT3, thyroid-stimulating hormone (TSH), fT4, anti-TPO Ab, transaminases (ALT, AST), alkaline phosphatase... (truncated)

Title: Pharmacokinetics of bisoprolol during repeated oral administration to healthy volunteers and patients with kidney or liver disease.
The pharmacokinetics of bisoprolol were investigated following oral administration of 10mg once daily for 7 days in 8 healthy subjects, in 14 patients with different degrees of renal impairment and in 18 patients with liver disease. In healthy subjects peak and trough steady-state concentrations of 52 micrograms/L and 11 micrograms/L, respectively, an elimination half-life of 10.0 hours and total body clearance of 14.2 L/h were observed. 5.21 mg/24 hours of unchanged bisoprolol were recovered following urinary excretion during the dosage interval. In 11 patients with renal impairment (mean CLCR = 28 +/- 5 ml/min/1.72m2) half-life was prolonged to 18.5 hours,... (truncated)

Title: Amiodarone-induced reversible and irreversible hepatotoxicity: two case reports.
Amiodarone is a highly effective treatment for supraventricular and ventricular tachyarrhythmia; however, it could be associated with several serious adverse effects, including liver injury.

Title: Anastrozole-related acute hepatitis with autoimmune features: a case report.
Two cases of acute hepatitis occurring during treatment with anastrozole have previously been reported, but the underlying mechanisms of liver injury are still uncertain. We report the case of anastrozole-related acute hepatitis with some autoimmune features.

MESH:D000077336 - caspofungin

Summary:

LLM Prediction Score	0.500 (normalized)
LLM Confidence Score	0.990
Golden Answer (Severity Class)	0.875 (normalized)
Prediction Error	0.375

Retrieved Context:

Title: Hepatotoxicity of Antimycotics Used for Invasive Fungal Infections: In Vitro Results.
<i>Purpose.</i> Drug-induced liver injury (DILI) is the most common cause of liver injury and a serious clinical problem; antimycotics are involved in approximately 3% of all DILI cases. The hepatotoxicity of many drugs, including the antimycotics, is poorly screened in human models. <i>Methods.</i> In a standardized assay the cytotoxicity on hepatocytes of different concentrations (<i>C</i>max, 5x <i>C</i>max, and 10x <i>C</i>max) of the antimycotics used for systemic infections was tested. Anidulafungin (ANI), liposomal amphotericerin B (L-AmB), caspofungin (CASPO), fluconazole (FLUCO), and voriconazole (VORI) were incubated with HepG2/C3A cells. After incubation, the viability of cells (XTT test, LDH release, trypan blue staining),... (truncated)

Title: Caspofungin versus micafungin in the incidence of hepatotoxicity in patients with normal to moderate liver failure.
One of the major adverse events of caspofungin and micafungin is hepatotoxicity, however, there are few reports compared the incidence of hepatotoxicity between caspofungin and micafungin. Herein, the primary objective of this study was to compare the incidence of hepatotoxicity between caspofungin and micafungin treatments for patients with fungal or suspected fungal infection.

Title: Echinocandins: A ray of hope in antifungal drug therapy.
Invasive fungal infections are on the rise. Amphotericin B and azole antifungals have been the mainstay of antifungal therapy so far. The high incidence of infusion related toxicity and nephrotoxicity with amphotericin B and the emergence of fluconazole resistant strains of Candida glabrata egged on the search for alternatives. Echinocandins are a new class of antifungal drugs that act by inhibition of beta (1, 3)-D- glucan synthase, a key enzyme necessary for integrity of the fungal cell wall.Caspofungin was the first drug in this class to be approved. It is indicated for esophageal candidiasis, candidemia, invasive candidiasis, empirical therapy in... (truncated)

Title: Switching to anidulafungin from caspofungin in cancer patients in the setting of liver dysfunction is associated with improvement of liver function tests.
Anidulafungin does not undergo hepatic metabolism like the other echinocandins. Therefore, there is a perception that anidulafungin may be less hepatotoxic or less likely to exacerbate existing liver damage. This has not been substantiated in the literature.

Title: Antifungal Drugs and Drug-Induced Liver Injury: A Real-World Study Leveraging the FDA Adverse Event Reporting System Database.
Aims: We aimed to estimate the risk of drug-induced liver injury (DILI) from various antifungal treatments with azoles and echinocandins causing in real-world practice. Methods: We performed disproportionality and Bayesian analyses based on data from the first quarter in 2004 to the third quarter in 2021 in the Food and Drug Administration Adverse Event Reporting System to characterize the signal differences of antifungal drugs-related DILI. We also compared the onset time and mortality differences of different antifungal agents. Results: A total of 2943 antifungal drugs-related DILI were identified. Affected patients tended to be aged >45 years (51.38%), with more males than... (truncated)

MESH:D005702 - galantamine

Summary:

LLM Prediction Score	0.000 (normalized)
LLM Confidence Score	0.980
Golden Answer (Severity Class)	0.375 (normalized)
Prediction Error	0.375

Retrieved Context:

Title: Galanthamine.
Galanthamine is a selective acetylcholinesterase inhibitor which has shown potential for the treatment of Alzheimer's disease. Galanthamine is selective for acetylcholinesterase versus butyrylcholinesterase; however, the drug produces greater enzyme inhibition in human erythrocytes than in human brain tissue. Galanthamine attenuates drug-and lesion-induced cognitive deficits in animal models of learning and memory. Preliminary results in patients with Alzheimer's disease have reported galanthamine to be associated with a reduction in cognitive deterioration on some neuropsychiatric rating scales. Nausea and vomiting are the most commonly reported adverse effects; liver toxicity has not been reported to date.

Title: Treatment of dementia with neurotransmission modulation.
The prevalence of dementia is growing in developed countries where elderly patients are increasing in numbers. Neurotransmission modulation is one approach to the treatment of dementia. Cholinergic precursors, anticholinesterases, nicotine receptor agonists and muscarinic M(2) receptor antagonists are agents that enhance cholinergic neurotransmission and that depend on having some intact cholinergic innervation to be effective in the treatment of dementia. The cholinergic precursor choline alfoscerate may be emerging as a potential useful drug in the treatment of dementia, with few adverse effects. Of the anticholinesterases, donepezil, in addition to having a similar efficacy to tacrine in mild-to-moderate Alzheimer's disease (AD),... (truncated)

Title: Treatment of cognitive impairment in Alzheimer's disease.
In Alzheimer's disease, cognition now responds to several drugs. Anticholinesterases target the acetylcholine deficit. In mild-to-moderate Alzheimer's disease, they all provide significant benefit versus placebo on the Alzheimer's Disease Assessment ScheduleCognitive Section (ADAS-Cog), Side effects, in 5% to 15% of cases, include nausea, vomiting, diarrhea, anorexia, and dizziness. Tacrine, the leading anticholinesterase, caused frequent hepatic enzyme elevation and was withdrawn; once-daily donepezil spares the liver and improves global measures of change in severe dementia; rivasiigmine is indicated in comorbid vascular disease; while galaniamine modulates the cerebral nicotinic acetylcholine receptors that potentiate the response to acetylcholine. Alternative agents include the N-methyl-D-aspartate... (truncated)

Title: Mushroom Polysaccharides as Potential Candidates for Alleviating Neurodegenerative Diseases.
Neurodegenerative diseases (NDs) are a widespread and serious global public health burden, particularly among the older population. At present, effective therapies do not exist, despite the increasing understanding of the different mechanisms of NDs. In recent years, some drugs, such as galantamine, entacapone, riluzole, and edaravone, have been proposed for the treatment of different NDs; however, they mainly concentrate on symptom management and confer undesirable side effects and adverse reactions. Therefore, there is an urgent need to find novel drugs with fewer disadvantages and higher efficacy for the treatment of NDs. Mushroom polysaccharides are macromolecular complexes with multi-targeting bioactivities, low... (truncated)

Title: [Anti-dementia drugs for Alzheimer disease in present and future].
Alzheimer disease(AD) is characterized as neurodegenerative disease showing impairment of cognitive function, death of neuronal cells, numerous numbers of senile plaques and tangle of neurofilaments. There are two different hypotheses that neurotoxicity of aggregated amyloid beta protein(A beta) and hyperphosphorylation of tau protein are the causes of AD. The dysfunction of cholinergic neuronal system is observed in the early stage of AD. Therefore, the strategy to increase of acetylcholine (ACh) level in brain by using ACh esterase inhibitor is mainstream in the present. We have tacrine, donepezil, rivastigmine and galantamine. Tacrine, the first drug for AD, is replaced by other... (truncated)

MESH:C121345 - desloratadine

Summary:

LLM Prediction Score	0.000 (normalized)
LLM Confidence Score	0.980
Golden Answer (Severity Class)	0.375 (normalized)
Prediction Error	0.375

Retrieved Context:

Title: Safety of Cyproheptadine, an Orexigenic Drug. Analysis of the French National Pharmacovigilance Data-Base and Systematic Review.
Objectives: Cyproheptadine is a first-generation H1-antihistamine drug first that was distributed in the 1960s. While its orexigenic effect was observed early, cyproheptadine is not yet authorized for this indication in all countries today. There is an increasing medical interest and demand for the orexigenic effect of cyproheptadine, especially in children with poor appetite. As cyproheptadine might be evaluated in future clinical trials, we wanted to assess its safety profile. Methods: Using the French national pharmacovigilance database, we retrospectively analyzed all pediatric and adult reports of adverse effects of cyproheptadine recorded since its first distribution in France. Next, we performed a... (truncated)

Title: H1 antihistamines: current status and future directions.
In this review, we compare and contrast the clinical pharmacology, efficacy, and safety of first-generation H1 antihistamines and second-generation H1 antihistamines. First-generation H1 antihistamines cross the blood-brain barrier, and in usual doses, they potentially cause sedation and impair cognitive function and psychomotor performance. These medications, some of which have been in use for more than 6 decades, have never been optimally investigated. Second-generation H1 antihistamines such as cetirizine, desloratadine, fexofenadine, levocetirizine, and loratadine cross the blood-brain barrier to a significantly smaller extent than their predecessors. The clinical pharmacology, efficacy, and safety of these medications have been extensively studied. They are... (truncated)

Title: Focus on the cetirizine use in clinical practice: a reappraisal 30 years later.
Antihistamines are currently one of the most commonly administered categories of drugs. They are used to treat symptoms that are secondary to histamine release, which is typical of certain allergic conditions, including rhinitis, conjunctivitis, asthma, urticaria, and anaphylaxis. Cetirizine belongs to the second-generation family, so, it is very selective for peripheral H1 receptors, is potent and quickly relieves symptoms, exerts additional anti-allergic/anti-inflammatory effects, and is usually well-tolerated. It has been marketed 30 years ago. In these years, a remarkable body of evidence has been built. The current review provides a practical update on the use of cetirizine in clinical practice.

Title: Prediction of drug-induced liver injury and cardiotoxicity using chemical structure and in vitro assay data.
Drug-induced liver injury (DILI) and cardiotoxicity (DICT) are major adverse effects triggered by many clinically important drugs. To provide an alternative to in vivo toxicity testing, the U.S. Tox21 consortium has screened a collection of ~10K compounds, including drugs in clinical use, against >70 cell-based assays in a quantitative high-throughput screening (qHTS) format. In this study, we compiled reference compound lists for DILI and DICT and compared the potential of Tox21 assay data with chemical structure information in building prediction models for human in vivo hepatotoxicity and cardiotoxicity. Models were built with four different machine learning algorithms (e.g., Random Forest,... (truncated)

Title: Lamotrigine hypersensitivity syndrome and spiking Fever.
We report a case of a 26 year old woman with rash, lymphadenopathy, liver enzyme abnormalities and spiking fever. She was diagnosed with drug-induced hypersensitivity syndrome (DHS) to lamotrigine. Spiking fever in relation to drug-induced hypersensitivity syndrome has not earlier been described in adults. Spiking fever is an important symptom of the wide spectrum of disease presentation. The syndrome is commonly referred to as either Drug Rash with Eosinophilia and Systemic Symptoms (DRESS) or DHS. In accord with previous authors we see both syndromes as two ends of a spectrum, with a wide range of symptoms and presentations. Therefore we... (truncated)

MESH:D000077362 - verteporfin

Summary:

LLM Prediction Score	0.000 (normalized)
LLM Confidence Score	0.970
Golden Answer (Severity Class)	0.375 (normalized)
Prediction Error	0.375

Retrieved Context:

Title: Increased CHCHD2 expression promotes liver fibrosis in nonalcoholic steatohepatitis via Notch/osteopontin signaling.
Nonalcoholic steatohepatitis (NASH) is closely related to liver fibrosis. The role of coiled-coil-helix-coiled-coil-helix domain-containing 2 (CHCHD2) in NASH remains unknown. CHCHD2's functions as a transcription factor have received much less attention than those in mitochondria. Herein, we systematically characterized the role of CHCHD2 as a transcription factor by chromatin immunoprecipitation sequencing and found its target genes were enriched in nonalcoholic fatty liver disease (NAFLD). Overall, CHCHD2 expression was found to be increased in the livers of patients with NAFLD and those of NASH mice. In line with these findings, CHCHD2 deficiency ameliorated NASH- and thioacetamide-induced liver fibrosis, whereas hepatocyte-specific CHCHD2... (truncated)

Title: COL1A1 expression induced by overexpression of both a 15-amino acid peptide from the fibrinogen domain of tenascin-X and integrin α11 in LX-2 cells.
Extracellular matrix tenascin‑X (TNX) is the largest member of the tenascin family. Our previous study demonstrated that TNX was involved in hepatic dysfunction, including fibrosis, in mice that were administered a high‑fat and high‑cholesterol diet with high levels of phosphorus and calcium. The present study investigated whether overexpression of both the fibrinogen domain of TNX (TNX‑FG) and integrin α11, one of the TNX cell surface receptors, induces <i>in vitro</i> fibrosis in LX‑2 human hepatic stellate cells. Overexpression of both a 15‑amino acid peptide (hTNX‑FGFFFF) derived from the TNX‑FG domain and integrin α11 induced the expression of type I collagen α1 chain (COL1A1).... (truncated)

Title: Increased YAP Activation Is Associated With Hepatic Cyst Epithelial Cell Proliferation in ARPKD/CHF.
Autosomal recessive polycystic kidney disease/congenital hepatic fibrosis (ARPKD/CHF) is a rare but fatal genetic disease characterized by progressive cyst development in the kidneys and liver. Liver cysts arise from aberrantly proliferative cholangiocytes accompanied by pericystic fibrosis and inflammation. Yes-associated protein (YAP), the downstream effector of the Hippo signaling pathway, is implicated in human hepatic malignancies such as hepatocellular carcinoma, cholangiocarcinoma, and hepatoblastoma, but its role in hepatic cystogenesis in ARPKD/CHF is unknown. We studied the role of the YAP in hepatic cyst development using polycystic kidney (PCK) rats, an orthologous model of ARPKD, and in human ARPKD/CHF patients. The liver... (truncated)

Title: Hepatic connective tissue growth factor expression and regulation differ between non-steatotic and non-alcoholic steatotic livers from brain-dead donor.
Accurate evaluation of liver steatosis is required from brain-dead donors (BDDs) with nonalcoholic fatty liver disease (NAFLD). Our purposes were to investigate expression and regulation of connective tissue growth factor (CTGF) expression in livers from human and rat after brain death, and further evaluate its potential application. NAFLD and brain death models were established in rats. LX2 cells were cultured under hypoxia/reoxygenation. CTGF protein and mRNA levels were measured in liver samples from BDDs of human and rat by immunohistochemistry and reverse transcription-quantitative polymerase chain reaction. YAP-regulated CTGF expression was investigated in LX2 cells via YAP small interfering RNA and... (truncated)

Title: PNPLA3 I148M Up-Regulates Hedgehog and Yap Signaling in Human Hepatic Stellate Cells.
Liver fibrosis represents the wound healing response to sustained hepatic injury with activation of hepatic stellate cells (HSCs). The I148M variant of the <i>PNPLA3</i> gene represents a risk factor for development of severe liver fibrosis. Activated HSCs carrying the I148M variant display exacerbated pro-inflammatory and pro-fibrogenic features. We aimed to examine whether the I148M variant may impair Hedgehog and Yap signaling, as key pathways implicated in the control of energy expenditure and maintenance of myofibroblastic traits. First, we show that TGF-β rapidly up-regulated the PNPLA3 transcript and protein and Yap/Hedgehog target gene expression. In addition, HSCs overexpressing

MESH:D000077300 - bosentan

Summary:

LLM Prediction Score	0.500 (normalized)
LLM Confidence Score	0.970
Golden Answer (Severity Class)	0.875 (normalized)
Prediction Error	0.375

Retrieved Context:

Title: Combined use of ursodeoxycholic acid and bosentan prevents liver toxicity caused by endothelin receptor antagonist bosentan monotherapy: two case reports.
Pulmonary arterial hypertension is a fatal disease characterized by progressive remodeling of the pulmonary arteries and an increase in pulmonary vascular resistance. Up to 50% of patients with systemic sclerosis have pulmonary arterial hypertension, which significantly affects the prognosis. The endothelin receptor antagonist bosentan is used for the treatment of pulmonary arterial hypertension and shows a great beneficial effect. However, the most frequent side effect of bosentan is liver toxicity, which often requires dose reduction and discontinuation.

Title: Hepatotoxicity by bosentan in a patient with portopulmonary hypertension: a case-report and review of the literature.
Bosentan is an endothelin receptor antagonist approved for treatment of pulmonary arterial hypertension. Mild liver reactions occur in about 10% of treated patients but severe hepatotoxicity is rare. We present clinical data and treatment outcome of a severe drug induced liver injury due to bosentan in a patient with non-cirrhotic portopulmonary hypertension. After 18 months of uncomplicated therapy with bosentan 125 mg b.i.d., the patient developed a severe mixed hepatic injury. Serum levels of bilirubin were 316 µmol/l (ref. value <20 micromol/l), AST 14 µkat/l (ref. value < 0.9 µkat/l), ALT 10 µkat/l (ref. value < 0.9 µkat/l), ALP 8... (truncated)

Title: Multiple compound-related adverse properties contribute to liver injury caused by endothelin receptor antagonists.
Drug-induced liver injury has been observed in patients treated with the endothelin receptor antagonists sitaxentan and bosentan, but not following treatment with ambrisentan. The aim of our studies was to assess the possible role of multiple contributory mechanisms in this clinically relevant toxicity. Inhibition of the bile salt export pump (BSEP) and multidrug resistance-associated protein 2 was quantified using membrane vesicle assays. Inhibition of mitochondrial respiration in human liver-derived HuH-7 cells was determined using a Seahorse XF(e96) analyzer. Cytochrome P450 (P450)-independent and P450-mediated cell toxicity was assessed using transfected SV40-T-antigen-immortalized human liver epithelial (THLE) cell lines. Exposure-adjusted assay ratios were... (truncated)

Title: Omics-based responses induced by bosentan in human hepatoma HepaRG cell cultures.
Bosentan is well known to induce cholestatic liver toxicity in humans. The present study was set up to characterize the hepatotoxic effects of this drug at the transcriptomic, proteomic, and metabolomic levels. For this purpose, human hepatoma-derived HepaRG cells were exposed to a number of concentrations of bosentan during different periods of time. Bosentan was found to functionally and transcriptionally suppress the bile salt export pump as well as to alter bile acid levels. Pathway analysis of both transcriptomics and proteomics data identified cholestasis as a major toxicological event. Transcriptomics results further showed several gene changes related to the activation... (truncated)

Title: CYP2C9, SLCO1B1, SLCO1B3, and ABCB11 polymorphisms in patients with bosentan-induced liver toxicity.
Bosentan is an endothelin receptor antagonist used as a first-line treatment in pulmonary arterial hypertension (PAH). Its main adverse effect is a dose-dependent liver toxicity. CYP2C9*2 has recently been shown to be associated with hepatotoxicity in PAH patients. We conducted a nested case-control study to further explore the relationship between functional polymorphisms of gene products involved in bosentan pharmacokinetics (OATP1B1, OATP1B3, and CYP2C9) or hepatobiliary transporters affected by bosentan (ABCB11) and bosentan-induced liver toxicity.

MESH:D000077443 - acamprosate

Summary:

LLM Prediction Score	0.000 (normalized)
LLM Confidence Score	0.990
Golden Answer (Severity Class)	0.375 (normalized)
Prediction Error	0.375

Retrieved Context:

Title: Treatment of alcohol use disorder in patients with liver disease.
Alcohol contributes to more than 5% of global mortality, and causes more than half of all liver-related deaths. The Alcohol Use Disorders Identification Test (AUDIT) can be used to detect those patients with hazardous drinking and alcohol dependence who will benefit from psychosocial and pharmacological alcohol treatment. Psychosocial treatments range from brief interventions and cognitive behavioral therapy, to experimental neuropsychological treatments. Psychosocial intervention can be combined with acamprosate or naltrexone as first line pharmacological treatments. For patients with liver disease, abstinence increases survival and is therefore an important treatment goal. Acamprosate is a good choice, as it prevents relapse to... (truncated)

Title: [Treatment of alcoholic liver diseases and psychiatric and psychosocial problems].
Only about 15% of the subjects abusing ethanol will eventually develop cirrhosis of the liver, suggesting that other factors in addition to the consumption of large quantities of ethanol play a role in the pathogenesis of alcoholic cirrhosis. Important contributors may be infection with hepatitis viruses, in particular HCV, protein-calorie malnutrition and immunologic factors. Abstinence improves the prognosis of patients with alcoholic cirrhosis, provided that the liver disease is not too far advanced. No pharmacotherapeutic intervention has shown a convincing improvement of the prognosis of alcoholic liver disease, so that the therapeutic efforts should be mainly directed towards abstinence. The... (truncated)

Title: Management of alcohol dependence in patients with liver disease.
Alcohol dependence represents a chronic and relapsing disease affecting nearly 10 % of the general population both in the USA and in Europe, with a widespread burden of morbidity and mortality. Alcohol dependence represents the most common cause of liver damage in the Western world. Although alcoholic liver disease is associated primarily with heavy drinking, continued alcohol consumption, even in low doses after the onset of liver disease, increases the risk of severe consequences, including mortality. Consequently, the ideal treatment of patients affected by alcohol dependence and alcoholic liver disease should aim at achieving long-term total alcohol abstinence and preventing... (truncated)

Title: [Acamprosate and psychosocial intervention. An integrative treatment approach for prevention of alcohol dependent patients in Switzerland].
105 patients with severe alcohol dependence, who were treated in 13 centers in Switzerland, took part in this open study. The abstinence rate achieved under treatment with Acamprosat, which was used within the framework of established psychotherapeutic intervention programmes in which the doctors could choose between five different procedures, was determined over a period of 24 weeks. In addition, a sociodemographic profile was drawn up, a physical examination was carried out and data were collected on the safety aspect of Acamprosat. It was also of interest to ascertain whether, and if so how, the patients' quality of life changed under... (truncated)

Title: Acamprosate for treatment of alcohol dependence: mechanisms, efficacy, and clinical utility.
Acamprosate, or N-acetyl homotaurine, is an N-methyl-D-aspartate receptor modulator approved by the Food and Drug Administration (FDA) as a pharmacological treatment for alcohol dependence. The exact mechanism of action of acamprosate is still under investigation, but the drug appears to work by promoting a balance between the excitatory and inhibitory neurotransmitters, glutamate and gamma-aminobutyric acid, respectively, and it may help individuals with alcohol dependence by reducing withdrawal-associated distress. Acamprosate has low bioavailability, but also has an excellent tolerability and safety profile. In comparison with naltrexone and disulfiram, which are the other FDA-approved treatments for alcohol dependence, acamprosate is unique in... (truncated)

MESH:C059240 - cevimeline

Summary:

LLM Prediction Score	0.000 (normalized)
LLM Confidence Score	0.970
Golden Answer (Severity Class)	0.375 (normalized)
Prediction Error	0.375

Retrieved Context:

Title: Primary Biliary Cirrhosis and Primary Sclerosing Cholangitis: a Review Featuring a Women's Health Perspective.
Primary biliary cirrhosis (PBC) and primary sclerosing cholangitis (PSC) are two major types of chronic cholestatic liver disease. Each disorder has distinguishing features and variable progression, but both may ultimately result in cirrhosis and hepatic failure. The following offers a review of PBC and PSC, beginning with a general overview of disease etiology, pathogenesis, diagnosis, clinical features, natural course, and treatment. In addition to commonly associated manifestations of fatigue, pruritus, and fat-soluble vitamin deficiency, select disease-related topics pertaining to women's health are discussed including metabolic bone disease, hyperlipidemia and cardiovascular risk, and pregnancy-related issues influencing maternal disease course and birth... (truncated)

Title: Primary biliary cholangitis: Epidemiology, prognosis, and treatment.
Primary biliary cholangitis (PBC) is a chronic cholestatic autoimmune liver disease characterized by a destructive, small duct, and lymphocytic cholangitis, and marked by the presence of antimitochondrial antibodies. The incidence and prevalence of PBC vary widely in different regions and time periods, and although disproportionally more common among White non-Hispanic females, contemporary data show a higher prevalence in males and racial minorities than previously described. Outcomes largely depend on early recognition of the disease and prompt institution of treatment, which, in turn, are directly influenced by provider bias and socioeconomic factors. Ursodeoxycholic acid remains the initial treatment of choice for... (truncated)

Title: Preventative care in cholestatic liver disease: Pearls for the specialist and subspecialist.
Cholestatic liver diseases (CLDs) encompass a variety of disorders of abnormal bile formation and/or flow. CLDs often lead to progressive hepatic insult and injury and following the development of cirrhosis and associated complications. Many such complications are clinically silent until they manifest with severe sequelae, including but not limited to life-altering symptoms, metabolic disturbances, cirrhosis, and hepatobiliary diseases as well as other malignancies. Primary sclerosing cholangitis (PSC) and primary biliary cholangitis (PBC) are the most common CLDs, and both relate to mutual as well as unique complications. This review provides an overview of PSC and PBC, with a focus on... (truncated)

Title: Current understanding of primary biliary cholangitis.
Primary biliary cholangitis (PBC) causes chronic and persistent cholestasis in the liver, eventually resulting in cirrhosis and hepatic failure without appropriate treatment. PBC mainly develops in middle-aged women, but it is also common in young women and men. PBC is considered a model of autoimmune disease because of the presence of diseasespecific autoantibodies, that is, antimitochondrial antibodies (AMAs), intense infiltration of mononuclear cells into the bile ducts, and a high prevalence of autoimmune diseases such as comorbidities. Histologically, PBC is characterized by degeneration and necrosis of intrahepatic biliary epithelial cells surrounded by a dense infiltration of mononuclear cells, coined as... (truncated)

Title: Orofacial manifestations of adverse drug reactions: a review study.
Adverse reaction to medication is common and may have a variety of clinical manifestations in the oral cavity. The present review paper aimed to describe adverse drug reactions (ADRs) which might be encountered by dental practitioners in every discipline.

MESH:D002230 - carbidopa

Summary:

LLM Prediction Score	0.000 (normalized)
LLM Confidence Score	0.990
Golden Answer (Severity Class)	0.375 (normalized)
Prediction Error	0.375

Retrieved Context:

Title: Levodopa + carbidopa + entacapone. Entacapone: a second look: new preparations. Parkinson's disease: a modest effect.
(1) If patients with Parkinson's disease treated with levodopa develop end-of-dose motor fluctuations, the standard therapy is to add bromocriptine, a dopamine receptor agonist, to their ongoing treatment. (2) Evaluation data available in 1999 on entacapone, a catechol-o-methyltransferase (COMT) inhibitor, failed to show whether the balance of benefits versus harm was at least equivalent to that of bromocriptine. (3) Entacapone is now also available as a triple fixed-dose combination with levodopa + carbidopa. (4) Three double-blind trials have compared triple combinations of levodopa + carbidopa (or benserazide) + entacapone with levodopa + carbidopa (or benserazide) + placebo. Two of these... (truncated)

Title: Clinical pharmacology, therapeutic use and potential of COMT inhibitors in Parkinson's disease.
When peripheral decarboxylation is blocked by carbidopa or benserazide, the main metabolic pathway of levodopa is O-methylation by catechol-O-methyltransferase (COMT). Entacapone and tolcapone are new potent, selective and reversible nitrocatechol-type COMT inhibitors. Animal studies have demonstrated that entacapone mainly has a peripheral effect whereas tolcapone also inhibits O-methylation in the brain. In human volunteers, both entacapone and tolcapone dose-dependently inhibit the COMT activity in erythrocytes, improve the bioavailability and decrease the elimination of levodopa, and inhibit the formation of 3-O-methyldopa (3-OMD). Entacapone is administered with every scheduled dose of levodopa whereas tolcapone is administered 3 times daily. The different administration... (truncated)

Title: The Design and Evaluation of an l-Dopa-Lazabemide Prodrug for the Treatment of Parkinson's Disease.
l-Dopa, the metabolic precursor of dopamine, is the treatment of choice for the symptomatic relief of the advanced stages of Parkinson's disease. The oral bioavailability of l-dopa, however, is only about 10% to 30%, and less than 1% of the oral dose is estimated to reach the brain unchanged. l-Dopa's physicochemical properties are responsible for its poor bioavailability, short half-life and the wide range of inter- and intrapatient variations of plasma levels. An l-dopa-lazabemide prodrug is proposed to overcome the problems associated with l-dopa absorption. Lazabemide is a monoamine oxidase (MAO)-B inhibitor, a class of compounds that slows the depletion... (truncated)

Title: Clinical advantages of COMT inhibition with entacapone - a review.
Two catechol- O-methyltransferase (COMT) inhibitors, entacapone and tolcapone, were developed during the 1990's to be used as adjuncts to levodopa (LD) - dopa decarboxylase (DDC) inhibitors in the treatment of Parkinson's disease (PD). Entacapone is currently in wide clinical use, while tolcapone can be used in restricted indications only, due to its hepatotoxicity. COMT inhibitors prolong the elimination of LD, while DDC inhibitors mainly increase its absorption; both mechanisms leading to increased bioavailability of LD. The pharmacokinetic properties of LD, carbidopa and entacapone are quite similar, and entacapone is administered concomitantly with LD plus carbidopa. Entacapone prolongs the clinical effect... (truncated)

Title: Prediction of metabolism-induced hepatotoxicity on three-dimensional hepatic cell culture and enzyme microarrays.
Human liver contains various oxidative and conjugative enzymes that can convert nontoxic parent compounds to toxic metabolites or, conversely, toxic parent compounds to nontoxic metabolites. Unlike primary hepatocytes, which contain myriad drug-metabolizing enzymes (DMEs), but are difficult to culture and maintain physiological levels of DMEs, immortalized hepatic cell lines used in predictive toxicity assays are easy to culture, but lack the ability to metabolize compounds. To address this limitation and predict metabolism-induced hepatotoxicity in high-throughput, we developed an advanced miniaturized three-dimensional (3D) cell culture array (DataChip 2.0) and an advanced metabolizing enzyme microarray (MetaChip 2.0). The DataChip is a functionalized... (truncated)

MESH:D000077608 - aprepitant

Summary:

LLM Prediction Score	0.000 (normalized)
LLM Confidence Score	0.980
Golden Answer (Severity Class)	0.375 (normalized)
Prediction Error	0.375

Retrieved Context:

Title: A novel effect of Aprepitant: Protection for cisplatin-induced nephrotoxicity and hepatotoxicity.
Cisplatin is widely used chemotherapeutic drug and have some serious side effects as tissue toxicity and nausea and vomiting. Aprepitant is used in clinic as an anti-emetic drug for cisplatin treated patient to prevent nausea and vomiting. We aimed to investigate the protective effects of Aprepitant on cisplatin-induced nephrotoxicity and hepatotoxicity. In total 42 male rats were separated into six groups (n = 7). A single dose of cisplatin (10 mg/kg i.p.) was administered to induce toxicity on first day. Different doses of Aprepitant (5, 10 and 20 mg/kg, p.o.) were given to treatment groups during 3 days. After the... (truncated)

Title: NK1 receptor antagonists versus other antiemetics in the prevention of postoperative nausea and vomiting following laparoscopic surgical procedures: a systematic review and meta-analysis.
A systematic electronic search of MEDLINE, EMBASE, and CINAHL databases aimed at comparing neurokinin-1 receptor antagonists with other antiemetics in their prevention of postoperative nausea and vomiting in adult patients undergoing laparoscopic surgery identified seven randomized controlled trials for review and meta-analysis. Preoperative aprepitant 80 mg was found to reduce nausea (RR: 0.56, 95% CI: 0.41-0.75, I² = 0%, <i>P</i> = 0.89) and vomiting (RR: 0.20, 95% CI: 0.05-0.77, I² = 0%, <i>P</i> = 0.96) and resulted in complete response (RR: 1.61 (1.25-2.08), I² = 0%, <i>P</i> = 0.70) within the first 2 hours following surgery as well as vomiting... (truncated)

Title: Aprepitant Inhibits JNK and p38/MAPK to Attenuate Inflammation and Suppresses Inflammatory Pain.
Substance P contributes to the pathogenesis of pain by acting on NK-1R, specialized sensory neurons that detect noxious stimuli. Aprepitant, an antagonist of NK-1R, is widely used to treat chemotherapy-induced nausea and vomiting. In this study, we used LPS-stimulated BV-2 microglia cell line and animal models of inflammatory pain to explore the analgesic effect of aprepitant on inflammatory pain and its underlying mechanism. The excitability of DRG neurons were measured using whole-cell patch-clamp recordings. The behavioral tests were measured and the morphological changes on inflamed paw sections were determined by HE staining. Changes in the expressions of cytokine were measured... (truncated)

Title: The effects of intravenous fosaprepitant and ondansetron for the prevention of postoperative nausea and vomiting in neurosurgery patients: a prospective, randomized, double-blinded study.
The incidence of postoperative nausea and vomiting (PONV) is 30-50% after surgery. PONV occurs frequently, especially after craniotomy. In this study, we investigated the preventive effects on PONV in a randomized study by comparing patients who had been administered fosaprepitant, a neurokinin-1 (NK1) receptor antagonist, or ondansetron intravenously. Sixty-four patients undergoing craniotomy were randomly allocated to receive fosaprepitant 150 mg i.v. (NK1 group, n = 32) or ondansetron 4 mg i.v. (ONS group, n = 32) before anesthesia. The incidence of vomiting was significantly less in the NK1 group, where 2 of 32 (6%) patients experienced vomiting compared to 16... (truncated)

Title: A Comparison of Fosaprepitant and Ondansetron for Preventing Postoperative Nausea and Vomiting in Moderate to High Risk Patients: A Retrospective Database Analysis.
Postoperative nausea and vomiting (PONV) occur in 30-50% of patients undergoing general anesthesia and in 70-80% of high PONV risk patients. In this study, we investigated the efficacy of fosaprepitant, a neurokinin-1 (NK1) receptor antagonist, compared to ondansetron, a selective 5-hydroxytryptamine type 3 (5-HT3) receptor antagonist, in moderate to high PONV risk patients from our previous randomized controlled trials. Patients (171 patients from 4 pooled studies) with the Apfel simplified score ≥ 2 and undergoing general anesthesia were randomly allocated to receive intravenous fosaprepitant 150 mg (NK1 group, <i>n</i> = 82) and intravenous ondansetron 4 mg (ONS group, <i>n</i> = 89) before... (truncated)

MESH:C084656 - paricalcitol

Summary:

LLM Prediction Score	0.000 (normalized)
LLM Confidence Score	0.980
Golden Answer (Severity Class)	0.375 (normalized)
Prediction Error	0.375

Retrieved Context:

Title: Paricalcitol inhibits oxidative stress-induced cell senescence of the bile duct epithelium dependent on modulating Sirt1 pathway in cholestatic mice.
Clinical studies indicate that vitamin D receptor (VDR) expression is reduced in primary biliary cirrhosis patient livers. However, the mechanism by which activated VDR effect cholestatic liver injury remains unclear.

Title: The vitamin D analogue paricalcitol attenuates hepatic ischemia/reperfusion injury through down-regulation of Toll-like receptor 4 signaling in rats.
Recent studies have revealed that vitamin D and its synthetic analogues have a protective effect on experimental ischemia/reperfusion (I/R) models in several organs, but little is known about its effect on the liver. The aim of this study was to evaluate the beneficial effects of vitamin D in a model of liver I/R in rats, focusing on Toll-like receptor (TLR) 4 signaling, which has been shown to be involved in I/R injury.

Title: Paricalcitol Ameliorates Acute Kidney Injury in Mice by Suppressing Oxidative Stress and Inflammation via Nrf2/HO-1 Signaling.
Effective and targeted prevention and treatment methods for acute kidney injury (AKI), a common clinical complication, still needs to be explored. Paricalcitol is a biologically active chemical that binds to vitamin D receptors in the body to exert anti-oxidant and anti-inflammatory effects. However, the molecular mechanism of the effect of paricalcitol on AKI remains unclear. The current study uses a paricalcitol pretreatment with a mouse AKI model induced by cisplatin to detect changes in renal function, pathology and ultrastructure. Results showed that paricalcitol significantly improved renal function in mice and reduced inflammatory cell infiltration and mitochondrial damage in renal tissue.... (truncated)

Title: Calcitriol Protects against Acetaminophen-Induced Hepatotoxicity in Mice.
Acetaminophen (APAP) overdose is one of the major causes of acute liver failure. Severe liver inflammation and the production of oxidative stress occur due to toxic APAP metabolites and glutathione depletion. Growing evidence has proved that vitamin D (VD) exerts anti-inflammatory and antioxidative functions. Our objective was to explore the protective role of calcitriol (VD3) in acute APAP-induced liver injury. Methods: Adult male mice were randomized into three groups; control (n = 8), APAP (n = 8), and VD3 group (n = 8). All mice, except controls, received oral administration of APAP (400 mg/kg) and were sacrificed 24 h later.... (truncated)

Title: The role of vitamin d in primary biliary cirrhosis: possible genetic and cell signaling mechanisms.
Primary biliary cirrhosis (PBC) is an immune-mediated chronic inflammatory disease of the liver of unknown etiology. Vitamin D deficiency is highly prevalent in patients with PBC, and many studies have demonstrated the significant effect of calcitriol on liver cell physiology. Vitamin D has antiproliferative and antifibrotic effects on liver fibrosis. Genetic studies have provided an opportunity to determine which proteins link vitamin D to PBC pathology (e.g., the major histocompatibility complex class II molecules, the vitamin D receptor, toll-like receptors, apolipoprotein E, Nramp1, and cytotoxic T lymphocyte antigen-4). Vitamin D also exerts its effect on PBC through cell signaling mechanisms,... (truncated)

MESH:D020008 - delavirdine

Summary:

LLM Prediction Score	0.250 (normalized)
LLM Confidence Score	0.950
Golden Answer (Severity Class)	0.625 (normalized)
Prediction Error	0.375

Retrieved Context:

Title: Toxicity of non-nucleoside analogue reverse transcriptase inhibitors.
The non-nucleoside reverse transcriptase inhibitors (NNRTI) nevirapine (NVP), efavirenz (EFV), and delaviridine (DLV) are increasingly being used to treat HIV infection. Studies have shown excellent tolerance and efficacy and less development of virological resistance with HIV regimens that include NNRTIs. Nevertheless, abnormalities in liver enzymes are common in patients with HIV infection, and there are multiple etiologies for these abnormalities, including drug toxicity, viral hepatitis, opportunistic infections, and substance abuse. In particular, highly active antiretroviral therapy (HAART) can result in hepatotoxicity through a variety of mechanisms, such as mitochondrial toxicity, lipodystrophy syndrome, and steatohepatitis. The NNRTIs have been most frequently... (truncated)

Title: Delavirdine: a review of its use in HIV infection.
Delavirdine, a bisheteroarylpiperazine derivative, is a non-nucleoside reverse transcriptase inhibitor (NNRTI) that allosterically binds to HIV-1 reverse transcriptase, inhibiting both the RNA- and DNA-directed DNA polymerase functions of the enzyme. Delavirdine in combination with nucleoside reverse transcriptase inhibitors (NRTIs) produced sustained reductions in plasma viral loads and improvements in immunological responses in large randomised, double-blind, placebo-controlled studies of 48 to 54 weeks' duration. In patients with advanced HIV infection, triple therapy with delavirdine, zidovudine and lamivudine, didanosine or zalcitabine for 1 year significantly prolonged the time to virological failure compared with dual therapy (delavirdine plus zidovudine or 2 NRTIs; p... (truncated)

Title: Lack of hepatotoxicity associated with nonnucleoside reverse transcriptase inhibitors.
Nonnucleoside reverse transcriptase inhibitors (NNRTIs), particularly nevirapine, have been associated with hepatotoxicity. We performed a retrospective study to determine the incidence of NNRTI hepatotoxicity in a group of HIV-infected patients from a New York City practice. These patients are predominantly homosexual white males. We also analyzed the effect of coinfection with hepatitis B (HBV) or hepatitis C (HCV) virus. In total, 272 patients received NNRTIS: 40 (15%) received delavirdine, 91 (33%) received efavirenz, and 141 (52%) received nevirapine. Of the patients with known hepatitis status, 18 of 190 (9%) were coinfected with HBV, and 24 of 205 were coinfected (12%)... (truncated)

Title: Mechanisms of Hepatic Cholestatic Drug Injury.
Drug-induced cholestasis represents a form of drug-induced liver disease that can lead to severe impairment of liver function. Numerous drugs have been shown to cause cholestasis and consequently bile duct toxicity. However, there is still lack of therapeutic tools that can prevent progression to advanced stages of liver injury. This review focuses on the various pathological mechanisms by which drugs express their hepatotoxic effects, as well as consequences of increased bile acid and toxin accumulation in the hepatocytes.

Title: NNRTI and Liver Damage: Evidence of Their Association and the Mechanisms Involved.
Due to the improved effectiveness and safety of combined antiretroviral therapy, human immunodeficiency virus (HIV) infection has become a manageable, chronic condition rather than a mortal disease. However, HIV patients are at increased risk of experiencing non-AIDS-defining illnesses, with liver-related injury standing out as one of the leading causes of death among these patients. In addition to more HIV-specific processes, such as antiretroviral drug-related toxicity and direct injury to the liver by the virus itself, its pathogenesis is related to conditions that are also common in the general population, such as alcoholic and non-alcoholic fatty liver disease, viral hepatitis, and... (truncated)

MESH:D017275 - isradipine

Summary:

LLM Prediction Score	0.000 (normalized)
LLM Confidence Score	0.950
Golden Answer (Severity Class)	0.375 (normalized)
Prediction Error	0.375

Retrieved Context:

Title: Pharmacokinetics of calcium antagonists under development.
Calcium antagonist drugs under clinical development are of the Type I (verapamil, diltiazem-like) and Type II (nifedipine-like) classes. Tiapamil, the only Type I drug currently available, is a high clearance, widely distributed drug which undergoes extensive presystemic elimination. Pharmacokinetically it is quite similar to verapamil; however, it does have increased biliary excretion and decreased binding to plasma proteins. Eight Type II (dihydropyridine) drugs are reviewed. Seven of these drugs (felodipine, isradipine, nicardipine, nilvadipine, nimodipine, nisoldipine and nitrendipine) are pharmacokinetically similar to nifedipine, with high clearance, extensive distribution, and significant presystemic elimination. Amlodipine has lower clearance, even greater peripheral distribution, and... (truncated)

Title: Successful drug development despite adverse preclinical findings part 2: examples.
To illustrate the process of addressing adverse preclinical findings (APFs) as outlined in the first part of this review, a number of cases with unexpected APF in toxicity studies with drug candidates is discussed in this second part. The emphasis is on risk characterization, especially regarding the mode of action (MoA), and risk evaluation regarding relevance for man. While severe APFs such as retinal toxicity may turn out to be of little human relevance, minor findings particularly in early toxicity studies, such as vasculitis, may later pose a real problem. Rodents are imperfect models for endocrine APFs, non-rodents for human... (truncated)

Title: Isradipine enhancement of virtual reality cue exposure for smoking cessation: Rationale and study protocol for a double-blind randomized controlled trial.
Cigarette smoking remains a leading cause of preventable death in the United States, contributing to over 480,000 deaths each year. Although significant strides have been made in the development of effective smoking cessation treatments, most established interventions are associated with high relapse rates. One avenue for increasing the effectiveness of smoking cessation interventions is to design focused, efficient, and rigorous experiments testing engagement of well-defined mechanistic targets. Toward this aim, the current protocol will apply a pharmacologic augmentation strategy informed by basic research in animal models of addiction. Our goal is to evaluate the enhancing effect of isradipine, an FDA-approved... (truncated)

Title: Novel Score-based Decision Approach in Chronic Myeloid Leukemia Patients After Acute Toxic Imatinib-induced Liver Injury.
The tyrosine kinase inhibitor (TKI) imatinib in rare cases can cause acute toxic hepatitis, hepatic failure, and death. Currently, the choice of further chronic myeloid leukemia (CML) therapy in patients after acute hepatotoxicity is still a difficult question, which requires a complex individual approach based on the clinical guidelines of adverse event management. Data about the further follow-up strategy approach in patients with CML after acute toxic imatinib-induced liver injury are of concern, and at times controversial. In addition, one of the questions is about the necessity and safety of the imatinib therapy resumption after acute hepatotoxicity. In some publications, imatinib... (truncated)

Title: Fulminant liver failure associated with clarithromycin.
To report a patient developing fulminant liver failure while being treated with clarithromycin for pneumonia.

MESH:D000068577 - nebivolol

Summary:

LLM Prediction Score	0.000 (normalized)
LLM Confidence Score	0.980
Golden Answer (Severity Class)	0.375 (normalized)
Prediction Error	0.375

Retrieved Context:

Title: Protective role of nebivolol in cadmium-induced hepatotoxicity via downregulation of oxidative stress, apoptosis and inflammatory pathways.
Cadmium (Cd) intoxication in human occurs through inhalation of cigarette smoke and ingestion of contaminated water and food. We investigated the role of nebivolol (NEB) in Cd induced hepatotoxicity. In our study; NEB was given as (10 mg/kg/d) orally to rats for 6 weeks, in the presence or absence of hepatotoxicity induced by oral administration of Cd (7 mg/kg/d) for 6 weeks. Levels of serum liver enzyme biomarkers; alanine transaminase (ALT), aspartate transaminase (AST) and serum total antioxidant capacity (TAC) were measured. In addition; mean arterial pressure and total cholesterol levels were measured. Hepatic superoxide dismutase (SOD) and malondialdehyde (MDA)... (truncated)

Title: Hypoglycemia, polycythemia and hyponatremia in a newborn exposed to nebivolol during pregnancy.
Nebivolol is a third-generation beta blocker that exerts selective antagonistic activity on β1 receptors. It has vasodilating properties that result from direct stimulation of endothelial nitric oxide synthase. Nebivolol is indicated for the treatment of hypertension and heart failure, and is generally well tolerated. In this article, we report a case of an infant who was admitted to the Pediatrics and Neonatology Unit of the Moscati Hospital (Aversa, Italy) about 24 hours after birth. The reason for hospitalization was persistent severe hypoglycemia (blood glucose = 30 mg/dL) and jaundice (total bilirubin = 12.5 mg/dL, indirect bilirubin 11.75 mg/dL). He was... (truncated)

Title: Acute hepatitis after starting pinaverium bromide in a patient taking mirtazapine.
A 56-year-old man presented with chronic abdominal pain. He had been evaluated extensively in the recent past undergoing upper gastrointestinal endoscopy, colonoscopy and CT scan of the abdomen with normal results. The provisional diagnosis of irritable bowel syndrome was performed and pinaverium bromide was started. The patient had pre-existing hypertension, a major depressive disorder and gastro-oesophageal reflux disease. He had been taking nebivolol and pantoprazole for several years and mirtazapine for the last 1 year. The patient developed nausea, vomiting and anorexia after 5 days of starting pinaverium bromide. Investigations revealed marked elevation of liver enzymes and bilirubin. He was... (truncated)

Title: The effect of nebivolol versus metoprolol succinate extended release on asymmetric dimethylarginine in hypertension.
This study sought to determine if metoprolol succinate ER (MET), and nebivolol (NEB), a β1-AR with increased bioavailability of nitric oxide (NO), would have differing effects on plasma asymmetric dimethylarginine concentration in hypertensives. It was hypothesized that NEB, a β1-AR antagonist and β3-AR agonist with NO-releasing properties, and MET, only a β1-AR antagonist, would have different effects on plasma asymmetric dimethylarginine (ADMA) concentration. Forty-one hypertensive subjects randomly received either 50 mg of MET (n = 19) or 5 mg of NEB (n = 22) for 4 weeks followed by 100 mg MET and 10 mg NEB for 4 weeks. ADMA... (truncated)

Title: Rhabdomyolysis in a patient taking nebivolol.
β Blockers such as propranolol and labetalol are known to induce toxic myopathy because of their partial β2 adrenoceptor agonistic effect. Nebivolol has the highest β1 receptor affinity among β blockers, and it has never been reported to induce rhabdomyolysis until now. We report a patient who developed rhabdomyolysis after changing medication to nebivolol. A 75-year-old woman was admitted to our hospital because of generalized weakness originating 2 weeks before visiting. Approximately 1 month before her admission, her medication was changed from carvedilol 12.5 mg to nebivolol 5 mg. Over this time span, she had no other lifestyle changes causing... (truncated)

MESH:D017273 - goserelin

Summary:

LLM Prediction Score	0.000 (normalized)
LLM Confidence Score	0.990
Golden Answer (Severity Class)	0.375 (normalized)
Prediction Error	0.375

Retrieved Context:

Title: [Severe toxic hepatitis during flutamide (Eulexin) treatment].
A case of severe toxic hepatitis in a patient with metastatic prostatic cancer treated for three months with flutamide (Eulexin) combined with an LHRH analogue, goserelin (Zoladex) is described. The patient developed severe liver failure with jaundice, ascites and severe encephalopathy. The condition reversed after discontinuation of flutamide. Less severe, but otherwise similar, adverse reactions have been reported in the literature, and the importance of considering the drug as a potential hepatotoxin is stressed.

Title: Frequency of flutamide induced hepatotoxicity in patients with prostate carcinoma.
To identify and describe the frequency and severity of hepatotoxicity in patients who received flutamide therapy for prostate cancer, 22 patients were treated with the combination of flutamide and goserilin or orchiectomy. After diagnosis and staging of prostate cancer, baseline results were obtained for a set of five liver function tests (LF Ts). Hepatotoxicity was assessed according to the WHO criteria. After initiation of flutamide therapy, LF Ts were performed at 4, 8 and 12 weeks and every 2 months thereafter. Severe hepatotoxicity appeared in two of 22 (9%) patients. Following the discontinuation of flutamide, one patient died due to... (truncated)

Title: [A case of fulminant hepatitis caused by antiandrogen, flutamide in a patient with prostate cancer].
Luteinizing hormone-releasing hormone analogue, 3.6 mg goserelin acetate and flutamide, 375 mg per day were used to treat metastatic prostate cancer in a 66-year-old male. Marked increase in serum aminotransferases and total bilirubin levels was noted after 7 weeks of treatment. The enzyme levels returned to normal at 2 months following discontinuation of the treatment. Though incidences of severe hepatotoxicity are rare (0.003-0.18%), cases of flutamide-induced death have been reported. Careful monitoring of patients is mandatory while administering flutamide.

Title: Phase II trial of tamoxifen and goserelin in recurrent epithelial ovarian cancer.
Endocrine therapy is a recognised option in the treatment of chemo-resistant ovarian cancer. We conducted a nonrandomised phase II evaluation of combination endocrine therapy with tamoxifen and goserelin in patients with advanced ovarian cancer that had recurred following chemotherapy. In total, 26 patients entered the study, of which 17 had platinum-resistant disease. The median age was 63 years and enrolled patients had received a median of three chemotherapy regimens prior to trial entry. Patients were given oral tamoxifen 20 mg twice daily on a continuous basis and subcutaneous goserelin 3.6 mg once a month until disease progression. Using the definition... (truncated)

Title: A multicenter randomized trial comparing the luteinizing hormone-releasing hormone analogue goserelin acetate alone and with flutamide in the treatment of advanced prostate cancer. The International Prostate Cancer Study Group.
A prospective randomized trial was conducted to compare the effects of the nonsteroidal antiandrogen flutamide (250 mg. 3 times daily) plus the luteinizing hormone-releasing hormone analogue goserelin acetate (Zoladex) (3.6 mg. subcutaneous depot injection every 28 days) with goserelin acetate alone in advanced prostatic carcinoma. A total of 571 eligible patients, of whom 57% had distant metastases, showed no difference in subjective or objective response rates, interval to progression, treatment failure or survival after a median followup of 2 years. In the combination group more patients had an early decrease in elevated levels of tumor markers and the small number... (truncated)

MESH:D020105 - milrinone

Summary:

LLM Prediction Score	0.000 (normalized)
LLM Confidence Score	0.970
Golden Answer (Severity Class)	0.375 (normalized)
Prediction Error	0.375

Retrieved Context:

Title: Drug-induced kidney injury in Chinese critically ill pediatric patients.
Background: Drug-induced acute kidney injury (DIKI) is a common adverse drug reaction event but is less known in pediatric patients. The study explored the DIKI in Chinese pediatric patients using the Pediatric Intensive Care database (PIC). Method: We screened pediatric patients with acute kidney injury (AKI) using the KDIGO criteria from the PIC and then assessed the relationship between their drugs and DIKI using the Naranjo scale. For the fifteen frequently used DIKI-suspected drugs, we divided patients into drug-exposed and non-exposed groups, using the outcome of whether DIKI was presented or not. Propensity score matching (PSM) was used to control... (truncated)

Title: Inhaled pulmonary vasodilators: a narrative review.
Pulmonary hypertension (PH) is a severe disease that affects people of all ages. It can occur as an idiopathic disorder at birth or as part of a variety of cardiovascular and pulmonary disorders. Inhaled pulmonary vasodilators (IPV) can reduce pulmonary vascular resistance (PVR) and improve RV function with minimal systemic effects. IPV includes inhaled nitric oxide (iNO), inhaled aerosolized prostacyclin, or analogs, including epoprostenol, iloprost, treprostinil, and other vasodilators. In addition to pulmonary vasodilating effects, IPV can also be used to improve oxygenation, reduce inflammation, and protect cell. Off-label use of IPV is common in daily clinical practice. However, evidence... (truncated)

Title: The circadian rhythm gene Bmal1 ameliorates acute deoxynivalenol-induced liver damage.
Deoxynivalenol (DON) is widely emerging in various grain crops, milk, and wine products, which can trigger different toxic effects on humans and animals by inhalation or ingestion. It also imposes a considerable financial loss on the agriculture and food industry each year. Previous studies have reported acute and chronic toxicity of DON in liver, and liver is not only the main detoxification organ for DON but also the circadian clock oscillator directly or indirectly regulates critical physiologically hepatic functions under different physiological and pathological conditions. However, researches on the association of circadian rhythm in DON-induced liver damage are limited. In... (truncated)

Title: Acute liver failure secondary to yellow phosphorus rodenticide poisoning: Outcomes at a center with dedicated liver intensive care and transplant unit.
Accidental or suicidal poisoning with yellow phosphorus or metal phosphides (YPMP) such as aluminum (AlP) zinc phosphide (Zn₃P₂) commonly cause acute liver failure (ALF) and cardiotoxicity. These are used as household, agricultural and industrial rodenticides and in production of ammunitions, firecrackers and fertilizers. In absence of a clinically available laboratory test for diagnosis or toxin measurement or an antidote, managing their poisoning is challenging even at a tertiary care center with a dedicated liver intensive care unit (LICU) and liver transplant facility.

Title: Parenteral Nutrition, Sepsis, Acute Heart Failure and Hepatotoxic Drugs Are Related to Liver Test Disturbances in Critically Ill Patients.
Parenteral nutrition (PN) is often associated with liver dysfunction in the ICU, although other factors such as sepsis, acute heart failure (AHF), and hepatotoxic drugs can be equally present. The relative impact of PN on liver dysfunction in critically ill patients is largely unknown.

MESH:D004091 - hydromorphone

Summary:

LLM Prediction Score	0.000 (normalized)
LLM Confidence Score	0.990
Golden Answer (Severity Class)	0.375 (normalized)
Prediction Error	0.375

Retrieved Context:

Title: Safe Use of Opioids in Chronic Kidney Disease and Hemodialysis Patients: Tips and Tricks for Non-Pain Specialists. In patients suffering from moderate-to-severe chronic kidney disease (CKD) or end-stage renal disease (ESRD), subjected to hemodialysis (HD), pain is very common, but often underestimated. Opioids are still the mainstay of severe chronic pain management; however, their prescription in CKD and HD patients is still significantly low and pain is often under-treated. Altered pharmacokinetics and the lack of clinical trials on the use of opioids in patients with renal impairment increase physicians' concerns in this specific population. This narrative review focused on the correct and safe use of opioids in patients with CKD and HD. Morphine and codeine are not... (truncated)

Title: The efficacy and safety of epidural morphine/hydromorphone in the treatment of intractable postherpetic neuralgia: A single-center, double-blinded, randomized controlled, prospective, and non-inferiority study.
Objective: Postherpetic neuralgia (PHN) is a clinical puzzle, especially in patients who still suffered from moderate and severe pain after standard treatment. This single-center, double-blinded, randomized controlled, prospective, and non-inferiority study observed the safety and effectiveness of the epidural application of morphine or hydromorphone, trying to provide an alternative method for those patients with refractory PHN. Methods: Eighty PHN patients with a visual analogue scale (VAS) still greater than 50 mm after routine management were randomly divided into two groups according to 1:1, respectively. One group received epidural morphine (EMO group), and the other group received epidural hydromorphone (EHM group). VAS,... (truncated)

Title: Long-Term Intravenous Ketamine for Analgesia in a Child with Severe Chronic Intestinal Graft versus Host Disease. Ketamine is reported to be an effective adjuvant to opioids in the treatment of refractory cancer pain; however, the use of high doses of ketamine for extended periods in pediatric patients has not been described. We present a five-year-old male with grade IV intestinal GVHD whose abdominal pain required both hydromorphone and ketamine for a period of over four months. There was no evidence of hepatotoxicity, hemorrhagic cystitis, or other adverse effects. Possible withdrawal symptoms were mild and were readily mitigated by gradually weaning ketamine.

Title: Preclinical and Clinical Pharmacology of Hydrocodone for Chronic Pain: A Mini Review.
Hydrocodone is one of the most prescribed oral analgesic drugs and it is one of the most abused drugs in general population. It is a mu-opioid agonist predominantly metabolized to the O-demethylated product hydromorphone and to the N-demethylated product norhydrocodone. The purpose of the study is to summarize the preclinical and clinical characteristics of hydrocodone. Pharmacokinetic aspect (terminal half-life, maximum serum concentration, and time to maximum serum concentration) of hydrocodone and the influence of metabolic genetic polymorphism in analgesic response to hydrocodone are also illustrated and commented. Literature on experimental preclinical pharmacology investigating analgesic activity in laboratory animals is furtherly... (truncated)

Title: A randomized, double-blind, non-inferiority study of hydromorphone hydrochloride immediate-release tablets versus oxycodone hydrochloride immediate-release powder for cancer pain: efficacy and safety in Japanese cancer patients. Hydromorphone is a standard opioid analgesic for cancer pain that, prior to this study, was not approved in Japan, where options for opioid switching are limited. We aimed to investigate the efficacy and safety of hydromorphone (DS-7113b) immediate-release tablets in opioid-naïve cancer patients with moderate to severe cancer pain.

MESH:C050739 - interferon alfacon-1

Summary:

LLM Prediction Score	0.500 (normalized)
LLM Confidence Score	0.910
Golden Answer (Severity Class)	0.875 (normalized)
Prediction Error	0.375

Retrieved Context:

Title: First phase hepatitis c viral kinetics in previous nonresponders patients.
A large proportion of patients fails to respond to treatment for hepatitis C. Initiation of interferon therapy is associated with a rapid first phase decline in viremia, reflecting inhibition of viral production or release from infected cells. We characterized first phase viral kinetics in previous nonresponder patients and compared the antiviral efficacy of interferon in nonresponders to that observed in naive patients. Twenty nonresponders with genotype 1 infection were evaluated. Ten received a single 15 mcg dose of interferon alfacon-1 and ten were given a 30 mcg dose. Viral kinetic data from previously untreated historical control patients with genotype 1... (truncated)

Title: Efficacy of consensus interferon in treatment of HbeAg-positive chronic hepatitis B: a multicentre, randomized controlled trial.
Consensus interferon (CIFN) is a newly developed type I interferon.

MESH:C100416 - peginterferon alfa-2a

Summary:

LLM Prediction Score	0.500 (normalized)
LLM Confidence Score	0.970
Golden Answer (Severity Class)	0.875 (normalized)
Prediction Error	0.375

Retrieved Context:

Title: Safety of pegylated interferon-alpha-2a in adjuvant therapy of intermediate and high-risk melanomas.
Pegylated (PEG)-IFN-alpha-2a is a modified form of recombinant human IFN-alpha-2a with sustained absorption and prolonged half-life. Our aim was to evaluate its safety profile in adjuvant treatment of high-risk melanoma patients in a single centre setting and to compare this safety profile with data obtained from the literature for a) low dose IFN-alpha and b) high dose IFN. Eighteen consecutive melanoma patients (AJCC 2002 stages IIa-IIIc) were retrospectively analyzed for toxicities associated with adjuvant PEG-IFN-alpha-2a (180 microg/week s.c.). The most frequently reported adverse events were constitutional side effects (78%), myelosuppression (83%) and hepatotoxicity (78%). The proportion of patients receiving PEG-IFN-alpha-2a... (truncated)

Title: Reversible bilateral ototoxicity in a patient with chronic hepatitis B during peginterferon alpha-2a treatment.
Peginterferon alpha-2a (PEG IFN α-2a) is frequently used in chronic hepatitis B (CHB)treatment. Numerous adverse events can be noted during this therapy such as flu-like disease, rash, weight loss and depression. However, PEG IFN α-2a related ototoxicity seems to be an uncommon entity. Ototoxicity can be detected objectively by audiometry. In this paper, we present a case of CHB who developed reversible bilateral ototoxicity during PEG IFN α-2a treatment. Due to ototoxicity detected objectively by audiogram, treatment was ceased at sixth month and ototoxicity completely recovered one month after stopping the drug.

Title: Pegylated Interferon α-2a Triggers NK-Cell Functionality and Specific T-Cell Responses in Patients with Chronic HBV Infection without HBsAg Seroconversion.
Pegylated interferon α-2a (Peg-IFN-α) represents a therapeutic alternative to the prolonged use of nucleos(t)ide analog (NA) in chronic hepatitis B (CHB) infection. The mechanisms leading to a positive clinical outcome remain unclear. As immune responses are critical for virus control, we investigated the effects of Peg-IFN-α on both innate and adaptive immunity, and related it to the clinical evolution. The phenotypic and functional features of the dendritic cells (DCs), natural killer (NK) cells and HBV-specific CD4/CD8 T cells were analyzed in HBeAg-negative CHB patients treated for 48-weeks with NA alone or together with Peg-IFN-α, before, during and up to 2-years... (truncated)

Title: Entecavir and Peginterferon Alfa-2a in Adults With Hepatitis B e Antigen-Positive Immune-Tolerant Chronic Hepatitis B Virus Infection.
Monotherapy with interferon or nucleoside analog is generally not recommended during the immune-tolerant (IT) phase of chronic hepatitis B virus (HBV) infection. Recognition that high HBV DNA levels are associated with hepatocellular carcinoma has increased interest in treating HBV in the IT phase. Small pediatric studies reported efficacy with combination nucleoside analog and interferon therapy. The aim of this study was to evaluate the safety and efficacy of the combination of entecavir and peginterferon in adults in the IT phase of chronic HBV infection. Hepatitis B e antigen (HBeAg)-positive adults with HBV DNA > 10⁷ IU/mL and alanine aminotransferase (ALT)... (truncated)

Title: [A case with chronic hepatitis C who developed liver cirrhosis due to liver dysfunction caused by pegylated interferon plus ribavirin treatment despite negativity of serum HCV RNA, during therapy].
We report a case of chronic hepatitis C in whom liver cirrhosis was later diagnosed following abnormality of ALT levels during pegylated interferon α2a and ribavirin treatment. A 62-year-old woman with chronic hepatitis C was treated with pegylated interferon α2a plus ribavirin for 72 weeks. Her HCV RNA became negative 16 weeks after the start of treatment and continued to be negative for most of the treatment duration. Her AST/ALT, ALP/γ-GTP levels became elevated soon after the initiation of treatment and thereafter remained unchanged. However, most of these levels normalized after the end of treatment. Post-treatment liver biopsy showed liver... (truncated)

MESH:D001398 - aztreonam

Summary:

LLM Prediction Score	0.250 (normalized)
LLM Confidence Score	0.970
Golden Answer (Severity Class)	0.625 (normalized)
Prediction Error	0.375

Retrieved Context:

Title: Safety profile of aztreonam in clinical trials.
The clinical safety of aztreonam in the treatment of suspected aerobic gram-negative infections was assessed in 346 patients who received single doses and in 2,388 patients who received multiple doses. Of those administered multiple doses, 163 (6.8%) experienced 172 adverse clinical effects. The most common were local reactions at the injection site, rash, diarrhea, and nausea and/or vomiting. Among aztreonam and control groups, three-fold increases in serum aspartate aminotransferase (SGOT) and serum alanine aminotransferase (SGPT) values occurred at comparably low frequencies; the mean values of SGOT and SGPT were slightly higher in patients administered aztreonam than in those given cefamandole.... (truncated)

Title: Aztreonam compared with gentamicin for treatment of serious urinary tract infections.
52 patients with serious urinary tract infections were randomised to receive either aztreonam (35) or gentamicin (17). In the aztreonam group 23 patients had unqualified cures, 6 cures with relapse, and 6 cures with reinfection; the comparable numbers in the gentamicin group were 9, 1, and 4. There were no failures with aztreonam and 3 with gentamicin. The most important determinant of outcome was the presence or absence of urological abnormalities. 11 further patients, with renal failure or gentamicin-resistant isolates, treated with aztreonam were all cured. Toxic effects were limited to symptomless liver-function-test abnormalities with aztreonam , whereas deterioration in... (truncated)

Title: [The use of endobronchial aztreonam in the treatment of bronchiectatic suppuration].
The authors submitted 8 patients with bronchiectasis to endobronchial therapy with Aztreonam 2 gr twice a week for 4 weeks after endobronchial lavage with sodium chloride solution. They observed a definite clinical and radiological improvement and a stabilized condition of the disease was observed. No hepatic or renal toxicity was detected.

Title: A Phase III, randomized, controlled, non-inferiority trial of ceftaroline fosamil 600 mg every 8 h versus vancomycin plus aztreonam in patients with complicated skin and soft tissue infection with systemic inflammatory response or underlying comorbidities.
Increasing the ceftaroline fosamil dose beyond 600 mg every 12 h may provide additional benefit for patients with complicated skin and soft tissue infections (cSSTIs) with severe inflammation and/or reduced pathogen susceptibility. A Phase III multicentre, randomized trial evaluated the safety and efficacy of ceftaroline fosamil 600 mg every 8 h in this setting.

Title: Hepatotoxicity Due to Azole Antimycotic Agents in a HLA B*35:02-Positive Patient.
We will present a 42-year-old woman with acute myeloid leukemia and pulmonary aspergillosis. She was treated with several antifungal agents, including three triazoles. Voriconazole, posaconazole, and isavuconazole all led to hepatocellular liver injury. Voriconazole administration led to a peak alanine aminotransferase (ALT) value of 1,793 U/L (normal range, 9-59 U/L). After posaconazole and isavuconazole treatment, ALT rose over 500 U/L. The typical course of events, exclusion of differential diagnoses, and normalization of the liver function tests (LFTs) after stopping the triazoles were highly suspicious for a drug-induced liver injury (DILI). Interestingly, our patient carries a rare HLA B allele (HLA... (truncated)

MESH:D000068879 - adalimumab

Summary:

LLM Prediction Score	0.500 (normalized)
LLM Confidence Score	0.970
Golden Answer (Severity Class)	0.875 (normalized)
Prediction Error	0.375

Retrieved Context:

Title: Subacute liver failure induced by adalimumab.
Most cases of liver toxicity associated with TNF-antagonists have been linked to infliximab and to a lesser extent to etanercept. So far only mild elevations of liver enzymes during therapy with adalimumab have been reported. In general, patients who developed ALT and AST elevations were asymptomatic and the abnormalities decreased or resolved with either continuation or discontinuation of adalimumab, or modification of concomitant medications. In this case report, we are presenting the first case of a patient without previous history of liver disease or concomitant risk factors for liver disease who developed subacute liver failure during therapy with adalimumab for... (truncated)

Title: Severe cholestasis due to adalimumab in a Crohn's disease patient.
Elevation of liver biochemistry has been reported with anti-tumor necrosis factor agents, but overt liver failure rarely reported. Autoimmune hepatitis has been more commonly reported with infliximab than adalimumab (ADA). Our case, however, describes the first reported case of ADA-associated severe cholestatic injury. A 39-year-old female with Crohn's disease developed severe jaundice after initiation of ADA. All serologic tests and imaging studies were normal. Liver biopsy showed prominent pericentral canalicular cholestasis, without features of steatosis or sclerosing cholangitis, consistent with drug-induced cholestasis. The serum total bilirubin peaked at 280 μmol/L, and improvement was seen after 5 wk with eventual normalization... (truncated)

Title: Successful treatment with etanercept of a patient with psoriatic arthritis after adalimumab-related hepatotoxicity.
Inhibitors of tumor necrosis factor (TNF) alpha (infliximab, etanercept, adalimumab) are nowadays widely used for the treatment of rheumatoid arthritis (RA), psoriatic arthritis (PsA) and ankylosing spondylitis (AS), not responding to conventional therapies. Anti-TNF alpha drugs have demonstrated great efficacy in slowing the disease, however, to date, concern still remains regarding acute and long-term toxicity related to TNF block. Increase in liver tests may be observed during treatment with anti-TNF agents, more often related to concomitant drugs (i.e. NSAIDS, methotrexate) or to reactivation of chronic HBV or HCV infections. However, liver damage directly induced by the drug has been described... (truncated)

Title: Drug induced autoimmune hepatitis and TNF-α blocking agents: is there a real relationship?
Hepatotoxicity is an expected side effect of tumour necrosis factor-α (anti-TNF-α) blocking agents including, infliximab, etanercept and adalimumab. Although mild to moderate elevations of liver enzymes have been recognised after the use of these agents, severe hepatitis is rarely reported. Reactivation of viral hepatitis and drug induced liver injury is two main causes of liver dysfunction in these patients. A broad spectrum, ranging from minor immunological alterations to systemic autoimmune disease, has been reported during treatment with anti-TNF-α. Therefore, in recent studies TNF-α blocking agents have been considered a potential cause of drug induced autoimmune hepatitis. Taking into account the... (truncated)

Title: Drug-induced liver injury caused by adalimumab: a case report and review of the bibliography.
The most serious adverse drug reaction of adalimumab (ADR) is tuberculosis reactivation. We describe a case of a 35-year-old man, with rheumatoid arthritis (RA) and hepatitis C virus genotype 1a with a liver biopsy in 2001 with a METAVIR score pattern A1 F0; he received interferon alpha 2b for six months, but treatment was suspended because of reactivation of RA. Liver function tests after treatment were similar to previous ones showing a minimal cholestatic pattern. In 2008, methotrexate was prescribed, but the drug was withdrawn at the third month because of the appearance of pruritus and Ggt rise. Viral load... (truncated)

MESH:D015774 - ganciclovir

Summary:

LLM Prediction Score	0.500 (normalized)
LLM Confidence Score	0.990
Golden Answer (Severity Class)	0.875 (normalized)
Prediction Error	0.375

Retrieved Context:

Title: Ganciclovir hepatotoxicity.
A 33-year-old male with acquired immunodeficiency syndrome received ganciclovir for presumed cytomegalovirus retinitis. Although results of baseline liver function tests were abnormal, marked elevations of transaminases and alkaline phosphatase occurred when the drug was first instituted, as well as after rechallenge. These elevated laboratory values declined on each occasion that the drug was withdrawn. As no other toxic or infectious insults could clearly be incriminated in these acute, self-limited episodes of hepatic function abnormalities, ganciclovir was most likely responsible for the toxicity observed in this patient.

Title: Ganciclovir for the treatment of disseminated CMV disease without pneumonia in allogeneic T-lymphocyte depleted bone marrow transplantation.
Treatment with ganciclovir was assessed in 13 patients who underwent allogeneic T-lymphocyte depleted bone marrow transplantation (BMT) for a variety of malignant hematological disorders and subsequently developed severe cytomegalovirus (CMV) disease without pneumonia. The manifestations of CMV disease appeared on days 23-105 (median 51) post BMT, and included gastrointestinal symptoms, weight loss, fever, disturbed liver function, leukopenia and thrombocytopenia. Ganciclovir was administered for 14 days, without the addition of intravenous immunoglobulins. Following therapy, the clinical manifestations subsided in most of the patients, while leukopenia, thrombocytopenia and liver dysfunction resolved in about half of the patients. One patient who experienced recurrent... (truncated)

Title: Cytomegalovirus hepatitis and ganciclovir treatment in immunocompetent children.
Ganciclovir treatment in children with cytomegalovirus (CMV) infection is still controversial and only indicated in selected cases. The aim of thi study was to evaluate clinical and demographic features of CMV hepatitis in immunocompetent children and to determine the effect of ganciclovir treatment in these patients retrospectively. The study was carried out in a group o 29 children with CMV hepatitis. All the patients were investigated for signs of infection, inborn errors of metabolism, genetic diseases, extrahepatic biliary atresia and other causes of hepatitis. Two patients with congenital CMV infection and two patients with biliary atresia were excluded from the... (truncated)

Title: Standard ganciclovir dosing results in slow decline of cytomegalovirus viral loads.
Cytomegalovirus (CMV) can cause severe disease, including rejection in transplant recipients. Ganciclovir and its oral prodrug valganciclovir have been used as first-line therapy for CMV disease in transplant recipients. The exposure targets of ganciclovir are not exactly known, and toxicity and resistance have interfered with ganciclovir therapy.

Title: Ganciclovir therapeutic drug monitoring in transplant recipients.
The use of (val)ganciclovir is complicated by toxicity, slow response to treatment and acquired resistance.

MESH:C579707 - fosaprepitant

Summary:

LLM Prediction Score	0.000 (normalized)
LLM Confidence Score	0.970
Golden Answer (Severity Class)	0.375 (normalized)
Prediction Error	0.375

Retrieved Context:

Title: The effects of intravenous fosaprepitant and ondansetron for the prevention of postoperative nausea and vomiting in neurosurgery patients: a prospective, randomized, double-blinded study.
The incidence of postoperative nausea and vomiting (PONV) is 30-50% after surgery. PONV occurs frequently, especially after craniotomy. In this study, we investigated the preventive effects on PONV in a randomized study by comparing patients who had been administered fosaprepitant, a neurokinin-1 (NK1) receptor antagonist, or ondansetron intravenously. Sixty-four patients undergoing craniotomy were randomly allocated to receive fosaprepitant 150 mg i.v. (NK1 group, n = 32) or ondansetron 4 mg i.v. (ONS group, n = 32) before anesthesia. The incidence of vomiting was significantly less in the NK1 group, where 2 of 32 (6%) patients experienced vomiting compared to 16... (truncated)

Title: A Comparison of Fosaprepitant and Ondansetron for Preventing Postoperative Nausea and Vomiting in Moderate to High Risk Patients: A Retrospective Database Analysis.
Postoperative nausea and vomiting (PONV) occur in 30-50% of patients undergoing general anesthesia and in 70-80% of high PONV risk patients. In this study, we investigated the efficacy of fosaprepitant, a neurokinin-1 (NK1) receptor antagonist, compared to ondansetron, a selective 5-hydroxytryptamine type 3 (5-HT3) receptor antagonist, in moderate to high PONV risk patients from our previous randomized controlled trials. Patients (171 patients from 4 pooled studies) with the Apfel simplified score ≥ 2 and undergoing general anesthesia were randomly allocated to receive intravenous fosaprepitant 150 mg (NK1 group, <i>n</i> = 82) and intravenous ondansetron 4 mg (ONS group, <i>n</i> = 89) before... (truncated)

Title: Safety of Polysorbate 80 in the Oncology Setting.
Polysorbate 80 is a synthetic nonionic surfactant used as an excipient in drug formulation. Various products formulated with polysorbate 80 are used in the oncology setting for chemotherapy, supportive care, or prevention, including docetaxel, epoetin/darbepoetin, and fosaprepitant. However, polysorbate 80, like some other surfactants, is not an inert compound and has been implicated in a number of systemic and injection- and infusion-site adverse events (ISAEs). The current formulation of intravenous fosaprepitant has been associated with an increased risk of hypersensitivity systemic reactions (HSRs). Factors that have been associated with an increased risk of fosaprepitant-related ISAEs include the site of administration... (truncated)

Title: Economic Value of Fosaprepitant-Containing Regimen in the Prevention of Chemotherapy-Induced Nausea and Vomiting in China: Cost-Effectiveness and Budget Impact Analysis.
The purpose of this study was to evaluate the cost-effectiveness and budget impact of fosaprepitant (FosAPR)-containing regimen for the prevention of chemotherapy-induced nausea and vomiting (CINV) among patients receiving high emetogenic chemotherapy (HEC) from the Chinese payer's perspective.

Title: NK1 receptor antagonists versus other antiemetics in the prevention of postoperative nausea and vomiting following laparoscopic surgical procedures: a systematic review and meta-analysis.
A systematic electronic search of MEDLINE, EMBASE, and CINAHL databases aimed at comparing neurokinin-1 receptor antagonists with other antiemetics in their prevention of postoperative nausea and vomiting in adult patients undergoing laparoscopic surgery identified seven randomized controlled trials for review and meta-analysis. Preoperative aprepitant 80 mg was found to reduce nausea (RR: 0.56, 95% CI: 0.41-0.75, I² = 0%, <i>P</i> = 0.89) and vomiting (RR: 0.20, 95% CI: 0.05-0.77, I² = 0%, <i>P</i> = 0.96) and resulted in complete response (RR: 1.61 (1.25-2.08), I² = 0%, <i>P</i> = 0.70) within the first 2 hours following surgery as well as vomiting... (truncated)

MESH:C105050 - tegaserod

Summary:

LLM Prediction Score	0.000 (normalized)
LLM Confidence Score	0.980
Golden Answer (Severity Class)	0.375 (normalized)
Prediction Error	0.375

Retrieved Context:

Title: Tegaserod for the Treatment of Irritable Bowel Syndrome.
Tegaserod (Zelnorm®) is a 5-hydroxytryptamine (serotonin) type 4 receptor agonist for the treatment of hypomotility disorders of the lower gastrointestinal tract associated with the irritable bowel syndrome with constipation (IBS-C).

Title: Repurposing the serotonin agonist Tegaserod as an anticancer agent in melanoma: molecular mechanisms and clinical implications.
New therapies are urgently needed in melanoma particularly in late-stage patients not responsive to immunotherapies and kinase inhibitors.

Title: The Zelnorm epidemiologic study (ZEST): a cohort study evaluating incidence of abdominal and pelvic surgery related to tegaserod treatment.
Pre-marketing clinical studies of tegaserod suggested an increased risk of abdominal surgery, particularly cholecystectomy. We sought to quantify the association between tegaserod use and the occurrence of abdominal or pelvic surgery, including cholecystectomy.

Title: Successful drug development despite adverse preclinical findings part 2: examples.
To illustrate the process of addressing adverse preclinical findings (APFs) as outlined in the first part of this review, a number of cases with unexpected APF in toxicity studies with drug candidates is discussed in this second part. The emphasis is on risk characterization, especially regarding the mode of action (MoA), and risk evaluation regarding relevance for man. While severe APFs such as retinal toxicity may turn out to be of little human relevance, minor findings particularly in early toxicity studies, such as vasculitis, may later pose a real problem. Rodents are imperfect models for endocrine APFs, non-rodents for human... (truncated)

Title: The safety of drugs used in acid-related disorders and functional gastrointestinal disorders.
Medicines are frequently used in the management of acid-related disorders and functional gastrointestinal disorders. With the exception of complicated peptic ulcer disease, these disorders are not associated with appreciable mortality. Drug treatments have consequently been held to the highest standards of safety. Some medicines have been withdrawn or restricted based on assessments and perceptions of risk. However, the risk of serious toxicity is low for most of the agents discussed in this article. Assessments are made of the safety and adverse-event profiles of certain drug classes and, where appropriate, individual medicines. For conditions with a low risk of mortality or... (truncated)

MESH:D000077604 - fomepizole

Summary:

LLM Prediction Score	0.000 (normalized)
LLM Confidence Score	0.970
Golden Answer (Severity Class)	0.375 (normalized)
Prediction Error	0.375

Retrieved Context:

Title: Use of fomepizole (4-methylpyrazole) for acetaminophen poisoning: A scoping review.
Acetaminophen (paracetamol, APAP) poisoning is a prominent global cause of drug-induced liver injury. While N-acetylcysteine (NAC) is an effective antidote, it has therapeutic limitations in massive overdose or delayed presentation. The objective is to comprehensively review the literature on fomepizole as a potential adjunct antidote for acetaminophen toxicity.

Title: Fomepizole as an adjunct in acetylcysteine treated acetaminophen overdose patients: a case series.
Acetaminophen (N-acetyl-para-aminophenol or APAP) is the leading cause of acute liver failure worldwide. Standard therapy for APAP overdose is with IV N-acetylcysteine (NAC). However, overdose patients treated with NAC can still incur hepatotoxicity in some circumstances. Fomepizole has proven safety in methanol and ethylene glycol poisoning and is a potent CYP2E1 and c-Jun-N-terminal Kinase (JNK) inhibitor that is effective even in the metabolic phase.

Title: Comparing N-acetylcysteine and 4-methylpyrazole as antidotes for acetaminophen overdose.
Acetaminophen (APAP) overdose can cause hepatotoxicity and even liver failure. N-acetylcysteine (NAC) is still the only FDA-approved antidote against APAP overdose 40 years after its introduction. The standard oral or intravenous dosing regimen of NAC is highly effective for patients with moderate overdoses who present within 8 h of APAP ingestion. However, for late-presenting patients or after ingestion of very large overdoses, the efficacy of NAC is diminished. Thus, additional antidotes with an extended therapeutic window may be needed for these patients. Fomepizole (4-methylpyrazole), a clinically approved antidote against methanol and ethylene glycol poisoning, recently emerged as a promising candidate.... (truncated)

Title: Novel Therapies for the Treatment of Drug-Induced Liver Injury: A Systematic Review.
Many drugs with different mechanisms of action and indications available on the market today are capable of inducing hepatotoxicity. Drug-induced liver injury (DILI) has been a treatment challenge nowadays as it was in the past. We searched Medline (<i>via</i> PubMed), CENTRAL, Science Citation Index Expanded, clinical trials registries and databases of DILI and hepatotoxicity up to 2021 for novel therapies for the management of adult patients with DILI based on the combination of three main search terms: 1) treatment, 2) novel, and 3) drug-induced liver injury. The mechanism of action of novel therapies, the potential of their benefit in clinical... (truncated)

Title: 4-methylpyrazole protects against acetaminophen-induced acute kidney injury.
Acetaminophen (APAP) hepatotoxicity is the most common cause of acute liver failure in the United States, and while a significant percentage of APAP overdose patients develop kidney injury, molecular mechanisms involved in APAP-induced nephrotoxicity are relatively unknown. We have shown that 4-methylpyrazole (4MP, Fomepizole) protects against APAP-induced liver injury by inhibiting reactive metabolite formation through Cyp2E1, and analysis of data from APAP overdose patients indicated that kidney dysfunction strongly correlated with severe liver injury. Since Cyp2E1 is also expressed in the kidney, this study explored protection by 4MP against APAP-induced nephrotoxicity. Male C57BL/6 J mice were treated with either 300... (truncated)

MESH:D015283 - citalopram

Summary:

LLM Prediction Score	0.500 (normalized)
LLM Confidence Score	0.990
Golden Answer (Severity Class)	0.875 (normalized)
Prediction Error	0.375

Retrieved Context:

Title: Liver injury associated with antidepressants.
Antidepressants are commonly prescribed and used in the management of depression, anxiety disorders, and other psychiatric illnesses. Antidepressants used in therapeutic dosing ranges are associated with causing several adverse drug reactions including hepatotoxicity. Paroxetine, fluoxetine, fluvoxamine, citalopram, mirtazapine and venlafaxine are associated with reversible liver injury upon discontinuation of the agent. Patient cases of hepatotoxicity involving the use of nefazodone, trazodone, duloxetine, bupropion, and sertraline are linked to causing death in its users. Due to the idiosyncratic nature of hepatotoxicity, monitoring of liver function tests and immediate discontinuation upon abnormal lab findings or signs and symptoms of liver dysfunction are... (truncated)

Title: Citalopram-Induced Long QT Syndrome and the Mammalian Dive Reflex.
While SCUBA diving, a 44-year-old Caucasian patient had an abnormal cardiac rhythm, presumably Torsade de Pointes (TdP), during the initial descent to depth. Upon surfacing, she developed ventricular fibrillation and died. The patient had been treated for mild depression for nearly a year with citalopram 60 mg per day, a drug known to cause prolonged QT interval. She had also been treated with two potentially hepatotoxic drugs. Liver impairment causes selective loss of cytochrome P450 (CYP) 2C19 activity, the major pathway for metabolism of citalopram. The post mortem blood level of citalopram was 1300 ng/mL. The patient was found to... (truncated)

Title: Antidepressants and Hepatotoxicity: A Cohort Study among 5 Million Individuals Registered in the French National Health Insurance Database.
Hepatotoxicity may be a concern when prescribing antidepressants. Nevertheless, this risk remains poorly understood for serotonin and noradrenaline reuptake inhibitors (SNRIs: venlafaxine, milnacipran, duloxetine) and 'other antidepressants' (mianserin, mirtazapine, tianeptine and agomelatine), particularly in comparison with selective serotonin reuptake inhibitors (SSRIs: fluoxetine, citalopram, paroxetine, sertraline, fluvoxamine, escitalopram), which are by far the most commonly prescribed antidepressants.

Title: Histological changes in the liver of fetuses of pregnant rats following citalopram administration.
Depression is a dilapidating disorder, which may occur during pregnancy. Citalopram is an antidepressant drug often prescribed to pregnant women. The purpose of the present study is to determine whether maternal administration of citalopram affects fetal liver histology.

Title: Forensic Aspects of a Fatal Intoxication Involving Acetaminophen, Citalopram and Trazodone: A Case Report.
We report the case of a young man, a former heroin addict, found dead at home by the Police Forces in an advanced state of decomposition. Numerous blisters and unpacked tablets of medications were found all over the bed and on the floor of the room. Multiple injuries to the face, left arm and neck of the deceased were noted. The latter damages were attributed to post-mortem dog bites, since no indications of a possible defense against the animal were observed. The autopsy findings were unremarkable. Toxicological investigations performed on peripheral blood and urine by gas chromatography-mass spectrometry (GC-MS) technique... (truncated)

MESH:D016572 - cyclosporine

Summary:

LLM Prediction Score	0.500 (normalized)
LLM Confidence Score	0.990
Golden Answer (Severity Class)	0.875 (normalized)
Prediction Error	0.375

Retrieved Context:

Title: [Effects of cyclosporin A on various indices of cholestasis in kidney transplant recipients].
A cholestatic syndrome has been reported as one of the main side effects of CyA therapy. The aim of the present study was to evaluate frequency and degree of severity of the cholestatic syndrome in a group of patients with renal transplant treated with CyA. In 55 patients we evaluated both clinical: jaundice, pruritus, presence of biliary lithiasis and biochemical parameters: total serum biliary salts (TBS), total bilirubin (TB), alkaline phosphatase (AP), gammaglutamyl transpeptidase (GGT), transaminase (AST, ALT), cholesterol (CT), triglycerides (TG), HDL-cholesterol (HDL-C) and compared them with a control group matched for sex and age. In the transplant patients... (truncated)

Title: Hepatotoxicity caused by both tacrolimus and cyclosporine after living donor liver transplantation.
We present a case report of a posttransplant patient who had hepatotoxicity due to both tacrolimus and cyclosporine and cholestatic jaundice due to tacrolimus. The patient did not show sustained improvement in enzyme and bilirubin abnormalities after an initial change from tacrolimus to cyclosporine or with a change back to tacrolimus, but he ultimately showed improvement when the blood concentration of tacrolimus was lowered. A 56-year-old man with subacute fulminant hepatitis induced by acarbose was admitted to our hospital for living donor liver transplantation. The liver graft consisted of the left lobe from his ABO-identical son. The early posttransplant course... (truncated)

Title: Ciclosporin metabolite pattern in blood and urine of kidney graft patients in relation to liver function.
Ciclosporin, an immunosuppressant, is metabolized by the liver cytochrome P450 system. Changes in the pattern of its metabolites in blood and urine in patients with disturbed liver function have been studied. Forty seven kidney graft patients receiving 2.9 mg/kg/d ciclosporin b.i.d., and no additional medication that would interfere with ciclosporin metabolism, were allocated to three groups according to liver function: I with normal liver function (n = 19), II with elevated liver enzyme activity or bilirubin concentration in serum (n = 20), and III with cholestasis (n = 8). Ciclosporin and 17 metabolites were determined in blood and 24 h-urine.... (truncated)

Title: Cyclosporine-induced worsening of hepatic dysfunction in a patient with Crohn's disease and enterocutaneous fistula.
Cyclosporine recently has become part of the treatment regimen for patients with refractory inflammatory bowel disease, though it is still considered investigational for that purpose. However, little attention has been given to the potential hepatotoxicity associated with cyclosporine administration. This can be especially significant in patients with preexisting abnormalities of liver function, including those induced by total parenteral nutrition.

Title: Involvement of oxidative species in cyclosporine-mediated cholestasis.
Cyclosporine is an established medication for the prevention of transplant rejection. However, adverse consequences such as nephrotoxicity, hepatotoxicity, and cholestasis have been associated with prolonged usage. In cyclosporine-induced obstructive and chronic cholestasis, for example, the overproduction of oxidative stress is significantly increased. Additionally, cyclosporine exerts adverse effects on liver function and redox balance responses in treated rats, as evidenced by its increasing levels of aspartate aminotransferase (AST), alanine aminotransferase (ALT), and bilirubin while also decreasing the levels of glutathione and NADPH. Cyclosporine binds to cyclophilin to produce its therapeutic effects, and the resulting complex inhibits calcineurin, causing calcium to accumulate... (truncated)

MESH:D000077267 - fulvestrant

Summary:

LLM Prediction Score	0.500 (normalized)
LLM Confidence Score	0.980
Golden Answer (Severity Class)	0.875 (normalized)
Prediction Error	0.375

Retrieved Context:

Title: Grade 3 Hepatotoxicity following Fulvestrant, Palbociclib, and Erdafitinib Therapy in a Patient with ER-Positive/PR-Negative/HER2-Negative Metastatic Breast Cancer: A Case Report.
A 49-year-old woman with ER-positive/PR-negative/HER2-negative metastatic breast cancer experienced Grade 3 hepatotoxicity following initiation of a clinical trial of fulvestrant, palbociclib, and erdafitinib. Fulvestrant was determined to be the drug most likely responsible for this hepatotoxic effect. This case report details the timing and nature of this drug-induced liver injury, adding support to an area that has yet to be described adequately in the existing literature.

Title: A phase II neoadjuvant trial of anastrozole, fulvestrant, and gefitinib in patients with newly diagnosed estrogen receptor positive breast cancer.
Endocrine therapy in patients with breast cancer can be limited by the problem of resistance. Preclinical studies suggest that complete blockade of the estrogen receptor (ER) combined with inhibition of the epidermal growth factor receptor can overcome endocrine resistance. We tested this hypothesis in a phase II neoadjuvant trial of anastrozole and fulvestrant combined with gefitinib in postmenopausal women with newly diagnosed ER-positive breast cancer. After a baseline tumor core biopsy, patients were randomized to receive anastrozole and fulvestrant or anastrozole, fulvestrant, and gefitinib (AFG) for 3 weeks. After a second biopsy at 3 weeks, all patients received AFG for... (truncated)

Title: Short-term tamoxifen administration improves hepatic steatosis and glucose intolerance through JNK/MAPK in mice.
Nonalcoholic fatty liver disease (NAFLD) which is a leading cause of chronic liver diseases lacks effective treatment. Tamoxifen has been proven to be the first-line chemotherapy for several solid tumors in clinics, however, its therapeutic role in NAFLD has never been elucidated before. In vitro experiments, tamoxifen protected hepatocytes against sodium palmitate-induced lipotoxicity. In male and female mice fed with normal diets, continuous tamoxifen administration inhibited lipid accumulation in liver, and improved glucose and insulin intolerance. Short-term tamoxifen administration largely improved hepatic steatosis and insulin resistance, however, the phenotypes manifesting inflammation and fibrosis remained unchanged in abovementioned models. In addition,... (truncated)

Title: Fulvestrant-Based Combination Therapy for Second-Line Treatment of Hormone Receptor-Positive Advanced Breast Cancer.
Fulvestrant is recommended for patients with hormone receptor-positive (HR+) advanced breast cancer (ABC) who progress after aromatase inhibitor therapy. As most patients in this setting have already developed mechanisms of resistance to endocrine therapy, targeting biological pathways associated with endocrine resistance in combination with fulvestrant may improve outcomes. Therefore, evidence supporting a combinatorial treatment approach in the second-line setting was investigated based on a search of PubMed and ClinicalTrials.gov . Twenty-eight studies of targeted therapies plus fulvestrant as second-line treatment for HR+ ABC were identified, including three and six key randomized trials exploring cyclin-dependent kinase 4/6 (CDK4/6) inhibitors and phosphatidylinositol... (truncated)

Title: Hepatotoxicity After CDK 4/6 Inhibitor Initiation in the Treatment of Hormone-Positive Metastatic Breast Cancer.
Cancer cells proliferate using various mechanisms. One mechanism of preventing tumor cell growth is blockade of the cyclin-dependent kinase (CDK) 4/6 axis. Multiple CDK 4/6 inhibitors - ribociclib, palbociclib, and abemaciclib - have significantly improved progression-free survival rates. However, they can cause hepatotoxicity. We present a case of a 67-year-old female who was diagnosed with stage 1C invasive ductal carcinoma. She was treated with letrozole and ribociclib due to recurrence as metastatic disease, but within 10 days, she developed transaminitis. She then started palbociclib but experienced elevated transaminases within two weeks, needing discontinuation of palbociclib. Subsequent positron-emission tomography/computed tomography imaging... (truncated)

MESH:C062876 - cetrorelix

Summary:

LLM Prediction Score	0.000 (normalized)
LLM Confidence Score	0.950
Golden Answer (Severity Class)	0.375 (normalized)
Prediction Error	0.375

Retrieved Context:

Title: Melatonin inhibits hypothalamic gonadotropin-releasing hormone release and reduces biliary hyperplasia and fibrosis in cholestatic rats.
Melatonin is a hormone produced by the pineal gland with increased circulating levels shown to inhibit biliary hyperplasia and fibrosis during cholestatic liver injury. Melatonin also has the capability to suppress the release of hypothalamic gonadotropin-releasing hormone (GnRH), a hormone that promotes cholangiocyte proliferation when serum levels are elevated. However, the interplay and contribution of neural melatonin and GnRH to cholangiocyte proliferation and fibrosis in bile duct-ligated (BDL) rats have not been investigated. To test this, cranial levels of melatonin were increased by implanting osmotic minipumps that performed an intracerebroventricular (ICV) infusion of melatonin or saline for 7 days starting... (truncated)

Title: Unsupervised identification of disease states from high-dimensional physiological and histopathological profiles.
The liver and kidney in mammals play central roles in protecting the organism from xenobiotics and are at high risk of xenobiotic-induced injury. Xenobiotic-induced tissue injury has been extensively studied from both classical histopathological and biochemical perspectives. Here, we introduce a machine-learning approach to analyze toxicological response. Unsupervised characterization of physiological and histological changes in a large toxicogenomic dataset revealed nine discrete toxin-induced disease states, some of which correspond to known pathology, but others were novel. Analysis of dynamics revealed transitions between disease states at constant toxin exposure, mostly toward decreased pathology, implying induction of tolerance. Tolerance correlated with induction... (truncated)

Title: Disruption of the FasL/Fas axis protects against inflammation-derived tumorigenesis in chronic liver disease.
Fas Ligand (FasL) and Fas (APO-1/CD95) are members of the TNFR superfamily and may trigger apoptosis. Here, we aimed to elucidate the functional role of Fas signaling in an experimental model of chronic liver disease, the hepatocyte-specific NEMO knockout (NEMO^{Δhepa}) mice. We generated NEMO^{Δhepa} /Fas^{lpr} mice, while NEMO^{Δhepa}, NEMO^{f/f} as well as Fas^{lpr}animals were used as controls, and characterized their phenotype during liver disease progression. Liver damage was evaluated by serum transaminases, histological, immunofluorescence procedures, and biochemical and molecular biology techniques. Proteins were detected by western Blot, expression of mRNA by RT-PCR, and infiltration of inflammatory cells was determined by... (truncated)

Title: TJ-M2010-5, A self-developed MyD88 inhibitor, attenuates liver fibrosis by inhibiting the NF-κB pathway.
Liver fibrosis is the result of most chronic inflammatory liver damage and seriously endangers human health. However, no drugs have been approved to treat this disease. Previous studies showed that the Toll-like receptors (TLRs)/myeloid differentiation factor-88 (MyD88)/nuclear factor-κB (NF-κB) pathway plays a key role in liver fibrosis. TJ-M2010-5 is a self-developed small molecule MyD88 inhibitor, which has been proven to have a good protective effect in a variety of inflammatory disease models. In the present study, to investigate the anti-fibrotic effect of TJ-M2010-5, mice were injected with carbon tetrachloride (CCl₄) in vivo and LX2 cells (a human hepatic stellate cell... (truncated)

Title: Effect of trichloroacetaldehyde on the activation of CD4⁺T cells in occupational medicamentosa-like dermatitis: An in vivo and in vitro study.
Occupational medicamentosa-like dermatitis induced by trichloroethylene (OMLDT) is a hypersensitivity disease with autoimmune liver injury, which has increasingly become a serious occupational health problem in China. However, the pathogenesis of OMLDT remained undefined. In this study, 30 TCE-induced OMLDT patients, 58 exposure controls, and 40 non-exposure controls were recruited. We showed that the ratio of activated CD4⁺ T cells (downregulation of CD62 L) was dramatically increased in OMLDT patients compared to exposure and non-exposure control, suggesting that CD4⁺ T cells activation was a key cellular event in the development of

MESH:D011224 - prazosin

Summary:

LLM Prediction Score	0.000 (normalized)
LLM Confidence Score	0.980
Golden Answer (Severity Class)	0.375 (normalized)
Prediction Error	0.375

Retrieved Context:

Title: Propranolol, a β-adrenoceptor antagonist, worsens liver injury in a model of non-alcoholic steatohepatitis.
Prazosin an α1-adrenoceptor (AR) antagonist has been shown to reduce liver injury in a mouse model of non-alcoholic steatohepatitis (NASH) and is suggested as a potential treatment of NASH especially given its concomitant anti-fibrotic properties. The effect however, of β-AR blockade in non-cirrhotic NASH is unknown and is as such investigated here. In the presence of the β-blocker propranolol (PRL), mice fed normal chow or a half methionine and choline deficient diet, supplemented with ethionine (HMCDE), to induce NASH, showed significantly enhanced liver injury, as evidenced by higher hepatic necrosis scores and elevated serum aminotransferases (ALT). Mechanistically, we showed that... (truncated)

Title: Define Mesenchymal Stem Cell from Its Fate: Biodisposition of Human Mesenchymal Stem Cells in Normal and Concanavalin A-Induced Liver Injury Mice.
The pharmaceutical industry and clinical trials have been revolutionized mesenchymal stem cell-based therapeutics. However, the pharmacokinetics of transplanted cells has been little characterized in their target tissues under healthy or disease condition. A quantitative polymerase chain reaction analytical method with matrix effect was developed to track the biodistribution of human mesenchymal stem cells in normal mice and those with Concanavalin A (Con A)-induced liver injury. Mesenchymal stem/stromal cell (MSC) disposition in blood and different organs were compared, and relevant pharmacokinetic parameters were calculated. Human MSCs (hMSCs) and mouse MSCs (mMSCs) displayed a very similar pharmacokinetic profile in all tested doses:... (truncated)

Title: Doxazosin Attenuates Liver Fibrosis by Inhibiting Autophagy in Hepatic Stellate Cells via Activation of the PI3K/Akt/mTOR Signaling Pathway.
To investigate the effect of doxazosin on autophagy and the activation of hepatic stellate cells (HSCs) in vivo and in vitro and determine the underlying mechanism.

Title: Altered Function and Expression of ABC Transporters at the Blood-Brain Barrier and Increased Brain Distribution of Phenobarbital in Acute Liver Failure Mice.
This study investigated alterations in the function and expression of P-glycoprotein (P-GP), breast cancer resistance protein (BCRP), and multidrug resistance-associated protein 2 (MRP2) at the blood-brain barrier (BBB) of acute liver failure (ALF) mice and its clinical significance. ALF mice were developed using intraperitoneal injection of thioacetamide. P-GP, BCRP, and MRP2 functions were determined by measuring the ratios of brain-to-plasma concentration of rhodamine 123, prazosin, and dinitrophenyl-<i>S</i>-glutathione, respectively. The mRNA and proteins expression levels of P-GP, BCRP, and MRP2 were evaluated with quantitative real-time PCR and western blot, respectively. MDCK-MDR1 and HCMEC/D3 cells were used to document the effects of... (truncated)

Title: Neuroimmunomodulation of adrenoblockers during liver cirrhosis: modulation of hepatic stellate cell activity.
The sympathetic nervous system and the immune system are responsible for producing neurotransmitters and cytokines that interact by binding to receptors; due to this, there is communication between these systems. Liver immune cells and nerve fibres are systematically distributed in the liver, and the partial overlap of both patterns may favour interactions between certain elements. Dendritic cells are attached to fibroblasts, and nerve fibres are connected via the dendritic cell-fibroblast complex. Receptors for most neuroactive substances, such as catecholamines, have been discovered on dendritic cells. The sympathetic nervous system regulates hepatic fibrosis through sympathetic fibres and adrenaline from the adrenal... (truncated)

MESH:D015766 - albendazole

Summary:

LLM Prediction Score	0.500 (normalized)
LLM Confidence Score	0.990
Golden Answer (Severity Class)	0.875 (normalized)
Prediction Error	0.375

Retrieved Context:

Title: [Drug Induced Liver Injury by Prophylactic Administration of Albendazole].
Albendazole is used as a typical antiparasitic agent worldwide. The side effects of albendazole may include nausea, vomiting, abdominal pain, dizziness, headache, alopecia, and increased liver enzymes. Mild elevation of the liver enzyme has been reported in more than 10% of cases, but drug induced liver injury was reported to be very rare. A 30-year-old woman visited the Dong-A University Hospital with anorexia, nausea, jaundice, and elevated liver enzyme. For diagnosis, other acute hepatitis etiologies were excluded, but the prophylactic administration of albendazole was verified. This paper introduces a case of drug-induced liver injury through the prophylactic administration of albendazole.... (truncated)

Title: Albendazole Induced Recurrent Acute Toxic Hepatitis: A Case Report.
Drug induced acute toxic hepatitis can be idiosyncratic. Albendazole, a widely used broad spectrum antiparasitic drug is generally accepted as a safe drug. It may cause asymptomatic transient liver enzyme abnormalities but acute toxic hepatitis is very rare. Case Report : Herein, we present the case of 47 year old woman with recurrent acute toxic hepatitis after a single intake of albendazole in 2010 and 2014. The patient was presented with symptoms and findings of anorexia, vomiting and jaundice. For diagnosis, other acute hepatitis etiologies were excluded. Roussel Uclaf Causality Assessment Method (RUCAM) score was calculated and found to be... (truncated)

Title: Acute drug-induced hepatitis caused by albendazole.
Albendazole binds to parasite's tubulin inhibiting its glucose absorption. Its common adverse effects are nausea, vomiting, constipation, thirst, dizziness, headache, hair loss and pruritus. Although mainly metabolized in the liver, abnormal liver function tests were a rare adverse effect during clinical trials and we found no literature about albendazole-induced hepatitis requiring admission. This patient had a previous history of albendazole ingestion in 2002 resulting in increase of liver function tests. And in 2005, the episode repeated. We evaluated the patient for viral hepatitis, alcoholic liver disease, and autoimmune hepatitis, but no other cause of hepatic injury could be found. Liver... (truncated)

Title: HLA-targeted sequencing reveals the pathogenic role of HLA-B*15:02/HLA-B*13:01 in albendazole-induced liver failure: a case report and a review of the literature.
Drug-induced liver injury (DILI) is one of the serious adverse drug reactions (ADRs), which belongs to immune-mediated adverse drug reactions (IM-ADRs). As an essential health drug, albendazole has rarely been reported to cause serious liver damage. A young man in his 30 s developed severe jaundice, abnormal transaminases, and poor blood coagulation mechanism after taking albendazole, and eventually developed into severe liver failure. The patient was found heterozygous of <i>HLA-B*15:02</i> and <i>HLA-B*13:01</i> through HLA-targeted sequencing, which may have a pathogenic role in the disease. This case report summarizes his presentation, treatment, and prognosis. A useful summary of the diagnosis and associated... (truncated)

Title: Albendazole-induced liver injury: a case report.
We report a case of a 47-year-old male, who was referred to the clinical hepatology services at Pablo Tobón Uribe Hospital for evaluation of a jaundice syndrome. After undergoing several exams, we diagnosed hepatic hydatidosis and the patient was treated with albendazole; however, after five months of uninterrupted treatment the patient again consulted and his liver test showed marked hepatocellular damage. This time, the patient was diagnosed with drug-induced liver injury due to albendazole, based on information from the clinical record, history of drug consumption, clinical and laboratory tests improved after discontinuing the medication and after discarding other possible causes;... (truncated)

MESH:D000068799 - prasugrel hydrochloride

Summary:

LLM Prediction Score	0.374 (normalized)
LLM Confidence Score	0.940
Golden Answer (Severity Class)	0.0 (normalized)
Prediction Error	0.374

Retrieved Context:

Title: Gastrointestinal adverse events after dual antiplatelet therapy: clopidogrel is safer than ticagrelor, but prasugrel data are lacking or inconclusive.
Current guidelines offer a choice of P2Y12 receptor antagonist among clopidogrel, prasugrel or ticagrelor on top of aspirin (ASA) for dual antiplatelet therapy (DAPT) in patients after acute coronary syndromes (ACS). However, the comparative risks of gastrointestinal (GI) adverse events during DAPT are not clear. Two large ACS trials (TRITON and PLATO) provide a valuable opportunity to directly match the risks of GI complications among current antiplatelet regimens. We compared the rates of GI adverse events after prasugrel and ticagrelor versus clopidogrel based on the Food and Drug Administration (FDA) clinical safety reviews. When compared with ticagrelor, clopidogrel is safer... (truncated)

Title: Prasugrel-related hepatotoxicity.
Prasugrel is usually preferred over Clopidogrel to reduce the risk of recurrent coronary thrombosis in patients who undergo percutaneous coronary interventions during an acute coronary syndrome owing to its more potent and more rapid antithrombotic activation. Little is known about Prasugrel-induced hepatotoxicity, although mild-to-moderate alanine transaminase (ALT) and gamma glutamyl transpeptidase (GGT) elevations have been noticed in post-marketing surveillance. Herein, we report the case of a patient with Prasugrel-related hepatotoxicity that was reverted after switching from Prasugrel to Ticagrelor.

Title: Erosive arthritis and hepatic granuloma formation induced by peptidoglycan polysaccharide in rats is aggravated by prasugrel treatment.
Administration of the thienopyridine P2Y12 receptor antagonist, clopidogrel, increased the erosive arthritis induced by peptidoglycan polysaccharide (PG-PS) in rats or by injection of the arthritogenic K/BxN serum in mice. To determine if the detrimental effects are caused exclusively by clopidogrel, we evaluated prasugrel, a third-generation thienopyridine pro-drug, that contrary to clopidogrel is mostly metabolized into its active metabolite in the intestine. Prasugrel effects were examined on the PG-PS-induced arthritis rat model. Erosive arthritis was induced in Lewis rats followed by treatment with prasugrel for 21 days. Prasugrel treated arthritic animals showed a significant increase in the inflammatory response, compared with... (truncated)

Title: Proton pump inhibitors and gastroprotection in patients treated with antithrombotic drugs: A cardiologic point of view.
Aspirin, other antiplatelet agents, and anticoagulant drugs are used across a wide spectrum of cardiovascular and cerebrovascular diseases. A concomitant proton pump inhibitor (PPI) treatment is often prescribed in these patients, as gastrointestinal complications are relatively frequent. On the other hand, a potential increased risk of cardiovascular events has been suggested in patients treated with PPIs; in particular, it has been discussed whether these drugs may reduce the cardiovascular protection of clopidogrel, due to pharmacodynamic and pharmacokinetic interactions through hepatic metabolism. Previously, the concomitant use of clopidogrel and omeprazole or esomeprazole has been discouraged. In contrast, it remains less known... (truncated)

Title: Prasugrel effectively reduces the platelet reactivity units in patients with genetically metabolic dysfunction of cytochrome P450 2C19 who are treated with long-term dual antiplatelet therapy after undergoing drug-eluting stent implantation.
Dual antiplatelet therapy (DAPT) with aspirin and P2Y₁₂ inhibitor is administered following percutaneous coronary intervention (PCI) with coronary stent implantation. Several studies have reported the effects of switching between P2Y₁₂ inhibitors on platelet reactivity (P2Y₁₂ reaction units: PRU), from acute to late phase after PCI. However, the effect of switching at very late phase is unknown. This study examined the effect on PRU in Japanese coronary heart disease patients with long-term DAPT (aspirin + clopidogrel) when switching from clopidogrel to prasugrel. Ninety-six patients were enrolled in this study. The median DAPT duration at enrollment was 1824.0 days. Twenty-three patients with PRU ≥ 208 at enrollment... (truncated)

MESH:D008687 - metformin

Summary:

LLM Prediction Score	0.374 (normalized)
LLM Confidence Score	0.990
Golden Answer (Severity Class)	0.0 (normalized)
Prediction Error	0.374

Retrieved Context:

Title: Literature review of the clinical characteristics of metformin-induced hepatotoxicity.
Background: Knowledge of metformin-induced hepatotoxicity is based on case reports. The aim of this study was to investigate the clinical features of metformin-induced hepatotoxicity. Methods: We collected relevant literature on metformin-induced hepatotoxicity published from January 1994 to February 2022 by searching Chinese and English databases. Results: Thirty patients (19 males and 11 females) from 29 articles were included, with a median age of 61 years (range 29-83). The median time to onset of liver injury was 4 weeks (range 0.3-648) after metformin administration. Clinical symptoms occurred in 28 patients, including gastrointestinal reactions (56.7%), jaundice (50.0%), fatigue (36.7%), anorexia (23.3%), pruritus (13.3%), dark... (truncated)

Title: [Metformin-associated hepatotoxicity].
Metformin is an oral biguanide widely used in the management of patients with type 2 diabetes. It produces non-specific gastrointestinal symptoms in 10-30% of the patients. Lactic acidosis is the most serious side effect, so it must not be administered to patients with renal, liver, or heart insufficiency. Only a few cases of hepatotoxicity due to this drug have been documented. We report the case of a patient with type 2 diabetes mellitus and recent use of metformin who developed serious liver injury, followed by a favorable evolution.

Title: Metformin as a Rare Cause of Drug-Induced Liver Injury, a Case Report and Literature Review.
Metformin is an oral hypoglycemic agent that is commonly used in the treatment of type 2 diabetes mellitus. Although metformin-associated gastrointestinal upset and metabolic acidosis is widely recognized side effect of this drug, metformin-induced liver injury has been rarely reported in the literature. In most cases reported, metformin-induced liver injury was associated with concomitant intake of other hepatotoxic drugs. Here, we report a case of a 70-year-old white woman who suffered metformin-induced liver injury 5 weeks after starting on this medication, and she was not on any other hepatotoxic agent. With increasing prescription of metformin, this case deserves particular attention... (truncated)

Title: Hepatotoxicity associated with metformin therapy in treatment of type 2 diabetes mellitus with nonalcoholic fatty liver disease.
To report a case of idiosyncratic hepatotoxicity associated with metformin in the treatment of type 2 diabetes with nonalcoholic fatty liver disease (NAFLD).

Title: Editor's Highlight: Metformin Protects Against Acetaminophen Hepatotoxicity by Attenuation of Mitochondrial Oxidant Stress and Dysfunction.
Overdose of acetaminophen (APAP) causes severe liver injury and even acute liver failure in both mice and human. A recent study by Kim et al. (2015, Metformin ameliorates acetaminophen hepatotoxicity via Gadd45β-dependent regulation of JNK signaling in mice. J. Hepatol. 63, 75-82) showed that metformin, a first-line drug to treat type 2 diabetes mellitus, protected against APAP hepatotoxicity in mice. However, its exact protective mechanism has not been well clarified. To investigate this, C57BL/6J mice were treated with 400 mg/kg APAP and 350 mg/kg metformin was given 0.5 h pre- or 2 h post-APAP. Our data showed that pretreatment with... (truncated)

MESH:D007981 - levorphanol

Summary:

LLM Prediction Score	0.371 (normalized)
LLM Confidence Score	0.980
Golden Answer (Severity Class)	0.0 (normalized)
Prediction Error	0.371

Retrieved Context:

Title: Ensuring competency in end-of-life care: controlling symptoms.
BACKGROUND: Palliative medicine is assuming an increasingly important role in patient care. The Education for Physicians in End-of-life Care (EPEC) Project is an ambitious program to increase core palliative care skills for all physicians. It is not intended to transmit specialty level competencies in palliative care. METHOD: The EPEC Curriculum was developed to be a comprehensive syllabus including trainer notes, multiple approaches to teaching the material, slides, and videos of clinical encounters to trigger discussion are provided. The content was developed through a combination of expert opinion, participant feedback and selected literature review. Content development was guided by the goal... (truncated)

Title: What Do We Know about Opioids and the Kidney?
Evidence suggests a link between opioid use and kidney disease. This review summarizes the known renal manifestations of opioid use including its role in acute and chronic kidney injury. Both the direct and indirect effects of the drug, and the context which leads to the development of renal failure, are explored. While commonly used safely for pain control and anesthesia in those with kidney disease, the concerns with respect to side effects and toxicity of opioids are addressed. This is especially relevant with the worldwide increase in the use of opioids for medical and recreational use.

Title: Genetically determined oxidation polymorphism and drug hepatotoxicity. Study of 51 patients.
The influence of genetically determined oxidation polymorphism on drug hepatotoxicity has been poorly investigated and results are controversial. We studied drug oxidation capacity in 51 patients with hepatitis caused mainly by drugs undergoing oxidative metabolism, using dextromethorphan, a test compound recently proposed as a substitute for debrisoquine. Phenotyping was performed using the metabolic ratio (MR) calculated as MR = 0-10 h urinary output of dextromethorphan/0-10 h urinary output of dextrorphan (the main oxidative metabolite), after oral administration of 40 mg dextromethorphan hydrobromide. Dextromethorphan oxidation capacity was similar in patients and in 103 control subjects as judged by: (a) the prevalence... (truncated)

Title: State of the Art and Uses for the Biopharmaceutics Drug Disposition Classification System (BDDCS): New Additions, Revisions, and Citation References.
The Biopharmaceutics Drug Disposition Classification system (BDDCS) is a four-class approach based on water solubility and extent of metabolism/permeability rate. Based on the BDDCS class to which a drug is assigned, it is possible to predict the role of metabolic enzymes and transporters on the drug disposition of a new molecular entity (NME) prior to its administration to animals or humans. Here, we report a total of 1475 drugs and active metabolites to which the BDDCS is applied. Of these, 379 are new entries, and 1096 are revisions of former classification studies with the addition of references for the approved... (truncated)

Title: Antipsychotic Drugs Efficacy in Dextromethorphan-Induced Psychosis.
Psychosis is known as a broad term of symptoms that cause serious disorganization of behavior, thinking, and perception of reality. One of the medicines that recently gained much attention in terms of its psychotic potential is dextromethorphan (DXM). DXM, a widely used antitussive drug, is a commonly abused drug because of its euphoric, hallucinogenic, and dissociative properties. To date, DXM is a legally marketed cough suppressant that is neither a controlled substance nor a regulated chemical under the Controlled Substances Act. The management of DXM-related psychosis is dependent on the type of psychotic symptoms. Atypical neuroleptics (i.e., olanzapine, risperidone, quetiapine)... (truncated)

MESH:D015122 - mercaptopurine

Summary:

LLM Prediction Score	0.630 (normalized)
LLM Confidence Score	0.990
Golden Answer (Severity Class)	1.0 (normalized)
Prediction Error	0.370

Retrieved Context:

Title: Thiopurine-induced liver injury in patients with inflammatory bowel disease: a systematic review.
The mean prevalence of azathioprine (AZA) or 6-mercaptopurine (MP)-induced liver injury in patients with inflammatory bowel disease was approximately 3%, and the mean annual drug-induced liver disorder rate was only 1.4%. However, this low figure calculated from retrospective studies contrasts with a much higher incidence (>10%) reported by a prospective study. Thiopurine-induced hepatotoxicity can be grouped into three syndromes: hypersensitivity, idiosyncratic cholestatic reaction, and endothelial cell injury (with resultant raised portal pressures, veno-occlusive disease, or peliosis hepatis). A small percentage of patients present with a slight elevation of liver tests (LTs) that do not have clinical implications and LTs return... (truncated)

Title: Mercaptopurine rescue after azathioprine-induced liver injury in inflammatory bowel disease.
Azathioprine (AZA) liver toxicity arises in approximately 3% of inflammatory bowel disease patients and may result in treatment discontinuation.

Title: Mercaptopurine-induced hepatoportal sclerosis in a patient with Crohn's disease.
Thiopurines play a pivotal role in the management of inflammatory bowel disease. Azathioprine and mercaptopurine have been associated with a number of liver abnormalities, including hepatitis, veno-occlusive disease, nodular regenerative hyperplasia, and peliosis hepatitis. Patients treated with azathioprine and mercaptopurine have their liver chemistry tests routinely checked due to this potential for hepatotoxicity. Hepatoportal sclerosis is a cause of non-cirrhotic portal hypertension that is increasingly being recognized; its etiopathogenesis is not well defined. We present the first case report of mercaptopurine-induced hepatoportal sclerosis leading to non-cirrhotic portal hypertension in a patient with Crohn's disease. He had been treated with mercaptopurine... (truncated)

Title: Diagnosis of 6 mercaptopurine hepatotoxicity post liver transplantation utilizing metabolite assays.
Azathioprine and 6-mercaptopurine (6 MP) are commonly used as immunosuppression postsolid organ transplantation. Recently, a better understanding of the metabolism of these drugs has developed. 6 Mercaptopurine is metabolized by thiopurine methyl transferase (TPMT) which is under the control of a common genetic polymorphism. Genetic testing and measurement of levels of 6 MP metabolites allow identification of patients at risk of toxicity. We report two cases of cholestatic hepatocellular injury associated with 6 MP toxicity occurring after orthotopic liver transplantation. Cholestasis developed after the introduction of 6 MP. Patients underwent extensive investigation and 6 MP toxicity was considered only after... (truncated)

Title: [Thiopurine-induced hyperammonaemic encephalopathy in a patient with Crohn's disease].
Thiopurine drugs, azathioprine (Imuran) and 6-mercaptopurine (6-MP), are immunomodulators that have been shown to be effective at inducing and maintaining remission in inflammatory bowel disease. Although usually well-tolerated, the occurrence of side effects, typically myelotoxicity and hepatotoxicity, is a major drawback. The side effects can be classified as dose-dependent and independent. Both cholestatic hepatitis and endothelial injury, leading to vascular congestion and nodular regenerative hyperplasia, have been described during therapy with thiopurines, which can end up with portal hypertension. These injuries are potentially mediated by different metabolites. In this article we present a case of hyperammonaemic encephalopathy during therapy with... (truncated)

MESH:D000077592 - maraviroc

Summary:

LLM Prediction Score	0.005 (normalized)
LLM Confidence Score	0.990
Golden Answer (Severity Class)	0.375 (normalized)
Prediction Error	0.370

Retrieved Context:

Title: Common adverse effects of antiretroviral therapy for HIV disease.
Family physicians are treating patients infected with human immunodeficiency virus in their practices more often. Long-term complications of this disease are multifactorial and can be related to the virus itself or to adverse effects of antiretroviral therapy. Each drug class has side effects: nucleoside/nucleotide reverse transcriptase inhibitors are associated with lactic acidosis, lipodystrophy, and hyperlipidemia; non-nucleoside reverse transcriptase inhibitors are associated with neuropsychiatric symptoms, rash, liver toxicity, and lipid abnormalities; and protease inhibitors are associated with gastrointestinal intolerance and glucose and lipid abnormalities. The entry inhibitor maraviroc and the integrase inhibitor raltegravir have been approved for treatment-naive and treatment-experienced patients.... (truncated)

Title: [Secondary effects of treatment with maraviroc and other CCR5 antagonists. Potential impact of the CCR5 blocker].
Maraviroc is the first inhibitor of CCR5 co-receptors to be marketed as an antiretroviral. The pre-clinical studies and phase III trials have shown that it has a very favourable safety profile. No characteristic adverse effect of maraviroc has been identified. Unlike with aplaviroc, where its clinical development was stopped due to serious hepatoxicity, no increase in liver toxicity has been demonstrated in patients treated with maraviroc even if they are co-infected by hepatotropic virus. Nor was there any evidence of an increase in the incidence of neoplasms or serious infections in patients treated with maraviroc. In a study on naive... (truncated)

Title: Maraviroc: new drug. Multiple antiretroviral treatment failure: too soon to reach conclusions.
(1) The choice of treatment for HIV-infected patients in whom several lines of antiretroviral therapy have failed is particularly difficult. Some antiretroviral drugs (enfuvirtide and some HIV protease inhibitors) remain effective, at least in the short term. (2) Maraviroc is the first CCR5 antagonist to be licensed for use in this setting. It acts by blocking one of the two coreceptors, CCR5, needed for HIV entry into CD4+ lymphocytes. It is approved for use in HIV-infected patients with multiple antiretroviral failure. (3) Two double-blind placebo-controlled trials including 1076 patients infected by HIV strains using only the CCR5 coreceptor tested the... (truncated)

Title: Maraviroc, a CCR5 antagonist, prevents development of hepatocellular carcinoma in a mouse model.
Chronic liver disease may result in a sequential progression through fibrosis, cirrhosis and lead, eventually, to hepatocellular carcinoma (HCC). Hepatic stellate cells (HSC) seem to be responsible for the fibrogenic response through the activation of an autocrine loop involving the chemokine receptor, CCR5. However, the role of CCR5 in HCC remains poorly understood. Since this receptor is also one of the main ports of entry for the human immunodeficiency virus (HIV), several CCR5 inhibitors are being used in the clinic to reduce viral load. We used one of these inhibitors, maraviroc (MVC), in a mouse model of diet-induced HCC to... (truncated)

Title: Maraviroc: a review of its use in HIV infection and beyond.
The human immunodeficiency virus-1 (HIV-1) enters target cells by binding its envelope glycoprotein gp120 to the CD4 receptor and/or coreceptors such as C-C chemokine receptor type 5 (CCR5; R5) and C-X-C chemokine receptor type 4 (CXCR4; X4), and R5-tropic viruses predominate during the early stages of infection. CCR5 antagonists bind to CCR5 to prevent viral entry. Maraviroc (MVC) is the only CCR5 antagonist currently approved by the United States Food and Drug Administration, the European Commission, Health Canada, and several other countries for the treatment of patients infected with R5-tropic HIV-1. MVC has been shown to be effective at inhibiting... (truncated)

MESH:D016316 - guanfacine

Summary:

LLM Prediction Score	0.006 (normalized)
LLM Confidence Score	0.990
Golden Answer (Severity Class)	0.375 (normalized)
Prediction Error	0.369

Retrieved Context:

Title: A Retrospective Analysis of Guanfacine for the Pharmacological Management of Delirium.
Background Delirium is a syndrome of acute brain failure that represents a change from an individual's baseline cognitive functioning characterized by deficits in attention and multiple aspects of cognition that fluctuate in severity over time. The symptomatic management of delirium's behavioral manifestations remains difficult. The alpha-2 agonists, dexmedetomidine and clonidine, are efficacious, but their potential cardiovascular adverse effects limit their utilization. Guanfacine is an oral alpha-2 agonist with a lower potential for such adverse outcomes; however, its use in delirium has not been studied. Methods A retrospective descriptive analysis of guanfacine for managing hyperactive or mixed delirium at Tampa General... (truncated)

Title: Consideration of sex and gender differences in addiction medication response.
Substance use continues to contribute to significant morbidity and mortality in the United States, for both women and men, more so than another other preventable health condition. To reduce the public health burden attributable to substances, the National Institute on Drug Abuse and the National Institute on Alcohol Abuse and Alcoholism have identified that medication development for substance use disorder is a high priority research area. Furthermore, both Institutes have stated that research on sex and gender differences in substance use medication development is a critical area. The purpose of the current narrative review is to highlight how sex and... (truncated)

Title: Medications for alcohol use disorders: An overview.
Patients who suffer from alcohol use disorders (AUDs) usually go through various socio-behavioral and pathophysiological changes that take place in the brain and other organs. Recently, consumption of unhealthy food and excess alcohol along with a sedentary lifestyle has become a norm in both developed and developing countries. Despite the beneficial effects of moderate alcohol consumption, chronic and/or excessive alcohol intake is reported to negatively affect the brain, liver and other organs, resulting in cell death, organ damage/failure and death. The most effective therapy for alcoholism and alcohol related comorbidities is alcohol abstinence, however, chronic alcoholic patients cannot stop drinking... (truncated)

Title: Benzodiazepines I: Upping the Care on Downers: The Evidence of Risks, Benefits and Alternatives.
Benzodiazepines are some of the most commonly prescribed medications in the world. These sedative-hypnotics can provide rapid relief for symptoms like anxiety and insomnia, but are also linked to a variety of adverse effects (whether used long-term, short-term, or as needed). Many patients take benzodiazepines long-term without ever receiving evidence-based first-line treatments (e.g., psychotherapy, relaxation techniques, sleep hygiene education, serotonergic agents). This review discusses the risks and benefits of, and alternatives to benzodiazepines. We discuss evidence-based indications and contraindications, and the theoretical biopsychosocial bases for effectiveness, ineffectiveness and harm. Potential adverse effects and drug-drug interactions are summarized. Finally, both fast-acting/acute... (truncated)

Title: Clinical predictors of adverse cardiovascular events for acute pediatric drug exposures.
Context: Risk factors for adverse cardiovascular events (ACVE) from drug exposures have been well-characterized in adults but not studied in children. The objective of the present study is to describe the incidence, characteristics, and risk factors for in-hospital ACVEs among pediatric emergency department (ED) patients with acute drug exposures.Methods: This is a prospective cohort design evaluating patients in the Toxicology Investigators Consortium (ToxIC) Registry. Pediatric patients (age <18 years) who were evaluated at the bedside by a medical toxicologist for a suspected acute drug exposure were included. The primary outcome was in-hospital ACVE (myocardial injury, shock, ventricular dysrhythmia, or cardiac... (truncated)

MESH:D002744 - chlorpheniramine

Summary:

LLM Prediction Score	0.368 (normalized)
LLM Confidence Score	0.980
Golden Answer (Severity Class)	0.0 (normalized)
Prediction Error	0.368

Retrieved Context:

Title: Focus on Over-the-Counter Drugs' Misuse: A Systematic Review on Antihistamines, Cough Medicines, and Decongestants.
Background: Over the past 20 years or so, the drug misuse scenario has seen the emergence of both prescription-only and over-the-counter (OTC) medications being reported as ingested for recreational purposes. OTC drugs such as antihistamines, cough/cold medications, and decongestants are reportedly the most popular in being diverted and misused. Objective: While the current related knowledge is limited, the aim here was to examine the published clinical data on OTC misuse, focusing on antihistamines (e.g., diphenhydramine, promethazine, chlorpheniramine, and dimenhydrinate), dextromethorphan (DXM)- and codeine-based cough medicines, and the nasal decongestant pseudoephedrine. Methods: A systematic literature review was carried out with the... (truncated)

Title: [Stevens-Johnson syndrome plus intrahepatic cholestasis caused by clindamycin or chlorpheniramine].
A 48-year-old woman was hospitalized with the diagnosis of hepatitis. She presented with symptoms of jaundice, headache, elevated bilirubin, and elevated hepatic enzymes. She related a recent episode of a bronchial infection that was treated during the previous eight days with paracetamol (500mg, 2 doses only), chlorpheniramine, betamethasone and clindamycin. After an initial clinical and laboratorial improvement, she began to complain of pruritus of the palms and soles. Thereafter, vesicles evolving to blisters developed and a deterioration of her general health ensued. Serologies for hepatitis A, B, and C viruses were negative. Intrahepatic cholestasis and Stevens Johnson Syndrome (SJS) were... (truncated)

Title: Hepatotoxicity induced by methimazole in a previously healthy patient.
We report a case of hepatotoxicity induced by methimazole treatment in a patient affected by hyperthyroidism. A 54-year-old man, presented to our observation for palpitations, excessive sweating, weakness, heat intolerance and weight loss. On physical examination, his blood pressure was 140/90 mmHg and heart beat was 100/min regular. He had mild tremors and left exophthalmos. Laboratory test revealed a significant increase in serum thyroid hormone levels with a decrease in thyroid stimulating hormone levels. A diagnosis of hyperthyroidism was made and he began treatment with methimazole (30 mg/day). Fourteen days later, he returned for the development of scleral icterus, followed... (truncated)

Title: Pancreatitis and Acute Liver Failure From Coricidin® HBP Intoxication.
The use of over-the-counter medications as recreational drugs of abuse in adolescents is increasing. We present the case of a patient who presented with abdominal pain after the ingestion of Coricidin®, an over-the-counter cold medication that contains acetaminophen, chlorpheniramine maleate, and dextromethorphan hydrobromide. The case was complicated by acute liver failure and concomitant pancreatitis that, in a few reported cases, has been associated with high doses of acetaminophen.

Title: Potential deleterious effects of paracetamol dose regime used in Nigeria versus that of the United States of America.
Paracetamol, also known as acetaminophen (N-acetyl-para-aminophenol, APAP) is the world's most used over-the-counter analgesic-antipyretic drug. Despite its good safety profile, acetaminophen can cause severe hepatotoxicity in overdose, and poisoning from paracetamol has become a major public health concern. Paracetamol is now the major cause of acute liver failure in the United States and Europe. This systematic review aims at examining the likelihood of paracetamol use in Nigeria causing more liver toxicity vis-à-vis the reduced maximum recommended daily adult dose of 3 g for the 500 mg tablet. Online searches were conducted in the databases of PubMed, Google Scholar and MEDLINE... (truncated)

MESH:C446481 - aliskiren

Summary:

LLM Prediction Score	0.367 (normalized)
LLM Confidence Score	0.950
Golden Answer (Severity Class)	0.0 (normalized)
Prediction Error	0.367

Retrieved Context:

Title: Insight into the hepatoprotective, hypolipidemic, and antidiabetic impacts of aliskiren in streptozotocin-induced diabetic liver disease in mice.
Diabetic hepatopathy is a serious complication of poorly controlled diabetes mellitus. An efficient antidiabetic drug which keeps normal liver tissues is not available. The renin-angiotensin system has been reported to be involved in both diabetic state and liver function. Aliskiren is a direct renin inhibitor and a recently antihypertensive drug with poly-pharmacological properties. The aim of the current study is to explore the possible hepatoprotective effects and mechanisms of action of aliskiren against streptozotocin (STZ) induced liver toxicity.

Title: Food-drug interactions precipitated by fruit juices other than grapefruit juice: An update review.
This review addressed drug interactions precipitated by fruit juices other than grapefruit juice based on randomized controlled trials (RCTs). Literature was identified by searching PubMed, Cochrane Library, Scopus and Web of Science till December 30 2017. Among 46 finally included RCTs, six RCTs simply addressed pharmacodynamic interactions and 33 RCTs studied pharmacokinetic interactions, whereas seven RCTs investigated both pharmacokinetic and pharmacodynamic interactions. Twenty-two juice-drug combinations showed potential clinical relevance. The beneficial combinations included orange juice-ferrous fumarate, lemon juice-^{99m}Tc-tetrofosmin, pomegranate juice-intravenous iron during hemodialysis, cranberry juice-triple therapy medications for H. pylori, blueberry juice-etanercept, lime juice-antimalarials, and wheat grass juice-chemotherapy. The potential... (truncated)

Title: Aliskiren Reduces Hepatic steatosis and Epididymal Fat Mass and Increases Skeletal Muscle Insulin Sensitivity in High-Fat Diet-Fed Mice.
Aliskiren has been found to reduce chronic injury and steatosis in the liver of methionine-choline-deficient (MCD) diet-fed mice. This study investigated whether aliskiren has an anti-steatotic effect in HFD-fed mice, which are more relevant to human patients with non-alcoholic fatty liver disease than MCD mice. Mice fed with 4-week normal chow or HFD randomly received aliskiren (50 mg/kg/day) or vehicle via osmotic minipumps for further 4 weeks. Aliskiren reduced systemic insulin resistance, hepatic steatosis, epididymal fat mass and increased gastrocnemius muscle glucose transporter type 4 levels with lower tissue angiotensin II levels in the HFD-fed mice. In addition, aliskiren lowered... (truncated)

Title: Aliskiren effect on non-alcoholic steatohepatitis in metabolic syndrome.
Non-alcoholic steatohepatitis (NASH) is highly associated with metabolic syndrome, a major cause of morbidity in the globalized society. The renin-angiotensin system (RAS) influences hepatic fatty acid metabolism, inflammation and fibrosis. Thus, in the present study, we aimed to evaluate the effect of aliskiren, a direct renin inhibitor, on metabolic syndrome-related NASH.

Title: Probable drug-induced liver injury associated with aliskiren: case report and review of adverse event reports from pharmacovigilance databases.
A case of probable drug-induced liver injury (DILI) attributed to use of the antihypertensive agent aliskiren is reported.

MESH:C481642 - eculizumab

Summary:

LLM Prediction Score	0.367 (normalized)
LLM Confidence Score	0.960
Golden Answer (Severity Class)	0.0 (normalized)
Prediction Error	0.367

Retrieved Context:

Title: Hepatotoxicity associated with eculizumab in a patient with atypical hemolytic uremic syndrome.
No abstract available.

Title: [Acute HCV-induced hepatitis in a patient affected by atypical hemolytic uremic syndrome (aHUS) treated with Eculizumab - case report].
Atypical hemolytic uremic syndrome (aHUS) is a rare and heterogenous disease caused by a disregulation of the alternative pathway of the complement cascade. Specifically, microvascular damage is produced that can lead to acute kidney disease, hemolytic anemia and thrombocytopenia. It accounts for 10% of all hemolytic uremic syndromes and can result in death or in end stage renal disease since the first episode. We can differentiate two forms of aHUS: a sporadic form (80%), affecting adult people, and a familial form (20%) that usually became manifest during infancy. In the acute phase of the disease, frequent and severe anemia requires... (truncated)

Title: Eculizumab Treatment for Postpartum HELLP Syndrome and aHUS-Case Report.
Preeclampsia is a pregnancy-specific disorder affecting ca 3% of all pregnant women. Preeclampsia is the source of severe pregnancy complications. Later life consequences for mother and infant include increased risk of cardiovascular disease. Preeclampsia is caused by the dysfunction of the endothelium with subsequent activation of complement and coagulation systems. HELLP syndrome is considered to be an extreme complication of preeclampsia but it can also present independently. Diagnostic symptoms in HELLP syndrome are Hemolysis, Elevated Liver enzymes, and Low Platelets. Similar phenotype is present in thrombotic microangiopathies (TMAs) and HELLP syndrome is considered part of the TMA spectrum. Here, we... (truncated)

Title: Eculizumab hepatotoxicity in pediatric aHUS.
Eculizumab is a humanized anti-C5 antibody approved for the treatment of atypical hemolytic uremic syndrome (aHUS). Its use is increasing in children following reports of its safety and efficacy.

Title: C3 inhibition with pegcetacoplan in subjects with paroxysmal nocturnal hemoglobinuria treated with eculizumab.
Paroxysmal nocturnal hemoglobinuria (PNH) is an acquired, life-threatening hematologic disease characterized by chronic complement-mediated hemolysis and thrombosis. Despite treatment with eculizumab, a C5 inhibitor, 72% of individuals remain anemic. Pegcetacoplan (APL-2), a PEGylated C3 inhibitor, has the potential to provide more complete hemolysis control in patients with PNH. This open-label, phase Ib study was designed to assess the safety, tolerability, and pharmacokinetics of pegcetacoplan in subjects with PNH who remained anemic during treatment with eculizumab. Pharmacodynamic endpoints were also assessed as an exploratory objective of this study. Data are presented for six subjects in cohort 4 who received treatment for... (truncated)

MESH:D009248 - nadolol

Summary:

LLM Prediction Score	0.008 (normalized)
LLM Confidence Score	0.950
Golden Answer (Severity Class)	0.375 (normalized)
Prediction Error	0.367

Retrieved Context:

Title: Antiarrhythmic effects of beta-adrenergic blocking agents in benign or potentially lethal ventricular arrhythmias.
Classification of ventricular arrhythmias into those that are benign, potentially lethal and lethal is based on their associated risk for producing sudden cardiac death. This classification system is useful in defining indications for the treatment of ventricular arrhythmias and predicting differential rates of antiarrhythmic drug efficacy and toxicity. Whether the reduction of potentially lethal ventricular arrhythmias will prevent sudden cardiac death remains to be determined. The class II antiarrhythmic agents--the beta-adrenergic blocking drugs--have been shown to reduce sudden cardiac death in postmyocardial infarction patients, but the precise mechanism of their effect has not been defined. beta blockers are efficacious in... (truncated)

Title: Nadolol for lithium tremor in the presence of liver damage.
Lithium-induced tremor classically responds to treatment with propranolol. Since it is metabolized in the liver, propranolol may not be the drug of choice in those patients who have compromised liver function or who are recovering from prior liver diseases. Another nonselective beta-adrenergic blocker, nadolol, has no hepatic biotransformation. We present here the first case report of successful treatment of lithium-induced tremor with nadolol, which was selected because the patient had compromised liver function. The patient's liver function tests remained stable with the therapy.

Title: Current and future pharmacological therapies for managing cirrhosis and its complications.
Due to the restrictions of liver transplantation, complication-guided pharmacological therapy has become the mainstay of long-term management of cirrhosis. This article aims to provide a complete overview of pharmacotherapy options that may be commenced in the outpatient setting which are available for managing cirrhosis and its complications, together with discussion of current controversies and potential future directions. PubMed/Medline/Cochrane Library were electronically searched up to December 2018 to identify studies evaluating safety, efficacy and therapeutic mechanisms of pharmacological agents in cirrhotic adults and animal models of cirrhosis. Non-selective beta-blockers effectively reduce variceal re-bleeding risk in cirrhotic patients with moderate/large varices, but... (truncated)

Title: Risk factors for idiosyncratic drug-induced liver injury.
Idiosyncratic drug-induced liver injury (DILI) is a rare disorder that is not related directly to dosage and little is known about individuals who are at increased risk. There are no suitable preclinical models for the study of idiosyncratic DILI and its pathogenesis is poorly understood. It is likely to arise from complex interactions among genetic, nongenetic host susceptibility, and environmental factors. Nongenetic risk factors include age, sex, and other diseases (eg, chronic liver disease or human immunodeficiency virus infection). Compound-specific risk factors include daily dose, metabolism characteristics, and propensity for drug interactions. Alcohol consumption has been proposed as a risk... (truncated)

Title: Current and investigational drugs in early clinical development for portal hypertension.
The development of portal hypertension leads to a majority of complications associated with chronic liver disease. Therefore, adequate treatment of portal hypertension is crucial in the management of such patients. Current treatment options are limited and consist mainly of medications that decrease the hyperdynamic circulation, such as non-selective beta blockers, and treatment of hypervolemia with diuretics. Despite these options, mortality rates have not improved over the last two decades. Newer, more effective treatment options are necessary to help improve survival and quality of life in these patients.

MESH:C516667 - pazopanib

Summary:

LLM Prediction Score	0.634 (normalized)
LLM Confidence Score	0.970
Golden Answer (Severity Class)	1.0 (normalized)
Prediction Error	0.366

Retrieved Context:

Title: Pazopanib-induced mixed liver injury in a patient with soft-tissue sarcoma, but without the UGT1A1*28 mutation: a case report.
A 72-year-old man who underwent pazopanib therapy for soft-tissue sarcoma in the left leg was referred to our department because of elevated levels of liver enzymes. Laboratory tests showed high alanine aminotransferase, alkaline phosphatase, and total bilirubin levels. He was treated with intravenous methylprednisolone (mPSL) therapy (125 mg/day) followed by oral prednisolone and ursodeoxycholic acid therapy, but his liver enzyme abnormality deteriorated and he presented with jaundice. The intravenous mPSL (250 mg/day) treatment was effective and the abnormal levels of liver enzymes and jaundice were improved. He does not carry the UGT1A1*28 mutant allele. Based on our findings, patients presenting... (truncated)

Title: Pazopanib-Induced Liver Toxicity in Patients With Metastatic Renal Cell Carcinoma: Effect of UGT1A1 Polymorphism on Pazopanib Dose Reduction, Safety, and Patient Outcomes.
Pazopanib can induce liver toxicity in patients with metastatic renal cell carcinoma (mRCC). We assessed the effect of a TA repeat polymorphism in the UGT1A1 (uridine diphosphate glucuronosyltransferase 1A1) gene encoding uridine diphosphate glucuronosyltransferase 1A1 on liver toxicity, dose reductions, and patient outcomes.

Title: A remarkable response to pazopanib, despite recurrent liver toxicity, in a patient with a high grade endometrial stromal sarcoma, a case report.
Pazopanib is an oral tyrosine kinase inhibitor registered for metastatic renal cell carcinoma and soft tissue sarcoma. Liver toxicity is a common side effect for this class of agents. The current opinion is that in case of severe liver toxicity pazopanib should be interrupted and restarted at a lower dose after returning to Common Terminology Criteria for Adverse Events (CTCAE) grade 1. After recurrence of liver toxicity at the lower dose it is advised to permanently stop pazopanib. We describe a patient with an YWHAE-FAM22 translocated endometrial stromal sarcoma with a remarkable response to pazopanib despite recurrent liver toxicity.

Title: Severe tyrosine-kinase inhibitor induced liver injury in metastatic renal cell carcinoma patients: two case reports assessed for causality using the updated RUCAM and review of the literature.
Sunitinib and pazopanib are both oral small molecule multityrosine kinase inhibitors (MTKI) used in the treatment of renal cell carcinoma (RCC). Hepatotoxicity or "liver injury" is the most important adverse effect of pazopanib administration, but little is known about the underlying mechanism. Liver injury may also occur in patients treated with sunitinib, but severe toxicity is extremely rare. Herein we report two new cases of severe liver injury induced by MTKI. Both cases are unique and exceptional. We assessed both cases for drug-induced liver injury (DILI) using the updated score Roussel Uclaf causality assessment method (RUCAM). The literature on potential... (truncated)

Title: Protective effects of taxifolin on pazopanib-induced liver toxicity: an experimental rat model.
Pazopanib is a tyrosine kinase inhibitor that is generally used for the treatment of metastatic renal cell cancer and advanced soft tissue sarcoma. It can cause various degrees of hepatotoxicity. Our study aimed to investigate the effect of taxifolin on pazopanib-induced liver toxicity. A total of 18 rats were divided into three groups: the pazopanib (PP), pazopanib plus taxifolin (TPP), and control (C) group. Taxifolin was administered to the TPP (n=6) group with a dose of 50 mg/kg. Distilled water was orally admnistered to the C (n=6) and PP (n=6) groups as a solvent. Subsequently, pazopanib 200 mg/kg was administered... (truncated)

MESH:C044946 - benazepril

Summary:

LLM Prediction Score	0.135 (normalized)
LLM Confidence Score	0.980
Golden Answer (Severity Class)	0.5 (normalized)
Prediction Error	0.365

Retrieved Context:

Title: The influence of hepatic cirrhosis on the pharmacokinetics of benazepril hydrochloride.
The influence of hepatic disease on the pharmacokinetics of the new ACE inhibitor, benazepril hydrochloride, was evaluated in 12 male patients suffering from liver cirrhosis. The patients received a single oral 20 mg dose. The plasma concentrations and urinary excretion of unchanged benazepril and its active metabolite benazeprilat were determined. Compared with a historical control group of healthy volunteers treated with the same benazepril. HC1 dose, the plasma concentrations of benazepril were doubled in the cirrhotic patients. However, the time to reach maximum concentration (0.5 h) was not affected. The plasma kinetics and the urinary excretion of the metabolite benazeprilat... (truncated)

Title: Safety assessment of a highly bioavailable curcumin-galactomannoside complex (CurQfen) in healthy volunteers, with a special reference to the recent hepatotoxic reports of curcumin supplements: A 90-days prospective study.
Recently, there is a growing concern about the use of curcumin supplements owing to a few reported hepatotoxicity related adverse events among some of the long-term consumers. Even though no clear evidence was elucidated for the suspected toxicity, the addition of adjuvants that inhibits body's essential detoxification pathways, adulteration with synthetic curcumin, and presence of contaminants including heavy metals, chromate, illegal dyes, non-steroidal anti-inflammatory agents, and pyrrole alkaloids were suggested as plausible reasons. Considering these incidences and speculations, there is a need to critically evaluate the safety of curcumin supplements for prolonged intake. The present study is an evaluation of... (truncated)

Title: Effects of Benazepril on Survival of Dogs with Chronic Kidney Disease: A Multicenter, Randomized, Blinded, Placebo-Controlled Clinical Trial.
Chronic kidney disease (CKD) is an important cause of morbidity and mortality in dogs.

Title: Association of CYP1A1 and CYP1B1 inhibition in in vitro assays with drug-induced liver injury.
Drug-induced liver injury (DILI) is one of the major causes for the discontinuation of drug development and withdrawal of drugs from the market. Since it is known that reactive metabolite formation and being substrates or inhibitors of cytochrome P450s (P450s) are associated with DILI, we systematically investigated the association between human P450 inhibition and DILI. The inhibitory activity of 266 DILI-positive drugs (DILI drugs) and 92 DILI-negative drugs (no-DILI drugs), which were selected from Liver Toxicity Knowledge Base (US Food and Drug Administration), against 8 human P450 forms was assessed using recombinant enzymes and luminescent substrates, and the threshold values... (truncated)

Title: TJ-M2010-5, A self-developed MyD88 inhibitor, attenuates liver fibrosis by inhibiting the NF-κB pathway.
Liver fibrosis is the result of most chronic inflammatory liver damage and seriously endangers human health. However, no drugs have been approved to treat this disease. Previous studies showed that the Toll-like receptors (TLRs)/myeloid differentiation factor-88 (MyD88)/nuclear factor-κB (NF-κB) pathway plays a key role in liver fibrosis. TJ-M2010-5 is a self-developed small molecule MyD88 inhibitor, which has been proven to have a good protective effect in a variety of inflammatory disease models. In the present study, to investigate the anti-fibrotic effect of TJ-M2010-5, mice were injected with carbon tetrachloride (CCl₄) in vivo and LX2 cells (a human hepatic stellate cell... (truncated)

MESH:D000077144 - clopidogrel

Summary:

LLM Prediction Score	0.511 (normalized)
LLM Confidence Score	0.990
Golden Answer (Severity Class)	0.875 (normalized)
Prediction Error	0.364

Retrieved Context:

Title: [Clopidogrel- induced hepatotoxicity in hemodialyzed patient: a case report].
Drug-induced liver injury is a frequent cause of acute liver failure. It may cause clinical manifestations ranging from simple alteration of the common liver function tests until more severe manifestations including encephalopathy, coagulopathy, and in many cases progressive multi-organ dysfunction. The condition, therefore, may be associated with higher morbidity and mortality as well as higher consumption of economic resources. In this paper, we present the case of a 71-year-old patient treated with hemodialysis, diabetic, with ischemic cardiopathy and severe peripheral vascular disease. The patient presented a progressive clinical deterioration with the development of ascites, jaundice and significant deterioration of liver... (truncated)

Title: Clopidogrel-induced hepatocellular injury and cholestatic jaundice in an elderly patient: case report and review of the literature.
In patients undergoing percutaneous coronary intervention and in those with acute coronary syndromes, clopidogrel plus aspirin is the first-line antiplatelet therapy for reducing cardiovascular events. Although clopidogrel is generally well tolerated, with rash, indigestion, vomiting, diarrhea, and bleeding being the most common adverse effects, rare but serious complications may occur. We describe a 78-year-old woman who underwent percutaneous coronary intervention with drug-eluting stents; clopidogrel and aspirin were started as antiplatelet therapy. Three weeks later, the patient developed mixed hepatocellular and cholestatic liver injury. Clopidogrel was discontinued, and her liver profile results began to improve. Her diagnostic work-up included screening for... (truncated)

Title: [Cholestasic toxic hepatitis due to clopidogrel in a patient with multiple conditions].
Clopidogrel is a thienopyridine-class antiplatelet drug commonly used in ischemic heart disease,cerebrovascular disease and peripheral artery disease.Liver toxicity due to this drug is very infrequent.We found 16 cases in the literature, and in only two of them liver biopsy was carried out. We report the case of a 78 year old patient with multiple conditions affected by severe toxic cholestatic hepatitis due to clopidogrel and the results of the liver biopsy performed. Hepatitis was resolved after discontinuing the drug.Based on the characteristics of this case and other previously published cases, we review the characteristics of toxic hepatitis due to clopidogrel... (truncated)

Title: Clopidogrel-induced liver damage: A case report and review of the literature.
Liver damage is a rare side effect of clopidogrel. That is reversible in most cases. Considering the widespread use of this medication in cardiovascular diseases, the management of hepatotoxicity requires further meticulous investigation.

Title: Clopidogrel-induced hepatotoxicity and fever.
A 59-year-old woman developed fever and elevated hepatic enzyme levels within days of starting clopidogrel, which had been prescribed in conjunction with a percutaneous coronary intervention. When she discontinued the clopidogrel, her liver enzyme levels returned to baseline and her fever disappeared. These signs and symptoms returned after rechallenge with clopidogrel. Monitoring for fever and elevation of liver enzyme levels in patients taking clopidogrel may be warranted. If a patient has signs of hepatotoxicity with or without fever, discontinuation of clopidogrel should be considered, along with substitution with ticlopidine if clinically warranted.

MESH:D000077291 - terbinafine

Summary:

LLM Prediction Score	0.636 (normalized)
LLM Confidence Score	0.990
Golden Answer (Severity Class)	1.0 (normalized)
Prediction Error	0.364

Retrieved Context:

Title: A curious case of cholestasis: oral terbinafine associated with cholestatic jaundice and subsequent erythema nodosum.
Terbinafine is a commonly prescribed antifungal agent used in the treatment of trichophytic onychomycosis and chronic cutaneous mycosis that are resistant to other treatments. This case report highlights a rarely documented but important adverse hepatic reaction that was caused by the use of oral terbinafine. A woman in her thirties presented with a 3-week history of jaundice, malaise, itching, nausea, decreased appetite, weight loss, dark orange urine and intermittent non-radiating epigastric pain. She had recently finished a 3-week course of oral terbinafine for a fungal nail infection. Liver biopsy findings were consistent with chronic active hepatitis secondary to a drug... (truncated)

Title: Terbinafine-induced hepatic dysfunction.
A 41-year-old man developed severe hepatic dysfunction following a 3.5-week course of terbinafine (250 mg/day). He suffered marked pruritus, jaundice, malaise, anorexia and loin pain. Serum bilirubin rose to a peak value of 718 micromol/l with alkaline phosphatase at 569 U/l, alanine aminotransferase at 90 U/l, aspartate aminotransferase at 63 U/l and a prolonged prothrombin time of 21 s, unresponsive to vitamin K. Transjugular liver biopsy showed canalicular cholestasis consistent with a drug reaction. Symptoms resolved 11 months after drug cessation, with liver function tests returning to normal values after 15 months. This case represents the most severe cholestatic reaction... (truncated)

Title: Terbinafine hepatotoxicity: case report and review of the literature.
We report a patient who developed significant liver dysfunction following therapy with terbinafine. At the end of a 3 1/2-wk course of terbinafine, he developed progressive jaundice and pruritus. His serum bilirubin peaked at 30.9 mg/dl 3 wk after discontinuing terbinafine. A liver biopsy revealed mild to moderate mixed cellular infiltrate in the portal tracts, and hepatocellular and canicular cholestasis. His liver tests normalized 100 days after stopping terbinafine.

Title: Terbinafine-induced prolonged cholestasis with reduction of interlobular bile ducts.
The antifungal drug terbinafine has infrequently been incriminated in the occurrence of acute liver injury. We report a case of prolonged cholestasis that occurred in a 75-year-old woman, following terbinafine administration. Jaundice followed by pruritus appeared after four weeks of therapy and was associated with mixed hepatocellular and cholestatic liver tests abnormalities. Following drug withdrawal, serum bilirubin returned to normal values within three months, but anicteric cholestasis persisted for over six months. A liver biopsy performed after six months showed centrilobular cholestasis, discrete portal fibrosis, and a reduction in the number of interlobular biliary ducts. Terbinafine should be added to... (truncated)

Title: [Hepatitis attributed to the use of terbinafine].
A 71-year-old woman was admitted to our hospital with jaundice after she had been using terbinafine for a few weeks. The liver function tests showed a mixed cholestatic-hepatocellular pattern. A liver biopsy revealed large amounts of intracellular bile pigment. Causes of the liver disorder other than the use of the aforementioned antimycotic drug were excluded. Ten months after cessation of the drug the patient had recovered completely. The Netherlands Inspectorate for Health Care received 20 reports of liver enzyme elevations due to terbinafine in 1991-1994.

MESH:D015080 - mesna

Summary:

LLM Prediction Score	0.012 (normalized)
LLM Confidence Score	0.970
Golden Answer (Severity Class)	0.375 (normalized)
Prediction Error	0.363

Retrieved Context:

Title: Toxicity of high-dose ifosfamide in children.
Ifosfamide has been shown to be an active agent in the treatment of several childhood cancers. However, the optimal dose and method of administration remains to be established. The dose/response relationship of ifosfamide suggests that a maximum tolerable, fractionated dose be given, and to reduce hospitalisation this dose should be given in the shortest possible time. A total of 20 patients aged 1-23 years received 124 courses (mean, 6 courses/patient; range, 1-16); 9 subjects had either relapsed or resistant disease, and all of these had previously received cyclophosphamide. A dose of 3 g/m2 ifosfamide was given for 2 (five patients)... (truncated)

Title: Protective effects of MESNA (2-mercaptoethane sulphonate) against acetaminophen-induced hepatorenal oxidative damage in mice.
Acetaminophen, a widely used analgesic and antipyretic, is known to cause hepatic and renal injury in humans and experimental animals when administered in high doses. It was reported that these toxic effects of acetaminophen are due to oxidative reactions that take place during its metabolism. In this study we aimed to investigate the possible beneficial effect of 2-mercaptoethane sulphonate (MESNA), an antioxidant agent, against acetaminophen toxicity in mice. Balb-c mice were injected i.p. with: vehicle (the control group); a single dose of 150 mg kg(-1) MESNA (MES group); a single dose of 900 mg kg(-1) i.p. acetaminophen (AA4h and AA24h... (truncated)

Title: Acrolein, a highly toxic aldehyde generated under oxidative stress in vivo, aggravates the mouse liver damage after acetaminophen overdose.
Although acetaminophen-induced liver injury in mice has been extensively studied as a model of human acute drug-induced hepatitis, the mechanism of liver injury remains unclear. Liver injury is believed to be initiated by metabolic conversion of acetaminophen to the highly reactive intermediate N-acetyl p-benzoquinoneimine, and is aggravated by subsequent oxidative stress via reactive oxygen species (ROS), including hydrogen peroxide (H2O2) and the hydroxyl radical (•OH). In this study, we found that a highly toxic unsaturated aldehyde acrolein, a byproduct of oxidative stress, has a major role in acetaminophen-induced liver injury. Acetaminophen administration in mice resulted in liver damage and increased... (truncated)

Title: Increased sensitivity of glutathione S-transferase P-null mice to cyclophosphamide-induced urinary bladder toxicity.
Hemorrhagic cystitis and diffuse inflammation of the bladder, common side effects of cyclophosphamide (CY) treatment, have been linked to the generation of acrolein derived from CY metabolism. Metabolic removal of acrolein involves multiple pathways, which include reduction, oxidation, and conjugation with glutathione. Herein, we tested the hypothesis that glutathione S-transferase P (GSTP), the GST isoform that displays high catalytic efficiency with acrolein, protects against CY-induced urotoxicity by detoxifying acrolein. Treatment of wild-type (WT) and mGstP1/P2 null (GSTP-null) mice with CY caused hemorrhagic cystitis, edema, albumin extravasation, and sloughing of bladder epithelium; however, CY-induced bladder ulcerations of the lamina propria were... (truncated)

Title: Evaluation of azathioprine-induced cytotoxicity in an in vitro rat hepatocyte system.
Azathioprine (AZA) is widely used in clinical practice for preventing graft rejection in organ transplantations and various autoimmune and dermatological diseases with documented unpredictable hepatotoxicity. The potential molecular cytotoxic mechanisms of AZA towards isolated rat hepatocytes were investigated in this study using "Accelerated Cytotoxicity Mechanism Screening" techniques. The concentration of AZA required to cause 50% cytotoxicity in 2 hrs at 37°C was found to be 400 μM. A significant increase in AZA-induced cytotoxicity and reactive oxygen species (ROS) formation was observed when glutathione- (GSH-) depleted hepatocytes were used. The addition of N-acetylcysteine decreased cytotoxicity and ROS formation. Xanthine oxidase inhibition... (truncated)

MESH:D013307 - streptomycin

Summary:

LLM Prediction Score	0.362 (normalized)
LLM Confidence Score	0.990
Golden Answer (Severity Class)	0.0 (normalized)
Prediction Error	0.362

Retrieved Context:

Title: Hepatotoxicity to different antituberculosis drug combinations.
Hepatotoxicity to different combinations of anti-tuberculosis drugs containing, Rifampicin (R), Streptomycin (S), Isoniazid (H), Pyrazinamide (Z) and Myambutol (E) is described in 47 patients who completed 6 to 9 months therapy. Seven cases (15%) showed signs of toxicity and in 4 patients (8.5%) the drugs had to be withdrawn. Two patients developed hepatitis, one with jaundice and the other with fever and deranged liver functions, while others 2 developed severe hypersensitivity reactions. Burning palms, difficulty in micturition, itching and giddiness were complained of by one patient each, which settled in due course without recourse to withdrawal of drugs.

Title: Etiological role of brucellosis in autoimmune hepatitis.
To show that brucellosis may trigger autoimmune hepatitis (AIH), in addition to nonspecific liver involvement and toxic hepatitis, due to a class effect of tetracycline family used for treatment. We present a female patient admitted to our hospital due to partially improved fatigue and elevated liver enzymes following doxycycline and streptomycin usage for brucellosis. Brucellosis is endemic in our country, Turkey. It may involve any organ in the body. Liver is frequently involved. Doxycycline used for treatment occasionally may lead to hepatotoxicity. AIH is a necroinflammatory disease of the liver. Certain drugs (e.g. minocycline), toxins, and viruses (hepatitis B, hepatitis... (truncated)

Title: Study on hepatotoxicity and other side-effects of antituberculosis drugs.
A prospective study of different side-effects and toxicity of different antituberculosis drugs was made on 125 cases of pulmonary tuberculosis, divided into 3 groups according to the regime of treatment. Group A consisted of 50 patients, taking streptomycin, ethambutol and isoniazid. Group B of 50 patients received streptomycin plus ethambutol plus isoniazid and rifampicin and 25 patients comprising group C received streptomycin plus isoniazid plus ethambutol and pyrazinamide. The group B showed hepatotoxicity in 30% cases, out of which clinical jaundice with abnormal liver function tests being 26% and rest 4% cases were of anicteric hepatitis, while group A showed... (truncated)

Title: [Adverse effects of antitubercular drugs: epidemiology, mechanisms, and patient management].
Tuberculosis, what ever its localization, is an infectious disease which can be totally cured by combining antitubercular drugs. Current therapeutic regimens with isoniazid, rifampicin, pyrazinamide, ethambutol, and streptomycin have proved successful in treating tuberculosis. However, they are associated to a high rate of adverse effects that can lead to therapeutic failure. Understanding the nature and the severity of these adverse effects allows for their appropriate management. Toxic neuropathy and hepatitis are the most common adverse reactions to isoniazid. Rifampicin is generally well tolerated but some severe immuno-allergic reactions may occur in case of intermittent regimen. Pyrazinamide-induced liver injury is rare... (truncated)

Title: Adverse reactions to short-course regimens containing streptomycin, isoniazid, pyrazinamide and rifampicin in Hong Kong.
Three studies of drug toxicity were made in Chinese adults with pulmonary tuberculosis admitted concurrently to short-course antituberculosis regimens. The first was of streptomycin plus isoniazid plus pyrazinamide given daily (SHZ regimen), three times a week (S3H3Z3 regimen) or twice a week (S2H2Z2 regimen). The second was of pyrazinamide in the SHZ regimen and PAS in the standard daily combination of streptomycin plus isoniazid plus PAS (SPH regimen). The third was of the SHZ regimen and these 3 drugs plus rifampicin daily (SHRZ regimen). In study 1 (174 SHZ, 185 S3H3Z3, 182 S2H2Z2 patients), the incidence of arthralgia was associated... (truncated)

MESH:C527517 - ofatumumab

Summary:

LLM Prediction Score	0.362 (normalized)
LLM Confidence Score	0.940
Golden Answer (Severity Class)	0.0 (normalized)
Prediction Error	0.362

Retrieved Context:

Title: Idelalisib given front-line for treatment of chronic lymphocytic leukemia causes frequent immune-mediated hepatotoxicity.
Idelalisib is a small-molecule inhibitor of PI3Kδ with demonstrated efficacy for the treatment of relapsed/refractory chronic lymphocytic leukemia (CLL). To evaluate idelalisib as front-line therapy, we enrolled 24 subjects in a phase 2 study consisting of 2 months of idelalisib monotherapy followed by 6 months of combination therapy with idelalisib and the anti-CD20 antibody ofatumumab. After a median follow-up period of 14.7 months, hepatotoxicity was found to be a frequent and often severe adverse event. A total of 19 subjects (79%) experienced either grade ≥1 ALT or AST elevation during the study, and 13 subjects (54%) experienced grade ≥3 transaminitis.... (truncated)

Title: The emerging role of ofatumumab in the treatment of chronic lymphocytic leukemia.
The treatment of chronic lymphocytic leukemia (CLL) has evolved over the past decade. Our better understanding of disease biology and risk stratification has allowed delivering more effective therapies. In fact, front-line chemoimmunotherapy has demonstrated improvement in overall survival when compared to chemotherapy in randomized studies. Yet, treatment of relapsed CLL remains challenging and few agents are effective in that setting. Ofatumumab (Ofa) is a humanized monoclonal antibody targeted against CD20 with demonstrable activity in rituximab-resistant CLL cell lines. This agent was recently approved for the treatment of relapsed/refractory CLL patients who have failed fludarabine and alemtuzumab. In this review, we... (truncated)

Title: Clinical utility and patient considerations in the use of ofatumumab in chronic lymphocytic leukemia.
Treatment aim for chronic lymphocytic leukemia has been radically changed over the past years from providing only a palliative approach to reaching disease eradication and improving survival. Ofatumumab is a monoclonal humanized antibody with peculiar in vitro and in vivo properties, at present approved for double fludarabine and alemtuzumab refractory chronic lymphocytic leukemia. Its efficacy in this subset of patients, who typically have an unfavorable prognosis, facilitated its use in different Phase II and III trials. Ofatumumab as single agent or combined with chemotherapeutic or biologic agents, led to sundry results in the setting of both previously treated or untreated... (truncated)

Title: Ofatumumab: a novel monoclonal anti-CD20 antibody.
Ofatumumab, a novel humanized monoclonal anti-CD20 antibody, was recently approved by the FDA for the treatment of fludarabine and alemtuzumab refractory chronic lymphocytic leukemia (CLL). Ofatumumab effectively induces complement-dependent cytotoxicity (CDC) in vitro, and recent studies demonstrated that ofatumumab also effectively mediates antibody-dependent cellular cytotoxicity (ADCC). Pharmacokinetic studies indicated that increased exposure to the antibody correlated with improved clinical outcome in CLL. Thus, pharmacogenomics may be important in identifying which patients are more likely to respond to ofatumumab therapy, although such studies have not yet been performed. Patients with the high-affinity FCGR3a 158 V/V polymorphism may be more likely to... (truncated)

Title: The role of ofatumumab in the treatment of chronic lymphocytic leukemia resistant to previous therapies.
Chronic lymphocytic leukemia (CLL) is an indolent but incurable disease. Despite the improvement of the available therapies, the management of heavily-treated CLL patients represents a challenge for modern practitioners. Ofatumumab is a second-generation, fully human anti-CD20 monoclonal antibody that has shown activity in CLL patients who have failed very effective therapies such as fludarabine, alemtuzumab and rituximab. Potential benefits of ofatumumab include powerful complement-dependent cytotoxicity, less immunogenicity, faster infusions and activity in resistant CLL patients. Recently, the FDA has approved ofatumumab for the treatment of CLL patients who have failed fludarabine and alemtuzumab-based regimens. The aim of this review is... (truncated)

MESH:D007741 - labetalol

Summary:

LLM Prediction Score	0.639 (normalized)
LLM Confidence Score	0.990
Golden Answer (Severity Class)	1.0 (normalized)
Prediction Error	0.361

Retrieved Context:

Title: Drug-induced fulminant hepatic failure in pregnancy.
Liver disease in pregnancy can be classified as predating, co-incidental or unique to pregnancy. Medications are often overlooked as a significant cause of liver disease. We present the case of a 39-year-old patient who presented at 20 weeks with jaundice, elevated liver enzymes, and abnormal liver function progressing eventually to fulminant hepatic failure. The patient was on methyldopa and labetalol from 12 weeks' gestational age. Liver biopsy was consistent with drug-induced liver injury. Both methyldopa and labetalol have been associated with hepatotoxicity including liver failure. This case highlights the importance of including medications as a cause of liver failure in... (truncated)

Title: Nicardipine-induced acute hepatitis in an intensive care unit patient.
Drug-related hepatotoxicity is now the leading cause of acute liver failure in the United States, especially among patients who have no prior liver disease. Nicardipine is the only IV calcium channel blocker available for the short-term treatment of hypertension with a considerably good safety profile. We report a case of nicardipine-induced hepatitis. A patient with history of hypertension was admitted because of right middle cerebral artery infarction. Computed tomography of the brain showed evolving stroke. The patient went for cerebral angiography and stent placement, and during the procedure he had cerebral hemorrhage. He was transferred to neurosurgery. After surgery, he... (truncated)

Title: Labetalol hepatotoxicity.
The Food and Drug Administration has received 11 reports of cases (three fatal) in the United States in which hepatocellular damage was associated with labetalol. The temporal circumstances strongly implicate labetalol; the conditions of nine patients improved after cessation of labetalol therapy, and one patient had a recurrence after therapy was restarted. Follow-up with each reporting physician failed to provide historic or laboratory evidence for other viral, toxic, or drug-induced causes of hepatocellular damage, and the case series did not show the demographic and historic risk factors that would be expected if non-A, non-B hepatitis were the cause. Reports of... (truncated)

Title: Cellular imaging predictions of clinical drug-induced liver injury.
Drug-induced liver injury (DILI) is the most common adverse event causing drug nonapprovals and drug withdrawals. Using drugs as test agents and measuring a panel of cellular phenotypes that are directly linked to key mechanisms of hepatotoxicity, we have developed an in vitro testing strategy that is predictive of many clinical outcomes of DILI. Mitochondrial damage, oxidative stress, and intracellular glutathione, all measured by high content cellular imaging in primary human hepatocyte cultures, are the three most important features contributing to the hepatotoxicity prediction. When applied to over 300 drugs and chemicals including many that caused rare and idiosyncratic liver... (truncated)

Title: Metabolic Activation and Cytotoxicity of Labetalol Hydrochloride Mediated by Sulfotransferases.
Labetalol hydrochloride (LHCl), an α- and β-adrenoreceptor blocker, is widely used for the treatment of hypertension as well as angina pectoris. Previous reports have demonstrated the adverse events during clinical application of LHCl, such as liver injury and acute renal failure. The present study aimed to investigate metabolic activation of LHCl to initiate the elucidation of the mechanisms of its liver toxicity. One glutathione (GSH) conjugate was detected in rat and human primary hepatocytes as well as bile of rats after exposure to LHCl. The GSH conjugate was chemically synthesized and characterized by Q-TOF and ¹H NMR. Pretreatment of 2,6-dichloro-4-nitrophenol... (truncated)

MESH:D018120 - finasteride

Summary:

LLM Prediction Score	0.359 (normalized)
LLM Confidence Score	0.990
Golden Answer (Severity Class)	0.0 (normalized)
Prediction Error	0.359

Retrieved Context:

Title: The Interplay between Finasteride-Induced Androgen Imbalance, Endoplasmic Reticulum Stress, Oxidative Stress, and Liver Disorders in Paternal and Filial Generation.
Finasteride (Fin) causes androgen imbalance by inhibiting the conversion of testosterone (T) to its more active metabolite, dihydrotestosterone (DHT). Androgen receptors (AR) are present (e.g., in hepatocytes), which have well-developed endoplasmic reticulum (ERet). Cellular protein quality control is carried out by ERet in two paths: (i) unfolded protein response (UPR) and/or (ii) endoplasmic reticulum associated degradation (ERAD). ERet under continuous stress can generate changes in the UPR and can direct the cell on the pathway of life or death. It has been demonstrated that genes involved in ERet stress are among the genes controlled by androgens in some tissues. Oxidative... (truncated)

Title: Successful drug development despite adverse preclinical findings part 2: examples.
To illustrate the process of addressing adverse preclinical findings (APFs) as outlined in the first part of this review, a number of cases with unexpected APF in toxicity studies with drug candidates is discussed in this second part. The emphasis is on risk characterization, especially regarding the mode of action (MoA), and risk evaluation regarding relevance for man. While severe APFs such as retinal toxicity may turn out to be of little human relevance, minor findings particularly in early toxicity studies, such as vasculitis, may later pose a real problem. Rodents are imperfect models for endocrine APFs, non-rodents for human... (truncated)

Title: Drug-Induced Liver Injury From Enobosarm (Ostarine), a Selective Androgen Receptor Modulator.
Anabolic steroids are well-known to cause liver injury, which may manifest with jaundice and elevated liver enzymes. Selective androgen receptor modulators (SARMs) have been developed to enhance muscle bulk without the side effects associated with exogenous androgen steroids. We report a case of significant cholestatic liver injury associated with a SARM, ostarine (enobosarm), similar to that associated with anabolic steroids. Liver injury from SARMs has not been reported frequently, and we speculate that this may be seen more often as the consumption of SARMs increases in the athletic market.

Title: Dual-5α-Reductase Inhibition Promotes Hepatic Lipid Accumulation in Man.
5α-Reductase 1 and 2 (SRD5A1, SRD5A2) inactivate cortisol to 5α-dihydrocortisol in addition to their role in the generation of DHT. Dutasteride (dual SRD5A1 and SRD5A2 inhibitor) and finasteride (selective SRD5A2 inhibitor) are commonly prescribed, but their potential metabolic effects have only recently been identified.

Title: Selective Androgen Receptor Modulators: An Emerging Liver Toxin.
Selective androgen receptor modulators (SARMs) are a class of nonsteroidal drugs that are favored over anabolic androgenic steroids (AASs) for their tissue-selectivity and improved side-effect profile. These drugs have been evaluated for treatment of various diseases including muscle-wasting disorders, osteoporosis, and breast cancer. Despite lacking approval for therapeutic use, SARMs are widely used recreationally as performance enhancing drugs by bodybuilders and athletes. In recent years, cases of drug-induced liver injury (DILI) secondary to SARMs have begun to emerge, but little is known regarding their hepatotoxicity. In this review, we provide current knowledge regarding DILI from SARMs. A literature search was... (truncated)

MESH:D004087 - dihydroergotamine

Summary:

LLM Prediction Score	0.357 (normalized)
LLM Confidence Score	0.990
Golden Answer (Severity Class)	0.0 (normalized)
Prediction Error	0.357

Retrieved Context:

Title: A Biologically-Based Computational Approach to Drug Repurposing for Anthrax Infection.
Developing drugs to treat the toxic effects of lethal toxin (LT) and edema toxin (ET) produced by <i>B. anthracis</i> is of global interest<i>.</i> We utilized a computational approach to score 474 drugs/compounds for their ability to reverse the toxic effects of anthrax toxins. For each toxin or drug/compound, we constructed an activity network by using its differentially expressed genes, molecular targets, and protein interactions. Gene expression profiles of drugs were obtained from the Connectivity Map and those of anthrax toxins in human alveolar macrophages were obtained from the Gene Expression Omnibus. Drug rankings were based on the ability of a... (truncated)

Title: Getting to the Heart of the Matter: Migraine, Triptans, DHE, Ditans, CGRP Antibodies, First/Second-Generation Gepants, and Cardiovascular Risk.
The science of migraine pathophysiology has advanced significantly since the 1930's. Imaging techniques, neurochemical analysis, clinical trials, and the clinical experience of providers treating migraine patients have not only sharpened our understanding of the disease, but have also led to the development of novel neural-based targets. Targeted therapies such as calcitonin gene-related peptide (CGRP) antibodies and "Second Generation" CGRP receptor antagonists (Gepants) have not only demonstrated efficacy, but have not resulted in any significant cardiovascular nor other serious adverse events. "First Generation" Gepants were associated with liver toxicity.

Title: New Approaches to Shifting the Migraine Treatment Paradigm.
The standard of care paradigm for migraine treatment has been based almost exclusively on approaches that grew out of the happenstance use of market pharmaceuticals. Only methysergide, which has long since been removed from use for safety concerns, the ergotamine family of drugs, and the triptans were explicitly developed with migraine and other vascular headaches in mind. While the forward and innovative thinking to utilize the broad array of agents to treat migraine served millions well, their therapeutic efficacy was often low, and adverse event profiles were troublesome in the least. Advances in biochemical and molecular biology and the application... (truncated)

Title: Coalescing beneficial host and deleterious antiparasitic actions as an antischistosomal strategy.
Conventional approaches for antiparasitic drug discovery center upon discovering selective agents that adversely impact parasites with minimal host side effects. Here, we show that agents with a broad polypharmacology, often considered 'dirtier' drugs, can have unique efficacy if they combine deleterious effects on the parasite with beneficial actions in the host. This principle is evidenced through a screen for drugs to treat schistosomiasis, a parasitic flatworm disease that impacts over 230 million people. A target-based screen of a <i>Schistosoma</i> serotoninergic G protein coupled receptor yielded the potent agonist, ergotamine, which disrupted worm movement. In vivo, ergotamine decreased mortality, parasite load... (truncated)

Title: Association of CYP1A1 and CYP1B1 inhibition in in vitro assays with drug-induced liver injury.
Drug-induced liver injury (DILI) is one of the major causes for the discontinuation of drug development and withdrawal of drugs from the market. Since it is known that reactive metabolite formation and being substrates or inhibitors of cytochrome P450s (P450s) are associated with DILI, we systematically investigated the association between human P450 inhibition and DILI. The inhibitory activity of 266 DILI-positive drugs (DILI drugs) and 92 DILI-negative drugs (no-DILI drugs), which were selected from Liver Toxicity Knowledge Base (US Food and Drug Administration), against 8 human P450 forms was assessed using recombinant enzymes and luminescent substrates, and the threshold values... (truncated)

MESH:D002328 - carisoprodol

Summary:

LLM Prediction Score	0.357 (normalized)
LLM Confidence Score	0.990
Golden Answer (Severity Class)	0.0 (normalized)
Prediction Error	0.357

Retrieved Context:

Title: The Importance of Patient-Specific Factors for Hepatic Drug Response and Toxicity.
Responses to drugs and pharmacological treatments differ considerably between individuals. Importantly, only 50%-75% of patients have been shown to react adequately to pharmacological interventions, whereas the others experience either a lack of efficacy or suffer from adverse events. The liver is of central importance in the metabolism of most drugs. Because of this exposed status, hepatotoxicity is amongst the most common adverse drug reactions and hepatic liabilities are the most prevalent reason for the termination of development programs of novel drug candidates. In recent years, more and more factors were unveiled that shape hepatic drug responses and thus underlie the... (truncated)

Title: Detection of Acetaminophen-Protein Adducts in Decedents with Suspected Opioid-Acetaminophen Combination Product Overdose.
Acetaminophen overdose is a leading cause of drug-induced liver failure in the United States. Acetaminophen-protein adducts have been suggested as a biomarker of hepatotoxicity. The purpose of this study was to determine whether protein-derived acetaminophen-protein adducts are quantifiable in postmortem samples. Heart blood, femoral blood, and liver tissue were collected at autopsy from 22 decedents suspected of opioid-acetaminophen overdose. Samples were assayed for protein-derived acetaminophen-protein adducts, acetaminophen, and selected opioids found in combination products containing acetaminophen. Protein-derived APAP-CYS was detected in 17 of 22 decedents and was measurable in blood that was not degraded or hemolyzed. Heart blood concentrations ranged... (truncated)

Title: Alcohol and medication interactions.
Many medications can interact with alcohol, thereby altering the metabolism or effects of alcohol and/or the medication. Some of these interactions can occur even at moderate drinking levels and result in adverse health effects for the drinker. Two types of alcohol-medication interactions exist: (1) pharmacokinetic interactions, in which alcohol interferes with the metabolism of the medication, and (2) pharmacodynamic interactions, in which alcohol enhances the effects of the medication, particularly in the central nervous system (e.g., sedation). Pharmacokinetic interactions generally occur in the liver, where both alcohol and many medications are metabolized, frequently by the same enzymes. Numerous classes of... (truncated)

Title: Hepatotoxicity Induced by "the 3Ks": Kava, Kratom and Khat.
The 3Ks (kava, kratom and khat) are herbals that can potentially induce liver injuries. On the one hand, growing controversial data have been reported about the hepatotoxicity of kratom, while, on the other hand, even though kava and khat hepatotoxicity has been investigated, the hepatotoxic effects are still not clear. Chronic recreational use of kratom has been associated with rare instances of acute liver injury. Several studies and case reports have suggested that khat is hepatotoxic, leading to deranged liver enzymes and also histopathological evidence of acute hepatocellular degeneration. Numerous reports of severe hepatotoxicity potentially induced by kava have also... (truncated)

Title: Clinically important alterations in pharmacogene expression in histologically severe nonalcoholic fatty liver disease.
Polypharmacy is common in patients with nonalcoholic fatty liver disease (NAFLD) and previous reports suggest that NAFLD is associated with altered drug disposition. This study aims to determine if patients with NAFLD are at risk for altered drug response by characterizing changes in hepatic mRNA expression of genes mediating drug disposition (pharmacogenes) across the histological NAFLD severity spectrum. We utilize RNA-seq for 93 liver biopsies with histologically staged NAFLD Activity Score (NAS), fibrosis stage, and steatohepatitis (NASH). We identify 37 significant pharmacogene-NAFLD severity associations including CYP2C19 downregulation. We chose to validate CYP2C19 due to its actionability in drug prescribing. Meta-analysis... (truncated)

MESH:D014229 - triazolam

Summary:

LLM Prediction Score	0.144 (normalized)
LLM Confidence Score	0.980
Golden Answer (Severity Class)	0.5 (normalized)
Prediction Error	0.356

Retrieved Context:

Title: Clinical safety and tolerability issues in use of triazole derivatives in management of fungal infections.
There has been an increase in the number of patients susceptible to invasive fungal infections (IFIs) leading to a greater need for effective, well tolerated, and easily administered antifungal agents. The advent of triazoles has revolutionized the care of patients requiring treatment or prophylaxis for IFIs. However, triazoles have been associated with a number of adverse events and significant drug-drug interactions. While commonly used, physicians and patients should be aware of the distinct properties of these agents in order to ensure that patients are optimally treated with the least amount of toxicity possible. Clinicians should have a full understanding of... (truncated)

Title: [Voluntary drug poisoning: epidemiology, performance and limits of the emergency laboratory].
The aim of this study is to determine the efficiency ot toxicologic screening (detection of barbiturates, benzodiazepines, tricyclic antidepressants, salicylates, phenothiazines, meprobamate and ethanol assay), during acute drug poisoning. In 1988, 898 patients are enclose in this study. Screenings are negative in 17% of cases; benzodiazepines, alcohol and antidepressants are often found. The recovery is very good for barbiturates and salicylates but it's not perfect for benzodiazepines, particularly flunitrazepam triazolam, loflazepate, oxazepam, and non tricyclic antidepressants. This failure probably depends on these emergency methods.

Title: Pharmacokinetics and pharmacodynamics of antifungals in children and their clinical implications.
Invasive fungal infections are a significant cause of morbidity and mortality in children. Successful management of these systemic infections requires identification of the causative pathogen, appropriate antifungal selection, and optimisation of its pharmacokinetic and pharmacodynamic properties to maximise its antifungal activity and minimise toxicity and the emergence of resistance. This review highlights salient scientific advancements in paediatric antifungal pharmacotherapies and focuses on pharmacokinetic and pharmacodynamic studies that underpin current clinical decision making. Four classes of drugs are widely used in the treatment of invasive fungal infections in children, including the polyenes, triazoles, pyrimidine analogues and echinocandins. Several lipidic formulations of... (truncated)

Title: Stimulation of Hepatic Apolipoprotein A-I Production by Novel Thieno-Triazolodiazepines: Roles of the Classical Benzodiazepine Receptor, PAF Receptor, and Bromodomain Binding.
Expression and secretion of apolipoprotein A-I (apoA-I) by cultured liver cells can be markedly stimulated by triazolodiazepines (TZDs). It has been shown previously that the thieno-TZD Ro 11-1464 increases plasma levels of apoA-I and in vivomacrophage reverse cholesterol transport in mice. However, these effects were only seen at high doses, at which the compound could act on central benzodiazepine (BZD) receptors or platelet activating factor (PAF) receptors, interfering with its potential utility. In this work, we describe 2 new thieno-TZDs MDCO-3770 and MDCO-3783, both derived from Ro 11-1464. These compounds display the same high efficacy on apoA-I production, metabolic stability,... (truncated)

Title: Novel Pathways of Ponatinib Disposition Catalyzed By CYP1A1 Involving Generation of Potentially Toxic Metabolites.
Ponatinib, a pan-BCR-ABL tyrosine kinase inhibitor for the treatment of chronic myeloid leukemia (CML), causes severe side effects including vascular occlusions, pancreatitis, and liver toxicity, although the underlying mechanisms remain unclear. Modifications of critical proteins through reactive metabolites are thought to be responsible for a number of adverse drug reactions. In vitro metabolite screening of ponatinib with human liver microsomes and glutathione revealed unambiguous signals of ponatinib-glutathione (P-GSH) adducts. Further profiling of human cytochrome P450 (P450) indicated that CYP1A1 was the predominant P450 enzyme driving this reaction. P-GSH conjugate formation paralleled the disappearance of hydroxylated ponatinib metabolites, suggesting the initial... (truncated)

MESH:D014640 - vancomycin

Summary:

LLM Prediction Score	0.355 (normalized)
LLM Confidence Score	0.990
Golden Answer (Severity Class)	0.0 (normalized)
Prediction Error	0.355

Retrieved Context:

Title: Adverse Effects of Intravenous Vancomycin-Based Prophylaxis during Therapy for Pediatric Acute Myeloid Leukemia.
Children and adolescents with acute myeloid leukemia (AML) are at risk of life-threatening bacterial infections, especially with viridans group streptococci. Primary antibacterial prophylaxis with vancomycin-based regimens reduces this risk but might increase the risks of renal or liver toxicity or <i>Clostridium difficile</i> infection (CDI). A retrospective review of data for patients treated for newly diagnosed AML at St. Jude Children's Research Hospital between 2002 and 2008 was conducted. Nephrotoxicity was classified according to pediatric risk, injury, failure, loss, and end-stage renal disease (pRIFLE) criteria and hepatotoxicity according to Common Terminology Criteria for Adverse Events (CTCAE) criteria. The risks of nephrotoxicity,... (truncated)

Title: Influence of Antibiotics on Functionality and Viability of Liver Cells In Vitro.
(1) Antibiotics are an important weapon in the fight against serious bacterial infections and are considered a common cause of drug-induced liver injury (DILI). The hepatotoxicity of many drugs, including antibiotics, is poorly analyzed in human in vitro models. (2) A standardized assay with a human hepatoma cell line was used to test the hepatotoxicity of various concentrations (Cmax, 5× Cmax, and 10× Cmax) of antibiotics. In an ICU, the most frequently prescribed antibiotics, ampicillin, cefepime, cefuroxime, levofloxacin, linezolid, meropenem, rifampicin, tigecycline, and vancomycin, were incubated with HepG2/C3A cells for 6 days. Cell viability (XTT assay, LDH release, and vitality),... (truncated)

Title: A Patient with Nafcillin-Associated Drug-Induced Liver Failure.
Nafcillin-induced acute liver injury is a rare and potentially fatal complication that has been known since the 1960s but inadequately studied. At this time, the only proven treatment is early discontinuation of the drug. Because of the high prevalence of nafcillin class antibiotic use in the United States, it is important for clinicians to have a high clinical suspicion for this diagnosis. We present a case of liver failure attributable to nafcillin use in a 68-year-old male with a history methicillin-sensitive <i>Staphylococcus</i> and L3/L4 osteomyelitis. After starting long-term antibiotic therapy, he presented with painless jaundice which necessitated discontinuation of the... (truncated)

Title: A challenge for diagnosing acute liver injury with concomitant/sequential exposure to multiple drugs: can causality assessment scales be utilized to identify the offending drug?
Drug-induced hepatotoxicity most commonly manifests as an acute hepatitis syndrome and remains the leading cause of drug-induced death/mortality and the primary reason for withdrawal of drugs from the pharmaceutical market. We report a case of acute liver injury in a 12-year-old Hispanic boy, who received a series of five antibiotics (amoxicillin, ceftriaxone, vancomycin, ampicillin/sulbactam, and clindamycin) for cervical lymphadenitis/retropharyngeal cellulitis. Histopathology of the liver biopsy specimen revealed acute cholestatic hepatitis. All known causes of acute liver injury were appropriately excluded and (only) drug-induced liver injury was left as a cause of his cholestasis. Liver-specific causality assessment scales such as Council... (truncated)

Title: [Examination of factors affecting efficacy and adverse effect, for the retrospective study of vancomycin hydrochloride (VCM)].
Vancomycin hydrochloride (VCM) is widely used for treatment of methicillin-resistant Staphylococcus aureus (MRSA) infections. However, this drug can cause sever adverse reactions, such as red neck syndrome, nephrotoxicity and ototoxicity. Thus, therapeutic drug monitoring (TDM) was bringing into effect for well effectiveness and to prevent side effects. In Kanto Medical Center NTT EC, TDM of VCM has been brought into effect since 1994. The date were accumulated from 200 patients. In this study, the retrospective research was carried out based on 117 cases selected from the above accumulated data, and then several factors such as VCM inducing side effect, a... (truncated)

MESH:C054920 - loracarbef

Summary:

LLM Prediction Score	0.022 (normalized)
LLM Confidence Score	0.950
Golden Answer (Severity Class)	0.375 (normalized)
Prediction Error	0.353

Retrieved Context:

Title: Antimicrobial stewardship's new weapon? A review of antibiotic allergy and pathways to 'de-labeling'.
The continued emergence of multiresistant pathogens and widespread antimicrobial use has led to a greater emphasis on antimicrobial stewardship programs. Concurrently, an increased awareness of the rising number of antibiotic allergy labels and impact on antimicrobial use has surfaced. The integration of antibiotic allergy de-labeling and antimicrobial stewardship programs may be a pathway worthy of further focus and investigation.

Title: Emerging Strategies to Combat ESKAPE Pathogens in the Era of Antimicrobial Resistance: A Review.
The acronym ESKAPE includes six nosocomial pathogens that exhibit multidrug resistance and virulence: <i>Enterococcus faecium, Staphylococcus aureus, Klebsiella pneumoniae, Acinetobacter baumannii, Pseudomonas aeruginosa</i>, and <i>Enterobacter</i> spp. Persistent use of antibiotics has provoked the emergence of multidrug resistant (MDR) and extensively drug resistant (XDR) bacteria, which render even the most effective drugs ineffective. Extended spectrum β-lactamase (ESBL) and carbapenemase producing Gram negative bacteria have emerged as an important therapeutic challenge. Development of novel therapeutics to treat drug resistant infections, especially those caused by ESKAPE pathogens is the need of the hour. Alternative therapies such as use of antibiotics in combination or... (truncated)

Title: The evolving role of chemical synthesis in antibacterial drug discovery.
The discovery and implementation of antibiotics in the early twentieth century transformed human health and wellbeing. Chemical synthesis enabled the development of the first antibacterial substances, organoarsenicals and sulfa drugs, but these were soon outshone by a host of more powerful and vastly more complex antibiotics from nature: penicillin, streptomycin, tetracycline, and erythromycin, among others. These primary defences are now significantly less effective as an unavoidable consequence of rapid evolution of resistance within pathogenic bacteria, made worse by widespread misuse of antibiotics. For decades medicinal chemists replenished the arsenal of antibiotics by semisynthetic and to a lesser degree fully synthetic... (truncated)

Title: Antibiotics: Conventional Therapy and Natural Compounds with Antibacterial Activity-A Pharmaco-Toxicological Screening.
Antibiotics are considered as a cornerstone of modern medicine and their discovery offers the resolution to the infectious diseases problem. However, the excessive use of antibiotics worldwide has generated a critical public health issue and the bacterial resistance correlated with antibiotics inefficiency is still unsolved. Finding novel therapeutic approaches to overcome bacterial resistance is imperative, and natural compounds with antibacterial effects could be considered a promising option. The role played by antibiotics in tumorigenesis and their interrelation with the microbiota are still debatable and are far from being elucidated. Thus, the present manuscript offers a global perspective on antibiotics in... (truncated)

Title: Diagnosis and management of immediate hypersensitivity reactions to cephalosporins.
Cephalosporins can cause a range of hypersensitivity reactions, including IgE-mediated, immediate reactions. Cephalosporin allergy has been reported with use of a specific cephalosporin, as a cross-reaction between different cephalosporins or as a cross-reaction to other β-lactam antibiotics. Unlike penicillins, the exact allergenic determinants of cephalosporins are less well understood and thus, standardized diagnostic skin testing is not available. Nevertheless, skin testing with diluted solutions of cephalosporins can be valuable in confirming IgE-mediated hypersensitivity reactions. In vitro tests are in development using recent technological advances and can be used as complementary tests. However, they are not commonly used because of their... (truncated)

MESH:D003973 - diatrizoate

Summary:

LLM Prediction Score	0.348 (normalized)
LLM Confidence Score	0.920
Golden Answer (Severity Class)	0.0 (normalized)
Prediction Error	0.348

Retrieved Context:

Title: Atypia of hepatic histiocytes induced by Renografin-60.
Recurrent bouts of abdominal pain in a 12-year-old patient were diagnosed as due to intermittent obstruction of the choledochus. The condition was corrected by choledochostomy. Incidental liver biopsy showed atypia of intrahepatic histiocytes. However, electron microscopy identified associated foreign bodies suspected of being an iodinated compound. Six months later a repeat liver biopsy and extensive search showed no carcinoma present. The hepatic atypia was thought to have been induced by diatrizoate meglumine/diatrizoate sodium (Renografin-60), an iodinated compound (E. R. Squibb, Princeton, NJ, U.S.A.) used during percutaneous transhepatic cholangiography.

Title: The effectiveness and biosafety of diatrizoate contrast media in complicated cholelithiasis.
Adverse reactions after intravascular administration of iodine-based contrast media are well-known. Nevertheless the same type of contrast media is also used for endoscopic retrograde cholangiopancreatography and systemic absorption of contrast media after mentioned procedure routinely occurs, not much is known about effects of widely used diatrizoates (Triombrast) on the hepato-pancreato-biliary system in case of cholelithiasis treatment.

Title: CT densities in delayed iodine hepatic scanning.
Sixty patients underwent CT scanning of the liver prior to, immediately after, and four hours after intravenous administration of 60% meglumine diatrizoate. Twenty patients received a 50 ml bolus of contrast material (14.6 g of iodine), 20 received 100 ml (29.2 g of iodine), and 20 received 200 ml (58.4 g of iodine). In each group, delayed CT scanning safely raised the inherent density of the liver significantly (increase of 14.3% using 50 ml; 23.9% using 100 ml; and 40.7% using 200 ml). Thus, delayed scanning with doses presently used in abdominal and neurological CT examinations may be helpful in... (truncated)

Title: Discovery of a 2'-Fluoro,2'-Bromouridine Phosphoramidate Prodrug Exhibiting Anti-Yellow Fever Virus Activity in Culture and in Mice.
Yellow fever virus (YFV) is a potentially lethal, zoonotic, blood-borne flavivirus transmitted to humans and non-human primates by mosquitoes. Owing to multiple deadly epidemics, the WHO classifies YFV as a "high impact, high threat disease" with resurgent epidemic potential. At present, there are no approved antiviral therapies to combat YFV infection. Herein we report on 2'-halogen-modified nucleoside analogs as potential anti-YFV agents. Of 11 compounds evaluated, three showed great promise with low toxicity, high intracellular metabolism into the active nucleoside triphosphate form, and sub-micromolar anti-YFV activity. Notably, we investigated a 2'-fluoro,2'-bromouridine phosphate prodrug (C9), a known anti-HCV agent with good... (truncated)

Title: Gilbert syndrome combined with prolonged jaundice caused by contrast agent: Case report.
This case highlights a patient with Gilbert syndrome who underwent endoscopic retrograde cholangiopancreatography (ERCP) with removal of bile duct stones, who then experienced an unexplained increase in bilirubin, with total bilirubin (TBIL) levels increasing from 159.5 μmol/L to 396.2 μmol/L and to a maximum of 502.8 μmol/L after 9 d. Following the decrease in the TBIL level, enhanced magnetic resonance cholangiopancreatography (MRCP) was performed to exclude any possible remaining choledocholithiasis. Nevertheless, the serum bilirubin level increased again, with TBIL levels rising from 455.7 μmol/L to 594.8 μmol/L and a maximum level of 660.3 μmol/L with no remaining bile duct stones.... (truncated)

MESH:C046649 - ropinirole

Summary:

LLM Prediction Score	0.153 (normalized)
LLM Confidence Score	0.980
Golden Answer (Severity Class)	0.5 (normalized)
Prediction Error	0.347

Retrieved Context:

Title: Association of CYP1A1 and CYP1B1 inhibition in in vitro assays with drug-induced liver injury.
Drug-induced liver injury (DILI) is one of the major causes for the discontinuation of drug development and withdrawal of drugs from the market. Since it is known that reactive metabolite formation and being substrates or inhibitors of cytochrome P450s (P450s) are associated with DILI, we systematically investigated the association between human P450 inhibition and DILI. The inhibitory activity of 266 DILI-positive drugs (DILI drugs) and 92 DILI-negative drugs (no-DILI drugs), which were selected from Liver Toxicity Knowledge Base (US Food and Drug Administration), against 8 human P450 forms was assessed using recombinant enzymes and luminescent substrates, and the threshold values... (truncated)

Title: Recent updates on structural insights of MAO-B inhibitors: a review on target-based approach.
Parkinson's disease is a neurodegenerative disorder characterized by slow movement, tremors, and stiffness caused due to loss of dopaminergic neurons caused in the brain's substantia nigra. The concentration of dopamine is decreased in the brain. Parkinson's disease may be happened because of various genetic and environmental factors. Parkinson's disease is related to the irregular expression of the monoamine oxidase (MAO) enzyme, precisely type B, which causes the oxidative deamination of biogenic amines such as dopamine. MAO-B inhibitors, available currently in the market, carry various adverse effects such as dizziness, nausea, vomiting, lightheadedness, fainting, etc. So, there is an urgent need... (truncated)

Title: A Patient With CKD Develops Cholestatic Liver Injury During a Clinical Trial.
No abstract available.

Title: Human hepatic in vitro models reveal distinct anti-NASH potencies of PPAR agonists.
Non-alcoholic steatohepatitis (NASH) is a highly prevalent, chronic liver disease characterized by hepatic lipid accumulation, inflammation, and concomitant fibrosis. Up to date, no anti-NASH drugs have been approved. In this study, we reproduced key NASH characteristics in vitro by exposing primary human hepatocytes (PHH), human skin stem cell-derived hepatic cells (hSKP-HPC), HepaRG and HepG2 cell lines, as well as LX-2 cells to multiple factors that play a role in the onset of NASH. The obtained in vitro disease models showed intracellular lipid accumulation, secretion of inflammatory chemokines, induced ATP content, apoptosis, and increased pro-fibrotic gene expression. These cell systems were... (truncated)

Title: Quantitative NTCP pharmacophore and lack of association between DILI and NTCP Inhibition.
The human sodium taurocholate cotransporting polypeptide (NTCP) is a hepatic bile acid transporter. Inhibition of NTCP uptake may potentially also prevent hepatitis B virus (HBV) infection. The first objective was to develop a quantitative pharmacophore for NTCP inhibition. Recent studies showed that hepatotoxic drugs could inhibit bile acid uptake into hepatocytes, without inhibiting canalicular efflux, and cause bile acid elevation in plasma. Hence, a second objective was to examine whether NTCP inhibition is associated with drug induced liver injury (DILI). Twenty-seven drugs from our previous study were used as the training set to develop a quantitative pharmacophore. From secondary screening... (truncated)

MESH:C052035 - trandolapril

Summary:

LLM Prediction Score	0.154 (normalized)
LLM Confidence Score	0.950
Golden Answer (Severity Class)	0.5 (normalized)
Prediction Error	0.346

Retrieved Context:

Title: Association of CYP1A1 and CYP1B1 inhibition in in vitro assays with drug-induced liver injury.
Drug-induced liver injury (DILI) is one of the major causes for the discontinuation of drug development and withdrawal of drugs from the market. Since it is known that reactive metabolite formation and being substrates or inhibitors of cytochrome P450s (P450s) are associated with DILI, we systematically investigated the association between human P450 inhibition and DILI. The inhibitory activity of 266 DILI-positive drugs (DILI drugs) and 92 DILI-negative drugs (no-DILI drugs), which were selected from Liver Toxicity Knowledge Base (US Food and Drug Administration), against 8 human P450 forms was assessed using recombinant enzymes and luminescent substrates, and the threshold values... (truncated)

Title: Compromised glutathione synthesis results in high susceptibility to acetaminophen hepatotoxicity in acatalasemic mice.
Acatalasemia is caused by genetic defect in the catalase gene. Human achatalasemia patients are able to scavenge physiological hydrogen peroxide but are vulnerable to exogenous oxidative stress. In the present study, we used an acetaminophen-induced hepatotoxicity model in acatalasemic mice to explore this vulnerability. Interestingly, the acetaminophen-induced decrease in total glutathione levels was more prolonged in acatalasemic mice. While the subunits of glutamate-cysteine ligase, a glutathione synthase enzyme, were increased by acetaminophen in the liver of wild-type mice, their expression was lower and was further reduced by acetaminophen in acatalasemic mice. This feature was also observed in immortalized hepatocytes derived... (truncated)

Title: From congestive hepatopathy to hepatocellular carcinoma, how can we improve patient management?
Heart failure and liver disease often coexist because of systemic disorders and diseases that affect both organs as well as complex cardio-hepatic interactions. Heart failure can cause acute or chronic liver injury due to ischaemia and passive venous congestion, respectively. Congestive hepatopathy is frequently observed in patients with congenital heart disease and after the Fontan procedure, but also in older patients with chronic heart failure. As congestive hepatopathy can evolve into cirrhosis and hepatocellular carcinoma, screening for liver injury should be performed in patients with chronic cardiac diseases and after Fontan surgery. Fibrosis starts in the centro-lobular zone and will... (truncated)

Title: Update on New Aspects of the Renin-Angiotensin System in Hepatic Fibrosis and Portal Hypertension: Implications for Novel Therapeutic Options.
There is considerable experimental evidence that the renin angiotensin system (RAS) plays a central role in both hepatic fibrogenesis and portal hypertension. Angiotensin converting enzyme (ACE), a key enzyme of the classical RAS, converts angiotensin I (Ang I) to angiotensin II (Ang II), which acts via the Ang II type 1 receptor (AT1R) to stimulate hepatic fibrosis and increase intrahepatic vascular tone and portal pressure. Inhibitors of the classical RAS, drugs which are widely used in clinical practice in patients with hypertension, have been shown to inhibit liver fibrosis in animal models but their efficacy in human liver disease is... (truncated)

Title: The effect of angiotensin-blocking agents on liver fibrosis in patients with hepatitis C.
Multiple studies implicate the renin-angiotensin system in hepatic fibrogenesis. Few studies have examined the effects of angiotensin blockade on liver fibrosis via human histology.

MESH:D019829 - nevirapine

Summary:

LLM Prediction Score	0.654 (normalized)
LLM Confidence Score	0.990
Golden Answer (Severity Class)	1.0 (normalized)
Prediction Error	0.346

Retrieved Context:

Title: Jaundice and hepatocellular damage associated with nevirapine therapy.
Nevirapine is a nonnucleoside reverse transcription inhibitor that is used as part of highly active antiretroviral therapeutic combinations. Nevirapine has been associated with a skin rash in 32 to 48% of patients. Recent reports indicate that hepatic toxicity also occurs.

Title: Liver toxicity caused by nevirapine.
Nevirapine plasma levels were measured in 70 HIV-infected patients, 33 of whom developed transaminase elevations. Higher nevirapine levels and hepatitis C virus infection were found to be independent predictors of liver toxicity. Moreover, in individuals with chronic hepatitis C, nevirapine concentrations greater than 6 microg/ml were associated with a 92% risk of liver toxicity. Therefore, monitoring nevirapine levels, especially in individuals with chronic hepatitis C, may be warranted.

Title: Liver toxicity induced by non-nucleoside reverse transcriptase inhibitors.
Liver toxicity is one of the most relevant adverse effects of antiretroviral therapy. Within the non-nucleoside reverse transcriptase inhibitors (NNRTIs), efavirenz can be considered a safer drug for the liver than nevirapine. In fact, the frequency of severe increased liver enzymes in patients on efavirenz ranges from 1 to 8%, whereas in patients treated with nevirapine, it ranges from 4 to 18%. Likewise, nevirapine is more commonly associated than efavirenz with early acute hepatitis, which is produced by a hypersensitivity mechanism and has a defined risk profile that often makes it avoidable. Despite the fact that most cases of NNRTI-induced... (truncated)

Title: RAT CYP3A and CYP2B1/2 were not associated with nevirapine-induced hepatotoxicity.
Nevirapine is an antiretroviral drug that is used for treatment as well as for the prevention of mother-to-child transmission of the human immunodeficiency virus (HIV). Unfortunately, its adverse effects, mainly hypersensitivity skin reactions and hepatotoxicity, have hampered the use of nevirapine. Since nevirapine-induced hepatotoxicity commonly occurs between 2-12 weeks of treatment, and nevirapine is a known inducer of human CYP3A and CYP2B6 isozymes, it was envisaged that the hepatotoxicity was due to activation of nevirapine to toxic metabolites by the induced enzymes. Therefore, the aim of this study was to use a rat model and determine the role of the... (truncated)

Title: Incidence of liver injury after beginning antiretroviral therapy with efavirenz or nevirapine.
To compare the incidence and define the risk factors associated with liver toxicity in patients beginning treatment with nevirapine (NVP) and efavirenz (EFZ).

MESH:D019469 - indinavir

Summary:

LLM Prediction Score	0.655 (normalized)
LLM Confidence Score	0.970
Golden Answer (Severity Class)	1.0 (normalized)
Prediction Error	0.345

Retrieved Context:

Title: Incidence of Severe Hepatotoxicity Related to Antiretroviral Therapy in HIV/HCV Coinfected Patients.
Introduction. Hepatotoxicity is a concern in HIV/hepatitis C virus (HCV) coinfected patients due to their underlying liver disease. This study assessed the incidence of hepatotoxicity in HIV/HCV co-infected patients in two outpatient infectious diseases clinics. Methods. HIV/HCV co-infected adults were included in this retrospective study if they were PI or NNRTI naïve at their first clinic visit and were initiated on an NNRTI- and/or PI-based antiretroviral regimen. Patients were excluded if they had active or chronic hepatitis B virus (HBV). The primary objective was to determine the overall incidence of severe hepatotoxicity. Results. Fifty-six of the 544 patients identified met... (truncated)

Title: Liver damage and kinetics of hepatitis C virus and human immunodeficiency virus replication during the early phases of combination antiretroviral treatment.
In order to assess the relationship between human immunodeficiency virus (HIV) RNA, hepatitis C virus (HCV) RNA, CD4, CD8, and liver enzymes during combination antiretroviral therapy, these parameters were measured in 12 HIV-HCV-coinfected patients (who were naive for antiretrovirals) on the day before and 3, 7, 14, 28, 56, and 84 days after initiating the following treatments: stavudine and lamivudine in all patients, indinavir in 6 patients, and nevirapine in 6 patients. HIV RNA declined rapidly, CD4 cells increased slowly, and CD8 cells and liver enzymes were stable. HCV RNA showed a transient significant increase at days 14 and 21... (truncated)

Title: Acute hepatitis in a patient treated with saquinavir and ritonavir: absence of cross-toxicity with indinavir.
No abstract available.

Title: Severe hepatitis in three AIDS patients treated with indinavir.
No abstract available.

Title: Severe hepatitis in patients with AIDS and haemophilia B treated with indinavir.
No abstract available.

MESH:C106389 - conivaptan

Summary:

LLM Prediction Score	0.344 (normalized)
LLM Confidence Score	0.940
Golden Answer (Severity Class)	0.0 (normalized)
Prediction Error	0.344

Retrieved Context:

Title: Cardiorenal Syndrome: Role of Arginine Vasopressin and Vaptans in Heart Failure.
Heart and kidney failure continued to be of increasing prevalence in today's society, and their comorbidity has synergistic effect on the morbidity and mortality of patients. Cardiorenal syndrome (CRS) is a complex disease with multifactorial pathophysiology. Better understanding of this pathophysiological network is crucial for the successful intervention to prevent advancement of the disease process. One of the major factors in this process is neurohormonal activation, predominantly involving renin-angiotensin-aldosterone system (RAAS) and arginine vasopressin (AVP). Heart failure causes reduced cardiac output/cardiac index (CO/CI) and fall in renal perfusion pressures resulting in activation of baroreceptors and RAAS, respectively. Activated baroreceptors and... (truncated)

Title: Conivaptan and its role in the treatment of hyponatremia.
Hyponatremia is the most common electrolyte abnormality in hospitalized patients and is associated with increased morbidity and mortality. The recognition of the central role that arginin vasopressin plays in the pathogenesis of hyponatremia and the discovery that its actions are mediated by stimulation of V(1A) and V(2) receptors have led to the development of a new class of drugs, the arginin vasopressin antagonists. Conivaptan is a nonselective V(1A) and V(2) receptors antagonist that was the first of this class to be approved by the FDA for the management of euvolemic and hypervolemic hyponatremia. Its short-term safety and efficacy for the... (truncated)

Title: Pharmacokinetics of conivaptan use in patients with severe hepatic impairment.
Label="PURPOSE" NlmCategory="OBJECTIVE">Conivaptan is an intravenous dual V_{1A}/V₂ vasopressin antagonist approved for the treatment of euvolemic and hypervolemic hyponatremia. Earlier studies showed that patients with moderate liver disease could be safely treated with conivaptan by reducing the dose by 50%, whereas patients with mild hepatic impairment needed no dose adjustment. The objective of this Phase 1, open-label study was to assess the pharmacokinetics, protein binding, and safety of 48 h of conivaptan infusion in individuals with severe hepatic impairment.

Title: Hyponatremia in Patients with Cirrhosis of the Liver.
Hyponatremia is common in cirrhosis. It mostly occurs in an advanced stage of the disease and is associated with complications and increased mortality. Either hypovolemic or, more commonly, hypervolemic hyponatremia can be seen in cirrhosis. Impaired renal sodium handling due to renal hypoperfusion and increased arginine-vasopressin secretion secondary to reduced effective volemia due to peripheral arterial vasodilation represent the main mechanisms leading to dilutional hyponatremia in this setting. Patients with cirrhosis usually develop slowly progressing hyponatremia. In different clinical contexts, it is associated with neurological manifestations due to increased brain water content, where the intensity is often magnified by concomitant... (truncated)

Title: Tolvaptan: the evidence for its therapeutic value in acute heart failure syndrome.
Acute heart failure syndrome (AHFS) is one of the leading causes of hospital admission in the US. Tolvaptan is a vasopressin V(2) receptor antagonist that blocks the effect of arginine vasopressin (AVP) in reabsorbing water from the collecting ducts of the nephrons in congestive heart failure.

MESH:D009599 - nitroprusside

Summary:

LLM Prediction Score	0.343 (normalized)
LLM Confidence Score	0.990
Golden Answer (Severity Class)	0.0 (normalized)
Prediction Error	0.343

Retrieved Context:

Title: Organotoxic effects of excessive doses of sodium nitroprusside in the rabbit.
The simultaneous iv. infusion in conscious rabbits of 7.5 mg/kg.h sodium nitroprusside (SNP) plus sodium thiosulfate in the molar ratios 1:5 or 1:10, respectively, for 4 h produced perilobular necroses of liver cells. 21 days after the infusion, regeneration of the damaged cells was complete. No histological changes were found in various other organs after this high dose of SNP. No signs of liver toxicity were found in rabbits that had received 0.75 mg/kg.h SNP for 8 h daily during a period of 5 consecutive days. This dose was in the range of SNP doses recommended for clinical use in... (truncated)

Title: Interleukin-1 and nitric oxide protect against tumor necrosis factor alpha-induced liver injury through distinct pathways.
Mice sensitized with D-galactosamine (GalN) and challenged with recombinant murine tumor necrosis factor alpha (TNF alpha) developed severe apoptotic and secondary necrotic liver injury as assessed by histology, measurement of cytosolic DNA fragments, and determination of liver specific enzymes in plasma. Pretreatment with recombinant human interleukin-1 beta (IL-1) rendered mice insensitive to this TNF alpha toxicity. Coadministration of the liver-specific transcriptional inhibitor GalN with IL-1 prevented the development of tolerance, implicating de novo synthesis of liver specific proteins in the induction of tolerance. Pretreatment of mice with IL-1 resulted in elevated levels of nitrite/nitrate in serum and in enhanced nitric... (truncated)

Title: The effects of sodium nitroprusside-induced hypotension on splanchnic perfusion and hepatocellular integrity.
The purpose of our study was to investigate the effects of sodium nitroprusside-induced hypotension on splanchnic perfusion and hepatocellular integrity. Thirty patients undergoing radical prostatectomy were allocated randomly to a sodium nitroprusside (SNP) or control group (control). Regional pco2 was measured using gastric tonometry, and the regional to arterial difference in partial pressure of CO2 and intramucosal pH were calculated. The cytosolic liver enzyme alpha-glutathione S-transferase and standard liver enzyme markers (alanine aminotransferase, aspartate aminotransferase, and gamma-glutamyltransferase) were also measured. Mean arterial pressure in the SNP group was 50 mm Hg for 97 min during surgery. A significant increase from... (truncated)

Title: Perhexiline Therapy in Patients with Type 2 Diabetes: Incremental Insulin Resistance despite Potentiation of Nitric Oxide Signaling.
Perhexiline (Px) inhibits carnitine palmitoyltransferase 1 (CPT1), which controls uptake of long chain fatty acids into mitochondria. However, occasional cases of hypoglycaemia have been reported in Px-treated patients, raising the possibility that Px may also increase sensitivity to insulin. Furthermore, Px increases anti-aggregatory responses to nitric oxide (NO), an effect which may theoretically parallel insulin sensitization. We therefore sought to examine these relationships in patients with stable Type 2 diabetes (T2D) and cardiovascular disease (n = 30). Px was initiated, and dosage was titrated, to reach the therapeutic range and thus prevent toxicity. Investigations were performed before and after 2... (truncated)

Title: Ischemic pancreatitis and hepatitis secondary to ergotamine poisoning.
Acute ergotamine intoxication in a 29-year-old man was complicated by peripheral ischemia, pancreatitis, and hepatitis. The patient was treated with sodium nitroprusside infusion. Complications and treatment of ergotamine poisoning are discussed.

MESH:D007545 - isoproterenol

Summary:

LLM Prediction Score	0.343 (normalized)
LLM Confidence Score	0.970
Golden Answer (Severity Class)	0.0 (normalized)
Prediction Error	0.343

Retrieved Context:

Title: A Comparative Study of Rat Urine ¹H-NMR Metabolome Changes Presumably Arising from Isoproterenol-Induced Heart Necrosis Versus Clarithromycin-Induced QT Interval Prolongation.
Cardiotoxicity remains a challenging concern both in drug development and in the management of various clinical situations. There are a lot of examples of drugs withdrawn from the market or stopped during clinical trials due to unpredicted cardiac adverse events. Obviously, current conventional methods for cardiotoxicity assessment suffer from a lack of predictivity and sensitivity. Therefore, there is a need for developing new tools to better identify and characterize any cardiotoxicity that can occur during the pre-clinical and clinical phases of drug development as well as after marketing in exposed patients. In this study, isoproterenol and clarithromycin were used as... (truncated)

Title: Crosstalk between beta-adrenergic and insulin signaling mediates mechanistic target of rapamycin hyperactivation in liver of high-fat diet-fed male mice.
Nonalcoholic fatty liver disease (NAFLD) is the most common cause of chronic liver disease. While increased nutrient intake and sympathetic activity have been associated with the disease, the pathogenesis of NAFLD remains incompletely understood. We investigated the impact of the interaction of high dietary fat and sugar intake with increased beta-adrenergic receptor (β-AR) signaling on the activity of nutrient-sensing pathways and fuel storage in the liver. C57BL/6J mice were fed a standard rodent diet (STD), a high-fat diet (HFD), a high-fat/high-sugar Western diet (WD), a high-sugar diet with mixed carbohydrates (HCD), or a high-sucrose diet (HSD). After 6 week on... (truncated)

Title: Natural Sympathomimetic Drugs: From Pharmacology to Toxicology.
Sympathomimetic agents are a group of chemical compounds that are able to activate the sympathetic nervous system either directly via adrenergic receptors or indirectly by increasing endogenous catecholamine levels or mimicking their intracellular signaling pathways. Compounds from this group, both used therapeutically or abused, comprise endogenous catecholamines (such as adrenaline and noradrenaline), synthetic amines (e.g., isoproterenol and dobutamine), trace amines (e.g., tyramine, tryptamine, histamine and octopamine), illicit drugs (e.g., ephedrine, cathinone, and cocaine), or even caffeine and synephrine. In addition to the effects triggered by stimulation of the sympathetic system, the discovery of trace amine associated receptors (TAARs) in humans... (truncated)

Title: Hepatoprotective effects of betaine on liver damages followed by myocardial infarction.
Myocardial infarction is commonly considered as a leading cause of cardiovascular disease taking the lives of seven million people annually. Liver dysfunction is associated with cardiac diseases. The profile of abnormal liver functions in heart failure is not clearly defined. This study was designed to investigate the protective effects of betaine on liver injury after myocardial infarction induced by isoprenaline in rats. Forty-eight male rats were divided into four groups: the control group received normal diet and the experimental groups received 50, 150, and 250 mg kg⁻¹ body weight of betaine daily through gastric gavages for 60 days. All of... (truncated)

Title: Hepatoprotective Role of Carvedilol against Ischemic Hepatitis Associated with Acute Heart Failure via Targeting miRNA-17 and Mitochondrial Dynamics-Related Proteins: An In Vivo and In Silico Study.
Acute heart failure (AHF) is one of the most common diseases in old age that can lead to mortality. Systemic hypoperfusion is associated with hepatic ischemia-reperfusion injury, which may be irreversible. Ischemic hepatitis due to AHF has been linked to the pathogenesis of liver damage. In the present study, we extensively investigated the role of mitochondrial dynamics-related proteins and their epigenetic regulation in ischemic liver injury following AHF and explored the possible hepatoprotective role of carvedilol. The biochemical analysis revealed that the ischemic liver injury following AHF significantly elevated the activity of alanine aminotransferase (ALT), aspartate aminotransferase (AST), and alkaline... (truncated)

MESH:C017717 - sodium thiosulfate

Summary:

LLM Prediction Score	0.340 (normalized)
LLM Confidence Score	0.990
Golden Answer (Severity Class)	0.0 (normalized)
Prediction Error	0.340

Retrieved Context:

Title: Organotoxic effects of excessive doses of sodium nitroprusside in the rabbit.
The simultaneous iv. infusion in conscious rabbits of 7.5 mg/kg.h sodium nitroprusside (SNP) plus sodium thiosulfate in the molar ratios 1:5 or 1:10, respectively, for 4 h produced perilobular necroses of liver cells. 21 days after the infusion, regeneration of the damaged cells was complete. No histological changes were found in various other organs after this high dose of SNP. No signs of liver toxicity were found in rabbits that had received 0.75 mg/kg.h SNP for 8 h daily during a period of 5 consecutive days. This dose was in the range of SNP doses recommended for clinical use in... (truncated)

Title: Hydrogen Sulfide as a Novel Regulatory Factor in Liver Health and Disease.
Hydrogen sulfide (H₂S), a colorless gas smelling of rotten egg, has long been recognized as a toxic gas and environment pollutant. However, increasing evidence suggests that H₂S acts as a novel gasotransmitter and plays important roles in a variety of physiological and pathological processes in mammals. H₂S is involved in many hepatic functions, including the regulation of oxidative stress, glucose and lipid metabolism, vasculature, mitochondrial function, differentiation, and circadian rhythm. In addition, H₂S contributes to the pathogenesis and treatment of a number of liver diseases, such as hepatic fibrosis, liver cirrhosis, liver cancer, hepatic ischemia/reperfusion injury, nonalcoholic fatty liver disease/nonalcoholic... (truncated)

Title: The H₂S Donor Sodium Thiosulfate (Na₂S₂O₃) Does Not Improve Inflammation and Organ Damage After Hemorrhagic Shock in Cardiovascular Healthy Swine.
We previously demonstrated marked lung-protective properties of the H₂S donor sodium thiosulfate (Na₂S₂O₃, STS) in a blinded, randomized, controlled, long-term, resuscitated porcine model of swine with coronary artery disease, i.e., with decreased expression of the H₂S-producing enzyme cystathionine-γ-lyase (CSE). We confirmed these beneficial effects of STS by attenuation of lung, liver and kidney injury in mice with genetic CSE deletion (CSE-ko) undergoing trauma-and-hemorrhage and subsequent intensive care-based resuscitation. However, we had previously also shown that any possible efficacy of a therapeutic intervention in shock states depends both on the severity of shock as well as on the presence or absence... (truncated)

Title: Effect of Short-Term Tacrolimus Exposure on Rat Liver: An Insight into Serum Antioxidant Status, Liver Lipid Peroxidation, and Inflammation.
Tacrolimus (TAC) is an immunosuppressive drug, optimally used for liver, kidney, and heart transplant to avoid immune rejection. In retrospect, a multitude of studies have reported effects of TAC, such as nephrotoxicity, diabetes, and other complications. However, limited information is available regarding short-term exposure of TAC on the liver. Therefore, the present study was designed to unravel the effects of short-term exposure of TAC on a rat model. The animal model was established by TAC administration for 6, 12, 24, and 48 h time points. Liver histopathological changes were observed with PAS-D, reticulin stain, and immunostaining of PCNA and CK-7... (truncated)

Title: Emerging pharmacological tools to control hydrogen sulfide signaling in critical illness.
Hydrogen sulfide (H₂S) has long been known as a toxic environmental hazard. Discovery of physiological roles of H₂S as a neurotransmitter by Kimura and colleagues triggered an intensive research in the biological roles of H₂S in the past decades. Manipulation of H₂S levels by inhibiting H₂S synthesis or administration of H₂S-releasing molecules revealed beneficial as well as harmful effects of H₂S. As a result, it is now established that H₂S levels are tightly controlled and too much or too little H₂S levels cause harm. Nonetheless, translation of sulfide-based therapy to clinical practice has been stymied due to the very low... (truncated)

MESH:D011718 - pyrazinamide

Summary:

LLM Prediction Score	0.713 (normalized)
LLM Confidence Score	0.990
Golden Answer (Severity Class)	0.375 (normalized)
Prediction Error	0.338

Retrieved Context:

Title: Pyrazinamide-induced granulomatous hepatitis.
Noncaseating granulomatous hepatitis may be caused by a variety of drugs, but we have not found, by computer search of the literature, a previous describe of granulomatous hepatitis associated with pyrazinamide. We describe a 52-year-old man with hectic fever, chills, extreme fatigue, liver damage, and hyperuricemia about 4 weeks after commencing pyrazinamide therapy. A liver biopsy specimen showed noncaseating epithelioid granulomas. The patient recovered soon after the interruption of tuberculostatic treatment.

Title: Deleterious influence of pyrazinamide on the outcome of patients with fulminant or subfulminant liver failure during antituberculous treatment including isoniazid.
Isoniazid and pyrazinamide are well-known hepatotoxic drugs, often used in combination. The aim of this study was to assess the prognostic influence of pyrazinamide on the outcome of fulminant or subfulminant liver failure caused by antituberculous therapy. Eighteen patients with fulminant or subfulminant liver failure due to antituberculous therapy were studied. Nine patients received isoniazid and rifampicin without pyrazinamide (group 1), and nine patients received isoniazid and rifampicin together with pyrazinamide (group 2). The severity of fulminant and subfulminant liver failure, as judged by the prevalence of coma and the lowest level of factor V, was similar in the two... (truncated)

Title: [Liver injury under tuberculostatic treatment].
We report the case of a patient with nausea, loss of appetite and increase of the aminotransferase levels to eight times the upper normal limit occurring two weeks after she was started on isoniazide, rifampicine and pyrazinamide for treatment of tuberculosis. Isoniazide is the most likely cause of liver injury occurring during combined antituberculosis therapy, whereas pyrazinamide or rifampicine are only rarely responsible. The case presented is used to review and compare the different recommendations concerning the monitoring of patients receiving antituberculous therapy and the clinical management of patients developing liver injury.

Title: Recurrent dysosmia induced by pyrazinamide.
Pyrazinamide can have adverse effects such as hepatic toxicity, hyperuricemia or digestive disorders. In rare cases, alterations in taste and smell function have been reported for pyrazinamide when combined with other drugs. We report a case of reversible olfactory disorder related to pyrazinamide in a woman, with a positive rechallenge. The patient presented every day a sensation of smelling something burning 15 min after drug intake. Dysosmia disappeared completely after pyrazinamide withdrawal and recurred after its rechallenge. The case was reported to the Tunisian Centre of Pharmacovigilance.

Title: Clinical characteristics of pyrazinamide-associated hepatotoxicity in patients at a hospital in Lima, Peru.
In order to determine the characteristics of drug-induced liver injury (DILI), adult patients diagnosed with tuberculosis and with an anti-tuberculosis treatment scheme including pyrazinamide were studied. The re-exposure process was used for the cause-effect analysis of the DILI. A total of 10 patients were found with pyrazinamide-associated DILI; the median age and hospital stay were 40.5 years (from 22 to 76 years) and 41 days (from 11 to 130 days), respectively. The median time in which the events appeared was 14 days (from 3 to 46 days); jaundice was observed in 4 patients and radiological patterns such as hepatocellular, mixed... (truncated)

MESH:D007612 - kanamycin

Summary:

LLM Prediction Score	0.335 (normalized)
LLM Confidence Score	0.990
Golden Answer (Severity Class)	0.0 (normalized)
Prediction Error	0.335

Retrieved Context:

Title: A Comprehensive Assessment of the Safety of <i>Blautia producta</i> DSM 2950.
In recent years, <i>Blautia</i> has attracted attention for its role in ameliorating host diseases. In particular, <i>Blautia producta</i> DSM 2950 has been considered a potential probiotic due to its ability to mitigate inflammation in poly(I:C) induced HT-29 cells. Thus, to promote the development of indigenous intestinal microorganisms with potential probiotic function, we conducted a comprehensive experimental analysis of DSM 2950 to determine its safety. This comprised a study of its potential virulence genes, antibiotic resistance genes, genomic islands, antibiotic resistance, and hemolytic activity and a 14-day test of its acute oral toxicity in mice. The results indicated no toxin-related virulence... (truncated)

Title: [Antibiotic sensitivity of Yersinia enterocolitica and Yersinia pseudotuberculosis isolated from patients with the jaundice form of acute hepatitis].
Sensitivity to 12 antibiotics of Y. enterocolitica and Y. pseudotuberculosis isolated from patients with acute hepatitis in the jaundice form was studied. It was found that the representatives of Y. enterocolitica markedly differed from the strains of Y. pseudotuberculosis by their sensitivity to penicillins: the MIC for the first was greater than 1600 micrograms/ml and for the latter it was 0.1--3.2 micrograms/ml. Among the aminoglycosides gentamicin proved to be the most active against both species. Its MIC with respect to these organisms was 0.1--3.2 micrograms/ml. The other aminoglycoside antibiotics by their activity levels may be arranged as follows: tobramycin, sisomicin,... (truncated)

Title: [Evaluation of hepatic function in new cases of pulmonary tuberculosis due to the use of standard chemotherapy regimens I and IIB].
The frequency and magnitude of hepatotoxic reactions were compared in 147 new cases of pulmonary tuberculosis within the first three months of chemotherapy (CT) by standard regimen 1 [H, R, Z, S (E)] (Group 1) and regimen 2B [the same drugs + kanamycin (amikacin) and fluoroquinolones] (Group 2). Their efficiency was evaluated from 6 serum indices--the level of bilirubin, the activities of alanine aminotransferase (ALT), aspartate aminotransferase (AST), alkaline phosphatase (AP), and gamma-glutamyl transpeptidase (GGTP), and thymol test results. Tests were monthly carried out. The results were separately analyzed in patients with and without baseline abnormalities in the indices being... (truncated)

Title: A case of severe acute hepatitis of unknown etiology treated with the Chinese herbal medicine Inchinko-to.
A prolonged severe hepatitis of unknown etiology was treated with Inchinko-to, a Chinese herbal medicine, and this case is herein described. Inchinko-to was given with ursodeoxycholic acid (UDCA) and glycyrrhizin. The improvement in the patient's liver function seemed to be accelerated after the treatment, especially after stopping the administration of kanamycin sulfate which might possibly inhibit the conversion of geniposide, one of the constituents of Inchinko-to, to an active ingredient through the suppression of the bacterial growth in intestinal flora, suggesting the usefulness of Inchinko-to for treatment of severe hepatitis.

Title: Cefoperazone versus cefotaxime, plus amikacin or sisomicin, in fever and infection in hematologic granulocytopenic patients.
Forty patients with leukemia or aplastic anemia were randomized to receive one of the following antibiotic regimens at the onset of fever during granulocytopenia: cefoperazone + amikacin (regimen A), cefoperazone + sisomicin (regimen B), cefotaxime + amikacin (regimen C), cefotaxime + sisomicin (regimen D). All patients were receiving gut decontamination at the time of randomization. Patients were monitored twice weekly with swabs and cultures for bacteria and fungi. Overall, there were 56 febrile episodes: 31 were proven bacterial, 3 were probable, and 16 were of unknown origin. Response rates were comparable in all 4 treatment regimens: 90%, 91%, 92% and... (truncated)

MESH:D016595 - misoprostol

Summary:

LLM Prediction Score	0.041 (normalized)
LLM Confidence Score	0.980
Golden Answer (Severity Class)	0.375 (normalized)
Prediction Error	0.334

Retrieved Context:

Title: Misoprostol therapeutics revisited.
Misoprostol, a prostaglandin E1 analog, is a racemate of four stereoisomers. On administration it rapidly de-esterifies to its active form, misoprostolic acid. Misoprostolic acid is 85% albumin bound and has a half-life of approximately 30 minutes. It is excreted in urine as inactive metabolites. No significant drug interactions have been reported. Besides its gastrointestinal protective and uterotonic activities, misoprostol regulates various immunologic cascades. It inhibits platelet-activating factor and leukocyte adherence, and modulates adhesion molecule expression. It protects against gut irradiation injury, experimental gastric cancer, enteropathy, and constipation. It improves nutrient absorption in cystic fibrosis. Misoprostol has utility in acetaminophen and... (truncated)

Title: Histopathology and oxidative stress analysis of concomitant misoprostol and celecoxib administration.
Nonsteroidal anti-inflammatory drugs (NSAIDs), non-selective or selective inhibitors of cyclooxygenase (COX-1 and -2), reduce pain and inflammation associated with arthritic diseases. Celecoxib, a COX-2-selective inhibitor providing decreased gastric injury relative to non-selective NSAIDs, is commonly prescribed. Misoprostol, a prostaglandin analog, supplements NSAID-inhibited prostaglandin levels. As concomitant celecoxib and misoprostol administration has been shown to intensify renal adverse effects, this article examined the influence of concomitant administration on hepatic histopathology, oxidative stress, and celecoxib concentration. On days 1 and 2, rat groups (n = 6) were gavaged twice daily (two groups with vehicle and two groups with 100 µg/kg misoprostol). From... (truncated)

Title: Misoprostol modulates cytokine expression through a cAMP pathway: Potential therapeutic implication for liver disease.
Dysregulated cytokine metabolism plays a critical role in the pathogenesis of many forms of liver disease, including alcoholic and non-alcoholic liver disease. In this study we examined the efficacy of Misoprostol in modulating LPS-inducible TNFα and IL-10 expression in healthy human subjects and evaluated molecular mechanisms for Misoprostol modulation of cytokines in vitro. Healthy subjects were given 14day courses of Misoprostol at doses of 100, 200, and 300µg four times a day, in random order. Baseline and LPS-inducible cytokine levels were examined ex vivo in whole blood at the beginning and the end of the study. Additionally, in vitro studies... (truncated)

Title: Gastrointestinal adverse effects of non-steroidal anti-inflammatory drugs.
NSAIDs are used extensively worldwide at a cost of billions of dollars annu-ally. Adverse side effects, especially in the gastrointestinal (GI) tract, are uncommon but cause a substantial burden of illness because of the volume of use. Important upper GI complications include dyspepsia, gastric erosions and peptic ulcers and complications such as bleeding, perforation or gastric outlet obstruction. Dyspeptic symptoms may occur without correlation to endoscopic findings. Topical injury and COX-1 inhibition resulting in gastric prostaglandin suppression are two commonly postulated mechanisms of gastroduodenal damage. Advanced age, previous peptic ulcers or ulcer complications, concomitant use of glucocorticoids or anticoagulants, and... (truncated)

Title: Prostaglandin I(2) and E(2) mediate the protective effects of cyclooxygenase-2 in a mouse model of immune-mediated liver injury.
Studies of the molecular and cellular mechanisms of concanavalin A (ConA)-induced liver injury have provided important knowledge on the pathogenesis of many liver diseases involving hepatic inflammation. However, studies identifying hepato-protective factors based on the mechanistic understanding of this model are lacking. Evidence suggests that certain prostaglandin (PG) products of cyclooxygenase (COX)-1 and COX-2 provide important anti-inflammatory and cytoprotective functions in some pathophysiological states. In the present study, we demonstrate a protective role of COX-2 derived PGs in ConA-induced liver injury. COX-2(-/-) mice developed much more severe liver damage upon ConA treatment compared with wild-type and COX-1(-/-) mice. Treatment of... (truncated)

MESH:D004008 - diclofenac

Summary:

LLM Prediction Score	0.666 (normalized)
LLM Confidence Score	0.990
Golden Answer (Severity Class)	1.0 (normalized)
Prediction Error	0.334

Retrieved Context:

Title: Diclofenac hepatitis.
The characteristics of liver damage associated with the use of diclofenac, a popular nonsteroidal anti-inflammatory drug, were investigated by reviewing adverse drug reaction reports for Australia. Twenty six patients were reported for whom diclofenac was the sole suspected drug cause of their liver damage. The average age of the patients was 64 years (range 37-84 years); 19 (70%) were women. The most common clinical features were jaundice, hepatomegaly, anorexia, and nausea. Features of drug hypersensitivity were not reported. Duration of treatment with diclofenac before the onset of the illness ranged from 6-417 days (median 76 days). The most prominent biochemical... (truncated)

Title: Diclofenac induced hepatitis. 3 cases with features of autoimmune chronic active hepatitis.
Diclofenac is a frequently prescribed nonsteroidal antiinflammatory drug (NSAID). Significant hepatotoxicity related to diclofenac may be more common than previously recognized, as three patients with diclofenac-associated hepatitis were seen by one clinician in a single year. All patients were ANA positive during the hepatitis and had histologic features of chronic active hepatitis. Two had been inappropriately treated with corticosteroids. The third patient presented more acutely with jaundice and symptoms of hepatitis. Two of the patients developed the same hepatic reaction when rechallenged with diclofenac. The third patient was changed to tiaprofenic acid, a NSAID of the same family, and redeveloped... (truncated)

Title: Diclofenac-associated hepatotoxicity.
Diclofenac sodium, a phenylacetic acid-derived nonsteroidal anti-inflammatory drug (NSAID) recently released in the United States, was associated with the development of significant hepatitis in seven patients, with one associated death. Signs and symptoms developed within several weeks of initiation of drug use and generally resolved 4 to 6 weeks following discontinuation of use of the drug. The only patient rechallenged with the drug developed a recurrence of her hepatic abnormalities. In one patient, fatal, fulminant hepatitis developed despite early withdrawal of the drug. Review of the European literature disclosed three additional fatalities associated with diclofenac therapy. It is unclear whether... (truncated)

Title: Diclofenac hepatitis.
We report five cases of biopsy-proven hepatitis developing between six and 20 weeks after administration of diclofenac. In one patient jaundice had previously developed following use of ibuprofen. In another the clinical, biochemical and histopathological features were those of chronic active hepatitis and treatment with corticosteroids was required. All patients recovered from their liver injury without sequelae. Resolution of symptoms occurred between three and 12 weeks following cessation of the drug, while liver function tests returned to normal between seven and 16 weeks after drug withdrawal, except in the patient with chronic active hepatitis who remained biochemically abnormal for eight... (truncated)

Title: Diclofenac associated hepatitis.
Diclofenac is a widely used non-steroidal anti-inflammatory drug, being the most commonly prescribed of its kind in the world. This paper describes five cases of hepatitis with clinical features indicating a direct link with diclofenac. All the patients presented with an acute hepatitis, three being jaundiced. They gave a history of taking diclofenac up to the time of presentation, four of the five having started the drug within the previous 3 months. There were no other features in the histories to suggest alternative causes for the liver dysfunction. Liver function tests were grossly abnormal in all cases, showing a hepatitic... (truncated)

MESH:D015215 - zidovudine

Summary:

LLM Prediction Score	0.668 (normalized)
LLM Confidence Score	0.990
Golden Answer (Severity Class)	1.0 (normalized)
Prediction Error	0.332

Retrieved Context:

Title: Comparative pharmacokinetics of zidovudine in healthy volunteers and in patients with AIDS with and without hepatic disease.
To understand whether disease caused by the human immunodeficiency virus (HIV) affects zidovudine disposition, we compared the drug's pharmacokinetics in six healthy volunteers; six persons with the acquired immunodeficiency syndrome (AIDS) and no evidence of gastrointestinal (nausea, vomiting, diarrhea), renal (elevated blood urea nitrogen, serum creatinine), or hepatic (elevated liver function tests) disease; and three patients with AIDS and hepatic disease. After a single oral dose of zidovudine, serial blood samples were analyzed for drug concentration by radioimmunoassay. A one-compartment oral absorption model was fit to the concentration-time data. The absorption rate constant (4.05 vs 2.11 hr-1) and time to... (truncated)

Title: Zidovudine-induced mitochondrial disorder with massive liver steatosis, myopathy, lactic acidosis, and mitochondrial DNA depletion.
Zidovudine is known to be responsible for a mitochondrial myopathy with ragged-red fibres and mitochondrial DNA depletion in muscle. Lactic acidosis alone or associated with hepatic abnormalities has also been reported. A single report mentioned the concomitant occurrence of muscular and hepatic disturbances and lactic acidosis in a patient receiving zidovudine, but muscle and liver tissues were not studied. A 57-year-old man with AIDS, who had been treated with zidovudine for 3 years, developed fatigue and weight loss. Serum creatine kinase and hepatic enzyme levels were high. Lactic acidosis was present. Liver biopsy showed diffuse macrovacuolar and microvacuolar steatosis. After... (truncated)

Title: Combination therapy with recombinant human soluble CD4-immunoglobulin G and zidovudine in patients with HIV infection: a phase I study.
To determine the effect of zidovudine (ZDV) on the pharmacokinetic disposition of recombinant soluble CD4 immunoglobulin G (rCD4-IgG) and to evaluate the safety and preliminary activity of concurrent administration of ZDV with rCD4-IgG, we undertook an open-label, dose-escalating, 12-week study. The regimens of intravenous rCD4-IgG and oral ZDV we used were (a) 300 micrograms/kg rCD4-IgG twice per week and 300 mg ZDV per day, (b) 300 micrograms/kg rCD4-IgG twice per week and 600 mg ZDV per day, (c) 1,000 micrograms/kg rCD4-IgG twice per week and 300 mg ZDV per day, (d) 1,000 micrograms/kg rCD4-IgG twice per week and 600 mg... (truncated)

Title: Massive hepatic steatosis and lactic acidosis in a patient with AIDS who was receiving zidovudine.
Massive steatosis has recently been described among a few human immunodeficiency virus-seropositive patients who were receiving antiretroviral therapy. Although clinical and light-microscopic pathological findings were carefully described, no ultrastructural studies of the liver were performed in these cases. We report the light-microscopic and ultrastructural findings at autopsy of a 35-year-old woman with AIDS who developed severe lactic acidosis and hepatic failure. The patient had been receiving standard doses of zidovudine for 5 months when she was hospitalized because of the rapid onset of abdominal pain, nausea, and vomiting. The most significant findings at autopsy were massive hepatomegaly and steatosis. Ultrastructural... (truncated)

Title: Zidovudine (AZT) and hepatic lipid accumulation: implication of inflammation, oxidative and endoplasmic reticulum stress mediators.
The clinical effectiveness of Zidovudine (AZT) is constrained due to its side-effects including hepatic steatosis and toxicity. However, the mechanism(s) of hepatic lipid accumulation in AZT-treated individuals is unknown. We hypothesized that AZT-mediated oxidative and endoplasmic reticulum (ER) stress may play a role in the AZT-induced hepatic lipid accumulation. AZT treatment of C57BL/6J female mice (400 mg/day/kg body weight, i.p.) for 10 consecutive days significantly increased hepatic triglyceride levels and inflammation. Markers of oxidative stress such as protein oxidation, nitration, glycation and lipid peroxidation were significantly higher in the AZT-treated mice compared to vehicle controls. Further, the levels of ER... (truncated)

MESH:D005479 - flurazepam

Summary:

LLM Prediction Score	0.171 (normalized)
LLM Confidence Score	0.980
Golden Answer (Severity Class)	0.5 (normalized)
Prediction Error	0.329

Retrieved Context:

Title: Cimetidine protects against acetaminophen toxicity.
Generally, acetaminophen (APAP) overdoses with elimination half-lives over 4 hr. sustain liver damage. In the following cases, cimetidine (C) seems to have protected against APAP toxicity. An 18 yr. old, 64 kg female smoker presented 6 hr. after taking 10Rg APAP, 1200+ mg C, and small amounts of flurazepam and Sleepeze (methaprilene + scopolamine). Three plasma APAP levels (by HPLC) revealed an elimination half-life of 4.4 hr. C did not interfere with the APAP assay. Despite the long half-life in a patient with microsomal enzymes induced by smoking, no evidence of hepatotoxicity developed. A month later, the same patient overdosed... (truncated)

Title: Psychotropic drugs and liver disease: A critical review of pharmacokinetics and liver toxicity.
The liver is the organ by which the majority of substances are metabolized, including psychotropic drugs. There are several pharmacokinetic changes in end-stage liver disease that can interfere with the metabolization of psychotropic drugs. This fact is particularly true in drugs with extensive first-pass metabolism, highly protein bound drugs and drugs depending on phase I hepatic metabolic reactions. Psychopharmacological agents are also associated with a risk of hepatotoxicity. The evidence is insufficient for definite conclusions regarding the prevalence and severity of psychiatric drug-induced liver injury. High-risk psychotropics are not advised when there is pre-existing liver disease, and after starting a... (truncated)

Title: Deletion of Constitutive Androstane Receptor Led to Intestinal Alterations and Increased Imidacloprid in Murine Liver.
Imidacloprid (IMI) is the most frequently detected neonicotinoid pesticide in the environment. Despite typically low toxicity in vertebrates, IMI exposure is associated with liver and gastrointestinal toxicity. The mechanism underlying IMI toxicity in mammals is unclear. Pesticide exposure frequently activates xenobiotic nuclear receptors, such as the constitutive androstane receptor (CAR), to induce detoxification phase I and phase II genes. This study examined the role of CAR in mediating IMI off-target toxicity. Female <i>Car^{-/-}</i> and wild-type (WT) mice were orally administered imidacloprid (50 mg/kg, twice daily) for 21 days, following which serum, liver, and intestinal tissues were collected. Liver tissue analysis... (truncated)

Title: Continuous Infusion of Flumazenil in the Management of Benzodiazepines Detoxification.
An effective approach in the treatment of benzodiazepine (BZD) overdosing and detoxification is flumazenil (FLU). Studies in chronic users who discontinued BZD in a clinical setting suggested that multiple slow bolus infusions of FLU reduce BZD withdrawal symptoms. The aim of this study was to confirm FLU efficacy for reducing BZD withdrawal syndrome by means of continuous elastomeric infusion, correlated to drugs plasma level and patients' compliance. Methods: Seven-day FLU 1 mg/day subcutaneously injected through an elastomeric pump and BZDs lormetazepam, clonazepam, and lorazepam were assessed by HPLC-MS/MS in serum of patients before and after 4 and 7 days of... (truncated)

Title: Benzodiazepines reported in NFLIS-Drug, 2015 to 2018.
The National Forensic Laboratory Information System (NFLIS) is a program of the U.S. Drug Enforcement Administration, Diversion Control Division. The NFLIS-Drug component collects drug identification results and associated information from drug cases submitted to and analyzed by federal, state, and local forensic laboratories. This paper presents national annual estimates and national and regional yearly trend differences for clonazepam, diazepam, flubromazolam, clonazolam, and etizolam using annual report rates per 100,000 persons aged 15 or older between 2015 and 2018. An estimated 263,538 benzodiazepine reports were identified by state and local laboratories between 2015 and 2018. Methamphetamine, cocaine, and heroin accounted for... (truncated)

MESH:D010672 - phenytoin

Summary:

LLM Prediction Score	0.671 (normalized)
LLM Confidence Score	0.990
Golden Answer (Severity Class)	1.0 (normalized)
Prediction Error	0.329

Retrieved Context:

Title: Phenytoin hepatotoxicity: a review of the literature.
Phenytoin hepatotoxicity is a serious idiosyncratic reaction that occurs in less than one percent of patients. The onset of symptoms occurs early in therapy, usually within the first six weeks. Presenting symptoms often include fever, rash, lymphadenopathy, hepatomegaly, anorexia, and myalgias or arthralgias. Other significant findings that may develop throughout hospitalization are jaundice, periorbital or facial edema, and splenomegaly. The following alterations in liver function tests are associated with phenytoin hepatotoxicity: elevations in serum aminotransferases, lactic dehydrogenase, alkaline phosphatase, bilirubin, and prothrombin time. Rechallenges, lymphocyte stimulation test, and liver biopsy have been used to aid in the diagnosis. Rechallenge is... (truncated)

Title: Phenytoin-induced hypersensitivity reactions.
A case of phenytoin-induced hepatitis with mononucleosis is reported, and syndromes associated with phenytoin hypersensitivity reactions are discussed. A 23-year-old black woman with a two-month history of seizure disorder was admitted to a hospital with nausea, vomiting, fever, lymphadenopathy, diffuse maculopapular rash, left-upper-quadrant tenderness, and hepatomegaly. She was receiving phenytoin sodium 300 mg/day; carbamazepine 200 mg four times daily had been discontinued four days before admission because of leukopenia. Phenytoin was discontinued after admission; however, phenytoin 1 g i.v. was given for a tonic-clonic seizure two days after admission, after which swelling of the face and legs and pruritus developed.... (truncated)

Title: Free phenytoin toxicity.
Phenytoin has a narrow therapeutic window, and when managing cases of toxicity, clinicians are very wary of this fact. Typically, if patient presents with symptoms suggestive of phenytoin toxicity, total serum phenytoin is promptly ordered. That could be falsely low especially in elderly or critically ill patients, which may lead to a low albumin level resulting in this discrepancy. The free phenytoin can be best estimated using the Sheiner-Tozer equation. Herein, we describe a case of an elderly male patient who presented with drowsiness, gait changes, and elevated liver enzymes and a normal total serum phenytoin level of 18 ng/dL... (truncated)

Title: Phenytoin-induced toxic cholestatic hepatitis in a patient with skin lesions: case report.
Phenytoin is a highly effective and widely prescribed anticonvulsant agent, but it can be associated with dose-related side effects and hypersensitivity reactions. We present a case of phenytoin-induced cholestatic hepatotoxicity in a 47-year-old woman who had exfoliative dermatitis, an increase in liver enzymes with a cholestatic pattern, and eosinophilia after 25 days of phenytoin therapy. The diagnostic workup showed no other possible causes, and the results of a percutaneous liver biopsy were consistent with drug-induced toxic hepatitis. Within 3 weeks after discontinuing phenytoin therapy, her liver function tests returned to normal values.

Title: Cholestatic liver dysfunction after long-term phenytoin therapy.
Cholestatic jaundice developed in a 64-year-old woman who had received phenytoin sodium for more than 40 years. Discontinuation of phenytoin therapy resulted in resolution of the hepatic abnormalities, which recurred on rechallenge, strongly suggesting a causal relation to the drug. Phenytoin therapy was discontinued again, with complete resolution of the hepatic abnormalities. The liver biopsy specimen obtained during therapy showed cholestasis compatible with obstruction of the biliary tree, although an obstructive process was never demonstrated. The biochemical abnormalities and histologic features observed most likely represent an unusual response to phenytoin therapy.

MESH:C100294 - phendimetrazine

Summary:

LLM Prediction Score	0.329 (normalized)
LLM Confidence Score	0.950
Golden Answer (Severity Class)	0.0 (normalized)
Prediction Error	0.329

Retrieved Context:

Title: Anti-obesity effects of pectinase and cellulase enzyme-treated Ecklonia cava extract in high-fat diet-fed C57BL/6N mice.
The present study investigated the anti-obesity effects of enzyme-treated Ecklonia cava extract (EEc) in C57BL/6N mice with high-fat diet (HFD)-induced obesity. The EEc was separated and purified with the digestive enzymes pectinase (Rapidase X-Press L) and cellulase (Rohament CL) and its effects on the progression of HFD-induced obesity were examined over 10 weeks. The mice were divided into 6 groups (n=10/group) as follows: Normal diet group, HFD group, mice fed a HFD with 25 mg/kg/day Garcinia cambogia extract and mice fed a HFD with 5, 25 or 150 mg/kg/day EEc (EHD groups). Changes in body weight, fat, serum lipid levels... (truncated)

Title: The Evaluation of Drug Delivery Nanocarrier Development and Pharmacological Briefing for Metabolic-Associated Fatty Liver Disease (MAFLD): An Update.
Current research indicates that the next silent epidemic will be linked to chronic liver diseases, specifically non-alcoholic fatty liver disease (NAFLD), which was renamed as metabolic-associated fatty liver disease (MAFLD) in 2020. Globally, MAFLD mortality is on the rise. The etiology of MAFLD is multifactorial and still incompletely understood, but includes the accumulation of intrahepatic lipids, alterations in energy metabolism, insulin resistance, and inflammatory processes. The available MAFLD treatment, therefore, relies on improving the patient's lifestyle and multidisciplinary pharmacotherapeutic options, whereas the option of surgery is useless without managing the comorbidities of the MAFLD. Nanotechnology is an emerging approach addressing... (truncated)

Title: Bariatric Surgery and Liver Disease: General Considerations and Role of the Gut-Liver Axis.
Weight loss is a therapeutic solution for many metabolic disorders, such as obesity and its complications. Bariatric surgery aims to achieve lasting weight loss in all patients who have failed after multiple dietary attempts. Among its many benefits, it has been associated with the regression of non-alcoholic fatty liver disease (NAFLD), which is often associated with obesity, with evidence of substantial improvement in tissue inflammation and fibrosis. These benefits are mediated not only by weight loss, but also by favorable changes in systemic inflammation and in the composition of the gut microbiota. Changes in microbial metabolites such as short-chain fatty... (truncated)

Title: Obesity Management in the Primary Prevention of Hepatocellular Carcinoma.
Hepatocellular carcinoma (HCC) is the most frequent primary hepatic malignancy and a leading cause of cancer-related death globally. HCC is associated with an indolent clinical presentation, resulting in frequent advanced stage diagnoses where surgical resection or transplant therapies are not an option and medical therapies are largely ineffective at improving survival. As such, there is a critical need to identify and enhance primary prevention strategies to mitigate HCC-related morbidity and mortality. Obesity is an independent risk factor for the onset and progression of HCC. Furthermore, obesity is a leading cause of nonalcoholic steatohepatitis (NASH), the fasting growing etiological factor of... (truncated)

Title: Post-marketing withdrawal of anti-obesity medicinal products because of adverse drug reactions: a systematic review.
We identified anti-obesity medications withdrawn since 1950 because of adverse drug reactions after regulatory approval, and examined the evidence used to support such withdrawals, investigated the mechanisms of the adverse reactions, and explored the trends over time.

MESH:D013752 - tetracycline

Summary:

LLM Prediction Score	0.578 (normalized)
LLM Confidence Score	0.990
Golden Answer (Severity Class)	0.25 (normalized)
Prediction Error	0.328

Retrieved Context:

Title: [Tetracyclin intoxication versus idiopathic pancreatitis: report of a case with multiple organ involvement (author's transl)].
The tetracycline class of antibiotics is infrequently used in clinical pediatrics due to its side effects: they include anorexia, nausea, vomiting and diarrhea. Hypersensitivity, a photosensibility reaction and a brownish discoloration of teeth is less frequently, a pseudotumor cerebri is rarely seen. Once therapeutic plasma levels are exceeded however, either by overdosage or decreased renal or hepatic clearance of the drug, serious complications like a secondary Fanconi-Syndrom or a nephrogenic diabetes insipidus can occur. The increased toxicity of tetracyclines in pregnant women is well known. We would like to report a fatal case, where serious complications like a secondary Fanconi-Syndrom,... (truncated)

Title: Tetracycline-induced bile duct paucity and prolonged cholestasis.
Acute self-limited liver disease has been associated with tetracycline use. However, severe prolonged cholestatic hepatitis and bile duct paucity have not been previously attributed to tetracyclines. Hepatitis, characterized by prolonged jaundice, severe pruritus, and moderate increased transaminase values, occurred within 2 months of ingesting tetracyclines in two female patients. Serum bilirubin levels normalized 12 and 34 months after tetracycline ingestion. Liver histology revealed bile duct paucity, severe cholestasis, and minimal necrosis and inflammation. Tetracyclines may infrequently induce bile duct paucity and prolonged, severe, and reversible cholestasis.

Title: Association between tetracycline or doxycycline and hepatotoxicity: a population based case-control study.
An FDA Working Group, along with representatives of PhRMA and the American Association for the Study of Liver Diseases, as well as the Institute of Medicine Report 'To Err is Human: Building a Safer Health Care System' have suggested that post-marketing drug surveillance is a important method to decrease adverse drug events. While tetracyclines are known to cause hepatotoxicity, no post-marketing drug surveillance studies have examined the risk of developing hepatotoxicity with tetracyclines. Therefore, the objective of this study is to determine the difference in risk of hepatotoxicity in patients receiving doxycycline or tetracycline using California Medicaid claims.

Title: Doxycycline and hepatotoxicity.
The hepatotoxicity of tetracyclines is well known. If microvesicular steatosis due to a high dose of tetracycline has virtually disappeared, it can also be observed with other drugs belonging to the tetracycline family. To our knowledge, hepatotoxicity induced by doxycycline has never been reported. In our patient, the abrupt onset of hepatic failure, five days after the start of doxycycline and the rapid normalization after the drug was stopped, leads to suspect a causal relationship between doxycycline and liver insufficiency. We must however be careful before concluding, because our patient received also acetylsalicylic acid and paracetamol, two other potential hepatotoxic... (truncated)

Title: Hepatic safety of antibiotics used in primary care.
Antibiotics used by general practitioners frequently appear in adverse-event reports of drug-induced hepatotoxicity. Most cases are idiosyncratic (the adverse reaction cannot be predicted from the drug's pharmacological profile or from pre-clinical toxicology tests) and occur via an immunological reaction or in response to the presence of hepatotoxic metabolites. With the exception of trovafloxacin and telithromycin (now severely restricted), hepatotoxicity crude incidence remains globally low but variable. Thus, amoxicillin/clavulanate and co-trimoxazole, as well as flucloxacillin, cause hepatotoxic reactions at rates that make them visible in general practice (cases are often isolated, may have a delayed onset, sometimes appear only after cessation... (truncated)

MESH:C043114 - fosphenytoin

Summary:

LLM Prediction Score	0.673 (normalized)
LLM Confidence Score	0.980
Golden Answer (Severity Class)	1.0 (normalized)
Prediction Error	0.327

Retrieved Context:

Title: Antiepileptic Overdose.
Antiepileptics include various groups of drugs that have different mechanisms of actions and adverse effects. They are often also used to treat other disorders such as psychosis, chronic pain, and migraine. The most common drugs implicated in overdose include phenytoin, sodium valproate, carbamazepine, and phenobarbital. Common signs of toxicity of these drugs are central nervous system manifestations such as altered sensorium, lethargy, ataxia, and nystagmus. Some ingestions can paradoxically precipitate seizures and even status epilepticus. Sodium valproate can cause hyperammonemic encephalopathy and cerebral edema. Carbamazepine is implicated in cardiac arrhythmias and hyponatremia. Phenobarbital causes sedation, respiratory depression, and hypotension. In... (truncated)

Title: Data on novel DNA methylation changes induced by valproic acid in human hepatocytes.
Valproic acid (VPA) is a widely prescribed antiepileptic drug in the world. Despite its pharmacological importance, it may cause liver toxicity and steatosis. However the exact mechanism of the steatosis formation is unknown. The data presented in this DIB publication is used to further investigate the VPA-induced mechanisms of steatosis by analyzing changes in patterns of methylation. Therefore, primary human hepatocytes (PHHs) were exposed to VPA at a concentration which was shown to cause steatosis without inducing overt cytotoxicity. VPA was administered for 5 days daily to PHHs. Furthermore, after 5 days VPA-treatment parts of the PHHs were followed for... (truncated)

Title: Probable Anticonvulsant Hypersensitivity Syndrome Due to Fosphenytoin in a Pediatric Patient with Streptococcus pneumoniae Meningitis.
An 8-year-old previously healthy girl with Streptococcus pneumoniae meningitis developed probable anticonvulsant hypersensitivity syndrome (AHS) within 5 days of starting fosphenytoin. She experienced fever, rash, periorbital edema, profound hepatotoxicity and coagulopathy. Her sudden and dramatic rise in aspartate aminotransferase (AST) and alanine aminotransferase (ALT) to greater than 80 times the upper limit of normal in combination with an elevated INR were very concerning. Mortality from AHS has been correlated with the degree of hepatic involvement. Fosphenytoin was immediately discontinued and, within 48 hours, AST, ALT and INR began to decrease and were within normal limits by hospital day 23. Prompt... (truncated)

Title: Anticonvulsant hypersensitivity syndrome after phenytoin administration in an adolescent patient: a case report and review of literature.
Hypersensitivity is a rare adverse drug reaction (ADR) associated with anti-epileptic medications. Phenytoin is one of the commonly used drugs for treatment of epilepsy that encounters a hypersensitivity reaction. This reaction can be ranged from mild cutaneous rash to anticonvulsant hypersensitivity syndrome (AHS) or drug reaction with eosinophilia and systemic symptoms (DRESS) that includes fever, rash, eosinophilia and involvement of multiple internal organs.

Title: Changes in Real-world Practice Patterns of Antiepileptic Drugs for Status Epilepticus: A Nationwide Observational Study in Japan.
Intravenous (i.v.) phenytoin/fosphenytoin is recommended as the second-line therapy of antiepileptic drugs in patients with status epilepticus (SE). i.v. Levetiracetam is regarded as an effective and safe equivalent with i.v. phenytoin/fosphenytoin. However, i.v. levetiracetam is not covered by public health insurance for SE in most countries. For this study, we performed the real-world practice pattern survey of antiepileptic drugs for status epilepticus using the nationwide inpatient database. We used the Japanese Diagnosis Procedure Combination inpatient database in Japan and identified all cases of emergency admission attributable to status epilepticus from March 2011 through March 2018. We described the patient characteristics... (truncated)

MESH:D017828 - rifabutin

Summary:

LLM Prediction Score	0.300 (normalized)
LLM Confidence Score	0.980
Golden Answer (Severity Class)	0.625 (normalized)
Prediction Error	0.325

Retrieved Context:

Title: Adverse events associated with high-dose rifabutin in macrolide-containing regimens for the treatment of Mycobacterium avium complex lung disease.
We initiated a multidrug trial that included high-dose rifabutin for the treatment of pulmonary Mycobacterium avium complex (MAC) disease. Twenty-six patients received rifabutin (600 mg/d) in combination with ethambutol, streptomycin, and either clarithromycin (500 mg b.i.d.; 15 patients) or azithromycin (600 mg/d; 11 patients). Rifabutin-related adverse events occurred in 77% of patients. Fifty-eight percent of patients required a dosage adjustment or discontinuance of rifabutin therapy. The most common adverse event was a reduction in the mean total white blood cell (WBC) count, which decreased from 8,600 +/- 2,800/mm3 before treatment to 4,500 +/- 2,100/mm3 during treatment (P = .0001). Although... (truncated)

Title: Unexpected Hepatotoxicity of Rifampin and Saquinavir/Ritonavir in Healthy Male Volunteers.
OBJECTIVES: Rifampin is a potent inducer of the cytochrome P450 3A4 isoenzyme (CYP3A4) that metabolizes most protease inhibitor (PI) antiretrovirals. This study was designed to evaluate the steady-state pharmacokinetics and tolerability of the coadministration of the PIs saquinavir and ritonavir (a CYP3A4 inhibitor used as a pharmacoenhancer of other PIs) and rifampin when coadministered in healthy HIV-negative volunteers. METHODS: In an open-label, randomized, one sequence, two-period crossover study involving 28 healthy HIV-negative volunteers, arm 1 was randomized to receive saquinavir/ritonavir 1000/100 mg twice daily while arm 2 received rifampin 600 mg once daily for 14 days. Both arms were then... (truncated)

Title: Does cytochrome P450 liver isoenzyme induction increase the risk of liver toxicity after paracetamol overdose?
Paracetamol (acetaminophen, N-acetyl-p-aminophenol, 4-hydroxyacetanilide) is the most common cause of acute liver failure in developed countries. There are a number of factors which potentially impact on the risk of an individual developing hepatotoxicity following an acute paracetamol overdose. These include the dose of paracetamol ingested, time to presentation, decreased liver glutathione, and induction of cytochrome P450 (CYP) isoenzymes responsible for the metabolism of paracetamol to its toxic metabolite N-acetyl-p-benzoquinoneimine (NAPQI). In this paper, we review the currently published literature to determine whether induction of relevant CYP isoenzymes is a risk factor for hepatotoxicity in patients with acute paracetamol overdose. Animal... (truncated)

Title: Global access of rifabutin for the treatment of tuberculosis - why should we prioritize this?
Rifabutin, a rifamycin of equivalent potency to rifampicin, has several advantages in its pharmacokinetic and toxicity profile, particularly in HIV co-infected patients on combined antiretroviral therapy (cART). In this commentary, we evaluate evidence supporting increased global use of rifabutin and highlight key recommendations for action.

Title: Targeting Xenobiotic Nuclear Receptors PXR and CAR to Prevent Cobicistat Hepatotoxicity.
Liver-related diseases including drug-induced liver injury are becoming increasingly prominent in AIDS patients. Cobicistat (COBI) is the backbone of multiple regimens for antiretroviral therapy. The current work investigated the mechanisms of adverse drug-drug interactions associated with COBI that lead to liver damage. For individuals co-infected with HIV and tuberculosis (TB), the World Health Organization recommends the initiation of TB treatment followed by antiretroviral therapy. Rifampicin (RIF), a first line anti-TB drug, is a human specific activator of pregnane X receptor (PXR). Using PXR-humanized mice, we found that RIF-mediated PXR activation potentiates COBI hepatotoxicity. In contrast, rifabutin, a PXR-neutral analog of... (truncated)

MESH:D000077209 - decitabine

Summary:

LLM Prediction Score	0.177 (normalized)
LLM Confidence Score	0.980
Golden Answer (Severity Class)	0.5 (normalized)
Prediction Error	0.323

Retrieved Context:

Title: A prospective, multicenter study of low dose decitabine in adult patients with refractory immune thrombocytopenia.
We conducted a prospective, multicenter study to evaluate the efficacy and safety of low-dose decitabine in adult patients with refractory immune thrombocytopenia. Adult patients who did not respond to, did not tolerate, or were unwilling to undergo splenectomy, with either a baseline platelet count less than 30 × 10⁹ /L or the presence of bleeding symptoms and further need of ITP-specific treatments, were enrolled. Patients received decitabine at 3.5 mg/m² intravenously for three consecutive days per cycle, for three cycles with a four-week interval between cycles. All patients were assessed every week during the first 12 weeks and at four-week intervals thereafter. We... (truncated)

Title: Long-Term Follow-Up of Elderly Patients with Acute Myeloid Leukemia Treated with Decitabine: A Real-World Study of the Apulian Hematological Network.
Decitabine, a DNA hypomethylating agent, was approved for use in adults with acute myeloid leukemia (AML) not eligible for standard chemotherapy and is now widely accepted as standard treatment. Although a number of clinical trials demonstrated its benefits in elderly AML patients, older adults and patients with frequent comorbidities are typically under-represented in such settings. Thus, the aim of the present study is to evaluate, in a real-world setting, the effectiveness and toxicity of decitabine administered as a single agent in unselected previously untreated elderly AML patients not eligible for intensive chemotherapy. In nine hematological departments of the Apulian Hematological... (truncated)

Title: [The safety of decitabine as bridging pretreatment regimen before hematopoietic stem cell transplantation in pediatric hematological malignancies].
The safety of decitabine as bridging treatment before allogeneic hematopoietic stem cell transplantation (allo-HSCT) in children with refractory hematological malignancies was evaluated. All 11 cases succeeded in hematopoietic reconstitution. The main adverse reaction was hematological toxicity. Neither did infections occur, nor drug-induced liver damage and renal impairment during decitabine administration. Most cases showed grade I-IIgastrointestinal adverse events. One case was diagnosed as severe acute graft versus host disease and died of intracranial hemorrhage on day 61 after allo-HSCT. The other 10 patients survived. Decitabine bridge is a safe regimen before allo-HSCT in children with refractory hematological malignancies.

Title: Safety and clinical activity of 5-aza-2'-deoxycytidine (decitabine) with or without Hyper-CVAD in relapsed/refractory acute lymphocytic leukaemia.
To test the safety and activity of 5-aza-2'-deoxycytidine (decitabine) in patients with relapsed/refractory acute lymphocytic leukaemia (ALL), we conducted a phase 1 study with two parts: administering decitabine alone or in combination with Hyper-CVAD (fractionated cyclophosphamide, vincristine, doxorubicin and dexamethasone alternating with high-dose methotrexate and cytarabine). Patients participated in either part of the study or in both parts sequentially. In the initial part, decitabine was administered intravenously at doses of 10-120 mg/m(2) per d for 5 d every other week in cycles of 28 d. In the combination part, patients were treated on the first 5 d of Hyper-CVAD with... (truncated)

Title: A Perspective on the Comparative Antileukemic Activity of 5-Aza-2'-deoxycytidine (Decitabine) and 5-Azacytidine (Vidaza).
5-Aza-2'-deoxycytidine (5-AZA-CdR, decitabine, Dacogen®) and 5-azacytidine (5-AC, Vidaza®) are epigenetic agents that have been approved for the clinical treatment of the hematological malignancy myelodysplastic syndrome (MDS) and are currently under clinical evaluation for the treatment of acute myeloid leukemia (AML). Most investigators currently classify 5-AZA-CdR and 5-AC as inhibitors of DNA methylation, which can reactivate tumor suppressor genes silenced by this epigenetic event. Examination of the pharmacology of these analogues reveals important differences with respect to their molecular mechanism of action. The action of 5-AZA-CdR is due to its incorporation into DNA. 5-AC is a riboside

MESH:D000077208 - remifentanil

Summary:

LLM Prediction Score	0.056 (normalized)
LLM Confidence Score	0.990
Golden Answer (Severity Class)	0.375 (normalized)
Prediction Error	0.319

Retrieved Context:

Title: Effects of dexmedetomidine on perioperative stress response, inflammation and immune function in patients with different degrees of liver cirrhosis.
Effects of dexmedetomidine (DEX) on perioperative stress response, inflammation and immune function in patients with different degrees of liver cirrhosis were investigated. A total of 94 patients with liver cirrhosis who were admitted to the Affiliated Hospital of Shandong University of Traditional Chinese Medicine from December 2016 to November 2017 were included, and randomly divided into control and observation group (n=47). Patients in control group were given remifentanil for anesthesia, while patients in observation group were treated with remifentanil and for DEX anesthesia. Venous blood was collected immediately before induction of anesthesia (T1), 10 min (T2) after the beginning of... (truncated)

Title: Remifentanil Preconditioning Attenuates Hepatic Ischemia-Reperfusion Injury in Rats via Neuronal Activation in Dorsal Vagal Complex.
Remifentanil, an ultra-short acting opiate, has been reported to protect against hepatic ischemia-reperfusion injury, which is a major cause of postoperative liver dysfunction. The objective of this study was to determine whether a central vagal pathway is involved in this protective procedure. Rat models of hepatic ischemia-reperfusion were used in the experimental procedures. The results revealed that intravenous pretreatment with remifentanil decreased serum aminotransferases and hepatic histologic damage; however, an intraperitoneal injection of <i>μ</i>-opioid receptor antagonist did not abolish the protection of remifentanil preconditioning. c-Fos immunofluorescence of the brain stem showed that dorsal motor nucleus of the vagus was activated... (truncated)

Title: Effects of remifentanil on hemodynamics, liver function and ICAM-1 expression in liver cancer patients undergoing surgery.
The objective of the present study was to investigate the effects of remifentanil on hemodynamics, liver function, and expression of intercellular adhesion molecule-1 (ICAM-1) in patients with liver cancer undergoing surgery. A total of 60 patients who underwent liver cancer resection in The First People's Hospital of Xiangyang, Hubei University of Medicine from January 2014 to January 2016 were selected, including 33 males and 27 females, with an average age of 54.12±4.77 years. Patients were randomly divided into the control group and experimental group (n=30 each). The control group and experimental group were anesthetized with propofol/isoflurane and remifentanil/propofol, respectively. In... (truncated)

Title: Pharmacokinetics and pharmacodynamics of remifentanil in volunteer subjects with severe liver disease.
Remifentanil, a new mu-opioid agonist with an extremely short duration of action, is metabolized by circulating and tissue esterases; therefore, its clearance should be relatively unaffected by changes in hepatic or renal function. This study was designed to determine whether severe hepatic disease affects the pharmacokinetics or pharmacodynamics of remifentanil.

Title: The influence of the severity of chronic virus-related liver disease on propofol requirements during propofol-remifentanil anesthesia.
The purpose of this study was to investigate the influence of chronic virus- related liver disease severity on propofol requirements.

MESH:D004206 - disopyramide

Summary:

LLM Prediction Score	0.569 (normalized)
LLM Confidence Score	0.920
Golden Answer (Severity Class)	0.25 (normalized)
Prediction Error	0.319

Retrieved Context:

Title: Poisoning due to class IA antiarrhythmic drugs. Quinidine, procainamide and disopyramide.
Quinidine, procainamide and disopyramide are antiarrhythmic drugs in the class 1A category. These drugs have a low toxic to therapeutic ratio, and their use is associated with a number of serious adverse effects during long term therapy and life-threatening sequelae following acute overdose. Class 1A agents inhibit the fast inward sodium current and decrease the maximum rate of rise and amplitude of the cardiac action potential. Prolonged Q-T interval and, to a lesser extent, QRS duration may be observed at therapeutic concentrations of quinidine. With increasing plasma concentrations, progressive depression of automaticity and conduction velocity occur. 'Quinidine syncope' (a transient... (truncated)

Title: Acute cardiac failure and hepatic ischemia induced by disopyramide phosphate.
Two patients abruptly developed congestive heart failure and elevation in serum transaminase levels when given disopyramide phosphate; enzyme abnormalities and hemodynamic status corrected upon withdrawal of the drug. Both patients had underlying ischemic cardiomyopathy. Myocardial infarction, pulmonary embolism, and viral hepatitis were ruled out in both patients. One patient had a liver biopsy documenting central hepatic necrosis with congestion, consistent with hepatic ischemia and not toxic hepatitis. In the other patient, cardiac decompensation and hepatocellular enzyme elevation were reproduced on rechallenge with the drug. Disopyramide should be used with caution in patients with heart failure.

Title: Disopyramide hepatotoxicity and disseminated intravascular coagulation.
A 55-year-old white woman had severe hepatocellular damage after taking disopyramide. Simultaneously, disseminated intravascular coagulation developed with thrombocytopenia, prolonged prothrombin time, and elevated fibrin split products. Both problems gradually subsided 14 days after cessation of disopyramide therapy. This case report shows that disopyramide can have the serious side effect of hepatocellular toxicity and provides evidence of the association of hepatocellular damage and disseminated intravascular coagulation.

Title: Antiarrhythmic effects of beta-adrenergic blocking agents in benign or potentially lethal ventricular arrhythmias.
Classification of ventricular arrhythmias into those that are benign, potentially lethal and lethal is based on their associated risk for producing sudden cardiac death. This classification system is useful in defining indications for the treatment of ventricular arrhythmias and predicting differential rates of antiarrhythmic drug efficacy and toxicity. Whether the reduction of potentially lethal ventricular arrhythmias will prevent sudden cardiac death remains to be determined. The class II antiarrhythmic agents--the beta-adrenergic blocking drugs--have been shown to reduce sudden cardiac death in postmyocardial infarction patients, but the precise mechanism of their effect has not been defined. beta blockers are efficacious in... (truncated)

Title: Drug-induced hypoglycemia. A review of 1418 cases.
The present review catalogues 1418 reported cases of drug-induced hypoglycemia. The main findings are that sulfonylureas (especially chlorpropamide and glyburide), either alone or with a second hypoglycemic or potentiating agent, still account for 63% of all cases; that alcohol, propranolol, and salicylate, either singly or with another hypoglycemic drug, are the next most frequent offenders (19% of the total); and that one older drug (quinine) and three new ones (pentamidine, ritodrine, and disopyramide) have caused an additional 7% of all episodes of severe hypoglycemia. The clinical factors that set the stage for drug-induced hypoglycemia are still restricted food intake, age,... (truncated)

MESH:D000198 - spectinomycin

Summary:

LLM Prediction Score	0.057 (normalized)
LLM Confidence Score	0.960
Golden Answer (Severity Class)	0.375 (normalized)
Prediction Error	0.318

Retrieved Context:

Title: Relationship between the treatment and the evolution of the clinical course in scouring Merino lambs from "La Serena" (Southwest Spain).
This work investigated the link between the type of treatment and the clinical evolution of lambs suffering from diarrhoea attributed to non-enterotoxigenic <i>Escherichia coli</i>. Two hundred and forty scouring lambs, and 25 healthy lambs selected as control, were used in this trial. The faecal samples from the scouring lambs were positive to non-enterotoxigenic <i>E. coli</i>. All the scouring lambs received supportive care and they were randomly allotted to two groups of 120 animals (treated group and untreated group). The lambs in the treated group were given two daily doses of 20 mg/kg live weight spectinomycin for 3 days, while the other... (truncated)

Title: The public health issue of antibiotic residues in food and feed: Causes, consequences, and potential solutions.
Antibiotics are among the essential veterinary medicine compounds associated with animal feed and food animal production. The use of antibiotics for the treatment of bacterial infections is almost unavoidable, with less need to demonstrate their importance. Although banned as a growth factor for a few years, their use in animals can add residues in foodstuffs, presenting several environmental, technological, animal health, and consumer health risks. With regard to human health risks, antibiotic residues induce and accelerate antibiotic resistance development, promote the transfer of antibiotic-resistant bacteria to humans, cause allergies (penicillin), and induce other severe pathologies, such as cancers (sulfamethazine, oxytetracycline,... (truncated)

Title: Prediction of liver injury induced by chemicals in human with a multiparametric assay on isolated mouse liver mitochondria.
Drug-induced liver injury (DILI) in humans is difficult to predict using classical in vitro cytotoxicity screening and regulatory animal studies. This explains why numerous compounds are stopped during clinical trials or withdrawn from the market due to hepatotoxicity. Thus, it is important to improve early prediction of DILI in human. In this study, we hypothesized that this goal could be achieved by investigating drug-induced mitochondrial dysfunction as this toxic effect is a major mechanism of DILI. To this end, we developed a high-throughput screening platform using isolated mouse liver mitochondria. Our broad spectrum multiparametric assay was designed to detect the... (truncated)

Title: Diagnostic and therapeutic strategies for non-alcoholic fatty liver disease.
The global incidence rate of non-alcoholic fatty liver disease (NAFLD) is approximately 25%. With the global increase in obesity and its associated metabolic syndromes, NAFLD has become an important cause of chronic liver disease in many countries. Despite recent advances in pathogenesis, diagnosis, and therapeutics, there are still challenges in its treatment. In this review, we briefly describe diagnostic methods, therapeutic targets, and drugs related to NAFLD. In particular, we focus on evaluating carbohydrate and lipid metabolism, lipotoxicity, cell death, inflammation, and fibrosis as potential therapeutic targets for NAFLD. We also summarized the clinical research progress in terms of drug... (truncated)

Title: Influence of lincomycin-spectinomycin treatment on the outcome of Enterococcus cecorum infection and on the cecal microbiota in broilers.
Enterococcus cecorum (EC) is one of the main reasons for skeletal disease in meat type chickens. Intervention strategies are still rare and focus mainly on early antibiotic treatment of the disease, although there are no data available concerning the effectivity of this procedure. The present study aimed to investigate the effectivity of early lincomycin-spectinomycin treatment during the first week of life after EC-infection. Furthermore, the impact of lincomycin-spectinomycin treatment and EC infection on the development of cecal microbiota was investigated.

MESH:D011739 - pyrimethamine

Summary:

LLM Prediction Score	0.692 (normalized)
LLM Confidence Score	0.980
Golden Answer (Severity Class)	0.375 (normalized)
Prediction Error	0.317

Retrieved Context:

Title: Pyrimethamine-clarithromycin combination for therapy of acute Toxoplasma encephalitis in patients with AIDS.
Clarithromycin, a new macrolide, is effective in treating experimental Toxoplasma gondii infection. A pyrimethamine-clarithromycin combination was evaluated for the treatment of acute Toxoplasma encephalitis in 13 AIDS patients. The scheduled regimen was 2 g of clarithromycin per day and 75 mg of pyrimethamine per day for 6 weeks. The protocol was completed in eight patients and stopped in five patients (because of voluntary withdrawal by two patients, deterioration of neurological condition and thrombocytopenia in two patients, and suspicion of liver toxicity in one patient). The clinical and computed tomography scan responses at week 6 of treatment were 80 and 50%,... (truncated)

Title: Treatment of servere psoriasis with pyrimethamine.
Pyrimethamine, a folate antagonist similar to methrotrexate without known hepatotoxcity in man, was administer orally, once a week, to seven patients with severe, long-standing psoriasis. Four patients obtained a good to excellent response; a fifth withdrew from the study early after a partial response; a sixth had minimal response to high doses, and the seventh was withdrawn due to a probable drug-induced hepatotoxic reaction. Hepatotoxic reaction was suspected in another patient with abnormal liver biopsy and experienced severe gastrointestinal toxic reactions and two developed significant thrombocytopenia, which resolved after oral treatment with folic acid or discontinuation of the drug. These... (truncated)

Title: A study of toxicity and differential gene expression in murine liver following exposure to anti-malarial drugs: amodiaquine and sulphadoxine-pyrimethamine.
Amodiaquine (AQ) along with sulphadoxine-pyrimethamine (SP) offers effective and cheaper treatment against chloroquine-resistant falciparum malaria in many parts of sub-Saharan Africa. Considering the previous history of hepatitis, agranulocytosis and neutrocytopenia associated with AQ monotherapy, it becomes imperative to study the toxicity of co-administration of AQ and SP. In this study, toxicity and resulting global differential gene expression was analyzed following exposure to these drugs in experimental Swiss mice.

Title: Fatal hepatic necrosis due to pyrimethamine-sulfadoxine (Fansidar).
Pyrimethamine-sulfadoxine has been associated with severe and fatal cutaneous reactions as well as transient liver damage. We report the case of a patient who died of progressive hepatic failure caused by pyrimethamine-sulfadoxine administration. In addition, we summarize reports made to the Food and Drug Administration since 1982 that focus on hepatotoxic reactions to pyrimethamine-sulfadoxine. We suggest that fatal hepatic injury can occur after treatment with pyrimethamine-sulfadoxine and that physicians who prescribe the drug should be aware of this possibility.

Title: [Acute hepatitis following administration of fansidar].
Since 1971 pyrimethamine-sulfadoxine (Fansidar, Roche) has been used worldwide for prophylaxis and therapy of chloroquine resistant Plasmodium falciparum malaria. The drug monitoring team of the producing firm has received reports of a number of cutaneous adverse reactions, some severe, and a few even with fatal outcome. Liver reactions were also encountered, with severe cases only in the recent literature. We report on two patients with hepatitis in temporal relationship to pyrimethamine-sulfadoxine, the first with a second event after later exposure to the same drug. After discontinuing the medication the liver function abnormalities returned to normal limits within a few weeks.... (truncated)

MESH:D005045 - etomidate

Summary:

LLM Prediction Score	0.317 (normalized)
LLM Confidence Score	0.990
Golden Answer (Severity Class)	0.0 (normalized)
Prediction Error	0.317

Retrieved Context:

Title: Therapy of Cushing's syndrome with steroid biosynthesis inhibitors.
Several substances with different inhibitory effects on adrenal steroid biosynthesis were investigated in patients with Cushing's syndrome. It has been shown that trilostane, a 3 beta-hydroxysteroid-dehydrogenase inhibitor, is not potent enough to block cortisol biosynthesis in patients with hypercortisolism. Aminoglutethimide inhibits side chain cleavage of cortisol synthesis, but it has been demonstrated that the blocking effect on cortisol secretion is not strong enough to normalize urinary cortisol excretion in patients with Cushing's disease. For metyrapone, an inhibitor of adrenal 11 beta-hydroxylase, promising results were reported for the treatment of Cushing's syndrome. However, the drug has several side effects and depending... (truncated)

Title: [Anesthesia and hepatic porphyria].
Three of the acute hepatic porphyrias, acute intermittent porphyria, variegata porphyria and hereditary coproporphyria, are characterized by an idiosyncratic reaction to many common drugs; the resulting excessive excretion of porphyrin precursors is responsible for episodes of acute neurological dysfunction. This review aimed to focus the attention of the anaesthesiologist on the porphyrinogenic properties of all the drugs used in anaesthesia and intensive care. An outline of the chemistry of porphyrins and the enzymatic pathways were recalled, so as to place the acute porphyrias in their proper perspective. There follows a reminder of the clinical aspect of acute porphyric crises. The... (truncated)

Title: Pharmacological management of severe Cushing's syndrome: the role of etomidate.
Cushing's syndrome (CS) is an endocrine disease characterized by excessive adrenocortical steroid production. One of the mainstay pharmacological treatments for CS are steroidogenesis enzyme inhibitors, including the antifungal agent ketoconazole along with metyrapone, mitotane, and aminoglutethimide. Recently, osilodrostat was added to this drug class and approved by the US Food and Drug Administration (FDA) for the treatment of Cushing's Disease. Steroidogenesis enzyme inhibitors inhibit various enzymes along the cortisol biosynthetic pathway and may be used preoperatively to lower cortisol levels and reduce surgical risk associated with tumor resection or postoperatively when surgery and/or radiation therapies are not curative. Because their... (truncated)

Title: Polymorphisms of pharmacogenetic candidate genes affect etomidate anesthesia susceptibility.
Purpose: Etomidate is widely used in general anesthesia and sedation, and significant individual differences are observed during anesthesia induction. This study aimed to explore the molecular mechanisms of different etomidate susceptibility at the genetic level. Methods: 128 patients were enrolled in the study. The bispectral index (BIS), mean arterial pressure (MAP) and heart rate (HR) were recorded when the patients entered the operating room for 5 min, before the administration of etomidate, 30 s, 60 s, 90 s, 120 s and 150 s after the administration of etomidate, and the corresponding single nucleotide polymorphisms (SNPs) were analyzed. Results: Significant individual differences were observed in etomidate anesthesia.... (truncated)

Title: Liver enzyme studies with continuous intravenous anaesthesia.
A battery of liver function tests was carried out before operation and on the 3rd--5th and 13th--15th postoperative days in patients anaesthetised with continuous infusion of thiopentone, Althesin or etomidate for an intermediate operation. Some derangement of enzyme activity was found in one quarter to one third of the patients, and was most marked after Althesin. The findings are compared with published data on ketamine, which had an effect on enzyme activities similar to that of Althesin. On pooling data from different studies it became very apparent that large doses of intravenous anaesthetics cause a greater derangement of liver function... (truncated)

MESH:D000069349 - linezolid

Summary:

LLM Prediction Score	0.691 (normalized)
LLM Confidence Score	0.990
Golden Answer (Severity Class)	0.375 (normalized)
Prediction Error	0.316

Retrieved Context:

Title: Severe drug-induced liver injury associated with prolonged use of linezolid.
This study aims to describe a patient developing concomitant severe liver failure and lactic acidosis after long-term treatment with linezolid. A 55-year-old Caucasian woman developed concomitant severe liver failure and lactic acidosis after a treatment with linezolid for 50 days because of infected hip prosthesis. Other causes of liver failure and lactic acidosis were excluded by extensive diagnostic workup. A liver biopsy showed microvesicular steatosis. As linezolid toxicity was considered to be the cause of the lactic acidosis and the severe hepatic failure, the antibiotic was withdrawn. After 4 days of supportive therapy and hemodialysis, the serum lactate level returned... (truncated)

Title: Linezolid use and drug-induced liver injury.
Linezolid is a frequently prescribed antibiotic for chronic infection suppression, such as in prosthetic joint infections. While well tolerated, its prolonged use can increase the risk of rare and serious side effects. We present a case of linezolid toxicity presenting with a constellation of adverse effects including bone marrow suppression, lactic acidosis, and drug-induced liver injury. Our case highlights the increased risk of acute multiorgan failure in patients using the antibiotic for extended durations, notably those with preexisting comorbidities.

Title: Linezolid-induced pure red cell aplasia: a case report and literature review.
Linezolid (LZD) is the first oxazolidinone with excellent safety and efficacy profiles against refractory infections caused by gram-positive organisms. Hematological toxicities such as thrombocytopenia, anemia, and leukocytopenia are common in LZD therapy; however, LZD-induced pure red cell aplasia (PRCA) is rare. An 83-year-old man diagnosed with pleural empyema caused by Staphylococcus aureus received LZD after developing resistance to multiple antibiotics. Although his infection-related symptoms were improved by LZD, progressive anemia was noticed after LZD therapy was initiated. Eight weeks after LZD administration began, his hemoglobin level was 5.7 g/dL and reticulocyte proportion was 0.36%, while his white blood cell and... (truncated)

Title: Linezolid-associated acute interstitial nephritis and drug rash with eosinophilia and systemic symptoms (DRESS) syndrome.
Linezolid is a recent addition to the antibiotic armamentarium against Gram-positive bacteria, including multiresistant staphylococci and enterococci. Linezolid is relatively well tolerated and is not believed to be nephrotoxic. However, we report the case of an 88-year-old woman who was treated for prosthetic joint infection and methicillin-resistant Staphylococcus aureus bacteremia with vancomycin followed by linezolid therapy. On day 7 of linezolid treatment, the patient developed severe pruritus, macular rash, facial edema, eosinophilia, marked increase in serum creatinine level, and mild hepatitis. Renal biopsy showed acute interstitial nephritis with eosinophilic cells. Discontinuation of linezolid and a short course of prednisone led... (truncated)

Title: Concise Clinical Review of Hematologic Toxicity of Linezolid in Multidrug-Resistant and Extensively Drug-Resistant Tuberculosis: Role of Mitochondria.
Multidrug-resistant tuberculosis (MDR-TB) is caused by an organism that is resistant to both rifampicin and isoniazid. Extensively drug-resistant TB, a rare type of MDR-TB, is caused by an organism that is resistant to quinolone and one of group A TB drugs (i.e., linezolid and bedaquiline). In 2018, the World Health Organization revised the groupings of TB medicines and reclassified linezolid as a group A drug for the treatment of MDR-TB. Linezolid is a synthetic antimicrobial agent in the oxazolidinone class. Although linezolid has a good efficacy, it can cause substantial adverse events, especially hematologic toxicity. In both TB infection and... (truncated)

MESH:D012299 - rimantadine

Summary:

LLM Prediction Score	0.315 (normalized)
LLM Confidence Score	0.970
Golden Answer (Severity Class)	0.0 (normalized)
Prediction Error	0.315

Retrieved Context:

Title: Compounds with anti-influenza activity: present and future of strategies for the optimal treatment and management of influenza. Part I: Influenza life-cycle and currently available drugs.
Influenza is a contagious respiratory acute viral disease characterized by a short incubation period, high fever and respiratory and systemic symptoms. The burden of influenza is very heavy. Indeed, the World Health Organization (WHO) estimates that annual epidemics affect 5-15% of the world's population, causing up to 4-5 million severe cases and from 250,000 to 500,000 deaths. In order to design anti-influenza molecules and compounds, it is important to understand the complex replication cycle of the influenza virus. Replication is achieved through various stages. First, the virus must engage the sialic acid receptors present on the free surface of the... (truncated)

Title: Inhibitor Development against p7 Channel in Hepatitis C Virus.
Hepatitis C Virus (HCV) is the key cause of chronic and severe liver diseases. The recent direct-acting antiviral agents have shown the clinical success on HCV-related diseases, but the rapid HCV mutations of the virus highlight the sustaining necessity to develop new drugs. p7, the viroporin protein from HCV, has been sought after as a potential anti-HCV drug target. Several classes of compounds, such as amantadine and rimantadine have been testified for p7 inhibition. However, the efficacies of these compounds are not high. Here, we screened some novel p7 inhibitors with amantadine scaffold for the inhibitor development. The dissociation constant... (truncated)

Title: Relevance of Liver Failure for Anti-Infective Agents: From Pharmacokinetic Alterations to Dosage Adjustments.
The liver is a complex organ with great ability to influence drug pharmacokinetics. Due to its wide array of function, its impairment has the potential to affect bioavailability, enterohepatic circulation, drug distribution, metabolism, clearance, and biliary elimination. These alterations differ widely depending on the cause of the liver failure, if it is acute or chronic in nature, the extent of impairment, and comorbid conditions. In addition, effects on liver functions do not occur in a proportional or predictable manner for escalating degrees of liver impairment. The ability of hepatic alterations to influence PK is also dependent on drug characteristics, such... (truncated)

Title: A randomized, crossover study to evaluate the pharmacokinetics of amantadine and oseltamivir administered alone and in combination.
The threat of potential pandemic influenza requires a reevaluation of licensed therapies for the prophylaxis or treatment of avian H5N1 infection that may adapt to man. Among the therapies considered for use in pandemic influenza is the co-administration of ion channel and neuraminidase inhibitors, both to potentially increase efficacy as well as to decrease the emergence of resistant isolates. To better understand the potential for drug interactions, a cross-over, randomized, open-label trial was conducted with amantadine, 100 mg po bid, and oseltamivir, 75 mg po bid, given alone or in combination for 5 days. Each subject (N = 17) served... (truncated)

Title: Anti-infective Properties of the Golden Spice Curcumin.
The search for novel anti-infectives is one of the most important challenges in natural product research, as diseases caused by bacteria, viruses, and fungi are influencing the human society all over the world. Natural compounds are a continuing source of novel anti-infectives. Accordingly, curcumin, has been used for centuries in Asian traditional medicine to treat various disorders. Numerous studies have shown that curcumin possesses a wide spectrum of biological and pharmacological properties, acting, for example, as anti-inflammatory, anti-angiogenic and anti-neoplastic, while no toxicity is associated with the compound. Recently, curcumin's antiviral and antibacterial activity was investigated, and it was shown... (truncated)

MESH:D002747 - chlorpropamide

Summary:

LLM Prediction Score	0.562 (normalized)
LLM Confidence Score	0.930
Golden Answer (Severity Class)	0.25 (normalized)
Prediction Error	0.312

Retrieved Context:

Title: A case of chronic liver disease due to tolazamide.
Although chlorpropamide and tolbutamide are well recognized as causes of hepatotoxicity, there are only 3 reported cases of hepatic injury caused by a third oral hypoglycemic agent, tolazamide. In 2 of these cases, the liver-function tests returned to normal when the drug was discontinued. In the third case, the patient had cholestasis from chlorpropamide before administration of tolazamide and developed chronic liver disease. We are reporting the second instance of chronic liver disease induced by tolazamide. Our patient had been taking chlorpropamide, but she had no evidence of liver disease before administration of tolazamide. Tolazamide should be considered as a... (truncated)

Title: Drug-induced acute liver disease.
Fifty-three patients with drug-induced acute liver disease are reported. There were 35 females and 18 males with a mean age of 41 years. All but one patient had jaundice or hyperbilirubinaemia and 51 had abnormal liver enzymes. Histologically 38 patients (72%) had cholestatic injury while 15 had cytotoxic parenchymal damage. Methyldopa, chlorpropamide, chlorpromazine, halothane and the contraceptive pill accounted for 60% of cases. Fifty-one patients recovered after drug withdrawal while 2 died of hepatic failure. Drug-induced liver injury must be considered in patients presenting with evidence of hepatic disease as the majority will recover on withdrawal.

Title: Granulomatous hepatitis associated with glyburide.
A wide variety of diseases and injuries can cause granulomatous hepatitis, and drug-induced granulomatous hepatitis is a well-described entity. Sulfonylurea derivatives, which are commonly used oral hypoglycemic agents in the treatment of non-insulin-dependent diabetes mellitus, have been implicated in liver disease. However, glyburide, a second-generation sulfonylurea and a potent hypoglycemic drug, is considered to have less hepatic side effects than chlorpropamide. It has been reportedly associated with cholestatic jaundice and hepatitis and with hypersensitivity angitis. A case of necrotizing granuloma has been reported. We present a second case of granulomatous hepatitis occurring in a patient who had been taking glyburide... (truncated)

Title: Chlorpropamide-induced granulomas. A probable hypersensitivity reaction in liver and bone marrow.
Anicteric hepatitis, associated with fever and exfoliative dermatitis, developed in a diabetic patient two weeks after intake of a long-acting sulfonylurea, chlorpropamide (Diabinese). Granulomas showing heavy infiltration with eosinophils were found in the liver and bone marrow. These were interpreted as manifestations of an allergic reaction. The clinical signs, abnormal laboratory findings, and hepatic lesions subsided spontaneously on withdrawal of the drug. Bone marrow changes, however, persisted seven months after cessation of the drug. To our knowledge, this is the first report of a patient with liver and bone marrow inflammation characterized by granulomas with eosinophilic infiltration following intake of... (truncated)

Title: Comparative study of glibenclamide & chlorpropamide in newly diagnosed maturity onset diabetics.
The clinical effectiveness of glibenclamide and chlorpropamide was compared in 107, uncomplicated, newly diagnosed maturity onset diabetics. The glibenclamide and chlorpropamide groups comprised of 49 and 58 patients respectively and were highly comparable. After a follow up period ranging from 6 months to 2-1/2 years, the failure rate in glibenclamide treated patients was 22.5% and 12% in those taking chlorpropamide. The changes in weight were similar and both drugs were devoid of serious toxic effects in the dosage prescribed. Symptoms of hypoglycaemia were seen in 2 patients on glibenclamide, while 3 patients in each group showed a modest rise of... (truncated)

MESH:D001310 - auranofin

Summary:

LLM Prediction Score	0.064 (normalized)
LLM Confidence Score	0.970
Golden Answer (Severity Class)	0.375 (normalized)
Prediction Error	0.311

Retrieved Context:

Title: Auranofin protects against cocaine-induced hepatic injury through induction of heme oxygenase-1.
Auranofin, a disease-modifying gold compound, has been empirically applying to the management of rheumatoid arthritis. We investigated a protective effect of auranofin against hepatic injury induced by cocaine. Cocaine (75 mg/kg) markedly increased serum alanine amino transferase (ALT) (4,130 IU/l) and aspartate amino transferase (AST) (1,730 IU/l) activities at 16 hr after treatment, and induced hepatic necrosis surrounding central veins in mice. Concurrently, overexpression of heme oxygenase-1 (HO-1), a rate-limiting enzyme for heme degradation and an oxidative stress marker, was identified at the edges of cocaine-mediated necrotic area. Auranofin (10 mg/ml, i.p.) significantly induced hepatic HO-1 protein in mice from... (truncated)

Title: Auranofin mitigates systemic iron overload and induces ferroptosis via distinct mechanisms.
Iron homeostasis is essential for health; moreover, hepcidin-deficiency results in iron overload in both hereditary hemochromatosis and iron-loading anemia. Here, we identified iron modulators by functionally screening hepcidin agonists using a library of 640 FDA-approved drugs in human hepatic Huh7 cells. We validated the results in C57BL/6J mice and a mouse model of hemochromatosis (Hfe^{-/-} mice). Our screen revealed that the anti-rheumatoid arthritis drug auranofin (AUR) potently upregulates hepcidin expression. Interestingly, we found that canonical signaling pathways that regulate iron, including the Bmp/Smad and IL-6/Jak2/Stat3 pathways, play indispensable roles in mediating AUR's effects. In addition, AUR induces IL-6 via the... (truncated)

Title: Counteraction of HCV-Induced Oxidative Stress Concurs to Establish Chronic Infection in Liver Cell Cultures.
Hepatitis C virus (HCV) is a blood-borne pathogen causing acute and chronic hepatitis. A significant number of people chronically infected with HCV develop cirrhosis and/or liver cancer. The pathophysiologic mechanisms of hepatocyte damage associated with chronic HCV infection are not fully understood yet, mainly due to the lack of an <i>in vitro</i> system able to recapitulate the stages of infection <i>in vivo</i>. Several studies underline that HCV virus replication depends on redox-sensitive cellular pathways; in addition, it is known that virus itself induces alterations of the cellular redox state. However, the exact interplay between HCV replication and oxidative stress has... (truncated)

Title: Anti-fibrotic effect of aurocyanide, the active metabolite of auranofin.
Drug repositioning has gained significant attention over the past several years. The anti-rheumatoid arthritis drug auranofin has been investigated for the treatment of other diseases, including liver fibrosis. Because auranofin is rapidly metabolized, it is necessary to identify the active metabolites of auranofin that have detectable levels in the blood and reflect its therapeutic effects. In the present study, we investigated whether aurocyanide as an active metabolite of auranofin, can be used to evaluate the anti-fibrotic effects of auranofin. Incubation of auranofin with liver microsomes showed that auranofin was susceptible to hepatic metabolism. Previously, we found that the anti-fibrotic effects of auranofin... (truncated)

Title: Auranofin prevents liver fibrosis by system Xc-mediated inhibition of NLRP3 inflammasome.
Demand for a cure of liver fibrosis is rising with its increasing morbidity and mortality. Therefore, it is an urgent issue to investigate its therapeutic candidates. Liver fibrosis progresses following 'multi-hit' processes involving hepatic stellate cells, macrophages, and hepatocytes. The NOD-like receptor protein 3 (NLRP3) inflammasome is emerging as a therapeutic target in liver fibrosis. Previous studies showed that the anti-rheumatic agent auranofin inhibits the NLRP3 inflammasome; thus, this study evaluates the antifibrotic effect of auranofin in vivo and explores the underlying molecular mechanism. The antifibrotic effect of auranofin is assessed in thioacetamide- and carbon tetrachloride-induced liver fibrosis models. Moreover,... (truncated)

MESH:D017706 - lisinopril

Summary:

LLM Prediction Score	0.685 (normalized)
LLM Confidence Score	0.990
Golden Answer (Severity Class)	0.375 (normalized)
Prediction Error	0.310

Retrieved Context:

Title: Lisinopril-Induced Liver Injury: An Unusual Presentation and Literature Review.
Lisinopril is an angiotensin converting enzyme inhibitor (ACE-I) that has been on market for more than 25 years. ACE-I are usually well tolerated and rarely have serious or life-threatening side effects. We describe an unusual presentation of fulminant hepatic cholestasis probably secondary to lisinopril. To our knowledge, this is the second case report which shows lisinopril-induced liver injury though a cholestatic mechanism. The patient was a 59-year-old woman with type 2 diabetes, a high body mass index and hypertension, who presented with a 5-week history of jaundice and itching. She had been started on lisinopril for diabetic nephropathy 8 weeks... (truncated)

Title: Fulminant hepatitis after lisinopril administration.
A case of fulminant hepatitis in a patient taking lisinopril for 5 weeks for arterial hypertension is reported. Jaundice, fever, myalgia, and marked increase in serum aminotransferase activities occurred after 2 weeks of treatment. Continuation of lisinopril administration for 3 weeks after the onset of jaundice was associated with the development of grade III encephalopathy and a marked decrease in prothrombin and proaccelerin levels. This case strongly suggests that lisinopril may induce acute hepatitis and that continuation of the treatment after the onset of jaundice can lead to life-threatening hepatic failure.

Title: Chronic hepatitis caused by lisinopril.
ACE inhibitors are used world-wide for treatment of hypertension and cardiac failure; liver damage is a rare but potentially severe side-effect of these drugs. In this case report we describe a patient with chronic liver damage due to lisinopril.

Title: Unique case of presumed lisinopril-induced hepatotoxicity.
A case of presumed lisinopril-induced hepatotoxicity is reported.

Title: A case of meropenem-induced liver injury and jaundice.
This report describes what we believe is the first reported case of clinically significant cholestasis and acute liver injury within three days of meropenem therapy. An 83-year-old Hispanic female was admitted for sepsis of unknown origin and was started on intravenous meropenem. Three days following initiation of the antibiotic, the patient developed mixed hepatocellular and cholestatic liver injury with jaundice and pruritus. Possible causes of cholestasis were excluded after extensive investigations. A drug-induced liver injury was suspected and meropenem was discontinued. Following discontinuation of meropenem, the patient demonstrated symptomatic and laboratory improvements, and her liver enzymes and bilirubin levels were... (truncated)

MESH:D008620 - meprobamate

Summary:

LLM Prediction Score	0.310 (normalized)
LLM Confidence Score	0.980
Golden Answer (Severity Class)	0.0 (normalized)
Prediction Error	0.310

Retrieved Context:

Title: [Voluntary drug poisoning: epidemiology, performance and limits of the emergency laboratory].
The aim of this study is to determine the efficiency ot toxicologic screening (detection of barbiturates, benzodiazepines, tricyclic antidepressants, salicylates, phenothiazines, meprobamate and ethanol assay), during acute drug poisoning. In 1988, 898 patients are enclose in this study. Screenings are negative in 17% of cases; benzodiazepines, alcohol and antidepressants are often found. The recovery is very good for barbiturates and salicylates but it's not perfect for benzodiazepines, particularly flunitrazepam triazolam, loflazepate, oxazepam, and non tricyclic antidepressants. This failure probably depends on these emergency methods.

Title: Value of toxicological investigation in the diagnosis of acute drug poisoning in children.
In the 2 years 1978 and 1979 specimens from 287 children aged between 10 days and 14 years were received for general toxicological investigations. Of the 95 (33%) cases of confirmed poisoning, the diagnosis was established as a direct result of the analyses in 48 patients. No diagnosis was made in at least 85 (30%) of the remaining cases. Benzodiazepines were the drugs most commonly encountered (33%), followed by barbiturates, glutethimide, and meprobamate (15%), salicylate and paracetamol (15%), tricyclic antidepressants (12%), and ethanol (11%). 36 patients were severely poisoned (grade 3 or 4 coma, or convulsions), although only 1 patent... (truncated)

Title: Declinol, a Complex Containing Kudzu, Bitter Herbs (Gentian, Tangerine Peel) and Bupleurum, Significantly Reduced Alcohol Use Disorders Identification Test (AUDIT) Scores in Moderate to Heavy Drinkers: A Pilot Study.
It is well established that inherited human aldehyde dehydrogenase 2 (ALDH-2) deficiency reduces the risk for alcoholism. Kudzu plants and extracts have been used for 1,000 years in traditional Chinese medicine to treat alcoholism. Kudzu contains daidzin, which inhibits ALDH-2 and suppresses heavy drinking in rodents. Decreased drinking due to ALDH-2 inhibition is attributed to aversive properties of acetaldehyde accumulated during alcohol consumption. However not all of the anti-alcohol properties of diadzin are due to inhibition of ALDH-2. This is in agreement with our earlier work showing significant interaction effects of both pyrozole (ALDH-2 inhibitor) and methyl-pyrozole (non-inhibitor) and ethanol's... (truncated)

Title: Alcohol and medication interactions.
Many medications can interact with alcohol, thereby altering the metabolism or effects of alcohol and/or the medication. Some of these interactions can occur even at moderate drinking levels and result in adverse health effects for the drinker. Two types of alcohol-medication interactions exist: (1) pharmacokinetic interactions, in which alcohol interferes with the metabolism of the medication, and (2) pharmacodynamic interactions, in which alcohol enhances the effects of the medication, particularly in the central nervous system (e.g., sedation). Pharmacokinetic interactions generally occur in the liver, where both alcohol and many medications are metabolized, frequently by the same enzymes. Numerous classes of... (truncated)

Title: Drug disposition in pathophysiological conditions.
Expression and activity of several key drug metabolizing enzymes (DMEs) and transporters are altered in various pathophysiological conditions, leading to altered drug metabolism and disposition. This can have profound impact on the pharmacotherapy of widely used clinically relevant medications in terms of safety and efficacy by causing inter-individual variabilities in drug responses. This review article highlights altered drug disposition in inflammation and infectious diseases, and commonly encountered disorders such as cancer, obesity/diabetes, fatty liver diseases, cardiovascular diseases and rheumatoid arthritis. Many of the clinically relevant drugs have a narrow therapeutic index. Thus any changes in the disposition of these drugs... (truncated)

MESH:D018119 - stavudine

Summary:

LLM Prediction Score	0.691 (normalized)
LLM Confidence Score	0.950
Golden Answer (Severity Class)	1.0 (normalized)
Prediction Error	0.309

Retrieved Context:

Title: Hepatic steatosis and lactic acidosis caused by stavudine in an HIV-infected patient.
Lactic acidosis and hepatic steatosis caused by mitochondrial toxicity of nucleoside reverse transcriptase inhibitors (NRTI) is a rare cause of liver disease with a high mortality rate. This report describes a male, HIV-positive patient with a 4-week history of nausea, vomiting and abdominal pain. His medication consisted of prednisone 5 mg od (because of auto-immune thrombocytopenia), didanosine (for 2 years) and stavudine (for 3 months). Laboratory studies showed cholestasis and elevation of aminotransferases. Lactic level was not measured. Liver biopsy revealed steatosis and cholestatic hepatitis. In the absence of other causes of liver disease a probable diagnosis of stavudine-induced hepatic... (truncated)

Title: Metabolic acidosis and hepatic steatosis in two HIV-infected patients on stavudine (d4T) treatment.
Nucleoside analog reverse transcriptase inhibitors (NRTI) have been used to treat HIV-infected patients for >10 years. Some severe adverse events have been attributed to mitochondrial dysfunction. Since 1991, cases of severe lactic acidosis have been reported in association with nucleoside therapy. Our objective was to report two cases of metabolic acidosis and hepatic steatosis in patients receiving stavudine (d4T) and to review the literature. A male and a female, 47 and 45 years of age, respectively, presented with abdominal pain, nausea, vomiting, and weakness after 9 and 6 months, respectively, of treatment with stavudine. At presentation, both patients had severe... (truncated)

Title: Dideoxynucleoside HIV reverse transcriptase inhibitors and drug-related hepatotoxicity: a case report.
This report regards the case of a 43 year-old HIV-positive woman who developed an episode of serious transaminase elevation during stavudine-including antiretroviral therapy. Diagnostic assessment ruled out hepatitis virus co-infection, alcohol abuse besides other possible causes of liver damage. No signs of lactic acidosis were present. Liver biopsy showed portal inflammatory infiltrate, spotty necrosis, vacuoles of macro- and micro-vesicular steatosis, acidophil and foamy hepatocytes degeneration with organelles clumping, poorly formed Mallory bodies and neutrophil granulocytes attraction (satellitosis). A dramatic improvement in liver function tests occurred when stavudine was discontinued and a new antiretroviral regimen with different nucleoside reverse transcriptase inhibitors... (truncated)

Title: Drug-induced increased mitochondrial biogenesis in a liver biopsy.
Oncocytic changes seen in hepatocytes in patients receiving highly active antiretroviral therapy (HAART) are a result of mitochondrial damage. This is the first report that provides the electron microscopy illustration of mitochondrial proliferation as a result of the HAART drug Stavudine (Zerit) hepatotoxicity. The drug's effect on mitochondrial DNA replication leads to depleted mitochondrial-encoded proteins and configurational defects of the mitochondrial inner membrane leading to reduced and abnormal cristae, which house the electron transport chain and elementary bodies. This results in a decrease in the NAD/NADH ratio and reduces oxidative phosphorylation. The shift in the NAD/NADH ratio decreases the rate... (truncated)

Title: Stavudine:pharmacology,clinical use and future role.
Stavudine is a nucleoside analogue reverse transcriptase inhibitor of HIV-1 and HIV-2 and demonstrates in vitro activity with an acceptable therapeutic index in a range of T-lymphocyte and haematopoietic precursor cell lines. It is additive or synergistic in vitro with a range of other antiretrovirals, including the proteinase inhibitor saquinavir, in two- and three-way combinations and is active against zidovudine (ZDV)-resistant virus. It exhibits excellent oral bioavailability, with cerebrospinal fluid (CSF)/plasma penetration. In clinical use, stavudine monotherapy exhibits similar antiretroviral activity to ZDV, and is of proven clinical benefit in ZDV-pre-treated patients. In combination with ddI and/or nelfinavir it results... (truncated)

MESH:D002707 - chlordiazepoxide

Summary:

LLM Prediction Score	0.318 (normalized)
LLM Confidence Score	0.990
Golden Answer (Severity Class)	0.625 (normalized)
Prediction Error	0.307

Retrieved Context:

Title: Psychotropic drugs and liver disease: A critical review of pharmacokinetics and liver toxicity.
The liver is the organ by which the majority of substances are metabolized, including psychotropic drugs. There are several pharmacokinetic changes in end-stage liver disease that can interfere with the metabolization of psychotropic drugs. This fact is particularly true in drugs with extensive first-pass metabolism, highly protein bound drugs and drugs depending on phase I hepatic metabolic reactions. Psychopharmacological agents are also associated with a risk of hepatotoxicity. The evidence is insufficient for definite conclusions regarding the prevalence and severity of psychiatric drug-induced liver injury. High-risk psychotropics are not advised when there is pre-existing liver disease, and after starting a... (truncated)

Title: Granulomas and cholestatic--hepatocellular injury associated with phenylbutazone. Report of two cases.
A combined cholestatic-hepatocellular injury and noncaseating granulomas occurred in two patients 1 and 4 weeks after phenylbutazone therapy. Both patients were jaundiced, one had a macular rash, and both had peripheral blood eosinophilia. Symptoms and signs subsided, and abnormal findings from tests of hepatic function rapidly returned to normal following withdrawal of the drug. Sections of liver biopsy specimens 6 months later showed no granulomas or other pathologic changes. Previously reported cases are reviewed.

Title: Interference with bile salt export pump function is a susceptibility factor for human liver injury in drug development.
The bile salt export pump (BSEP) is an efflux transporter, driving the elimination of endobiotic and xenobiotic substrates from hepatocytes into the bile. More specifically, it is responsible for the elimination of monovalent, conjugated bile salts, with little or no assistance from other apical transporters. Disruption of BSEP activity through genetic disorders is known to manifest in clinical liver injury such as progressive familial intrahepatic cholestasis type 2. Drug-induced disruption of BSEP is hypothesized to play a role in the development of liver injury for several marketed or withdrawn therapeutics. Unfortunately, preclinical animal models have been poor predictors of the... (truncated)

Title: The effect of cimetidine on hepatic drug elimination in cirrhosis.
Both cimetidine therapy and cirrhosis individually interfere with normal elimination of various drugs. Cimetidine is often prescribed in patients with cirrhosis but there is incomplete data on its effect on drug elimination in cirrhotics. The purpose of this study was to address this issue. Eight stable cirrhotics were studied prior to and following 7 days of cimetidine administration, (300 mg orally q.i.d.). Chlordiazepoxide (Librium), which is eliminated by the liver after demethylation, and indocyanine green, which is removed by the liver without biotransformation, were used as probes. Consistent with the concept that cimetidine interferes with drug metabolism by inhibiting microsomal... (truncated)

Title: A multidisciplinary approach to the management of liver disease and alcohol disorders in psychiatric settings (Review).
Society is burdened with the uncontrolled use of alcohol, an ongoing issue, with a substantial associated morbidity and a pressing economical reverberation. It is inevitable that a series of psychiatric patients who display alcohol disorders will be admitted to hospital while also suffering from health conditions, such as liver disease, due to the consumption of alcohol. Managing comorbid patients in a psychiatric facility is a delicate matter that requires a collaborative team. The aim of this systematic paper is to highlight the following: The possibility of treating alcohol use disorder (AUD) and alcohol withdrawal syndrome (AWS) overlapping alcohol liver disease... (truncated)

MESH:D005474 - fluoxymesterone

Summary:

LLM Prediction Score	0.694 (normalized)
LLM Confidence Score	0.980
Golden Answer (Severity Class)	1.0 (normalized)
Prediction Error	0.306

Retrieved Context:

Title: Evaluation of tamoxifen dose in advanced breast cancer: a progress report.
The results of an ongoing trial randomizing patients with progressive, metastatic breast carcinoma between tamoxifen (Tam, NSC-180973) and Tam plus fluoxymesterone (Flu) (7 mg/m2 bid) are reported. Each patient received a single dose level of Tam in the range of 2-100 mg/m2 bid. The combination had a higher response rate overall (45% vs 28%) and when only the patients' soft tissue sites were analyzed (54% vs 9%, P=0.04). The time to treatment failure was longer for the combination among those patients with a response or disease stabilization (P=0.08). Response rates with Tam doses less than 12 mg/m2 bid were also... (truncated)

Title: Peliosis hepatis. Twelve cases associated with oral androgen therapy.
Peliosis hepatis was encountered in 12 patients treated with high-dose oxymetholone or fluoxymesterone therapy. In three cases liver failure was the primary cause of death. In one case, the diagnosis was established by biopsy, the androgen therapy was discontinued, and the lesion was absent at autopsy two years later. In eight cases peliosis hepatis was an incidental finding at postmortem examination. The clinical and anatomic features of these cases are described, and previously reported cases of peliosis are briefly reviewed. A hypothesis is offered to explain the association of this peculiar lesion with anabolic androgen therapy.

Title: Prospective evaluation of carcinoembryonic antigen levels and alternating chemotherapeutic regimens in metastatic breast cancer.
Ninety-seven eligible and evaluable women with metastatic breast cancer were placed on a prospective clinical protocol to evaluate the use of continuous cyclic therapy with dibromodulcitol, doxorubicin, vincristine, tamoxifen, and fluoxymesterone (DAVTH) v DAVTH alternating with cyclophosphamide, methotrexate, 5-fluorouracil, and prednisone (CMFP); and the use of pretreatment and serial carcinoembryonic antigen (CEA) levels in these patients. Continuous DAVTH and DAVTH/CMFP were equivalent therapies with respect to response rates, time to treatment failure (TTF), and survival. Pretreatment CEA levels were elevated (greater than 5 ng/mL) in 42/97 patients and less than 5 ng/mL in the remaining patients. Patients with elevated pretreatment... (truncated)

Title: Drug, Herb, and Dietary Supplement Hepatotoxicity.
The past decade has witnessed drugs, herbs, and dietary supplements share the common feature of potential liver injury in a few susceptible individuals.[...].

Title: Hepatotoxicity by Dietary Supplements: A Tabular Listing and Clinical Characteristics.
Dietary supplements (DS) are extensively consumed worldwide despite unproven efficacy. The true incidence of DS-induced liver injury (DSILI) is unknown but is probably under-diagnosed due to the general belief of safety of these products. Reported cases of herbals and DS-induced liver injury are increasing worldwide. The aim of this manuscript is to report a tabular listing with a description of DS associated with hepatotoxicity as well as review the phenotype and severity of DSILI. Natural remedies related to hepatotoxicity can be divided into herbal product-induced liver injury and DS-induced liver injury. In this article, we describe different DS associated with... (truncated)

MESH:D002065 - buspirone

Summary:

LLM Prediction Score	0.070 (normalized)
LLM Confidence Score	0.990
Golden Answer (Severity Class)	0.375 (normalized)
Prediction Error	0.305

Retrieved Context:

Title: Exploring Chronic Drug Effects on Microengineered Human Liver Cultures Using Global Gene Expression Profiling.
Global gene expression profiling is useful for elucidating a drug's mechanism of action on the liver; however, such profiling in rats is not very sensitive for predicting human drug-induced liver injury, while dedifferentiated monolayers of primary human hepatocytes (PHHs) do not permit chronic drug treatment. In contrast, micropatterned cocultures (MPCCs) containing PHH colonies and 3T3-J2 fibroblasts maintain a stable liver phenotype for 4-6 weeks. Here, we used MPCCs to test the hypothesis that global gene expression patterns in stable PHHs can be used to distinguish clinical hepatotoxic drugs from their non-liver-toxic analogs and understand the mechanism of action prior to... (truncated)

Title: Buspirone for early satiety and symptoms of gastroparesis: A multi-centre, randomised, placebo-controlled, double-masked trial (BESST).
Label="BACKGROUND">Patients with gastroparesis and related disorders have symptoms including early satiety, postprandial fullness and bloating. Buspirone, a 5-HT₁ receptor agonist, may improve fundic accommodation.

Title: [A new generation of tranquilizing agents].
The newer anxiolytics include compounds whose the molecular structure, the mechanisms, and the pharmacological properties are heterogeneous. Nevertheless, the most of them have clinical and adverse effects like to the most known: buspirone, after the commercial shrinking for hepatic toxicity of alpidem. These anxiolytics are efficacious against generalized anxiety disorder, like the benzodiazepines. The principal interest consists in minimal adverse effects and the safety of the use. These compounds have not a sedative effect, do not induce rebound, dependence, abuse and withdrawal, do not impair the psychomotor, cognitive and memory performances. The big ratio efficacy/tolerance allows to use them in... (truncated)

Title: Synergistic drug-cytokine induction of hepatocellular death as an in vitro approach for the study of inflammation-associated idiosyncratic drug hepatotoxicity.
Idiosyncratic drug hepatotoxicity represents a major problem in drug development due to inadequacy of current preclinical screening assays, but recently established rodent models utilizing bacterial LPS co-administration to induce an inflammatory background have successfully reproduced idiosyncratic hepatotoxicity signatures for certain drugs. However, the low-throughput nature of these models renders them problematic for employment as preclinical screening assays. Here, we present an analogous, but high-throughput, in vitro approach in which drugs are administered to a variety of cell types (primary human and rat hepatocytes and the human HepG2 cell line) across a landscape of inflammatory contexts containing LPS and cytokines TNF,... (truncated)

Title: Treatment of Irritability in Huntington's Disease.
Irritability is a common neuropsychiatric feature of Huntington's disease (HD), with prevalences varying from 38% to 73%. Similar prevalences of irritability are reported in other neurodegenerative disorders and traumatic brain injury, especially when the frontal lobe is involved. Before therapeutic interventions are initiated, the clinician should analyze the severity and frequency of the irritable behavior. By examining irritability in a broader spectrum, a tailor-made treatment can be provided.In general, I recommend as a first step a selective serotonin reuptake inhibitor (SSRI), such as sertraline, or the mood stabilizer valproate; they both have a mild side effect profile. Next, if the... (truncated)

MESH:C027235 - gallium nitrate

Summary:

LLM Prediction Score	0.071 (normalized)
LLM Confidence Score	0.930
Golden Answer (Severity Class)	0.375 (normalized)
Prediction Error	0.304

Retrieved Context:

Title: Gallium nitrate suppresses the production of nitric oxide and liver damage in a murine model of LPS-induced septic shock.
The efficacy of gallium (Ga) nitrate was examined in a murine model of sepsis. Male Balb/c mice (6-8 weeks) were randomized into 3 groups: 1) vehicle-treated controls 2) mice with sepsis induced by treatment with 0.3 mg i.v. of Propionibacterium acnes followed one week later by 0.01 microg lipopolysaccharide (LPS) and 10 mg of D-galactosamine (GalN) 3) mice with sepsis injected with 45 mg/kg s.c. of gallium nitrate (calculated as elemental Ga) 24 hours prior to LPS/GalN. Two hours after LPS/GalN or vehicle, plasma concentrations of tumor necrosis factor (TNF-alpha) in groups 1, 2 and 3 were 54+/-31 (n=6), 21,390+/-5139... (truncated)

Title: Phase II evaluation of gallium nitrate by continuous infusion in breast cancer.
We evaluated the role of gallium nitrate infusion in the treatment of metastatic breast cancer. Gallium nitrate was administered at 300 mg/m2/day for 7 days every 3 weeks by continuous infusion concomitantly with oral calcium supplement of 500 mg twice daily and oral hydration. Fifteen patients with refractory metastatic breast cancer received such treatment for a total of 30 courses. Median age was 51, and median performance status (Zubrod scale) was 1. These patients had minimal prior chemotherapy (median 1 regimen). All patients were evaluable for toxicity and 14 for response. Nine patients had one to two metastatic sites, five... (truncated)

Title: Gallium Nanoparticle-Mediated Reduction of Brain Specific Serine Protease-4 in an Experimental Metastatic Cancer Model
Purpose: Tumor growth and metastasis depend on angiogenesis; therefore, efforts are being made to develop specific angiogenic inhibitors. Gallium (Ga) is the second most common metal ion, after platinum, used in cancer treatment. Its activities are numerous and various. In the present study, we aimed to investigate the effect of Ga on brain metastasis arising from hepatocellular carcinoma (HCC). Materials and methods: Forty experimental rats (divided into 4 groups) received diethylnitrosamine (DEN) at a dose (20 mg/kg.b.wt.; for 6 weeks) to induce HCC and were treated with Ga nanoparticles (GaNPs) with the bacterium Bacillus licheniformis (1mg/kg.b.wt.). Liver functions (alanine aminotransferase;... (truncated)

Title: Cancer Inhibition and In Vivo Osteointegration and Compatibility of Gallium-Doped Bioactive Glasses for Osteosarcoma Applications.
Traditional osteosarcoma therapies tend to focus solely on eradicating residual cancer cells and often fail to promote local bone regeneration and even inhibit it due to lack of precise control over target cells, i.e., the treatment affects both normal and cancer cells. Typically, multistep procedures are required for optimal efficacy. Here, we found that a silica-based bioactive material containing 3 mol % gallium oxide selectively kills human osteosarcoma cells and presents excellent in vivo osteointegration, while showing no local or systemic toxicity. Cell culture media conditioned with the proposed material was able to kill 41% of osteosarcoma cells, and no... (truncated)

Title: Compromised glutathione synthesis results in high susceptibility to acetaminophen hepatotoxicity in acatalasemic mice.
Acatalasemia is caused by genetic defect in the catalase gene. Human achatalasemia patients are able to scavenge physiological hydrogen peroxide but are vulnerable to exogenous oxidative stress. In the present study, we used an acetaminophen-induced hepatotoxicity model in acatalasemic mice to explore this vulnerability. Interestingly, the acetaminophen-induced decrease in total glutathione levels was more prolonged in acatalasemic mice. While the subunits of glutamate-cysteine ligase, a glutathione synthase enzyme, were increased by acetaminophen in the liver of wild-type mice, their expression was lower and was further reduced by acetaminophen in acatalasemic mice. This feature was also observed in immortalized hepatocytes derived... (truncated)

MESH:D003609 - dactinomycin

Summary:

LLM Prediction Score	0.699 (normalized)
LLM Confidence Score	0.980
Golden Answer (Severity Class)	1.0 (normalized)
Prediction Error	0.301

Retrieved Context:

Title: [Veno-occlusive disease of the liver as a treatment complication in children with Wilm's tumor].
We report on three children aged 1 1/2, 2 and 9 1/2 years with Wilms' tumor, who developed a tender hepatomegaly and ascites associated with elevated liver enzymes, anemia and thrombocytopenia during chemotherapy. This clinical picture and liver sonography abnormality are best explained by veno-occlusive disease (VOD) of the liver, while other causes of liver disease could not be identified. Actinomycin D dosage was 0.045 mg/kg as bolus injection in two patients and 0.075 mg/kg split over five days in a third patient. Presumable, this drug was the causative agent. VOD was observed after preoperative and postoperative chemotherapy. No child... (truncated)

Title: Age is a risk factor for chemotherapy-induced hepatopathy with vincristine, dactinomycin, and cyclophosphamide.
To evaluate the spectrum of and determine the risk factors for the development of liver toxicity (hepatopathy) after therapy with vincristine, dactinomycin, and cyclophosphamide (VAC) for rhabdomyosarcoma in children and adolescents.

Title: Hepatopathy-thrombocytopenia syndrome--a complication of dactinomycin therapy for Wilms' tumor: a report from the United Kingdom Childrens Cancer Study Group.
We have observed hepatopathy, associated with thrombocytopenia, in children receiving chemotherapy for Wilms' tumor. We have studied this hepatopathy-thrombocytopenia syndrome (HTS) in patients enrolled in the United Kingdom Childrens' Cancer Study Group (UKCCSG) Wilms' tumor trials (UKW1 and UKW2). At the time of this study, 501 patients had completed therapy. Treatment flow sheets were examined for evidence of hepatopathy (hepatomegaly with abnormal liver function tests) and severe thrombocytopenia (platelet count less than 25 x 10(9)/L). No child who developed the syndrome had received irradiation. HTS was seen in five of 355 (1.4%) of patients treated with combination chemotherapy but in... (truncated)

Title: Severe hepatic toxicity after treatment with single-dose dactinomycin and vincristine. A report of the National Wilms' Tumor Study.
Dactinomycin is an antitumor antibiotic with known activity against many pediatric solid tumors. Administration of dactinomycin using a single-dose schedule was incorporated into the design of the National Wilms' Tumor Study 4 (NWTS-4). This was done to determine whether laboratory and preliminary clinical data, suggesting that such a schedule was associated with increased antitumor effect and/or decreased normal tissue toxicity, could be validated in a large clinical trial. Five patients treated with regimens EE-4 or K-4, regimens that included single-dose dactinomycin and no abdominal irradiation, experienced severe hepatic toxicity. The clinical courses of these patients suggested that multiple factors, including... (truncated)

Title: Single-dose versus fractionated-dose dactinomycin in the treatment of Wilms' tumor. Preliminary results of a clinical trial. The Brazilian Wilms' Tumor Study Group.
A clinical trial was conducted by the Brazilian Wilms' Tumor (WT) Study Group to compare the single-dose (60 micrograms/kg x 1 day) administration of dactinomycin (AMD) with the standard fractionated dose (15 micrograms/kg/d x 5 days) used in the US National WT Study. Except for the AMD administration, treatment for all patients followed that of the latter study. Patients were randomized to receive either of the two AMD regimens in the schedules most appropriate for their stage and histologic condition. One hundred seventy-six children with WT entered the study until December 1988. No significant differences in overall or relapse-free survival... (truncated)

MESH:D003533 - cyproheptadine

Summary:

LLM Prediction Score	0.575 (normalized)
LLM Confidence Score	0.980
Golden Answer (Severity Class)	0.875 (normalized)
Prediction Error	0.300

Retrieved Context:

Title: Apetamin Hepatotoxicity: Potential Consequences of Purchasing a Body Enhancement Drug Off the Internet.
We report Apetamin (cyproheptadine lysine and vitamin syrup), a non-US Food and Drug Administration-approved weight gain supplement, causing drug-induced autoimmune hepatitis. A 40-year-old previously healthy woman presented with fatigue, right-sided abdominal discomfort, and jaundice 6 weeks after starting Apetamin, which she learned from social media for figure augmentation. Labs were significant for elevated transaminases, positive smooth muscle antibody, and increased immunoglobulins. Biopsy indicated drug-induced autoimmune hepatitis. Symptoms improved with prednisone, azathioprine, and stopping Apetamin which contains cyproheptadine, a known hepatotoxin. The case reveals the influence of social media and its impact on health and the importance of a complete drug... (truncated)

Title: [Hepatitis caused by cyproheptadine (Periactine). A case and review of the literature].
We report the case of a patient who developed jaundice after receiving cyproheptadine for 29 days. Complete recovery occurred within 3 months after cyproheptadine withdrawal. Analysis of 3 previously reported cases and this observation shows that cyproheptadine-induced hepatitis is uncommon. Hepatitis occurs within the first month of treatment and is of the cytolytic or mixed-pattern type. Jaundice is constant. Most often, recovery occurs quickly after the discontinuation of cyproheptadine. However, acute hepatitis can be followed by prolonged anicteric cholestasis.

Title: Safety of Cyproheptadine, an Orexigenic Drug. Analysis of the French National Pharmacovigilance Data-Base and Systematic Review.
Objectives: Cyproheptadine is a first-generation H1-antihistamine drug first that was distributed in the 1960s. While its orexigenic effect was observed early, cyproheptadine is not yet authorized for this indication in all countries today. There is an increasing medical interest and demand for the orexigenic effect of cyproheptadine, especially in children with poor appetite. As cyproheptadine might be evaluated in future clinical trials, we wanted to assess its safety profile. Methods: Using the French national pharmacovigilance database, we retrospectively analyzed all pediatric and adult reports of adverse effects of cyproheptadine recorded since its first distribution in France. Next, we performed a... (truncated)

Title: Poisoning due to an over-the-counter hypnotic, Sleep-Qik (hyoscine, cyproheptadine, valerian).
The clinical features and risk of hepatotoxicity of 'Sleep-Qik' (valerian dry extract 75 mg, hyoscine hydrobromide 0.25 mg, cyproheptadine hydrochloride 2 mg) were determined in 23 patients treated in our hospital between 1988 and 1991. The main clinical problems were central nervous system depression and anticholinergic poisoning. There was no clinical evidence of acute hepatitis in the 23 patients after taking an average of 2.5 g of valerian (range 0.5 to 12 g). There was no evidence of subclinical liver damage in 12 patients who had routine liver function tests performed approximately 6-12 hours after ingestion. Delayed onset of severe... (truncated)

Title: Prolonged cholestasis after cyproheptadine-induced acute hepatitis.
We report a patient in whom cyproheptadine-induced hepatitis was followed by prolonged cholestasis marked by elevation of serum alkaline phosphatase levels, gammaglutamyltransferase and bile acid levels, and disappearance of small bile ducts. Chlorpromazine and imipramine, which can induce a similar acute hepatitis followed by protracted cholestasis, have a close chemical structure (i.e., a tricyclic ring). We suggest that this structure might be involved in this type of hepatotoxicity.

MESH:D017329 - triptorelin pamoate

Summary:

LLM Prediction Score	0.077 (normalized)
LLM Confidence Score	0.960
Golden Answer (Severity Class)	0.375 (normalized)
Prediction Error	0.298

Retrieved Context:

Title: The efficacy and safety of triptorelin-therapy following conservative surgery for deep infiltrating endometriosis: A multicenter, prospective, non-interventional study in China.
Triptorelin is one of the most commonly used gonadotropin-releasing hormone agonists and has been used in the treatment of deep infiltrating endometriosis (DIE). This study aimed to evaluate the efficacy and safety of up to 24 weeks of triptorelin treatment after conservative surgery for DIE.This prospective, non-interventional study was performed in 18 tertiary hospitals in China. Premenopausal women aged ≥18 years treated with triptorelin 3.75 mg once every 28 days for up to 24 weeks after conservative surgery for DIE were included. Endometriosis symptoms were assessed, using a visual analogue scale (0-10 cm) or numerical range (0-10), at baseline (pre-surgery)... (truncated)

Title: Prospective randomized study comparing the long-acting gonadotropin-releasing hormone agonist triptorelin, flutamide, and cyproterone acetate, used in combination with an oral contraceptive, in the treatment of hirsutism.
To compare triptorelin, cyproterone acetate (CPA), and flutamide, in combination with an oral contraceptive, in the treatment of hirsutism.

Title: Long-Term Treatment With Letrozole in a Boy With Familial Male-Limited Precocious Puberty.
The long-term follow-up in children with familial male-limited precocious puberty (FMPP) who were treated with letrozole, triptorelin, and spironolactone is limited, especially considering the efficiency and safety.

Title: Influence of exogenous gonadotropin-releasing hormone on seasonal reproductive behavior of the coyote (Canis latrans).
Wild Canis species such as the coyote (C. latrans) express a suite of reproductive traits unusual among mammals, including perennial pair-bonds and paternal care of the young. Coyotes also are monestrous, and both sexes are fertile only in winter; thus, they depend upon social and physiologic synchrony for successful reproduction. To investigate the mutability of seasonal reproduction in coyotes, we attempted to evoke an out-of-season estrus in October using one of two short-acting gonadotropin-releasing hormone (GnRH) agents: (1) a GnRH analogue, deslorelin (6-D-tryptophan-9-(N-ethyl-L-prolinamide)-10-deglycinamide), 2.1mg pellet sc; or (2) gonadorelin, a GnRH (5-oxoPro-His-Trp-Ser-Tyr-Gly-Leu-Arg-Pro-GlyNH(2)) porcine hypothalamic extract, 2.0 microg/kg im once daily... (truncated)

Title: A pilot study of gonadotropin-releasing hormone agonist combined with aromatase inhibitor as fertility-sparing treatment in obese patients with endometrial cancer.
This study aims to evaluate the effects and pregnancy outcomes of gonadotropin-releasing hormone agonist (GnRH agonist) combined with aromatase inhibitor (AI) in preserving the fertility of obese women with grade 1 endometrial cancer (EC).

MESH:C011481 - methenamine hippurate

Summary:

LLM Prediction Score	0.078 (normalized)
LLM Confidence Score	0.920
Golden Answer (Severity Class)	0.375 (normalized)
Prediction Error	0.297

Retrieved Context:

Title: Sulfasalazine-Induced Delayed Hypersensitivity Reaction Presenting as Fever, Aseptic Meningitis, and Mesenteric Panniculitis in a Patient with Seronegative Arthritis.
BACKGROUND An 82-year-old woman presented with acute pyrexial illness and mesenteric panniculitis and developed biochemical aseptic meningitis (cerebrospinal fluid pleocytosis with no identifiable pathogen). Investigation determined her illness was likely a delayed hypersensitivity reaction caused by sulfasalazine. Sulfasalazine-induced aseptic meningitis is a rare condition often diagnosed late in a patient's admission owing to initial non-specific illness symptomatology requiring the exclusion of more common "red flag" etiologies, such as infection and malignancy. CASE REPORT An 82-year-old woman with a history of recurrent urinary tract infections and seronegative arthritis presented with a 3-day history of fatigue, headache, dyspnea, and lassitude. On admission,... (truncated)

Title: Development of an LC-MS/MS Assay and Toxicokinetic Characterization of Hexamethylenetetramine in Rats.
Hexamethylenetetramine, an aldehyde-releasing agent, is used as a preservative in various food, cosmetics, and medical treatments, such as a treatment for urinary tract infections. It has been reported to be allergenic on contact with the skin, with the additional possibility of causing toxicity once absorbed systemically. Despite its potential toxicity, there are no reports on the in vivo bioavailability of hexamethylenetetramine following oral or dermal administration. In this study, we developed a new simple and sensitive LC-MS/MS method for the determination of hexamethylenetetramine in plasma and applied this method to characterize the toxicokinetics. The developed assay had a sufficient specificity... (truncated)

Title: Bacterial and Viral Infection and Sepsis in Kidney Transplanted Patients.
Kidney transplanted patients are a unique population with intrinsic susceptibility to viral and bacterial infections, mainly (but not exclusively) due to continuous immunosuppression. In this setting, infectious episodes remain among the most important causes of death, with different risks according to the degree of immunosuppression, time after transplantation, type of infection, and patient conditions. Prevention, early diagnosis, and appropriate therapy are the goals of infective management, taking into account that some specific characteristics of transplanted patients may cause a delay (the absence of fever or inflammatory symptoms, the negativity of serological tests commonly adopted for the general population, or the... (truncated)

Title: Pycnogenol® Supplementation Prevents Recurrent Urinary Tract Infections/Inflammation and Interstitial Cystitis.
This open pilot registry study aimed to evaluate and compare the prophylactic effects of Pycnogenol® or cranberry extract in subjects with previous, recurrent urinary tract infections (UTI) or interstitial cystitis (IC). <i>Methods</i>. Inclusion criteria were recurrent UTI or IC. One subject group was supplemented with 150 mg/day Pycnogenol®, another with 400 mg/day cranberry extract, and a group served as a control in a 2-month open follow-up. <i>Results</i>. 64 subjects with recurrent UTI/IC completed the study. The 3 groups of subjects were comparable at baseline. All subjects had significant symptoms (minor pain, stranguria, repeated need for urination, and lower, anterior abdominal pain) at... (truncated)

Title: A double-blinded randomized placebo-controlled non-inferiority trial protocol for postoperative infections associated with canine pyometra.
Pyometra is a common infectious condition, especially in elderly bitches. In addition to an infected uterus, dogs may have concurrent urinary tract infection (UTI). The preferred treatment is surgical removal of the ovaries and uterus, whereupon the general prognosis is excellent. In addition, antimicrobial therapy is frequently prescribed for postoperative treatment. However, no research exists on the benefit of postoperative antimicrobial treatment in uncomplicated canine pyometra. Antimicrobial resistance has become a major challenge in treatment of bacterial infections. Diminishing overuse of antimicrobial agents is essential for controlling the development of antimicrobial resistance in both animals and humans.

MESH:D008012 - lidocaine

Summary:

LLM Prediction Score	0.291 (normalized)
LLM Confidence Score	0.990
Golden Answer (Severity Class)	0.0 (normalized)
Prediction Error	0.291

Retrieved Context:

Title: [Unknown fever and abnormal liver functions after repeated epidural blocks with lidocaine for management of herpes zoster pain].
We present a case of unknown fever and abnormal liver functions which developed during the course of pain management for herpes zoster with repeated epidural blocks with 0.5% lidocaine 10 ml. The patient was a 67 year old woman. At her first admission to dermatology, there were no abnormal findings in her blood examinations. She complained of severe pain from herpes zoster. She was admitted to the pain clinic. She received thoracic epidural blocks with 0.5% lidocaine 10 ml repeatedly three or four times a week. Two weeks later, she developed general fatigue, appetite loss, nausea and a high fever.... (truncated)

Title: Lido-OH, a Hydroxyl Derivative of Lidocaine, Produced a Similar Local Anesthesia Profile as Lidocaine With Reduced Systemic Toxicities.
Background: lidocaine is one of the most commonly used local anesthetics for the treatment of pain and arrhythmia. However, it could cause systemic toxicities when plasma concentration is raised. To reduce lidocaine's toxicity, we designed a hydroxyl derivative of lidocaine (lido-OH), and its local anesthesia effects and systemic toxicity <i>in vivo</i> were quantitively investigated. Method: the effectiveness for lido-OH was studied using mouse tail nerve block, rat dorsal subcutaneous infiltration, and rat sciatic nerve block models. The systemic toxicities for lido-OH were evaluated with altered state of consciousness (ASC), arrhythmia, and death in mice. Lidocaine and saline were used as... (truncated)

Title: Lignocaine toxicity after anterior nasal packing.
No abstract available.

Title: Lidocaine Infusion: An Antiarrhythmic With Neurologic Toxicities.
As a renowned local anesthetic agent of choice, lidocaine is also a class 1b antiarrhythmic agent that is primarily used for the treatment of ventricular arrhythmias. Although lidocaine systemic toxicity is rare, it may be life-threatening; thus, its early identification and management are of vital importance. This case report details the clinical scenario of intravenous lidocaine administration to a patient at high risk of toxicity in a 64-year-old patient, who initially presented with sustained monomorphic ventricular tachycardia received lidocaine and subsequently developed neurologic manifestations of lidocaine toxicity, including altered mental status and seizure. The patient was treated promptly with benzodiazepine... (truncated)

Title: Neurologic toxicity of lidocaine during awake intubation in a patient with tongue base abscess. Case report.
Lidocaine is commonly used for topical anesthesia of the upper airway in patient with anticipated difficult tracheal intubation undergoing awake fiberoptic intubation. Lidocaine toxicity is dose related and proportional to its plasma level. Although neurologic toxicity has been frequently observed with intravenous use, it has also been reported for topical use. We report on a case of a patient with base tongue abscess who developed sudden seizures and coma during application of topical anesthesia with lidocaine for awake fiberoptic intubation. The presence of a deep neck infection that causes hyperemia and edema of the pharyngolaryngeal mucosa may enhance transmucosal systemic... (truncated)

MESH:D000077431 - oxaprozin

Summary:

LLM Prediction Score	0.662 (normalized)
LLM Confidence Score	0.940
Golden Answer (Severity Class)	0.375 (normalized)
Prediction Error	0.287

Retrieved Context:

Title: Development a novel robust method to enhance the solubility of Oxaprozin as nonsteroidal anti-inflammatory drug based on machine-learning.
Accurate specification of the drugs' solubility is known as an important activity to appropriately manage the supercritical impregnation process. Over the last decades, the application of supercritical fluids (SCFs), mainly CO₂, has found great interest as a promising solution to dominate the limitations of traditional methods including high toxicity, difficulty of control, high expense and low stability. Oxaprozin is an efficient off-patent nonsteroidal anti-inflammatory drug (NSAID), which is being extensively used for the pain management of patients suffering from chronic musculoskeletal disorders such as rheumatoid arthritis. In this paper, the prominent purpose of the authors is to predict and consequently... (truncated)

Title: Oxaprozin-induced symptomatic hepatotoxicity.
To describe a case of symptomatic hepatotoxicity attributed to oxaprozin use.

Title: Clinical pharmacokinetics of oxaprozin.
Oxaprozin is a nonsteroidal anti-inflammatory drug which reaches peak plasma concentrations 2 to 6 hours after oral administration. Oxaprozin binds extensively, in a concentration-dependent manner, to plasma albumin. The area under the plasma concentration-time curve (AUC) of oxaprozin is linearly proportional to the dose for oral doses up to 1200 mg. At doses greater than 1200 mg there is an increase in the unbound fraction of drug, leading to an increased clearance and volume of distribution (Vd) of total oxaprozin. Accumulation of the drug at steady state is between 40 and 58% lower than predicted by single dose data. After... (truncated)

Title: Hepatic safety of two analgesics used over the counter: ibuprofen and aspirin.
We evaluated the potential hepatic toxicity of ibuprofen, aspirin, and oxaprozin in 1468 patients with rheumatoid arthritis and osteoarthritis by slightly modifying an algorithm that was developed to evaluate the drug relatedness of renal toxicity associated with therapeutic doses of these agents in the same population. Ibuprofen proved to be the safest of these nonsteroidal antiinflammatory drugs; it was associated with no AST elevation that was considered probably drug related as determined by application of the algorithm to laboratory values and information from case report forms. The frequency of probably drug-related AST elevations was highest (5%) with aspirin; with oxaprozin,... (truncated)

Title: Oxaprozin-induced fulminant hepatitis.
To report oxaprozin-induced fulminant hepatic failure.

MESH:D014042 - tolazamide

Summary:

LLM Prediction Score	0.536 (normalized)
LLM Confidence Score	0.880
Golden Answer (Severity Class)	0.25 (normalized)
Prediction Error	0.286

Retrieved Context:

Title: A case of chronic liver disease due to tolazamide.
Although chlorpropamide and tolbutamide are well recognized as causes of hepatotoxicity, there are only 3 reported cases of hepatic injury caused by a third oral hypoglycemic agent, tolazamide. In 2 of these cases, the liver-function tests returned to normal when the drug was discontinued. In the third case, the patient had cholestasis from chlorpropamide before administration of tolazamide and developed chronic liver disease. We are reporting the second instance of chronic liver disease induced by tolazamide. Our patient had been taking chlorpropamide, but she had no evidence of liver disease before administration of tolazamide. Tolazamide should be considered as a... (truncated)

Title: Alcohol and medication interactions.
Many medications can interact with alcohol, thereby altering the metabolism or effects of alcohol and/or the medication. Some of these interactions can occur even at moderate drinking levels and result in adverse health effects for the drinker. Two types of alcohol-medication interactions exist: (1) pharmacokinetic interactions, in which alcohol interferes with the metabolism of the medication, and (2) pharmacodynamic interactions, in which alcohol enhances the effects of the medication, particularly in the central nervous system (e.g., sedation). Pharmacokinetic interactions generally occur in the liver, where both alcohol and many medications are metabolized, frequently by the same enzymes. Numerous classes of... (truncated)

Title: Association of CYP1A1 and CYP1B1 inhibition in in vitro assays with drug-induced liver injury.
Drug-induced liver injury (DILI) is one of the major causes for the discontinuation of drug development and withdrawal of drugs from the market. Since it is known that reactive metabolite formation and being substrates or inhibitors of cytochrome P450s (P450s) are associated with DILI, we systematically investigated the association between human P450 inhibition and DILI. The inhibitory activity of 266 DILI-positive drugs (DILI drugs) and 92 DILI-negative drugs (no-DILI drugs), which were selected from Liver Toxicity Knowledge Base (US Food and Drug Administration), against 8 human P450 forms was assessed using recombinant enzymes and luminescent substrates, and the threshold values... (truncated)

Title: Interactions between Food and Drugs, and Nutritional Status in Renal Patients: A Narrative Review.
Drugs and food interact mutually: drugs may affect the nutritional status of the body, acting on senses, appetite, resting energy expenditure, and food intake; conversely, food or one of its components may affect bioavailability and half-life, circulating plasma concentrations of drugs resulting in an increased risk of toxicity and its adverse effects, or therapeutic failure. Therefore, the knowledge of these possible interactions is fundamental for the implementation of a nutritional treatment in the presence of a pharmacological therapy. This is the case of chronic kidney disease (CKD), for which the medication burden could be a problem, and nutritional therapy plays... (truncated)

Title: Post-Authorization Safety Studies of Acute Liver Injury and Severe Complications of Urinary Tract Infection in Patients with Type 2 Diabetes Exposed to Dapagliflozin in a Real-World Setting.
At the time of dapagliflozin's approval in Europe (2012) to treat patients with type 2 diabetes mellitus, concerns regarding acute liver injury and severe complications of urinary tract infection (sUTI) led to two post-authorization safety (PAS) studies of these outcomes to monitor the safety of dapagliflozin in real-world use.

MESH:D000068836 - rivastigmine

Summary:

LLM Prediction Score	0.090 (normalized)
LLM Confidence Score	0.990
Golden Answer (Severity Class)	0.375 (normalized)
Prediction Error	0.285

Retrieved Context:

Title: Treatment of dementia with neurotransmission modulation.
The prevalence of dementia is growing in developed countries where elderly patients are increasing in numbers. Neurotransmission modulation is one approach to the treatment of dementia. Cholinergic precursors, anticholinesterases, nicotine receptor agonists and muscarinic M(2) receptor antagonists are agents that enhance cholinergic neurotransmission and that depend on having some intact cholinergic innervation to be effective in the treatment of dementia. The cholinergic precursor choline alfoscerate may be emerging as a potential useful drug in the treatment of dementia, with few adverse effects. Of the anticholinesterases, donepezil, in addition to having a similar efficacy to tacrine in mild-to-moderate Alzheimer's disease (AD),... (truncated)

Title: In silico and in vitro studies confirm Ondansetron as a novel acetylcholinesterase and butyrylcholinesterase inhibitor.
Alzheimer's disease (AD) is a progressive neurodegenerative disorder that is growing rapidly among the elderly population around the world. Studies show that a lack of acetylcholine and butyrylcholine due to the overexpression of enzymes Acetylcholinesterase (AChE) and Butyrylcholinesterase (BChE) may lead to reduced communication between neuron cells. As a result, seeking novel inhibitors targeting these enzymes might be vital for the future treatment of AD. Ondansetron is used to prevent nausea and vomiting caused by chemotherapy or radiation treatments and is herein shown to be a potent inhibitor of cholinesterase. Comparison is made between Ondansetron and FDA-approved cholinesterase inhibitors Rivastigmine and Tacrine.... (truncated)

Title: Treatment of cognitive impairment in Alzheimer's disease.
In Alzheimer's disease, cognition now responds to several drugs. Anticholinesterases target the acetylcholine deficit. In mild-to-moderate Alzheimer's disease, they all provide significant benefit versus placebo on the Alzheimer's Disease Assessment ScheduleCognitive Section (ADAS-Cog), Side effects, in 5% to 15% of cases, include nausea, vomiting, diarrhea, anorexia, and dizziness. Tacrine, the leading anticholinesterase, caused frequent hepatic enzyme elevation and was withdrawn; once-daily donepezil spares the liver and improves global measures of change in severe dementia; rivasiigmine is indicated in comorbid vascular disease; while galaniamine modulates the cerebral nicotinic acetylcholine receptors that potentiate the response to acetylcholine. Alternative agents include the N-methyl-D-aspartate... (truncated)

Title: Acetylcholinesterase Inhibitors for Alzheimer's Disease Treatment Ameliorate Acetaminophen-Induced Liver Injury in Mice via Central Cholinergic System Regulation.
Acetaminophen (APAP) is widely used as an analgesic and antipyretic agent, but it may induce acute liver injury at high doses. Alzheimer's disease patients, while treated with acetylcholinesterase inhibitor (AChEI), may take APAP when they suffer from cold or pain. It is generally recognized that inhibiting acetylcholinesterase activity may also result in liver injury. To clarify whether AChEI could deteriorate or attenuate APAP hepatotoxicity, the effects of AChEI on APAP hepatotoxicity were investigated. Male C57BL/6J mice were administrated with the muscarinic acetylcholine receptor (mAChR) blocker atropine (Atr), or classic α7 nicotine acetylcholine receptor (α7nAChR) antagonist methyllycaconitine (MLA) 1 hour before... (truncated)

Title: Brain selective inhibition of acetylcholinesterase: a novel approach to therapy for Alzheimer's disease.
It could be argued that clinical experience with cholinergic drugs in the therapy of AD has not yet shown relevant symptomatic improvements. The main reasons for this might be attributed to peripheral cholinergic effects and the liver toxicity of some of these drugs, which limit their use and prevent confirmation of the cholinergic hypothesis (Gray et al., 1989). The main disadvantages of the cholinesterase inhibitors used in clinical trials are the short duration of action in the case of physostigmine and the potential for liver toxicity seen with the aminoacridine derivatives. The results presented with SDZ ENA 713 indicate that... (truncated)

MESH:C075156 - ebrotidine

Summary:

LLM Prediction Score	0.716 (normalized)
LLM Confidence Score	0.640
Golden Answer (Severity Class)	1.0 (normalized)
Prediction Error	0.284

Retrieved Context:

Title: Intrahepatic cholestatic jaundice related to administration of ranitidine. A case report with histologic and ultramicroscopic study.
Ranitidine may cause liver injuries ranging from transient, subclinical serum transaminases increase every 100-1,000 treated patients to cholestatic hepatitis in less than 1/100,000. Other H2-receptor antagonists are more dangerous: 11 toxic hepatitis cases have been reported as adverse effect after 1 year of marketed ebrotidine. A 75-year-old male with ischemic cardiopathy history was started on an 8 days treatment of oral ranitidine due to pirosis, without any other changes of therapy; 48 h after drug withdrawal, light-coloured stools, dark urine and icteric scleras developed. On hospital admission, 10 days later, physical examination showed slight hepatomegaly and severe jaundice with skin... (truncated)

Title: Acute liver injury associated with the use of ebrotidine, a new H2-receptor antagonist.
Ebrotidine is a new H2-receptor antagonist marketed in Spain in early 1997 and withdrawn in July 1998. We report 11 cases of acute liver injury related to ebrotidine and submitted to a Regional Registry of Hepatotoxicity between June 1997 and August 1998.

Title: Drug-Induced Liver Toxicity and Prevention by Herbal Antioxidants: An Overview.
The liver is the center for drug and xenobiotic metabolism, which is influenced most with medication/xenobiotic-mediated toxic activity. Drug-induced hepatotoxicity is common and its actual frequency is hard to determine due to underreporting, difficulties in detection or diagnosis, and incomplete observation of exposure. The death rate is high, up to about 10% for drug-induced liver damage. Endorsed medications represented >50% of instances of intense liver failure in a study from the Acute Liver Failure Study Group of the patients admitted in 17 US healing facilities. Albeit different studies are accessible uncovering the mechanistic aspects of medication prompted hepatotoxicity, we are... (truncated)

Title: Drug-induced liver injury: Do we know everything?
Interest in drug-induced liver injury (DILI) has dramatically increased over the past decade, and it has become a hot topic for clinicians, academics, pharmaceutical companies and regulatory bodies. By investigating the current state of the art, the latest scientific findings, controversies, and guidelines, this review will attempt to answer the question: Do we know everything? Since the first descriptions of hepatotoxicity over 70 years ago, more than 1000 drugs have been identified to date, however, much of our knowledge of diagnostic and pathophysiologic principles remains unchanged. Clinically ranging from asymptomatic transaminitis and acute or chronic hepatitis, to acute liver failure,... (truncated)

Title: A Critical Perspective on 3D Liver Models for Drug Metabolism and Toxicology Studies.
The poor predictability of human liver toxicity is still causing high attrition rates of drug candidates in the pharmaceutical industry at the non-clinical, clinical, and post-marketing authorization stages. This is in part caused by animal models that fail to predict various human adverse drug reactions (ADRs), resulting in undetected hepatotoxicity at the non-clinical phase of drug development. In an effort to increase the prediction of human hepatotoxicity, different approaches to enhance the physiological relevance of hepatic <i>in vitro</i> systems are being pursued. Three-dimensional (3D) or microfluidic technologies allow to better recapitulate hepatocyte organization and cell-matrix contacts, to include additional cell... (truncated)

MESH:D009966 - orphenadrine

Summary:

LLM Prediction Score	0.283 (normalized)
LLM Confidence Score	0.960
Golden Answer (Severity Class)	0.0 (normalized)
Prediction Error	0.283

Retrieved Context:

Title: Utility of spherical human liver microtissues for prediction of clinical drug-induced liver injury.
Drug-induced liver injury (DILI) continues to be a major source of clinical attrition, precautionary warnings, and post-market withdrawal of drugs. Accordingly, there is a need for more predictive tools to assess hepatotoxicity risk in drug discovery. Three-dimensional (3D) spheroid hepatic cultures have emerged as promising tools to assess mechanisms of hepatotoxicity, as they demonstrate enhanced liver phenotype, metabolic activity, and stability in culture not attainable with conventional two-dimensional hepatic models. Increased sensitivity of these models to drug-induced cytotoxicity has been demonstrated with relatively small panels of hepatotoxicants. However, a comprehensive evaluation of these models is lacking. Here, the predictive value... (truncated)

Title: Embracing the Dark Side: Computational Approaches to Unveil the Functionality of Genes Lacking Biological Annotation in Drug-Induced Liver Injury.
In toxicogenomics, functional annotation is an important step to gain additional insights into genes with aberrant expression that drive pathophysiological mechanisms. Nevertheless, there exists a gap on annotation of these genes which often hampers the interpretation of results and limits their applicability in translational medicine. In this study, we evaluated the coverage of functional annotations of differentially expressed genes (DEGs) induced by 10 selected compounds from the TG-GATEs database identified as high- or no-risk in causing drug-induced liver injury (most-DILI or no-DILI, respectively) using <i>in vitro</i> human data. Functional roles of DEGs not present in the most common biological annotation... (truncated)

Title: A scoping review to identify and map the multidimensional domains of pain in adults with advanced liver disease.
Background: Pain is a significant problem in adults living with advanced liver disease, having limited guidance available for its clinical management. While pain is considered a multidimensional experience, there have been limited reviews of the pain literature in advanced liver disease conducted with a multidimensional framework. Aims: The goal of this scoping review was to identify and map the multidimensional domains of pain in adults with advanced liver disease using the biopsychosocial model. Methods: We used Arksey and O'Malley's scoping framework. A search was conducted in MEDLINE, Embase, AMED, and CINAHL databases and the gray literature using specific eligibility criteria... (truncated)

Title: Metabolism and toxicity of usnic acid and barbatic acid based on microsomes, S9 fraction, and 3T3 fibroblasts <i>in vitro</i> combined with a UPLC-Q-TOF-MS method.
Introduction: Usnic acid (UA) and barbatic acid (BA), two typical dibenzofurans and depsides in lichen, have a wide range of pharmacological activities and hepatotoxicity concerns. This study aimed to clarify the metabolic pathway of UA and BA and illuminate the relationship between metabolism and toxicity. Methods: An UPLC-Q-TOF-MS method was developed for metabolite identification of UA and BA in human liver microsomes (HLMs), rat liver microsomes (RLMs), and S9 fraction (RS9). The key metabolic enzymes responsible for UA and BA were identified by enzyme inhibitors combined with recombinant human cytochrome P450 (CYP450) enzymes. The cytotoxicity and metabolic toxicity mechanism of... (truncated)

Title: Prediction of drug-induced liver injury and cardiotoxicity using chemical structure and in vitro assay data.
Drug-induced liver injury (DILI) and cardiotoxicity (DICT) are major adverse effects triggered by many clinically important drugs. To provide an alternative to in vivo toxicity testing, the U.S. Tox21 consortium has screened a collection of ~10K compounds, including drugs in clinical use, against >70 cell-based assays in a quantitative high-throughput screening (qHTS) format. In this study, we compiled reference compound lists for DILI and DICT and compared the potential of Tox21 assay data with chemical structure information in building prediction models for human in vivo hepatotoxicity and cardiotoxicity. Models were built with four different machine learning algorithms (e.g., Random Forest,... (truncated)

MESH:C004704 - cyclobenzaprine

Summary:

LLM Prediction Score	0.343 (normalized)
LLM Confidence Score	0.990
Golden Answer (Severity Class)	0.625 (normalized)
Prediction Error	0.282

Retrieved Context:

Title: Rhabdomyolysis: a manifestation of cyclobenzaprine toxicity.
A case of cyclobenzaprine (flexeril) overdose and the resultant rhabdomyolysis is presented. A review of the range of clinical toxicity, management of overdose is described. The similarity of cyclobenzaprine to the tricyclic antidepressant class is emphasized; this report attempts to disseminate related information on this commonly prescribed centrally acting muscle relaxant.

Title: Detective work in drug-induced liver injury: sometimes it is all about interviewing the right witness.
Diagnosing drug-induced liver injury (DILI) relies primarily on history taking. We report 4 cases in which DILI was missed or the drug was misidentified when physicians relied solely on patient history. We reviewed 27 cases referred with possible DILI from August 1, 2009, to February 1, 2010. Four patients seemed to be reliable historians, but their cases were greatly clarified by a call to their pharmacist. One subject, who forgot a new medication, underwent an unfruitful evaluation including surgery. Another patient had acetaminophen toxicity that was missed because she grossly underreported her pain medication use. The third and fourth patients... (truncated)

Title: Interaction of Energy Drinks with Prescription Medication and Drugs of Abuse.
In recent years, the consumption of energy drinks (EDs) has become increasingly popular, especially among adolescents. Caffeine, a psychostimulant, is the main compound of EDs which also contain other substances with pharmacological effects. This review aims to compile current evidence concerning the potential interactions between EDs, medicines, and drugs of abuse as they are frequently consumed in combination. The substances involved are mainly substrates, inductors or inhibitors of CYP1A2, psychostimulants, alcohol and other depressant drugs. Furthermore, intoxications reported with EDs and other substances have also been screened to describe acute toxicity. The results of our review show that the consumption... (truncated)

Title: Alcohol and medication interactions.
Many medications can interact with alcohol, thereby altering the metabolism or effects of alcohol and/or the medication. Some of these interactions can occur even at moderate drinking levels and result in adverse health effects for the drinker. Two types of alcohol-medication interactions exist: (1) pharmacokinetic interactions, in which alcohol interferes with the metabolism of the medication, and (2) pharmacodynamic interactions, in which alcohol enhances the effects of the medication, particularly in the central nervous system (e.g., sedation). Pharmacokinetic interactions generally occur in the liver, where both alcohol and many medications are metabolized, frequently by the same enzymes. Numerous classes of... (truncated)

Title: Duloxetine: a review of its safety and efficacy in the management of fibromyalgia syndrome.
Fibromyalgia (FM) is a chronic disorder characterized by widespread pain and other associated symptoms including fatigue, insomnia, cognitive/memory problems, and even psychological distress. Duloxetine is one of three FDA approved medications (the other two being milnacipran and pregabalin) for the treatment of FM. It has been demonstrated that FM patients possess low central nervous system levels of serotonin and norepinephrine. Duloxetine, which is classified pharmacologically as a serotonin-norepinephrine reuptake inhibitor (SNRI), may be beneficial for FM patients by increasing these levels. This review will touch briefly upon the pathophysiology of FM, diagnostic tools, currently available therapeutic options (both pharmacologic and... (truncated)

MESH:D008795 - metronidazole

Summary:

LLM Prediction Score	0.657 (normalized)
LLM Confidence Score	0.990
Golden Answer (Severity Class)	0.375 (normalized)
Prediction Error	0.282

Retrieved Context:

Title: Metronidazol as a probable cause of severe liver injury.
Metronidazol, a commonly used antibiotic drug, has been very rarely associated with hepatotoxicity. In particular, no reports have appeared in the literature about cases of metronidazol-associated severe hepatotoxicity, leading to liver transplantation or death. We report on a case of acute fulminant liver failure in a young woman, who had, two years previously, developed jaundice after intake of metronidazol. During the current hospitalization, metronidazol treatment had been undertaken two weeks previously and also this time the patient developed severe hepatocellular injury and cholestasis. A viral etiology was ruled out as well as vascular, metabolic and malignant etiology. Although, the cause... (truncated)

Title: Hepatotoxicity of metronidazole in Cockayne syndrome: A clinical report.
Cockayne syndrome (CS) is a rare autosomal recessive genetic disorder characterized by growth failure and progressive multisystem dysfunction caused by deficient nucleotide excision repair. Whereas metronidazole (MTZ) hepatotoxicity is quite rare in the general population, cases of severe hepatic reaction to MTZ have been reported in CS patients. We report here the case of a 21-year-old CS patient who presented with jaundice following one week of treatment with MTZ combined with spiramycin for dental care. This case is the first one documented with a liver biopsy. Histopathological analysis revealed portal and lobular inflammation with predominance of neutrophils, ballooning degeneration and... (truncated)

Title: Hepatic Failure following Metronidazole in Children with Cockayne Syndrome.
Cockayne syndrome is an uncommon autosomal recessive disease characterized by microcephaly, abnormal growth, and pathologic premature aging. The purpose of this report is to evaluate liver failure in children with Cockayne syndrome following metronidazole administration. The first case was a 2-year-old boy with Cockayne syndrome. He had been treated with metronidazole for gastroenteritis. 48 hours after treatment initiation, he was hospitalized due to jaundice, intractable vomiting, and agitation. Unfortunately, he died of acute liver failure. The second case was a 5-year-old boy with Cockayne syndrome as well, who had been treated with amoxicillin and metronidazole for a dental infection. He... (truncated)

Title: Severe hepatotoxicity associated with the combination of spiramycin plus metronidazole.
Drug-induced liver injury (DILI) is a leading cause of acute liver failure and is the most frequent reason for post-marketing drug withdrawal. The spectrum of liver injury is wide, ranging from mild and subclinical injury, noticeable only on routine biochemical testing, to fulminant liver failure and death. Antibiotics, as a group, are a leading cause of DILI. We herein describe 4 patients who developed moderate to severe hepatotoxicity after exposure to a commercially - available combination of two antibiotics - spiramycin and metronidazole - commonly used for the treatment and prevention of periodontal infections. No other aetiology for liver injury... (truncated)

Title: Ornidazole-induced liver damage: report of three cases and review of the literature.
Metronidazole and ornidazole, synthetic nitroimidazole derivatives, are used in the treatment of infections caused by anaerobic bacteria and protozoa. The drugs are well tolerated and serious side effects are very rarely encountered. Hepatotoxicity is a rare side effect and hitherto only six cases have been reported. We describe three patients who developed hepatitis after ornidazole use and review the previously reported cases. All three cases used ornidazole in conventional doses and developed hepatitis and associated cholestasis. They improved 1-2 months after discontinuation. We concluded that nitroimidazole derivatives may cause hepatotoxic damage resembling acute cholestatic hepatitis. Early recognition and withdrawal of... (truncated)

MESH:D009627 - nomifensine

Summary:

LLM Prediction Score	0.719 (normalized)
LLM Confidence Score	0.800
Golden Answer (Severity Class)	1.0 (normalized)
Prediction Error	0.281

Retrieved Context:

Title: Identification of multiple glutathione conjugates of 8-amino- 2-methyl-4-phenyl-1,2,3,4-tetrahydroisoquinoline maleate (nomifensine) in liver microsomes and hepatocyte preparations: evidence of the bioactivation of nomifensine.
8-Amino-2-methyl-4-phenyl-1,2,3,4-tetrahydroisoquinoline maleate (nomifensine), an antidepressant drug, was withdrawn from the market because of increased incidence of hemolytic anemia, as well as kidney and liver toxicity. Although the nature of the potentially reactive metabolites formed after nomifensine metabolism remains unknown and no glutathione (GSH) adducts of these nomifensine reactive metabolites have been reported, bioactivation has been postulated as a potential mechanism for the toxicity of nomifensine. This study was conducted to probe the potential bioactivation pathways of nomifensine in human and animal hepatocytes and in liver microsomes using GSH as a trapping agent. Two types of GSH conjugates were characterized by... (truncated)

Title: [Granulomatous hepatitis caused by nomifensine].
Four patients are described who developed granulomatous hepatitis 4-6 weeks after treatment with nomifensin. The granulomas were located both in the portal tract and the liver parenchyma, and contained eosinophils and a few polynuclear giant cells. Serum alkaline phosphatase, alanine aminotransferase and bilirubin were raised in three, two and one patient respectively. After medication was discontinued all abnormalities disappeared within 6-10 weeks. After oral intake of 25-50 mg nomifensin all patients experienced paroxysms of high body temperature for several hours. Reexposure in one patient resulted in another fever spike 6 hours after ingestion. No other cause of the fever or... (truncated)

Title: [Demonstration of drug-specific IgE and IgG antibodies using RIA: clinical importance as shown with nomifensin (Alival)].
Serum samples from 41 patients who developed adverse reactions during therapy with nomifensine were screened by RAST-based immunoassay for specific IgE and IgG antibodies against nomifensine and three of its metabolites. The results were compared with those of 10 patients without side effects and with 8 non-treated controls. Nomifensine-specific IgE antibodies were found in none of the subjects. However, all patients treated with nomifensine (with and without side effects) had specific IgG antibodies. The antibody cross-reacted in all cases with the metabolites. The titers did not discriminate clearly between the different side reactions and only partially between the presence or... (truncated)

Title: A correlation between the in vitro drug toxicity of drugs to cell lines that express human P450s and their propensity to cause liver injury in humans.
Drug toxicity to T-antigen-immortalized human liver epithelial (THLE) cells stably transfected with plasmid vectors that encoded human cytochrome P450s 1A2, 2C9, 2C19, 2D6, or 3A4, or an empty plasmid vector (THLE-Null), was investigated. An automated screening platform, which included 1% dimethyl sulfoxide (DMSO) vehicle, 2.7% bovine serum in the culture medium, and assessed
3-(4,5-dimethylthiazol-2-yl)-5-(3-carboxymethoxyphenyl)-2-(4-sulfophenyl)-2H-tetrazolium reduction, was used to evaluate the cytotoxicity of 103 drugs after 24h. Twenty-two drugs caused cytotoxicity to THLE-Null cells, with $EC_{50} \leq 200$ μM; 21 of these drugs (95%) have been reported to cause human liver injury. Eleven drugs exhibited lower EC_{50} values in cells transfected... (truncated)

Title: The Immunological Mechanisms and Immune-Based Biomarkers of Drug-Induced Liver Injury.
Drug-induced liver injury (DILI) has become one of the major challenges of drug safety all over the word. So far, about 1,100 commonly used drugs including the medications used regularly, herbal and/or dietary supplements, have been reported to induce liver injury. Moreover, DILI is the main cause of the interruption of new drugs development and drugs withdrawn from the pharmaceutical market. Acute DILI may evolve into chronic DILI or even worse, commonly lead to life-threatening acute liver failure in Western countries. It is generally considered to have a close relationship to genetic factors, environmental risk factors, and host immunity, through... (truncated)

MESH:D012254 - ribavirin

Summary:

LLM Prediction Score	0.594 (normalized)
LLM Confidence Score	0.990
Golden Answer (Severity Class)	0.875 (normalized)
Prediction Error	0.281

Retrieved Context:

Title: Hepatitis C virus cures after direct acting antiviral-related drug-induced liver injury: Case report.
The United States Food and Drug Administration recently warned that the direct acting antiviral (DAA) combination hepatitis C virus (HCV) treatment of Paritaprevir, Ombitasvir, Dasabuvir, Ritonavir, and Ribavirin (PODr + R) can cause severe liver injury in patients with advanced liver disease. Drug induced liver injury was observed in a small number of patients with decompensated cirrhosis treated with other DAAs, but has not been reported in patients with compensated cirrhosis. We report a case of a 74-year-old woman with chronic HCV and Child-Pugh class A cirrhosis (compensated cirrhosis) treated with PODr + R. The patient presented on day 14... (truncated)

Title: Liver toxicity associated with sofosbuvir, an NS5A inhibitor and ribavirin use.
Hepatitis C virus (HCV) infection is a major cause of end-stage liver disease and hepatocellular carcinoma. There have been rapid advances in HCV treatment with the development of oral direct-acting antivirals (DAAs). Studies have shown sustained virological response rates above 90% with combinations of DAAs, including patients with compensated cirrhosis. Thus far, significant drug toxicity has not been seen with these agents, but there is limited experience of using DAAs in decompensated HCV cirrhosis. This report describes the first experience of serious drug-induced hepatotoxicity with the new DAAs. The mechanism underlying these drug reactions is currently unknown. Few patients with... (truncated)

Title: A pilot study of ribavirin therapy for recurrent hepatitis C virus infection after liver transplantation.
Ribavirin is a guanosine analogue that normalizes serum liver enzymes in most nontransplant patients with chronic hepatitis C virus (HCV) infection. We conducted an uncontrolled pilot study of ribavirin in 9 liver transplantation recipients that had persistently elevated liver enzymes, active hepatitis by liver biopsy, and HCV RNA in serum by polymerase chain reaction. Ribavirin was given orally at dosages of 800-1200 mg per day for 3 mo. All 9 patients promptly responded to ribavirin: mean (+/- SD) ALT decreased from 392 +/- 377 IU/L immediately before treatment to 199 +/- 185 and 68 +/- 37 IU/L after 1 and... (truncated)

Title: Antiviral hepatitis and antiretroviral drug interactions.
More and more HIV-infected patients are treated for viral hepatitis, increasing interactions. HEPATITIS C: The concomitant use of didanosine and ribavirin increases the risk of mitochondrial toxicity, responsible for pancreatitis and/or lactic acidosis. Lactic acidosis is characterized by a high mortality rate. Thus, didanosine, but also stavudine, should not be co-administered with ribavirin. Cases of hepatic decompensation have been reported in cirrhotics concomitantly receiving ribavirin and didanosine. Thus, this co-admininistration should be contraindicated in patients with advanced liver fibrosis. Anemia is a frequent side effect of ribavirin. In patients with zidovudine-related anemia, this drug should be discontinued before prescribing ribavirin.... (truncated)

Title: Effect of ribavirin, levovirin and viramidine on liver toxicological gene expression in rats.
The ribavirin/interferon-alpha combination is currently the standard therapy for patients with chronic hepatitis C. However, ribavirin causes hemolytic anemia as a significant side-effect. Levovirin, an L-enantiomer of ribavirin, possesses similar immunomodulatory activity to ribavirin but lacks direct antiviral activity or hemolytic anemia. Viramidine is a liver-targeting prodrug of ribavirin with much less potential for hemolytic anemia. The aim of the present study is to profile the hepatic toxicological gene response to ribavirin, levovirin and viramidine. Rats were dosed orally with 120 mg kg(-1) day(-1) of ribavirin and viramidine and 2000 mg kg(-1) day(-1) of levovirin for 8 days. Ribavirin did... (truncated)

MESH:D008788 - metolazone

Summary:

LLM Prediction Score	0.095 (normalized)
LLM Confidence Score	0.960
Golden Answer (Severity Class)	0.375 (normalized)
Prediction Error	0.280

Retrieved Context:

Title: Risk of acute and serious liver injury associated to nimesulide and other NSAIDs: data from drug-induced liver injury case-control study in Italy.
Drug-induced liver injury is one of the most serious adverse drug reactions and the most frequent reason for restriction of indications or withdrawal of drugs. Some nonsteroidal anti-inflammatory drugs (NSAIDs) were withdrawn from the market because of serious hepatotoxicity. We estimated the risk of acute and serious liver injury associated with the use of nimesulide and other NSAIDs, with a prevalence of use greater than or equal to 5%.

Title: Acute Cocaine Intoxication: An Approach to Severe Hepatic and Renal Dysfunctions.
Cocaine is a highly addictive substance. Its poisoning can lead to potentially fatal multi-organ dysfunction. We report a case of cocaine overdose with severe multi-organ dysfunction. A healthy 51-year-old man was admitted to the emergency room due to behaviour changes and seizure after inhaling crack. Multiple dysfunctions were developed, with emphasis on liver and kidney dysfunction, due to their severity. The patient had marked hepatic cytolysis with a peak on the third day with alanine aminotransferase (ALT) and aspartate aminotransferase (AST): 7941 and 4453 IU/L, respectively with mild coagulopathy and hyperbilirubinemia. Underwent empirical treatment with acetylcysteine with good clinical response.... (truncated)

Title: An updated review on drug-induced cholestasis: mechanisms and investigation of physicochemical properties and pharmacokinetic parameters.
Drug-induced cholestasis is an important form of acquired liver disease and is associated with significant morbidity and mortality. Bile acids are key signaling molecules, but they can exert toxic responses when they accumulate in hepatocytes. This review focuses on the physiological mechanisms of drug-induced cholestasis associated with altered bile acid homeostasis due to direct (e.g., bile acid transporter inhibition) or indirect (e.g., activation of nuclear receptors, altered function/expression of bile acid transporters) processes. Mechanistic information about the effects of a drug on bile acid homeostasis is important when evaluating the cholestatic potential of a compound, but experimental data often are... (truncated)

Title: Positive Outcome in Catastrophic Momordica charantia-Associated Herb-Induced Liver Injury: A Tale of Two Cities - From Gonaives, Haiti to New York City.
Herb-induced liver injury (HILI) is a global concern due to the uptrend in Complementary and Alternative Medicine (CAM). The authors add to the current literature by reporting a case of a 61-year-old man with recent travel to Haiti. His past medical history include hepatitis C virus treated in 2021 with a sustained virologic response (SVR). He presented with profound weakness and abnormal liver transaminases in the thousands. It was initially unclear what the etiology of the patient's hepatocellular necrosis was, however, the level of abnormality was most consistent with either toxic metabolic injury or vascular ischemic injury. We initiated N-acetylcysteine and... (truncated)

Title: Drug-induced cholestasis.
Recent progress in understanding the molecular mechanisms of bile formation and cholestasis have led to new insights into the pathogenesis of drug-induced cholestasis. This review summarizes their variable clinical presentations, examines the role of transport proteins in hepatic drug clearance and toxicity, and addresses the increasing importance of genetic determinants, as well as practical aspects of diagnosis and management.

MESH:D008914 - minoxidil

Summary:

LLM Prediction Score	0.279 (normalized)
LLM Confidence Score	0.990
Golden Answer (Severity Class)	0.0 (normalized)
Prediction Error	0.279

Retrieved Context:

Title: [Polymyalgia induced by topical minoxidil].
Topical minoxidil, used in the treatment of baldness, has been commercially available since 1987. Its systemic side effects are rare. We observed an as yet unreported "polymyalgia syndrome" in four otherwise healthy males whose sole medication was topically applied minoxidil. They experienced fatigue, weight loss and severe pain in the shoulders and pelvic girdle, suggesting connective tissue disease. Three patients had a transient rise in liver enzymes, while other laboratory analyses remained normal. Tritanomaly was detected in two patients who underwent systematic color vision testing. All symptoms disappeared after withdrawal of minoxidil. Rechallenge was positive once in one patient and... (truncated)

Title: The hydroxypropyl-β-cyclodextrin-minoxidil inclusion complex improves the cardiovascular and proliferative adverse effects of minoxidil in male rats: Implications in the treatment of alopecia.
The efficacy of minoxidil (MXD) ethanolic solutions (1%-5% w/v) in the treatment of androgenetic alopecia is limited by adverse reactions. The toxicological effects of repeated topical applications of escalating dose (0.035%-3.5% w/v) and of single and twice daily doses (3.5% w/v) of a novel hydroxypropyl-β-cyclodextrin MXD GEL formulation (MXD/HP-β-CD) and a MXD solution were investigated in male rats. The cardiovascular effects were evaluated by telemetric monitoring of ECG and arterial pressure in free-moving rats. Ultrasonographic evaluation of cardiac morphology and function, and histopathological and biochemical analysis of the tissues, were performed. A pharmacovigilance investigation was undertaken using the EudraVigilance database... (truncated)

Title: Human Vascular Wall Microfluidic Model for Preclinical Evaluation of Drug-Induced Vascular Injury.
Drug-induced vascular injury (DIVI) in preclinical animal models often leads to candidate compound termination during drug development. DIVI has not been documented in human clinical trials with drugs that cause DIVI in preclinical animals. A robust human preclinical assay for DIVI is needed as an early vascular injury screen. A human vascular wall microfluidic tissue chip was developed with a human umbilical vein endothelial cell (HUVEC)-umbilical artery smooth muscle cell (vascular smooth muscle cell, VSMC) bilayer matured under physiological shear stress. Optimized temporal flow profiles produced HUVEC-VSMC bilayers with quiescent endothelial cell (EC) monolayers, EC tight junctions, and contractile VSMC... (truncated)

Title: Successful drug development despite adverse preclinical findings part 2: examples.
To illustrate the process of addressing adverse preclinical findings (APFs) as outlined in the first part of this review, a number of cases with unexpected APF in toxicity studies with drug candidates is discussed in this second part. The emphasis is on risk characterization, especially regarding the mode of action (MoA), and risk evaluation regarding relevance for man. While severe APFs such as retinal toxicity may turn out to be of little human relevance, minor findings particularly in early toxicity studies, such as vasculitis, may later pose a real problem. Rodents are imperfect models for endocrine APFs, non-rodents for human... (truncated)

Title: Atypical Case of Minoxidil-Induced Generalized Anasarca and Pleuropericardial Effusion.
Minoxidil is an antihypertensive medication used to control blood pressure that is resistant to three or more antihypertensive medications including diuretics. There have only been a few reported cases of minoxidil-induced pleuropericardial effusion with anasarca. Here, we discuss the case of a 70-year-old male with a history of uncontrolled hypertension who presented to the hospital with swelling of the extremities. He was on minoxidil 10 mg twice a day and complained of generalized body swelling and extremity pain with symptoms of dyspnea on exertion, paroxysmal nocturnal dyspnea, weight gain, abdominal distention, and intermittent, throbbing extremity pain (8/10). The patient denied... (truncated)

MESH:D019819 - budesonide

Summary:

LLM Prediction Score	0.278 (normalized)
LLM Confidence Score	0.990
Golden Answer (Severity Class)	0.0 (normalized)
Prediction Error	0.278

Retrieved Context:

Title: A Time to Pause and Reflect: When a Patient with Autoimmune Hepatitis Stops Responding to Corticosteroids.
Drug-induced liver injury (DILI) with features of autoimmunity (AI) is a challenging diagnosis to make particularly due to its apparent corticosteroid responsiveness. We present the case of a 74-year-old woman who presented with a 2-week history of jaundice and fatigue. She was initially diagnosed with autoimmune hepatitis (AIH) based on biochemical and histological characteristics and prompt response with budesonide but a biochemical relapse occurred soon after inadvertent rechallenge with irbesartan, a drug that she had discontinued prior to her presentation but was not initially considered to be a cause of her symptoms.

Title: Budesonide combined with ursodeoxycholic acid in primary biliary cirrhosis with advanced liver damage.
We describe two patients with primary biliary cirrhosis who rapidly suffered progressive liver failure and developed jaundice, despite having undergone correct therapy using ursodeoxycholic acid. Both cases showed an extraordinary clinical and biochemical response 2 months after budesonide was added to standard therapy, leading to recovery of normal liver function.

Title: Budesonide with Low-Dose 6-Mercaptopurine as a Possible New Treatment for IgG4-Related Sclerosing Cholangitis and Systemic IgG4-Related Disease: A Case Report.
BACKGROUND Systemic IgG4-related disease is a rare disease that can affect the hepatobiliary system and may lead to tissue fibrosis and organ failure. Diagnostic criteria for IgG4-related disease are well established, and systemic glucocorticoids are recommended for initiation of treatment. Besides the beneficial properties of glucocorticoids, the long-term treatment with systemic steroids carries the risk of toxicity, especially in elderly patients, in whom IgG4-related disease is more common. Furthermore, disease relapses may occur during the tapering of steroids. Overall, the optimal treatment approach for maintenance therapy has not been clarified yet and is an area of current clinical research. CASE... (truncated)

Title: Montelukast induced acute hepatocellular liver injury.
A 46-year-old male with uncontrolled asthma on inhaled albuterol and formoterol with budesonide was commenced on montelukast. He developed abdominal pain and jaundice 48 days after initiating montelukast therapy. His liver tests showed an increase in serum total bilirubin, conjugated bilirubin, aspartate aminotransferase, alanine aminotransferase, and alkaline phosphatase. The patient was evaluated for possible non-drug related liver injury. Montelukast was discontinued suspecting montelukast induced hepatocellular liver injury. Liver tests began to improve and returned to normal 55 days after drug cessation. Causality of this adverse drug reaction by the Council for International Organizations of Medical Sciences or Roussel Uclaf Causality... (truncated)

Title: Nonstandard drugs and feasible new interventions for autoimmune hepatitis: part I.
Nonstandard drugs that target critical pathogenic pathways or immune regulatory mechanisms constitute the next generation of treatments for autoimmune hepatitis. Mycophenolate mofetil impairs the proliferation of lymphocytes, decreases autoantibody production, and induces apoptosis of activated immunocytes. Patients treated for azathioprine intolerance improve more frequently than patients treated for refractory liver disease (54% versus 10%), and mycophenolate mofetil is emerging as a rescue therapy for this population. Complete corticosteroid withdrawal is possible in 40% of patients treated with mycophenolate mofetil, and the frequency of side effects ranges from 3-34%. Budesonide in combination with azathioprine has normalized liver tests more frequently (47%... (truncated)

MESH:D000068581 - tadalafil

Summary:

LLM Prediction Score	0.097 (normalized)
LLM Confidence Score	0.990
Golden Answer (Severity Class)	0.375 (normalized)
Prediction Error	0.278

Retrieved Context:

Title: Hepatic safety of ambrisentan alone and in combination with tadalafil: a post-hoc analysis of the AMBITION trial.
Treatment with endothelin receptor antagonists (ERA) can result in adverse hepatic effects in patients with pulmonary arterial hypertension (PAH). We evaluated the hepatic safety of ambrisentan (ABS), an ERA, used as monotherapy, or with tadalafil (TAD), a phosphodiesterase-5 (PDE5) inhibitor as initial combination therapy (ABS + TAD) in the AMBITION trial. This was a retrospective analysis set in academic and private outpatient clinics and research centers. This analysis included 596 patients with PAH who were randomized to ABS or TAD as monotherapy or ABS + TAD as initial combination therapy and received at least one dose of study drug, and... (truncated)

Title: The emergence of oral tadalafil as a once-daily treatment for pulmonary arterial hypertension.
Pulmonary hypertension (PH) is found in a vast array of diseases, with a minority representing pulmonary arterial hypertension (PAH). Idiopathic PAH or PAH in association with other disorders has been associated with poor survival, poor exercise tolerance, progressive symptoms of dyspnea, and decreased quality of life. Left untreated, patients with PAH typically have a progressive decline in function with high morbidity ultimately leading to death. Advances in medical therapy for PAH over the past decade have made significant inroads into improved function, quality of life, and even survival in this patient population. Three classes of pulmonary artery-specific vasodilators are currently... (truncated)

Title: Sexual function in hypertensive patients receiving treatment.
In many forms of erectile dysfunction (ED), cardiovascular risk factors, in particular arterial hypertension, seem to be extremely common. While causes for ED are related to a broad spectrum of diseases, a generalized vascular process seems to be the underlying mechanism in many patients, which in a large portion of clinical cases involves endothelial dysfunction, ie, inadequate vasodilation in response to endothelium-dependent stimuli, both in the systemic vasculature and the penile arteries. Due to this close association of cardiovascular disease and ED, patients with ED should be evaluated as to whether they may suffer from cardiovascular risk factors including hypertension,... (truncated)

Title: Tadalafil: the evidence for its clinical potential in the treatment of pulmonary arterial hypertension.
Pulmonary arterial hypertension (PAH), characterized by increased pulmonary artery pressures in the absence of elevated pulmonary venous pressures, is a progressive disease associated with reduced exercise capacity and increased mortality risk. Current treatments for PAH include nonspecific vasodilators, prostacyclin and related analogs, and endothelin receptor antagonists. Since phosphodiesterase type 5 is highly expressed in pulmonary vascular tissues, agents that selectively inhibit phosphodiesterase type 5 activity induce pulmonary arterial vasodilatation, and are being developed for the treatment of PAH.

Title: Perioperative management with phosphodiesterase type 5 inhibitor and prostaglandin E1 for moderate portopulmonary hypertension following adult-to-adult living-donor liver transplantation: a case report.
Portopulmonary hypertension (PPH) is a relatively rare but well-recognized complication of end-stage liver disease. Moderate or severe PPH (mean pulmonary artery pressure [mPAP] ≥ 35 mmHg) is usually a contraindication for liver transplantation due to high operation-related mortality. Here, we report on a patient with moderate PPH whose condition was successfully managed with a phosphodiesterase type 5 (PDE5) inhibitor (tadalafil) and prostaglandin E1, who experienced rapid improvement of PPH after living-donor liver transplantation (LDLT).

MESH:C095285 - silodosin

Summary:

LLM Prediction Score	0.098 (normalized)
LLM Confidence Score	0.940
Golden Answer (Severity Class)	0.375 (normalized)
Prediction Error	0.277

Retrieved Context:

Title: Acute hepatocellular drug induced liver injury probably by alfuzosin.
Alpha blockers are the drugs that exert their effects by binding to alpha receptors and relaxing smooth muscles and are currently used for treatment of benign prostate hyperplasia (BPH). These drugs are often tolerated well by the patients. However, they also possess some common side effects. Hepatotoxicity, on the other hand, is quite rare. We report herein a case with the rare complication of acute hepatocellular drug induced liver injury (DILI) by administration of Alfuzosin.

Title: Silodosin in the treatment of benign prostatic hyperplasia.
Benign prostatic hyperplasia (BPH)-associated lower urinary tract symptoms (LUTS) are highly prevalent in older men. Medical therapy is the first-line treatment for LUTS due to BPH. Alpha-adrenergic receptor blockers remain one of the mainstays in the treatment of male LUTS and clinical BPH. They exhibit early onset of efficacy with regard to both symptoms and flow rate improvement, and this is clearly demonstrated in placebo-controlled trials with extensions out to five years. These agents have been shown to prevent symptomatic progression of the disease. The aim of this article is to offer a critical review of the current literature on... (truncated)

Title: Manifestations of Liver Impairment and the Effects of MH-76, a Non-Quinazoline α1-Adrenoceptor Antagonist, and Prazosin on Liver Tissue in Fructose-Induced Metabolic Syndrome.
Excessive fructose consumption may lead to metabolic syndrome, metabolic dysfunction-associated fatty liver disease (MAFLD) and hypertension. α1-adrenoceptors antagonists are antihypertensive agents that exert mild beneficial effects on the metabolic profile in hypertensive patients. However, they are no longer used as a first-line therapy for hypertension based on Antihypertensive and Lipid-Lowering Treatment to Prevent Heart Attack Trial (ALLHAT) outcomes. Later studies have shown that quinazoline-based α1-adrenolytics (prazosin, doxazosin) induce apoptosis; however, this effect was independent of α1-adrenoceptor blockade and was associated with the presence of quinazoline moiety. Recent studies showed that α1-adrenoceptors antagonists may reduce mortality in COVID-19 patients due to... (truncated)

Title: Pharmacokinetics and pharmacodynamics of antifungals in children and their clinical implications.
Invasive fungal infections are a significant cause of morbidity and mortality in children. Successful management of these systemic infections requires identification of the causative pathogen, appropriate antifungal selection, and optimisation of its pharmacokinetic and pharmacodynamic properties to maximise its antifungal activity and minimise toxicity and the emergence of resistance. This review highlights salient scientific advancements in paediatric antifungal pharmacotherapies and focuses on pharmacokinetic and pharmacodynamic studies that underpin current clinical decision making. Four classes of drugs are widely used in the treatment of invasive fungal infections in children, including the polyenes, triazoles, pyrimidine analogues and echinocandins. Several lipidic formulations of... (truncated)

Title: Efficacy of silodosin on the outcome of semi-rigid ureteroscopy for the management of large distal ureteric stones: blinded randomised trial.
To evaluate the efficacy of silodosin therapy, as a new α-adrenergic receptor (α-AR) blocker, on the success rate of semi-rigid ureteroscopy (URS) for the management of large distal ureteric stones.

MESH:D002939 - ciprofloxacin

Summary:

LLM Prediction Score	0.600 (normalized)
LLM Confidence Score	0.990
Golden Answer (Severity Class)	0.875 (normalized)
Prediction Error	0.275

Retrieved Context:

Title: Ciprofloxacin Exposure Leading to Fatal Hepatotoxicity: An Unusual Correlation.
BACKGROUND Ciprofloxacin is a commonly used fluoroquinolone antibiotic. It is occasionally associated with benign elevations in liver enzymes. Few reports in the literature correlate ciprofloxacin with significant liver injury. We present a fatal case of ciprofloxacin-induced liver failure. CASE REPORT A 74-year-old female was successfully treated with ciprofloxacin for a urinary tract infection (UTI), but immediately began having new-onset symptoms, including fatigue and nausea. This continued for two months, at which time she presented to the hospital; she was found to have elevated liver enzymes and another UTI. She was treated with ciprofloxacin again for UTI and discharged three days... (truncated)

Title: [Acute liver failure after ingestion of ciprofloxacin].
A 23-year old female patient is referred to our intensive care unit from another hospital because of progredient neurological deterioration with sopor. One week before, she had experienced non-specific pain in her upper right stomach combined with vomitus and nausea. For two days, she had been treated with ciprofloxacin 2 × 500 mg / d by her primary care physician. Except for appendectomy in childhood, no other diseases were reported. Clinical investigation: Physical examination reveals mild scleral and dermal jaundice. There is tenderness of the upper right abdomen. Initially, no neurological pathological findings are obvious. Laboratory results show an increase... (truncated)

Title: Idiosyncratic Drug-Induced Liver Injury Due to Ciprofloxacin: A Report of Two Cases and Review of the Literature.
BACKGROUND Drug-induced liver injury (DILI) can present clinically as a spectrum that includes asymptomatic elevation of transaminases, acute or chronic hepatitis, and acute liver failure. Idiosyncratic DILI is more likely to affect individuals with comorbidities, and to have a wide range of clinical presentations. Although antibiotics are associated with DILI, the fluoroquinolone, ciprofloxacin, is a rarely reported cause. Two cases of idiosyncratic DILI following ciprofloxacin treatment are described, including a review of the literature. CASE REPORT Case 1: A 35-year-old man was treated with ciprofloxacin for periorbital cellulitis. On the second day of ciprofloxacin treatment, he developed abdominal pain, nausea,... (truncated)

Title: Acute Cholestatic Liver Injury Due to Ciprofloxacin in a Young Healthy Adult.
Ciprofloxacin is a commonly prescribed antibiotic due to its broad spectrum and good safety profile. However, recent evidence suggests that it has the propensity to cause idiosyncratic drug-induced liver injury. There are 25 reported cases of ciprofloxacin induced severe liver injury in the literature. Here, we describe another case of acute cholestatic liver injury due to ciprofloxacin. A 32-year-old female presented to the gastroenterology department with a week's history of pruritus, jaundice, and abdominal pain. Her symptoms started three days after completing a ciprofloxacin course for urinary tract infection. Her hepatic enzymes were elevated and showed a cholestatic pattern. An... (truncated)

Title: A follow-up safety study of ciprofloxacin users.
We followed 37,233 outpatients for 45 days after receiving a prescription for ciprofloxacin to identify any newly diagnosed, important illnesses that might have been caused by the drug. For 29 users the role of ciprofloxacin in the etiology of the illness could not be confidently ruled out (7.79/10,000 persons; 95% CI 5.42-11.18). In only seven was a causal relation to ciprofloxacin considered likely: three skin reactions and one case each of thrombocytopenia, "headache, nausea, and shakes," hallucinations, and palpitations. No fatal illnesses occurred, and all patients recovered after discontinuing the drug. In addition, few cases of photosensitivity were associated with... (truncated)

MESH:D002945 - cisplatin

Summary:

LLM Prediction Score	0.649 (normalized)
LLM Confidence Score	0.990
Golden Answer (Severity Class)	0.375 (normalized)
Prediction Error	0.274

Retrieved Context:

Title: Lethal hepatotoxicity following 5-fluorouracil/cisplatin chemotherapy: a relevant case report.
Some articles have reported severe toxicities induced by cisplatin/5-fluorouracil regimens, nevertheless, severe and lethal liver toxicity has not been previously reported. In this article, we report the case of a 72-year-old woman, who developed fulminant hepatitis, hypoglycemia and hypotension with atrial fibrillation not responding to treatment. After ruling out all other possible causes of hepatitis, the toxicity was more likely attributed to 5-fluorouracil. Genotyping was performed and the patient was found to be a homozygote carrier of the T variant of the MTHFR gene. The patient died two days later. Several factors, including genetic factors, could explain this severe toxicity.... (truncated)

Title: Platinum-based drug-induced depletion of amino acids in the kidneys and liver.
Cisplatin (cis-diamminedichloroplatinum II; CDDP) is a widely used cytostatic agent; however, it tends to promote kidney and liver disease, which are a major signs of drug-induced toxicity. Platinum compounds are often presented as alternative therapeutics and subsequently easily dispersed in the environment as contaminants. Due to the major roles of the liver and kidneys in removing toxic materials from the human body, we performed a comparative study of the amino acid profiles in chicken liver and kidneys before and after the application of CDDP and platinum nanoparticles (PtNPs-10 and PtNPs-40). The treatment of the liver with the selected drugs affected... (truncated)

Title: A case of severe hepatotoxicity induced by cisplatin and 5-fluorouracil.
Severe hepatotoxicity from combination chemotherapy with cisplatin and 5-fluorouracil is a rare adverse effect. In this case report, we present a case with severe hepatotoxicity immediately following chemotherapy with cisplatin and 5-fluorouracil. This female patient had previously been treated with this combination with no hepatotoxicity. The elevated liver enzymes quickly normalized after chemotherapy was stopped. There were no specific changes in liver imaging. As hepatotoxicity occurred after repeated administration of cisplatin, we suggest that this hepatotoxicity might represent a case of allergic hepatitis caused by cisplatin. Severe hepatotoxicity should be watched for with repeated administration of cisplatin.

Title: Biochemical and histological study of rat liver and kidney injury induced by Cisplatin.
Cisplatin is a chemotherapeutic agent widely used in treatment of several cancers. It is documented as a major cause of clinical nephrotoxicity and hepatotoxicity. The purpose of this study was to investigate the involvement of oxidative stress in the pathogenesis of cisplatin-induced liver and kidney injury. Wistar rats were divided into four groups. Group 1 (control) was intraperitoneally (IP) injected with a single dose of 0.85% normal saline. Groups 2, 3 and 4 were IP injected with single doses of cisplatin at 10, 25 and 50 mg/kg body weight (BW), respectively. At 24, 48, 72, 96 and 120 h after... (truncated)

Title: Silymarin ameliorates cisplatin-induced hepatotoxicity in rats: histopathological and ultrastructural studies.
The benefit of silymarin, a plant extract with strong antioxidant activity against hepatotoxicity induced by cisplatin in rats was investigated in this study. Cisplatin is one of the most effective chemotherapeutic drugs, yet it alone does not achieve a satisfactory therapeutic outcome and at high doses it can produce undesirable side effects. Five equal-sized groups (18 rats each) of male Sprague Dawley rats [control, vehicle; cisplatin; silymarin 2 h after cisplatin injection; and silymarin 2 h before cisplatin injection] were used. Histopathological and ultrastructural observation of livers were carried out using light and electron microscopy. Results documented that cisplatin produced... (truncated)

MESH:D000077588 - deferasirox

Summary:

LLM Prediction Score	0.603 (normalized)
LLM Confidence Score	0.990
Golden Answer (Severity Class)	0.875 (normalized)
Prediction Error	0.272

Retrieved Context:

Title: Efficacy and safety of deferasirox compared with deferoxamine in sickle cell disease: two-year results including pharmacokinetics and concomitant hydroxyurea.
We report a prospective, randomized, Phase II study of deferasirox and deferoxamine (DFO) in sickle cell disease patients with transfusional iron overload, with all patients continuing on deferasirox after 24 weeks, for up to 2 years. The primary objective was to evaluate deferasirox safety compared with DFO; long-term efficacy and safety of deferasirox was also assessed. We also report, for the first time, the safety and pharmacokinetics of deferasirox in patients concomitantly receiving hydroxyurea. Deferasirox (n = 135) and DFO (n = 68) had comparable safety profiles over 24 weeks. Adverse events (AEs) secondary to drug administration were reported in... (truncated)

Title: A randomised comparison of deferasirox versus deferoxamine for the treatment of transfusional iron overload in sickle cell disease.
Deferasirox is a once-daily, oral iron chelator developed for treating transfusional iron overload. Preclinical studies indicated that the kidney was a potential target organ of toxicity. As patients with sickle cell disease often have abnormal baseline renal function, the primary objective of this randomised, open-label, phase II trial was to evaluate the safety and tolerability of deferasirox in comparison with deferoxamine in this population. Assessment of efficacy, as measured by change in liver iron concentration (LIC) using biosusceptometry, was a secondary objective. A total of 195 adult and paediatric patients received deferasirox (n = 132) or deferoxamine (n = 63).... (truncated)

Title: Deferasirox: uncertain future following renal failure fatalities, agranulocytosis and other toxicities.
Cases of fatal, acute, irreversible renal failure and cytopenias, including agranulocytosis and thrombocytopenia, have been disclosed in a postmarketing report on deferasirox, a few months after the European Union authorities and about a year after the FDA proceeded to its accelerated approval. No details on the incidence rate or the cause of these toxicities have yet been reported. Other toxic side effects include skin, gastric, auditory and ocular abnormalities, and hepatitis. Regular serum creatinine, blood counts and other toxicity monitoring as well as withdrawal of deferasirox from the patients affected and those with serum ferritin < 0.5 mg/l was recommended.... (truncated)

Title: Deferasirox induced liver injury in haemochromatosis.
Durg-induced liver injury is a common side-effect of many medicines. It is particularly problem when the original condition under treatment is already causing liver damage. This report describes the hepatotoxicity induced by Deferasirox in a patient with haemochromatosis with a discussion of possible pathogenetic mechanism.

Title: Significant Hyperbilirubinemia and Acute Hepatocellular Jaundice in a Pediatric Patient Receiving Deferasirox: A Case Report.
Despite a boxed warning, postmarketing reports of deferasirox-associated hepatic injury in patients with chronic transfusions are not well described. Hepatic impairment, including failure, has been reported to occur more frequently in patients older than 55 years and in those with significant comorbidities, including liver cirrhosis and multiorgan failure. In this case report, we describe significant hyperbilirubinemia and acute hepatocellular jaundice related to deferasirox in a 7-year-old female being treated for iron overload secondary to chronic transfusions. This report outlines a unique case without preexisting risk factors in which other causes of liver injury are excluded as defined by the Roussel... (truncated)

MESH:D020910 - ketorolac

Summary:

LLM Prediction Score	0.104 (normalized)
LLM Confidence Score	0.990
Golden Answer (Severity Class)	0.375 (normalized)
Prediction Error	0.271

Retrieved Context:

Title: Ketogal Safety Profile in Human Primary Colonic Epithelial Cells and in Mice.
In our previous studies, a ketorolac-galactose conjugate (ketogal) showed prolonged anti-inflammatory and analgesic activity, causing less gastric ulcerogenic effect and renal toxicity than its parent drug ketorolac. In order to demonstrate the safer profile of ketogal compared to ketorolac, histopathological changes in the small intestine and liver using three staining techniques before and after repeated oral administration in mice with ketorolac or an equimolecular dose of its galactosylated prodrug ketogal were assessed. Cytotoxicity and oxidative stress parameters were evaluated and compared in ketorolac- and ketogal-treated Human Primary Colonic Epithelial cells at different concentrations and incubation times. Evidence of mitochondrial oxidative... (truncated)

Title: A comparative study of the analgesic effects of intravenous ketorolac, paracetamol, and morphine in patients undergoing video-assisted thoracoscopic surgery: A double-blind, active-controlled, randomized clinical trial.
Opioids are traditionally used as the drug of choice for the management of postoperative pain. However, their use is limited in patients undergoing Video-assisted thoracic surgery (VATS), due to their side effects, such as respiratory depression, nausea, and vomiting.

Title: Lack of hepatotoxic effects of parenteral ketorolac in the hospital setting.
No large controlled studies to date have examined the hepatic safety of parenteral ketorolac, which is used to treat acutely ill hospitalized patients who may be at greatest risk of liver injury.

Title: Ruptured subcapsular hematoma after laparoscopic cholecystectomy attributed to ketorolac-induced coagulopathy.
Ketorolac is the first injectable nonsteroidal antiinflammatory drug used as an analgesic in the perioperative period. However, gastrointestinal bleeding is a risk associated with its perioperative administration. A 23-year-old woman was admitted for elective laparoscopic cholecystectomy. Her medical history was unremarkable except for a complaint of intermittent right upper quadrant pain for several months. The operative procedure was uneventful. Thirty milligrams of ketorolac were given intravenously just prior to termination of surgery. Eighteen hours after surgery, the patient developed right upper quadrant pain associated with tachycardia and hypotension. Abdominal computed tomography (CT) scan demonstrated a large subcapsular hematoma of the... (truncated)

Title: Recommendations of the national football league physician society task force on the use of toradol(®) ketorolac in the national football league.
Ketorolac tromethamine (Toradol(®)) is a non-steroidal anti-inflammatory drug that has potent analgesic and anti-inflammatory properties. It can be administered orally, intravenously, intramuscularly, or via a nasal route. Ketorolac injections have been used for several years in the National Football League (NFL), in both the oral and injectable forms, to treat musculoskeletal injuries and to prevent post-game soreness. In an attempt to determine the appropriate use of this medication in NFL players, the NFL Team Physician Society appointed a Task Force to consider the best available evidence as to how ketorolac should be used for pain management in professional football players.... (truncated)

MESH:D013467 - sulindac

Summary:

LLM Prediction Score	0.729 (normalized)
LLM Confidence Score	0.980
Golden Answer (Severity Class)	1.0 (normalized)
Prediction Error	0.271

Retrieved Context:

Title: Sulindac hepatotoxicity.
Two patients who developed painless jaundice while taking sulindac are described. Rechallenge in one case confirmed the association of hepatic damage with sulindac administration. Laboratory data and liver biopsy findings suggested a hepatitis with cholestatic features.

Title: Serious adverse reactions associated with sulindac.
Sulindac is a nonsteroidal anti-inflammatory agent that has been associated with serious adverse reactions. We saw four patients with reactions associated with sulindac. Our patients, one of whom died, had high temperatures and involvement of one or more organs, including the skin, liver, CNS, lymph nodes, bone marrow, and lungs. Eight similar previously reported cases also are summarized. In view of these cases of sulindac-induced toxicity, six of which were proved unequivocally by drug rechallenge, we suggest that physicians be cautious in prescribing this agent.

Title: Hepatic toxicity of nonsteroidal anti-inflammatory drugs.
The hepatic toxicity associated with the use of nonsteroidal anti-inflammatory drugs (NSAIDs) is reviewed. NSAIDs include agents in more than 10 classes of compounds, many of which are capable of producing hepatic injury. When NSAIDs are used to treat rheumatic disease, the hepatic effects of the disease itself may complicate the diagnosis of NSAID-induced hepatic injury. Hepatotoxicity caused by drugs may be either intrinsic or idiosyncratic in nature and may be manifested by hepatocellular injury, cholestasis, or a combination of both types of injury. Intrinsic hepatotoxins, such as salicylates, produce injury in a large percentage of exposed individuals that is... (truncated)

Title: Nonsteroidal anti-inflammatory drug-induced hepatotoxicity.
Nonsteroidal anti-inflammatory drugs are among the most common drugs associated with drug-induced liver injury, with an estimated incidence of between 3 and 23 per 100,000 patient years. Nimesulide, sulindac, and diclofenac seem to be associated with the highest risk and the only risk factor consistently identified is the concomitant use of other hepatotoxic drugs. Diclofenac-induced liver injury is a paradigm for drug-related hepatotoxicity. Recent studies suggest that genetic factors favoring the formation and accumulation of the reactive acylglucuronide metabolite of diclofenac and an enhanced immune response to the metabolite-protein adducts are associated with increased susceptibility to hepatotoxicity.

Title: Sulindac metabolism and synergy with tumor necrosis factor-alpha in a drug-inflammation interaction model of idiosyncratic liver injury.
Sulindac (SLD) is a nonsteroidal anti-inflammatory drug (NSAID) that has been associated with a greater incidence of idiosyncratic hepatotoxicity in human patients than other NSAIDs. In previous studies, cotreatment of rats with SLD and a modestly inflammatory dose of lipopolysaccharide (LPS) led to liver injury, whereas neither SLD nor LPS alone caused liver damage. In studies presented here, further investigation of this animal model revealed that the concentration of tumor necrosis factor-alpha (TNF-alpha) in plasma was significantly increased by LPS at 1 h, and SLD enhanced this response. Etanercept, a soluble TNF-alpha receptor, reduced SLD/LPS-induced liver injury, suggesting a role... (truncated)

MESH:D001971 - bromocriptine

Summary:

LLM Prediction Score	0.107 (normalized)
LLM Confidence Score	0.980
Golden Answer (Severity Class)	0.375 (normalized)
Prediction Error	0.268

Retrieved Context:

Title: Neuroleptic malignant syndrome associated with the use of injection zuclopenthixol acetate.
Zuclopenthixol is usually used in parental form to manage acute agitation and psychosis.[1] It has high affinity for dopamine D1 and D2 receptors. There are very few reports of Neuroleptic Malignant Syndrome (NMS) with use of zuclopenthixol monotherapy. In this case report, we present a 35 year old male with alcohol dependence, presented to the emergency with altered sensorium, fever and stiffness of limbs. He had history of receiving Injection Zuclopenthixol acetate 200 mg thrice over 24 hours. Within 12-14 hours of the last injection, patient developed features suggestive of NMS. On investigations he was found to have raised serum... (truncated)

Title: Levodopa + carbidopa + entacapone. Entacapone: a second look: new preparations. Parkinson's disease: a modest effect.
(1) If patients with Parkinson's disease treated with levodopa develop end-of-dose motor fluctuations, the standard therapy is to add bromocriptine, a dopamine receptor agonist, to their ongoing treatment. (2) Evaluation data available in 1999 on entacapone, a catechol-o-methyltransferase (COMT) inhibitor, failed to show whether the balance of benefits versus harm was at least equivalent to that of bromocriptine. (3) Entacapone is now also available as a triple fixed-dose combination with levodopa + carbidopa. (4) Three double-blind trials have compared triple combinations of levodopa + carbidopa (or benserazide) + entacapone with levodopa + carbidopa (or benserazide) + placebo. Two of these... (truncated)

Title: Effects of various drugs on alcohol-induced oxidative stress in the liver.
The major aim of this work was to investigate how alcohol-induced oxidative stress in combined chemotherapy changes the metabolic function of the liver in experimental animals. This research was conducted to establish how bromocriptine, haloperidol and azithromycin, applied to the experimental model, affected the antioxidative status of the liver. The following parameters were determined: reduced glutathione, activities of glutathione peroxidase, glutathione reductase, peroxidase, catalase, xanthine oxidase and lipid peroxidation intensity. Alanine transaminase was measured in serum. Alcohol stress (AO group) reduced glutathione and the activity of xanthine oxidase and glutathione peroxidase, but increased catalase and alanine transaminase activity. The best... (truncated)

Title: Tolcapone: new drug. In Parkinson's disease: unacceptable risk of severe hepatitis.
(1) When patients with Parkinson's disease who are taking levodopa develop motor fluctuations that do not respond to dose adjustments, the standard treatment is the addition of bromocriptine, a dopaminergic agonist. Evaluation of entacapone fails to show whether the risk-benefit balance of this catechol-O-methyltransferase (COMT) inhibitor is at least as favourable as that of bromocriptine. (2) Tolcapone, another COMT inhibitor, is back on the French market after being withdrawn because of serious hepatic effects. The summary of product characteristics (SPC) specifies that tolcapone must only be used when entacapone treatment fails or is poorly tolerated. (3) Renewal of marketing authorisation... (truncated)

Title: Entacapone: new preparation. Comparative data are lacking.
(1) Entacapone, a catechol-O-methyltransferase inhibitor, is indicated, in combination with levodopa + a dopadecarboxylase inhibitor, for the treatment of parkinsonian patients who have motor fluctuations on levodopa therapy. (2) The clinical file contains only placebo-controlled trials. (3) The two main clinical trials involved patients with moderate fluctuations while on levodopa, usually combined with other antiparkinsonian drugs. They showed a moderate effect of entacapone, with an increased duration of motor improvement ("on" periods) of approximately one hour, but one trial showed no increase in overall patient satisfaction. (4) In clinical trials the main adverse effects of entacapone were dyskinesias and gastrointestinal... (truncated)

MESH:D003061 - codeine

Summary:

LLM Prediction Score	0.266 (normalized)
LLM Confidence Score	0.990
Golden Answer (Severity Class)	0.0 (normalized)
Prediction Error	0.266

Retrieved Context:

Title: Analgesics in patients with hepatic impairment: pharmacology and clinical implications.
The physiological changes that accompany hepatic impairment alter drug disposition. Porto-systemic shunting might decrease the first-pass metabolism of a drug and lead to increased oral bioavailability of highly extracted drugs. Distribution can also be altered as a result of impaired production of drug-binding proteins or changes in body composition. Furthermore, the activity and capacity of hepatic drug metabolizing enzymes might be affected to various degrees in patients with chronic liver disease. These changes would result in increased concentrations and reduced plasma clearance of drugs, which is often difficult to predict. The pharmacology of analgesics is also altered in liver disease.... (truncated)

Title: [Conventional techniques for analgesia: opioids and non-opioids. Indications, adverse effects and monitoring].
Morphine dosage must be carefully adapted in patients with renal failure or severe liver failure. The i.v. route is used for morphine titration in the post anaesthesia care unit (PACU), or for analgesia in children. Systematic (not on demand) intramuscular or subcutaneous morphine must be administered at intervals not longer than 4 hours. Dosage is best determined after i.v. titration in the PACU. Codeine, administered orally, is metabolised into morphine. Codeine has almost no effect in 7% of Caucasians and at least 15% of Asians. Nalbuphine, which has a sedative effect and a short half-life, is mainly used in children.... (truncated)

Title: Glucuronidation of paracetamol by human liver microsomes in vitro / enzyme kinetic parameters and interactions with short-chain aliphatic alcohols and opiates.
In this study, glucuronidation of paracetamol (CAS 103-90-2) by human liver microsomes and the effects of aliphatic alcohols and opiates were investigated. Paracetamol glucuronidation was optimised for various incubation conditions. Ten different aliphatic alcohols and the opiates morphine, codeine and dihydrocodeine were analysed as inhibitors of paracetamol glucuronidation. Furthermore, the effects of paracetamol on morphine-3 and codeine glucuronidation were investigated. Enzyme kinetic analysis was carried out via determination of the parameters Km, Vmax, Ki and the type of inhibition. Except for methanol and ethanol, all Investigated alcohols inhibited glucuronidation of paracetamol. Ki values ranged between 4.59 mmol/l (n-pentanol) and 340.54... (truncated)

Title: Can NSAIDs cause acute biliary pain with cholestasis?
Two patients had many acute episodes of biliary pain with elevated liver function tests 12-48 h after the last ingestion of nonsteroidal anti-inflammatory drugs (NSAIDs) (including paracetamol) alone or in combination with codeine. One had known intolerance to NSAIDs, but paracetamol had not been previously incriminated in the pathogenesis of the attacks. In this patient the combined use of paracetamol and codeine probably also increased the severity of the episodes. We conclude that in some patients in whom endoscopic cholangiography is normal, biliary pain and abnormal liver function tests could be the result of NSAIDs. A thorough drug history is... (truncated)

Title: Pharmacological Aspects of Over-the-Counter Opioid Drugs Misuse.
Several over-the-counter (OTC) drugs are known to be misused. Among them are opioids such as codeine, dihydrocodeine, and loperamide. This work elucidates their pharmacology, interactions, safety profiles, and how pharmacology is being manipulated to misuse these common medications, with the aim to expand on the subject outlined by the authors focusing on abuse prevention and prevalence rates. The reviewed literature was identified in several online databases through searches conducted with phrases created by combining the international non-proprietary names of the drugs with terms related to drug misuse. The results show that OTC opioids are misused as an alternative for illicit... (truncated)

MESH:D000638 - amiodarone

Summary:

LLM Prediction Score	0.734 (normalized)
LLM Confidence Score	0.990
Golden Answer (Severity Class)	1.0 (normalized)
Prediction Error	0.266

Retrieved Context:

Title: Amiodarone-Induced Acute Liver Injury.
Amiodarone is a lipophilic structure with a half-life of 25-100 days. Long-term oral amiodarone is associated with photosensitivity, thyroid dysfunction, and pulmonary and hepatic toxicity. Intravenous amiodarone can lead to sweating, heating sensation, nausea, phlebitis at the injection site, and rarely acute hepatitis. This is a compelling case of a 60-year-old male who developed acute liver injury 24-36 h after starting amiodarone. All the possible causes of acute liver injury were ruled out, and his liver enzymes improved after discontinuing amiodarone.

Title: Hepatotoxicity associated with amiodarone therapy.
Amiodarone has been reported to cause asymptomatic increases in liver function tests in 15-55% of patients. Clinically apparent, symptomatic hepatic disease occurs less frequently, but patients have been reported to have hepatomegaly, jaundice, cirrhosis, or chronic active hepatitis. Less well recognized is the fact that amiodarone has been attributed to six deaths. We cared for a patient with amiodarone hepatotoxicity, which led us to review the literature associated with this serious condition.

Title: Prolonged Jaundice Secondary to Amiodarone Use: A Case Report and Literature Review.
Adverse reactions to the antiarrhythmic medication amiodarone are severe, potentially life-threatening, and not rare. One in three patients on long-term therapy experience elevated liver enzymes, and clinically apparent liver toxicity occurs in 1% of patients treated. We report the case of a 76-year-old patient with amiodarone-induced intrahepatic cholestasis and prolonged hyperbilirubinemia despite the discontinuation of the offending agent. Current research hypothesizes that amiodarone leads to hepatic injury both by direct hepatotoxicity and by increasing the likelihood of hepatocytes to create abnormal, toxic metabolites. Increased awareness of such an adverse effect can guide clinicians toward the possible underlying etiologies of prolonged... (truncated)

Title: Amiodarone hepatotoxicity. A clinicopathologic study of five patients.
Five patients had amiodarone hepatotoxicity detected on routine biochemical monitoring. Symptoms attributable to hepatotoxicity were minimal or absent; reversible hepatomegaly was seen in two patients, whereas three patients had signs of nonhepatic amiodarone toxicity before or with hepatotoxicity. Serum aminotransferase levels were elevated in all patients and alkaline phosphatase levels in four; no patient had hyperbilirubinemia or prolongation of the prothrombin time. Light microscopy showed steatosis, cellular degeneration, and cellular necrosis in the biopsy samples of four patients, whereas the fifth patient's sample had a granulomatous injury pattern. Electron microscopic study of liver tissue done in two patients showed phospholipid-laden... (truncated)

Title: Amiodarone hepatotoxicity.
Potential hepatotoxicity related to amiodarone therapy is often a concern when deciding whether to initiate or continue treatment with this medication. While mostly associated with long-term oral administration of the drug, toxicity has also been reported early during intravenous administration and months after discontinuation of therapy. In the majority of patients, it is discovered incidentally during routine testing of liver biochemistry and rarely do the hepatic effects develop into symptomatic liver injury or failure. Despite the widespread use of amiodarone, prospective clinical studies have been sparse and there has been little consensus among experts in the field regarding optimum monitoring... (truncated)

MESH:D010646 - phentolamine

Summary:

LLM Prediction Score	0.265 (normalized)
LLM Confidence Score	0.960
Golden Answer (Severity Class)	0.0 (normalized)
Prediction Error	0.265

Retrieved Context:

Title: Norepinephrine released by intestinal Paneth cells exacerbates ischemic AKI.
Small intestinal Paneth cells play a critical role in acute kidney injury (AKI) and remote organ dysfunction by synthesizing and releasing IL-17A. In addition, intestine-derived norepinephrine is a major mediator of hepatic injury and systemic inflammation in sepsis. We tested the hypothesis that small intestinal Paneth cells synthesize and release norepinephrine to exacerbate ischemic AKI. After ischemic AKI, we demonstrated larger increases in portal venous norepinephrine levels compared with plasma norepinephrine in mice, consistent with an intestinal source of norepinephrine release after renal ischemia and reperfusion. We demonstrated that murine small intestinal Paneth cells express tyrosine hydroxylase mRNA and protein,... (truncated)

Title: Adrenergic regulation during acute hepatic infection with Entamoeba histolytica in the hamster: involvement of oxidative stress, Nrf2 and NF-KappaB.
Oxidative stress and transcriptional pathways of nuclear factor erythroid 2-related factor 2 (Nrf2) and nuclear factor kappa-B (NF-κB) are critically involved in the etiopathology of amebic liver abscess (ALA). In this work, we studied the relationship between the adrenergic nervous system and ALA in the hamster. ALA was visible at 12 h of infection. While 6-hydroxidopamine (6-OHDA) decreased infection, propranolol (β-adrenergic blocker) treatment was associated with less extensive liver damage, and phentolamine treatment (α-adrenergic blocker) significantly reduced ALA compared to 6-OHDA and propranolol. Serum enzymatic activities of alanine aminotransferase (ALT) and γ-glutamyl transpeptidase (γ-GTP) were increased at 12 h post-infection.... (truncated)

Title: Side effects of self-administration of intracavernous papaverine and phentolamine for the treatment of impotence.
Beginning October 1985, 111 men agreed to enter a prospective study of the side effects of low dose papaverine/phentolamine therapy. A total of 46 men dropped out, 30 during the initial phase. The percentage of men with painless nodules almost consistently doubled from one followup examination to the next: 8 per cent at 1 month, 17 per cent at 3 months, 32 per cent at 6 months and 57 per cent at 12 months. The average injection frequency of those with nodules was 2 1/2 times higher than those without nodules. Of the men 41 per cent required an increased... (truncated)

Title: The treatment of pheochromocytoma associated with pseudo-obstruction and perforation of the colon, hepatic failure, and DIC.
The case of a 59-year-old man with paralytic ileus (pseudo-obstruction) associated with pheochromocytoma is reviewed. Paralytic ileus is believed to have been the result of overstimulation of alpha and beta receptors on the intestine by catecholamines. Phentolamine, bunazocin, propranolol, bethanechol and midaglizole in single administrations or in combination were administered. Phentolamine infusion clearly relieved the symptom, but ileus recurred, and the patient died of respiratory failure, liver dysfunction and disseminated intravascular coagulation syndrome. The significant role of catecholamines in causing these symptoms is discussed, and the management of this relatively rare complication is reviewed.

Title: Hepatotoxicity related to intracavernous pharmacotherapy with papaverine.
To determine the incidence of hepatotoxicity related to self-administration of intracavernous papaverine or papaverine/phentolamine (bimix).

MESH:D065819 - voriconazole

Summary:

LLM Prediction Score	0.735 (normalized)
LLM Confidence Score	0.990
Golden Answer (Severity Class)	1.0 (normalized)
Prediction Error	0.265

Retrieved Context:

Title: Rechallenge of voriconazole successfully tolerated after hepatic toxicity.
Infections caused by Aspergillus species are often life-threatening. Drugs effective for Aspergillus infection are limited. Voriconazole is one of the most important drugs, however, considerable portion of patients experience liver toxicity and have to stop the drug administration. We frequently experience liver toxicity even though the serum concentration of voriconazole is within the target range. Historically, in some life-threatening situations like tuberculosis, where a suitable alternative is unavailable, rechallenge has been attempted. However, there have been no report on the rechallenge of voriconazole. We report cases of successful re-administration of voriconazole after liver toxicity.

Title: Voriconazole-Induced Hepatotoxicity Presenting With Severe Hepatic Encephalopathy After Liver Transplantation.
Voriconazole-induced hepatotoxicity is a relatively rare but serious clinicopathologic entity. This drug is frequently used for invasive aspergillosis and other fungal infections. We report a patient with alcoholic cirrhosis who developed hepatic encephalopathy due to voriconazole administered for invasive pulmonary aspergillosis and subsequently showed marked improvement in mental status with dose adjustment of the drug. The patient eventually underwent an uneventful liver transplant. Histopathologic examination of the diseased liver specimen revealed numerous rhomboid-shaped crystals, deemed secondary to liver injury after voriconazole-induced hepatotoxicity. Additionally, this article briefly reviews the available data on voriconazole-induced hepatotoxicity with special emphasis on plasma drug concentration... (truncated)

Title: Voriconazole hepatotoxicity in severe liver dysfunction.
There are no studies regarding to these effects in patients with severe liver dysfunction.

Title: Metabolomics analysis of plasma reveals voriconazole-induced hepatotoxicity is associated with oxidative stress.
Voriconazole is one of the most frequently used antifungal drugs for the initial treatment of invasive aspergillosis, but liver-related adverse events occur frequently and usually lead to drug discontinuation. Moreover, the mechanism of voriconazole-induced hepatotoxicity remains unsettled. A holistic understanding of its mechanism is critical to prevent liver-related adverse events. Metabolomics has been demonstrated to be a helpful strategy for investigating drug-induced toxicity. This study aimed to utilize human plasma samples to investigate the mechanism of voriconazole-induced hepatotoxicity through a metabolomics approach. Patients that were administered voriconazole were classified into a voriconazole-induced hepatotoxicity group and control group (n = 65,... (truncated)

Title: Posaconazole achieves prompt recovery of voriconazole-induced liver injury in a case of invasive aspergillosis.
Azole antifungals have frequently been linked to the presence of hepatotoxicity, but there is scarce information on cross-toxicity between these drugs or on the possibility of using some of them when this type of toxicity occurs. We report the case of a 64-year-old man with invasive aspergillosis (IA) leading to spondylodiscitis with neurological involvement. Early management included intravenous (iv) voriconazole, which had to be interrupted after 1 week due to liver damage. Therapeutic drug monitoring (TDM) of voriconazole showed that the plasma concentration was within the therapeutic range. However, it was replaced by a combination therapy of oral posaconazole plus... (truncated)

MESH:D001418 - baclofen

Summary:

LLM Prediction Score	0.110 (normalized)
LLM Confidence Score	0.990
Golden Answer (Severity Class)	0.375 (normalized)
Prediction Error	0.265

Retrieved Context:

Title: Multidisciplinary View of Alcohol Use Disorder: From a Psychiatric Illness to a Major Liver Disease.
Alcohol use disorder is a significant health problem being a cause of increased morbidity and mortality worldwide. Alcohol-related illness has a relevant economic impact on the society and a negative influence on the life of patients and their family members. Psychosocial support might be useful in the management of people affected by alcohol use disorder since psychiatric and pharmaceutical approaches show some limits. In fact, many drugs are accessible for the treatment of alcohol disorder, but only Baclofen is functional as an anti-craving drug in patients with advanced liver disease. The alcohol-related liver damage represents the most frequent cause of... (truncated)

Title: Baclofen therapeutics, toxicity, and withdrawal: A narrative review.
Baclofen is an effective therapeutic for the treatment of spasticity related to multiple sclerosis, spinal cord injuries, and other spinal cord pathologies. It has been increasingly used off-label for the management of several disorders, including musculoskeletal pain, gastroesophageal reflux disease, and alcohol use disorder. Baclofen therapy is associated with potential complications, including life-threatening toxicity and withdrawal syndrome. These disorders require prompt recognition and a high index of suspicion. While these complications can develop following administration of either oral or intrathecal baclofen, the risk is greater with the intrathecal route. The management of baclofen toxicity is largely supportive while baclofen withdrawal... (truncated)

Title: Baclofen for the Treatment of Alcohol Use Disorder in Patients With Liver Cirrhosis: 10 Years After the First Evidence.
Alcohol Use Disorder (AUD) is a chronic and relapsing condition characterized by harmful alcohol intake and behavioral-cognitive changes. AUD is the most common cause of liver disease in the Western world. Alcohol abstinence is the cornerstone of therapy in alcoholic patients affected with liver disease. Medical recommendations, brief motivational interventions and psychosocial approach are essential pieces of the treatment for these patients; however, their efficacy alone may not be enough to achieve total alcohol abstinence. The addition of pharmacological treatment could improve clinical outcomes in AUD patients. Moreover, pharmacological treatments for AUD are limited in patients with advanced liver disease,... (truncated)

Title: Management of alcohol dependence in patients with liver disease.
Alcohol dependence represents a chronic and relapsing disease affecting nearly 10 % of the general population both in the USA and in Europe, with a widespread burden of morbidity and mortality. Alcohol dependence represents the most common cause of liver damage in the Western world. Although alcoholic liver disease is associated primarily with heavy drinking, continued alcohol consumption, even in low doses after the onset of liver disease, increases the risk of severe consequences, including mortality. Consequently, the ideal treatment of patients affected by alcohol dependence and alcoholic liver disease should aim at achieving long-term total alcohol abstinence and preventing... (truncated)

Title: Baclofen promotes alcohol abstinence in alcohol dependent cirrhotic patients with hepatitis C virus (HCV) infection.
Hepatitis C virus (HCV) and alcoholic liver disease (ALD), either alone or in combination, count for more than two thirds of all liver diseases in the Western world. There is no safe level of drinking in HCV-infected patients and the most effective goal for these patients is total abstinence. Baclofen, a GABA(B) receptor agonist, represents a promising pharmacotherapy for alcohol dependence (AD). Previously, we performed a randomized clinical trial (RCT), which demonstrated the safety and efficacy of baclofen in patients affected by AD and cirrhosis. The goal of this post-hoc analysis was to explore baclofen's effect in a subgroup of... (truncated)

MESH:D006879 - hydroxocobalamin

Summary:

LLM Prediction Score	0.112 (normalized)
LLM Confidence Score	0.990
Golden Answer (Severity Class)	0.375 (normalized)
Prediction Error	0.263

Retrieved Context:

Title: Pernicious Anemia Associated Cobalamin Deficiency and Thrombotic Microangiopathy: Case Report and Review of the Literature.
A 43-year-old Hispanic male without significant previous medical history was brought to emergency department for syncope following a blood draw to investigate a 40 lbs weight loss during the past 6 months associated with decreased appetite and progressive fatigue. The patient also reported a 1-month history of jaundice. On examination, he was hemodynamically stable and afebrile with pallor and diffuse jaundice but without skin rash or palpable purpura. Normal sensations and power in all extremities were evident on neurological exam. Presence of hemolytic anemia, schistocytosis, thrombocytopenia, and elevated lactate dehydrogenase (LDH) was suggestive of thrombotic thrombocytopenic purpura (TTP). However, presence... (truncated)

Title: Pharmacological treatment of inhalation injury after nuclear or radiological incidents: The Chinese and German approach.
Inhalation injury is often associated with burns and significantly increases morbidity and mortality. The main toxic components of fire smoke are carbon monoxide, hydrogen cyanide, and irritants. In the case of an incident at a nuclear power plant or recycling facility associated with fire, smoke may also contain radioactive material. Medical treatments may vary in different countries, and in this paper, we discuss the similarities and differences in the treatments between China and Germany. Carbon monoxide poisoning is treated by 100% oxygen administration and, if available, hyperbaric oxygenation in China as well as in Germany. In addition, antidotes binding the... (truncated)

Title: Ornipural® Mitigates Malathion-Induced Hepato-Renal Damage in Rats via Amelioration of Oxidative Stress Biomarkers, Restoration of Antioxidant Activity, and Attenuation of Inflammatory Response.
The current study was instigated by investigating the ameliorative potential of Ornipural® solution against the hepato-renal toxicity of malathion. A total number of 35 male Wistar albino rats were divided equally into five groups. Group 1 served as control and received normal saline intraperitoneally. Group 2, the sham group, were administered only corn oil (vehicle of malathion) orally. Group 3 was orally intoxicated by malathion in corn oil at a dose of 135 mg/kg BW via intra-gastric gavage. Group 4 received malathion orally concomitantly with Ornipural® intraperitoneally. Group 5 was given Ornipural® solution in saline via intraperitoneal injection at a... (truncated)

Title: Hydroxocobalamin-Induced Oxalate Nephropathy in a Patient With Smoke Inhalation.
No abstract available.

Title: A New, Atypical Case of Cobalamin F Disorder Diagnosed by Whole Exome Sequencing.
Cobalamin F (cblF) disorder, caused by homozygous or compound heterozygous mutations in the LMBRD1 gene, is a recognised cause of developmental delay, pancytopaenia and failure to thrive which may present in the neonatal period. A handful of cases have been reported in the medical literature. We report a new case, diagnosed at the age of 6 years through whole exome sequencing, with atypical features including prominent metopic suture, cleft palate, unilateral renal agenesis and liver abnormalities, which broaden the phenotypic spectrum.

MESH:D004176 - dipyridamole

Summary:

LLM Prediction Score	0.113 (normalized)
LLM Confidence Score	0.980
Golden Answer (Severity Class)	0.375 (normalized)
Prediction Error	0.262

Retrieved Context:

Title: Equilibrative nucleoside transporter (ENT)-1-dependent elevation of extracellular adenosine protects the liver during ischemia and reperfusion.
Ischemia and reperfusion-elicited tissue injury contributes to morbidity and mortality of hepatic surgery and during liver transplantation. Previous studies implicated extracellular adenosine signaling in liver protection. Based on the notion that extracellular adenosine signaling is terminated by uptake from the extracellular towards the intracellular compartment by way of equilibrative nucleoside transporters (ENTs), we hypothesized a functional role of ENTs in liver protection from ischemia. During orthotopic liver transplantation in humans, we observed higher expressional levels of ENT1 than ENT2, in conjunction with repression of ENT1 and ENT2 transcript and protein levels following warm ischemia and reperfusion. Treatment with the pharmacologic... (truncated)

Title: The effect of eplerenone on adenosine formation in humans in vivo: a double-blinded randomised controlled study.
It has been suggested that mineralocorticoid receptor antagonists have direct cardioprotective properties, because these drugs reduce mortality in patients with heart failure. In murine models of myocardial infarction, mineralocorticoid receptor antagonists reduce infarct size. Using gene deletion and pharmacological approaches, it has been shown that extracellular formation of the endogenous nucleoside adenosine is crucial for this protective effect. We now aim to translate this finding to humans, by investigating the effects of the selective mineralocorticoid receptor antagonist eplerenone on the vasodilator effect of the adenosine uptake inhibitor dipyridamole, which is a well-validated surrogate marker for extracellular adenosine formation.

Title: Palmitate increases the susceptibility of cells to drug-induced toxicity: an in vitro method to identify drugs with potential contraindications in patients with metabolic disease.
Fatty acids are an important source of energy. Excessive energy intake results in elevated levels of free fatty acids that are thought to be the pathogenic factors causing metabolic disorders such as dyslipidemia, obesity, insulin resistance, diabetes, and fatty liver. Underlying metabolic disorders have been suggested to be a predisposing factor for drug-induced liver injury. The steadily expanding population with metabolic disease may pose a higher risk for drug-induced toxicity. In order to understand the interaction of free fatty acids and drug-induced toxicity at the cellular level, we explored whether the saturated free fatty acid palmitate could modulate drug-induced cytotoxicity... (truncated)

Title: Agranulocytosis and hepatic toxicity with ticlopidine therapy: a case report.
Ticlopidine is a platelet inhibitor used to prevent thrombosis in patients with cerebrovascular or coronary artery disease. The most common side effects are mild and transitory: diarrhea, dyspepsia, nausea and rashes. More serious, but less frequent, adverse effects are hematological dyscrasia and cholestatic hepatitis. We report a rare case of agranulocytosis associated with hepatic toxicity, probably related to the use of ticlopidine.

Title: A novel pipeline of 2-(benzenesulfonamide)-N-(4-hydroxyphenyl) acetamide analgesics that lack hepatotoxicity and retain antipyresis.
Although acetaminophen (ApAP) is one of the most commonly used medicines worldwide, hepatotoxicity is a risk with overdose or in patients with compromised liver function. ApAP overdose is the most common cause of acute fulminant hepatic failure. Oxidation of ApAP to N-acetyl-p-benzoquinone imine (NAPQI) is the mechanism for hepatotoxicity. 1 is a non-hepatotoxic, metabolically unstable lipophilic ApAP analog that is not antipyretic. The newly synthesized 3 is a non-hepatotoxic ApAP analog that is stable, lipophilic, and retains analgesia and antipyresis. Intraperitoneal or po administration of the new chemical entities (NCEs), 3b and 3r, in concentrations equal to a toxic dose... (truncated)

MESH:D015737 - nisoldipine

Summary:

LLM Prediction Score	0.115 (normalized)
LLM Confidence Score	0.940
Golden Answer (Severity Class)	0.375 (normalized)
Prediction Error	0.260

Retrieved Context:

Title: Influence of Inflammation on Cytochromes P450 Activity in Adults: A Systematic Review of the Literature.
Background: Available in-vitro and animal studies indicate that inflammation impacts cytochromes P450 (CYP) activity <i>via</i> multiple and complex transcriptional and post-transcriptional mechanisms, depending on the specific CYP isoforms and the nature of inflammation mediators. It is essential to review the current published data on the impact of inflammation on CYP activities in adults to support drug individualization based on comorbidities and diseases in clinical practice. Methods: This systematic review was conducted in PubMed through 7th January 2021 looking for articles that investigated the consequences of inflammation on CYP activities in adults. Information on the source of inflammation, victim drugs (and... (truncated)

Title: Pharmacokinetics of calcium antagonists under development.
Calcium antagonist drugs under clinical development are of the Type I (verapamil, diltiazem-like) and Type II (nifedipine-like) classes. Tiapamil, the only Type I drug currently available, is a high clearance, widely distributed drug which undergoes extensive presystemic elimination. Pharmacokinetically it is quite similar to verapamil; however, it does have increased biliary excretion and decreased binding to plasma proteins. Eight Type II (dihydropyridine) drugs are reviewed. Seven of these drugs (felodipine, isradipine, nicardipine, nilvadipine, nimodipine, nisoldipine and nitrendipine) are pharmacokinetically similar to nifedipine, with high clearance, extensive distribution, and significant presystemic elimination. Amlodipine has lower clearance, even greater peripheral distribution, and... (truncated)

Title: Stereoselective pharmacokinetics of dihydropyridine calcium antagonists.
Many dihydropyridine calcium antagonists are widely used for the treatment of angina and hypertension, and many more are under development. Most of these drugs have one or more chiral centre, and the pharmacological activity between the enantiomers for these drugs is known to be markedly different. First, the stereospecific assay methods for these drugs in plasma or serum are reviewed with emphasis on chiral stationary phase high-performance liquid chromatography for their determination. Next, the stereoselective pharmacokinetics of these drugs (nilvadipine, nitrendipine, felodipine, nimodipine, manidipine, benidipine and nisoldipine) in animals, healthy subjects and patients with hepatic disease is reviewed. Enantiomer-enantiomer interaction,... (truncated)

Title: Interactions between Food and Drugs, and Nutritional Status in Renal Patients: A Narrative Review.
Drugs and food interact mutually: drugs may affect the nutritional status of the body, acting on senses, appetite, resting energy expenditure, and food intake; conversely, food or one of its components may affect bioavailability and half-life, circulating plasma concentrations of drugs resulting in an increased risk of toxicity and its adverse effects, or therapeutic failure. Therefore, the knowledge of these possible interactions is fundamental for the implementation of a nutritional treatment in the presence of a pharmacological therapy. This is the case of chronic kidney disease (CKD), for which the medication burden could be a problem, and nutritional therapy plays... (truncated)

Title: Effectiveness of itraconazole on clinical symptoms and radiologic findings in patients with recurrent chronic rhinosinusitis and nasal polyposis.
This study was done to evaluate the effect of itraconazole on clinical symptoms and radiologic findings in patients with chronic rhinosinusitis and nasal polyposis after surgery.

MESH:D014044 - tolbutamide

Summary:

LLM Prediction Score	0.510 (normalized)
LLM Confidence Score	0.960
Golden Answer (Severity Class)	0.25 (normalized)
Prediction Error	0.260

Retrieved Context:

Title: A case of chronic liver disease due to tolazamide.
Although chlorpropamide and tolbutamide are well recognized as causes of hepatotoxicity, there are only 3 reported cases of hepatic injury caused by a third oral hypoglycemic agent, tolazamide. In 2 of these cases, the liver-function tests returned to normal when the drug was discontinued. In the third case, the patient had cholestasis from chlorpropamide before administration of tolazamide and developed chronic liver disease. We are reporting the second instance of chronic liver disease induced by tolazamide. Our patient had been taking chlorpropamide, but she had no evidence of liver disease before administration of tolazamide. Tolazamide should be considered as a... (truncated)

Title: Favorable effects of glibenclamide in a patient exhibiting idiosyncratic hepatotoxic reactions to both chlorpropamide and tolbutamide.
A middle-aged diabetic woman after four weeks of chlorpropamide treatment developed cholestatic hepatitis with systemic manifestations of idiosyncratic reaction. After recovery, unintended rechallenge with the same drug induced a brisk exacerbation of the symptoms and signs that reversed completely following chlorpropamide withdrawal. Tolbutamide medication was subsequently well tolerated for several weeks, followed by another flare of cholestatic liver lesion and cutaneous eruption with eosinophilia (after each reaction the patient was treated with insulin). Eventually glibenclamide (glyburide) was instituted resulting in very satisfactory control of diabetes, with no untoward reaction.

Title: Serum aminotransferase activity as a predictor of clearance of drugs metabolized by CYP isoforms in rats with acute hepatic failure induced by carbon tetrachloride.
The values of serum aminotransferase activity (AST) in untreated rats and rats with acute hepatic failure at 24h after an oral administration of CCl(4) (0.5 ml/kg) were 85+/-9 IU/l and 4260+/-620 IU/l (mean+/-S.D., n=6), respectively. The values of total clearance (CL(tot)) after intravenous administration of caffeine, tolbutamide, chlorzoxazone or lidocaine (as probe drugs for various CYP isoforms) to CCl(4)-treated rats were decreased to about 1/8, 1/3, 1/3 or 1/2 compared with those in untreated rats. Good correlations were observed between mRNA expression and enzyme activity of CYP2C11, CYP2E1, CYP3A2 and CYP1A2 in livers of rats given various doses of CCl(4).... (truncated)

Title: Differential Effects of 1α,25-Dihydroxyvitamin D₃ on the Expressions and Functions of Hepatic CYP and UGT Enzymes and Its Pharmacokinetic Consequences In Vivo.
The compound 1α,25-Dihydroxyvitamin D₃ (1,25(OH)₂D₃) is the active form of vitamin D₃ and a representative ligand of the vitamin D receptor (VDR). Previous studies have described the impacts of 1,25(OH)₂D₃ on a small number of cytochrome P450 (CYP) and uridine diphosphate-glucuronyltransferase (UGT) enzymes, but comparatively little is known about interactions between several important CYP and UGT isoforms and 1,25(OH)₂D₃ in vitro and/or in vivo. Thus, we investigated the effects of 1,25(OH)₂D₃ on the gene and protein expressions and functional activities of selected CYPs and UGTs and their impacts on drug pharmacokinetics in rats. The mRNA/protein expressions of Cyp2b1 and Cyp2c11... (truncated)

Title: A correlation between the in vitro drug toxicity of drugs to cell lines that express human P450s and their propensity to cause liver injury in humans.
Drug toxicity to T-antigen-immortalized human liver epithelial (THLE) cells stably transfected with plasmid vectors that encoded human cytochrome P450s 1A2, 2C9, 2C19, 2D6, or 3A4, or an empty plasmid vector (THLE-Null), was investigated. An automated screening platform, which included 1% dimethyl sulfoxide (DMSO) vehicle, 2.7% bovine serum in the culture medium, and assessed 3-(4,5-dimethylthiazol-2-yl)-5-(3-carboxymethoxyphenyl)-2-(4-sulfophenyl)-2H-tetrazolium reduction, was used to evaluate the cytotoxicity of 103 drugs after 24h. Twenty-two drugs caused cytotoxicity to THLE-Null cells, with $EC_{50} \leq 200$ μM; 21 of these drugs (95%) have been reported to cause human liver injury. Eleven drugs exhibited lower EC_{50} values in cells

MESH:D009525 - niacin

Summary:

LLM Prediction Score	0.615 (normalized)
LLM Confidence Score	0.990
Golden Answer (Severity Class)	0.875 (normalized)
Prediction Error	0.260

Retrieved Context:

Title: Hypercholesterolemia. Use of niacin and niacin combinations in therapy.
Niacin or niacin combinations were administered in dosage of 1.0 to 6.0 gm. to 31 hypercholesterolemic patients for periods up to three years. Eighty per cent were able to continue medication for long periods without significant side effects. Jaundice, apparently due to nicotinic acid, occurred in one patient. Liver toxicity will probably be a hazard in the use of this therapy. Significant and maintained serum cholesterol depression was achieved in 80 per cent of the patients who were able to take adequate dosage. Reduction in xanthomata was observed with cholesterol reduction. In some cases, when larger doses were not tolerated... (truncated)

Title: Acute liver failure secondary to niacin toxicity.
A 17-year-old male was transferred to the pediatric intensive care unit for evaluation of acute liver failure. He was recently released from an alcohol treatment center with acute onset of chest pain. Cardiac workup was negative but he was found to have abnormal coagulation studies and elevated liver transaminases. Other evaluations included a normal toxicology screen and negative acetaminophen level. Autoimmune and infectious workups were normal providing no identifiable cause of his acute liver failure. He initially denied any ingestions or illicit drug use but on further query he admitted taking niacin in an attempt to obscure the results of... (truncated)

Title: New developments in the use of niacin for treatment of hyperlipidemia: new considerations in the use of an old drug.
Niacin has been used for many years to treat hyperlipidemia. It has been shown to reduce coronary death and non-fatal myocardial infarction and, in a separate analysis of long-term (15-year) follow-up, all cause mortality. It reduces total cholesterol, low density lipoprotein cholesterol (LDL-C) and triglycerides and increases high density lipoprotein cholesterol (HDL-C). Sustained-release niacin may be associated with more dramatic changes in LDL-C and triglyceride, whereas the short acting preparation causes greater increases in HDL-C. The increase of HDL-C occurs at a lower dose (1500 mg/day) than the reduction of LDL-C (> 1500 mg/day). Niacin also favorably influences other lipid... (truncated)

Title: Safety considerations with niacin therapy.
Niacin has beneficial effects on plasma lipoproteins and has demonstrated clinical benefits in reducing cardiovascular events and atherosclerosis progression. The side effects of niacin, however, have limited its use in general clinical practice. An understanding of cutaneous flushing based on the best available evidence should enhance patient education efforts and improve adherence. Although serious hepatic toxicity from niacin administration has been reported, it is largely confined to the use of slow-release formulations given as unregulated nutritional supplements. Niacin has been shown to induce insulin resistance in short-term trials, but the glycemic response in subjects with and without diabetes is usually... (truncated)

Title: Niacin as antidyslipidemic drug.
Niacin is an important vitamin (B3) that can be used in gram doses to positively modify pathogenetically relevant lipid disorders: elevated LDL cholesterol, elevated non-HDL cholesterol, elevated triglycerides, elevated lipoprotein(a), and reduced HDL cholesterol. This review reports the latest published findings with respect to niacin's mechanisms of action on these lipids and its anti-inflammatory and anti-atherosclerotic effects. In the pre-statin era, niacin was shown to have beneficial effects on cardiovascular end-points; but in recent years, two major studies performed in patients whose LDL cholesterol levels had been optimized by a statin therapy did not demonstrate an additional significant effect on... (truncated)

MESH:D002439 - cefotaxime

Summary:

LLM Prediction Score	0.365 (normalized)
LLM Confidence Score	0.980
Golden Answer (Severity Class)	0.625 (normalized)
Prediction Error	0.260

Retrieved Context:

Title: [Spontaneous bacterial peritonitis].
We describe five patients with spontaneous bacterial peritonitis. The condition is reported more frequently than before and survival has improved. Important clinical features are increasing ascites and unexpected derangement of liver function. Possible predisposing factors, as well as diagnostic and therapeutic measures, are discussed. We emphasize the significance of ascitic polymorph nuclear cell count and bedside inoculation of ascites on blood culture medium, and stress the importance of prompt antibiotic therapy. The choice of empiric antimicrobial treatment is based on the reported frequency of causative agent and toxicity to drugs. Our experience so far indicates that cefotaxime administered as monotherapy... (truncated)

Title: A hunt for the source of sepsis.
A 42-year-old woman presented with a 2-day history of drowsiness, confusion, worsening headache, high fevers, urticarial rash, bilateral leg pains and urinary retention. Preceding this was a 1-month history of headache unresponsive to various analgesics for which her general practitioner started carbamazepine. Suspected central nervous system infection was investigated and treated with cefotaxime. A full septic screen, lumbar puncture and MRI of the spine were all inconclusive. After 3 days, the patient deteriorated and repeated blood tests-initially unremarkable-revealed neutropaenia and acutely deranged liver function. Connective tissue disorder was considered due to a negative septic screen and lack of response to... (truncated)

Title: Optimal dose of cefotaxime in neonates with early-onset sepsis: A developmental pharmacokinetic model-based evaluation.
Objective: The perspective of real-world study is especially relevant to newborns, enabling dosage regimen optimization and regulatory approval of medications for use in newborns. The aim of the present study was to conduct a pharmacokinetic analysis of cefotaxime and evaluate the dosage used in newborns with early-onset sepsis (EOS) using real-world data in order to support the rational use in the clinical practice. Methods: This prospective, open-label study was performed in newborns with EOS. A developmental pharmacokinetic-pharmacodynamic model of cefotaxime in EOS patients was established based on an opportunistic sampling method. Then, clinical evaluation of cefotaxime was conducted in newborns... (truncated)

Title: [Therapeutic effect of ceftizoxime on severe infectious complications in blood disorders. Tohkai Research Group on Infections in Hematopoietic Disorders].
Ceftizoxime (CZX) was given by intravenous injection in daily doses of 2-8 g to 103 patients with severe infections complicating hematopoietic disorders. The clinical effect was evaluated in 95 of the 103 patients. The causative organisms were identified in 22 patients but were unknown in the remaining 73. Infected sites were the respiratory tract, urinary tract, soft tissue, and blood. The overall effectiveness rate (inclusive of marked and moderate) was 61.1% (58/95). The effectiveness rate was 63.6% (14/22) in patients in whom the causative organisms were identified and 60.3% (44/73) in patients in whom the causative organisms could not be... (truncated)

Title: Comparative toxicities of third-generation cephalosporins.
Data on the adverse effects experienced by 2,539 patients who received ceftazidime were compared with adverse effects reported with cefoperazone, cefotaxime, ceftizoxime, and moxalactam. There were 216 such reactions among the ceftazidime-treated patients; 158 patients (6.2 percent) had reactions that were possibly or probably drug-related. The clinical and laboratory safety profile of ceftazidime in regard to renal, hepatic, hematopoietic, and hemostatic parameters compared favorably with that of other third-generation cephalosporins. An increased serum creatinine level was observed in 0.8 percent of ceftazidime-treated patients, an increased blood urea nitrogen level in 1.6 percent, hepatic abnormalities in approximately 6 percent, diarrhea in... (truncated)

MESH:D008750 - methyldopa

Summary:

LLM Prediction Score	0.742 (normalized)
LLM Confidence Score	0.980
Golden Answer (Severity Class)	1.0 (normalized)
Prediction Error	0.258

Retrieved Context:

Title: Patterns of hepatic injury induced by methyldopa.
Twelve patients with liver disease related to methyldopa were seen between 1967 and 1977. Illness occurred within 1--9 weeks of commencement of therapy in 9 patients, the remaining 3 patients having received the drug for 13 months, 15 months and 7 years before experiencing symptoms. Jaundice with tender hepatomegaly, usually preceded by symptoms of malaise, anorexia, nausea and vomiting, and associated with upper abdominal pain, was an invariable finding in all patients. Biochemical liver function tests indicated hepatocellular necrosis and correlated with histopathological evidence of hepatic injury, the spectrum of which ranged from fatty change and focal hepatocellular necrosis to... (truncated)

Title: Morphologic alterations in patients with alpha-methyldopa-induced liver damage after short- and long-term exposure.
Alpha-methyldopa-induced histologic alterations were investigated in 21 patients with hepatic injury after short- and long-term exposure. Seven patients developed liver injury within 6 months and 24 after several years (mean, 5 years) of exposure. Histologic findings and clinical and biochemical data differed significantly in the two groups. Morphologic analysis of the short-term-treated group revealed marked parenchymatous degeneration, focal, confluent and massive necrosis, and inflammation. Fatty accumulation and increased fibrous trabeculae were characteristic for the patients treated for long term. All patients in the short-term-exposed group had acute and severe hepatitis. Four of them had icterus. Two patients died of hepatic... (truncated)

Title: Methyldopa Hepatitis. A report of six cases and review of the literature.
Six cases of methyldopa hepatitis, including two in which the patients died are reported; and 77 cases from the literature are reviewed. Patients in whom severe hepatotoxic reactions to methyldopa develop usually complain of prodromal symptoms typical of hepatitis, often with fever, one to four weeks after therapy is initiated. Jaundice, when it occurs, is usually manifest within three months. Asymptomatic, transient elevations of serum transaminase levels may occur in patients receiving methyldopa. However, since the clinical and histologic features of hepatic injury from methyldopa are indistinguishable from viral hepatitis, it is suggested that the incidence of this iatrogenic disease... (truncated)

Title: Methyldopa: an often overlooked cause of fever and transient hepatocellular dysfunction.
The clinical features and laboratory findings of 78 cases of methyldopa fever are reported. This drug reaction masqueraded as a variety of acute infectious diseases including septicaemia, meningitis, hepatitis and gastroenteritis, occurred within five weeks of starting the drug and appeared to be unrelated to its dosage. Eosinophilia and skin rashes were conspicuous by their absence. In the majority of patients, symptoms were relieved within 48 hours of the withdrawal of the drug. Sixty-one per cent of patients had biochemical evidence of liver damage but jaundice was uncommon. This pattern of mild hepatotoxicity in patients with early febrile reactions to... (truncated)

Title: α-Methyldopa-induced hepatitis during the postpartum period.
A 34-year-old woman, with a history of pre-eclampsia, was diagnosed with α-methyldopa-induced hepatotoxicity, after she presented with severe jaundice and hepatitis 8 weeks following delivery. Laboratory investigations and liver biopsy ruled out other causes of hepatitis. She continued to improve clinically after cessation of α-methyldopa, and was discharged 10 days after admission. This case report emphasises that it may not be possible to predict which patients may develop α-methyldopa-induced hepatitis, hence regular monitoring of liver function tests during treatment should be implemented.

MESH:D004157 - diphenoxylate

Summary:

LLM Prediction Score	0.257 (normalized)
LLM Confidence Score	0.980
Golden Answer (Severity Class)	0.0 (normalized)
Prediction Error	0.257

Retrieved Context:

Title: Practical Guidance for the Management of Adverse Events in Patients with KRASG12C-Mutated Non-Small Cell Lung Cancer Receiving Adagrasib.
Adagrasib (MRTX849) is a KRASG12C inhibitor with favorable properties, including long half-life (23 h), dose-dependent pharmacokinetics, and central nervous system (CNS) penetration. As of September 1, 2022, a total of 853 patients with KRASG12C-mutated solid tumors, including patients with CNS metastases, had received adagrasib (monotherapy or in combination). Adagrasib-related treatment-related adverse events (TRAEs) are generally mild to moderate in severity, start early in treatment, resolve quickly with appropriate intervention, and result in a low rate of treatment discontinuation. Common TRAEs seen in clinical trials included gastrointestinal-related toxicities (diarrhea, nausea, and vomiting); hepatic toxicities (increased alanine aminotransferase/aspartate aminotransferase) and fatigue, which... (truncated)

Title: Review of Immune-Related Adverse Events (irAEs) in Non-Small-Cell Lung Cancer (NSCLC)-Their Incidence, Management, Multiorgan irAEs, and Rechallenge.
Immune checkpoint inhibitors (ICIs) have revolutionized the treatment of advanced malignancies, including non-small cell lung cancer (NSCLC). These agents have improved clinical outcomes and have become quite an attractive alternative alone or combined with other treatments. Although ICIs are tolerated better, they also lead to unique toxicities, termed immune-related adverse events (irAEs). A reconstituted immune system may lead to dysregulation in normal immune self-tolerance and cause inflammatory side effects (irAEs). Although any organ system can be affected, immune-related adverse events most commonly involve the gastrointestinal tract, endocrine glands, skin, and liver. They can occur anytime during the treatment course and... (truncated)

Title: Causes, clinical features, and outcomes from a prospective study of drug-induced liver injury in the United States.
Idiosyncratic drug-induced liver injury (DILI) is among the most common causes of acute liver failure in the United States, accounting for approximately 13% of cases. A prospective study was begun in 2003 to recruit patients with suspected DILI and create a repository of biological samples for analysis. This report summarizes the causes, clinical features, and outcomes from the first 300 patients enrolled.

Title: The Useage of Opioids and their Adverse Effects in Gastrointestinal Practice: A Review.
Opium is one of the oldest herbal medicines currently used as an analgesic, sedative and antidiarrheal treatment. The effects of opium are principally mediated by the μ-, κ- and δ-opioid receptors. Opioid substances consist of all natural and synthetic alkaloids that are derived from opium. Most of their effects on gastrointestinal motility and secretion result from suppression of neural activity. Inhibition of gastric emptying, increase in sphincter tone, changes in motor patterns, and blockage of peristalsis result from opioid use. Common adverse effects of opioid administration include sedation, dizziness, nausea, vomiting, constipation, dependency and tolerance, and respiratory depression. The most... (truncated)

Title: Development and Validation of a Model Consisting of Comorbidity Burden to Calculate Risk of Death Within 6 Months for Patients With Suspected Drug-Induced Liver Injury.
Patients with drug-induced liver injury (DILI) frequently have comorbid conditions, but the effects of non-liver comorbidities on outcomes are not well understood. We investigated the association between comorbidity burden and outcomes of patients with DILI, and developed and validated a model to calculate risk of death within 6 months.

MESH:D002444 - cefuroxime

Summary:

LLM Prediction Score	0.368 (normalized)
LLM Confidence Score	0.990
Golden Answer (Severity Class)	0.625 (normalized)
Prediction Error	0.257

Retrieved Context:

Title: Worsening cholestasis and possible cefuroxime-induced liver injury following "successful" therapeutic endoscopic retrograde cholangiopancreatography for a distal common bile duct stone: a case report.
Cefuroxime very rarely causes drug-induced liver injury. We present a case of a patient with paradoxical worsening of jaundice caused by cefuroxime-induced cholestasis following therapeutic endoscopic retrograde cholangiopancreatography for a distal common bile duct stone.

Title: Influence of Antibiotics on Functionality and Viability of Liver Cells In Vitro.
(1) Antibiotics are an important weapon in the fight against serious bacterial infections and are considered a common cause of drug-induced liver injury (DILI). The hepatotoxicity of many drugs, including antibiotics, is poorly analyzed in human in vitro models. (2) A standardized assay with a human hepatoma cell line was used to test the hepatotoxicity of various concentrations (Cmax, 5× Cmax, and 10× Cmax) of antibiotics. In an ICU, the most frequently prescribed antibiotics, ampicillin, cefepime, cefuroxime, levofloxacin, linezolid, meropenem, rifampicin, tigecycline, and vancomycin, were incubated with HepG2/C3A cells for 6 days. Cell viability (XTT assay, LDH release, and vitality),... (truncated)

Title: Risk of acute liver injury associated with the use of moxifloxacin and other oral antimicrobials: a retrospective, population-based cohort study.
To estimate the incidence and relative risk of a hospitalization or emergency visit for noninfectious liver injury in users of eight oral antimicrobials-amoxicillin, amoxicillin-clavulanic acid, clarithromycin, cefuroxime, doxycycline, levofloxacin, moxifloxacin, telithromycin-compared with nonusers of these antimicrobials.

Title: A rare case of cefepime-induced cholestatic liver injury.
Cefepime is widely used in the hospital setting, and only a few studies have reported neurotoxicity and nephrotoxicity as side effects of this drug. Herein, we present a 93-year-old man who exhibited features of cholestatic hepatitis including elevated blood transaminases and direct-form predominant bilirubin levels after administration of cefepime. Blood liver tests showed total recovery after discontinuing the offending agent. Cefepime was probable to cause drug-induced cholestatic hepatitis in our patient since the Roussel Uclaf Causality Assessment Method score for cefepime was 7. No drug interactions were likely according to the Drug Interaction Probability Scale for this patient. No similar... (truncated)

Title: Antibiotics in Yersinia enterocolitica infections.
A prospective study was undertaken to evaluate the incidence, course, effects of treatment and outcome of patients with Yersinia enterocolitica infections. A total of 189 patients were followed: 62.5% had enteric forms of illness, 20.6% extramesenteric forms, 23.2% arthritis and erythema nodosum. Lymphadenopathy with high fever and weight loss, a septic syndrome and hepatitis were predominant manifestations of the extramesenteric form. Ten per cent of the isolates (135) were susceptible to amoxycillin (4 mg/l), 38% to cephradine (8 mg/l), 82% to doxycycline (4 mg/l), 83% to chloramphenicol (4 mg/l), 85% to trimethoprim (1 mg/l), 87% to cefuroxime (8 mg/l), 92%... (truncated)

MESH:D008790 - metoprolol

Summary:

LLM Prediction Score	0.370 (normalized)
LLM Confidence Score	0.990
Golden Answer (Severity Class)	0.625 (normalized)
Prediction Error	0.255

Retrieved Context:

Title: [Jaundice and liver injury with cholestatic pattern after treatment with Metoprololsuccinat].
Drug-induced liver injury is a well-known adverse event to numerous medications with clinical presentations from asymptomatic liver enzyme elevation to liver failure. However, liver injury after administration with metoprolol is not common, and only few case reports have been published. This is a case report of an 80-year-old woman with liver injury with cholestatic pattern and jaundice after two months of treatment with Metoprololsuccinat. With no evidence of other disease, liver function normalized in the following months after discontinuation of Metoprololsuccinat.

Title: Nicardipine-induced acute hepatitis in an intensive care unit patient.
Drug-related hepatotoxicity is now the leading cause of acute liver failure in the United States, especially among patients who have no prior liver disease. Nicardipine is the only IV calcium channel blocker available for the short-term treatment of hypertension with a considerably good safety profile. We report a case of nicardipine-induced hepatitis. A patient with history of hypertension was admitted because of right middle cerebral artery infarction. Computed tomography of the brain showed evolving stroke. The patient went for cerebral angiography and stent placement, and during the procedure he had cerebral hemorrhage. He was transferred to neurosurgery. After surgery, he... (truncated)

Title: A One-Two Punch: Hydralazine-Induced Liver Injury in a Recovering Ischemic Hepatitis.
A 77-year-old woman presented to the emergency department with a 2-day history of nausea and vomiting. Her medical history included diabetes mellitus, hypertension, atrial fibrillation, dilated cardiomyopathy, and coronary artery disease. Her home medications included aspirin, clopidogrel, warfarin, digoxin, metoprolol, losartan, simvastatin, isosorbide dinitrate, furosemide, and spironolactone. Initial physical examination showed blood pressure of 170/80 mm Hg with a heart rate of 69 beats per minute, otherwise unremarkable. Initial laboratory workup was significant for INR of 3.6, with slightly elevated troponin I and creatinine of 0.06 ng/mL and 1.4 mg/dL, respectively. The patient was admitted to the medicine floor. However,... (truncated)

Title: Reversible Fulminant Hepatitis Secondary to Cocaine in the Setting of β-Blocker Use.
<i>Background</i>. Fulminant hepatitis is acute hepatic injury with severe decline in hepatic function manifested by encephalopathy, hypercoagulable state, jaundice, renal failure, hypoglycemia, or a constellation of these symptoms in patients without preexisting liver disease. Etiologies include viral infections, hepatotoxic drugs, autoimmune diseases, vaso-occlusive diseases, sepsis, and malignant infiltration. <i>Case Report.</i> A 56-year-old man presented with acute heart failure in the setting of cocaine use. The patient subsequently developed fulminant hepatic failure manifested by acute hypoglycemia, elevated liver enzyme, and worsening liver function, which resolved over 1 week with supportive care. The patient was on β-blocker, which was stopped during the... (truncated)

Title: Macrophage polarization is involved in liver fibrosis induced by β ₁-adrenoceptor autoantibody.
Accumulating evidence suggests that liver injury can be induced by the over-expression of β ₁-adrenergic receptors (β ₁-ARs). High titers of autoantibodies specific to β ₁-adrenergic receptors (β ₁-AA) are detected in the sera of heart failure patients, potentially playing agonist-like roles. However, the role of β ₁-AA in liver function has not been characterized. In this study, we collect the sera of primary biliary cholangitis (PBC) patients, a condition which easily develops into liver fibrosis, and analyze the relationship between PBC and β ₁-AA. A passive immunization model is established to assess the effect of β ₁-AA on the... (truncated)

MESH:D000077237 - arsenic trioxide

Summary:

LLM Prediction Score	0.629 (normalized)
LLM Confidence Score	0.990
Golden Answer (Severity Class)	0.375 (normalized)
Prediction Error	0.254

Retrieved Context:

Title: Cytotoxicity and oxidative stress in human liver carcinoma cells exposed to arsenic trioxide (HepG(2)).
Arsenic is a trace element that occurs naturally in the earth's crust. It has been found to be a major contaminant in groundwater supply in several countries of the world. Whether ingested or inhaled, arsenic induces both systemic (skin disorders, cardiovascular diseases, anemia, peripheral neuropathy, liver and kidney damage) and carcinogenic (skin, lung, bladder and liver neoplasms) effects. However, its molecular mechanisms of toxicity are not completely understood. In this research, we used HepG(2) cells as a model to study the cytotoxicity and oxidative stress associated with exposure to arsenic trioxide. We hypothesized that oxidative stress plays a role in... (truncated)

Title: In Vivo Effect of Arsenic Trioxide on Keap1-p62-Nrf2 Signaling Pathway in Mouse Liver: Expression of Antioxidant Responsive Element-Driven Genes Related to Glutathione Metabolism.
Arsenic is a Group I human carcinogen, and chronic arsenic exposure through drinking water is a major threat to human population. Liver is one of the major organs for the detoxification of arsenic. The present study was carried out in mice in vivo after arsenic treatment through drinking water at different doses and time of exposure. Arsenic toxicity is found to be mediated by reactive oxygen species. Nuclear factor (erythroid-2 related) factor 2 (Nrf2)/Keap1 (Kelch-like ECH-associated protein 1)/ARE (antioxidant response element)-driven target gene system protects cells against oxidative stress and maintains cellular oxidative homeostasis. Our result showed 0.4 ppm, 2... (truncated)

Title: L-Ascorbic Acid and α-Tocopherol Reduces Hepatotoxicity Associated with Arsenic Trioxide Chemotherapy by Modulating Nrf2 and Bcl2 Transcription Factors in Chang liver Cells.
Arsenic trioxide (As₂O₃) is a promising new regimen for the treatment of acute promyelocytic leukemia (APL). The induction of oxidative stress mediated by reactive oxygen species (ROS) and excessive intracellular calcium influx are the main reasons behind As₂O₃ toxicity. Since liver is the major organ for xenobiotic metabolism, it is always under stress. Antioxidant vitamins such as L-Ascorbic acid (L-AA) and α-Tocopherol (α-TOC) have been proposed to have beneficial effects against a variety of pathological conditions and are known by their free radical scavenging properties. The present study evaluates the curative efficacy of L-AA and α-TOC against As₂O₃ toxicity using... (truncated)

Title: Melatonin protects against arsenic trioxide-induced liver injury by the upregulation of Nrf2 expression through the activation of PI3K/AKT pathway.
Melatonin has been demonstrated to have anti-inflammatory and antioxidant effects. The aim of this study was to investigate the protective effects of melatonin on arsenic trioxide (As2O3)-induced toxicity in liver and oxidative stress in rats. The rats were injected with 3mg/kg As2O3 on alternate days and melatonin was given with an intraperitoneal injection (i.p.) 1 h before As2O3 treatment. On the 8th days, the rats were killed to determine liver histological injury, antioxidant activities and accumulation of arsenic in liver tissues. Our results showed that melatonin attenuated As2O3-induced hepatic pathological damage, liver parameters, liver ROS level, MDA level, and the... (truncated)

Title: Metformin ameliorates arsenic trioxide hepatotoxicity via inhibiting mitochondrial complex I.
Arsenic trioxide (ATO) is a well-accepted chemotherapy agent in managing promyelocytic leukemia. ATO often causes severe health hazards such as hepatotoxicity, dermatosis, neurotoxicity, nephrotoxicity and cardiotoxicity. The production of reactive oxygen species, (ROS) play a significant role in ATO-induced hepatotoxicity. The oral hypoglycemic drug, metformin, is considered to be a potential novel agent for chemoprevention in the treatment of cancer. Moreover, metformin has also been shown to have hepatoprotective effects. In the present study, we demonstrated that metformin protected normal hepatocytes from ATO-induced apoptotic cell death in vitro and in vivo. Gene expression screening revealed that glucose metabolism might be... (truncated)

MESH:D000077562 - valganciclovir

Summary:

LLM Prediction Score	0.122 (normalized)
LLM Confidence Score	0.990
Golden Answer (Severity Class)	0.375 (normalized)
Prediction Error	0.253

Retrieved Context:

Title: Incidence and risk factors for the development of cytomegalovirus viremia in a steroid sparing liver transplant center.
Cytomegalovirus (CMV) is a common opportunistic infection in patients after liver transplant (LT). Guidelines recommend 900 mg daily of valganciclovir; however, valganciclovir commonly causes dose-dependent hematologic toxicities. Use of a low-dose valganciclovir (450 mg) has been used to prevent these adverse effects, but the data regarding this dosing strategy are not as robust in a steroid sparing LT center.

Title: Ganciclovir therapeutic drug monitoring in transplant recipients.
The use of (val)ganciclovir is complicated by toxicity, slow response to treatment and acquired resistance.

Title: Valganciclovir inhibits human adenovirus replication and pathology in permissive immunosuppressed female and male Syrian hamsters.
Adenovirus infections of immunocompromised pediatric hematopoietic stem cell transplant patients can develop into serious and often deadly multi-organ disease. There are no drugs approved for adenovirus infections. Cidofovir (an analog of 2-deoxycytidine monophosphate) is used at times but it can be nephrotoxic and its efficacy has not been proven in clinical trials. Brincidofovir, a promising lipid-linked derivative of cidofovir, is in clinical trials. Ganciclovir, an analog of 2-deoxyguanosine, has been employed occasionally but with unknown efficacy in the clinic. In this study, we evaluated valganciclovir against disseminated adenovirus type 5 (Ad5) infection in our permissive immunosuppressed Syrian hamster model. We... (truncated)

Title: Standard ganciclovir dosing results in slow decline of cytomegalovirus viral loads.
Cytomegalovirus (CMV) can cause severe disease, including rejection in transplant recipients. Ganciclovir and its oral prodrug valganciclovir have been used as first-line therapy for CMV disease in transplant recipients. The exposure targets of ganciclovir are not exactly known, and toxicity and resistance have interfered with ganciclovir therapy.

Title: Filociclovir is a potent inhibitor of human adenovirus F41.
Clusters of acute non HepA-E hepatitis cases in previously healthy children have been reported globally. At least, 1010 cases were identified in 35 countries, 5% of those cases required liver transplantation and 2% died. The exact cause is not yet known, but there is circumstantial evidence suggesting that human adenovirus F41 (HAdV-F41) might be playing a role. No antiviral drug has been approved for treating human adenovirus infections. Furthermore, HAdV-F41 is notoriously difficult to grow in cell culture, which hindered studying the efficacy of an antiviral compound against this virus. Here, we show that filociclovir (FCV), a nucleoside analog, is... (truncated)

MESH:C502994 - saxagliptin

Summary:

LLM Prediction Score	0.253 (normalized)
LLM Confidence Score	0.970
Golden Answer (Severity Class)	0.0 (normalized)
Prediction Error	0.253

Retrieved Context:

Title: Effect of Saxagliptin, a Dipeptidyl Peptidase 4 Inhibitor, on Non-Alcoholic Fatty Liver Disease.
Non-alcoholic fatty liver disease (NAFLD) represents a broad spectrum of chronic liver disease characterized by aberrant accumulation of triglycerides (TG) in hepatocytes without excessive alcohol consumption. Hepatic lipotoxicity derived from overaccumulation of free fatty acids is considered as one of the typical hallmarks of NAFLD. Insulin resistance (IR) and chronic inflammation are widely recognized as the key etiological factors associated with NAFLD. Dipeptidyl peptidase 4 inhibitor (DPP4i) is a novel pharmacological agent extensively applied in the treatment of Type 2 Diabetes Mellitus (T2DM) for decades which also have a liver protective effect.

Title: Kombiglyze (metformin and saxagliptin)-induced hepatotoxicity in a patient with non-alcoholic fatty liver disease.
A 33-year-old man was referred with hyperosmotic symptoms of 4 weeks. Clinical examination showed palpable hepatomegaly and no stigmata of liver disease. Findings were random glucose 16.6 mmol/L, HbA1c 12.4%, triglyceride 6.2 mmol/L, normal LFTs and ultrasound liver: increased echogenicity. Management consisted of dietician referral and commencement of metformin 500 mg bd, diamicron MR 60 mg od, and fenofibrate 145 mg od. He was non-compliant, complaining of "heaviness of head" after consuming oral diabetic agents, without symptoms of hypoglycemia. Treatment was switched to Kombiglyze XR (saxaglipitin 5 mg + metformin 1000 mg) and empagliflozin 25 mg od. He presented 1... (truncated)

Title: The therapeutic effects of berberine plus sitagliptin in a rat model of fatty liver disease.
Fatty liver disease (FLD) is a disorder related to accumulation of excess fat within the hepatocytes. In this study, the effects of Berberine, a natural compound, and Sitagliptin as a DPP-4 inhibitor, were observed in a rat model of FLD.

Title: Combined effects of synbiotic and sitagliptin versus sitagliptin alone in patients with nonalcoholic fatty liver disease.
Non-alcoholic fatty liver disease (NAFLD) is one of the most prevalent chronic liver diseases in recent years. The aim of this study was to evaluate the effects of sitagliptin with and without a synbiotic supplement in the treatment of patients with NAFLD.

Title: Bioequivalence of saxagliptin/dapagliflozin fixed-dose combination tablets compared with coadministration of the individual tablets to healthy subjects.
Saxagliptin and dapagliflozin are individually indicated as an adjunct to diet and exercise to improve glycemic control in adults with type 2 diabetes mellitus. The bioequivalence of saxagliptin/dapagliflozin 2.5/5 mg and 5/10 mg fixed-dose combination (FDC) tablets compared with coadministration of the individual tablets and the food effect on both strengths of saxagliptin/dapagliflozin FDCs were evaluated in this open-label, randomized, single-dose crossover study. Healthy subjects were randomized to saxagliptin 2.5 mg + dapagliflozin 5 mg fasted, 2.5/5 mg FDC fasted, 2.5/5 mg FDC fed (Cohort 1) or saxagliptin 5 mg + dapagliflozin 10 mg fasted, 5/10 mg FDC fasted, 5/10... (truncated)

MESH:D000069287 - capecitabine

Summary:

LLM Prediction Score	0.500 (normalized)
LLM Confidence Score	0.990
Golden Answer (Severity Class)	0.25 (normalized)
Prediction Error	0.250

Retrieved Context:

Title: Drug-Induced Liver Injury Caused by Capecitabine: A Case Report and a Literature Review.
Chemotherapy is widely used in cancer treatment, and the drug Capecitabine is often used in treatment of breast cancer and usually well-tolerated. Toxicity from Capecitabine typically involves hand-foot syndrome, fatigue, nausea, reduced appetite, and diarrhea, while severe liver toxicity is rarely seen. We present a case of a 63-year-old female with metastatic breast cancer, without liver metastasis, who developed a severe drug-induced liver injury (DILI) with critically elevated liver enzyme levels as reaction to Capecitabine treatment with seemingly no evident explanation as to why. The patient had a RUCAM score of 7 and a Naranjo score of 6 implying that... (truncated)

Title: Severe and Late Acute Liver Injury Induced by Capecitabine.
Capecitabine (CAP) is an antineoplastic agent that is known to cause mild hepatotoxicity. However, severe and late acute liver injury was not reported previously as an adverse reaction of CAP. This report discusses the case of a 63-year-old man with colon cancer who was receiving the fifth cycle of CAP as a monotherapy and presented with fatigue and jaundice during the fifth cycle of CAP. Laboratory tests showed markedly elevated transaminases (aspartate transaminase: 2,448 U/L; alanine transaminase: 1,984 U/L). Eventually, discontinuation of CAP was enough to reverse the delayed CAP-induced acute hepatic injury in clinical and laboratory terms.

Title: A phase II study of fixed-dose capecitabine and assessment of predictors of toxicity in patients with advanced/metastatic colorectal cancer.
The purpose of this study was to evaluate the safety and activity of fixed-dose capecitabine in patients with advanced colorectal cancer and to correlate pretreatment plasma concentrations of homocysteine and serum and red cell folate with toxicity. Patients received capecitabine 2000 mg (4 x 500 mg tablets) twice daily on days 1-14 every 3 weeks. They were reviewed weekly during the first cycle and then three weekly for safety assessment. Eligibility criteria were advanced/metastatic colorectal cancer, < or = 2 prior chemotherapy regimens, ECOG performance status 0-2 and life expectancy >12 weeks. A total of 60 patients were enrolled and... (truncated)

Title: Phase 1b study of the oral gemcitabine 'Pro-drug' LY2334737 in combination with capecitabine in patients with advanced solid tumors.
Background This Phase 1b study aimed to determine the recommended Phase 2 dose of LY2334737, an oral pro-drug of gemcitabine, in combination with capecitabine, an oral pro-drug of 5-fluorouracil, in patients with advanced solid tumors. In addition, pharmacokinetics (PK) and tumor response were evaluated. Patients and methods Patients with advanced/metastatic solid tumors received 650 mg/m(2) capecitabine twice daily (BID) and escalating doses of LY2334737 once daily (QD; initial dose 10 mg/day), both for 14 days followed by 7-day drug holiday. Cycles were repeated until progressive disease (PD) or unacceptable toxicity. Results Fifteen patients received a median of 2 (range 1-7)... (truncated)

Title: Lethal cardiotoxicity, steatohepatitis, chronic pancreatitis, and acute enteritis induced by capecitabine and oxaliplatin in a 36-year-old woman.
A 36-year-old female was hospitalized with symptoms suggesting intestinal occlusion. She was diagnosed with adenocarcinoma of the ampulla of Vater (pT4N0 stage) and underwent cephalic duodenopancreatectomy 8 months ago. Five cycles of postoperative chemotherapy were administrated using capecitabine and oxaliplatin (CAPOX or XELOX), the last one being completed 1 month ago. During the present hospitalization, because of normal computed tomography and ultrasound abdominal examination, rehydration and antibiotherapy were administrated. However, 4 days after hospital admission, the patient died. At autopsy and histological examination, we found a severe myocardial sclerosis with large scarring areas, severe steatohepatitis, chronic pancreatitis with large fibrotic... (truncated)

MESH:C498826 - nilotinib

Summary:

LLM Prediction Score	0.750 (normalized)
LLM Confidence Score	0.970
Golden Answer (Severity Class)	0.5 (normalized)
Prediction Error	0.250

Retrieved Context:

Title: A Case of Severe, Nilotinib-Induced Liver Injury.
Idiosyncratic hepatotoxicity is a leading reason for the discontinuation or dose modification of Food and Drug Administration (FDA)-approved medications in the United States. We report the case of a 53-year-old woman with chronic myeloid leukemia who developed acute cholestatic hepatitis in response to the tyrosine kinase inhibitor nilotinib. Nilotinib was discontinued, and the patient's liver function tests normalized over the next 3 months. We conclude that nilotinib may cause life-threatening hepatotoxicity and recommend that patients on the medication undergo regular monitoring of their liver tests.

Title: Antifibrotic Effect of Combination of Nilotinib and Stem Cell-Conditioned Media on CCl₄-Induced Liver Fibrosis.
Liver fibrosis is the excessive extracellular matrix accumulation of proteins, such as collagen, which follows the chronic liver diseases. Advanced liver fibrosis leads to cirrhosis and liver failure. Nilotinib is a second-generation tyrosine kinase inhibitor, which showed antifibrotic efficacy. Stem cell therapy still has some limitations such as oncogenesis, unexpected differentiation, and ethical consideration. Stem cells secrete cytokines and growth factors that showed paracrine-mediated antifibrotic and anti-inflammatory effects in vivo and in vitro. Thus, stem cell-conditioned medium (SC-CM), which contains the secretory proteins of stem cells, may have an antifibrotic role. This study was carried out to examine the antifibrotic... (truncated)

Title: Experience with the Use of Nilotinib in Indian Patients.
Important genetic and ethnic factors could affect the toxicity and efficacy of tyrosine kinase inhibitors. Though nilotinib has been available in India since 2010, there is no report on its safety and toxicity from Indian patients with chronic myeloid leukemia. This is an analysis of efficacy and toxicity of nilotinib when used as a <i>second line drug</i> after failure or intolerance to imatinib. Thirty-seven patients started nilotinib [median age 46 years, median duration from diagnosis 5 years, 73% in chronic phase at time of switch] between 2010 to 2016. Reason for switch: failure of imatinib in 33 (89%) and intolerance in 4 (11%).... (truncated)

Title: Nilotinib interferes with the signalling pathways implicated in acetaminophen hepatotoxicity.
Nilotinib, a second-generation tyrosine kinase inhibitor, has been recently approved for the treatment for chronic myeloid leukaemia. The objective of this study was to explore the potential effects of clinically relevant doses of nilotinib against acetaminophen (APAP)-induced hepatotoxicity in mice. To simulate the clinical application in human beings, nilotinib (25 and 50 mg/kg) was administered to mice 2 hr after APAP intoxication (500 mg/kg). The results indicated that nilotinib (25 mg/kg) (i) abolished APAP-induced liver injury and necro-inflammation, (ii) increased hepatic-reduced glutathione (GSH) and its related enzymes synthesis, (iii) suppressed hepatic oxidative/nitrosative stress cascades, (iv) decreased neutrophil accumulation in the... (truncated)

Title: Nilotinib-Induced Immune-Mediated Liver Injury: Corticosteroid as a Possible Therapeutic Option.
Introduction: Nilotinib is a BCR-ABL tyrosine kinase inhibitor approved for chronic myeloid leukemia. We present a case of severe immune-mediated liver injury by nilotinib treatment. Case report: A 59-year-old woman was referred to the liver clinic because of elevated liver enzyme levels. One year prior, she was diagnosed as having chronic myeloid leukemia and treated with nilotinib therapy. The level of aspartate aminotransferase and alanine aminotransferase were 578 IU/L and 499 IU/L, respectively. Percutaneous needle liver biopsy showed extensive centrilobular infiltration of immune cells and destruction of the lobular architecture with minimal inflammation in the portal triad. Immunohistochemical staining showed... (truncated)

MESH:C053091 - lomefloxacin

Summary:

LLM Prediction Score	0.250 (normalized)
LLM Confidence Score	0.910
Golden Answer (Severity Class)	0.5 (normalized)
Prediction Error	0.250

Retrieved Context:

Title: Comparative tolerability of the newer fluoroquinolone antibacterials.
The most common adverse effects of the fluoroquinolones involve the gastrointestinal tract, skin and CNS, and are mainly mild and reversible. Of the gastrointestinal events, nausea and vomiting are the most common. Mild hepatic reactions are a class effect, usually presenting as mild transaminase level increases without clinical symptoms. However, postmarketing surveillance has revealed significant hepatotoxicity with trovafloxacin. It is not currently known whether the severe reactions to trovafloxacin are specific to that agent or simply represent an extreme of an emerging class effect. The enormous worldwide usage of, and extensive published adverse effect data on the other fluoroquinolones and... (truncated)

Title: [Lomefloxacin in phthisiatric practice].
The efficacy and safety of lomefloxacin in the treatment of patients with hepatitis due to the use of routine antituberculosis agents were estimated. The trial group included 20 patients (10 with increased activity of enzymes such as alanine and asparagine transaminases, alkaline phosphatase and gamma-glutamate dehydrogenase) who were treated for various forms of tuberculosis with antitubeculosis drugs. The treatment course with lomefloxacin was 4 weeks (400 mg twice a day at 12-hour intervals). The criteria of the enrolment to the trial group were a more than 2-3 times higher activity of the enzymes and the absence of the markers of... (truncated)

Title: Fluoroquinolone-related adverse events resulting in health service use and costs: A systematic review.
Adverse events (AEs) associated with the use of fluoroquinolone antimicrobials include Clostridium difficile associated diarrhea (CDAD), liver injury and seizures. Yet, the economic impact of these AEs is seldom acknowledged. The aim of this review was to identify health service use and subsequent costs associated with ciprofloxacin, levofloxacin, moxifloxacin, norfloxacin and ofloxacin -related AEs.

Title: Current progress of fluoroquinolones-increased risk of aortic aneurysm and dissection.
Aortic aneurysm (AA) and aortic dissection (AD) are major life-threatening diseases around the world. AA is a localized or diffuse dilation of the aorta, while AD is the separation of the layers creating a false lumen within the aortic wall. Fluoroquinolones (FQ) remain one of the most important kind of antibiotics and have a wider clinical use and broad antibacterial spectrum. FQ were also reported to treat infected AA. The most common adverse events (AEs) of FQ are mild and reversible, like headaches, diarrhea and nausea. Due to FQ-related serious AEs, such as tendonitis and tendon rupture, chondrotoxicity, or retinal... (truncated)

Title: Overview of Side-Effects of Antibacterial Fluoroquinolones: New Drugs versus Old Drugs, a Step Forward in the Safety Profile?
Antibacterial fluoroquinolones (FQs) are frequently used in treating infections. However, the value of FQs is debatable due to their association with severe adverse effects (AEs). The Food and Drug Administration (FDA) issued safety warnings concerning their side-effects in 2008, followed by the European Medicine Agency (EMA) and regulatory authorities from other countries. Severe AEs associated with some FQs have been reported, leading to their withdrawal from the market. New systemic FQs have been recently approved. The FDA and EMA approved delafloxacin. Additionally, lascufloxacin, levonadifloxacin, nemonoxacin, sitafloxacin, and zabofloxacin were approved in their origin countries. The relevant AEs of FQs and... (truncated)

MESH:D000077487 - pramipexole

Summary:

LLM Prediction Score	0.000 (normalized)
LLM Confidence Score	0.980
Golden Answer (Severity Class)	0.25 (normalized)
Prediction Error	0.250

Retrieved Context:

Title: Successful drug development despite adverse preclinical findings part 2: examples.
To illustrate the process of addressing adverse preclinical findings (APFs) as outlined in the first part of this review, a number of cases with unexpected APF in toxicity studies with drug candidates is discussed in this second part. The emphasis is on risk characterization, especially regarding the mode of action (MoA), and risk evaluation regarding relevance for man. While severe APFs such as retinal toxicity may turn out to be of little human relevance, minor findings particularly in early toxicity studies, such as vasculitis, may later pose a real problem. Rodents are imperfect models for endocrine APFs, non-rodents for human... (truncated)

Title: Preliminary Evidence of Efficacy and Target Engagement of Pramipexole in Anhedonic Depression.
To investigate feasibility and target engagement of high-dose, add-on pramipexole treatment in anhedonic depression.

Title: Add-on pramipexole for anhedonic depression: study protocol for a randomised controlled trial and open-label follow-up in Lund, Sweden.
Many depressed patients do not achieve remission with available treatments. Anhedonia is a common residual symptom associated with treatment resistance as well as low function and quality of life. There are currently no specific and effective treatments for anhedonia. Some trials have shown that dopamine agonist pramipexole is efficacious for treating depression, but more data is needed before it could become ready for clinical prime time. Given its mechanism of action, pramipexole might be a useful treatment for a depression subtype characterised by significant anhedonia and lack of motivation-symptoms associated with dopaminergic hypofunction. We recently showed, in an open-label pilot... (truncated)

Title: Effects of pramipexole treatment on the α-synuclein content in serum exosomes of Parkinson's disease patients.
Advances approaches in the treatment of Parkinson's disease are needed. The study was aimed to evaluate the therapeutic value of the new dopamine receptor agonist pramipexole. The effects of pramipexole on serum exosomes were investigated, and the possible mechanisms of action of the drug were explored. Initially, 68 patients were included in the study, of whom 3 cases did not complete the study. The remaining 65 patients were administered pramipexole at increasing doses starting at 0.25 mg twice a day for the 1st week, and reaching 1.5 mg three times daily at the 8th week. The doses were tapered during... (truncated)

Title: Long-term safety and sustained efficacy of extended-release pramipexole in early and advanced Parkinson's disease.
To assess the long-term safety and efficacy of pramipexole as a once-daily (q.d.) extended-release oral formulation in early or advanced Parkinson's disease (PD).

MESH:D003620 - dantrolene

Summary:

LLM Prediction Score	0.750 (normalized)
LLM Confidence Score	0.990
Golden Answer (Severity Class)	1.0 (normalized)
Prediction Error	0.250

Retrieved Context:

Title: Oral Dantrolene for Myopathic Symptoms in Malignant Hyperthermia-Susceptible Patients: A 25-Year Retrospective Cohort Study of Adverse Effects and Tolerability.
Patients susceptible to malignant hyperthermia (MH) may experience disabling manifestations of an unspecified myopathy outside the context of anesthesia, including myalgia, fatigue, or episodic rhabdomyolysis. Clinical observations suggest that oral dantrolene may relief myopathic symptoms in MH-susceptible (MHS) patients. However, high-dose oral dantrolene has been associated with severe hepatotoxicity.

Title: Dantrolene sodium and hepatic injury.
This is a report on hepatic adverse events associated with dantrolene therapy. All cases reported to the manufacturer are included, from all sources, through 1987. Of 122 cases containing sufficient data to analyze, 47 patients had asymptomatic transaminase elevations, 12 had additional mild (less than or equal to 2.5 mg/dl) hyperbilirubinemia, 36 had jaundice, and 27 patients died. There is an overrepresentation of women over 35 years and patients with multiple sclerosis in the fatal group compared with the study population as a whole (not statistically significant). Mean dantrolene dose was 582 mg/d in fatal cases and 263 mg/d in... (truncated)

Title: [Dantrolene-induced pleurisy: a case report].
We report the case of a patient who developed dantrolene-induced pleurisy. Dantrolene (Dantrium) is a muscle relaxing agent used for the treatment of spastic neurological manifestations which has known liver toxicity. Lung toxicity is rarely reported. Six cases of dantrolene-induced pleurisy occurring after chronic administration (> 60 days) have been described in the literature. The pleurisy is associated with pleural and peripheral eosinophilia. Spontaneous regression a few days after withdrawal and radiological cure a few months later is the rule. The precise mechanism of this drug-induced pleural reaction remains unknown.

Title: Hepatitis from dantrolene sodium.
The clinical course and histological changes in the liver during a presumed adverse reaction to the drug dantrolene sodium are described in four patients. After a typical prodrome one developed a moderately severe hepatitis-like illness. Another also had a prodrome but never became jaundiced. In the other two, abnormal liver function tests were detected on routine screening. In each case liver biopsy showed changes typical of an acute hepatitis, but the severity was unrelated to the clinical presentation. In addition, there were also changes in the portal tracts resembling ascending cholangitis. In each case liver function tests returned to normal... (truncated)

Title: Dantrolene. A review of its pharmacodynamic and pharmacokinetic properties and therapeutic use in malignant hyperthermia, the neuroleptic malignant syndrome and an update of its use in muscle spasticity.
Dantrolene sodium acts primarily by affecting calcium flux across the sarcoplasmic reticulum of skeletal muscle. Recently, dantrolene has been used very successfully in the treatment of several rare hypercatabolic syndromes which have previously been associated with high mortality rates. In malignant hyperthermia, where early diagnosis and treatment usually with intravenous dantrolene in association with other supportive measures (and often subsequent dantrolene therapy) is performed, recovery is seen in virtually 100% of patients. There is a rapid resolution of hyperthermia, dysrhythmias, muscle rigidity, tachycardia, hypercapnia, mottled or cyanotic skin, and metabolic acidosis, and a slower normalisation of myoglobinuria and elevated serum... (truncated)

MESH:D004970 - estrone

Summary:

LLM Prediction Score	0.500 (normalized)
LLM Confidence Score	0.980
Golden Answer (Severity Class)	0.25 (normalized)
Prediction Error	0.250

Retrieved Context:

Title: The Pathological Mechanisms of Estrogen-Induced Cholestasis: Current Perspectives.
Estrogens are steroid hormones with a wide range of biological activities. The excess of estrogens can lead to decreased bile flow, toxic bile acid (BA) accumulation, subsequently causing intrahepatic cholestasis. Estrogen-induced cholestasis (EIC) may have increased incidence during pregnancy, and within women taking oral contraception and postmenopausal hormone replacement therapy, and result in liver injury, preterm birth, meconium-stained amniotic fluid, and intrauterine fetal death in pregnant women. The main pathogenic mechanisms of EIC may include deregulation of BA synthetic or metabolic enzymes, and BA transporters. In addition, impaired cell membrane fluidity, inflammatory responses and change of hepatocyte tight junctions are... (truncated)

Title: Potential Therapeutic Application of Estrogen in Gender Disparity of Nonalcoholic Fatty Liver Disease/Nonalcoholic Steatohepatitis.
Nonalcoholic fatty liver disease (NAFLD) caused by fat accumulation in the liver is globally the most common cause of chronic liver disease. Simple steatosis can progress to nonalcoholic steatohepatitis (NASH), a more severe form of NAFLD. The most potent driver for NASH is hepatocyte death induced by lipotoxicity, which triggers inflammation and fibrosis, leading to cirrhosis and/or liver cancer. Despite the significant burden of NAFLD, there is no therapy for NAFLD/NASH. Accumulating evidence indicates gender-related NAFLD progression. A higher incidence of NAFLD is found in men and postmenopausal women than premenopausal women, and the experimental results, showing protective actions of... (truncated)

Title: Clinical significance of estrogens in chronic aggressive hepatitis.
For a long time, it has been assumed that stagnation of active estrogens in the blood gives rise to liver injury and causes a severe inflammatory process in the liver as well as affecting the clinical course of chronic aggressive hepatitis in women. During reproductive years, estrogen production, as gaged by the values of urinary excretion, follows a cyclic pattern. As the menopause is approached, urinary excretion of estrogens gradually diminishes and the cyclic fluctuation becomes more shallow. The titer continues to fall progressively in the post-menopausal years although some estrogen may be found even in aged women. Even though... (truncated)

Title: Studies on the effects of estrogen on in vitro antibody production in autoimmune liver diseases, including lupoid hepatitis and primary biliary cirrhosis.
Antibody-forming cells produced by adding trinitrophenylated sheep red blood cells (TNP-SRBC) were induced, when peripheral blood mononuclear cells from normal individuals and patients with autoimmune liver diseases, including lupoid hepatitis and primary biliary cirrhosis, were stimulated in vitro with pokeweed mitogen (PWM). Although antibody responses were significantly augmented by adding estrogen simultaneously with PWM to mononuclear cell cultures prepared from normal individuals and autoimmune liver diseases patients, a significant difference was observed according to the concentrations of estrogen between the normal subjects and patients. These observations suggest that a different responsiveness to the different concentrations of estrogen underlines the immunological... (truncated)

Title: Urinary excretion of oestrone, oestradiol-17beta and oestriol in patients with chronic liver damage.
No abstract available.

MESH:D003078 - colchicine

Summary:

LLM Prediction Score	0.500 (normalized)
LLM Confidence Score	0.990
Golden Answer (Severity Class)	0.75 (normalized)
Prediction Error	0.250

Retrieved Context:

Title: Colchicine-associated ring mitosis in liver biopsy and their clinical implications.
Colchicine is an alkaloid with antimitotic activity used to treat a variety of medical conditions. Colchicine toxicity can result in multiorgan failure and death. In recent years, the histopathologic features of colchicine toxicity in various organs that is, gastrointestinal tract, kidneys, salivary glands, muscle, and skin have been described. However, the histopathologic manifestations of colchicine in the liver have not been reported. We present the case of a 54-year-old man with transfusion acquired chronic hepatitis C and longstanding gout managed with colchicine. A liver biopsy performed showed changes consistent with chronic hepatitis C along with many scattered mitoses arrested in... (truncated)

Title: Electrophilicities and Protein Covalent Binding of Demethylation Metabolites of Colchicine.
Colchicine, an alkaloid existing in plants of Liliaceous colchicum, has been widely used in the treatment of gout and familial Mediterranean fever. The administration of colchicine was found to cause liver injury in humans. The mechanisms of colchicine-induced liver toxicity remain unknown. The objectives of this study were to determine the electrophilicities of demethylation metabolites of colchicine and investigate the protein adductions derived from the reactive metabolites of colchicine. Four demethylated colchicine (1-, 2-, 3-, and 10-DMCs), namely, M1-M4, were detected in colchicine-fortified microsomal incubations. Four N-acetyl cysteine (NAC) conjugates (M5-M8) derived from colchicine were detected in the microsomes in... (truncated)

Title: Colchicine-Induced Hepatotoxicity.
Drug-induced injury (DILI) is a frequent cause of abnormal liver tests and a leading cause of liver failure in the United States. Colchicine has long been used as a systemic anti-inflammatory agent for treatment of gout by inhibiting mitotic activity and neutrophil function. We present the first case of colchicine-induced hepatotoxicity, supported by histopathologic findings characteristic of colchicine-induced injury and resolution of liver enzyme abnormalities after its discontinuation. Colchicine-associated DILI has implications for the evaluation of patients with abnormal liver tests and gout, especially for patients with alcoholism and non-alcoholic fatty liver disease, in whom there is an increased incidence... (truncated)

Title: Hemophagocytic lymphohistiocytosis and pelger-huët anomaly associated with colchicine intoxication.
Colchicine is frequently used in the treatment of familial Mediterranean fever (FMF). First symptoms of colchicine intoxication are gastrointestinal disturbances, such as abdominal cramps, diarrhea, pancytopenia and so on. Herein, we report a female FMF patient with pancytopenia and hemophagocytic lymphohitiocytosis (HLH), following colchicine intoxication for committing suicide. To our knowledge, this is the first reported case of a patient with HLH associated with colchicine intoxication.

Title: Severe hypertriglyceridemia and colchicine intoxication following suicide attempt.
Colchicine overdose is uncommon but potentially life threatening. Due to its serious adverse systemic effects, overdose must be recognized and treated. We report a case of an 18-year-old female who ingested 18 mg (~0.4 mg/kg) of colchicine in a suicide attempt. The patient's clinical manifestations included abdominal cramps, vomiting, pancytopenia, hypocholesterolemia, and rhabdomyolysis. Two unique manifestations of toxicity in this patient were profound and persistent, severe hypertriglyceridemia and electrolyte imbalance, mainly hypophosphatemia, with no other evident cause except the colchicine intoxication. Following intensive supportive treatment, including ventilator support, N-acetylcysteine, granulocyte colony stimulating factor, electrolyte repletion, and zinc supplementation, the patient... (truncated)

MESH:D000077289 - letrozole

Summary:

LLM Prediction Score	0.500 (normalized)
LLM Confidence Score	0.990
Golden Answer (Severity Class)	0.25 (normalized)
Prediction Error	0.250

Retrieved Context:

Title: Palbociclib-induced severe hepatitis: A case study and literature review.
Palbociclib is a selective and reversible CDK4/6 inhibitor approved for patients presenting with HR+ HER2- locally advanced or metastatic breast cancer. Its adverse effect (AE) is mainly reported on the occurrence of leukopenia and fatigue. Even though palbociclib has an extensive hepatic metabolism, there are rare reports about significant liver toxicity. We present the case of a 61-year-old female with metastatic breast cancer treated with palbociclib and an aromatase inhibitor (letrozole). The patient developed a rare AE of severe acute drug-induced hepatitis but improved dramatically after stopping the palbociclib and receiving treatment with <i>N</i>-acetylcysteine (NAC). The treatment with NAC may... (truncated)

Title: Letrozole-induced hepatitis with autoimmune features: a rare adverse drug reaction with review of the relevant literature.
While aromatase inhibitors (AIs) have been known to cause minor elevations in liver enzymes, severe hepatotoxicity is rare. To the best of our knowledge, this is the first reported case of Letrozole-induced hepatitis with autoimmune features. A 70-year-old female with estrogen positive, invasive ductal carcinoma of the breast, presented with jaundice 3 months after starting letrozole. Hepatic transaminases were markedly elevated and her ANA and anti-smooth muscle antibody was positive. Liver biopsy featured drug-induced hepatitis. After stopping letrozole, liver tests trended back to normal within 3 weeks. She scored 9 for Roussel-Uclaf Causality Assessment Method (RUCAM). Over the last 10... (truncated)

Title: Denosumab-Induced Immune Hepatitis.
Denosumab-Prolia®, Xgeva® (Amgen) is a fully human antibody to the receptor activator of the nuclear factor-K ligand (RANKL). Hepatotoxicity is extremely rare, with only one reported case of immune origin. We present a second case of hepatotoxicity resulting from an immune reaction to denosumab. A 43-year-old female was referred to the Endocrinology, Diabetes & Metabolism Department for treatment of low bone mineral density (BMD) following endocrine therapy with letrozole and lucrin because of breast cancer. She developed premature menopause at the age of 36 years when she underwent a left lumpectomy due to an infiltrating duct carcinoma of the breast... (truncated)

Title: Drug-Induced Liver Injury Caused by Capecitabine: A Case Report and a Literature Review.
Chemotherapy is widely used in cancer treatment, and the drug Capecitabine is often used in treatment of breast cancer and usually well-tolerated. Toxicity from Capecitabine typically involves hand-foot syndrome, fatigue, nausea, reduced appetite, and diarrhea, while severe liver toxicity is rarely seen. We present a case of a 63-year-old female with metastatic breast cancer, without liver metastasis, who developed a severe drug-induced liver injury (DILI) with critically elevated liver enzyme levels as reaction to Capecitabine treatment with seemingly no evident explanation as to why. The patient had a RUCAM score of 7 and a Naranjo score of 6 implying that... (truncated)

Title: [A real-world study of the effects of endocrine therapy on liver function in breast cancer].
Objective: To compare the effect of different endocrine therapy drugs on liver function in patients with early breast cancer. Methods: A retrospective cohort study was conducted to include 4 318 patients with early breast cancer who received adjuvant endocrine therapy in Department of Breast Surgery, Peking Union Medical College Hospital from January 1, 2013 to December 31, 2021. All the patients were female, aged (51.2±11.3) years (range: 20 to 87 years), including 1 182 patients in the anastrozole group, 592 patients in the letrozole group, 332 patients in the exemestane group, and 2 212 patients in the toremifene group. The... (truncated)

MESH:D002927 - cimetidine

Summary:

LLM Prediction Score	0.500 (normalized)
LLM Confidence Score	0.990
Golden Answer (Severity Class)	0.25 (normalized)
Prediction Error	0.250

Retrieved Context:

Title: Cimetidine: adverse reactions and acute toxicity.
Recent reports of cimetidine toxicity are summarized. Summaries of specific cases and categorized according to cardiovascular, central nervous system, dermatologic, endocrine, gastrointestinal, hematologic, or renal toxicity, or overdosage. Adverse reactions reported secondary to cimetidine during its investigational period and shortly after marketing were minimal. In several studies in which over 1200 patients were treated with cimetidine, the incidences of adverse clinical symptoms was no higher than in the nearly 500 placebo-treated patients. However, subsequent reports indicate that elderly patients, patients with impaired renal function, and patients with liver disease appear quite susceptible to mental confusion. Potentially serious hematologic depression, cardiac... (truncated)

Title: Potential use of cimetidine for treatment of acetaminophen overdose.
Acetaminophen, a drug frequently taken in intentional and accidental overdose, causes liver toxicity when concentration of the cytochrome P-450-derived metabolite exceeds the metabolic capacity of available glutathione. Present treatment of acetaminophen overdose involves oral N-acetylcysteine (NAC), which enhances liver glutathione synthesis. An alternative or additive approach to therapy would be to inhibit the formation of the toxic metabolite by inhibiting the cytochrome P-450 system. The H2-receptor antagonist cimetidine inhibits the cytochrome P-450 system, does not interfere with the administration or function of NAC, and therefore affords additive protection. Also, it has little effect on the nontoxic routes of elimination of... (truncated)

Title: Prevention of acetaminophen and cocaine hepatotoxicity in mice by cimetidine treatment.
Hepatotoxicity occurs in animals after administration of large doses of acetaminophen and cocaine and is thought to result from production of reactive metabolites of these parent drugs by cytochrome P450. Because cimetidine binds to cytochrome P450 and inhibits hepatic drug metabolism in both humans and animals, we determined the effects of cimetidine coadministration on acetaminophen and cocaine hepatotoxicity in mice. Marked elevations of serum glutamic pyruvic transaminase and severe pericentral hepatocellular necrosis occurred in animals receiving intraperitoneal doses of 350 mg/kg acetaminophen or 35 mg/kg cocaine, while minimal serum glutamic pyruvic transaminase elevations and liver necrosis were seen in animals... (truncated)

Title: The risk of acute liver injury associated with cimetidine and other acid-suppressing anti-ulcer drugs.
The objective of this study was to estimate the risk of acute liver injury associated with individual acid-suppressing drugs and assess the role of dose and duration of treatment.

Title: Cimetidine-induced liver injury. Report of three cases.
Cimetidine-induced liver injury has only very rarely been reported. Three patients are described who developed signs of hepatic damage after the institution of cimetidine therapy. Transient signs of acute liver failure were noticed in one patient. Histologically, a cytotoxic type of injury with centrilobular confluent and bridging portal-central necrosis, accompanied by a mixed mono- and polymorphonuclear infiltrate with signs of cholangiolitis in the portal tracts was observed in two patients, whereas a hepatocanalicular type of cholestatic hepatitis was noticed in another patient. It is proposed that the mechanism of cimetidine-induced liver injury may vary in different patients: it may be... (truncated)

MESH:D000493 - allopurinol

Summary:

LLM Prediction Score	0.750 (normalized)
LLM Confidence Score	0.990
Golden Answer (Severity Class)	1.0 (normalized)
Prediction Error	0.250

Retrieved Context:

Title: Allopurinol-Induced Granulomatous Hepatitis: A Case Report and Review of Literature.
Liver enzyme elevation is a common reason for referral to a gastroenterologist. Drugs are one of the most common reasons for asymptomatic elevation of liver enzymes. We present here a case of granulomatous hepatitis (GH) secondary to long-term use of allopurinol. An 83-year-old male with a history of chronic gout and hypertension was evaluated for elevation of liver enzymes. He denies any complaints of abdominal pain, nausea, fever, chills, weight loss, night sweats, or yellowness of skin. He denies any use of herbal medications. He was on losartan and allopurinol for years. No new medications reported. Physical examination was unremarkable.... (truncated)

Title: [Severe acute hepatitis due to allopurinol in a patient with asymptomatic hyperuricemia and kidney failure. A review of the literature and an analysis of the risk factors].
The case of a 39-year-old female with mild renal failure and asymptomatic hyperuricemia who developed generalized exanthema, fever and eosinophilia followed by progressive jaundice and worsening of renal function 19 days after the initiation of treatment with alopurinol (300 mg/day) is reported. Liver biopsy showed a combination of mixed inflammatory infiltrate with abundant eosinophils and periportal necrosis and bridging, together with cholestasis and moderate steatosis. A review of the literature is made providing detailed analysis of other cases with preexisting renal failure and the role of renal dysfunction as a risk factor is discussed.

Title: Allopurinol hepatotoxicity. Report of two cases and review of the literature.
Allopurinol hepatotoxicity occurred in two patients. Data from the literature suggest that allopurinol can occasionally cause liver injury, particularly in persons receiving diuretic drugs or with compromised renal function. Clinical and laboratory findings are consistent with hepatocellular injury mediated by a hypersensitivity reaction. Most patients recover when the drug is withdrawn; the possible benefits of corticosteroid treatment remain to be established.

Title: Allopurinol-induced DRESS syndrome.
A 70-year-old man was admitted to our clinic with complaints of fever, jaundice, dyspnea, and generalized rash after 3 months of allopurinol treatment for gout. On physical examination, he was found to have fever (38.5°C), jaundice, and generalized maculopapular rash. Leukocytosis, eosinophilia, elevation of liver enzymes, and hyperbilirubinemia were detected in his blood analysis. Skin biopsy was consistent with drug-induced hypersensitivity. He was diagnosed as Drug Rash with Eosinophilia and Systemic Symptoms (DRESS). Allopurinol treatment was stopped and steroid treatment was launched. At day 24 of admission, the patient died because of multiple organ failure.

Title: Allopurinol-induced DRESS syndrome mimicking biliary obstruction.
An 84-year-old man was admitted to our hospital with fever, jaundice, and itching. He had been diagnosed previously with chronic renal failure and diabetes, and had been taking allopurinol medication for 2 months. A physical examination revealed that he had a fever (38.8°C), jaundice, and a generalized maculopapular rash. Azotemia, eosinophilia, atypical lymphocytosis, elevation of liver enzymes, and hyperbilirubinemia were detected by blood analysis. Magnetic resonance cholangiography revealed multiple cysts similar to choledochal cysts in the liver along the biliary tree. Obstructive jaundice was suspected clinically, and so an endoscopic ultrasound examination was performed, which ruled out a diagnosis of... (truncated)

MESH:D003606 - dacarbazine

Summary:

LLM Prediction Score	1.000 (normalized)
LLM Confidence Score	0.980
Golden Answer (Severity Class)	0.75 (normalized)
Prediction Error	0.250

Retrieved Context:

Title: Phase II study of dacarbazine given with modern prophylactic anti-emetics and growth factor support to patients with metastatic, resistant soft tissue, and bone sarcoma.
Historically, administration of dacarbazine to sarcoma patients was limited by frequent treat-ment-related nausea/vomiting and neutropenia. These toxicities are now largely preventable with contemporary antiemetics and growth factor support. In this single-arm, phase II study, dacarbazine 850 mg/m² was given on day 1 of each 3-week cycle until disease progression or intolerance with prophylactic serotonin-3 receptor, neurokinin-1 antagonists, corticosteroids, and pegfilgrastim. Coprimary endpoints included clinical benefit rate (CBR), and any grade of nausea/vomiting and/or grade 3-4 neutropenia. With a sample size of 80 patients, >24 patients with clinical benefit would indicate that the CBR exceeds the historical (<20%) [Power 0.80; alpha... (truncated)

Title: [Veno-occlusive syndrome with acute liver dystrophy following decarbazine therapy of malignant melanoma (author's transl)].
Case report about death due to veno-occlusive liver disease following Dacarbazine treatment: 9 years after surgical treatment of malignant melanoma of the trunk a 68-years old patient developed lymph node metastases in the right axilla, which were removed immediately by surgical excision. One month before the patient had undergone surgical treatment of empyema of the gallbladder: Cholecystectomy and appendectomy were performed, postoperative recovery was uncomplicated. On account of the second lymph node metastasis within nine months adjuvant treatment with Dacarbazine was agreed and started one month later. After having performed the first course of treatment without any hints to intolerance... (truncated)

Title: Pathophysiological aspects of dacarbazine-induced human liver damage.
Hepatic Veno-occlusive Disease (VOD) and Budd Chiari syndrome (BCS) have been reported as fatal complications of Dacarbazine therapy. Peripheral eosinophilia and eosinophilic infiltrates after repeated Dacarbazine therapy in some cases were interpreted as evidence of an allergic drug reaction. Since no mention of an allergic drug reaction is to be found in other reports on veno-occlusive disease or Budd Chiari syndrome due to Dacarbazine, the pathophysiological mechanism of these fatal complications of Dacarbazine remain to be elucidated. We describe a case of VOD due to Dacarbazine which showed peripheral eosinophilia after the first cycle of Dacarbazine treatment with no prior... (truncated)

Title: Acute temozolomide induced liver injury: mixed type hepatocellular and cholestatic toxicity.
Temozolomide (TMZ) is an oral imidazotetrazine methylating agent which is used for the treatment of glioblastoma multiforme (GBM). We report a case of acute hepatotoxicity in a 53-year old male patient after administration of TMZ for GBM. He had fatigue, nausea, anorexia and jaundice. His laboratory analysis showed alanine aminotransferase(ALT): 632 IU/L (normal range 0-40); aspartate aminotransferase(AST): 554 IU/L (normal range 5-34); alkaline phosphatase(ALP): 1143 IU/L (normal range 40-150); γ-glutamyl transpeptidase(GGT): 514 IU/L (normal range 9-64 IU/L); total bilirubin: 15.1 mg/dL (normal range 0-1.2); direct bilirubin: 13.2 mg/dL and prothrombin time(PT): 13.5 s, with international normalized ratio (INR): 1.1 (normal... (truncated)

Title: Acute temozolomide induced liver injury : Mixed type hepatocellular and cholestatic toxicity.
Temozolomide (TMZ) is an oral imidazotetrazine methylating agent which is used for the treatment of glioblastoma multiforme (GBM). We report a case of acute hepatotoxicity in a 53-year old male patient after administration of TMZ for GBM. He had fatigue, nausea, anorexia and jaundice. His laboratory analysis showed alanine aminotransferase(ALT) : 632 IU/L (normal range 0-40) ; aspartate aminotransferase(AST) : 554 IU/L (normal range 5-34) ; alkaline phosphatase(ALP) : 1143 IU/L (normal range 40-150) ; γ-glutamyl transpeptidase(GGT) : 514 IU/L (normal range 9-64 IU/L) ; total bilirubin : 15.1 mg/dL (normal range 0-1.2) ; direct bilirubin : 13.2 mg/dL and... (truncated)

MESH:D011346 - prochlorperazine

Summary:

LLM Prediction Score	0.500 (normalized)
LLM Confidence Score	0.980
Golden Answer (Severity Class)	0.25 (normalized)
Prediction Error	0.250

Retrieved Context:

Title: Hepatobiliary diseases during pregnancy and their management: An update.
Liver diseases in pregnancy although rare but they can seriously affect mother and fetus. Signs and symptoms are often not specific and consist of jaundice, nausea, vomiting, and abdominal pain. Although any type of liver disease can develop during pregnancy or pregnancy may occur in a patient already having chronic liver disease. All liver diseases with pregnancy can lead to increased maternal and fetal morbidity and mortality. It is difficult to identify features of liver disease in pregnant women because of physiological changes. Physiological changes of normal pregnancy can be confounding with that of sign and symptoms of liver diseases.... (truncated)

Title: A woman with recent jaundice and pruritus.
A middle-aged woman suffering from jaundice and pruritus that had begun a month previously was presented to a physician.At the first assessment, laboratory findings had revealed a cholestatic pattern and serologic tests for hepatitis B virus (HBV), hepatitis C virus (HCV) and hepatitis A virus (HAV) were negative. Normal findings of abdominal computed tomography (CT) scan and endoscopic retrograde cholangiopancreatography (ERCP) ruled out extrahepatic causes of cholestasis. A liver biopsy was done and showed intrahepatic cholestasis without destruction of the bile ducts or granuloma.We assessed the intrahepatic causes of cholestasis. Finally the diagnosis was confirmed by means of a simple... (truncated)

Title: Lisinopril-Induced Liver Injury: An Unusual Presentation and Literature Review.
Lisinopril is an angiotensin converting enzyme inhibitor (ACE-I) that has been on market for more than 25 years. ACE-I are usually well tolerated and rarely have serious or life-threatening side effects. We describe an unusual presentation of fulminant hepatic cholestasis probably secondary to lisinopril. To our knowledge, this is the second case report which shows lisinopril-induced liver injury though a cholestatic mechanism. The patient was a 59-year-old woman with type 2 diabetes, a high body mass index and hypertension, who presented with a 5-week history of jaundice and itching. She had been started on lisinopril for diabetic nephropathy 8 weeks... (truncated)

Title: Pharmacological screening of silibinin for antischizophrenic activity along with its acute toxicity evaluation in experimental animals.
Silibinin (SIL), a flavolignan extracted from the medicinal plant "silybum marianum (milk thistle)", has traditionally been used to treat liver disease. This phytochemical has displayed neuroprotective properties, its activity against schizophrenia is not elucidated. The present study was designed to evaluate the antipsychotic potential of silibinin and probe its toxic potential. The acute oral toxicity study was assessed as per OECD 425 guidelines. Animals were divided into two groups of female rats (n = 6): one group served as the normal control and the other group received a 2,000 mg/kg dose of SIL. We also evaluated the antipsychotic potential of SIL.... (truncated)

Title: Detection of anti-isoniazid and anti-cytochrome P450 antibodies in patients with isoniazid-induced liver failure.
Isoniazid (INH)-induced hepatotoxicity remains one of the most common causes of drug-induced idiosyncratic liver injury and liver failure. This form of liver injury is not believed to be immune-mediated because it is not usually associated with fever or rash, does not recur more rapidly on rechallenge, and previous studies have failed to identify anti-INH antibodies (Abs). In this study, we found Abs present in sera of 15 of 19 cases of INH-induced liver failure. Anti-INH Abs were present in 8 sera; 11 had anti-cytochrome P450 (CYP)2E1 Abs, 14 had Abs against CYP2E1 modified by INH, 14 had anti-CYP3A4 antibodies, and... (truncated)

MESH:D000068580 - varenicline

Summary:

LLM Prediction Score	0.500 (normalized)
LLM Confidence Score	0.990
Golden Answer (Severity Class)	0.25 (normalized)
Prediction Error	0.250

Retrieved Context:

Title: Levamisole-induced Wegener's granulomatosis following contaminated cocaine abuse.
A 44-year-old woman with a medical history of chronic pain syndrome presented with a 3-day history of a painful "rash" that started on her face and spread to her legs. Further history revealed that she recently started a new medication, varenicline, 7 weeks prior to admission and had a long-standing history of intranasal cocaine use. Review of systems was significant for rhinitis, nasal congestion, joint pain, and a febrile episode 2 days prior to admission. Physical examination revealed centrally violaceous, tender, stellate, and retiform purpuric patches and plaques on her extremities, nasal dorsum, and cheeks. Approximately 1.0-centimeter tender purpuric nodules... (truncated)

Title: Drug-induced liver injury due to varenicline: a case report.
Liver injury due to prescription and nonprescription medications is an expanding public health concern in the United States, with drug-induced liver injury (DILI) being the single most common reason for regulatory actions instituted by the Food and Drug Administration against certain medications and supplements.

Title: Two-step progression of varenicline-induced autoimmune hepatitis.
We describe a rare case of drug-induced hepatitis due to the smoking cessation agent varenicline in a 46-year-old Asian woman. The liver injury progressed in two steps. First, the liver injury started in the absence of viral/autoimmune responses, and withdrawal of varenicline lowered the increase in the levels of liver enzymes immediately. Such findings suggested varenicline-induced liver injury. Second, hepatitis recurred in association with conversion of antinuclear antibody from negative to positive about 8 weeks after the initial episode. Histology upon recurrence of liver injury revealed interface hepatitis with lymphocytic and lymphoplasmacytic portal inflammatory infiltrates extending into lobules. Such findings... (truncated)

Title: Acute hepatic injury associated with varenicline in a patient with underlying liver disease.
To report a case of acute hepatic injury associated with varenicline.

Title: Alcohol Interaction with Cocaine, Methamphetamine, Opioids, Nicotine, Cannabis, and γ-Hydroxybutyric Acid.
Millions of people around the world drink alcoholic beverages to cope with the stress of modern lifestyle. Although moderate alcohol drinking may have some relaxing and euphoric effects, uncontrolled drinking exacerbates the problems associated with alcohol abuse that are exploding in quantity and intensity in the United States and around the world. Recently, mixing of alcohol with other drugs of abuse (such as opioids, cocaine, methamphetamine, nicotine, cannabis, and γ-hydroxybutyric acid) and medications has become an emerging trend, exacerbating the public health concerns. Mixing of alcohol with other drugs may additively or synergistically augment the seriousness of the adverse effects... (truncated)

MESH:D011339 - probenecid

Summary:

LLM Prediction Score	0.500 (normalized)
LLM Confidence Score	0.980
Golden Answer (Severity Class)	0.75 (normalized)
Prediction Error	0.250

Retrieved Context:

Title: Influence of Probenecid on the Pharmacokinetics and Pharmacodynamics of Sorafenib.
Prior studies have demonstrated an organic anion transporter 6 (OAT6)-mediated accumulation of sorafenib in keratinocytes. The OAT6 inhibitor probenecid decreases sorafenib uptake in skin and might, therefore, decrease sorafenib-induced cutaneous adverse events. Here, the influence of probenecid on sorafenib pharmacokinetics and toxicity was investigated. Pharmacokinetic sampling was performed in 16 patients on steady-state sorafenib treatment at days 1 and 15 of the study. Patients received sorafenib (200-800 mg daily) in combination with probenecid (500 mg two times daily (b.i.d.)) on days 2-15. This study was designed to determine bioequivalence with geometric mean Area under the curve from zero to twelve... (truncated)

Title: Emodin-induced hepatotoxicity was exacerbated by probenecid through inhibiting UGTs and MRP2.
Aggravating effect of probenecid (a traditional anti-gout agent) on emodin-induced hepatotoxicity was evaluated in this study. 33.3% rats died in combination group, while no death was observed in rats treated with emodin alone or probenecid alone, indicating that emodin-induced (150 mg/kg) hepatotoxicity was exacerbated by probenecid (100 mg/kg). In toxicokinetics-toxicodynamics (TK-TD) study, aspartate aminotransferase (AST) and systemic exposure (area under the serum concentration-time curve, AUC) of emodin and its glucuronide were significantly increased in rats after co-administrated with emodin and probenecid for 28 consecutive days. Results showed that the increased AUC (increased by 85.9%) of emodin was mainly caused by... (truncated)

Title: Probenecid, an Old Drug with Potential New Uses for Central Nervous System Disorders and Neuroinflammation.
Probenecid is an old uricosuric agent used in clinics to treat gout and reduce the renal excretion of antibiotics. In recent years, probenecid has gained attention due to its ability to interact with membrane proteins such as TRPV2 channels, organic anion transporters, and pannexin 1 hemichannels, which suggests new potential therapeutic utilities in medicine. Some current functions of probenecid include their use as an adjuvant to increase the bioavailability of several drugs in the Central Nervous System (CNS). Numerous studies also suggest that this drug has important neuroprotective, antiepileptic, and anti-inflammatory properties, as evidenced by their effect against neurological and... (truncated)

Title: Probenecid, an organic anion transporter 1 and 3 inhibitor, increases plasma and brain exposure of N-acetylcysteine.
1. N-acetylcysteine (NAC) is being investigated as an antioxidant for several conditions including traumatic brain injury, but the mechanism by which it crosses membrane barriers is unknown. We have attempted to understand how the transporter inhibitor, probenecid, affects NAC pharmacokinetics and to evaluate the interaction of NAC with transporters. 2. Juvenile Sprague-Dawley rats were administered NAC alone or in combination with probenecid intraperitoneally. Plasma and brain samples were collected serially and NAC concentrations were measured. Transporter studies were conducted with human embryonic kidney-293 cells that overexpress organic anion transporter (OAT)1 or OAT3 and with human multi-drug resistance-associated protein (MRP)1 or... (truncated)

Title: Mechanisms of Hepatic Cholestatic Drug Injury.
Drug-induced cholestasis represents a form of drug-induced liver disease that can lead to severe impairment of liver function. Numerous drugs have been shown to cause cholestasis and consequently bile duct toxicity. However, there is still lack of therapeutic tools that can prevent progression to advanced stages of liver injury. This review focuses on the various pathological mechanisms by which drugs express their hepatotoxic effects, as well as consequences of increased bile acid and toxin accumulation in the hepatocytes.

MESH:D002066 - busulfan

Summary:

LLM Prediction Score	0.750 (normalized)
LLM Confidence Score	0.980
Golden Answer (Severity Class)	1.0 (normalized)
Prediction Error	0.250

Retrieved Context:

Title: Portal hypertension in a patient with chronic myeloid leukaemia.
A patient with chronic myeloid leukaemia treated with busulphan for 4-5 years, developed signs of busulphan toxicity and portal hypertension with ascites, oesophageal varices and jaundice. At post-mortem there was minimal leukaemic infiltration but there were alterations in the liver architecture sufficient to explain the portal hypertension. The pathogenesis of the liver changes and their possible relationship to splenomegaly and busulphan toxicity are considered.

Title: Toxicity of high-dose busulphan and cyclophosphamide as conditioning therapy for allogeneic bone marrow transplantation in adults with haematological malignancies.
The toxicity of the conditioning regimen high-dose busulfan (BU) 16 mg/kg followed by cyclophosphamide (CY) 200 mg/kg has been analysed in 60 adult patients (mean age 36 +/- 9 years) with haematological malignancies, a third of whom had advanced disease, all received the graft from fully HLA-identical siblings. Significant nausea and vomiting were rare during BU administration but occurred in 44% of the patients with CY. Severe mucositis occurred in 30% of patients. Haemorrhagic cystitis occurred in 16% of patients; interstitial pneumonia occurred in 3 patients and was fatal in one. Veno-occlusive disease of the liver occurred in 2 patients... (truncated)

Title: Veno-occlusive disease of the liver after busulfan, melphalan, and thiotepa conditioning therapy: incidence, risk factors, and outcome.
The purpose of this study was to determine the incidence of veno-occlusive disease (VOD) after a high-dose regimen of busulfan, melphalan, and thiotepa and the risk factors for a more severe outcome. We followed 253 consecutive patients with malignant disorders who received autologous transplants after stem cell harvest followed by 12 mg/kg busulfan, 100 mg/m2 melphalan, and 500 mg/m2 thiotepa. Diagnosis of VOD was based on weight gain, hepatomegaly, and jaundice. Risk factors for moderate or severe VOD were identified using logistic regression models. VOD occurred in 70 of 253 patients (28%), of whom 31 (12%) had moderate and 11... (truncated)

Title: Evaluation of Reversed Administration Order of Busulfan (BU) and Cyclophosphamide (CY) as Conditioning on Liver Toxicity in Allogenic Hematopoietic Stem Cell Transplantation (ALL-HSCT).
Background: Busulfan (BU) in combination with cyclophosphamide (CY) is used as an effective conditioning regimen in hematopoietic SCT. Busulfan, depletes glutathione level in liver and causes elevated levels of CY metabolites. Cyclophosphamide metabolites are highly toxic for sinusoidal endothelial cells and cause VOD/ SOS with high mortality rate. Materials and Methods: Between September 2013 and September 2015, all adult patients with acute leukemia who were candidates for myeloablative allogenic SCT and were admitted to Stem Cell Transplantation center were enrolled in this prospective randomized clinical trial. We tested the hypothesis that reverse administration from BU-CY (n=28) to CY-BU group (n=27)... (truncated)

Title: Influence of underlying disease on busulfan disposition in pediatric bone marrow transplant recipients: a nonparametric population pharmacokinetic study.
Busulfan is an alkylating agent used in a conditioning regimen prior to bone marrow transplantation. Busulfan has a narrow therapeutic index, giving rise to major liver toxicity (veno-occlusive disease), and a wide interpatient and intrapatient pharmacokinetic variability. This report presents the results of a population pharmacokinetic analysis leading to models based on underlying diseases requiring bone marrow transplantation. One hundred children received oral busulfan-based conditioning regimens between March 1998 and February 2006. Busulfan pharmacokinetic parameter estimates (Ka, first order absorption rate constant; Vs, volume of distribution related to the body weight; and Cl/F, apparent clearance) were estimated by using the... (truncated)

MESH:C049073 - droxicam

Summary:

LLM Prediction Score	0.750 (normalized)
LLM Confidence Score	0.610
Golden Answer (Severity Class)	1.0 (normalized)
Prediction Error	0.250

Retrieved Context:

Title: [Droxicam-induced hepatitis. Description of 3 new cases and review of the literature].
Three new cases of cholestatic hepatitis caused by droxicam are described, along with a revision of the other eight cases published to date. Itching, asthenia, and jaundice were the most common symptoms. Average age was 62.8 years (range: 45-82 years), and the median time of exposition was 22.7 days (range: 5-50 days). Biochemistry of the liver showed primarily cholestasis and in 4/11 cases hypereosinophilia. Two patients presented elevated levels of cholesterol and triglycerides which disappeared within the month. Clinical manifestations persisted in one patient for eight weeks after the cessation of treatment. The three patients presented in the present series... (truncated)

Title: [Droxicam-induced toxic hepatitis].
We present four cases of hepatitis with clinical features indicating a direct link with Droxicam. In all the cases, the presentation was that of acute hepatitis with subsequent resolution, whereas one patient developed autoimmune chronic active hepatitis. A full evaluation including ultrasound, liver biopsy, and serologic markers supported the diagnosis. Due to the fact that the "Dirección General de Farmacia y Productos Sanitarios" has approved the postponement of dispensation of products with Droxicam from February 25, 1995, this drug should not be considered anymore in the future as a potential cause of liver injury (acute or chronic hepatitis).

Title: [Hepatotoxicity induced by Droxicam: presentation of 4 cases].
Hepatotoxicity has been reported with most of the nonsteroidal antiinflammatory drugs (NSAID). We describe four patients who presented hepatic injury after of treatment with Droxicam, a new NSAID prodrug of piroxicam. Hepatitis was attributed to Droxicam because of the absence of other etiological factors, temporal relation with drug administration, clinical, laboratory and histological picture and evolution favorable after the drug suppression. The hepatic injury was manifested as cholestasis and withdrawal of the drug was followed by biochemical and clinical improvement until the complete normalization in three of the four patients. Postmarketing surveillance is necessary in new drugs with unknown hepatotoxicity.

Title: Risk of acute and serious liver injury associated to nimesulide and other NSAIDs: data from drug-induced liver injury case-control study in Italy.
Drug-induced liver injury is one of the most serious adverse drug reactions and the most frequent reason for restriction of indications or withdrawal of drugs. Some nonsteroidal anti-inflammatory drugs (NSAIDs) were withdrawn from the market because of serious hepatotoxicity. We estimated the risk of acute and serious liver injury associated with the use of nimesulide and other NSAIDs, with a prevalence of use greater than or equal to 5%.

Title: Association of CYP1A1 and CYP1B1 inhibition in in vitro assays with drug-induced liver injury.
Drug-induced liver injury (DILI) is one of the major causes for the discontinuation of drug development and withdrawal of drugs from the market. Since it is known that reactive metabolite formation and being substrates or inhibitors of cytochrome P450s (P450s) are associated with DILI, we systematically investigated the association between human P450 inhibition and DILI. The inhibitory activity of 266 DILI-positive drugs (DILI drugs) and 92 DILI-negative drugs (no-DILI drugs), which were selected from Liver Toxicity Knowledge Base (US Food and Drug Administration), against 8 human P450 forms was assessed using recombinant enzymes and luminescent substrates, and the threshold values... (truncated)

MESH:C006012 - fenofibric acid

Summary:

LLM Prediction Score	0.500 (normalized)
LLM Confidence Score	0.930
Golden Answer (Severity Class)	0.25 (normalized)
Prediction Error	0.250

Retrieved Context:

Title: Biopsy-confirmed fenofibrate-induced severe jaundice: A case report.
Drug-induced liver injury (DILI) is the leading cause of acute liver failure in the United States. DILI is mainly caused by painkillers and fever reducers, and it is often characterized by the type of hepatic injury (hepatocellular or cholestatic). This report presents a case of fenofibrate-induced severe jaundice in a 65-year-old Korean male with no prior history of liver disease. We offer a strategy for patients who present signs of severe liver injury with jaundice and high elevations in serum transaminases.

Title: Fenofibric acid: a new fibrate approved for use in combination with statin for the treatment of mixed dyslipidemia.
The last two to three decades have seen an explosive growth in interest and information regarding cardiovascular disease (CVD) risk assessment and treatment. Evidence for the role of low-density lipoprotein (LDL) in risk has led to a series of clinical guidelines/recommendations on the importance of LDL lowering with statin treatment. There is also substantial evidence on a number of lipoproteins in the initiation and progression of atherosclerosis and CV events. Health care professionals have not embraced easily novel approaches to identifying those at increased risk and more aggressive treatment. This is especially true for non-LDL factors. The use of non-statin... (truncated)

Title: A review on the rationale and clinical use of concomitant rosuvastatin and fenofibrate/fenofibric acid therapy.
Mixed dyslipidemia, characterized by a lipid triad of elevated triglycerides (TG), elevated low-density lipoprotein-cholesterol (LDL-C) and reduced high-density lipoprotein-cholesterol (HDL-C), is a common and frequently difficult to manage condition. The use of combination medications is often needed to effectively treat the lipid triad. The co-administration of statins and fibrates may provide the desired endpoints but safety issues such as toxicity to the muscles, liver and kidneys are a concern. Given the potency of rosuvastatin to lower LDL-C and fenofibrate's effectiveness in lowering TG, the use of this specific combination may be desirable in treating mixed dyslipidemia. Pharmacokinetic studies revealed no... (truncated)

Title: Fenofibrate metabolism in the cynomolgus monkey using ultraperformance liquid chromatography-quadrupole time-of-flight mass spectrometry-based metabolomics.
Fenofibrate, widely used for the treatment of dyslipidemia, activates the nuclear receptor, peroxisome proliferator-activated receptor alpha. However, liver toxicity, including liver cancer, occurs in rodents treated with fibrate drugs. Marked species differences occur in response to fibrate drugs, especially between rodents and humans, the latter of which are resistant to fibrate-induced cancer. Fenofibrate metabolism, which also shows species differences, has not been fully determined in humans and surrogate primates. In the present study, the metabolism of fenofibrate was investigated in cynomolgus monkeys by ultraperformance liquid chromatography-quadrupole time-of-flight mass spectrometry (UPLC-QTOFMS)-based metabolomics. Urine samples were collected before and after oral doses... (truncated)

Title: PPARalpha regulates the hepatotoxic biomarker alanine aminotransferase (ALT1) gene expression in human hepatocytes.
In this work, we investigated a potential mechanism behind the observation of increased aminotransferase levels in a phase I clinical trial using a lipid-lowering drug, the peroxisome proliferator-activated receptor (PPAR) alpha agonist, AZD4619. In healthy volunteers treated with AZD4619, serum alanine aminotransferase (ALT) and aspartate aminotransferase (AST) activities were elevated without an increase in other markers for liver injury. These increases in serum aminotransferases have previously been reported in some patients receiving another PPARalpha agonist, fenofibrate. In subsequent in vitro studies, we observed increased expression of ALT1 protein and mRNA in human hepatocytes after treatment with fenofibric acid. The PPAR... (truncated)

MESH:C073460 - cefditoren

Summary:

LLM Prediction Score	0.500 (normalized)
LLM Confidence Score	0.960
Golden Answer (Severity Class)	0.25 (normalized)
Prediction Error	0.250

Retrieved Context:

Title: Cefditoren-induced hepatitis.
There are many daily antibiotic prescriptions, especially beta-lactams in trivial infections such as cystitis in young women. Many of these drugs carry an implicit probability of producing hepatotoxicity, manifested by a nonspecific general picture and elevated analytical transaminases. We must take it into account when making the differential diagnosis in the hepatotoxicity study and suspend it as soon as we recognize it.

Title: Cefditoren versus levofloxacin in patients with exacerbations of chronic bronchitis: serum inflammatory biomarkers, clinical efficacy, and microbiological eradication.
The aim of this open-label, randomized, parallel-group pilot study was to evaluate the efficacy of cefditoren pivoxil and levofloxacin in terms of speed of reduction in inflammatory parameters, clinical recovery, and microbiological eradication.

Title: Nephroprotective Plants: A Review on the Use in Pre-Renal and Post-Renal Diseases.
Kidney diseases are expected to become the fifth leading cause of death by 2040. Several physiological failures classified as pre-, intra-, and post-renal factors induce kidney damage. Diabetes, liver pathologies, rhabdomyolysis, and intestinal microbiota have been identified as pre-renal factors, and lithiasis or blood clots in the ureters, prostate cancer, urethral obstructions, prostate elongation, and urinary tract infections are post-renal factors. Additionally, the nephrotoxicity of drugs has been highlighted as a crucial factor inducing kidney injuries. Due to the adverse effects of drugs, it is necessary to point to other alternatives to complement the treatment of these diseases, such as... (truncated)

Title: The influence of alcoholic liver disease on serum PIVKA-II levels in patients without hepatocellular carcinoma.
Prothrombin induced by vitamin K defi-ciency or antagonist II (PIVKA-II) is a widely used diagnostic marker for hepatocellular carcinoma (HCC). We evaluated the correlation between alcoholic liver disease (ALD) and serum PIVKA-II levels in chronic liver disease (CLD) patients.

Title: [Safety in the selection of oral antibiotic treatment in community infections, beyond COVID-19].
Oral antibiotics are among the most frequently used medications in the community. Its adverse effects are generally considered to be infrequent and mild, and include allergies, toxicities and drug interactions. Antibiotics are able to harm patients by various mechanisms, not always well known. Knowledge of the clinically relevant antibiotic-associated adverse effects can allow a judicious use based on the principle first do no harm, primun non nocere. In this review we explore the main adverse effects of oral antibiotics with specific focus on β-lactams, macrolides, and fluoroquinolones.

MESH:C473384 - lumiracoxib

Summary:

LLM Prediction Score	0.750 (normalized)
LLM Confidence Score	0.850
Golden Answer (Severity Class)	1.0 (normalized)
Prediction Error	0.250

Retrieved Context:

Title: Tolerability of selective cyclooxygenase 2 inhibitors used for the treatment of rheumatological manifestations of inflammatory bowel disease.
Nonsteroidal anti-inflammatory drugs (NSAIDs) are used to reduce inflammatory pain and swelling in inflammatory bowel disease (IBD) patients with rheumatological manifestations. While these drugs effectively reduce musculoskeletal pain and stiffness, long-term use is limited by gastrointestinal (GI) adverse effects (AEs) and disease exacerbation. As an alternative to NSAIDs, selective cyclooxygenase 2 (COX-2) inhibitors were developed to improve GI safety and tolerability. COX-2 inhibitors include drugs such as celecoxib, rofecoxib, valdecoxib, etoricoxib, and lumiracoxib. Rofecoxib and valdecoxib have been withdrawn from the market worldwide due to safety concerns (most importantly for cardiovascular adverse events) and lumiracoxib has been withdrawn in many... (truncated)

Title: Mechanisms of drug-induced liver injury: from bedside to bench.
The low incidence of idiosyncratic drug-induced liver injury (DILI), together with the lack of a reliable diagnostic biomarker and robust preclinical and in vitro toxicology test systems for the condition have limited our ability to define the mechanisms of DILI. A notable exception is acetaminophen hepatotoxicity, which is associated with the formation of a well-characterized and highly reactive intermediate metabolite, N-acetyl-p-benzoquinone imine. However, studies have also suggested a role for the host immune response and variation in the expression of the lymphocyte CD44 gene in the pathogenesis of acetaminophen hepatotoxicity. A careful review of the laboratory, clinical and histological phenotype... (truncated)

Title: A genome-wide study identifies HLA alleles associated with lumiracoxib-related liver injury.
Lumiracoxib is a selective cyclooxygenase-2 inhibitor developed for the symptomatic treatment of osteoarthritis and acute pain. Concerns over hepatotoxicity have contributed to the withdrawal or non-approval of lumiracoxib in most major drug markets worldwide. We performed a case-control genome-wide association study on 41 lumiracoxib-treated patients with liver injury (cases) and 176 matched lumiracoxib-treated patients without liver injury (controls). Several SNPs from the MHC class II region showed strong evidence of association (the top SNP was rs9270986 with P = 2.8 x 10(-10)). These findings were replicated in an independent set of 98 lumiracoxib-treated cases and 405 matched lumiracoxib-treated controls (top... (truncated)

Title: Efficacy and tolerability of lumiracoxib, a highly selective cyclo-oxygenase-2 (COX2) inhibitor, in the management of pain and osteoarthritis.
Lumiracoxib is a COX2 inhibitor that is highly selective, is more effective than placebo on pain in osteoarthritis (OA), with similar analgesic and anti-inflammatory effects as non-selective NSAIDs and the selective COX2 inhibitor celecoxib, has a lower incidence of upper gastrointestinal (GI) side effects in patients not taking aspirin, and a similar incidence of cardiovascular (CV) side effects compared to naproxen or ibuprofen. In the context of earlier guidelines and taking into account the GI and CV safety results of the TARGET study, lumiracoxib had secured European Medicines Agency (EMEA) approval with as indication symptomatic treatment of OA as well... (truncated)

Title: Clinical pharmacology of lumiracoxib, a second-generation cyclooxygenase 2 selective inhibitor.
Although highly selective cyclooxygenase (COX)-2 inhibitors have been shown to be less toxic to the gastrointestinal tract than conventional non-steroidal anti-inflammatory drugs (NSAIDs), their overall safety profile is questioned. Since different selective COX-2 inhibitors were found to be associated with increased cardiovascular thrombotic events, the thrombotic hazard may be a class effect. Furthermore, warnings have been issued regarding serious skin and hypersensitivity reactions associated with valdecoxib. Lumiracoxib is a novel COX-2 selective inhibitor (coxib) with improved biochemical selectivity over that of currently available coxibs. It is structurally distinct from other drugs in the class and has weakly acidic properties. Clinical... (truncated)

MESH:D014268 - trifluoperazine

Summary:

LLM Prediction Score	0.500 (normalized)
LLM Confidence Score	0.980
Golden Answer (Severity Class)	0.25 (normalized)
Prediction Error	0.250

Retrieved Context:

Title: Trifluoperazine-induced cholestatic jaundice.
Liver injury occurs with many drugs; therefore, a thorough work up is important for establishing the diagnosis. We report a case of trifluoperazine-induced cholestatic jaundice. A 44-year old male with schizoaffective disorder developed an increase in liver enzymes and jaundice after starting treatment with trifluoperazine. Workup for other potential etiologies was negative.

Title: Trifluoperazine inhibits acetaminophen-induced hepatotoxicity and hepatic reactive nitrogen formation in mice and in freshly isolated hepatocytes.
The hepatotoxicity of acetaminophen (APAP) occurs by initial metabolism to N-acetyl-p-benzoquinone imine which depletes GSH and forms APAP-protein adducts. Subsequently, the reactive nitrogen species peroxynitrite is formed from nitric oxide (NO) and superoxide leading to 3-nitrotyrosine in proteins. Toxicity occurs with inhibited mitochondrial function. We previously reported that in hepatocytes the nNOS (NOS1) inhibitor NANT inhibited APAP toxicity, reactive nitrogen and oxygen species formation, and mitochondrial dysfunction. In this work we examined the effect of trifluoperazine (TFP), a calmodulin antagonist that inhibits calcium induced nNOS activation, on APAP hepatotoxicity and reactive nitrogen formation in murine hepatocytes and <i>in vivo</i>. In... (truncated)

Title: Mechanisms of acetaminophen-induced liver necrosis.
Although considered safe at therapeutic doses, at higher doses, acetaminophen produces a centrilobular hepatic necrosis that can be fatal. Acetaminophen poisoning accounts for approximately one-half of all cases of acute liver failure in the United States and Great Britain today. The mechanism occurs by a complex sequence of events. These events include: (1) CYP metabolism to a reactive metabolite which depletes glutathione and covalently binds to proteins; (2) loss of glutathione with an increased formation of reactive oxygen and nitrogen species in hepatocytes undergoing necrotic changes; (3) increased oxidative stress, associated with alterations in calcium homeostasis and initiation of signal... (truncated)

Title: The development and hepatotoxicity of acetaminophen: reviewing over a century of progress.
Acetaminophen (APAP) was first synthesized in the 1800s, and came on the market approximately 65 years ago. Since then, it has become one of the most used drugs in the world. However, it is also a major cause of acute liver failure. Early investigations of the mechanisms of toxicity revealed that cytochrome P450 enzymes catalyze formation of a reactive metabolite in the liver that depletes glutathione and covalently binds to proteins. That work led to the introduction of N-acetylcysteine (NAC) as an antidote for APAP overdose. Subsequent studies identified the reactive metabolite N-acetyl-<i>p</i>-benzoquinone imine, specific P450 enzymes involved, the mechanism of... (truncated)

Title: Repositioning of the antipsychotic drug TFP for sepsis treatment.
Sepsis is a disease responsible for the death of almost all critical patients. Once infected by virus or bacteria, patients can die due to systemic inflammation within a short period of time. Cytokine storm plays an essential role in causing organ dysfunction and septic shock. Thus, inhibition of cytokine secretion is considered very important in sepsis therapy. In this study, we found that TFP, an antipsychotic drug mainly used to treat schizophrenia by suppressing dopamine secretion, inhibited cytokine release from activated immune cells both in vitro and in vivo. Trifluoperazine (TFP) decreased the levels of pro-inflammatory cytokines without altering their... (truncated)

MESH:D010118 - oxytetracycline

Summary:

LLM Prediction Score	0.500 (normalized)
LLM Confidence Score	0.970
Golden Answer (Severity Class)	0.25 (normalized)
Prediction Error	0.250

Retrieved Context:

Title: [A case of severe methacycline damage to the liver and bone marrow].
A case of a male patient with bronchopneumonia incorrectly treated for a long time with methacycline (rondomycin), an oxytetracycline drug, is reported. methacycline was applied in a dose of 8 capsules daily (2 capsules 4 times) in the course of 2 1/2 months, the total dose amounting to about 150 g. The patient developed severe toxic hepatitis as a result of this incorrect treatment. The hepatitis was manifested by jaundice and cytolysis. The bone marrow was also affected--hypoplasia marked by combined depression of leuko-, erythro- and thrombopoiesis and peripheral pancytopenia. In addition chloramphenicol treatment was applied which increased the toxic... (truncated)

Title: Hepatic safety of antibiotics used in primary care.
Antibiotics used by general practitioners frequently appear in adverse-event reports of drug-induced hepatotoxicity. Most cases are idiosyncratic (the adverse reaction cannot be predicted from the drug's pharmacological profile or from pre-clinical toxicology tests) and occur via an immunological reaction or in response to the presence of hepatotoxic metabolites. With the exception of trovafloxacin and telithromycin (now severely restricted), hepatotoxicity crude incidence remains globally low but variable. Thus, amoxicillin/clavulanate and co-trimoxazole, as well as flucloxacillin, cause hepatotoxic reactions at rates that make them visible in general practice (cases are often isolated, may have a delayed onset, sometimes appear only after cessation... (truncated)

Title: Cinnamon Aqueous Extract Attenuates Diclofenac Sodium and Oxytetracycline Mediated Hepato-Renal Toxicity and Modulates Oxidative Stress, Cell Apoptosis, and Inflammation in Male Albino Rats.
Among commonly consumed anti-inflammatory and antimicrobial drugs are diclofenac sodium (DFS) and oxytetracycline (OTC), especially in developing countries because they are highly effective and cheap. However, the concomitant administration of anti-inflammatory drugs with antibiotics may exaggerate massive toxic effects on many organs. Cinnamon (<i>Cinnamomum zeylanicum</i>, Cin) is considered one of the most broadly utilized plants with various antioxidant and anti-inflammatory actions. This study aimed to evaluate the possible protective effects of cinnamon aqueous extract (Cin) against DFS and OTC hepato-renal toxicity. Eight groups (8/group) of adult male albino rats were treated orally for 15 days with physiological saline (control), Cin... (truncated)

Title: The risk of liver damage associated with minocycline: a comparative study.
Using the General Practice Research Database, the authors performed (1) a cohort analysis comparing the incidence of liver dysfunction in new users of minocycline with new users of oxytetracycline/tetracycline and (2) a case control study assessing antibiotic exposure in new cases of liver dysfunction and controls without liver dysfunction. In new users, the incidence of liver dysfunction in those exposed to minocycline was 1.04 cases/10,000 exposed person months (EPM) and 0.69 cases/10,000 EPM in those exposed to oxytetracycline/tetracycline (relative risk 1.51 [CI95: 0.63, 3.65]). The risk in both groups was greatest in the first month of use. The adjusted odds... (truncated)

Title: Detection of Oxytetracycline Residuein Infant Formulaby High-Performance Liquid Chromatography)HPLC(.
Determination of drug residues in food is of great importance due to their toxicity. Long-term exposure with low level of drug residues could be important, especially in children. Based on document study, oxytetracycline)OTC) is a prophylaxis and treatment agent for great number of diseases and possesses a broad spectrum activity against many pathogenic organisms and can be toxic or cause allergic reactions in some hypersensitive individual's if the residues of drug exist in the infant formula. The previous studies show that using high-performance liquid chromatography (HPLC) is useful for OTC detection in milk. Therefore, we decided to measure OTC... (truncated)

MESH:D008911 - minocycline

Summary:

LLM Prediction Score	0.750 (normalized)
LLM Confidence Score	0.990
Golden Answer (Severity Class)	1.0 (normalized)
Prediction Error	0.250

Retrieved Context:

Title: Acute hepatic failure associated with oral minocycline: a case report.
A 39-year-old woman was evaluated for possible liver transplantation due to rapidly developing hepatic failure 4 weeks after initiation of oral minocycline 100 mg twice a day for the treatment of acne. The patient developed a maculopapular rash, malaise, fever, nausea, and vomiting 2 weeks prior to admission to the hospital. On admission, her symptoms rapidly progressed to liver failure characterized by rapidly rising liver enzyme levels, worsening encephalopathy, and coagulopathy. Viral hepatitis serologies and blood cultures were all negative. After intensive supportive care for 2 weeks, the patient's condition gradually improved and she was discharged with mildly elevated liver... (truncated)

Title: Minocycline hepatitis.
Minocycline is an effective antibiotic widely used in the treatment of acne vulgaris. We report a previously well 20-year-old woman who developed liver dysfunction with jaundice and malaise following a 1 year course of minocycline for acne vulgaris. Serum antinuclear antibody was strongly positive (1 : 2560) and liver transaminases were grossly deranged. All other causes of liver disease were excluded. Both the clinical symptoms and laboratory abnormalities resolved spontaneously on stopping the drug. We review the three different types of hepatotoxicity associated with minocycline and draw evidence to support the diagnosis of minocycline-induced autoimmune hepatitis. This case supports the... (truncated)

Title: Drug-induced hepatitis with autoimmune features during minocycline therapy.
A 25-year-old woman with no history of liver disease developed liver dysfunction associated with severe jaundice and general malaise following a prolonged therapy with minocycline for acne vulgaris. Serum anti-nuclear antibody was detected and immunoglobulin G level was elevated. Symptoms resolved and liver function normalized following minocycline discontinuation and corticosteroid administration. Our diagnosis was drug-induced hepatitis with autoimmune features, as liver histology revealed acute hepatitis. Drug-induced hepatitis should be considered when liver dysfunction or systemic symptoms develops during long-term minocycline therapy.

Title: Interstitial pneumonia and hepatitis caused by minocycline.
A 28-year-old patient is described who presented with progressive dyspnoea and jaundice due to interstitial pneumonia and hepatitis. The most likely cause is a drug-related reaction to minocycline. We discuss the different kinds of drug-related reactions that are most likely involved.

Title: [Cutaneous xanthomas associated with minocycline-induced cholestatic jaundice].
Minocycline is an effective treatment of acne vulgaris, especially for inflammatory forms. Prescription rates have increased in recent years accompanied by a number of reports concerning drug-induced side effects. An otherwise healthy woman developed an erythema multiform-like rash and and toxic hepatic damage causing cholestatic jaundice following long-term minocycline use. Unusual cutaneous lipid deposition also developed. Minocycline-induced side effects are reviewed.

MESH:D010389 - pemoline

Summary:

LLM Prediction Score	0.750 (normalized)
LLM Confidence Score	0.940
Golden Answer (Severity Class)	1.0 (normalized)
Prediction Error	0.250

Retrieved Context:

Title: Hepatotoxicity due to pemoline. Report of two cases.
Pemoline, a central nervous system stimulant, has been used extensively in Great Britain and Europe for a variety of conditions including memory failure and depression. In the 10 years that the drug has been used no report of hepatotoxicity has appeared. A prospective study of efficacy and safety of pemoline was started in the United States approximately 3 years ago. This report describes two patients who developed reversible liver abnormalities while receiving pemoline during the study and following rechallenge with the drug.

Title: Hepatotoxicity due to pemoline (Cylert): a report of two cases.
Pemoline (Cylert) is an agent used to treat attention deficit disorders and other behavioral syndromes. There have, however, been three published reports of mild hepatic dysfunction in five patients coincident with pemoline therapy. We report two further cases of probable pemoline hepatotoxicity. One case involved a mild aminotransferase elevation in a 6-year-old who was on pemoline for 5 months. The second case, in an 11-year-old, developed hepatic failure with marked prolongation in prothrombin time nonresponsive to parenteral vitamin K, deep jaundice, and submassive hepatic necrosis. This patient had a long history of pemoline usage. Pharmacokinetics are briefly discussed. A spectrum... (truncated)

Title: Stimulant medication therapy in the treatment of children with attention deficit hyperactivity disorder.
Despite the tremendous research advances that have increased our knowledge regarding the pharmacodynamics, clinical pharmacology, pharmacokinetics, and adverse effects of stimulant medications in the treatment of children with ADHD, our knowledge is yet incomplete. Perhaps the most central unresolved issue concerns our understanding of the pathogenesis, pathophysiology, and diagnosis of ADHD. This review has touched briefly on the controversy and confusion surrounding this issue. Although our understanding of the use of stimulant medications in this disorder is similarly incomplete, a review of the literature does allow certain conclusions to be made that are helpful to the practitioner. 1. Stimulant medications... (truncated)

Title: Ornidazole-induced autoimmune hepatitis.
Certain drugs including oxyphenisatin, methyldopa, nitrofurantoin, diclofenac, interferon, infliximab, pemoline, minocycline, atorvastatin, and rosuvastatin can induce hepatocellular injury that mimics autoimmune hepatitis. Whether drugs and herbs unmask or induce autoimmune hepatitis or simply cause a drug-induced hepatitis with accompanying autoimmune features is unclear. We describe the clinicopathologic details of eight cases with ornidazole-induced hepatitis with autoimmune features.

Title: Pemoline-induced autoimmune hepatitis.
We present a case of pemoline-induced autoimmune hepatitis in a 46-yr-old woman who received the drug for management of her multiple sclerosis. The autoimmune nature of the hepatitis is based on elevation of her anti-nuclear antibodies, anti-thyroid antibodies, IgA, and IgM globulins. These features of autoimmunity disappeared with normalization of her liver-associated enzymes and have remained negative for at least 6 months after completion of prednisone therapy.

MESH:D012293 - rifampin

Summary:

LLM Prediction Score	0.750 (normalized)
LLM Confidence Score	0.990
Golden Answer (Severity Class)	1.0 (normalized)
Prediction Error	0.250

Retrieved Context:

Title: Isoniazid-rifampicin-induced submassive hepatic necrosis.
A 58-year-old woman with tuberculosis received antituberculous drugs which included isoniazid, rifampicin, and ethambutol. Nausea and anorexia were initial symptoms while jaundice and abdominal pain were late manifestations. She became comatose and died 7 weeks after therapy. Autopsy revealed submassive necrosis of the liver and active advanced pulmonary tuberculosis. It is, thus, necessary for the physician to be alert for this serious complication in prescribing a combination of these antituberculous drugs.

Title: [Toxic hepatitis caused by rifampin and isoniazid in treatment of tuberculosis (author's transl)].
Five children with tuberculosis were treated with isoniazid (20 mg./Kg./day) and rifampin (15 mg./Kg./day). After five to twenty seven days of treatment they presented anorexia, vomiting and jaundice. Hepatomegaly was found in two of them. High indirect bilirubin, S.G.O.T. and S.G.P.T. and low prothrombin levels were present in all of them. Eight to thirty one days after withdrawal of rifampin, all patients were well and their laboratory data was normal.

Title: Rifampicin-isoniazid induced fatal fulminant hepatitis during treatment of latent tuberculosis: A case report and literature review.
A 42-year-old Indian man received 450 mg rifampicin (RIF) and 150 mg isoniazid (INH) daily after being diagnosed of a latent tuberculosis infection. Baseline serum aminotransferase and total bilirubin levels were within normal limits. On day 31 of treatment, the patient experienced epigastric discomfort and general malaise and one week later he developed nausea and episodic vomiting. The patient missed his first scheduled clinic appointment and he continued taking RIF-INH despite his symptoms. He visited the tuberculosis clinic on day 47 of treatment where he was found to be jaundiced and his liver enzymes were elevated. RIF-INH was stopped and... (truncated)

Title: Adverse effects of rifampin.
Rifampin has proved to be a valuable antibiotic with relatively few major adverse effects. Its toxicity is predominantly hepatic and immunoallergic in character. While hepatic toxicity is dose related and has been observed mainly in patients with underlying liver disease, the immunoallergic effects are usually associated with intermittent or prolonged therapy. These immunoallergic effects may be minor (a cutaneous, gastrointestinal, or influenzalike syndrome) or major (hemolytic anemia, shock, or acute renal failure).

Title: Isoniazid-rifampin fulminant hepatitis. A possible consequence of the enhancement of isoniazid hepatotoxicity by enzyme induction.
The authors report 6 cases of fulminant hepatitis in patients treated with isoniazid and rifampin. In 4 of these patients, the treatment had been started within 3 days after a general anesthesia. The course of the disease was remarkably similar in all 6 patients: (1) the time interval from the beginning of the isoniazid-rifampin administration to the onset of jaundice was 6 to 10 days; (2) disorders of consciousness appeared less than 3 days after the onset of jaundice; (3) serum transaminases were 26 to 80 times the upper limit of normal; (4) the main liver lesion was centrilobular necrosis;... (truncated)

MESH:C031967 - rasagiline

Summary:

LLM Prediction Score	0.126 (normalized)
LLM Confidence Score	0.980
Golden Answer (Severity Class)	0.375 (normalized)
Prediction Error	0.249

Retrieved Context:

Title: Long-term, open-label, phase 3 study of rasagiline in Japanese patients with early Parkinson's disease.
Rasagiline is a monoamine oxidase B inhibitor with demonstrated efficacy and safety in patients with Parkinson's disease (PD). We recently conducted the first randomized, double-blind, placebo-controlled trial of rasagiline in Japanese patients with early PD and now report the results of its open-label extension (clinicaltrials.gov, NCT02337751). In the double-blind trial, patients aged 30-79 years with PD diagnosis within 5 years and Movement Disorder Society-Unified Parkinson's Disease Rating Scale (MDS-UPDRS) Part II + Part III total score ≥ 14 were randomized to placebo or rasagiline 1 mg/day for 26 weeks. Of 210 patients who completed the randomized trial, 198 (95 placebo,... (truncated)

Title: Safety comparisons among monoamine oxidase inhibitors against Parkinson's disease using FDA adverse event reporting system.
Monoamine oxidase B (MAO-B) inhibitors are used to control Parkinson's disease (PD). Selegiline, rasagiline, and safinamide are widely used as MAO-B inhibitors worldwide. Although these drugs inhibit MAO-B, there are pharmacological and chemical differences, such as the inhibitory activity, the non-dopaminergic properties in safinamide, and the amphetamine-like structure in selegiline. MAO-B inhibitors may differ in adverse events (AEs). However, differences in actual practical clinics are not fully investigated. A retrospective study was conducted using FAERS, the largest database of spontaneous adverse events. AE signals for MAO-B inhibitors, including selegiline, rasagiline, and safinamide, were detected using the reporting odds ratio method... (truncated)

Title: Acute myeloid leukaemia presenting as acute liver failure-a case report and literature review.
A 75-year-old woman presented with rapidly progressive fatigue, abdominal pain and jaundice. Physical examination revealed tender abdomen and splenomegaly. Magnetic resonance cholangiogram showed marked hepatomegaly, splenomegaly and scattered nodules or masses in the liver and spleen. The patient expired from multiorgan failure. Autopsy revealed infiltration of the liver, spleen and bone marrow by acute myeloid leukaemia.

Title: Drug treatment of Parkinson's disease.
Parkinson's disease (PD) is a common neurodegenerative disease. While its cause remains elusive, much progress has been made regarding its treatment. Available drugs have a good symptomatic effect, but none has yet been shown to slow the progression of the disease in humans. The most efficacious drug is levodopa, but it remains unclear whether the symptomatic benefit is associated with neurotoxic effects and long-term deterioration. The long-term problem associated with levodopa is the appearance of dyskinesias, which is significantly delayed among patients treated with dopamine agonists as initial therapy. Less clear is the role of other drugs in PD, such... (truncated)

Title: Adjunctive therapy in Parkinson's disease: the role of rasagiline.
Parkinson's disease is the second most common neurodegenerative disorder, currently affecting 1.5 million people in the US. In this review, we describe the diagnostic and pathological features of Parkinson's disease, as well as its clinical course. We then review pharmacologic treatments for the disease, with a particular focus on therapies adjunctive to levodopa and specifically the role of rasagiline. We review the four pivotal rasagiline trials, and discuss rasagiline and its use as adjunctive therapy for Parkinson's disease. Finally, we discuss potential side effects, drug interactions, and other practical aspects concerning the use of rasagiline in Parkinson's disease.

MESH:D010643 - phenoxybenzamine

Summary:

LLM Prediction Score	0.244 (normalized)
LLM Confidence Score	0.960
Golden Answer (Severity Class)	0.0 (normalized)
Prediction Error	0.244

Retrieved Context:

Title: Utility of spherical human liver microtissues for prediction of clinical drug-induced liver injury.
Drug-induced liver injury (DILI) continues to be a major source of clinical attrition, precautionary warnings, and post-market withdrawal of drugs. Accordingly, there is a need for more predictive tools to assess hepatotoxicity risk in drug discovery. Three-dimensional (3D) spheroid hepatic cultures have emerged as promising tools to assess mechanisms of hepatotoxicity, as they demonstrate enhanced liver phenotype, metabolic activity, and stability in culture not attainable with conventional two-dimensional hepatic models. Increased sensitivity of these models to drug-induced cytotoxicity has been demonstrated with relatively small panels of hepatotoxicants. However, a comprehensive evaluation of these models is lacking. Here, the predictive value... (truncated)

Title: Effect of phenoxybenzamine (POB) on portal venous pressure in patients with portal hypertension.
In order to assess an effect of phenoxybenzamine (POB) on portal circulation, POB (0.5-1.0 mg./kg.) was administered intravenously to six patients with portal hypertension and two patients without portal hypertension. In patients with portal hypertension, POB reduced portal venous pressure (PVP) from 362.5 +/- 53.8 mm. H2O to 282.5 +/- 50.4 mm. H2O (P less than 0.001) where central venous pressure (CVP) was maintained constant. In patients without portal hypertension, change in PVP was in parallel with that in CVP where the decrease in PVP was regarded as not specific. This preferential reduction of PVP in portal hypertension seemed to... (truncated)

Title: The evolution of strategies to minimise the risk of human drug-induced liver injury (DILI) in drug discovery and development.
Early identification of toxicity associated with new chemical entities (NCEs) is critical in preventing late-stage drug development attrition. Liver injury remains a leading cause of drug failures in clinical trials and post-approval withdrawals reflecting the poor translation between traditional preclinical animal models and human clinical outcomes. For this reason, preclinical strategies have evolved over recent years to incorporate more sophisticated human in vitro cell-based models with multi-parametric endpoints. This review aims to highlight the evolution of the strategies adopted to improve human hepatotoxicity prediction in drug discovery and compares/contrasts these with recent activities in our lab. The key role of... (truncated)

Title: Relationships Between Pharmacovigilance, Molecular, Structural, and Pathway Data: Revealing Mechanisms for Immune-Mediated Drug-Induced Liver Injury.
Immune-mediated drug-induced liver injury (IMDILI) can be devastating, irreversible, and fatal in the absence of successful transplantation surgery. We present a novel approach that combines the methods of pharmacoepidemiology with in silico molecular modeling to identify specific features in toxic ligands that are associated with clinical features of IMDILI. Specifically, from pharmacovigilance data multivariate logistic regression identified 18 drugs associated with IMDILI ($P < 0.00015$). Eleven of these drugs, along with their known and proposed metabolites, constituted a training set used to develop a four-point pharmacophore model (sensitivity 75%; specificity 85%). Subsequently, this information was combined with information from immune-pathway... (truncated)

Title: Pheochromocytoma with markedly abnormal liver function tests and severe leukocytosis.
Pheochromocytoma is a rare neuroendocrine tumor arising from the medulla of the adrenal glands, which causes an overproduction of catecholamines. The common symptoms are headache, palpitations, and sweating; however, various other clinical manifestations might also be present. Accurate diagnosis of pheochromocytoma is important because surgical treatment is usually successful, and associated clinical problems are reversible if treated early. A 49-year-old man with a history of uncontrolled hypertension and diabetes mellitus presented with chest pain, fever, and sweating. His liver function tests and white blood cell counts were markedly increased and his echocardiography results suggested stress-induced cardiomyopathy. His abdominal computed tomography... (truncated)

MESH:C011941 - nabilone

Summary:

LLM Prediction Score	0.241 (normalized)
LLM Confidence Score	0.980
Golden Answer (Severity Class)	0.0 (normalized)
Prediction Error	0.241

Retrieved Context:

Title: Safety Considerations in Cannabinoid-Based Medicine.
Cannabinoids are a diverse class of chemical compounds that are increasingly recognized as potential therapeutic options for a range of conditions. While many studies and reviews of cannabinoids focus on efficacy, safety is much less well reported. Overall assessment of the safety of cannabinoid-based medicines is confounded by confusion with recreational cannabis use as well as different study designs, indications, dosing, and administration methods. However, clinical studies in registered products are increasingly available, and this article aims to discuss and clarify what is known regarding the safety profiles of cannabinoid-based medicines, focusing on the medical and clinical safety evidence and... (truncated)

Title: Therapeutic potential and safety considerations for the clinical use of synthetic cannabinoids.
The phytocannabinoid Δ⁹-tetrahydrocannabinol (THC) was isolated and synthesized in the 1960s. Since then, two synthetic cannabinoids (SCBs) targeting the cannabinoid 1 (CB1R) and 2 (CB2R) receptors were approved for medical use based on clinical safety and efficacy data: dronabinol (synthetic THC) and nabilone (synthetic THC analog). To probe the function of the endocannabinoid system further, hundreds of investigational compounds were developed; in particular, agonists with (1) greater CB1/2R affinity relative to THC and (2) full CB1/2R agonist activity. This pharmacological profile may pose greater risks for misuse and adverse effects relative to THC, and these SCBs proliferated in retail markets... (truncated)

Title: Medicinal Cannabis-Potential Drug Interactions.
The endocannabinoids system (ECS) has garnered considerable interest as a potential therapeutic target in various carcinomas and cancer-related conditions alongside neurodegenerative diseases. Cannabinoids are implemented in several physiological processes such as appetite stimulation, energy balance, pain modulation and the control of chemotherapy-induced nausea and vomiting (CINV). However, pharmacokinetics and pharmacodynamics interactions could be perceived in drug combinations, so in this short review we tried to shed light on the potential drug interactions of medicinal cannabis. Hitherto, few data have been provided to the healthcare practitioners about the drug^{-}drug interactions of cannabinoids with other prescription medications. In general, cannabinoids are usually... (truncated)

Title: Small Molecules from Nature Targeting G-Protein Coupled Cannabinoid Receptors: Potential Leads for Drug Discovery and Development.
The cannabinoid molecules are derived from Cannabis sativa plant which acts on the cannabinoid receptors types 1 and 2 (CB1 and CB2) which have been explored as potential therapeutic targets for drug discovery and development. Currently, there are numerous cannabinoid based synthetic drugs used in clinical practice like the popular ones such as nabilone, dronabinol, and Δ(9)-tetrahydrocannabinol mediates its action through CB1/CB2 receptors. However, these synthetic based Cannabis derived compounds are known to exert adverse psychiatric effect and have also been exploited for drug abuse. This encourages us to find out an alternative and safe drug with the least psychiatric... (truncated)

Title: Toxicological properties of Δ9-tetrahydrocannabinol and cannabidiol.
Cannabis sativa L. contains more than 100 phytocannabinoids that can interact with cannabinoid receptors CB1 and CB2. None of the cannabinoid receptor ligands is entirely CB1- or CB2-specific. The effects of cannabinoids therefore differ not just because of different potency at cannabinoid receptors but also because they can interact with other non-CB1 and non-CB2 targets, such as TRPV1, GPR55, and GPR119. The most studied phytocannabinoid is Δ9-tetrahydrocannabinol (THC). THC is a partial agonist at both cannabinoid receptors, but its psychotomimetic effect is produced primarily via activation of the CB1 receptor, which is strongly expressed in the central nervous system, with... (truncated)

MESH:D003622 - dapsone

Summary:

LLM Prediction Score	0.615 (normalized)
LLM Confidence Score	0.990
Golden Answer (Severity Class)	0.375 (normalized)
Prediction Error	0.240

Retrieved Context:

Title: Dapsone hypersensitivity syndrome with circulating 190-kDa and 230-kDa autoantibodies.
Dapsone has potent anti-inflammatory effects, and is used in the treatment of leprosy, cutaneous vasculitis, neutrophilic dermatoses, and dermatitis herpetiformis and other blistering disorders. However, it may cause severe adverse reactions such as hypersensitivity syndrome, which is characterized by fever, skin rash, hepatitis and lymphadenopathy. We report a 44-year-old female Korean patient with dapsone hypersensitivity syndrome (DHS) that presented as a bullous skin eruption. The patient had a 1-year history of urticarial vasculitis, treated with antihistamines, prednisolone and dapsone. Although the skin lesions improved, she reported fever, nausea, abdominal pain, jaundice, fatigue and skin rashes. On physical examination, there were... (truncated)

Title: Dapsone hypersensitivity syndrome: a clinico-epidemiological review.
Diaminodiphenyl sulphone (dapsone) is a drug of choice in the treatment of leprosy. It is also useful for the treatment of many neutrophilic and other dermatoses. Dapsone hypersensitivity syndrome is a rare but well recognized serious adverse effect characterized by fever, skin rashes, generalized lymphadenopathy, hepatitis, and hepato-splenomegaly. Twenty-six patients with dapsone hypersensitivity syndrome were studied for clinical profile, outcome, and prognosis. The male:female ratio was 2.2:1, and the mean age was 33.19 years (range 13 to 64 years). The interval between start of dapsone therapy and appearance of symptoms varied from 2-7 weeks (mean 29.82 days). Twenty-four patients received... (truncated)

Title: Dapsone-induced pure red cell aplasia and cholestatic jaundice: A new experience for diagnosis and management.
Dapsone (4,4'- diaminodiphenylsulfone) is the parent compound of the sulfones, and it has potent antiparasitic, anti-inflammatory, and immunomodulatory effects. It is used in the treatment of leprosy, dermatitis herpetiformis, and prophylactically to prevent Pneumocystis pneumonia and toxoplasmosis in patients unable to tolerate trimethoprim with sulfamethoxazole. We hereby report a case of dapsone toxicity who developed pure red cell aplasia and cholestatic jaundice in a suspected case of dermatitis herpetiformis. Patient had an excellent response to corticosteroids after withdrawal of dapsone.

Title: [Adverse effects of Disulone; results of the France pharmacovigilance inquiry. Regional Centers of Pharmacovigilance].
Disulone (dapsone + ferrous oxalate) is a sulphone marketed in France since 1958 and authorized in P. Carinii prophylaxis in HIV+ cotrimoxazole intolerant patients, bullous dermatosis, leprosy and polychondritis. Between 1983 and 1998, 249 adverse reactions were reported to French pharmacovigilance centres and Aventis, the manufacturer. Every side-effect was reviewed and the causal relationship was assessed on the basis of the French method for causality assessment. Main side-effects were divided as follows: 117 blood dyscrasias (generally neutropenia and agranulocytosis, rarely methaemoglobinaemia, haemolysis, macrocytosis, anaemia, aplastic anaemia, haemochromatosis and sulphaemoglobinaemia); 29 hypersensitivity syndrome; 39 cutaneous reactions, generally rash; 27 liver injuries... (truncated)

Title: Dapsone in rheumatoid arthritis.
Dapsone, a synthetic sulfone with chemical similarities to sulfapyridine, has been used for a number of years to treat leprosy and dermatitis herpetiformis. Recently, a number of prospective, randomized, double-blind trials have shown their success in the management of rheumatoid arthritis, with dapsone being superior to placebo and comparable to chloroquine and hydroxychloroquine. Its mode of anti-inflammatory actions in rheumatoid arthritis is not clearly understood, but modulation of neutrophil activity or inhibition of neutrophil inflammatory product formation or release appear to play a role. The major limiting side effect is hemolytic anemia, which may be mitigated through careful patient selection,... (truncated)

MESH:D009553 - nimodipine

Summary:

LLM Prediction Score	0.135 (normalized)
LLM Confidence Score	0.980
Golden Answer (Severity Class)	0.375 (normalized)
Prediction Error	0.240

Retrieved Context:

Title: Calcium channel blockers ameliorate iron overload-associated hepatic fibrosis by altering iron transport and stellate cell apoptosis.
Liver fibrosis is the principal cause of morbidity and mortality in patients with iron overload. Calcium channel blockers (CCBs) can antagonize divalent cation entry into renal and myocardial cells and inhibit fibrogenic gene expression. We investigated the potential of CCBs to resolve iron overload-associated hepatic fibrosis. Kunming mice were assigned to nine groups (n=8 per group): control, iron overload, deferoxamine, high and low dose verapamil, high and low dose nimodipine, and high and low dose diltiazem. Iron deposition and hepatic fibrosis were measured in mouse livers. Expression levels of molecules associated with transmembrane iron transport were determined by molecular biology... (truncated)

Title: Inconsistencies and Ambiguities in Liver-Disease-Related Contraindications-A Systematic Analysis of SmPCs/PI of Major Drug Markets.
Liver disease is a common condition worldwide that can cause alterations in drug disposition and susceptibility to drug toxicities, with increased risk of adverse drug reactions. European Summaries of Product Characteristics (SmPCs) and United States Prescribing Information (US PI) should therefore be comprehensible to prescribers regarding their liver-associated contraindications to ensure safe prescribing. This study aimed to evaluate the ambiguity of terminology used in communicating liver-associated absolute contraindications in SmPCs/PI of commonly prescribed drugs in four major drug markets (Germany, Switzerland, the United Kingdom, and the United States) by assigning wordings to different categories and analyzing their clinical comprehensibility. For... (truncated)

Title: Pharmacokinetics of calcium antagonists under development.
Calcium antagonist drugs under clinical development are of the Type I (verapamil, diltiazem-like) and Type II (nifedipine-like) classes. Tiapamil, the only Type I drug currently available, is a high clearance, widely distributed drug which undergoes extensive presystemic elimination. Pharmacokinetically it is quite similar to verapamil; however, it does have increased biliary excretion and decreased binding to plasma proteins. Eight Type II (dihydropyridine) drugs are reviewed. Seven of these drugs (felodipine, isradipine, nicardipine, nilvadipine, nimodipine, nisoldipine and nitrendipine) are pharmacokinetically similar to nifedipine, with high clearance, extensive distribution, and significant presystemic elimination. Amlodipine has lower clearance, even greater peripheral distribution, and... (truncated)

Title: Palmitate increases the susceptibility of cells to drug-induced toxicity: an in vitro method to identify drugs with potential contraindications in patients with metabolic disease.
Fatty acids are an important source of energy. Excessive energy intake results in elevated levels of free fatty acids that are thought to be the pathogenic factors causing metabolic disorders such as dyslipidemia, obesity, insulin resistance, diabetes, and fatty liver. Underlying metabolic disorders have been suggested to be a predisposing factor for drug-induced liver injury. The steadily expanding population with metabolic disease may pose a higher risk for drug-induced toxicity. In order to understand the interaction of free fatty acids and drug-induced toxicity at the cellular level, we explored whether the saturated free fatty acid palmitate could modulate drug-induced cytotoxicity... (truncated)

Title: Novel Score-based Decision Approach in Chronic Myeloid Leukemia Patients After Acute Toxic Imatinib-induced Liver Injury.
The tyrosine kinase inhibitor (TKI) imatinib in rare cases can cause acute toxic hepatitis, hepatic failure, and death. Currently, the choice of further chronic myeloid leukemia (CML) therapy in patients after acute hepatotoxicity is still a difficult question, which requires a complex individual approach based on the clinical guidelines of adverse event management. Data about the further follow-up strategy approach in patients with CML after acute toxic imatinib-induced liver injury are of concern, and at times controversial. In addition, one of the questions is about the necessity and safety of the imatinib therapy resumption after acute hepatotoxicity. In some publications, imatinib...

MESH:D017292 - doxazosin

Summary:

LLM Prediction Score	0.137 (normalized)
LLM Confidence Score	0.980
Golden Answer (Severity Class)	0.375 (normalized)
Prediction Error	0.238

Retrieved Context:

Title: Curcumin and <i>α</i>/<i>β</i>-Adrenergic Antagonists Cotreatment Reverse Liver Cirrhosis in Hamsters: Participation of Nrf-2 and NF-<i>κ</i>B.
Liver cirrhosis is the result of an uncontrolled fibrogenetic process, due to the activation and subsequent differentiation into myofibroblasts of the hepatic stellate cells (HSC). It is known that HSC express adrenoreceptors (AR), and the use of AR antagonists protects experimental animals from cirrhosis. However, several studies suggest that the toxicity generated by metabolism of these antagonists would hinder its use in cirrhotic patients. In addition, liver fibrosis may be associated with a decrease of the antioxidant response of the nuclear factor erythroid 2-related factor 2 (Nrf-2) and the overregulation of the proinflammatory pathway of nuclear factor kappa B (NF-<i>κ</i>B).... (truncated)

Title: Doxazosin Attenuates Liver Fibrosis by Inhibiting Autophagy in Hepatic Stellate Cells via Activation of the PI3K/Akt/mTOR Signaling Pathway.
To investigate the effect of doxazosin on autophagy and the activation of hepatic stellate cells (HSCs) in vivo and in vitro and determine the underlying mechanism.

Title: Neuroimmunomodulation of adrenoblockers during liver cirrhosis: modulation of hepatic stellate cell activity.
The sympathetic nervous system and the immune system are responsible for producing neurotransmitters and cytokines that interact by binding to receptors; due to this, there is communication between these systems. Liver immune cells and nerve fibres are systematically distributed in the liver, and the partial overlap of both patterns may favour interactions between certain elements. Dendritic cells are attached to fibroblasts, and nerve fibres are connected via the dendritic cell-fibroblast complex. Receptors for most neuroactive substances, such as catecholamines, have been discovered on dendritic cells. The sympathetic nervous system regulates hepatic fibrosis through sympathetic fibres and adrenaline from the adrenal... (truncated)

Title: Adenoviral-bone morphogenetic protein-7 and/or doxazosin therapies promote the reversion of fibrosis/cirrhosis in a cirrhotic hamster model.
Liver fibrosis occurs in the presence of continuous insults, including toxic or biological agents. Novel treatments must focus on ceasing the progression of cellular damage, promoting the regeneration of the parenchyma and inhibition of the fibrotic process. The present study analyzed the effect of bone morphogenetic protein (BMP)-7 gene therapy with or without co-treatment with doxazosin in a model of liver cirrhosis in hamsters. The serum alanine aminotransferase, aspartate aminotransferase and albumin levels were analyzed spectrophotometrically. Tissue hepatic samples were analyzed by hematoxylin and eosin for parenchymal structure and Sirius red for collagen fiber content. BMP-7 and α-smooth muscle actin... (truncated)

Title: Acute hepatocellular drug induced liver injury probably by alfuzosin.
Alpha blockers are the drugs that exert their effects by binding to alpha receptors and relaxing smooth muscles and are currently used for treatment of benign prostate hyperplasia (BPH). These drugs are often tolerated well by the patients. However, they also possess some common side effects. Hepatotoxicity, on the other hand, is quite rare. We report herein a case with the rare complication of acute hepatocellular drug induced liver injury (DILI) by administration of Alfuzosin.

MESH:D000077268 - pamidronate

Summary:

LLM Prediction Score	0.139 (normalized)
LLM Confidence Score	0.980
Golden Answer (Severity Class)	0.375 (normalized)
Prediction Error	0.236

Retrieved Context:

Title: Antiresorptive effect of a single infusion of microgram quantities of zoledronate in Paget's disease of bone.
Zoledronate (CGP 42446) is a third generation imidazole ring containing bisphosphonate that has been found in animal studies to be up to 850 times more potent than pamidronate. In this first study reporting the effects of this drug in humans, 16 patients with active Paget's disease of bone [baseline serum alkaline phosphatase activity (SAP) at least twice the upper limit of normal] were treated in a fixed ascending dose-ranging protocol with a single 1-hour infusion of either 24, 72, 216, or 400 microg of zoledronate (four patients per dose). SAP and two markers of bone resorption, 24-hour urinary hydroxyproline/creatinine excretion... (truncated)

Title: Pathologic hepatic Tc-99m-MDP uptake in polyostotic fibrous dysplasia.
Fibrous dysplasia of bone is a congenital, sporadic developmental disorder characterized by immature fibrous connective tissue and bone deformities. Hepatic Tc-99m-MDP uptake is a rare, serendipitous finding during bone scanning studies. The present patient was a 25-year-old male who had severe polyostotic fibrous dysplasia. On Tc-99m-MDP (methylene diphosphonate) bone scintigraphy, increased activity accumulations were seen on multiple ribs, vertebrae and base of the cranium. In addition, diffuse increased pathologic uptake of Tc-99m-MDP in the liver was shown. Intravenous pamidronate was administered monthly for two months. In the third week of the last administration Tc-99m-MDP bone scintigraphy was performed again, but... (truncated)

Title: The Effects of Polyphenol, Tannic Acid, or Tannic Acid in Combination with Pamidronate on Human Osteoblast Cell Line Metabolism.
This study investigates the effect of tannic acid (TA) combined with pamidronate (PAM) on a human osteoblast cell line.

Title: [Zoledronate-associated end stage renal failure and hypocalcaemia].
In an 81-year-old patient with a history of long-standing stable chronic renal failure a diagnosis of multiple myeloma was made. After an initial chemotherapy, a therapy with intravenous pamidronate, 90 mg monthly, was initiated. After four years of well tolerated therapy, pamidronate was stopped and zoledronate, 4 mg intravenously every four weeks, was started. After approximately one year, an elevated plasma creatinine was noted for the'first time, progressing to end stage renal failure within the next months. At admission, besides end-stage renal failure, severe asymptomatic hypocalcemia was noted. Renal biopsy findings included severe tubulointerstitial damage compatible with drug-induced tubular injury.... (truncated)

Title: Hepatic Osteodystrophy: The Mechanism of Bone Loss in Hepatocellular Disease and the Effects of Pamidronate Treatment.
The present study was designed to evaluate the bone phenotypes and mechanisms involved in bone disorders associated with hepatic osteodystrophy. Hepatocellular disease was induced by carbon tetrachloride (CCl4). In addition, the effects of disodium pamidronate on bone tissue were evaluated.

MESH:D003022 - clotrimazole

Summary:

LLM Prediction Score	0.140 (normalized)
LLM Confidence Score	0.990
Golden Answer (Severity Class)	0.375 (normalized)
Prediction Error	0.235

Retrieved Context:

Title: Comparison of efficacy of alternative medicine with allopathy in treatment of oral fungal infection.
This clinical study assessed and compared the efficacy of tea tree oil (TTO), an alternative form of medicine, with clotrimazole (i.e., allopathy) and a conservative form of management in the treatment of oral fungal infection. In this interventional, observational, and comparative study, we enrolled 36 medically fit individuals of both sexes who were aged 20-60 years old. The participants were randomly assigned to three groups. Group I was given TTO (0.25% rinse) as medicament, Group II was given clotrimazole, and Group III was managed with conservative treatment. The results were analyzed from the clinical evaluation of lesions, changes in four... (truncated)

Title: Bioavailability of the imidazole antifungal agent clotrimazole and its effects on key biotransformation genes in the common carp (Cyprinus carpio).
Clotrimazole (CTZ) is a persistent imidazole antifungal agent which is frequently detected in the aquatic environment and predicted to bio-concentrate in fish. Common carp (Cyprinus carpio) were exposed to mean measured concentrations of either 1.02 or 14.63μgl(-1) CTZ for 4 and 10 days, followed by a depuration period of 4 days in a further group of animals. Following each exposure regimen, plasma and liver CTZ concentrations were measured. Mean measured plasma concentrations of CTZ in animals exposed to the lower concentration of CTZ were 30 and 44μgl(-1) on days 4 and 10, respectively, and in the higher concentration were 318... (truncated)

Title: Mechanisms of Hepatic Cholestatic Drug Injury.
Drug-induced cholestasis represents a form of drug-induced liver disease that can lead to severe impairment of liver function. Numerous drugs have been shown to cause cholestasis and consequently bile duct toxicity. However, there is still lack of therapeutic tools that can prevent progression to advanced stages of liver injury. This review focuses on the various pathological mechanisms by which drugs express their hepatotoxic effects, as well as consequences of increased bile acid and toxin accumulation in the hepatocytes.

Title: Review of Recurrent Otomycosis and Clotrimazole in Its Treatment.
Otomycosis is a disease whose acute form affects four in 1,000 persons annually and the chronic form affects 3-5% of the population. It is brought on by various fungi, primarily saprophytes which most commonly include the Candida albicans and Aspergillus niger. The disease rarely poses a life-threatening danger, but as it requires prolonged treatment and follow-up and has a significant chance of recurrence, it has a difficult and taxing course. Numerous therapeutic modalities are available for the treatment of otomycosis. In the beginning, the fungal elements are removed by ear toilet - washing or suctioning of the ear canal followed... (truncated)

Title: Role of posaconazole in the management of oropharyngeal and esophageal candidiasis.
Mucocutaneous candidiasis (MC) is one of the first signs of human immunodeficiency virus (HIV) infection. Over 90% of patients with AIDS will eventually develop oropharyngeal candidiasis (OPC) at some time during their illness, and an additional 10% will develop esophageal candidiasis (EC). Although numerous antifungal agents are available, azoles, both topical (clotrimazole) and systemic (fluconazole, itraconazole), have replaced older topical antifungals (gentian violet and nystatin) in the management of MC in these patients. The systemic azoles, itraconazole and fluconazole, are generally safe and effective agents in HIV-infected patients with MC. A concern in these patients is the clinical relapse, which... (truncated)

MESH:D004005 - dichlorphenamide

Summary:

LLM Prediction Score	0.234 (normalized)
LLM Confidence Score	0.890
Golden Answer (Severity Class)	0.0 (normalized)
Prediction Error	0.234

Retrieved Context:

Title: Febuxostat attenuates aluminum chloride-induced hepatorenal injury in rats with the impact of Nrf2, Crat, Car3, and MNK-mediated apoptosis.
Aluminum (Al) is a ubiquitous xenobiotic with known toxicity for both humans and animals. Our study was conducted to investigate the protective role of febuxostat (Feb) against aluminum chloride (AlCl3)-induced hepatorenal injury in rats. Hepatorenal injury was induced by oral administration of AlCl3 (40 mg/kg b.w.), for 2 months. Twenty-four male Sprague-Dawley rats were randomly allocated into four groups (six rats/group). The first group received the vehicle thought the experiment. The second group was considered as a control positive group. The third and fourth groups received oral treatment of Feb (10 mg/kg.b.w.) and (15 mg/kg.b.w.), respectively with AlCl3, concurrently for 2 months. Twenty-four hours, after... (truncated)

Title: Association of CYP1A1 and CYP1B1 inhibition in in vitro assays with drug-induced liver injury.
Drug-induced liver injury (DILI) is one of the major causes for the discontinuation of drug development and withdrawal of drugs from the market. Since it is known that reactive metabolite formation and being substrates or inhibitors of cytochrome P450s (P450s) are associated with DILI, we systematically investigated the association between human P450 inhibition and DILI. The inhibitory activity of 266 DILI-positive drugs (DILI drugs) and 92 DILI-negative drugs (no-DILI drugs), which were selected from Liver Toxicity Knowledge Base (US Food and Drug Administration), against 8 human P450 forms was assessed using recombinant enzymes and luminescent substrates, and the threshold values... (truncated)

Title: State of the Art and Uses for the Biopharmaceutics Drug Disposition Classification System (BDDCS): New Additions, Revisions, and Citation References.
The Biopharmaceutics Drug Disposition Classification system (BDDCS) is a four-class approach based on water solubility and extent of metabolism/permeability rate. Based on the BDDCS class to which a drug is assigned, it is possible to predict the role of metabolic enzymes and transporters on the drug disposition of a new molecular entity (NME) prior to its administration to animals or humans. Here, we report a total of 1475 drugs and active metabolites to which the BDDCS is applied. Of these, 379 are new entries, and 1096 are revisions of former classification studies with the addition of references for the approved... (truncated)

Title: Evaluation of the reproductive and developmental risks of caffeine.
A risk analysis of in utero caffeine exposure is presented utilizing epidemiological studies and animal studies dealing with congenital malformation, pregnancy loss, and weight reduction. These effects are of interest to teratologists, because animal studies are useful in their evaluation. Many of the epidemiology studies did not evaluate the impact of the "pregnancy signal," which identifies healthy pregnancies and permits investigators to identify subjects with low pregnancy risks. The spontaneous abortion epidemiology studies were inconsistent and the majority did not consider the confounding introduced by not considering the pregnancy signal. The animal studies do not support the concept that caffeine... (truncated)

Title: COVID-19: A review of the proposed pharmacological treatments.
The emerging pandemic of coronavirus disease 2019 (COVID-19) caused by the severe acute respiratory syndrome coronavirus 2 (SARS-CoV-2) presents an unprecedented challenge for healthcare systems globally. The clinical course of COVID-19 and its ability to rapidly create widespread infection has major implications, warranting vigorous infection prevention and control measures. As the confirmed number of cases has surpassed 5.6 million worldwide and continues to grow, the potential severity of the disease and its deadly complications requires urgent development of novel therapeutic agents to both prevent and treat COVID-19. Although vaccines and specific drug therapies have yet to be discovered, ongoing research... (truncated)

MESH:C106783 - naratriptan

Summary:

LLM Prediction Score	0.267 (normalized)
LLM Confidence Score	0.960
Golden Answer (Severity Class)	0.5 (normalized)
Prediction Error	0.233

Retrieved Context:

Title: Hepatobiliary Events in Migraine Therapy with Herbs-The Case of Petadolex, A Petasites Hybridus Extract.
Petadolex®, a defined butterbur extract has clinically proven efficacy against migraine attacks. However, spontaneous reports indicate cases of herbal induced liver injury (HILI). While most HILI patients presented mild serum biochemistry changes (<3 ULN, dose range 50 to 225 mg/day; treatment duration 4-730 days) nine developed severe HILI (average time-to-onset 103 days, ALT-range 3-153; AST 2-104-fold ULN). HILI cases resolved after medication withdrawal though two patients required liver transplantation. Liver biopsies revealed an inconsistent injury pattern, i.e. necrosis, macrovesicular steatosis, inflammation, cholestasis, and bile duct proliferation. Causality assessment rated 3 cases likely, 13 possible, 8 unlikely and 24 as unclassifiable/unclassified.... (truncated)

Title: Sumatriptan (subcutaneous route of administration) for acute migraine attacks in adults.
Migraine is a highly disabling condition for the individual and also has wide-reaching implications for society, healthcare services, and the economy. Sumatriptan is an abortive medication for migraine attacks, belonging to the triptan family. Subcutaneous administration may be preferable to oral for individuals experiencing nausea and/or vomiting

Title: Petasites for Migraine Prevention: New Data on Mode of Action, Pharmacology and Safety. A Narrative Review.
Petasins are the pharmacologically active ingredients of butterbur and of therapeutic benefit in the treatment of migraine and tension headaches. Here, we summarize the pharmacology, safety and clinical efficacy of butterbur in the prevention of migraine attacks and present new data on its mode of action. We review published literature and study reports on the safety and clinical efficacy of the butterbur root extract Petadolex® and report new findings on petasins in dampening nociception by desensitizing calcium-conducting TRP ion channels of primary sensory neurons. Importantly, butterbur diminishes the production of inflammatory mediators by inhibiting activities of cyclooxygenases, lipoxygenases and phospholipase... (truncated)

Title: Data-driven identification of structural alerts for mitigating the risk of drug-induced human liver injuries.
The use of structural alerts to de-prioritize compounds with undesirable features as drug candidates has been gaining in popularity. Hundreds of molecular structural moieties have been proposed as structural alerts. An emerging issue is that strict application of these alerts will result in a significant reduction of the chemistry space for new drug discovery, as more than half of the oral drugs on the market match at least one of the alerts. To mitigate this issue, we propose to apply a rigorous statistical analysis to derive/validate structural alerts before use.

Title: The Effect of Sumatriptan, Theophylline, Pregabalin and Caffeine on Prevention of Headache Caused By Spinal Anaesthesia (PDPH): A Systematic Review.
Spinal anaesthesia (SA) is a common method during surgery due to easy administration, rapid effects, relaxes muscles and controls pain. But, post-dural puncture headache (PDPH) is a common problem after SA that occurs in 6%-36% of SA. We assessed the effect of four common treatment drugs sumatriptan, theophylline, pregabalin and oral caffeine on prevention of PDPH. In this systematic review, all randomized clinical trials (RCTs) during January 2015 and December 2021 were searched from PubMed, Google Scholar, Web of Science, Cochrane review and Clinical Key with a specific search strategy. The article qualities were assessed by two independent authors and... (truncated)

MESH:D002515 - cephradine

Summary:

LLM Prediction Score	0.267 (normalized)
LLM Confidence Score	0.940
Golden Answer (Severity Class)	0.5 (normalized)
Prediction Error	0.233

Retrieved Context:

Title: Promising Antidiabetic and Antimicrobial Agents Based on Fused Pyrimidine Derivatives: Molecular Modeling and Biological Evaluation with Histopathological Effect.
Diabetes is the most common metabolic disorder in both developing and non-developing countries, and a well-recognized global health problem. The WHO anticipates an increase in cases from 171 million in 2000 to 366 million by 2030. In the present study, we focus on the preparation of pyrimidine derivatives as potential antidiabetic and antimicrobial agents. Thein vivoeffect on total serum glucose concentration, cholesterol and antioxidant activity was assessed in adult male albino Wister rats and compared to the reference drug glimperide. Promising results were observed for compound 5. The histopathological study confirms that compound 5 results in significant activity with liver... (truncated)

Title: An evaluation of tolerance of roxithromycin in adults.
This review deals with tolerance of a new macrolide, roxithromycin from data collected from a number of studies in adults. A total of 2917 adults, 2519 given roxithromycin 150 mg bid, were recruited into 17 multicentre comparative or non-comparative studies. Nine studies were double-blind, against doxycycline, erythromycin estolate (EES), lymecycline or cephradine. Overall the drug was well tolerated: side-effects possibly or probably related to roxithromycin were noted in only 4.1% (120/2917) of all patients, and in 3.1% (15/480) of elderly subjects. The gastrointestinal tolerance of roxithromycin was significantly better than that of doxycycline in four trials, and better than that... (truncated)

Title: Antibiotics in Yersinia enterocolitica infections.
A prospective study was undertaken to evaluate the incidence, course, effects of treatment and outcome of patients with Yersinia enterocolitica infections. A total of 189 patients were followed: 62.5% had enteric forms of illness, 20.6% extramesenteric forms, 23.2% arthritis and erythema nodosum. Lymphadenopathy with high fever and weight loss, a septic syndrome and hepatitis were predominant manifestations of the extramesenteric form. Ten per cent of the isolates (135) were susceptible to amoxycillin (4 mg/l), 38% to cephradine (8 mg/l), 82% to doxycycline (4 mg/l), 83% to chloramphenicol (4 mg/l), 85% to trimethoprim (1 mg/l), 87% to cefuroxime (8 mg/l), 92%... (truncated)

Title: Teicoplanin vs cephradine and metronidazole in the prophylaxis of sepsis following vascular surgery: an interim analysis of an ongoing trial.
This paper presents further preliminary results of a trial of the prophylaxis of sepsis in 165 patients undergoing vascular surgery. The efficacy and safety of a single dose of teicoplanin was examined and compared with three doses of cephradine plus metronidazole. No significant differences were detected in the prophylactic efficacy in either group. The first interim report indicated abnormalities in liver function, maximum at 7 days, in both groups. These findings are confirmed in this second interim report. Raised levels of GGT and alkaline phosphatase are more prominent in patients receiving teicoplanin. Liver function improved by 28 days, however, suggesting... (truncated)

Title: A comparison of teicoplanin vs. cephradine and metronidazole in surgical prophylaxis: an interim analysis.
The preliminary results of a trial to examine and compare the safety, tolerability and efficacy of a single dose of teicoplanin with three doses of cephradine combined with metronidazole are presented in a series of 113 patients undergoing elective vascular surgery. There were no obvious differences in the infection rates and sepsis indicators in either group. Neither drug regimen produced any evidence of renal or hepatic damage, though the levels of three hepatocellular enzymes, aspartate aminotransferase (AST), alanine aminotransferase (ALT) and gamma-glutamyl-transferase (GGT), were seen to be transiently elevated, peaking 7 days post-operatively. The trial continues.

MESH:D008774 - methylphenidate

Summary:

LLM Prediction Score	0.608 (normalized)
LLM Confidence Score	0.990
Golden Answer (Severity Class)	0.375 (normalized)
Prediction Error	0.233

Retrieved Context:

Title: Liver Transplant in a Patient under Methylphenidate Therapy: A Case Report and Review of the Literature.
Background. Methylphenidate (MPH) is widely used in treating children with attention-deficit-hyperactivity disorder. Hepatotoxicity is a rare phenomenon; only few cases are described with no liver failure. Case. We report on the case of a 12-year-old boy who received MPH for attention-deficit-hyperactivity disorder. Two months later the patient presented with signs and symptoms of hepatitis and MPH was discontinued, showing progressive worsening and developing liver failure and a liver transplantation was required. Other causes of liver failure were ruled out and the liver biopsy was suggestive of drug toxicity. Discussion. One rare adverse reaction of MPH is hepatotoxicity. The review of... (truncated)

Title: Multiple organ failure resulting from intravenous abuse of methylphenidate hydrochloride.
A 32-year-old woman who abused methylphenidate hydrochloride developed complications including hepatic, renal, pancreatic, pulmonary, and CNS toxicities. These were manifested by abnormal liver function enzymes, poor urine output, hypotension, tachypnea, tachycardia, abnormal blood gases, rising serum BUN and creatinine, and hyperactive deep-tendon reflexes. All were reversible with medication withdrawal and supportive therapy. Toxicities of this medication and their implications are discussed.

Title: Hepato-Protection Effect of Curcumin Against Methylphenidate-Induced Hepatotoxicity: Histological and Biochemical Evidences.
As a psychostimulant agent, methylphenidate (MPH) abuse can cause serious liver damage. Studies have documented the hepatoprotective impacts of curcumin on liver damage. According to this definition, the purpose of this study is to explain the hapatoprotective effects of curcumin against the hepatotoxicity induced by MPH.

Title: Stimulant medication therapy in the treatment of children with attention deficit hyperactivity disorder.
Despite the tremendous research advances that have increased our knowledge regarding the pharmacodynamics, clinical pharmacology, pharmacokinetics, and adverse effects of stimulant medications in the treatment of children with ADHD, our knowledge is yet incomplete. Perhaps the most central unresolved issue concerns our understanding of the pathogenesis, pathophysiology, and diagnosis of ADHD. This review has touched briefly on the controversy and confusion surrounding this issue. Although our understanding of the use of stimulant medications in this disorder is similarly incomplete, a review of the literature does allow certain conclusions to be made that are helpful to the practitioner. 1. Stimulant medications... (truncated)

Title: Methylphenidate has mild hyperglycemic and hypokalemia effects and increases leukocyte and neutrophil counts.
Various psychotropic drugs may affect the hematological and biochemical profiles of plasma and its metabolism. Carbamazepine, the most well-known psychotropic drug, can cause substantial hyponatremia. Methylphenidate, a piperidine derivative structurally related to amphetamines, acts as a central nervous system stimulant. The current study evaluated whether methylphenidate affects hematological and biochemical parameters of patients diagnosed with attention deficit hyperactivity disorder.Patients undergoing treatment for attention deficit hyperactivity disorder at our Adolescent Psychiatric Clinic were enrolled in the study. Blood samples for complete blood count and common biochemical analyses were collected before patients started methylphenidate and after 3 months of continuous treatment.Participants included... (truncated)

MESH:C406545 - pegvisomant

Summary:

LLM Prediction Score	0.608 (normalized)
LLM Confidence Score	0.960
Golden Answer (Severity Class)	0.375 (normalized)
Prediction Error	0.233

Retrieved Context:

Title: Pegvisomant-induced cholestatic hepatitis with jaundice in a patient with Gilbert's syndrome.
We report on a patient with active acromegaly and Gilbert's syndrome who developed severe hepatic dysfunction during pegvisomant (PEGv) monotherapy. She was partially resistant to all previous therapies, including long-acting somatostatin analogs and cabergoline. Five months after starting PEGv therapy, with an already normalized IGF1, she developed cholestatic liver dysfunction with jaundice. Liver or biliary diseases including biliary sludge, cholelithiasis or liver steatosis were excluded. A liver biopsy was in keeping with drug-induced liver injury. The discontinuation of PEGv was followed by full clinical and biochemical recovery in 6 weeks. PEGv therapy was not resumed. Apart from a minimal increase... (truncated)

Title: Pegvisomant-induced liver injury is related to the UGT1A1*28 polymorphism of Gilbert's syndrome.
Pegvisomant (PEG) therapy has been associated with drug-induced liver dysfunction in acromegalic patients. The mechanism of its toxicity remains unknown.

Title: Role of UGT1A1 and ADH gene polymorphisms in pegvisomant-induced liver toxicity in acromegalic patients.
Hepatotoxicity is one of the most serious adverse effects in acromegalic patients treated with pegvisomant (PEG-V). Recent studies have found an association between this adverse event and the UGT1A1 allele 28 polymorphism associated with Gilbert's syndrome.

Title: Pegvisomant-Induced Cholestatic Hepatitis in an Acromegalic Patient with UGT1A1(*)28 Mutation.
Pegvisomant (PEGv) is a growth hormone receptor antagonist approved for the treatment of acromegaly; one of its documented adverse effects is reversible elevation of hepatic enzymes. We report a 39-year-old male acromegalic patient with a pituitary macroadenoma who underwent transsphenoidal surgery. The patient's condition improved but GH and IGF-I levels did not normalize; as a consequence, we first administered dopamine agonists and then somatostatin receptor ligands (SRLs) with poor response. PEGv 15 mg every other day was added to lanreotide 120 mg monthly. The patient developed a severe hepatitis five months after starting the combination therapy. Elevated ferritin, iron, and... (truncated)

Title: Pegvisomant: new preparation. A last resort in acromegaly.
(1) The first-line treatment for acromegaly is transsphenoidal surgery. As an adjunct to surgery, and for patients with inoperable tumours, the first-line drug therapy is a somatostatin analogue (octreotide or lanreotide). (2) Pegvisomant, a growth hormone (GH) receptor antagonist, is licensed for patients who have a poor response to surgery and/or radiation therapy and in whom somatostatin analogue therapy has failed. (3) There are no published comparative trials evaluating pegvisomant as alternative for patients who have already tried a somatostatin analogue. Most of the evaluation data comes from a single randomised, placebo-controlled trial in 112 patients treated for three months.... (truncated)

MESH:D013988 - ticlopidine

Summary:

LLM Prediction Score	0.730 (normalized)
LLM Confidence Score	0.970
Golden Answer (Severity Class)	0.5 (normalized)
Prediction Error	0.230

Retrieved Context:

Title: [A case of ticlopidine induced acute cholestatic hepatitis and pure red cell aplasia].
Ticlopidine inhibits platelet aggregation and provides beneficial secondary prevention of cerebrovascular and coronary artery disease. Frequently reported adverse effects of ticlopidine include diarrhea, nausea, and rash. However, to our knowledge, there are only a few published reports of the simultaneous occurrence of cholestatic hepatitis and pure red cell aplasia. Here we report a patient with simultaneous severe cholestatic hepatitis and pure red cell aplasia associated with ticlopidine. Although these adverse effects are rare, periodic hematological and liver function tests are recommended after starting ticlopidine.

Title: Ticlopidine-induced severe cholestatic hepatitis.
We report a case study of an 86-year-old female patient with severe cholestatic hepatitis who was undergoing treatment with oral ticlopidine 250 mg daily for coronary artery disease. The patient had nausea and vomiting and was jaundiced after taking ticlopidine for 6 weeks. She was admitted to the hospital for further evaluation. Ultrasound and endoscopic retrograde cholangiopancreatography eliminated the presence of biliary obstruction. Results from a liver biopsy showed a histopathologic picture consistent with cholestatic hepatitis. Ticlopidine-induced cholestatic hepatitis has been reported 32 times in the foreign literature. This is the first reported severe cholestatic hepatitis (total bilirubin up to... (truncated)

Title: Ticlopidine-induced cholestatic hepatitis with anti-nuclear antibody in serum.
We describe a case of severe cholestatic hepatitis following administration of ticlopidine. A 57-year-old man without known liver disease developed jaundice approximately 3 weeks after initiation of ticlopidine for secondary prevention of stroke. Hyperbilirubinemia and abnormal liver function test values resolved 5 months after withdrawal of ticlopidine. The diagnosis of ticlopidine-induced cholestasis was made after thorough investigations had excluded other causes of jaundice. He was not retreated with ticlopidine. This case may serve to illustrate the possibility of ticlopidine hepatotoxicity, which has rarely been reported. Furthermore, to the best of our knowledge, ticlopidine-induced cholestatic hepatitis accompanied by autoantibody has not... (truncated)

Title: Agranulocytosis and hepatic toxicity with ticlopidine therapy: a case report.
Ticlopidine is a platelet inhibitor used to prevent thrombosis in patients with cerebrovascular or coronary artery disease. The most common side effects are mild and transitory: diarrhea, dyspepsia, nausea and rashes. More serious, but less frequent, adverse effects are hematological dyscrasia and cholestatic hepatitis. We report a rare case of agranulocytosis associated with hepatic toxicity, probably related to the use of ticlopidine.

Title: [2 cases of acute cholestasis caused by ticlopidine].
We report the case of two patients suffered from cholestatic jaundice occurred 3-4 weeks after starting ticlopidine therapy. In both cases the diagnosis was made by ruling out any other known cause of acute hepatitis or cholestasis. One patient underwent liver biopsy, which showed a typical intralobular cholestatic pattern and a slight lymphocytic infiltration of the portal tracts. The other patient, a 29 year-old woman, was taking ticlopidine as the sole drug, further to an ischemic stroke occurred while she was taking oral contraceptives; she presented a diffuse itchy dermatitis, fever and slight eosinophilia besides cholestasis. In both patients ticlopidine... (truncated)

MESH:D000077405 - irbesartan

Summary:

LLM Prediction Score	0.396 (normalized)
LLM Confidence Score	0.970
Golden Answer (Severity Class)	0.625 (normalized)
Prediction Error	0.229

Retrieved Context:

Title: Cholestatic hepatitis related to use of irbesartan: a case report and a literature review of angiotensin II antagonist-associated hepatotoxicity.
We report a patient who developed cholestatic hepatitis shortly after starting therapy with irbesartan, one of the new, recently marketed angiotensin II antagonists. Serological studies and ultrasonography ruled out viral hepatitis and extrahepatic obstructive jaundice, respectively. A percutaneous liver biopsy showed a portal inflammatory infiltrate with eosinophils and marked cholestatic features in the perivenular area. Irbesartan was discontinued and the patient's jaundice resolved slowly over a period of several weeks, although mild biochemical cholestasis lasted for more than 1 year. There have been seven prior cases of angiotensin II antagonist-induced hepatotoxicity reported in the literature. A class warning for hepatotoxicity... (truncated)

Title: Targeting the proinflammatory cytokines, oxidative stress, apoptosis and TGF-β1/STAT-3 signaling by irbesartan to ameliorate doxorubicin-induced hepatotoxicity.
Doxorubicin (DOX) is an anthracycline antibiotic that is used frequently for treatment of various types of malignancies. Hepatotoxicity is one of the serious complications of DOX. The aim of this study was to explore the effect of different doses of irbesartan on doxorubicin-induced hepatotoxicity in mice. Sixty male BALB/c mice were divided into six equal groups as follows: Control group; DOX group; Irbesartan (Small dose) group; Irbesartan (Large dose) group; DOX + Irbesartan (Small dose) group and DOX + Irbesartan (Large dose) group. Liver weight/body weight ratio, food intake, serum albumin, alanine transaminase (ALT), aspartate transaminase (AST), alkaline phosphatase (ALP)... (truncated)

Title: Irbesartan Ameliorates Lipid Deposition by Enhancing Autophagy via PKC/AMPK/ULK1 Axis in Free Fatty Acid Induced Hepatocytes.
Irbesartan has shown significant therapeutic effects in hypertensive patients with non-alcoholic fatty liver disease (NAFLD). To determine the underlying mechanisms of its action, we established an <i>in vitro</i> model of NAFLD by treating human and mouse hepatocytes with free fatty acids (FFAs) and angiotensin (Ang) II. Irbesartan significantly reversed AngII/FFA-induced lipid deposition and mitochondrial dysfunction by restoring ATP production and the mitochondrial membrane potential (MMP), and decreasing the levels of reactive oxygen species (ROS) and inflammatory markers. In addition, irbesartan also increased the autophagy flux, in terms of increased numbers of autolysosomes and autophagosomes, and the upregulation and mitochondrial localization... (truncated)

Title: Angiotensin-converting-enzyme 2 inhibits liver fibrosis in mice.
The renin-angiotensin system (RAS) plays a major role in liver fibrosis. Recently, a homolog of angiotensin-converting-enzyme 1 (ACE1), termed ACE2, has been identified that appears to be a negative regulator of the RAS by degrading Ang II to Ang(1-7). The aim of this study was to characterize the long-term effects of gene deletion of ACE2 in the liver, to define the role of ACE2 in acute and chronic liver disease, and to characterize the role of Ang(1-7) in hepatic stellate cell (HSC) activation. Ace2 knockout (KO) mice and wild-type (wt) littermates underwent different models of acute and chronic liver injury.... (truncated)

Title: Sprue-Like Enteropathy and Liver Injury: A Rare Emerging Association with Olmesartan.
Olmesartan-induced enteropathy is an underreported phenomenon, first described in 2012. While olmesartan's antihypertensive properties were confirmed early on, its association with a sprue-like enteropathy was subsequently noted. Although this association has been reported with olmesartan, there have been few reports of this association with other angiotensin-receptor blockers. We present a case of a 79-year-old male who presented with diarrhea, weight loss, jaundice, and transaminitis. Further history revealed that he had been taking olmesartan 40 mg daily for hypertension. Workup of his diarrhea and jaundice included duodenal and liver biopsies revealed findings consistent with a sprue-like enteropathy and an autoimmune hepatitis-like... (truncated)

MESH:D002955 - leucovorin

Summary:

LLM Prediction Score	0.228 (normalized)
LLM Confidence Score	0.990
Golden Answer (Severity Class)	0.0 (normalized)
Prediction Error	0.228

Retrieved Context:

Title: Substitution of l-leucovorin for d,l-leucovorin in the rescue from high-dose methotrexate treatment in patients with osteosarcoma.
Studies in which high-dose methotrexate (HDMTX) is used for the treatment of osteosarcoma have utilized commercial formulations of d,l-leucovorin (leucovorin calcium) for rescue from potential methotrexate (MTX) toxicity. These formulations are racemic mixtures containing equal amounts of d and l isomers of leucovorin. All of the available data indicate that the l isomer is the pharmacologically active diastereomer. A clinical study was conducted to determine if l-leucovorin was as safe and efficacious as d,l-leucovorin in the rescue of patients with osteosarcoma who were treated with HDMTX (12.5 g/m2 over 6 h). Because d,l-leucovorin consists of equal proportions of d and... (truncated)

Title: The use of folates concomitantly with low-dose pulse methotrexate.
Toxicities related to low-dose weekly methotrexate are largely due to its antifolate properties. Preexisting folate deficiency is associated with methotrexate toxicity in some patients. At the onset of methotrexate therapy and throughout therapy, the physician should be vigilant regarding one or more nutrient deficiencies. A multivitamin and, where appropriate, specific daily folic acid supplements should be employed. The only regimen known presently (through controlled trials) to treat side effects is the low-dose folinic acid (leucovorin) protocol outlined herein. Folic acid may be helpful to treat mild gastrointestinal symptoms. Folinic acid supplementation should be considered prophylactically in those requiring methotrexate who... (truncated)

Title: The Clinical Spectrum and Diagnosis of Oxaliplatin Liver Injury in the Era of Nonalcoholic Fatty Liver Disease.
Oxaliplatin is an alkylating agent given with fluorouracil and leucovorin as a mainstay adjuvant chemotherapy for stage III colorectal cancer (CRC). Liver injury from oxaliplatin ranges from mild liver enzyme increases in 42% to 57% of patients in clinical trials¹ to rare severe injury leading to acute liver failure.² Chronic injury from endothelial cell damage and architectural distortion may manifest years later with nodular regenerative hyperplasia (NRH), portal sclerosis, and noncirrhotic portal hypertension (NCPH).²[,]³ Chronic subclinical injury occurs in up to 78% of patients.³ Diagnosis may be confounded by nonalcoholic fatty liver disease (NAFLD), and long-term outcomes from chronic injury... (truncated)

Title: Methotrexate in psoriasis with and without leucovorin: effect of different dosage schedules on acute liver toxicity.
Studies on thirty-six psoriatics revealed no differences in acute liver toxicity of four different intermittent dosage schedules of methotrexate with or without addition of leucovorin, as judged by daily determinations of SGOT for one week. Three patients with psoriatic erythroderma receiving high-dosage methotrexate (100 mg i.v.) with leucovorin rescue responded extremely well to treatment and did not distinguish themselves from the other patients with regard to acute liver toxicity.

Title: Toxicity during l-LV/5FU adjuvant chemotherapy as a modified RPMI regimen for patients with colorectal cancer.
l-leucovorin (LV)/5-fluorouracil (5FU) may play an important role, as an adjuvant chemotherapy, in improving the survival of patients with stage III colorectal cancer. However, severe toxicity of the chemotherapeutic agent could be fatal. Adverse effects, including bone marrow suppression, liver damage, renal damage, and glucose tolerance, were evaluated daily during 3 courses of l-LV/5FU-modified RPMI regimen adjuvant chemotherapy for 22 patients with stage III colorectal cancer. Decrease in the serum levels of neutrophils and platelets occurred in the 1st course, which became more obvious after three or four administrations of l-LV/5FU in the 1st course. Furthermore, serum levels of leukocytes,... (truncated)

MESH:C541220 - canakinumab

Summary:

LLM Prediction Score	0.148 (normalized)
LLM Confidence Score	0.940
Golden Answer (Severity Class)	0.375 (normalized)
Prediction Error	0.227

Retrieved Context:

Title: Systematic review of immunomodulatory therapies for hidradenitis suppurativa.
Background: Greater understanding of the roles of tumor necrosis factor-α, IL-1β, IL-10, and the IL-23/T-helper (Th) 17 and IL-12/Th1 pathways in immune dysregulation in moderate/severe hidradenitis suppurativa (HS) has helped in developing new regimens. We aim to review the use of different immunomodulatory therapies used to manage HS. Methods: A comprehensive literature search was conducted on the PubMed and Clinicaltrials.gov databases from 1 January 1947 to 31 December 2018. Only clinical trials, case reports, case series and retrospective analyses published in the English language were included. Results: Our search yielded 107 articles and 35 clinical trials, of which 15 are... (truncated)

Title: IL-1 Signal Inhibition In Alcoholic Hepatitis (ISAIAH): a study protocol for a multicentre, randomised, placebo-controlled trial to explore the potential benefits of canakinumab in the treatment of alcoholic hepatitis.
Alcohol consumption causes a spectrum of liver abnormalities and leads to over 3 million deaths per year. Alcoholic hepatitis (AH) is a florid presentation of alcoholic liver disease characterized by liver failure in the context of recent and heavy alcohol consumption. The aim of this study is to explore the potential benefits of the IL-1β antibody, canakinumab, in the treatment of AH.

Title: Development and Role in Therapy of Canakinumab in Adult-Onset Still's Disease.
Adult-onset Still's disease (AOSD) is a rare inflammatory disease of unknown etiology typically characterized by episodes of spiking fever, evanescent rash, arthralgia, leukocytosis, and hyperferritinemia. The pivotal role of interleukin (IL)-1 and other pro-inflammatory cytokines gives rise to the development of new targeted therapies. Currently, AOSD patients can benefit from efficient and well tolerated biologic agents, such as IL-1, IL-6, and tumour necrosis factor (TNF)-α antagonists. Canakinumab, a human monoclonal anti-IL-1β antibody, is indicated for the treatment of different autoinflammatory syndromes in adults, adolescents, and children and it has recently been approved for AOSD treatment. In this article, we summarize... (truncated)

Title: Improvement of Liver Involvement in Familial Mediterranean Fever After the Introduction of Canakinumab: A Case Report.
Hepatic involvement in familial Mediterranean fever (FMF) ranges from a nonspecific increase in liver enzymes to cryptogenic cirrhosis, and the liver is mostly involved in patients bearing the M694V <i>MEFV</i> mutation in homozygosis. A 44-year-old Jewish woman with FMF developed nonalcoholic steatohepatitis during colchicine treatment (2,5 mg per day), confirmed by both elastography and liver biopsy. Therefore, combined therapy with the interleukin-1 (IL-1) blocking agent canakinumab (150 mg every four weeks) and colchicine (at a reduced dose of 1.5 mg per day) was started. Three months later, transaminases became normal, and after further six months, there was a marked improvement... (truncated)

Title: Therapeutic Perspectives of IL1 Family Members in Liver Diseases: An Update.
Interleukin (IL) 1 superfamily members are a cornerstone of a variety of inflammatory processes occurring in various organs including the liver. Progression of acute and chronic liver diseases regardless of etiology depends on the stage of hepatocyte damage, the release of inflammatory cytokines and disturbances in gut microbiota. IL1 cytokines and receptors can have pro- or anti-inflammatory roles, even dual functionalities conditioned by the microenvironment. Developing novel therapeutic strategies to block the IL1/IL1R signaling pathways seems like a reasonable option. This mode of action is now exploited by anakinra and canakinumab, which are used to treat different inflammatory illnesses, and... (truncated)

MESH:C068373 - eprosartan

Summary:

LLM Prediction Score	0.149 (normalized)
LLM Confidence Score	0.930
Golden Answer (Severity Class)	0.375 (normalized)
Prediction Error	0.226

Retrieved Context:

Title: Protective and therapeutic role of mango pulp and eprosartan drug and their anti-synergistic effects against thioacetamide-induced hepatotoxicity in male rats.
The present study was done to evaluate the protective and therapeutic role of mango pulp (M), eprosartan drug (E), and their co-administration (EM) against hepatotoxicity induced by thioacetamide (T). Seven groups of rats were prepared as follows: the control (C) group (normal rats), T group (the rats were injected with T), T-M group (the rats were injected with T, and then treated with M), T-E group (the rats were injected with T, and then treated with E), T-EM group (the rats were injected with T, and then treated with E and M), M-TM-M group (the rats were administered with M... (truncated)

Title: Drug-induced liver injury after switching from tamoxifen to anastrozole in a patient with a history of breast cancer being treated for hypertension and diabetes.
Anastrozole is a selective non-steroidal aromatase inhibitor that blocks the conversion of androgens to estrogens in peripheral tissues. It is used as adjuvant therapy for early-stage hormone-sensitive breast cancer in postmenopausal women. Significant side effects of anastrozole include osteoporosis and increased levels of cholesterol. To date, seven case reports on anastrozole hepatotoxicity have been published. We report the case of an 81-year-old woman with a history of breast cancer, arterial hypertension, type 2 diabetes mellitus, hyperlipidemia, and chronic renal insufficiency. Four days after switching hormone therapy from tamoxifen to anastrozole, icterus developed along with a significant increase in liver enzymes... (truncated)

Title: Effect of hepatic disease on the pharmacokinetics and plasma protein binding of eprosartan.
To evaluate the pharmacokinetics and plasma protein binding of eprosartan in hepatic disease.

Title: Angiotensin II type 1 receptor blockers increase tolerance of cells to copper and cisplatin.
The human pathology Wilson disease (WD) is characterized by toxic copper (Cu) accumulation in brain and liver, resulting in, among other indications, mitochondrial dysfunction and apoptosis of hepatocytes. In an effort to identify novel compounds that can alleviate Cu-induced toxicity, we screened the Pharmakon 1600 repositioning library using a Cu-toxicity yeast screen. We identified 2 members of the drug class of Angiotensin II Type 1 receptor blockers (ARBs) that could increase yeast tolerance to Cu, namely Candesartan and Losartan. Subsequently, we show that specific ARBs can increase yeast tolerance to Cu and/or the chemotherapeutic agent cisplatin (Cp). The latter also... (truncated)

Title: Hepatotoxicity Associated with a Short Course of Rosuvastatin.
No abstract available.

MESH:D000077123 - rocuronium

Summary:

LLM Prediction Score	0.226 (normalized)
LLM Confidence Score	0.990
Golden Answer (Severity Class)	0.0 (normalized)
Prediction Error	0.226

Retrieved Context:

Title: Influence of obstructive jaundice on pharmacodynamics of rocuronium.
Anesthetics are variable in patients with obstructive jaundice. The minimum alveolar concentration awake of desflurane is reduced in patients with obstructive jaundice, while it has no effect on pharmacodynamics and pharmacokinetics of propofol. In this study, we investigated the influence of obstructive jaundice on the pharmacodynamics and blood concentration of rocuronium.

Title: [A Case of Suspected Delayed Postoperative Malignant Hyperthermia].
Malignant hyperthermia occurred 10 hours after surgery in a 72-year-old man who had received emergency laparoscopic cholecystectomy for severe acute cholecystitis with cholelethiasis. He had a high fever (39.4 degrees C) with liver damage before surgery. Anesthesia was induced with propofol and fentanyl and maintained with sevoflurane and epidural block using ropivacaine. Rocuronium was used as a muscle relaxant During surgery, body temperature decreased by cooling the body surface, but tachycardia continued. Ten hours after surgery, body temperature increased to the maximum of 40.6 degrees C and he went into shock. Then another 10 hours later, he developed cardiac arrest... (truncated)

Title: Increased Renal Clearance of Rocuronium Compensates for Chronic Loss of Bile Excretion, via upregulation of Oatp2.
Requirement for rocuronium upon surgery changes only minimally in patients with end-stage liver diseases. Our study consisted of both human and rat studies to explore the reason. The reduction rate of rocuronium infusion required to maintain neuromuscular blockade during the anhepatic phase (relative to paleohepatic phase) was examined in 16 children with congenital biliary atresia receiving orthotopic liver transplantation. Pharmacodynamics and pharmacokinetics of rocuronium were studied based on BDL rats. The role of increased Oatp2 and decrease Oatp1 expressions in renal compensation were explored. The reduction of rocuronium requirements significantly decreased in obstructively jaundiced children (24 ± 9 vs. 39... (truncated)

Title: Rocuronium bromide and organ function.
Neuromuscular blocking drugs can be divided into those that are: (i) excreted entirely by the kidney; (ii) predominantly by the kidney but also by the liver; (iii) mainly by the liver but also by the kidney or; (iv) removed by other metabolic pathways. Rocuronium is mainly excreted by the liver and pharmacokinetic and pharmacodynamic studies in humans suggest that its duration of action may be prolonged to a greater extent in patients with hepatic disease than in patients with renal disease. The effect is likely to be modest and not a contra-indication to its use.

Title: Recent advances in neuromuscular blocking agents.
Factors driving the development of neuromuscular blocking agents are discussed. The goal of recent development of neuromuscular blocking agents is to develop agents with fewer adverse effects than succinylcholine and greater control. Greater control can be achieved through a short duration of action and a fast onset, similar to that found with succinylcholine. Duration control can be achieved through rapid, reliable metabolism that is organ independent, as with cisatracurium, or that occurs in the liver, because this will fail only in patients with severe hepatic disease. Rapid onset can be achieved by giving higher doses of drugs that have a... (truncated)

MESH:C093622 - rizatriptan

Summary:

LLM Prediction Score	0.226 (normalized)
LLM Confidence Score	0.980
Golden Answer (Severity Class)	0.0 (normalized)
Prediction Error	0.226

Retrieved Context:

Title: Hepatobiliary Events in Migraine Therapy with Herbs-The Case of Petadolex, A Petasites Hybridus Extract.
Petadolex®, a defined butterbur extract has clinically proven efficacy against migraine attacks. However, spontaneous reports indicate cases of herbal induced liver injury (HILI). While most HILI patients presented mild serum biochemistry changes (<3 ULN, dose range 50 to 225 mg/day; treatment duration 4-730 days) nine developed severe HILI (average time-to-onset 103 days, ALT-range 3-153; AST 2-104-fold ULN). HILI cases resolved after medication withdrawal though two patients required liver transplantation. Liver biopsies revealed an inconsistent injury pattern, i.e. necrosis, macrovesicular steatosis, inflammation, cholestasis, and bile duct proliferation. Causality assessment rated 3 cases likely, 13 possible, 8 unlikely and 24 as unclassifiable/unclassified.... (truncated)

Title: Sumatriptan (intranasal route of administration) for acute migraine attacks in adults.
Migraine is a highly disabling condition for the individual and also has wide-reaching implications for society, healthcare services, and the economy. Sumatriptan is an abortive medication for migraine attacks, belonging to the triptan family. Intranasal administration may be preferable to oral for individuals experiencing nausea and/or vomiting, although it is primarily absorbed in the gut, not the nasal mucosa.

Title: Targeted CGRP Small Molecule Antagonists for Acute Migraine Therapy.
Migraine is a highly prevalent, severe, and disabling neurological condition with a significant unmet need for effective acute therapies. Patients (~50%) are dissatisfied with their currently available therapies. Calcitonin gene-related peptide (CGRP) has emerged as a key neuropeptide involved in the pathophysiology of migraines. As reviewed in this manuscript, a number of small molecule antagonists of the CGRP receptor have been developed for migraine therapy. Incredibly, the majority of the clinical trials conducted have proven positive, demonstrating the importance of this signalling pathway in migraine. Unfortunately, a number of these molecules raised liver toxicity concerns when used daily for as... (truncated)

Title: Molecular Idiosyncratic Toxicology of Drugs in the Human Liver Compared with Animals: Basic Considerations.
Drug induced liver injury (DILI) occurs in patients exposed to drugs at recommended doses that leads to idiosyncratic DILI and provides an excellent human model with well described clinical features, liver injury pattern, and diagnostic criteria, based on patients assessed for causality using RUCAM (Roussel Uclaf Causality Assessment Method) as original method of 1993 or its update of 2016. Overall, 81,856 RUCAM based DILI cases have been published until mid of 2020, allowing now for an analysis of mechanistic issues of the disease. From selected DILI cases with verified diagnosis by using RUCAM, direct evidence was provided for the involvement... (truncated)

Title: Efficacy of frovatriptan as compared to other triptans in migraine with aura.
The treatment of migraine attacks with aura by triptans is difficult since triptans most probably are not efficacious when taken during the aura phase. Moreover, there are insufficient data from randomised studies whether triptans are efficacious in migraine attacks with aura when taken during the headache phase. In this metaanalysis, we aimed to compare the efficacy of frovatriptan versus rizatriptan, zolmitriptan, and almotriptan.

MESH:C059896 - miglustat

Summary:

LLM Prediction Score	0.226 (normalized)
LLM Confidence Score	0.970
Golden Answer (Severity Class)	0.0 (normalized)
Prediction Error	0.226

Retrieved Context:

Title: Iminosugar Glucosidase Inhibitors Reduce Hepatic Inflammation in Hepatitis A Virus-Infected <i>Ifnar1^{-/-}</i> Mice.
Iminosugar compounds are monosaccharide mimetics with broad but generally weak antiviral activities related to inhibition of enzymes involved in glycobiology. Miglustat (<i>N</i>-butyl-1-deoxynojirimycin), which is approved for the treatment of lipid storage diseases in humans, and UV-4 [<i>N</i>-(9-methoxynonyl)-1-deoxynojirimycin] inhibit the replication of hepatitis A virus (HAV) in cell culture (50% inhibitory concentrations [IC₅₀s] of 32.13 μM and 8.05 μM, respectively) by blocking the synthesis of gangliosides essential for HAV cell entry. We used a murine model of hepatitis A and targeted mass spectrometry to assess the capacity of these compounds to deplete hepatic gangliosides and modify the course of HAV infection <i>in vivo</i>.... (truncated)

Title: Evaluation of Two Liver Treatment Strategies in a Mouse Model of Niemann-Pick-Disease Type C1.
Niemann-Pick-disease type C1 (NPC1) is an autosomal-recessive cholesterol-storage disorder. Besides other symptoms, NPC1 patients develop liver dysfunction and hepatosplenomegaly. The mechanisms of hepatomegaly and alterations of lipid metabolism-related genes in NPC1 disease are still poorly understood. Here, we used an NPC1 mouse model to study an additive hepatoprotective effect of a combination of 2-hydroxypropyl-β-cyclodextrin (HPβCD), miglustat and allopregnanolone (combination therapy) with the previously established monotherapy using HPβCD. We examined transgene effects as well as treatment effects on liver morphology and hepatic lipid metabolism, focusing on hepatic cholesterol transporter genes. Livers of <i>Npc1</i>^{-/-} mice showed hepatic cholesterol sequestration with consecutive liver... (truncated)

Title: Differential Effects of 2-Hydroxypropyl-Cyclodextrins on Lipid Accumulation in <i>Npc1</i>-Null Cells.
Niemann-Pick disease type C (NPC) is an autosomal recessive disorder characterized by abnormal accumulation of free cholesterol and sphingolipids in lysosomes. The iminosugar miglustat, which inhibits hexosylceramide synthesis, is used for NPC treatment, and 2-hydroxypropyl-β-cyclodextrin (HP-β-CD), a cyclic oligosaccharide derivative, is being developed to treat NPC. Moreover, therapeutic potential of 2-hydroxypropyl-γ-cyclodextrin (HP-γ-CD) was shown in NPC models, although its mechanism of action remains unclear. Here, we investigated the effects of HP-β-CD, HP-γ-CD, and their homolog 2-hydroxypropyl-α-cyclodextrin (HP-α-CD) on lipid accumulation in <i>Npc1</i>-null Chinese hamster ovary (CHO) cells compared with those of miglustat. HP-β-CD and HP-γ-CD, unlike HP-α-CD, reduced intracellular free... (truncated)

Title: Favorable outcomes following early onset oral miglustat in early infantile Niemann Pick Type C.
Niemann-Pick disease Type C (NPC) is a rare autosomal recessive neurovisceral lysosomal disorder. Perinatal and early infantile onset NPC are the most severe types of the disease. Early infantile type is characterized by a rapidly progressive neurodegenerative course, which entails significant morbidity and usually results in death within 5 years. Miglustat, an iminosugar that selectively inhibits the glycosylceramide synthase enzyme, is known to stabilize or delay neurological progression in individuals with NPC, but its impact on affected infants is yet to be elucidated. We present two siblings with early infantile NPC due to the previously reported devastating homozygous mutation c.2279_2281delTCT in... (truncated)

Title: Eye movement impairment recovery in a Gaucher patient treated with miglustat.
In Gaucher Disease (GD) the enzyme (imiglucerase) replacement therapy (ERT) is not able to stop the progression of the neurological involvement, while the substrate reduction therapy (SRT), performed by N-Butyldeoxynojirimycin (miglustat), is an alternative that should be evaluated. Two sisters, presenting the same genotype (R353G/R353G), were diagnosed as suffering from GD; one of them later developed neurological alterations identified by quantitative saccadic eye movements analysis. The aim of the study was to quantitatively measure the miglustat effects in this GD neurological patient. Eye movement analysis during subsequent controls was performed by estimating the characteristic parameters of saccadic main sequence. The... (truncated)

MESH:D000077403 - orlistat

Summary:

LLM Prediction Score	0.776 (normalized)
LLM Confidence Score	0.990
Golden Answer (Severity Class)	1.0 (normalized)
Prediction Error	0.224

Retrieved Context:

Title: Orlistat-induced fulminant hepatic failure.
Orlistat was approved by the Food and Drug Administration in 1998 and has been shown to be superior to placebo in achieving weight loss. It is generally well tolerated. However, severe liver injury has been reported. We present a case of hepatic failure in a patient taking orlistat. A 54-year-old African-American woman with hypertension presented with hepatic failure. She had noticed increasing fatigue, jaundice and confusion. She used alcohol sparingly and denied tobacco or illicit drug use, but had been taking over-the-counter orlistat for the past two months. Physical examination revealed scleral icterus, jaundice, asterixis and slow speech. Laboratory testing... (truncated)

Title: [A case of acute cholestatic hepatitis associated with Orlistat].
Orlistat(Xenical(R), Roche) is considered a safe and effective drug to treat obesity by reduced absorption of 30% digested fat. To date, no serious adverse effects affecting the liver have been published except a case of subacute hepatic failure leading to liver transplantation in a young women with moderate obesity treated with orlistat. We report a case of acute cholestatic hepatitis in a young woman with moderate obesity treated with orlistat: a 33-year-old female admitted for the evaluation of jaundice. Abdominal ultrasonography, ERCP, routine chemistry, viral markers, and a fine needle biopsy of liver were performed. Microscopic findings of the liver... (truncated)

Title: Carboxylesterase-2 is a highly sensitive target of the antiobesity agent orlistat with profound implications in the activation of anticancer prodrugs.
Orlistat has been the most used anti-obesity drug and the mechanism of its action is to reduce lipid absorption by inhibiting gastrointestinal lipases. These enzymes, like carboxylesterases (CESs), structurally belong to the α/β hydrolase fold superfamily. Lipases and CESs are functionally related as well. Some CESs (e.g., human CES1) have been shown to hydrolyze lipids. This study was designed to test the hypothesis that orlistat inhibits CESs with higher potency toward CES1 than CES2, a carboxylesterase with little lipase activity. Liver microsomes and recombinant CESs were tested for the inhibition of the hydrolysis of standard substrates and the anticancer prodrugs... (truncated)

Title: Treatment of nonalcoholic steatohepatitis in adults: present and future.
Nonalcoholic steatohepatitis has become one of the most common liver-related health problems. This condition has been linked to an unhealthy diet and weight gain, but it can also be observed in nonobese people. The standard of care is represented by the lifestyle intervention. However, because this approach has several limitations, such as a lack of compliance, the use of many drugs has been proposed. The first-line pharmacological choices are vitamin E and pioglitazone, both showing a positive effect on transaminases, fat accumulation, and inflammation. Nevertheless, vitamin E has no proven effect on fibrosis and on long-term morbidity and mortality and... (truncated)

Title: Mechanistically acting anti-obesity compositions/formulations of natural origin: a patent review (2010-2021).
Current health trends indicate that the rate of incidence of obesity has risen considerably. According to the World Health Organization (WHO) report 2017, the issue of obesity has grown to an epidemic proportion, with over 4 million people dying every year. Orlistat, a potent pancreatic lipase (PL) inhibitor for long-term treatment of obesity has been recently reported to cause hepatic and renal toxicities. Hence, there is a need to develop newer, safer and efficacious therapeutics that targets obesity and its associated disorders.

MESH:D015296 - ceftizoxime

Summary:

LLM Prediction Score	0.276 (normalized)
LLM Confidence Score	0.950
Golden Answer (Severity Class)	0.5 (normalized)
Prediction Error	0.224

Retrieved Context:

Title: Comparative toxicities of third-generation cephalosporins.
Data on the adverse effects experienced by 2,539 patients who received ceftazidime were compared with adverse effects reported with cefoperazone, cefotaxime, ceftizoxime, and moxalactam. There were 216 such reactions among the ceftazidime-treated patients; 158 patients (6.2 percent) had reactions that were possibly or probably drug-related. The clinical and laboratory safety profile of ceftazidime in regard to renal, hepatic, hematopoietic, and hemostatic parameters compared favorably with that of other third-generation cephalosporins. An increased serum creatinine level was observed in 0.8 percent of ceftazidime-treated patients, an increased blood urea nitrogen level in 1.6 percent, hepatic abnormalities in approximately 6 percent, diarrhea in... (truncated)

Title: [Therapeutic effect of ceftizoxime on severe infectious complications in blood disorders. Tohkai Research Group on Infections in Hematopoietic Disorders].
Ceftizoxime (CZX) was given by intravenous injection in daily doses of 2-8 g to 103 patients with severe infections complicating hematopoietic disorders. The clinical effect was evaluated in 95 of the 103 patients. The causative organisms were identified in 22 patients but were unknown in the remaining 73. Infected sites were the respiratory tract, urinary tract, soft tissue, and blood. The overall effectiveness rate (inclusive of marked and moderate) was 61.1% (58/95). The effectiveness rate was 63.6% (14/22) in patients in whom the causative organisms were identified and 60.3% (44/73) in patients in whom the causative organisms could not be... (truncated)

Title: Hypersensitivity Pneumonitis Caused by Cephalosporins With Identical R1 Side Chains.
Drug-induced hypersensitivity pneumonitis results from interactions between pharmacologic agents and the human immune system. We describe a 54-year-old man with hypersensitivity pneumonitis caused by cephalosporins with identical R1 side chains. The patient, who complained of cough with sputum, was prescribed ceftriaxone and clarithromycin at a local clinic. The following day, he complained of dyspnea, and chest X-ray revealed worsening of inflammation. Upon admission to our hospital, antibiotics were changed to cefepime with levofloxacin, but his pneumonia appeared to progress. Changing antibiotics to meropenem with ciprofloxacin improved his symptoms and radiologic findings. Antibiotics were de-escalated to ceftazidime with levofloxacin, and his... (truncated)

Title: Clinical pharmacokinetics of the third generation cephalosporins.
At the present time, the third generation cephalosporins that are already on the market or close to this point include cefsulodin, cefotaxime, cefoperazone, latamoxef, ceftriaxone, ceftazidime, ceftizoxime and cefotetan. Other newer compounds are also under development but have not been included in this review. None of the third generation compounds is suitable for oral administration and, accordingly, their pharmacokinetics have been studied only after intravenous and intramuscular administration. Microbiological assays and HPLC methods have been used for the measurement of plasma/serum, urine, bile and cerebrospinal fluid (CSF) concentrations. As found with cefotaxime, microbiological assays should only be used when the... (truncated)

Title: Potential of a polyherbal drug to prevent antimicrobial resistance in bacteria to antibiotics.
Persistence of antibacterial drugs for prolonged period in milk increases the probability of antimicrobial resistance progress. Ceftizoxime was found to be excreted in milk for a prolonged period in goats, cows and buffaloes following intravenous injection of ceftriaxone and ceftizoxime. A single dose of ceftriaxone was administered intravenously in healthy control goats (group I) and a single oral dose of the commercial mammary protective polyherbal drug (1.9 gm) was given one hour prior to intravenous ceftriaxone injection in healthy (group II) and induced mastitic (group III) goats to evaluate milk disposition of ceftizoxime following single intravenous dosing of ceftriaxone at 42.25 mg kg⁻¹.Ceftriaxone/ceftizoxime... (truncated)

MESH:D004656 - enalapril

Summary:

LLM Prediction Score	0.654 (normalized)
LLM Confidence Score	0.990
Golden Answer (Severity Class)	0.875 (normalized)
Prediction Error	0.221

Retrieved Context:

Title: [Enalapril (Reniten)-associated toxic hepatitis].
A 52-year-old female was hospitalized with malaise, pruritus, jaundice, abdominal discomfort and vomiting. For 20 weeks she had been taking enalapril (Reniten) for hypertension. Serum aminotransferases and bilirubin were highly elevated with prolonged thromboplastin time. There was no evidence for extrahepatic cholestasis in ultrasonography. Serological investigations for a viral etiology of the liver failure were negative and the patient had no risk factors for viral hepatitis or exposure to hepatotoxic substances. Liver puncture revealed hepatitis of the fulminant viral hepatitis type, a picture that can be seen in a drug-induced hepatitis. The complete recovery of liver function after cessation of... (truncated)

Title: Acute liver failure due to enalapril.
This report presents a 46-year-old man who was treated for hypertension with the angiotensin-converting-enzyme (ACE) inhibitor enalapril. After 3 years of continuous treatment he presented with jaundice and progressive liver failure that continued despite withdrawal of the medication. The patient was taking no other medication. All known causes of acute liver failure could be excluded indicating a drug-induced liver damage after long-term treatment with enalapril. Analysis of liver biopsies revealed a pathomorphological pattern comparable to than observed in severe halothane hepatitis. Serological studies including T-cell stimulation with enalapril and a broad spectrum of tests for autoimmunity including autoantibodies against calreticulin,... (truncated)

Title: Disulfiram-induced hepatitis: case report and review of the literature.
A case of hepatitis is reported in a 38-year-old alcoholic woman taking disulfiram to aid in maintaining sobriety. She presented with anorexia, vomiting, fatigue, right upper-quadrant pain, pruritus, darkened urine, and jaundice after about two weeks of disulfiram 500 mg/d. The patient also had been taking enalapril 10 mg/d for one year. Hepatocellular enzymes, total bilirubin, and eosinophils were significantly elevated. Hepatitis B core antibody, hepatitis A antibody, hepatitis B surface antibody, and antinuclear antibody were negative. After discontinuation of disulfiram, the clinical and biochemical manifestations of hepatitis began to resolve and the patient was discharged in a much improved... (truncated)

Title: Establishment of a mouse model of enalapril-induced liver injury and investigation of the pathogenesis.
Drug-induced liver injury (DILI) is a major concern in drug development and clinical drug therapy. Since the underlying mechanisms of DILI have not been fully understood in most cases, elucidation of the hepatotoxic mechanisms of drugs is expected. Although enalapril (ELP), an angiotensin-converting enzyme inhibitor, has been reported to cause liver injuries with a low incidence in humans, the precise mechanisms by which ELP causes liver injury remains unknown. In this study, we established a mouse model of ELP-induced liver injury and analyzed the mechanisms of its hepatotoxicity. Mice that were administered ELP alone did not develop liver injury, and... (truncated)

Title: Enalapril hepatotoxicity in the rat. Effects of modulators of cytochrome P450 and glutathione.
The effects of modulators of cytochrome P450 and reduced glutathione (GSH) on the hepatotoxicity of enalapril maleate (EN) were investigated in Fischer 344 rats. Twenty-four hours following the administration of EN (1.5 to 1.8 g/kg), increased serum transaminases (ALT and AST) and hepatic necrosis were observed. Pretreatment of the animals with pregnenolone-16 alpha-carbonitrile, a selective inducer of the cytochrome P450IIIA gene subfamily, enhanced EN-induced hepatotoxicity, whereas pretreatment with the cytochrome P450 inhibitor, cobalt protoporphyrin, reduced the liver injury. Depletion of hepatic non-protein sulfhydryls (NPSHs), an indicator of GSH, by combined treatment with buthionine sulfoximine (BSO) and diethyl maleate (DEM) produced... (truncated)

MESH:D000077715 - nateglinide

Summary:

LLM Prediction Score	0.155 (normalized)
LLM Confidence Score	0.900
Golden Answer (Severity Class)	0.375 (normalized)
Prediction Error	0.220

Retrieved Context:

Title: Drug interaction study of flavonoids toward OATP1B1 and their 3D structure activity relationship analysis for predicting hepatoprotective effects.
Organic anion transporting polypeptide 1B1 (OATP1B1), a liver-specific uptake transporter, was associated with drug induced liver injury (DILI). Screening and identifying potent OATP1B1 inhibitors with little toxicity is of great value in reducing OATP1B1-mediated DILI. Flavonoids are a group of polyphenols ubiquitously present in vegetables, fruits and herbal products, some of them were reported to produce transporter-mediated DDI. Our objective was to investigate potential inhibitors of OATP1B1 from 99 flavonoids, and to assess the hepatoprotective effects on bosentan induced liver injury. Eight flavonoids, including biochanin A, hispidulin, isoliquiritigenin, isosinensetin, kaempferol, licochalcone A, luteolin and sinensetin exhibited significant inhibition (>50 %)... (truncated)

Title: DDIT4 S-Nitrosylation Aids p38-MAPK Signaling Complex Assembly to Promote Hepatic Reactive Oxygen Species Production.
Mitogen-activated protein kinase (MAPK) signaling plays a significant role in reactive oxygen species (ROS) production. The authors have previously shown that Brahma-related gene 1 (BRG1), a chromatin remodeling protein, contributes to hepatic ROS accumulation in multiple animal and cellular models of liver injury. Here it is reported that DNA damage-induced transcript 4 (DDIT4) is identified as a direct transcriptional target for BRG1. DDIT4 overexpression overcomes BRG1 deficiency to restore ROS production whereas DDIT4 knockdown phenocopies BRG1 deficiency in suppressing ROS production in vitro and in vivo. Mechanistically, DDIT4 coordinates the assembly of the p38-MAPK signaling complex to drive ROS production... (truncated)

Title: Valsartan improves {beta}-cell function and insulin sensitivity in subjects with impaired glucose metabolism: a randomized controlled trial.
Recently, the Nateglinide and Valsartan in Impaired Glucose Tolerance Outcomes Research Trial demonstrated that treatment with the angiotensin receptor blocker (ARB) valsartan for 5 years resulted in a relative reduction of 14% in the incidence of type 2 diabetes in subjects with impaired glucose metabolism (IGM). We investigated whether improvements in β-cell function and/or insulin sensitivity underlie these preventive effects of the ARB valsartan in the onset of type 2 diabetes.

Title: Effects of glucose load and nateglinide intervention on endothelial function and oxidative stress.
We analysed endothelial function and oxidative stress in patients with abnormal glucose metabolism, the effect of glucose load, and the impact of nateglinide. 109 participants were grouped into newly diagnosed diabetes, prediabetes, and control. Fasting plasma glucose (FPG), postprandial plasma glucose (PPG), glycosylated haemoglobin (HbA1c), and glycated albumin (GA) varied significantly among the study groups ($P < 0.01$). Nitric oxide (NO) and insulin resistance index (HOMA-IRI) levels were markedly different between the newly diagnosed diabetes and the control ($P < 0.01$). Glucose loading lowered flow-mediated endothelium-dependent dilation (FMEDD), NO, and superoxide dismutase (SOD) ($P < 0.01$). Fasting and glucose loading... (truncated)

Title: Association of CYP1A1 and CYP1B1 inhibition in in vitro assays with drug-induced liver injury.
Drug-induced liver injury (DILI) is one of the major causes for the discontinuation of drug development and withdrawal of drugs from the market. Since it is known that reactive metabolite formation and being substrates or inhibitors of cytochrome P450s (P450s) are associated with DILI, we systematically investigated the association between human P450 inhibition and DILI. The inhibitory activity of 266 DILI-positive drugs (DILI drugs) and 92 DILI-negative drugs (no-DILI drugs), which were selected from Liver Toxicity Knowledge Base (US Food and Drug Administration), against 8 human P450 forms was assessed using recombinant enzymes and luminescent substrates, and the threshold values... (truncated)

MESH:D005476 - fluphenazine

Summary:

LLM Prediction Score	0.155 (normalized)
LLM Confidence Score	0.980
Golden Answer (Severity Class)	0.375 (normalized)
Prediction Error	0.220

Retrieved Context:

Title: Fluphenazine reduces proteotoxicity in C. elegans and mammalian models of alpha-1-antitrypsin deficiency.
The classical form of α1-antitrypsin deficiency (ATD) is associated with hepatic fibrosis and hepatocellular carcinoma. It is caused by the proteotoxic effect of a mutant secretory protein that aberrantly accumulates in the endoplasmic reticulum of liver cells. Recently we developed a model of this deficiency in C. elegans and adapted it for high-content drug screening using an automated, image-based array scanning. Screening of the Library of Pharmacologically Active Compounds identified fluphenazine (Flu) among several other compounds as a drug which reduced intracellular accumulation of mutant α1-antitrypsin Z (ATZ). Because it is representative of the phenothiazine drug class that appears to... (truncated)

Title: Analysis of Mitochondrial Function in Cell Membranes as Indicator of Tissue Vulnerability to Drugs in Humans.
Drug side effects are one of the main reasons for treatment withdrawal during clinical trials. Reactive oxygen species formation is involved in many of the drug side effects, mainly by interacting with the components of the cellular respiration. Thus, the early detection of these effects in the drug discovery process is a key aspect for the optimization of pharmacological research. To this end, the superoxide formation of a series of drugs and compounds with antidepressant, antipsychotic, anticholinergic, narcotic, and analgesic properties was evaluated in isolated bovine heart membranes and on cell membrane microarrays from a collection of human tissues, together... (truncated)

Title: Disturbances of liver function of long acting neuroleptic drugs.
In a study in 97 patients on the occurrence of disturbances of liver functions by use of long acting neuroleptic drugs (fluphenazine decanoate, flupenthixol decanoate and fluspirilene) no indications were found that these agents, even in relatively high dosages, are hepatotoxic. Furthermore, in 40% of the patients slight disturbances were found, which nevertheless, were not specific in nature, and mostly concerned the turbidity tests. In spite of these findings a regular control (for instances, once a year) of the liver functions in patients with long acting neuroleptic drugs seems to be called for.

Title: Antipsychotic Drug Fluphenazine against Human Cancer Cells.
Drug repurposing is a strategy that can speed up and find novel clinical uses for already-approved drugs for several diseases, such as cancer. This process is accelerated compared to the development of new drugs because these compounds have already been tested in clinical trials and data related to their pharmacokinetics is already described, reducing the costs and time associated with the development of new anticancer therapeutics. Several studies suggest that the repurposing of fluphenazine for cancer therapy may be a promising approach, as this drug proved to reduce the viability of diverse cancer cell lines. In this review, intensive research... (truncated)

Title: Drug-Associated Liver Injury Related to Antipsychotics: Exploratory Analysis of Pharmacovigilance Data.
Drug-associated liver injury is one of the most common causes for acute liver failure and market withdrawal of approved drugs. In addition, the potential for hepatotoxicity related to specific substances has to be considered in psychopharmacotherapy. However, systematic evaluations of hepatotoxicity related to antipsychotics are limited.

MESH:D015282 - octreotide

Summary:

LLM Prediction Score	0.468 (normalized)
LLM Confidence Score	0.990
Golden Answer (Severity Class)	0.25 (normalized)
Prediction Error	0.218

Retrieved Context:

Title: [Treatment of acromegaly with octreotide, a synthetic analog of somatostatin with extended action].
Octreotide, an analog of somatostatin, is a valid tool for the cure of acromegalic disease. This compound has a prolonged half-life and is more selective than native somatostatin in suppressing growth hormone (GH) secretion. Octreotide, 100 micrograms tid sc, decreases GH levels and improves clinical symptoms in about 85% of acromegalic patients, lowering GH to below 5 ng/ml in 45% and to below 2 ng/ml in 17-21%. Octreotide normalizes somatomedin-C (IGF-I) levels in 36-50% of patients. The increase of dosage up to 1500 micrograms/day does not appear useful in poor responsive patients. No adverse effects on other endocrine functions submitted... (truncated)

Title: Abnormal liver function tests in a patient fed with total parenteral nutrition and treated with octreotide.
Octreotide is a long-acting analog of somatostatin, a hypothalamic release-inhibiting hormone. It is sometimes used therapeutically to relieve symptoms associated with acromegaly and gastroenteropancreatic endocrine tumors. It has also been used in the treatment of short-bowel syndrome and gastrointestinal and biliary fistulae. We report a patient with biliary leakage due to Marizzi's syndrome treated with total parenteral nutrition and octreotide who developed abnormal liver function tests that improved when the two treatments were stopped. We also review the literature regarding abnormal liver function tests in patients on total parenteral nutrition.

Title: Hepatitis in an infant treated with octreotide for congenital hyperinsulinism.
Congenital hyperinsulinism is characterized by hypoglycemia caused by several genetic disorders of inappropriate insulin secretion. Octreotide, an analogue of somatostatin, plays a major role in the pharmaceutical treatment of this condition. A 9-month-old infant treated with octreotide developed anicteric hepatitis with no other proven cause. After the discontinuation of this drug, the liver enzymes declined rapidly. Liver function tests should be followed in patients receiving octreotide.

Title: Octreotide-associated cholestasis and hepatitis in an infant with congenital hyperinsulinism.
Congenital hyperinsulinism (CHI) is the most common cause of prolonged hypoglycemia in the neonate. It is caused by several genetic mutations that interfere with the cascade of normal insulin secretion from pancreatic beta cells. Octreotide, a somatostatin analog, suppresses insulin secretion from pancreatic beta cells, and is an effective therapy used for both short and long term in the treatment of CHI. It is well tolerated in most patients; however, several adverse effects have been reported, most of them mild and transient. Impaired liver function has been described previously in few children. Here, we describe about a child with CHI... (truncated)

Title: Overexpression of P-glycoprotein, MRP2, and CYP3A4 impairs intestinal absorption of octreotide in rats with portal hypertension.
Portal hypertension (PH) is the main cause of complications and death in liver cirrhosis. The effect of oral administration of octreotide (OCT), a drug that reduces PH by the constriction of mesenteric arteries, is limited by a remarkable intestinal first-pass elimination.

MESH:C072379 - repaglinide

Summary:

LLM Prediction Score	0.409 (normalized)
LLM Confidence Score	0.960
Golden Answer (Severity Class)	0.625 (normalized)
Prediction Error	0.216

Retrieved Context:

Title: Repaglinide induced acute hepotototoxicity.
Repaglinide is considered a safe drug; adverse events are mild to moderate which includes hypoglycemia, headache, nausea, vomiting, diarrhea and dyspepsia as similar to sulphonylureas. This case report describes a rare side effect of repaglinide. In rare cases, elevated liver enzymes have been noted. We report a case of acute hepatotoxicity in a 78-year-old woman who developed acute hepatotoxicity while taking repaglinide.

Title: Formulation and Optimization of Repaglinide Nanoparticles Using Microfluidics for Enhanced Bioavailability and Management of Diabetes.
The technologies for fabrication of nanocrystals have an immense potential to improve solubility of a variety of the poor water-soluble drugs with subsequent enhanced bioavailability. Repaglinide (Rp) is an antihyperglycemic drug having low bioavailability due to its extensive first-pass metabolism. Microfluidics is a cutting-edge technique that provides a new approach for producing nanoparticles (NPs) with controlled properties for a variety of applications. The current study's goal was to engineer repaglinide smart nanoparticles (Rp-Nc) utilizing microfluidic technology (Dolomite Y shape), and then to perform in-vitro, in-vivo, and toxicity evaluations of them. This method effectively generated nanocrystals with average particle sizes of... (truncated)

Title: Non-uniformity of Changes in Drug-Metabolizing Enzymes and Transporters in Liver Cirrhosis: Implications for Drug Dosage Adjustment.
Liver cirrhosis is a chronic disease that affects the liver structure, protein expression, and overall metabolic function. Abundance data for drug-metabolizing enzymes and transporters (DMET) across all stages of disease severity are scarce. Levels of these proteins are crucial for the accurate prediction of drug clearance in hepatically impaired patients using physiologically based pharmacokinetic (PBPK) models, which can be used to guide the selection of more precise dosing. This study aimed to experimentally quantify these proteins in human liver samples and assess how they can impact the predictive performance of the PBPK models. We determined the absolute abundance of 51... (truncated)

Title: Comparison of twelve single-drug regimens for the treatment of type 2 diabetes mellitus.
We performed a network meta-analysis to compare the efficacy of 12 single-drug regimens (Glibenclamide, Glimepiride, Pioglitazone, Rosiglitazone, Repaglinide, Metformin, Sitaglitin, Exenatide, Liraglutide, Acarbose, Benfluorex, and Glipizide) in the treatment of type 2 diabetes mellitus (T2DM). Fifteen relevant randomized controlled trials (RCTs) were included; direct and indirect evidence from these studies was combined, and weighted mean difference (WMD) and surface under the cumulative ranking curves (SUCRAs) were examined to evaluate the monotherapies. Liraglutide was more effective than Glimepiride, Pioglitazone, Sitaglitin, Exenatide, and Glipizide at reducing glycated hemoglobin (HbA1c) levels. In contrast, Acarbose was less effective than Glibenclamide, Glimepiride, Pioglitazone, Rosiglitazone, Repaglinide,... (truncated)

Title: Repaglinide/metformin fixed-dose combination to improve glycemic control in patients with type 2 diabetes: an update.
Type 2 diabetes is a progressive disease associated with high levels of morbidity and mortality and for which there is both a large and growing prevalence worldwide. Lifestyle advice plus metformin is commonly recommended initially to manage hyperglycemia and to minimize the risk of vascular complications. However, additional agents are required when glycemic targets cannot be achieved or maintained due to the progressive nature of the disease. Repaglinide/metformin fixed-dose combination (FDC) therapy (PrandiMet(®); Novo Nordisk, Bagsværd, Denmark) has been approved for use in the USA. This FDC is a rational second-line therapy given the complementary mechanisms of action of the... (truncated)

MESH:D003023 - cloxacillin

Summary:

LLM Prediction Score	0.591 (normalized)
LLM Confidence Score	0.980
Golden Answer (Severity Class)	0.375 (normalized)
Prediction Error	0.216

Retrieved Context:

Title: Flucloxacillin-induced cholestatic liver damage.
Two female patients (75 and 68 yr old) developed jaundice 4 and 7 weeks respectively after treatment with flucloxacillin. Liver biopsies showed intrahepatic cholestasis. After cessation of the drug, the liver tests became normal.

Title: Cloxacillin-induced acute vanishing bile duct syndrome: A case study and literature review.
Ductopenia is often regarded as a chronic process where ≥50% of portal tracts lack bile ducts, which is also known as vanishing bile duct syndrome (VBDS). One aetiology is drug-induced liver injury. Cloxacillin, an antistaphylococcal penicillin, typically causes "bland" cholestasis. We present the first case of cloxacillin-induced acute ductopenia or VBDS and a review of published cloxacillin-induced liver injuries. A 66-year-old woman with no prior liver disease, but known penicillin allergy, was treated for postcarotid angioplasty staphylococcal infection with 6 weeks of cloxacillin. She presented with a 2-week history of weakness and jaundice. Laboratory work-up showed elevated liver enzymes with... (truncated)

Title: Flucloxacillin induced delayed cholestatic hepatitis.
We report four cases of severe delayed cholestatic hepatitis induced by flucloxacillin. All patients presented with deep jaundice and pruritus which developed soon after ceasing flucloxacillin. Liver function tests were abnormal in all patients with markedly elevated serum bilirubin concentration, alkaline phosphatase and aspartate transaminase levels. Extrahepatic biliary obstruction and infective hepatitis were excluded in all cases. Liver biopsies showed centrilobular cholestasis with portal and lobular inflammation and eosinophil infiltration. Although symptoms resolved within six weeks in all patients, cholestatic liver function tests have persisted in two patients for more than six months. With the increasing usage of this drug... (truncated)

Title: Hepatic safety of antibiotics used in primary care.
Antibiotics used by general practitioners frequently appear in adverse-event reports of drug-induced hepatotoxicity. Most cases are idiosyncratic (the adverse reaction cannot be predicted from the drug's pharmacological profile or from pre-clinical toxicology tests) and occur via an immunological reaction or in response to the presence of hepatotoxic metabolites. With the exception of trovafloxacin and telithromycin (now severely restricted), hepatotoxicity crude incidence remains globally low but variable. Thus, amoxicillin/clavulanate and co-trimoxazole, as well as flucloxacillin, cause hepatotoxic reactions at rates that make them visible in general practice (cases are often isolated, may have a delayed onset, sometimes appear only after cessation... (truncated)

Title: [Hepatotoxicity caused by cloxacillin].
We describe acute mixed hepatitis (cholestatic and hepatocellular) in two women probably induced by cloxacillin. Viral causes, autoimmune hepatitis, biliary tree obstruction and other risk factors were excluded. After discontinuation of the drug evolution was favorable and clinical signs and hepatic laboratory tests became normal in both cases. We also review the literature about hepatotoxicity induced by anti-Staphylococci semisynthetic penicillins.

MESH:D008723 - methohexital

Summary:

LLM Prediction Score	0.160 (normalized)
LLM Confidence Score	0.970
Golden Answer (Severity Class)	0.375 (normalized)
Prediction Error	0.215

Retrieved Context:

Title: Comparison of Bolus Dosing of Methohexital and Propofol in Elective Direct Current Cardioversion.
Background Methohexital and propofol can both be used as sedation for direct current cardioversion (DCCV). However, there are limited data comparing these medications in this setting. We hypothesized that patients receiving methohexital for elective DCCV would be sedated more quickly, recover from sedation faster, and experience less adverse effects. Methods and Results This was a prospective, blinded randomized controlled trial conducted at a single academic medical center. Eligible participants were randomly assigned to receive either methohexital (0.5 mg/kg) or propofol (0.8 mg/kg) as a bolus for elective DCCV. The times from bolus of the medication to achieving a Ramsay Sedation Scale score... (truncated)

Title: Intravenous hypnotic regimens in patients with liver disease; a review article.
The liver as an important organ in the body has many essential functions in physiological processes. One of the major activities of liver is drug metabolism. Hepatic dysfunction affecting hepatic physiological activities, especially drug metabolism can cause many problems during anesthesia and administration of different drugs to patients.

Title: Drug Repurposing for the Management of Depression: Where Do We Stand Currently?
A slow rate of new drug discovery and higher costs of new drug development attracted the attention of scientists and physicians for the repurposing and repositioning of old medications. Experimental studies and off-label use of drugs have helped drive data for further studies of approving these medications. A deeper understanding of the pathogenesis of depression encourages novel discoveries through drug repurposing and drug repositioning to treat depression. In addition to reducing neurotransmitters like epinephrine and serotonin, other mechanisms such as inflammation, insufficient blood supply, and neurotoxicants are now considered as the possible involved mechanisms. Considering the mentioned mechanisms has resulted... (truncated)

Title: Nicotine self-administration with menthol and audiovisual cue facilitates differential packaging of CYP2A6 and cytokines/chemokines in rat plasma extracellular vesicles.
In this study, we investigated whether intravenously self-administered nicotine with menthol and audiovisual cue modulates nicotine-metabolizing CYP2A6, oxidative stress modulators, and cytokines/chemokines in plasma extracellular vesicles (EVs) in rats. We assigned rats to self-administered nicotine with: (a) audiovisual cue (AV), (b) menthol, and (c) menthol and AV cue. We found increased levels of CD9 in plasma EVs after self-administered nicotine with menthol and AV cue. Moreover, expression of CYP2A6 in plasma EVs was significantly increased after self-administered nicotine in response to menthol and AV cue. However, despite an upward trend on SOD1 and catalase, increase was not found to be... (truncated)

Title: Ketamine and depression: a narrative review.
Depression is the third leading cause of disability in the world. Depressive symptoms may be reduced within several weeks after the start of conventional antidepressants, but treatment resistance concerns one-third of patients who fail to achieve recovery. Over the last 20 years, ketamine, an antagonist of the N-methyl-D-aspartate receptor, has been described to have antidepressant properties. A literature review was conducted through an exhaustive electronic search. It was restricted to Cochrane reviews, meta-analyses, and randomized controlled trials (RCTs) of ketamine for major depressive disorder and/or bipolar disorder. This review included two Cochrane reviews, 14 meta-analyses and 15 trials. Ketamine was... (truncated)

MESH:D010862 - pilocarpine

Summary:

LLM Prediction Score	0.161 (normalized)
LLM Confidence Score	0.980
Golden Answer (Severity Class)	0.375 (normalized)
Prediction Error	0.214

Retrieved Context:

Title: Anticonvulsant Effect of Turmeric and Resveratrol in Lithium/Pilocarpine-Induced Status Epilepticus in Wistar Rats.
Epilepsy is a chronic neurological disorder that lacks a cure. The use of plant-derived antioxidant molecules such as those contained in turmeric powder and resveratrol may produce short-term anticonvulsant effects. A total of 42 three-month-old male Wistar rats were divided into six groups (<i>n</i> = 7 in each group): Vehicle (purified water), turmeric (150 and 300 mg/kg, respectively), and resveratrol (30 and 60 mg/kg, respectively), administered <i>per os</i> (p.o.) every 24 h for 35 days. Carbamazepine (300 mg/kg/5 days) was used as a pharmacological control for anticonvulsant activity. At the end of the treatment, status epilepticus was induced using the... (truncated)

Title: Effects of modulating M3 muscarinic receptor activity on azoxymethane-induced liver injury in mice.
Previously, we reported that azoxymethane (AOM)-induced liver injury is robustly exacerbated in M3 muscarinic receptor (M3R)-deficient mice. We used the same mouse model to test the hypothesis that selective pharmacological modulation of M3R activity regulates the liver injury response. Initial experiments confirmed that giving a selective M3R antagonist, darifenacin, to AOM-treated mice mimicked M3R gene ablation. Compared to vehicle controls, mice treated with the M3R antagonist had reduced survival and increased liver nodularity and fibrosis. We next assessed AOM-induced liver injury in mice treated with a selective M3R agonist, pilocarpine. After pilocarpine treatment, stimulation of post-M3R signaling in the liver... (truncated)

Title: Celecoxib Decrease Seizures Susceptibility in a Rat Model of Inflammation by Inhibiting HMGB1 Translocation.
The risk of developing epilepsy is strongly linked to peripheral inflammatory disorders in humans. High-mobility group box protein 1 (HMGB1) has the most focus for being a suspect in this scenario. The current study aimed to detect the celecoxib effect, an anti-inflammatory drug, on decreasing seizure susceptibility and organ damage in lipopolysaccharides (LPS)/pilocarpine (PILO) pretreated Wistar rats. Rats were divided into 6 groups (8 each): group 1 (control), group 2 (PILO), group 3 (PILO+LPS), group 4 (PILO+LPS+(VPA) Valproic acid), group 5 (PILO+LPS+Celecoxib), and group 6 (PILO+LPS+VPA+Celecoxib). LPS was used to induce sepsis and PILO to induce seizures. Oxidative stress markers,... (truncated)

Title: The standardized extract of Centella asiatica L. Urb attenuates the convulsant effect induced by lithium/pilocarpine without affecting biochemical and haematological parameters in rats.
Status epilepticus (SE) is a type of epileptic activity characterized by a failure of the inhibitory mechanisms that limit seizures, which are mainly regulated by the GABAergic system. This imbalance increases glutamatergic neurotransmission and consequently produces epileptic activity. It is also associated with oxidative stress due to an imbalance between reactive oxygen species (ROS) and antioxidant defences. Unfortunately, long-term treatment with anti-epileptic drugs (AEDs) may produce hepatotoxicity, nephrotoxicity, and haematological alterations. In this way, some secondary metabolites of plants have been used to ameliorate the deterioration of nervous system disorders through their antioxidant properties, in addition to their anticonvulsant effects.... (truncated)

Title: The muscarinic agonist pilocarpine modifies cocaine-reinforced and food-reinforced responding in rats: comparison with the cholinesterase inhibitor tacrine.
Activation of muscarinic receptors in the brain antagonizes the actions of cocaine, blocking both its discriminative stimulus and reinforcing properties. Pilocarpine is a nonselective muscarinic agonist that is used clinically, but has not been well characterized for its actions during cocaine-reinforced behavior. This study evaluated its effects on cocaine-reinforced and food-reinforced behaviors in rats, using the cholinesterase inhibitor tacrine as a comparator. Intraperitoneal pilocarpine or tacrine at doses of 1.0 mg/kg or more attenuated self-administration of low-dose cocaine (0.1 mg/kg injection) but also increased oral movements. Pilocarpine was less potent than tacrine in decreasing responding supported by low or intermediate... (truncated)

MESH:D013311 - streptozocin

Summary:

LLM Prediction Score	0.589 (normalized)
LLM Confidence Score	0.970
Golden Answer (Severity Class)	0.375 (normalized)
Prediction Error	0.214

Retrieved Context:

Title: Induction of diabetes in cynomolgus monkey with one shot of analytical grade streptozotocin.
Streptozotocin (STZ)- induced diabetic monkey is a wide used preclinical animal model for the investigation of diabetes such as islet transplantation and development of diabetic drugs. There are serious side effects of this method, including nausea, emesis, weight loss, liver damage, renal failure, and metabolic acidosis. In order to reduce the side effects, diabetic monkeys were induced using clinical-grade STZ. However, clinical-grade STZ is not available in China. Here, we establised a method by using 100 mg/kg analytical-grade STZ to induce complete diabetes in cynomolgus monkey without generating adverse effects to liver and renal.

Title: Successful pharmaceutical-grade streptozotocin (STZ)-induced hyperglycemia in a conscious tethered baboon (Papio hamadryas) model.
Non-human primate (NHP) diabetic models using chemical ablation of β-cells with STZ have been achieved by several research groups. Chemotherapeutic STZ could lead to serious adverse events including nephrotoxicity, hepatotoxicity, and mortality.

Title: Preclinical toxicity study of streptozocin infused into the internal carotid artery of dogs and baboons.
Mature male and female dogs (10) and male baboons (4) were each given a single dose of streptozocin (STZ) (1000 mg/m2) by infusion directly into the left internal carotid artery over a two-hour time period. Serial hematology and serum chemistry profiles and physical observations were made, the animals necropsied at varying times after dosage, and the major organs examined histologically. In dogs, severe weight loss, neutrophilia, electrolyte disturbances, decreased liver function, diabetes and decreased serum amylase levels were the major toxicities. In baboons, weight loss, hypoglycemic coma, diabetes, decreased liver function and electrolyte disturbances were observed. While systemic toxicities were... (truncated)

Title: Insight into the hepatoprotective, hypolipidemic, and antidiabetic impacts of aliskiren in streptozotocin-induced diabetic liver disease in mice.
Diabetic hepatopathy is a serious complication of poorly controlled diabetes mellitus. An efficient antidiabetic drug which keeps normal liver tissues is not available. The renin-angiotensin system has been reported to be involved in both diabetic state and liver function. Aliskiren is a direct renin inhibitor and a recently antihypertensive drug with poly-pharmacological properties. The aim of the current study is to explore the possible hepatoprotective effects and mechanisms of action of aliskiren against streptozotocin (STZ) induced liver toxicity.

Title: Influence of diabetes on liver injury induced by antitubercular drugs and on silymarin hepatoprotection in rats.
Isoniazid, rifampicin and pyrazinamide during short-course chemotherapy for tuberculosis can result in liver injury. The coexistence of tuberculosis and diabetes is common in patients who receive inadequate treatment. The risk of hepatotoxicity from many toxicants is increased in diabetic rats. Silymarin provides protection against liver injury caused by many hepatotoxicants, including antitubercular drugs (ATDs). In the wake of increased severity of ATD-induced hepatotoxicity in diabetes we report here the results of a study on the influence of diabetes on silymarin hepatoprotection in rats. Rats with diabetes induced via intraperitoneally injected streptozotocin (50 mg/kg), nondiabetic rats and insulin-treated diabetic rats received... (truncated)

MESH:D002440 - cefoxitin

Summary:

LLM Prediction Score	0.164 (normalized)
LLM Confidence Score	0.980
Golden Answer (Severity Class)	0.375 (normalized)
Prediction Error	0.211

Retrieved Context:

Title: [Clinical experience of cefoxitin in the field of obstetrics and gynecology (author's transl)].
Cefoxitin was given to the 7 patients of infections in the field of obstetrics and gynecology, and the following results were obtained: 1) The clinical response was excellent in 2 patients, good in 4 and poor in 1 patient with the efficacy rate of 85.7%. Out of the 4 patients resistant to the previous therapy with other antibiotics, 3 patients responded to cefoxitin, and all the 3 patients of anaerobic infections responded satisfactorily to cefoxitin. 2) Microorganisms isolated were 2 strains each of E. coli and Staphylococcus aureus, 3 strains of Peptococcus and 1 strain of Eubacterium lentum. All the... (truncated)

Title: Pharmacokinetics, efficacy and tolerance of cefoxitin in the treatment of cefoxitin-susceptible extended-spectrum beta-lactamase producing Enterobacterales infections in critically ill patients: a retrospective single-center study.
Cefoxitin is active against some extended-spectrum beta-lactamase-producing Enterobacterales (ESBL-PE), but has not been evaluated so far in the intensive care unit (ICU) settings. Data upon its pharmacokinetics (PK), tolerance and efficacy in critical conditions are scanty. We performed a retrospective single-center study in a university hospital medical ICU, in subjects presenting with cefoxitin-susceptible ESBL-PE infection and treated with cefoxitin. The primary aim was to determine cefoxitin PK. Secondary endpoints were efficacy, tolerance, and emergence of cephamycin-resistance.

Title: Hepatitis following famotidine: a case report.
H2 receptor antagonists can rarely cause idiosyncratic drug reactions leading to acute hepatitis. Famotidine, however, is considered a relatively safe drug with regards to hepatotoxicity. We report a case of a 47 year old male with a history of hepatitis C who developed acute hepatitis on the third day of hospitalization with a dramatic rise in his liver enzymes from normal values at the time of admission. The acute rise in liver enzymes made us consider an adverse drug reaction and famotidine was discontinued. Subsequently his liver enzymes came back to normal in seven days. Thus, physicians should consider famotidine... (truncated)

Title: Second-Generation Cephalosporins-Associated Drug-Induced Liver Disease: A Study in VigiBase with a Focus on the Elderly.
The objective of this study was to characterize individual case safety reports (ICSRs) and adverse drug reactions (ADRs) related to second-generation cephalosporins and resulting in hepatobiliary disorders, in VigiBase, WHO global database.

Title: Extraintestinal Manifestation of <i>Yersinia pseudotuberculosis</i> Bacteremia as Acute Hepatitis: Case Report and Review of the Literature.
<i>Yersinia pseudotuberculosis</i> is a causative agent of foodborne zoonosis that usually causes self-limiting pseudoappendicitis. <i>Y. pseudotuberculosis</i> infection also causes systemic spread or extraintestinal manifestations in patients with predisposing conditions. Here, we present a case of acute hepatitis with <i>Y. pseudotuberculosis</i> bacteremia in a 30-year-old man. He was previously healthy without significant medical history other than obesity and current smoking. At the time of admission, he presented with high fever accompanied by chills, jaundice, abdominal pain, and watery diarrhea. Laboratory studies revealed leukocytosis and elevated liver function parameters. A stool culture showed no causative pathogens. Empiric antibiotic therapy with ceftriaxone and... (truncated)

MESH:D000068818 - cetuximab

Summary:

LLM Prediction Score	0.211 (normalized)
LLM Confidence Score	0.990
Golden Answer (Severity Class)	0.0 (normalized)
Prediction Error	0.211

Retrieved Context:

Title: Reversible grade 4 hyperbilirubinemia in a patient with UGT1A1 7/7 genotype treated with irinotecan and cetuximab.
Irinotecan-induced gastrointestinal toxicities are common and typically present in the form of diarrhea or nausea and vomiting. However, severe hyperbilirubinemia (grade 3/4) has not been previously reported in association with this chemotherapeutic agent. We report a case of prolonged grade 4 hyperbilirubinemia after a single dose of irinotecan at 125 mg/m(2). This severe toxicity was attributed to a UGT1A1 7/7 genotype and resolved to grade 2 after 8 weeks of supportive care. This case outlines the possibility of severe hepatic toxicity with moderate doses of irinotecan in patients with a UGT1A1 7/7 genotype. Despite the severity and prolonged duration of... (truncated)

Title: Cetuximab for the treatment of locally advanced and recurrent/metastatic oral cancer: An investigation of distant metastasis.
The aim of this retrospective study was to assess the efficacy and safety of cetuximab therapy for patients with locally advanced (LA) and recurrent/metastatic (R/M) oral squamous cell carcinoma (OSCC), with a specific focus on distant metastases (DMs). Data from 21 patients with unresectable LA and R/M OSCC treated with cetuximab therapy in our department between December, 2012 and July, 2015 were reviewed. The endpoint was the time-to-progression and the assessments made were tumor response rate, progression-free survival (PFS), overall survival (OS) and safety. The overall response rate was 57.1%, with a complete response (CR) rate of 33.3%. The overall... (truncated)

Title: Cetuximab as treatment for head and neck cancer patients with a previous liver transplant: report of two cases.
Cetuximab is a monoclonal antibody against epidermal growth factor receptor useful in the treatment of patients with Head and Neck Squamous Cell Carcinoma combined with radiotherapy or chemotherapy. Its pharmacokinetics are not influenced by hepatic status and there are no specific warnings concerning its indication in patients with impaired hepatic function. Patients with a previous liver transplant are at risk for hepatic toxicity and use immunosupressants to avoid rejection that can interact with other drugs. We present two cases of patients with a previous liver transplant in which cetuximab was administered to treat head and neck cancer.

Title: [Two Cases of Colon Cancer with Severe Liver Dysfunction Due to Multiple Liver Metastases Effectively Treated with Hepatic Arterial Infusion Chemotherapy plus CetuximabFollowed by Systemic Chemotherapy].
Case 1: An 80-year-old man was diagnosed with cecal cancer plus multiple liver metastases and peritoneal disseminations. He underwent surgical resection of the primary tumor to prevent bowel obstruction. Initially, hepatic arterial infusion(HAI) plus cetuximab(Cmab)was administered to reduce the size of the metastatic tumors and prevent liver failure. A partial response(PR)was observed in the liver metastases after 12 courses of treatment and S-1 plus oxaliplatin(SOX)plus bevacizumab was started. Case 2: A 44-year-old man was diagnosed with sigmoid colon cancer with multiple liver, lung and bone metastases, and with obstructive jaundice and cholangitis due to severe liver hilum lymph node metastases.... (truncated)

Title: Hepatic toxicities associated with the use of preoperative systemic therapy in patients with metastatic colorectal adenocarcinoma to the liver.
Colorectal cancer patients with isolated liver metastasis are potentially cured with surgical resection. Recent advances in systemic chemotherapy have increased the ability to convert unresectable metastatic liver lesions to resectable lesions. The cost in toxicity of these therapeutic advances is increasingly being recognized. Numerous reports have demonstrated an association between irinotecan and steatohepatitis as well as between oxaliplatin and sinusoidal dilation. In this review, we summarize the current clinical experience with these hepatic toxicities and discuss the role they play in determining postoperative morbidity. We also review emerging safety data regarding the use of bevacizumab and cetuximab. Finally, we give... (truncated)

MESH:D015736 - felodipine

Summary:

LLM Prediction Score	0.164 (normalized)
LLM Confidence Score	0.950
Golden Answer (Severity Class)	0.375 (normalized)
Prediction Error	0.211

Retrieved Context:

Title: A risk-benefit assessment of losartan potassium in the treatment of hypertension.
Losartan potassium is the first of a new class of orally active antihypertensive drugs which antagonise the action of angiotensin (AT) II at the AT1 receptor subtype. Losartan potassium is converted by the liver to the active metabolite E-3174, which is a more potent antagonist at the AT1 receptor. E-3174 is responsible for most of the pharmacological effects of losartan potassium, and its long half-life contributes to the extended duration of action of the drug. Losartan potassium is effective as a once-daily antihypertensive agent. In mild to moderate hypertension, losartan potassium has similar efficacy to enalapril, atenolol and felodipine extended... (truncated)

Title: Food-drug interactions precipitated by fruit juices other than grapefruit juice: An update review.
This review addressed drug interactions precipitated by fruit juices other than grapefruit juice based on randomized controlled trials (RCTs). Literature was identified by searching PubMed, Cochrane Library, Scopus and Web of Science till December 30 2017. Among 46 finally included RCTs, six RCTs simply addressed pharmacodynamic interactions and 33 RCTs studied pharmacokinetic interactions, whereas seven RCTs investigated both pharmacokinetic and pharmacodynamic interactions. Twenty-two juice-drug combinations showed potential clinical relevance. The beneficial combinations included orange juice-ferrous fumarate, lemon juice-^{99m}Tc-tetrofosmin, pomegranate juice-intravenous iron during hemodialysis, cranberry juice-triple therapy medications for H. pylori, blueberry juice-etanercept, lime juice-antimalarials, and wheat grass juice-chemotherapy. The potential... (truncated)

Title: Pharmacokinetics of calcium antagonists under development.
Calcium antagonist drugs under clinical development are of the Type I (verapamil, diltiazem-like) and Type II (nifedipine-like) classes. Tiapamil, the only Type I drug currently available, is a high clearance, widely distributed drug which undergoes extensive presystemic elimination. Pharmacokinetically it is quite similar to verapamil; however, it does have increased biliary excretion and decreased binding to plasma proteins. Eight Type II (dihydropyridine) drugs are reviewed. Seven of these drugs (felodipine, isradipine, nicardipine, nilvadipine, nimodipine, nisoldipine and nitrendipine) are pharmacokinetically similar to nifedipine, with high clearance, extensive distribution, and significant presystemic elimination. Amlodipine has lower clearance, even greater peripheral distribution, and... (truncated)

Title: Stereoselective pharmacokinetics of dihydropyridine calcium antagonists.
Many dihydropyridine calcium antagonists are widely used for the treatment of angina and hypertension, and many more are under development. Most of these drugs have one or more chiral centre, and the pharmacological activity between the enantiomers for these drugs is known to be markedly different. First, the stereospecific assay methods for these drugs in plasma or serum are reviewed with emphasis on chiral stationary phase high-performance liquid chromatography for their determination. Next, the stereoselective pharmacokinetics of these drugs (nilvadipine, nitrendipine, felodipine, nimodipine, manidipine, benidipine and nisoldipine) in animals, healthy subjects and patients with hepatic disease is reviewed. Enantiomer-enantiomer interaction,... (truncated)

Title: Beverage-induced enhanced bioavailability of carbamazepine and its consequent effect on antiepileptic activity and toxicity.
The present study was undertaken to investigate the food-drug interaction of carbamazepine (CBZ). Common fruit juices [grapefruit juice (GFJ), lime juice (LJ)], known to inhibit the enzyme cytochrome P450 3A4 (CYP3A4), and some widely consumed beverages [milk (M), black tea (BT)] were involved in this study in the presence of CBZ, as might happen during clinical therapy. The effects of the beverages on the pharmacokinetics and drug-induced toxicity of CBZ was observed after concomitant administration for a period of 28 days. Accordingly, the influence of altered bioavailability of CBZ on its antiepileptic activity was investigated. A significant shift in the... (truncated)

MESH:D006852 - hydrochlorothiazide

Summary:

LLM Prediction Score	0.460 (normalized)
LLM Confidence Score	0.990
Golden Answer (Severity Class)	0.25 (normalized)
Prediction Error	0.210

Retrieved Context:

Title: Hydrochlorothiazide-induced hepatotoxicity: A rare case of DILI.
Thiazide diuretics are prescribed daily and rarely hepatotoxic. We report the case of 86-year-old woman who was admitted in hospital for jaundice after taking hydrochlorothiazide. All differential diagnoses have been eliminated. The liver biopsy was compatible with drug-induced hepatitis. Clinical and biological manifestations improved after discontinuation of the treatment. The reported case is compared to three other cases in the literature.

Title: Jaundice and rash associated with the use of phenobarbital and hydrochlorothiazide.
Rash, lymphadenopaty, splenomegaly, periorbital edema, and hepatitis occurred in an 18-year-old woman who was taking phenobarbital and hydrochlorothiazide. Tests for fluorescent antinuclear antibody and hepatitis-associated antigen and antibody were negative. Liver biopsy was not characteristic of viral hepatitis. Clinical recovery occurred within two weeks. Treatment consisted of withdrawal of the above drugs plus the administration of methylprednisolone and diphenhydramine.

Title: Drug-induced liver injury after switching from tamoxifen to anastrozole in a patient with a history of breast cancer being treated for hypertension and diabetes.
Anastrozole is a selective non-steroidal aromatase inhibitor that blocks the conversion of androgens to estrogens in peripheral tissues. It is used as adjuvant therapy for early-stage hormone-sensitive breast cancer in postmenopausal women. Significant side effects of anastrozole include osteoporosis and increased levels of cholesterol. To date, seven case reports on anastrozole hepatotoxicity have been published. We report the case of an 81-year-old woman with a history of breast cancer, arterial hypertension, type 2 diabetes mellitus, hyperlipidemia, and chronic renal insufficiency. Four days after switching hormone therapy from tamoxifen to anastrozole, icterus developed along with a significant increase in liver enzymes... (truncated)

Title: Telmisartan/hydrochlorothiazide-induced hepatotoxicity.
No abstract available.

Title: Clopidogrel-induced liver damage: A case report and review of the literature.
Liver damage is a rare side effect of clopidogrel. That is reversible in most cases. Considering the widespread use of this medication in cardiovascular diseases, the management of hepatotoxicity requires further meticulous investigation.

MESH:D014859 - warfarin

Summary:

LLM Prediction Score	0.415 (normalized)
LLM Confidence Score	0.990
Golden Answer (Severity Class)	0.625 (normalized)
Prediction Error	0.210

Retrieved Context:

Title: Acute Warfarin Toxicity as Initial Manifestation of Metastatic Liver Disease.
Near complete infiltration of the liver secondary to metastasis from the head and neck cancer is a rare occurrence. The prognosis of liver failure associated with malignant infiltration is extremely poor; the survival time of patients is extremely low. We present a case of acute warfarin toxicity as initial manifestation of metastatic liver disease. Our patient is a 64-year-old woman presenting with epigastric pain and discomfort, found to have unrecordable International Normalized Ratio. She rapidly deteriorated with acute respiratory failure requiring mechanical ventilation, profound shock requiring high dose vasopressor infusion, severe coagulopathy, worsening liver enzymes with worsening of lactic acidosis... (truncated)

Title: [Thrombosed St. Jude Medical prosthesis with drug induced hepatitis due to warfarin potassium--a case report].
A case was presented of a 51-year-old woman who underwent aortic valve replacement with a St. Jude Medical prosthesis two and a half years before. The patient was initially placed on Warfarin potassium after the surgery. However she gradually developed jaundice during the period of two months Warfarin was replaced with ticlopidine hydrochloride as it was thought to be the most probable cause of jaundice. The prosthesis was subsequently thrombosed and had to be replaced with a Carpentier bovine pericardial valve. Warfarin potassium rarely induces the hepatic dysfunction as a result of drug allergy. However, the prompt diagnosis and adequate... (truncated)

Title: Hepatotoxicity of New Oral Anticoagulants (NOACs).
Case reports and analyses of clinical studies and of pharmacovigilance data suggest that new oral anticoagulants (NOACs) are associated with a small risk for hepatotoxicity. The objective of this publication is to summarize the current data about this subject, with a special emphasis on pharmacovigilance data in the World Health Organization (WHO) Global Individual Case Safety Reports (ICSR) database and on potential mechanisms of hepatotoxicity. For that, all available case reports as well as published analyses of clinical studies were obtained with a detailed search in PubMed. In addition, pharmacovigilance data from VigiBase(®), the WHO Global ICRS database, were extracted... (truncated)

Title: Abnormal serum transaminases following therapeutic doses of acetaminophen in the absence of known risk factors.
J.M., a healthy, 25-year-old male, volunteered for a study involving warfarin and acetaminophen. Acetaminophen 1 g four times a day was started for 21 days. Liver function tests taken at regular intervals for the first 12 days were unremarkable. On day 18, however, aspartate aminotransferase (AST) was 527 IU/liter and alanine aminotransferase (ALT) was 166 IU/liter. Acetaminophen was discontinued and serum transaminase levels returned to baseline levels two weeks later (AST = 26, ALT = 20). Analysis of J.M.'s urine samples over the first 18 days showed excretion patterns of glucuronide, sulfate, and glutathione derived cysteine and mercapturic acid conjugates... (truncated)

Title: Acetaminophen causes an increased International Normalized Ratio by reducing functional factor VII.
Acetaminophen may increase International Normalized Ratio (INR) in patients taking anticoagulation medication, and in patients with acetaminophen poisoning without hepatic injury. The objective of this study was to describe and investigate the effect of acetaminophen on INR. The authors studied patients admitted to a regional toxicology treatment center with acetaminophen poisoning with INR and without potentially confounding coingestion or hepatic injury. Exposed and nonexposed (control) cohorts were recruited from admissions with acetaminophen poisoning and psychotropic drug poisoning, respectively. From 1,437 acetaminophen poisonings, after exclusions, there were 143 admissions with 205 estimations of INR. INR showed a time-dependent increase. Fifty percent... (truncated)

MESH:C042705 - pegaspargase

Summary:

LLM Prediction Score	0.584 (normalized)
LLM Confidence Score	0.960
Golden Answer (Severity Class)	0.375 (normalized)
Prediction Error	0.209

Retrieved Context:

Title: Clinical Utility of Pegaspargase in Children, Adolescents and Young Adult Patients with Acute Lymphoblastic Leukemia: A Review.
Acute lymphoblastic leukemia (ALL) is a heterogenous hematological malignancy representing 25% of all cancers in children less than 15 years of age. Significant improvements in survival and cure rates have been made over the past four decades in pediatric ALL treatment. Asparaginases, derived from <i>Escherichia coli</i> and <i>Erwinia chrysanthemi</i>, have become a critical component of ALL therapy since the 1960s. Asparaginases cause depletion of serum asparagine, leading to deprivation of this critical amino acid for protein synthesis, and hence limit survival of lymphoblasts. Pegaspargase, a conjugate of monomethoxypolyethylene glycol (mPEG) and L-asparaginase, has become an integral component of pediatric upfront... (truncated)

Title: L-Carnitine for Treatment of Pegasparaginase-Induced Hepatotoxicity.
Similar to pediatric regimens, multiple doses of L-asparaginase (PEG-Asp) are being increasingly used in adults with newly diagnosed acute lymphoblastic leukemia (ALL) with promising results. One of the most common side effects of the drug in adults is high-grade hyperbilirubinemia and transaminitis. Despite being almost always reversible and may not recur, clinicians may still be reluctant to continue with PEG-Asp in patients with liver toxicity, losing the benefit from multiple doses of the drug.

Title: Asparaginase-induced hepatotoxicity: rapid development of cholestasis and hepatic steatosis.
L-Asparaginase is a bacterial enzyme used in the treatment of acute lymphoblastic leukemia. In the ongoing U.S. Drug-Induced Liver Injury Network (DILIN) prospective study, standard and pegylated asparaginase were the most frequent cause of liver injury with jaundice among anti-cancer agents (8 of 40: 20%). The unique features of this hepatotoxicity are described.

Title: Liver failure after treatment with inotuzumab and polychemotherapy including PEG-asparaginase in a patient with relapsed Philadelphia chromosome-negative acute lymphoblastic leukemia.
We present the case of a 58-year-old female patient who presented with an extramedullary B-ALL relapse after prior allogenic HSCT and blinatumomab therapy. The patient died from complications of a drug-induced acute liver failure after a salvage therapy combining inotuzumab ozogamicin (InO)-based induction followed by consolidation with high dose MTX and pegaspargase based on the GMALL protocol for older ALL patients. After a diagnosis of the extramedullary relapse in the form of a retro vesical chloroma, the patient received an individualized multi-agent chemotherapy based on induction chemotherapy for older patients in combination with InO. After four administrations of InO, in... (truncated)

Title: Clinical and demographic factors contributing to asparaginase-associated toxicities in children with acute lymphoblastic leukemia.
A total of 548 patients (age range: 1-22 years, 60.4% Hispanic, 55.8% male) diagnosed with acute lymphoblastic leukemia were reviewed for pegaspargase-associated hypersensitivity (14.8%), hyperbilirubinemia (9.7%), venous thromboembolism (VTE, 9.7%), and pancreatitis (5.3%). Odds ratios (OR) and 95% confidence intervals (CI) evaluated associations between clinical factors and each toxicity, cumulative number of toxicities, and toxicity clusters identified using k-mode analysis. Most (68.9%) did not experience any toxicity, 24.6% experienced one toxicity, and 6.3% two or more. Age >10 years was associated with hyperbilirubinemia (OR = 3.83; 95% CI: 1.64-8.95), pancreatitis (OR = 3.72; 95% CI: 1.29-10.68), VTE (OR = 4.65;... (truncated)

MESH:D000077727 - ertapenem

Summary:

LLM Prediction Score	0.171 (normalized)
LLM Confidence Score	0.990
Golden Answer (Severity Class)	0.375 (normalized)
Prediction Error	0.204

Retrieved Context:

Title: Eravacycline: A Review in Complicated Intra-Abdominal Infections.
Eravacycline (Xerava™), a novel fully synthetic fluorocycline, consists of the tetracyclic core scaffold with unique modifications in the tetracyclic D ring; consequently, it exhibits potent in vitro activity against Gram-positive and -negative bacterial strains expressing certain common tetracycline-specific acquired resistance mechanisms. In vitro, eravacycline exhibits potent activity against a broad spectrum of clinically relevant Gram-positive and -negative aerobic and anaerobic bacteria. Intravenous eravacycline is approved in several countries for the treatment of complicated intra-abdominal infections (cIAIs) in adult patients. In two pivotal double-blind, multinational trials in this patient population, eravacycline (infusion ≈ 1 h) was noninferior to intravenous ertapenem or... (truncated)

Title: Frequency, clinical presentation, and outcomes of drug-induced liver injury after liver transplantation.
Drug-induced liver injury (DILI) is increasingly being recognized as a common cause of acute hepatitis. The clinical impact of DILI after liver transplantation (LT) is not known. The aim of this study was to describe the frequency, clinical presentation, and outcomes of DILI in LT recipients. LT recipients with possible DILI were identified with electronic pathology records and clinical note database retrieval tools. Diagnostic criteria were applied to identify cases of DILI. Twenty-nine of 1689 LT recipients (1.7%) were identified with DILI. The mean age was 52 years, and 52% were women. The major indications for LT were primary sclerosing... (truncated)

Title: Investigation and Analysis of the Colonization and Prevalence of Carbapenem-Resistant <i>Enterobacteriaceae</i> in Pediatric Liver Transplant Recipients.
Label="OBJECTIVE" NlmCategory="OBJECTIVE">This study aimed to investigate the colonization and prevalence of carbapenem-resistant <i>Enterobacteriaceae</i> (CRE) in pediatric liver transplant recipients and analyze the high-risk factors and prognosis of CRE infection.

Title: Ertapenem for treatment of osteomyelitis: a case series.
Ertapenem is a once-daily broad spectrum carbapenem that is increasingly used to treat polymicrobial osteomyelitis due to diabetic foot and traumatic wound infections. However, limited data exists on ertapenem use for osteomyelitis. This study aimed to characterize outcomes and adverse effects with empiric use of ertapenem for osteomyelitis.

Title: Single Dose Based Ertapenem Prophylaxis Reduces Surgical Site Infection after Selective Hepatectomy of Hepatocellular Carcinoma: A Propensity Score Matching Study.
This study aimed to assess whether a single dose of ertapenem prophylaxis was more effective than other antibiotics to prevent surgical site infection (SSI) after selective hepatectomy for hepatocellular carcinoma (HCC).

MESH:D003000 - clonidine

Summary:

LLM Prediction Score	0.173 (normalized)
LLM Confidence Score	0.990
Golden Answer (Severity Class)	0.375 (normalized)
Prediction Error	0.202

Retrieved Context:

Title: Aspects of tolerability of centrally acting antihypertensive drugs.
Traditional centrally acting antihypertensives have been associated with a high incidence of adverse effects and are no longer recommended as first-line therapy. The newer imidazoline receptor agonists must overcome this reputation if they are to gain recognition as potential first-line agents for hypertension. Methyldopa, a centrally acting alpha(2)-agonist, is characterized by a number of serious adverse reactions that limit its use. Although unpredictable idiosyncratic or hypersensitivity reactions are uncommon, these include hepatitis, myocarditis, and hemolytic anaemia. Less serious problems such as abnormal liver function tests, positive Coombs test, drug-induced fever, and pancreatitis also occur. Central side effects include drowsiness, fatigue,... (truncated)

Title: Transdermal estradiol priming during clonidine stimulation test in non-growth hormone deficient children with short stature: a pilot study.
The diagnosis of growth hormone (GH) deficiency is strongly influenced by age, body mass index and presence of gonadal steroids. Priming with oral estradiol (E2) is one possible way to overcome the impact of variable levels of sex steroids. We describe the effects of transdermal estradiol (E2-t) priming on GH response after clonidine stimulation in prepubertal children with familial short stature (group 1, n = 12) or constitutional growth delay (group 2, n = 22). All patients underwent a clonidine test (0.1 mg/m2, p.o.) followed by a clonidine plus E2-t test (50 microg/day) with a 7-day interval. Before E2-t, basal... (truncated)

Title: The activation of α₂-adrenergic receptor in the spinal cord lowers sepsis-induced mortality.
The effect of clonidine administered intrathecally (i.t.) on the mortality and the blood glucose level induced by sepsis was examined in mice. To produce sepsis, the mixture of D-galactosamine (GaLN; 0.6 g/10 ml)/lipopolysaccharide (LPS; 27 µg/27 µl) was treated intraperitoneally (i.p.). The i.t. pretreatment with clonidine (5 µg/5 µl) increased the blood glucose level and attenuated mortality induced by sepsis in a dose-dependent manner. The i.t. post-treatment with clonidine up to 3 h caused an elevation of the blood glucose level and protected sepsis-induced mortality, whereas clonidine post-treated at 6, 9, or 12 h did not affect. The pre-treatment with... (truncated)

Title: The effects of novel α₂-adrenoreceptor agonist dexmedetomidine on shivering in patients underwent caesarean section.
Objective: Meperidine used to control shivering during perioperative period has associated side effects. The present study compared the safety of selective α2-adrenoreceptor agonist dexmedetomidine and meperidine for anti-shivering in primiparas after caesarean delivery under combined spinal-epidural anesthesia (CSEA).Methods: 100 primiparas scheduled for caesarean delivery were randomly allocated to dexmedetomidine group (Group D, <i>n</i>=50) and meperidine positive control group (Group M, <i>n</i>=50). Primiparas experienced shivering that continued to cord clamping were treated with dexmedetomidine (0.5 μg/kg) or meperidine (0.5 mg/kg) after cord clamping. The primary outcome measures were incidence of nausea, vomiting, and respiratory depression. Secondary outcome measures were shivering score, vital signs... (truncated)

Title: Case study: adverse response to clonidine.
The use of clonidine alone and in combination to treat a variety of problems has increased in child and adolescent patients. Four cases of adverse experiences with clonidine are described. Clinical guidelines for the use of clonidine in particular and the use of polypharmacy in general are presented.

MESH:D000078308 - tiagabine

Summary:

LLM Prediction Score	0.176 (normalized)
LLM Confidence Score	0.980
Golden Answer (Severity Class)	0.375 (normalized)
Prediction Error	0.199

Retrieved Context:

Title: Choice and use of newer anticonvulsant drugs in older patients.
Epilepsy is common in the elderly. The incidence of epilepsy is age-dependent, with a peak during the first year of life and higher incidence in those older than 75 years. Cerebrovascular disease is a common cause of epilepsy in the elderly. Drug treatment of the elderly is a challenge because of pharmacokinetic changes with aging, including impaired drug protein binding or displacement of drug from protein binding sites, potentially causing drug toxicity as a result of increased free drug concentrations. With aging, hepatic mass and blood flow decline along with renal function. Established anticonvulsant drugs have adverse effects and drug... (truncated)

Title: Use of antiepileptic drugs in hepatic and renal disease.
The use of antiepileptic drugs in patients with renal or hepatic disease is common in clinical practice. Since the liver and kidney are the main organs involved in the elimination of most drugs, their dysfunction can have important effects on the disposition of antiepileptic drugs. Renal or hepatic disease can prolong the elimination of the parent drug or an active metabolite leading to accumulation and clinical toxicity. It can also affect the protein binding, distribution, and metabolism of a drug. The protein binding of anionic acidic drugs, such as phenytoin and valproate, can be reduced significantly by renal failure, causing... (truncated)

Title: [Clinical implications of pharmacology and pharmacokinetics of tiagabine].
Tiagabine is a new antiepileptic drug which acts by blocking neuronal and glial GABA uptake and it is indicated in the treatment of partial epilepsies. Its pharmacokinetics is lineal, being extensively metabolized in the liver by means of CYP3A4 isoenzyme. Plasma elimination half life ranges between 5-8 hours in healthy volunteers, being markedly reduced when the drug is administered concomitantly with enzyme-inducing antinconvulsants. Tiagabine does not induce nor inhibit hepatic enzymes and, consequently, it does not modify the kinetics of simultaneously prescribed antiepileptic drugs. No relevant kinetic differences have been observed between adults and elderly subjects. Renal impairment does not... (truncated)

Title: An Updated Overview on Therapeutic Drug Monitoring of Recent Antiepileptic Drugs.
Given the distinctive characteristics of both epilepsy and antiepileptic drugs (AEDs), therapeutic drug monitoring (TDM) can make a significant contribution to the field of epilepsy. The measurement and interpretation of serum drug concentrations can be of benefit in the treatment of uncontrollable seizures and in cases of clinical toxicity; it can aid in the individualization of therapy and in adjusting for variable or nonlinear pharmacokinetics; and can be useful in special populations such as pregnancy. This review examines the potential for TDM of newer AEDs such as eslicarbazepine acetate, felbamate, gabapentin, lacosamide, lamotrigine, levetiracetam, perampanel, pregabalin, rufinamide, retigabine, stiripentol, tiagabine,... (truncated)

Title: Influence of tiagabine maintenance on cannabis effects and related behaviors in daily cannabis users.
No medications are approved for cannabis use disorder (CUD). Gamma-aminobutyric acid (GABA) reuptake is modulated by cannabinoid (CB) receptor agonists, and there are shared effects between CB agonists and the GABA reuptake inhibitor tiagabine. This overlapping neuropharmacology suggested that tiagabine might be useful for CUD. The study determined the ability of tiagabine maintenance to reduce cannabis self-administration using a placebo-controlled, double-blind, counterbalanced, within-subjects design. Nontreatment-seeking daily cannabis users (N = 12; 3 female, 9 male) completed two 12-day outpatient maintenance phases (0 or 12 mg of tiagabine/day). Each phase consisted of a safety session, 7 maintenance days, and 4 experimental... (truncated)

MESH:C089750 - zolmitriptan

Summary:

LLM Prediction Score	0.177 (normalized)
LLM Confidence Score	0.990
Golden Answer (Severity Class)	0.375 (normalized)
Prediction Error	0.198

Retrieved Context:

Title: Paracetamol (acetaminophen) use in infants and children was never shown to be safe for neurodevelopment: a systematic review with citation tracking.
Although widely believed by pediatricians and parents to be safe for use in infants and children when used as directed, increasing evidence indicates that early life exposure to paracetamol (acetaminophen) may cause long-term neurodevelopmental problems. Furthermore, recent studies in animal models demonstrate that cognitive development is exquisitely sensitive to paracetamol exposure during early development. In this study, evidence for the claim that paracetamol is safe was evaluated using a systematic literature search. Publications on PubMed between 1974 and 2017 that contained the keywords "infant" and either "paracetamol" or "acetaminophen" were considered. Of those initial 3096 papers, 218 were identified that... (truncated)

Title: Hepatobiliary Events in Migraine Therapy with Herbs-The Case of Petadolex, A Petasites Hybridus Extract.
Petadolex®, a defined butterbur extract has clinically proven efficacy against migraine attacks. However, spontaneous reports indicate cases of herbal induced liver injury (HILI). While most HILI patients presented mild serum biochemistry changes (<3 ULN, dose range 50 to 225 mg/day; treatment duration 4-730 days) nine developed severe HILI (average time-to-onset 103 days, ALT-range 3-153; AST 2-104-fold ULN). HILI cases resolved after medication withdrawal though two patients required liver transplantation. Liver biopsies revealed an inconsistent injury pattern, i.e. necrosis, macrovesicular steatosis, inflammation, cholestasis, and bile duct proliferation. Causality assessment rated 3 cases likely, 13 possible, 8 unlikely and 24 as unclassifiable/unclassified.... (truncated)

Title: Targeted CGRP Small Molecule Antagonists for Acute Migraine Therapy.
Migraine is a highly prevalent, severe, and disabling neurological condition with a significant unmet need for effective acute therapies. Patients (~50%) are dissatisfied with their currently available therapies. Calcitonin gene-related peptide (CGRP) has emerged as a key neuropeptide involved in the pathophysiology of migraines. As reviewed in this manuscript, a number of small molecule antagonists of the CGRP receptor have been developed for migraine therapy. Incredibly, the majority of the clinical trials conducted have proven positive, demonstrating the importance of this signalling pathway in migraine. Unfortunately, a number of these molecules raised liver toxicity concerns when used daily for as... (truncated)

Title: Gepants - a long way to cure: a narrative review.
Calcitonin gene-related peptide (CGRP) is probably the most potent vasodilator in cerebral circulation. Forty years after its discovery, the new CGRP-targeted therapy monoclonal antibodies, and the small molecule gepants, are now available for clinical practice. While randomized controlled trials and real-world experience consistently demonstrated the high efficacy and tolerability of monoclonal antibodies, limited evidence is available to characterize gepants fully. Depending on pharmacokinetics, these CGRP receptor antagonists can be used for acute (ubrogepant, rimegepant, and the not yet approved zavegepant) or preventive (atogepant and rimegepant) migraine treatment. Randomized placebo-controlled trials demonstrated gepants efficacy in treating acute attacks to obtain 2... (truncated)

Title: Efficacy of frovatriptan as compared to other triptans in migraine with aura.
The treatment of migraine attacks with aura by triptans is difficult since triptans most probably are not efficacious when taken during the aura phase. Moreover, there are insufficient data from randomised studies whether triptans are efficacious in migraine attacks with aura when taken during the headache phase. In this metaanalysis, we aimed to compare the efficacy of frovatriptan versus rizatriptan, zolmitriptan, and almotriptan.

MESH:D011139 - polythiazide

Summary:

LLM Prediction Score	0.055 (normalized)
LLM Confidence Score	0.750
Golden Answer (Severity Class)	0.25 (normalized)
Prediction Error	0.195

Retrieved Context:

Title: Data-driven identification of structural alerts for mitigating the risk of drug-induced human liver injuries.
The use of structural alerts to de-prioritize compounds with undesirable features as drug candidates has been gaining in popularity. Hundreds of molecular structural moieties have been proposed as structural alerts. An emerging issue is that strict application of these alerts will result in a significant reduction of the chemistry space for new drug discovery, as more than half of the oral drugs on the market match at least one of the alerts. To mitigate this issue, we propose to apply a rigorous statistical analysis to derive/validate structural alerts before use.

Title: Indapamide-Induced Rhabdomyolysis: An Evaluation of Case Reports in VigiBase Using the Bradford Hill Criteria.
Indapamide can cause hypokalaemia and hyponatraemia. Rhabdomyolysis associated with these electrolyte abnormalities has been reported.

Title: 20th ISoP Annual Meeting "Integrated pharmacovigilance for safer patients" 8-10 November 2021 Muscat, Oman (Hybrid meeting).
No abstract available.

Title: The frequency of photosensitizing drug dispensings in Austria and Germany: a correlation with their photosensitizing potential based on published literature.
Drug-induced photosensitivity refers to the development of cutaneous adverse events due to interaction between a pharmaceutical compound and sunlight. Although photosensitivity is a very commonly listed side-effect of systemic drugs, reliable data on its actual incidence are lacking so far.

MESH:D000077266 - moxifloxacin

Summary:

LLM Prediction Score	0.806 (normalized)
LLM Confidence Score	0.990
Golden Answer (Severity Class)	1.0 (normalized)
Prediction Error	0.194

Retrieved Context:

Title: Moxifloxacin induced fatal hepatotoxicity in a 72-year-old man: a case report.
Moxifloxacin is a newer-generation synthetic fluoroquinolone that is used for treatment of acute bacterial sinusitis, acute exacerbation of chronic bronchitis, community acquired pneumonia, intra-abdominal infections and skin/skin structure infections. We describe a case of fatal hepatotoxicity caused by Moxifloxacin in a 72-year-old man. He presented with jaundice and epigastric tenderness that started one week after being treated for acute exacerbation of his chronic bronchitis with Moxifloxacin by his primary care physician. He was admitted to intensive care unit for close monitoring. His labs showed marked elevation in liver enzymes and bilirubin. His condition continued to deteriorate in intensive care unit... (truncated)

Title: Fluoroquinolone therapy and idiosyncratic acute liver injury: a population-based study.
Although fluoroquinolones are sometimes associated with mild, transient elevations in aminotransferase levels, serious acute liver injury is uncommon. Regulatory warnings have identified moxifloxacin as presenting a particular risk of hepatotoxicity. Thus, we examined the risk of idiosyncratic acute liver injury associated with the use of moxifloxacin relative to other selected antibiotic agents.

Title: Dose- and time-dependent manners of moxifloxacin induced liver injury by targeted metabolomics study.
Moxifloxacin is the most widely prescribed antibiotics due to its excellent oral bioavailability and broad-spectrum antibacterial effect. Despite of its popularity, the rare and severe liver injury induced by moxifloxacin is a big concern that cannot be ignored in clinical practice. However, the early warning and related metabolic disturbances of moxifloxacin induced hepatoxicity were rarely reported. In this study, the dose- and time-dependent manners of moxifloxacin induced liver injury were investigated by a targeted metabolomics method. In dose-dependent experiment, three different dosages of moxifloxacin were administered to the rats, including 36 mg kg⁻¹ d⁻¹, 72 mg kg⁻¹ d⁻¹, and 108 mg kg⁻¹ d⁻¹. In time-dependent experiment,... (truncated)

Title: Moxifloxacin Induced Liver Injury by Causing <i>Lachnospiraceae</i> Deficiency and Interfering with Butyric Acid Production through Gut-Liver Axis.
Cases of unpredictable, idiosyncratic liver damage of moxifloxacin (MXF) have been occasionally reported. However, the health effects of MXF exposure remain controversial. The current study examined the metabolic phenotypes and intestinal flora characteristics of hepatotoxicity induced by MXF. Rats were administered moxifloxacin hydrochloride tablets at doses of 36, 72, and 108 mg/kg body weight/day for 21 days. The levels of tricarboxylic acid cycle intermediates were decreased, whereas those of lipids (arachidonic acid, hexadecanoic acid, and linoleic acid) were increased, reflecting disorders of energy-related and lipid metabolism. Enrichment analysis of the differential metabolites suggested that butanoate metabolism was associated with MXF-induced liver... (truncated)

Title: [Novel side effects of moxifloxacin: making a balanced decision again].
Earlier this year, a 'Dear Doctor' letter was sent to Dutch health care professionals, describing the rare occurrence of fulminant hepatitis and the Stevens-Johnson syndrome or toxic epidermal necrolysis in patients using moxifloxacin. This resulted in media attention, questions in parliament and moxifloxacin being banned from the formulary in several hospitals. Was this reaction justified? In the Netherlands, moxifloxacin is only mentioned in the practice guideline on the treatment of severe community-acquired pneumonia. Alternatives for moxifloxacin for this indication are penicillin combined with ciprofloxacin, or cephalosporins in combination with erythromycin. The associated risks, in particular fatal anaphylaxis and sudden cardiac... (truncated)

MESH:D006857 - hydroflumethiazide

Summary:

LLM Prediction Score	0.060 (normalized)
LLM Confidence Score	0.580
Golden Answer (Severity Class)	0.25 (normalized)
Prediction Error	0.190

Retrieved Context:

Title: Investigating Core Signaling Pathways of Hepatitis B Virus Pathogenesis for Biomarkers Identification and Drug Discovery via Systems Biology and Deep Learning Method.
Hepatitis B Virus (HBV) infection is a major cause of morbidity and mortality worldwide. However, poor understanding of its pathogenesis often gives rise to intractable immune escape and prognosis recurrence. Thus, a valid systematic approach based on big data mining and genome-wide RNA-seq data is imperative to further investigate the pathogenetic mechanism and identify biomarkers for drug design. In this study, systems biology method was applied to trim false positives from the host/pathogen genetic and epigenetic interaction network (HPI-GEN) under HBV infection by two-side RNA-seq data. Then, via the principal network projection (PNP) approach and the annotation of KEGG (Kyoto... (truncated)

Title: 20th ISoP Annual Meeting "Integrated pharmacovigilance for safer patients" 8-10 November 2021 Muscat, Oman (Hybrid meeting).
No abstract available.

Title: The frequency of photosensitizing drug dispensings in Austria and Germany: a correlation with their photosensitizing potential based on published literature.
Drug-induced photosensitivity refers to the development of cutaneous adverse events due to interaction between a pharmaceutical compound and sunlight. Although photosensitivity is a very commonly listed side-effect of systemic drugs, reliable data on its actual incidence are lacking so far.

MESH:D006830 - hydralazine

Summary:

LLM Prediction Score	0.565 (normalized)
LLM Confidence Score	0.990
Golden Answer (Severity Class)	0.375 (normalized)
Prediction Error	0.190

Retrieved Context:

Title: A Suspected Case of Hydralazine-Induced Hepatotoxicity: A Case Report and Review of Literature.
BACKGROUND Hydralazine is an effective antihypertensive agent but may rarely have devastating hepatotoxic effects that are extremely variable, thus making the diagnosis difficult. CASE REPORT We report the case of a 74-year-old male patient who had transaminitis after being started on hydralazine by his cardiologist for poorly controlled hypertension. He had extreme dizziness, nausea, and weakness, which all resolved after discontinuation of hydralazine, and liver function test results also dramatically improved. CONCLUSIONS It is imperative that clinicians be aware of the possible hepatotoxicity of hydralazine and its clinical features so that the medication can be promptly discontinued to help promote... (truncated)

Title: Acute Cholestatic Liver Injury From Hydralazine Intake.
Hydralazine is a commonly used oral antihypertensive agent. We report a rare case of hydralazine-induced hepatotoxicity in the form of subacute hepatic necrosis. A 75-year-old African American woman presented with jaundice of 7-day duration. She was started on hydralazine 100 mg 3 times a day 10 weeks before presentation. On physical examination, scleral icterus was noted. Workup revealed elevated liver transaminases, alkaline phosphatase, and conjugated bilirubin. She had no history of liver disease, and liver function tests had been normal before starting hydralazine. Other etiologies, including viruses, common toxins, drugs, autoimmune, and copper-induced hepatitis, were excluded. Abdominal imaging studies did... (truncated)

Title: Hydralazine-induced cholestatic hepatitis.
Hydralazine has been widely used for treating hypertension, particularly in patients with renal failure. We report a case on a patient in whom we believe the drug was implicated in an otherwise unexplained disturbance of liver function. A 63-year-old African-American female with medical history of hypertension and end-stage renal disease (on hemodialysis) was admitted to the hospital with epigastric pain and jaundice. The symptoms started about 1 week ago. Initial laboratory tests showed abnormal liver enzymes with elevated conjugated bilirubin and alkaline phosphatase suggestive of cholestatic jaundice. Amylase and lipase were normal. Abdominal ultrasound showed normal caliber common bile duct... (truncated)

Title: A One-Two Punch: Hydralazine-Induced Liver Injury in a Recovering Ischemic Hepatitis.
A 77-year-old woman presented to the emergency department with a 2-day history of nausea and vomiting. Her medical history included diabetes mellitus, hypertension, atrial fibrillation, dilated cardiomyopathy, and coronary artery disease. Her home medications included aspirin, clopidogrel, warfarin, digoxin, metoprolol, losartan, simvastatin, isosorbide dinitrate, furosemide, and spironolactone. Initial physical examination showed blood pressure of 170/80 mm Hg with a heart rate of 69 beats per minute, otherwise unremarkable. Initial laboratory workup was significant for INR of 3.6, with slightly elevated troponin I and creatinine of 0.06 ng/mL and 1.4 mg/dL, respectively. The patient was admitted to the medicine floor. However,... (truncated)

Title: Hydralazine-induced liver injury: a review and discussion.
Hydralazine is a commonly prescribed antihypertensive agent. Some of its labelled adverse reactions include lupus-like syndrome, tachycardia, headache and fever. Despite its well-known side effects, little is known about hydralazine's hepatotoxic effects. We report the case of a 54-year-old female patient who was started on hydralazine for hypertension management but later presented with hydralazine-induced liver injury. Her initial presentation consisted of non-specific symptoms and a hepatocellular injury pattern. Liver biopsy revealed hepatic steatosis. Three weeks after discontinuation of hydralazine, the patient's liver enzymes normalised, and her symptoms resolved. Few studies have examined the incidence and mechanism by which hydralazine induces... (truncated)

MESH:D001279 - atracurium

Summary:

LLM Prediction Score	0.189 (normalized)
LLM Confidence Score	0.980
Golden Answer (Severity Class)	0.0 (normalized)
Prediction Error	0.189

Retrieved Context:

Title: Atracurium and severe hepatic disease: a case report.
Atracurium is a new non-depolarising neuromuscular blocking agent, metabolized through Hofmann elimination. A case is presented in which a 45-year-old patient with severe liver disease showed a decreased response to atracurium. The possible causes of resistance to atracurium are discussed. We conclude that atracurium may be used in patients with severe hepatic disease.

Title: Recent advances in neuromuscular blocking agents.
Factors driving the development of neuromuscular blocking agents are discussed. The goal of recent development of neuromuscular blocking agents is to develop agents with fewer adverse effects than succinylcholine and greater control. Greater control can be achieved through a short duration of action and a fast onset, similar to that found with succinylcholine. Duration control can be achieved through rapid, reliable metabolism that is organ independent, as with cisatracurium, or that occurs in the liver, because this will fail only in patients with severe hepatic disease. Rapid onset can be achieved by giving higher doses of drugs that have a... (truncated)

Title: Pharmacokinetics of atracurium and its metabolites.
The pharmacokinetic profile of atracurium was studied in normal patients and in patients with renal failure, renal-hepatic failure, or hepatic disease. Its short elimination half-life was not significantly altered by renal failure, but in patients with severe liver disease elimination of its metabolites was prolonged, necessitating care during long-term i.v. infusions in patients with hepatic dysfunction.

Title: [Resistance to non-depolarizing myorelaxants. Our experience with 3 clinical cases].
The Authors report their own experience in three patients that showed reduced sensitivity to atracurium or vecuronium. Two patients were affected by neoplastic diseases and one by hand trauma. Doses of 0.5 mg/kg of atracurium and 0.08 mg/kg of vecuronium were unable to establish a complete neuromuscular blockade. The Authors review the main clinical situations in which resistance to non-depolarizing muscle relaxants is known: burns, hepatic diseases, chronic therapy with anticonvulsant drugs. Possibly, such events are due to fast elimination of the drug, or to an increased number of acetylcholine nicotinic receptors, or to an unknown acutely acting circulating factor,... (truncated)

Title: Atracurium and vecuronium: two unique neuromuscular blocking agents.
Atracurium and vecuronium are two new nondepolarizing skeletal muscle relaxants that were developed to overcome the deficiencies seen with currently available agents (tubocurarine, metocurine, pancuronium, and gallamine). Both compounds have unique metabolic profiles, separating them from other nondepolarizing agents. Neither drug depends on normal renal function for excretion and each can safely be given to patients with renal failure. Atracurium also does not depend on hepatic function for metabolism; however, vecuronium may require dosing adjustments in hepatic disease. Atracurium and vecuronium have similar onset times for muscle relaxation but shorter durations of action than other nondepolarizing muscle relaxants. Both agents... (truncated)

MESH:D002077 - butorphanol

Summary:

LLM Prediction Score	0.187 (normalized)
LLM Confidence Score	0.980
Golden Answer (Severity Class)	0.0 (normalized)
Prediction Error	0.187

Retrieved Context:

Title: Butorphanol suppresses fentanyl-induced cough during general anesthesia induction: A randomized, double-blinded, placebo-controlled clinical trial.
Fentanyl-induced cough (FIC) is unwanted in the patients requiring stable induction of general anesthesia. This study was designed to evaluate the suppressive effects of butorphanol pretreatment on the incidence and severity of FIC during the induction of general anesthesia. A total of 315 patients of American Society of Anesthesiologists physical status I and II, scheduled for elective surgery under general anesthesia were randomized into 3 equally sized groups (n = 0105). Two minutes before fentanyl bolus, group I received intravenously 5 mL normal saline, groups II and III received butorphanol 0.015 and 0.03 mg/kg (diluted with saline to 5 mL),... (truncated)

Title: The absolute bioavailability and pharmacokinetics of butorphanol nasal spray in patients with hepatic impairment.
The objective of the study was to investigate the effects of hepatic impairment on the absolute transnasal bioavailability and pharmacokinetics of butorphanol.

Title: Chronic intake of high-dose of blueberry leaf extract does not augment the harmful effects of ethanol in rats.
Excessive alcohol consumption is a risk factor for liver diseases. Enhancement of alcohol metabolism could be an effective strategy to prevent these adverse effects since it promotes the clearance of ethanol and acetaldehyde from the serum. Polyphenol-rich products have shown to protect against alcohol-related liver damage. Blueberry leaves have attracted attention as they are rich polyphenols such as proantocyanidins and chlorogenic acid. In this study, we investigated the effects of a high dose of blueberry leaf extract (BLEx) on alcohol metabolism during chronic intake of ethanol. Seven-week old Sprague-Dawley (SD) rats were divided into four groups: normal liquid diet group... (truncated)

Title: Opioid Use Is More Common in Nonalcoholic Fatty Liver Disease Patients with Cirrhosis, Higher BMI, and Psychiatric Disease.
Opioid use is a topic of growing concern among patients with nonalcoholic fatty liver disease (NAFLD). Given safety concerns of opioids, proactively identifying subgroups of patients with an increased probability of opioid use may encourage practitioners to recommend alternative therapies for pain, thus reducing the likelihood of opioid misuse. This work assessed the prevalence and patient characteristics associated with opioid use in a real-world cohort of patients with NAFLD.

Title: The 3Rs in Experimental Liver Disease.
Patients with cirrhosis present multiple physiological and immunological alterations that play a very important role in the development of clinically relevant secondary complications to the disease. Experimentation in animal models is essential to understand the pathogenesis of human diseases and, considering the high prevalence of liver disease worldwide, to understand the pathophysiology of disease progression and the molecular pathways involved, due to the complexity of the liver as an organ and its relationship with the rest of the organism. However, today there is a growing awareness about the sensitivity and suffering of animals, causing opposition to animal research among a... (truncated)

MESH:D000077595 - famciclovir

Summary:

LLM Prediction Score	0.188 (normalized)
LLM Confidence Score	0.980
Golden Answer (Severity Class)	0.375 (normalized)
Prediction Error	0.187

Retrieved Context:

Title: Clinical potential of emerging new agents in hepatitis B.
Treatment of chronic hepatitis B is directed at interrupting the natural history and clinical outcomes of the disease. It needs to take into account the virology and replication cycle of the hepatitis B virus (HBV), and the host immune response to HBV. Long term follow-up of patients treated with interferon supports the paradigm that a sustained, major suppression of HBV replication, particularly that associated with hepatitis B e antigen (HBeAg) seroconversion, interrupts the natural history of hepatitis B. The availability of potent but well tolerated and orally available HBV antivirals, of which lamivudine is the prototype, has allowed clearer treatment... (truncated)

Title: Therapy for chronic hepatitis B. Present status.
Up to now Interferon (IFN) has been the only licensed treatment for chronic type B and D viral hepatitis. However, IFN monotherapy is efficacious only in HBeAg positive chronic hepatitis B and is aggravated by important side effects in many patients. To overcome the limits of IFN monotherapy, combination therapies of this cytokine together with other synergistic drugs have been proposed and many antivirals that directly act on Hepatitis B Virus (HBV) synthesis have been developed. Combination therapies with acyclovir, levamisole, and cortisone have not been more advantageous than IFN alone. Of the antivirals, Adenine Arabinoside monophosphate, though active against... (truncated)

Title: Liver transplantation for chronic viral liver disease.
Liver transplantation (LTx) for chronic viral liver disease has evolved rapidly during the last two decades. The major problem in cases of LTx for viral hepatitis is the extremely high rate of recurrent viral infection in the liver allograft. While recurrent hepatitis C virus (HCV) infection typically causes a mild hepatitis and has a slow progression, hepatitis B virus (HBV) infection of the liver allograft has been reported to result in cirrhosis in as short a period of time as 1 year. The risk of graft infection is greatest for patients with actively replicating virus. The high rate of disease... (truncated)

Title: Hepatitis B and C viruses: molecular identification and targeted antiviral therapies.
Four agents are in clinical development for the treatment of chronic hepatitis B infection. These nucleoside analogs are incorporated into the growing DNA chain and terminate replication. Lamivudine, a cytadine analog that inhibits the synthesis of negative strand DNA from pre-genomic RNA, predictably inhibits replication and improves liver enzymes and histology in infected individuals. Following cessation of treatment, relapse is common, and genetic causes of viral resistance have been described. Other drugs for HBV infection include famciclovir, a guanosine analog that has also shown to suppress replication in immunocompetent as well as in immunocompromised patients; lobucavir, a guanosine analog; and... (truncated)

Title: Perspectives for the treatment of hepatitis B virus infections.
Primarily resulting as a spin-off of the search for effective anti-HSV or anti-HIV agents, several compounds have been identified as effective and promising candidate anti-HBV drugs, i.e. famciclovir (penciclovir), BMS-200475, lamivudine (3TC), (-)FTC, L(-)Fd4C, L-FMAU, DAPD (DXG), bis(POM)-PMEA and bis(POC)-PMPA. They all inhibit HBV replication in Hep G2 2.2.15 at concentrations that are well below the cytotoxicity threshold. All these nucleoside analogues require three phosphorylation steps to be active, in their triphosphate form, as inhibitors of the HBV DNA polymerase, except for PMEA (adefovir) and PMPA (tenofovir), which need only two phosphorylation steps, to PMEApp and PMPApp, respectively, to interact... (truncated)

MESH:D013693 - temazepam

Summary:

LLM Prediction Score	0.189 (normalized)
LLM Confidence Score	0.990
Golden Answer (Severity Class)	0.375 (normalized)
Prediction Error	0.186

Retrieved Context:

Title: Feline hepatic biotransformation of diazepam: Differences between cats and dogs.
In contrast to humans and dogs, diazepam has been reported to induce severe hepatic side effects in cats, particularly after repeated dosing. With the aim to elucidate the mechanisms underlying this apparent sensitivity of cats to drug-induced liver injury, in a series of in vitro experiments, the feline-specific biotransformation of diazepam was studied with liver microsomes obtained from cats and dogs and the possible inhibition of the bile salt export pump (Bsep) was measured in isolated membrane vesicles overexpressing feline and canine Bsep. In line with previous in vivo studies, the phase I metabolites nordiazepam, temazepam and oxazepam were measurable... (truncated)

Title: Psychotropic drugs and liver disease: A critical review of pharmacokinetics and liver toxicity.
The liver is the organ by which the majority of substances are metabolized, including psychotropic drugs. There are several pharmacokinetic changes in end-stage liver disease that can interfere with the metabolization of psychotropic drugs. This fact is particularly true in drugs with extensive first-pass metabolism, highly protein bound drugs and drugs depending on phase I hepatic metabolic reactions. Psychopharmacological agents are also associated with a risk of hepatotoxicity. The evidence is insufficient for definite conclusions regarding the prevalence and severity of psychiatric drug-induced liver injury. High-risk psychotropics are not advised when there is pre-existing liver disease, and after starting a... (truncated)

Title: Detection of nanolevel drug metabolites in an organotypic culture of primary human hepatocytes and porcine hepatocytes with special reference to a two-compartment model.
The quantification of drug metabolites produced during drug metabolism is a growing concern for the pharmaceutical industry, regulatory agencies such as the US Food and Drug Administration, the European Medicines Agency, and others. As 70% of drugs are known reactive metabolites and have black box warnings, they are a major cause of drug-induced injury and lead to drug attrition in early or late clinical stages. According to a 2006 survey report of pharmaceutical companies, drug-induced liver injury was ranked first in terms of adverse events, and it remains the most common reason for restriction or withdrawal of a drug from... (truncated)

Title: Detection of Acetaminophen-Protein Adducts in Decedents with Suspected Opioid-Acetaminophen Combination Product Overdose.
Acetaminophen overdose is a leading cause of drug-induced liver failure in the United States. Acetaminophen-protein adducts have been suggested as a biomarker of hepatotoxicity. The purpose of this study was to determine whether protein-derived acetaminophen-protein adducts are quantifiable in postmortem samples. Heart blood, femoral blood, and liver tissue were collected at autopsy from 22 decedents suspected of opioid-acetaminophen overdose. Samples were assayed for protein-derived acetaminophen-protein adducts, acetaminophen, and selected opioids found in combination products containing acetaminophen. Protein-derived APAP-CYS was detected in 17 of 22 decedents and was measurable in blood that was not degraded or hemolyzed. Heart blood concentrations ranged... (truncated)

Title: Aliphatic Halogenated Hydrocarbons: Report and Analysis of Liver Injury in 60 Patients.
<i>Background and Aims:</i> Intoxications by aliphatic halogenated hydrocarbons (AHH), used as effective solvents, are rare and may cause life-threatening liver injury. Patients with acute intoxications by AHH received an innovative treatment. <i>Methods:</i> Analyzed were data of 60 patients intoxicated by AHH, such as dichloromethane (<i>n</i> = 3), chloroform (<i>n</i> = 2), carbon tetrachloride (<i>n</i> = 12), 1,2-dichloroethane (<i>n</i> = 18), 1,1,2-trichloroethane (<i>n</i> = 2), trichloroethylene (<i>n</i> = 2), tetrachloroethylene (<i>n</i> = 13) or mixed AHH chemicals (<i>n</i> = 8), who received a new treatment consisting of CO₂-induced hyperventilation to accelerate toxin removal via the lungs. <i>Results:</i> Added to the inspiration... (truncated)

MESH:D000077339 - leflunomide

Summary:

LLM Prediction Score	0.814 (normalized)
LLM Confidence Score	0.980
Golden Answer (Severity Class)	1.0 (normalized)
Prediction Error	0.186

Retrieved Context:

Title: Reappraisal of the clinical use of leflunomide in rheumatoid arthritis and psoriatic arthritis.
Leflunomide is a disease-modifying antirheumatic drug (DMARD) that has been in routine clinical use for the treatment of rheumatoid arthritis (RA) and psoriatic arthritis for a decade. In RA, clinical trials of up to two years' duration showed that leflunomide monotherapy was equivalent to methotrexate in clinical and radiographic disease outcomes (tender and swollen joint counts, physician and patient global assessments, American College of Rheumatology and Disease Activity Score responses, slowing or halting of radiographic progression). In a number of studies, quality of life measurements indicated that leflunomide is superior to methotrexate. Leflunomide has been studied in combination with methotrexate... (truncated)

Title: Inhibition of hepatic cytochrome P450 enzymes and sodium/bile acid cotransporter exacerbates leflunomide-induced hepatotoxicity.
Leflunomide is an immunosuppressive agent marketed as a disease-modifying antirheumatic drug. But it causes severe side effects, including fatal hepatitis and liver failure. In this study we investigated the contributions of hepatic metabolism and transport of leflunomide and its major metabolite teriflunomide to leflunomide induced hepatotoxicity in vitro and in vivo.

Title: Leflunomide-induced acute hepatitis.
Leflunomide, a new immunomodulatory agent, was prescribed to a 67-year-old female patient with rheumatoid arthritis. Fifteen days later she developed diarrhoea and elevated liver enzymes. A liver biopsy showed a pattern of acute hepatitis. The patient was homozygous for the rare CYP2C9*3 allele, which determines the slowest metabolic rate for CYP2C9 enzymatic activity, that is probably involved in the metabolism of leflunomide. Liver damage subsided in few weeks. This case illustrates the risk of hepatotoxicity by leflunomide and suggests that it is possibly related to CYP2C9 polymorphism.

Title: Mitochondrial dysfunction induced by leflunomide and its active metabolite.
Leflunomide, an anti-inflammatory drug used for the treatment of rheumatoid arthritis, has been marked with a black box warning regarding an increased risk of liver injury. The active metabolite of leflunomide, A771726, which also carries a boxed warning about potential hepatotoxicity, has been marketed as teriflunomide for the treatment of relapsing multiple sclerosis. Thus far, however, the mechanism of liver injury associated with the two drugs has remained elusive. In this study, cytotoxicity assays showed that ATP depletion and subsequent LDH release were induced in a time- and concentration-dependent manner by leflunomide in HepG2 cells, and to a lesser extent,... (truncated)

Title: Thrombotic microangiopathy and liver toxicity due to a combination therapy of leflunomide and methotrexate: a case report.
Treatment of active rheumatoid arthritis may necessitate a methotrexate mono- or combination therapy. As in the present case, novel side effects may occur, when escalating therapy.

MESH:D008727 - methotrexate

Summary:

LLM Prediction Score	0.560 (normalized)
LLM Confidence Score	0.990
Golden Answer (Severity Class)	0.375 (normalized)
Prediction Error	0.185

Retrieved Context:

Title: Risk of liver disease in methotrexate treated patients.
Methotrexate is the first line drug treatment for a number of rheumatic and non-rheumatic diseases. It is effective in controlling disease activity and preventing disease-related damage, and significantly cheaper than many alternatives. Use in rheumatoid arthritis infers a significant morbidity and mortality benefit. Methotrexate is generally well tolerated but can cause symptomatic adverse events. Multiple serious adverse events have been attributed to methotrexate, based largely on older reports using high or daily doses, and subsequent case reports and circumstantial evidence. The risk with modern dosing regimens: Lower doses, weekly schedules, and concomitant folic acid is less clear. Clarification and dissemination... (truncated)

Title: Pre-administration of turmeric prevents methotrexate-induced liver toxicity and oxidative stress.
Methotrexate (MTX) is an antimetabolite broadly used in treatment of cancer and autoimmune diseases. MTX-induced hepatotoxicity limits its application. We investigated hepatoprotective effects of turmeric in MTX-induced liver toxicity.

Title: [The liver and methotrexate].
Methotrexate is proposed for the treatment of inflammatory disorders such as rheumatoid arthritis, psoriasis and Crohn's disease. The liver toxicity of methotrexate has been investigated and prolonged treatment can induce liver fibrosis. Moreover, alcohol consumption, diabetes and obesity are associated with liver fibrosis in patients treated with this drug. Therefore, liver fibrosis associated with methotrexate could be due to associated factors instead of methotrexate itself. Recommendations to monitor and diagnose methotrexate induced liver damage vary depending on the disease. Frequent evaluation of liver fibrosis with liver biopsy is recommended during therapy, especially in patients treated for psoriasis. Noninvasive methods, such... (truncated)

Title: Cumulative dose and length of methotrexate treatment were not shown to be predictors of hepatic fibrosis by elastography - a monocentric cohort study.
Methotrexate is used in several inflammatory diseases, such as rheumatoid arthritis (RA), spondyloarthritis (SpA) or inflammatory bowel disease (IBD). There has been some controversy regarding methotrexate liver toxicity, especially since the use of newer techniques. We aim to evaluate the prevalence of liver injury in methotrexate-treated patients with inflammatory diseases.

Title: Methotrexate-associated liver toxicity in a patient with breast cancer: case report and literature review.
A patient with breast cancer developed severe asthenia, accompanied with progressively increasing transaminases, during adjuvant chemotherapy with CMF (cyclophosphamide, methotrexate and 5-fluorouracil). Additional blood tests and imaging were negative. A liver biopsy revealed a grade II toxic hepatitis. Because methotrexate was suspected to be the cause of the hepatotoxicity, the administration of this drug was stopped and mitoxantrone was given instead. A recovery of clinical symptoms and normalisation of the liver function tests was observed afterwards. In that sense, mitoxantrone appears to be a valuable alternative to methotrexate in cases of hepatotoxicity in patients with breast cancer. An overview of... (truncated)

MESH:D014299 - trimipramine

Summary:

LLM Prediction Score	0.434 (normalized)
LLM Confidence Score	0.950
Golden Answer (Severity Class)	0.25 (normalized)
Prediction Error	0.184

Retrieved Context:

Title: Imipramine Accelerates Nonalcoholic Fatty Liver Disease, Renal Impairment, Diabetic Retinopathy, Insulin Resistance, and Urinary Chromium Loss in Obese Mice.
Imipramine is a tricyclic antidepressant that has been approved for treating depression and anxiety in patients and animals and that has relatively mild side effects. However, the mechanisms of imipramine-associated disruption to metabolism and negative hepatic, renal, and retinal effects are not well defined. In this study, we evaluated C57BL6/J mice subjected to a high-fat diet (HFD) to study imipramine's influences on obesity, fatty liver scores, glucose homeostasis, hepatic damage, distribution of chromium, and retinal/renal impairments. Obese mice receiving imipramine treatment had higher body, epididymal fat pad, and liver weights; higher serum triglyceride, aspartate and alanine aminotransferase, creatinine, blood urea... (truncated)

Title: Drug-Induced Liver Injury during Antidepressant Treatment: Results of AMSP, a Drug Surveillance Program.
Drug-induced liver injury is a common cause of liver damage and the most frequent reason for withdrawal of a drug in the United States. The symptoms of drug-induced liver damage are extremely diverse, with some patients remaining asymptomatic.

Title: The Importance of Patient-Specific Factors for Hepatic Drug Response and Toxicity.
Responses to drugs and pharmacological treatments differ considerably between individuals. Importantly, only 50%-75% of patients have been shown to react adequately to pharmacological interventions, whereas the others experience either a lack of efficacy or suffer from adverse events. The liver is of central importance in the metabolism of most drugs. Because of this exposed status, hepatotoxicity is amongst the most common adverse drug reactions and hepatic liabilities are the most prevalent reason for the termination of development programs of novel drug candidates. In recent years, more and more factors were unveiled that shape hepatic drug responses and thus underlie the... (truncated)

Title: Antidepressant Drugs Effects on Blood Pressure.
Individuals suffering from depressive disorders display a greater incidence of hypertension compared with the general population, despite reports of the association between depression and hypotension. This phenomenon may depend, at least in part, on the use of antidepressant drugs, which may influence blood pressure through different effects on adrenergic and serotoninergic pathways, as well as on histaminergic, dopaminergic, and cholinergic systems. This review summarizes extant literature on the effect of antidepressant drugs on blood pressure. Selective serotonin reuptake inhibitors are characterized by limited effects on autonomic system activity and a lower impact on blood pressure. Thus, they represent the safest... (truncated)

Title: Risk factors for idiosyncratic drug-induced liver injury.
Idiosyncratic drug-induced liver injury (DILI) is a rare disorder that is not related directly to dosage and little is known about individuals who are at increased risk. There are no suitable preclinical models for the study of idiosyncratic DILI and its pathogenesis is poorly understood. It is likely to arise from complex interactions among genetic, nongenetic host susceptibility, and environmental factors. Nongenetic risk factors include age, sex, and other diseases (eg, chronic liver disease or human immunodeficiency virus infection). Compound-specific risk factors include daily dose, metabolism characteristics, and propensity for drug interactions. Alcohol consumption has been proposed as a risk... (truncated)

MESH:C017136 - clometacin

Summary:

LLM Prediction Score	0.817 (normalized)
LLM Confidence Score	0.780
Golden Answer (Severity Class)	1.0 (normalized)
Prediction Error	0.183

Retrieved Context:

Title: [Hepatitis caused by clometacin (Dupéran). Retrospective study of 30 cases. A model of autoimmune drug-induced hepatitis?].
Thirty cases of clometacin-induced hepatitis were retrospectively collected over a nine-year period in hepatogastroenterological units of non university, public hospitals. There was a strong female predominance (90 percent). Clometacin (Dupéran) was taken because of arthritis in 8 out of 10 cases. Administration was continuous in 85 percent of cases and median duration was 445 days. median dose was 450 mg per day. Jaundice, fatigue, and weight loss were the most frequent symptoms, but edema, ascites and palmar erythema were not uncommon. Thrombopenia (38 percent) was the most frequent hematologic abnormality. Renal failure, always with benign course, was present in 1/4... (truncated)

Title: [Liver damage following clomethacin treatment. 6 cases, including 2 deaths (author's transl)].
The authors report on 6 cases of severe liver damage following clomethacin treatment. Three patients developed prolonged hepatitis, lasting more than 3 months, with biochemical and histological signs of activity; one patient committed suicide by overdosage and died of subacute necrotizing hepatitis; one patient died of chronic cirrhotic hepatitis after prolonged clomethacin-alpha-methyldopa combined treatment, and there was one case of active hepatitis on latent cirrhosis. Drug toxicity was suggested by the presence of jaundice with occasional fever, urticaria or pruritus and eosinophilia, and by the lack of any other cause. It was confirmed in 3 cases by relapse of the... (truncated)

Title: Chronic active hepatitis and giant multinucleated hepatocytes in adults treated with clometacin.
The authors report the cases of 2 adults who became jaundiced during prolonged administration of clometacin, a new analgesic drug. Jaundice and serum aminotransferase activity progressively increased while the drug administration was continued but quickly decreased when it was eventually interrupted. 1 patient resumed the intake of clometacin and died with jaundice and ascites. In both patients, liver lesions were those of severe chronic active hepatitis with numerous giant multinucleated hepatocytes.

Title: [Chronic active hepatitis associated with anti-native DNA antibodies: incidence of drug etiology].
Of 75 patients with HBsAg negative chronic active hepatitis (CAH), 28 had antinuclear antibodies in their serum. We have tested these patients' sera for serum antibodies against double stranded (native) DNA (anti-ds-DNA), by immunofluorescence with Crithidia luciliae as substrate. They were found in 14 patients (50 p. 100). Thirteen of the patients with anti-ds-DNA and 11 of those without were female; the mean ages were 64 +/- 16 and 56 +/- 19 years, respectively. The clinical and biological signs seemed to be more severe in patients with anti-ds-DNA than in those without. Liver histological activity and frequency of cirrhosis (about... (truncated)

Title: [Clometacine hepatitis. 2 cases (author's transl)].
Two women (aged 72 and 78 years) developed hepatitis due to clometacine. The main laboratory abnormalities were raised transaminases and blood eosinophil count. In one of these cases, in which the initial histological lesions had the appearance of aggressive chronic hepatitis, there was progression to cirrhosis despite the interruption of treatment. The mechanism of this hepatotoxicity is probably a hypersensitivity reaction.

MESH:D000069286 - bortezomib

Summary:

LLM Prediction Score	0.692 (normalized)
LLM Confidence Score	0.990
Golden Answer (Severity Class)	0.875 (normalized)
Prediction Error	0.183

Retrieved Context:

Title: Bortezomib-induced severe hepatitis in multiple myeloma: a case report.
Bortezomib is a novel proteasome inhibitor with significant antimyeloma activity. Its toxicity is manageable, and the most frequent adverse effects mainly consist of gastrointestinal symptoms, peripheral neuropathy, neuropatic pain, and thrombocytopenia. Severe liver toxicity has not been previously recognized. A patient with relapsed multiple myeloma who developed bortezomib-induced severe recurrent hepatitis is described. The importance of recognizing this rare potential toxicity is highlighted in order to discontinue this agent if liver adverse reaction is suspected.

Title: Bortezomib-associated fatal liver failure in a haemodialysis patient with multiple myeloma.
Bortezomib is a proteasome inhibitor with excellent antimyeloma activity. The most frequent toxic side effects are gastrointestinal, neuropathy and thrombocytopenia. The liver was not considered an important target organ for toxicity until one case of bortezomib-induced severe hepatitis was reported in a patient with multiple myeloma.

Title: Phase 1 clinical trial of bortezomib in adults with recurrent malignant glioma.
Bortezomib selectively binds and inhibits the 20S proteasome enzyme's active sites. This study was conducted to determine the side effects and maximum tolerated dose (MTD) of bortezomib in patients with recurrent malignant glioma. Separate dose escalations were conducted in patients taking or not taking enzyme-inducing anti-seizure drugs (+/-EIASD). The starting dose in both groups was 0.9 mg/m(2) intravenously twice weekly for the first three of each 4 week cycle. Imaging assessment of response was carried out and Plasma 20S proteasome activity inhibition and imaging was conducted to monitor efficacy. The 66 patients enrolled had a median age of 51 years,... (truncated)

Title: Bortezomib alleviates drug-induced liver injury by regulating CYP2E1 gene transcription.
Acute liver failure, i.e., the fatal deterioration of liver function, is the most common indication that emergency liver transplantation is necessary. Moreover, in the USA, drug-induced liver injury (DILI), including acetaminophen (APAP)-induced hepatotoxicity, is the main cause of acute liver failure. Matching a donor for liver transplantation is extremely difficult, and thus the development of a novel therapy for DILI is urgently needed. Following recent approval by the FDA of the proteasomal inhibitor bortezomib, its therapeutic effects on various human diseases, including solid and hematologic malignancies, have been validated. However, the specific action of proteasomal inhibition in cases of DILI... (truncated)

Title: A Case of Drug-Induced Hepatitis due to Bortezomib in Multiple Myeloma.
We report on a case of severe hepatotoxicity in a 52-year-old male with multiple myeloma (MM) who had received bortezomib therapy. At patient presentation, liver enzymes were normal, but started to markedly increase 3 days after the patient's second dose of bortezomib was administered, when free kappa light chains were noticeably reduced in the serum. After discontinuation of bortezomib, liver enzymes recovered gradually to baseline. Then, the patient was started on a thalidomide-containing regimen, which he was able to tolerate well. The patient achieved complete remission prior to autologous stem cell transplantation (ASCT). The patient underwent ASCT without occurrence of... (truncated)

MESH:D010634 - phenobarbital

Summary:

LLM Prediction Score	0.558 (normalized)
LLM Confidence Score	0.990
Golden Answer (Severity Class)	0.375 (normalized)
Prediction Error	0.183

Retrieved Context:

Title: [Acute hepatic failure associated with valproic acid in children. Report of 3 cases].
We report the cases of three epileptic children who developed hepatotoxicity induced by valproic acid. Two patients had developmental delay. Including the one who died, all patients were receiving polytherapy (carbamazepine in two and phenobarbital in one). The patients age ranged from 2 years and 8 months to 5 years and 1 month. The onset of hepatic complications occurred within 6 months of valproate therapy in two patients and 12 months in one. All patients developed the classical clinical signs of hepatotoxicity. Vomiting, edema and jaundice were the initial symptoms. Fever occurred in two patients. The serum levels of glutamic... (truncated)

Title: Jaundice and rash associated with the use of phenobarbital and hydrochlorothiazide.
Rash, lymphadenopaty, splenomegaly, periorbital edema, and hepatitis occurred in an 18-year-old woman who was taking phenobarbital and hydrochlorothiazide. Tests for fluorescent antinuclear antibody and hepatitis-associated antigen and antibody were negative. Liver biopsy was not characteristic of viral hepatitis. Clinical recovery occurred within two weeks. Treatment consisted of withdrawal of the above drugs plus the administration of methylprednisolone and diphenhydramine.

Title: Phenobarbital hepatotoxicity in an 8-month-old infant.
Severe hepatotoxicity from phenobarbital occurred in an infant boy who had a complicated illness with chronic bilateral subdural hematomas and sepsis. Skin rash began after 2 weeks of treatment, and signs of hepatocellular failure developed 3 weeks after phenobarbital had been started. Signs of severe liver disease included elevated aminotransferases, conjugated hyperbilirubinemia, significant coagulopathy, hepatosplenomegaly and ascites. Other features of this adverse drug reaction were unremitting fever, leukocytosis with eosinophilia and atypical lymphocytosis, and proteinuria. Sepsis, viral hepatitis, and metabolic liver disease were excluded. The child was on no other medication and had been previously well. In-vitro rechallenge of the... (truncated)

Title: Drug-induced liver injury. In vitro demonstration of hypersensitivity to both phenytoin and phenobarbital.
Fever, lymphadenopathy, exfoliative dermatitis, and evidence of drug-induced liver injury developed in a 16-year-old girl three weeks after beginning therapy with phenytoin and phenobarbital. This clinical syndrome can be caused by either of these structurally related drugs but has been more frequently attributed to phenytoin. In vitro studies disclosed marked reactivity of this patient's lymphocytes to concentrations of both drugs, which encompassed their measured serum levels. The demonstration of dual reactivity raises concerns about continuing administration of phenobarbital during an apparent phenytoin-induced reaction. Whether this potential risk is greater than the risk of stopping all anticonvulsant medications in a patient... (truncated)

Title: Antiepileptic Drugs and Liver Disease.
Acute, symptomatic seizures or epilepsy may complicate the course of hepatic disease. Choosing the most appropriate antiepileptic drug in this setting represents a difficult challenge, as most medications are metabolized by the liver. This article focuses on the acute and chronic treatment of seizures in patients with advanced liver disease and reviews the hepatotoxic potential of specific antiepileptic drugs. Newer antiepileptic drugs without, or with minimal, hepatic metabolism, such as levetiracetam, lacosamide, topiramate, gabapentin, and pregabalin should be used as first-line therapy. Medications undergoing extensive hepatic metabolism, such as valproic acid, phenytoin, and felbamate should be used as drugs of... (truncated)

MESH:D002981 - clindamycin

Summary:

LLM Prediction Score	0.556 (normalized)
LLM Confidence Score	0.990
Golden Answer (Severity Class)	0.375 (normalized)
Prediction Error	0.181

Retrieved Context:

Title: Clindamycin-induced acute cholestatic hepatitis.
We report a case of acute hepatotoxicity in a 42-year-old woman after administration of clindamycin for a dental infection. After 6 d of treatment, she had fatigue, nausea, vomiting, anorexia, pruritus and jaundice. Her laboratory analysis showed alanine aminotransferase (ALT), 1795 IU/L (normal range 0-40); aspartate aminotransferase (AST), 1337 IU/L (normal range 5-34); alkaline phosphatase (ALP), 339 IU/L (normal range 40-150); gamma-glutamyl transpeptidase (GGT), 148 IU/L (normal range 9-64 IU/L); total bilirubin, 4.1 mg/dL; direct bilirubin, 2.9 mg/dL and prothrombin time (PT), 13.5 s, with international normalized ratio (INR), 1.04. She was hospitalized, with immediate drug discontinuation. Her liver biopsy... (truncated)

Title: Hepatotoxicity due to Clindamycin in Combination with Acetaminophen in a 62-Year-Old African American Female: A Case Report and Review of the Literature.
Clindamycin is a bacteriostatic lincosamide antibiotic with a broad spectrum. Side effects include nausea, vomiting, diarrhea, and metallic taste; however, hepatotoxicity is rare. The incidence is unknown. It is characterized by increases in aspartate and alanine transaminases. There may be no symptoms and the treatment is to stop the administration of clindamycin. We have described a 62-year-old African American female medicated with acetaminophen and clindamycin who had initially presented to the dental clinic for the evaluation of gum pain following tooth extraction. She had significantly increased levels of liver transaminases, which trended downwards on quitting the medication.

Title: Oral clindamycin causing acute cholestatic hepatitis without ductopenia: a brief review of idiosyncratic drug-induced liver injury and a case report.
Clindamycin is a lincosamide antibiotic active against most of the anaerobes, protozoans, and Gram-positive bacteria, including community-acquired methicillin-resistant Staphylococcus aureus. Its use has increased greatly in the recent past due to wide spectrum of activity and good bioavailability in oral form. Close to 20% of the patients taking clindamycin experience diarrhea as the most common side effect. Hepatotoxicity is a rare side effect. Systemic clindamycin therapy has been linked to two forms of hepatotoxicity: transient serum aminotransferase elevation and an acute idiosyncratic liver injury that occurs 1-3 weeks after starting therapy. This article is a case report of oral clindamycin... (truncated)

Title: Cholestatic liver disease with ductopenia (vanishing bile duct syndrome) after administration of clindamycin and trimethoprim-sulfamethoxazole.
Two patients who developed cholestatic liver disease after exposure to antibiotics are described. One patient who received clindamycin had liver biopsy findings of marked cholestasis, portal inflammation, bile duct injury and bile duct paucity (ductopenia). A second biopsy after clinical improvement showed resolution of cholestasis but persistence of duct paucity. Three years later, treatment with ampicillin caused another episode of cholestatic hepatitis with cholestasis and duct paucity on rebiopsy. The second patient, who developed cholestasis after receiving trimethoprim-sulfamethoxazole, had marked duct paucity in the liver biopsy. This is the first description, to our knowledge, of ductopenia apparently caused by clindamycin.... (truncated)

Title: A Case of Levofloxacin-Induced Hepatotoxicity.
BACKGROUND Levofloxacin covers a broad spectrum of pathogens and is readily prescribed by clinicians. Hepatotoxicity is a known but unusual complication of levofloxacin use. Here, we present a case of severe transaminitis caused by levofloxacin. CASE REPORT A young man in his thirties with a history of asthma, chronic alcoholism, methamphetamine intravenous drug abuse (IVDA), and non-compliant insulin-dependent diabetes mellitus (IDDM) presented to an emergency department with suicidal ideation. Vital signs were stable and the patient was noted to have cellulitis of the right forearm, for which cultures were drawn, and he received IV clindamycin. He was admitted to behavioral... (truncated)

MESH:D011398 - promethazine

Summary:

LLM Prediction Score	0.445 (normalized)
LLM Confidence Score	0.990
Golden Answer (Severity Class)	0.625 (normalized)
Prediction Error	0.180

Retrieved Context:

Title: Enhanced activation of human NK cells by drug-exposed hepatocytes.
Drug-induced liver injury (DILI) represents one of the major causes why drugs have to be withdrawn from the market. In this study, we describe a new interaction between drug-exposed hepatocytes and natural killer (NK) cells. In a previous genome-wide expression analysis of primary human hepatocytes that had been exposed to clinically relevant concentrations of 148 drugs, we found that several activating ligands for NK cell receptors were regulated by various drugs (e.g., valproic acid, ketoconazole, promethazine, isoniazid). Especially expression of the activating NKG2D ligands (MICA, MICB and ULBPs) and the NKp30 ligand B7-H6 were upregulated in primary human hepatocytes upon... (truncated)

Title: [Psychiatric drugs as risk factor in fatal heat stroke].
Two men aged 33 and 31 years suffered a fatal heat stroke on a warm summer day. One of them used pimozide and clomipramine, the other zuclopenthixol, dexetimide, droperidol, promethazine and propranolol as psychiatric medication. Both of them had a body temperature > 42.3 degrees C, without perspiring. At first only a comatose situation with practically normal laboratory values existed; this was rapidly followed by massive liver damage, disseminated intravascular coagulation, anaemia, thrombopenia and acute renal failure. In spite of adequate and rapid treatment these complications were fatal. Both patients used medication with an antidopaminergic and anticholinergic (side) effect. The... (truncated)

Title: Clinically approved heterocyclics act on a mitochondrial target and reduce stroke-induced pathology.
Substantial evidence indicates that mitochondria are a major checkpoint in several pathways leading to neuronal cell death, but discerning critical propagation stages from downstream consequences has been difficult. The mitochondrial permeability transition (mPT) may be critical in stroke-related injury. To address this hypothesis, identify potential therapeutics, and screen for new uses for established drugs with known toxicity, 1,040 FDA-approved drugs and other bioactive compounds were tested as potential mPT inhibitors. We report the identification of 28 structurally related drugs, including tricyclic antidepressants and antipsychotics, capable of delaying the mPT. Clinically achievable doses of one drug in this general structural class... (truncated)

Title: Histamine H1 receptor antagonist attenuates catecholamine surge and organ injury after severe burns.
Severe burns induce a catecholamine surge, causing severe damage to the organism and raising the possibility of multisystem organ failure. Few strategies are generally acceptable to reduce catecholamine surge and organ injury post-burn. We have previously shown that histamine can amplify the catecholamine surge. In addition, promethazine, a first-generation histamine H1 receptor antagonist, alleviates catecholamine surge and organ injury after severe burns in rats. However, evidence is lacking on whether promethazine benefits patients after severe burns. Currently, sedation and analgesia (such as midazolam and fentanyl) are commonly required for patients after severe burns. It remains unclear if patients after severe... (truncated)

Title: Phenothiazines Enhance the Hypothermic Preservation of Liver Grafts: A Pilot in Vitro Study.
In vitro liver conservation is an issue of ongoing critical importance in graft transplantation. In this study, we investigated the possibility of augmenting the standard pre-transplant liver conservation protocol (University of Wisconsin (UW) cold solution) with the phenothiazines chlorpromazine and promethazine. Livers from male Sprague-Dawley rats were preserved either in UW solution alone, or in UW solution plus either 2.4, 3.6, or 4.8 mg chlorpromazine and promethazine (C+P, 1:1). The extent of liver injury following preservation was determined by alanine aminotransferase (ALT) and aspartate aminotransferase (AST) activities, the ratio of AST/ALT, morphological changes as assessed by hematoxylin-eosin staining, apoptotic cell... (truncated)

MESH:D004280 - dobutamine

Summary:

LLM Prediction Score	0.179 (normalized)
LLM Confidence Score	0.990
Golden Answer (Severity Class)	0.0 (normalized)
Prediction Error	0.179

Retrieved Context:

Title: Inflammation-induced hepatotoxicity in humans.
Because severe sepsis is frequently complicated by multiple organ failure, it is of importance to monitor organ function. Unfortunately, conventional liver function markers are either relatively unspecific or have a long half-life, which make them poor predictors of acute liver injury. Glutathione S-transferase A1-1 (GSTA1-1) has a relatively short half-life (1 h), is more specific, and is rapidly released into the blood after liver damage. In the present study, we measured plasma GSTA1-1 levels by enzyme-linked immunosorbent assay in seven healthy volunteers after repeated experimental endotoxemia induced by 2 ng kg Escherichia coli endotoxin per day (to investigate inflammation-induced hepatic... (truncated)

Title: [Ischemic hepatitis due to anthracycline-induced cardiac insufficiency in a patient with acute myelocytic leukemia (M0)].
A 25 year-old woman diagnosed as acute myelocytic leukemia (M0) suffered a fourth relapse in February 1992 at which time she already had anthracycline-induced cardiac dysfunction. Although remission was induced by low dose cytosine arabinoside and etoposide combined with pirarubicin, she developed acute heart failure followed by extreme elevation of transaminases level and DIC. Abdominal echography and CT revealed small round lesions in the liver. We diagnosed this episode as ischemic hepatitis because of the following clinical findings; serological markers of virus hepatitis were negative, hypotension and reduced blood flow to the liver were seen, and both transaminases and LDH... (truncated)

Title: Increased transaminases in psychiatry: a case report.
We report the case of a patient admitted to the hospital with psychiatric troubles. Soon after admission, he presented severe hepatitis of unknown origin. Careful review of the charts, transvenous liver biopsy, right heart and hepatic pressure measurements, negative toxicologic and viral screenings were highly suggestive of hypoxic hepatitis. Indeed, the patient had previously been treated for a decompensated cardiomyopathy and medications stopped prior to the current admission. Without clear clinical evidence of heart failure he presented a brief malaise two days before the increase in liver enzymes. Holter heart recording showed afterwards bouts of ventricular tachycardia. Treatment with Dobutamine... (truncated)

Title: Daptomycin-Induced Acute Liver Failure: A Rare Case Report.
Acute liver failure (ALF) is characterized by severe liver injury, encephalopathy, and impaired coagulation/synthetic function. Drug-induced liver injury (DILI) can rarely, in a dose-dependent manner, lead to ALF. This article presents a rare case of daptomycin-induced acute liver failure in a patient with no prior liver disease. A 73-year-old male with multiple comorbidities including heart failure, diabetes, and chronic kidney disease received daptomycin treatment for diabetic left foot osteomyelitis. Five days after starting therapy, he developed weakness, jaundice, and drowsiness, leading to ICU admission. Physical examination and labs revealed hepatomegaly, elevated liver enzymes and abnormal ultrasound findings. Autoimmune and infectious... (truncated)

Title: Liver perfusion and hepatocellular inflammatory response in sepsis.
Sepsis is characterized by disturbances in liver perfusion and alterations in intrahepatic cellular functions and interactions. This provokes structural and functional liver damage as well as hepatocellular activation that is believed to perpetuate the immuno-inflammatory response. Changes in hepatic perfusion during sepsis are still poorly understood due to the heterogeneity of septic animal models and the difficult accessibility of the hepatic circulation in humans. Sinusoidal blood flow is severely compromised during sepsis due to a decline in perfused sinusoidal area in association with a decrease in sinusoidal flow velocity. Imbalances in the production of nitric oxide may account for these... (truncated)

MESH:D009543 - nifedipine

Summary:

LLM Prediction Score	0.553 (normalized)
LLM Confidence Score	0.990
Golden Answer (Severity Class)	0.375 (normalized)
Prediction Error	0.178

Retrieved Context:

Title: Nifedipine hepatitis.
An 80-year-old woman developed acute hepatitis following her first exposure to nifedipine. This adverse effect was characterised by fever, chills, anorexia, nausea, liver tenderness, hepatitic liver function tests and peripheral blood eosinophilia. On liver biopsy the portal tracts were expanded with a mixed inflammatory cell infiltrate rich in eosinophils. The potential for the occurrence of this adverse effect must increase with the current expansion of indications for the use of nifedipine.

Title: A case report of nifedipine-induced hepatitis with jaundice.
Nifedipine is a generic, well-known and commonly-prescribed dihydropyridine calcium channel blocker used in the treatment of hypertension and Prinzmetal's angina. A known but very rare and serious adverse effect of nifedipine is clinically-apparent hepatitis which can take months to resolve.

Title: Effect of subclinical, clinical and supraclinical doses of calcium channel blockers on models of drug-induced hepatotoxicity in rats.
Drug-related hepatotoxicity is the leading cause of acute liver failure, and hepatic problems are responsible for a significant number of liver transplantations and deaths worldwide. Calcium has been associated with various metabolic processes that lead to cell death and apoptosis, and increased cytosolic Ca(2+) has been implicated in hepatotoxicity. This study was designed to investigate the effects of calcium channel blockers (CCBs) on isoniazid-rifampicin, zidovudine and erythromycin-induced hepatotoxicity in rats. Treatment groups comprised control, hepatotoxicant, hepatotoxicant along with each of silymarin, nifedipine, verapamil and diltiazem at subclinical, clinical and supraclinical doses. A day to the end of treatment for each... (truncated)

Title: Hepatotoxicity with the administration of nifedipine for treatment of preterm labor.
Nifedipine, a calcium channel blocker, is becoming increasingly popular as a tocolytic medication. Previous reports of hepatitis associated with nifedipine in the medical literature have been published. We present the first case reported of a patient with preterm labor treated with nifedipine who had development of a concurrent elevation in serum liver enzyme levels.

Title: Alcoholic-like liver lesions induced by nifedipine.
We report the case of a 78-year-old patient suffering from alcoholic-like liver injury, consisting of steatosis and Mallory bodies, caused by the calcium channel blocker, nifedipine.

MESH:D000077408 - modafinil

Summary:

LLM Prediction Score	0.197 (normalized)
LLM Confidence Score	0.990
Golden Answer (Severity Class)	0.375 (normalized)
Prediction Error	0.178

Retrieved Context:

Title: Approved and investigational uses of modafinil : an evidence-based review.
Modafinil is a wake-promoting agent that is pharmacologically different from other stimulants. It has been investigated in healthy volunteers, and in individuals with clinical disorders associated with excessive sleepiness, fatigue, impaired cognition and other symptoms. This review examines the use of modafinil in clinical practice based on the results of randomized, double-blind, placebo-controlled clinical trials available in the English language in the MEDLINE database. In sleep-deprived individuals, modafinil improves mood, fatigue, sleepiness and cognition to a similar extent as caffeine but has a longer duration of action. Evidence for improved cognition in non-sleep-deprived healthy volunteers is controversial.Modafinil improves excessive sleepiness... (truncated)

Title: Modafinil-induced drug reaction with eosinophilia and systemic symptoms syndrome.
No abstract available.

Title: Narcolepsy: current treatment options and future approaches.
The management of narcolepsy is presently at a turning point. Three main avenues are considered in this review: 1) Two tendencies characterize the conventional treatment of narcolepsy. Modafinil has replaced methylphenidate and amphetamine as the first-line treatment of excessive daytime sleepiness (EDS) and sleep attacks, based on randomized, double blind, placebo-controlled clinical trials of modafinil, but on no direct comparison of modafinil versus traditional stimulants. For cataplexy, sleep paralysis, and hypnagogic hallucinations, new antidepressants tend to replace tricyclic antidepressants and selective serotonin reuptake inhibitors (SSRIs) in spite of a lack of randomized, double blind, placebo-controlled clinical trials of these compounds;... (truncated)

Title: Inflammation in Depression and the Potential for Anti-Inflammatory Treatment.
Accumulating evidence supports an association between depression and inflammatory processes, a connection that seems to be bidirectional. Clinical trials have indicated antidepressant treatment effects for anti-inflammatory agents, both as add-on treatment and as monotherapy. In particular, nonsteroidal anti-inflammatory drugs (NSAIDs) and cytokine-inhibitors have shown antidepressant treatment effects compared to placebo, but also statins, poly-unsaturated fatty acids, pioglitazone, minocycline, modafinil, and corticosteroids may yield antidepressant treatment effects. However, the complexity of the inflammatory cascade, limited clinical evidence, and the risk for side effects stress cautiousness before clinical application. Thus, despite proof-of-concept studies of anti-inflammatory treatment effects in depression, important challenges remain... (truncated)

Title: Fatigue in chronic liver disease patients: prevalence, pathophysiology, and management.
Fatigue is the most commonly encountered symptom in patients with chronic liver disease (CLD). The resulting decrease in quality of life contributes markedly to the societal costs of fatigue. Moreover, fatigue is associated with social dysfunction, increased daytime somnolence, impaired working ability, and increased risk of mortality. Fatigue is not related to the severity of the underlying liver fibrosis or dysfunction. In CLD patients, fatigue manifests with both central symptoms, characterised by cognitive impairment, sleep disturbance, apathy, and autonomic dysfunction, and peripheral symptoms, characterised by decreased exercise tolerance and reduced physical activity levels. The pathogenesis of fatigue in CLD is... (truncated)

MESH:D013888 - thiothixene

Summary:

LLM Prediction Score	0.428 (normalized)
LLM Confidence Score	0.940
Golden Answer (Severity Class)	0.25 (normalized)
Prediction Error	0.178

Retrieved Context:

Title: Toxic hepatitis and single daily dosage imipramine therapy.
Liver function abnormalities have occasionally been associated with imipramine since the drug was introduced in 1957. The mechanism by which the drug produces hepatotoxicity has not been determined but may involve a direct toxic effect or a hypersensitivity reaction. In this case, a patient receiving 6 mg/kg (300 mg) of imipramine daily developed elevated liver enzymes while the plasma imipramine concentration was found to be in a therapeutic range. This case suggests that single daily dosing of tricyclic antidepressants may be more hazardous to the liver than divided doses.

Title: Quantitative NTCP pharmacophore and lack of association between DILI and NTCP Inhibition.
The human sodium taurocholate cotransporting polypeptide (NTCP) is a hepatic bile acid transporter. Inhibition of NTCP uptake may potentially also prevent hepatitis B virus (HBV) infection. The first objective was to develop a quantitative pharmacophore for NTCP inhibition. Recent studies showed that hepatotoxic drugs could inhibit bile acid uptake into hepatocytes, without inhibiting canalicular efflux, and cause bile acid elevation in plasma. Hence, a second objective was to examine whether NTCP inhibition is associated with drug induced liver injury (DILI). Twenty-seven drugs from our previous study were used as the training set to develop a quantitative pharmacophore. From secondary screening... (truncated)

Title: Antipsychotic Drugs Efficacy in Dextromethorphan-Induced Psychosis.
Psychosis is known as a broad term of symptoms that cause serious disorganization of behavior, thinking, and perception of reality. One of the medicines that recently gained much attention in terms of its psychotic potential is dextromethorphan (DXM). DXM, a widely used antitussive drug, is a commonly abused drug because of its euphoric, hallucinogenic, and dissociative properties. To date, DXM is a legally marketed cough suppressant that is neither a controlled substance nor a regulated chemical under the Controlled Substances Act. The management of DXM-related psychosis is dependent on the type of psychotic symptoms. Atypical neuroleptics (i.e., olanzapine, risperidone, quetiapine)... (truncated)

Title: Fluphenazine (oral) versus placebo for schizophrenia.
Fluphenazine is one of the first drugs to be classed as an 'antipsychotic' and has been widely available for five decades.

Title: Clinically approved heterocyclics act on a mitochondrial target and reduce stroke-induced pathology.
Substantial evidence indicates that mitochondria are a major checkpoint in several pathways leading to neuronal cell death, but discerning critical propagation stages from downstream consequences has been difficult. The mitochondrial permeability transition (mPT) may be critical in stroke-related injury. To address this hypothesis, identify potential therapeutics, and screen for new uses for established drugs with known toxicity, 1,040 FDA-approved drugs and other bioactive compounds were tested as potential mPT inhibitors. We report the identification of 28 structurally related drugs, including tricyclic antidepressants and antipsychotics, capable of delaying the mPT. Clinically achievable doses of one drug in this general structural class... (truncated)

MESH:C041226 - terazosin

Summary:

LLM Prediction Score	0.177 (normalized)
LLM Confidence Score	0.970
Golden Answer (Severity Class)	0.0 (normalized)
Prediction Error	0.177

Retrieved Context:

Title: Acute hepatocellular drug induced liver injury probably by alfuzosin.
Alpha blockers are the drugs that exert their effects by binding to alpha receptors and relaxing smooth muscles and are currently used for treatment of benign prostate hyperplasia (BPH). These drugs are often tolerated well by the patients. However, they also possess some common side effects. Hepatotoxicity, on the other hand, is quite rare. We report herein a case with the rare complication of acute hepatocellular drug induced liver injury (DILI) by administration of Alfuzosin.

Title: The Role of α1-Adrenoceptor Antagonists in the Treatment of Prostate and Other Cancers.
This review evaluates the role of α-adrenoceptor antagonists as a potential treatment of prostate cancer (PCa). Cochrane, Google Scholar and Pubmed were accessed to retrieve sixty-two articles for analysis. In vitro studies demonstrate that doxazosin, prazosin and terazosin (quinazoline α-antagonists) induce apoptosis, decrease cell growth, and proliferation in PC-3, LNCaP and DU-145 cell lines. Similarly, the piperazine based naftopidil induced cell cycle arrest and death in LNCaP-E9 cell lines. In contrast, sulphonamide based tamsulosin did not exhibit these effects. In vivo data was consistent with in vitro findings as the quinazoline based α-antagonists prevented angiogenesis and decreased tumour mass in... (truncated)

Title: Addressing the Side Effects of Contemporary Antidepressant Drugs: A Comprehensive Review.
Randomized trials have shown that selective serotonin reuptake inhibitors (SSRIs) and serotonin-norepinephrine reuptake inhibitors (SNRIs) have better safety profiles than classical tricyclic antidepressants (TCAs). However, an increasing number of studies, including meta-analyses, naturalistic studies, and longer-term studies suggested that SSRIs and SNRIs are no less safe than TCAs. We focused on comparing the common side effects of TCAs with those of newer generation antidepressants including SSRIs, SNRIs, mirtazapine, and bupropion. The main purpose was to investigate safety profile differences among drug classes rather than the individual antidepressants, so studies containing comparison data on drug groups were prioritized. In terms of... (truncated)

Title: Manifestations of Liver Impairment and the Effects of MH-76, a Non-Quinazoline α1-Adrenoceptor Antagonist, and Prazosin on Liver Tissue in Fructose-Induced Metabolic Syndrome.
Excessive fructose consumption may lead to metabolic syndrome, metabolic dysfunction-associated fatty liver disease (MAFLD) and hypertension. α1-adrenoceptors antagonists are antihypertensive agents that exert mild beneficial effects on the metabolic profile in hypertensive patients. However, they are no longer used as a first-line therapy for hypertension based on Antihypertensive and Lipid-Lowering Treatment to Prevent Heart Attack Trial (ALLHAT) outcomes. Later studies have shown that quinazoline-based α1-adrenolytics (prazosin, doxazosin) induce apoptosis; however, this effect was independent of α1-adrenoceptor blockade and was associated with the presence of quinazoline moiety. Recent studies showed that α1-adrenoceptors antagonists may reduce mortality in COVID-19 patients due to... (truncated)

Title: Alcohol and medication interactions.
Many medications can interact with alcohol, thereby altering the metabolism or effects of alcohol and/or the medication. Some of these interactions can occur even at moderate drinking levels and result in adverse health effects for the drinker. Two types of alcohol-medication interactions exist: (1) pharmacokinetic interactions, in which alcohol interferes with the metabolism of the medication, and (2) pharmacodynamic interactions, in which alcohol enhances the effects of the medication, particularly in the central nervous system (e.g., sedation). Pharmacokinetic interactions generally occur in the liver, where both alcohol and many medications are metabolized, frequently by the same enzymes. Numerous classes of... (truncated)

MESH:D000068582 - certolizumab pegol

Summary:

LLM Prediction Score	0.323 (normalized)
LLM Confidence Score	0.950
Golden Answer (Severity Class)	0.5 (normalized)
Prediction Error	0.177

Retrieved Context:

Title: Liver-side of inflammatory bowel diseases: Hepatobiliary and drug-induced disorders.
Hepatobiliary disorders are among the most common extraintestinal manifestations in inflammatory bowel diseases (IBD), both in Crohn's disease and ulcerative colitis (UC), and therefore represent a diagnostic challenge. Immune-mediated conditions include primary sclerosing cholangitis (PSC) as the main form, variant forms of PSC (namely small-duct PSC, PSC-autoimmune hepatitis overlap syndrome and IgG4-related sclerosing cholangitis) and granulomatous hepatitis. PSC is by far the most common, presenting in up to 8% of IBD patients, more frequently in UC. Several genetic foci have been identified, but environmental factors are preponderant on disease pathogenesis. The course of the two diseases is typically independent. PSC... (truncated)

Title: One-year risk of serious infection in patients treated with certolizumab pegol as compared with other TNF inhibitors in a real-world setting: data from a national U.S. rheumatoid arthritis registry.
Registry studies provide a valuable source of comparative safety data for tumor necrosis factor inhibitors (TNFi) used in rheumatoid arthritis (RA), but they are subject to channeling bias. Comparing safety outcomes without accounting for channeling bias can lead to inaccurate comparisons between TNFi prescribed at different stages of the disease. In the present study, we examined the incidence of serious infection and other adverse events during certolizumab pegol (CZP) use vs other TNFi in a U.S. RA cohort before and after using a methodological approach to minimize channeling bias.

Title: Long-term safety and clinical outcomes of certolizumab pegol treatment in patients with active non-radiographic axial spondyloarthritis: 3-year results from the phase 3 C-axSpAnd study.
52-week results from C-axSpAnd demonstrated the safety and efficacy of certolizumab pegol (CZP) in patients with active non-radiographic axial spondyloarthritis (nr-axSpA) and objective signs of inflammation (sacroiliitis on MRI and/or elevated C-reactive protein levels). Long-term safety and clinical outcomes, including MRI assessments, are evaluated up to 3 years for CZP-treated patients with nr-axSpA.

Title: When Autoimmunity 'DRESSes up': A Case after Certolizumab Therapy.
Drug Reaction with Eosinophilia and Systemic Symptoms (DRESS) is characterised by skin rash together with visceral organ involvement, lymphadenopathy, eosinophilia and atypical lymphocytosis. The syndrome is clinically heterogeneous, making diagnosis challenging. It has an annual incidence of 2 per 100,000 population and a mortality rate of 2-10%. We describe the first case of DRESS induced by certolizumab, a biologic disease-modifying antirheumatic drug (bioDMARD).

Title: Long-term safety of certolizumab pegol in plaque psoriasis: pooled analysis over 3 years from three phase III, randomized, placebo-controlled studies.
Certolizumab pegol (CZP) is an Fc-free, PEGylated anti-tumour necrosis factor biologic.

MESH:D000077154 - rosiglitazone

Summary:

LLM Prediction Score	0.552 (normalized)
LLM Confidence Score	0.980
Golden Answer (Severity Class)	0.375 (normalized)
Prediction Error	0.177

Retrieved Context:

Title: Severe cholestatic hepatitis caused by thiazolidinediones: risks associated with substituting rosiglitazone for troglitazone.
Troglitazone maleate (Rezulin) has been associated with severe hepatotoxicity, which led to its withdrawal from the U.S. market in March 2000. Rosiglitazone maleate (Avandia) is being marketed as a safe alternative in the treatment of type 2 diabetes mellitus. We report a case of severe thiazolidinedione-induced cholestatic hepatitis in a 56-year-old female patient at a university hospital who was given rosiglitazone, 8 mg/day, after she developed milder hepatotoxicity while taking troglitazone. Rosiglitazone was discontinued, and the patient was treated with prednisone, azathioprine, and ursodiol. Clinical evaluation and liver biopsy were performed and liver function tests were monitored. After being switched... (truncated)

Title: Case series of liver failure associated with rosiglitazone and pioglitazone.
The thiazolidinedione drugs rosiglitazone and pioglitazone are not widely known to be hepatotoxic. We evaluated the FDA Adverse Event Reporting System (AERS) to determine the number of reported cases of liver failure associated with rosiglitazone and pioglitazone between 1997 and 2006, and described their clinical characteristics.

Title: Thiazolidinediones and liver toxicity.
Thiazolidinediones or glitazones specifically target insulin resistance. They have proven efficacy for reducing plasma glucose levels of type 2 diabetic patients treated with diet alone, sulphonylureas, metformin or insulin. In addition, they may be associated to some improvement of cardiovascular risk profile. However, troglitazone, the first compound approved by the FDA in the US, proved to be hepatotoxic and was withdrawn from the market after the report of several dozens of deaths or cases of severe hepatic failure requiring liver transplantation. It remains unclear whether or not hepatotoxicity is a class effect or is related to the unique tocopherol side... (truncated)

Title: Evaluation of liver function in type 2 diabetic patients during clinical trials: evidence that rosiglitazone does not cause hepatic dysfunction.
Troglitazone treatment has been associated with idiosyncratic hepatic reaction leading to hepatic failure and death in some patients. This raises questions regarding whether all thiazolidinediones or peroxisomal proliferator-activated receptor-gamma (PPAR-gamma) agonists are hepatotoxic and whether data from clinical trials are adequate to detect a signal of potentially serious drug-related hepatotoxicity. The purpose of this study was to assess whether the idiosyncratic liver toxicity reported with troglitazone is molecule-specific or a thiazolidinedione class effect, based on liver enzyme data collected prospectively during phase 2/3 clinical trials with rosiglitazone, a new, potent, and specific member of the thiazolidinedione class.

Title: Hepatotoxicity with thiazolidinediones: is it a class effect?
Decreased insulin sensitivity plays a major role in various human diseases. particularly type 2 diabetes mellitus, and is associated with a higher risk of atherosclerosis and cardiovascular complications. Thiazolidinediones, more commonly termed glitazones, are the first drugs to specifically target muscular insulin resistance. They have proven efficacy for reducing plasma glucose levels in patients with type 2 diabetes mellitus treated with diet alone, sulphonylureas, metformin or insulin. In addition, they are associated with some improvement of the cardiovascular risk profile. However, troglitazone, the first compound approved by the Food and Drug Administration in the US, proved to be hepatotoxic and... (truncated)

MESH:D008775 - methylprednisolone

Summary:

LLM Prediction Score	0.550 (normalized)
LLM Confidence Score	0.990
Golden Answer (Severity Class)	0.375 (normalized)
Prediction Error	0.175

Retrieved Context:

Title: Methylprednisolone-induced hepatotoxicity in a 16-year-old girl with multiple sclerosis.
Multiple sclerosis (MS) is a chronic inflammatory disease with demyelination of the central nervous system. High-dosage corticosteroids are the first-line therapy in the acute relapsing of MS. We report a case of severe high-dose methylprednisolone-induced acute hepatitis in a patient with a new diagnosis of MS. A 16-year-old girl was admitted for urticaria, angioedema, nausea and vomiting a month later she had been diagnosed with MS and treated with high-dosage methylprednisolone. Laboratory investigations showed hepatic insufficiency with grossly elevated liver enzymes. A liver biopsy showed focal centrilobular hepatocyte necrosis with interface hepatitis. Methylprednisolone-induced hepatotoxicity can confuse the clinical picture of... (truncated)

Title: [Recurrent acute liver toxicity from intravenous methylprednisolone].
Adverse drug reactions (hepatotoxicity) are a frequent cause of acute liver injury with a wide clinical and histological spectrum. An early recognition of drug-related liver disease has been considered essential in clinical practice due to potential risks. In most cases exposure discontinuation improves the clinical picture.Steroids are used in a variety of clinical settings. However, intravenous steroids have rarely been associated with hepatotoxicity. We report the case of a middle-aged woman with multiple sclerosis who received a bolus of methylprednisolone on three occasions for the management of relapsing disease, with the development of repeated episodes of elevated liver enzymes after... (truncated)

Title: Methylprednisolone-induced acute liver injury in a patient treated for multiple sclerosis relapse.
Drug-induced liver injury is the fourth most common cause of liver disease in industrialised countries. Methylprednisolone is often considered to be a treatment with a low hepatotoxicity. We report a case of methylprednisolone-induced liver injury in a 35-year-old woman. She was admitted to our department for acute liver injury 2 months after a treatment with high dose of methylprednisolone (1 g/day) for a multiple sclerosis relapse. No other cause of liver injury could be found (screening for hepatotropic viruses, autoimmune antibodies, ceruloplasmin, abdominal ultrasonography and liver biopsy). Liver function tests spontaneously improved and returned to normal range within 6 weeks.... (truncated)

Title: Hepatotoxicity after high-dose methylprednisolone for demyelinating disease.
Liver toxicity, although not mentioned among the possible adverse effects of corticosteroids, has been occasionally reported in literature. We observed 2 cases of hepatotoxicity after a high-dose methylprednisolone treatment of a demyelinating disease and evaluated the potential relationship in the light of available evidence. The first patient developed a histologically documented acute hepatitis and recovered after 3 weeks. In the second patient, a mild augmentation of liver enzymes occurred, followed by normalization in a few days. The causal relationship between hepatotoxicity and methylprednisolone treatment was deemed probable in both cases. Careful review of the literature suggests that corticosteroid-induced liver damage... (truncated)

Title: Acute liver toxicity due to methylprednisolone: consider this diagnosis in the context of autoimmunity.
The occurrence of corticosteroid-induced hepatitis is a rare event that has been recently described in the literature. We report the case of an acute cytolytic hepatitis in a patient treated with methylprednisolone for multiple sclerosis associated with an autoimmune thyroid dysfunction. After ruling out other etiologies, we concluded that the acute liver injury was due to steroids, and we analyzed the specific circumstances in the literature where methylprednisolone may have been responsible for acute hepatitis.

MESH:D004315 - doxapram

Summary:

LLM Prediction Score	0.175 (normalized)
LLM Confidence Score	0.960
Golden Answer (Severity Class)	0.0 (normalized)
Prediction Error	0.175

Retrieved Context:

Title: A New Structure-Activity Relationship (SAR) Model for Predicting Drug-Induced Liver Injury, Based on Statistical and Expert-Based Structural Alerts.
The prompt identification of chemical molecules with potential effects on liver may help in drug discovery and in raising the levels of protection for human health. Besides <i>in vitro</i> approaches, computational methods in toxicology are drawing attention. We built a structure-activity relationship (SAR) model for evaluating hepatotoxicity. After compiling a data set of 950 compounds using data from the literature, we randomly split it into training (80%) and test sets (20%). We also compiled an external validation set (101 compounds) for evaluating the performance of the model. To extract structural alerts (SAs) related to hepatotoxicity and non-hepatotoxicity we used SARpy,... (truncated)

Title: State of the Art and Uses for the Biopharmaceutics Drug Disposition Classification System (BDDCS): New Additions, Revisions, and Citation References.
The Biopharmaceutics Drug Disposition Classification system (BDDCS) is a four-class approach based on water solubility and extent of metabolism/permeability rate. Based on the BDDCS class to which a drug is assigned, it is possible to predict the role of metabolic enzymes and transporters on the drug disposition of a new molecular entity (NME) prior to its administration to animals or humans. Here, we report a total of 1475 drugs and active metabolites to which the BDDCS is applied. Of these, 379 are new entries, and 1096 are revisions of former classification studies with the addition of references for the approved... (truncated)

Title: Fatal Neurotoxicosis in Dogs Associated with Tychoplanktic, Anatoxin-a Producing Tychonema sp. in Mesotrophic Lake Tegel, Berlin.
In May 2017, at least 12 dogs showed signs of acute neurotoxicosis after swimming in or drinking from Lake Tegel, a mesotrophic lake in Berlin, Germany, and several of the affected dogs died shortly afterwards despite intensive veterinary treatment. Cyanobacterial blooms were not visible at the water surface or the shorelines. However, detached and floating water moss (<i>Fontinalis antipyretica</i>) with high amounts of <i>Tychonema</i> sp., a potential anatoxin-a (ATX) producing cyanobacterium, was found near the beaches where the dogs had been swimming and playing. Necropsies of two of the dogs revealed no specific lesions beside the anamnestic neurotoxicosis. ATX was... (truncated)

Title: Pharmacologic advances in canine and feline reproduction.
Substantial improvements in therapeutic options for companion animal reproduction and gynecologic emergencies have been made over the last decade. New, alternative drug treatments, with fewer side effects and improved efficacy, are available. This has widened the spectrum of therapeutic possibilities for diseases that were previously treated only by surgical intervention. New drugs are available for estrus induction and pregnancy termination, as well as for the treatment of pyometra. This review summarizes the pharmacology and toxicology of reproductive agents currently in use for contraception, pyometra, dystocia, eclampsia, premature labor, agalactia, mastitis, metritis, and prostatic disorders, and compares their efficacy and safety... (truncated)

Title: Brucella sp. vertebral osteomyelitis with intercurrent fatal Staphylococcus aureus toxigenic enteritis in a bottlenose dolphin (Tursiops truncatus).
A previously beach-stranded, juvenile, male, bottlenose dolphin (Tursiops truncatus) was diagnosed with vertebral osteomyelitis of unknown etiology. Antemortem serological testing suggested past or current Brucella sp. infection; however, this could not be confirmed prior to death despite multiple isolation attempts from aspirates, blood, and biopsies. Systemic antibiotics were administered for over a year to control the suspected infection; however, the animal succumbed peracutely to infection by a highly pathogenic, enterotoxin-secreting Staphylococcus sp. Gross necropsy findings included a fistulous tract leading to locally extensive osteomyelitis of a coccygeal vertebra with sequestra and osteophytes from which a Brucella species was isolated. Histopathological... (truncated)

MESH:D064750 - rabeprazole

Summary:

LLM Prediction Score	0.200 (normalized)
LLM Confidence Score	0.970
Golden Answer (Severity Class)	0.375 (normalized)
Prediction Error	0.175

Retrieved Context:

Title: Review article: rabeprazole's tolerability profile in clinical trials.
Rabeprazole is a new member of a class of substituted benzimidazole drugs known as proton pump inhibitors. Comparative trials have demonstrated that it is at least as effective as omeprazole for the treatment of gastrooesophageal reflux disease (GERD), duodenal ulcers, or gastric ulcers. It is significantly more effective than histamine2-receptor antagonists for acid suppression, GERD healing and pain relief, and duodenal ulcer healing and pain relief. Adverse events reported during clinical trials provide an important indication of a medication's tolerability. We demonstrate that rabeprazole has a favourable adverse events profile. It is well tolerated in placebo-controlled studies and comparative trials... (truncated)

Title: Controlling on-demand gastric acidity in obese subjects: a randomized, controlled trial comparing a single dose of 20 mg rabeprazole and 20 mg omeprazole.
Obesity is associated with a risk of gastroesophageal reflux disease. The pharmacodynamic efficacy of proton pump inhibitors has not been specifically evaluated in obese subjects. The aim of this study was to compare the antisecretory response to a single oral dose of 20 mg rabeprazole, 20 mg omeprazole and placebo in obese subjects.

Title: Postmarketing surveillance of rabeprazole in upper gastrointestinal peptic lesions in Japanese patients with coexisting hepatic disorders.
Many Japanese patients with hepatic disorders confirmed on diagnostic imaging and coexisting upper gastrointestinal (GI) peptic lesions receive treatment with proton pump inhibitors. Some pharmacotherapies used to treat peptic ulcers have been associated with adverse drug reactions (ADRs), including elevated liver enzyme levels.

Title: Proton pump inhibitors in the elderly population.
Accurate diagnosis and effective treatment of acid-related diseases, including gastro-oesophageal reflux disease (GORD), are important health care priorities, particularly in the elderly. Both the prevalence and severity of GORD are increased in older individuals. The reason for the age-related increase in the prevalence of GORD is not completely understood, but it appears to result from both age-related changes in physiology and effects of the medications often taken by older people. The diagnosis of GORD in the elderly is also difficult because of its potential atypical presentation in these patients, as well as the overlap between GORD symptoms and those of... (truncated)

Title: Effect of Rabeprazole and Rebamipide in the Treatment of Upper Gastrointestinal Hemorrhage Associated with Dual Antiplatelet Therapy in Elderly Patients with Coronary Heart Disease.
To investigate the therapeutic effect of rabeprazole and rebamipide on patient age over 60 with dual antiplatelet therapy (DAPT)-related upper gastrointestinal hemorrhage following percutaneous coronary intervention (PCI). A total of 360 patients age over 60 undergoing PCI were recruited for antiplatelet therapy involving a combined treatment of aspirin (100 mg/d) and clopidogrel (75 mg/d). The enrolled patients were divided into 4 groups: the control group, the rabeprazole group, the rebamipide group, and the rabeprazole + rebamipide group. The incidence and severity of any upper gastrointestinal hemorrhage and the incidence of major adverse cardiac events (MACEs) were observed 6 months after the operation. The... (truncated)

MESH:D013148 - spironolactone

Summary:

LLM Prediction Score	0.425 (normalized)
LLM Confidence Score	0.990
Golden Answer (Severity Class)	0.25 (normalized)
Prediction Error	0.175

Retrieved Context:

Title: Spironolactone-induced hepatitis.
There is widespread use of spironolactone in medical practice and the indications for its use well established and side effect profile well known. Herein, we present a case of drug-induced hepatitis occurring in a 50-year-old woman using spironolactone for the treatment of androgenetic alopecia. Six weeks after commencement of spironolactone the patient became unwell, complained of an extensive itch, but no icterus or jaundice. Liver function tests found abnormally elevated bilirubin and enzymes levels. After withdrawal of spironolactone, the patient's symptoms resolved and liver function improved. To date, there has only been one other report of spironolactone-induced hepatitis.

Title: A One-Two Punch: Hydralazine-Induced Liver Injury in a Recovering Ischemic Hepatitis.
A 77-year-old woman presented to the emergency department with a 2-day history of nausea and vomiting. Her medical history included diabetes mellitus, hypertension, atrial fibrillation, dilated cardiomyopathy, and coronary artery disease. Her home medications included aspirin, clopidogrel, warfarin, digoxin, metoprolol, losartan, simvastatin, isosorbide dinitrate, furosemide, and spironolactone. Initial physical examination showed blood pressure of 170/80 mm Hg with a heart rate of 69 beats per minute, otherwise unremarkable. Initial laboratory workup was significant for INR of 3.6, with slightly elevated troponin I and creatinine of 0.06 ng/mL and 1.4 mg/dL, respectively. The patient was admitted to the medicine floor. However,... (truncated)

Title: The Effects of Spironolactone in Preventing Bile Duct Ligation-induced Hepatitis in A Rat Model.
Cholestasis is associated with the accumulation of bile acids and bilirubin in the hepatocytes and leads to liver injury. Pregnane X Receptor (PXR) coordinates protective hepatic responses to toxic stimuli, and this receptor was reported to stimulate bile secretion by increasing MRP2 expression. Since PXR activators were reported to be anti-inflammatory in the liver, PXR was proposed as a drug target for the treatment of chronic inflammatory liver diseases. We investigated the potential protective effect of spironolactone (SPL), an enzyme inducer, in hepatotoxicity induced by bile duct ligation in rats. Wistar Albino (250-300 g) rats were divided into the control... (truncated)

Title: Spironolactone Effect in Hepatic Ischemia/Reperfusion Injury in Wistar Rats.
Ischemia/reperfusion (IR) injury, often associated with liver surgery, is an unresolved problem in the clinical practice. Spironolactone is an antagonist of aldosterone that has shown benefits over IR injury in several tissues, but its effects in hepatic IR are unknown.

Title: Efficacy of Spironolactone as an Adjunctive Therapy to Risperidone to Improve Symptoms of Schizophrenia: A Double-Blind, Randomized, Placebo-Controlled, Clinical Trial.
Objective: Spironolactone (C24H32O4S), a potent mineralocorticoid receptor (MR) inhibitor, is a potassium-sparing diuretic that is traditionally used to treat fluid build-up in the body or for its anti-androgenic properties. This study is a double-blind, placebo-controlled, randomized clinical trial assessing the beneficial effects of spironolactone in addition to risperidone in improving negative symptoms of schizophrenia. Method: 40 patients with chronic schizophrenia, aged 18-60 years, were assigned to two groups: risperidone + spironolactone or risperidone + placebo. Risperidone was administered to both the spironolactone and placebo groups with a dose up to 6 mg/day throughout the trial. Spironolactone (C24H32O4S) was ordered 100... (truncated)

MESH:D004110 - diltiazem

Summary:

LLM Prediction Score	0.326 (normalized)
LLM Confidence Score	0.990
Golden Answer (Severity Class)	0.5 (normalized)
Prediction Error	0.174

Retrieved Context:

Title: Considerations for safe use of statins: liver enzyme abnormalities and muscle toxicity.
Statins play an important role in the care of patients with cardiovascular disease and have a good safety record in clinical practice. The risk of hepatic injury caused by statins is estimated to be about 1 percent, similar to that of patients taking a placebo. Patients with transaminase levels no more than three times the upper limit of normal can continue taking statins; often the elevations will resolve spontaneously. Coexisting elevations of transaminase levels from nonalcoholic fatty liver disease and stable hepatitis B and C viral infections are not contra- indications to statin use. Although myalgias are common with statin... (truncated)

Title: Effect of subclinical, clinical and supraclinical doses of calcium channel blockers on models of drug-induced hepatotoxicity in rats.
Drug-related hepatotoxicity is the leading cause of acute liver failure, and hepatic problems are responsible for a significant number of liver transplantations and deaths worldwide. Calcium has been associated with various metabolic processes that lead to cell death and apoptosis, and increased cytosolic Ca(2+) has been implicated in hepatotoxicity. This study was designed to investigate the effects of calcium channel blockers (CCBs) on isoniazid-rifampicin, zidovudine and erythromycin-induced hepatotoxicity in rats. Treatment groups comprised control, hepatotoxicant, hepatotoxicant along with each of silymarin, nifedipine, verapamil and diltiazem at subclinical, clinical and supraclinical doses. A day to the end of treatment for each... (truncated)

Title: Simvastatin-diltiazem drug interaction resulting in rhabdomyolysis and hepatitis.
Simvastatin, a hydroxymethyl glutarate coenzyme A (HMG-CoA) reductase inhibitor, is a commonly used cholesterol lowering agent. The long-term safety profile of simvastatin, established over ten-years of clinical use, is excellent. Both rhabdomyolysis and hepatitis, however, are recognized toxic effects of this medication, and generally occur when the patients are taking more than 40 mg of simvastatin a day. Potent inhibitors of the cytochrome P450 3A4 (CYP3A4) enzyme increase the incidence of simvastatin toxicity. Calcium channel blockers are weak inhibitors of the CYP3A4 enzyme. Diltiazem is known to increase the serum concentration of simvastatin. Many patients who take both simvastatin and... (truncated)

Title: Toxic effects of diltiazem in a patient with chronic renal failure.
No abstract available.

Title: Protective effect of carvedilol alone and coadministered with diltiazem and prednisolone on doxorubicin and 5-fluorouracil-induced hepatotoxicity and nephrotoxicity in rats.
This study investigated the protective effects of carvedilol alone and coadministered with prednisolone and diltiazem on doxorubicin (DOX) and 5-fluorouracil (5-FU)-induced toxicity. Each of 2 pools of 70 female rats were randomly allotted into 10 groups of 7 animals each and treated as follows: Group 1: normal saline (10 mL/kg); Group 2: normal saline and DOX (40 mg/kg)/5-FU (20 mg/kg) alone; Group 3: gallic acid (200 mg/kg) and DOX/5-FU; Group 4: carvedilol (0.075 mg/kg) and DOX/5-FU; Group 5: carvedilol (0.15 mg/kg) and DOX/5-FU; Group 6: carvedilol (0.30 mg/kg) and DOX/5-FU; Group 7: diltiazem (3.43 mg/kg) and DOX/5-FU; Group 8: diltiazem... (truncated)

MESH:C106791 - telithromycin

Summary:

LLM Prediction Score	0.828 (normalized)
LLM Confidence Score	0.950
Golden Answer (Severity Class)	1.0 (normalized)
Prediction Error	0.172

Retrieved Context:

Title: [Hepatotoxicity by antibiotics: update in 2008].
Although antibiotics are the most commonly incriminated drugs in instances of hepatotoxicity in medical literature. However, it is mainly due to its wide prescription and the absolute risk of hepatotoxicity related to antibiotic use is thought to be low. Nevertheless, among the different penicillins, amoxicillin-clavulanate is the single leading drug involved in hepatotoxicity in cohorts of patients with drug-induced liver injury (DILI), representing between 12.8% to 14% of the cases. It is the most frequent cause of hospitalization for DILI. The incidence of amoxicillin-clavulanate induced hepatotoxicity has been estimated to be 9.91 per 100,000 users and its clinical presentation varies,... (truncated)

Title: Telithromycin: review of adverse effects.
Telithromycin is a macrolide antibiotic that has been marketed since the early 2000s. It has not been shown to be more effective against any bacteria than other macrolide antibiotics. Its antibacterial activity is in no way remarkable. In early 2014, we reviewed its adverse effect profile using data from periodic safety update reports, drug regulatory agencies, and detailed published case reports. In addition to the adverse effect profile telithromycin shares with the other macrolides, it provokes several specific adverse effects: visual disturbances due to impaired accommodation; taste and smell disorders; severe liver damage; worsening of myasthenia gravis; rhabdomyolysis; and loss... (truncated)

Title: Hepatic safety of antibiotics used in primary care.
Antibiotics used by general practitioners frequently appear in adverse-event reports of drug-induced hepatotoxicity. Most cases are idiosyncratic (the adverse reaction cannot be predicted from the drug's pharmacological profile or from pre-clinical toxicology tests) and occur via an immunological reaction or in response to the presence of hepatotoxic metabolites. With the exception of trovafloxacin and telithromycin (now severely restricted), hepatotoxicity crude incidence remains globally low but variable. Thus, amoxicillin/clavulanate and co-trimoxazole, as well as flucloxacillin, cause hepatotoxic reactions at rates that make them visible in general practice (cases are often isolated, may have a delayed onset, sometimes appear only after cessation... (truncated)

Title: Cellular imaging predictions of clinical drug-induced liver injury.
Drug-induced liver injury (DILI) is the most common adverse event causing drug nonapprovals and drug withdrawals. Using drugs as test agents and measuring a panel of cellular phenotypes that are directly linked to key mechanisms of hepatotoxicity, we have developed an in vitro testing strategy that is predictive of many clinical outcomes of DILI. Mitochondrial damage, oxidative stress, and intracellular glutathione, all measured by high content cellular imaging in primary human hepatocyte cultures, are the three most important features contributing to the hepatotoxicity prediction. When applied to over 300 drugs and chemicals including many that caused rare and idiosyncratic liver... (truncated)

Title: Analyzing the Mechanisms Behind Macrolide Antibiotic-Induced Liver Injury Using Quantitative Systems Toxicology Modeling.
Macrolide antibiotics are commonly prescribed treatments for drug-resistant bacterial infections; however, many macrolides have been shown to cause liver enzyme elevations and one macrolide, telithromycin, has been pulled from the market by its provider due to liver toxicity. This work seeks to assess the mechanisms responsible for the toxicity of macrolide antibiotics.

MESH:D000077265 - donepezil

Summary:

LLM Prediction Score	0.203 (normalized)
LLM Confidence Score	0.990
Golden Answer (Severity Class)	0.375 (normalized)
Prediction Error	0.172

Retrieved Context:

Title: Donepezil: a clinical review of current and emerging indications.
This article reviews the piperidine derivative, donepezil hydrochloride (E2020, Aricept), a reversible central acetylcholinesterase inhibitor currently approved for treatment of mild-to-moderate Alzheimer's disease. Donepezil is well absorbed orally, unaffected by food or by time of administration; it reaches therapeutic levels in doses of 5-10 mg/day and peak plasma concentrations are obtained 3-4 h after oral administration. A single bedtime dose is recommended due to the long elimination half-life of the drug (70 h). Donepezil does not cause liver toxicity or significant drug interactions and is relatively well-tolerated. Initial side effects include nausea, vomiting, diarrhoea, insomnia, muscle cramps, fatigue, anorexia and... (truncated)

Title: Treatment of cognitive impairment in Alzheimer's disease.
In Alzheimer's disease, cognition now responds to several drugs. Anticholinesterases target the acetylcholine deficit. In mild-to-moderate Alzheimer's disease, they all provide significant benefit versus placebo on the Alzheimer's Disease Assessment ScheduleCognitive Section (ADAS-Cog), Side effects, in 5% to 15% of cases, include nausea, vomiting, diarrhea, anorexia, and dizziness. Tacrine, the leading anticholinesterase, caused frequent hepatic enzyme elevation and was withdrawn; once-daily donepezil spares the liver and improves global measures of change in severe dementia; rivasiigmine is indicated in comorbid vascular disease; while galaniamine modulates the cerebral nicotinic acetylcholine receptors that potentiate the response to acetylcholine. Alternative agents include the N-methyl-D-aspartate... (truncated)

Title: In Vitro Metabolism of Donepezil in Liver Microsomes Using Non-Targeted Metabolomics.
Donepezil is a reversible acetylcholinesterase inhibitor that is currently the most commonly prescribed drug for the treatment of Alzheimer's disease. In general, donepezil is known as a safe and well-tolerated drug, and it was not associated with liver abnormalities in several clinical trials. However, rare cases of drug-related liver toxicity have been reported since it has become commercially available. Few studies have investigated the metabolic profile of donepezil, and the mechanism of liver damage caused by donepezil has not been elucidated. In this study, the in vitro metabolism of donepezil was investigated using liquid chromatography-tandem mass spectrometry based on a... (truncated)

Title: Donepezil-related toxic hepatitis.
Alzheimer's disease (AD) is the leading cause of dementia. It is characterized by the presence of senile plaques and neurofibrillary tangles in the brain, and impairment of the central cholinergic system, which contribute to memory loss and cognitive dysfunction. Cholinesterase inhibitors prevent the hydrolysis of acetylcholine and are currently approved for the symptomatic treatment of Alzheimer's disease. Donepezil, a piperidine-based, reversible and specific inhibitor of acetylcholinesterase, has been demonstrated to be clinically effective in the treatment of patients with mild to moderate AD. To date, clinical trials have not reported an association between treatment with donepezil and hepatotoxicity. We describe... (truncated)

Title: Perspectives in the management of Alzheimer's disease: clinical profile of donepezil.
Donepezil HCl is a piperidine-based reversible acetylcholinesterase (AChE) inhibitor, chemically distinct from other cholinesterase (ChE) inhibitors and rationally designed to treat the symptoms of Alzheimer's disease (AD). It is highly selective for AChE in the central nervous system (CNS), with little or no affinity for butyrylcholinesterase (BuChE). In preclinical studies in animals, donepezil produced increased CNS acetylcholine. The resultant enhancement of cholinergic activity gave rise to improved performance by rats on tests of learning and memory, with no evidence of hepatic or renal toxicity. In subsequent phase I clinical evaluations in healthy volunteers, donepezil demonstrated favorable pharmacokinetic, pharmacodynamic and safety... (truncated)

MESH:C422802 - ibritumomab tiuxetan

Summary:

LLM Prediction Score	0.171 (normalized)
LLM Confidence Score	0.900
Golden Answer (Severity Class)	0.0 (normalized)
Prediction Error	0.171

Retrieved Context:

Title: High activity 90Y-ibritumomab tiuxetan (Zevalin) with peripheral blood progenitor cells support in patients with refractory/resistant B-cell non-Hodgkin lymphomas.
Radioimmunotherapy (RIT) is an alternative approach in the treatment of resistant/refractory B-cell non-Hodgkin lymphoma (NHL). We performed a feasibility and toxicity pilot study of escalating activity of 90Y-ibritumomab tiuxetan followed by autologous stem cell transplantation (ASCT). Three activity levels were fixed--30 MBq/kg (0.8 mCi/kg), 45 MBq/kg (1.2 mCi/kg) and 56 MBq/kg (1.5 mCi/kg)--and 13 patients enrolled. One week before treatment all patients underwent dosimetry. ASCT was performed 13 d after Zevalin administration. Treatment was well tolerated and all patients engrafted promptly. No differences in terms of haematological toxicities were observed among the three levels, apart from a delayed platelet recovery... (truncated)

Title: Y⁹⁰ -Ibritumomab tiuxetan (Y⁹⁰ -IT) and high-dose melphalan as conditioning regimen before autologous stem cell transplantation for elderly patients with lymphoma in relapse or resistant to chemotherapy: a feasibility trial (SAKK 37/05).
Standard conditioning regimens for autologous stem cell transplantation (ASCT) are often not tolerated by elderly patients, on one hand. Single high-dose melphalan, on the other hand, has been shown to be safe and active as a pretransplant preparative regimen in elderly patients. Y⁹⁰ -Ibritumomab tiuxetan (Y⁹⁰ -IT) is well tolerated and feasible in the transplantation setting. We therefore investigated the combination of high-dose melphalan and Y⁹⁰ -IT as a conditioning regimen for patients ≥65 years of age. Patients with relapsed or resistant CD20-positive lymphoma in remission after salvage chemotherapy could be enrolled. High-dose therapy consisted of standard dose Y⁹⁰ -IT (0.4-mCi/kg... (truncated)

Title: Megadose 90Y-ibritumomab tiuxetan prior to allogeneic transplantation is effective for aggressive large B-cell lymphoma.
Allogeneic hematopoietic cell transplantation (allo-HCT) can be curative for relapsed or refractory B-cell lymphomas (BCLs), although outcomes are worse in aggressive disease, and most patients will still experience relapse. Radioimmunotherapy using 90Y-ibritumomab tiuxetan can induce disease control across lymphoma subtypes in a dose-dependent fashion. We hypothesized that megadoses of 90Y-ibritumomab tiuxetan with reduced-intensity conditioning could safely produce deeper remissions in aggressive BCL further maintained with the immunologic effect of allo-HCT. In this phase 2 study, CD20+ BCL patients received outpatient 90Y-ibritumomab tiuxetan (1.5 mCi/kg; maximum, 120 mCi), fludarabine, and then 2 Gy total body irradiation before HLA-matched allo-HCT. Twenty patients... (truncated)

Title: Phase II Study of Yttrium-90 Ibritumomab Tiuxetan Plus High-Dose BCNU, Etoposide, Cytarabine, and Melphalan for Non-Hodgkin Lymphoma: The Role of Histology.
Standard-dose ⁹⁰yttrium-ibritumomab tiuxetan (.4 mci/kg) together with high-dose BEAM (BCNU, etoposide, cytarabine, and melphalan) (Z-BEAM) has been shown to be a well-tolerated autologous hematopoietic stem cell transplantation preparative regimen for non-Hodgkin lymphoma. We report the outcomes of a single-center, single-arm phase II trial of Z-BEAM conditioning in high-risk CD20⁺ non-Hodgkin lymphoma histologic strata: diffuse large B cell (DLBCL), mantle cell, follicular, and transformed. Robust overall survival and notably low nonrelapse mortality rates (.9% at day +100 for the entire cohort), with few short- and long-term toxicities, confirm the safety and tolerability of the regimen. In addition, despite a high proportion... (truncated)

Title: Radioimmunotherapy of B-Cell Non-Hodgkin's Lymphoma.
This manuscript reviews current advances in the use of radioimmunotherapy (RIT) for the treatment of B-cell non-Hodgkin's lymphoma (NHL). RIT has been in use for more than 20 years and has progressed significantly with the discovery of new molecular targets, the development of new stable chelates, the humanization of monoclonal antibodies

MESH:D008736 - methyclothiazide

Summary:

LLM Prediction Score	0.080 (normalized)
LLM Confidence Score	0.720
Golden Answer (Severity Class)	0.25 (normalized)
Prediction Error	0.170

Retrieved Context:

Title: Data-driven identification of structural alerts for mitigating the risk of drug-induced human liver injuries.
The use of structural alerts to de-prioritize compounds with undesirable features as drug candidates has been gaining in popularity. Hundreds of molecular structural moieties have been proposed as structural alerts. An emerging issue is that strict application of these alerts will result in a significant reduction of the chemistry space for new drug discovery, as more than half of the oral drugs on the market match at least one of the alerts. To mitigate this issue, we propose to apply a rigorous statistical analysis to derive/validate structural alerts before use.

Title: Fluorescent molecularly imprinted polymer nanocomposite for solid-phase extraction and fluorimetric determination of hydrochlorothiazide.
We report herein a fluorescent molecularly imprinted polymer (FMIP) for the solid-phase extraction (SPE) and fluorimetric determination of hydrochlorothiazide (HCTZ) in water. The FMIP is based on fluorescent polystyrene nanoparticles embedded within a molecularly imprinted polyaniline (PANI) matrix. The operational adsorption parameters such as the initial HCTZ concentration, incubation time and the solution pH were found to influence the removal efficiency. At optimum conditions, a high adsorption capacity of the FMIP was found (2.08 mg g⁻¹). Evidence of the adsorption process was confirmed by the change in the FMIP physicochemical properties measured by FTIR absorption spectroscopy and electron microscopy. Based... (truncated)

Title: 20th ISoP Annual Meeting "Integrated pharmacovigilance for safer patients" 8-10 November 2021 Muscat, Oman (Hybrid meeting).
No abstract available.

Title: The frequency of photosensitizing drug dispensings in Austria and Germany: a correlation with their photosensitizing potential based on published literature.
Drug-induced photosensitivity refers to the development of cutaneous adverse events due to interaction between a pharmaceutical compound and sunlight. Although photosensitivity is a very commonly listed side-effect of systemic drugs, reliable data on its actual incidence are lacking so far.

MESH:C108475 - pitavastatin

Summary:

LLM Prediction Score	0.206 (normalized)
LLM Confidence Score	0.970
Golden Answer (Severity Class)	0.375 (normalized)
Prediction Error	0.169

Retrieved Context:

Title: Pitavastatin up-regulates the induction of iNOS through enhanced stabilization of its mRNA in pro-inflammatory cytokine-stimulated hepatocytes.
Studies have indicated that protective effects of statins (HMG-CoA reductase inhibitor) are associated with the regulation of endothelial nitric oxide synthase (eNOS) or inducible NOS (iNOS) in heart and liver diseases. Statins have been reported to enhance hepatic NO production and decrease the vascular tone in patients with cirrhosis. However, it is unclear which NOS contributes to the increased NO production. We hypothesized that statins are involved in the up-regulation of iNOS in inflammatory liver, resulting in decreased hepatic resistance. Primary cultured rat hepatocytes were treated with pro-inflammatory cytokine interleukin (IL)-1beta in the presence or absence of pitavastatin. Pretreatment of... (truncated)

Title: Pitavastatin: evidence for its place in treatment of hypercholesterolemia.
Statins, inhibitors of 3-hydroxy-3-methylglutaryl-coenzyme A reductase, are the most potent pharmacologic agents for lowering total cholesterol (TC) and low-density lipoprotein cholesterol (LDL-C). They have become an accepted standard of care in the treatment of patients with known atherosclerotic cardiovascular disease (secondary prevention) and also those at increased risk of cardiovascular events. There are currently six statin drugs commercially available in the US. Although they are chemically similar and have the same primary mechanisms of action in lowering TC and LDL-C, there are differences in their efficacy or potency, metabolism, drug-drug interactions, and individual tolerability. Considering the numbers of patients who... (truncated)

Title: Additional Treatment with Fenofibrate for Patients Treated with Pitavastatin Under Ordinary Medical Practice for Hypertriglyceridemia in Japan (APPROACH-J Study).
Safety and efficacy of combination therapy of pitavastatin and fenofibrate were examined in consecutive case series with fasting serum triglycerides ≥ 150 mg/dL despite receiving pitavastatin 1 or 2 mg daily for over 2 months and additionally administered micronized fenofibrate 67 mg daily for another 4 to 16 weeks. Such low doses were selected in consideration of safety, and normal liver and renal functions were incorporated in inclusion criteria. In result, a total of 56 cases were examined. The addition of fenofibrate 67 mg to pitavastatin 1 mg/2 mg yielded a 36.8%/35.6% reduction in triglycerides and 6.4%/12.4% elevation in high-density... (truncated)

Title: Comparison of effects of pitavastatin and atorvastatin on plasma coenzyme Q10 in heterozygous familial hypercholesterolemia: results from a crossover study.
An open, randomized, four-phased crossover study using 4 mg of pitavastatin or 20 mg of atorvastatin was performed to compare their efficacy and safety, especially regarding plasma levels of coenzyme Q10 (CoQ10) in 19 Japanese patients with heterozygous familial hypercholesterolemia. Pitavastatin and atorvastatin caused significant and almost comparable reductions in serum levels of total cholesterol (-35.4 vs. -33.8%), low-density lipoprotein cholesterol (-42.8 vs. -40.7%), and triglyceride (-26.1 vs. -29.4%), and significantly increased serum levels of high-density lipoprotein cholesterol (12.1 vs. 11.4%). Under these conditions, plasma levels of CoQ10 were reduced by atorvastatin (-26.1%, P=0.0007) but not by pitavastatin (-7.7%, P=0.39),... (truncated)

Title: Diabetogenic effect of pravastatin is associated with insulin resistance and myotoxicity in hypercholesterolemic mice.
Label="BACKGROUND">HMG-CoA reductase inhibitors (statins) are cholesterol-lowering drugs widely used to treat hypercholesterolemia and prevent cardiovascular disease. Statins are generally well tolerated, but adverse reactions may occur, particularly myopathy and new onset of diabetes. The exact mechanism of statin-induced myopathy and diabetes has not been fully elucidated. We have previously shown that treatment of hypercholesterolemic (LDLr^{-/-}) mice with pravastatin for 2 months decreased pancreatic islet insulin secretion and increased oxidative stress and cell death, but no glucose intolerance was observed. The purpose of the current work was to study long-term pravastatin effects on glucose homeostasis, insulin sensitivity, muscle protein turnover and cell... (truncated)

MESH:D004837 - epinephrine

Summary:

LLM Prediction Score	0.168 (normalized)
LLM Confidence Score	0.990
Golden Answer (Severity Class)	0.0 (normalized)
Prediction Error	0.168

Retrieved Context:

Title: Traumatic hemobilia: a complication of percutaneous liver biopsy.
Two patients with hemobilia are presented. The first patient, with alcoholic liver disease, had a percutaneous liver biopsy. Subsequently he developed jaundice, with an enlarged tender gallbladder, biliary colic, and gastrointestinal bleeding. Hemobilia was demonstrated by superselective hepatic angiography and bleeding was stopped by intraarterial infusion of epinephrine and propranolol. The second patient, with primary biliary cirrhosis at an advanced stage, had a percutaneous liver biopsy followed by gastrointestinal bleeding, severe abdominal pain, and finally death. In both cases hemobilia was suggested by gastroduodenoscopy.

Title: Sympathetic nervous system catecholamines and neuropeptide Y neurotransmitters are upregulated in human NAFLD and modulate the fibrogenic function of hepatic stellate cells.
Sympathetic nervous system (SNS) signalling regulates murine hepatic fibrogenesis through effects on hepatic stellate cells (HSC), and obesity-related hypertension with SNS activation accelerates progression of non-alcoholic fatty liver disease (NAFLD), the commonest cause of chronic liver disease. NAFLD may lead to cirrhosis. The effects of the SNS neurotransmitters norepinephrine (NE), epinephrine (EPI) and neuropeptide Y (NPY) on human primary HSC (hHSC) function and in NAFLD pathogenesis are poorly understood.

Title: Sympathomimetic amine compounds and hepatotoxicity: Not all are alike-Key distinctions noted in a short review.
Sympathomimetic amine compounds are often pooled together and incorrectly assumed to be interchangeable with respect to potential adverse effects. A brief and specific review of sympathomimetic compounds and one instance (i.e., hepatotoxicity) where these compounds have been improperly grouped together is covered. A review of the proposed mechanisms through which known hepatotoxic sympathomimetic agents (e.g., 3,4-methylenedioxymethamphetamine or MDMA, methamphetamine and amphetamine) cause liver injury, along with a corresponding review of in vitro data, interventional data, animal model studies and observational data allow for a comparison/contrast of different agents and reveals a lack of potential toxicity for some agents (e.g., pseudoephedrine,... (truncated)

Title: The Role of Catecholamines in Pathophysiological Liver Processes.
Over the last few years, the number of research publications about the role of catecholamines (epinephrine, norepinephrine, and dopamine) in the development of liver diseases such as liver fibrosis, fatty liver diseases, or liver cancers is constantly increasing. However, the mechanisms involved in these effects are not well understood. In this review, we first recapitulate the way the liver is in contact with catecholamines and consider liver implications in their metabolism. A focus on the expression of the adrenergic and dopaminergic receptors by the liver cells is also discussed. Involvement of catecholamines in physiological (glucose metabolism, lipids metabolism, and liver... (truncated)

Title: A case of idiopathic anaphylaxis followed by acute liver injury.
Idiopathic anaphylaxis is characterized by recurrent anaphylaxis without a known trigger. The coexistence of acute liver injury with idiopathic anaphylaxis is rare, even in cases of severe anaphylaxis such as shock. An unusual case involving repeated episodes of anaphylactic shock accompanied by acute liver injury is described here. A 36-year-old woman who experienced anaphylaxis due to an unknown cause was referred to our hospital because of marked elevations in her liver enzyme levels. After a thorough evaluation to determine the cause of the acute liver injury, viral infection, drug use, and autoimmune hepatitis were excluded. The episodes were accompanied by... (truncated)

MESH:D013411 - sulfadiazine

Summary:

LLM Prediction Score	0.543 (normalized)
LLM Confidence Score	0.980
Golden Answer (Severity Class)	0.375 (normalized)
Prediction Error	0.168

Retrieved Context:

Title: Case report: Sulfasalazine-induced hypersensitivity.
Drug-induced hypersensitivity syndrome (DiHS)/drug reaction with eosinophilia and systemic symptoms (DRESS) is a systemic inflammatory condition that is characterized by multisystemic involvement (liver, blood, and skin), heterogeneous manifestations (fever, rash, lymphadenopathy, and eosinophilia), and an unpredictable course; cases of DiHS/DRESS caused by sulfasalazine are rare in children compared to adults. We report a case of a 12-year-old girl with juvenile idiopathic arthritis (JIA) and sulfasalazine-related hypersensitivity who developed fever, rash, blood abnormalities, and hepatitis complicated with hypocoagulation. The treatment with intravenous and then oral glucocorticosteroids was effective. We also reviewed 15 cases (67% male patients) of childhood-onset sulfasalazine-related DiHS/DRESS from... (truncated)

Title: Idiosyncratic NSAID drug induced oxidative stress.
Many idiosyncratic non-steroidal anti-inflammatory drugs (NSAIDs) cause GI, liver and bone marrow toxicity in some patients which results in GI bleeding/ulceration/fulminant hepatic failure/hepatitis or agranulocytosis/aplastic anemia. The toxic mechanisms proposed have been reviewed. Evidence is presented showing that idiosyncratic NSAID drugs form prooxidant radicals when metabolised by peroxidases known to be present in these tissues. Thus GSH, NADH and/or ascorbate were cooxidised by catalytic amounts of NSAIDs and hydrogen peroxide in the presence of peroxidase. During GSH and NADH cooxidation, oxygen uptake and activation occurred. Furthermore the formation of NSAID oxidation products was prevented during the cooxidation indicating that the... (truncated)

Title: Idiosyncratic toxicity associated with potentiated sulfonamides in the dog.
Idiosyncratic toxicity to potentiated sulfonamides occurs in both humans and dogs, with considerable clinical similarities. The syndrome in dogs can consist of fever, arthropathy, blood dyscrasias (neutropenia, thrombocytopenia, or hemolytic anemia), hepatopathy consisting of cholestasis or necrosis, skin eruptions, uveitis, or keratoconjunctivitis sicca. Other manifestations seen less commonly include protein-losing nephropathy, meningitis, pancreatitis, pneumonitis, or facial nerve palsy. The pathogenesis of these reactions is not completely understood, but may be due to a T-cell-mediated response to proteins haptenated by oxidative sulfonamide metabolites. Our laboratory is working on tests to characterize dogs with possible idiosyncratic sulfonamide reactions, to include ELISA for... (truncated)

Title: Anti-<i>Toxoplasma</i> activity of silver nanoparticles green synthesized with <i>Phoenix dactylifera</i> and <i>Ziziphus spina-christi</i> extracts which inhibits inflammation through liver regulation of cytokines in Balb/c mice.
Toxoplasmosis constitutes a global infection caused by oblige intracellular apicomplexan protozoan parasite <i>Toxoplasma gondii</i> Although often asymptomatic, infection can result in more severe, potentially life threatening symptoms particularly in immunocompromised individuals. The present study evaluated the anti-<i>Toxoplasma</i> effects in experimental animals of silver nanoparticles synthesized in combination with extracts of natural plants (<i>Phoenix dactylifera</i> and <i>Ziziphus spina-christi</i>) as an alternative method to standard sulfadiazine drug therapy. Liver functions estimated by and AST and ALT were significantly increased in <i>T. gondii</i>-infected mice compared with the control group as well as hepatic nitric oxide (NO), lipid peroxidation (LPO) levels and caused significant... (truncated)

Title: In Vitro and in Vivo Effects of Nitrofurantoin on Experimental Toxoplasmosis.
Toxoplasma gondii is an important opportunistic pathogen that causes toxoplasmosis, which has very few therapeutic treatment options. The most effective therapy is a combination of pyrimethamine and sulfadiazine; however, their utility is limited because of drug toxicity and serious side effects. For these reasons, new drugs with lower toxicity are urgently needed. In this study, the compound, (Z)-1-[(5-nitrofuran-2-yl)methyleneamino]-imidazolidine-2,4-dione (nitrofurantoin), showed anti-T. gondii effects in vitro and in vivo. In HeLa cells, the selectivity of nitrofurantoin was 2.3, which was greater than that of pyrimethamine (0.9). In T. gondii-infected female ICR mice, the inhibition rate of T. gondii growth in the...

MESH:C409045 - almotriptan

Summary:

LLM Prediction Score	0.167 (normalized)
LLM Confidence Score	0.950
Golden Answer (Severity Class)	0.0 (normalized)
Prediction Error	0.167

Retrieved Context:

Title: Association of CYP1A1 and CYP1B1 inhibition in in vitro assays with drug-induced liver injury.
Drug-induced liver injury (DILI) is one of the major causes for the discontinuation of drug development and withdrawal of drugs from the market. Since it is known that reactive metabolite formation and being substrates or inhibitors of cytochrome P450s (P450s) are associated with DILI, we systematically investigated the association between human P450 inhibition and DILI. The inhibitory activity of 266 DILI-positive drugs (DILI drugs) and 92 DILI-negative drugs (no-DILI drugs), which were selected from Liver Toxicity Knowledge Base (US Food and Drug Administration), against 8 human P450 forms was assessed using recombinant enzymes and luminescent substrates, and the threshold values... (truncated)

Title: Efficacy of frovatriptan as compared to other triptans in migraine with aura.
The treatment of migraine attacks with aura by triptans is difficult since triptans most probably are not efficacious when taken during the aura phase. Moreover, there are insufficient data from randomised studies whether triptans are efficacious in migraine attacks with aura when taken during the headache phase. In this metaanalysis, we aimed to compare the efficacy of frovatriptan versus rizatriptan, zolmitriptan, and almotriptan.

Title: New drugs for migraine.
After the triptans, a calcitonin gene-related peptide blocker (telcagepant) is the first acute medicine that has been developed primarily for treatment of acute migraine. Otherwise, the new drugs have been developed first for other purposes, like anticonvulsants, antihypertensives and antidepressants used for migraine prophylaxis. For acute attacks, a new way to administer a traditional drug like dihydroergotamine is under way, and documentation of efficacy in migraine has been gained for some commonly used painkillers and anti-inflammatory drugs, and for some herbal extracts. Based on insights into the basic pathophysiological mechanisms of the disorder, some drugs have been developed which seem... (truncated)

Title: Data-driven identification of structural alerts for mitigating the risk of drug-induced human liver injuries.
The use of structural alerts to de-prioritize compounds with undesirable features as drug candidates has been gaining in popularity. Hundreds of molecular structural moieties have been proposed as structural alerts. An emerging issue is that strict application of these alerts will result in a significant reduction of the chemistry space for new drug discovery, as more than half of the oral drugs on the market match at least one of the alerts. To mitigate this issue, we propose to apply a rigorous statistical analysis to derive/validate structural alerts before use.

Title: Pharmacokinetics and Pharmacodynamics of Intranasal Solid Lipid Nanoparticles and Nanostructured Lipid Carriers for Nose-to-Brain Delivery.
Nose-to-brain drug delivery has been of great interest for the treatment of many central nervous system (CNS) diseases and psychiatric disorders over past decades. Several nasally administered formulations have been developed to circumvent the blood-brain barrier and directly deliver drugs to the CNS through the olfactory and trigeminal pathways. However, the nasal mucosa's drug absorption is insufficient and the volume of the nasal cavity is small, which, in combination, make nose-to-brain drug delivery challenging. These problems could be minimized using formulations based on solid lipid nanoparticles (SLNs) or nanostructured lipid carriers (NLCs), which are effective nose-to-brain drug delivery systems that... (truncated)

MESH:D008653 - mesoridazine

Summary:

LLM Prediction Score	0.459 (normalized)
LLM Confidence Score	0.950
Golden Answer (Severity Class)	0.625 (normalized)
Prediction Error	0.166

Retrieved Context:

Title: Evaluating the safety, tolerability, pharmacokinetics and efficacy of clofazimine in cryptosporidiosis (CRYPTOFAZ): study protocol for a randomized controlled trial.
Cryptosporidium infection and diarrhea (cryptosporidiosis) is a life-threatening infection in persons with HIV and also in children of 6-18 months of age in the developing world. To date, only nitazoxanide is licensed for treatment of cryptosporidiosis, and only in persons after the first year of life and with healthy immune systems. Clofazimine (CFZ: Lamprene®), an established drug that has been used for leprosy for more than 50 years, recently has been described as effective against Cryptosporidium in vitro and in mouse infections. The efficacy and pharmacokinetics of CFZ in vivo, in HIV-infected patients with cryptosporidial diarrhea are not known.

Title: Drug Repurposing for the Management of Depression: Where Do We Stand Currently?
A slow rate of new drug discovery and higher costs of new drug development attracted the attention of scientists and physicians for the repurposing and repositioning of old medications. Experimental studies and off-label use of drugs have helped drive data for further studies of approving these medications. A deeper understanding of the pathogenesis of depression encourages novel discoveries through drug repurposing and drug repositioning to treat depression. In addition to reducing neurotransmitters like epinephrine and serotonin, other mechanisms such as inflammation, insufficient blood supply, and neurotoxicants are now considered as the possible involved mechanisms. Considering the mentioned mechanisms has resulted... (truncated)

Title: Mebendazole Inhibits <i>Histoplasma capsulatum</i> In Vitro Growth and Decreases Mitochondrion and Cytoskeleton Protein Levels.
Histoplasmosis is a frequent mycosis in people living with HIV/AIDS and other immunocompromised hosts. Histoplasmosis has high rates of mortality in these patients if treatment is unsuccessful. Itraconazole and amphotericin B are used to treat histoplasmosis; however, both antifungals have potentially severe pharmacokinetic drug interactions and toxicity. The present study determined the minimal inhibitory and fungicidal concentrations of mebendazole, a drug present in the NIH Clinical Collection, to establish whether it has fungicidal or fungistatic activity against <i>Histoplasma capsulatum</i>. Protein extracts from <i>H. capsulatum</i> yeasts, treated or not with mebendazole, were analyzed by proteomics to understand the metabolic changes driven... (truncated)

Title: Management of Systemic Medical Emergencies Associated with Psychotropic Medications.
No abstract available.

Title: Adverse Effects and Toxicity of the Atypical Antipsychotics: What is Important for the Pediatric Emergency Medicine Practitioner.
Medications are being used with greater frequency to address pediatric mental health problems, and in recent years atypical antipsychotic (AAP) prescriptions have increased more than any other class. Acute care practitioners must be aware of the pharmacology of AAPs and the conditions, on- and off-label, for which they are prescribed. This involves identifying and managing side effects that manifest both mentally and physically. Although "atypicality" confers a lower risk of movement side effects compared to conventional agents, children are more sensitive than adults to extrapyramidal reactions. Like adults, they also may present with toxic sedation, confusion, cardiovascular dysfunction, and metabolic... (truncated)

MESH:C082598 - aldesleukin

Summary:

LLM Prediction Score	0.209 (normalized)
LLM Confidence Score	0.970
Golden Answer (Severity Class)	0.375 (normalized)
Prediction Error	0.166

Retrieved Context:

Title: Low-dose interleukin-2 in patients with stable ischaemic heart disease and acute coronary syndromes (LILACS): protocol and study rationale for a randomised, double-blind, placebo-controlled, phase I/II clinical trial.
Label="INTRODUCTION">Inflammation and dysregulated immune responses play a crucial role in atherosclerosis, underlying ischaemic heart disease (IHD) and acute coronary syndromes (ACSs). Immune responses are also major determinants of the postischaemic injury in myocardial infarction. Regulatory T cells (CD4⁺CD25⁺FOXP3⁺; Treg) induce immune tolerance and preserve immune homeostasis. Recent in vivo studies suggested that low-dose interleukin-2 (IL-2) can increase Treg cell numbers. Aldesleukin is a human recombinant form of IL-2 that has been used therapeutically in several autoimmune diseases. However, its safety and efficacy is unknown in the setting of coronary artery disease.

Title: Clinical and immunologic effects of intranodal autologous tumor lysate-dendritic cell vaccine with Aldesleukin (Interleukin 2) and IFN-{alpha}2a therapy in metastatic renal cell carcinoma patients.
To evaluate the clinical and immunologic outcomes of DC (dendritic cell) vaccine with interleukin (IL)-2 and IFN-alpha 2a in metastatic renal cell carcinoma patients.

Title: Low-dose interleukin-2 promotes STAT-5 phosphorylation, T_{reg} survival and CTLA-4-dependent function in autoimmune liver diseases.
CD4⁺ CD25^{high} CD127^{low} forkhead box protein 3 (FoxP3⁺) regulatory T cells (T_{reg}) are essential for the maintenance of peripheral tolerance. Impaired T_{reg} function and an imbalance between effector and T_{regs} contribute to the pathogenesis of autoimmune diseases. We reported recently that the hepatic microenvironment is deficient in interleukin (IL)-2, a cytokine essential for T_{reg} survival and function. Consequently, few liver-infiltrating T_{reg} demonstrate signal transducer and activator of transcription-5 (STAT-5) phosphorylation. To establish the potential of IL-2 to enhance T_{reg} therapy, we investigated the effects of very low dose Proleukin (VLDP) on the phosphorylation of STAT-5 and the subsequent... (truncated)

MESH:D002443 - ceftriaxone

Summary:

LLM Prediction Score	0.335 (normalized)
LLM Confidence Score	0.990
Golden Answer (Severity Class)	0.5 (normalized)
Prediction Error	0.165

Retrieved Context:

Title: [Use of ceftriaxone in urinary and respiratory tract infections].
Efficiency of ceftriaxone (Rocephin Hoffman Laroche) was assessed in 16 children aged between 3 and 14 years and in 4 adults aged between 17 and 70 years with severe infections of the urinary and respiratory tracts caused by E. coli. S. pneumoniae, P. aeruginosa, P. mirabilis or enterococci. Pyelonephritis as a sole pathology was diagnosed in 10 patients whereas in further 8 patients it complicated other diseases (nephrotic syndrome, hepatitis, cholangitis, leukemia). Pneumonia complicated nephritis leukemia or lymphoma in 8 children. Peritonitis was diagnosed in 1 adult patient. Ceftriaxone was given in a single daily dose of 50 mg/kg to... (truncated)

Title: A case of ceftriaxone-induced liver injury and literature review.
Liver injury evoked by drugs spans various clinical manifestations ranging from mild biochemical abnormalities to acute liver failure. Ceftriaxone is a third-generation cephalosporin often used in clinical practice for its long half-life, high tissue penetration rate, wide spectrum and good safety profile. Ceftriaxone, as other cephalosporins have little hepatotoxicity; however, few cases of toxic hepatitis induced by this antibiotic have been reported.

Title: Worsening cholestasis and possible cefuroxime-induced liver injury following "successful" therapeutic endoscopic retrograde cholangiopancreatography for a distal common bile duct stone: a case report.
Cefuroxime very rarely causes drug-induced liver injury. We present a case of a patient with paradoxical worsening of jaundice caused by cefuroxime-induced cholestasis following therapeutic endoscopic retrograde cholangiopancreatography for a distal common bile duct stone.

Title: Ceftriaxone-induced toxic hepatitis.
Toxic hepatitis or drug-induced liver injury encompasses a spectrum of clinical disease ranging from mild biochemical abnormalities to acute liver failure. The advantages of a long half-life, wide spectrum, high tissue penetration rate, and a good safety profile, make ceftriaxone, a third-generation cephalosporin, a frequent choice in the treatment of childhood infections. Previous studies have reported a few cases of high aspartate aminotransferase and alanine aminotransferase levels, along with three cases of hepatitis caused by ceftriaxone. Here, we report a case of drug-induced toxic hepatitis in a patient who was treated with ceftriaxone for acute tonsillitis.

Title: Hepatotoxicity of antibiotics.
Several antibiotics can cause severe hepatic injury. It is the purpose of this paper to review the main antibiotics that can cause hepatic injury and discuss the presentation, pattern, and outcome of hepatic injury. In the case of the penicillins, the combination amoxycillin-clavulanate and the penicillinase-resistant penicillins oxacillin, (di-)cloxacillin, and flucloxacillin can cause (mainly cholestatic) hepatitis. Cephalosporins have little hepatotoxicity; ceftriaxone can cause drug-induced gallstones. The potential of erythromycin and several other macrolides to cause (usually cholestatic) hepatitis is well established. Tetracyclines can cause a syndrome mimicking acute fatty liver of pregnancy, but this complication has virtually disappeared. Quinolones seem... (truncated)

MESH:D004961 - estramustine

Summary:

LLM Prediction Score	0.335 (normalized)
LLM Confidence Score	0.960
Golden Answer (Severity Class)	0.5 (normalized)
Prediction Error	0.165

Retrieved Context:

Title: Comparison of estramustine phosphate, methotrexate and cis-platinum in patients with advanced, hormone refractory prostate cancer.
In this clinical trial of men with advanced prostatic cancer no longer responsive to hormone therapy 189 were randomized to receive estramustine phosphate, methotrexate or cis-platinum. Response evaluations were done in 158 cases. Objective response rates (complete, partial or stabilization of disease) were 34 per cent for estramustine phosphate, 36 per cent for cis-platinum and 41 per cent for methotrexate. Subjective parameters indicated a substantial advantage for pain improvement with methotrexate or cis-platinum over estramustine phosphate. Probabilities of continued response indicated some advantage for methotrexate and median response durations at this time were twice as long for methotrexate (32 weeks)... (truncated)

Title: Hemolytic-uremic syndrome during therapy with estramustine phosphate for advanced prostatic cancer.
3 weeks after commencing treatment with estramustine phosphate, typical manifestations of hemolytic-uremic syndrome occurred in a 66-year-old patient with prostate cancer. Urinary tract obstructions were excluded and no renal damage could be identified. An improvement in renal function was achieved by stopping estramustine phosphate and infusing adequate amounts of fluids and electrolytes. Anemia and thrombocytopenia also progressively improved after the discontinuation of chemotherapy. Nausea and vomiting, hepatotoxicity, impotence, reduced libido and hypercalcemia are major side effects of estramustine phosphate, and would be difficult to explain our observations without considering the role played by estramustine phosphate. Our observations suggest that estramustine... (truncated)

Title: Oral estramustine phosphate (NSC-89199) in the treatment of advanced (stage D) carcinoma of the prostate.
Thirty-two patients with stage D carcinoma of the prostate were treated with oral estramustine phosphate at a dose of 15 mg/kg/day from 3 to 15 months. Objective remissions, reduction of greater than 50 percent of measurable lesions such as soft tissue masses, lymph nodes, and prostatic masses, were seen in seven of 32 patients (22 percent response rate). Subjective response, ie, relief of pain, weight gain, sense of well being, snd improved performance status, occurred in all objective responders and in seven other patients with stable disease (15 of 32 patients = 47 percent). No hematologic, hepatic, or renal toxic... (truncated)

Title: Phase I study of paclitaxel and estramustine: preliminary activity in hormone-refractory prostate cancer.
Estramustine phosphate is a unique antimitotic agent that binds to tubulin and microtubule-associated proteins. Preclinically, estramustine combined with other microtubule inhibitors, like vinblastine or paclitaxel (Taxol; Bristol-Myers Squibb Company, Princeton, NJ), produced additive or greater antimitotic and cytotoxic effects. Clinically, the estramustine/vinblastine combination has significant activity in hormone-refractory prostate cancer. We have begun a phase I study of paclitaxel by 96-hour continuous infusion every 3 weeks combined with daily oral estramustine (600 mg/m2). Eighteen patients with refractory solid tumors have received paclitaxel doses ranging from 80 to 140 mg/m2. Grade 3 or 4 granulocytopenia occurred in one of seven and... (truncated)

Title: Estramustine phosphate in secondary hormone-resistant carcinoma of the prostate.
In this retrospective study, 71 patients with secondary hormone-refractory prostatic carcinomas were treated with estramustine phosphate (EMP), at three different dosages (280, 560, 840 mg orally). All patients were completely followed up until cancer-induced death. In 12 cases of further progression polychemotherapy was administered. As this was not a randomized study, an analysis of statistical significance was not performed. The higher dosages of EMP caused an extended progression-free interval accompanied by an equally considerable alleviation of carcinoma-induced pain. The overall survival time was not influenced by subsequent polychemotherapy. An elevation of liver function parameters was observed in 5 patients. One... (truncated)

MESH:D017312 - toremifene

Summary:

LLM Prediction Score	0.540 (normalized)
LLM Confidence Score	0.960
Golden Answer (Severity Class)	0.375 (normalized)
Prediction Error	0.165

Retrieved Context:

Title: [A Case of Liver Failure Induced by Toremifene in a Patient with Metastatic Breast Cancer].
A 67-year-old woman underwent total mastectomy, postoperative radiation therapy, and adjuvant hormonal therapy more than 9 years 4 months previously. There were no symptoms of recurrence for 3.5 years after completing adjuvant hormonal therapy. A hard mass appeared on the front chest wall and was diagnosed as recurrence of breast cancer histopathologically. A computed tomography (CT) scan revealed multiple metastases in the left side of the chest wall, in the left Level II axillary lymph nodes, and in the left lung. The patient was prescribed high-dose tremifene (HD-TOR 120 mg/day). After less than 4 months, she presented with general fatigue... (truncated)

Title: [A real-world study of the effects of endocrine therapy on liver function in breast cancer].
Objective: To compare the effect of different endocrine therapy drugs on liver function in patients with early breast cancer. Methods: A retrospective cohort study was conducted to include 4 318 patients with early breast cancer who received adjuvant endocrine therapy in Department of Breast Surgery, Peking Union Medical College Hospital from January 1, 2013 to December 31, 2021. All the patients were female, aged (51.2±11.3) years (range: 20 to 87 years), including 1 182 patients in the anastrozole group, 592 patients in the letrozole group, 332 patients in the exemestane group, and 2 212 patients in the toremifene group. The... (truncated)

Title: Toremifene-induced fatty liver and NASH in breast cancer patients with breast-conservation treatment.
We have described fatty liver, diagnosed by computed tomography scanning (CT) in more than 30% of patients with breast cancer who received tamoxifen. Therefore, it is urgent to elucidate the frequency and the degree of fatty liver induced by toremifene, an analogue of tamoxifen, which is also used in breast cancer. We enrolled 52 breast cancer patients who were treated with breast-conservation treatment and administered oral toremifene for 3-5 years as adjuvant endocrine therapy. We evaluated the degree of fatty liver by abdominal CT performed annually. CT demonstrated toremifene-induced fatty liver in four (7.7%) of 52 breast cancer patients. Toremifene-induced... (truncated)

Title: Genotoxic potential of tamoxifen and analogues in female Fischer F344/n rats, DBA/2 and C57BL/6 mice and in human MCL-5 cells.
Chronic administration of tamoxifen to female rats causes hepatocellular carcinomas. We have investigated damage to liver DNA caused by the administration of tamoxifen to female Fischer F344/N rats or C57B1/6 or DBA/2 mice using 32P-postlabelling. Following the administration of tamoxifen for 7 days (45 mg/kg/day) and extraction of hepatic DNA, up to 7 radiolabelled adduct spots could be detected after PEI-cellulose chromatography of the 32P-labelled DNA digests. Tamoxifen caused a time-dependent increase in the level of adduct detected up to a value of at least 1 adduct/10(6) nucleotides after 7 days dosing. A dose response relationship was demonstrated over the... (truncated)

Title: Repurposing of FDA-Approved Toremifene to Treat COVID-19 by Blocking the Spike Glycoprotein and NSP14 of SARS-CoV-2.
The global pandemic of Coronavirus Disease 2019 (COVID-19), caused by severe acute respiratory syndrome coronavirus 2 (SARS-CoV-2), has led to the death of more than 675,000 worldwide and over 150,000 in the United States alone. However, there are currently no approved effective pharmacotherapies for COVID-19. Here, we combine homology modeling, molecular docking, molecular dynamics simulation, and binding affinity calculations to determine potential targets for toremifene, a selective estrogen receptor modulator which we have previously identified as a SARS-CoV-2 inhibitor. Our results indicate the possibility of inhibition of the spike glycoprotein by toremifene, responsible for aiding in fusion of the viral... (truncated)

MESH:D017963 - azithromycin

Summary:

LLM Prediction Score	0.710 (normalized)
LLM Confidence Score	0.990
Golden Answer (Severity Class)	0.875 (normalized)
Prediction Error	0.165

Retrieved Context:

Title: Liver transplantation for azithromycin-induced severe liver injury.
Drug-induced liver injury is the most common cause of acute liver failure in Western countries by prescription drugs and herbal medications. Liver injury due to azithromycin has rarely been reported. This is a brief report of a patient administered azithromycin and who developed acute liver failure leading to liver transplantation. We report the case of a 68-year-old woman who developed jaundice 1 week after she started taking a azithromycin. On the 3rd day of hospitalization, her hepatic function rapidly deteriorated and level of consciousness decreased to drowsiness. The model for end-stage liver disease score was confirmed to be 33, and... (truncated)

Title: Azithromycin induced hepatocellular toxicity and hepatic encephalopathy in asymptomatic dilated cardiomyopathy.
Azithromycin is a widely used macrolide derivative and has generally been considered to be a very safe medication. Though gastrointestinal symptoms and reversible hearing loss are common, potentially serious side effects including angioedema and cholestatic jaundice occurred in less than one percent of patients. We report a case of asymptomatic dilated cardiomyopathy with Azithromycin induced severe hepatocellular toxicity and hepatic encephalopathy.

Title: Azithromycin-induced cholestatic hepatitis.
Since its introduction >20 years ago, Azithromycin has been widely used owing to its broad spectrum and good tolerability, especially when used for <7 days. In literature, there are only very few, sporadic reports available of patients developing cholestatic hepatitis following treatment with it. The current case study describes a 69-year old patient, with a medical history that included significant alcohol consumption, who presented with jaundice following a 3-day course of Azithromycin. Following a transjugular liver biopsy, he was managed with a short course of corticosteroids and his liver function gradually improved and finally normalized ~2 months after discontinuation of... (truncated)

Title: The long-term safety of chronic azithromycin use in adult patients with cystic fibrosis, evaluating biomarkers for renal function, hepatic function and electrical properties of the heart.
<i>Background</i>: Azithromycin maintenance therapy is widely used in cystic fibrosis (CF), but little is known about its long-term safety. We investigated whether chronic azithromycin use is safe regarding renal function, hepatic cell toxicity and QTc-interval prolongation.<i>Methods</i>: Adult CF patients (72 patients using azithromycin for a cumulative period of 364.8 years and 19 controls, 108.8 years) from two CF-centers in the Netherlands with azithromycin (non)-use for at least three uninterrupted years were studied retrospectively.<i>Results</i>: There was no difference in mean decline of estimated glomerular filtration rate (eGFR), nor in occurrence of eGFR-events. No drug-induced liver injury could be attributed to azithromycin. Of the... (truncated)

Title: Clinical and histologic features of azithromycin-induced liver injury.
Rare cases of azithromycin-induced hepatotoxicity have been reported, with variable clinical and histologic features. We characterized clinical features and outcomes of azithromycin-induced liver injury.

MESH:C064925 - gadodiamide

Summary:

LLM Prediction Score	0.211 (normalized)
LLM Confidence Score	0.900
Golden Answer (Severity Class)	0.375 (normalized)
Prediction Error	0.164

Retrieved Context:

Title: Cumulative administrations of gadolinium-based contrast agents: risks of accumulation and toxicity of linear vs macrocyclic agents.
Ever since gadolinium was found to deposit in the brain of patients with normal kidney function by Kanda et al. in 2014, several studies have been conducted to evaluate its effect on the patients' health. However, conflicting results were obtained regarding imaging in gadolinium retention. These finding were attributed to the chelating structure of the administered gadolinium-based contrast agent (GBCA): linear agents were found to accumulate in the dentate nucleus (DN) and the globus pallidus (GP) of subjects even after one dose. There are some contradictory results when assessing macrocyclic agents. In the following article, we review the basis of... (truncated)

Title: Nephrogenic systemic fibrosis in liver disease: a systematic review.
Nephrogenic systemic fibrosis (NSF) may develop in patients with liver disease, a fact highlighted by Food and Drug Administration (FDA) announcements cautioning against the use of gadolinium-based contrast agents (GBCAs) in select liver disease patients. The purpose of this systematic literature review is to characterize the risk of NSF in patients with liver disease. All published articles on NSF from September 2000 through August 2008, were identified via PubMed searches and examination of articles' reference lists. Two reviewers independently read each article and identified unique patients with biopsy-proven or suspected NSF. Data on demographics, liver status, renal status, and GBCA... (truncated)

Title: Comparison of hepatocellular carcinoma conspicuity on hepatobiliary phase images with gadoxetate disodium vs. delayed phase images with extracellular cellular contrast agent.
To compare the conspicuity of hepatocellular carcinoma (HCC) on hepatobiliary phase of gadoxetate disodium-enhanced vs. delayed phase of gadodiamide-enhanced MR images, relative to liver function.

Title: Benefits and Detriments of Gadolinium from Medical Advances to Health and Ecological Risks.
Gadolinium (Gd)-containing chelates have been established as diagnostics tools. However, extensive use in magnetic resonance imaging has led to increased Gd levels in industrialized parts of the world, adding to natural occurrence and causing environmental and health concerns. A vast amount of data shows that metal may accumulate in the human body and its deposition has been detected in organs such as brain and liver. Moreover, the disease nephrogenic systemic fibrosis has been linked to increased Gd³⁺ levels. Investigation of Gd³⁺ effects at the cellular and molecular levels mostly revolves around calcium-dependent proteins, since Gd³⁺ competes with calcium due to... (truncated)

Title: Nephrogenic systemic fibrosis risk after liver magnetic resonance imaging with gadoxetate disodium in patients with moderate to severe renal impairment: results of a prospective, open-label, multicenter study.
The objective of this study was to assess the risk of gadoxetate disodium in liver imaging for the development of nephrogenic systemic fibrosis (NSF) in patients with moderate to severe renal impairment.

MESH:C006358 - acetohydroxamic acid

Summary:

LLM Prediction Score	0.211 (normalized)
LLM Confidence Score	0.910
Golden Answer (Severity Class)	0.375 (normalized)
Prediction Error	0.164

Retrieved Context:

Title: Cheminformatics analysis of assertions mined from literature that describe drug-induced liver injury in different species.
Drug-induced liver injury is one of the main causes of drug attrition. The ability to predict the liver effects of drug candidates from their chemical structures is critical to help guide experimental drug discovery projects toward safer medicines. In this study, we have compiled a data set of 951 compounds reported to produce a wide range of effects in the liver in different species, comprising humans, rodents, and nonrodents. The liver effects for this data set were obtained as assertional metadata, generated from MEDLINE abstracts using a unique combination of lexical and linguistic methods and ontological rules. We have analyzed... (truncated)

Title: FADS2-dependent fatty acid desaturation dictates cellular sensitivity to ferroptosis and permissiveness for hepatitis C virus replication.
The metabolic oxidative degradation of cellular lipids severely restricts replication of hepatitis C virus (HCV), a leading cause of chronic liver disease, but little is known about the factors regulating this process in infected cells. Here we show that HCV is restricted by an iron-dependent mechanism resembling the one triggering ferroptosis, an iron-dependent form of non-apoptotic cell death, and mediated by the non-canonical desaturation of oleate to Mead acid and other highly unsaturated fatty acids by fatty acid desaturase 2 (FADS2). Genetic depletion and ectopic expression experiments show FADS2 is a key determinant of cellular sensitivity to ferroptosis. Inhibiting FADS2... (truncated)

Title: Differential Metabolic Pathways and Metabolites in a C57BL/6J Mouse Model of Alcoholic Liver Disease.
BACKGROUND Alcoholic liver disease (ALD), an important cause of acute or chronic liver injury, results from binge drinking or long-term alcohol consumption. To date, there is no well-established mouse model with a comprehensive metabolic profile that mimics ALD in humans. This study aimed to explore the differential metabolic pathways and related differential metabolites in the liver of an ALD mouse model. MATERIAL AND METHODS A C57BL/6J mouse model of ALD was induced by alcohol feeding for 10 days plus binge alcohol feeding. The metabolomic profiles in the liver of the ALD mouse model was detected through ultra-high-pressure liquid chromatography-quadrupole time-of-flight... (truncated)

Title: Nitidine chloride, a benzophenanthridine alkaloid from <i>Zanthoxylum nitidum</i> (Roxb.) DC., exerts multiple beneficial properties, especially in tumors and inflammation-related diseases.
Plant-derived alkaloids are a kind of very important natural organic compounds. Nitidine chloride is one of the main active ingredients in <i>Zanthoxylum nitidum</i> (Roxb.) DC. which is a frequently-used Chinese herbal medicine. <i>Z. nitidum</i> has many kinds of efficacy, such as activating blood circulation and removing stasis, promoting qi circulation and relieving pain, and detoxication and detumescence. In China, <i>Z. nitidum</i> is usually used for the treatment of gastrointestinal diseases, toothache, and traumatic injury. At present, there are numerous studies of nitidine chloride with regard to its pharmacology, pharmacokinetics, toxicology, etc. However, a systematic, cutting-edge review of nitidine-related studies is... (truncated)

Title: Flavonoids and related privileged scaffolds as potential urease inhibitors: a review.
Infections caused by bacteria are a significant issue on a global scale, and imperative action is required to discover novel or improved therapeutic agents. Flavonoids are a class of plant-derived compounds that have a variety of potentially useful bioactivities. These activities include immediate antimicrobial properties, synergistic effect with antimicrobials, ferocious repression of pathogenicity, anti-urease activity <i>etc.</i> This review summarizes current studies concerning anti-urease actions of flavonoids as well as structural-activity correlation investigations of the flavonoid core structure. It is possible that if researchers investigate the many structural changes that may be made in flavonoid rings, they'll be able to build... (truncated)

MESH:D012968 - etidronic acid

Summary:

LLM Prediction Score	0.164 (normalized)
LLM Confidence Score	0.940
Golden Answer (Severity Class)	0.0 (normalized)
Prediction Error	0.164

Retrieved Context:

Title: Primary biliary cirrhosis.
Primary biliary cirrhosis (PBC) is an immune-mediated chronic cholestatic liver disease with a slowly progressive course. Without treatment, most patients eventually develop fibrosis and cirrhosis of the liver and may need liver transplantation in the late stage of disease. PBC primarily affects women (female preponderance 9-10:1) with a prevalence of up to 1 in 1,000 women over 40 years of age. Common symptoms of the disease are fatigue and pruritus, but most patients are asymptomatic at first presentation. The diagnosis is based on sustained elevation of serum markers of cholestasis, i.e., alkaline phosphatase and gamma-glutamyl transferase, and the presence of... (truncated)

Title: First-in-Human Phase I Study of MBC-11, a Novel Bone-Targeted Cytarabine-Etidronate Conjugate in Patients with Cancer-Induced Bone Disease.
Results are consistent with MBC-11 targeting and treating cancer-induced bone lesions by concentrating cytarabine and etidronate at the site of disease.MBC-11 was well tolerated, with an maximum tolerated dose of 5 mg/kg per day and myelosuppression as the principal toxicity.Treatment significantly reduced cancer cell activity in over half of bone lesions detected at baseline.MBC-11 pharmacokinetic and pharmacodynamic parameters are consistent with the novel drug design goals, and encouraging results warrant further clinical development.

Title: The toxicological mechanisms and detoxification of depleted uranium exposure.
Depleted uranium (DU) has been widely applied in industrial and military activities, and is often obtained from producing fuel for nuclear reactors. DU may be released into the environment, polluting air, soil, and water, and is considered to exert both radiological and chemical toxicity. In humans and animals, DU can induce multiple health effects, such as renal tubular necrosis and bone malignancies. This review summarizes the known information on DU's routes of entry, mechanisms of toxicity, and health effects. In addition, we survey the chelating agents used in ameliorating DU toxicity.

Title: Comprehensive cytotoxicity studies of superparamagnetic iron oxide nanoparticles.
Recently lots of efforts have been taken to develop superparamagnetic iron oxide nanoparticles (SPIONs) for biomedical applications. So it is utmost necessary to have in depth knowledge of the toxicity occurred by this material. This article is designed in such way that it covers all the associated toxicity issues of SPIONs. It mainly emphasis on toxicity occurred at different levels including cellular alterations in the form of damage to nucleic acids due to oxidative stress and altered cellular response. In addition focus is been devoted for in vitro and in vivo toxicity of SPIONs, so that a better therapeutics can... (truncated)

Title: Characterization of the small molecule ARC39, a direct and specific inhibitor of acid sphingomyelinase in vitro.
Inhibition of acid sphingomyelinase (ASM), a lysosomal enzyme that catalyzes the hydrolysis of sphingomyelin into ceramide and phosphorylcholine, may serve as an investigational tool or a therapeutic intervention to control many diseases. Specific ASM inhibitors are currently not sufficiently characterized. Here, we found that 1-aminodecylidene bis-phosphonic acid (ARC39) specifically and efficiently (>90%) inhibits both lysosomal and secretory ASM in vitro. Results from investigating sphingomyelin phosphodiesterase 1 (<i>SMPD1/Smpd1</i>) mRNA and ASM protein levels suggested that ARC39 directly inhibits ASM's catalytic activity in cultured cells, a mechanism that differs from that of functional inhibitors of ASM. We further provide evidence that ARC39... (truncated)

MESH:D002740 - chlorothiazide

Summary:

LLM Prediction Score	0.413 (normalized)
LLM Confidence Score	0.970
Golden Answer (Severity Class)	0.25 (normalized)
Prediction Error	0.163

Retrieved Context:

Title: Drug induced interstitial nephritis, hepatitis and exfoliative dermatitis.
Acute interstitial nephritis associated with hepatitis, exfoliative dermatitis, fever and eosinophilia is uncommon. The syndrome has been described previously in association with phenindione administration, leptospirosis and heavy metal poisoning. Four cases are described, two of which were due to phenindione sensitivity. The other two patients had been exposed to a number of toxins including allopurinol, frusemide, chlorothiazide and methyldopa so that the exact aetiological agent is unclear. Interstitial nephritis should be considered as a cause of acute renal failure in patients with other features of drug hypersensitivity.

Title: An updated review on drug-induced cholestasis: mechanisms and investigation of physicochemical properties and pharmacokinetic parameters.
Drug-induced cholestasis is an important form of acquired liver disease and is associated with significant morbidity and mortality. Bile acids are key signaling molecules, but they can exert toxic responses when they accumulate in hepatocytes. This review focuses on the physiological mechanisms of drug-induced cholestasis associated with altered bile acid homeostasis due to direct (e.g., bile acid transporter inhibition) or indirect (e.g., activation of nuclear receptors, altered function/expression of bile acid transporters) processes. Mechanistic information about the effects of a drug on bile acid homeostasis is important when evaluating the cholestatic potential of a compound, but experimental data often are... (truncated)

Title: The comparative effect of chlorothiazide and meralluride on the development of hepatic encephalopathy in cirrhotic patients.
No abstract available.

Title: Data-driven identification of structural alerts for mitigating the risk of drug-induced human liver injuries.
The use of structural alerts to de-prioritize compounds with undesirable features as drug candidates has been gaining in popularity. Hundreds of molecular structural moieties have been proposed as structural alerts. An emerging issue is that strict application of these alerts will result in a significant reduction of the chemistry space for new drug discovery, as more than half of the oral drugs on the market match at least one of the alerts. To mitigate this issue, we propose to apply a rigorous statistical analysis to derive/validate structural alerts before use.

Title: Management of portal hypertension and ascites in polycystic liver disease.
Patients suffering from polycystic liver disease may develop Hepatic Venous Outflow Obstruction, Portal Vein Obstruction and/or Inferior Caval Vein Syndrome because of cystic mass effect. This can cause portal hypertension, leading to ascites, variceal haemorrhage or splenomegaly. For this review, we evaluate the evidence to provide clinical guidance for physicians faced with this complication. Diagnosis is made with imaging such as ultrasound, computed tomography or magnetic resonance imaging. Therapy includes conventional therapy with diuretics and paracentesis, and medical therapy using somatostatin analogues. Based on disease phenotype various (non-)surgical liver-volume reducing therapies, hepatic or portal venous stenting, transjugular intrahepatic portosystemic shunts... (truncated)

MESH:D017255 - acitretin

Summary:

LLM Prediction Score	0.463 (normalized)
LLM Confidence Score	0.980
Golden Answer (Severity Class)	0.625 (normalized)
Prediction Error	0.162

Retrieved Context:

Title: Psoriasis and Nonalcoholic Fatty Liver Disease.
Nonalcoholic fatty liver disease (NAFLD) is the most prevalent liver condition in the West. The prevalence and severity of NAFLD is higher and the prognosis worse in patients with psoriasis. The pathogenic link between psoriasis and NAFLD is chronic inflammation and peripheral insulin resistance, a common finding in diseases associated with psoriasis. NAFLD should therefore be ruled out during the initial evaluation of patients with psoriasis, in particular if they show signs of metabolic syndrome and require systemic treatment. Concomitant psoriasis and NAFLD and the likelihood of synergy between them place limitations on general recommendations and treatment for these patients... (truncated)

Title: Acitretin : A Review of its Pharmacology and Therapeutic Use.
Acitretin (etretin), a second generation monoaromatic retinoid for use in the treatment of severe psoriasis and other dermatoses, is the major active metabolite of etretinate and possesses a similar therapeutic index; i.e. a similar ratio of clinical efficacy to adverse effects. When used alone at a maintenance dosage of 30 to 50mg daily, acitretin is effective in the treatment of psoriasis, causing a reduction in the severity of scaling, erythema and induration. Efficacy appears to be further enhanced by combination with psoralen-ultraviolet A photochemotherapy (PUVA) or ultraviolet B irradiation (UVB). These combinations reduce the time to lesion clearance and reduce... (truncated)

Title: Effects of acitretin on the liver.
Therapy with aromatic retinoids for psoriasis is associated with abnormal liver function test findings and toxic hepatitis (in 1.5% of patients).

Title: Long-term safety of retinoid therapy.
The concern about long-term toxicity of oral synthetic retinoids has developed because many patients, especially those with genodermatoses, require lifelong therapy. Several organ systems are at risk, especially the hepatic, skeletal, and cardiovascular systems. Although acute hepatotoxicity is a rare side effect of etretinate and acitretin therapy, prospective studies have not demonstrated chronic liver toxicity. The frequency of bone changes induced by retinoids is difficult to estimate, because this adverse effect is usually asymptomatic and requires x-ray or scintigraphic examination for detection. Atherosclerosis develops in many patients who receive long-term retinoid therapy, but the extent to which the process is... (truncated)

Title: Retinoids, methotrexate and cyclosporine.
Acitretin alone is efficient (PASI 90: 40%). In responders, it is the best long-term maintenance treatment (up to 29 years of continuous treatment). The main side effect is its teratogenicity in females. It is necessary to begin retinoid treatment at low doses (10 mg/day), increasing the dose step by step, looking for the maximum well-tolerated dose (usually defined as a mild cheilitis). Doses higher than the highest well-tolerated dose are frequently responsible for the Kobner phenomenon. In children, retinoids are very efficient and nearly always well tolerated, but it seems important to never give more than 0.5 mg/kg/day. Methotrexate is... (truncated)

MESH:D005996 - nitroglycerin

Summary:

LLM Prediction Score	0.161 (normalized)
LLM Confidence Score	0.990
Golden Answer (Severity Class)	0.0 (normalized)
Prediction Error	0.161

Retrieved Context:

Title: [Ischemic hepatitis].
The patients with chronic congestive heart failure and acute deterioration of heart failure (pulmonary oedema, significant reduction of blood pressure) have decrease liver's perfusion with signs of acute damage of liver's cells--ischemic hepatitis. Aspat, AIAT and LDH in blood rich very high level. The level of bilirubin, alkaline phosphatase and glucose increase slightly. Hepatotoxic viruses are never observed. The authors described a case of 34 years old man, who two years earlier had large myocardial infarction with aneurysm of heart and congestive heart failure. He was admitted to hospital in shock. The shock was caused probably by overdose of nitroglycerin.... (truncated)

Title: The Protective Effect of Nitroglycerin, N-Acetyl Cysteine and Metoprolol in CCL4 Induced Animal Model of Acute Liver Injury.
The current study was designed to determine the hepatoprotective effect of well-known drugs. Nitroglycerin, N-acetyl cysteine and Metoprolol in acute liver injury induced by CCL4. The antioxidant effects of b-blockers, especially carvedilol, have been described by several investigators. However, for metoprolol, the effect is a bit query as there is only one in-vitro study showing a little hepatoprotective effect. Thus, it is worthy to re-study the hepatoprotective effect of metoprolol.

Title: Is Glyceryl Trinitrate, a Nitric Oxide Donor Responsible for Ameliorating the Chemical-Induced Tissue Injury In Vivo?
Oxidative stress induced by well-known toxins including ferric nitrilotriacetate (Fe-NTA), carbon tetrachloride (CCl₄) and thioacetamide (TAA) has been attributed to causing tissue injury in the liver and kidney. In this study, the effect of glyceryl trinitrate (GTN), a donor of nitric oxide and NG-nitroarginine methyl ester (l-NAME), a nitric oxide inhibitor on TAA-induced hepatic oxidative stress, GSH and GSH-dependent enzymes, serum transaminases and tumor promotion markers such as ornithine decarboxylase (ODC) activity and [³H]-thymidine incorporation in rats were examined. The animals were divided into seven groups consisting of six healthy rats per group. The six rats were injected intraperitoneally with... (truncated)

Title: Nitrovasodilators inhibit platelet-derived growth factor-induced proliferation and migration of activated human hepatic stellate cells.
Nitrovasodilators have been proposed for the treatment of portal hypertension alone or in combination with beta-blockers. In addition to their vasodilatory properties, nitric oxide (NO) donors may exert direct antifibrogenic properties. We evaluated the effect of nitroglycerin (NTG) and S-nitroso-N-acetyl penicillamine (SNAP) on the mitogenic and chemotactic properties of platelet-derived growth factor (PDGF)-BB and the modulation of the relative intracellular signaling pathways in fully activated human hepatic stellate cells (HSCs), a cell type that plays an active role in liver fibrogenesis and portal hypertension.

Title: Liver function monitoring: a prospective nested case-control study of Salvia miltiorrhiza polyphenol injection.
Instructions for Salvia miltiorrhiza polyphenol injections indicate abnormal liver function as an occasional adverse reaction, but the incidence of this adverse drug reaction (ADR) has increased in recent years. We assessed S. miltiorrhiza polyphenol ADRs by performing a nested case-control study(NCCS) and meta-analysis. In the NCCS, 2633 patients receiving this treatment in the First Affiliated Hospital of Bengbu Medical College were enrolled. Logistic regression models found that in 58 (2.2%) patients experiencing abnormal liver function, the risk for liver dysfunction was associated with sulfa drug allergy (OR = 7.874, 95%CI (1.280, 48.447), P = 0.026), payment methods (OR = 0.106,... (truncated)

MESH:D000068338 - everolimus

Summary:

LLM Prediction Score	0.659 (normalized)
LLM Confidence Score	0.990
Golden Answer (Severity Class)	0.5 (normalized)
Prediction Error	0.159

Retrieved Context:

Title: Progressive liver failure induced by everolimus for renal cell carcinoma in a 58-year-old male hepatitis B virus carrier.
A 58-year-old man was diagnosed as a hepatitis B virus (HBV) carrier approximately 30 years ago. He was diagnosed with renal cell carcinoma when he was 57 years old. Radical nephrectomy was performed, and everolimus was administered to treat his lung metastasis. After beginning the everolimus, intermittent fever, general fatigue, and jaundice developed. He was admitted under a diagnosis of flare (acute exacerbation) of chronic B hepatitis due to HBV reactivation. Despite intensive care, he died of hepatic failure and fungus infection. The autopsy findings were compatible with hepatic failure due to HBV reactivation by everolimus. Antiviral prophylaxis must be... (truncated)

Title: Risk of fatigue and hepatic and metabolic toxicities in patients with solid tumors treated with everolimus: a meta-analysis.
We performed a systematic review and meta-analysis of fatigue, hepatic and metabolic toxicities associated with everolimus intake in patients with solid tumors.

Title: [Liver damage caused by atorvastatin and cyclosporine in patients with renal transplant].
Kidney transplantation is the preferred method of treatment of end-stage renal disease, which significantly improves the quality of life, but also increases survival when compared to dialysis. Prevention of acute or chronic rejection demands the use of immunosuppression. However, nephrotoxicity, hepatotoxicity, cardiovascular disease, post-transplantation diabetes mellitus, chronic graft dysfunction and dyslipidemia may all occur as complications of immunosuppressive therapy. Dyslipidemia is a significant problem in renal transplant recipients due to the fact that it increases the risk of cardiovascular mortality in patients in whom the risk is already higher than in the general population. Very often, there is an interaction... (truncated)

Title: Everolimus is a potent inhibitor of activated hepatic stellate cell functions in vitro and in vivo, while demonstrating anti-angiogenic activities.
Progression of liver fibrosis to HCC (hepatocellular carcinoma) is a very complex process which involves several pathological phenomena, including hepatic stellate cell activation, inflammation, fibrosis and angiogenesis. Therefore inhibiting multiple pathological processes using a single drug can be an effective choice to curb the progression of HCC. In the present study, we used the mTOR inhibitor everolimus to observe its effect on the in vitro activation of hepatic stellate cells and angiogenesis. The results of the present study demonstrated that everolimus treatment blocked the functions of the immortalized human activated hepatic stellate cell line LX-2 without affecting the viability and... (truncated)

Title: Epithelial to mesenchymal transition in the liver field: the double face of Everolimus in vitro.
Everolimus (EVE), a mammalian target of rapamycin inhibitor, has been proposed as liver transplant immunosuppressive drug, gaining wide interest also for the treatment of cancer. Although an appropriate tolerance, it may induce several adverse effects, such as fibro-interstitial pneumonitis due to the acquisition of activated myofibroblasts. The exact molecular mechanism associated with epithelial to mesenchymal transition (EMT) may be crucial also in the liver context. This work examines the role and the molecular mediators of EMT in hepatic stellate cell (HSC) and human liver cancer cells (HepG2) and the potential role of EVE to maintain the epithelial phenotype rather than... (truncated)

MESH:D000077157 - sorafenib

Summary:

LLM Prediction Score	0.841 (normalized)
LLM Confidence Score	0.990
Golden Answer (Severity Class)	1.0 (normalized)
Prediction Error	0.159

Retrieved Context:

Title: Fulminant hepatitis in a patient with hepatocellular carcinoma related to nonalcoholic steatohepatitis treated with sorafenib.
We describe a case of acute liver failure in a patient with advanced hepatocellular carcinoma related to nonalcoholic steatohepatitis during sorafenib treatment. A 74-year-old man with diabetes mellitus and hypertension was diagnosed with hepatocellular carcinoma associated with fatty liver. Three weeks after sorafenib therapy, at Eastern Cooperative Oncology Group performance status 3, he developed jaundice, general weakness, flapping tremor, nausea, and anorexia. Sorafenib was stopped: laboratory tests showed a relevant elevation of transaminases suggesting diagnosis of acute hepatitis. During hospital admission, the patient died of liver failure. Sorafenib is the first successful target therapy effective for advanced hepatocellular carcinoma. The... (truncated)

Title: A rare case of sorafenib-induced severe hyponatremia.
Sorafenib is an anti-angiogenic tyrosine kinase inhibitor used to treat patients with renal cell cancer and advanced hepatocellular cancer. Common adverse effects of sorafenib are rash, diarrhea, nausea, and abnormal liver function test and hand-foot syndrome.

Title: Elucidation of the Molecular Mechanisms Underlying Sorafenib-Induced Hepatotoxicity.
Sorafenib is a small, orally-active multikinase inhibitor that is most frequently used for the management of renal cell carcinoma, hepatocellular carcinoma, and radioactive iodine-resistant thyroid carcinoma. However, recent reports have associated sorafenib with hepatotoxicity that can limit its clinical application, although the mechanism of hepatotoxicity is still to be elucidated. Thus, our study was designed to explore the molecular mechanisms underlying sorafenib-induced hepatotoxicity in an <i>in vivo</i> model. Twenty male adult Wistar rats were randomly placed into two groups; the first group received an oral dose of normal saline (vehicle), and the second received sorafenib (30 mg/kg) once daily for twenty-one... (truncated)

Title: Safety and efficacy of sorafenib in the treatment of hepatocellular carcinoma.
Hepatocellular carcinoma (HCC) is frequently diagnosed in the setting of chronic liver disease and cirrhosis. The median survival after diagnosis is dismal. The treatment options that may offer cure are either resection or liver transplantation. Unfortunately most patients are not eligible for either treatment modality at diagnosis because of advanced stage and underlying liver dysfunction. Until recently, there was no effective systemic therapy for patients with advanced HCC. Sorafenib, an oral multikinase inhibitor of the vascular endothelial growth factor receptor, the platelet-derived growth factor receptor and Raf, has shown antitumor activity in patients with advanced HCC in phase III trials.... (truncated)

Title: Sorafenib-Induced Grade Four Hepatotoxicity in a Patient with Recurrent Gastrointestinal Stromal Tumor (GIST): A Case Report and Review of Literature.
Gastrointestinal stromal tumor is a rare mesenchymal tumor. Sorafenib is an effective medication in these tumors based on two phase II clinical trials and a retrospective analysis. We report a rare case of a 57-year-old male with acute hepatotoxicity from sorafenib. He was treated conservatively with IV fluids and prednisolone. Liver function tests improved over 2 months. We conclude that sorafenib could cause life-threatening hepatotoxicity and patients taking sorafenib need to be closely monitored.

MESH:D009661 - nortriptyline

Summary:

LLM Prediction Score	0.842 (normalized)
LLM Confidence Score	0.980
Golden Answer (Severity Class)	1.0 (normalized)
Prediction Error	0.158

Retrieved Context:

Title: Nortriptyline-induced fulminant hepatic failure.
We describe, to our knowledge, the first reported case of nortriptyline-induced fulminant hepatic failure. This tricyclic antidepressant drug was taken by a postmenopausal woman for 64 days before her presentation. The absence of fever, rash, or marked eosinophilia, the predominant zone 3 necrosis with bridging, and the latent period favor a metabolic idiosyncratic reaction. The fatal outcome underscores the importance of recognizing the association and discontinuing the offending agent.

Title: Trazodone-induced hepatotoxicity: a case report with comments on drug-induced hepatotoxicity.
Trazodone (Desyrel) is a second-generation, nontricyclic antidepressant that has been in use in North America since the early 1980s. It has the advantage of being more sedating and having less anticholinergic side effects than other secondary amines in the piperazine class, namely, desipramine and nortriptyline. Five previous cases of trazodone hepatotoxicity have been reported in the literature, one describing chronic damage and the others, more acute cellular and cholestatic injury. We describe a case of acute reversible liver injury with the use of trazodone. This case is unique in that injury occurred after protracted (18 months) drug use and while... (truncated)

Title: Nortriptyline-induced hepatic failure.
We report a case of hepatic injury after treatment with nortriptyline in a therapeutic dose. There were symptoms of hepatitis and increased prothrombin time, serum alanine aminotransferase and alkaline phosphatases. The patient recovered after discontinuation of the drug.

Title: The Importance of Patient-Specific Factors for Hepatic Drug Response and Toxicity.
Responses to drugs and pharmacological treatments differ considerably between individuals. Importantly, only 50%-75% of patients have been shown to react adequately to pharmacological interventions, whereas the others experience either a lack of efficacy or suffer from adverse events. The liver is of central importance in the metabolism of most drugs. Because of this exposed status, hepatotoxicity is amongst the most common adverse drug reactions and hepatic liabilities are the most prevalent reason for the termination of development programs of novel drug candidates. In recent years, more and more factors were unveiled that shape hepatic drug responses and thus underlie the... (truncated)

Title: Drug-Induced Liver Injury during Antidepressant Treatment: Results of AMSP, a Drug Surveillance Program.
Drug-induced liver injury is a common cause of liver damage and the most frequent reason for withdrawal of a drug in the United States. The symptoms of drug-induced liver damage are extremely diverse, with some patients remaining asymptomatic.

MESH:D000077146 - irinotecan

Summary:

LLM Prediction Score	0.532 (normalized)
LLM Confidence Score	0.990
Golden Answer (Severity Class)	0.375 (normalized)
Prediction Error	0.157

Retrieved Context:

Title: Phase I study of cisplatin, irinotecan, and epirubicin administered every 3 weeks in patients with advanced solid tumours.
This phase I study was conducted to determine the recommended phase II doses, safety profile, and antitumour activity of a combination regimen of cisplatin, irinotecan, and epirubicin administered every 3 weeks in patients with advanced solid tumours. Cisplatin and epirubicin were given at fixed doses of 50 and 60 mg m(-2), respectively. The irinotecan dose was escalated at 10 mg m(-2) increments from a starting dose level of 70 mg m(-2). Epirubicin, irinotecan, and their metabolites were measured with HPLC methods. In all, 35 patients received 141 courses of treatment. Irinotecan dose was escalated in seven cohorts up to 130... (truncated)

Title: Reversible grade 4 hyperbilirubinemia in a patient with UGT1A1 7/7 genotype treated with irinotecan and cetuximab.
Irinotecan-induced gastrointestinal toxicities are common and typically present in the form of diarrhea or nausea and vomiting. However, severe hyperbilirubinemia (grade 3/4) has not been previously reported in association with this chemotherapeutic agent. We report a case of prolonged grade 4 hyperbilirubinemia after a single dose of irinotecan at 125 mg/m(2). This severe toxicity was attributed to a UGT1A1 7/7 genotype and resolved to grade 2 after 8 weeks of supportive care. This case outlines the possibility of severe hepatic toxicity with moderate doses of irinotecan in patients with a UGT1A1 7/7 genotype. Despite the severity and prolonged duration of... (truncated)

Title: Drug-Drug Interactions and Disease Status Are Associated With Irinotecan-Induced Hepatotoxicity: A Cross-Sectional Study in Shanghai.
Irinotecan-induced hepatotoxicity can cause severe clinical complications in patients; however, the underlying mechanism and factors affecting hepatotoxicity have rarely been investigated. In this cross-sectional study, we screened all clinical, demographic, medication, and genetic variables among 126 patients receiving irinotecan and explored potential associations with the incidence and time to onset of irinotecan-induced hepatotoxicity. Approximately 38.9% of the patients suffered from hepatotoxicity after irinotecan administration. The presence of cardiovascular diseases increases the incidence of hepatotoxicity ≈2.9-fold and doubles the hazard of time to hepatotoxicity. Patients with liver metastasis had a >4-fold higher risk of hepatotoxicity and a 3.5-fold increased hazard of... (truncated)

Title: Irinotecan and Δ^8-Tetrahydrocannabinol Interactions in Rat Liver: A Preliminary Evaluation Using Biochemical and Genotoxicity Markers.
There is growing interest regarding the use of herbal preparations based on <i>Cannabis sativa</i> for medicinal purposes, despite the poorly understood interactions of their main constituent Δ⁸-tetrahydrocannabinol (THC) with conventional drugs, especially cytostatics. The objective of this pilot study was to prove whether the concomitant intake of THC impaired liver function in male Wistar rats treated with the anticancer drug irinotecan (IRI), and evaluate the toxic effects associated with this exposure. IRI was administered once intraperitoneally (at 100 mg/kg of the body weight (b.w.)), while THC was administered per os repeatedly for 1, 3, and 7 days (at 7 mg/kg... (truncated)

Title: Risk factors for irinotecan-induced liver injury: a retrospective multicentre cross-sectional study in China.
The hepatotoxicity of irinotecan has been widely implicated in the treatment of multiple solid tumours. However, there are few studies on the influencing factors of irinotecan-induced hepatotoxicity. Herein, we investigated the risk factors for irinotecan-induced liver injury among 421 patients receiving irinotecan-based regimens (IBRs).

MESH:D006585 - altretamine

Summary:

LLM Prediction Score	0.093 (normalized)
LLM Confidence Score	0.860
Golden Answer (Severity Class)	0.25 (normalized)
Prediction Error	0.157

Retrieved Context:

Title: Pentamethylmelamine (PMM): Phase I clinical and pharmacokinetic studies.
PMM is a water-soluble alternative to HMM. PMM has been administered as an intravenous infusion to 17 patients in a Phase I clinical trial. The dose-limiting toxicities were nausea and vomiting which were observed in all patients at 500 mg m-2 and above. The dose was not escalated above 1300 mg m-2 where nausea and vomiting were severe, prolonged (greater than 24 h) and poorly controlled by anti-emetics. Haematological, hepatic and renal toxicities were not observed. Neurological toxicity was not observed at low doses (less than 500 mg/m2) but could not be determined at higher doses due to intensive anti-emetic... (truncated)

Title: Pharmacology of pentamethylmelamine in humans.
A rapid, specific high-pressure liquid chromatographic assay was used to study the pharmacology of pentamethylmelamine in 21 patients (28 infusions) receiving 80 to 1500 mg/sq m. In patients with normal liver function, pentamethylmelamine was rapidly cleared from the plasma with a terminal half-life of 2.2 hr. Abnormal liver function tended to correlate with increased half-life and reduced total clearance. In addition, increased neurological toxicity was associated with hepatic abnormalities. The N2,N2,N4,N6-tetramethylmelamine, N2,N4,N6-trimethylmelamine, dimethylmelamine, and monomethylmelamine metabolites were detected in plasma. The terminal plasma half-lives of these metabolites increased with decreasing number of methyl group. With liver dysfunction, the plasma clearance... (truncated)

Title: Morin encapsulated chitosan nanoparticles (MCNPs) ameliorate arsenic induced liver damage through improvement of the antioxidant system and prevention of apoptosis and inflammation in mice.
Chronic exposure to arsenic over a period of time induces toxicity, primarily in the liver but gradually in all systems of the body. Morin hydrate (MH; 2',3,4',5,7-pentahydroxyflavone), a potent flavonoid abundantly present in plants of the Moraceae family, is thought to be a major bioactive compound that may be used to prevent a wide range of disease pathologies including hepatotoxicity. Therapeutic applications of morin (MOR) are however seriously constrained because of its insolubility, poor bioavailability, high metabolism and rapid elimination from the human body. Nanoformulation of MOR is a possible solution to these problems. In the present study we investigated... (truncated)

Title: A human liver microphysiology platform for investigating physiology, drug safety, and disease models.
This paper describes the development and characterization of a microphysiology platform for drug safety and efficacy in liver models of disease that includes a human, 3D, microfluidic, four-cell, sequentially layered, self-assembly liver model (SQL-SAL); fluorescent protein biosensors for mechanistic readouts; as well as a microphysiology system database (MPS-Db) to manage, analyze, and model data. The goal of our approach is to create the simplest design in terms of cells, matrix materials, and microfluidic device parameters that will support a physiologically relevant liver model that is robust and reproducible for at least 28 days for stand-alone liver studies and microfluidic integration... (truncated)

Title: Generation of hepatic spheroids using human hepatocyte-derived liver progenitor-like cells for hepatotoxicity screening.
Rationale: The idiosyncratic drug-induced liver injury (iDILI) is a major cause of acute liver injury and a key challenge in late-stage drug development. Individual heterogeneity is considered to be an essential factor of iDILI. However, few <i>in vitro</i> model can predict heterogeneity in iDILI. We have previously shown that mouse and human hepatocytes can be converted to expandable liver progenitor-like cells <i>in vitro</i> (HepLPCs). However, the limited proliferation potential of human HepLPCs confines its industrial application. Here, we reported the generation of a novel hepatocyte model not only to provide unlimited cell sources for human hepatocytes but also to establish... (truncated)

MESH:D000077735 - gemifloxacin

Summary:

LLM Prediction Score	0.219 (normalized)
LLM Confidence Score	0.940
Golden Answer (Severity Class)	0.375 (normalized)
Prediction Error	0.156

Retrieved Context:

Title: The safety profile of the fluoroquinolones.
Premarketing trials showed the fluoroquinolone agents to have a favorable side-effect profile, with treatment-related adverse events comprising gastrointestinal, central nervous system, and dermatologic effects that were generally mild and reversible on cessation of treatment. However, postmarketing surveillance studies have identified severe adverse events, including severe anaphylaxis, QTc-interval prolongation, and potential cardiotoxicity, associated with 3 quinolone agents that either resulted in the removal of the agent from the market (temafloxacin and grepafloxacin) or significantly restricted its use due to substantial mortality and morbidity associated with liver toxicity (trovafloxacin). To date, there have been no such significant adverse events associated with the... (truncated)

Title: Hepatic safety of antibiotics used in primary care.
Antibiotics used by general practitioners frequently appear in adverse-event reports of drug-induced hepatotoxicity. Most cases are idiosyncratic (the adverse reaction cannot be predicted from the drug's pharmacological profile or from pre-clinical toxicology tests) and occur via an immunological reaction or in response to the presence of hepatotoxic metabolites. With the exception of trovafloxacin and telithromycin (now severely restricted), hepatotoxicity crude incidence remains globally low but variable. Thus, amoxicillin/clavulanate and co-trimoxazole, as well as flucloxacillin, cause hepatotoxic reactions at rates that make them visible in general practice (cases are often isolated, may have a delayed onset, sometimes appear only after cessation... (truncated)

Title: Moxifloxacin safety: an analysis of 14 years of clinical data.
Moxifloxacin, a fluoroquinolone antibiotic, is used for the treatment of respiratory tract, pelvic inflammatory disease, skin, and intra-abdominal infections. Its safety profile is considered favorable in most reviews but has been challenged with respect to rare but potentially fatal toxicities (e.g. hepatic, cardiac, or skin reactions).

Title: Guide to selection of fluoroquinolones in patients with lower respiratory tract infections.
Newer fluoroquinolones such as levofloxacin, moxifloxacin, gatifloxacin and gemifloxacin have several attributes that make them excellent choices for the therapy of lower respiratory tract infections. In particular, they have excellent intrinsic activity against Streptococcus pneumoniae, Haemophilus influenzae, Moraxella catarrhalis and the atypical respiratory pathogens. Fluoroquinolones may be used as monotherapy to treat high-risk patients with acute exacerbation of chronic bronchitis, and for patients with community-acquired pneumonia requiring hospitalisation, but not admission to intensive care. Overall, the newer fluoroquinolones often achieve clinical cure rates in > or =90% of these patients. However, rates may be lower in hospital-acquired pneumonia, and this... (truncated)

Title: Efficacy and safety of gemifloxacin in the treatment of community-acquired pneumonia: a randomized, double-blind comparison with trovafloxacin.
This multicentre, randomized, double blind, parallel group study compared the efficacy and safety of gemifloxacin (320 mg once daily) with trovafloxacin (200 mg once daily) in 571 patients with community-acquired pneumonia (CAP). Although treatment was given routinely for 7 days it could be extended to 14 days; two-thirds of patients were treated for 7 days. High clinical success rates were noted at follow-up in the per-protocol population in both the gemifloxacin group (95.8%) and the trovafloxacin group (93.6%), non-inferiority with 95% CI. In the intent-to-treat population, the clinical success rate at follow-up was significantly superior for gemifloxacin (87.6%) compared with... (truncated)

MESH:C108128 - frovatriptan

Summary:

LLM Prediction Score	0.155 (normalized)
LLM Confidence Score	0.930
Golden Answer (Severity Class)	0.0 (normalized)
Prediction Error	0.155

Retrieved Context:

Title: Managing migraine by patient profile: role of frovatriptan.
For the last quarter of a century, triptans have been available for acute treatment of migraine but with little guidance on which of the different triptan products to use for which patient or which attack of migraine. In this article, we propose a structured approach to analysis of individual migraine attacks and patient characteristics as a means of defining and optimizing acute intervention. Assessment of patient and attack profiles includes the "5-Ps": pattern, phenotype, patient, pharmacology, and precipitants. Attending to these five components of information can assist in developing an individualized behavioral, pharmacological, and nonpharmacological comprehensive treatment plan for most... (truncated)

Title: Efficacy of frovatriptan as compared to other triptans in migraine with aura.
The treatment of migraine attacks with aura by triptans is difficult since triptans most probably are not efficacious when taken during the aura phase. Moreover, there are insufficient data from randomised studies whether triptans are efficacious in migraine attacks with aura when taken during the headache phase. In this metaanalysis, we aimed to compare the efficacy of frovatriptan versus rizatriptan, zolmitriptan, and almotriptan.

Title: Pharmacogenetics in Primary Headache Disorders.
Primary headache disorders, such as migraine, tension-type headache (TTH), and cluster headache, belong to the most common neurological disorders affecting a high percentage of people worldwide. Headache induces a high burden for the affected individuals on the personal level, with a strong impact on life quality, daily life management, and causes immense costs for the healthcare systems. Although a relatively broad spectrum of different pharmacological classes for the treatment of headache disorders are available, treatment effectiveness is often limited by high variances in therapy responses. Genetic variants can influence the individual treatment success by influencing pharmacokinetics or pharmacodynamics of the... (truncated)

Title: Prediction of adverse drug reactions based on knowledge graph embedding.
Adverse drug reactions (ADRs) are an important concern in the medication process and can pose a substantial economic burden for patients and hospitals. Because of the limitations of clinical trials, it is difficult to identify all possible ADRs of a drug before it is marketed. We developed a new model based on data mining technology to predict potential ADRs based on available drug data.

Title: Comparison of New Pharmacologic Agents With Triptans for Treatment of Migraine: A Systematic Review and Meta-analysis.
New therapeutic classes of migraine-specific treatment have been developed, including 5-hydroxytryptamine1F receptor agonists (lasmiditan) and calcitonin gene-related peptide antagonists (rimegepant and ubrogepant).

MESH:D016559 - tacrolimus

Summary:

LLM Prediction Score	0.471 (normalized)
LLM Confidence Score	0.990
Golden Answer (Severity Class)	0.625 (normalized)
Prediction Error	0.154

Retrieved Context:

Title: Tacrolimus therapy causes hepatotoxicity in patients with a history of liver disease.
Tacrolimus is known to have little hepatotoxicity. Nevertheless, a few case studies have shown liver toxicities of tacrolimus, particularly in patients on multiple medications. This study is a retrospective data analysis on the potential of tacrolimus hepatotoxicity.

Title: Hepatotoxicity caused by both tacrolimus and cyclosporine after living donor liver transplantation.
We present a case report of a posttransplant patient who had hepatotoxicity due to both tacrolimus and cyclosporine and cholestatic jaundice due to tacrolimus. The patient did not show sustained improvement in enzyme and bilirubin abnormalities after an initial change from tacrolimus to cyclosporine or with a change back to tacrolimus, but he ultimately showed improvement when the blood concentration of tacrolimus was lowered. A 56-year-old man with subacute fulminant hepatitis induced by acarbose was admitted to our hospital for living donor liver transplantation. The liver graft consisted of the left lobe from his ABO-identical son. The early posttransplant course... (truncated)

Title: Protective effect of silymarin on tacrolimus-induced kidney and liver toxicity.
Tacrolimus (FK506) is an immunosuppressive agent and has toxic side effects such as nephrotoxicity, hepatotoxicity, and neurotoxicity. In our study, we aimed to investigate the protective effect of silymarin on renal and hepatic toxicity considered to be tacrolimus related.

Title: Effect of Short-Term Tacrolimus Exposure on Rat Liver: An Insight into Serum Antioxidant Status, Liver Lipid Peroxidation, and Inflammation.
Tacrolimus (TAC) is an immunosuppressive drug, optimally used for liver, kidney, and heart transplant to avoid immune rejection. In retrospect, a multitude of studies have reported effects of TAC, such as nephrotoxicity, diabetes, and other complications. However, limited information is available regarding short-term exposure of TAC on the liver. Therefore, the present study was designed to unravel the effects of short-term exposure of TAC on a rat model. The animal model was established by TAC administration for 6, 12, 24, and 48 h time points. Liver histopathological changes were observed with PAS-D, reticulin stain, and immunostaining of PCNA and CK-7... (truncated)

Title: Controversial Interactions of Tacrolimus with Dietary Supplements, Herbs and Food.
Tacrolimus is an immunosuppressive calcineurin inhibitor used to prevent rejection in allogeneic organ transplant recipients, such as kidney, liver, heart or lung. It is metabolized in the liver, involving the cytochrome P450 (CYP3A4) isoform CYP3A4, and is characterized by a narrow therapeutic window, dose-dependent toxicity and high inter-individual and intra-individual variability. In view of the abovementioned facts, the aim of the study is to present selected interactions between tacrolimus and the commonly used dietary supplements, herbs and food. The review was based on the available scientific literature found in the PubMed, Scopus and Cochrane databases. An increase in the serum... (truncated)

MESH:D001553 - benzbromarone

Summary:

LLM Prediction Score	0.846 (normalized)
LLM Confidence Score	0.880
Golden Answer (Severity Class)	1.0 (normalized)
Prediction Error	0.154

Retrieved Context:

Title: Benzbromarone aggravates hepatic steatosis in obese individuals.
As a widely used anti-gout drug, benzbromarone has been found to induce hepatic toxicity in patients during clinical treatment. Previous studies have reported that benzbromarone is metabolized via cytochrome P450, thus causing mitochondrial toxicity in hepatocytes. In this study, we found that benzbromarone significantly aggravated hepatic steatosis in both obese db/db mice and high fat diet (HFD)-induced obese (DIO) mouse models. However, benzbromarone had less effect on the liver of lean mice. It was found that the expression of mRNAs encoding lipid metabolism and some liver-specific genes were obviously disturbed in benzbromarone-treated DIO mice compared to the control group. The... (truncated)

Title: Hepatocellular toxicity of benzbromarone: effects on mitochondrial function and structure.
Benzbromarone is an uricosuric structurally related to amiodarone and a known mitochondrial toxicant. The aim of the current study was to improve our understanding in the molecular mechanisms of benzbromarone-associated hepatic mitochondrial toxicity. In HepG2 cells and primary human hepatocytes, ATP levels started to decrease in the presence of 25-50μM benzbromarone for 24-48h, whereas cytotoxicity was observed only at 100μM. In HepG2 cells, benzbromarone decreased the mitochondrial membrane potential starting at 50μM following incubation for 24h. Additionally, in HepG2 cells, 50μM benzbromarone for 24h induced mitochondrial uncoupling,and decreased mitochondrial ATP turnover and maximal respiration. This was accompanied by an increased... (truncated)

Title: Liver failure associated with benzbromarone: A case report and review of the literature.
Benzbromarone is a uricosuric agent that reduces proximal tubular reabsorption of uric acid. Because of hepatotoxicity, it has been withdrawn from the market in Europe. Recently, some benefit-risk assessments of benzbromarone suggest that benzbromarone has greater benefits than risks, and the application of benzbromarone in the treatment of gout and hyperuricemia is still under debate.

Title: Sequential metabolism and bioactivation of the hepatotoxin benzbromarone: formation of glutathione adducts from a catechol intermediate.
Benzbromarone (BBR) is a uricosuric agent that has been used as a treatment for chronic gout. Although never approved in the United States, BBR was recently withdrawn from European markets due to several clinical cases linking the drug to an idiosyncratic hepatotoxicity that is sometimes fatal. We report here a possible mechanism of toxicity that involves the bioactivation of BBR through sequential hydroxylation of the benzofuran ring to a catechol, which can then be further oxidized to a reactive quinone intermediate capable of adducting protein. NADPH-supplemented human liver microsomes generated a single metabolite that was identified as 6-OH BBR by... (truncated)

Title: Inactivation of CYP3A4 by Benzbromarone in Human Liver Microsomes.
Benzbromarone is a uricosuric drug in current clinical use that can cause serious hepatotoxicity. Chemically reactive and/or cytotoxic metabolites of benzbromarone have been identified; however there is a lack of available information on their role in benzbromarone hepatotoxicity. The reactive metabolites of some hepatotoxic drugs are known to covalently bind, or alternatively are targeted, to specific cytochrome P450 (P450) enzymes, a process that is often described as mechanism-based inhibition.

MESH:D020280 - sertraline

Summary:

LLM Prediction Score	0.528 (normalized)
LLM Confidence Score	0.990
Golden Answer (Severity Class)	0.375 (normalized)
Prediction Error	0.153

Retrieved Context:

Title: Acute liver injury secondary to sertraline.
Sertraline is widely prescribed to treat depression and anxiety disorders. However, hepatitis secondary to its use is a rare entity. We report the case of a 26-year-old woman in her 20th week of pregnancy presented with nausea, vomiting, malaise and dark urine. This occurred 6 months after sertraline 50 mg daily was started for the treatment of depression. Three weeks prior to her presentation, the dose of sertraline was increased to 100 mg daily. The patient's liver biochemical profile demonstrated increased transaminases. The biopsy of the liver showed lobular hepatitis, with a mild prominence of eosinophils, suggestive of a drug-induced... (truncated)

Title: The role of hepatic cytochrome P450s in the cytotoxicity of sertraline.
Sertraline, an antidepressant, is commonly used to manage mental health symptoms related to depression, anxiety disorders, and obsessive-compulsive disorder. The use of sertraline has been associated with rare but severe hepatotoxicity. Previous research demonstrated that mitochondrial dysfunction, apoptosis, and endoplasmic reticulum stress were involved in sertraline-associated cytotoxicity. In this study, we reported that after a 24-h treatment in HepG2 cells, sertraline caused cytotoxicity, suppressed topoisomerase I and IIα, and damaged DNA in a concentration-dependent manner. We also investigated the role of cytochrome P450 (CYP)-mediated metabolism in sertraline-induced toxicity using our previously established HepG2 cell lines individually expressing 14 CYPs (1A1,... (truncated)

Title: Sertraline, an antidepressant, induces apoptosis in hepatic cells through the mitogen-activated protein kinase pathway.
Sertraline is generally used for the treatment of depression and is also approved for the treatment of panic, obsessive-compulsive, and posttraumatic stress disorders. Previously, using rat primary hepatocytes and isolated mitochondria, we demonstrated that sertraline caused hepatic cytotoxicity and mitochondrial impairment. In the current study, we investigated and characterized molecular mechanisms of sertraline toxicity in human hepatoma HepG2 cells. Sertraline decreased cell viability and induced apoptosis in a dose- and time-dependent manner. Sertraline activated the intrinsic checkpoint protein caspase-9 and caused the release of cytochrome c from mitochondria to cytosol; this process was Bcl-2 family dependent because antiapoptotic Bcl-2 family... (truncated)

Title: Liver injury associated with antidepressants.
Antidepressants are commonly prescribed and used in the management of depression, anxiety disorders, and other psychiatric illnesses. Antidepressants used in therapeutic dosing ranges are associated with causing several adverse drug reactions including hepatotoxicity. Paroxetine, fluoxetine, fluvoxamine, citalopram, mirtazapine and venlafaxine are associated with reversible liver injury upon discontinuation of the agent. Patient cases of hepatotoxicity involving the use of nefazodone, trazodone, duloxetine, bupropion, and sertraline are linked to causing death in its users. Due to the idiosyncratic nature of hepatotoxicity, monitoring of liver function tests and immediate discontinuation upon abnormal lab findings or signs and symptoms of liver dysfunction are... (truncated)

Title: Severe liver toxicity in a chronic myeloid leukemia patient probably induced by a drug interaction between imatinib and sertraline.
Our objective is to describe a chronic myeloid leukemia patient with a severe liver toxicity likely due to a drug-drug interaction between imatinib and sertraline. The patient started treatment with sertraline three months after starting imatinib. From the beginning of sertraline treatment, the patient developed vomiting, and five weeks later she developed a severe hepatic failure and was admitted to the hospital. The Naranjo nomogram showed a probable correlation between this adverse effect and the interaction between imatinib and sertraline. This interaction is extremely rare and the mechanism of action is not clear; it could be a mix of pharmacokinetic... (truncated)

MESH:D000584 - amiloride

Summary:

LLM Prediction Score	0.222 (normalized)
LLM Confidence Score	0.980
Golden Answer (Severity Class)	0.375 (normalized)
Prediction Error	0.153

Retrieved Context:

Title: Amiloride reduces portal hypertension in rat liver cirrhosis.
This study aimed to investigate the effect of amiloride on portal hypertension. Amiloride is known to inhibit Na(+)/H(+) exchangers on activated hepatic stellate cells.

Title: The long-term follow-up after idiosyncratic drug-induced liver injury with jaundice.
Chronic evolution after drug-induced liver injury (DILI) has been reported. How often this leads to liver-related morbidity and mortality is unexplored.

Title: In vitro platforms for evaluating liver toxicity.
The liver is a heterogeneous organ with many vital functions, including metabolism of pharmaceutical drugs and is highly susceptible to injury from these substances. The etiology of drug-induced liver disease is still debated although generally regarded as a continuum between an activated immune response and hepatocyte metabolic dysfunction, most often resulting from an intermediate reactive metabolite. This debate stems from the fact that current animal and in vitro models provide limited physiologically relevant information, and their shortcomings have resulted in "silent" hepatotoxic drugs being introduced into clinical trials, garnering huge financial losses for drug companies through withdrawals and late stage... (truncated)

Title: Molecular mechanisms of ursodeoxycholic acid toxicity & side effects: ursodeoxycholic acid freezes regeneration & induces hibernation mode.
Ursodeoxycholic acid (UDCA) is a steroid bile acid approved for primary biliary cirrhosis (PBC). UDCA is reported to have "hepato-protective properties". Yet, UDCA has "unanticipated" toxicity, pronounced by more than double number of deaths, and eligibility for liver transplantation compared to the control group in 28 mg/kg/day in primary sclerosing cholangitis, necessitating trial halt in North America. UDCA is associated with increase in hepatocellular carcinoma in PBC especially when it fails to achieve biochemical response (10 and 15 years incidence of 9% and 20% respectively). "Unanticipated" UDCA toxicity includes hepatitis, pruritus, cholangitis, ascites, vanishing bile duct syndrome, liver cell failure,... (truncated)

Title: Pharmacokinetics of amiloride in renal and hepatic disease.
The pharmacokinetics of the antikaliuretic amiloride has been studied in healthy controls and in patients with chronic renal failure or hepatitis. It was 40% bound to protein. In healthy volunteers 49% of an oral dose was recovered unchanged in the urine. The renal clearance of amiloride was about 3 times the creatinine clearance, which means that it was predominantly excreted via tubular secretion. Renal impairment reduced the clearance of amiloride, causing a prolongation of the t1/2 and drug accumulation in plasma. In hepatitis the t1/2 of amiloride was prolonged and the AUC increased. Urinary recovery (Ae) of amiloride was greater... (truncated)

MESH:D007548 - isosorbide dinitrate

Summary:

LLM Prediction Score	0.153 (normalized)
LLM Confidence Score	0.990
Golden Answer (Severity Class)	0.0 (normalized)
Prediction Error	0.153

Retrieved Context:

Title: A One-Two Punch: Hydralazine-Induced Liver Injury in a Recovering Ischemic Hepatitis.
A 77-year-old woman presented to the emergency department with a 2-day history of nausea and vomiting. Her medical history included diabetes mellitus, hypertension, atrial fibrillation, dilated cardiomyopathy, and coronary artery disease. Her home medications included aspirin, clopidogrel, warfarin, digoxin, metoprolol, losartan, simvastatin, isosorbide dinitrate, furosemide, and spironolactone. Initial physical examination showed blood pressure of 170/80 mm Hg with a heart rate of 69 beats per minute, otherwise unremarkable. Initial laboratory workup was significant for INR of 3.6, with slightly elevated troponin I and creatinine of 0.06 ng/mL and 1.4 mg/dL, respectively. The patient was admitted to the medicine floor. However,... (truncated)

Title: A comparative study on the anti-schistosomal and hepatoprotective effects of vinpocetine and isosorbide-5-mononitrate on Schistosoma mansoni-infected mice.
Schistosomiasis is a remarkable public health problem in developing countries. Presently, praziquantel is the optional drug for all human schistosomiasis. Owing to the increased praziquantel resistance, there is an urgent need to develop new alternatives. This study aims at determining the anti-schistosomal and/or the hepatoprotective effects of the anti-inflammatory drug; vinpocetine, and the vasodilator and the nitric oxide donor; isosorbide-5-mononitrate, in comparison to praziquantel. In the present research, the therapeutic efficacies of these drugs were assessed in Swiss albino female mice (CD-I strain) experimentally infected with an Egyptian strain of Schistosoma mansoni, using some general, parasitological, and histopathological parameters. In... (truncated)

Title: [Biliary atresias operated with favourable results: predictable outcome].
Since 1975, our experience in the treatment of biliary atresia with Kasai's technique has improved little by little, achieving 65% favourable outcome in the last five years. We define "good results" as the complete restoration of biliary flow and normalization of bilirrubin levels. The long-term evolution of these good results can be diverse. The objective of the present work is to analyze the outcome of patients in our series in whom a favourable initial response was achieved, as well as evaluating their present situation and future perspectives. The authors present a total of 17 patients operated by Kasai's technique since... (truncated)

Title: Diagnosis and treatment of alcoholic liver disease and its complications.
Alcoholic liver disease (ALD) is a serious and potentially fatal consequence of alcohol use. The diagnosis of ALD is based on drinking history, physical signs and symptoms, and laboratory tests. Treatment strategies for ALD include lifestyle changes to reduce alcohol consumption, cigarette smoking, and obesity; nutrition therapy; and pharmacological therapy. The diagnosis and management of the complications of ALD are important for alleviating the symptoms of the disease, improving quality of life, and decreasing mortality.

Title: Association of CYP1A1 and CYP1B1 inhibition in in vitro assays with drug-induced liver injury.
Drug-induced liver injury (DILI) is one of the major causes for the discontinuation of drug development and withdrawal of drugs from the market. Since it is known that reactive metabolite formation and being substrates or inhibitors of cytochrome P450s (P450s) are associated with DILI, we systematically investigated the association between human P450 inhibition and DILI. The inhibitory activity of 266 DILI-positive drugs (DILI drugs) and 92 DILI-negative drugs (no-DILI drugs), which were selected from Liver Toxicity Knowledge Base (US Food and Drug Administration), against 8 human P450 forms was assessed using recombinant enzymes and luminescent substrates, and the threshold values... (truncated)

MESH:C009927 - estropipate

Summary:

LLM Prediction Score	0.098 (normalized)
LLM Confidence Score	0.870
Golden Answer (Severity Class)	0.25 (normalized)
Prediction Error	0.152

Retrieved Context:

Title: Acute alcohol-induced liver injury.
Alcohol consumption is customary in most cultures and alcohol abuse is common worldwide. For example, more than 50% of Americans consume alcohol, with an estimated 23.1% of Americans participating in heavy and/or binge drinking at least once a month. A safe and effective therapy for alcoholic liver disease (ALD) in humans is still elusive, despite significant advances in our understanding of how the disease is initiated and progresses. It is now clear that acute alcohol binges not only can be acutely toxic to the liver, but also can contribute to the chronicity of ALD. Potential mechanisms by which acute alcohol... (truncated)

Title: Role of hemostatic factors in hepatic injury and disease: animal models de-liver.
Chronic liver damage is associated with unique changes in the hemostatic system. Patients with liver disease often show a precariously rebalanced hemostatic system, which is easily tipped towards bleeding or thrombotic complications by otherwise benign stimuli. In addition, some clinical studies have shown that hemostatic system components contribute to the progression of liver disease. There is a strong basic science foundation for clinical studies with this particular focus. Chronic and acute liver disease can be modeled in rodents and large animals with a variety of approaches, which span chronic exposure to toxic xenobiotics, diet-induced obesity, and surgical intervention. These experimental... (truncated)

Title: Alcoholic liver disease and the potential role of plasminogen activator inhibitor-1 and fibrin metabolism.
Plasminogen activator inhibitor-1 (PAI-1) is a major player in fibrinolysis due to its classical role of inhibiting plasminogen activators. Although increased fibrinolysis is common in alcoholic cirrhosis, decreased fibrinolysis (driven mostly by elevated levels of PAI-1) is common during the development of alcoholic liver disease (ALD). However, whether or not PAI-1 plays a causal role in the development of early ALD was unclear. Recent studies in experimental models have suggested that PAI-1 may contribute to the development of early (steatosis), intermediate (steatohepatitis) and late (fibrosis) stages of ALD. For example, fatty liver owing to both acute and chronic ethanol was... (truncated)

Title: Role of Fibrin(ogen) in Progression of Liver Disease: Guilt by Association?
Strong experimental evidence indicates that components of the hemostatic system, including thrombin, exacerbate diverse features of experimental liver disease. Clinical studies have also begun to address this connection and some studies have suggested that anticoagulants can improve outcome in patients with liver disease. Among the evidence of coagulation cascade activation in models of liver injury and disease is the frequent observation of thrombin-driven hepatic fibrin(ogen) deposition. Indeed, hepatic fibrin(ogen) deposition has long been recognized as a consequence of hepatic injury. Although commonly inferred as pathologic due to protective effects of anticoagulants in mouse models, the role of fibrin(ogen) in acute... (truncated)

Title: Advances in alcoholic liver disease.
Alcoholic liver disease (ALD) remains a leading cause of death from liver disease in the United States. In studies from the Veterans Administration, patients with cirrhosis and superimposed alcoholic hepatitis had greater than 60% mortality over a 4-year period, with most of those deaths occurring in the first month. Thus, the prognosis for this disease is more ominous than for many common types of cancer (eg, breast, prostate, and colon). Moreover, ALD imposes a significant economic burden from lost wages, health care costs, and lost productivity. Unfortunately, there is still no Food and Drug Administration-approved or widely accepted drug therapy... (truncated)

MESH:D002974 - clemastine

Summary:

LLM Prediction Score	0.149 (normalized)
LLM Confidence Score	0.980
Golden Answer (Severity Class)	0.0 (normalized)
Prediction Error	0.149

Retrieved Context:

Title: [Cholestatic jaundice after ingestion of amoxicillin and clavulanic acid].
A 61-year-old woman fell ill with recurrent nausea, loss of appetite and tiredness. Five days later she noted increasing jaundice of skin and sclerae, pale stools and dark urine, and she developed itching over the whole body. Among biochemical tests alkaline phosphatase (537 U/l) and bilirubin (32.0 mg/dl) were markedly increased, while both GOT (102 U/l) and GPT (39 U/l) were only slightly elevated. Ultrasonography was normal and extrahepatic cholestasis appeared unlikely on endoscopic retrograde cholangiopancreatography. Cholestasis due to virus hepatitis was also excluded. It was only on repeated and direct questioning that the patient reported having taken three tablets... (truncated)

Title: Treatment-Related Toxicities During Anti-GD2 Immunotherapy in High-Risk Neuroblastoma Patients.
The introduction of immunotherapy using an anti-GD2 antibody (dinutuximab, ch14.18) has significantly improved survival rates for high-risk neuroblastoma patients. However, this improvement in survival is accompanied by a substantial immunotherapy-related toxicity burden. The primary objective of this study was to describe treatment-related toxicities during immunotherapy with dinutuximab, IL-2, GM-CSF, and isotretinoin. A retrospective, single center analysis of immunotherapy-related toxicities was performed in twenty-six consecutive high-risk neuroblastoma patients who received immunotherapy as maintenance therapy in the Princess Máxima Center (Utrecht, Netherlands). Toxicities were recorded and graded according to the CTCAE. Particular attention was drawn to pain and fever management and toxicities... (truncated)

Title: Alcohol and medication interactions.
Many medications can interact with alcohol, thereby altering the metabolism or effects of alcohol and/or the medication. Some of these interactions can occur even at moderate drinking levels and result in adverse health effects for the drinker. Two types of alcohol-medication interactions exist: (1) pharmacokinetic interactions, in which alcohol interferes with the metabolism of the medication, and (2) pharmacodynamic interactions, in which alcohol enhances the effects of the medication, particularly in the central nervous system (e.g., sedation). Pharmacokinetic interactions generally occur in the liver, where both alcohol and many medications are metabolized, frequently by the same enzymes. Numerous classes of... (truncated)

Title: Additional hepatic ¹⁶⁶Ho-radioembolization in patients with neuroendocrine tumours treated with ¹⁷⁷Lu-DOTATATE; a single center, interventional, non-randomized, non-comparative, open label, phase II study (HEPAR PLUS trial).
Label="BACKGROUND" NlmCategory="BACKGROUND">Neuroendocrine tumours (NET) consist of a heterogeneous group of neoplasms with various organs of origin. At diagnosis 21% of the patients with a Grade 1 NET and 30% with a Grade 2 NET have distant metastases. Treatment with peptide receptor radionuclide therapy (PRRT) shows a high objective response rate and long median survival after treatment. However, complete remission is almost never achieved. The liver is the most commonly affected organ in metastatic disease and is the most incriminating factor for patient survival. Additional treatment of liver disease after PRRT may improve outcome in NET patients. Radioembolization is an established... (truncated)

Title: Relationships Between Pharmacovigilance, Molecular, Structural, and Pathway Data: Revealing Mechanisms for Immune-Mediated Drug-Induced Liver Injury.
Immune-mediated drug-induced liver injury (IMDILI) can be devastating, irreversible, and fatal in the absence of successful transplantation surgery. We present a novel approach that combines the methods of pharmacoepidemiology with in silico molecular modeling to identify specific features in toxic ligands that are associated with clinical features of IMDILI. Specifically, from pharmacovigilance data multivariate logistic regression identified 18 drugs associated with IMDILI ($P < 0.00015$). Eleven of these drugs, along with their known and proposed metabolites, constituted a training set used to develop a four-point pharmacophore model (sensitivity 75%; specificity 85%). Subsequently, this information was

MESH:C065507 - ferumoxides

Summary:

LLM Prediction Score	0.148 (normalized)
LLM Confidence Score	0.950
Golden Answer (Severity Class)	0.0 (normalized)
Prediction Error	0.148

Retrieved Context:

Title: Assessment and comparison of magnetic nanoparticles as MRI contrast agents in a rodent model of human hepatocellular carcinoma.
The purpose of this study was to synthesize, characterize and tailor the surface properties of magnetic nanoparticles with biocompatible copolymer coatings and to evaluate the efficiency of the resulting nanoconjugates as magnetic resonance imaging (MRI) contrast agents for liver imaging. Magnetic nanoparticles with core diameters of 10 and 30 nm were synthesized by pyrolysis and were subsequently coated with a copolymer containing either carboxyl (SHP) or methoxy groups as termini. All four formulas, and ferumoxides (Feridex I.V.(®)), were individually injected intravenously into separate, normal Balb/C mice (at 2.5, 1.0 and 0.56 mg Fe kg(-1)), and the animals underwent T(2)-weighted MRI... (truncated)

Title: MR imaging in the evaluation of hepatic metastases.
Optimal detection of focal hepatic lesions in patients with metastases can alter patient management and result in significant cost savings by reducing the number of unnecessary laparotomies for unresectable disease. Liver-specific MR imaging contrast agents (reticuloendothelial and hepatobiliary agents) offer greater lesion-to-liver contrast than the conventional extracellular fluid space MR imaging contrast agents (gadolinium chelates), which have a nonspecific distribution. For the detection of hepatic metastases, although the work of Seneterre et al suggests that the accuracy of ferumoxide-enhanced MR imaging is equivalent to that of CTAP, other studies find CTAP to be superior. Comparisons of reticuloendothelial agents and hepatobiliary... (truncated)

Title: Superparamagnetic iron oxide hepatic MR imaging: efficacy and safety using conventional and fast spin-echo pulse sequences.
The purpose of this study was to evaluate the technical efficacy and safety of iv ferumoxides (Feridex), a superparamagnetic iron oxide contrast agent for detection of hepatic lesions using conventional spin-echo and fast spin-echo MR images. Precontrast and postcontrast MR studies were performed on 25 patients with suspected focal hepatic lesions. Conventional T1- and T2-weighted MR images, as well as fast spin-echo and fat suppressed fast spin-echo MR images, were evaluated. Quantitative assessment of the contrast agent was performed obtaining region of interest measurements of the liver, spleen, and selected hepatic lesions. The pulse sequences were also evaluated subjectively for... (truncated)

Title: Conspicuity of hepatocellular nodular lesions in cirrhotic livers at ferumoxides-enhanced MR imaging: importance of Kupffer cell number.
To correlate the conspicuity of hepatocellular carcinomas and dysplastic nodules on ferumoxides-enhanced magnetic resonance (MR) images with the number of Kupffer cells in the hepatic lesions, as compared with that in background liver in histopathologic findings.

Title: Detection of hepatocellular carcinoma arising in cirrhotic livers: comparison of gadolinium- and ferumoxides-enhanced MR imaging.
We prospectively compared the detectability of hepatocellular carcinoma (HCC) arising in cirrhotic livers using dynamic gadolinium-enhanced fast low-angle shot (FLASH), ferumoxides-enhanced T2-weighted turbo spin-echo, and ferumoxides-enhanced T2*-weighted FLASH MR imaging.

MESH:D000077204 - temozolomide

Summary:

LLM Prediction Score	0.647 (normalized)
LLM Confidence Score	0.990
Golden Answer (Severity Class)	0.5 (normalized)
Prediction Error	0.147

Retrieved Context:

Title: Acute temozolomide induced liver injury: mixed type hepatocellular and cholestatic toxicity.
Temozolomide (TMZ) is an oral imidazotetrazine methylating agent which is used for the treatment of glioblastoma multiforme (GBM). We report a case of acute hepatotoxicity in a 53-year old male patient after administration of TMZ for GBM. He had fatigue, nausea, anorexia and jaundice. His laboratory analysis showed alanine aminotransferase(ALT): 632 IU/L (normal range 0-40); aspartate aminotransferase(AST): 554 IU/L (normal range 5-34); alkaline phosphatase(ALP): 1143 IU/L (normal range 40-150); γ-glutamyl transpeptidase(GGT): 514 IU/L (normal range 9-64 IU/L); total bilirubin: 15.1 mg/dL (normal range 0-1.2); direct bilirubin: 13.2 mg/dL and prothrombin time(PT): 13.5 s, with international normalized ratio (INR): 1.1 (normal... (truncated)

Title: Acute temozolomide induced liver injury : Mixed type hepatocellular and cholestatic toxicity.
Temozolomide (TMZ) is an oral imidazotetrazine methylating agent which is used for the treatment of glioblastoma multiforme (GBM). We report a case of acute hepatotoxicity in a 53-year old male patient after administration of TMZ for GBM. He had fatigue, nausea, anorexia and jaundice. His laboratory analysis showed alanine aminotransferase(ALT) : 632 IU/L (normal range 0-40) ; aspartate aminotransferase(AST) : 554 IU/L (normal range 5-34) ; alkaline phosphatase(ALP) : 1143 IU/L (normal range 40-150) ; γ-glutamyl transpeptidase(GGT) : 514 IU/L (normal range 9-64 IU/L) ; total bilirubin : 15.1 mg/dL (normal range 0-1.2) ; direct bilirubin : 13.2 mg/dL and... (truncated)

Title: Temozolomide-induced biliary ductopenia: a case report.
Temozolomide is an alkylating agent used along with concurrent radiation therapy in the treatment of glioblastoma. The primary adverse effect of temozolomide is bone marrow suppression with resulting cytopenias. There have been reported cases of temozolomide-induced hepatotoxicity, including fatal liver failure, associated with reactivation of the hepatitis virus or with concurrent use of other hepatotoxic drugs. In this report, we describe a unique mechanism of temozolomide-induced liver injury with supporting histopathology.

Title: Severe cholestatic hepatitis due to temozolomide: an adverse drug effect to keep in mind. Case report and review of literature.
Temozolomide is the current standard of therapy for postoperative patients with glioblastoma starting adjuvant radiotherapy. Hematologic adverse events are the most frequent side effects of temozolomide, while liver toxicity has been reported only in the post-marketing period. Here we report a case of severe temozolomide-induced liver injury during concurrent radiotherapy treatment, at a dose level of 75 mg/m2. The aim of this case report is to focus on the problems of temozolomide-induced hepatotoxicity. In conclusion, a close monitoring of liver function tests is recommended during treatment with temozolomide.

Title: Liver toxicity during temozolomide chemotherapy caused by Chinese herbs.
Complementary and alternative medicine is often used by patients with malignant glioma. Although several interactions of various alternative agents with chemotherapy are known, none has been described for temozolomide so far.

MESH:D011345 - fenofibrate

Summary:

LLM Prediction Score	0.521 (normalized)
LLM Confidence Score	0.980
Golden Answer (Severity Class)	0.375 (normalized)
Prediction Error	0.146

Retrieved Context:

Title: Biopsy-confirmed fenofibrate-induced severe jaundice: A case report.
Drug-induced liver injury (DILI) is the leading cause of acute liver failure in the United States. DILI is mainly caused by painkillers and fever reducers, and it is often characterized by the type of hepatic injury (hepatocellular or cholestatic). This report presents a case of fenofibrate-induced severe jaundice in a 65-year-old Korean male with no prior history of liver disease. We offer a strategy for patients who present signs of severe liver injury with jaundice and high elevations in serum transaminases.

Title: Fenofibrate Improves Liver Function and Reduces the Toxicity of the Bile Acid Pool in Patients With Primary Biliary Cholangitis and Primary Sclerosing Cholangitis Who Are Partial Responders to Ursodiol.
Cholestatic liver diseases result in the hepatic retention of bile acids, causing subsequent liver toxicity. Peroxisome proliferator-activated receptor alpha (PPARα) regulates bile acid metabolism. In this retrospective observational study, we assessed the effects of fenofibrate (a PPARα agonist) therapy on bile acid metabolism when given to patients with primary biliary cholangitis (PBC) and primary sclerosing cholangitis (PSC) who have had an incomplete response to Ursodiol monotherapy. When fenofibrate was added to Ursodiol therapy there was a significant reduction and in some cases normalization of serum alkaline phosphatase, alanine aminotransferase, and aspartate aminotransferase abnormalities, as well as pro-inflammatory cytokines. Combination fenofibrate... (truncated)

Title: Hepatotoxic effects of fenofibrate in spontaneously hypertensive rats expressing human C-reactive protein.
Dyslipidemia and inflammation play an important role in the pathogenesis of cardiovascular and liver disease. Fenofibrate has a well-known efficacy to reduce cholesterol and triglycerides. Combination with statins can ameliorate hypolipidemic and anti-inflammatory effects of fibrates. In the current study, we tested the anti-inflammatory and metabolic effects of fenofibrate alone and in combination with rosuvastatin in a model of inflammation and metabolic syndrome, using spontaneously hypertensive rats expressing the human C-reactive protein transgene (SHR-CRP transgenic rats). SHR-CRP rats treated with fenofibrate alone (100 mg/kg body weight) or in combination with rosuvastatin (20 mg/kg body weight) vs. SHR-CRP untreated controls showed... (truncated)

Title: Identification and Characterization of Fenofibrate-Induced Liver Injury.
Fenofibrate is a commonly used hypolipidemic associated with rare instances of hepatotoxicity, and routine liver biochemistry monitoring is recommended.

Title: Fenofibrate-induced hepatotoxicity: A case with a special feature that is different from those in the LiverTox database.
We report a special case of fenofibrate-induced acute severe DILI with sudden onset and rapid recovery, which is different from those in the LiverTox database.

MESH:D013881 - thioridazine

Summary:

LLM Prediction Score	0.479 (normalized)
LLM Confidence Score	0.970
Golden Answer (Severity Class)	0.625 (normalized)
Prediction Error	0.146

Retrieved Context:

Title: Pimozide in chronic schizophrenic outpatients.
In a double blind placebo controlled clinical evaluation of maintenance therapy in chronic schizophrenic female outpatients, thiordazine in single daily doses not exceeding 375 mg./day for 6 months was shown to be effective maintenance treatment compared with PL, thereby establishing the sensitivity of the experiment. Pimozide was also shown to be effective in a single oral dose not exceeding 16 mg./day and comparable overall to the standard drug. The experimental design was based on the anticipated retrogression of PL treated subjects during the 6-month study period, which was reflected in 5 of 9 (56%) "treatment failures" in the PL group... (truncated)

Title: Psychotropic drugs and liver disease: A critical review of pharmacokinetics and liver toxicity.
The liver is the organ by which the majority of substances are metabolized, including psychotropic drugs. There are several pharmacokinetic changes in end-stage liver disease that can interfere with the metabolization of psychotropic drugs. This fact is particularly true in drugs with extensive first-pass metabolism, highly protein bound drugs and drugs depending on phase I hepatic metabolic reactions. Psychopharmacological agents are also associated with a risk of hepatotoxicity. The evidence is insufficient for definite conclusions regarding the prevalence and severity of psychiatric drug-induced liver injury. High-risk psychotropics are not advised when there is pre-existing liver disease, and after starting a... (truncated)

Title: [Therapeutic effect and plasma level of thioridazine in schizophrenic patients (author's transl)].
1. In 18 Patients the plasma concentration of the neuroleptic drug thioridazine was measured twice a week by a fluorometric method during a period of treatment lasting for an average of 27 days. At the same time the psychopathological findings were recorded by means of the AMP system. 2. A significant correlation was found both between dosage per kg body weight and plasma level, and between age and plasma level. No connection with the sex of the patient could be demonstrated. 3. A curve-linear correlation appears to exist between plasma concentration and remission of symptoms. 4. In the course of... (truncated)

Title: Targeting Ca²⁺ and Mitochondrial Homeostasis by Antipsychotic Thioridazine in Leukemia Cells.
Mitochondria have pivotal roles in cellular physiology including energy metabolism, reactive oxygen species production, Ca²⁺ homeostasis, and apoptosis. Altered mitochondrial morphology and function is a common feature of cancer cells and the regulation of mitochondrial homeostasis has been identified as a key to the response to chemotherapeutic agents in human leukemias. Here, we explore the mechanistic aspects of cytotoxicity produced by thioridazine (TR), an antipsychotic drug that has been investigated for its anticancer potential in human leukemia cellular models. TR exerts selective cytotoxicity against human leukemia cells in vitro. A PCR array provided a general view of the expression of... (truncated)

Title: Drug-Associated Liver Injury Related to Antipsychotics: Exploratory Analysis of Pharmacovigilance Data.
Drug-associated liver injury is one of the most common causes for acute liver failure and market withdrawal of approved drugs. In addition, the potential for hepatotoxicity related to specific substances has to be considered in psychopharmacotherapy. However, systematic evaluations of hepatotoxicity related to antipsychotics are limited.

MESH:D016597 - trimetrexate

Summary:

LLM Prediction Score	0.520 (normalized)
LLM Confidence Score	0.920
Golden Answer (Severity Class)	0.375 (normalized)
Prediction Error	0.145

Retrieved Context:

Title: Correlates of severe or life-threatening toxic effects from trimetrexate.
Trimetrexate, an investigational antifol, has been associated with marked variability in drug tolerance among patients. The agent is extensively protein bound, and hepatic biotransformation plays a major role in its elimination. In early phase II testing, nine of 15 patients who experienced life-threatening or fatal toxic effects from trimetrexate had albumin levels less than or equal to 3.5 g/dL prior to treatment. This prompted a review of the data base on 272 patients entered in phase I clinical trails. The incidence of severe or life-threatening anemia, leukopenia, neutropenia, thrombocytopenia, mucositis, and hepatic toxic effects during the first course of trimetrexate... (truncated)

Title: Fungal infections: their diagnosis and treatment in transplant recipients.
Systemic fungal infections typically occur in individuals who are seriously ill with recognized risk factors such as those frequently found in transplant recipients. Unfortunately, they are often diagnosed late, when the efficacy of the available treatments is low, often less than 50%, and the cost in terms of lives lost, hospital length of stay, and total hospital costs is substantially increased. The application of antifungal therapies associated with reported efficacy rates greater than 50% are those used prophylactically. When used prophylactically, these infections are reduced in greater than 95% of the expected cases. The choice of a prophylactic agent should... (truncated)

Title: Pharmacokinetics and Biodistribution of Pegylated Methotrexate after IV Administration to Mice.
The efficacy of methotrexate (MTX) as an antimetabolite chemotherapeutic agent highly depends on its blood circulation half-life. In our previous study, different conjugates of MTX (MTX-PEG) were synthesized, their physicochemical properties were investigated and MTX-PEG5000 was finally selected as optimum drug-conjugate for further investigations. In the current work, first the stability of MTX-PEG5000 was studied at 37 °C and the results indicated its high stability in plasma (T_{1/2} = 144 h) and a relatively rapid degradation in tissue homogenate (T_{1/2} = 24 h). The study of protein binding pointed out that the conjugate was highly protein-bound (95%). The results of... (truncated)

Title: Management of Pneumocystis Jirovecii pneumonia in HIV infected patients: current options, challenges and future directions.
The discovery of the Human Immunodeficiency Virus (HIV) was led by the merge of clustered cases of Pneumocystis jirovecii Pneumonia (PCP) in otherwise healthy people in the early 80's.1,2 In the face of sophisticated treatment now available for HIV infection, life expectancy approaches normal limits. It has dramatically changed the natural course of HIV from a nearly fatal infection to a chronic disease.3-5 However, PCP still remains a relatively common presentation of uncontrolled HIV. Despite the knowledge and advances gained in the prevention and management of PCP infection, it continues to have high morbidity and mortality rates. Trimethoprim-sulfamethoxazole (TMP-SMZ) remains... (truncated)

Title: Sunitinib malate in the treatment of recurrent or persistent uterine leiomyosarcoma: a Gynecologic Oncology Group phase II study.
New agents are needed for patients with metastatic uterine leiomyosarcoma who progress after treatment with doxorubicin or gemcitabine-docetaxel. Agents targeting tumor vasculature have potential for activity in leiomyosarcoma. We aimed to assess the activity of sunitinib in patients with recurrent uterine leiomyosarcoma who had received one or two prior therapies by determining the frequency of patients who survived progression-free for at least 6 months or who achieved objective tumor response. We also aimed to characterize the toxicity of sunitinib and to estimate time-to-progression.

MESH:D014700 - verapamil

Summary:

LLM Prediction Score	0.519 (normalized)
LLM Confidence Score	0.990
Golden Answer (Severity Class)	0.375 (normalized)
Prediction Error	0.144

Retrieved Context:

Title: Liver injury due to verapamil.
A 56-year-old female patient on verapamil for hypertension experienced two episodes of jaundice, pruritus and upper abdominal pain with transaminase elevated up to six-fold and alkaline phosphatase up to four-fold when inadvertently re-challenged with the drug. Liver biopsy showed marked cholestasis. Verapamil can occasionally cause mixed cytotoxic-cholestatic liver injury.

Title: [Verapamil-associated liver injury].
Hepatotoxicity due to verapamil is very rare and to the best of our knowledge only 10 cases have been reported. A 54-year-old woman developed cholestatic liver injury and pruritus following treatment with sustained-release verapamil (240 mg/day) for arterial hypertension. The pruritus and all hepatic biochemical abnormalities completely resolved after withdrawal of the drug. Similar to previously reported cases, the pathogenic mechanism of verapamil-associated liver injury in our patient was, most probably, idiosyncratic. These cases emphasize the need for awareness of the possibility that verapamil may occasionally induce liver injury, sometimes severe and potentially fatal.

Title: Effect of subclinical, clinical and supraclinical doses of calcium channel blockers on models of drug-induced hepatotoxicity in rats.
Drug-related hepatotoxicity is the leading cause of acute liver failure, and hepatic problems are responsible for a significant number of liver transplantations and deaths worldwide. Calcium has been associated with various metabolic processes that lead to cell death and apoptosis, and increased cytosolic Ca^{2+} has been implicated in hepatotoxicity. This study was designed to investigate the effects of calcium channel blockers (CCBs) on isoniazid-rifampicin, zidovudine and erythromycin-induced hepatotoxicity in rats. Treatment groups comprised control, hepatotoxicant, hepatotoxicant along with each of silymarin, nifedipine, verapamil and diltiazem at subclinical, clinical and supraclinical doses. A day to the end of treatment for each... (truncated)

Title: Comparative Study of Protective Effect of Cimetidine and Verapamil on Paracetamol-Induced Hepatotoxicity in Mice.
Paracetamol, chemically known as acetaminophen, if taken in higher doses has hepatotoxic potential. Cimetidine by inhibiting the cytochromal enzymes and reducing the production of the toxic metabolite can reduce the hepatotoxic potential while Verapamil can act as a hepatoprotective by maintaining calcium homeostasis. The present study was conducted to study the hepatoprotective activity of Cimetidine and Verapamil against the toxicity induced by paracetamol. In addition to the group receiving only distilled water or 300 mg/kg paracetamol additional groups were added treated with 150 mg/kg Cimetidine and Verapamil alone or both. The Liver function tests and histopathology revealed hepatotoxicity in the... (truncated)

Title: Verapamil-loaded supramolecular hydrogel patch attenuates metabolic dysfunction-associated fatty liver disease via restoration of autophagic clearance of aggregated proteins and inhibition of NLRP3.
Obesity, a serious threat to public health, is linked to chronic metabolic complications including insulin resistance, type-2 diabetes, and metabolic dysfunction-associated fatty liver disease (MAFLD). Current obesity medications are challenged by poor effectiveness, poor patient compliance, and potential side effects. Verapamil is an inhibitor of L-type calcium channels, FDA-approved for the treatment of hypertension. We previously investigated the effect of verapamil on modulating autophagy to treat obesity-associated lipotoxicity. This study aims to develop a verapamil transdermal patch and to evaluate its anti-obesity effects.

MESH:D006220 - haloperidol

Summary:

LLM Prediction Score	0.482 (normalized)
LLM Confidence Score	0.990
Golden Answer (Severity Class)	0.625 (normalized)
Prediction Error	0.143

Retrieved Context:

Title: [Hepatic tolerance of atypical antipsychotic drugs].
The strategy in the choice of antipsychotic agent must take into account the hepatic tolerance according to non-negligible incidence of liver disorders among psychiatric population (presence of risk factors like alcoholism, drugs of abuse intake, polymedication including potentially hepatotoxic drugs.). More than 1 000 drugs have been listed as being responsible of hepatic side effects; 16% of these agents were neuropsychiatric drugs. Antidepressive drugs (tricyclic agents or SSRI), mood stabilizing agents and neuroleptic drugs have been implicated in biological or/and clinical hepatotoxicity. For these reasons, some psychotropic agents have been withdrawn of the pharmaceutical market like alpidem or medifoxamine. Atrium*,... (truncated)

Title: Identifying olanzapine induced liver injury in the setting of acute hepatitis C: A case report.
Olanzapine is linked to asymptomatic, transient elevations of liver aminotransferases but is historically thought to rarely cause significant hepatotoxicity. Underlying liver disease is a risk factor for drug-induced liver injury and may complicate the differential diagnosis of acute transaminitis in patients taking medications associated with hepatotoxicity. Ms L presented with 2 months of new psychotic symptoms resulting in hospitalizations. Although psychosis previously improved with haloperidol, she reported symptoms concerning for akathisia. Restlessness improved and psychotic symptoms resolved after initiation of olanzapine. Concurrently, her alanine aminotransferase (ALT) was elevated, prompting further workup and new diagnosis of acute hepatitis C. Over the... (truncated)

Title: [Cytolytic hepatitis during treatment with phenothiazines: apropos of a case].
In contrast to the well known chlorpromazine-induced cholestatic hepatitis, we report the case of a schizophrenic patient who presents a cytolytic hepatitis, without any prior hepatic disease. Mr G. was first hospitalized for depressive symptomatology. A pseudo-nevrotic schizophrenia was diagnosed. Pretherapeutic clinical and biological data were normal. A treatment with chlorpromazine 400 mg/day was given. At day 8, the patient was still anxious and began to be agitated. An increase to 500 mg/day of chlorpromazine posology and an addition of haloperidol 200 mg/day was implemented. At day 10, the following clinical symptoms appeared: 38.6 degrees C fever; headache; myalgia; epigastralgia... (truncated)

Title: Effect of chronic treatment of haloperidol on the rat liver: a stereological and histopathological study.
Haloperidol is commonly used in therapy for patients with acute and chronic schizophrenia. Because it can have some adverse effects on specific target organs such as the liver, we analyzed whether haloperidol exerts a toxic effect on rat liver by means of stereological and histopathological methods. Fifteen adult male rats, divided into three groups, were used in the experiments. Once a day for 6 weeks, either saline or 0.4 or 0.8 mg kg(-1) doses of haloperidol were given interperitoneally to the control, low-dose, and high-dose groups, respectively. At the end of the experiment, rats were killed by an overdose of... (truncated)

Title: Gene expression on liver toxicity induced by administration of haloperidol in rats with severe fatty liver.
Sudden deaths are often encountered in schizophrenic patients prescribed with antipsychotic drugs, and fatty liver may be more prevalent among patients with schizophrenia. The aim of this study is to investigate the adverse effects of antipsychotic drugs on fatty liver. We administered haloperidol intraperitoneally to fatty liver rats and examined the mRNA expression in the liver. Basic expressions of cytochrome P450 (CYP)1A2, CYP2C11 and CYP3A2 decreased, and response of these CYPs to haloperidol was reduced in the fatty liver. Metabolism of haloperidol was also suppressed in the fatty liver rats. Moreover, hepatic injury by administration of haloperidol was shown pathohistologically... (truncated)

MESH:D000547 - amantadine

Summary:

LLM Prediction Score	0.233 (normalized)
LLM Confidence Score	0.990
Golden Answer (Severity Class)	0.375 (normalized)
Prediction Error	0.142

Retrieved Context:

Title: Amantadine-associated delirium in patients with maintenance dialysis: Insomnia-associated recovery and uneven seasonal distribution.
Amantadine hydrochloride is a risky drug for triggering delirium in dialysis patients; however, it is often administered casually. Furthermore, little is known regarding the recovery and prognosis of dialysis patients with amantadine-associated delirium. Data of this retrospective cohort study were collected from a local hospital database for hospitalizations between January 2011 and December 2020. Patients were divided into 2 cohorts: early recovery (recovery within 14 days) and delayed recovery (recovery more than 14 days). The cases were analyzed together with the intermonth temperature using descriptive statistics. A Kaplan-Meier survival curve and binary logistic regression were applied for the analyses of... (truncated)

Title: Inhibitor Development against p7 Channel in Hepatitis C Virus.
Hepatitis C Virus (HCV) is the key cause of chronic and severe liver diseases. The recent direct-acting antiviral agents have shown the clinical success on HCV-related diseases, but the rapid HCV mutations of the virus highlight the sustaining necessity to develop new drugs. p7, the viroporin protein from HCV, has been sought after as a potential anti-HCV drug target. Several classes of compounds, such as amantadine and rimantadine have been testified for p7 inhibition. However, the efficacies of these compounds are not high. Here, we screened some novel p7 inhibitors with amantadine scaffold for the inhibitor development. The dissociation constant... (truncated)

Title: A randomized, crossover study to evaluate the pharmacokinetics of amantadine and oseltamivir administered alone and in combination.
The threat of potential pandemic influenza requires a reevaluation of licensed therapies for the prophylaxis or treatment of avian H5N1 infection that may adapt to man. Among the therapies considered for use in pandemic influenza is the co-administration of ion channel and neuraminidase inhibitors, both to potentially increase efficacy as well as to decrease the emergence of resistant isolates. To better understand the potential for drug interactions, a cross-over, randomized, open-label trial was conducted with amantadine, 100 mg po bid, and oseltamivir, 75 mg po bid, given alone or in combination for 5 days. Each subject (N = 17) served... (truncated)

Title: Effect of ribavirin and amantadine on early hepatitis C virus RNA rebound and clearance in serum during daily high-dose interferon.
The early rebound in serum HCV RNA during HCV dynamic studies with high-dose interferon may be due to de novo infection with interferon escape quasispecies. We simultaneously measured serum alanine aminotransferase (ALT) and HCV RNA at rapid intervals in chronic HCV liver disease patients during interferon therapy alone or in combination with ribavirin and amantadine. HCV RNA declined rapidly between 0 and 48 hr in all patients (phase 1). Ribavirin and amantadine significantly increased this phase 1 decline. In all four monotherapy patients with viral rebound, the increasing levels of HCV RNA were associated with a parallel increase in serum... (truncated)

Title: Triple-combination antiviral drug for pandemic H1N1 influenza virus infection in critically ill patients on mechanical ventilation.
A recent in vitro study showed that the three compounds of antiviral drugs with different mechanisms of action (amantadine, ribavirin, and oseltamivir) could result in synergistic antiviral activity against influenza virus. However, no clinical studies have evaluated the efficacy and safety of combination antiviral therapy in patients with severe influenza illness. A total of 245 adult patients who were critically ill with confirmed pandemic influenza A/H1N1 2009 (pH1N1) virus infection and were admitted to one of the intensive care units of 28 hospitals in Korea were reviewed. Patients who required ventilator support and received either triple-combination antiviral drug (TCAD) therapy... (truncated)

MESH:D014293 - trimethadione

Summary:

LLM Prediction Score	0.484 (normalized)
LLM Confidence Score	0.900
Golden Answer (Severity Class)	0.625 (normalized)
Prediction Error	0.141

Retrieved Context:

Title: Trimethadione as a probe drug to estimate hepatic oxidizing capacity in humans.
Trimethadione (TMO) has the properties required of probe drugs for the evaluation of hepatic drug-oxidizing capacity in humans in vivo. TMO is demethylated to dimethadione (DMO), its only metabolite, in the liver after oral administration. Involvement of two cytochrome P450's--CYP2C9 and 3A4--in TMO metabolism has been seen in humans, but involvement of 1A2 is not clearly established. In humans with various types of liver disease and hepatectomy, the serum DMO/TMO ratios, which were measured on blood samples obtained by a single collection 4 hr after oral administration of TMO, correlated well with the degree of hepatic damage. This finding suggests... (truncated)

Title: Anticonvulsant drugs. An update.
A considerable amount of information is now available concerning the clinical pharmacology of the anticonvulsant drugs. Some of the more important data are reviewed in this article. In recent years, valproic acid (or sodium valproate) has found a place as a major anticonvulsant agent, while older drugs such as troxidone and sulthiame seem to be disappearing from use. Although much information is available, the essential mechanisms of action of the anticonvulsant drugs are still not understood, either at a molecular or at an electrophysiological level. The pharmacokinetics of the anticonvulsants in common use are now reasonably well documented, though some... (truncated)

Title: Safety of anticonvulsants in hepatic porphyrias.
Because acute attacks of porphyria may be precipitated by anticonvulsants, a therapeutic dilemma arises when seizures complicate hepatic porphyria. The list of unsafe agents includes barbiturates, primidone, phenytoin, mephenytoin, ethotoin, ethosuximide, methsuximide, phensuximide, and trimethadione. Agents are considered unsafe if they induce experimental porphyria in animals, and short trials in patients are unreliable for screening. Using drug incubation in chick-embryo hepatocyte culture, we found that porphyrin was increased by carbamazepine, clonazepam, and valproate. These agents should probably be avoided or used with caution in porphyric patients. Alternative approaches to acute porphyric attacks with seizures are discussed.

Title: Trimethadione tolerance test for one-point estimation of the severity of liver damage in cirrhotic patients.
We evaluated the adequacy of the trimethadione (TMO) tolerance test (the method of estimation from the serum dimethadione [DMO]/TMO ratio, DMO is only one metabolite of TMO, at 4 hours after oral administration of TMO) for estimating the severity of liver damage in 40 cirrhotic patients with and without hepatic encephalopathy. Serum dimethadione (DMO)/TMO ratios in a single blood sample after oral administration of TMO were significantly lower in cirrhotic patients with (0.07 +/- 0.02, p less than 0.05) or without (0.29 +/- 0.12, p less than 0.05) hepatic encephalopathy than in normal subjects (0.63 +/- 0.04). Serum DMO/TMO rations... (truncated)

Title: We are all aging, and here's why.
Here, through this review, we aim to serve this purpose by first discussing the statistics and aging demographics, including the life expectancy of the world and India, along with the gender life expectancy gap observed throughout the world, followed by explaining the hallmarks and integral causes of aging, along with the role played by senescent cells in controlling inflammation and the effect of senescence associated secretory phenotype on longevity. A few of the molecular pathways which are crucial in modulating the process of aging, such as the nutrient-sensing mTOR pathway, insulin signaling, Nrf2, FOXO, PI3-Akt, Sirtuins, and AMPK, and their... (truncated)

MESH:D014282 - trihexyphenidyl

Summary:

LLM Prediction Score	0.139 (normalized)
LLM Confidence Score	0.980
Golden Answer (Severity Class)	0.0 (normalized)
Prediction Error	0.139

Retrieved Context:

Title: Dyskinesia from manganism in a hepatic dysfunction patient.
A 14-year-old boy came to the neurological clinic because of involuntary movement. He represented a case of common variable hypogammaglobulinemia, with hepatosplenomegaly noted for 5 years and jaundice for 1 month. Neurological and laboratory examinations revealed choreoathetosis and hyperbilirubinemia, hypoalbuminemia, increased hepatic aminotransferase, and decreased indocyanine green clearance; as well as increased signal change over the globus pallidus, subthalamic area, internal capsule, tegmentum, brain stem and pituitary gland revealed by a brain magnetic resonance (T1-weighted) imaging study. A manganese study confirmed high body manganese loading. Trihexyphenidyl administration ameliorated the dyskinesia; however, the patient died from hepatic failure later. Though rare... (truncated)

Title: Computational Models Using Multiple Machine Learning Algorithms for Predicting Drug Hepatotoxicity with the DILIrank Dataset.
Drug-induced liver injury (DILI) remains one of the challenges in the safety profile of both authorized and candidate drugs, and predicting hepatotoxicity from the chemical structure of a substance remains a task worth pursuing. Such an approach is coherent with the current tendency for replacing non-clinical tests with in vitro or in silico alternatives. In 2016, a group of researchers from the FDA published an improved annotated list of drugs with respect to their DILI risk, constituting "the largest reference drug list ranked by the risk for developing drug-induced liver injury in humans" (DILIrank). This paper is one of the... (truncated)

Title: Management Perspective of Wilson's Disease: Early Diagnosis and Individualized Therapy.
Wilson's disease (WD) is an inherited disease caused by mutations in ATP7B and is characterized by the pathological accumulation of copper in the liver and brain. Common clinical manifestations of WD include a wide range of liver disease and neurological symptoms. In some patients, psychiatric symptoms may be the only manifestation at the time of diagnosis. The clinical features of WD are highly variable and can mimic any disease of internal medicine. Therefore, for unexplained medical diseases, the possibility of WD should not be ignored. Early diagnosis and treatment can improve the prognosis of WD patients and reduce disability and... (truncated)

Title: Wilson's disease with depression and parkinsonism.
Wilson's disease (WD) is an autosomal recessive disorder with reduced biliary excretion of copper plus impaired formation of ceruloplasmin, leading to copper accumulation in the liver, brain, kidney, and cornea. Clinical manifestations include liver damage, psychiatric symptoms, and neurological features. We report a 35-year-old woman with a history of deranged liver functions who had severe depression several years later and eventually presented with parkinsonian features. The underlying diagnosis is WD and family screening revealed WD in 2 other siblings. She could not tolerate penicillamine because of fever and leucopenia. While taking trientine hydrochloride and zinc sulphate, her parkinsonism improved and... (truncated)

Title: An in vitro coculture system of human peripheral blood mononuclear cells with hepatocellular carcinoma-derived cells for predicting drug-induced liver injury.
Preventing clinical drug-induced liver injury (DILI) remains a major challenge, because DILI develops via multifactorial mechanisms. Immune and inflammatory reactions are considered important mechanisms of DILI; however, biomarkers from in vitro systems using immune cells have not been comprehensively studied. The aims of this study were (1) to identify promising biomarker genes for predicting DILI in an in vitro coculture model of peripheral blood mononuclear cells (PBMCs) with a human liver cell line, and (2) to evaluate these genes as predictors of DILI using a panel of drugs with different clinical DILI risk. Transcriptome-wide analysis of PBMCs cocultured with HepG2... (truncated)

MESH:D011441 - propylthiouracil

Summary:

LLM Prediction Score	0.862 (normalized)
LLM Confidence Score	0.980
Golden Answer (Severity Class)	1.0 (normalized)
Prediction Error	0.138

Retrieved Context:

Title: Propylthiouracil hepatotoxicity. A review and case presentation.
Propylthiouracil (PTU) is widely used to treat patients with hyperthyroidism. In rare cases, this drug has been found to have severe toxic effects on the liver. The case of a 14-year-old girl treated with PTU for hyperthyroidism who developed jaundice, severe hepatocellular dysfunction, and hepatomegaly is reported. Her condition gradually deteriorated, and she developed paranoid ideation, profound lethargy, and peripheral edema. After three weeks of prednisone therapy, clinical and laboratory signs of improvement were observed. This patient was one of only five pediatric cases among the 16 reported cases of PTU liver toxicity reported to date. Her history and the... (truncated)

Title: Propylthiouracil-related Toxic Hepatitis: Impact of Silent Cases.
Liver is the main organ which can metabolize many drugs or chemical agents. Toxic events developed by drugs are one of the most common causes of liver damage. Toxic hepatitis can be encountered in different clinical situations, such as acute hepatitis, fulminant hepatitis chronic hepatitis or cirrhosis. We aimed to report a case of asymptomatic toxic hepatitis in a patient taking propylthiouracil (PTU). A 38 years old female patient admitted to hospital complained of fatigue. She had no special medical history except Graves' disease. She had been taking PTU 300 mg/day for 1 month. She had no history of another... (truncated)

Title: Propylthiouracil and hepatitis. Two cases and a review of the literature.
Propylthiouracil-induced hepatitis is an uncommon entity. Two further cases are reported herein, and the clinical and laboratory features of the other six cases in the English literature are reviewed. The initial appearance of the disease is similar to that of viral hepatitis, characterized by nausea, vomiting, and jaundice. The biochemical pattern of injury is predominantly hepatocellular, with marked elevation of transaminase valves and less striking elevation of alkaline phosphatase values. Recovery is usually complete after withdrawal of the drug, but there have been at least two fatalities, including the first patient (to our knowledge) whose case is reported herein. Despite... (truncated)

Title: Propylthiouracil-induced cholestatic jaundice.
The typical propylthiouracil (PTU)-linked hepatotoxicity, is known to manifest itself by hepatocellular injury with greatly increased serum transaminase values and evidence of hepatic necrosis on liver biopsy. Herewith presented is a 33-year old, thyrotoxic woman who developed cholestatic jaundice two weeks after initiation of PTU therapy. The diagnosis was confirmed by liver biopsy. A causal link between PTU treatment and the cholestatic jaundice was suggested by: the time of onset, typical skin rash and a positive migration inhibition factor (MIF) test to PTU. Awareness of this rare complication of PTU treatment may prevent the performance of unnecessary, expensive and possibly... (truncated)

Title: Propylthiouracil-induced severe hepatitis: a case report and review of the literature.
A 21-year-old woman was diagnosed as having Graves' disease in April, 1995. Thiamazole was administered; about a month later the patient had a skin rash and propylthiouracil (PTU) was given instead. Two months after commencing PTU, she rapidly developed jaundice, accompanied by severe liver damage. The drug-induced lymphocyte stimulating test was positive for PTU and she was diagnosed as having severe hepatitis induced by PTU. After pulse therapy with 500 mg of methylprednisolone was given for 3 days, liver function test results were gradually improved, and became normalized 1 1/2 months after admission. The pathology findings of the liver biopsy... (truncated)

MESH:C095108 - amprenavir

Summary:

LLM Prediction Score	0.513 (normalized)
LLM Confidence Score	0.940
Golden Answer (Severity Class)	0.375 (normalized)
Prediction Error	0.138

Retrieved Context:

Title: Liver function parameters in HIV/HCV co-infected patients treated with amprenavir and ritonavir and correlation with plasma levels.
Acute liver toxicity is a frequent adverse event that occurs during antiretroviral therapy and was observed in 6-30% of the patients on treatment, especially in presence of HCV coinfection (Cooper et al., 2002, Maida et al., 2006, Sulkowski et al., 2000). A correlation between HCV-associated liver-fibrosis severity and the risk of HAART associated hepatoxicity has been demonstrated (Aranzabal et al., 2005, Sulkowski et al., 2004). This high liver toxicity rate might be due to increased drug exposure in patients with liver disease (Veronese et al., 2000). It has been reported that patients with chronic hepatitis C show significantly reduced CPY3A4... (truncated)

Title: Relevance of Liver Failure for Anti-Infective Agents: From Pharmacokinetic Alterations to Dosage Adjustments.
The liver is a complex organ with great ability to influence drug pharmacokinetics. Due to its wide array of function, its impairment has the potential to affect bioavailability, enterohepatic circulation, drug distribution, metabolism, clearance, and biliary elimination. These alterations differ widely depending on the cause of the liver failure, if it is acute or chronic in nature, the extent of impairment, and comorbid conditions. In addition, effects on liver functions do not occur in a proportional or predictable manner for escalating degrees of liver impairment. The ability of hepatic alterations to influence PK is also dependent on drug characteristics, such... (truncated)

Title: Amprenavir and ritonavir plasma concentrations in HIV-infected patients treated with fosamprenavir/ritonavir with various degrees of liver impairment.
The purpose of this study was to evaluate the steady-state pharmacokinetics of amprenavir and ritonavir in HIV-infected patients with different degrees of hepatic impairment.

Title: Interactions of Antiretroviral Drugs with Food, Beverages, Dietary Supplements, and Alcohol: A Systematic Review and Meta-analyses.
Multiple factors may affect combined antiretroviral therapy (cART). We investigated the impact of food, beverages, dietary supplements, and alcohol on the pharmacokinetic and pharmacodynamic parameters of 33 antiretroviral drugs. Systematic review in adherence to PRISMA guidelines was performed, with 109 reports of 120 studies included. For each drug, meta-analyses or qualitative analyses were conducted. We have found clinically significant interactions with food for more than half of antiretroviral agents. The following drugs should be taken with or immediately after the meal: tenofovir disoproxil, etravirine, rilpivirine, dolutegravir, elvitegravir, atazanavir, darunavir, lopinavir, nelfinavir, ritonavir, saquinavir. Didanosine, zalcitabine, zidovudine, efavirenz, amprenavir, fosamprenavir, and... (truncated)

Title: Molecular Factors and Pathways of Hepatotoxicity Associated with HIV/SARS-CoV-2 Protease Inhibitors.
Antiviral protease inhibitors are peptidomimetic molecules that block the active catalytic center of viral proteases and, thereby, prevent the cleavage of viral polyprotein precursors into maturation. They continue to be a key class of antiviral drugs that can be used either as boosters for other classes of antivirals or as major components of current regimens in therapies for the treatment of infections with human immunodeficiency virus (HIV) and severe acute respiratory syndrome coronavirus 2 (SARS-CoV-2). However, sustained/lifelong treatment with the drugs or drugs combined with other substance(s) often leads to severe hepatic side effects such as lipid abnormalities, insulin resistance,... (truncated)

MESH:D001262 - atenolol

Summary:

LLM Prediction Score	0.362 (normalized)
LLM Confidence Score	0.980
Golden Answer (Severity Class)	0.5 (normalized)
Prediction Error	0.138

Retrieved Context:

Title: Synthesis, Molecular Docking, and Preclinical Evaluation of a New Succinimide Derivative for Cardioprotective, Hepatoprotective and Lipid-Lowering Effects.
Cardiac and hepatotoxicities are major concerns in the development of new drugs. Better alternatives to other treatments are being sought to protect these vital organs from the toxicities of these pharmaceuticals. In this regard, a preclinical study is designed to investigate the histopathological effects of a new succinimide derivative (Comp-1) on myocardial and liver tissues, and the biochemical effects on selected cardiac biomarkers, hepatic enzymes, and lipid profiles. For this, an initially lethal/toxic dose was determined, followed by a grouping of selected albino rats into five groups (each group had n = 6). The control group received daily oral saline... (truncated)

Title: Atenolol hepatotoxicity: report of a complicated case.
To report a case of acute hepatitis related to atenolol administration in a liver transplant (LT) recipient.

Title: Clinical experience with atenolol in patients with chronic liver disease.
The pharmacokinetics of atenolol were investigated following single intravenous (25 mg) and oral administration (100 mg) of atenolol in 13 patients with chronic liver disease and normal renal function and in 12 normal healthy volunteers. Four of the patients with chronic liver disease were not included in the statistical evaluation of kinetic data, since a reduction of creatinine clearance was observed during the course the study after ingestion of atenolol. A tendency to an increased distribution volume of atenolol could be observed in subjects with liver disease compared to normal volunteers. After intravenous and oral administration of atenolol, pharmacokinetic parameters... (truncated)

Title: A risk-benefit assessment of losartan potassium in the treatment of hypertension.
Losartan potassium is the first of a new class of orally active antihypertensive drugs which antagonise the action of angiotensin (AT) II at the AT1 receptor subtype. Losartan potassium is converted by the liver to the active metabolite E-3174, which is a more potent antagonist at the AT1 receptor. E-3174 is responsible for most of the pharmacological effects of losartan potassium, and its long half-life contributes to the extended duration of action of the drug. Losartan potassium is effective as a once-daily antihypertensive agent. In mild to moderate hypertension, losartan potassium has similar efficacy to enalapril, atenolol and felodipine extended... (truncated)

Title: In vitro platforms for evaluating liver toxicity.
The liver is a heterogeneous organ with many vital functions, including metabolism of pharmaceutical drugs and is highly susceptible to injury from these substances. The etiology of drug-induced liver disease is still debated although generally regarded as a continuum between an activated immune response and hepatocyte metabolic dysfunction, most often resulting from an intermediate reactive metabolite. This debate stems from the fact that current animal and in vitro models provide limited physiologically relevant information, and their shortcomings have resulted in "silent" hepatotoxic drugs being introduced into clinical trials, garnering huge financial losses for drug companies through withdrawals and late stage... (truncated)

MESH:D000068180 - aripiprazole

Summary:

LLM Prediction Score	0.487 (normalized)
LLM Confidence Score	0.990
Golden Answer (Severity Class)	0.625 (normalized)
Prediction Error	0.138

Retrieved Context:

Title: Aripiprazole-induced liver injury: a spontaneous reporting database study.
Background: There have been individual case reports of aripiprazole in recent years, both domestically and internationally, but no analysis of the characteristics of the occurrence of adverse reactions/events of drug-induced liver injury with aripiprazole using spontaneous reports has been seen. Methods: Using a retrospective study approach, the 452 adverse reaction/event reports of aripiprazole-induced liver injury collected by the China Adverse Drug Reaction Monitoring System from 1 January 2012 to 31 December 2016 were analyzed and evaluated, and exploring it's the clinical characteristics and related risk factors for liver injury occurrence. Results: Among 452 cases of aripiprazole-induced liver injury ADR/ADE reports,... (truncated)

Title: Suspected Aripiprazole-induced neutropenia in a geriatric patient: a case report.
Aripiprazole, a third-generation antipsychotic medication, has been used to treat a range of psychiatric disorders. According to the U.S. Food and Drug Administration's prescribing information, the most common adverse reactions in adult patients in clinical trials (≥10%) were nausea, vomiting, constipation, headache, dizziness, akathisia, anxiety, and insomnia. While hematological adverse effects may occur with aripiprazole, there is very limited information in the published literature on such adverse outcomes.

Title: Aripiprazole-induced Hepatitis: A Case Report.
Aripiprazole is an atypical antipsychotic that acts as a partial agonist of dopamine type 2 receptors as well as 5-HT1A receptors. It is used in the treatment of schizophrenia and in type 1 bipolar disorder for mania. Because aripiprazole is well tolerated with few side effects it is used off-label in other psychotic disorders. The prevalence of abnormal liver function tests with antipsychotic use is 32%, with clinically significant effects in 4% of cases. No cases of aripiprazole-induced liver injury have been published. We report a 28-year-old female who presented with non-affective first-episode psychosis and who was treated with aripiprazole.... (truncated)

Title: Aripiprazole reduces liver cell division.
Effects of aripiprazole on dopamine regulation are being tested as a treatment for patients with a dual diagnosis of schizophrenia and addictions, often cocaine dependence. Aripiprazole has one of the fewest side-effects among the second-generation antipsychotics. Nevertheless, severe aripiprazole hepatotoxicity was reported in persons with a history of cocaine and alcohol abuse. Here we report that therapeutically relevant aripiprazole concentrations, equal to laboratory alert levels in patients' serum, reduce the rate of hepatocytes' division. This could be an underlying mechanism of severe liver injury development in the patients with a history of alcohol and cocaine abuse, the two hepatotoxic agents... (truncated)

Title: Psychotropic drug-related eosinophilia with systemic symptoms after acute caffeine ingestion.
Drug-related eosinophilia with systemic symptoms (DRESS) is a potentially life-threatening, multiorgan condition that can result from drug treatment. Antiepileptic medications have provided the best-studied link of any class of medications. Here, we report the case of a 16-year-old boy with long-standing bipolar disorder who was chronically treated with aripiprazole and fluoxetine and developed DRESS syndrome after ingestion of high doses of caffeine. His classic presentation with fever, morbilliform rash, lymphadenopathy, and visceral involvement, including leukocytosis, eosinophilia, and hepatitis, was consistent with this diagnosis. Furthermore, the patient's symptoms dramatically improved after corticosteroid therapy and discontinuation of all psychotropic medications. We propose... (truncated)

MESH:D010068 - oxacillin

Summary:

LLM Prediction Score	0.512 (normalized)
LLM Confidence Score	0.980
Golden Answer (Severity Class)	0.375 (normalized)
Prediction Error	0.137

Retrieved Context:

Title: Severe hepatitis associated with oxacillin therapy.
A 6-year-old girl had fever, abdominal pain, and severe anicteric hepatitis during intravenous oxacillin therapy for staphylococcal osteomyelitis. She had greatly elevated liver enzymes, prolonged prothrombin time, leukopenia, and eosinophilia. Clinical symptoms resolved and laboratory data returned to normal after withdrawing oxacillin and substituting cefazolin. This hepatotoxicity appears to be specific to oxacillin and not to other beta-lactams. Monitoring liver function tests during oxacillin therapy, especially in patients receiving prolonged treatment, may be warranted.

Title: Oxacillin hepatitis. Two patients with liver biopsy, and review of the literature.
Hepatotoxicity associated with intravenous sodium oxacillin therapy is reported in two drug abusers cured of staphylococcal endocarditis. Coincident with the administration of oxacillin, marked increases in hepatic transaminase were observed and liver biopsy showed nonspecific hepatitis. Upon cessation of oxacillin therapy, liver enzyme values returned to(ward) normal. Reports of oxacillin-associated changes in hepatic enzyme levels are reviewed; further observation of oxacillin-associated hepatotoxicity is warranted.

Title: Reversible oxacillin hepatotoxicity.
Eight patients developed elevations of hepatic enzymes while receiving oxacillin intravenously. In all instances the patients were asymptomatic and anicteric. Peripheral eosinophilia was present in five of eight patients. In each patient change of therapy to a different pencillinase-resistant penicillin or to penicillin G was associated with a rapid decrease in liver function abnormalities and evenutal return of the enzymatic values to normal. Change of medication to an alternative penicillinase-resistant penicillin or to penicillin G is suggested as a safe procedure for completion of antistaphylococcal therapy in patient who develop oxacillin-related hepatotoxicity.

Title: Hepatic safety of antibiotics used in primary care.
Antibiotics used by general practitioners frequently appear in adverse-event reports of drug-induced hepatotoxicity. Most cases are idiosyncratic (the adverse reaction cannot be predicted from the drug's pharmacological profile or from pre-clinical toxicology tests) and occur via an immunological reaction or in response to the presence of hepatotoxic metabolites. With the exception of trovafloxacin and telithromycin (now severely restricted), hepatotoxicity crude incidence remains globally low but variable. Thus, amoxicillin/clavulanate and co-trimoxazole, as well as flucloxacillin, cause hepatotoxic reactions at rates that make them visible in general practice (cases are often isolated, may have a delayed onset, sometimes appear only after cessation... (truncated)

Title: Hepatotoxicity of antibiotics.
Several antibiotics can cause severe hepatic injury. It is the purpose of this paper to review the main antibiotics that can cause hepatic injury and discuss the presentation, pattern, and outcome of hepatic injury. In the case of the penicillins, the combination amoxycillin-clavulanate and the penicillinase-resistant penicillins oxacillin, (di-)cloxacillin, and flucloxacillin can cause (mainly cholestatic) hepatitis. Cephalosporins have little hepatotoxicity; ceftriaxone can cause drug-induced gallstones. The potential of erythromycin and several other macrolides to cause (usually cholestatic) hepatitis is well established. Tetracyclines can cause a syndrome mimicking acute fatty liver of pregnancy, but this complication has virtually disappeared. Quinolones seem... (truncated)

MESH:D002752 - chlorthalidone

Summary:

LLM Prediction Score	0.114 (normalized)
LLM Confidence Score	0.970
Golden Answer (Severity Class)	0.25 (normalized)
Prediction Error	0.136

Retrieved Context:

Title: A randomized titrate-to-target study comparing fixed-dose combinations of azilsartan medoxomil and chlorthalidone with olmesartan and hydrochlorothiazide in stage-2 systolic hypertension.
Azilsartan medoxomil (AZL-M), an angiotensin II receptor blocker, has been developed in fixed-dose combinations (FDCs) with chlorthalidone (CTD).

Title: Comparison of efficacy and safety between third-dose triple and third-dose dual antihypertensive combination therapies in patients with hypertension.
We compared the efficacy and safety of third-standard-dose triple and third-standard-dose dual antihypertensive combination therapies in patients with mild to moderate hypertension. This was a phase II multicenter, randomized, double-blind, parallel-group trial. After a 4-week placebo run-in period, 245 participants were randomized to the third-dose triple combination (ALC group; amlodipine 1.67 mg + losartan potassium 16.67 mg + chlorthalidone 4.17 mg) or third-dose dual combination (AL group; amlodipine 1.67 mg + losartan potassium 16.67 mg, LC group; losartan potassium 16.67 mg + chlorthalidone 4.17 mg, AC group; amlodipine 1.67 mg + chlorthalidone 4.17 mg) therapy groups and followed up for... (truncated)

Title: Medications for alcohol use disorders: An overview.
Patients who suffer from alcohol use disorders (AUDs) usually go through various socio-behavioral and pathophysiological changes that take place in the brain and other organs. Recently, consumption of unhealthy food and excess alcohol along with a sedentary lifestyle has become a norm in both developed and developing countries. Despite the beneficial effects of moderate alcohol consumption, chronic and/or excessive alcohol intake is reported to negatively affect the brain, liver and other organs, resulting in cell death, organ damage/failure and death. The most effective therapy for alcoholism and alcohol related comorbidities is alcohol abstinence, however, chronic alcoholic patients cannot stop drinking... (truncated)

Title: Prolonged pyrexia and hepatitis: Q fever.
No abstract available.

Title: Amyloidosis: an unusual cause of portal hypertension.
Amyloidosis comprises a group of diseases that occurs in five to nine cases per million patients per year worldwide irrespective of its classification. Although the hepatic involvement in primary amyloidosis is frequent, the clinical manifestations of liver amyloidosis are mild or even absent. The authors report the case of an aged man who complained of diffuse abdominal pain and marked weight loss and presented clinical signs of hepatopathy. Clinical workup revealed portal hypertension with ascites, hemorrhoids, and esophageal varices. The laboratory tests showed the cholestatic pattern of liver enzymes, hyperbilirubinemia, renal insufficiency and massive proteinuria accompanied by the presence of... (truncated)

MESH:D008879 - midodrine

Summary:

LLM Prediction Score	0.136 (normalized)
LLM Confidence Score	0.980
Golden Answer (Severity Class)	0.0 (normalized)
Prediction Error	0.136

Retrieved Context:

Title: Norepinephrine is More Effective Than Midodrine/Octreotide in Patients With Hepatorenal Syndrome-Acute Kidney Injury: A Randomized Controlled Trial.
Background: Terlipressin is the first-line pharmacological treatment for hepatorenal syndrome. When terlipressin is unavailable, midodrine/octreotide or norepinephrine, with albumin, represent the alternative treatments. The comparative efficacy of these alternative regimens remains unclear. Objective: To compare the efficacy of midodrine/octreotide to that of norepinephrine for the treatment of patients with hepatorenal syndrome. Methods: In the intensive care setting, sixty patients with hepatorenal syndrome were randomized to initially receive either 0.5 mg/h of norepinephrine (maximum 3 mg/h) or 5 mg of oral midodrine three times/day (maximum 12.5 mg three times/day) plus octreotide (100 μg/6 h) as subcutaneous injection (maximum 200 μg/6 h), together with albumin (20-40 g/day). Treatment was allowed for... (truncated)

Title: A Comprehensive Systematic Review of the Latest Management Strategies for Hepatorenal Syndrome: A Complicated Syndrome to Tackle.
Hepatorenal syndrome (HRS), defined by the extreme manifestation of renal impairment in patients with cirrhosis, is characterized by reduced renal blood flow and glomerular filtration rate. It is diagnosed with reduced kidney function confirming the absence of intrinsic kidney disease, such as hematuria or proteinuria. HRS is potentially reversible with liver transplantation or vasoconstrictor drugs. The condition carries a poor prognosis with high mortality rates, particularly in patients with advanced cirrhosis. The latest management for HRS involves a combination of pharmacological and non-pharmacological interventions, aiming to improve renal function and reduce the risk of mortality. Pharmacological treatments include vasoconstrictors, such... (truncated)

Title: Hepatorenal Syndrome.
Hepatorenal syndrome (HRS) is the most serious hepatorenal disorder and one of the most difficult to treat. To date, the best treatment options are those that reverse the mechanisms underlying HRS: portal hypertension, splanchnic vasodilation, and/or renal vasoconstriction. Therefore, liver transplantation is the preferred definitive treatment option. The role of other therapies is predominantly to prolong survival sufficiently to allow patients to undergo transplantation. Terlipressin with the addition of adjunctive albumin volume expansion is the preferred pharmacologic therapy for the treatment of patients with HRS. Norepinephrine and vasopressin are acceptable alternatives in countries where terlipressin is not yet available. For... (truncated)

Title: Case Report of Isoniazid-Related Acute Liver Failure Requiring Liver Transplantation.
The prevalence of latent tuberculosis infection (LTBI) in the United States in 2011 and 2012 was estimated at 4.4¯4.8%. As of 2015, 12.4 million people still possessed LTBI. Isoniazid, or isonicotinic acid hydrazine (INH), is the most commonly used medication among varying regimens that exist in the treatment of tuberculosis and LTBI. INH-related hepatotoxicity is a well-known adverse effect of its use, often causing asymptomatic elevations in serum aminotransferase levels. These elevations are typically transient and reversible, but can cause acute, clinically-significant liver injury in rare cases. We report a case of a 67-year old male who developed subacute hepatic... (truncated)

Title: Current and future pharmacological therapies for managing cirrhosis and its complications.
Due to the restrictions of liver transplantation, complication-guided pharmacological therapy has become the mainstay of long-term management of cirrhosis. This article aims to provide a complete overview of pharmacotherapy options that may be commenced in the outpatient setting which are available for managing cirrhosis and its complications, together with discussion of current controversies and potential future directions. PubMed/Medline/Cochrane Library were electronically searched up to December 2018 to identify studies evaluating safety, efficacy and therapeutic mechanisms of pharmacological agents in cirrhotic adults and animal models of cirrhosis. Non-selective beta-blockers effectively reduce variceal re-bleeding risk in cirrhotic patients with moderate/large varices, but... (truncated)

MESH:D000658 - amoxicillin

Summary:

LLM Prediction Score	0.489 (normalized)
LLM Confidence Score	0.990
Golden Answer (Severity Class)	0.625 (normalized)
Prediction Error	0.136

Retrieved Context:

Title: Amoxicillin-Clavulanate Induced Liver Injury in a Young Female.
Amoxicillin-clavulanate (AC) is an antibiotic widely used for various infections. It has rarely been associated with drug-induced liver injury (DILI), mainly in males 55 or older with associated alcohol consumption or medications causing liver injury. Here we present an atypical case of a 22-year-old female with a past medical history of celiac disease and alopecia areata who was prescribed AC in urgent care for bilateral cervical lymphadenopathy, nausea, and chills. Her nausea and vomiting worsened after taking AC for three days, and she developed jaundice. On workup, she was found to have deranged liver functions, and pan-lobular hepatitis was confirmed... (truncated)

Title: Does Genetics Play a Role in Acute Liver Injury After Amoxicillin Exposure?
Amoxicillin-clavulanate has long been associated with drug-induced liver injury (DILI) and although approximately 4 times less common, amoxicillin has also been implicated. Many studies have associated possible genetic factors with susceptibility to DILI, but there is currently no literature with evidence of instances of DILI within the same family. Two sisters presented with similar symptoms and signs of liver injury including jaundice, scleral icterus, abdominal pain, and anorexia with transaminitis and abnormal coagulation studies. Both sisters were started on amoxicillin approximately 2-3 weeks before presentation. They both had progression of the liver injury, and on biopsies, they had similar findings... (truncated)

Title: Hepatitis associated with amoxycillin-clavulanic acid combination report of 15 cases.
Fifteen cases of hepatitis related to a combination of amoxycillin and clavulanic acid are reported. Most patients were aged 60 years or more and there were more men than women (sex ratio 4:1). The amoxycillin-clavulanic acid had been given at doses ranging from 0.5 to 6 g/day (mean 2 g/day) for seven to 60 days (mean 18 days). In 11 cases, the first symptoms appeared one to four weeks after stopping treatment. Jaundice was observed in all patients and was frequently associated with pruritus. Serum aminotransferase activities were increased in all patients and were generally two to 10 times the... (truncated)

Title: An Uncommon Side Effect of a Commonly Used Antibiotic: Amoxicillin-Clavulanic Acid Induced Hepatitis.
Amoxicillin-Clavulanic acid continues to be one of the most commonly used antibiotic combinations. Hepatic injury due to this antibiotic is rare. We report a case of amoxicillin-clavulanic acid induced hepatitis causing painless jaundice to bring to attention this rare side effect of this commonly used antibiotic. This is a case of a 62-year-old Caucasian female, who presented with acute onset severe painless jaundice, nausea, vomiting, and pruritus of less than 1-week duration. She had completed a course of amoxicillin-clavulanic acid 3 weeks prior to presentation. A careful history pointed to this simple diagnosis. It may be easily missed without an... (truncated)

Title: Risk of acute liver injury associated with the combination of amoxicillin and clavulanic acid.
Amoxicillin-clavulanic acid combination-associated hepatitis and jaundice was first identified in 1988. Numerous case reports and case series have been published since then, but there is no precise estimate of this risk.

MESH:D017245 - foscarnet

Summary:

LLM Prediction Score	0.240 (normalized)
LLM Confidence Score	0.980
Golden Answer (Severity Class)	0.375 (normalized)
Prediction Error	0.135

Retrieved Context:

Title: Foscarnet for treatment of cytomegalovirus infections in bone marrow transplant recipients.
42 episodes of verified or clinically suspected cytomegalovirus (CMV) infection in 40 bone marrow transplant (BMT) recipients were treated with foscarnet (trisodium phosphonophormate hexahydrate). CMV infection was verified in 31/42 treatment episodes. Symptoms treated were pneumonia (n = 17), pancytopenia with or without fever (n = 12), enteritis (n = 5), fever (n = 4), encephalitis (n = 2), retinitis (n = 1) and hepatitis (n = 1). Foscarnet was given as a continuous intravenous infusion. Side-effects observed were increase in serum creatinine (38%), decrease in serum calcium (19%), increase in serum bilirubin (12%), decrease in hemoglobin concentration (7%), increase... (truncated)

Title: Brincidofovir Use after Foscarnet Crystal Nephropathy in a Kidney Transplant Recipient with Multiresistant Cytomegalovirus Infection.
<i>Background</i>. Cytomegalovirus (CMV) antiviral drug resistance constitutes an increasing challenge in transplantation. Foscarnet is usually proposed when resistance for ganciclovir is suspected, but its use is limited by its nephrotoxicity. <i>Case Presentation</i>. We report a case of multiresistant CMV disease in a kidney transplant recipient. Foscarnet was prescribed after ganciclovir treatment failure in a patient with two mutations in the UL97 viral gene. Foscarnet induced biopsy-proven kidney crystal precipitation that resulted in severe acute transplant failure and nephrotic syndrome. Despite a large decrease in immunosuppression, CMV disease was not controlled and a salvage therapy with Brincidofovir (BCV), which is an... (truncated)

Title: Cytomegalovirus infection as a common complication following liver transplantation.
The aim of our study was to assess the incidence course, influence on liver function, diagnostic methods, prophylaxis of, and cost treatment effectiveness of CMV infection among 123 consecutive liver transplant recipients. All patients received immunoglobulin and parenterall gancyclovir as prophylaxis. CMV IgM and IgG antibodies were determined using an ELISA method. Thirty seven patients (30.0%) developed CMV infection. Main indications for primary LTX were: immune liver disease (n = 22), viral hepatitis (n = 5), and other (n = 10). CMV infection occurred between the days 5 and 416. Ten patients (27.0%) developed more than one infection (52 infections... (truncated)

Title: Adverse effects of drugs used in the management of opportunistic infections associated with HIV infection.
Pneumocystis carinii pneumonia (PCP) is one of the most common AIDS-defining diagnoses. First-line therapy is cotrimoxazole (trimethoprim-sulfamethoxazole), despite a high incidence of toxic effects, and a greater incidence of hypersensitivity reactions among HIV-positive patients compared with the seronegative population. Alternative agents such as intravenous pentamidine, or clindamycin with primaquine, and trimethoprim with dapsone, also have a wide range of serious adverse effects, but remain treatment options. Atovaquone appears promising for the treatment of both PCP and toxoplasmosis, and has a lower reported incidence of toxicity than the alternative agents. The most toxic antifungal drugs are reserved for serious infections, such... (truncated)

Title: The search for new therapies for human cytomegalovirus infections.
Ganciclovir (GCV), the therapy of choice for human cytomegalovirus (CMV) infections and foscarnet, a drug used to treat GCV-resistant CMV infections was approved more than twenty years ago. Although cidofovir and a prodrug of GCV have since been added to the armamentarium, a highly effective drug without significant toxicities has yet to be approved. Such a therapeutic agent is required for treatment of immunocompromised hosts and infants, which bear the greatest burden of disease. The modest antiviral activity of existing drugs is insufficient to completely suppress viral replication, which results in the selection of drug-resistant variants that remain pathogenic, continue... (truncated)

MESH:D000069458 - ranolazine

Summary:

LLM Prediction Score	0.135 (normalized)
LLM Confidence Score	0.960
Golden Answer (Severity Class)	0.0 (normalized)
Prediction Error	0.135

Retrieved Context:

Title: Novel anti-arrhythmic medications in the treatment of atrial fibrillation.
Atrial fibrillation (AF) is a prevalent condition particularly amongst the elderly, which contributes to both morbidity and mortality. The burden of disease has lead to significant increases in health care utilization and cost in recent years. Treatment of Atrial fibrillation consists of either a rate or rhythm control strategy. Rhythm control is achieved using medical management and/or catheter ablation. In spite of major strides in catheter ablation, this procedure remains a second line treatment of AF. Anti-arrhythmic medications represent the main treatment modality for the maintenance of sinus rhythm. Amiodarone has been used for decades because of its efficacy and... (truncated)

Title: Possible Interaction between Dabigatran and Ranolazine in Patients with Renal Failure.
Dabigatran etexilate is a direct oral anticoagulant (thrombin inhibitor) used for the prevention of stroke and systemic thromboembolic events in patients with permanent atrial fibrillation; prevention of venous thromboembolic events and deep veins thrombosis; treatment and prevention of pulmonary embolism. Dabigatran is a relatively new drug, and as a result, its interactions with other medications and their significance are not fully known. A 72 years old male, having a medical history of heart and renal failure, was hospitalized for pneumonia treatment. The patient was taking several drugs, including dabigatran 150 mg twice daily and ranolazine 750 mg twice daily. His... (truncated)

Title: Modulation of myocardial energetics: An important category of agents in the multimodal treatment of coronary artery disease and heart failure.
The combined and relative contribution of glucose and fatty acid oxidation generates myocardial energy, which regulates the cardiac function and efficiency. Any dysregulation in this metabolic homeostasis can adversely affect the function of heart and contribute to cardiac conditions such as angina and heart failure. Metabolic agents ameliorate this internal metabolic anomaly, by shifting the energy production pathway from free fatty acids to glucose, resulting in a better performance of the heart. Metabolic therapy is relatively a new modality, which functions through optimization of cardiac substrate metabolism. Among the metabolic therapies, trimetazidine and ranolazine are the agents presently available in... (truncated)

Title: Efficacy of ranolazine in preventing atrial fibrillation following cardiac surgery: Results from a meta-analysis.
Atrial fibrillation (AF) is a common complication after cardiac surgery. Ranolazine is a Food and Drug Administration approved anti-ischemic drug, which also has anti-arrhythmic properties. Recent studies have demonstrated the benefit of ranolazine in preventing post-operative AF (POAF) in patients undergoing cardiac surgery. Hence, we performed a meta-analysis of published studies comparing ranolazine plus standard therapy versus standard therapy for POAF prevention in patients undergoing cardiac surgery.

Title: Molecular mechanisms of statin intolerance.
Statins reduce cardiovascular morbidity and mortality in primary and secondary prevention. Despite their efficacy, many persons are unable to tolerate statins due to adverse events such as hepatotoxicity and myalgia/myopathy. In the case of most patients, it seems that mild-to-moderate abnormalities in liver and muscle enzymes are not serious adverse effects and do not outweigh the benefits of coronary heart disease risk reduction. The risk for mortality or permanent organ damage ascribed to statin use is very small and limited to cases of myopathy and rhabdomyolysis. Statin-induced muscle-related adverse events comprise a highly heterogeneous clinical disorder with numerous, complex etiologies... (truncated)

MESH:D004025 - dicyclomine

Summary:

LLM Prediction Score	0.135 (normalized)
LLM Confidence Score	0.950
Golden Answer (Severity Class)	0.0 (normalized)
Prediction Error	0.135

Retrieved Context:

Title: A Pilot Study of Silymarin as Supplementation to Reduce Toxicities in Metastatic Colorectal Cancer Patients Treated With First-Line FOLFIRI Plus Bevacizumab.
Irinotecan, a topoisomerase inhibitor, is a common cytotoxic agent prescribed for metastatic colorectal cancer (mCRC) patients. Diarrhea is the most common adverse event (AE). The underlying mechanism of irinotecan-induced diarrhea is intestinal mucosal damage caused by SN-38 (active metabolite of irinotecan) hydrolyzed from SN-38G (inactive metabolite) by bacterial -glucuronidase (G). According to an animal study, silymarin reduces the activity of bacterial G without impairing antitumor efficacy. We conducted a prospective open-label pilot study to evaluate the effect of silymarin as supplementation in reducing toxicities of mCRC patients undergoing irinotecan-based chemotherapy. We enrolled and randomized 70 mCRC patients receiving first-line FOLFIRI... (truncated)

Title: The safety of drugs used in acid-related disorders and functional gastrointestinal disorders.
Medicines are frequently used in the management of acid-related disorders and functional gastrointestinal disorders. With the exception of complicated peptic ulcer disease, these disorders are not associated with appreciable mortality. Drug treatments have consequently been held to the highest standards of safety. Some medicines have been withdrawn or restricted based on assessments and perceptions of risk. However, the risk of serious toxicity is low for most of the agents discussed in this article. Assessments are made of the safety and adverse-event profiles of certain drug classes and, where appropriate, individual medicines. For conditions with a low risk of mortality or... (truncated)

Title: A case report and literature review of daptomycin-induced liver injury.
Daptomycin is a lipopeptide antimicrobial used to treat gram positive organisms including multi-drug resistant infections. It has been shown to occasionally cause abnormalities in liver function but more commonly is associated with elevations in serum creatinine phosphokinase (CK) (Hair and Keam, 2007) [1]. We describe a case where a patient being treated for methicillin-resistant <i>Staphylococcus aureus</i> (MRSA) bacteremia with daptomycin developed asymptomatic elevated transaminases without evidence of multiorgan failure, hyperbilirubinemia or elevation of CK levels. Other etiologies for liver injury were considered and ruled out, and after daptomycin was discontinued, the transaminases returned to normal levels. We also provide a... (truncated)

Title: Isoniazid-induced liver disorder in the treatment of tuberculosis.
No abstract available.

Title: Retrospective Study of Reported Adverse Events Due to Complementary Health Products in Singapore From 2010 to 2016.
The objective of this study is to collate and analyse adverse event reports associated with the use of complementary health products (CHP) submitted to the Health Sciences Authority (HSA) of Singapore for the period 2010-2016 to identify various trends and signals for pharmacovigilance purposes. A total of 147,215 adverse event reports suspected to be associated with pharmaceutical products and CHP were received by HSA between 2010 and 2016. Of these, 143,191 (97.3%) were associated with chemical drugs, 1,807 (1.2%) with vaccines, 1,324 (0.9%) with biological drugs (biologics), and 893 (0.6%) with CHP. The number of adverse event reports associated with... (truncated)

MESH:C047638 - alfuzosin

Summary:

LLM Prediction Score	0.491 (normalized)
LLM Confidence Score	0.940
Golden Answer (Severity Class)	0.625 (normalized)
Prediction Error	0.134

Retrieved Context:

Title: Acute hepatocellular drug induced liver injury probably by alfuzosin.
Alpha blockers are the drugs that exert their effects by binding to alpha receptors and relaxing smooth muscles and are currently used for treatment of benign prostate hyperplasia (BPH). These drugs are often tolerated well by the patients. However, they also possess some common side effects. Hepatotoxicity, on the other hand, is quite rare. We report herein a case with the rare complication of acute hepatocellular drug induced liver injury (DILI) by administration of Alfuzosin.

Title: Alfuzosin-induced acute liver injury.
We describe a 56-year-old man who developed an acute liver injury after taking alfuzosin for 1 month to control his newly diagnosed benign prostatic hypertrophy (BPH). There was no history of alcohol consumption or the taking herbal or traditional remedies. Viral causes, autoimmune hepatitis, and biliary tree obstruction were excluded. Other rare causes of hepatitis such as hemochromatosis, primary biliary cirrhosis and Wilson's disease were also absent in this patient. His liver test results began to improve after discontinuing the alfuzosin. Two weeks later, alfuzosin was administered again because the patient complained of dysuria. After 10 days of alfuzosin reuse,... (truncated)

Title: Alfuzosin-induced acute hepatitis in a patient with chronic liver disease.
To report a new case of probable alfuzosin-induced hepatitis.

Title: Detective work in drug-induced liver injury: sometimes it is all about interviewing the right witness.
Diagnosing drug-induced liver injury (DILI) relies primarily on history taking. We report 4 cases in which DILI was missed or the drug was misidentified when physicians relied solely on patient history. We reviewed 27 cases referred with possible DILI from August 1, 2009, to February 1, 2010. Four patients seemed to be reliable historians, but their cases were greatly clarified by a call to their pharmacist. One subject, who forgot a new medication, underwent an unfruitful evaluation including surgery. Another patient had acetaminophen toxicity that was missed because she grossly underreported her pain medication use. The third and fourth patients... (truncated)

Title: Manifestations of Liver Impairment and the Effects of MH-76, a Non-Quinazoline α1-Adrenoceptor Antagonist, and Prazosin on Liver Tissue in Fructose-Induced Metabolic Syndrome.
Excessive fructose consumption may lead to metabolic syndrome, metabolic dysfunction-associated fatty liver disease (MAFLD) and hypertension. α1-adrenoceptors antagonists are antihypertensive agents that exert mild beneficial effects on the metabolic profile in hypertensive patients. However, they are no longer used as a first-line therapy for hypertension based on Antihypertensive and Lipid-Lowering Treatment to Prevent Heart Attack Trial (ALLHAT) outcomes. Later studies have shown that quinazoline-based α1-adrenolytics (prazosin, doxazosin) induce apoptosis; however, this effect was independent of α1-adrenoceptor blockade and was associated with the presence of quinazoline moiety. Recent studies showed that α1-adrenoceptors antagonists may reduce mortality in COVID-19 patients due to... (truncated)

MESH:D000077205 - pioglitazone

Summary:

LLM Prediction Score	0.508 (normalized)
LLM Confidence Score	0.980
Golden Answer (Severity Class)	0.375 (normalized)
Prediction Error	0.133

Retrieved Context:

Title: Thiazolidinediones and liver toxicity.
Thiazolidinediones or glitazones specifically target insulin resistance. They have proven efficacy for reducing plasma glucose levels of type 2 diabetic patients treated with diet alone, sulphonylureas, metformin or insulin. In addition, they may be associated to some improvement of cardiovascular risk profile. However, troglitazone, the first compound approved by the FDA in the US, proved to be hepatotoxic and was withdrawn from the market after the report of several dozens of deaths or cases of severe hepatic failure requiring liver transplantation. It remains unclear whether or not hepatotoxicity is a class effect or is related to the unique tocopherol side... (truncated)

Title: Second-generation thiazolidinediones and hepatotoxicity.
To report a case of hepatotoxicity probably caused by pioglitazone, summarize case reports of hepatotoxicity induced by rosiglitazone or pioglitazone, and make recommendations regarding routine liver enzyme measurement in patients taking these agents.

Title: Mixed hepatocellular-cholestatic liver injury after pioglitazone therapy.
Pioglitazone is an oral hypoglycemic agent in the thiazolidinedione class. Only one case of hepatotoxicity related to this agent has previously been reported.

Title: Case series of liver failure associated with rosiglitazone and pioglitazone.
The thiazolidinedione drugs rosiglitazone and pioglitazone are not widely known to be hepatotoxic. We evaluated the FDA Adverse Event Reporting System (AERS) to determine the number of reported cases of liver failure associated with rosiglitazone and pioglitazone between 1997 and 2006, and described their clinical characteristics.

Title: Hepatotoxicity with thiazolidinediones: is it a class effect?
Decreased insulin sensitivity plays a major role in various human diseases. particularly type 2 diabetes mellitus, and is associated with a higher risk of atherosclerosis and cardiovascular complications. Thiazolidinediones, more commonly termed glitazones, are the first drugs to specifically target muscular insulin resistance. They have proven efficacy for reducing plasma glucose levels in patients with type 2 diabetes mellitus treated with diet alone, sulphonylureas, metformin or insulin. In addition, they are associated with some improvement of the cardiovascular risk profile. However, troglitazone, the first compound approved by the Food and Drug Administration in the US, proved to be hepatotoxic and... (truncated)

MESH:D016190 - carboplatin

Summary:

LLM Prediction Score	0.508 (normalized)
LLM Confidence Score	0.990
Golden Answer (Severity Class)	0.375 (normalized)
Prediction Error	0.133

Retrieved Context:

Title: Metastatic human epidermal growth factor 2 (HER2/neu) amplified breast cancer with acute fulminant hepatitis responding to trastuzumab, pertuzumab and carboplatin.
A 30-year-old woman presented to an outside hospital with pain in the right upper abdomen. Imaging revealed over 100 liver lesions, the largest measuring 74 mm×71 mm, and multiple lytic bone lesions. An outpatient liver biopsy showed a poorly differentiated adenocarcinoma favouring a breast primary. The tumour was oestrogen and progesterone receptor negative, but human epidermal growth factor 2 (HER2/neu) amplified. In her second clinic visit she had decompensated liver failure manifested by new-onset ascites and jaundice. Initially, the chemotherapy plan was for docetaxel, pertuzumab and trastuzumab, but given her severe liver dysfunction we used a combination of carboplatin, pertuzumab... (truncated)

Title: High-dose carboplatin, cyclophosphamide, and BCNU with autologous bone marrow support: excessive hepatic toxicity.
Intensive doses of carboplatin, cyclophosphamide, and BCNU with autologous bone marrow support were given to four patients with advanced melanoma. Three developed clinically diagnosed, severe veno-occlusive liver disease, which was fatal in two cases. The dose of carboplatin (450 mg/m2) was comparable with that used in ambulatory regimens. At the doses and schedule employed, this three-drug combination produced excessive hepatic toxicity. Caution is suggested when giving carboplatin in combination with intensive doses of other chemotherapeutic agents with known hepatotoxic potential.

Title: Phase I study of carboplatin (CBDCA) in children with cancer.
A phase I study of carboplatin (CBDCA) was performed in 40 children with advanced cancer. A single course of CBDCA consisted of 4 weekly 1-hour infusions followed by a 2-week rest. The starting dose of 100 mg/m2/week was 66% of the maximum tolerated dose in adults. Escalated dose levels given were: 125, 150, 175, and 210 mg/m2. Myelosuppression was dose limiting, with thrombocytopenia more pronounced than leukopenia. There was no evidence of cumulative toxicity. The maximum tolerated dose for children with solid tumors was 210 mg/m2/week X 4. Other side effects included transient nausea and vomiting at the higher dose... (truncated)

Title: Pharmacological Effects of Cisplatin Combination with Natural Products in Cancer Chemotherapy.
Cisplatin and other platinum-based drugs, such as carboplatin, ormaplatin, and oxaliplatin, have been widely used to treat a multitude of human cancers. However, a considerable proportion of patients often relapse due to drug resistance and/or toxicity to multiple organs including the liver, kidneys, gastrointestinal tract, and the cardiovascular, hematologic, and nervous systems. In this study, we sought to provide a comprehensive review of the current state of the science highlighting the use of cisplatin in cancer therapy, with a special emphasis on its molecular mechanisms of action, and treatment modalities including the combination therapy with natural products. Hence, we searched... (truncated)

Title: Hepatotoxicity induced by paclitaxel interaction with turmeric in association with a microcystin from a contaminated dietary supplement.
A 67-year-old Caucasian male with lung cancer was presented to the Emergency Department with asthenia, anorexia, jaundice and choluria. The patient's lung cancer was being treated medically by a combination of paclitaxel/carboplatin with bi-monthly frequency. The patient was also self-medicating with several natural products, including Chlorella (520 mg/day), Silybum marianum (total of 13.5 mg silymarin/day), zinc sulphate (5.5 mg), selenium (50 µg) and 15 g/day of Curcuma longa. In first chemotherapy cycle no toxicity was observed even he was taking other medications as budesonide and sitagliptin. The toxic events started only after the introduction of the dietary products. Chlorella had... (truncated)

MESH:D007538 - isoniazid

Summary:

LLM Prediction Score	0.867 (normalized)
LLM Confidence Score	0.990
Golden Answer (Severity Class)	1.0 (normalized)
Prediction Error	0.133

Retrieved Context:

Title: Isoniazid-associated hepatitis in 114 patients.
Analysis of the overt hepatic disease that developed in 114 patients while taking isoniazid for chemoprophylaxis of tuberculosis showed it to be mainly hepatocellular. The severity of the hepatic injury was manifested by the 13 fatalities (12.3%) and by the histological demonstration of submassive necrosis in 9 and massive necrosis in 4 patients. The 20 other patients from whom hepatic tissue was available for study included 16 with moderately severe acute hepatocellular injury (4 had a mixed hepatocellular-cholestatic pattern), and 4 with chronic hepatic diseases (1 had cirrhosis). Clinical manifestations of hepatic disease prior to the onset of jaundice included... (truncated)

Title: Toxic hepatitis with jaundice occuring in a patient treated with isoniazid.
No abstract available.

Title: Isoniazid-induced acute liver failure during preventive therapy for latent tuberculosis infection.
Treating latent tuberculosis infection is a strategy for eliminating tuberculosis, and isoniazid is recommended as preventive therapy. However, concerns have been raised regarding the application of isoniazid due to its toxicity, particularly hepatotoxicity; however, biochemical monitoring is not routinely performed during treatment. We herein present a case of fatal isoniazid-induced acute liver failure. The patient's liver function was not periodically examined and isoniazid therapy was continued for 10 days despite the onset of symptoms associated with hepatitis. The patient died four months after hospitalization. It is essential to consider the potential toxicities of isoniazid and establish strategies to prevent acute... (truncated)

Title: Hepatotoxicity caused by the combined action of isoniazid and rifampicin.
A 35 year old black Somalian woman with miliary tuberculosis developed hepatotoxicity after a few days of treatment with isoniazid, rifampicin, pyrazinamide, and ethambutol. After withdrawal of all drugs the liver profile returned to normal and remained so after challenge with isoniazid. Hepatotoxicity recurred when rifampicin was added, but it was well tolerated when reintroduced without isoniazid.

Title: Isoniazid metabolism and hepatotoxicity.
Isoniazid (INH) is highly effective for the management of tuberculosis. However, it can cause liver injury and even liver failure. INH metabolism has been thought to be associated with INH-induced liver injury. This review summarized the metabolic pathways of INH and discussed their associations with INH-induced liver injury.

MESH:D015760 - alfentanil

Summary:

LLM Prediction Score	0.133 (normalized)
LLM Confidence Score	0.970
Golden Answer (Severity Class)	0.0 (normalized)
Prediction Error	0.133

Retrieved Context:

Title: Relevance of Liver Failure for Anti-Infective Agents: From Pharmacokinetic Alterations to Dosage Adjustments.
The liver is a complex organ with great ability to influence drug pharmacokinetics. Due to its wide array of function, its impairment has the potential to affect bioavailability, enterohepatic circulation, drug distribution, metabolism, clearance, and biliary elimination. These alterations differ widely depending on the cause of the liver failure, if it is acute or chronic in nature, the extent of impairment, and comorbid conditions. In addition, effects on liver functions do not occur in a proportional or predictable manner for escalating degrees of liver impairment. The ability of hepatic alterations to influence PK is also dependent on drug characteristics, such... (truncated)

Title: Analgesics in patients with hepatic impairment: pharmacology and clinical implications.
The physiological changes that accompany hepatic impairment alter drug disposition. Porto-systemic shunting might decrease the first-pass metabolism of a drug and lead to increased oral bioavailability of highly extracted drugs. Distribution can also be altered as a result of impaired production of drug-binding proteins or changes in body composition. Furthermore, the activity and capacity of hepatic drug metabolizing enzymes might be affected to various degrees in patients with chronic liver disease. These changes would result in increased concentrations and reduced plasma clearance of drugs, which is often difficult to predict. The pharmacology of analgesics is also altered in liver disease.... (truncated)

Title: Drug Interactions With New Synthetic Opioids.
Fentanyl, fentanyl analogs, and other new synthetic opioids (NSO) have burst onto the illegal drug market as new psychoactive substances (NPS). They are often sold as heroin to unsuspecting users and produce euphoria through their agonist action on μ- opioid receptors. Their high consumption, often combined with other substances, has led to multiple intoxications during recent years. In some countries, such as the United States, the consumption of opioids, whether for medical or recreational purposes, has become epidemic and is considered a public health problem. Fentanyl analogs are more potent than fentanyl which in turn is 50 times more potent... (truncated)

Title: Role of cytochrome P4502B6 in methadone metabolism and clearance.
Methadone N-demethylation in vitro is catalyzed by hepatic cytochrome P4502B6 (CYP2B6) and CYP3A4, but clinical disposition is often attributed to CYP3A4. This investigation tested the hypothesis that CYP2B6 is a prominent CYP isoform responsible for clinical methadone N-demethylation and clearance, using the in vivo mechanism-based CYP2B6 inhibitor ticlopidine, given orally for 4 days. A preliminary clinical investigation with the CYP3A4/5 substrate probe alfentanil established that ticlopidine did not inhibit intestinal or hepatic CYP3A4/5. Subjects received intravenous plus oral (deuterium-labeled) racemic methadone before and after ticlopidine. Ticlopidine significantly and stereoselectively (S > R) inhibited methadone N-demethylation, decreasing plasma metabolite/methadone area under... (truncated)

Title: Pharmacokinetics of Fentanyl and Its Derivatives in Children: A Comprehensive Review.
Fentanyl and its derivatives sufentanil, alfentanil, and remifentanil are potent opioids. A comprehensive review of the use of fentanyl and its derivatives in the pediatric population was performed using the National Library of Medicine PubMed. Studies were included if they contained original pharmacokinetic parameters or models using established routes of administration in patients younger than 18 years of age. Of 372 retrieved articles, 44 eligible pharmacokinetic studies contained data of 821 patients younger than 18 years of age, including more than 46 preterm infants, 64 full-term neonates, 115 infants/toddlers, 188 children, and 28 adolescents. Underlying diagnoses included congenital heart and pulmonary disease... (truncated)

MESH:D009270 - naloxone

Summary:

LLM Prediction Score	0.133 (normalized)
LLM Confidence Score	0.990
Golden Answer (Severity Class)	0.0 (normalized)
Prediction Error	0.133

Retrieved Context:

Title: Buprenorphine-naloxone treatment in opioid dependence and risk of liver enzyme elevation: results from a 12-month observational study.
Some case series mention possible liver toxicity in opioid-dependent patients under buprenorphine treatment.

Title: Identifying olanzapine induced liver injury in the setting of acute hepatitis C: A case report.
Olanzapine is linked to asymptomatic, transient elevations of liver aminotransferases but is historically thought to rarely cause significant hepatotoxicity. Underlying liver disease is a risk factor for drug-induced liver injury and may complicate the differential diagnosis of acute transaminitis in patients taking medications associated with hepatotoxicity. Ms L presented with 2 months of new psychotic symptoms resulting in hospitalizations. Although psychosis previously improved with haloperidol, she reported symptoms concerning for akathisia. Restlessness improved and psychotic symptoms resolved after initiation of olanzapine. Concurrently, her alanine aminotransferase (ALT) was elevated, prompting further workup and new diagnosis of acute hepatitis C. Over the... (truncated)

Title: Buprenorphine/Naloxone and methadone effects on laboratory indices of liver health: a randomized trial.
Buprenorphine/naloxone (BUP) and methadone (MET) are efficacious treatments for opioid dependence, although concerns about a link between BUP and drug-induced hepatitis have been raised. This study compares the effects of BUP and MET on liver health in opioid-dependent participants.

Title: Hepatotoxicity in a 52-week randomized trial of short-term versus long-term treatment with buprenorphine/naloxone in HIV-negative injection opioid users in China and Thailand.
Buprenorphine/naloxone (BUP/NX), an effective treatment for opioid dependence, has been implicated in hepatic toxicity. However, as persons taking BUP/NX have multiple hepatic risk factors, comparative data are needed to quantify the risk of hepatoxicity with BUP/NX.

Title: Cocaine and Opioid-Induced Acute Liver Injury: A Rare Case Report.
Drug overdose has been a public health burden in the United States. Repeated use of cocaine and heroin may increase the risk of severe acute liver failure. We present the case of a middle-aged man with no significant past medical condition except a chronic history of drug abuse who presented to our hospital after an overdose of cocaine and heroin. Patient received Narcan by paramedics and continued treatment in the emergency room (ER). Patient has exhibited multiple organ failures, such as acute liver failure, rhabdomyolysis, acute kidney injury, and acute respiratory hypoxic hypercapnic respiratory failure likely due to respiratory center... (truncated)

MESH:C041747 - nitazoxanide

Summary:

LLM Prediction Score	0.132 (normalized)
LLM Confidence Score	0.980
Golden Answer (Severity Class)	0.0 (normalized)
Prediction Error	0.132

Retrieved Context:

Title: In vitro and in vivo effects of 3-bromopyruvate against Echinococcus metacestodes.
While searching for novel anti-echinococcosis drugs, we have been focusing on glycolysis which is relied on by Echinococcus for energy production and intermediates for other metabolic processes. The aim of this study was to investigate the potential therapeutic implication of glycolytic inhibitors on Echinococcus. Our results demonstrate that at an initial concentration of 40 μM, all inhibitors of glycolysis used in the current experiment [3-bromopyruvate (3-BrPA), ornidazole, clorsulon (CLS), sodium oxamate and 2,6-dihydroxynaphthalene (NA-P₂)] show considerable in vitro effects against Echinococcus granulosus protoscoleces and Echinococcus multilocularis metacestodes. Among them, 3-BrPA exhibited the highest activity which was similar to that of nitazoxanide... (truncated)

Title: Current trends in the treatment of hepatic encephalopathy.
Hepatic encephalopathy (HE) is a common reversible neuropsychiatric syndrome associated with chronic and acute liver dysfunction and significant morbidity and mortality. Although a clear pathogenesis is yet to be determined, elevated ammonia in the serum and central nervous system are the mainstay for pathogenesis and treatment. Management includes early diagnosis and prompt treatment of precipitating factors (infection, gastrointestinal bleeding, electrolyte disturbances, hepatocellular carcinoma, dehydration, hypotension, and use of benzodiazepines, psychoactive drugs, and/or alcohol). Clinical trials have established the efficacy of lactulose and lactitol enemas in the treatment of acute hepatic encephalopathy. Extensive clinical experience has demonstrated the efficacy of oral... (truncated)

Title: Active Clinical Trials in Hepatic Encephalopathy: Something Old, Something New and Something Borrowed.
Hepatic encephalopathy (HE) is a potentially reversible neurocognitive syndrome that occurs in patients with acute or chronic liver disease. Currently, most of the therapies for HE aim to reduce ammonia production or increase its elimination. To date, only two agents have been approved as treatments for HE: lactulose and rifaximin. Many other drugs have also been used, but data to support their use are limited, preliminary or lacking. The aim of this review is to provide an overview and discussion of the current development of treatments for HE. Data from ongoing clinical trials in HE were obtained from the ClinicalTrials.gov... (truncated)

Title: Synthesis and antimicrobial evaluation of nitazoxanide-based analogues: identification of selective and broad spectrum activity.
A library composed of nitazoxanide-based analogues was synthesized and assayed for increased antibacterial efficacy against the pyruvate-ferredoxin oxidoreductase (PFOR) using microorganisms Helicobacter pylori, Campylobacter jejuni and Clostridium difficile. Derivatives were found to recapitulate and improve activity against these organisms and select analogues were tested for their ability to disrupt the PFOR enzyme directly. The library was also screened for activity against staphylococci and resulted in the identification of analogues capable of inhibiting both staphylococci and all PFOR organisms at low micromolar minimum inhibitory concentrations with low toxicity to human foreskin cells.

Title: Thiazolides as novel antiviral agents. 1. Inhibition of hepatitis B virus replication.
We report the syntheses and activities of a wide range of thiazolides [viz., 2-hydroxyaroyl-N-(thiazol-2-yl)amides] against hepatitis B virus replication, with QSAR analysis of our results. The prototypical thiazolide, nitazoxanide [2-hydroxybenzoyl-N-(5-nitrothiazol-2-yl)amide, NTZ] 1 is a broad spectrum antiinfective agent effective against anaerobic bacteria, viruses, and parasites. By contrast, 2-hydroxybenzoyl-N-(5-chlorothiazol-2-yl)amide 3 is a novel, potent, and selective inhibitor of hepatitis B replication (EC(50) = 0.33 μm) but is inactive against anaerobes. Several 4'- and 5'-substituted thiazolides show good activity against HBV; by contrast, some related salicyloylanilides show a narrower spectrum of activity. The ADME properties of 3 are similar to 1; viz.,... (truncated)

MESH:D004949 - estazolam

Summary:

LLM Prediction Score	0.131 (normalized)
LLM Confidence Score	0.830
Golden Answer (Severity Class)	0.0 (normalized)
Prediction Error	0.131

Retrieved Context:

Title: Insomnia Promotes Hepatic Steatosis in Rats Possibly by Mediating Sympathetic Overactivation.
Background: Insomnia is a widespread problem that can lead to the occurrence of other diseases and correlates closely with sympathetic nerve hyperactivation. Obesity-induced hepatic steatosis is mediated by sympathetic overactivation. However, it remains unclear whether insomnia may cause hepatic steatosis. The goal of this study was to preliminarily investigate whether insomnia caused hepatic steatosis in rats <i>via</i> sympathetic hyperactivation. Methods: A total of 32 Sprague-Dawley male rats were divided randomly into four groups: model, sympathetic denervation (Sd), estazolam, and control (eight rats/group). Model group received sustained sleep deprivation using the modified multiple platform method. In the Sd group, rats underwent... (truncated)

Title: Editorial: Alcohol-associated liver disease-From pathogenesis to treatment.
No abstract available.

Title: Midazolam Ameliorates Acute Liver Injury Induced by Carbon Tetrachloride <i>via</i> Enhancing Nrf2 Signaling Pathway.
Oxidative stress contributes greatly to initiation and progression of liver injury. Activation of nuclear-factor erythroid 2-related factor 2 (Nrf2) has been considered as an attractive strategy for preventing and treating the oxidative damage related to liver injury. This study aimed to find an efficacious agent to activate Nrf2/HO-1 signaling pathway from clinically used therapeutic agents and to characterize the usefulness for preventing and treating CCl₄-induced acute liver injury. For this purpose, a series of clinically used therapeutic agents were collected and their activation potentials on Nrf2 were assayed by using 293T-Nrf2-luc cell line. Among all tested therapeutic agents, midazolam was... (truncated)

Title: Serum alanine transaminase total bilirubin concentrations predict CYP3A activity as measured by midazolam and 1'-hydroxylation.
Microsomal enzyme P450 (CYP450) plays an important role in metabolism of most xenobiotics. The activity of CYP3A decreases in patients with liver dysfunction. However, whether serum concentrations of liver enzymes reflect the activity of CYP3A is unclear. We aimed to search for a new clue to predict the activity of CYP3A and guide clinical medication.

Title: Clinical Presentations and Predictors of In-Hospital Mortality in Illicit Drug Users in the New Psychoactive Substances (NPS) Endemic Era in Taiwan.
Predictors of mortality in illicit drug users involving Novel Psychoactive Substances (NPS) and multiple substances have not been elucidated. We aimed to define predictors of mortality in the NPS endemic era's illicit drug users to strengthen patient care in emergency treatment. This was a retrospective study. LC-MS/MS-confirmed positive illicit drug users who visited the emergency departments (ED) of six medical systems were enrolled. Demographic information, physical examinations, and laboratory data were abstracted for mortality analysis. There were 16 fatalities in 355 enrolled patients. The most frequently used illicit drugs were amphetamines, followed by opioids, cathinones, and ketamine. The most frequently... (truncated)

MESH:D007654 - ketoconazole

Summary:

LLM Prediction Score	0.869 (normalized)
LLM Confidence Score	0.990
Golden Answer (Severity Class)	1.0 (normalized)
Prediction Error	0.131

Retrieved Context:

Title: Liver cirrhosis developed after ketoconazole-induced acute hepatic injury.
We describe a previously healthy woman who developed liver cirrhosis as a sequela of acute hepatic injury that was induced by ketoconazole administration to treat onychomycosis. The initial presentation of the disease was of a typical acute hepatitis, characterized by nausea, anorexia, fatigue, and jaundice that developed during the administration of ketoconazole. Many other causes of hepatitis were absent in the patient. Even though the hepatic injury was gradually resolved for several months after cessation of the drug, the liver function was not completely restored. Six months after the onset of illness, a follow-up abdominal computed tomography and peritoneoscopic liver... (truncated)

Title: Hepatic injury associated with ketoconazole therapy. Analysis of 33 cases.
Ketoconazole has only recently been recognized as a cause of hepatic injury, with most reports coming from outside the United States. In order to characterize more fully the U.S. experience, we undertook an analysis of 54 reports of alleged ketoconazole-induced liver injury submitted to the Food and Drug Administration from the time of initial marketing in 1980. Thirty-three reports were considered likely instances of ketoconazole-induced hepatitis. The majority of these cases occurred in women more than 40 yr of age. Jaundice was recorded in 27 individuals after therapy of 11-168 days with an average daily dose of 200 mg. Anorexia,... (truncated)

Title: Hepatic reactions associated with ketoconazole in the United Kingdom.
Ketoconazole was introduced in the United Kingdom in 1981. By November 1984 the Committee on Safety of Medicines had received 82 reports of possible hepatotoxicity associated with the drug, including five deaths. An analysis of the 75 cases that had been adequately followed up suggested that 16, including three deaths, were probably related to treatment with the drug. Of the remainder, 48 were possibly related to treatment, five were unlikely to be so, and six were unclassifiable. The mean age of patients in the 16 probable cases was 57.9, with hepatotoxicity being more common in women. The average duration of... (truncated)

Title: Prolonged jaundice following ketoconazole-induced hepatic injury.
Two patients developed prolonged and progressive jaundice associated with ketoconazole-induced hepatic injury although the drug was discontinued before or shortly after the onset of symptoms of hepatic toxicity. One patient, who had been jaundiced for eight weeks and was not improving, showed prompt clinical improvement and progressive resolution of jaundice following therapy with prednisolone. Liver biopsy before therapy showed marked cholestasis in all acinar zones and moderately severe fibrosis in the space of Disse. The other patient, who was less severely jaundiced, showed spontaneous resolution although he remained jaundiced for 11 weeks. Liver biopsy performed three weeks after onset of... (truncated)

Title: Adverse effects of ketoconazole in dogs--a retrospective study.
Although ketoconazole has been used extensively in dogs for the treatment of various fungal infections, information about adverse effects is mainly anecdotal. Common adverse effects in humans include dose-dependant anorexia, nausea and vomiting, allergic rashes and pruritus. Drug-induced hepatitis is very rare, but potentially fatal. The aim of this study was to evaluate the type and frequency of adverse effects associated with ketoconazole therapy in dogs treated for skin diseases and any possible influence of dosage, duration of therapy, signalment or concurrent medication. The medical records of 632 dogs treated with ketoconazole (2.6-33.4 mg/kg) were reviewed. Adverse effects occurred in... (truncated)

MESH:D000077731 - meropenem

Summary:

LLM Prediction Score	0.506 (normalized)
LLM Confidence Score	0.990
Golden Answer (Severity Class)	0.375 (normalized)
Prediction Error	0.131

Retrieved Context:

Title: A case of meropenem-induced liver injury and jaundice.
This report describes what we believe is the first reported case of clinically significant cholestasis and acute liver injury within three days of meropenem therapy. An 83-year-old Hispanic female was admitted for sepsis of unknown origin and was started on intravenous meropenem. Three days following initiation of the antibiotic, the patient developed mixed hepatocellular and cholestatic liver injury with jaundice and pruritus. Possible causes of cholestasis were excluded after extensive investigations. A drug-induced liver injury was suspected and meropenem was discontinued. Following discontinuation of meropenem, the patient demonstrated symptomatic and laboratory improvements, and her liver enzymes and bilirubin levels were... (truncated)

Title: Meropenem-induced vanishing bile duct syndrome.
Vanishing bile duct syndrome (VBDS) is a rare and potentially life-threatening disorder in which progressive destruction and disappearance of small intrahepatic bile ducts occur, with resultant cholestasis. The mechanism by which biliary epithelial cells are damaged and intrahepatic bile ducts are lost has not been fully elucidated. However, many etiologies have been reported,and several drugs have been implicated. Meropenem is a widely used, well tolerated broad-spectrum carbapenem antibiotic indicated for the treatment of intraabdominal infections, complicated skin and skin structure infections, and pediatric bacterial meningitis. We describe what we believe is the first reported case of meropenem-induced VBDS. A 60-year-old... (truncated)

Title: Influence of Antibiotics on Functionality and Viability of Liver Cells In Vitro.
(1) Antibiotics are an important weapon in the fight against serious bacterial infections and are considered a common cause of drug-induced liver injury (DILI). The hepatotoxicity of many drugs, including antibiotics, is poorly analyzed in human in vitro models. (2) A standardized assay with a human hepatoma cell line was used to test the hepatotoxicity of various concentrations (Cmax, 5× Cmax, and 10× Cmax) of antibiotics. In an ICU, the most frequently prescribed antibiotics, ampicillin, cefepime, cefuroxime, levofloxacin, linezolid, meropenem, rifampicin, tigecycline, and vancomycin, were incubated with HepG2/C3A cells for 6 days. Cell viability (XTT assay, LDH release, and vitality),... (truncated)

Title: Pharmacokinetics and Pharmacodynamics of Meropenem by Extended or Continuous Infusion in Low Body Weight Critically Ill Patients.
<i>Background</i>: Pathophysiological changes such as extreme body weights in critically ill patients with severe infections may alter the pharmacokinetics (PK) of antimicrobials, leading to treatment failure or toxicity. There are almost no PK data on meropenem in critically ill patients with low body weight (LwBW) and therefore information is lacking on the most appropriate dosing regimens, especially when administered by extended infusion. <i>Objectives</i>: To assess if the current administered doses of meropenem could lead to supratherapeutic concentrations in LwBW patients and to identify the factors independently associated with overexposure. Methods: A matched case-control 1:1 study of surgical critically ill patients... (truncated)

Title: Signal Detection of Potential Hepatotoxic Drugs: Case-Control Study Using Both a Spontaneous Reporting System and Electronic Medical Records.
Drug-induced liver injury (DILI) is a common adverse drug event. Spontaneous reporting systems such as the Japanese Adverse Event Report Database (JADER) have been used to evaluate the association between drugs and adverse drug events. However, the association of drugs with adverse drug events may be overestimated due to reporting biases. Therefore, it is important to objectively evaluate the association using liver function test values. The aim of the present study was to predict potential hepatotoxic drugs using real-world data including electronic medical records and the JADER database. A total of 70009 (2779 with DILI and 67230 without DILI) and... (truncated)

MESH:D000077384 - anastrozole

Summary:

LLM Prediction Score	0.504 (normalized)
LLM Confidence Score	0.980
Golden Answer (Severity Class)	0.375 (normalized)
Prediction Error	0.129

Retrieved Context:

Title: An unusual cause of jaundice in a patient with breast cancer.
A 48-year old woman with metastatic breast cancer and extensive bone marrow infiltration was admitted with extreme lethargy, jaundice and deranged liver function tests. She had been started on anastrozole in May 2013 for bony metastases, detected on a bone scan. A CT scan performed at that time had shown no evidence of metastatic or nodal disease elsewhere. Over the subsequent 2 months, the patient had become progressively jaundiced. Outpatient abdominal ultrasound and CT liver had shown a fatty liver with no focal lesions. She was admitted in August 2013 with bilirubin 567, alkaline phosphatase 385, alanine aminotransferase 98, albumin... (truncated)

Title: Drug-induced liver injury after switching from tamoxifen to anastrozole in a patient with a history of breast cancer being treated for hypertension and diabetes.
Anastrozole is a selective non-steroidal aromatase inhibitor that blocks the conversion of androgens to estrogens in peripheral tissues. It is used as adjuvant therapy for early-stage hormone-sensitive breast cancer in postmenopausal women. Significant side effects of anastrozole include osteoporosis and increased levels of cholesterol. To date, seven case reports on anastrozole hepatotoxicity have been published. We report the case of an 81-year-old woman with a history of breast cancer, arterial hypertension, type 2 diabetes mellitus, hyperlipidemia, and chronic renal insufficiency. Four days after switching hormone therapy from tamoxifen to anastrozole, icterus developed along with a significant increase in liver enzymes... (truncated)

Title: A phase II neoadjuvant trial of anastrozole, fulvestrant, and gefitinib in patients with newly diagnosed estrogen receptor positive breast cancer.
Endocrine therapy in patients with breast cancer can be limited by the problem of resistance. Preclinical studies suggest that complete blockade of the estrogen receptor (ER) combined with inhibition of the epidermal growth factor receptor can overcome endocrine resistance. We tested this hypothesis in a phase II neoadjuvant trial of anastrozole and fulvestrant combined with gefitinib in postmenopausal women with newly diagnosed ER-positive breast cancer. After a baseline tumor core biopsy, patients were randomized to receive anastrozole and fulvestrant or anastrozole, fulvestrant, and gefitinib (AFG) for 3 weeks. After a second biopsy at 3 weeks, all patients received AFG for... (truncated)

Title: Anastrozole-induced liver injury after a prolonged latency: a very rare complication of a commonly prescribed medication.
Anastrozole is an aromatase inhibitor that has been used more frequently over the last decade especially for oestrogen receptor-positive breast cancer. It has a relatively safe side effect profile. However, occasionally it has been associated with serious adverse events. Here, we present the case of a 58-year-old woman who presented with significantly elevated liver enzymes 4 years after starting anastrozole. She was not taking any other medications and an extensive workup did not reveal any other cause for her liver injury. The patient's liver enzymes normalised after discounting the anastrozole. She scored 4 on the updated Roussel Uclaf Causality Assessment... (truncated)

Title: A prospective, randomized study on hepatotoxicity of anastrozole compared with tamoxifen in women with breast cancer.
Tamoxifen and anastrozole are widely used as adjuvant treatment for early stage breast cancer, but their hepatotoxicity is not fully defined. We aimed to compare hepatotoxicity of anastrozole with tamoxifen in the adjuvant setting in postmenopausal breast cancer patients. Three hundred and fifty-three Chinese postmenopausal women with hormone receptor-positive early breast cancer were randomized to anastrozole or tamoxifen after optimal primary therapy. The primary end-point was fatty liver disease, defined as a liver-spleen ratio <0.9 as determined using a computed tomography scan. The secondary end-points included abnormal liver function and treatment failure during the 3-year follow up. The cumulative incidence... (truncated)

MESH:D000077402 - pantoprazole

Summary:

LLM Prediction Score	0.504 (normalized)
LLM Confidence Score	0.990
Golden Answer (Severity Class)	0.375 (normalized)
Prediction Error	0.129

Retrieved Context:

Title: Liver hepatotoxicity associated with pantoprazole: a rare case report.
Hepatotoxicity may occasionally develop over the course of treatment with proton pump inhibitors (PPIs). Although skin reactions, interstitial nephritis, pancytopenia, anaphylaxis, and generalized edema have been reported to be associated with PPIs, hepatotoxicity associated with oral pantoprazole is very rare. In this report, we present a case of hepatotoxicity in a 35-year-old man who received pantoprazole (40 mg/day) for acute gastritis. One week after discontinuation of pantoprazole, his liver function began to improve, and the patient gradually fully recovered. Although this toxicity occurs only infrequently, pantoprazole should be considered as a rare hepatotoxic agent in the literature.

Title: Acute hepatitis after starting pinaverium bromide in a patient taking mirtazapine.
A 56-year-old man presented with chronic abdominal pain. He had been evaluated extensively in the recent past undergoing upper gastrointestinal endoscopy, colonoscopy and CT scan of the abdomen with normal results. The provisional diagnosis of irritable bowel syndrome was performed and pinaverium bromide was started. The patient had pre-existing hypertension, a major depressive disorder and gastro-oesophageal reflux disease. He had been taking nebivolol and pantoprazole for several years and mirtazapine for the last 1 year. The patient developed nausea, vomiting and anorexia after 5 days of starting pinaverium bromide. Investigations revealed marked elevation of liver enzymes and bilirubin. He was... (truncated)

Title: A case of probable esomeprazole-induced transient liver injury in a pregnant woman with hyperemesis.
We report a case of 22-year-old primigravida presented to Women's Hospital - Hamad Medical Corporation emergency with severe epigastric pain, nausea, and vomiting. On admission, she was dehydrated with remarkably worsening symptoms. Laboratory findings revealed significantly elevated liver enzymes with unknown etiology. Her past medical history showed an admission for nausea and vomiting 3 weeks previously and she was discharged on antiemetics, and esomeprazole for the first time. Due to the predominantly elevated liver enzymes, the clinical pharmacist discussed the possibility of esomeprazole-induced adverse effects and suggested to suspend esomeprazole based on the evidence from literature review. The liver enzymes... (truncated)

Title: Pantoprazole ameliorates liver fibrosis and suppresses hepatic stellate cell activation in bile duct ligation rats by promoting YAP degradation.
Liver fibrosis is one of the most severe pathologic consequences of chronic liver diseases, and effective therapeutic strategies are urgently needed. Proton pump inhibitors (PPIs) are H⁺/K⁺-ATPase inhibitors and currently used to treat acid-related diseases such as gastric ulcers, which have shown other therapeutic effects in addition to inhibiting acid secretion. However, few studies have focused on PPIs from the perspective of inhibiting hepatic fibrosis. In the present study, we investigated the effects of pantoprazole (PPZ), a PPI, against liver fibrosis in a bile duct ligation (BDL) rat model, human hepatic stellate cell (HSC) line LX-2 and mouse primary HSCs... (truncated)

Title: Pantoprazole induces severe acute hepatitis.
A female patient receiving pantoprazole during a corticosteroid therapy for encephalomyelitis disseminata developed severe acute hepatitis one month after initiation of pantoprazole treatment. Other causes of hepatic dysfunction including viral hepatitis, autoimmune hepatitis, primary sclerosing cholangitis, primary biliary cirrhosis, haemochromatosis or Wilson's disease were excluded. Liver biopsy showed severe hepatic lesions with extensive necroses of the parenchyma. One week after discontinuation of pantoprazole the liver function began to improve and gradually the patient fully recovered. One year earlier the patient had been treated with pantoprazole before and had developed a milder form of hepatitis then. This case argues for an... (truncated)

MESH:C084606 - ethotoin

Summary:

LLM Prediction Score	0.504 (normalized)
LLM Confidence Score	0.900
Golden Answer (Severity Class)	0.375 (normalized)
Prediction Error	0.129

Retrieved Context:

Title: Safety of anticonvulsants in hepatic porphyrias.
Because acute attacks of porphyria may be precipitated by anticonvulsants, a therapeutic dilemma arises when seizures complicate hepatic porphyria. The list of unsafe agents includes barbiturates, primidone, phenytoin, mephenytoin, ethotoin, ethosuximide, methsuximide, phensuximide, and trimethadione. Agents are considered unsafe if they induce experimental porphyria in animals, and short trials in patients are unreliable for screening. Using drug incubation in chick-embryo hepatocyte culture, we found that porphyrin was increased by carbamazepine, clonazepam, and valproate. These agents should probably be avoided or used with caution in porphyric patients. Alternative approaches to acute porphyric attacks with seizures are discussed.

Title: Utility of spherical human liver microtissues for prediction of clinical drug-induced liver injury.
Drug-induced liver injury (DILI) continues to be a major source of clinical attrition, precautionary warnings, and post-market withdrawal of drugs. Accordingly, there is a need for more predictive tools to assess hepatotoxicity risk in drug discovery. Three-dimensional (3D) spheroid hepatic cultures have emerged as promising tools to assess mechanisms of hepatotoxicity, as they demonstrate enhanced liver phenotype, metabolic activity, and stability in culture not attainable with conventional two-dimensional hepatic models. Increased sensitivity of these models to drug-induced cytotoxicity has been demonstrated with relatively small panels of hepatotoxicants. However, a comprehensive evaluation of these models is lacking. Here, the predictive value... (truncated)

MESH:D000077156 - gefitinib

Summary:

LLM Prediction Score	0.629 (normalized)
LLM Confidence Score	0.980
Golden Answer (Severity Class)	0.5 (normalized)
Prediction Error	0.129

Retrieved Context:

Title: [Safety analysis of eight patients treated with erlotinib after severe gefitinib-induced liver injury].
Gefitinib and erlotinib are commercially available epidermal growth factor receptor tyrosine kinase inhibitors (EGFR-TKIs) that are widely used for the treatment of non-small-cell lung cancer (NSCLC). Acute severe liver injury, although rare, has been observed in patients receiving these EGFR-TKIs. Some studies have reported that erlotinib treatment does not cause severe liver toxicity in patients with NSCLC who previously presented with severe liver injury during the course of gefitinib treatment. We retrospectively assessed the occurrence of liver toxicity in patients with NSCLC who were receiving erlotinib and had previously presented with severe gefitinib-induced liver injury.Severe liver injury occurred in only... (truncated)

Title: Treatment of Non-Small-Cell Lung Cancer with Erlotinib following Gefitinib-Induced Hepatotoxicity: Review of 8 Clinical Cases.
Objective. Gefitinib often induces liver damage. A few reports have described that the subsequent administration of erlotinib was associated with less hepatotoxicity, but the safety and efficacy of this treatment are still not fully investigated. Therefore, we evaluated retrospectively the patients with erlotinib following gefitinib-induced hepatotoxicity. Methods and Patients. We retrospectively reviewed the medical records between December 2007 and March 2010. The patients were evaluated including the following information: age, gender, histology of lung cancer, performance status, smoking status, epidermal growth factor receptor (EGFR) mutation status, liver metastasis, viral hepatitis, alcoholic liver injury, clinical response, and hepatotoxicity due to EGFR... (truncated)

Title: [Transient liver injury caused by gefitinib].
Gefitinib blocks epidermal growth factor receptor autophosphorylation and subsequently the signal transduction pathways implicated in proliferation, metastasis, invasion, and angiogenesis. Reported adverse reactions to gefitinib include liver injury that is not fully understood. Liver injury was observed in 5 (12.2%) of 41 patients with non-small cell lung cancer who received gefinitib monotherapy. Onset of liver injury was seen between 28 and 56 days after initiation of administration. Two patients had Grade 2 liver injury and 3 patients, Grade 3. In 4 patients, liver injury was temporary, lasting during a period of continuous gefitinib administration. In another patient, gefitinib was discontinued... (truncated)

Title: Rechallenge with gefitinib following severe drug-induced hepatotoxicity in a patient with advanced non-small cell lung cancer: A case report and literature review.
Gefitinib has come to be the most widely used epidermal growth factor receptor-tyrosine kinase inhibitor in the treatment of advanced non-small cell lung cancer (NSCLC) in Asian patients. Common side effects include mild to moderate skin rash and diarrhea, however, drug-induced liver injury of varying severity is overlooked in long-term gefitinib administration and rarely reported. The current case report presents a female Chinese NSCLC patient who developed severe gefitinib-induced hepatotoxicity and was rechallenged with gefitinib following a 3-month break. The patient achieved partial clinical remission but developed drug-induced grade 4 hepatotoxicity following gefitinib administration for 14 months. As an alternative,... (truncated)

Title: Treatment with gefitinib after erlotinib-induced liver injury: a case report.
Gefitinib and erlotinib have minor differences in their chemical structures, and thus it remains unclear whether the hepatotoxicity induced by one compound is affected by the other. The case of a patient who developed erlotinib-induced liver injury and was then treated with gefitinib without hepatic toxicity or disease progression is presented.

MESH:D010869 - pindolol

Summary:

LLM Prediction Score	0.128 (normalized)
LLM Confidence Score	0.890
Golden Answer (Severity Class)	0.0 (normalized)
Prediction Error	0.128

Retrieved Context:

Title: [Comparative study on the elimination of pindolol (visken) and antipyrin in patients with liver diseases].
25 patients suffering from different hepatic diseases were studied in order to investigate the elimination of the beta-receptor blocking drug pindolol in hepatic disease. Following an overnight fasting period the patients received simultaneously 3 mg pindolol intravenously and 1000 mg antipyrine orally. Plasma samples were taken at certain time intervals for 24 hs and urine was collected for 72 hs for the measurements of drug concentrations in plasma and urine. From these measurements different pharmacokinetic parameters were calculated for both drugs used in the present study according to a one-compartment open model. The total body clearance of antipyrine was selected... (truncated)

Title: The emergence of new antidepressants for clinical use: Agomelatine paradox versus other novel agents.
This study was designed with the rational aim of discussing the emerging antidepressant agents that are likely to bring positive landmark, tremendous improvement and significant impact to the management of patients with depression disorders. It also elaborates on the Agomelatine paradox vis-a-vis the other novel antidepressant agents. The emerging antidepressants are: selective monoamine oxidase inhibitors (MAOIs) such as bifemelane, pirlindole, toloxatone, selegiline, rasagiline and safinamide; serotonin-norepinephrine reuptake inhibitors (SNRIs) such as ansofaxine, nefopam and levomilnacipran; norepinephrine reuptake inhibitors (NRIs) such as Reboxetine, viloxazine, teniloxazine (also known as sulfoxazine or sufoxazine), and atomoxetine; Vilazodone (a serotonin 5-HT_{1A} autoreceptor partial agonist with... (truncated)

Title: A preliminary metabolites identification of a novel compound with β-adrenolytic activity.
The identification of main metabolites and assessment of renal excretion of a novel compound with β-adrenolytic activity (2RS)-1-(1H-indol-4-yloxy)-3-((2-(2-methoxyphenoxy)ethyl)amino)propan-2-ol, briefly called (RS)-9 or 2F109, were studied in vivo in rat serum, urine, faeces, liver, intestine, lungs and kidneys, and in vitro in rat liver microsomes.

Title: Rhabdomyolysis in a patient taking nebivolol.
β Blockers such as propranolol and labetalol are known to induce toxic myopathy because of their partial β2 adrenoceptor agonistic effect. Nebivolol has the highest β1 receptor affinity among β blockers, and it has never been reported to induce rhabdomyolysis until now. We report a patient who developed rhabdomyolysis after changing medication to nebivolol. A 75-year-old woman was admitted to our hospital because of generalized weakness originating 2 weeks before visiting. Approximately 1 month before her admission, her medication was changed from carvedilol 12.5 mg to nebivolol 5 mg. Over this time span, she had no other lifestyle changes causing... (truncated)

Title: Psychiatric side effects of medications prescribed in internal medicine.
Several pharmacological treatments used in internal medicine can induce psychiatric side effects (PSEs) that mimic diagnoses seen in psychiatry. PSEs may occur upon withdrawal or intoxication, and also at usual therapeutic doses. Drugs that may lead to depressive, anxious, or psychotic syndromes include corticosteroids, isotretinoin, levo-dopar mefloquine, interferon-a, and anabolic steroids, as well as some over-the-counter medications. PSEs are often difficult to diagnose and can be very harmful to patients. PSEs are discussed in this review, as well as diagnostic clues to facilitate their identification.

MESH:C100273 - fenclozic acid

Summary:

LLM Prediction Score	0.873 (normalized)
LLM Confidence Score	0.660
Golden Answer (Severity Class)	1.0 (normalized)
Prediction Error	0.127

Retrieved Context:

Title: In vitro exploration of potential mechanisms of toxicity of the human hepatotoxic drug fenclozic acid.
The carboxylic acid NSAID fenclozic acid exhibited an excellent preclinical safety profile and promising clinical efficacy, yet was withdrawn from clinical development in 1971 due to hepatotoxicity observed in clinical trials. A variety of modern in vitro approaches have been used to explore potential underlying mechanisms. Covalent binding studies were undertaken with [(14)C]-fenclozic acid to investigate the possible role of reactive metabolites. Time-dependent covalent binding to protein was observed in NADPH-supplemented liver microsomes, although no metabolites were detected in these incubations or in reactive metabolite trapping experiments. In human hepatocytes, covalent binding was observed at lower levels than in microsomes... (truncated)

Title: Acute liver effects, disposition and metabolic fate of [¹⁴C]-fenclozic acid following oral administration to normal and bile-cannulated male C57BL/6J mice.
The distribution, metabolism, excretion and hepatic effects of the human hepatotoxin fenclozic acid were investigated following single oral doses of 10 mg/kg to normal and bile duct-cannulated male C57BL/6J mice. Whole body autoradiography showed distribution into all tissues except the brain, with radioactivity still detectable in blood, kidney and liver at 72 h post-dose. Mice dosed with [¹⁴C]-fenclozic acid showed acute centrilobular hepatocellular necrosis, but no other regions of the liver were affected. The majority of the [¹⁴C]-fenclozic acid-related material recovered was found in the urine/aqueous cage wash, (49%) whilst a smaller portion (13%) was eliminated via the faeces. Metabolic profiles for... (truncated)

Title: Identification of the reactive metabolites of fenclozic acid in bile duct cannulated rats.
Fenclozic acid (Myalex) was developed by ICI pharmaceuticals in the 1960s for the treatment of rheumatoid arthritis and was a promising compound with a good preclinical safety profile and efficacy. While it did not show adverse hepatic effects in preclinical animal tests or initial studies in man [Chalmers et al. Ann. Rheum. Dis. 1969 , 28 , 595 and Chalmers et al. Ann. Rheum. Dis. 1969 , 28 , 590], it was later withdrawn from clinical development. Hepatotoxicity was observed in humans at daily doses of 400 mg but was not replicated in any of the animal species... (truncated)

Title: The metabolic fate of fenclozic acid in chimeric mice with a humanized liver.
The metabolic fate of the human hepatotoxin fenclozic acid ([2-(4-chlorophenyl)-1,3-thiazol-4-yl]acetic acid) (Myalex) was studied in normal and bile-cannulated chimeric mice with a humanized liver, following oral administration of 10 mg/kg. This in vivo animal model was investigated to assess its utility to study "human" metabolism of fenclozic acid, and in particular to explore the formation of electrophilic reactive metabolites (RMs), potentially unique to humans. Metabolism was extensive, particularly involving the carboxylic acid-containing side chain. Metabolism resulted in the formation of a large number of metabolites and involved biotransformation via both oxidative and conjugative routes. The oxidative metabolites detected included a... (truncated)

Title: A correlation between the in vitro drug toxicity of drugs to cell lines that express human P450s and their propensity to cause liver injury in humans.
Drug toxicity to T-antigen-immortalized human liver epithelial (THLE) cells stably transfected with plasmid vectors that encoded human cytochrome P450s 1A2, 2C9, 2C19, 2D6, or 3A4, or an empty plasmid vector (THLE-Null), was investigated. An automated screening platform, which included 1% dimethyl sulfoxide (DMSO) vehicle, 2.7% bovine serum in the culture medium, and assessed 3-(4,5-dimethylthiazol-2-yl)-5-(3-carboxymethoxyphenyl)-2-(4-sulfophenyl)-2H-tetrazolium reduction, was used to evaluate the cytotoxicity of 103 drugs after 24h. Twenty-two drugs caused cytotoxicity to THLE-Null cells, with EC_{50} ≤ 200 μM; 21 of these drugs (95%) have been reported to cause human liver injury. Eleven drugs exhibited lower EC_{50} values in cells transfected... (truncated)

MESH:D007099 - imipramine

Summary:

LLM Prediction Score	0.502 (normalized)
LLM Confidence Score	0.990
Golden Answer (Severity Class)	0.375 (normalized)
Prediction Error	0.127

Retrieved Context:

Title: Toxic hepatitis and single daily dosage imipramine therapy.
Liver function abnormalities have occasionally been associated with imipramine since the drug was introduced in 1957. The mechanism by which the drug produces hepatotoxicity has not been determined but may involve a direct toxic effect or a hypersensitivity reaction. In this case, a patient receiving 6 mg/kg (300 mg) of imipramine daily developed elevated liver enzymes while the plasma imipramine concentration was found to be in a therapeutic range. This case suggests that single daily dosing of tricyclic antidepressants may be more hazardous to the liver than divided doses.

Title: The role of NLRP3 inflammasome in psychotropic drug-induced hepatotoxicity.
Increased medical application of psychotropic drugs raised attention concerning their toxicological effects. In fact, more than 160 psychotropic drugs including antidepressants and antipsychotics, have been shown to cause liver side effects, but the underlying mechanisms are still poorly understood. Here, we discovered that fluoxetine, a common antidepressant, was specifically sensed by NLRP3 inflammasome, whose subsequent activation resulted in the maturation of caspase-1 and IL-1β, as well as gasdermin D (GSDMD) cleavage, which could be completely abrogated by a selective NLRP3 inhibitor MCC950 or Nlrp3 knockout (Nlrp3^{-/-}). Mechanistically, mitochondrial damage and the subsequent mitochondrial reactive oxygen species (mtROS) accumulation were crucial... (truncated)

Title: Imipramine Accelerates Nonalcoholic Fatty Liver Disease, Renal Impairment, Diabetic Retinopathy, Insulin Resistance, and Urinary Chromium Loss in Obese Mice.
Imipramine is a tricyclic antidepressant that has been approved for treating depression and anxiety in patients and animals and that has relatively mild side effects. However, the mechanisms of imipramine-associated disruption to metabolism and negative hepatic, renal, and retinal effects are not well defined. In this study, we evaluated C57BL6/J mice subjected to a high-fat diet (HFD) to study imipramine's influences on obesity, fatty liver scores, glucose homeostasis, hepatic damage, distribution of chromium, and retinal/renal impairments. Obese mice receiving imipramine treatment had higher body, epididymal fat pad, and liver weights; higher serum triglyceride, aspartate and alanine aminotransferase, creatinine, blood urea... (truncated)

Title: Acute and chronic drug-induced hepatitis.
Adverse drug reactions may mimic almost any kind of liver disease. Acute hepatitis is often due to the formation of reactive metabolites in the liver. Despite several protective mechanisms (epoxide hydrolases, conjugation with glutathione), this formation may lead to predictable toxic hepatitis after hugh overdoses (e.g. paracetamol), or to idiosyncratic toxic hepatitis after therapeutic doses (e.g. isoniazid). Both genetic factors (e.g. constitutive levels of cytochrome P-450 isoenzymes, or defects in protective mechanisms) and acquired factors (e.g. malnutrition, or chronic intake of alcohol or other microsomal enzyme inducers) may explain the unique susceptibility of some patients. Formation of chemically reactive metabolites... (truncated)

Title: Evaluation of the potential for drug-induced liver injury based on in vitro covalent binding to human liver proteins.
Prediction of idiosyncratic drug-induced liver injury (DILI) is difficult, and the underlying mechanisms are not fully understood. However, many drugs causing DILI are considered to form reactive metabolites and covalently bind to cellular macromolecules in the liver. The objective of this study was to clarify whether the risk of idiosyncratic DILI can be estimated by comparing in vitro covalent binding (CB) levels among 12 positive compounds (acetaminophen, alpidem, bromfenac, carbamazepine, diclofenac, flutamide, imipramine, nefazodone, tacrine, ticlopidine, tienilic acid, and troglitazone) for DILI and 12 negative compounds (acetylsalicylic acid, caffeine, dexamethasone, losartan, ibuprofen, paroxetine, pioglitazone, rosiglitazone, sertraline, theophylline, venlafaxine, and zolpidem).... (truncated)

MESH:D004917 - erythromycin

Summary:

LLM Prediction Score	0.498 (normalized)
LLM Confidence Score	0.990
Golden Answer (Severity Class)	0.625 (normalized)
Prediction Error	0.127

Retrieved Context:

Title: [Hepatotoxicity by antibiotics: update in 2008].
Although antibiotics are the most commonly incriminated drugs in instances of hepatotoxicity in medical literature. However, it is mainly due to its wide prescription and the absolute risk of hepatotoxicity related to antibiotic use is thought to be low. Nevertheless, among the different penicillins, amoxicillin-clavulanate is the single leading drug involved in hepatotoxicity in cohorts of patients with drug-induced liver injury (DILI), representing between 12.8% to 14% of the cases. It is the most frequent cause of hospitalization for DILI. The incidence of amoxicillin-clavulanate induced hepatotoxicity has been estimated to be 9.91 per 100,000 users and its clinical presentation varies,... (truncated)

Title: Cholestasis and liver cell damage due to hypersensitivity to erythromycin stearate--recurrence following therapy with erythromycin succinate.
Erythromycin is a frequently used antibiotic in patients with atypical respiratory infection and/or an allergy to penicillin. We report the case of a young woman who developed severe cholestasis and jaundice following treatment with erythromycin stearate. Two years later her general practitioner prescribed erythromycin succinate for pharyngitis. She experienced a severe second episode of jaundice and malaise. Different esters of erythromycin have been introduced to reduce side effects such as allergic reactions to erythromycin. The findings in our patient underline the fact that hypersensitivity is caused by the erythromycin molecule, independent from the type of esterification. Because of these side... (truncated)

Title: Drug interactions and hepatitis produced by some macrolide antibiotics.
Drug interactions involving macrolides have been mainly reported in subjects receiving troleandomycin and in a few receiving erythromycin derivatives. In rats and in humans, troleandomycin, erythromycin and erythromycin derivatives induce microsomal enzymes; the induced isozymes of cytochrome P-450 have a high activity for these macrolides but a poor activity with several other substrates. These isozymes actively demethylate and oxidize these macrolides into nitrosoalkanes which form stable, inactive complexes with the iron of cytochrome P-450. Eventually, the oxidative metabolism of other drugs may be decreased. These effects are marked after administration of troleandomycin, moderate after administration of erythromycin derivatives and absent... (truncated)

Title: ¹H NMR-based serum metabolomics reveals erythromycin-induced liver toxicity in albino Wistar rats.
Erythromycin (ERY) is known to induce hepatic toxicity which mimics other liver diseases. Thus, ERY is often used to produce experimental models of drug-induced liver-toxicity. The serum metabolic profiles can be used to evaluate the liver-toxicity and to further improve the understanding of underlying mechanism.

Title: Liver transplantation for azithromycin-induced severe liver injury.
Drug-induced liver injury is the most common cause of acute liver failure in Western countries by prescription drugs and herbal medications. Liver injury due to azithromycin has rarely been reported. This is a brief report of a patient administered azithromycin and who developed acute liver failure leading to liver transplantation. We report the case of a 68-year-old woman who developed jaundice 1 week after she started taking a azithromycin. On the 3rd day of hospitalization, her hepatic function rapidly deteriorated and level of consciousness decreased to drowsiness. The model for end-stage liver disease score was confirmed to be 33, and... (truncated)

MESH:D012460 - sulfasalazine

Summary:

LLM Prediction Score	0.751 (normalized)
LLM Confidence Score	0.980
Golden Answer (Severity Class)	0.625 (normalized)
Prediction Error	0.126

Retrieved Context:

Title: Adverse effects of sulfasalazine and treatment of ulcerative colitis with mesalazine.
We investigated the adverse effects of sulfasalazine in the treatment of inflammatory bowel disease in Japan. In our first survey, adverse effects were observed in 52 out of 751 patients with ulcerative colitis (6.9%) and in 8 out of 221 patients with Crohn's disease (3.6%); i.e., such effects were observed in 60 out of 972 patients with inflammatory bowel disease. In our second survey, we examined 94 adverse reactions in 64 patients with ulcerative colitis. The main adverse reactions were exanthema, fever, nausea and vomiting, angioedema, and liver damage, in decreasing order of frequency; these reactions accounted for about 80%... (truncated)

Title: Hepatotoxicity after treatment with sulfasalazine.
A 42-year-old man with fever, right lower quadrant abdominal pain, and occasional rectal bleeding was diagnosed as having Crohn's disease. He was started on sulfasalazine (Azulfidine) therapy, and a generalized rash subsequently developed. When laboratory data indicated possible sulfasalazine hepatotoxicity, use of the drug was discontinued. Symptoms continued to worsen, however, and the patient died of fulminant hepatic necrosis. Comparison with similar cases showed that a hypersensitivity reaction was probably responsible for the liver damage, with a latent period that made recognition difficult. Thus, while fulminant hepatic necrosis is rare, this possibility must always be considered by the clinician when... (truncated)

Title: Effects of sulfasalazine on lipid peroxidation and histologic liver damage in a rat model of obstructive jaundice and obstructive jaundice with lipopolysaccharide-induced sepsis.
Sulfasalazine, an inhibitor of cyclooxygenase, 5-lipoxygenase, and nuclear factor κB (NF-κB), has been found to alleviate oxidative damage, proinflammatory cytokine production, bile-duct proliferation, neutrophil infiltration, and fibrosis. Therefore, it may have a potential effect in attenuating lipid peroxidation and histologic liver damage in patients with biliary obstruction and biliary obstruction with sepsis.

Title: Sulfasalazine hepatotoxicity.
Hepatotoxicity has been recently described as a rare complication of sulfasalazine therapy. Two patients with hepatic damage after sulfasalazine treatment are reported. Liver biopsy performed in one patient revealed granulomatous hepatitis. On rechallenge with 5 aminosalicylic acid enema the second patient developed a hypersensitivity reaction without hepatic involvement.

Title: Unusual Case of an Alcoholic with Liver Injury from Sulfasalazine Use.
A 57-year-old male with a history of alcoholism presented to the emergency room with abdominal pain, jaundice, transaminitis, and hyperbilirubinemia. Due to the history of alcoholism, it was initially presumed that the patient had alcoholic hepatitis but further investigation revealed that he was recently started on sulfasalazine for the treatment of rheumatoid arthritis. Upon cessation of the drug, the patient's liver function tests significantly improved over a few days and eventually normalized within weeks. This case was interesting as the patient's history of alcoholism disguised the actual diagnosis. Furthermore, the late presentation of sulfasalazine-induced liver injury is uncommon as it... (truncated)

MESH:D000078305 - zonisamide

Summary:

LLM Prediction Score	0.124 (normalized)
LLM Confidence Score	0.980
Golden Answer (Severity Class)	0.25 (normalized)
Prediction Error	0.126

Retrieved Context:

Title: Thinking beyond the obvious: hepatotoxicity secondary to idiosyncratic depakote toxicity.
Depakote-induced hepatotoxicity has been well established as an adverse effect, and periodic monitoring of drug level is often required. Depakote-induced hepatotoxicity mostly occurs at supratherapeutic drug level. Rarely, an idiosyncratic response is triggered, and hepatotoxicity can occur at the therapeutic drug level mostly in chronic users. Here, we describe a rare case of idiosyncratic depakote-induced hepatotoxicity. A 25-year-old female with non-insulin-dependent diabetes mellitus, hypothyroidism, seizure disorder, and Dandy Walker Syndrome presented with an unwitnessed seizure and altered mental status. The patient's medication list included zonisamide, depakote, and synthroid. She was noted to be lethargic, disoriented, nonverbal, but awake. An arterial... (truncated)

Title: A prospective audit of adjunctive zonisamide in an everyday clinical setting.
This audit examined outcomes for 203 patients prescribed zonisamide (ZNS) for various uncontrolled seizure types at a specialist outpatient service. Forty-two (20.7%) patients achieved 6 months of seizure freedom, and an additional 37 (18.2%) had a 50% seizure reduction for 6 months on a stable ZNS dose. Seizure freedom was more likely in patients with primary generalized (24/61, 39%) than in those with partial-onset (18/141, 12.7%) seizures (P<0.001). Eight patients (5 seizure free) were maintained on ZNS monotherapy. More patients became seizure free with ZNS as monotherapy or first add-on, compared with those in whom ZNS was the second, third,... (truncated)

Title: [Newer antiepileptic drugs].
Ten newer antiepileptic drugs have been developed since 1990s. These drugs have wider therapeutic spectra, fewer side-effects, and lesser drug-to-drug interactions compared with the older typical antiepileptic drugs. Among them, zonisamide was developed in Japan and has been used from 1989. Gabapentin was at length approved in 2006. The other newer antiepileptic drugs are not approved yet in Japan. Felbamate can not be used in Europe because it may induce lethal hepatic toxicity and aplastic anemia. Vigabatrin is not approved in USA because it may induce permanent visual field deficit. The USA guideline for epilepsy treatment recommends that patients with... (truncated)

Title: [Newer antiepileptic drugs].
Ten newer antiepileptic drugs have been developed since 1990s. These drugs have wider therapeutic spectra, fewer side-effects, and lesser drug-to-drug interactions compared with the older typical antiepileptic drugs. Among them, zonisamide was developed in Japan and has been used from 1989. Gabapentin was at length approved in 2006. The other newer antiepileptic drugs are not approved yet in Japan. Felbamate can not be used in Europe because it may induce lethal hepatic toxicity and aplastic anemia. Vigabatrin is not approved in USA because it may induce permanent visual field deficit. The USA guideline for epilepsy treatment recommends that patients with... (truncated)

Title: Zonisamide Therapy Reduces Metabolic Consequences and Diminishes Nonalcoholic Fatty Liver Disease in Patients with Epilepsy.
Antiepileptic drugs that can reduce aberrant metabolism are beneficial for patients. Zonisamide (ZNS) is a chemical with antiepileptic and antioxidant activities. Here, we evaluate the efficacy of ZNS therapy on reducing obesity and decreasing risks of vascular diseases and hepatic steatosis. Clinical and metabolic indicators including body weight, body mass index (BMI), serum lipid profiles, glycated hemoglobin (HbA1c), homocysteine, and an inflammatory marker, high-sensitivity C-reactive protein (hs-CRP), were assessed at baseline and at the end of 12 and 24 weeks of treatment. Nonalcoholic fatty liver disease was evaluated using the hepatic steatosis index (HSI). A body weight reduction of ≥5%... (truncated)

MESH:D006919 - hydroxyzine

Summary:

LLM Prediction Score	0.125 (normalized)
LLM Confidence Score	0.990
Golden Answer (Severity Class)	0.0 (normalized)
Prediction Error	0.125

Retrieved Context:

Title: Safety of Cyproheptadine, an Orexigenic Drug. Analysis of the French National Pharmacovigilance Data-Base and Systematic Review.
Objectives: Cyproheptadine is a first-generation H1-antihistamine drug first that was distributed in the 1960s. While its orexigenic effect was observed early, cyproheptadine is not yet authorized for this indication in all countries today. There is an increasing medical interest and demand for the orexigenic effect of cyproheptadine, especially in children with poor appetite. As cyproheptadine might be evaluated in future clinical trials, we wanted to assess its safety profile. Methods: Using the French national pharmacovigilance database, we retrospectively analyzed all pediatric and adult reports of adverse effects of cyproheptadine recorded since its first distribution in France. Next, we performed a... (truncated)

Title: Embracing the Dark Side: Computational Approaches to Unveil the Functionality of Genes Lacking Biological Annotation in Drug-Induced Liver Injury.
In toxicogenomics, functional annotation is an important step to gain additional insights into genes with aberrant expression that drive pathophysiological mechanisms. Nevertheless, there exists a gap on annotation of these genes which often hampers the interpretation of results and limits their applicability in translational medicine. In this study, we evaluated the coverage of functional annotations of differentially expressed genes (DEGs) induced by 10 selected compounds from the TG-GATEs database identified as high- or no-risk in causing drug-induced liver injury (most-DILI or no-DILI, respectively) using <i>in vitro</i> human data. Functional roles of DEGs not present in the most common biological annotation... (truncated)

Title: Case Report of Isoniazid-Related Acute Liver Failure Requiring Liver Transplantation.
The prevalence of latent tuberculosis infection (LTBI) in the United States in 2011 and 2012 was estimated at 4.4⁻4.8%. As of 2015, 12.4 million people still possessed LTBI. Isoniazid, or isonicotinic acid hydrazine (INH), is the most commonly used medication among varying regimens that exist in the treatment of tuberculosis and LTBI. INH-related hepatotoxicity is a well-known adverse effect of its use, often causing asymptomatic elevations in serum aminotransferase levels. These elevations are typically transient and reversible, but can cause acute, clinically-significant liver injury in rare cases. We report a case of a 67-year old male who developed subacute hepatic... (truncated)

Title: Identifying olanzapine induced liver injury in the setting of acute hepatitis C: A case report.
Olanzapine is linked to asymptomatic, transient elevations of liver aminotransferases but is historically thought to rarely cause significant hepatotoxicity. Underlying liver disease is a risk factor for drug-induced liver injury and may complicate the differential diagnosis of acute transaminitis in patients taking medications associated with hepatotoxicity. Ms L presented with 2 months of new psychotic symptoms resulting in hospitalizations. Although psychosis previously improved with haloperidol, she reported symptoms concerning for akathisia. Restlessness improved and psychotic symptoms resolved after initiation of olanzapine. Concurrently, her alanine aminotransferase (ALT) was elevated, prompting further workup and new diagnosis of acute hepatitis C. Over the... (truncated)

Title: Cholestatic jaundice as a result of combination designer supplement ingestion.
To report a case of cholestatic jaundice as a result of combination herbal and designer supplement use.

MESH:D011899 - ranitidine

Summary:

LLM Prediction Score	0.500 (normalized)
LLM Confidence Score	0.990
Golden Answer (Severity Class)	0.625 (normalized)
Prediction Error	0.125

Retrieved Context:

Title: Intrahepatic cholestatic jaundice related to administration of ranitidine. A case report with histologic and ultramicroscopic study.
Ranitidine may cause liver injuries ranging from transient, subclinical serum transaminases increase every 100-1,000 treated patients to cholestatic hepatitis in less than 1/100,000. Other H2-receptor antagonists are more dangerous: 11 toxic hepatitis cases have been reported as adverse effect after 1 year of marketed ebrotidine. A 75-year-old male with ischemic cardiopathy history was started on an 8 days treatment of oral ranitidine due to pirosis, without any other changes of therapy; 48 h after drug withdrawal, light-coloured stools, dark urine and icteric scleras developed. On hospital admission, 10 days later, physical examination showed slight hepatomegaly and severe jaundice with skin... (truncated)

Title: Side effects of ranitidine.
Ranitidine was first marketed in 1981; since then many patients have been treated such that much experience has been accumulated on the safety of this histamine H2-receptor antagonist in the treatment of gastroduodenal disease. A wide array of ranitidine-associated side effects has been described, but infrequently. As so much information is now available, the aim of this review is to assess the weight of evidence for a causal link between ranitidine and the reported side effects. Overall, ranitidine is well tolerated. The incidence of general side effects at less than 2% is very similar to placebo. Headaches, tiredness, dizziness and... (truncated)

Title: Ranitidine-related Stevens-Johnson syndrome in patients with severe liver diseases: a report of two cases.
Ranitidine is widely used and well tolerated. Ranitidine-related skin eruption is rare and usually mild. We presented two patients who developed Stevens-Johnson syndrome after ranitidine therapy. Before therapy, both patients had severe liver disease and jaundice. The first was a case of ketoconazole-induced hepatitis, and the second was a case of hepatitis C-related liver cirrhosis with hepatocellular carcinoma. Ranitidine was the most likely drug responsible for causing Stevens-Johnson syndrome in these two patients.

Title: Nephrotoxicity and hepatotoxicity of histamine H2 receptor antagonists.
The extensive use of selective histamine H2 receptor antagonists provides a unique opportunity to describe very rare adverse drug reactions. Although mild elevation of serum creatinine level following the administration of cimetidine is relatively common, acute interstitial nephritis (AIN) is a rare hypersensitivity reaction. There have been 25 published reports of AIN associated with H2 antagonist therapy and we also identified 16 cases from the Australian Adverse Drug Reaction Advisory Committee (ADRAC) database. AIN was reported most commonly following cimetidine administration. AIN was supported by renal biopsy in 28 patients and by rechallenge in 6. H2 antagonist-induced AIN was more... (truncated)

Title: Potential use of cimetidine for treatment of acetaminophen overdose.
Acetaminophen, a drug frequently taken in intentional and accidental overdose, causes liver toxicity when concentration of the cytochrome P-450-derived metabolite exceeds the metabolic capacity of available glutathione. Present treatment of acetaminophen overdose involves oral N-acetylcysteine (NAC), which enhances liver glutathione synthesis. An alternative or additive approach to therapy would be to inhibit the formation of the toxic metabolite by inhibiting the cytochrome P-450 system. The H2-receptor antagonist cimetidine inhibits the cytochrome P-450 system, does not interfere with the administration or function of NAC, and therefore affords additive protection. Also, it has little effect on the nontoxic routes of elimination of... (truncated)

MESH:D000077261 - carvedilol

Summary:

LLM Prediction Score	0.500 (normalized)
LLM Confidence Score	0.990
Golden Answer (Severity Class)	0.375 (normalized)
Prediction Error	0.125

Retrieved Context:

Title: Carvedilol-Induced Liver Injury, a Rare Cause of Mixed Hepatitis: A Clinical Case.
Drug-induced liver injury is an increasingly prevalent consequence of the diversification of available therapeutic weapons, mostly idiosyncratic and with several possible mechanisms and patterns of specific damage for each drug. Carvedilol, a widely used non-selective alpha and beta blocker leads, in very rare cases, to injury of the bile ducts by toxic metabolites, resulting in a mixed-pattern hepatitis with possible progression to chronic cholestatic syndrome and cirrhosis. The authors report the second known case of this important toxicity.

Title: Curcumin and <i>α</i>/<i>β</i>-Adrenergic Antagonists Cotreatment Reverse Liver Cirrhosis in Hamsters: Participation of Nrf-2 and NF-<i>κ</i>B.
Liver cirrhosis is the result of an uncontrolled fibrogenetic process, due to the activation and subsequent differentiation into myofibroblasts of the hepatic stellate cells (HSC). It is known that HSC express adrenoreceptors (AR), and the use of AR antagonists protects experimental animals from cirrhosis. However, several studies suggest that the toxicity generated by metabolism of these antagonists would hinder its use in cirrhotic patients. In addition, liver fibrosis may be associated with a decrease of the antioxidant response of the nuclear factor erythroid 2-related factor 2 (Nrf-2) and the overregulation of the proinflammatory pathway of nuclear factor kappa B (NF-<i>κ</i>B).... (truncated)

Title: Hepatotoxicity associated with carvedilol.
To report a severe episode of pruritus and elevated serum transaminases in a patient who was receiving carvedilol.

Title: Protective effect of carvedilol alone and coadministered with diltiazem and prednisolone on doxorubicin and 5-fluorouracil-induced hepatotoxicity and nephrotoxicity in rats.
This study investigated the protective effects of carvedilol alone and coadministered with prednisolone and diltiazem on doxorubicin (DOX) and 5-fluorouracil (5-FU)-induced toxicity. Each of 2 pools of 70 female rats were randomly allotted into 10 groups of 7 animals each and treated as follows: Group 1: normal saline (10 mL/kg); Group 2: normal saline and DOX (40 mg/kg)/5-FU (20 mg/kg) alone; Group 3: gallic acid (200 mg/kg) and DOX/5-FU; Group 4: carvedilol (0.075 mg/kg) and DOX/5-FU; Group 5: carvedilol (0.15 mg/kg) and DOX/5-FU; Group 6: carvedilol (0.30 mg/kg) and DOX/5-FU; Group 7: diltiazem (3.43 mg/kg) and DOX/5-FU; Group 8: diltiazem... (truncated)

Title: Carvedilol Improves Inflammatory Response, Oxidative Stress and Fibrosis in the Alcohol-Induced Liver Injury in Rats by Regulating Kuppfer Cells and Hepatic Stellate Cells.
To evaluate the anti-inflammatory, anti-oxidant and antifibrotic effects of carvedilol (CARV) in rats with ethanol-induced liver injury.

MESH:D011405 - propafenone

Summary:

LLM Prediction Score	0.500 (normalized)
LLM Confidence Score	0.960
Golden Answer (Severity Class)	0.375 (normalized)
Prediction Error	0.125

Retrieved Context:

Title: Propafenone-related cholestatic hepatitis in an elderly patient.
Hepatic toxicity caused by propafenone is extremely rare. We here describe a case of acute cholestasis secondary to propafenone treatment in an elderly male with no history of preexistent liver disease. The clinical picture and time course of the symptoms seem to be compatible with a direct toxic reaction. Age-related delays in hepatic drug metabolism should explain the hazardous increase in the bioavailability of this drug. Despite the rarity of this complication, propafenone should be considered as a potential cause of drug-induced cholestasis in elderly patients. The literature on the hepatotoxicity of propafenone is also reviewed.

Title: Propafenone hepatotoxicity: report of a new case and review of the literature.
Propafenone is a class Ic antiarrhythmic drug. It is a beta-adrenergic blocker that causes bradycardia and bronchospasm. It is metabolized primarily in the liver. Its bioavailability and plasma concentration differ among patients under long-term therapy. They are genetically determined by the hepatic cytochrome P-450 2D6. Hepatic toxicity is highly uncommon. To date, only eight patients were reported in the reviewed world literature. In this article, one new case will be reported emphasizing the importance of medication history taking in patients presenting with new-onset liver enzymes abnormalities.

Title: Propafenone hepatotoxicity: report of two new cases.
Two patients developed acute cholestatic hepatitis during treatment with propafenone. Viral infections, alcohol abuse, hepatotoxicity by other drugs, and biliary obstruction were excluded as causes. In one patient, liver biopsy showed changes consistent with a drug-associated injury. Another patient had autoimmune antibodies (ANA) in the serum. Following propafenone withdrawal, the clinical and biochemical profiles of both patients improved. Hepatic toxicity from the antiarrhythmic drug propafenone is highly uncommon. Moreover, the drug produces hepatocellular injury by an unknown mechanism. Most of the seven cases reported here had acute cholestatic hepatitis after a latency period of two to four weeks.

Title: [Hepatic toxicity of propafenone: a case description].
A case of acute liver injury associated with the use of the antiarrhythmic drug propafenone in a 62-year-old woman undergoing clinical observation for recurrent atrial fibrillation is reported. Propafenone hydrochloride, a class 1C antiarrhythmic drug widely used in the clinical practice for the treatment of supraventricular and ventricular arrhythmias, rarely (0.1-0.2% of incidence) causes liver injury characterized by a rise in hepatic cell enzymes or cholestatic enzymes or both. Within 2 months of the discontinuation of therapy the liver function tests return to normal, therefore there are no known fatalities secondary to propafenone liver injury including fulminant hepatitis and death.... (truncated)

Title: [Acute cholestatic hepatitis caused by propafenone. Report of a case and review of the literature].
A case of acute cholestatic hepatitis associated with use of propafenone is reported. Hepatitis developed 3 weeks after the beginning of administration of this drug. The close time relationship between the administration of the antiarrhythmic drug and the acute onset of the liver damage, the exclusion of hepatobiliary disease and the rapid normalisation of biochemical parameters following withdrawal of the drug strongly suggest that propafenone was involved in the pathogenesis of this syndrome. Meticulous taking of patient history and clinical assessment are mandatory for the early identification of drug-induced hepatotoxicity and avoidance of more severe types of reactions, such as... (truncated)

MESH:D007520 - isocarboxazid

Summary:

LLM Prediction Score	0.500 (normalized)
LLM Confidence Score	0.950
Golden Answer (Severity Class)	0.625 (normalized)
Prediction Error	0.125

Retrieved Context:

Title: Drug-induced liver injury and microsomal arylamidase activity in needle biopsy of human liver.
The relation between the activities of arylamidase (one of the non-specific carboxylesterases) located exclusively in smooth endoplasmic reticulum (SER), and microscopical changes of the hepatocytes, was studied in three patients treated with phenobarbital and twenty-five patients injured by various other drugs. A micro-method was used for the determination of the enzyme activity in small pieces of liver (5-15 mg) obtained by needle biopsy. The arylamidase activity in seven normal livers was 0.43 +/- 0.05 (SD) nmol benzylhydrazine/micron protein/hr formed from isocarboxazid. The mean value in the livers treated with phenobarbital was statistically different from the value before phenobarbital treatment (P... (truncated)

Title: Predicting therapeutic drugs for hepatocellular carcinoma based on tissue-specific pathways.
Hepatocellular carcinoma (HCC) is a significant health problem worldwide with poor prognosis. Drug repositioning represents a profitable strategy to accelerate drug discovery in the treatment of HCC. In this study, we developed a new approach for predicting therapeutic drugs for HCC based on tissue-specific pathways and identified three newly predicted drugs that are likely to be therapeutic drugs for the treatment of HCC. We validated these predicted drugs by analyzing their overlapping drug indications reported in PubMed literature. By using the cancer cell line data in the database, we constructed a Connectivity Map (CMap) profile similarity analysis and KEGG enrichment... (truncated)

Title: Microbiota-Host-Irinotecan Axis: A New Insight Toward Irinotecan Chemotherapy.
Irinotecan (CPT11) and its active metabolite ethyl-10-hydroxy-camptothecin (SN38) are broad-spectrum cytotoxic anticancer agents. Both cause cell death in rapidly dividing cells (e.g., cancer cells, epithelial cells, hematopoietic cells) and commensal bacteria. Therefore, CPT11 can induce a series of toxic side-effects, of which the most conspicuous is gastrointestinal toxicity (nausea, vomiting, diarrhea). Studies have shown that the gut microbiota modulates the host response to chemotherapeutic drugs. Targeting the gut microbiota influences the efficacy and toxicity of CPT11 chemotherapy through three key mechanisms: microbial ecocline, catalysis of microbial enzymes, and immunoregulation. This review summarizes and explores how the gut microbiota participates in... (truncated)

Title: A neglected modality in psychiatric treatment--the monoamine oxidase inhibitors.
The monoamine oxidase inhibitors are at present being used relatively infrequently in my opinion because of reports of severe and dangerous side effects such as toxic hepatocellular damage and hypertensive crises and also on account of several studies which have not given a very encouraging picture regarding the efficacy of this group of drugs. The purpose of this article is to demonstrate that this group of antidepressant drugs is very useful when the proper indications for their employment are observed and are relatively safe provided that appropriate precautions such as the avoidance of cheese and other foods high in tyramine... (truncated)

Title: Therapeutic targeting of CPT-11 induced diarrhea: a case for prophylaxis.
CPT-11 (irinotecan), a DNA topoisomerase I inhibitor is one of the main treatments for colorectal cancer. The main dose limiting toxicities are neutropenia and late onset diarrhea. Though neutropenia is manageable, CPT-11 induced diarrhea is frequently severe, resulting in hospitalizations, dose reductions or omissions leading to ineffective treatment administration. Many potential agents have been tested in preclinical and clinical studies to prevent or ameliorate CPT-11 induced late onset diarrhea. It is predicted that prophylaxis of CPT-11 induced diarrhea will reduce sub-therapeutic dosing as well as hospitalizations and will eventually lead to dose escalations resulting in better response rates. This article... (truncated)

MESH:D008801 - mexiletine

Summary:

LLM Prediction Score	0.500 (normalized)
LLM Confidence Score	0.950
Golden Answer (Severity Class)	0.375 (normalized)
Prediction Error	0.125

Retrieved Context:

Title: Mexiletine-induced severe skin eruption, fever, eosinophilia, atypical lymphocytosis, and liver dysfunction.
A 64-year-old man developed a severe generalized pruritic morbilliform skin eruption, fever, eosinophilia, atypical lymphocytosis, and liver dysfunction 30 days after ingestion of mexiletine, a sodium channel blocker, prescribed to treat postherpetic neuralgia. Following intravenous dexamethasone, body temperature normalized the next day. However, the skin eruption did not disappear completely for 4 weeks. The patch test was positive for mexiletine. Clinical features and the result of patch test indicated that the patient developed hypersensitivity syndrome, a severe adverse cutaneous drug reaction, caused by mexiletine. We propose that mexiletine be added to the list of drugs that can cause severe adverse... (truncated)

Title: Flos Carthami Exerts Hepatoprotective Action in a Rat Model of Alcoholic Liver Injury via Modulating the Metabolomics Profile.
This study was intended to identify the shifts in the metabolomics profile of the hepatic tissue damaged by alcohol consumption and verify the potential restorative action of flos carthami (the flowers of <i>Carthamus tinctorius</i>, FC) in the protection of alcohol-induced injury by attenuating the level of identified metabolites. Rats were treated with FC and subsequently subjected to alcohol administration. The serum samples were subjected to liquid chromatography-mass spectrometry (LC-MS)-based metabolomics followed by statistical and bioinformatics analyses. The clustering of the samples showed an obvious separation in the principal component analysis (PCA) plot, and the scores plot of the orthogonal partial... (truncated)

Title: Drug-induced hypersensitivity syndrome due to mexiletine hydrochloride associated with reactivation of human herpesvirus 7.
It has been suggested that reactivation of human herpesvirus 6 (HHV-6) infection may be involved in the pathogenesis of drug-induced hypersensitivity syndrome. We report a 45-year-old Japanese man who developed a generalized papuloerythematous rash, fever, hepatitis, lymphadenopathy and lymphocytosis with an increased number of atypical lymphocytes. He was diagnosed with DIHS due to mexiletine hydrochloride based on laboratory data, results of a patch test and the clinical course of his complaint, and was treated with systemic steroids. In order to determine whether HHV-6 or -7 was associated with the patient's disease, serological assays and PCR were carried out. Significant increases... (truncated)

Title: [Blood level of mexiletine in liver-damaged patients].
No abstract available.

Title: Population pharmacokinetic parameters in patients treated with oral mexiletine.
A new data analysis approach, NON-MEM, proposed by Sheiner and Beal, has been employed to estimate the population pharmacokinetic parameters of oral mexiletine in patients treated for arrhythmias. 452 serum concentration measurements in 58 patients were available for analysis. 27 patients had congestive heart failure and 8 had abnormal liver function tests at the time of the study. The population averages of the pharmacokinetic parameters and their interindividual variability were: oral total body clearance (Cl) 0.38 l/h/kg +/- 43% (C.V.), apparent volume of distribution (Vd) 5.3 l/kg +/- 40%, absorption rate constant 3.1 h-1 +/- 205%, absorption time-lag 0.3 h.... (truncated)

MESH:C033563 - haloperidol decanoate

Summary:

LLM Prediction Score	0.250 (normalized)
LLM Confidence Score	0.950
Golden Answer (Severity Class)	0.375 (normalized)
Prediction Error	0.125

Retrieved Context:

Title: Identifying olanzapine induced liver injury in the setting of acute hepatitis C: A case report.
Olanzapine is linked to asymptomatic, transient elevations of liver aminotransferases but is historically thought to rarely cause significant hepatotoxicity. Underlying liver disease is a risk factor for drug-induced liver injury and may complicate the differential diagnosis of acute transaminitis in patients taking medications associated with hepatotoxicity. Ms L presented with 2 months of new psychotic symptoms resulting in hospitalizations. Although psychosis previously improved with haloperidol, she reported symptoms concerning for akathisia. Restlessness improved and psychotic symptoms resolved after initiation of olanzapine. Concurrently, her alanine aminotransferase (ALT) was elevated, prompting further workup and new diagnosis of acute hepatitis C. Over the... (truncated)

Title: Effects of various drugs on alcohol-induced oxidative stress in the liver.
The major aim of this work was to investigate how alcohol-induced oxidative stress in combined chemotherapy changes the metabolic function of the liver in experimental animals. This research was conducted to establish how bromocriptine, haloperidol and azithromycin, applied to the experimental model, affected the antioxidative status of the liver. The following parameters were determined: reduced glutathione, activities of glutathione peroxidase, glutathione reductase, peroxidase, catalase, xanthine oxidase and lipid peroxidation intensity. Alanine transaminase was measured in serum. Alcohol stress (AO group) reduced glutathione and the activity of xanthine oxidase and glutathione peroxidase, but increased catalase and alanine transaminase activity. The best... (truncated)

Title: Pharmacokinetic Characteristics of Long-Acting Injectable Antipsychotics for Schizophrenia: An Overview.
The availability of long-acting injectable (LAI) antipsychotics for the treatment of schizophrenia provides clinicians with options that deliver continuous drug exposure and may improve adherence compared with daily oral antipsychotics. However, all LAI antipsychotics have unique formulations and pharmacokinetic characteristics that have implications for medication selection, administration interval, and injection site. This review outlines key differences in drug formulations and pharmacokinetics among LAI antipsychotics. A systematic search of the PubMed database was conducted to identify physical and formulation properties and pharmacokinetic data of commercially available LAI antipsychotics, including flupentixol decanoate, fluphenazine decanoate, haloperidol decanoate, zuclopenthixol decanoate, aripiprazole monohydrate, aripiprazole lauroxil,... (truncated)

Title: Acute Necrotizing Pancreatitis following Long-Term Antipsychotic Use.
<i>Introduction</i>. Psychiatrists commonly use antipsychotic medications in the treatment of psychotic and mood disorders. A rare but known side effect of atypical antipsychotics is acute pancreatitis. Most cases of antipsychotic-induced pancreatitis occur within six months of initiation. The mechanism believed to cause this reaction is hypertriglyceridemia. Here, we present a unique case of antipsychotic-induced pancreatitis that deviates from previous cases in the time to onset of the pancreatitis and the mechanism of presentation. <i>Case Presentation</i>. We present a case of a patient with treatment-resistant schizophrenia managed for over a decade on olanzapine and haloperidol. Twelve years after stabilization on this... (truncated)

Title: A 6-Month Prospective Trial of a Personalized Behavioral Intervention + Long-Acting Injectable Antipsychotic in Individuals With Schizophrenia at Risk of Treatment Nonadherence and Homelessness.
Long-acting injectable antipsychotics (LAI) can optimize adherence for high-risk serious mental illness (SMI). This customized adherence-enhancement approach delivered by social worker interventionists was combined with LAI (CAE-L) of paliperidone palmitate for homeless, poorly adherent individuals with SMI.

MESH:D009640 - norethindrone

Summary:

LLM Prediction Score	0.500 (normalized)
LLM Confidence Score	0.980
Golden Answer (Severity Class)	0.375 (normalized)
Prediction Error	0.125

Retrieved Context:

Title: A new and practical oral contraceptive agent: norethindrone with mestranol.
The usefulness of a new oral contraceptive compound containing norethindrone 5 mg. and mestranol 0.075 mg. (Ortho-Novum) was studied. One hundred and seventeen private patients used this preparation for a total of 998 menstrual cycles. There were no pregnancies. Side effects were minimal and caused few (11 patients) to discontinue use. The most significant of these was weight gain and a premenstrual tension syndrome including nausea, breakthrough bleeding and skin effects. Other symptoms were minor and easily controlled. Instruction in use of the preparation is easy. Patient acceptance is excellent. Possible masculinizing effects, liver damage, or harmful effects on the... (truncated)

Title: [Experience in the treatment of metastatic breast carcinoma with norethisterone acetate in a clinical study].
Twenty-nine patients with progressive advanced carcinoma of the breast were treated with 40 mg/day norethisterone acetate orally in this clinical trial. 27 of 29 patients were evaluable for response to the drug. One patient achieved a partial response (duration 2 months), 6 had a stable disease (median duration 7.6 months) and 20 developed a progressive disease on treatment. There was a high rate of liver toxicity in 23 patients [grade 3 and 4 according to WHO (1)]. The literature on norethisterone acetate is reviewed and compared with present results. The role of progestogenic agents in treatment of advanced mammary carcinoma... (truncated)

Title: Rare case of norethisterone-induced hepatitis: A case report.
We report a case of probable norethisterone-related liver injury, manifesting as a significant rise in liver transaminases in a 62-year-old woman. Upon discontinuation of norethisterone, liver transaminases decreased to normal level within two weeks. Knowledge of rare adverse effects of drugs such as norethisterone is necessary for rapid identification and management, especially in patients with risk factors such as non-alcoholic liver disease and obesity.

Title: Drug-Induced Liver Injury: Highlights of the Recent Literature.
Drug-induced liver injury (DILI), herbal-induced liver injury, and herbal and dietary supplement (HDS)-induced liver injury are an important aspect of drug safety. Knowledge regarding responsible drugs, mechanisms, risk factors, and the diagnostic tools to detect liver injury have continued to grow in the past year. This review highlights what we considered the most significant publications from among more than 1800 articles relating to liver injury from medications, herbal products, and dietary supplements in 2017 and 2018. The US Drug-Induced Liver Injury Network (DILIN) prospective study highlighted several areas of ongoing study, including the potential utility of human leukocyte antigens and... (truncated)

Title: Seven cases of jaundice in women taking an oral contraceptive, anovlar.
No abstract available.

MESH:D019821 - simvastatin

Summary:

LLM Prediction Score	0.500 (normalized)
LLM Confidence Score	0.990
Golden Answer (Severity Class)	0.375 (normalized)
Prediction Error	0.125

Retrieved Context:

Title: Flutamide-induced hepatotoxicity with possible potentiation by simvastatin.
We report a 65 years old male who presented with jaundice in February 2000. He was operated for prostate carcinoma and started on flutamide in May 1999. He developed anorexia in mid January and frank jaundice by end February. LFT showed cholestatic picture. Liver biopsy showed cholestasis. Flutamide was stopped and patient made slow recovery. Possible additional hepatotoxicity of simvastatin is discussed.

Title: [Acute cholestatic hepatitis caused by simvastatine in a 67-year-old patient].
The following work presents the case of undesired during side effect (toxic hepatocellular damage) by Simvastatine (Cardin). The main syptoms were jaundice and pruritus. Conclusions draw the attention to the role of history taking in the diagnosis of the described disease, as well as the importance of regular patient checkups whoin those use statines.

Title: An effective assessment of simvastatin-induced toxicity with NMR-based metabonomics approach.
Simvastatin, which is used to control elevated cholesterol levels, is one of the most widely prescribed drugs. However, a daily excessive dose can induce drug-toxicity, especially in muscle and liver. Current markers for toxicity reflect mostly the late stages of tissue damage; thus, more efficient methods of toxicity evaluation are desired.

Title: Hepatotoxicity Induced by Fluvastatin: A Reversible Acute Cholestatic Liver Injury.
BACKGROUND Fluvastatin, a commonly prescribed statin, is indicated for treatment of hypercholesterolemia in persons at high risk for coronary, cerebrovascular, and peripheral artery disease. However, there have been rare reports of liver injury or renal failure associated with use of fluvastatin. CASE REPORT We describe the case of a 69-year-old Saudi man with a medical history of diabetes mellitus and hypercholesterolemia for 2 years, on metformin, gliclazide modified release, daily aspirin, and simvastatin. Fluvastatin 40 mg daily was administered instead of simvastatin for 7 weeks before the patient was admitted to the hospital with fatigue, weakness, abdominal pain, loss of... (truncated)

Title: Effects of simvastatin 40 mg daily on muscle and liver adverse effects in a 5-year randomized placebo-controlled trial in 20,536 high-risk people.
Simvastatin reduces cardiovascular mortality and morbidity but, as with other HMG-CoA reductase inhibitors, can cause significant muscle toxicity and has been associated with elevations of liver transaminases.

MESH:D009529 - nicardipine

Summary:

LLM Prediction Score	0.500 (normalized)
LLM Confidence Score	0.980
Golden Answer (Severity Class)	0.375 (normalized)
Prediction Error	0.125

Retrieved Context:

Title: Nicardipine-induced acute hepatitis in an intensive care unit patient.
Drug-related hepatotoxicity is now the leading cause of acute liver failure in the United States, especially among patients who have no prior liver disease. Nicardipine is the only IV calcium channel blocker available for the short-term treatment of hypertension with a considerably good safety profile. We report a case of nicardipine-induced hepatitis. A patient with history of hypertension was admitted because of right middle cerebral artery infarction. Computed tomography of the brain showed evolving stroke. The patient went for cerebral angiography and stent placement, and during the procedure he had cerebral hemorrhage. He was transferred to neurosurgery. After surgery, he... (truncated)

Title: Nilvadipine. A review of its pharmacodynamic and pharmacokinetic properties, therapeutic use in hypertension and potential in cerebrovascular disease and angina.
Nilvadipine, a calcium antagonist of the dihydropyridine class, selectively blocks calcium channels in vascular smooth muscle. Compared with nifedipine, the prototype of the dihydropyridines, nilvadipine has a longer duration of action. The antihypertensive efficacy of nilvadipine appears to be comparable with that of nicardipine and nitrendipine, enalapril and captopril and hydrochlorothiazide/triamterene, although further clinical experience is required to establish the claimed advantages nilvadipine may have over the other dihydropyridine derivatives currently used to treat hypertension. Preliminary studies suggest that nilvadipine may also be useful in the treatment of patients with stable exertional or variant angina. Studies conducted in Japan indicate... (truncated)

Title: Effect of nicardipine infusion on the release of glutathione S-transferase following halothane anaesthesia.
To assess the possible protective effect of calcium channel blockade on hepatic function after halothane anaesthesia, 80 patients were allocated randomly to receive an i.v. infusion of either nicardipine or normal saline. Plasma concentration of glutathione S-transferase B1 subunits (GST) was measured as a sensitive index of hepatic damage. Data from 53 patients were analysed. Plasma GST concentration increased significantly at 3 and 6 h after induction of anaesthesia in the placebo group (P less than 0.01), and at 3 h (P less than 0.01) and 6 h (P less than 0.05) in the nicardipine group. The administration of nicardipine... (truncated)

Title: Intravenous nicardipine and labetalol use in hypertensive patients with signs or symptoms suggestive of end-organ damage in the emergency department: a subgroup analysis of the CLUE trial.
To compare the efficacy of Food and Drug Administration recommended dosing of nicardipine versus labetalol for the management of hypertensive patients with signs and/or symptoms (S/S) suggestive of end-organ damage (EOD).

Title: [Effects of nicardipine on the functional state of the liver in patients with ischemic heart disease].
Hepatic function was studied in 31 patients with coronary heart disease before and after treatment with nicardipine. Clinical and biochemical findings (enhanced gamma-glutamate transferase and high cholesterol, hepatobiliary syndrome) provide evidence for hepatic function disturbance associated with coronary heart disease. Nicardipine produced a good subjective response, the attenuation of hepatobiliary syndrome. Biochemical indices did not change.

MESH:D000078785 - mirtazapine

Summary:

LLM Prediction Score	0.500 (normalized)
LLM Confidence Score	0.990
Golden Answer (Severity Class)	0.375 (normalized)
Prediction Error	0.125

Retrieved Context:

Title: Mirtazapine-induced hepatotoxicity.
We report two cases of hepatotoxicity induced by mirtazapine. Two women treated for depression with mirtazapine developed severe hepatotoxicity because of the drug. In the first case, mirtazapine was prescribed for 3 years and the patient developed derangement in liver function with prolonged jaundice, which improved after the withdrawal of mirtazapine. The use of mirtazapine in the second case was associated with prolonged jaundice, which only recovered 3 months after the withdrawal of mirtazapine. The cause of mirtazapine-induced liver toxicity was unknown, and to our knowledge, these are the first reported cases of mirtazapine-induced prolonged jaundice.

Title: Acute hepatitis after starting pinaverium bromide in a patient taking mirtazapine.
A 56-year-old man presented with chronic abdominal pain. He had been evaluated extensively in the recent past undergoing upper gastrointestinal endoscopy, colonoscopy and CT scan of the abdomen with normal results. The provisional diagnosis of irritable bowel syndrome was performed and pinaverium bromide was started. The patient had pre-existing hypertension, a major depressive disorder and gastro-oesophageal reflux disease. He had been taking nebivolol and pantoprazole for several years and mirtazapine for the last 1 year. The patient developed nausea, vomiting and anorexia after 5 days of starting pinaverium bromide. Investigations revealed marked elevation of liver enzymes and bilirubin. He was... (truncated)

Title: Liver injury associated with antidepressants.
Antidepressants are commonly prescribed and used in the management of depression, anxiety disorders, and other psychiatric illnesses. Antidepressants used in therapeutic dosing ranges are associated with causing several adverse drug reactions including hepatotoxicity. Paroxetine, fluoxetine, fluvoxamine, citalopram, mirtazapine and venlafaxine are associated with reversible liver injury upon discontinuation of the agent. Patient cases of hepatotoxicity involving the use of nefazodone, trazodone, duloxetine, bupropion, and sertraline are linked to causing death in its users. Due to the idiosyncratic nature of hepatotoxicity, monitoring of liver function tests and immediate discontinuation upon abnormal lab findings or signs and symptoms of liver dysfunction are... (truncated)

Title: The pharmacovigilance of mirtazapine: results of a prescription event monitoring study on 13554 patients in England.
Mirtazpine is the first noradrenaline and serotonin specific antidepressant. We monitored the safety of mirtazapine as reported in primary practice in England. The exposure data were provided by monitoring the dispensed prescriptions issued between September 1997 and February 1999. Questionnaires sent to GPs provided outcome data. Drowsiness/sedation and malaise/lassitude were the most frequent ADRs (116, 71 respectively) and had the highest incidence density (per 1000 patient-months) in the first month of treatment (58.1, 27.8 respectively). Agitation (73), aggression (70), rash (20), hallucinations (13) and abnormal dreams (31 were unlabelled AES while abnormal liver function tests (12), syncope (8), abnormal behaviour... (truncated)

Title: Addressing the Side Effects of Contemporary Antidepressant Drugs: A Comprehensive Review.
Randomized trials have shown that selective serotonin reuptake inhibitors (SSRIs) and serotonin-norepinephrine reuptake inhibitors (SNRIs) have better safety profiles than classical tricyclic antidepressants (TCAs). However, an increasing number of studies, including meta-analyses, naturalistic studies, and longer-term studies suggested that SSRIs and SNRIs are no less safe than TCAs. We focused on comparing the common side effects of TCAs with those of newer generation antidepressants including SSRIs, SNRIs, mirtazapine, and bupropion. The main purpose was to investigate safety profile differences among drug classes rather than the individual antidepressants, so studies containing comparison data on drug groups were prioritized. In terms of... (truncated)

MESH:D008376 - maprotiline

Summary:

LLM Prediction Score	0.500 (normalized)
LLM Confidence Score	0.960
Golden Answer (Severity Class)	0.625 (normalized)
Prediction Error	0.125

Retrieved Context:

Title: Hepatitis caused by antidepressive therapy with maprotiline and opipramol.
There are few published reports of antidepressive therapy induced hepatotoxicity. In most cases antidepressants cause only slight elevation of liver enzymes without clinical relevance. However, our patient with recidivation of unipolar depressive disorder developed severe laboratory abnormalities and clinical symptoms during therapy with maprotiline (Ludiomil) and opipramol (Insidon). To our knowledge, this is the first case report of bioptically proven severe acute hepatitis caused by these antidepressants. After their withdrawal, the patient's fatigue symptoms, scleric jaundice, and marked increase of liver enzymes completely disappeared. Hepatic side effects should be considered during antidepressive therapy with maprotiline and opipramol especially when additional... (truncated)

Title: Hepatotoxicity associated with maprotiline therapy: case report.
The author reports a case of markedly elevated liver enzymes associated with maprotiline therapy for depression. The patient was cachexic but was anicteric and had no evidence of viral hepatitis. The serum enzyme levels returned to normal shortly after the medication was discontinued.

Title: Case report of hepatotoxicity associated with maprotiline.
A patient with unipolar affective disorder developed elevations in her liver enzymes shortly after initiation of therapy with maprotiline. The enzyme levels rapidly returned to normal after cessation of the drug with no residual liver disease.

Title: [Photoallergic dermatitis caused by the herbicide phenmedipham].
The case of a 51 year old woman, working in the field of agriculture, is reported; she developed a photoallergic dermatitis and a fulminant hepatitis after the use of the herbicide phenmedipham (Betanal). Photopatch-testing revealed sensitization to phenmedipham and, additionally, to maprotiline hydrochloride (Ludiomil), an antidepressant taken by the patient at about the same time. The possible relationship of these reactions with the clinical symptoms are discussed.

Title: Drug-Induced Liver Injury during Antidepressant Treatment: Results of AMSP, a Drug Surveillance Program.
Drug-induced liver injury is a common cause of liver damage and the most frequent reason for withdrawal of a drug in the United States. The symptoms of drug-induced liver damage are extremely diverse, with some patients remaining asymptomatic.

MESH:D000077213 - lamotrigine

Summary:

LLM Prediction Score	0.750 (normalized)
LLM Confidence Score	0.990
Golden Answer (Severity Class)	0.875 (normalized)
Prediction Error	0.125

Retrieved Context:

Title: Liver dysfunction induced by systemic hypersensitivity reaction to lamotrigine: case report.
Lamotrigine is an anticonvulsant drug used to treat partial and generalized seizure disorders. Hypersensitivity to lamotrigine usually causes mild symptoms such as fever, rash, and slight invasion of internal organs. However, a 33-year-old male patient who was admitted with Stevens-Johnson syndrome after taking lamotrigine for 15 days experienced hepatic failure and died 5 days after admission. This case demonstrates the importance of realizing that lamotrigine can lead to fatal hepatic failure, and that tests for the normal liver function should be performed when administering lamotrigine.

Title: Potential hepatotoxicity of lamotrigine.
Lamotrigine is a new antiepileptic drug that is effective for a broad range of seizures in adults and children. Three children with seizures of different causes who were treated with lamotrigine and developed reversible hepatotoxicity are reported. In one child, this therapy led to relatively severe hepatic failure that required and responded to aggressive therapy. Unlike most of the previously reported six patients with similar severe hepatic involvement, this patient's liver function and blood hepatic enzymes became normal. All three patients were on multiple drugs, and two were in epilepsia partialis continua secondary to encephalitis. Two of the patients had... (truncated)

Title: Fatal progressive hepatic necrosis associated with lamotrigine treatment: a case report and literature review.
We present a case of fatal, progressive hepatotoxicity in a patient treated with lamotrigine. After presenting with a rash and fever, she developed elevated liver function tests and clinical sequelae of hepatic failure. The subacute course of her progressive liver damage is documented in serial liver biopsies. While her initial biopsy showed approximately 50% hepatocyte necrosis, her post mortem examination performed three weeks later displayed massive hepatic necrosis with extensive bile duct proliferation. Although she was taking other antipsychotics at the time, her clinical course best parallels other reports of lamotrigine-associated hepatotoxicity. Here we discuss not only the clinicopathologic findings... (truncated)

Title: Pediatric ingestion of lamotrigine.
A 3-year-old female presented to the emergency department after ingesting forty-six 25-mg tablets of lamotrigine that resulted in sedation, rash, and transient elevation of liver function tests. Her initial physical examination was significant for marked somnolence and a lacy reticular blanching rash. Laboratory studies were all within normal limits except for mildly elevated liver function tests. Initial plasma lamotrigine level was found to be elevated above adult therapeutic levels (25.3 microg/mL). Treatment consisted of gastric lavage followed by activated charcoal. The patient was subsequently observed in the pediatric intensive care unit where symptoms and laboratory abnormalities promptly resolved, and she... (truncated)

Title: Case report: Progressive skin rash and lymphadenopathy associated with lamotrigine-valproic acid combination in a bipolar adolescent.
Background: Lamotrigine may cause severe skin reactions. There is a known interaction between lamotrigine and valproic acid with an increase in lamotrigine levels and lamotrigine toxicity risk. Few cases of severe rash and systemic reactions in bipolar patients using lamotrigine and valproate have been reported. Here, we report a rare case of severe skin rash and lymphadenopathy associated with lamotrigine-valproic acid combination. Case presentation: An 18-year-old female adolescent with bipolar disorder type I was treated with lamotrigine, magnesium valproate, and perospirone for 12 days. After the last dose of lamotrigine, she abruptly developed generalized rash and swollen lymph nodes, which continued... (truncated)

MESH:D015738 - famotidine

Summary:

LLM Prediction Score	0.250 (normalized)
LLM Confidence Score	0.990
Golden Answer (Severity Class)	0.375 (normalized)
Prediction Error	0.125

Retrieved Context:

Title: Hepatitis following famotidine: a case report.
H2 receptor antagonists can rarely cause idiosyncratic drug reactions leading to acute hepatitis. Famotidine, however, is considered a relatively safe drug with regards to hepatotoxicity. We report a case of a 47 year old male with a history of hepatitis C who developed acute hepatitis on the third day of hospitalization with a dramatic rise in his liver enzymes from normal values at the time of admission. The acute rise in liver enzymes made us consider an adverse drug reaction and famotidine was discontinued. Subsequently his liver enzymes came back to normal in seven days. Thus, physicians should consider famotidine... (truncated)

Title: Ranitidine treatment during a modest inflammatory response precipitates idiosyncrasy-like liver injury in rats.
Drug idiosyncrasy is an adverse event of unknown etiology that occurs in a small fraction of people taking a drug. Some idiosyncratic drug reactions may occur from episodic decreases in the threshold for drug hepatotoxicity. Previous studies in rats have shown that modest underlying inflammation triggered by bacterial lipopolysaccharide (LPS) can decrease the threshold for xenobiotic hepatotoxicity. The histamine-2 (H2)-receptor antagonist ranitidine (RAN) causes idiosyncratic reactions in people, with liver as a usual target. We tested the hypothesis that RAN could be rendered hepatotoxic in animals undergoing a modest inflammatory response. Male rats were treated with a nonhepatotoxic dose of... (truncated)

Title: Hepatic effects of drugs used in the treatment of peptic ulcer disease.
Although one member of the H2 blocker family (oxmetidine) proved to be intrinsically hepatotoxic, overt hepatic injury attributable to cimetidine and ranitidine is decidedly rare, given the tens of millions of patients who have received these medications. Even considering the fact that serious adverse reactions may be underreported by as much as a factor of 100, the number of reports submitted to the FDA-DDE of possible hepatic toxicity associated with these agents supports this low incidence. Whether or not a similarly low incidence will be seen with famotidine and the newer H2 receptor antagonists still under investigation must await additional... (truncated)

Title: Synergistic drug-cytokine induction of hepatocellular death as an in vitro approach for the study of inflammation-associated idiosyncratic drug hepatotoxicity.
Idiosyncratic drug hepatotoxicity represents a major problem in drug development due to inadequacy of current preclinical screening assays, but recently established rodent models utilizing bacterial LPS co-administration to induce an inflammatory background have successfully reproduced idiosyncratic hepatotoxicity signatures for certain drugs. However, the low-throughput nature of these models renders them problematic for employment as preclinical screening assays. Here, we present an analogous, but high-throughput, in vitro approach in which drugs are administered to a variety of cell types (primary human and rat hepatocytes and the human HepG2 cell line) across a landscape of inflammatory contexts containing LPS and cytokines TNF,... (truncated)

Title: Anaphylaxis due to First-Time Intravenous Infusion of N-Acetylcysteine in a Dog.
A 4-year-old female spayed Pomeranian was referred to the emergency service for intermittent trouble breathing and an enlarged liver found on ultrasound. A severe mixed hepatopathy was found on bloodwork, and ultrasound-guided liver aspirates showed marked hepatocellular vacuolar changes and rare neutrophils. An intravenous (IV) loading dose of n-acetylcysteine (NAC) was given for the first time in this patient, and immediately after the infusion the patient collapsed, became hypotensive, hypothermic, tachycardic, and developed gallbladder wall edema. Treatment for anaphylaxis was immediately initiated with IV fluids, an epinephrine bolus and then continuous rate infusion, diphenhydramine, and famotidine. Clinical signs resolved within... (truncated)

MESH:D008152 - loxapine

Summary:

LLM Prediction Score	0.500 (normalized)
LLM Confidence Score	0.980
Golden Answer (Severity Class)	0.625 (normalized)
Prediction Error	0.125

Retrieved Context:

Title: Drug-Associated Liver Injury Related to Antipsychotics: Exploratory Analysis of Pharmacovigilance Data.
Drug-associated liver injury is one of the most common causes for acute liver failure and market withdrawal of approved drugs. In addition, the potential for hepatotoxicity related to specific substances has to be considered in psychopharmacotherapy. However, systematic evaluations of hepatotoxicity related to antipsychotics are limited.

Title: Safety, tolerability, and risks associated with first- and second-generation antipsychotics: a state-of-the-art clinical review.
Since the discovery of chlorpromazine (CPZ) in 1952, first-generation antipsychotics (FGAs) have revolutionized psychiatric care in terms of facilitating discharge from hospital and enabling large numbers of patients with severe mental illness (SMI) to be treated in the community. Second-generation antipsychotics (SGAs) ushered in a progressive shift from the paternalistic management of SMI symptoms to a patient-centered approach, which emphasized targets important to patients - psychosocial functioning, quality of life, and recovery. These drugs are no longer limited to specific <i>Diagnostic and Statistical Manual of Mental Disorders</i> (<i>DSM</i>) categories. Evidence indicates that SGAs show an improved safety and tolerability profile... (truncated)

Title: Adverse Drug Reactions of Olanzapine, Clozapine and Loxapine in Children and Youth: A Systematic Pharmacogenetic Review.
Children and youth treated with antipsychotic drugs (APs) are particularly vulnerable to adverse drug reactions (ADRs) and prone to poor treatment response. In particular, interindividual variations in drug exposure can result from differential metabolism of APs by cytochromes, subject to genetic polymorphism. <i>CYP1A2</i> is pivotal in the metabolism of the APs olanzapine, clozapine, and loxapine, whose safety profile warrants caution. We aimed to shed some light on the pharmacogenetic profiles possibly associated with these drugs' ADRs and loss of efficacy in children and youth. We conducted a systematic review relying on four databases, following the Preferred Reporting Items for Systematic... (truncated)

Title: Antipsychotic Safety in Liver Disease: A Narrative Review and Practical Guide for the Clinician.
Clinicians treating psychiatric disorders in medically ill patients need a comprehensive resource for comparing the risk and types of liver injury associated with antipsychotic therapy.

Title: Hepatitis B and C in individuals with a history of antipsychotic medication use: A population-based evaluation.
A better understanding of links between mental illness and risk of bloodborne infectious disease could inform preventive and therapeutic strategies in individuals with mental illness.

MESH:D008148 - lovastatin

Summary:

LLM Prediction Score	0.500 (normalized)
LLM Confidence Score	0.990
Golden Answer (Severity Class)	0.375 (normalized)
Prediction Error	0.125

Retrieved Context:

Title: [Lovastatin-induced acute cholestatic hepatitis].
Acute cholestatic hepatitis developed in two patients, a 58-year-old man and a 54-year-old woman, who had been treated for hypercholesterolaemia with the cholesterol-synthesis inhibitor lovastatin for 3 years and 2 months, respectively. Both of them at first complained only of tiredness and loss of appetite, but then developed jaundice with colourless stool and dark urine. Alkaline phosphatase concentration rose up to maximally 1227 and 569 U/l, gamma-GT to 403 and 410 U/l, respectively. The transaminases and glutamate dehydrogenase were also elevated, while serum cholinesterase had fallen to 2346 and 2418 U/l, respectively. Histological examination of liver biopsies 6 months and... (truncated)

Title: Defining patient risks from expanded preventive therapies.
In clinical trials, all lipid-lowering agents have been associated with mild, asymptomatic elevations of alanine aminotransferase (ALT) and asparate aminotransferase enzymes. This, along with the fact that 3-hydroxy-3-methylglutaryl-coenzyme A (HMG-CoA) reductase inhibitors are hepatotoxic in some animals, led the US Food and Drug Administration (FDA) to recommend monitoring of liver enzymes for all lipid-lowering agents, except the bile acid sequestrants. Because the drugs act by different mechanisms, ALT elevations may be a pharmacodynamic effect related to lipid lowering, rather than a direct effect of the drug. Animal studies support this assumption. ALT elevations of 3 times the upper limit of... (truncated)

Title: Acute hepatitis induced by HMG-CoA reductase inhibitor, lovastatin.
The HMG-CoA reductase inhibitor, lovastatin, is known to induce asymptomatic liver dysfunction in a few patients. We report the case of an adult who suffered from clinical hepatitis three months after the onset of lovastatin administration. Manifestations included asthenia, jaundice, and increased aminotransferase and alkaline phosphatase activities. Histologic examination showed centrilobular necrosis, centrilobular cholestasis, and infiltrates with mononuclear and polymorphonuclear cells, including eosinophils. Withdrawal of lovastatin was followed by complete normalization of liver tests within two months.

Title: Hepatic effects of lovastatin exposure in patients with liver disease: a retrospective cohort study.
Little is known about the potential adverse hepatic effects of HMG-CoA reductase inhibitors ('statins') in patients with existing liver disease; therefore, we examined the risk of liver toxicity with lovastatin exposure in these patients.

Title: The liver and lovastatin.
The cholesterol-lowering agents, known as statins, have been in use for 15 years and are among the most commonly prescribed drugs. Animal studies and premarketing clinical trials have given signals of hepatotoxicity, primarily minor elevations in serum alanine aminotransferase enzyme (ALT) levels. For that reason, all of the cholesterol-lowering drugs have labeling that requires monitoring of liver enzymes. Postmarketing experience, however, suggests that hepatotoxicity is rare and thus it is timely to revisit the issue. The first of the statins, lovastatin, was approved in 1986 and has acquired 24 million patient-years of clinical experience. Minor elevations in liver enzymes, i.e.,... (truncated)

MESH:D017311 - amlodipine

Summary:

LLM Prediction Score	0.500 (normalized)
LLM Confidence Score	0.990
Golden Answer (Severity Class)	0.625 (normalized)
Prediction Error	0.125

Retrieved Context:

Title: A possible increase in liver enzymes due to amlodipine: A case report.
Amlodipine is a commonly prescribed antihypertensive drug, well tolerated and has rarely been attributed as a cause for elevated liver enzymes. Here, we present a 47-year-old male patient known to be hypertensive and admitted to our rehabilitation facility after an acute stroke. During his stay, amlodipine was started in addition to other antihypertensive medications to control his blood pressure. His liver transaminases after 4 days (notably alanine aminotransferase) were found to be markedly elevated. After reviewing the medications and investigating probable causes, amlodipine was suspended. After 5 days of suspending amlodipine, the transaminases started to trend downward. The Naranjo Adverse... (truncated)

Title: Severe Drug-Induced Liver Injury From Over-the-Counter Medication.
Drug-induced liver injury (DILI) is difficult to diagnose as it presents with a wide variety of clinical manifestations and there is no established specific biomarker. However, clinicians require expertise in diagnosing DILI as it can lead to critical illness, is relatively common, and can be caused by a variety of drugs, herbal medicines, and supplements. A 67-year-old male was admitted to the hospital with a fever, jaundice, and fatigue. Abdominal ultrasonography, computed tomography, and magnetic resonance cholangiopancreatography revealed no morphological abnormalities in the hepatobiliary system. On the third day of hospitalization, the liver damage and acute kidney injury progressed, and... (truncated)

Title: Possible amlodipine-induced hepatotoxicity after stem cell transplant.
To report the occurrence of hepatotoxicity in a patient initiated on amlodipine. A review of other reports of amlodipine hepatotoxicity is also provided.

Title: Amlodipine-Induced Liver Injury.
This is a case of an 88-year-old female with a history of hypertension who was started on amlodipine about three weeks prior to presentation. After about two weeks of amlodipine therapy, she developed intermittent right upper quadrant pain as well as pruritus which continued for a few days before she presented to medical attention. Her labs showed significantly elevated liver enzymes so she presented to the hospital for further evaluation. Imaging was unremarkable, her infectious and autoimmune workups were all negative. The amlodipine was discontinued and her liver enzymes slowly normalized after about seven weeks.

Title: Is Dysguesia Going to be a Rare or a Common Side-effect of Amlodipine?
A very rare side-effect of amlodipine is dysguesia. A review of the literature produced only one case. We report a case about a female with essential hypertension on drug treatment with amlodipine developed loss of taste sensation. Condition moderately improved on stoppage of the drug for 25 days. We conclude that amlodipine can cause dysguesia. Here, we describe the clinical presentation and review the relevant literature on amlodipine and dysguesia.

MESH:D008140 - lorazepam

Summary:

LLM Prediction Score	0.500 (normalized)
LLM Confidence Score	0.990
Golden Answer (Severity Class)	0.375 (normalized)
Prediction Error	0.125

Retrieved Context:

Title: Lorazepam as a Cause of Drug-Induced Liver Injury.
Lorazepam is a benzodiazepine derivative that is globally used for the therapy of anxiety and insomnia. A 51-year-old Japanese man with yellowish discoloration of the eyes and skin and pruritus was admitted due to liver dysfunction. He had taken lorazepam approximately 5 months prior to this admission. The clinical presentation and pathologic findings in the liver were consistent with drug-induced liver injury. After cessation of lorazepam, treatment with Stronger neo-minophagen C and ursodeoxycholic acid was started, and his liver injury resolved after 59 days. This case must serve as a warning to physicians to be aware of the possibility of... (truncated)

Title: Psychotropic drugs and liver disease: A critical review of pharmacokinetics and liver toxicity.
The liver is the organ by which the majority of substances are metabolized, including psychotropic drugs. There are several pharmacokinetic changes in end-stage liver disease that can interfere with the metabolization of psychotropic drugs. This fact is particularly true in drugs with extensive first-pass metabolism, highly protein bound drugs and drugs depending on phase I hepatic metabolic reactions. Psychopharmacological agents are also associated with a risk of hepatotoxicity. The evidence is insufficient for definite conclusions regarding the prevalence and severity of psychiatric drug-induced liver injury. High-risk psychotropics are not advised when there is pre-existing liver disease, and after starting a... (truncated)

Title: Simultaneous toxicities in a child on multiple anticonvulsants.
It is rare to develop simultaneous toxicities while on anticonvulsants. This article presents a 3(1/2)-year-old child on valproic acid, lamotrigine, and phenytoin who developed simultaneous hepatotoxicity and bone marrow toxicity during a parainfluenza virus type 3 infection. These toxicities resolved after the cessation of anticonvulsants, and her seizures were managed acutely with scheduled lorazepam. This article discusses the possibility that simultaneous use of valproic acid, lamotrigine, and phenytoin could give this combination of toxicities and that concurrent viral infection may increase this risk.

Title: Identifying olanzapine induced liver injury in the setting of acute hepatitis C: A case report.
Olanzapine is linked to asymptomatic, transient elevations of liver aminotransferases but is historically thought to rarely cause significant hepatotoxicity. Underlying liver disease is a risk factor for drug-induced liver injury and may complicate the differential diagnosis of acute transaminitis in patients taking medications associated with hepatotoxicity. Ms L presented with 2 months of new psychotic symptoms resulting in hospitalizations. Although psychosis previously improved with haloperidol, she reported symptoms concerning for akathisia. Restlessness improved and psychotic symptoms resolved after initiation of olanzapine. Concurrently, her alanine aminotransferase (ALT) was elevated, prompting further workup and new diagnosis of acute hepatitis C. Over the... (truncated)

Title: Aripiprazole reduces liver cell division.
Effects of aripiprazole on dopamine regulation are being tested as a treatment for patients with a dual diagnosis of schizophrenia and addictions, often cocaine dependence. Aripiprazole has one of the fewest side-effects among the second-generation antipsychotics. Nevertheless, severe aripiprazole hepatotoxicity was reported in persons with a history of cocaine and alcohol abuse. Here we report that therapeutically relevant aripiprazole concentrations, equal to laboratory alert levels in patients' serum, reduce the rate of hepatocytes' division. This could be an underlying mechanism of severe liver injury development in the patients with a history of alcohol and cocaine abuse, the two hepatotoxic agents... (truncated)

MESH:D008469 - meclofenamic acid

Summary:

LLM Prediction Score	0.500 (normalized)
LLM Confidence Score	0.890
Golden Answer (Severity Class)	0.375 (normalized)
Prediction Error	0.125

Retrieved Context:

Title: Mefenamic Acid-Upregulated Nrf2/SQSTM1 Protects Hepatocytes against Oxidative Stress-Induced Cell Damage.
Mefenamic acid (MFA) is a commonly prescribed non-steroidal anti-inflammatory drug (NSAID) with anti-inflammatory and analgesic properties. MFA is known to have potent antioxidant properties and a neuroprotective effect against oxidative stress. However, its impact on the liver is unclear. This study aimed to elucidate the antioxidative effects of MFA and their underlying mechanisms. We observed that MFA treatment upregulated the nuclear factor erythroid 2-related factor 2 (Nrf2) pathway. Treatment with various anthranilic acid derivative-class NSAIDs, including MFA, increased the expression of sequestosome 1 (SQSTM1) in HepG2 cells. MFA disrupted the interaction between Kelch-like ECH-associated protein 1 (Keap1) and Nrf2, activating... (truncated)

Title: Idiosyncratic NSAID drug induced oxidative stress.
Many idiosyncratic non-steroidal anti-inflammatory drugs (NSAIDs) cause GI, liver and bone marrow toxicity in some patients which results in GI bleeding/ulceration/fulminant hepatic failure/hepatitis or agranulocytosis/aplastic anemia. The toxic mechanisms proposed have been reviewed. Evidence is presented showing that idiosyncratic NSAID drugs form prooxidant radicals when metabolised by peroxidases known to be present in these tissues. Thus GSH, NADH and/or ascorbate were cooxidised by catalytic amounts of NSAIDs and hydrogen peroxide in the presence of peroxidase. During GSH and NADH cooxidation, oxygen uptake and activation occurred. Furthermore the formation of NSAID oxidation products was prevented during the cooxidation indicating that the... (truncated)

Title: The patterns of toxicity and management of acute nonsteroidal anti-inflammatory drug (NSAID) overdose.
The nonsteroidal anti-inflammatory drugs (NSAIDs) are widely used for their analgesic, anti-inflammatory and antipyretic actions. They are commonly taken in overdose in many areas of the world. The majority of patients with acute NSAID overdose will remain asymptomatic or develop minor self-limiting gastrointestinal symptoms. However, serious clinical sequelae have been reported in patients with acute NSAID overdose and these include convulsions, metabolic acidosis, coma and acute renal failure. There appear to be some differences between the NSAIDs in terms of the relative risk of these complications; in particular mefenamic acid is most commonly associated with convulsions. The management of these... (truncated)

Title: Significance of Multiple Bioactivation Pathways for Meclofenamate as Revealed through Modeling and Reaction Kinetics.
Meclofenamate is a nonsteroidal anti-inflammatory drug used in the treatment of mild-to-moderate pain yet poses a rare risk of hepatotoxicity through an unknown mechanism. Nonsteroidal anti-inflammatory drug (NSAID) bioactivation is a common molecular initiating event for hepatotoxicity. Thus, we hypothesized a similar mechanism for meclofenamate and leveraged computational and experimental approaches to identify and characterize its bioactivation. Analyses employing our XenoNet model indicated possible pathways to meclofenamate bioactivation into 19 reactive metabolites subsequently trapped into glutathione adducts. We describe the first reported bioactivation kinetics for meclofenamate and relative importance of those pathways using human liver microsomes. The findings validated only... (truncated)

Title: Toxicological Evaluation of Novel Cyclohexenone Derivative in an Animal Model through Histopathological and Biochemical Techniques.
Toxicity studies were conducted to provide safety data of potential drug candidates by determining lethal and toxic doses. This study was designed for pre-clinical evaluation of novel cyclohexenone derivative with respect to the acute and sub-acute toxicity along with the diabetogenic potential. Acute and sub-acute toxicity were assessed after intraperitoneal (i.p) injection of the investigational compound through selected doses for 21 days. This was followed by assessment of isolated body organs (liver, kidney, heart and pancreas) via biochemical indicators and histopathological techniques. No signs of toxicity were revealed in the study of acute toxicity. Similarly, a sub-acute toxicity study showed... (truncated)

MESH:D000077340 - fluvastatin

Summary:

LLM Prediction Score	0.500 (normalized)
LLM Confidence Score	0.970
Golden Answer (Severity Class)	0.375 (normalized)
Prediction Error	0.125

Retrieved Context:

Title: Hepatotoxicity Induced by Fluvastatin: A Reversible Acute Cholestatic Liver Injury.
BACKGROUND Fluvastatin, a commonly prescribed statin, is indicated for treatment of hypercholesterolemia in persons at high risk for coronary, cerebrovascular, and peripheral artery disease. However, there have been rare reports of liver injury or renal failure associated with use of fluvastatin. CASE REPORT We describe the case of a 69-year-old Saudi man with a medical history of diabetes mellitus and hypercholesterolemia for 2 years, on metformin, gliclazide modified release, daily aspirin, and simvastatin. Fluvastatin 40 mg daily was administered instead of simvastatin for 7 weeks before the patient was admitted to the hospital with fatigue, weakness, abdominal pain, loss of... (truncated)

Title: Drug-Induced Acute Liver Injury Within 12 Hours After Fluvastatin Therapy.
Although statins are generally well-tolerated drugs, recent cases of drug-induced liver injury associated with their use have been reported. A 52-year-old Chinese man reported with liver damage, which appeared 12 hours after beginning treatment with fluvastatin. Patient presented with complaints of increasing nausea, anorexia, and upper abdominal pain. His laboratory values showed elevated creatine kinase and transaminases. Testing for autoantibodies was also negative. The liver biochemistries eventually normalized within 3 weeks of stopping the fluvastatin. Therefore, when prescribing statins, the possibility of hepatic damage should be taken into account.

Title: Fluvastatin and hepatic reactions: a signal from spontaneous reporting in Italy.
Signal detection is a crucial element in recognising new adverse drug reactions (ADRs) as soon as possible. HMG-CoA reductase inhibitors ('statins'), the most potent cholesterol-lowering drugs, are generally well tolerated but can occasionally lead to liver toxicity. Pre- and postmarketing studies on statins revealed an incidence of 0.1-3% elevation in hepatic transaminase levels. However, these elevations are asymptomatic, reversible, dose related or probably due to other causes. Postmarketing studies clearly showed the lack of evidence of hepatotoxicity from statins, apart from some isolated case reports of serious hepatic damage described in the literature. It is still unclear whether serious hepatic... (truncated)

Title: Lipid-lowering drugs and serum liver enzymes: the effects of body weight and baseline enzyme levels.
The most important side effects of fibrate and 3-hydroxy-3-methylglutaryl-coenzyme A (HMG-CoA) reductase inhibitor (statin) treatment are hepatic toxicity and myopathy. Obese individuals may have higher levels of serum transaminases than their lean counterparts. The main purpose of this study was to examine the effects of statins and fibrates on liver enzymes in obese patients and to compare them with their effects on patients with various body mass indexes (BMI). Two hundred and sixty-three hyperlipidemic patients of both sexes aged 31-74 years were studied for 24 weeks. One hundred and three patients received fluvastatin (40 mg/day), 62 atorvastatin (10-20 mg/day), 45... (truncated)

Title: Fluvastatin.
Fluvastatin was the first wholly synthetic statin to the market and is effective in reducing total and low density lipoprotein cholesterol, which translates into reductions in coronary heart disease events. The Lescol Intervention Prevention Study has established the effectiveness of the early use of statins in reducing recurrent events in high-risk patients with coronary heart disease post percutaneous coronary interventions. Fluvastatin is well-tolerated with few side effects. The occurrence of significant abnormalities in liver enzymes is infrequent, and the risk of myositis and rhabdomyolysis seems to be less than with other statins. There have been no reports of fatal rhabdomyolysis... (truncated)

MESH:D010546 - perphenazine

Summary:

LLM Prediction Score	0.500 (normalized)
LLM Confidence Score	0.980
Golden Answer (Severity Class)	0.375 (normalized)
Prediction Error	0.125

Retrieved Context:

Title: Lysosomal membrane permeabilization mediated apoptosis involve in perphenazine-induced hepatotoxicity in vitro and in vivo.
Antipsychotic drugs represent a class of lysosomotropic drugs widely used in clinical practice. However, the hepatotoxicity of these drugs has been reported in recent years. Therefore, understanding the changes in cellular homeostasis mediated by these drugs is of great significance for revealing the true mechanisms underlying hepatotoxicity. Perphenazine is a classical antipsychotic drug that can reportedly induce extrapyramidal and sympatholytic side effects. The present research focuses on the toxicity effect of perphenazine on normal human hepatocytes. To assess the hepatotoxicity of continuous administration of perphenazine and investigate potential mechanisms related to apoptosis, human normal L02 hepatocytes were exposed to 10-40... (truncated)

Title: Fatal massive hepatic necrosis: a probable hypersensitivity reaction to sulfasalazine.
Sulfasalazine (salicylazosulfapyridine) is a commonly prescribed oral medication for inflammatory bowel disease. We report a case of a 15-yr-old boy with ulcerative colitis who developed a generalized hypersensitivity reaction with a serum sickness-like syndrome and severe hepatotoxicity while taking sulfasalazine, perphenazine, and amitriptyline. The injury to the liver persisted for 5 months after withdrawal of the drugs, and the patient died of terminal hepatic failure with massive hepatic necrosis. Severe hepatic toxicity to sulfasalazine is uncommon, but it can be fatal.

Title: Aripiprazole-induced liver injury: a spontaneous reporting database study.
Background: There have been individual case reports of aripiprazole in recent years, both domestically and internationally, but no analysis of the characteristics of the occurrence of adverse reactions/events of drug-induced liver injury with aripiprazole using spontaneous reports has been seen. Methods: Using a retrospective study approach, the 452 adverse reaction/event reports of aripiprazole-induced liver injury collected by the China Adverse Drug Reaction Monitoring System from 1 January 2012 to 31 December 2016 were analyzed and evaluated, and exploring it's the clinical characteristics and related risk factors for liver injury occurrence. Results: Among 452 cases of aripiprazole-induced liver injury ADR/ADE reports,... (truncated)

Title: Safety, tolerability, and risks associated with first- and second-generation antipsychotics: a state-of-the-art clinical review.
Since the discovery of chlorpromazine (CPZ) in 1952, first-generation antipsychotics (FGAs) have revolutionized psychiatric care in terms of facilitating discharge from hospital and enabling large numbers of patients with severe mental illness (SMI) to be treated in the community. Second-generation antipsychotics (SGAs) ushered in a progressive shift from the paternalistic management of SMI symptoms to a patient-centered approach, which emphasized targets important to patients - psychosocial functioning, quality of life, and recovery. These drugs are no longer limited to specific <i>Diagnostic and Statistical Manual of Mental Disorders</i> (<i>DSM</i>) categories. Evidence indicates that SGAs show an improved safety and tolerability profile... (truncated)

Title: Relevance of liver enzyme elevations with four different neuroleptics: a retrospective review of 7,263 treatment courses.
Data on liver enzyme elevations were collected in a retrospective study of 7,263 treatment courses with haloperidol, clozapine, perphenazine, and perazine. Charts of 233 patients hospitalized between 1980 and 1992 at Tübingen University Psychiatric Clinic were selected because clinically relevant increases of liver enzymes had been detected during monotherapy with one of the four examined neuroleptics. At least one hepatic enzyme (mostly alanine aminotransferase [ALAT]) exceeded the established reference range of 3-fold elevations of ALAT, aspartate aminotransferase, gamma-glutamyl transpeptidase, and glutamate dehydrogenase and 2-fold elevations of alkaline phosphatase (AP) during monotherapy with clozapine in 15%, perazine in 7.6%, perphenazine in... (truncated)

MESH:D008130 - lomustine

Summary:

LLM Prediction Score	0.500 (normalized)
LLM Confidence Score	0.980
Golden Answer (Severity Class)	0.375 (normalized)
Prediction Error	0.125

Retrieved Context:

Title: Toxicity Profile of Procarbazine Lomustine and Vincristine Chemotherapy in Low-Grade Glioma - Retrospective Review.
Background The role of Procarbazine Lomustine and Vincristine (PCV) chemotherapy is already established in terms of improving survival in low-grade glioma (LGG). This improved survival has led to the increasing administration of PCV to LGG patients over the past years. However, like other chemotherapies, serious hematological and non-hematological toxicities may occur. The purpose of this study was to evaluate the toxicity profile of PCV and its clinical relevance in our practice. Materials and Methods We reviewed 63 patients of LGG retrospectively who received chemotherapy PCV between January 2015 and January 2018 at Shaukat Khanum Memorial Cancer Hospital & Research Centre,... (truncated)

Title: Alkylation and Carbamylation Effects of Lomustine and Its Major Metabolites and MGMT Expression in Canine Cells.
DNA Alkylation is thought to be the reason for the efficacy of lomustine while carbamylation has been implicated as the cause for the side effects seen with lomustine treatment such as hepatotoxicity. In the alkylation study we show that lomustine and its metabolites form similar levels of the DNA adducts N^7 hydroxyethylguanine and O^6 hydroxyethyldeoxyguanosine. In terms of carbamylation, lomustine showed greater extent of carbamylation in the canine hepatocytes and lymphoma cell lines. The DNA repair enzyme O^6 methylguanine DNA methyltransferase (MGMT) causes resistance of tumor cells to bifunctional nitrosourea, like lomustine. There is no data available regarding MGMT expression/activity... (truncated)

Title: Evaluation of toxicity of carmustine with or without bevacizumab in patients with recurrent or progressive high grade gliomas.
An increased incidence in hematologic toxicity has been reported with the addition of bevacizumab to lomustine for patients with recurrent or progressive high grade gliomas (HGG). Data regarding incidence of toxicity with combination bevacizumab and carmustine is limited. The purpose of this study is to compare toxicity of single agent carmustine and carmustine plus bevacizumab for patients with HGGs.

Title: Procarbazine and CCNU Chemotherapy for Recurrent Glioblastoma with MGMT Promoter Methylation.
Label="BACKGROUND" NlmCategory="BACKGROUND">While procarbazine, CCNU (lomustine), and vincristine (PCV) has been an alternative chemotherapy option for malignant gliomas, it is worth investigating whether the combination of only procarbazine and CCNU is comparable because vincristine adds toxicity with uncertain benefit. The purpose of this study was to evaluate the feasibility of procarbazine and CCNU chemotherapy for recurrent glioblastoma multiforme (GBM) with O⁶-methylguanine-DNA-methyltransferase (MGMT) promoter methylation.

Title: Phase II trial of lomustine plus temozolomide chemotherapy in addition to radiotherapy in newly diagnosed glioblastoma: UKT-03.
To evaluate toxicity and efficacy of the combination of lomustine, temozolomide (TMZ) and involved-field radiotherapy in patients with newly diagnosed glioblastoma (GBM).

MESH:D016853 - muromonab-cd3

Summary:

LLM Prediction Score	0.500 (normalized)
LLM Confidence Score	0.880
Golden Answer (Severity Class)	0.375 (normalized)
Prediction Error	0.125

Retrieved Context:

Title: Muromonab-CD3 for the successful treatment of early chronic rejection after pediatric liver transplantation: report of a case.
A four-and-a-half-year-old boy underwent living-donor liver transplantation (LDLT) for progressive familial intrahepatic cholestasis. Immunosuppressive therapy was commenced with tacrolimus and methylprednisolone, despite which derangement of liver function tests (LFTs) became evident on postoperative day (POD) 7. A diagnosis of acute cellular rejection was made and steroid pulse therapy (SPT) was initiated. Although the LFTs improved transiently after SPT, they deteriorated again, and failed to respond to repeated SPT. Jaundice was prolonged and transudative ascitic fluid accumulated. Liver needle biopsies on PODs 20 and 24 confirmed severe graft damage constituting early chronic rejection. Based on the poor response to steroid therapy,... (truncated)

Title: Comparative analysis of T-cell depletion method for clinical immunotherapy-anti-hepatitis c effects of natural killer cells via interferon-γ production.
Liver transplantation (LT) is a life-saving treatment for liver cirrhosis patients with hepatocellular carcinoma (HCC). However, 10%-20% HCC recurrence rate after LT is due to the immunosuppression inducing tumor growth. We recently reported a novel immunotherapy with donor liver natural killer (NK) cells to prevent HCC and hepatitis C virus (HCV) recurrence after LT. In this cell processing procedure, Muromonab-CD3 (Orthoclone OKT3, an anti-CD3 antibody) was added to the culture medium to deplete CD3(+) T cells to prevent graft-versus-host disease. However, the manufacture of OKT3 was discontinued in 2010, when other treatments with similar efficacy and fewer side effects became... (truncated)

Title: OKT3 (muromonab-CD3) associated hepatitis in a kidney transplant recipient.
The introduction of OKT3 (muromonab-CD3) revolutionized the management of steroid-resistant rejection in transplant patients. Indeed, after the success of OKT3 for treatment of rejection, some centers used OKT3 for perioperative prophylaxis against rejection after transplantation. Despite the success of this agent for prevention and treatment of rejection, its use is associated with side effects. These side effects, including the cytokine release syndrome and flash pulmonary edema, are well recognized in the transplant community. However, there have been no published reports of acute severe hepatitis associated with the use of OKT3 in non-liver transplant patients. We describe here a case of... (truncated)

Title: Engineering of a trispecific tumor-targeted immunotherapy incorporating 4-1BB co-stimulation and PD-L1 blockade.
Co-stimulatory 4-1BB receptors on tumor-infiltrating T cells are a compelling target for overcoming resistance to immune checkpoint inhibitors, but initial clinical studies of 4-1BB agonist mAbs were accompanied by liver toxicity. We sought to engineer a tri-specific antibody-based molecule that stimulates intratumoral 4-1BB and blocks PD-L1/PD-1 signaling without systemic toxicity and with clinically favorable pharmacokinetics. Recombinant fusion proteins were constructed using scMATCH3 technology and humanized antibody single-chain variable fragments against PD-L1, 4-1BB, and human serum albumin. Paratope affinities were optimized using single amino acid substitutions, leading to design of the drug candidate NM21-1480. Multiple <i>in vitro</i> experiments evaluated pharmacodynamic properties... (truncated)

Title: Oral Administration of OKT3 MAb to Patients with NASH, Promotes Regulatory T-cell Induction, and Alleviates Insulin Resistance: Results of a Phase IIa Blinded Placebo-Controlled Trial.
Oral administration of anti-CD3 antibodies induced regulatory T cells (Tregs) alleviating the insulin resistance and liver damage in animal models.

MESH:D002738 - chloroquine

Summary:

LLM Prediction Score	0.500 (normalized)
LLM Confidence Score	0.990
Golden Answer (Severity Class)	0.375 (normalized)
Prediction Error	0.125

Retrieved Context:

Title: Chloroquine in the treatment of porphyria cutanea tarda.
Porphyria cutanea tarda (PCT) is common in Ethiopia and invariably affects the liver. Treatment by abstension from alcohol and avoidance of direct sunlight often failed to achieve lasting improvement. Phlebotomy is unacceptable to most of our patients and impractical as a routine therapy. Chloroquine phosphate 500 mg (300 mg base) given daily for 10 days to 24 patients with confirmed PCT, was found to be uniformly effective. Both clinical and biochemical remissions were complete, The side effects of chloroquine include fever, nausea, vomiting and myalgia which predictably occur on the third day of therapy and subside within 72 hours. Since... (truncated)

Title: Evaluation of acute and sublethal effects of chloroquine (C₁₈H₂₆ClN₃) on certain enzymological and histopathological biomarker responses of a freshwater fish <i>Cyprinus carpio</i>.
In this study the toxicity of antimalarial drug chloroquine (CQ) on certain enzymological (GOT, GPT and LDH) and histopathological alterations (Gill, liver and kidney) of a freshwater fish <i>Cyprinus carpio</i> was studied after acute (96 h) and sublethal (35 days) exposure. The median lethal concentration (96 h) of CQ was 31.62 mg/ml. During acute treatment (CQ at 31.62 mg/ml) the treated fish groups showed a significant increase in GOT and GPT activities in blood plasma; whereas LDH activity was decreased when compare to control groups. To analyse the effects of drug at the lowest concentration, the fish were exposed to 3.16 mg/ml (1/10th of 96 h LC50... (truncated)

Title: Hepatotoxic effects of anti-rheumatic drugs in cultured rat hepatocytes.
Drug-induced liver injury has been suggested as a possible mechanism for the liver damage in rheumatic diseases. To evaluate the role of direct toxic action of drugs on hepatocytes, the effects of salicylate, chloroquine, prednisolone and indomethacin on LDH leakage from cultured rat hepatocytes were studied. Exposure for 24 h to the first two drugs induced liver damage, as reflected by LDH release, at concentrations 2-10 times as high as the therapeutic plasma levels in humans. Indomethacin and prednisolone at concentrations approx 50-100-fold higher than the therapeutic plasma levels, were not toxic to cultured hepatocytes. The data suggest that direct... (truncated)

Title: Chloroquine in treatment of porphyria cutanea tarda. Long-term efficacy of combined phlebotomy and high-dose chloroquine therapy.
The combined treatment with phlebotomy and high-dose chloroquine for PCT is described and 21 patients treated were followed up for periods of up to 6 years. Clinical and laboratory remissions were experienced by all patients. The urinary excretion of porphyrin and serum hepatic enzyme levels rose rapidly during therapy and then fell, mostly to levels well below that before treatment. The periods required for normalization of laboratory tests, median value, were 1.5 months for liver enzymes and 7 months for uroporphyrins. Clinical symptoms soon disappeared, blistering within 3 weeks and skin fragility within 12. Mild nausea or subfebrility was noted... (truncated)

Title: Protective effect of quercetin on chloroquine-induced oxidative stress and hepatotoxicity in mice.
The present study was aimed to find out the protective effect of quercetin on hepatotoxicity resulting by commonly used antimalarial drug chloroquine (CQ). Swiss albino mice were administered with different amounts of CQ ranging from human therapeutic equivalent of 360 mg/kg body wt. to as high as 2000 mg/kg body wt. We observed statistically significant generation of reactive oxygen species, liver toxicity, and oxidative stress. Our observation of alterations in biochemical parameters was strongly supported by real-time PCR measurement of mRNA expression of key biochemical enzymes involved in hepatic toxicity and oxidative stress. However, the observed hepatotoxicity and accompanying oxidative... (truncated)

MESH:D007559 - ivermectin

Summary:

LLM Prediction Score	0.500 (normalized)
LLM Confidence Score	0.990
Golden Answer (Severity Class)	0.375 (normalized)
Prediction Error	0.125

Retrieved Context:

Title: Ivermectin drug induced liver injury.
Ivermectin remains a popular, albeit unproven, therapy used in both the prevention and treatment of COVID-19. We discuss a patient who developed jaundice and a liver injury 3 weeks after initiating ivermectin for COVID prevention. Liver histology demonstrated a pattern of injury that was both portal and lobular, with a bile ductulitis as well with marked cholesasis. She was managed with low dose corticosteroids, later tapered and withdrawn. She remains well a year after presenting.

Title: Macrofilaricidal Activity, Acute and Biochemical Effects of Three Lichen Species Found on Mount Cameroon.
Onchocerciasis is a parasitic infection affecting a relatively small population globally but has very devastating pathological outcomes. Ivermectin and recently moxidectin are the only drugs approved for clinical management of the disease, both of which have several limitations. In particular, they are efficacious against microfilariae (microfilaricidal) with no activity against adult worms (nonmacrofilaricidal). Promising anthelmintic activity has been reported in some lichens. This study investigated three lichens, <i>Usnea articulata</i>, <i>Parmotrema tinctorum</i>, and <i>Heterodermia obscurata</i>, found on Mount Cameroon, for potential macrofilaricidal activity. Organic extracts were screened for anti-Onchocerca activity against <i>Onchocerca ochengi</i> isolated from cattle skin using worm motility and... (truncated)

Title: Possible pathogenic pathways in the adverse clinical events seen following ivermectin administration to onchocerciasis patients.
BACKGROUND: Reactions are commonly associated with the chemotherapy of onchocerciasis. However unmanageable reactions are uncommon when ivermectin (Mectizan(R)) is used for the treatment of this infection, and this drug has proved to be a great improvement over previously used agents. Serious adverse events (SAE) nevertheless have occurred, and there is considerable concern about the negative effect such events may have on mass drug administration programs.This paper reviews the basic pathogenic mechanisms that can be involved in the destruction of microfilaria by chemotherapeutic agents. A central challenge to filarial chemotherapy is the need to remove parasites from biologically sensitive tissues, a... (truncated)

Title: Serious adverse reactions associated with ivermectin: A systematic pharmacovigilance study in sub-Saharan Africa and in the rest of the World.
Ivermectin is known to cause severe encephalopathies in subjects infected with loiasis, an endemic parasite in Sub-Saharan Africa (SSA). In addition, case reports have described ivermectin-related serious adverse drug reactions (sADRs) such as toxidermias, hepatic and renal disorders. The aim of this study was to identify suspected sADRs reported after ivermectin administration in VigiBase, the World Health Organization's global individual case safety reports database and analyze their frequency relative to the frequency of these events after other antinematodal drugs reported in SSA and other areas of the world (ROW).

Title: First case of ivermectin-induced severe hepatitis.
Loiasis, caused by the filarial parasite Loa loa, is endemic in West and Central Africa. Ivermectin has been shown to be an effective treatment of loiasis. We report the case of a 20-year-old woman originally from Cameroon who was infected by the L. loa parasite and developed severe hepatitis, identified 1 month after a single dose of ivermectin. Liver biopsy showed intralobular inflammatory infiltrates, confluent necrosis and apoptosis, compatible with drug-induced liver disease. To our knowledge, this is the first case of ivermectin-induced severe liver disease published in the literature.

MESH:D003543 - cysteamine

Summary:

LLM Prediction Score	0.500 (normalized)
LLM Confidence Score	0.970
Golden Answer (Severity Class)	0.375 (normalized)
Prediction Error	0.125

Retrieved Context:

Title: Treatment of severe acetaminophen poisoning with intravenous acetylcysteine.
Intravenous (IV) acetylcysteine, cysteamine, and methionine treatments were compared in patients with severe acetaminophen poisoning; a control group consisted of patients receiving supportive therapy only. Acetylcysteine proved the safest and most effective mode of treatment. Acetylcysteine was effective in preventing liver damage, hepatic failure, renal damage, and death when given eight to ten hours after poisoning. When treatment was delayed for ten to 24 hours, results were the same as in the supportive-therapy group. The alanine aminotransferase (ALT) activity remained normal in 76% of the patients treated within ten hours, as compared with 40% in both cysteamine- and methionine-treated groups... (truncated)

Title: Hepatic lipid metabolism disorders and immunotoxicity induced by cysteamine in early developmental stages of zebrafish.
Cysteamine, a sulfhydryl compound, is an intermediate in the metabolism of coenzyme A to taurine in living organisms. However, the potential side effects of cysteamine such as hepatotoxicity in pediatric patients have been reported in some studies. To evaluate the impact of cysteamine on infants and children, larval zebrafish (a vertebrate model) were exposed to 0.18, 0.36 and 0.54 mM cysteamine from 72 hpf to 144 hpf. Alterations in general and pathological evaluation, biochemical parameters, cell proliferation, lipid metabolism factors, inflammatory factors and Wnt signaling pathway levels were examined. Increased liver area and lipid accumulation were observed in liver morphology,... (truncated)

Title: Intravenous delivery of cysteamine for the treatment of cystinosis: association with hepatotoxicity.
Nephropathic cystinosis is a lysosomal storage disorder, which, if untreated, results in renal failure by age 10 years. Oral cysteamine has been shown to preserve renal function in these patients. In this study, a 2-year-old girl with nephropathic cystinosis and severe gastrointestinal dysmotility was treated with intravenous (i.v.) administration of cysteamine hydrochloride (HCl). This is only the second report of long-term i.v. cysteamine therapy for nephropathic cystinosis. Unlike the treatment in the previous case, however, treatment in our patient was limited by liver toxicity.

Title: Early prediction of the outcome of a paracetamol overdose based on an analysis of 163 patients.
Clinical and biochemical data obtained from 163 patients who had taken an overdose of paracetamol were examined to determine which factors or measurements were of value in predicting the severity of ensuing liver damage early after ingestion of tablets. Although the overall severity of hepatic necrosis was found to increase with the dose of paracetamol ingested, correlation was not sufficiently close to provide an accurate prognostic index in individuals. Severe hepatic damage was less likely if the patient had vomited or had a stomach wash-out within 6 hr of overdose. The plasma concentrations of paracetamol, measured at known times after... (truncated)

Title: Cysteamine, methionine, and penicillamine in the treatment of paracetamol poisoning.
60 patients with paracetamol poisoning have been treated with intravenous cysteamine, L-methionine, or D-penicillamine and the incidence and severity of hepatic necrosis compared with those observed in 70 patients receiving supportive therapy only. Of 31 patients with 4-hour plasma-paracetamol concentrations greater than 250 mug/ml given supportive therapy 22 sustained severe liver damage, 3 died in hepatic failure, and 4 developed acute renal failure. None of 23 similarly poisoned patients given cysteamine within 10 hours of ingestion suffered severe liver damage or renal failure and none died. Cysteamine was partially effective at 10-12 hours, but ineffective 12 hours or more after... (truncated)

MESH:D004976 - ethacrynic acid

Summary:

LLM Prediction Score	0.500 (normalized)
LLM Confidence Score	0.880
Golden Answer (Severity Class)	0.625 (normalized)
Prediction Error	0.125

Retrieved Context:

Title: Ethacrynic Acid: A Promising Candidate for Drug Repurposing as an Anticancer Agent.
Ethacrynic acid (ECA) is a diuretic that inhibits Na-K-2Cl cotransporter (NKCC2) present in the thick ascending loop of Henle and muculo dens and is clinically used for the treatment of edema caused by excessive body fluid. However, its clinical use is limited due to its low bioavailability and side effects, such as liver damage and hearing loss at high doses. Despite this, ECA has recently emerged as a potential anticancer agent through the approach of drug repositioning, with a novel mechanism of action. ECA has been shown to regulate cancer hallmark processes such as proliferation, apoptosis, migration and invasion, angiogenesis,... (truncated)

Title: Pomegranate prevents binge alcohol-induced gut leakiness and hepatic inflammation by suppressing oxidative and nitrative stress.
Alcoholic liver disease (ALD) is a major chronic liver disease worldwide and can range from simple steatosis, inflammation to fibrosis/cirrhosis possibly through leaky gut and systemic endotoxemia. We investigated whether pomegranate (POM) protects against binge alcohol-induced gut leakiness, endotoxemia, and inflammatory liver damage. After POM pretreatment for 10 days, rats were exposed to 3 oral doses of binge alcohol (5 g/kg/dose) or dextrose (as control) at 12-h intervals. Binge alcohol exposure induced leaky gut with significantly elevated plasma endotoxin and inflammatory fatty liver by increasing the levels of oxidative and nitrative stress marker proteins such as ethanol-inducible CYP2E1, inducible nitric... (truncated)

Title: Strategies for Early Prediction and Timely Recognition of Drug-Induced Liver Injury: The Case of Cyclin-Dependent Kinase 4/6 Inhibitors.
The idiosyncratic nature of drug-induced liver injury (DILI) represents a current challenge for drug developers, regulators and clinicians. The myriad of agents (including medications, herbals, and dietary supplements) with recognized DILI potential not only strengthens the importance of the post-marketing phase, when urgent withdrawal sometimes occurs for rare unanticipated liver toxicity, but also shows the imperfect predictivity of pre-clinical models and the lack of validated biomarkers beyond traditional, non-specific liver function tests. After briefly reviewing proposed key mechanisms of DILI, we will focus on drug-related risk factors (physiochemical and pharmacokinetic properties) recently proposed as predictors of DILI and use cyclin-dependent... (truncated)

Title: Endogenous Antioxidant and LOX-Mediated Systems Contribute to the Hepatoprotective Activity of Aqueous Partition of Methanol Extract of <i>Muntingia calabura</i> L. Leaves against Paracetamol Intoxication.
Methanol extract of <i>Muntingia calabura</i> L. (family Muntingiaceae) leaf has been reported to exert various pharmacological activities including hepatoprotection. The present study was carried out to identify the most effective hepatoprotective partition derived from the extract and to determine the mechanisms of action involved. The extract was partitioned using solvents with different polarity to yield petroleum ether (PEMC), ethyl acetate (EAMC), and aqueous (AQMC) extracts. Each extract, at 250 mg/kg, was subjected to the paracetamol (PCM)-induced hepatotoxic assay and several parameters such as liver weight, liver/body weight ratio, serum liver enzymes' level, and histopathological examinations were determined. Each partition was... (truncated)

Title: Application of 3D Hepatic Plate-Like Liver Model for Statin-Induced Hepatotoxicity Evaluation.
Background: Drug-induced liver injury is one of the main reasons of withdrawals of drugs in postmarketing stages. However, an experimental model(s) which can accurately recapitulates liver functions and reflects the level of drug hepatotoxicity is lack. In this study, we assessed drug hepatotoxicity using a novel three-dimensional hepatic plate-like hydrogel fiber (3D-P) co-culture system. Methods: During the 28-days culture period, the liver-specific functions, hepatocyte polarity, sensitivity of drug-induced toxicity of 3D-P co-culture system were evaluated with 2D co-culture, collagen sandwich co-culture, 3D hybrid hydrogel fiber co-culture and human primary hepatocytes as controls. High-content imaging and analysis (HCA) methods were used... (truncated)

MESH:D002994 - clofibrate

Summary:

LLM Prediction Score	0.500 (normalized)
LLM Confidence Score	0.940
Golden Answer (Severity Class)	0.375 (normalized)
Prediction Error	0.125

Retrieved Context:

Title: Adverse effects of hypolipidaemic drugs.
Cholestyramine, colestipol, clofibrate, gemfibrozil, nicotinic acid (niacin), probucol, neomycin, and dextrothyroxine are the most commonly used drugs in the treatment of hyperlipoproteinaemic disorders. While adverse reaction data are available for all of them, definitive data regarding the frequency and severity of potential adverse effects from well-controlled trials using large numbers of patients (greater than 1000) are available only for cholestyramine, clofibrate, nicotinic acid and dextrothyroxine. In adult patients treated with cholestyramine, gastrointestinal complaints, especially constipation, abdominal pain and unpalatability are most frequently observed. Continued administration along with dietary manipulation (e.g. addition of dietary fibre) and/or stool softeners results in diminished... (truncated)

Title: The Effects of Clofibrate on Neonatal Jaundice: A Systematic Review.
Neonatal jaundice is a prevalent disease that causes many complications, including kernicterus and even death. Previous studies have shown that clofibrate as an aryloxy isobutyric acid derivate can be effectively applied for the treatment of neonatal jaundice. Thus, this review was carried out to investigate the effects and mechanism of action of clofibrate on neonatal jaundice.

Title: Mitochondria, PPARs, and Cancer: Is Receptor-Independent Action of PPAR Agonists a Key?
Before the discovery of peroxisome proliferator activated receptors (PPARs), it was well known that certain drugs considered as classical PPAR-alpha agonists induced hepatocarcinoma or peroxisome proliferation in rodents. These drugs were derivatives of fibric acid, and they included clofibrate, bezafibrate, and fenofibrate. However, such toxicity has never been observed in human patients treated with these hypolipidemic drugs. Thiazolidinediones are a new class of PPAR activators showing greater specificity for the gamma isoform of PPARs. These drugs are used as insulin sensitizers in the treatment of type II diabetes. In addition, they have been shown to induce cell differentiation or apoptosis... (truncated)

Title: Effects of di-isononyl phthalate, di-2-ethylhexyl phthalate, and clofibrate in cynomolgus monkeys.
The effects of the peroxisome proliferators di-isononyl phthalate (DINP) and di-2-ethylhexyl phthalate (DEHP) were evaluated in young adult male cynomolgus monkeys after 14 days of treatment, with emphasis on detecting hepatic and other effects seen in rats and mice after treatment with high doses of phthalates. Groups of 4 monkeys received DINP (500 mg/kg/day), DEHP (500 mg/kg/day), or vehicle (0.5% methyl cellulose, 10 ml/kg) by intragastric intubation for 14 consecutive days. Clofibrate (250 mg/kg/day), a hypolipidemic drug used for cholesterol reduction in human patients was used as a reference substance. None of the test substances had any effect on body... (truncated)

Title: Decrease of hepatic stellate cells in rats with enhanced sensitivity to clofibrate-induced hepatocarcinogenesis.
To examine the possible involvement of nonparenchymal cells in the development of preneoplastic hepatic lesions induced by clofibrate (CF), alterations of these cells were investigated immunohistochemically in glutathione S-transferase M1 gene polymorphic rats (KS and NC types) with different cancer susceptibilities. After CF administration for 8 weeks, α-smooth muscle actin (α-SMA)-positive hepatic stellate cells (HSC) were markedly decreased in sensitive KS-type rats, but not in the NC-type rats. Kupffer cells were decreased with similar extents between them. The sinusoidal endothelial cells were not changed in either type. The other markers for HSC, vimentin and CRBP1, also confirmed the decrease of... (truncated)

MESH:C078456 - denileukin diftitox

Summary:

LLM Prediction Score	0.500 (normalized)
LLM Confidence Score	0.940
Golden Answer (Severity Class)	0.375 (normalized)
Prediction Error	0.125

Retrieved Context:

Title: E7777 in Japanese patients with relapsed/refractory peripheral and cutaneous T-cell lymphoma: A phase I study.
E7777, a recombinant cytotoxic fusion protein comprising diphtheria toxin fragments A and B and human interleukin-2, shares an amino acid sequence with denileukin diftitox but has improved purity and an increased percentage of active protein monomer species. A phase I study was carried out to evaluate the tolerability, safety, pharmacokinetics, and antitumor activity of E7777 in Japanese patients with relapsed/refractory peripheral and cutaneous T-cell lymphoma. E7777 (6, 12, and expanded 9 μg/kg/day) was given to 13 patients by i.v. infusion on five consecutive days per 21-day cycle. Dose-limiting toxicities, including increased alanine aminotransferase, hyponatremia (n = 2), hypokalemia, lymphopenia, fatigue,... (truncated)

Title: Denileukin diftitox for the treatment of steroid-resistant acute graft-versus-host disease.
Acute graft-versus-host disease (aGVHD) is partly mediated through activated T cells, and these cells are known to express the high-affinity receptor for interleukin 2 (IL-2R). Denileukin diftitox is composed of human IL-2 and diphtheria toxin that is cytotoxic to activated lymphocytes expressing the high-affinity IL-2R. We describe the results of a phase II study of denileukin diftitox in 22 patients with steroid-resistant aGVHD. Twenty patients were treated at dose level 1 (4.5 microg/kg daily on days 1-5 and then weekly on study days 8, 15, 22, and 29), and 2 patients were treated at dose level 2 (9.0 microg/kg delivered... (truncated)

Title: Transient T cell depletion causes regression of melanoma metastases.
Cognate immunity against neoplastic cells depends on a balance between effector T cells and regulatory T (Treg) cells. Treg cells prevent immune attack against normal and neoplastic cells by directly suppressing the activation of effector CD4+ and CD8+ T cells. We postulated that a recombinant interleukin 2/diphtheria toxin conjugate (DAB/IL2; Denileukin Diftitox; Ontak) may serve as a useful strategy to deplete Treg cells and break tolerance against neoplastic tumors in humans.

MESH:D005000 - ethionamide

Summary:

LLM Prediction Score	0.500 (normalized)
LLM Confidence Score	0.970
Golden Answer (Severity Class)	0.375 (normalized)
Prediction Error	0.125

Retrieved Context:

Title: Hepatitis in leprosy patients treated by a daily combination of dapsone, rifampin, and a thioamide.
A 13% incidence of hepatitis was observed among 54 cases of multibacillary leprosy treated daily with the three-drug combination of dapsone, rifampin, and a thioamide (ethionamide or prothionamide). No hepatitis was observed among 109 cases of paucibacillary leprosy treated daily with the two-drug combination of dapsone and rifampin. Symptoms were jaundice in five cases and nausea plus vomiting associated with a significant increase of transaminase levels in two cases. In five cases, the symptoms appeared during the first two months of therapy and in two cases, later. Discontinuing treatment with rifampin and the thioamide but not dapsone resulted in recovery.... (truncated)

Title: Hepatic toxicity during chemotherapy for severe tuberculosis meningitis.
The possible development of hepatotoxic effects as a result of high dosages of isoniazid, rifampin, pyrazinamide, and ethionamide was assessed in 56 young children (median age, 22 months) treated for severe tuberculous meningitis (TBM). Only one of the 56 children became jaundiced, probably as result of hepatitis A infection. Of 33 children observed for at least eight weeks, only five (15%) had normal serum aspartate aminotransferase (AST), alanine aminotransferase (ALT), and gamma-glutamyl transferase levels throughout, but in only three patients were AST or ALT values greater than 200 U/L, and enzyme levels tended to normalize toward the end of the... (truncated)

Title: Metabolism of the anti-tuberculosis drug ethionamide by mouse and human FMO1, FMO2 and FMO3 and mouse and human lung microsomes.
Tuberculosis (TB) results from infection with Mycobacterium tuberculosis and remains endemic throughout the world with one-third of the world's population infected. The prevalence of multi-drug resistant strains necessitates the use of more toxic second-line drugs such as ethionamide (ETA), a pro-drug requiring bioactivation to exert toxicity. M. tuberculosis possesses a flavin monooxygenase (EtaA) that oxygenates ETA first to the sulfoxide and then to 2-ethyl-4-amidopyridine, presumably through a second oxygenation involving sulfinic acid. ETA is also a substrate for mammalian flavin-containing monooxygenases (FMOs). We examined activity of expressed human and mouse FMOs toward ETA, as well as liver and lung microsomes.... (truncated)

Title: [Hepatotoxic effect of ethionamide and para-amino-salicylic acid in initial chemotherapy of patients with pulmonary tuberculosis in the light of liver function tests].
No abstract available.

Title: [The hepatotoxicity of ethionamide remains a topical subject. Apropos of a case of acute hepatitis].
The authors discuss the case of a 22 year-old patient treated for lepromatous leprosy who developed acute hepatitis 2 years after beginning polychemotherapy associating thalidomide, rifampin, ethionamide and clofazimine. Besides the ethionamide, there was no hepato-toxic factor and the hepatic anomalies quickly disappeared upon termination of treatment with this product. This again draws attention to the hepato-toxicity of ethionamide.

MESH:D000078328 - felbamate

Summary:

LLM Prediction Score	0.750 (normalized)
LLM Confidence Score	0.980
Golden Answer (Severity Class)	0.875 (normalized)
Prediction Error	0.125

Retrieved Context:

Title: Felbamate.
The development and release of felbamate is characterized by several relatively unique features. Felbamate was submitted to innovative and unconventional clinical trials. It was the first antiepileptic drug (AED) to be tested in a double-blind fashion in patients withdrawn from AEDs for presurgical video/electroencephalogram (EEG) monitoring. In addition, felbamate was the first AED to be tested in double-blind monotherapy trials. Felbamate was also the first drug to be tested in a placebo-controlled trial in children with the Lennox-Gastaut syndrome. When it was first released in North America in 1993, felbamate was the first new antiepileptic drug in 15 years. Finally,... (truncated)

Title: Felbamate in epilepsy therapy: evaluating the risks.
Felbamate demonstrates a unique therapeutic profile and often results in seizure control when other agents fail. Its use has been associated with risks for aplastic anaemia and hepatic failure. A number of confounding factors makes the actual incidence rate for each adverse effect difficult to determine. However, certain risk factors are common in reported cases. In order to minimise the risk, at the present time, it is necessary to rely on the clinical profile of the patients reporting these adverse effects. The patient reporting aplastic anaemia is usually female, Caucasian, and an adult. The dose did not appear to be... (truncated)

Title: Antiepileptic Drugs and Liver Disease.
Acute, symptomatic seizures or epilepsy may complicate the course of hepatic disease. Choosing the most appropriate antiepileptic drug in this setting represents a difficult challenge, as most medications are metabolized by the liver. This article focuses on the acute and chronic treatment of seizures in patients with advanced liver disease and reviews the hepatotoxic potential of specific antiepileptic drugs. Newer antiepileptic drugs without, or with minimal, hepatic metabolism, such as levetiracetam, lacosamide, topiramate, gabapentin, and pregabalin should be used as first-line therapy. Medications undergoing extensive hepatic metabolism, such as valproic acid, phenytoin, and felbamate should be used as drugs of... (truncated)

Title: Development of a highly sensitive cytotoxicity assay system for CYP3A4-mediated metabolic activation.
Drug-induced hepatotoxicity, which is a rare but serious adverse reaction to a large number of pharmaceutical drugs, is sometimes associated with reactive metabolites produced by drug-metabolizing enzymes. In the present study, we constructed a cell-based system to evaluate the cytotoxicity of reactive metabolites produced by CYP3A4 using human hepatoma cells infected with an adenovirus vector expressing human CYP3A4 (AdCYP3A4). When seven hepatoma cell lines (HepG2, Hep3B, HLE, HLF, Huh6, Huh7, and Fa2N4 cells) were infected with AdCYP3A4, HepG2 cells showed the highest CYP3A4 protein expression and testosterone 6β-hydroxylase activity (670 pmol · min(-1) · mg(-1)). With the use of AdCYP3A4-infected... (truncated)

Title: Investigating the role of 2-phenylpropenal in felbamate-induced idiosyncratic drug reactions.
Felbamate (2-phenyl-1,3-propanediol dicarbamate, FBM) can cause aplastic anemia and hepatotoxicity. The mechanism of FBM-induced toxicities is unknown; however, it has been proposed that 2-phenylpropenal, a reactive metabolite of FBM, is responsible. The pathway leading to this metabolite involves hydrolysis of FBM to 2-phenyl-1,3-propandiol monocarbamate (MCF), oxidation to 3-carbamoyl-2-phenylpropionaldehyde (CBMA), and spontaneous loss of carbon dioxide and ammonia. We made a polyclonal antibody against 2-phenylpropenal bound to protein and confirmed its specificity using ELISA. We attempted to develop an animal model of FBM-induced aplastic anemia and/or hepatotoxicity, and we also used the antibody to try to detect covalent binding of 2-phenylpropenal... (truncated)

MESH:D010400 - penicillin g

Summary:

LLM Prediction Score	0.250 (normalized)
LLM Confidence Score	0.990
Golden Answer (Severity Class)	0.375 (normalized)
Prediction Error	0.125

Retrieved Context:

Title: [Hepatic involvement in secondary syphilis].
A case of secondary syphilitic hepatitis is reported. A 49-year old woman was admitted for weight-loss, fever, hepatomegaly and splenomegaly. Diagnosis of syphilitic hepatitis was based on cholestasis associated with positive serologic tests for syphilis without other immunological disturbances. Biopsy of the liver showed a moderate infiltration in and around the portal tracts. Immunofluorescence study for treponema was negative. Rapid improvement was obtained with penicillin initially associated with steroid therapy. Liver involvement in secondary syphilis is characterized by anicteric cholestasis, an inflammatory syndrome, and periportal infiltrate inconstantly associated with centrilobular necrosis, granulomatous reaction and presence of treponemas in the lesions.... (truncated)

Title: Reversible oxacillin hepatotoxicity.
Eight patients developed elevations of hepatic enzymes while receiving oxacillin intravenously. In all instances the patients were asymptomatic and anicteric. Peripheral eosinophilia was present in five of eight patients. In each patient change of therapy to a different pencillinase-resistant penicillin or to penicillin G was associated with a rapid decrease in liver function abnormalities and evenutal return of the enzymatic values to normal. Change of medication to an alternative penicillinase-resistant penicillin or to penicillin G is suggested as a safe procedure for completion of antistaphylococcal therapy in patient who develop oxacillin-related hepatotoxicity.

Title: Antibiotic Disruption of the Gut Microbiota Enhances the Murine Hepatic Dysfunction Associated With a High-Salt Diet.
Epidemiological and experimental evidence indicates that antibiotic exposure is related to metabolic malfunctions, such as obesity and non-alcoholic fatty liver disease (NAFLD). Liver impairment and hypertrophy of adipose cells are related to high salt consumption. This research aims to investigated the physiological mechanism of a high salt diet (HSD) enhanced antibiotic-induced hepatic injury and mitochondrial abnormalities in mice. The mice were fed a HSD with or without penicillin G (PEN) for 8 weeks and the gut metabolome, untargeted faecal metabolomics, and intestinal function were evaluated. The results revealed that HSD, PEN and their combination (HSPEN) significantly changed the gut microbial community.... (truncated)

Title: Intravenous desensitization to beta-lactam antibiotics.
Patients allergic to penicillin (PCN) often require treatment with beta-lactam antibiotics for life-threatening bacterial infections. In this article, we review our experience with rapid intravenous desensitization for patients who gave a history of PCN allergy and who had hypersensitivity demonstrated by skin tests. Skin testing was performed with both prick and intradermal techniques and with the recommended antibiotic as well as PCN G, penicilloyl polylysine, and a minor determinant mixture. Patients were transferred to the intensive care unit, and desensitization was performed with a buret technique that required minimal preparation and was easily applied to any antibiotic. Fifteen desensitizations in... (truncated)

Title: Nafcillin-Induced Allergic Eosinophilic Cholestatic Hepatitis.
A 71-year-old female with no history of liver disease or antibiotic allergy developed jaundice with elevated liver enzymes and eosinophilia following treatment with nafcillin for septic arthritis. Further workup demonstrated hepatocellular dysfunction and liver biopsy showed expansion of portal tracts by lymphocytes and eosinophils consistent with a hypersensitivity reaction. Nafcillin and related antibiotics were withdrawn, and her symptoms resolved 3 months later. We searched PubMed using terms of "nafcillin cholestasis" and "nafcillin hepatitis", and a review of the literature showed other reports of nafcillin-induced hepatitis and cholestasis. Avoidance and on occasion the guarded use of glucocorticoids can lead to recovery... (truncated)

MESH:D010878 - piperacillin

Summary:

LLM Prediction Score	0.500 (normalized)
LLM Confidence Score	0.980
Golden Answer (Severity Class)	0.375 (normalized)
Prediction Error	0.125

Retrieved Context:

Title: [Clinical case of the month. Cholestatic hepatitis after administration of piperacillin].
We describe a patient suffering from infection of the upper respiratory tract, who was treated with a dose of 4 x 4 g of piperacillin over 10 days. Two days after the end of the treatment, she developed jaundice and had elevated alkaline phosphatase, gammaglutamyltransferase and transaminases. After exclusion of viral hepatitis, a vascular problem, and gall stone disease, the possibility of piperacillin-induced hepatitis was discussed. Lymphocyte transformation test for piperacillin was positive, suggesting an immunological mechanism for the observed hepatopathy. The patient was discharged a few days after in good clinical condition and with reduced liver values. Cholestasis gradually... (truncated)

Title: Signal Detection of Potential Hepatotoxic Drugs: Case-Control Study Using Both a Spontaneous Reporting System and Electronic Medical Records.
Drug-induced liver injury (DILI) is a common adverse drug event. Spontaneous reporting systems such as the Japanese Adverse Event Report Database (JADER) have been used to evaluate the association between drugs and adverse drug events. However, the association of drugs with adverse drug events may be overestimated due to reporting biases. Therefore, it is important to objectively evaluate the association using liver function test values. The aim of the present study was to predict potential hepatotoxic drugs using real-world data including electronic medical records and the JADER database. A total of 70009 (2779 with DILI and 67230 without DILI) and... (truncated)

Title: [Comparative study of cefpiramide + amikacin versus piperacillin + amikacin in granulocytopenic patients: a randomized, prospective study].
Cefpiramide + amikacin (CPM + AMK) was compared in a prospective randomized trial with our standard regimen of piperacillin plus amikacin (PIPC + AMK) as an empiric therapy for fever in 252 episodes of 141 patients with granulocytopenia. Initial profound granulocytopenia (fewer than 100/mm3 mature granulocytes) was present in approximately 69% of the patient trials in both treatment groups. Both groups were equally distributed in regard to age, disease, pretreatment WBC count and duration of antibiotic treatment, although septic shock was more frequently seen in CPM + AMK. Responses to PIPC + AMK and CPM + AMK were similar for... (truncated)

Title: An unusual case of piperacillin-tazobactam-induced fever, eosinophilia, thrombocytopenia and liver damage.
Piperacillin-tazobactam is a broad-spectrum antimicrobial agent that is commonly used in clinical practice. The development of delayed drug hypersensitivity reaction (DHR) has been reported in several cases previously. Here we describe an unusual case of non-immediate DHR due to a prolonged course of piperacillin-tazobactam. We report a 22-year-old man who developed fever, eosinophilia, thrombocytopenia and elevated hepatic enzymes following 17 days of piperacillin-tazobactam for methicillin-sensitive <i>Staphylococcus aureus</i> (MSSA) pneumonia. These adverse reactions were reversed immediately after antibiotic cessation. Our case highlights that clinicians should be aware of delayed adverse effects in patients receiving long-term piperacillin-tazobactam treatment.

Title: Stevens-Johnson syndrome presenting as intravenous line sepsis.
A 25-year-old Hispanic female with insulin dependent diabetes mellitus (IDDM) and endstage renal disease on chronic hemodialysis was hospitalized with paroxysms of fever and chills for a day. A day after starting piperacillin for presumed intravascular line infection, she developed a maculopapular dermatitis and abnormal liver function tests, at which point the drug was discontinued. However, the rash persisted for 10 days, after which it progressively worsened. She continued to have high fevers, abnormal liver function tests, and marked leukocytosis, despite multiple negative cultures and other nondiagnostic examinations. She was treated as a patient with sepsis of unknown etiology and... (truncated)

MESH:C554125 - interferon gamma-1b

Summary:

LLM Prediction Score	0.500 (normalized)
LLM Confidence Score	0.990
Golden Answer (Severity Class)	0.375 (normalized)
Prediction Error	0.125

Retrieved Context:

Title: Important role of proinflammatory cytokines/other endogenous substances in drug-induced hepatotoxicity: depression of drug metabolism during infections/inflammation states, and genetic polymorphisms of drug-metabolizing enzymes/cytokines may markedly contribute to this pathology.
Analysis of literature data on drug-induced hepatotoxicity reveals that often upper respiratory febrile illnesses and/or inflammation states precede liver injury/diseases related to administration of drugs or hepatotoxicity associated with administration of therapeutic doses of acetaminophen in some genetically predisposed subjects. The goals of this paper are to review the potential role of alterations in the balance between TH1 cells producing cytokines associated with a cell-mediated response and TH2 cells associated with an antibody response, as well as other endogenous substances, eg, growth factors, leading to a shift in immune response to one that may participate in the liver cells injury... (truncated)

Title: Sex-Specific Regulation of Interferon-γ Cytotoxicity in Mouse Liver by Autophagy.
Interferon-γ (IFNγ) is a central activator of immune responses in the liver and other organs. IFNγ triggers tissue injury and inflammation in immune diseases, which occur predominantly in females for unknown reasons. Recent findings that autophagy regulates hepatotoxicity from proinflammatory cytokines led to an examination of whether defective hepatocyte autophagy underlies sex-specific liver injury and inflammation induced by IFNγ.

Title: The role of the liver in the response to LPS: experimental and clinical findings.
The liver plays an important physiological role in lipopolysaccharide (LPS) detoxification and, in particular, hepatocytes are involved in the clearance of endotoxin of intestinal derivation. In experimental shock models, tumor necrosis factor (TNF)-alpha induces hepatocyte apoptosis and lethal effects are due to secreted TNF-alpha and not to cell-associated TNF-alpha. An exaggerated production of TNF-alpha has been reported in murine viral infections, in which mice become sensitized to low amounts of LPS and both interferon (IFN)-gamma and IFN-alpha/beta are involved in the macrophage-induced release of TNF-alpha. The prominent role of LPS and TNF-alpha in liver injury is also supported by studies... (truncated)

Title: Severe liver dysfunction possibly caused by the combination of interferon beta-1b therapy and melilot (sweet clover) supplement.
We report a case of severe liver dysfunction exacerbated after interferon beta (IFNB)-1b injection in a patient with multiple sclerosis (MS) who had been taking a melilot (sweet clover) supplement. Although IFNB-1b therapy for MS can cause mild liver dysfunction, severe hepatotoxicity attributable to supplement use has been reported.

Title: Synergistic Cytotoxicity from Drugs and Cytokines In Vitro as an Approach to Classify Drugs According to Their Potential to Cause Idiosyncratic Hepatotoxicity: A Proof-of-Concept Study.
Idiosyncratic drug-induced liver injury (IDILI) typically occurs in a small fraction of patients and has resulted in removal of otherwise efficacious drugs from the market. Current preclinical testing methods are ineffective in predicting which drug candidates have IDILI liability. Recent results suggest that immune mediators such as tumor necrosis factor-<i>α</i> (TNF) and interferon-<i>γ</i> (IFN) interact with drugs that cause IDILI to kill hepatocytes. This proof-of-concept study was designed to test the hypothesis that drugs can be classified according to their ability to cause IDILI in humans using classification modeling with covariates derived from concentration-response relationships that describe cytotoxic interaction with... (truncated)

MESH:D008730 - methoxsalen

Summary:

LLM Prediction Score	0.500 (normalized)
LLM Confidence Score	0.960
Golden Answer (Severity Class)	0.375 (normalized)
Prediction Error	0.125

Retrieved Context:

Title: Toxic hepatitis from oral methoxsalen photochemotherapy (PUVA).
A 59-year-old white woman with long-standing psoriasis, who had a damaged liver from methotrexate, developed a toxic hepatitis while on oral methoxsalen photochemotherapy (PUVA). Fever and marked elevation of liver enzymes occurred after the fifteenth PUVA treatment and spontaneously returned to normal within 5 days. One week later, following the next PUVA treatment, the same toxic hepatitis developed and again spontaneously resolved. Topical methoxsalen photochemotherapy with ultraviolet A (UVA) did not produce the toxic hepatitis and provided a suppression of the psoriasis on a maintenance basis.

Title: The drug methoxsalen, a suicide substrate for cytochrome P-450, decreases the metabolic activation, and prevents the hepatotoxicity, of carbon tetrachloride in mice.
Methoxsalen, a potent suicide inhibitor of cytochrome P-450 that can be used in humans, might be of value for the prevention of hepatitis in subjects with carbon tetrachloride poisoning. As a preliminary step, we have determined its effects on the hepatotoxicity of carbon tetrachloride in mice. Several monooxygenase activities, the in vitro covalent binding of carbon tetrachloride metabolites to microsomal proteins, and in vitro microsomal lipid peroxidation initiated by carbon tetrachloride metabolites were decreased by 60-90% in microsomes from mice killed 2 hr after the administration of methoxsalen (250 mumol X kg-1); microsomal lipid peroxidation mediated by endogenous iron and... (truncated)

Title: Protective Effects of Methoxsalen Supplementation on Chronic Alcohol-Induced Osteopenia and Steatosis in Rats.
Osteopenia or osteoporosis occurs frequently in alcoholics and patients with alcoholic fatty liver disease. Methoxsalen (MTS), 8-methoxypsoralen, improved osteoporosis in ovariectomized and diabetic mouse models; however, its effects on alcohol-induced osteopenia and steatosis have not been reported. This study examined the effects of MTS on alcohol-induced bone loss and steatosis. Rats in the alcohol groups were fed a Liber-DeCarli liquid diet containing 36% of its calories as alcohol. MTS was at 0.005% in their diet, while alendronate (positive control; 500 µg/kg BW/day) was administered orally for eight weeks. The pair-fed group received the same volume of isocaloric liquid diet containing... (truncated)

Title: 8-Methoxypsoralen disrupts MDR3-mediated phospholipids efflux and bile acid homeostasis and its relevance to hepatotoxicity.
Since its discovery in 1987, multidrug resistance 3 P-glycoprotein (MDR3) had recognized to play a crucial role in the translocation of phospholipids from the inner to outer leaflets of bile canalicular membranes. An increasing number of reports suggest that drug-mediated functional disruption of MDR3 is responsible for drug-induced cholestasis. 8-Methoxypsoralen (8-MOP) is used clinically to treat psoriasis, vitiligo and other skin disorders. However, psoralens safety for long-term use is a concern. In the current study, we evaluate 8-MOP's potential hepatotoxicity and effects on bile formation. Sprague Dawley (SD) rats were treated daily 200mg/kg or 400mg/kg of 8-MOP orally for 28... (truncated)

Title: Therapeutic Potential of Plants and Plant Derived Phytochemicals against Acetaminophen-Induced Liver Injury.
Acetaminophen (APAP), which is also known as paracetamol or <i>N</i>-acetyl-<i>p</i>-aminophenol is a safe and potent drug for fever, pain and inflammation when used at its normal therapeutic doses. It is available as over-the-counter drug and used by all the age groups. The overdose results in acute liver failure that often requires liver transplantation. Current clinical therapy for APAP-induced liver toxicity is the administration of <i>N</i>-acetyl-cysteine (NAC), a sulphydryl compound an approved drug which acts by replenishing cellular glutathione (GSH) stores in the liver. Over the past five decades, several studies indicate that the safety and efficacy of herbal extracts or... (truncated)

MESH:D000068579 - celecoxib

Summary:

LLM Prediction Score	0.500 (normalized)
LLM Confidence Score	0.990
Golden Answer (Severity Class)	0.375 (normalized)
Prediction Error	0.125

Retrieved Context:

Title: Prolonged cholestasis associated with short-term use of celecoxib.
Drug-induced liver injury due to celecoxib, a first generation Cox-2 inhibitor, has been rarely reported. We describe one case of severe and prolonged cholestasis after treatment with celecoxib for 12 days in a young woman with no evidence of other causes of liver disease or allergy. Jaundice lasted for 3 months, pruritus and abnormal liver biochemistry persisted for 18 months after stopping the drug. Liver biopsy specimens showed a cholestatic pattern of liver injury with only minimal mononuclear infiltrate in the portal tracts. This case report supports the notion that celecoxib may cause bland, long term cholestasis.

Title: The hepatic safety and tolerability of the novel cyclooxygenase-2 inhibitor celecoxib.
Celecoxib is a cyclooxygenase- (COX)-1-sparing inhibitor of COX-2 that is indicated for the treatment of osteoarthritis and rheumatoid arthritis. Many agents used for treating these diseases, both symptom-modifying and disease-modifying, are associated with the potential for hepatotoxicity. This article presents an analysis of the hepatic effects of celecoxib in 14 controlled studies of patients with arthritis (2 to 24 weeks' duration), in a long-term, open-label safety study (as long as 2 years), in 11 studies of patients receiving treatment for pain after oral or orthopedic surgery (up to 5 days' duration), and in five pharmacology studies. The overall incidence of... (truncated)

Title: Celecoxib-induced gastrointestinal, liver and brain lesions in rats, counteraction by BPC 157 or L-arginine, aggravation by L-NAME.
To counteract/reveal celecoxib-induced toxicity and NO system involvement.

Title: [Acute colitis closely following the beginning of celecoxib treatment].
Selective inhibitors of cyclooxygenase-2 have less gastroduodenal toxicity than non selective anti-inflammatory drugs. However, there is little information on their effect on the distal gut. A 91 year old woman presented with acute diarrhoea 5 weeks after beginning celecoxib treatment. Laboratory results showed an inflammatory syndrome and increased alanine aminotransferase (ALT) to 13 N. Endoscopic examination of the colon showed diffuse erythematous lesions of the sigmoid and of part of the right colon. No aetiology has been found for colitis or hepatitis. Diarrhea and blood test anomalies disappeared one week after celecoxib was stopped. The role of celecoxib in the... (truncated)

Title: [Hypersensitivity to celecoxib].
Celecoxib is a non-steroidal anti-inflammatory drug (NSAID) which acts via specific inhibition of cyclooxygenase-2 (synthesis of prostaglandins mediating pathological inflammation) but which preserves the homeostatic action of cyclooxygenase-1. The efficacy of celecoxib is similar to that of classical NSAIDs and its tolerance is comparable with that of placebo. We nevertheless observed one patient with hypersensitivity to celecoxib who developed fever, toxic skin reaction, and liver involvement.

MESH:D012631 - secobarbital

Summary:

LLM Prediction Score	0.500 (normalized)
LLM Confidence Score	0.980
Golden Answer (Severity Class)	0.375 (normalized)
Prediction Error	0.125

Retrieved Context:

Title: Prevalence and factors associated with potentially inappropriate medication use in older medicare beneficiaries with cancer.
To assess the factors related to potentially inappropriate medication (PIM) use in elderly patients with cancer, as well as to compare the PIM prevalence in older adults with and without cancer.

Title: Recommendations for Toxicological Investigation of Drug-Impaired Driving and Motor Vehicle Fatalities-2021 Update.
This report describes updates to the National Safety Council's Alcohol, Drugs and Impairment Division's recommendations for drug testing in driving under the influence of drug (DUID) cases and motor vehicle fatalities. The updates are based on a survey of drug testing practices in laboratories in the USA and Canada, a comprehensive review of the prior recommendations and data and research on drugs most frequently detected in DUID cases. A consensus meeting was held with representative forensic science practitioners and the authors of this report to update recommendations. No changes were made to the Tier I scope; however, there were changes... (truncated)

Title: Resin hemoperfusion for acute drug intoxication.
Eight patients with drug intoxication were hemoperfused on ten occasions at a blood-flow rate of 300 ml/min with a 650gm column of Amberlite XAD-4 resin, which is a macroreticular resin with a specific adsorptive attraction for lipid-soluble organic molecules. Column clearances of glutethimide and a variety of barbiturates ranged from 207 to 300 ml/min for treatment sessions extending from 2 1/2 to ten hours. After ingestion of 75 gm of glutethimide, one patient received hemoperfusion on three successive days for nine, ten, and eight hours, respectively. She recovered after the column removed over 30 gm of drug. The patients demonstrated... (truncated)

Title: SARS-CoV-2 Seroprevalence and Drug Use in Trauma Patients from Six Sites in the United States.
In comparison to the general patient population, trauma patients show higher level detections of bloodborne infectious diseases, such as Hepatitis and Human Immunodeficiency Virus. In comparison to bloodborne pathogens, the prevalence of respiratory infections such as SARS-CoV-2 and how that relates with other variables, such as drug usage and trauma type, is currently unknown in trauma populations. Here, we evaluated SARS-CoV-2 seropositivity and antibody isotype profile in 2,542 trauma patients from six Level-1 trauma centers between April and October of 2020 during the first wave of the COVID-19 pandemic. We found that the seroprevalence in trauma victims 18-44 years old... (truncated)

Title: A History of the Pharmacological Treatment of Bipolar Disorder.
In this paper, the authors review the history of the pharmacological treatment of bipolar disorder, from the first nonspecific sedative agents introduced in the 19th and early 20th century, such as solanaceae alkaloids, bromides and barbiturates, to John Cade's experiments with lithium and the beginning of the so-called "Psychopharmacological Revolution" in the 1950s. We also describe the clinical studies and development processes, enabling the therapeutic introduction of pharmacological agents currently available for the treatment of bipolar disorder in its different phases and manifestations. Those drugs include lithium salts, valproic acid, carbamazepine, new antiepileptic drugs, basically lamotrigine and atypical antipsychotic agents... (truncated)

MESH:D014212 - tretinoin

Summary:

LLM Prediction Score	0.500 (normalized)
LLM Confidence Score	0.990
Golden Answer (Severity Class)	0.375 (normalized)
Prediction Error	0.125

Retrieved Context:

Title: Fatal Hepatotoxicity Due to Viaminate.
Viaminate is a derivative of retinoic acid (tretinoin) used in the treatment of severe acne and other disorders of keratinization. Liver dysfunction due to viaminate therapy has not been well described and there have been no reported deaths due to viaminate-induced hepatotoxicity. Herein, we report the case of a woman who underwent treatment at the hospital for 38 days and died of hepatic failure after taking viaminate for more than 2 months. This case provides further insight into viaminate-induced hepatotoxicity.

Title: Effect of oral 13-cis-retinoic acid on serum lipids.
Serum lipid concentrations were measured in eighteen patients with severe acne before, during and after treatment with 0.8 mg/kg 13-cis-retinoic acid for 3 months. Cholesterol and triglyceride concentrations increased and HDL-cholesterol concentrations fell significantly during therapy. There were significant abnormalities of liver function and small decreases in indices of thyroid function. These changes had reverted to normal by 1 month after treatment. The data suggest that use of the drug in acne is likely to be limited by its toxicity.

Title: Mefloquine use, psychosis, and violence: a retinoid toxicity hypothesis.
Mefloquine use has been linked to severe gastrointestinal and neuropsychiatric adverse effects, including cognitive disturbances, anxiety, depression, psychosis, and violence. The adverse effects of the drug are thought to result from the secondary consequences of hepatocellular injury; in fact, mefloquine is known to cause a transient, anicteric chemical hepatitis. However, the mechanism of mefloquine-associated liver damage and the associated neuropsychiatric and behavioral effects of the drug are not well understood. Mefloquine and other 8-amino-quinolines are the only antimalarial drugs that target the liver-stage malaria parasites, which selectively absorb vitamin A from the host. Vitamin A is also stored mainly in... (truncated)

Title: Disturbed Vitamin A Metabolism in Non-Alcoholic Fatty Liver Disease (NAFLD).
Vitamin A is required for important physiological processes, including embryogenesis, vision, cell proliferation and differentiation, immune regulation, and glucose and lipid metabolism. Many of vitamin A's functions are executed through retinoic acids that activate transcriptional networks controlled by retinoic acid receptors (RARs) and retinoid X receptors (RXRs).The liver plays a central role in vitamin A metabolism: (1) it produces bile supporting efficient intestinal absorption of fat-soluble nutrients like vitamin A; (2) it produces retinol binding protein 4 (RBP4) that distributes vitamin A, as retinol, to peripheral tissues; and (3) it harbors the largest body supply of vitamin A, mostly as... (truncated)

Title: A study of potential hepatotoxicity of etretinate used in the treatment of psoriasis.
Etretinate was used to treat twenty patients who had severe, disabling psoriasis and an increased risk of liver damage. Potential hepatotoxicity was evaluated by obtaining liver biopsies prior to starting therapy and after a 6-month course on a dosage of 0.75 mg/kg/day. In comparing pretreatment biopsies to posttreatment biopsies, five of twenty patients demonstrated a morphologic change in their liver. Three showed progressive fatty metamorphosis, and two showed liver cell necrosis and progressive fibrosis. One of these was due to heavy alcohol intake. Based on our 6-month evaluation, etretinate does not produce a consistent toxic effect on the liver.

MESH:D005013 - ethosuximide

Summary:

LLM Prediction Score	0.500 (normalized)
LLM Confidence Score	0.980
Golden Answer (Severity Class)	0.375 (normalized)
Prediction Error	0.125

Retrieved Context:

Title: Antiepileptic Drugs and Liver Disease.
Acute, symptomatic seizures or epilepsy may complicate the course of hepatic disease. Choosing the most appropriate antiepileptic drug in this setting represents a difficult challenge, as most medications are metabolized by the liver. This article focuses on the acute and chronic treatment of seizures in patients with advanced liver disease and reviews the hepatotoxic potential of specific antiepileptic drugs. Newer antiepileptic drugs without, or with minimal, hepatic metabolism, such as levetiracetam, lacosamide, topiramate, gabapentin, and pregabalin should be used as first-line therapy. Medications undergoing extensive hepatic metabolism, such as valproic acid, phenytoin, and felbamate should be used as drugs of... (truncated)

Title: Use of antiepileptic drugs in hepatic and renal disease.
The use of antiepileptic drugs in patients with renal or hepatic disease is common in clinical practice. Since the liver and kidney are the main organs involved in the elimination of most drugs, their dysfunction can have important effects on the disposition of antiepileptic drugs. Renal or hepatic disease can prolong the elimination of the parent drug or an active metabolite leading to accumulation and clinical toxicity. It can also affect the protein binding, distribution, and metabolism of a drug. The protein binding of anionic acidic drugs, such as phenytoin and valproate, can be reduced significantly by renal failure, causing... (truncated)

Title: Hypersensitivity syndrome due to 2 anticonvulsant drugs.
Drug-induced hypersensitivity syndrome is a multiorgan-system reaction characterized by fever, pleomorphic eruption, lymphadenopathy, eosinophilia, lymphocytosis and hepatitis. We report a drug hypersensitivity syndrome in a 6-year-old Tunisian child treated for epileptic absences with sodium valproate and ethosuximide. Imputability of these 2 drugs is probable because of the chronological and clinical features. Positive rechallenge with ethosuximide confirmed the toxicity of this drug. Sodium valproate was also responsible because patch testing was positive and followed by a generalized eruption. Human herpesvirus 6 (HHV6) antibody titers increased significantly within 15 days. There was a favourable outcome after discontinuation of the drugs and corticosteroid... (truncated)

Title: Safety of anticonvulsants in hepatic porphyrias.
Because acute attacks of porphyria may be precipitated by anticonvulsants, a therapeutic dilemma arises when seizures complicate hepatic porphyria. The list of unsafe agents includes barbiturates, primidone, phenytoin, mephenytoin, ethotoin, ethosuximide, methsuximide, phensuximide, and trimethadione. Agents are considered unsafe if they induce experimental porphyria in animals, and short trials in patients are unreliable for screening. Using drug incubation in chick-embryo hepatocyte culture, we found that porphyrin was increased by carbamazepine, clonazepam, and valproate. These agents should probably be avoided or used with caution in porphyric patients. Alternative approaches to acute porphyric attacks with seizures are discussed.

Title: Synthesis, Molecular Docking, and Preclinical Evaluation of a New Succinimide Derivative for Cardioprotective, Hepatoprotective and Lipid-Lowering Effects.
Cardiac and hepatotoxicities are major concerns in the development of new drugs. Better alternatives to other treatments are being sought to protect these vital organs from the toxicities of these pharmaceuticals. In this regard, a preclinical study is designed to investigate the histopathological effects of a new succinimide derivative (Comp-1) on myocardial and liver tissues, and the biochemical effects on selected cardiac biomarkers, hepatic enzymes, and lipid profiles. For this, an initially lethal/toxic dose was determined, followed by a grouping of selected albino rats into five groups (each group had n = 6). The control group received daily oral saline... (truncated)

MESH:D000069438 - ezetimibe

Summary:

LLM Prediction Score	0.500 (normalized)
LLM Confidence Score	0.980
Golden Answer (Severity Class)	0.375 (normalized)
Prediction Error	0.125

Retrieved Context:

Title: Severe jaundice following treatment with ezetimibe.
Ezetimibe is a cholesterol-lowering agent that modulates intestinal absorption of sterols. It is well tolerated but hepatic toxicity has been reported when ezetimibe is used in conjunction with a statin medication. In this case report, we report severe isolated hyperbilirubinaemia occurring in a patient with occult cirrhosis, probably owing to nonalcoholic steatohepatitis, who was treated with ezetimibe alone. The adverse event started after ezetimibe therapy was initiated and resolved when the drug was stopped. We propose a mechanism for this reaction and believe that liver function should be monitored in patients with abnormal liver tests who are treated with ezetimibe,... (truncated)

Title: Recurrence and Severe Worsening of Hepatotoxicity After Reintroduction of Atorvastatin in Combination With Ezetimibe.
Severe hepatotoxicity is a rare but well-known adverse reaction to statins. However, despite the widespread use of statins, only a few cases describing statin reexposure or switch to another statin after liver injury have been published. The literature on hepatotoxicity with ezetimibe alone or in combination with statins is also scarce. We report a case where a patient with a history of elevated liver enzymes while using atorvastatin, but prior and subsequent good tolerance to simvastatin and pravastatin, developed drug-induced liver injury on reexposure to a combination of atorvastatin and ezetimibe. The hepatotoxicity in our patient was most likely caused... (truncated)

Title: Serious drug-induced liver disease secondary to ezetimibe.
Ezetimibe is the first member of a new family of lipid-lowering drugs that inhibits uptake of dietary and biliary cholesterol. It was approved by the FDA in 2002 for hypercholesterolemia alone or in combination with statins. Its use has been spreading over the last years. Ezetimibe was considered a safe drug. We report a case of a woman who developed a serious hepatocellular drug-induced liver disease after 4 mo therapy with 10 mg daily of ezetimibe. After withdrawal of the drug, the patient recovered slowly. Ezetimibe may produce serious toxic hepatitis and prompt withdrawal is mandatory in case of a... (truncated)

Title: Ezetimibe: its novel effects on the prevention and the treatment of cholesterol gallstones and nonalcoholic Fatty liver disease.
The cholesterol absorption inhibitor ezetimibe can significantly reduce plasma cholesterol concentrations by inhibiting the Niemann-Pick C1-like 1 protein (NPC1L1), an intestinal sterol influx transporter that can actively facilitate the uptake of cholesterol for intestinal absorption. Unexpectedly, ezetimibe treatment also induces a complete resistance to cholesterol gallstone formation and nonalcoholic fatty liver disease (NAFLD) in addition to preventing hypercholesterolemia in mice on a Western diet. Because chylomicrons are the vehicles with which the enterocytes transport cholesterol and fatty acids into the body, ezetimibe could prevent these two most prevalent hepatobiliary diseases possibly through the regulation of chylomicron-derived cholesterol and fatty acid... (truncated)

Title: Ezetimibe reduced hepatic steatosis induced by dietary oxysterols in nonhuman primates.
Oxidized cholesterol (oxysterols) plays an important and multifaceted role in lipid metabolism. Here we examined whether dietary oxysterols accelerate hepatic lipid accumulation and inflammation in nonhuman primates. We also examined the effect of the Niemann-Pick C1-like1 inhibitor, ezetimibe (Ez). <i>Macaca fascicularis</i> (5-year-old males) were fed either regular cholesterol + high-fat diet (control-HFD) or oxysterols + high-fat diet (ox-HFD; with 0.015% of oxysterols cholesterol) for 24 weeks. Compared with control-HFD, ox-HFD did not affect plasma lipid levels, but it did affect hepatic lipid levels [total cholesterol, 40.9 mg·g⁻¹ (ox-HFD) versus 3.2 (control-HFD) mg·g⁻¹; triglycerides, 28.0 (ox-HFD) versus 5.7 (control-HFD) mg·g⁻¹]. Ox-HFD... (truncated)

MESH:C092292 - ziprasidone

Summary:

LLM Prediction Score	0.500 (normalized)
LLM Confidence Score	0.980
Golden Answer (Severity Class)	0.375 (normalized)
Prediction Error	0.125

Retrieved Context:

Title: Effects of several atypical antipsychotics closapine, sertindole or ziprasidone on hepatic antioxidant enzymes: Possible role in drug-induced liver dysfunction.
Chronic use of atypical antipsychotics may produce hepatic damage. Atypical antipsychotics, including clozapine, sertindole, and ziprasidone, are extensively metabolized by the liver and this process generates toxic-free radical metabolic intermediates which may contribute to liver damage. The aim of this study was to investigate whether clozapine, sertindole, or ziprasidone affected hepatic antioxidant defense enzymes which consequently led to disturbed redox homeostasis. The expression and activity of antioxidant enzymes superoxide dismutase (SOD), glutathione peroxidase (GPx), glutathione reductase (GR), catalase (CAT), and glutathione-S-transferases (GST) were measured in rat livers at doses corresponding to human antipsychotic therapy. Clozapine increased activity of SOD types... (truncated)

Title: Successful treatment of psychosis induced by interferon alpha and ribavirin with paliperidone: first case reported.
Several clinical studies have shown a large number of mental symptoms by immunomodulatory treatment with interferon (IFN). The most frequently described symptoms are depression, suicidal behaviour, manic symptoms, anxiety, psychosis and delirium, associated with other non-specific symptoms such as fatigue, irritability, psychomotor retardation, decreased libido, insomnia, difficulty in concentration and attention. Having a history of mental disorder contraindicates the use of IFN-alpha. These adverse effects that affect the mental state appear usually at the beginning of the treatment (most after 3 weeks of treatment). The incidence of psychotic episodes is low and the episodes usually remit when treatment is interrupted;... (truncated)

Title: Atypical Antipsychotics and Metabolic Syndrome: From Molecular Mechanisms to Clinical Differences.
Atypical antipsychotics (AAPs) are commonly prescribed medications to treat schizophrenia, bipolar disorders and other psychotic disorders. However, they might cause metabolic syndrome (MetS) in terms of weight gain, dyslipidemia, type 2 diabetes (T2D), and high blood pressure, which are responsible for reduced life expectancy and poor adherence. Importantly, there is clear evidence that early metabolic disturbances can precede weight gain, even if the latter still remains the hallmark of AAPs use. In fact, AAPs interfere profoundly with glucose and lipid homeostasis acting mostly on hypothalamus, liver, pancreatic β-cells, adipose tissue, and skeletal muscle. Their actions on hypothalamic centers via dopamine,... (truncated)

Title: Risperidone Exacerbates Glucose Intolerance, Nonalcoholic Fatty Liver Disease, and Renal Impairment in Obese Mice.
Risperidone, a second-generation antipsychotic drug used for schizophrenia treatment with less-severe side effects, has recently been applied in major depressive disorder treatment. The mechanism underlying risperidone-associated metabolic disturbances and liver and renal adverse effects warrants further exploration. This research explores how risperidone influences weight, glucose homeostasis, fatty liver scores, liver damage, and renal impairment in high-fat diet (HFD)-administered C57BL6/J mice. Compared with HFD control mice, risperidone-treated obese mice exhibited increases in body, liver, kidney, and retroperitoneal and epididymal fat pad weights, daily food efficiency, serum triglyceride, blood urea nitrogen, creatinine, hepatic triglyceride, and aspartate aminotransferase, and alanine aminotransferase levels, and... (truncated)

Title: Safety Profile of Antipsychotic Drugs: Analysis Based on a Provincial Spontaneous Reporting Systems Database.
Introduction: Antipsychotic drugs are the main therapy for schizophrenia and have been widely used in mental disorder fields. However, the research on the safety of antipsychotic drugs in the real-world is rare. The purpose of this research is to evaluate the safety of antipsychotic drugs based on real-world data. Methods: ADR reports collected by the Henan Adverse Drug Reaction Monitoring Center from 2016 to 2020 were analyzed. We described the safety of antipsychotic drugs by descriptive analysis and four signal mining methods. Meanwhile, the risk factors for serious adverse reactions of antipsychotics were identified. Results: A total of 3363 ADR... (truncated)

MESH:D000068437 - pemetrexed

Summary:

LLM Prediction Score	0.500 (normalized)
LLM Confidence Score	0.980
Golden Answer (Severity Class)	0.375 (normalized)
Prediction Error	0.125

Retrieved Context:

Title: Correlation Between Methylenetetrahydrofolate Reductase (MTHFR) C677T Polymorphisms and Pemetrexed Chemotherapy Efficacy/Toxicity in Non-Squamous Non-Small Cell Lung Cancer.
BACKGROUND In the present study, we aimed to retrospectively analyze the correlation between toxicity of pemetrexed (PEM) chemotherapy and methylenetetrahydrofolate reductase (MTHFR) C677T polymorphisms in patients with advanced non-squamous non-small cell lung cancer (non-sq NSCLC). MATERIAL AND METHODS We used polymerase chain reaction, gene scanning, and restriction fragment length polymorphism to analyze MTHFR C677T in 51 patients with advanced non-sq NSCLC. The patients received chemotherapies with single-agent PEM (monotherapy group) or with PEM combined with cisplatin (joint group). The correlation between MTHFR C677T polymorphisms and chemotherapy efficacy/toxicity was also assessed. RESULTS There were 40 patients in the monotherapy group and... (truncated)

Title: Pemetrexed: new drug. Pleural mesothelioma: a first encouraging trial.
(1) Chemotherapy does not appear to prolong the survival of patients with inoperable pleural mesothelioma, and the tumour response rate barely exceeds 20%. A combination of cisplatin + doxorubicin seems to provide the best response rates. (2) In a trial of second-line docetaxel therapy in patients with non small cell lung cancer, survival was extended by about 3 months compared with palliative care (7.5 versus 4.6 months). (3) Pemetrexed, an antifolate closely related to methotrexate and raltitrexed, has been authorized for use for both conditions. (4) In a randomised single-blind trial involving 456 patients with previously untreated pleural mesothelioma, survival... (truncated)

Title: Approval summary: pemetrexed maintenance therapy of advanced/metastatic nonsquamous, non-small cell lung cancer (NSCLC).
On July 2, 2009, the U.S. Food and Drug Administration approved pemetrexed injection (Alimta® Injection; Eli Lilly and Company, Indianapolis, IN) for maintenance treatment of patients with locally advanced or metastatic nonsquamous non-small cell lung cancer whose disease has not progressed after four cycles of platinum-based doublet induction chemotherapy. A double-blind study of pemetrexed plus best supportive care versus placebo plus best supportive care was conducted. Pemetrexed, 500 mg/m(2) i.v., was administered every 21 days until disease progression. Folic acid, vitamin B(12), and a corticosteroid were given to all study patients. There were 663 randomized patients (pemetrexed, 441; placebo, 222).... (truncated)

Title: Effect of liver toxicity on clinical outcome of patients with non-small-cell lung cancer treated with pemetrexed.
Liver toxicity (LT) is a common side effect of pemetrexed (PEM); however, the effect of LT on clinical outcome has not been investigated in patients with non-small-cell lung cancer (NSCLC) treated with PEM. Between June, 2009 and June, 2012, a total of 95 chemo-naive NSCLC patients received a PEM-containing regimen in our hospital. We reviewed the medical records of those 95 patients and evaluated the incidence of LT. Furthermore, we investigated the association between LT and clinical outcome. In this analysis, LT was defined as any grade of aspartate aminotransferase or alanine aminotransferase elevation. A total of 67 patients (70.5%)... (truncated)

Title: Immune checkpoint inhibitor (ICI)-induced hepatitis diagnosed by liver biopsy followed by ICI-free chemotherapy leading to therapeutic effect: A case of lung cancer treatment.
In recent years, the combination of platinum-based chemotherapy and immune checkpoint inhibitors (ICIs) has become the standard treatment for patients with lung cancer. Hepatitis is one of the common toxicities following ICI/chemotherapy. When drug-induced hepatitis occurs, the suspected drug must be discontinued. Since it may be difficult to determine the exact drug causing the hepatitis, liver biopsy may help identify this. We report the case of a patient diagnosed with immune-related adverse event hepatitis from liver biopsy and clinical course. A 45-year-old man with lung adenocarcinoma (stage IV, cT4N3M1c) negative for driver gene mutation was treated with carboplatin (CBDCA), pemetrexed... (truncated)

MESH:D000657 - amoxapine

Summary:

LLM Prediction Score	0.500 (normalized)
LLM Confidence Score	0.890
Golden Answer (Severity Class)	0.375 (normalized)
Prediction Error	0.125

Retrieved Context:

Title: Addressing the Side Effects of Contemporary Antidepressant Drugs: A Comprehensive Review.
Randomized trials have shown that selective serotonin reuptake inhibitors (SSRIs) and serotonin-norepinephrine reuptake inhibitors (SNRIs) have better safety profiles than classical tricyclic antidepressants (TCAs). However, an increasing number of studies, including meta-analyses, naturalistic studies, and longer-term studies suggested that SSRIs and SNRIs are no less safe than TCAs. We focused on comparing the common side effects of TCAs with those of newer generation antidepressants including SSRIs, SNRIs, mirtazapine, and bupropion. The main purpose was to investigate safety profile differences among drug classes rather than the individual antidepressants, so studies containing comparison data on drug groups were prioritized. In terms of... (truncated)

Title: Antimicrobial Properties of Antidepressants and Antipsychotics-Possibilities and Implications.
The spreading of antibiotic resistance is responsible annually for over 700,000 deaths worldwide, and the prevision is that this number will increase exponentially. The identification of new antimicrobial treatments is a challenge that requires scientists all over the world to collaborate. Developing new drugs is an extremely long and costly process, but it could be paralleled by drug repositioning. The latter aims at identifying new clinical targets of an "old" drug that has already been tested, approved, and even marketed. This approach is very intriguing as it could reduce costs and speed up approval timelines, since data from preclinical studies... (truncated)

Title: Prediction of Drug-Induced Hepatotoxicity Using Long-Term Stable Primary Hepatic 3D Spheroid Cultures in Chemically Defined Conditions.
High failure rates of drug candidates in the clinics, restricted-use warnings as well as withdrawals of drugs in postmarketing stages are of substantial concern for the pharmaceutical industry and drug-induced liver injury (DILI) constitutes one of the most frequent reasons for such safety failures. Importantly, as DILI cannot be accurately predicted using animal models, animal safety tests are commonly complemented with assessments in human in vitro systems. 3D spheroid cultures of primary human hepatocytes in chemically defined conditions, hereafter termed CD-spheroids, have recently emerged as a microphysiological model system in which hepatocytes retain their molecular phenotypes and hepatic functions for... (truncated)

Title: Severe skin complications in patients treated with antidepressants: a literature review.
Clinical manifestations of drug-induced skin reactions include a wide range of symptoms, from mild drug-induced exanthemas to dangerous and life-threatening generalized systematic reactions. Adverse drug reactions of low risk include phenomena such as drug-induced rashes, phototoxic reactions, eczemas and urticarias, which appear most often when the medication is being introduced. Drug-induced skin reactions to psychotropic medication are usually associated with antiepileptic drugs. However, a significant role can be assigned to selective serotonin reuptake inhibitors. The aim of this paper is to review a spectrum of severe skin complications in patients treated with antidepressants with the indication of their clinical monitoring... (truncated)

Title: Use of Prescribed Psychotropics during Pregnancy: A Systematic Review of Pregnancy, Neonatal, and Childhood Outcomes.
This paper reviews the findings from preclinical animal and human clinical research investigating maternal/fetal, neonatal, and child neurodevelopmental outcomes following prenatal exposure to psychotropic drugs. Evidence for the risks associated with prenatal exposure was examined, including teratogenicity, neurodevelopmental effects, neonatal toxicity, and long-term neurobehavioral consequences (i.e., behavioral teratogenicity). We conducted a comprehensive review of the recent results and conclusions of original research and reviews, respectively, which have investigated the short- and long-term impact of drugs commonly prescribed to pregnant women for psychological disorders, including mood, anxiety, and sleep disorders. Because mental illness in the mother is not a benign event,... (truncated)

MESH:D004009 - dicloxacillin

Summary:

LLM Prediction Score	0.500 (normalized)
LLM Confidence Score	0.960
Golden Answer (Severity Class)	0.375 (normalized)
Prediction Error	0.125

Retrieved Context:

Title: Hepatic safety of antibiotics used in primary care.
Antibiotics used by general practitioners frequently appear in adverse-event reports of drug-induced hepatotoxicity. Most cases are idiosyncratic (the adverse reaction cannot be predicted from the drug's pharmacological profile or from pre-clinical toxicology tests) and occur via an immunological reaction or in response to the presence of hepatotoxic metabolites. With the exception of trovafloxacin and telithromycin (now severely restricted), hepatotoxicity crude incidence remains globally low but variable. Thus, amoxicillin/clavulanate and co-trimoxazole, as well as flucloxacillin, cause hepatotoxic reactions at rates that make them visible in general practice (cases are often isolated, may have a delayed onset, sometimes appear only after cessation... (truncated)

Title: Hepatotoxicity of antibiotics.
Several antibiotics can cause severe hepatic injury. It is the purpose of this paper to review the main antibiotics that can cause hepatic injury and discuss the presentation, pattern, and outcome of hepatic injury. In the case of the penicillins, the combination amoxycillin-clavulanate and the penicillinase-resistant penicillins oxacillin, (di-)cloxacillin, and flucloxacillin can cause (mainly cholestatic) hepatitis. Cephalosporins have little hepatotoxicity; ceftriaxone can cause drug-induced gallstones. The potential of erythromycin and several other macrolides to cause (usually cholestatic) hepatitis is well established. Tetracyclines can cause a syndrome mimicking acute fatty liver of pregnancy, but this complication has virtually disappeared. Quinolones seem... (truncated)

Title: IL-4 mediates dicloxacillin-induced liver injury in mice.
Drug-induced liver injury (DILI) is a major problem in drug development and clinical drug therapy. In most cases, the mechanisms are still unknown. It is difficult to predict DILI in humans due to the lack of experimental animal models. Dicloxacillin, penicillinase-sensitive penicillin, rarely causes cholestatic or mixed liver injury, and there is some evidence for immunoallergic idiosyncratic reaction in human. In this study, we investigated the mechanisms of dicloxacillin-induced liver injury. Plasma ALT and total-bilirubin (T-Bil) levels were significantly increased in dicloxacillin-administered (600 mg/kg, i.p.) mice. Dicloxacillin administration induced Th2 (helper T cells)-mediated factors and increased the plasma interleukin (IL)-4... (truncated)

Title: Human leukocyte antigen (HLA)-B*57:01-restricted activation of drug-specific T cells provides the immunological basis for flucloxacillin-induced liver injury.
The role of the adaptive immune system in adverse drug reactions that target the liver has not been defined. For flucloxacillin, a delay in the reaction onset and identification of human leukocyte antigen (HLA)-B*57:01 as a susceptibility factor are indicative of an immune pathogenesis. Thus, we characterize flucloxacillin-responsive CD4+ and CD8+ T cells from patients with liver injury and show that naive CD45RA+CD8+ T cells from volunteers expressing HLA-B*57:01 are activated with flucloxacillin when dendritic cells present the drug antigen. T-cell clones expressing CCR4 and CCR9 migrated toward CCL17 and CCL 25, and secreted interferon-gamma (IFN-γ), T helper (Th)2 cytokines,... (truncated)

Title: Nafcillin-Induced Allergic Eosinophilic Cholestatic Hepatitis.
A 71-year-old female with no history of liver disease or antibiotic allergy developed jaundice with elevated liver enzymes and eosinophilia following treatment with nafcillin for septic arthritis. Further workup demonstrated hepatocellular dysfunction and liver biopsy showed expansion of portal tracts by lymphocytes and eosinophils consistent with a hypersensitivity reaction. Nafcillin and related antibiotics were withdrawn, and her symptoms resolved 3 months later. We searched PubMed using terms of "nafcillin cholestasis" and "nafcillin hepatitis", and a review of the literature showed other reports of nafcillin-induced hepatitis and cholestasis. Avoidance and on occasion the guarded use of glucocorticoids can lead to recovery... (truncated)

MESH:D013726 - terbutaline

Summary:

LLM Prediction Score	0.500 (normalized)
LLM Confidence Score	0.990
Golden Answer (Severity Class)	0.375 (normalized)
Prediction Error	0.125

Retrieved Context:

Title: Critical periods for the role of oxidative stress in the developmental neurotoxicity of chlorpyrifos and terbutaline, alone or in combination.
The developmental neurotoxicity of chlorpyrifos (CPF) involves mechanisms other than inhibition of cholinesterase. In the current study, we examined the ability of CPF to evoke lipid peroxidation in the developing brain of fetal and neonatal rats. CPF given to pregnant rats on gestational days 17-20 or to neonatal rats on postnatal days 1-4, failed to elicit increases in thiobarbituric acid-reactive species (TBARS) in brain regions even when the dose was raised above the threshold for systemic toxicity and hepatic damage. In contrast, CPF administration during the second postnatal week, the peak period of neuronal cell differentiation and synaptogenesis, did evoke... (truncated)

Title: Maternal liver impairment associated with prolonged high-dose administration of terbutaline for premature labor.
Widespread use of beta 2-sympathomimetic therapy for premature labor has resulted in a diverse number of serious maternal complications, ie, pulmonary edema, myocardial ischemia, etc. However, to the authors' knowledge, there has been only one previous report of mild maternal liver impairment associated with this therapy. Recently, a patient developed marked elevation of liver enzymes (serum glutamic oxaloacetic transaminase, serum glutamic pyruvic transaminase) during therapy with terbutaline sulfate for premature labor.

Title: A rare case of cefepime-induced cholestatic liver injury.
Cefepime is widely used in the hospital setting, and only a few studies have reported neurotoxicity and nephrotoxicity as side effects of this drug. Herein, we present a 93-year-old man who exhibited features of cholestatic hepatitis including elevated blood transaminases and direct-form predominant bilirubin levels after administration of cefepime. Blood liver tests showed total recovery after discontinuing the offending agent. Cefepime was probable to cause drug-induced cholestatic hepatitis in our patient since the Roussel Uclaf Causality Assessment Method score for cefepime was 7. No drug interactions were likely according to the Drug Interaction Probability Scale for this patient. No similar... (truncated)

Title: Hepatotoxicity with the administration of nifedipine for treatment of preterm labor.
Nifedipine, a calcium channel blocker, is becoming increasingly popular as a tocolytic medication. Previous reports of hepatitis associated with nifedipine in the medical literature have been published. We present the first case reported of a patient with preterm labor treated with nifedipine who had development of a concurrent elevation in serum liver enzyme levels.

Title: Computational Models Using Multiple Machine Learning Algorithms for Predicting Drug Hepatotoxicity with the DILIrank Dataset.
Drug-induced liver injury (DILI) remains one of the challenges in the safety profile of both authorized and candidate drugs, and predicting hepatotoxicity from the chemical structure of a substance remains a task worth pursuing. Such an approach is coherent with the current tendency for replacing non-clinical tests with in vitro or in silico alternatives. In 2016, a group of researchers from the FDA published an improved annotated list of drugs with respect to their DILI risk, constituting "the largest reference drug list ranked by the risk for developing drug-induced liver injury in humans" (DILIrank). This paper is one of the... (truncated)

MESH:D013015 - sotalol

Summary:

LLM Prediction Score	0.250 (normalized)
LLM Confidence Score	0.990
Golden Answer (Severity Class)	0.375 (normalized)
Prediction Error	0.125

Retrieved Context:

Title: [Chronic hepatitis ascribed to the use of sotalol].
In a 68-year-old woman with severe chronic hepatitis an extensive investigation revealed no other cause than the use of sotalol for 10 months due to atrial fibrillation. Once the use of the medication had been discontinued the patient's symptoms quickly disappeared and the liver function disorders normalised within 5 months. Sotalol is a beta-adrenergic receptor blocking and anti-arrhythmic agent. It is widely used in patients with supraventricular and ventricular arrhythmias. An adverse effect in terms of liver damage is not known. The pathogenesis of the observed hepatitis remained an enigma because sotalol is a hydrophilic substance which is not metabolized... (truncated)

Title: Drug-specific risk of severe QT prolongation following acute drug overdose.
Background: Severe QT prolongation (SQTP) has been identified as a strong predictor of adverse cardiovascular events in acute drug overdose, but drug-specific causes of SQTP in the setting of acute drug overdose remain unclear. We aimed to perform the most definitive study to date describing drug-specific risk of SQTP following acute drug overdose.Methods: This was a prospective multicenter cohort study at >50 hospital sites across the US using the ToxIC Registry between 2015 and 2018. Inclusion criteria were adults (≥18 years) receiving medical toxicology consultation for acute drug overdose. The primary outcome was SQTP, which was defined using the computer... (truncated)

Title: Prolonged Jaundice Secondary to Amiodarone Use: A Case Report and Literature Review.
Adverse reactions to the antiarrhythmic medication amiodarone are severe, potentially life-threatening, and not rare. One in three patients on long-term therapy experience elevated liver enzymes, and clinically apparent liver toxicity occurs in 1% of patients treated. We report the case of a 76-year-old patient with amiodarone-induced intrahepatic cholestasis and prolonged hyperbilirubinemia despite the discontinuation of the offending agent. Current research hypothesizes that amiodarone leads to hepatic injury both by direct hepatotoxicity and by increasing the likelihood of hepatocytes to create abnormal, toxic metabolites. Increased awareness of such an adverse effect can guide clinicians toward the possible underlying etiologies of prolonged... (truncated)

Title: Amiodarone-induced pulmonary toxicity--a fatal case report and literature review.
Amiodarone is a widely used and very potent antiarrhythmic substance. Among its adverse effects, pulmonary toxicity is the most dangerous without a causal treatment option. Due to a very long half-life, accumulation can only be prevented by strict adherence to certain dosage patterns. In this review, we outline different safe and proven dosing schemes of amiodarone and compare the incidence and description of pulmonary toxicity. Reason for this is a case of fatal pulmonary toxicity due to a subacute iatrogenic overdosing of amiodarone in a 74-year-old male patient with known severe coronary artery disease, congestive heart failure and ectopic atrial... (truncated)

Title: Antiarrhythmic Drug Dosing in Children-Review of the Literature.
Antiarrhythmic drugs represent a mainstay of pediatric arrhythmia treatment. However, official guidelines and consensus documents on this topic remain scarce. There are rather uniform recommendations for some medications (including adenosine, amiodarone, and esmolol), while there are only very broad dosage recommendations for others (such as sotalol or digoxin). To prevent potential uncertainties and even mistakes with regard to dosing, we summarized the published dosage recommendations for antiarrhythmic drugs in children. Because of the wide variations in availability, regulatory approval, and experience, we encourage centers to develop their own specific protocols for pediatric antiarrhythmic drug therapy.

MESH:D007660 - ketoprofen

Summary:

LLM Prediction Score	0.500 (normalized)
LLM Confidence Score	0.980
Golden Answer (Severity Class)	0.375 (normalized)
Prediction Error	0.125

Retrieved Context:

Title: Ketoprofen hypersensibility and idiosyncratic response - a case report.
Context: Ketoprofen is widely used to remove pain. A steady increase on allergic reactions and photoallergic contact dermatitis related to ketoprofen has been reported when there is topical use. However, there are few documented cases of hypersensitivity when it is administered systemically.Objective: Present a case of hypersensitivity reaction after systemic administration of ketoprofen for pain control in nephritis crisis.Case description: A 43-years-old Caucasian man diagnosed with renal colic (kidney lithiasis), who was initially treated with 100 mg of tramadol (IV), followed by 4 mg of thiocolchicoside (IM) which caused no relief. Then 100 mg of ketoprofen was administered (IV). Right after the patient... (truncated)

Title: Nonsteroidal anti-inflammatory drug-induced liver injury: a case-control study in primary care.
Several nonsteroidal anti-inflammatory drugs (NSAIDs) have been withdrawn from the market because of hepatic adverse drug reactions (ADRs). Moreover, some cases of liver diseases have been reported in patients taking NSAIDs (arylcarboxylic NSAIDs, piroxicam, sulindac, nimesulide, etc.). Pharmacoepidemiological studies have shown a risk of hepatic ADRs with NSAIDs used in association with other hepatotoxic drugs. In contrast, other studies performed in hospitalized patients did not found any association. The aim of this study was to assess the hepatic risk associated with the use of NSAID in the setting of primary care. The study design was a case-control study where cases... (truncated)

Title: The use and effect of analgesics in patients who regularly drink alcohol.
Analgesic consumption poses special risks for regular users of alcohol. Among the numerous adverse health effects are acetaminophen toxicity and gastrointestinal (GI) bleeding associated with nonsteroidal anti-inflammatory drug (NSAID) use. An alcohol-acetaminophen hypothesis contends that alcohol enhances acetaminophen toxicity. Because 22% of adults use acetaminophen each week and 5% to 10% of the population is alcoholic, the healthcare implications of serious adverse interactions are considerable. However, such interactions are rare when NSAID doses remain in the therapeutic range. Although clinical studies fail to support anecdotal case reports of liver damage associated with consumption of therapeutic doses of acetaminophen by alcohol... (truncated)

Title: The problems and pitfalls of NSAID therapy in the elderly (Part I).
Nonsteroidal anti-inflammatory drugs (NSAIDs) are the most commonly prescribed drugs worldwide when grouped by generic categories and account for 3 to 9% of total prescription numbers in various countries. While NSAIDs are responsible for approximately 25% of all reported adverse drug reactions, aging may substantially increase the risk of NSAID-induced reactions. Several factors may contribute to NSAID-related toxicity in the elderly. The increase in morbidity associated with aging may result in consumption of a wide range of potent drugs, while inappropriate drug therapy and aberrant compliance are also capable of contributing to adverse drug reactions in geriatric patients. Age-related alterations... (truncated)

Title: Gastrointestinal safety and tolerability of oral non-aspirin over-the-counter analgesics.
Over-the-counter (OTC) analgesics are routinely used worldwide for self-management of various painful conditions. Despite this, there has been little in-depth review of the safety of non-aspirin analgesics at OTC doses. This paper reviews the available literature on the gastrointestinal (GI) and hepatic safety of non-aspirin OTC analgesics, including nonsteroidal anti-inflammatory drugs (NSAIDs; ibuprofen, ketoprofen, diclofenac, and naproxen) and acetaminophen; safety in overdose is also reviewed. Each non-aspirin OTC analgesic has a distinct adverse event (AE) profile, with GI AE rates for OTC dosing in one study ranging from 37% for diclofenac to 7.2% for ibuprofen and 7.6% for acetaminophen; GI... (truncated)

MESH:D011344 - procarbazine

Summary:

LLM Prediction Score	0.500 (normalized)
LLM Confidence Score	0.980
Golden Answer (Severity Class)	0.375 (normalized)
Prediction Error	0.125

Retrieved Context:

Title: Procarbazine-induced hepatotoxicity: case report and review of the literature.
Procarbazine hydrochloride is an oral alkylating agent primarily used as a component of chemotherapy regimens for Hodgkin's lymphoma, as well as in regimens for primary central nervous system lymphoma and high-grade gliomas. Although the prescribing information for procarbazine lists hepatic dysfunction as a potential adverse reaction, we found only one published report with a probable link between procarbazine and liver injury. We describe a 65-year-old man who developed liver injury due to procarbazine during salvage chemotherapy for non-Hodgkin's lymphoma. The patient had no preexisting liver disease, his lymphoma was without hepatic involvement, and no liver injury developed after initial chemotherapy... (truncated)

Title: Toxicity Profile of Procarbazine Lomustine and Vincristine Chemotherapy in Low-Grade Glioma - Retrospective Review.
Background The role of Procarbazine Lomustine and Vincristine (PCV) chemotherapy is already established in terms of improving survival in low-grade glioma (LGG). This improved survival has led to the increasing administration of PCV to LGG patients over the past years. However, like other chemotherapies, serious hematological and non-hematological toxicities may occur. The purpose of this study was to evaluate the toxicity profile of PCV and its clinical relevance in our practice. Materials and Methods We reviewed 63 patients of LGG retrospectively who received chemotherapy PCV between January 2015 and January 2018 at Shaukat Khanum Memorial Cancer Hospital & Research Centre,... (truncated)

Title: Susceptibility of nonhuman primates to carcinogens of human relevance.
Nonhuman primates are a valuable experimental model for the evaluation of human carcinogenic risk but have not been widely used for various reasons, such as high cost and lack of availability. The present review discusses the findings from a long-term carcinogenesis study in nonhuman primates that was carried out under contract by the National Cancer Institute from 1961 to 1997. Among the classes of compounds investigated were model rodent carcinogens, food additives, food and environmental contaminants, heterocyclic amines, N-nitroso compounds, and antineoplastic and immunosuppressives. Of the model rodent carcinogens tested, only urethane was carcinogenic in monkeys. Long-term administration of saccharin... (truncated)

Title: First reported case of disease: peliosis hepatis as cardinal symptom of Hodgkin's lymphoma.
We present the case of a 25-year-old woman with a history of weakness, weight loss, anemia, and elevated liver enzymes. Outpatient diagnostic evaluation, including abdominal ultrasound and endoscopies, revealed no conclusive explanation for the clinical picture and the patient was admitted to our clinic. Because of the hepatosplenomegaly together with the elevated liver enzymes, one of our differential diagnoses was that of liver disease. To clarify this, we performed a minilaparoscopy, which showed multiple diffuse distributed spots of livid color without clear margins distributed all over both liver lobes. A biopsy taken from these areas revealed the diagnosis of peliosis... (truncated)

Title: [Late data in pediatric oncology].
This work reviews the most frequent late effects seen in long-term survivors and how they relate to individual therapeutic modalities: a) Growth: severe growth retardation is seen in patients treated by radiation therapy, related to dose, anatomical site and age of patient, along with bony abnormalities (scoliosis, atrophy or hypoplasia, osteoporosis). b) Fertility: chemotherapy, in particular alkylating agents and the methylhydrazine procarbazine, can interfere with gonadal function, especially when administered with abdomen and pelvic irradiation. This effect is often seen in Hodgkin disease. c) Cardiovascular function: the anthracyclines cardiotoxicity is well known and most commonly presents with cardiomyopathy, pericarditis or... (truncated)

MESH:D000077723 - cefepime

Summary:

LLM Prediction Score	0.500 (normalized)
LLM Confidence Score	0.990
Golden Answer (Severity Class)	0.375 (normalized)
Prediction Error	0.125

Retrieved Context:

Title: A rare case of cefepime-induced cholestatic liver injury.
Cefepime is widely used in the hospital setting, and only a few studies have reported neurotoxicity and nephrotoxicity as side effects of this drug. Herein, we present a 93-year-old man who exhibited features of cholestatic hepatitis including elevated blood transaminases and direct-form predominant bilirubin levels after administration of cefepime. Blood liver tests showed total recovery after discontinuing the offending agent. Cefepime was probable to cause drug-induced cholestatic hepatitis in our patient since the Roussel Uclaf Causality Assessment Method score for cefepime was 7. No drug interactions were likely according to the Drug Interaction Probability Scale for this patient. No similar... (truncated)

Title: Influence of Antibiotics on Functionality and Viability of Liver Cells In Vitro.
(1) Antibiotics are an important weapon in the fight against serious bacterial infections and are considered a common cause of drug-induced liver injury (DILI). The hepatotoxicity of many drugs, including antibiotics, is poorly analyzed in human in vitro models. (2) A standardized assay with a human hepatoma cell line was used to test the hepatotoxicity of various concentrations (Cmax, 5× Cmax, and 10× Cmax) of antibiotics. In an ICU, the most frequently prescribed antibiotics, ampicillin, cefepime, cefuroxime, levofloxacin, linezolid, meropenem, rifampicin, tigecycline, and vancomycin, were incubated with HepG2/C3A cells for 6 days. Cell viability (XTT assay, LDH release, and vitality),... (truncated)

Title: A Case-Report of Drug-Induced Mixed Liver Injury Resulting From Cefepime Exposure.
A 99-year-old African-American male presented to the hospital with severe sepsis secondary to a urinary tract infection. Upon initial presentation he was tachycardic, hypotensive and had leukocytosis. While he had signs of acute kidney injury, no signs of acute liver injury were present with his alanine transferase (ALT) and amino transferase (AST) levels measuring at 22 and 44 U/L, respectively. During the treatment course the patient began to show signs of clinical improvement. Despite this, his ALT and AST began to increase on day 2 of treatment and reached their peak of 210 and 239 U/L on day 4. Cefepime-induced... (truncated)

Title: Hypersensitivity Pneumonitis Caused by Cephalosporins With Identical R1 Side Chains.
Drug-induced hypersensitivity pneumonitis results from interactions between pharmacologic agents and the human immune system. We describe a 54-year-old man with hypersensitivity pneumonitis caused by cephalosporins with identical R1 side chains. The patient, who complained of cough with sputum, was prescribed ceftriaxone and clarithromycin at a local clinic. The following day, he complained of dyspnea, and chest X-ray revealed worsening of inflammation. Upon admission to our hospital, antibiotics were changed to cefepime with levofloxacin, but his pneumonia appeared to progress. Changing antibiotics to meropenem with ciprofloxacin improved his symptoms and radiologic findings. Antibiotics were de-escalated to ceftazidime with levofloxacin, and his... (truncated)

Title: Delayed diagnosis of cholestatic drug-induced liver injury treated with corticosteroid for adrenal insufficiency secondary to miliary tuberculosis.
Drug-induced liver injury (DILI) in a patient with multiple comorbidities is often challenging to diagnose because liver injury can be attributed to multiple disease processes. Delayed treatment of DILI could have fatal consequences and, therefore, understanding the features and risks of DILI is crucial. We report a unique case of a patient who was admitted for severe sepsis of unknown etiology. This patient was later found to have miliary tuberculosis (TB) with associated adrenal insufficiency, complicated by acute cholestatic liver injury. Liver injury fully improved after initiation of corticosteroid for the treatment of adrenal insufficiency. The most likely pathophysiology of... (truncated)

MESH:D019791 - guanidine

Summary:

LLM Prediction Score	0.500 (normalized)
LLM Confidence Score	0.980
Golden Answer (Severity Class)	0.375 (normalized)
Prediction Error	0.125

Retrieved Context:

Title: In Vitro and In Vivo Evaluation of the Toxic Effects of Dodecylguanidine Hydrochloride.
The toxicity profiles of the widely used guanidine-based chemicals have not been fully elucidated. Herein, we evaluated the in vitro and in vivo toxicity of eight guanidine-based chemicals, focusing on inhalation toxicity. Among the eight chemicals, dodecylguanidine hydrochloride (DGH) was found to be the most cytotoxic (IC₅₀: 0.39 μg/mL), as determined by the water soluble tetrazolium salts (WST) assay. An acute inhalation study for DGH was conducted using Sprague-Dawley rats at 8.6 ± 0.41, 21.3 ± 0.83, 68.0 ± 3.46 mg/m³ for low, middle, and high exposure groups, respectively. The levels of lactate dehydrogenase, polymorphonuclear leukocytes, and cytokines (MIP-2, TGF-β1,... (truncated)

Title: Disruption of Membrane Integrity as a Molecular Initiating Event Determines the Toxicity of Polyhexamethylene Guanidine Phosphate Depending on the Routes of Exposure.
Polyhexamethylene guanidine phosphate (PHMG-P), a cationic biocide, is widely used in household products due to its strong bactericidal activity and low toxicity. However, it causes fatal lung damage when inhaled. In this study, we investigated why PHMG-P causes fatal lung injury when inhaled, and demonstrated that the disruption of membrane integrity through ionic interaction-a molecular initiating event of PHMG-P-determines toxicity. Mice were injected intravenously with 0.9 or 7.2 mg/kg PHMG-P (IV group), or instilled intratracheally with 0.9 mg/kg PHMG-P (ITI group); they were euthanized at 4 h and on days 1 and 7 after treatment. Increased total BAL cell count... (truncated)

Title: N, N', N″-trisubstituted guanidines: Synthesis, characterization and evaluation of their leishmanicidal activity.
Leishmaniasis is a group of diseases caused by protozoan parasites from the genus Leishmania. There are estimated 1.3 million new cases annually with a mortality of 20,000-30,000 per year, when patients are left untreated. Current chemotherapeutic drugs available present high toxicity and low efficacy, the latter mainly due to the emergence of drug-resistant parasites, which makes discovery of novel, safe, and efficacious antileishmanial drugs mandatory. The present work reports the synthesis, characterization by ESI-MS, ¹H and ¹³C NMR, and FTIR techniques as well as in vitro and in vivo evaluation of leishmanicidal activity of guanidines derivatives presenting lower toxicity. Among ten investigated... (truncated)

Title: Metformin selectively targets redox control of complex I energy transduction.
Many guanide-containing drugs are antihyperglycaemic but most exhibit toxicity, to the extent that only the biguanide metformin has enjoyed sustained clinical use. Here, we have isolated unique mitochondrial redox control properties of metformin that are likely to account for this difference. In primary hepatocytes and H4IIE hepatoma cells we found that antihyperglycaemic diguanides DG5-DG10 and the biguanide phenformin were up to 1000-fold more potent than metformin on cell signalling responses, gluconeogenic promoter expression and hepatocyte glucose production. Each drug inhibited cellular oxygen consumption similarly but there were marked differences in other respects. All diguanides and phenformin but not metformin inhibited... (truncated)

Title: Inhibition of CYP2E1 and activation of Nrf2 signaling pathways by a fraction from <i>Entada africana</i> alleviate carbon tetrachloride-induced hepatotoxicity.
<i>Entada africana</i> is used in non-conventional medicine for the management of liver ailments. A fraction, designated EaF10 (methylene chloride/methanol 90:10, v/v) with promising hepatoprotective activity has been isolated. Since the mechanisms underlying EaF10 hepatoprotective action remain unknown, this study was undertaken to investigate the anti-hepatotoxic mechanism of the fraction against carbon tetrachloride (CCl₄)-induced hepatotoxicity and its antioxidant properties. Antioxidant activities of EaF10 were assessed through four chemical antioxidant assays and its anti-hepatotoxic effect evaluated <i>in vivo</i> and <i>in vitro</i> by post-treatment (25 or 100 mg/Kg) or co-treatment (6.25-100 μg/mL) in CCl₄-intoxicated mice and normal human liver cells line L-02 hepatocytes... (truncated)

MESH:D013713 - teniposide

Summary:

LLM Prediction Score	0.500 (normalized)
LLM Confidence Score	0.960
Golden Answer (Severity Class)	0.375 (normalized)
Prediction Error	0.125

Retrieved Context:

Title: PhytoNanotechnology: Enhancing Delivery of Plant Based Anti-cancer Drugs.
Natural resources continue to be an invaluable source of new, novel chemical entities of therapeutic utility due to the vast structural diversity observed in them. The quest for new and better drugs has witnessed an upsurge in exploring and harnessing nature especially for discovery of antimicrobial, antidiabetic, and anticancer agents. Nature has historically provide us with potent anticancer agents which include vinca alkaloids [vincristine (VCR), vinblastine, vindesine, vinorelbine], taxanes [paclitaxel (PTX), docetaxel], podophyllotoxin and its derivatives [etoposide (ETP), teniposide], camptothecin (CPT) and its derivatives (topotecan, irinotecan), anthracyclines (doxorubicin, daunorubicin, epirubicin, idarubicin), and others. In fact, half of all the anti-cancer... (truncated)

Title: Curative effect of methotrexate combined with teniposide in the treatment of primary central nervous system lymphoma.
The present study aimed to investigate the curative effect of high-dose methotrexate (HD-MTX) combined with teniposide (Vm26) vs. HD-MTX alone in the treatment of primary central nervous system lymphoma (PCNSL), in order to provide data for assisting decisions associated with clinical treatment. Data from 56 patients with PCNSL admitted in Shanghai Huashan Hospital (Shanghai, China) from January 2009 to December 2014 were included into the present study. Clinical data, curative effects and prognosis of patients in these two groups were retrospectively analyzed using SPSS 20 statistical software. In the HD-MTX+Vm26 group, 12 patients (42.85%) achieved complete remission (CR) and 10... (truncated)

Title: Alleviation of podophyllotoxin toxicity using coexisting flavonoids from Dysosma versipellis.
Podophyllotoxin (POD) is a lignan-type toxin existing in many herbs used in folk medicine. Until now, no effective strategy is available for the management of POD intoxication. This study aims to determine the protective effects of flavonoids (quercetin and kaempferol) on POD-induced toxicity. In Vero cells, both flavonoids protected POD-induced cytotoxicity by recovering alleviating G2/M arrest, decreasing ROS generation and changes of membrane potential, and recovering microtubule structure. In Swiss mice, the group given both POD and flavonoids group had significantly lower mortality rate and showed less damages in the liver and kidney than the group given POD alone. As... (truncated)

Title: Adverse reactions to targeted and non-targeted chemotherapeutic drugs with emphasis on hypersensitivity responses and the invasive metastatic switch.
More than 100 drugs are used to treat the many different cancers. They can be divided into agents with relatively broad, non-targeted specificity and targeted drugs developed on the basis of a more refined understanding of individual cancers and directed at specific molecular targets on different cancer cells. Individual drugs in both groups have been classified on the basis of their mechanism of action in killing cancer cells. The targeted drugs include proteasome inhibitors, toxic chimeric proteins and signal transduction inhibitors such as tyrosine kinase (non-receptor and receptor), serine/threonine kinase, histone deacetylase and mammalian target of rapamycin inhibitors. Increasingly used... (truncated)

Title: Metabolic map of the antiviral drug podophyllotoxin provides insights into hepatotoxicity.
Podophyllotoxin (POD) is a natural compound with antiviral and anticancer activities. The purpose of the present study was to determine the metabolic map of POD <i>in vitro</i> and <i>in vivo</i>.Mouse and human liver microsomes were employed to identify POD metabolites <i>in vitro</i> and recombinant drug-metabolizing enzymes were used to identify the mono-oxygenase enzymes involved in POD metabolism. All <i>in vitro</i> incubation mixtures and bile samples from mice treated with POD were analysed with ultra-performance liquid chromatography coupled with electrospray ionization quadrupole time-of-flight mass spectrometry.A total of 38metabolites, including six phase-I metabolites and 32 phase-II metabolites, of POD were identified from... (truncated)

MESH:D016729 - leuprolide

Summary:

LLM Prediction Score	0.500 (normalized)
LLM Confidence Score	0.990
Golden Answer (Severity Class)	0.375 (normalized)
Prediction Error	0.125

Retrieved Context:

Title: Attenuation of Metabolic Syndrome by EPA/DHA Ethyl Esters in Testosterone-Deficient Obese Rats.
Inducing testosterone deficiency, as the standard treatment of prostate cancer, may cause metabolic disorders including insulin resistance, dyslipidemia, central obesity, cardiovascular diseases, and type 2 diabetes. This study measured responses to testosterone deficiency in high-carbohydrate, high-fat (H) diet-fed rats. We then tested whether eicosapentaenoic acid (EPA)/docosahexaenoic acid (DHA) ethyl esters (Omacor) reversed these metabolic changes. Male Wistar rats (8⁻9 weeks old) were divided into eight groups with four groups fed corn starch and four groups fed H diet. For each diet, one group received diet only; one group was orchidectomized; one group was given leuprolide (gonadotrophin-releasing hormone agonist, 2 mg/kg... (truncated)

Title: Flutamide-induced toxic hepatitis. Potential utility of ursodeoxycholic acid administration in toxic hepatitis.
Flutamide is a nonsteroidal antiandrogen commonly used in the treatment of prostate cancer. Hepatic toxicity associated with flutamide has been reported with an incidence from less than 1% to about 5%. Ursodeoxycholic acid (UDCA), a hydrophilic bile acid, has been widely used in the treatment of cholesterol gallstones and of several liver diseases, but few data are now available concerning its use in the management of drug-induced hepatitis. The case of a patient who presented severe hepatitis with jaundice following use of flutamide is reported. UDCA treatment was started on admission and, contemporaneously, flutamide was withdrawn. Clinical and biochemical improvement... (truncated)

Title: Nonalcoholic fatty liver disease induced by leuprorelin acetate.
Leuprorelin acetate is an agonist of gonadotropin-releasing hormone, used as a first choice treatment in patients with prostate carcinoma. The impact of leuprorelin therapy in liver function and metabolism is largely unknown. We report about a patient who had been treated for 32 months with leuprorelin acetate, who developed a nonalcoholic fatty liver disease (NAFLD), associated with a focal lesion at the IV hepatic segment where histologic features appeared to be more severe. The patient, in addition to NAFLD, presented a marked iatrogenic hypotestosteronemia and full-criteria meeting the diagnosis of metabolic syndrome, including insulin resistance. The radiologic and clinical findings,... (truncated)

Title: Neoadjuvant Enzalutamide Prior to Prostatectomy.
Purpose: Prostate cancer is dependent on androgen receptor (AR) activation. Optimal AR antagonism may effectively cytoreduce local disease and suppress or eliminate micrometastases. We evaluated neoadjuvant therapy prior to prostatectomy with the potent AR antagonist enzalutamide (enza) either alone or in combination with dutasteride (dut) and leuprolide (enza/dut/luteinizing hormone-releasing hormone analogues [LHRHa]).Experimental Design: Forty-eight of 52 men with intermediate or high-risk localized prostate cancer proceeded to prostatectomy after neoadjuvant enzalutamide or enza/dut/LHRHa for 6 months. We assessed pathologic complete response (pCR), minimal residual disease (MRD; ≤3 mm maximum diameter of residual disease), residual cancer burden (RCB), and expression of PSA... (truncated)

Title: Unsupervised identification of disease states from high-dimensional physiological and histopathological profiles.
The liver and kidney in mammals play central roles in protecting the organism from xenobiotics and are at high risk of xenobiotic-induced injury. Xenobiotic-induced tissue injury has been extensively studied from both classical histopathological and biochemical perspectives. Here, we introduce a machine-learning approach to analyze toxicological response. Unsupervised characterization of physiological and histological changes in a large toxicogenomic dataset revealed nine discrete toxin-induced disease states, some of which correspond to known pathology, but others were novel. Analysis of dynamics revealed transitions between disease states at constant toxin exposure, mostly toward decreased pathology, implying induction of tolerance. Tolerance correlated with induction... (truncated)

MESH:C058302 - moexipril

Summary:

LLM Prediction Score	0.250 (normalized)
LLM Confidence Score	0.940
Golden Answer (Severity Class)	0.375 (normalized)
Prediction Error	0.125

Retrieved Context:

Title: Moexipril for treatment of primary biliary cirrhosis in patients with an incomplete response to ursodeoxycholic acid. Blockade of angiotensin II synthesis attenuates hepatic fibrosis in different experimental models of chronic liver injury. We evaluated the safety and efficacy of moexipril, an angiotensin-converting enzyme inhibitor, in patients with primary biliary cirrhosis (PBC) who have had a suboptimal response to ursodeoxycholic acid (UDCA). Twenty PBC patients on UDCA (13-15 mg/kg/day) therapy with an elevation of serum alkaline phosphatase at least twice the upper limit of normal were treated with oral moexipril 15 mg/day for one year. No significant changes in serum alkaline phosphatase (379 +/- 32 vs. 379 +/- 51), bilirubin (0.8 +/- 0.1 vs. 0.9 +/- 0.1),... (truncated)

Title: Ramipril-associated cholestasis in the setting of recurrent drug-induced liver injury.
Aim: Angiotensin-converting enzyme inhibitors (ACEIs) are commonly used to treat hypertension. Although generally well tolerated, the adverse effects of ACEIs include hypotension, cough, acute kidney injury and hyperkalemia. Rare reports of ACEI-induced hepatotoxicity have been described, most notably a cholestatic pattern of injury related to captopril. A 67-year-old male presented to the emergency department with a three-week history of jaundice, pruritis and weakness. Eight weeks before, he began taking ramipril and clopidogrel. His past medical history was significant for previous acute cholestatic liver injury approximately 20 years earlier, which was attributed to methimazole. Abnormal blood work demonstrated aspartate aminotransferase (AST)... (truncated)

Title: A Review of the Challenges Associated with the Diagnosis and Therapy of Primary Sclerosing Cholangitis.
Primary sclerosing cholangitis (PSC) is a chronic and progressive cholestatic liver disease that often leads to the development of cirrhosis. Complications of PSC include pruritus, fatigue, vitamin deficiencies, metabolic bone disease, dominant biliary strictures, gallstones, and hepatobiliary malignancies, most commonly cholangiocarcinoma (CCA). Despite the presumed autoimmune etiology of PSC, a clear benefit from immunosuppressive agents has not yet been established, and their use is limited by their side effects. Endoscopy is required in evaluation of biliary strictures in PSC to rule out the possibility of CCA. Liver transplantation is currently the only life-extending therapy for patients with end-stage disease. However,... (truncated)

Title: Advances in pharmacotherapy for primary biliary cirrhosis.
Primary biliary cirrhosis (PBC) is a chronic autoimmune liver disease mostly seen in middle-aged women characterized by progressive nonsuppurative destruction of small bile ducts resulting in intrahepatic cholestasis, parenchymal injury and ultimately end-stage liver disease. Despite major breakthroughs in our understanding of PBC, there remains only one FDA-approved agent for treatment: ursodeoxycholic acid (UDCA) to which one-third of patients are unresponsive.

Title: From congestive hepatopathy to hepatocellular carcinoma, how can we improve patient management?
Heart failure and liver disease often coexist because of systemic disorders and diseases that affect both organs as well as complex cardio-hepatic interactions. Heart failure can cause acute or chronic liver injury due to ischaemia and passive venous congestion, respectively. Congestive hepatopathy is frequently observed in patients with congenital heart disease and after the Fontan procedure, but also in older patients with chronic heart failure. As congestive hepatopathy can evolve into cirrhosis and hepatocellular carcinoma, screening for liver injury should be performed in patients with chronic cardiac diseases and after Fontan surgery. Fibrosis starts in the centro-lobular zone and will... (truncated)

MESH:D000676 - amrinone

Summary:

LLM Prediction Score	0.500 (normalized)
LLM Confidence Score	0.940
Golden Answer (Severity Class)	0.625 (normalized)
Prediction Error	0.125

Retrieved Context:

Title: Clinical effects and side effects of amrinone. A study of 24 patients with chronic congestive heart failure.
Amrinone, a new inotropic agent, was used to treat 24 patients with chronic congestive heart failure who were classified as clinically stable and who were in New York Heart Association's classes II and III. Patients were treated for up to 30 months (mean, 12.5 months). Exercise tolerance improved in 20 patients, but only eight experienced significant improvement in symptoms of fatigue, dyspnea, and orthopnea and only nine tolerated the drug without apparent side effects. Eight were unable to continue amrinone therapy because of limiting side effects. The most significant adverse effects were cardiac arrhythmias, thrombocytopenia, abnormal results of liver function... (truncated)

Title: Effect of trichloroacetaldehyde on the activation of CD4⁺T cells in occupational medicamentosa-like dermatitis: An in vivo and in vitro study.
Occupational medicamentosa-like dermatitis induced by trichloroethylene (OMLDT) is a hypersensitivity disease with autoimmune liver injury, which has increasingly become a serious occupational health problem in China. However, the pathogenesis of OMLDT remained undefined. In this study, 30 TCE-induced OMLDT patients, 58 exposure controls, and 40 non-exposure controls were recruited. We showed that the ratio of activated CD4⁺ T cells (downregulation of CD62 L) was dramatically increased in OMLDT patients compared to exposure and non-exposure control, suggesting that CD4⁺ T cells activation was a key cellular event in the development of OMLDT. In parallel, the expression of cytokine including IL-2, IFN-γ, TNF-α... (truncated)

Title: Human hepatic in vitro models reveal distinct anti-NASH potencies of PPAR agonists.
Non-alcoholic steatohepatitis (NASH) is a highly prevalent, chronic liver disease characterized by hepatic lipid accumulation, inflammation, and concomitant fibrosis. Up to date, no anti-NASH drugs have been approved. In this study, we reproduced key NASH characteristics in vitro by exposing primary human hepatocytes (PHH), human skin stem cell-derived hepatic cells (hSKP-HPC), HepaRG and HepG2 cell lines, as well as LX-2 cells to multiple factors that play a role in the onset of NASH. The obtained in vitro disease models showed intracellular lipid accumulation, secretion of inflammatory chemokines, induced ATP content, apoptosis, and increased pro-fibrotic gene expression. These cell systems were... (truncated)

Title: Recent Advances of Pyridinone in Medicinal Chemistry.
Pyridinones have been adopted as an important block in medicinal chemistry that could serve as hydrogen bond donors and acceptors. With the help of feasible synthesis routes <i>via</i> established condensation reactions, the physicochemical properties of such a scaffold could be manipulated by adjustment of polarity, lipophilicity, and hydrogen bonding, and eventually lead to its wide application in fragment-based drug design, biomolecular mimetics, and kinase hinge-binding motifs. In addition, most pyridinone derivatives exhibit various biological activities ranging from antitumor, antimicrobial, anti-inflammatory, and anticoagulant to cardiotonic effects. This review focuses on recent contributions of pyridinone cores to medicinal chemistry, and addresses the... (truncated)

Title: Free radical scavengers in anaesthesiology and critical care.
Free radicals are highly reactive and unstable compounds. These highly reactive molecules cause oxidative damage to cellular components such as DNA, proteins and lipids. They play central role in the mechanism of cell injury and cell death. Free radical scavengers either prevent these reactive species from being formed, or remove them before they can damage vital components of the cell. Oxidative stress defines an imbalance in production of oxidizing chemical species and their effective removal by protective antioxidants and scavenger enzymes. Evidence of massive oxidative stress is well established in critical illnesses characterized by tissue ischaemia-reperfusion injury and by an... (truncated)

MESH:D002699 - chlorambucil

Summary:

LLM Prediction Score	0.500 (normalized)
LLM Confidence Score	0.980
Golden Answer (Severity Class)	0.375 (normalized)
Prediction Error	0.125

Retrieved Context:

Title: Interferon alfa-2b and chlorambucil in the treatment of non-Hodgkin's lymphoma.
Twenty-two patients with non-Hodgkin's lymphoma (NHL) were treated with a combined regimen of interferon alfa-2b (Intron A; Schering-Plough) and chlorambucil to evaluate the response and efficacy in pre-treated or relapsed patients. Ten patients were classified as having follicular lymphoma and 12 diffuse lymphoma. The treatment schedule consisted of interferon alfa-2b 3 X 10(6) IU/m2 thrice-weekly and chlorambucil 10 mg daily for three weeks, with a week's rest between each cycle. Treatment continued for upto six cycles. We obtained two complete remissions (CR), 12 good partial remissions (GPR), seven no remissions (NR) and one stable disease. On histologic examination we observed... (truncated)

Title: Suggested guidelines for patient monitoring: hepatic and hematologic toxicity attributable to systemic dermatologic drugs.
Hepatic and hematologic toxicity are among the most fearful adverse effects that occasionally occur as a result of systemic drugs in the dermatologist's therapeutic armamentarium. Drugs of greatest interest concerning hepatic toxicity include methotrexate, azathioprine, dapsone, and acitretin. Somewhat overlapping are drugs that have important hematologic toxicities, including methotrexate, azathioprine, dapsone, sulfonamides, cyclophosphamide, and chlorambucil. Laboratory tests most commonly used include (1) hepatic monitoring: transaminases (AST/SGOT and ALT/SGPT) and the ultrasound-guided liver biopsy, and (2) hematologic monitoring: CBC with diff and platelets along with occasional use of the reticulocyte count. Important principles and specific guidelines for monitoring by drug group... (truncated)

Title: [Hepatitis B virus reactivation during chlorambucil and prednisolone treatment in an HBsAg-negative and anti-HBs-positive patient with B-cell chronic lymphocytic leukemia].
It is generally accepted that seroconversion of hepatitis B virus (HBV) surface antigen (HBsAg) to an antibody to HBsAg (anti-HBs) indicates clearance of HBV. Here we report a case of severe hepatitis that manifested during chemotherapy in a female patient with chronic lymphocytic leukemia (CLL) who had been initially seronegative for HBsAg and seropositive for anti-HBs. The patient received chlorambucil and prednisolone for the treatment of CLL. After 6 months the serum levels of aminotransferases were increased, and HBsAg and HBV DNA were present in serum. Lamivudine was administered immediately after confirming the HBV reactivation, which considerably improved jaundice and... (truncated)

Title: Liver transplantation in man. II. A report of two orthotopic liver transplants in adult recipients.
Two patients with primary hepatic malignancy were treated by hepatectomy and orthotopic liver transplantation. In both cases the donor liver was infused with cold solutions and kept chilled without continuous perfusion. There was immediate satisfactory hepatic function in both transplants.The first patient died after 11 weeks from overwhelming bacterial and fungal infections probably secondary to hepatic infarction due to thrombosis of the recipient hepatic artery. The thrombus occurred at the site of the arterial clamp. In an attempt to control the growth before transplantation, the patient had been treated with large doses of chlorambucil, which resulted in extreme marrow depression... (truncated)

Title: Honokiol-Chlorambucil Co-Prodrugs Selectively Enhance the Killing Effect through STAT3 Binding on Lymphocytic Leukemia Cells <i>In Vitro</i> and <i>In Vivo</i>.
The broad-spectrum DNA alkylating therapeutic, chlorambucil (CBL), has limited safety and shows lower therapy effect because of a short half-life while used in the clinic. Therefore, it is very necessary to develop a more efficient and safer type of CBL derivate against tumors with selective targeting of cancer cells. In addition, the natural product of honokiol (HN), the novel potent chemo-preventive or therapeutic entity/carrier, can target the mitochondria of cancer cells through STAT3 to prevent cancer from spreading and metastasizing. In this study, we designed and synthesized the honokiol-chlorambucil (HN-CBL) co-prodrugs through carbonate ester linkage conjugating with the targeted delivery... (truncated)

MESH:D013852 - thiotepa

Summary:

LLM Prediction Score	0.500 (normalized)
LLM Confidence Score	0.980
Golden Answer (Severity Class)	0.375 (normalized)
Prediction Error	0.125

Retrieved Context:

Title: Veno-occlusive disease of the liver after busulfan, melphalan, and thiotepa conditioning therapy: incidence, risk factors, and outcome.
The purpose of this study was to determine the incidence of veno-occlusive disease (VOD) after a high-dose regimen of busulfan, melphalan, and thiotepa and the risk factors for a more severe outcome. We followed 253 consecutive patients with malignant disorders who received autologous transplants after stem cell harvest followed by 12 mg/kg busulfan, 100 mg/m2 melphalan, and 500 mg/m2 thiotepa. Diagnosis of VOD was based on weight gain, hepatomegaly, and jaundice. Risk factors for moderate or severe VOD were identified using logistic regression models. VOD occurred in 70 of 253 patients (28%), of whom 31 (12%) had moderate and 11... (truncated)

Title: A phase I study of high-dose BCNU, etoposide and escalating-dose thiotepa (BTE) with hematopoietic progenitor cell support in adults with recurrent and high-risk brain tumors.
This phase I dose-escalation study was performed to determine the tolerability of three-drug combination high-dose BCNU (B) (450 mg/m2), escalating-dose thiotepa (500-800 mg/m2) and etoposide (1200 mg/m2) in divided doses over four days in 22 adults with malignant primary brain tumors. Patients received G-CSF and hematopoeitic support with peripheral blood progenitor cells (PBPC) (n = 18) or both PBPC and marrow (n = 4). The maximum tolerated dose of thiotepa with acceptable toxicity was determined as 800 mg/m2. The 100-day mortality rate was 9% (2/22). Grade III/IV toxicities included mucositis (71%), diarrhea (29%), nausea/vomiting (19%), and hepatic toxicity (14%). Neurological... (truncated)

Title: Inappropriate antidiuretic hormone secretion after high-dose thiotepa.
High-dose thiotepa has been successfully included in a variety of conditioning regimens for stem cell transplantation in hematological and solid neoplasms. Toxicity of high-dose thiotepa mainly manifests as profound myeloablation and some degree of liver damage. We report a case of inappropriate secretion of antidiuretic hormone (SIADH) in a patient with primary CNS lymphoma who underwent therapy with high-dose thiotepa.

Title: Toxicity of high-dose chemotherapy with etoposide, thiotepa and CY in treating poor-prognosis Ewing's sarcoma family tumors: the experience of the Bambino Gesù Children's Hospital.
We report the toxicity of high-dose chemotherapy (HDC) based on etoposide, thiotepa and CY (ETC) in children with poor-prognosis Ewing's sarcoma family tumors (ESFTs). A total of 26 patients with high-risk ESFT (metastasis or axis localization or tumor volume >200 ml or necrosis <95%) were reviewed. The conditioning was based on etoposide (600 mg/m(2)), thiotepa (750 mg/m(2)) and CY (120 mg/kg) followed by autologous BM or PBSC rescue. The conditioning regimen was well tolerated, without any toxic deaths. The median time from transplant to a neutrophil count of >0.5 x 10(9)/l was 10 days (range 6-27) and 22.5 days (range... (truncated)

Title: Phase II study of high-dose thiotepa and hematopoietic stem cell transplantation in children with solid tumors.
From 1987 to 1995, 22 children with refractory solid tumors entered a phase II study of high-dose thiotepa (HDT) (900 mg/m2) followed by stem cell transplantation (SCT) in the Pediatrics Department of the Institut Gustave Roussy. Tumor types were rhabdomyosarcoma (eight), osteosarcoma (seven), neuroblastoma (three), Ewing's sarcoma (three) and Burkitt's lymphoma (one). Before HDT, all had been extensively treated with conventional chemotherapy, surgical resection of the primary tumor (13/22) and of metastases (6/22), and radiotherapy of the primary tumor in three patients. All had measurable disease, at the site of the primary tumor (3 patients), of the metastases (9 patients)... (truncated)

MESH:C093875 - montelukast

Summary:

LLM Prediction Score	0.500 (normalized)
LLM Confidence Score	0.990
Golden Answer (Severity Class)	0.375 (normalized)
Prediction Error	0.125

Retrieved Context:

Title: Montelukast induced acute hepatocellular liver injury.
A 46-year-old male with uncontrolled asthma on inhaled albuterol and formoterol with budesonide was commenced on montelukast. He developed abdominal pain and jaundice 48 days after initiating montelukast therapy. His liver tests showed an increase in serum total bilirubin, conjugated bilirubin, aspartate aminotransferase, alanine aminotransferase, and alkaline phosphatase. The patient was evaluated for possible non-drug related liver injury. Montelukast was discontinued suspecting montelukast induced hepatocellular liver injury. Liver tests began to improve and returned to normal 55 days after drug cessation. Causality of this adverse drug reaction by the Council for International Organizations of Medical Sciences or Roussel Uclaf Causality... (truncated)

Title: The Effect of Montelukast on Liver Damage in an Experimental Obstructive Jaundice Model.
Montelukast is a cysteinyl-leukotriene type 1 (CysLT1) selective receptor antagonist. In recent years, investigations have shown that montelukast possesses secondary anti-inflammatory activities and also antioxidant effects. For this reason, we aimed to determine the possible effects of montelukast on liver damage in experimental obstructive jaundice.

Title: Hepatotoxicity caused by montelukast in a paediatric patient.
Montelukast is a selective and competitive cysteinyl leukotriene receptor antagonist (CystLTRA) which is increasingly used for the treatment of allergic asthma. Recently, hepatotoxicity has been reported with this drug in adult patients, but only one letter to the editor has reported a case of probable montelukast-induced hepatotoxicity in a child. We present a case of a 3.5-year-old boy, receiving treatment with montelukast, who developed hepatocellular injury. The exclusion of other causes of increased activity of aminotransferases (viral, metabolic, autoimmune), improvement after dechallenge, the morphological findings and previous reports of comparable cases support the diagnosis of montelukast-induced liver injury in this... (truncated)

Title: Montelukast Prevents Mice Against Acetaminophen-Induced Liver Injury.
Acetaminophen (APAP) is a widely used over-the-counter antipyretic and analgesic drug. Overdose of APAP is the leading cause of hospital admission for acute liver failure. Montelukast is an antagonist of cysteinyl leukotriene receptor 1 (Cysltr1), which protects from inflammation and oxidative stress. However, the function of montelukast in APAP-induced hepatotoxicity remains unknown. In this study, we examined whether pharmacological inhibition of Cystlr1 could protect mice against APAP-induced hepatic damage. We found that APAP treatment upregulated messenger RNA and protein levels of Cysltr1 both <i>in vitro</i> and <i>in vivo</i>. Pharmacological inhibition of Cysltr1 by montelukast ameliorated APAP-induced acute liver failure. The... (truncated)

Title: The use of 5-lipoxygenase inhibitors and leukotriene receptor antagonists in the treatment of chronic asthma.
With the elucidation of asthma as a chronic inflammatory disease, therapeutic approaches have shifted from treatment of symptoms with bronchodilators to treatment of the underlying disease with antiinflammatory agents. Along with concerns about corticosteroid side effects on the part of both physicians and patients, this shift has motivated researchers to develop and test new agents with antiinflammatory capabilities. The leukotrienes are endogenous mediators with three inflammatory effects: they increase vascular permeability, recruit other inflammatory leukocytes, and induce bronchoconstriction. A number of antileukotriene agents are in various stages of development. Zileuton, a leukotriene synthesis inhibitor, has been shown to improve airway... (truncated)

MESH:D011803 - quinine

Summary:

LLM Prediction Score	0.500 (normalized)
LLM Confidence Score	0.990
Golden Answer (Severity Class)	0.375 (normalized)
Prediction Error	0.125

Retrieved Context:

Title: Quinine-induced granulomatous hepatitis.
Many adverse reactions to quinine have been reported. A 65 year old woman taking quinine sulphate for nocturnal leg cramps presented for investigation of episodes of malaise, fever, nausea, vomiting, and polyarthralgia. Granulomatous hepatitis was diagnosed, for which no common cause was found. She was challenged with quinine sulphate; within hours her temperature had risen and her symptoms returned; transaminase activities rose within 48 hours, as did erythrocyte sedimentation rate. After withdrawal of the drug symptoms abated and transaminase activities returned to normal. The biochemical response to challenge with quinine implicates the drug as the cause of the liver disturbances.... (truncated)

Title: Diversity and severity of adverse reactions to quinine: A systematic review.
Quinine is a common cause of drug-induced thrombocytopenia and the most common cause of drug-induced thrombotic microangiopathy. Other quinine-induced systemic disorders have been described. To understand the complete clinical spectrum of adverse reactions to quinine we searched 11 databases for articles that provided sufficient data to allow evaluation of levels of evidence supporting a causal association with quinine. Three reviewers independently determined the levels of evidence, including both immune-mediated and toxic adverse reactions. The principal focus of this review was on acute, immune-mediated reactions. The source of quinine exposure, the involved organ systems, the severity of the adverse reactions, and... (truncated)

Title: Quinine allergy causing acute severe systemic illness: report of 4 patients manifesting multiple hematologic, renal, and hepatic abnormalities.
Quinine is widely used for the common symptom of leg cramps. Quinine tablets require a prescription, but quinine and the product from which it is derived, cinchona, are also available without prescription. They are components of over-the-counter remedies for many common symptoms, of nutrition products, and of beverages such as tonic water and bitter lemon. Although quinine has been used for centuries, initially as an extract from the bark of the cinchona tree, allergic reactions to quinine can be severe and can affect multiple organs. These allergic reactions can cause thrombocytopenia, neutropenia, anemia, disseminated intravascular coagulation, acute renal failure, liver... (truncated)

Title: Quinine hepatotoxicity. An underrecognized or rare phenomenon?
Quinine-induced hepatic injury has been reported very rarely. We report a case involving a patient with an apparent hypersensitivity to quinine, manifested by a clinical syndrome mimicking a viral illness and a mixed hepatocellular-cholestatic liver injury pattern. We briefly review the unusual literature on this topic, which features many cases of hypersensitivity to the optical isomer of quinine, quinidine; yet, to our knowledge, there has only been one prior report of quinine hepatotoxicity.

Title: Disseminated Intravascular Coagulation Due to Drinking Tonic Water.
Quinine has been used worldwide to treat malaria; however, it is now used as an agent for night-time muscle cramping. The compound, derived from Cinchona tree bark, is found in antimalaria medication, supplements for leg cramping, and beverages such as tonic water and bitter lemon. Quinine, however, is not without its side effect profile which includes a wide range of ailments ranging from nausea to disseminated intravascular coagulation. The authors present a case of a 35-year-old man diagnosed with disseminated intravascular coagulation due to an excessive intake of tonic water because his friend told him that it would help alleviate... (truncated)

MESH:D009173 - mycophenolic acid

Summary:

LLM Prediction Score	0.500 (normalized)
LLM Confidence Score	0.980
Golden Answer (Severity Class)	0.375 (normalized)
Prediction Error	0.125

Retrieved Context:

Title: Effect of long-term coadministration of compound glycyrrhizin tablets on the pharmacokinetics of mycophenolic acid in rats.
1. Mycophenolic acid (MPA), having high-protein affinity, is an immunodepressant and the genuine-active ingredient of enteric-coated mycophenolate sodium (EC-MPS) tablet that has been widely used in combination with tacrolimus or cyclosporine to prevent acute rejection after organ transplantation. Moreover, MPA mainly experiences glucuronidation and its metabolites are partly transported by multidrug resistance-associated protein (Mrp) 2 into bile then reforms MPA via enterohepatic circulation. 2. Glycyrrhizin (GL), having high-protein affinity, is the main active ingredient of compound glycyrrhizin tablet, which is often prescribed with EC-MPS, tacrolimus or cyclosporine to prevent drug-induced hepatitis. In addition, GL can inhibit Mrp2 and selectively induce CYP3A and... (truncated)

Title: Hepatotoxicity and liver enzyme alteration in patients with immunobullous diseases receiving immunosuppressive therapy.
To avoid complications of high dose corticosteroid, pemphigus patients are usually co-treated with other immunosuppressive agents. Liver enzyme abnormality occurs commonly during treatment and occasionally causes discontinuation of drugs. To assess the rate of therapy-induced hepatotoxicity in patients with immunobullous diseases, we conducted a study of 250 pemphigus patients under immunosuppressive therapy prospectively. Aspartate aminotransferase (AST), alanine aminotransferase (ALT) and alkaline phosphatase (ALP) plasma levels were recorded before the start of treatment and every week under treatment (up to 3 weeks). Hepatotoxicity was defined as the rise in the ALT plasma levels to greater than twice the upper normal limit.... (truncated)

Title: Population pharmacokinetics of unbound mycophenolic acid in pediatric and young adult patients undergoing allogeneic hematopoietic cell transplantation.
Mycophenolate mofetil (MMF) is an immunosuppressant routinely used in allogeneic hematopoietic cell transplantation (alloHCT) to promote stem cell engraftment and prevent acute graft vs host disease. Administered as a prodrug, MMF is converted by esterases to the active moiety, mycophenolic acid (MPA). The impact of clinical covariates on unbound MPA exposure was investigated with a population pharmacokinetic approach. Pharmacokinetic data were obtained from routine area under the curve (AUC) monitoring of unbound MPA drug levels in 36 pediatric (n = 31) and young adult (n = 5) patients undergoing alloHCT for a variety of malignant and nonmalignant disorders. Unbound MPA... (truncated)

Title: Nonstandard drugs and feasible new interventions for autoimmune hepatitis: part I.
Nonstandard drugs that target critical pathogenic pathways or immune regulatory mechanisms constitute the next generation of treatments for autoimmune hepatitis. Mycophenolate mofetil impairs the proliferation of lymphocytes, decreases autoantibody production, and induces apoptosis of activated immunocytes. Patients treated for azathioprine intolerance improve more frequently than patients treated for refractory liver disease (54% versus 10%), and mycophenolate mofetil is emerging as a rescue therapy for this population. Complete corticosteroid withdrawal is possible in 40% of patients treated with mycophenolate mofetil, and the frequency of side effects ranges from 3-34%. Budesonide in combination with azathioprine has normalized liver tests more frequently (47%... (truncated)

Title: Uncommon side effect of MMF in renal transplant recipients.
Mycophenolate mofetil (MMF) is a potent immunosuppressive agent used in renal transplantation. Gastrointestinal and hematological side effects are commonly observed, but hepatotoxicity has not been reported. In this study, we assessed MMF-related hepatotoxicity in renal transplant recipients. A total of 124 renal transplantation recipients (RTRs) were evaluated for elevated liver enzymes associated with MMF, and 79 patients were enrolled to the study. Patients used MMF 2 g/day. The patients who had progressive increase in liver enzymes after renal transplantation and their AST, ALT, GGT, ALP, bilirubin levels, hepatitis, cytomegalovirus (CMV), abdominal ultrasonography, duration of hepatotoxicity, and decreased dosage or withdrawal... (truncated)

MESH:D000077764 - dronedarone

Summary:

LLM Prediction Score	0.750 (normalized)
LLM Confidence Score	0.970
Golden Answer (Severity Class)	0.875 (normalized)
Prediction Error	0.125

Retrieved Context:

Title: Mitochondrial oxidative stress and respiratory chain dysfunction account for liver toxicity during amiodarone but not dronedarone administration.
The role played by oxidative stress in amiodarone-induced mitochondrial toxicity is debated. Dronedarone shows pharmacological properties similar to those of amiodarone but several differences in terms of toxicity. In this study, we analyzed the effects of the two drugs on liver mitochondrial function by administering an equivalent human dose to a rat model. Amiodarone increased mitochondrial H(2)O(2) synthesis, which in turn induced cardiolipin peroxidation. Moreover, amiodarone inhibited Complex I activity and uncoupled oxidative phosphorylation, leading to a reduction in the hepatic ATP content. We also observed a modification of membrane phospholipid composition after amiodarone administration. N-acetylcysteine completely prevented such effects.... (truncated)

Title: DNA damage-induced apoptosis and mitogen-activated protein kinase pathway contribute to the toxicity of dronedarone in hepatic cells.
Dronedarone, an antiarrhythmic drug, has been marketed as an alternative to amiodarone. The use of dronedarone has been associated with severe liver injury; however, the mechanisms remain unclear. In this study, the possible mechanisms of dronedarone induced liver toxicity were characterized in HepG2 cells. Dronedarone decreased cells viability and induced apoptosis and DNA damage in a concentration- and time-dependent manner. Pretreatment of the HepG2 cells with apoptosis inhibitors (caspase-3, -8, and -9) or the necrosis inhibitor (Necrox-5), partially, but significantly, reduced the release of lactate dehydrogenase. Dronedarone caused the release of cytochrome c from mitochondria to cytosol, a prominent feature... (truncated)

Title: The role of hepatic cytochrome P450s in the cytotoxicity of dronedarone.
Dronedarone is used to treat patients with cardiac arrhythmias and has been reported to be associated with liver injury. Our previous mechanistic work demonstrated that DNA damage-induced apoptosis contributes to the cytotoxicity of dronedarone. In this study, we examined further the underlying mechanisms and found that after a 24-h treatment of HepG2 cells, dronedarone caused cytotoxicity, G1-phase cell cycle arrest, suppression of topoisomerase II, and DNA damage in a concentration-dependent manner. We also investigated the role of cytochrome P450s (CYPs)-mediated metabolism in the dronedarone-induced toxicity using our previously established HepG2 cell lines expressing individually 14 human CYPs (1A1, 1A2, 1B1,... (truncated)

Title: Severe toxic hepatitis associated with dronedarone.
Dronedarone was introduced in 2009 as a new antiarrhythmic agent and since then has been increasingly prescribed in atrial fibrillation or flutter. To date, two cases of severe toxic hepatitis have been reported in patients treated with dronedarone, both requiring emergency liver transplantation, and the FDA as well as the EMA have issued warnings about possible severe hepatotoxicity of dronedarone. Here we report an additional case of toxic hepatitis associated with dronedarone presenting with acute liver failure, followed by spontaneous recovery, in a 69-year old woman.

Title: Mechanisms of hepatocellular toxicity associated with dronedarone--a comparison to amiodarone.
Dronedarone is a new antiarrhythmic drug with an amiodarone-like benzofuran structure. Shortly after its introduction, dronedarone became implicated in causing severe liver injury. Amiodarone is a well-known mitochondrial toxicant. The aim of our study was to investigate mechanisms of hepatotoxicity of dronedarone in vitro and to compare them with amiodarone. We used isolated rat liver mitochondria, primary human hepatocytes, and the human hepatoma cell line HepG2, which were exposed acutely or up to 24h. After exposure of primary hepatocytes or HepG2 cells for 24h, dronedarone and amiodarone caused cytotoxicity and apoptosis starting at 20 and 50 µM, respectively. The cellular... (truncated)

MESH:D000078222 - tinzaparin

Summary:

LLM Prediction Score	0.250 (normalized)
LLM Confidence Score	0.910
Golden Answer (Severity Class)	0.375 (normalized)
Prediction Error	0.125

Retrieved Context:

Title: A study of unfractionated and low molecular weight heparins in a model of cholestatic liver injury in the rat.
Forty-eight rats with biliary obstruction induced by double ligation and section of the common bile duct were randomly and blindly assigned to receive subcutaneous injection of either conventional heparin sodium (1000IU kg(-1)), three already marketed low molecular weight heparin (LMWH) preparations: nadroparin (1000 anti-Xa IU kg(-1)), tinzaparin (1000 anti-Xa IU kg(-1)), enoxaparin (180 anti-Xa IU kg(-1)) or saline. Drugs were administered once a day, starting 7 days after surgery and continued for 3 weeks. At the end of the treatment period, rats were killed and analyzed for blood biochemistry and liver pathology. Liver fibrosis was assessed by image analysis. Data... (truncated)

Title: Hepatotoxicity with cholestatic pattern secondary to enoxaparin treatment.
Treatment with low-molecular-weight heparins is very common in clinical practice. Exceptionally, some patients develop hepatitis within a few days of starting treatment, and rapid discontinuation of the drug is decisive to avoid chronification.

Title: Anticoagulants: A Review of the Pharmacology, Dosing, and Complications.
Anticoagulants remain the primary strategy for the prevention and treatment of thrombosis. Unfractionated heparin, low molecular weight heparin, fondaparinux, and warfarin have been studied and employed extensively with direct thrombin inhibitors typically reserved for patients with complications or those requiring intervention. Novel oral anticoagulants have emerged from clinical development and are expected to replace older agents with their ease of use and more favorable pharmacodynamic profiles. Hemorrhage is the main concerning adverse event with all anticoagulants. With their ubiquitous use, it becomes important for clinicians to have a sound understanding of anticoagulant pharmacology, dosing, and toxicity.

Title: Effects of Anticoagulants on Experimental Models of Established Chronic Liver Diseases: A Systematic Review and Meta-Analysis.
The role of anticoagulants in chronic liver diseases is inconclusive. A meta-analysis was thus undertaken to evaluate treatment-related survival and antifibrotic effects in animal models of chronic liver diseases.

Title: Enoxaparin-Induced Liver Injury: Case Report and Review of the Literature and FDA Adverse Event Reporting System (FAERS).
Anticoagulants are a well known cause of drug-induced liver injury (DILI). We recently encountered a 45-year-old male who developed DILI during treatment with enoxaparin, a low-molecular-weight heparin (LMWH), for dural venous thrombosis. The man received enoxaparin 80 mg subcutaneously, twice daily. After 4 days, the patient was asymptomatic but he developed liver aminotransferase elevations: AST 340 U/L and ALT 579 U/L. Investigation revealed an R ratio of 19.9 by day 5 and a Roussel Uclaf Causality Assessment Method score of 10, giving a high probable likelihood that enoxaparin was the cause of hepatic injury. Enoxaparin was discontinued on day 7,... (truncated)

MESH:D017332 - cetirizine

Summary:

LLM Prediction Score	0.500 (normalized)
LLM Confidence Score	0.990
Golden Answer (Severity Class)	0.375 (normalized)
Prediction Error	0.125

Retrieved Context:

Title: A case of levocetirizine-induced liver injury.
Levocetirizine is a second-generation nonsedative antihistaminic agent that has been demonstrated to be safe and effective for treating allergic disease. There was only one case report of levocetirizine-induced liver toxicity, but a liver biopsy was not performed. In this article, we present the first case of levocetirizine-induced liver injury with histologic findings. A 48-year-old man was hospitalized with jaundice and generalized pruritus that had developed after 2 months of therapy with levocetirizine for prurigo nodularis. Laboratory findings revealed acute hepatitis with cholestasis. A liver biopsy demonstrated portal inflammation and hepatitis with apoptotic hepatocytes. The patient fully recovered 3 weeks after... (truncated)

Title: Cetirizine-induced hepatotoxicity: case series and review of the literature.
Drug-induced liver damage is a frequently encountered clinical table caused by many drugs. Cetirizine is a widely preferred and prescribed antihistaminic agent for allergic disorders due to its non-sedative properties. In view of the literature, we present four cases of hepatotoxicity due to cetirizine use. We conclude that in patients with high levels of liver enzymes of unknown origin, cetirizine as well as other hepatotoxic drugs should be reconsidered.

Title: Focus on the cetirizine use in clinical practice: a reappraisal 30 years later.
Antihistamines are currently one of the most commonly administered categories of drugs. They are used to treat symptoms that are secondary to histamine release, which is typical of certain allergic conditions, including rhinitis, conjunctivitis, asthma, urticaria, and anaphylaxis. Cetirizine belongs to the second-generation family, so, it is very selective for peripheral H1 receptors, is potent and quickly relieves symptoms, exerts additional anti-allergic/anti-inflammatory effects, and is usually well-tolerated. It has been marketed 30 years ago. In these years, a remarkable body of evidence has been built. The current review provides a practical update on the use of cetirizine in clinical practice.

Title: Safety of Cyproheptadine, an Orexigenic Drug. Analysis of the French National Pharmacovigilance Data-Base and Systematic Review.
Objectives: Cyproheptadine is a first-generation H1-antihistamine drug first that was distributed in the 1960s. While its orexigenic effect was observed early, cyproheptadine is not yet authorized for this indication in all countries today. There is an increasing medical interest and demand for the orexigenic effect of cyproheptadine, especially in children with poor appetite. As cyproheptadine might be evaluated in future clinical trials, we wanted to assess its safety profile. Methods: Using the French national pharmacovigilance database, we retrospectively analyzed all pediatric and adult reports of adverse effects of cyproheptadine recorded since its first distribution in France. Next, we performed a... (truncated)

Title: Levocetirizine induced hepatotoxicity in a patient with chronic urticaria.
No abstract available.

MESH:D011802 - quinidine

Summary:

LLM Prediction Score	0.500 (normalized)
LLM Confidence Score	0.980
Golden Answer (Severity Class)	0.375 (normalized)
Prediction Error	0.125

Retrieved Context:

Title: [Hepatitis caused by quinidine. Study of a case and review of the literature].
The authors report the case of a 63 year-old woman who developed high-grade fever with chills, nausea, diarrhea, severe pain in the right hypochondrium, and jaundice after one month's treatment with 300 mg of hydroquinidine hydrochloride daily. Serum bilirubin and aminotransferases were slightly increased, while alkaline phosphatases and gamma-glutamyl-transpeptidase serum activities were markedly raised. Histological examination of a liver specimen obtained by the transvenous route showed numerous epithelioid granulomas with giant cell formation and eosinophils in hepatic lobules and portal tracts. Symptoms disappeared three days after withdrawal of the drug, but hepatomegaly and a mild increase in serum gamma-glutamyl-transpeptidase persisted... (truncated)

Title: Quinidine-induced hepatotoxicity revisited.
Although quinidine has been widely used since the beginning of the century, quinidine-induced hepatotoxicity has been recently reported in the literature. We describe a reversible case of quinidine-induced hepatotoxicity. A 62-y-old male with a past medical history of atrial flutter and adult onset diabetes was admitted to the hospital with a 3-d history of diarrhea, nausea, fever, chills and palpitations. Past medications included 7.5 mg glyburide daily for 4 y, 0.25 mg digoxin daily for 3 w, 324 mg quinidine gluconate 3 times daily for 2 w, and 150 mg papaverine daily for 2 y. On admission, liver enzyme levels... (truncated)

Title: Quinidine hypersensitivity: a side effect of a forgotten antiarrhythmic.
Quinidine is one of the oldest antiarrhythmics known. Over the years, its use has decreased along with its side effects. Our case describes a 69-year-old woman with recurrent resistant ventricular tachycardia on Quinidine and Amiodarone who presented with acute liver toxicity. Drug-induced liver toxicity was at the top of our differential diagnosis list. Taking multiple factors into consideration, a decision was made to discontinue Quinidine, the patient's symptoms and lab abnormalities resolved within 1 week, yielding the diagnosis of Quinidine hypersensitivity.

Title: Quinidine hepatitis.
Long-term administration of quinidine was associated with persistent elevation of serum concentrations of SGOT, lactic acid dehydrogenase, and alkaline phosphatase. Liver biopsy showed active hepatitis. Discontinuance of quinidine therapy led to normalization of liver function tests. A challenge dose of quinidine caused clinical symptoms and abrupt elevation of SGOT, alkaline phosphatase, and lactic acid dehydrogenase values. We concluded that this patient had quinidine hepatotoxicity and believe that this is the first case reported with liver biopsy documentation. This report also suggests that, even after long-term administration, the hepatic toxicity is reversible.

Title: Quinine hepatotoxicity. An underrecognized or rare phenomenon?
Quinine-induced hepatic injury has been reported very rarely. We report a case involving a patient with an apparent hypersensitivity to quinine, manifested by a clinical syndrome mimicking a viral illness and a mixed hepatocellular-cholestatic liver injury pattern. We briefly review the unusual literature on this topic, which features many cases of hypersensitivity to the optical isomer of quinine, quinidine; yet, to our knowledge, there has only been one prior report of quinine hepatotoxicity.

MESH:D008463 - mebendazole

Summary:

LLM Prediction Score	0.500 (normalized)
LLM Confidence Score	0.990
Golden Answer (Severity Class)	0.375 (normalized)
Prediction Error	0.125

Retrieved Context:

Title: Mebendazole Inhibits <i>Histoplasma capsulatum</i> In Vitro Growth and Decreases Mitochondrion and Cytoskeleton Protein Levels.
Histoplasmosis is a frequent mycosis in people living with HIV/AIDS and other immunocompromised hosts. Histoplasmosis has high rates of mortality in these patients if treatment is unsuccessful. Itraconazole and amphotericin B are used to treat histoplasmosis; however, both antifungals have potentially severe pharmacokinetic drug interactions and toxicity. The present study determined the minimal inhibitory and fungicidal concentrations of mebendazole, a drug present in the NIH Clinical Collection, to establish whether it has fungicidal or fungistatic activity against <i>Histoplasma capsulatum</i>. Protein extracts from <i>H. capsulatum</i> yeasts, treated or not with mebendazole, were analyzed by proteomics to understand the metabolic changes driven... (truncated)

Title: Hepatotoxicity of mebendazole. Relationship with serum concentrations of the drug.
We report the case of a 47-year-old female patient, suffering from multiple hydatid cysts of the liver, in whom hepatitis developed after mebendazole treatment. Clinical manifestations of hypersensitivity were absent. A correlation was found between serum mebendazole concentrations and the degree of cytolysis; this is compatible with a direct hepatotoxic effect of mebendazole.

Title: Stimulation of pro-inflammatory responses by mebendazole in human monocytic THP-1 cells through an ERK signaling pathway.
Oral helminthic mebendazole (MBZ) has been reported to cause liver injury with inflammatory responses. However, the underlying mechanism remains unknown. To examine the inflammatory reactions, we investigated whether MBZ and other helminthic drugs increase the release of pro-inflammatory cytokines and chemokines using human monocytic cells. The release of interleukin (IL)-8 and tumor necrosis factor (TNF) α from human monocytic THP-1 cells was significantly increased by treatment with MBZ, albendazole (ABZ), fenbendazole (FBZ), or oxibendazole (OBZ), but not by albendazole sulfoxide or praziquantel, suggesting that MBZ and structurally similar drugs can stimulate monocytes and increase the release of pro-inflammatory cytokines. MBZ... (truncated)

Title: Hepatotoxicity caused by mebendazole in a patient with Gilbert's syndrome.
Mebendazole (MBZ) is a broad-spectrum antihelminthic agent of the benzimidazole type. Although MBZ has been reported to cause hepatic injury, case reports of severe hepatic injury are very rare. We report a case of severe hepatitis after administration of MBZ in a patient with Gilbert's syndrome affected by pinworms infestation.

Title: Hepatic Capillariasis- Drug Targets.
Zoonotic infections are increasingly becoming public health menaces and are usually transmitted to humans due to unsuitable environmental conditions. One of them is hepatic capillariasis, caused by the parasite Capillaria hepatica, primarily a disease of rodents, with hepatic manifestations in humans. Although its prevalence is very low, it can cause significant morbidity and mortality, with cases reported from all over the world. The main infective form for humans is the embryonated egg of the parasite, which hatches in the intestine and ultimately colonize the liver. The larvae mature and reproduce, and eventually form embryonated eggs, which cause chronic focal inflammation... (truncated)

MESH:D000666 - amphotericin b

Summary:

LLM Prediction Score	0.250 (normalized)
LLM Confidence Score	0.990
Golden Answer (Severity Class)	0.375 (normalized)
Prediction Error	0.125

Retrieved Context:

Title: Amphotericin B and liver function.
Amphotericin B is well established as a highly efficacious agent against systemic fungal infections in humans. The therapeutic potential of amphotericin B is limited due to its side effects. Although in clinical use for more than 35 years, impairment of liver function is not considered to be a typical adverse effect of amphotericin B. Experimental data suggest that the drug may interfere with the hepatic cytochrome P450 and may thus influence the metabolic capacity of the liver. A confirmation of such a finding in patients treated with amphotericin B would be of value. The incidence of severe acute or subacute... (truncated)

Title: Clinical hepatotoxicity associated with antifungal agents.
Invasive fungal diseases (IFDs) are a leading cause of morbidity and mortality among immunocompromised patients with bone marrow failure syndromes, hematological malignancies, hematopoietic stem cell transplantation (HSCT), those admitted in intensive care units (ICUs) and those with prolonged febrile neutropenia. IFDs occur in a setting of multiple morbidities and are associated with case fatality rates between 30 and 70%. Along with the development of classes and compounds, the last two decades have seen substantial improvements in the prevention and management of these infections and an overall increased use of antifungal agents. Areas covered: All antifungal agents, including amphotericin B formulations,... (truncated)

Title: Reversible hepatotoxicity related to amphotericin B.
Hepatotoxicity is regarded as a rare side effect of amphotericin B therapy. A patient with acute myelogenous leukemia who had normal liver function was treated with amphotericin B for fungal pneumonia. While he was receiving the drug at high dosages asymptomatic elevation of the levels of alkaline phosphatase, serum glutamic oxaloacetic transaminase, serum glutamic pyruvic transaminase, lactic dehydrogenase and bilirubin was noted. The levels returned to normal when the drug was discontinued. Rechallenge with a lower dosage prompted a rapid rise in the levels, with subsequent return to normal when the medication was withdrawn.

Title: Adverse effects of antifungal therapies in invasive fungal infections: review and meta-analysis.
Amphotericin B is the main therapeutic agent for the treatment of invasive fungal infections; however, it is associated with significant toxicities that limit its use. Other systemic antifungal agents have been developed to improve tolerability while maintaining the efficacy profile of conventional amphotericin B. Fifty-four studies involving 9,228 patients were assessed for the frequency of adverse effects of the main systemic antifungal agents. While the results suggest that liposomal amphotericin B is the least nephrotoxic of the lipid formulations (14.6%), that conventional amphotericin B is the most nephrotoxic (33.2%), and that itraconazole is the most hepatotoxic (31.5%), the lack of... (truncated)

Title: Frequency and Associated Factors of Amphotericin B Nephrotoxicity in Hospitalized Patients in Hematology-Oncology Wards in the Southwest of Iran.
Nephrotoxicity is the most clinically significant adverse reaction of amphotericin B. Different aspects of amphotericin B (AmB) nephrotoxicity have not been studied well in our population.

MESH:D000667 - ampicillin

Summary:

LLM Prediction Score	0.500 (normalized)
LLM Confidence Score	0.990
Golden Answer (Severity Class)	0.375 (normalized)
Prediction Error	0.125

Retrieved Context:

Title: Literature review of the clinical characteristics of metformin-induced hepatotoxicity.
Background: Knowledge of metformin-induced hepatotoxicity is based on case reports. The aim of this study was to investigate the clinical features of metformin-induced hepatotoxicity. Methods: We collected relevant literature on metformin-induced hepatotoxicity published from January 1994 to February 2022 by searching Chinese and English databases. Results: Thirty patients (19 males and 11 females) from 29 articles were included, with a median age of 61 years (range 29-83). The median time to onset of liver injury was 4 weeks (range 0.3-648) after metformin administration. Clinical symptoms occurred in 28 patients, including gastrointestinal reactions (56.7%), jaundice (50.0%), fatigue (36.7%), anorexia (23.3%), pruritus (13.3%), dark... (truncated)

Title: Influence of Antibiotics on Functionality and Viability of Liver Cells In Vitro.
(1) Antibiotics are an important weapon in the fight against serious bacterial infections and are considered a common cause of drug-induced liver injury (DILI). The hepatotoxicity of many drugs, including antibiotics, is poorly analyzed in human in vitro models. (2) A standardized assay with a human hepatoma cell line was used to test the hepatotoxicity of various concentrations (Cmax, 5× Cmax, and 10× Cmax) of antibiotics. In an ICU, the most frequently prescribed antibiotics, ampicillin, cefepime, cefuroxime, levofloxacin, linezolid, meropenem, rifampicin, tigecycline, and vancomycin, were incubated with HepG2/C3A cells for 6 days. Cell viability (XTT assay, LDH release, and vitality),... (truncated)

Title: Cholestatic liver disease with ductopenia (vanishing bile duct syndrome) after administration of clindamycin and trimethoprim-sulfamethoxazole.
Two patients who developed cholestatic liver disease after exposure to antibiotics are described. One patient who received clindamycin had liver biopsy findings of marked cholestasis, portal inflammation, bile duct injury and bile duct paucity (ductopenia). A second biopsy after clinical improvement showed resolution of cholestasis but persistence of duct paucity. Three years later, treatment with ampicillin caused another episode of cholestatic hepatitis with cholestasis and duct paucity on rebiopsy. The second patient, who developed cholestasis after receiving trimethoprim-sulfamethoxazole, had marked duct paucity in the liver biopsy. This is the first description, to our knowledge, of ductopenia apparently caused by clindamycin.... (truncated)

Title: Outpatient drug-induced parenchymal liver disease requiring hospitalization.
A case-history study of drug-induced liver disorders requiring hospitalization was carried out at the Group Health Cooperative of Puget Sound, a health maintenance organization with about 280,000 members, for the five-year period from January 1, 1977 to December 31, 1981. During this time, there were 12 instances of hospitalization for liver disorders judged to be probably (nine cases) or possibly (three cases) attributable to outpatient drug ingestion (other than antitumor agents). The rate was on the order of one per 100,000 person-years at risk. Drugs implicated as probable causes were ampicillin (two cases), carbamazepine (one case), erythromycin (one case), methyldopa... (truncated)

Title: Updating the side effects of the penicillins.
Use of the penicillins in patients may result in a variety of side effects in addition to the well known occasional allergic reactions. Some of these side effects may be quite serious. Treatment with the various penicillins, expecially in the large amounts presently prescribed, may be attended by immunologic reactions, such as nephritis, and by direct toxic effects which may involve the blood platelets, the neurones of the central nervous system, and perhaps the parenchymal cells of the liver.

MESH:D019888 - nelfinavir

Summary:

LLM Prediction Score	0.500 (normalized)
LLM Confidence Score	0.980
Golden Answer (Severity Class)	0.375 (normalized)
Prediction Error	0.125

Retrieved Context:

Title: Incidence of and risk factors for severe hepatotoxicity of nelfinavir-containing regimens among HIV-infected patients with chronic hepatitis C.
To determine the incidence of and risk factors for severe hepatotoxicity of nelfinavir-containing regimens among human immunodeficiency virus/hepatitis C virus (HIV/HCV)-coinfected patients with known stage of liver fibrosis.

Title: Hepatotoxicity and nelfinavir: a meta-analysis.
The inclusion of protease inhibitors in 3-drug highly active antiretroviral regimens for treating patients who are infected with human immunodeficiency virus-1 has had a significant impact in increasing survival and decreasing morbidity. However, the effectiveness of this class of drugs may be compromised by the occurrence of drug-related hepatotoxicity, which is problematic especially in individuals co-infected with hepatitis viruses. Based on its clinical and pharmacologic profile, especially its unique pattern of resistance, nelfinavir has been used frequently as a first-line protease-inhibitor therapy for human immunodeficiency virus-1-infected patients. The aim of this study was to identify the relative potential for developing... (truncated)

Title: Incidence of Severe Hepatotoxicity Related to Antiretroviral Therapy in HIV/HCV Coinfected Patients.
Introduction. Hepatotoxicity is a concern in HIV/hepatitis C virus (HCV) coinfected patients due to their underlying liver disease. This study assessed the incidence of hepatotoxicity in HIV/HCV co-infected patients in two outpatient infectious diseases clinics. Methods. HIV/HCV co-infected adults were included in this retrospective study if they were PI or NNRTI naïve at their first clinic visit and were initiated on an NNRTI- and/or PI-based antiretroviral regimen. Patients were excluded if they had active or chronic hepatitis B virus (HBV). The primary objective was to determine the overall incidence of severe hepatotoxicity. Results. Fifty-six of the 544 patients identified met... (truncated)

Title: Safety, efficacy, and tolerability of nelfinavir-containing antiretroviral therapy for patients coinfected with HIV and hepatitis C undergoing methadone maintenance.
The safety, efficacy, and tolerability of nelfinavir (NFV)-containing antiretroviral therapy were evaluated in patients coinfected with HIV and hepatitis C undergoing methadone maintenance at an urban outpatient opioid treatment program serving a minority adult population. Eligibility covered methadone-maintained patients coinfected with HIV and hepatitis C who had received or were currently receiving NFV. The yield was 51 case patients. Parameters examined looked into safety, efficacy, and tolerability. Nelfinavir was discontinued in 2 patients for liver function abnormalities but resumed in 1 patient. One patient developed laboratory abnormalities during NFV therapy that were not present before NFV therapy; in 12 case... (truncated)

Title: Maternal Lopinavir/Ritonavir Is Associated with Fewer Adverse Events in Infants than Nelfinavir or Atazanavir.
Combination antiretroviral therapy (cART) is successfully used for prevention of perinatal HIV transmission. To investigate safety, we compared adverse events (AE) among infants exposed to different maternal cART regimens. We reviewed 158 HIV-uninfected infants born between 1997 and 2009, using logistic regression to model grade ≥1 AE and grade ≥3 AE as a function of maternal cART and confounding variables (preterm, C-section, illicit drug use, race, ethnicity, infant antiretrovirals, and maternal viremia). Frequently used cART regimens included zidovudine (63%), lamivudine (80%), ritonavir-boosted lopinavir (37%), nelfinavir (26%), and atazanavir (10%). At birth, anemia occurred in 13/140 infants (9%), neutropenia in 27/107... (truncated)

MESH:D003981 - diazoxide

Summary:

LLM Prediction Score	0.500 (normalized)
LLM Confidence Score	0.980
Golden Answer (Severity Class)	0.375 (normalized)
Prediction Error	0.125

Retrieved Context:

Title: Liver injury may increase the risk of diazoxide toxicity: a case report.
Stress-related hyperinsulinism (HI) may lead to recalcitrant hypoglycemia for weeks or months following perinatal stress, often in premature newborn infants. Diazoxide is an effective and usually safe medication to treat this type and other types of neonatal HI. We report a male infant born at 35-week gestation with severe respiratory distress who developed prolonged hypoglycemia requiring high glucose infusion rates. He also had abnormal liver function tests, including hypoalbuminemia. Laboratory tests were consistent with HI, which responded to diazoxide treatment (10 mg/kg/day started at 10 days of age). The patient developed cardiorespiratory failure, hepatomegaly, worsening liver function tests, and hyperglycemia... (truncated)

Title: A case of insulin-secreting tumor successfully treated with diazoxide.
A 72-year-old patient was referred to our hospital because of a hypoglycemic crisis possibly due to an insulin secreting-tumor. Although its localization was assumed to be at the head of the pancreas as assessed by transhepatic venography with sampling, no mass lesion was detected by selective angiography, ultrasound, computerized axial tomography, magnetic resonance imaging and 67Ga-citrate scan. The patient was treated with diazoxide, because of poor localization and his age, insulin secretion was improved after both 30 and 60 min in the 75 g oral glucose tolerance test. The total amount of urinary C-peptide excretion decreased from 70.8 to 29.2... (truncated)

Title: Drug-induced hypoglycemia. A review of 1418 cases.
The present review catalogues 1418 reported cases of drug-induced hypoglycemia. The main findings are that sulfonylureas (especially chlorpropamide and glyburide), either alone or with a second hypoglycemic or potentiating agent, still account for 63% of all cases; that alcohol, propranolol, and salicylate, either singly or with another hypoglycemic drug, are the next most frequent offenders (19% of the total); and that one older drug (quinine) and three new ones (pentamidine, ritodrine, and disopyramide) have caused an additional 7% of all episodes of severe hypoglycemia. The clinical factors that set the stage for drug-induced hypoglycemia are still restricted food intake, age,... (truncated)

Title: Remote Ischemic Preconditioning and Diazoxide Protect from Hepatic Ischemic Reperfusion Injury by Inhibiting HMGB1-Induced TLR4/MyD88/NF-κB Signaling.
Remote ischemic preconditioning (RIPC) is known to have a protective effect against hepatic ischemia-reperfusion (IR) injury in animal models. However, the underlying mechanism of action is not clearly understood. This study examined the effectiveness of RIPC in a mouse model of hepatic IR and aimed to clarify the mechanism and relationship of the ATP-sensitive potassium channel (K_{ATP}) and HMGB1-induced TLR4/MyD88/NF-κB signaling. C57BL/6 male mice were separated into six groups: (i) sham-operated control, (ii) IR, (iii) RIPC+IR, (iv) RIPC+IR+glyburide (K_{ATP} blocker), (v) RIPC+IR+diazoxide (K_{ATP} opener), and (vi) RIPC+IR+diazoxide+glyburide groups. Histological changes, including hepatic ischemia injury, were assessed. The levels of circulating... (truncated)

Title: Bench-to-bedside review: Antidotal treatment of sulfonylurea-induced hypoglycaemia with octreotide.
The major potential adverse effect of use of sulfonylurea agents (SUAs) is a hyperinsulinaemic state that causes hypoglycaemia. It may be observed during chronic therapeutic dosing, even with very low doses of a SUA, and especially in older patients. It may also result from accidental or intentional poisoning in both diabetic and nondiabetic patients. The traditional approach to SUA-induced hypoglycaemia includes administration of glucose, and glucagon or diazoxide in those who remain hypoglycaemic despite repeated or continuous glucose supplementation. However, these antidotal approaches are associated with several shortcomings, including further exacerbation of insulin release by glucose and glucagon, leading only... (truncated)

MESH:D002220 - carbamazepine

Summary:

LLM Prediction Score	0.750 (normalized)
LLM Confidence Score	0.980
Golden Answer (Severity Class)	0.875 (normalized)
Prediction Error	0.125

Retrieved Context:

Title: [Acute severe hepatic insufficiency caused by carbamazepine].
Acute liver toxicity caused by carbamazepine is a well known though infrequent event. Severe toxicity with hepatocellular insufficiency is even more rare. A case is presented of a patient who suffered of partial epilepsy on treatment with valproate and carbamazepine, who was admitted because of severe acute liver insufficiency attributable to carbamazepine. He had started treatment with the latter drug two weeks earlier, when he developed fever, jaundice, rash and signs of encephalopathy in association with elevation in serum transaminases levels and a decrease in prothrombin index (24%). Discontinuation of both antiepileptic drugs, together with the usual supportive measures, was... (truncated)

Title: [Acute hepatitis due to carbamazepine (Tegretol). Study of a case and review of the literature].
The case of a 28-year-old epileptic woman, treated for 3 months with carbamazepine and who developed jaundice, fever and exfoliative dermatitis is reported. Biological investigations showed an increase in the activity of serum transaminases and alkaline phosphatases as well as in the blood eosinophilic count. Liver biopsy revealed centrolobular necrosis and cholestasis with portal inflammatory infiltrate. Drug challenge was followed by the rapid recurrence of clinical and biological signs. The definitive outcome was favorable. The analysis of the 12 previously published cases, and particularly the frequency of granulomatous hepatitis, suggests an immunoallergic mechanism for the development of carbamazepine hepatitis.

Title: Carbamazepine-provoked hepatotoxicity and possible aetiopathological role of glutathione in the events. Retrospective review of old data and call for new investigation.
The antiepileptic drug (AED) carbamazepine is widely used in the treatment of different kinds of seizures as well as affective and behavioural disorders. This paper presents an epidemiological study of carbamazepine-induced hepatic injuries and death, and describes the possible mechanisms of its toxicity. A retrospective analysis of clinical data revealed that the likelihood of hepatic death was comparatively higher in children, particularly when they were receiving medication with multiple AEDs, whereas reversible hepatic injuries were more likely to be seen in elderly patients. As suggested in this paper, the development of carbamazepine hepatotoxicity is rare, and unpredictable with the present... (truncated)

Title: Acute hepatotoxicity after excessively high doses of carbamazepine on two occasions.
A 2 1/2-year-old child being treated with carbamazepine (CBZ) for a seizure disorder on two separate occasions experienced elevated CBZ serum concentrations (28 and 23.2 mg/L), severe liver damage (SGOT greater than 6000 IU, SGPT greater than 5000 IU), and central nervous system manifestations (coma, lethargy, seizures). During the first episode, the time course of CBZ concentrations exhibited a nonlinear decline and was accompanied by CBZ-10,11-epoxide concentrations that were elevated 4-fold compared to normal values. Cerebrospinal fluid concentrations of CBZ and CBZ-10,11-epoxide were also elevated, although their ratios to serum concentrations did not suggest enhanced permeability of the central nervous... (truncated)

Title: Acetaminophen toxicity with concomitant use of carbamazepine.
Acetaminophen is a widely used analgesic that can cause acute liver failure when consumed above a maximum daily dose. Certain patients may be at increased risk of hepatocellular damage even at conventional therapeutic doses. We report a case of a 34-year-old man on carbamazepine for complex partial seizures who developed acute liver and renal failure on less than 2.5 grams a day of acetaminophen. This raises caution that patients on carbamazepine should avoid chronic use of acetaminophen, and if required use at lower doses with vigilant monitoring for signs of liver damage.

MESH:D015251 - epirubicin

Summary:

LLM Prediction Score	0.500 (normalized)
LLM Confidence Score	0.980
Golden Answer (Severity Class)	0.375 (normalized)
Prediction Error	0.125

Retrieved Context:

Title: Phase I study of cisplatin, irinotecan, and epirubicin administered every 3 weeks in patients with advanced solid tumours.
This phase I study was conducted to determine the recommended phase II doses, safety profile, and antitumour activity of a combination regimen of cisplatin, irinotecan, and epirubicin administered every 3 weeks in patients with advanced solid tumours. Cisplatin and epirubicin were given at fixed doses of 50 and 60 mg m(-2), respectively. The irinotecan dose was escalated at 10 mg m(-2) increments from a starting dose level of 70 mg m(-2). Epirubicin, irinotecan, and their metabolites were measured with HPLC methods. In all, 35 patients received 141 courses of treatment. Irinotecan dose was escalated in seven cohorts up to 130... (truncated)

Title: [Feasibility and toxicity of EC-T dose-dense adjuvant chemotherapy: A real world study in Chinese early-stage breast cancer patients with high recurrence risk].
Objective: We aimed to examine the feasibility and toxicity of EC-T dose-dense regimen and to demonstrate the suitable dose of epirubicin in a Chinese early-stage breast cancer population with high recurrence risk. Methods: 370 patients with early-stage breast cancer at high risk of recurrence were treated with EC-T dose-dense adjuvant chemotherapy and prophylactic administration of recombinant human granulocyte stimulating factor (G-CSF). The incidence of delayed chemotherapy, drug reduction and adverse reactions were retrospectively analyzed. Results: 370 patients completed the planned eight cycles of chemotherapy, 50 patients experienced chemotherapy delay, and 90 had chemotherapy dose reductions. Overall, 61.1% of the patients... (truncated)

Title: Experience with intermediate-dose (110-120 mg/m2) epirubicin.
A total of 23 patients with advanced malignancies received escalating doses of epirubicin (100-120 mg/m2) i.v. at 3-week intervals; 15 had received previous chemotherapy. In all, 46 courses of chemotherapy were given. Mucositis (grade II or III) occurred in 47% of courses at 120 mg/m2, but in only 15% of courses at 115 mg/m2. Myelotoxicity was manifest as leucopenia, with a median white blood count nadir of 1.9 (range, 0.8-7.0) x 10(9)/l. Nausea and vomiting were generally well controlled by prophylactic antiemetic therapy. Alopecia was WHO grade 0 in 2 patients, grade I in 1, grade II in 5 and... (truncated)

Title: Drug-Induced Liver Injury Caused by Capecitabine: A Case Report and a Literature Review.
Chemotherapy is widely used in cancer treatment, and the drug Capecitabine is often used in treatment of breast cancer and usually well-tolerated. Toxicity from Capecitabine typically involves hand-foot syndrome, fatigue, nausea, reduced appetite, and diarrhea, while severe liver toxicity is rarely seen. We present a case of a 63-year-old female with metastatic breast cancer, without liver metastasis, who developed a severe drug-induced liver injury (DILI) with critically elevated liver enzyme levels as reaction to Capecitabine treatment with seemingly no evident explanation as to why. The patient had a RUCAM score of 7 and a Naranjo score of 6 implying that... (truncated)

Title: Evaluation of epirubicin-induced acute oxidative stress toxicity in rat liver cells and mitochondria, and the prevention of toxicity through quercetin administration.
Anticancer therapy with epirubicin (EPI) results in acute hepatotoxicity, likely due to the generation of free radicals. However, the oxidative status of rat liver cells and mitochondria after EPI toxicity has not been investigated. In the present study, we first investigated the pro-oxidant effect of EPI on both hepatic cells and mitochondrial function. Injection of EPI into rats at a dose of 9mg/kg (cumulative dose in human chemotherapy), induced hepatic dysfunction, as revealed by a significant increase in serum glutamate oxaloacetate transaminases (SGOT) and glutamate pyruvate transaminases (SGPT). Oxidative stress in liver cells and mitochondria was provoked by EPI because... (truncated)

MESH:D010645 - phentermine

Summary:

LLM Prediction Score	0.123 (normalized)
LLM Confidence Score	0.980
Golden Answer (Severity Class)	0.0 (normalized)
Prediction Error	0.123

Retrieved Context:

Title: Sympathomimetic amine compounds and hepatotoxicity: Not all are alike-Key distinctions noted in a short review.
Sympathomimetic amine compounds are often pooled together and incorrectly assumed to be interchangeable with respect to potential adverse effects. A brief and specific review of sympathomimetic compounds and one instance (i.e., hepatotoxicity) where these compounds have been improperly grouped together is covered. A review of the proposed mechanisms through which known hepatotoxic sympathomimetic agents (e.g., 3,4-methylenedioxymethamphetamine or MDMA, methamphetamine and amphetamine) cause liver injury, along with a corresponding review of in vitro data, interventional data, animal model studies and observational data allow for a comparison/contrast of different agents and reveals a lack of potential toxicity for some agents (e.g., pseudoephedrine,... (truncated)

Title: Effect of Weight Loss Medications on Hepatic Steatosis and Steatohepatitis: A Systematic Review.
Non-alcoholic fatty liver disease (NAFLD) is a common comorbidity in individuals with obesity. Although multiple pharmacotherapeutics are in development, currently there are limited strategies specifically targeting NAFLD. This systematic review summarizes the existing literature on hepatic effects of medications used for weight loss. Glucagon-like peptide 1 (GLP-1) agonists are the best-studied in this regard, and evidence consistently demonstrates reduction in liver fat content, sometimes accompanied by improvements in histological features of steatohepatitis and reductions in serum markers of hepatic injury such as alanine aminotransferase (ALT). It remains unclear whether these benefits are independent of the weight loss caused by these... (truncated)

Title: Current and future drug targets in weight management.
Obesity will continue to be one of the leading causes of chronic disease unless the ongoing rise in the prevalence of this condition is reversed. Accumulating morbidity figures and a shortage of effective drugs have generated substantial research activity with several molecular targets being investigated. However, pharmacological modulation of body weight is extremely complex, since it is essentially a battle against one of the strongest human instincts and highly efficient mechanisms of energy uptake and storage. This review provides an overview of the different molecular strategies intended to lower body weight or adipose tissue mass. Weight-loss drugs in development include... (truncated)

Title: Obesity: Current and potential pharmacotherapeutics and targets.
Obesity is a global epidemic that contributes to a number of health complications including cardiovascular disease, type 2 diabetes, cancer and neuropsychiatric disorders. Pharmacotherapeutic strategies to treat obesity are urgently needed. Research over the past two decades has increased substantially our knowledge of central and peripheral mechanisms underlying homeostatic energy balance. Homeostatic mechanisms involve multiple components including neuronal circuits, some originating in hypothalamus and brain stem, as well as peripherally-derived satiety, hunger and adiposity signals that modulate neural activity and regulate eating behavior. Dysregulation of one or more of these homeostatic components results in obesity. Coincident with obesity, reward mechanisms... (truncated)

Title: Comprehensive Review of Current and Upcoming Anti-Obesity Drugs.
Obesity is among the leading causes of morbidity and mortality worldwide and its prevalence continues to increase globally. Because obesity is a chronic, complex, and heterogeneous disease influenced by genetic, developmental, biological, and environmental factors, it is necessary to approach obesity with an integrated and comprehensive treatment strategy. As it is difficult to achieve and sustain successful long-term weight loss in most patients with obesity through lifestyle modifications (e.g., diet, exercise, and behavioral therapy), pharmacological approaches to the treatment of obesity should be considered as an adjunct therapy. Currently, four drugs (orlistat, naltrexone extended-release [ER]/bupropion ER, phentermine/topiramate controlled-release, and liraglutide)... (truncated)

MESH:D004298 - dopamine

Summary:

LLM Prediction Score	0.123 (normalized)
LLM Confidence Score	0.990
Golden Answer (Severity Class)	0.0 (normalized)
Prediction Error	0.123

Retrieved Context:

Title: [Clinical and experimental investigations of the effect of dopamine on haemodynamics and function of kidney and liver (author's transl)].
Increased incidence of renal insufficiency is observed in severe damage of liver parenchyma such as fulminant hepatitis, decompensated cirrhosis of the liver, septic cholangitis and the different forms of obstructive jaundice. Functional circulatory disturbances of the kidney, especially of the renal cortex, are of importance in the aetiology of this condition. Dopamine, at a dosage as low as 3 gamma/kg/min leads to an improvement in renal blood flow and also to an increase in hepatic blood flow. These observations are of therapeutic importance. Some important circulatory and functional parameters of both these organs, which influence each other under normal and... (truncated)

Title: Induction of mitochondrial permeability transition (MPT) pore opening and ROS formation as a mechanism for methamphetamine-induced mitochondrial toxicity.
During the past 10 years, the use of methamphetamine (METH) has significantly increased in Iran and around the world. The widespread use of 3,4-methylenedioxymethamphetamine as a recreational drug has been responsible for the incidence of several cases of liver failure in young people. This issue made researchers focus on METH toxicity due to the lack of effective treatment and human health risk assessment. There are several reports showing that its long-term use increases the risk for dopamine depletion, but the toxicity mechanisms of METH in liver are not well understood. Therefore, we aimed to investigate the mitochondrial toxicity mechanisms of... (truncated)

Title: Chlorogenic and caftaric acids in liver toxicity and oxidative stress induced by methamphetamine.
Methamphetamine intoxication can cause acute hepatic failure. Chlorogenic and caftaric acids are the major dietary polyphenols present in various foods. The aim of this study was to evaluate the protective role of chlorogenic and caftaric acids in liver toxicity and oxidative stress induced by methamphetamine in rats. Thirty-two male albino rats were divided into 4 equal groups. Group 1, which was control group, was injected (i.p) with saline (1 mL/kg) twice a day over seven-day period. Groups 2, 3, and 4 were injected (i.p) with methamphetamine (10 mg/kg) twice a day over seven-day period, where groups 3 and 4 were... (truncated)

Title: Aripiprazole reduces liver cell division.
Effects of aripiprazole on dopamine regulation are being tested as a treatment for patients with a dual diagnosis of schizophrenia and addictions, often cocaine dependence. Aripiprazole has one of the fewest side-effects among the second-generation antipsychotics. Nevertheless, severe aripiprazole hepatotoxicity was reported in persons with a history of cocaine and alcohol abuse. Here we report that therapeutically relevant aripiprazole concentrations, equal to laboratory alert levels in patients' serum, reduce the rate of hepatocytes' division. This could be an underlying mechanism of severe liver injury development in the patients with a history of alcohol and cocaine abuse, the two hepatotoxic agents... (truncated)

Title: Could Alcohol Abuse and Dependence on Junk Foods Inducing Obesity and/or Illicit Drug Use Represent Danger to Liver in Young People with Altered Psychological/Relational Spheres or Emotional Problems?
Recent data show that young people, mainly due to the pressure of some risk factors or due to disrupted interpersonal relationships, utilise greater reward value and display greater sensitivity to the reinforcing properties of "pleasurable stimuli", specifically in those situations in which an enhanced dopamine release is present. Alcoholic beverages, foods rich in sugar and fat, and illicit drug use are pleasurable feelings associated with rewards. Research shows that there is a link between substance abuse and obesity in brain functioning. Still, alcohol excess is central in leading to obesity and obesity-related morbidities, such as hepatic steatosis, mainly when associated... (truncated)

MESH:D016293 - moricizine

Summary:

LLM Prediction Score	0.503 (normalized)
LLM Confidence Score	0.760
Golden Answer (Severity Class)	0.625 (normalized)
Prediction Error	0.122

Retrieved Context:

Title: The effect of hepatic disease on the disposition of moricizine in humans.
The pharmacokinetics of moricizine and two of its metabolites, moricizine sulfoxide and phenothiazine-2-carbamic acid ethyl ester sulfoxide, were studied in healthy control subjects and in patients with chronic liver disease (cirrhosis). Moricizine disposition was significantly altered by hepatic cirrhosis. Compared to healthy subjects, the hepatic disease patients had an increased Cmax (59%), an increased t1/2 (141%), and a reduced plasma clearance (71%). Additionally, small but statistically significant increases were observed for tmax and the fraction of moricizine not bound to plasma proteins in patients with hepatic disease. The elimination of both moricizine metabolites was also altered by hepatic dysfunction as... (truncated)

Title: Association of CYP1A1 and CYP1B1 inhibition in in vitro assays with drug-induced liver injury.
Drug-induced liver injury (DILI) is one of the major causes for the discontinuation of drug development and withdrawal of drugs from the market. Since it is known that reactive metabolite formation and being substrates or inhibitors of cytochrome P450s (P450s) are associated with DILI, we systematically investigated the association between human P450 inhibition and DILI. The inhibitory activity of 266 DILI-positive drugs (DILI drugs) and 92 DILI-negative drugs (no-DILI drugs), which were selected from Liver Toxicity Knowledge Base (US Food and Drug Administration), against 8 human P450 forms was assessed using recombinant enzymes and luminescent substrates, and the threshold values... (truncated)

Title: Genophenotypic Factors and Pharmacogenomics in Adverse Drug Reactions.
Adverse drug reactions (ADRs) rank as one of the top 10 leading causes of death and illness in developed countries. ADRs show differential features depending upon genotype, age, sex, race, pathology, drug category, route of administration, and drug-drug interactions. Pharmacogenomics (PGx) provides the physician effective clues for optimizing drug efficacy and safety in major problems of health such as cardiovascular disease and associated disorders, cancer and brain disorders. Important aspects to be considered are also the impact of immunopharmacogenomics in cutaneous ADRs as well as the influence of genomic factors associated with COVID-19 and vaccination strategies. Major limitations for the... (truncated)

Title: Analysis of transcriptomic responses to SARS-CoV-2 reveals plausible defective pathways responsible for increased susceptibility to infection and complications and helps to develop fast-track repositioning of drugs against COVID-19.
To understand the transcriptomic response to SARS-CoV-2 infection, is of the utmost importance to design diagnostic tools predicting the severity of the infection.

Title: Rate-control or rhythm-control: where do we stand?
Atrial fibrillation is the most common sustained rhythm disturbance and its prevalence is increasing worldwide due to the progressive aging of the population. Current guidelines clearly depict the gold standard management of acute symptomatic atrial fibrillation but the best-long term approach for first or recurrent atrial fibrillation is still debated with regard to quality of life, risk of new hospitalizations, and possible disabling complications, such as thromboembolic stroke, major bleeds and death. Some authors propose that regaining sinus rhythm in all cases, thus re-establishing a physiologic cardiac function not requiring a prolonged antithrombotic therapy, avoids the threat of intracranial or... (truncated)

MESH:D015474 - isotretinoin

Summary:

LLM Prediction Score	0.503 (normalized)
LLM Confidence Score	0.990
Golden Answer (Severity Class)	0.625 (normalized)
Prediction Error	0.122

Retrieved Context:

Title: Prevalence of adverse events varies with the different oral isotretinoin brands in acne treatment: a retrospective observational study.
Oral isotretinoin remains the most effective treatment for acne. The aim of this retrospective single-center cohort study was to estimate the prevalence of adverse events with the different oral isotretinoin brands used in acne treatment. The population consisted of all patients who consulted for acne between January 2015 and January 2020. The inclusion criterion was the initiation of treatment with oral isotretinoin. The exclusion criteria were the use of two or more brands during the same course of treatment and previous treatment with oral isotretinoin. Statistical analysis was carried out using Chi-square and Mann-Whitney tests. We analyzed 468 patients of... (truncated)

Title: Isotretinoin treatment induces oxidative toxicity in blood of patients with acne vulgaris: a clinical pilot study.
Acne vulgaris is the one of the most common skin diseases. Although isotretinoin (13-cis-retinoic acid) is an effective and well-tolerated medication, it has a wide range of side effects. Because the effects of isotretinoin on oxidant and antioxidant systems have not yet been clarified, we investigated plasma and erythrocyte antioxidant vitamins, lipid peroxidation (LP), reduced glutathione (GSH) and glutathione peroxidase (GSH-Px) values in patients with acne vulgaris before and after isotretinoin treatment. The study was performed on the blood plasma and erythrocytes of 31 acne vulgaris patients. Blood samples were taken from the patients before treatment and after isotretinoin (oral... (truncated)

Title: Dietary Supplements, Isotretinoin, and Liver Toxicity in Adolescents: A Retrospective Case Series.
Isotretinoin is the most effective acne therapy available, but has the potential for a number of adverse side effects, including transaminitis. The iPLEDGE isotretinoin program recommends avoiding some herbals and supplements due to potential side effects. However, little is known about the effects of protein supplements on the liver, particularly in patients taking isotretinoin. We designed a retrospective chart review to evaluate the symptoms, diagnosis, treatment, and outcome of patients on or preparing to take isotretinoin therapy who were concurrently ingesting protein or herbal supplementation and who developed transaminitis. In 100% (8/8) of cases, dietary supplementation was determined to be... (truncated)

Title: Isotretinoin: a reappraisal.
Isotretinoin is remarkably effective in the treatment of severe cystic acne, however, many complications have been observed during treatment and new toxic effects have been reported. Hypertriglyceridemia associated with decreases in high density lipoprotein (HDL) cholesterol has occurred in 25 percent of patients and requires monitoring during treatment. Painful erosions with granulation tissue recently have been reported in patients with severe acne. Other complications have included corneal opacities, pseudotumor cerebri, hypercalcemia, photosensitivity reactions, abnormal liver function tests, and skeletal hyperostosis. Isotretinoin is teratogenic and should be avoided during pregnancy. With the increasing acceptance and use of isotretinoin for cystic acne,... (truncated)

Title: The relevance of nanotechnology, hepato-protective agents in reducing the toxicity and augmenting the bioavailability of isotretinoin.
Acne Vulgaris is one of the most common chronic inflammatory skin disorders that affect majority of teen-agers worldwide. Isotretinoin (ITT) is the drug of choice in the management of acne, but, it suffers from serious side-effects including hepatotoxicity, and some psychological disturbances following its oral intake. The objective of this study was to develop and optimize ITT loaded nanoemulsions (ITT-SNEDDS) and to incorporate resveratrol (RSV)in optimum formulation to decrease ITT side effects The ITT solubility was first tested in various essential oils, surfactants, and co-surfactants to select the essential nanoemulsion ingredients. Mixture design was applied to study the effect of... (truncated)

MESH:C437965 - olmesartan

Summary:

LLM Prediction Score	0.378 (normalized)
LLM Confidence Score	0.980
Golden Answer (Severity Class)	0.5 (normalized)
Prediction Error	0.122

Retrieved Context:

Title: Sprue-Like Enteropathy and Liver Injury: A Rare Emerging Association with Olmesartan.
Olmesartan-induced enteropathy is an underreported phenomenon, first described in 2012. While olmesartan's antihypertensive properties were confirmed early on, its association with a sprue-like enteropathy was subsequently noted. Although this association has been reported with olmesartan, there have been few reports of this association with other angiotensin-receptor blockers. We present a case of a 79-year-old male who presented with diarrhea, weight loss, jaundice, and transaminitis. Further history revealed that he had been taking olmesartan 40 mg daily for hypertension. Workup of his diarrhea and jaundice included duodenal and liver biopsies revealed findings consistent with a sprue-like enteropathy and an autoimmune hepatitis-like... (truncated)

Title: Modulation of carbon tetrachloride-induced hepatic oxidative stress, injury and fibrosis by olmesartan and omega-3.
This study was designed to investigate the potential effects of omega-3, olmesartan and their combination on established hepatic fibrosis in the carbon tetrachloride (CCl4) rat model. Male Wistar rats received subcutaneous injections of CCl4 twice weekly for 12weeks, as well as daily oral treatments of olmesartan (1 and 3mg/kg), omega-3 (75 and 150mg/kg) and their combination during the last 4weeks of intoxication. Our results indicated that omega-3 and, to a lesser extent, olmesartan dose-dependently blunted CCl4-induced necroinflammation scoring and elevation of liver injury parameters in serum. Besides, omega-3 and, to a lesser extent, olmesartan treatments in a dose dependent manner... (truncated)

Title: Olmesartan-induced Enteropathy: A Rare Side Effect of a Common Medication.
Ischemic heart disease and stroke are the leading causes of mortality worldwide according to the World Health Organization. Hypertension is a major factor in the development of these diseases. Olmesartan is an angiotensin II receptor blocker (ARB) indicated in the treatment of hypertension. There are several case reports describing sprue-like enteropathy caused by olmesartan. We report on a 72-year-old patient referred to our hospital for work-up of chronic diarrhoea, vomiting and weight-loss, and villous atrophy on intestinal biopsy. The patient's symptoms abated upon cessation of olmesartan. This case illustrates the need for a thorough medication history and regular review during... (truncated)

Title: Olmesartan-Induced Enteropathy: An Unusual Cause of Villous Atrophy.
We report a case of a 63-year-old-man presenting with chronic diarrhea and weight loss while on olmesartan treatment for hypertension. Investigation showed multiple nutritional deficiencies associated with diffuse intestinal villous atrophy. Serologies for celiac disease were negative and other causes of villous atrophy were excluded. Olmesartan as a precipitant agent was suspected and withdrawn. Clinical improvement occurred in days with no need for other therapeutic measures. Follow-up at three months showed clinical remission and almost complete recovery of intestinal atrophy. Olmesartan is an angiotensin receptor blocker commonly prescribed for the management of hypertension. Spruelike enteropathy associated with this drug is... (truncated)

Title: Drug-induced digestive tract injury: decoding some invisible offenders.
There is an ever-growing list of pharmacological agents, several of which are attributed to cause clinically significant gastrointestinal (GI) injury. Many patients present with significant but nonspecific symptoms, that in conjunction with the absence of relevant drug history on the requisition slip can make the histopathologic diagnosis challenging. To complicate this, although some drugs have relatively characteristic histopathologic features (such as doxycycline), there exist many other drugs that exhibit wide and varying spectra of histopathologic findings (such as immune checkpoint inhibitors or olmesartan) and have histomorphologic overlap with many other commonly encountered disease entities. This review discusses the histopathologic features... (truncated)

MESH:D000077552 - basiliximab

Summary:

LLM Prediction Score	0.120 (normalized)
LLM Confidence Score	0.950
Golden Answer (Severity Class)	0.0 (normalized)
Prediction Error	0.120

Retrieved Context:

Title: PD-1 Checkpoint Blockade in Patients for Acute Myeloid Leukemia after HSCT Relapse Resulted in Severe GVHD and sHLH.
Treatment with immune checkpoint inhibitors (ICI) such as carrizumab leads to immune-mediated adverse effects including severe acute graft versus host disease (aGVHD) and secondary hemophagocytic syndrome (sHLH). Herein, we present two cases where aGVHD and sHLH developed after ICI administration, which was treated using methylprednisolone (MP). They developed high-grade fever complicated with liver dysfunction and diarrhea 1 day after ICI administration. Treatment with MP does not alleviate symptoms because of steroid resistance. Hyperbilirubinemia, rash with blisters, and watery diarrhea showed severe aGVHD. Hyperferritinemia, hypertriglyceridemia, and cytopenias suggested the diagnosis of HLH and met the criteria for sHLH diagnosis. They were... (truncated)

Title: Rapid steroid withdrawal in hepatitis C virus-positive kidney transplant recipients.
The effects of rapid steroid withdrawal (SW) on kidney transplantation (KT) outcome were investigated in 12 HCV+ patients in a prospective cohort study. These results were compared with 17 HCV+ patients who received KT in the prior 2 yr and treated with a standard prednisone taper protocol. SW patients received only 6 d of steroid treatment after transplantation. Eleven received Thymoglobulin and one Basiliximab induction treatment along with a calcineurin inhibitor and mycophenolate mofetil. Patient and graft survival was 92% in SW group (median follow-up 12 months, range 6-17), and 92 and 82% in the historic control group respectively (median... (truncated)

Title: Double-blind comparison of hepatitis C histological recurrence Rate in HCV+ Liver transplant recipients given basiliximab + steroids or basiliximab + placebo, in addition to cyclosporine and azathioprine.
Hepatitis C virus (HCV) recurrence in HCV+ liver transplant recipients is almost inevitable and may be promoted by immunosuppression. We compared the amount of liver damage with regard to usage of steroids and basiliximab.

Title: Fulminant zygomycosis of graft liver following liver transplantation.
A 44-year-old man with hepatitis B virus (HBV)-related cirrhosis underwent living donor liver transplantation at our institute. Induction of immunosuppression was achieved with basiliximab, due to deranged renal function, and maintained with prednisolone, tacrolimus and mycophenolate mofetil. The intraoperative and immediate postoperative periods were fairly uneventful. A duplex scan, taken during the third week post-transplantation due to sudden rise in liver enzymes, revealed multifocal hypoechoic lesions in the graft liver with normal Doppler parameters. Multidetecor computed tomography (MDCT) showed multiple hypodense vessel-sparing lesions in the graft liver. Cultures from the aspirate grew filamentous fungi identified as Basidiobolus ranarum species. Despite... (truncated)

Title: Early Switch From Tacrolimus to Everolimus After Liver Transplantation: Outcomes at 2 Years.
The observational CERTITUDE study follows liver transplant patients who completed the SIMCER trial. SIMCER randomized patients at month 1 after transplant to everolimus (EVR) with stepwise tacrolimus (TAC) withdrawal or to standard TAC, both with basiliximab induction and mycophenolic acid ± steroids. After completing SIMCER at 6 months after transplant, 65 EVR-treated patients and 78 TAC-treated patients entered CERTITUDE. At month 24 after transplant, 34/65 (52.3%) EVR-treated patients remained calcineurin inhibitor (CNI) free. Mean estimated glomerular filtration rate (eGFR) was significantly higher with EVR versus TAC during months 3-12. At month 24, eGFR values were 83.6 versus 75.3 mL/minute/1.73 m² , respectively (P = 0.90)... (truncated)

MESH:C100118 - ibufenac

Summary:

LLM Prediction Score	0.880 (normalized)
LLM Confidence Score	0.690
Golden Answer (Severity Class)	1.0 (normalized)
Prediction Error	0.120

Retrieved Context:

Title: Effects of non-narcotic analgesics on the liver.
Serious hepatotoxicity is uncommon with the proper therapeutic use of non-narcotic analgesics but experience with new non-steroidal anti-inflammatory drugs (NSAIDs) is limited. Drugs such as ibufenac, fenclofenac and benoxaprofen were withdrawn from the market because of hepatotoxicity, and liver damage has been reported on occasion with virtually all non-narcotic analgesics. However, a clear pattern of toxicity with characteristic clinical, biochemical and histopathological abnormalities has emerged with relatively few. With the exception of acute hepatic necrosis following overdosage of paracetamol, little is known of the mechanisms of liver injury induced by non-narcotic analgesics. Involvement of the liver in a generalised drug... (truncated)

Title: Non-steroidal anti-inflammatory drugs: What is the actual risk of liver damage?
Non-steroidal anti-inflammatory drugs (NSAIDs) constitute a family of drugs, which taken as a group, represents one of the most frequently prescribed around the world. Thus, not surprisingly NSAIDs, along with anti-infectious agents, list on the top for causes of Drug-Induced Liver Injury (DILI). The incidence of liver disease induced by NSAIDs reported in clinical studies is fairly uniform ranging from 0.29/100 000 [95% confidence interval (CI): 0.17-051] to 9/100 000 (95% CI: 6-15). However, compared with these results, a higher risk of liver-related hospitalizations was reported (3-23 per 100 000 patients). NSAIDs exhibit a broad spectrum of liver damage ranging... (truncated)

Title: Exploring Chronic Drug Effects on Microengineered Human Liver Cultures Using Global Gene Expression Profiling.
Global gene expression profiling is useful for elucidating a drug's mechanism of action on the liver; however, such profiling in rats is not very sensitive for predicting human drug-induced liver injury, while dedifferentiated monolayers of primary human hepatocytes (PHHs) do not permit chronic drug treatment. In contrast, micropatterned cocultures (MPCCs) containing PHH colonies and 3T3-J2 fibroblasts maintain a stable liver phenotype for 4-6 weeks. Here, we used MPCCs to test the hypothesis that global gene expression patterns in stable PHHs can be used to distinguish clinical hepatotoxic drugs from their non-liver-toxic analogs and understand the mechanism of action prior to... (truncated)

Title: Ibuprofen Increases the Hepatotoxicity of Ethanol through Potentiating Oxidative Stress.
Over 30 million prescriptions of NSAIDs (non-steroidal anti-inflammatory drugs) are issued every year. Considering that these drugs are available without a prescription as over the counter (OTC) drugs, their use will be astronomical. With the increasing use of NSAIDs, their adverse effects are drawing attention. Especially, stomach bleeding, kidney toxicity, liver toxicity, and neurological toxicity are reported as common. Ibuprofen, one of the extensively used NSAIDs along with aspirin, can also induce liver toxicity, but few studies are addressing this point. Here we examined the liver toxicity of ibuprofen and investigated whether co-exposure to ethanol can manifest synergistic effects. We... (truncated)

Title: [Hepatitis due to nonsteroidal anti-inflammatory agents].
The extended prescription of non-steroidal anti-inflammatory drugs in medical practice involve numerous adverse effects. Among them, hepatic injuries, rather uncommon, are very diverse with regard to clinical type and evolution scheme, according to the derivatives used. Salicylates, when taken at high doses, increase serum transaminases, mostly without overt clinical symptoms. Phenylbutazone is obviously hepatotoxic: it induces cytolytic hepatitis, in some cases with fatal issue. Among the indole derivatives, indometacine was involved, especially in children; mixed hepatitis have been noted during sulindac therapy, mostly with favourable outcome. In the group of propionic acid derivatives, ibuprofen, pirprofen and naproxen have been implicated... (truncated)

MESH:D003676 - deferoxamine

Summary:

LLM Prediction Score	0.119 (normalized)
LLM Confidence Score	0.990
Golden Answer (Severity Class)	0.0 (normalized)
Prediction Error	0.119

Retrieved Context:

Title: Acute liver failure due to iron overdose in an adult.
The vast majority of acute iron toxicity cases occur in children less than 5 years of age. Moreover, clinical hepatic injury is uncommon with most symptoms stemming from the intestinal tract (eg, nausea, vomiting, diarrhea). Therefore, physicians, particularly those who do not routinely treat pediatric patients, are often unfamiliar with hepatotoxicity related to iron overdose. Nevertheless, hepatotoxicity caused by acute iron poisoning is associated with a high mortality rate. We report a case of severe hepatic injury in an adult who overdosed on iron tablets with suicidal intent. Tests for other hepatotoxins (eg, acetaminophen), hepatatrophic viruses, and other causes of... (truncated)

Title: The role of Iron in lipid peroxidation and protein nitration during acetaminophen-induced liver injury in mice.
Acetaminophen (APAP) hepatotoxicity, a leading cause of acute liver failure in western countries, is characterized by mitochondrial superoxide and peroxynitrite formation. However, the role of iron, especially as facilitator of lipid peroxidation (LPO), has been controversial. Our aim was to determine the mechanism by which iron promotes cell death in this context. Fasted male C57BL/6J mice were treated with the iron chelator deferoxamine, minocycline (inhibitor of the mitochondrial calcium uniporter) or vehicle 1 h before 300 mg/kg APAP. Deferoxamine and minocycline significantly attenuated APAP-induced elevations in serum alanine amino transferase levels and hepatic necrosis at 6 h. This protection correlated with reduced 3-nitro-tyrosine... (truncated)

Title: Iron-chelation therapy with oral chelators in patients with thalassemia major.
In thalassemia major (TM), without iron chelation therapy, iron-mediated free radical damage causes liver, endocrine, and myocardial toxicities. Deferoxamine has universally been the standard therapeutic option for iron chelation therapy; however, its usage is troublesome, leading to suboptimal patient compliance. In order to maximize the effectiveness of iron chelation therapy, oral iron chelators deferiprone and deferasirox constitute an important development, offering a potential to improve compliance. Although both oral drugs are effective, they have differences including different pharmacokinetics and side-effect profiles. Our retrospective evaluation of TM patients using oral chelators showed that oral chelators are effective in reducing iron overload... (truncated)

Title: Pediatric liver transplantation for fulminant hepatic failure secondary to intentional iron overdose.
Acute iron poisoning may lead to life-threatening hepatotoxicity. We present the cases of two pediatric patients with hepatotoxicity following intentional iron ingestion that progressed rapidly to fulminant hepatic failure despite treatment with deferoxamine. Liver transplantation was lifesaving in both patients. These cases emphasize the need for a high index of suspicion for iron ingestion, close monitoring for liver toxicity, and timely consideration for liver transplantation.

Title: Longitudinal Study on Liver Functions in Patients with Thalassemia Major before and after Deferasirox (DFX) Therapy.
By performing regular blood transfusion and iron chelation therapy, most patients with beta thalassemia major (BTM) now survive beyond the third decade of life. Liver disease is becoming an important cause of morbidity and mortality in these patients. Chronic hepatitis and/or severe iron overload are both important causes of liver pathology. Iron chelation with desferrioxamine (DFO) reduces excessive body iron, but its efficacy is limited by poor compliance and dose related toxicity. The recent use of Deferasirox (DFX), an oral single dose therapy, has improved the compliance to chelation.

MESH:C028797 - pralidoxime

Summary:

LLM Prediction Score	0.256 (normalized)
LLM Confidence Score	0.990
Golden Answer (Severity Class)	0.375 (normalized)
Prediction Error	0.119

Retrieved Context:

Title: Human HepaRG liver spheroids: cold storage protocol and study on pyridinium oxime-induced hepatotoxicity in vitro.
Oximes play an essential role in the therapy of organophosphorus compound (OP) poisoning by reactivating inhibited acetylcholinesterase. Impairment of liver function was observed in OP poisoning and associated with obidoxime treatment by some reports. In this study human three-dimensional HepaRG spheroids were used as complex in vitro model to investigate oxime-induced liver toxicity. In this context, cold storage of liver spheroids at 4 °C in standard culture medium and in optimized tissue preservation solutions of up to 72 h was assessed. Cold storage in standard culture medium resulted in a complete loss of viability whereas an optimized tissue preservation solution preserved viability.... (truncated)

Title: Pesticide poisoning.
Acute poisoning with pesticides is a global public health problem and accounts for as many as 300,000 deaths worldwide every year. The majority of deaths occur due to exposure to organophosphates, organochlorines and aluminium phosphide. Organophosphate compounds inhibit acetylcholinesterase resulting in acute toxicity. Intermediate syndrome can develop in a number of patients and may lead to respiratory paralysis and death. Management consists of proper oxygenation, atropine in escalating doses and pralidoxime in high doses. It is Important to decontaminate the skin while taking precautions to avoid secondary contamination of health personnel. Organochlorine pesticides are toxic to the central nervous system... (truncated)

Title: Successful management of a kitten with chlorpyrifos and cypermethrin toxicosis with pralidoxime and atropine.
Organophosphates and pyrethroids have been widely used as agricultural and domestic insecticides. This case report describes a 3-month-old free-roaming female kitten, weighing 930 g, that developed hypersalivation, hypothermia, dyspnoea due to increased bronchial secretion, bradycardia, miosis and neurological signs, including restlessness, ataxia, disorientation, apparent hallucination, muscle twitching and seizures within 6 h of accidental ingestion of an insecticide containing chlorpyrifos (500 g/l) and cypermethrin (50 g/l). The kitten was treated empirically with intramuscular atropine and dexamethasone, and rectal diazepam. The history of insecticide exposure was obtained after 6 h of treatment and intramuscular 2-pyridine aldoxime methochloride (pralidoxime [2-PAM]) and atropine... (truncated)

Title: The Traditional Uses, Phytochemistry, Pharmacology, Toxicology, and Clinical Uses of <i>Metagentiana Rhodantha</i> (Franch.) T.N.Ho and S.W.Liu, an Ethnomedicine in Southwest China.
Background: <i>Metagentiana rhodantha</i> (Franch.) T.N.Ho and S.W.Liu (MR) belongs to Gentianales<i>,</i> and it is often called Hong-hua-long-dan in Chinese. Traditionally, it has been used to cure acute icteric hepatitis, sore throat, dysentery, acute gastritis, carbuncle, and furuncle based on traditional Chinese medicine (TCM) concepts. Aim of Study: This review manages to provide a critical and comprehensive analysis on the traditional uses, phytochemistry, pharmacology, toxicology, and clinical uses of MR and to evaluate the therapeutic potential of this plant. Methods: Relevant data mainly literatures on MR were selected from available database. All the papers reviewed provided evidence that the source herbs... (truncated)

Title: A cembranoid protects acute hippocampal slices against paraoxon neurotoxicity.
Many neurotoxic organophosphates (OPs) inhibit acetylcholinesterase (AChE) and as a result can cause a life threatening cholinergic crisis. Current medical countermeasures, which typically include atropine and oximes target the cholinergic crisis and are effective in decreasing mortality but do not sufficiently protect against delayed neurological deficits. There is, therefore, a need to develop neuroprotective drugs to prevent long-term neurological deficits. We used acute hippocampal slices to test the hypothesis that 4R,6R-cembratrienediol (4R) protects against functional damage caused by the OP paraoxon (POX). To assess hippocampal function, we measured synaptically evoked population spikes (PSs). Application of 4R reversed POX inhibition of... (truncated)

MESH:D008942 - mitoxantrone

Summary:

LLM Prediction Score	0.494 (normalized)
LLM Confidence Score	0.980
Golden Answer (Severity Class)	0.375 (normalized)
Prediction Error	0.119

Retrieved Context:

Title: [Phase II study of mitoxantrone].
A phase II clinical trial of an anthraquinone, mitoxantrone was performed in a total of 31 patients with various advanced solid tumors and 2 patients with malignant lymphomas refractory to extensive prior chemotherapies. Mitoxantrone was administered at dosages of 2 to 4 mg/m2 with a 5-day schedule or 6 to 12 mg/m2 with a one day schedule, repeating at 4-week intervals. Of 18 evaluable patients with breast cancer, one out of 2 patients who had not been exposed to regimens containing adriamycin achieved partial response lasting 3.6 months, while the remaining 16 patients exposed to adriamycin did not respond. Leukopenia... (truncated)

Title: Treatment of refractory or relapsed adult acute leukemia by using mitoxantrone-containing regimens.
Twenty-eight adult patients with primary refractory or relapsed acute leukemia were treated. The regimens consisted of mitoxantrone plus cytosine arabinoside for 17 patients with acute non-lymphocytic leukemia (ANLL) and mitoxantrone accompanied with vincristine and prednisolone for 11 patients with acute lymphoblastic leukemia (ALL). In primary refractory patients, 1 of the 4 (25%) ANLL and 1 of the 3 (33%) ALL attained complete remission (CR). Excluding 2 patients who underwent bone marrow transplantation, 8 of the 13 (62%) relapsed ANLL and 4 of the 8 (50%) relapsed ALL achieved CR with a median duration of remission of 6.2 months and 3.8... (truncated)

Title: Long-term follow-up of pediatric patients treated with mitoxantrone for multiple sclerosis.
The chemotherapeutic agent mitoxantrone is approved for the treatment of aggressive multiple sclerosis (MS) in adults. Its use, however, is limited by the risk of severe adverse events including cardiotoxicity, myelosuppression, liver toxicity and secondary leukemia. The aim of this retrospective study is to present data on the safety, tolerability and efficacy of mitoxantrone in a small cohort of children with MS. 4 pediatric MS patients with a high relapse rate or severe, disabling relapses were treated with mitoxantrone and followed for 3.8-18 years. The cumulative dose of mitoxantrone was 36, 68, 84 and 120 mg/m (2), respectively. The frequency... (truncated)

Title: Mitoxantrone, cytosine-arabinoside and 6-thioguanine (MAT) in the treatment of newly diagnosed acute non-lymphoblastic leukemia in adults.
Between January 1986 and September 1989, 28 patients over the age of 14 years were treated with a combination of mitoxantrone, cytosine-arabinoside (Ara-C) and 6-thioguanine (MAT) at the Kuwait Cancer Control Centre (KCCC). All patients were newly diagnosed cases of acute non-lymphoblastic leukemia (ANLL). Fifty-eight courses of treatment were given as induction and consolidation therapy. The main toxicity was bone marrow suppression. Other toxicities were mainly nausea and vomiting, hepatotoxicity, renal dysfunction and alopecia. In most patients these were mild and tolerable. Complete remission (CR) was achieved in 18 out of 28 (64%) patients. In six patients it was achieved... (truncated)

Title: [Clinical study of cyclic intra-arterial chemotherapy using mitoxantrone for advanced hepatocellular carcinoma].
Cyclic intra-arterial infusion chemotherapy using Mitoxantrone via reservoir system was prescribed for 8 patients with advanced hepatocellular carcinoma. According to the clinical staging system, two patients were in Stage III and the other six patients in Stage IV-A. Vp-3 portal invasion was observed in six out of the eight patients. Mitoxantrone (5-8 mg/body) was administered intra-arterially via port-a-cath device every 4 weeks. For tumor response, there were two PR cases, four NC cases, and two PD cases. Thus, the response rate was 25%. The mean survival period was 6.5 months, and 1-year survival rate was 13%. In one of two... (truncated)

MESH:D010419 - pentamidine

Summary:

LLM Prediction Score	0.492 (normalized)
LLM Confidence Score	0.980
Golden Answer (Severity Class)	0.375 (normalized)
Prediction Error	0.117

Retrieved Context:

Title: Pentamidine aerosol versus trimethoprim-sulfamethoxazole for Pneumocystis carinii in acquired immune deficiency syndrome.
Pneumocystis carinii pneumonia remains one of the most common opportunistic infections in patients with acquired immune deficiency syndrome (AIDS). Treatment with either intravenous pentamidine or trimethoprim-sulfamethoxazole (TMP-SMX) is frequently complicated by serious adverse reactions. This study was a prospective, blinded comparison of 600 mg/d of pentamidine as an aerosol versus 15 mg/kg/d of trimethoprim plus 75 mg/kg/d of sulfamethoxazole for patients with mild or moderately severe P. carinii pneumonia (alveolar arterial oxygen difference of less than 55 mm Hg). Of 367 participants who were randomized to receive study therapies, 287 had proven and 16 had presumed Pneumocystis pneumonia. There were... (truncated)

Title: Adverse reactions associated with pentamidine isethionate in AIDS patients: recommendations for monitoring therapy.
Since pentamidine has become commercially available, there is renewed interest in using it as the initial treatment for Pneumocystis carinii pneumonia (PCP) in AIDS patients. We reviewed the use of pentamidine in 24 patients with PCP to gain information on the prevalence and severity of adverse effects from this drug. Twenty out of twenty-four patients (83 percent) experienced some kind of adverse effect. Hepatic abnormalities (58 percent), nausea and vomiting (46 percent), hypoglycemia (33 percent), azotemia (25 percent), and pain at the injection site (25 percent) were the most frequently seen effects. We recommend monitoring guidelines to be applied during... (truncated)

Title: Visceral leishmaniasis unresponsive to antimonial drugs. II. Response to high dosage sodium stibogluconate or prolonged treatment with pentamidine.
Ten Kenyan patients with visceral leishmaniasis unresponsive to sodium stibogluconate, at a dose of 16 to 20 mg Sb/kg body-weight/day given for 30 to 98 days, were treated with 20 mg Sb/kg bw given every eight hours. This regimen was modified or abandoned in six patients because of suspected toxicity, although toxicity was difficult to assess because of intercurrent illness. Toxic effects included lethargy, anorexia, vomiting, electrocardiographic changes, fall in haemoglobin and rise in liver enzymes. One patient died, probably from a cardiac arrhythmia. Two patients were cured, four responded partially and four showed no response. Pentamidine, at a dose... (truncated)

Title: Higher pentamidine levels in AIDS patients with hypoglycemia and azotemia during treatment of Pneumocystis carinii pneumonia.
Trimethoprim-sulfamethoxazole (TMP-SMZ) and pentamidine are both licensed for the treatment of Pneumocystis carinii pneumonia (PCP). However, their use is associated with various adverse side effects. In this prospective study, 26 AIDS patients with 32 episodes of PCP were treated with pentamidine (4 mg/kg/d). Each patient was treated for 12 to 21 days, depending on the rapidity of onset of the clinical response. During the 32 PCP episodes, hypoglycemia occurred in 16 instances, azotemia in 12, liver toxicity in 10, and leukopenia in 8. The occurrence of thrombopenia, leukopenia, and liver toxicity was not related to age, pentamidine levels, or other... (truncated)

Title: [Side effects of trimethoprim-sulfamethoxazole in patients with AIDS].
Forty-nine episodes of Pneumocystis carinii pneumonia, treated with trimethoprim-sulfamethoxazole among 46 patients with AIDS were analysed retrospectively for any side effects. For only two were there insufficient data. There were 39 toxic reactions, 15 of them serious: leukopenia (n = 23), abnormal liver functions (n = 14), skin changes (n = 12), gastrointestinal complaints (n = 10) and thrombocytopenia (n = 9). In nine patients the treatment had to be discontinued, in six of them so early that a switch to pentamidine was necessary. Gastrointestinal symptoms occurred as early as the sixth day of treatment, while leukopenia and thrombocytopenia were... (truncated)

MESH:D011223 - praziquantel

Summary:

LLM Prediction Score	0.258 (normalized)
LLM Confidence Score	0.990
Golden Answer (Severity Class)	0.375 (normalized)
Prediction Error	0.117

Retrieved Context:

Title: [Praziquantel in the treatment of Far Eastern hepatic distomiasis caused by Clonorchis sinensis or Opisthorchis viverrini].
Forty-six patients infected with Opistorchis viverrini and/or Clonorchis sinensis, 24 males and 22 females, all Laotian or Vietnamese, aged 7 to 68, weighing 18 to 70 kg, were detected by parasitological stool examinations, numeration of Eggs Per Gram of feces (E.P.G.). In this manner we observed 23 light infestations (1-999 E.P.G.), 20 moderate infestations (10,000-29,999 E.P.G.). Before treatment, all these patients were submitted to a complete history and clinical examination with biological controls that totaled up 47 parameters. These controls were repeated daily from the eve of the treatment to 5 days after. The treatment was given orally at the... (truncated)

Title: A retrospective survey on side effects of praziquantel among 25,693 cases of schistosomiasis japonica.
A retrospective survey involving 25,693 persons was carried out in four provinces and the city of Shanghai, China between December 1981 until May 1982 to document relatively serious side effects of praziquantel used in a mass treatment programme for schistosomiasis japonica. Only 122 or 0.47% of those participating in the study had experienced relatively serious side reactions to the drug. Most had only one kind of side effects but two or more were recorded in a few patients. Neuropsychiatric reactions were seen in 39 persons (0.15%,) cardiovascular reactions in 37 (0.14%), hepatic changes in four (0.02%), dermatological reactions in 18... (truncated)

Title: [Pseudotumor form of hepatic distomatosis: successful treatment with praziquantel].
Human Fasciola hepatica infection is usually discovered either early, during the acute (invasive) phase, or in the advanced phase, which is characterized by biliary complications. We report a case of liver distomatosis with nodular intra-hepatic lesions in a 58-year-old woman. Radiological investigations showed 3 nodular lesions in the VII segment, which were difficult to distinguish from liver metastases or liver abscesses. Distomatosis serology was positive with passive hemagglutination. After a 5-day treatment with praziquantel, clinical symptoms resolved quickly while serological tests became negative. Radiological images slowly decreased to a calcified scar at 13 months. No side-effects were noted. Praziquantel, whose... (truncated)

Title: [Hepatic distomatosis caused by Clonorchis/Opisthorchis spp. in refugees from South-East Asia. Effects of treatment with praziquantel].
The prevalence of liver infection (percentage of patients voiding Clonorchis or Opistorchis spp. eggs in faeces) is 18.5% in the South East Asian refugees. Both sexes are equally affected and the older the refugees are, the more often they are found infected. Laotians, who like to eat the fishes raw, are harboring leverflukes more often than other Far-east inhabitants. Praziquantel, prescribed at a dose of 25 mg/kg body weight given 3 times on a single day, is very efficient in the treatment of the Far-east hepatic distomatosis. Tolerability to praziquantel is excellent. Side effects are few and of moderate intensity.

Title: Ameliorative effects of Schisandrin B on Schistosoma mansoni-induced hepatic fibrosis in vivo.
Schistosomiasis is second only to malaria as the most devastating parasitic disease in the world. It is caused by the helminths Schistosoma mansoni (S. mansoni), S. haematobium, or S. japonicum. Typically, patients with schistosomiasis suffer from symptoms of liver fibrosis and hepatosplenomegaly. Currently, patients were treated with praziquantel. Although praziquantel effectively kills the worm, it cannot prevent re-infection or resolve liver fibrosis. Also, current treatment options are not ample to completely cure liver fibrosis and splenic damages. Moreover, resistance of praziquantel has been reported in vivo and in vitro studies. Therefore, finding new effective treatment agents is urgently needed. Schisandrin... (truncated)

MESH:D017336 - loratadine

Summary:

LLM Prediction Score	0.117 (normalized)
LLM Confidence Score	0.990
Golden Answer (Severity Class)	0.0 (normalized)
Prediction Error	0.117

Retrieved Context:

Title: Important considerations for drugs, nutritional, and herbal supplements in pediatric solid organ transplant recipients.
Pediatric transplant recipients are on multiple prescription and non-prescription drugs. Many patients also use dietary, nutritional, and herbal supplements. This manuscript researched formulations of immunosuppressive drugs currently available and presents information on generic immunosuppressive drugs, commonly used non-prescription medications, dietary supplements, and herbal supplements. Immunosuppressive drugs are available in various formulations. Not all formulations are interchangeable. A number of FDA-approved generic formulations are available commercially in the United States. Generally generic formulations produce similar blood concentration vs time profiles compared to brand name products in adults and are considered to be bioequivalent. NSAID should be avoided in transplant patients due... (truncated)

Title: Safety of Cyproheptadine, an Orexigenic Drug. Analysis of the French National Pharmacovigilance Data-Base and Systematic Review.
Objectives: Cyproheptadine is a first-generation H1-antihistamine drug first that was distributed in the 1960s. While its orexigenic effect was observed early, cyproheptadine is not yet authorized for this indication in all countries today. There is an increasing medical interest and demand for the orexigenic effect of cyproheptadine, especially in children with poor appetite. As cyproheptadine might be evaluated in future clinical trials, we wanted to assess its safety profile. Methods: Using the French national pharmacovigilance database, we retrospectively analyzed all pediatric and adult reports of adverse effects of cyproheptadine recorded since its first distribution in France. Next, we performed a... (truncated)

Title: Cetirizine-induced hepatotoxicity: case series and review of the literature.
Drug-induced liver damage is a frequently encountered clinical table caused by many drugs. Cetirizine is a widely preferred and prescribed antihistaminic agent for allergic disorders due to its non-sedative properties. In view of the literature, we present four cases of hepatotoxicity due to cetirizine use. We conclude that in patients with high levels of liver enzymes of unknown origin, cetirizine as well as other hepatotoxic drugs should be reconsidered.

Title: Palmitate increases the susceptibility of cells to drug-induced toxicity: an in vitro method to identify drugs with potential contraindications in patients with metabolic disease.
Fatty acids are an important source of energy. Excessive energy intake results in elevated levels of free fatty acids that are thought to be the pathogenic factors causing metabolic disorders such as dyslipidemia, obesity, insulin resistance, diabetes, and fatty liver. Underlying metabolic disorders have been suggested to be a predisposing factor for drug-induced liver injury. The steadily expanding population with metabolic disease may pose a higher risk for drug-induced toxicity. In order to understand the interaction of free fatty acids and drug-induced toxicity at the cellular level, we explored whether the saturated free fatty acid palmitate could modulate drug-induced cytotoxicity... (truncated)

Title: H1 antihistamines: current status and future directions.
In this review, we compare and contrast the clinical pharmacology, efficacy, and safety of first-generation H1 antihistamines and second-generation H1 antihistamines. First-generation H1 antihistamines cross the blood-brain barrier, and in usual doses, they potentially cause sedation and impair cognitive function and psychomotor performance. These medications, some of which have been in use for more than 6 decades, have never been optimally investigated. Second-generation H1 antihistamines such as cetirizine, desloratadine, fexofenadine, levocetirizine, and loratadine cross the blood-brain barrier to a significantly smaller extent than their predecessors. The clinical pharmacology, efficacy, and safety of these medications have been extensively studied. They are... (truncated)

MESH:D005438 - fludrocortisone

Summary:

LLM Prediction Score	0.116 (normalized)
LLM Confidence Score	0.950
Golden Answer (Severity Class)	0.0 (normalized)
Prediction Error	0.116

Retrieved Context:

Title: [Addison's disease as a rare cause of chronically elevated liver enzymes].
Common causes of chronically elevated serum liver enzymes include fatty liver disease, chronic viral hepatitis, autoimmune hepatitis, or hereditary metabolic disorders. Adrenocortical insufficiency can also cause elevated liver enzymes. Since 1990 only 11 cases have been reported. We here report a 52-year-old man with elevated liver enzymes (1.5 x upper limit of normal) over the past 10 years. Furthermore, hyponatremia and hyperkalemia were noted. He complained of fatigue and low blood pressure over the past few years. At physical examination a dark complexion was noted. After ruling out chronic viral hepatitis, autoimmune disease, metabolic or hereditary disorders, rare causes of... (truncated)

Title: Adrenal failure followed by status epilepticus and hemolytic anemia in primary antiphospholipid syndrome.
We report on a 14 year old boy who presented with the symptoms abdominal pain, fever and proteinuria. A hematoma in the region of the right pararenal space was diagnosed. Prothrombin time and activated partial thromboplastin time were prolonged, lupus anticoagulant and anticardiolipin antibodies were positive and serum cortisol was normal. Ten days after admission the boy suddenly suffered generalized seizures due to low serum sodium. As well, the patient developed hemolytic anemia, acute elevated liver enzymes, hematuria and increased proteinuria. At this time a second hemorrhage of the left adrenal gland was documented. Adrenal function tests revealed adrenal insufficiency.... (truncated)

Title: Phase 1 Studies to Define the Safety, Tolerability, and Pharmacokinetic and Pharmacodynamic Profiles of the Nonsteroidal Mineralocorticoid Receptor Antagonist Apararenone in Healthy Volunteers.
Apararenone is a long-acting, nonsteroidal mineralocorticoid receptor antagonist (MRA). The safety, tolerability, and pharmacokinetic (PK) and pharmacodynamic (PD) profiles of single- and multiple-dose apararenone were assessed in 3 phase 1 randomized, double-blind studies in 223 healthy adults. Study 1 assessed the PK, safety/tolerability, and PD of single-dose apararenone (3.75-640 mg) and multiple-dose apararenone (10-40 mg/day on days 1-14, 320 mg loading dose on day 1 + 10 mg/day on days 2-14, or 40-320 mg loading dose on day 1 + 2.5-20 mg/day on days 2-14) in Caucasian and Black men and women. Study 2 assessed the PK and safety of... (truncated)

Title: Management of hypoadrenocorticism (Addison's disease) in dogs.
Hypoadrenocorticism (HOAC; Addison's disease) is an endocrine condition seen in small animal practice. Dogs with this disease can present in a variety of ways from acute hypovolemic collapse to vague, chronic, waxing, and waning clinical signs. In the most common form of this disease, animals have both mineralocorticoid and glucocorticoid deficiency, resulting in hyponatremia and hyperkalemia, and signs of cortisol deficiency. The etiology may be immune-mediated destruction of the adrenal cortex, drug-induced adrenocortical necrosis (mitotane), enzyme inhibition (trilostane), or infiltrative processes such as neoplastic or fungal disease. Much less commonly, dogs have signs of cortisol deficiency, but no electrolyte changes.... (truncated)

Title: Association Between NR3C1 Gene Polymorphisms and Toxicity Induced by Glucocorticoids Therapy in Saudi Children with Acute Lymphoblastic Leukemia
Background: Glucocorticoids (GCs) are key hormones used for the treatment of acute lymphoblastic leukemia (ALL) in children, but their cytotoxic effects are not well defined. The aim of this study was to evaluate the association between polymorphisms in NR3C1 encoding for protein involved in the GCs metabolism and its role in the development of ALL and the toxicity outcome, in terms of liver toxicity, glucose abnormality and infections, in ALL Saudi children. Methods: The following polymorphisms BCII rs41423247, ER22/23 EK rs6189 and rs6190 and N363S rs6195 in NR3C1 were analyzed in 70 children with ALL treated according to the ALL... (truncated)

MESH:D008617 - mephenytoin

Summary:

LLM Prediction Score	0.509 (normalized)
LLM Confidence Score	0.950
Golden Answer (Severity Class)	0.625 (normalized)
Prediction Error	0.116

Retrieved Context:

Title: Modeling of Hepatic Drug Metabolism and Responses in CYP2C19 Poor Metabolizer Using Genetically Manipulated Human iPS cells.
Cytochrome P450 family 2 subfamily C member 19 (CYP2C19), in liver, plays important roles in terms of drug metabolism. It is known that CYP2C19 poor metabolizers (PMs) lack CYP2C19 metabolic capacity. Thus, unexpected drug-induced liver injury or decrease of drug efficacy would be caused in CYP2C19 substrate-treated CYP2C19 PMs. However, it is difficult to evaluate the safety and effectiveness of drugs and candidate compounds for CYP2C19 PMs because there is currently no model for this phenotype. Here, using human induced pluripotent stem cells (human iPS cells) and our highly efficient genome-editing and hepatocyte differentiation technologies, we generated CYP2C19-knockout human iPS... (truncated)

Title: Possible association between poor metabolism of mephenytoin and hepatotoxicity caused by Atrium, a fixed combination preparation containing phenobarbital, febarbamate and difebarbamate.
Drug hepatotoxicity is partially determined by genetic factors involved in drug metabolism, which may involve the debrisoquine oxidation polymorphism mediated by cytochrome (CYP) 2D6. The purpose of this study was to assess the relationship between drug hepatotoxicity and another genetic polymorphism of drug oxidation, namely that of S-mephenytoin metabolism mediated by CYP2CMP. Mephenytoin hydroxylation capacity was assessed by a hydroxylation index in 24 patients with drug-induced hepatitis and in 23 healthy controls. Hydroxylation index was calculated as the ratio of S-mephenytoin dose to the (0-10 h) urinary excretion of 4-hydroxymephenytoin after oral administration of 100 mg racemic mephenytoin. The test... (truncated)

Title: The effect of omeprazole pretreatment on acetaminophen metabolism in rapid and slow metabolizers of S-mephenytoin.
Omeprazole, a widely used and potent gastric proton pump inhibitor, induces cytochrome P450 (CYP) 1A2 in humans. Induction is most pronounced in slow metabolizers of S-mephenytoin because CYP2C19 (S-mephenytoin hydroxylase) is responsible for the elimination of omeprazole. Acetaminophen (INN, paracetamol), a widely used and effective analgesic and antipyretic agent, causes serious hepatic and renal toxicity at high doses by conversion of acetaminophen to the toxic intermediate N-acetyl-p-benzoquinone imine (NAPQI) through CYP1A2, CYP2E1, and CYP3A4. This study evaluated whether omeprazole pretreatment in five rapid and five slow metabolizers of S-mephenytoin could increase thioether (an estimate of NAPQI production) metabolite formation from... (truncated)

Title: Safety of anticonvulsants in hepatic porphyrias.
Because acute attacks of porphyria may be precipitated by anticonvulsants, a therapeutic dilemma arises when seizures complicate hepatic porphyria. The list of unsafe agents includes barbiturates, primidone, phenytoin, mephenytoin, ethotoin, ethosuximide, methsuximide, phensuximide, and trimethadione. Agents are considered unsafe if they induce experimental porphyria in animals, and short trials in patients are unreliable for screening. Using drug incubation in chick-embryo hepatocyte culture, we found that porphyrin was increased by carbamazepine, clonazepam, and valproate. These agents should probably be avoided or used with caution in porphyric patients. Alternative approaches to acute porphyric attacks with seizures are discussed.

Title: Drug metabolism and genetic polymorphism in subjects with previous halothane hepatitis.
To test the hypothesis that halothane hepatitis is caused by a combination of altered drug metabolism and an immunoallergic disposition, the metabolism of antipyrine, metronidazole, sparteine, phenytoin, and racemic R- and S-mephenytoin was investigated in seven subjects with previous halothane hepatitis. The HLA tissue types and the complement C3 phenotypes were also determined. The metabolism of antipyrine and metronidazole was within normal range in all subjects, and they were all fast or extensive metabolizers of sparteine, mephenytoin, and phenytoin. HLA tissue types were unremarkable. Five of the seven subjects had complement C3 phenotypes F or FS. In the general

MESH:D000068698 - tenofovir

Summary:

LLM Prediction Score	0.490 (normalized)
LLM Confidence Score	0.990
Golden Answer (Severity Class)	0.375 (normalized)
Prediction Error	0.115

Retrieved Context:

Title: Investigating Organ Toxicity Profile of Tenofovir and Tenofovir Nanoparticle on the Liver and Kidney: Experimental Animal Study.
Tenofovir nanoparticles are novel therapeutic intervention in human immunodeficiency virus (HIV) infection reaching the virus in their sanctuary sites. However, there has been no systemic toxicity testing of this formulation despite global concerns on the safety of nano drugs. Therefore, this study was designed to investigate the toxicity of Tenofovir nanoparticle (NTDF) on the liver and kidney using an animal model. Fifteen adult male Sprague-Dawley (SD) rats maintained at the animal house of the biomedical resources unit of the University of KwaZulu-Natal were weighed and divided into three groups. Control animals (A) were administered with normal saline (NS). The therapeutic... (truncated)

Title: Acute Liver Toxicity due to Efavirenz/Emtricitabine/Tenofovir.
The fixed-dose combination of Efavirenz/Emtricitabine/Tenofovir is a first-line agent for the treatment of HIV; however few cases have reported hepatotoxicity associated with the drug. We report a case of Efavirenz/Emtricitabine/Tenofovir-associated hepatotoxicity presenting mainly with hepatocellular injury characterized by extremely elevated aminotransferase levels, which resolved without acute liver failure or need for liver transplant referral.

Title: Adverse effects of oral antiviral therapy in chronic hepatitis B.
Oral nucleoside/nucleotide analogues (NAs) are currently the backbone of chronic hepatitis B (CHB) infection treatment. They are generally well-tolerated by patients and safe to use. To date, a significant number of patients have been treated with NAs. Safety data has accumulated over the years. The aim of this article is to review and update the adverse effects of oral NAs. NAs can cause class adverse effects (<i>i.e</i>., myopathy, neuropathy, lactic acidosis) and dissimilar adverse effects. All NAs carry a "Black Box" warning because of the potential risk for mitochondrial dysfunction. However, these adverse effects are rarely reported. The majority of... (truncated)

Title: Raltegravir, tenofovir, and emtricitabine in an HIV-infected patient with HCV chronic hepatitis, NNRTI intolerance and protease inhibitors-induced severe liver toxicity.
In HIV-infected patients with HCV-related chronic hepatitis, liver impairment and drug toxicity may substantially reduce the number of possible therapeutic options.

Title: Tenofovir disoproxil fumarate-induced severe liver injury in a patient with chronic hepatitis B virus infection.
No abstract available.

MESH:D015313 - cefotetan

Summary:

LLM Prediction Score	0.261 (normalized)
LLM Confidence Score	0.960
Golden Answer (Severity Class)	0.375 (normalized)
Prediction Error	0.114

Retrieved Context:

Title: [Evaluation of cefotetan in obstetrics and gynecology].
Fundamental and clinical studies on gynecological use of cefotetan (CTT), a new cephamycin antibiotic, were performed with following results. Following the intravenous administration of 2.0 g of CTT, T 1/2 beta in serum was 3.1 hours and longer than the previous cephamycin antibiotics. The yields of CTT from serum to various uterine tissues and discharge from retroperitoneum were about 30 approximately 50%. In clinical use, 14 patients with gynecological infections were administrated CTT, and it showed excellent or good efficacy in all patients. No side effects were noted except 1 transient disturbance in liver function.

Title: Clinical pharmacokinetics of the third generation cephalosporins.
At the present time, the third generation cephalosporins that are already on the market or close to this point include cefsulodin, cefotaxime, cefoperazone, latamoxef, ceftriaxone, ceftazidime, ceftizoxime and cefotetan. Other newer compounds are also under development but have not been included in this review. None of the third generation compounds is suitable for oral administration and, accordingly, their pharmacokinetics have been studied only after intravenous and intramuscular administration. Microbiological assays and HPLC methods have been used for the measurement of plasma/serum, urine, bile and cerebrospinal fluid (CSF) concentrations. As found with cefotaxime, microbiological assays should only be used when the... (truncated)

Title: Autoimmune hemolytic anemia induced by levofloxacin.
Drug-induced autoimmune hemolytic anemia is a rare condition. We report the case of a 32-year-old white female who presented to the emergency department with generalized fatigue, fever, and jaundice. The patient reported using levofloxacin few days prior to presentation for urinary tract infection. The patient had evidence of hemolytic anemia with a hemoglobin of 6.7 g/dL which dropped to 5 g/dL on day 2, the direct Coombs test was positive, indirect bilirubin was 5.5 mg/dL, and LDH was 1283 IU/L. Further testing ruled out autoimmune disease, lymphoma, and leukemia as etiologies for the patient's hemolytic anemia. Levofloxacin was immediately stopped... (truncated)

Title: Second-Generation Cephalosporins-Associated Drug-Induced Liver Disease: A Study in VigiBase with a Focus on the Elderly.
The objective of this study was to characterize individual case safety reports (ICSRs) and adverse drug reactions (ADRs) related to second-generation cephalosporins and resulting in hepatobiliary disorders, in VigiBase, WHO global database.

Title: A Repeat Case of Cefotetan-Induced Hemolytic Anemia in a Surgical Patient.
Drug-induced hemolytic anemia is a rare hematologic condition in which the immune system creates antibodies against red blood cell antigens in response to a medication exposure. This condition is commonly triggered by antibiotics, nonsteroidal anti-inflammatory drugs, and certain chemotherapies. Here, we describe a patient who experienced a repeat episode of drug-induced hemolytic anemia related to prophylactic cefotetan given before surgery. This case is important for increasing awareness of cephalosporin-induced hemolytic anemia, illustrating the significance of early detection and treatment of the condition, and highlighting the need for careful review of medication history in the surgical setting.

MESH:D000077150 - oxaliplatin

Summary:

LLM Prediction Score	0.613 (normalized)
LLM Confidence Score	0.990
Golden Answer (Severity Class)	0.5 (normalized)
Prediction Error	0.113

Retrieved Context:

Title: Oxaliplatin-induced hepatocellular injury and ototoxicity: a review of the literature and report of unusual side effects of a commonly used chemotherapeutic agent.
After extensive literature review utilizing PubMed and Medline searches, we present a rare case of oxaliplatin-induced grade 3/4 hepatocellular injury and ototoxicity. The patient is a 46-year-old female diagnosed with stage IIIC (pT3N2bM0) adenocarcinoma of the sigmoid colon. PET/CT prior to surgery and chemotherapy was negative for distant metastatic disease and baseline liver-associated enzymes were within normal limits. Following sigmoidectomy, patient began adjuvant chemotherapy with 5-florouracil, leucovorin, and oxaliplatin (mFOLFOX-6). Cycle 1 was complicated only by refractory nausea. However, cycle 2 was complicated by vertigo with refractory nausea, tinnitus, and marked elevation in liver enzymes in a hepatocellular pattern. Extensive... (truncated)

Title: Curcumin Attenuates Oxaliplatin-Induced Liver Injury and Oxidative Stress by Activating the Nrf2 Pathway.
Oxaliplatin (OXA)-induced liver injury is one of the main limiting factors affecting the efficacy of OXA-based chemotherapy in patients with colorectal liver metastases. In addition, oxidative stress is an important pathophysiological mechanism of OXA-induced liver injury. Therefore, dietary antioxidants may decrease or prevent hepatic toxicity in vivo and be beneficial to OXA-based chemotherapy.

Title: The Clinical Spectrum and Diagnosis of Oxaliplatin Liver Injury in the Era of Nonalcoholic Fatty Liver Disease.
Oxaliplatin is an alkylating agent given with fluorouracil and leucovorin as a mainstay adjuvant chemotherapy for stage III colorectal cancer (CRC). Liver injury from oxaliplatin ranges from mild liver enzyme increases in 42% to 57% of patients in clinical trials¹ to rare severe injury leading to acute liver failure.² Chronic injury from endothelial cell damage and architectural distortion may manifest years later with nodular regenerative hyperplasia (NRH), portal sclerosis, and noncirrhotic portal hypertension (NCPH).²[,]³ Chronic subclinical injury occurs in up to 78% of patients.³ Diagnosis may be confounded by nonalcoholic fatty liver disease (NAFLD), and long-term outcomes from chronic injury... (truncated)

Title: Non-Cirrhotic Portal Hypertension in a Patient With Colonic Carcinoma Treated With Oxaliplatin.
Oxaliplatin is a chemotherapeutic agent with direct toxic action on deoxyribonucleic acid (DNA), which is known to cause an arrest in its synthesis and inducing cell death. It is a crucial medication for colorectal carcinoma, and in combination with other medications has demonstrated to exhibit synergism, managing to increase patients' survival, especially when compared to monotherapy with 5-fluoracil. Neurotoxicity is its most well-known adverse effect. However, other less frequent secondary effects have been described in case reports, among them liver injury, which is usually secondary to liver sinusoid injury. Despite the wide frequency of the use of this drug, the... (truncated)

Title: Identification of molecular pathways involved in oxaliplatin-associated sinusoidal dilatation.
Oxaliplatin-based chemotherapy for colorectal liver metastases (CRLM) can result in vascular liver lesions such as sinusoidal dilatations. Physiopathology remains unclear and variability between patients suggests that there is individual susceptibility. A better understanding of the molecular mechanisms of oxaliplatin liver toxicity may allow the identification of biomarkers and adaptation of chemotherapy delivery.

MESH:D000069444 - omalizumab

Summary:

LLM Prediction Score	0.113 (normalized)
LLM Confidence Score	0.970
Golden Answer (Severity Class)	0.0 (normalized)
Prediction Error	0.113

Retrieved Context:

Title: Treatment of Indolent and Advanced Systemic Mastocytosis.
Management of Indolent and Smoldering SM is focused on preventing anaphylactic reactions and identifying and avoiding symptom triggers. Skin and gastrointestinal symptoms are managed with H1- and H2-antihistamines. When skin symptoms are not adequately controlled, leukotriene antagonists and oral psoralen combined with ultraviolet therapy may be added. Proton pump inhibitors, sodium cromolyn, and oral corticosteroids may be added for gastrointestinal symptoms. Patients should be prescribed self-injectable epinephrine and trained to treat recurrent cardiovascular symptoms or anaphylaxis. Depression and cognitive impairment require a psychiatric evaluation for tailored treatment. Bone involvement is managed with bisphosphonates and eventually interferon. Omalizumab is effective on... (truncated)

Title: Total antioxidant capacity, hydrogen peroxide, malondialdehyde and total nitric oxide concentrations in patients with severe persistent allergic asthma: its relation to omalizumab treatment.
Asthma is a chronic inflammatory disease and omalizumab is indicated for moderate-to-severe persistent asthma. The results of many studies have shown that oxidative stress is involved in asthma pathogenesis. However, there is no data available to evaluate the alterations in total antioxidant capacity, hydrogen peroxide, malondialdehyde, and total nitric oxide concentrations.

Title: Cytomegalic hepatitis in a patient receiving omalizumab.
Cytomegalovirus is a double stranded DNA virus that can be present in nearly all organs and body fluids. The primary infection is usually asymptomatic in the immunocompetent host and it is common among adolescents and young adults. The symptomatic form appears, in the majority of cases, as a mononucleosis syndrome with full recovery without specific treatment. We report a case of a 25 years old woman who presented with hepatitis due to CMV infection and history of omalizumab administration one month earlier. This recombinant monoclonal antibody is used to control refractory asthma and chronic spontaneous urticarial as it inhibits human... (truncated)

Title: Risks and safety of biologics: A practical guide for allergists.
Biologic agents are a rapidly expanding class of medications, and several options are now available for the management of allergic and immunologic disorders. The risks of biologic therapy need to be understood in order to adequately counsel patients and appropriately monitor for potential adverse events. We sought to provide a comprehensive review of the risks and adverse effects reported for the current FDA-approved biologics used in management of allergic and immunologic disorders, including omalizumab, benralizumab, dupilumab, mepolizumab, reslizumab, tezepelumab and tralokinumab. Our review focuses on the risk of hypersensitivity reactions, pregnancy-specific considerations, risk of infection and risk of malignancy. We... (truncated)

Title: Levofloxacin Induced Toxic Epidermal Necrolysis: Successful Therapy with Omalizumab (Anti-IgE) and Pulse Prednisolone.
BACKGROUND Toxic epidermal necrolysis (TEN) is characterized by widespread erythematous and bullous lesions on the skin. Nowadays, considerable progress has been made in the understanding of its pathogenesis. Immunologically it is similar to graft-versus-host disease. Therefore, we may propose that TEN is a disorder of cell-mediated immunity. CASE REPORT Our patient was a 74-year-old white female who had pneumonia and was positive for hepatitis C virus (HCV), and who had been on levofloxacin therapy. After the first levofloxacin dose, erythematous dusky red macules occurred on her extremities and trunk, and on the following day, confluent purpuric lesions tended to run... (truncated)

MESH:D005473 - fluoxetine

Summary:

LLM Prediction Score	0.488 (normalized)
LLM Confidence Score	0.990
Golden Answer (Severity Class)	0.375 (normalized)
Prediction Error	0.113

Retrieved Context:

Title: Liver injury associated with antidepressants.
Antidepressants are commonly prescribed and used in the management of depression, anxiety disorders, and other psychiatric illnesses. Antidepressants used in therapeutic dosing ranges are associated with causing several adverse drug reactions including hepatotoxicity. Paroxetine, fluoxetine, fluvoxamine, citalopram, mirtazapine and venlafaxine are associated with reversible liver injury upon discontinuation of the agent. Patient cases of hepatotoxicity involving the use of nefazodone, trazodone, duloxetine, bupropion, and sertraline are linked to causing death in its users. Due to the idiosyncratic nature of hepatotoxicity, monitoring of liver function tests and immediate discontinuation upon abnormal lab findings or signs and symptoms of liver dysfunction are... (truncated)

Title: Reaction of the Liver upon Long-Term Treatment of Fluoxetine and Atorvastatin Compared with Alcohol in a Mouse Model.
Alcoholism is known to cause liver toxicity and is extensively researched. On the other hand, stress, depression, and obesity are interrelated conditions with alcoholism, and their medications would affect the liver itself. In this study, we investigated the effects of the drugs fluoxetine and atorvastatin on the liver and compared with those of alcohol in a mouse model.

Title: The role of NLRP3 inflammasome in psychotropic drug-induced hepatotoxicity.
Increased medical application of psychotropic drugs raised attention concerning their toxicological effects. In fact, more than 160 psychotropic drugs including antidepressants and antipsychotics, have been shown to cause liver side effects, but the underlying mechanisms are still poorly understood. Here, we discovered that fluoxetine, a common antidepressant, was specifically sensed by NLRP3 inflammasome, whose subsequent activation resulted in the maturation of caspase-1 and IL-1β, as well as gasdermin D (GSDMD) cleavage, which could be completely abrogated by a selective NLRP3 inhibitor MCC950 or Nlrp3 knockout (Nlrp3^{-/-}). Mechanistically, mitochondrial damage and the subsequent mitochondrial reactive oxygen species (mtROS) accumulation were crucial... (truncated)

Title: Acute hepatitis due to fluoxetine therapy.
Fluoxetine-induced hepatotoxicity is generally considered of minimal clinical importance and is not well recognized. Asymptomatic increases in liver enzyme values have been observed in 0.5% of patients who take long-term fluoxetine therapy. This report details 2 cases of acute hepatitis believed to be caused by fluoxetine. Three cases of acute hepatitis caused by fluoxetine have been reported previously. The mechanism of fluoxetine-induced hepatotoxicity is unknown. Although routine monitoring of liver function may not be cost-effective, physicians should be alert to the possibility of fluoxetine-associated hepatitis and consider early discontinuation of the drug if this condition is suspected.

Title: Baicalin provides protection against fluoxetine-induced hepatotoxicity by modulation of oxidative stress and inflammation.
Label="BACKGROUND" NlmCategory="BACKGROUND">Fluoxetine is one of the most widely prescribed anti-depressant drugs belonging to the category of selective serotonin reuptake inhibitors. Long-term fluoxetine treatment results in hepatotoxicity. Baicalin, a natural compound obtained from the Chinese herb <i>Scutellaria baicalensis</i> is known to have antioxidant, hepatoprotective and anti-inflammatory effects. However, the beneficial effects of baicalin against fluoxetine-induced hepatic damage have not previously been reported.

MESH:D007208 - indocyanine green

Summary:

LLM Prediction Score	0.110 (normalized)
LLM Confidence Score	0.990
Golden Answer (Severity Class)	0.0 (normalized)
Prediction Error	0.110

Retrieved Context:

Title: Indocyanine green clearance varies as a function of N-acetylcysteine treatment in a murine model of acetaminophen toxicity.
Standard assays to assess acetaminophen (APAP) toxicity in animal models include determination of ALT (alanine aminotransferase) levels and examination of histopathology of liver sections. However, these assays do not reflect the functional capacity of the injured liver. To examine a functional marker of liver injury, the pharmacokinetics of indocyanine green (ICG) were examined in mice treated with APAP, saline, or APAP followed by N-acetylcysteine (NAC) treatment.Male B6C3F1 mice were administered APAP (200 mg/kg IP) or saline. Two additional groups of mice received APAP followed by NAC at 1 or 4 h after APAP. At 24 h, mice were injected with... (truncated)

Title: A novel liver-specific fluorescent anti-cancer drug delivery system using indocyanine green.
Indocyanine green (ICG) accumulates only in hepatocytes and their malignant counterpart, hepatocellular carcinoma (HCC). We have developed ICG-conjugated anti-cancer drugs and noted their significant accumulation in HCC cells both in vitro and in vivo. ICG-conjugated gemcitabine was less toxic to normal cells and it had superior anti-tumor action compared to gemcitabine alone in a subcutaneous tumor xenograft. ICG conjugation can provide a novel fluorescent drug delivery system for treatment of liver cancer and this system can be used to both diagnose and treat HCC.

Title: Indocyanine green liver function studies on women taking progestins.
The liver extraction of indocyanine green in concentrations of 0.2 mg./kg. and 0.5 mg./kg. in women taking oral contraceptive (norethindrone 2 mg. with mestranol 0.1 mg., and norethindrone 1 mg. with mestranol 0.05 mg.) is reported. No major alteration was noted in the hepatic function with the plasma concentrations of the dye employed. The probability of increased hepatic arterial flow or increased cardiac output due to norethindrone-mestranol combination is noted. The value of indocyanine green in the study of liver dysfunction is stated and its relative sensitivity at various concentrations is mentioned. The use of indocyanine green to predict early... (truncated)

Title: Indocyanine green clearance test as a predictor of chemotherapy liver toxicity and post-operative complications in patients with colorectal liver metastases.
This study aims to investigate the relationship between the pre-operative indocyanine green (ICG) test, the chemotherapy-associated liver injury (CALI), and the development of severe post-operative complications (POC) in patients operated of colorectal liver metastases (CRLMs).

Title: The stimulant effect of drugs on indocyanine green clearance by the liver.
The rate of removal of a standard dose of 25 mg indocyanine green was studied in 67 patients at the bedside using dichromatic ear densitometry. The determination of the percentage disappearance rate per minute and the half life of the dye permitted separation of patients into three groups: those with normal liver function, those with liver damage, and a group taking opiates (9), anticonvulsants (8), phenylbutazone (2), haloperidol (1), and nitrofurantoin (1). The last group showed enhanced clearance of indocyanine green from the circulation.

MESH:C063449 - zileuton

Summary:

LLM Prediction Score	0.515 (normalized)
LLM Confidence Score	0.920
Golden Answer (Severity Class)	0.625 (normalized)
Prediction Error	0.110

Retrieved Context:

Title: The use of 5-lipoxygenase inhibitors and leukotriene receptor antagonists in the treatment of chronic asthma.
With the elucidation of asthma as a chronic inflammatory disease, therapeutic approaches have shifted from treatment of symptoms with bronchodilators to treatment of the underlying disease with antiinflammatory agents. Along with concerns about corticosteroid side effects on the part of both physicians and patients, this shift has motivated researchers to develop and test new agents with antiinflammatory capabilities. The leukotrienes are endogenous mediators with three inflammatory effects: they increase vascular permeability, recruit other inflammatory leukocytes, and induce bronchoconstriction. A number of antileukotriene agents are in various stages of development. Zileuton, a leukotriene synthesis inhibitor, has been shown to improve airway... (truncated)

Title: Clinical pattern of zileuton-associated liver injury: results of a 12-month study in patients with chronic asthma.
Zileuton is a 5-lipoxygenase inhibitor approved by the US FDA in 1996 for the treatment of asthma in adults and children. During phase II/III clinical trials, zileuton was generally well tolerated, although elevations in ALT and AST levels were noted in some patients, and a single treated patient developed hepatocellular jaundice. To more fully characterise the hepatic effects of zileuton, and to establish appropriate monitoring guidelines, a 12-month open-label, safety surveillance study was conducted prior to FDA approval.

Title: Cellular imaging predictions of clinical drug-induced liver injury.
Drug-induced liver injury (DILI) is the most common adverse event causing drug nonapprovals and drug withdrawals. Using drugs as test agents and measuring a panel of cellular phenotypes that are directly linked to key mechanisms of hepatotoxicity, we have developed an in vitro testing strategy that is predictive of many clinical outcomes of DILI. Mitochondrial damage, oxidative stress, and intracellular glutathione, all measured by high content cellular imaging in primary human hepatocyte cultures, are the three most important features contributing to the hepatotoxicity prediction. When applied to over 300 drugs and chemicals including many that caused rare and idiosyncratic liver... (truncated)

Title: In vitro metabolism of 2-acetylbenzothiophene: relevance to zileuton hepatotoxicity.
Zileuton, an inhibitor of 5-lipooxygenase, the initial enzyme in the leukotriene pathway, was marketed as a new treatment for asthma. This drug has been associated with liver toxicity, which has limited its clinical usefulness. We provide evidence here that the liver toxicity likely involves a sequence of biotransformations leading to 2-acetylbenzothiophene (2-ABT), which is subsequently metabolized to give a reactive intermediate(s). In vitro experiments with the human lymphoblast MCL5 cell line demonstrated that 2-ABT is cytotoxic in a P450-dependent manner. Human liver microsome (HLM) incubations with 2-ABT revealed the formation of two short-lived oxidized species, "M + 16" and "M... (truncated)

Title: Nitrosative Stress and Lipid Homeostasis as a Mechanism for Zileuton Hepatotoxicity and Resistance in Genetically Sensitive Mice.
Zileuton is an orally active inhibitor of leukotriene synthesis for maintenance treatment of asthma, for which clinical usage has been associated with idiosyncratic liver injury. Mechanistic understanding of zileuton toxicity is hampered by the rarity of the cases and lack of an animal model. A promising model for mechanistic study of rare liver injury is the Diversity Outbred (J:DO) mouse population, with genetic variation similar to that found in humans. In this study, female DO mice were administered zileuton or vehicle daily for 7 days (i.g.). Serum liver enzymes were elevated in the zileuton group, with marked interindividual variability in... (truncated)

MESH:C020375 - isaxonine

Summary:

LLM Prediction Score	0.890 (normalized)
LLM Confidence Score	0.420
Golden Answer (Severity Class)	1.0 (normalized)
Prediction Error	0.110

Retrieved Context:

Title: Acute and chronic drug-induced hepatitis.
Adverse drug reactions may mimic almost any kind of liver disease. Acute hepatitis is often due to the formation of reactive metabolites in the liver. Despite several protective mechanisms (epoxide hydrolases, conjugation with glutathione), this formation may lead to predictable toxic hepatitis after hugh overdoses (e.g. paracetamol), or to idiosyncratic toxic hepatitis after therapeutic doses (e.g. isoniazid). Both genetic factors (e.g. constitutive levels of cytochrome P-450 isoenzymes, or defects in protective mechanisms) and acquired factors (e.g. malnutrition, or chronic intake of alcohol or other microsomal enzyme inducers) may explain the unique susceptibility of some patients. Formation of chemically reactive metabolites... (truncated)

Title: [Hepatotoxicity of isaxonine phosphate: 4 cases of severe subacute hepatitis].
Isaxonine phosphate is known to induce acute hepatitis which in most cases is reversible after withdrawal of the drug. The authors describe 4 new cases of hepatitis which differ from those previously reported by their evolutive and pathological features. In 3 cases, the outcome was fatal within a delay of 15-40 days despite discontinuation of the drug, and was associated with hepatic encephalopathy and ascites. In the 4 cases plasma concentration of alanine- and aspartate-aminotransferases were initially increased (up to 33 X upper normal range) and decreased to normal values in 2 cases. Plasma bilirubin levels were also elevated at... (truncated)

Title: Drug-induced hepatitis associated with anticytoplasmic organelle autoantibodies.
A study from five hepatology units documenting 157 cases of drug-induced hepatitis and a second study from a laboratory of immunology which tested more than 100,000 sera permitted us to establish the frequency of antiorganelle antibodies and their diagnostic value in drug-induced hepatitis. In drug-induced hepatitis caused by a heterogenous group of drugs consisting of ajmaline, aminopterine, isaxonine, isoniazid, perhexiline, phenylbutazone and troleandromycine, antiorganelle antibodies were absent or rare. In drug-induced hepatitis caused by another heterogenous group of drugs, including clometacin, fenofibrate, oxyphenisatin and papaverine, antismooth muscle, antinucleus and antimitochondria antibodies were found in isolation or in different combinations in... (truncated)

Title: Mechanism for isaxonine hepatitis. I. Metabolic activation by mouse and human cytochrome P-450.
The metabolism of isaxonine was first investigated in mice. Incubation, under air, of [2-14C] isaxonine (1 mM) with mouse liver microsomes and an NADPH-generating system resulted in the irreversible binding of a [14C] isaxonine metabolite to microsomal proteins; binding required active microsomes, NADPH and oxygen, it was inhibited by 4 mM piperonyl butoxide or by a CO-O2 (80:20) atmosphere. In the presence of various concentrations of isaxonine (0.125-2 mM), binding followed Michaelis-Menten kinetics; the Vmax was increased by both phenobarbital and 3-methylcholanthrene pretreatments. In vivo, 2.5 hr after the administration of [2-14C] isaxonine (4 mmol X kg-1 i.p.), a [14C]... (truncated)

Title: [Acute hepatitis caused by isaxonine phosphate (Nerfactor)].
We report the cases of four adult patients suffering from acute hepatitis due to isaxonine phosphate (Nerfactor), a drug recently proposed for the treatment of the lesions of peripheral nerves. Hepatitis developed 14 to 166 days after the beginning of the administration of the drug. In all the patients, predominantly centrilobular hepatocytic necrosis was present. In two of our patients, the course of hepatitis was fatal. Hepatitis induced by isaxonine phosphate is likely to be due to an immuno-allergic mechanism.

MESH:D005437 - flucytosine

Summary:

LLM Prediction Score	0.482 (normalized)
LLM Confidence Score	0.980
Golden Answer (Severity Class)	0.375 (normalized)
Prediction Error	0.107

Retrieved Context:

Title: Evolving role of flucytosine in immunocompromised patients: new insights into safety, pharmacokinetics, and antifungal therapy.
Flucytosine is an antifungal agent useful in combination with amphotericin B in the treatment of several deeply invasive mycoses. The potentially dose-limiting, hematologic, gastrointestinal, and hepatic toxicities of flucytosine lead to a reluctance to use it in myelosuppressed patients. To investigate the safety and tolerability of flucytosine in this setting, we evaluated its use in 17 patients with cancer or aplastic anemia during a 2 1/2-year period at our institution and reviewed the literature describing mechanisms of action, resistance, in vitro and in vivo antifungal activity, clinical antifungal activity, pharmacokinetics, and toxicity. The combination of amphotericin B plus flucytosine eradicated... (truncated)

Title: Toxicity of amphotericin B plus flucytosine in 194 patients with cryptococcal meningitis.
A multicenter prospective randomized trial of four versus six weeks of amphotericin B, 0.3 mg/kg per day, plus flucytosine, 150 mg/kg per day, was performed with 194 patients with cryptococcal meningitis. One or more toxic drug reactions developed in 103 patients: azotemia (51), renal tubular acidosis (two), leukopenia (30), thrombocytopenia (22), diarrhea (26), nausea/vomiting (10), and hepatitis (13). The four- and six-week regimens were complicated by toxicity in 44 percent and 43 percent of cases, respectively. Toxicity appeared during the first two weeks of therapy in 56 percent and during the first four weeks in 87 percent. Azotemia did not... (truncated)

Title: Evaluation of Hepatotoxicity with Treatment Doses of Flucytosine and Amphotericin B for Invasive Fungal Infections.
Invasive fungal infection is a well-known cause of morbidity and mortality in immunocompromised patients. In this study we aimed to evaluate the hepatotoxicity induced by combined therapy of flucytosine and amphotericin B, at three different doses administered to mice for 14 days: 50 mg/kg flucytosine and 300 μg/kg amphotericin B; 100 mg/kg flucytosine and 600 μg/kg amphotericin B; 150 mg/kg flucytosine and 900 μg/kg amphotericin B. Liver injuries were evaluated by analysis of optic and electron microscopy samples, changes in TNF-α, IL-6, and NF-κB inflammation markers levels of expression, and evaluation of mRNA profiles. Histological and ultrastructural analysis revealed an... (truncated)

Title: Association therapy as a prognostic factor in deep fungal infection complicating oncohaematological diseases.
A group of 31 oncohaemopathic patients (17 male, mean age 44 +/- 6 years), diagnosed as having primary deep fungal infection involving the lungs, were retrospectively evaluated. When infection was suspected on a clinical basis the major associated risks for death were the duration of bone marrow aplasia (12 +/- 7 versus 21 +/- 6 days, $P < 0.001$), increase in white blood cells and, in particular, prolonged granulocytopenia (11 +/- 5 versus 24 +/- 8 days, $P < 0.001$) when survivors were compared with patients, who died. Our therapeutic empirical approach was based on the association of i.v. amphotericin... (truncated)

Title: Synthesis, Molecular Docking, and Preclinical Evaluation of a New Succinimide Derivative for Cardioprotective, Hepatoprotective and Lipid-Lowering Effects.
Cardiac and hepatotoxicities are major concerns in the development of new drugs. Better alternatives to other treatments are being sought to protect these vital organs from the toxicities of these pharmaceuticals. In this regard, a preclinical study is designed to investigate the histopathological effects of a new succinimide derivative (Comp-1) on myocardial and liver tissues, and the biochemical effects on selected cardiac biomarkers, hepatic enzymes, and lipid profiles. For this, an initially lethal/toxic dose was determined, followed by a grouping of selected albino rats into five groups (each group had n = 6). The control group received daily oral saline... (truncated)

MESH:D000077239 - meloxicam

Summary:

LLM Prediction Score	0.482 (normalized)
LLM Confidence Score	0.990
Golden Answer (Severity Class)	0.375 (normalized)
Prediction Error	0.107

Retrieved Context:

Title: Meloxicam: selective COX-2 inhibition in clinical practice.
Nonsteroidal antiinflammatory drugs (NSAIDs) exert their actions by inhibiting cyclooxygenase (COX). It has recently been postulated that NSAIDs' antiinflammatory efficacy arises from inhibition of the COX-2 isoform of cyclooxygenase, whereas inhibition of the COX-1 isoform produces the troublesome and sometimes serious gastric and renal side effects of NSAIDs. A relatively selective COX-2 inhibitor, such as meloxicam, may combine antiinflammatory efficacy with improved tolerability. In volunteers, indomethacin 75 mg, but not meloxicam 7.5 mg, inhibited renal prostaglandin E2 excretion and platelet aggregation (COX-1 mediated effects). Double-blind, randomized trials in osteoarthritis and rheumatoid arthritis patients have shown equivalent antiinflammatory efficacy among meloxicam... (truncated)

Title: Meloxicam-induced liver toxicity.
We report the case of a female patient with rheumatoid arthritis who developed acute cytolytic hepatitis due to meloxicam. Recently introduced in Belgium, meloxicam is the first nonsteroidal antiinflammatory drug with selective action on the inducible form of cyclooxygenase 2. The acute cytolytic hepatitis occurred rapidly after meloxicam administration and was associated with the development of antinuclear antibodies suggesting a hypersensitivity mechanism. This first case of meloxicam related liver toxicity demonstrates the potential of this drug to induce hepatic damage.

Title: Incidence of spontaneous notifications of adverse reactions with aceclofenac, meloxicam, and rofecoxib during the first year after marketing in the United Kingdom.
The objective was to compare the incidence of adverse reactions reported with three nonsteroidal anti-inflammatory drugs with different cyclo-oxygenase (COX)-2 selectivity. All spontaneous adverse reaction notifications in the pharmacovigilance database of the World Health Organisation Collaborating Centre for International Drug Monitoring with aceclofenac, meloxicam, and rofecoxib that were recorded during the first year of marketing were included. The incidence rate (adverse reactions/10(6) defined daily dose) and 95% confidence interval for total adverse reactions was 8.7 (6.1-12.0) for aceclofenac, 24.8 (23.1-26.6) for meloxicam, and 52.6 (49.9-55.4) for rofecoxib. Aceclofenac had a lower incidence of gastrointestinal bleeding, abdominal pain, and arterial hypertension... (truncated)

Title: Nonsteroidal anti-inflammatory drug-induced liver injury: a case-control study in primary care.
Several nonsteroidal anti-inflammatory drugs (NSAIDs) have been withdrawn from the market because of hepatic adverse drug reactions (ADRs). Moreover, some cases of liver diseases have been reported in patients taking NSAIDs (arylcarboxylic NSAIDs, piroxicam, sulindac, nimesulide, etc.). Pharmacoepidemiological studies have shown a risk of hepatic ADRs with NSAIDs used in association with other hepatotoxic drugs. In contrast, other studies performed in hospitalized patients did not found any association. The aim of this study was to assess the hepatic risk associated with the use of NSAID in the setting of primary care. The study design was a case-control study where cases... (truncated)

Title: Meloxicam methyl group determines enzyme specificity for thiazole bioactivation compared to sudoxicam.
Meloxicam is a thiazole-containing NSAID that was approved for marketing with favorable clinical outcomes despite being structurally similar to the hepatotoxic sudoxicam. Introduction of a single methyl group on the thiazole results in an overall lower toxic risk, yet the group's impact on P450 isozyme bioactivation is unclear. Through analytical methods, we used inhibitor phenotyping and recombinant P450s to identify contributing P450s, and then measured steady-state kinetics for bioactivation of sudoxicam and meloxicam by the recombinant P450s to determine relative efficiencies. Experiments showed that CYP2C8, 2C19, and 3A4 catalyze sudoxicam bioactivation, and CYP1A2 catalyzes meloxicam bioactivation, indicating that the methyl... (truncated)

MESH:D064748 - dexlansoprazole

Summary:

LLM Prediction Score	0.393 (normalized)
LLM Confidence Score	0.960
Golden Answer (Severity Class)	0.5 (normalized)
Prediction Error	0.107

Retrieved Context:

Title: The Importance of Patient-Specific Factors for Hepatic Drug Response and Toxicity.
Responses to drugs and pharmacological treatments differ considerably between individuals. Importantly, only 50%-75% of patients have been shown to react adequately to pharmacological interventions, whereas the others experience either a lack of efficacy or suffer from adverse events. The liver is of central importance in the metabolism of most drugs. Because of this exposed status, hepatotoxicity is amongst the most common adverse drug reactions and hepatic liabilities are the most prevalent reason for the termination of development programs of novel drug candidates. In recent years, more and more factors were unveiled that shape hepatic drug responses and thus underlie the... (truncated)

Title: Hepatotoxicity-Related Adverse Effects of Proton Pump Inhibitors: A Cross-Sectional Study of Signal Mining and Analysis of the FDA Adverse Event Report System Database.
Background and Objectives: Mounting evidence demonstrates that proton pump inhibitors (PPIs) are associated with a number of adverse effects. However, the literatures about hepatotoxicity-related adverse effects (HRAEs) of PPIs are mostly case reports and a few clinical studies. Methods: We evaluated the association between PPIs and HAREs using the reporting odd ratio (ROR) for mining the adverse event report signals in the FDA Adverse Event Reporting System (FAERS) database. Results: There were 23,825 reports of PPIs as primary suspect drug or second suspect drug, of which 3,253 reports were HRAEs. The top five HRAE signals caused by PPIs were hepatitis... (truncated)

Title: The safety of drugs used in acid-related disorders and functional gastrointestinal disorders.
Medicines are frequently used in the management of acid-related disorders and functional gastrointestinal disorders. With the exception of complicated peptic ulcer disease, these disorders are not associated with appreciable mortality. Drug treatments have consequently been held to the highest standards of safety. Some medicines have been withdrawn or restricted based on assessments and perceptions of risk. However, the risk of serious toxicity is low for most of the agents discussed in this article. Assessments are made of the safety and adverse-event profiles of certain drug classes and, where appropriate, individual medicines. For conditions with a low risk of mortality or... (truncated)

Title: Association of direct oral anticoagulant-proton pump inhibitor cotherapy with adverse outcomes: protocol for a population-based cohort study.
Proton pump inhibitors (PPIs) are widely used for primary and secondary prevention of upper gastrointestinal bleeding. However, there remains controversy about the overall net clinical benefit of PPIs (omeprazole, rabeprazole, pantoprazole, lansoprazole) when coprescribed with direct oral anticoagulants (DOACs; dabigatran, rivaroxaban, apixaban, edoxaban). Our objective is to explore the risk of clinically relevant events, including bleeding, thromboembolic events and death, in patients prescribed DOACs while taking PPIs versus no PPI.

Title: Risk of Enteric Infection in Patients with Gastric Acid Supressive Drugs: A Population-Based Case-Control Study.
Long-term use of gastric-acid-suppressive drugs is known to be associated with several adverse effects. However, the association between enteric infection and acid suppression therapy is still uncertain. This study aimed to evaluate the association between gastric acid suppression and the risk of enteric infection. Materials and Methods: We conducted a population-based case-control study using the data from Chang Gung Research Database (CGRD) in Taiwan. Between January 2008 and December 2017, a total of 154,590 adult inpatients (age > 18) were identified. A pool of potential eligible controls according to four propensity scores matching by sex, age, and index year were... (truncated)

MESH:D009355 - neomycin

Summary:

LLM Prediction Score	0.107 (normalized)
LLM Confidence Score	0.990
Golden Answer (Severity Class)	0.0 (normalized)
Prediction Error	0.107

Retrieved Context:

Title: Adverse effects of hypolipidaemic drugs.
Cholestyramine, colestipol, clofibrate, gemfibrozil, nicotinic acid (niacin), probucol, neomycin, and dextrothyroxine are the most commonly used drugs in the treatment of hyperlipoproteinaemic disorders. While adverse reaction data are available for all of them, definitive data regarding the frequency and severity of potential adverse effects from well-controlled trials using large numbers of patients (greater than 1000) are available only for cholestyramine, clofibrate, nicotinic acid and dextrothyroxine. In adult patients treated with cholestyramine, gastrointestinal complaints, especially constipation, abdominal pain and unpalatability are most frequently observed. Continued administration along with dietary manipulation (e.g. addition of dietary fibre) and/or stool softeners results in diminished... (truncated)

Title: Reduction of endotoxin attenuates liver fibrosis through suppression of hepatic stellate cell activation and remission of intestinal permeability in a rat non-alcoholic steatohepatitis model.
Previous clinical studies have demonstrated that endotoxin/toll-like receptor 4 (TLR4) signaling is critical in the inflammatory pathways associated with non-alcoholic steatohepatitis (NASH). In human and animal studies, NASH was associated with portal lipopolysaccharide (LPS) and the plasma LPS level was hypothesized to be associated with small intestinal bacterial overgrowth, change in composition of the microbiota and increased intestinal permeability. The aim of the present study was to investigate the roles of endogenous endotoxin and TLR4 in the pathogenesis of NASH. The effects of antibiotics were assessed in vivo using a choline deficiency amino acid (CDAA)-induced experimental liver fibrosis model. Antibiotics,... (truncated)

Title: Effect of the antibiotic neomycin on the toxicity of the glycoside vicine in rats.
Vicine is hydrolyzed by microflora to highly reactive free radical generating compound divicine which causes mortality and other adverse effects. This study in the rats established the effect of a broad spectrum and poorly absorbed antibiotic, neomycin sulfate on the toxicity of vicine. The results showed extremely decrease in mortality rate in the group pretreated with neomycin. Hemoglobin (Hb) concentration, hematocrit (Hct) value, and red blood cells (RBCs) count were significantly decreased after injection of vicine and the improvement of these values in the group pretreated with neomycin. The same results were observed in white blood cells (WBCs). The results... (truncated)

Title: Abnormalities of liver function and hepatic damage associated with total parenteral nutrition.
Liver dysfunction associated with total parenteral nutrition (TPN) probably is of multifactorial etiology. Available data justify the following recommendations. First, patients should be given balanced and complete solutions tailored to their specific needs and should not be overfed. Second, serum liver enzymes should be measured at least once a week. Third, oral or enteral intake, even if minimal, should be instituted as soon as possible. Fourth, glutamine may prove useful, although this measure is experimental. Finally, if liver dysfunction is detected, oral metronidazole, neomycin, or gentamicin may prove beneficial.

Title: Current trends in the treatment of hepatic encephalopathy.
Hepatic encephalopathy (HE) is a common reversible neuropsychiatric syndrome associated with chronic and acute liver dysfunction and significant morbidity and mortality. Although a clear pathogenesis is yet to be determined, elevated ammonia in the serum and central nervous system are the mainstay for pathogenesis and treatment. Management includes early diagnosis and prompt treatment of precipitating factors (infection, gastrointestinal bleeding, electrolyte disturbances, hepatocellular carcinoma, dehydration, hypotension, and use of benzodiazepines, psychoactive drugs, and/or alcohol). Clinical trials have established the efficacy of lactulose and lactitol enemas in the treatment of acute hepatic encephalopathy. Extensive clinical experience has demonstrated the efficacy of oral... (truncated)

MESH:D017338 - cladribine

Summary:

LLM Prediction Score	0.482 (normalized)
LLM Confidence Score	0.980
Golden Answer (Severity Class)	0.375 (normalized)
Prediction Error	0.107

Retrieved Context:

Title: An open-label pilot trial of cladibrine (2-cholordeoxyadenosine) in patients with primary sclerosing cholangitis.
Cladribine (2-chlorodeoxyadenosine) is a nucleoside analog with specific antilymphocytic activity that has been used in patients with a variety of lymphoid malignancies and autoimmune diseases. Primary sclerosing cholangitis (PSC) is a chronic hepatic autoimmune disorder of unknown etiology, thought to be mediated by biliary autoreactive cytotoxic lymphocytes. Because cladribine is an effective antilymphocytic drug, it may have potential disease-modifying activity in patients with PSC. We studied four patients with stages I and II PSC in an open-label pilot trial of 6 months' duration and 2 years' follow-up. Drugs were administered at 0.1 mg/kg/d subcutaneously for 5 days per monthly cycle... (truncated)

Title: Expert Narrative Review of the Safety of Cladribine Tablets for the Management of Relapsing Multiple Sclerosis.
Cladribine tablets (CladT) is a highly active oral disease-modifying therapy (DMT) for the management of relapsing multiple sclerosis (RMS). CladT acts as an immune reconstitution therapy, in that two short courses of treatment 1 year apart have been shown to suppress disease activity for a prolonged period in most patients, without need for continued DMT. Each course of CladT induces a profound reduction in B lymphocytes that recovers over months, and serious lymphopenia (Grade 3-4) is uncommon. Smaller reductions in levels of T lymphocytes occur slightly later: on average, these remain within the normal range and repopulate progressively. A larger effect... (truncated)

Title: Acute idiosyncratic liver injury after Cladribine treatment for multiple sclerosis: first case report and review on associated hepatic disorders.
In recent years, several disease-modifying therapies have been developed for the treatment of multiple sclerosis (MS). Cladribine transiently depletes B and T lymphocytes, with subsequent gradual cell recovery. No cases are reported in literature describing Cladribine drug-induced liver injury (DILI). We describe the case of a 19-year-old woman who developed acute idiosyncratic liver injury 12 days after treatment with Cladribine. Post-marketing adverse event reporting is of paramount importance to allow an early recognition and treatment. Moreover, evaluation of the physiopathological mechanism underlying drug-induced hepatic toxicity can provide clinicians with valuable instruments for prevention and treatment.

Title: Use of clofarabine for acute childhood leukemia.
A second-generation of purine nucleoside analogs, starting with clofarabine, has been developed in the course of the search for new therapeutic agents for acute childhood leukemia, especially for refractory or relapsed disease. Clofarabine is a hybrid of fludarabine and cladribine, and has shown to have antileukemic activity in acute lymphoblastic leukemia as well as in myeloid disorders. As the only new antileukemic chemotherapeutic agent to enter clinical use in the last 10 years, clofarabine was approved as an orphan drug with the primary indication of use in pediatric patients. Toxicity has been tolerable in a heavily pretreated patient population, and... (truncated)

Title: Hepatobiliary Adverse Reactions during Treatment with Cladribine: Analysis of Data from the European Spontaneous Reporting System.
Cladribine belongs to the group of disease-modifying therapies (DMTs) used to treat multiple sclerosis (MS). According to the highlights of a meeting held by the Pharmacovigilance Risk Assessment Committee (PRAC) on 14 January 2022, cladribine may be associated with the occurrence of liver injury, and thus liver function monitoring is recommended.

MESH:D007052 - ibuprofen

Summary:

LLM Prediction Score	0.480 (normalized)
LLM Confidence Score	0.990
Golden Answer (Severity Class)	0.375 (normalized)
Prediction Error	0.105

Retrieved Context:

Title: Ibuprofen Increases the Hepatotoxicity of Ethanol through Potentiating Oxidative Stress.
Over 30 million prescriptions of NSAIDs (non-steroidal anti-inflammatory drugs) are issued every year. Considering that these drugs are available without a prescription as over the counter (OTC) drugs, their use will be astronomical. With the increasing use of NSAIDs, their adverse effects are drawing attention. Especially, stomach bleeding, kidney toxicity, liver toxicity, and neurological toxicity are reported as common. Ibuprofen, one of the extensively used NSAIDs along with aspirin, can also induce liver toxicity, but few studies are addressing this point. Here we examined the liver toxicity of ibuprofen and investigated whether co-exposure to ethanol can manifest synergistic effects. We... (truncated)

Title: Drug-induced liver disease.
The past year has seen several additions to the list of drugs that cause hepatic injury. Many of these agents produce fulminant hepatic necrosis and, in some cases, were withdrawn from the market (eg, bromfenac). Other drugs had warnings placed in their labeling along with stringent monitoring guidelines to alert physicians and patients alike to the potential for severe hepatic injury (eg, troglitazone, tolcapone). New reports of hepatotoxicity continued to appear for many older agents, in some cases expanding the hepatotoxic spectrum for the drugs. Vanishing bile duct syndrome has drawn increasing attention and is now associated with more than... (truncated)

Title: Systematic review: ibuprofen-induced liver injury.
Nonsteroidal anti-inflammatory drugs (NSAIDs) are a leading cause of drug-induced liver injury (DILI) across the world. Ibuprofen is one of the most commonly used and safest NSAIDs, nevertheless reports on ibuprofen-induced hepatotoxicity are available.

Title: Diclofenac hepatitis.
We report five cases of biopsy-proven hepatitis developing between six and 20 weeks after administration of diclofenac. In one patient jaundice had previously developed following use of ibuprofen. In another the clinical, biochemical and histopathological features were those of chronic active hepatitis and treatment with corticosteroids was required. All patients recovered from their liver injury without sequelae. Resolution of symptoms occurred between three and 12 weeks following cessation of the drug, while liver function tests returned to normal between seven and 16 weeks after drug withdrawal, except in the patient with chronic active hepatitis who remained biochemically abnormal for eight... (truncated)

Title: Protective role of c-Jun N-terminal kinase-2 (JNK2) in ibuprofen-induced acute liver injury.
Ibuprofen is a worldwide used non-steroidal anti-inflammatory drug which may cause acute liver injury (ALI) requiring liver transplantation. We aimed to unveil the molecular pathways involved in triggering ibuprofen-induced ALI, which, at present, remain elusive. First, we investigated activation of essential pathways in human liver sections of ibuprofen-induced ALI. Next, we assessed the cytotoxicity of ibuprofen in vitro and developed a novel murine model of ibuprofen intoxication. To assess the role of JNK, we used animals carrying constitutive deletion of c-Jun N-terminal kinase 1 (Jnk1^{-/-}) or Jnk2 (Jnk2^{-/-}) expression and included investigations using animals with hepatocyte-specific Jnk deletion... (truncated)

MESH:D019258 - saquinavir

Summary:

LLM Prediction Score	0.480 (normalized)
LLM Confidence Score	0.970
Golden Answer (Severity Class)	0.375 (normalized)
Prediction Error	0.105

Retrieved Context:

Title: Unexpected Hepatotoxicity of Rifampin and Saquinavir/Ritonavir in Healthy Male Volunteers.
OBJECTIVES: Rifampin is a potent inducer of the cytochrome P450 3A4 isoenzyme (CYP3A4) that metabolizes most protease inhibitor (PI) antiretrovirals. This study was designed to evaluate the steady-state pharmacokinetics and tolerability of the coadministration of the PIs saquinavir and ritonavir (a CYP3A4 inhibitor used as a pharmacoenhancer of other PIs) and rifampin when coadministered in healthy HIV-negative volunteers. METHODS: In an open-label, randomized, one sequence, two-period crossover study involving 28 healthy HIV-negative volunteers, arm 1 was randomized to receive saquinavir/ritonavir 1000/100 mg twice daily while arm 2 received rifampin 600 mg once daily for 14 days. Both arms were then... (truncated)

Title: A literature review of liver function test elevations in rifampin drug-drug interaction studies.
Although rifampin drug-drug interaction (DDI) studies are routinely conducted, there have been instances of liver function test (LFT) elevations, warranting further evaluation. A literature review was conducted to identify studies in which combination with rifampin resulted in hepatic events and evaluate any similarities. Over 600 abstracts and manuscripts describing rifampin DDI studies were first evaluated, of which 30 clinical studies reported LFT elevations. Out of these, 11 studies included ritonavir in combination with other drug(s) in the rifampin DDI study. The number of subjects that were discontinued from treatment on these studies ranged from 0 to 71 (0-100% of subjects... (truncated)

Title: Association of saquinavir plasma concentrations with side effects but not with antiretroviral outcome in patients infected with protease inhibitor-susceptible human immunodeficiency virus type 1.
The objective of this study was to identify parameters among saquinavir pharmacokinetics, patients' demographics or comedications, to be addressed for improved personalized therapy. The presence of human immunodeficiency virus type 1 (HIV-1) RNA at therapy week 48 (principal target parameter), CD4 cell count at week 48, infections and side effects during 48 weeks, indicators of liver toxicity and lipid abnormalities at week 48, and a 12-h saquinavir plasma concentration-versus-time profile were assessed in 56 patients receiving saquinavir-ritonavir (1,000 and 100 mg, respectively) twice daily (44 therapy-naïve and 12 antiretrovirally pretreated patients) for association with saquinavir plasma concentrations, demographics, baseline values... (truncated)

Title: Potential Hepatotoxicity of Efavirenz and Saquinavir/Ritonavir Coadministration in Healthy Volunteers.
OBJECTIVE: This study was designed to investigate the pharmacokinetic effects of coadministration of saquinavir/ritonavir with efavirenz at steady state. METHODS: Healthy volunteers in this open-label, two-arm, one-sequence, two-period crossover study (planned enrollment of 40 participants) were randomized to one of two treatment arms: those in Arm 1 were scheduled to receive saquinavir/ritonavir 1,000/100 mg orally twice daily for 29 days and efavirenz 600 mg orally once daily starting on day 15 and continuing through day 29; participants randomized to Arm 2 were to receive efavirenz once daily for 29 days and saquinavir/ritonavir 1,000/100 mg twice daily starting on day 15... (truncated)

Title: Antiviral hepatitis and antiretroviral drug interactions.
More and more HIV-infected patients are treated for viral hepatitis, increasing interactions. HEPATITIS C: The concomitant use of didanosine and ribavirin increases the risk of mitochondrial toxicity, responsible for pancreatitis and/or lactic acidosis. Lactic acidosis is characterized by a high mortality rate. Thus, didanosine, but also stavudine, should not be co-administered with ribavirin. Cases of hepatic decompensation have been reported in cirrhotics concomitantly receiving ribavirin and didanosine. Thus, this co-admininistration should be contraindicated in patients with advanced liver fibrosis. Anemia is a frequent side effect of ribavirin. In patients with zidovudine-related anemia, this drug should be discontinued before prescribing ribavirin.... (truncated)

MESH:D009271 - naltrexone

Summary:

LLM Prediction Score	0.270 (normalized)
LLM Confidence Score	0.990
Golden Answer (Severity Class)	0.375 (normalized)
Prediction Error	0.105

Retrieved Context:

Title: Naltrexone: report of lack of hepatotoxicity in acute viral hepatitis, with a review of the literature.
Many clinicians appear to be concerned about the potential hepatotoxicity of the opiate antagonist naltrexone (NTX) and this may be one reason why it is not used more widely in treating both heroin and alcohol abusers. Some much-quoted early studies noted abnormalities in liver function tests (LFTs) in very obese patients taking high doses, although there was no evidence of clinically significant liver dysfunction. These concerns may be reinforced by advice in the UK product information sheet to perform LFTs before and during treatment, by high infection rates with hepatitis C virus (HCV) among injecting heroin addicts and by the... (truncated)

Title: Efficacy and tolerability of naltrexone in the management of alcohol dependence.
Naltrexone, a broad opioid-receptor antagonist, was the first medication since disulfiram to be approved by the United States of America Food and Drug Administration for the treatment of alcohol dependence. In the initial clinical trials in the early 1990s, oral naltrexone, 50 mg, was shown to significantly reduce the risk of relapsing to heavy drinking compared to placebo. These early trials were followed by other trials throughout the world such that by 2010 about 4,000 individuals had been studied. Meta-analyses of these trials revealed that oral naltrexone is effective in reducing relapse to heavy drinking but less effective in enhancing... (truncated)

Title: Kratom-induced transaminitis with subsequent precipitated opioid withdrawal following naltrexone.
Kratom is an herbal supplement that has gained popularity for recreational use within the United States. Kratom exerts opioid-like effects and, although not US FDA approved, is commonly used for self-treatment of pain, withdrawal management from opioids, and euphoria. Drug-related hepatic injury has been associated with kratom use. All of this raises concern for patient safety and monitoring. The potential for additive liver toxicity must be considered when kratom is used concurrently with hepatotoxic, over-the-counter, herbal, and prescription medications. This case report describes a case of kratom-induced liver inflammation complicated by opioid withdrawal that was precipitated by initiation of IM... (truncated)

Title: Opioid peptides and primary biliary cirrhosis.
Patients with liver disease have increased plasma concentrations of the endogenous opioid peptides methionine enkephalin and leucine enkephalin. As an initial investigation to determine whether opioid peptides contribute to any of the clinical manifestations of hepatic disease nalmefene, a specific opioid antagonist devoid of agonist activity, was given to 11 patients with cirrhosis. They all experienced a severe opioid withdrawal reaction on starting the drug. In the nine patients with primary biliary cirrhosis pruritus was greatly alleviated, fatigue seemed to improve, and plasma bilirubin concentration, which had been rising, showed a modest fall in all except one patient. These results... (truncated)

Title: Study of hepatotoxicity of naltrexone in the treatment of alcoholism.
Since a black box warning was issued by the Food and Drug Administration regarding the use of the opiate antagonist naltrexone (NTX), many clinicians have been concerned about current labeling of the potential hepatotoxicity risk of NTX in the treatment of opiate dependence and alcoholism. Despite many reports that demonstrated that the use of NTX did not cause elevation of liver enzymes, controversy concerning whether NTX is hepatotoxic continues. The current study monitored 74 alcoholic patients who received 25mg of NTX daily in the first week and then 50mg of NTX daily for the rest of the 12-week period. After... (truncated)

MESH:D013390 - succinylcholine

Summary:

LLM Prediction Score	0.105 (normalized)
LLM Confidence Score	0.990
Golden Answer (Severity Class)	0.0 (normalized)
Prediction Error	0.105

Retrieved Context:

Title: Prolonged apnea of an oral surgery patient after administration of succinylcholine.
To properly diagnose the presence of a genetic pseudocholinesterase variation, the family history is important. Furthermore, the DN and pseudocholinesterase tests are most useful, but if facilities for these are not available, one should be wary of patients with abnormal liver function tests. Certainly medical history, family medical history, laboratory evaluation, and knowledge of the disease processes should avoid unnecessary complications.

Title: Recent advances in neuromuscular blocking agents.
Factors driving the development of neuromuscular blocking agents are discussed. The goal of recent development of neuromuscular blocking agents is to develop agents with fewer adverse effects than succinylcholine and greater control. Greater control can be achieved through a short duration of action and a fast onset, similar to that found with succinylcholine. Duration control can be achieved through rapid, reliable metabolism that is organ independent, as with cisatracurium, or that occurs in the liver, because this will fail only in patients with severe hepatic disease. Rapid onset can be achieved by giving higher doses of drugs that have a... (truncated)

Title: Anaesthesia for the patient with neonatal adrenoleukodystrophy.
The authors present and discuss the care of a nine-month-old with neonatal adrenoleukodystrophy who required general anaesthesia for gastrointestinal endoscopy. Neonatal adrenoleukodystrophy is an inherited disorder of peroxisomal enzymes. Anaesthetic care may be affected by the presence of hypotonia, liver function abnormalities, gastroesophageal reflux, and impaired adrenocortical function. Preoperative sedation is contraindicated because of the risk of precipitating airway obstruction due to pre-existing hypotonia. Anaesthetic induction and tracheal intubation should be performed to minimize the risk for aspiration of gastric contents. The choice of muscle relaxant should take into account the pre-existing hypotonia as well as the possibility of hyperkalaemia... (truncated)

Title: Halothane and children: the first quarter century.
For the past 25 years, halothane has been the primary anesthetic agent at Children's Hospital, Columbus, Ohio. To confirm our impression that adverse reactions to halothane are rarely a problem in children, we examined the records of 200,311 cases conducted with halothane from June 1, 1958, through May 31, 1983. Life-threatening complications due to side effects were identified in fifteen patients, and could be grouped into three areas: hepatitis (one), malignant hyperthermia (ten), and cardiac arrhythmias (four). No child died or sustained permanent sequelae. In eleven instances, other drugs (succinylcholine, atropine, cocaine, and epinephrine) possibly contributed to the adverse reactions.

Title: The Importance of Patient-Specific Factors for Hepatic Drug Response and Toxicity.
Responses to drugs and pharmacological treatments differ considerably between individuals. Importantly, only 50%-75% of patients have been shown to react adequately to pharmacological interventions, whereas the others experience either a lack of efficacy or suffer from adverse events. The liver is of central importance in the metabolism of most drugs. Because of this exposed status, hepatotoxicity is amongst the most common adverse drug reactions and hepatic liabilities are the most prevalent reason for the termination of development programs of novel drug candidates. In recent years, more and more factors were unveiled that shape hepatic drug responses and thus underlie the... (truncated)

MESH:D000068679 - emtricitabine

Summary:

LLM Prediction Score	0.145 (normalized)
LLM Confidence Score	0.990
Golden Answer (Severity Class)	0.25 (normalized)
Prediction Error	0.105

Retrieved Context:

Title: Synthesis of a long acting nanoformulated emtricitabine ProTide.
While antiretroviral therapy (ART) has revolutionized treatment and prevention of human immunodeficiency virus type one (HIV-1) infection, regimen adherence, viral mutations, drug toxicities and access stigma and fatigue are treatment limitations. These have led to new opportunities for the development of long acting (LA) ART including implantable devices and chemical drug modifications. Herein, medicinal and formulation chemistry were used to develop LA prodrug nanoformulations of emtricitabine (FTC). A potent lipophilic FTC phosphoramidate prodrug (M2FTC) was synthesized then encapsulated into a poloxamer surfactant (NM2FTC). These modifications extended the biology, apparent drug half-life and antiretroviral activities of the formulations. NM2FTC demonstrated a... (truncated)

Title: Safety and tolerability of Elvitegravir/Cobicistat/Emtricitabine/Tenofovir Disoproxil fumarate in a real life setting: Data from surveillance cohort long-term toxicity antiretrovirals/antivirals (SCOLTA) project.
The study aim was to evaluate the impact on Liver and Kidney toxicity of the single tablet regimen Elvitegravir/Cobicistat/Emtricitabine/Tenofovir Disoproxil Fumarate (EVG/COBI/FTC/TDF) on Antiretroviral Therapy (ART) experienced or naïve patients.

Title: Emtricitabine (Emory University/Glaxo Wellcome/Triangle Pharmaceuticals).
Triangle Pharmaceuticals has licensed the development rights to emtricitabine (FTC), an antiviral nucleoside analog that is structurally related to 3TC, from Emory University [207618]. It is in phase III trials for HIV [324921] and in phase I/II trials for hepatitis B (HBV) [305302]. In June 1999, Triangle and Abbott Laboratories entered into an alliance for the development and marketing of six antiviral products, including FTC [326824]. An NDA was expected to be filed in 2000 [324921]. US-05814639 has been issued covering FTC [300465]. In April 2000, a report appeared in the South African media which suggested that a Triangle-sponsored trial... (truncated)

Title: Spatial Distribution Profiles of Emtricitabine, Tenofovir, Efavirenz, and Rilpivirine in Murine Tissues Following <i>In Vivo</i> Dosing Correlate with Their Safety Profiles in Humans.
Emtricitabine (FTC), tenofovir (TFV), efavirenz (EFV), and rilpivirine (RPV) are currently used as components of HIV combination therapy. Although these drugs are widely used in antiretroviral therapy, several organ toxicities related to TFV and EFV have been observed clinically. TFV is associated with nephrotoxicity, whereas EFV-related hepatotoxicity and neurotoxicity have been reported. While the precise molecular mechanisms related to the above-mentioned clinically observed toxicities have yet to be elucidated, understanding the local tissue distribution profiles of these drugs could yield insights into their safety profiles. To date, the distributions of these drugs in tissue following <i>in vivo</i> exposure are poorly... (truncated)

Title: Discovery and Development of the Anti-Human Immunodeficiency Virus Drug, Emtricitabine (Emtriva, FTC).
The HIV/AIDS epidemic, which was first reported on in 1981, progressed in just 10 years to a disease afflicting 10 million people worldwide including 1 million in the US. In 1987, AZT was approved for treating HIV/AIDS. Unfortunately, its clinical usefullness was severly limited by associated toxicities and the emergence of resistance. Three other drugs that were approved in the early 1990s suffered from similar liabilities. In 1990, the Liotta group at Emory University developed a highly diastereoselective synthesis of racemic 3'-thia-2',3'-dideoxycytidine and 3'-thia-2',3'-5-fluorodideoxycytidine and demonstrated that these compounds exhibited excellent anti-HIV activity with no apparent cytotoxicity. Subsequently, the enantiomers... (truncated)

MESH:C062402 - gadoteridol

Summary:

LLM Prediction Score	0.104 (normalized)
LLM Confidence Score	0.970
Golden Answer (Severity Class)	0.0 (normalized)
Prediction Error	0.104

Retrieved Context:

Title: Gadoteridol-enhanced MR imaging of malignant hepatic tumors: effects of triple versus standard doses on lesion-liver contrast.
The purpose of this study was to compare liver signal-to-noise ratio (SNR), lesion SNR, and lesion-liver contrast-to-noise-ratio (CNR) in patients with malignant liver lesions after the administration of a standard dose (0.1 mmol/kg of body weight) or a triple dose (0.3 mmol/kg) of a gadolinium chelate (gadoteridol). We hypothesized that the higher dose would produce a higher lesion-liver CNR and therefore increase the conspicuity of hepatic lesions.

Title: Benefits and Detriments of Gadolinium from Medical Advances to Health and Ecological Risks.
Gadolinium (Gd)-containing chelates have been established as diagnostics tools. However, extensive use in magnetic resonance imaging has led to increased Gd levels in industrialized parts of the world, adding to natural occurrence and causing environmental and health concerns. A vast amount of data shows that metal may accumulate in the human body and its deposition has been detected in organs such as brain and liver. Moreover, the disease nephrogenic systemic fibrosis has been linked to increased Gd³⁺ levels. Investigation of Gd³⁺ effects at the cellular and molecular levels mostly revolves around calcium-dependent proteins, since Gd³⁺ competes with calcium due to... (truncated)

Title: Gadobutrol in India-A Comprehensive Review of Safety and Efficacy.
Gadobutrol is a gadolinium (Gd)-based contrast agent for magnetic resonance imaging (MRI). In India, gadobutrol is approved for MRI of the central nervous system (CNS), liver, kidneys, breast and for MR angiography for patients 2 years and older. The standard dose for all age groups is 0.1 mmol/kg body weight. The safety profile has been demonstrated in 42 clinical phase 2 to 4 studies (>6800 patients), 7 observational studies, and by assessing pharmacovigilance data of 29 million applications. Furthermore, studies in children, adults, and elderly and in patients with impaired liver or kidney function did not show any increased adverse... (truncated)

Title: Risk factors for developing gadolinium-induced nephrogenic systemic fibrosis.
To identify medications and other potential risk factors, in addition to renal dysfunction, for developing gadolinium-induced nephrogenic systemic fibrosis (NSF).

Title: Management of patients with risk factors.
This review addresses concomitant diseases and risk factors in patients treated for diseases of the ears, nose and throat in outpatient and hospital services. Besides heart disease, lung disease, liver disease and kidney disease, this article also covers disorders of coagulation (including therapy with new oral anticoagulants) and electrolyte imbalance. Special attention is paid to the prophylaxis, diagnosis and treatment of perioperative delirium. It is also intended to help optimise the preparation for surgical procedures and pharmacotherapy during the hospital stay.

MESH:D017035 - pravastatin

Summary:

LLM Prediction Score	0.272 (normalized)
LLM Confidence Score	0.980
Golden Answer (Severity Class)	0.375 (normalized)
Prediction Error	0.103

Retrieved Context:

Title: Autoimmune-like Drug-induced Liver Injury Caused by Atorvastatin and Demonstration of the Safety Profile of Pravastatin: A Case Report and Literature Review.
Statin-induced liver injury is a well-recognized but rare phenomenon with hepatocellular, cholestatic, and mixed phenotypes. Most studies do not recommend regular monitoring of liver function tests (LFTs) after starting statins unless clinically indicated. We report a case of autoimmune-like atorvastatin-induced liver injury (aminotransferases > 5 times the upper limit of normal) that was detected on routine follow-up after three months in an asymptomatic patient. In addition to elevation in transaminases, the patient had weakly positive ANAs. Anti-smooth muscle antibody (ASMA) was positive in titers of 1:680. Screening for viral hepatitis A-E was negative. Other diagnostic investigations showed complete blood examination,... (truncated)

Title: Recurrence and Severe Worsening of Hepatotoxicity After Reintroduction of Atorvastatin in Combination With Ezetimibe.
Severe hepatotoxicity is a rare but well-known adverse reaction to statins. However, despite the widespread use of statins, only a few cases describing statin reexposure or switch to another statin after liver injury have been published. The literature on hepatotoxicity with ezetimibe alone or in combination with statins is also scarce. We report a case where a patient with a history of elevated liver enzymes while using atorvastatin, but prior and subsequent good tolerance to simvastatin and pravastatin, developed drug-induced liver injury on reexposure to a combination of atorvastatin and ezetimibe. The hepatotoxicity in our patient was most likely caused... (truncated)

Title: Hepatotoxicity of hydroxy-methyl-glutaryl-coenzyme A reductase inhibitors.
The first clinical studies on hydroxy-methyl-glutaryl-coenzyme A (HMG-CoA) reductase inhibitors reported a low incidence of liver toxicity. The personal observation of a case of simvastatin-induced acute cholestatic hepatitis prompted us to evaluate the true incidence of hepatic side effects of these drugs in a large Italian population. One hundred patients taking simvastatin and ninety patients treated with pravastatin were followed-up six months with periodical evaluation of serum lipid levels and liver function test. In 5% of simvastatin-treated patients and 4.5% of pravastatin-treated patients significant liver toxicity was observed, which required drug discontinuation. The mechanism of liver damage was direct, idiosyncratic,... (truncated)

Title: Atorvastatin-induced acute elevation of hepatic enzymes and the absence of cross-toxicity of pravastatin.
Atorvastatin has been associated with liver injury. We reported here two cases of aminotransferases elevation within 12 h of low-dose atorvastatin therapy. Liver functions were fully recovered to the baseline level 11 days after discontinuation of atorvastatin treatment. The possible relative risk factors included advanced age, chronic and systemic diseases, and co-administration of cytochrome P450 3A (CYP3A) enzyme-dependent metabolic drugs or its inhibitors such as clopidogrel and diltiazem. No significant transaminase elevation was observed after switching to pravastatin. Thus, pravastatin might be safer than atorvastain in patients with chronic or systemic diseases, or with co-administration of CYP3A enzyme-dependent drugs.

Title: Coenzyme Q10 or Creatine Counteract Pravastatin-Induced Liver Redox Changes in Hypercholesterolemic Mice.
Statins are the preferred therapy to treat hypercholesterolemia. Their main action consists of inhibiting the cholesterol biosynthesis pathway. Previous studies report mitochondrial oxidative stress and membrane permeability transition (MPT) of several experimental models submitted to diverse statins treatments. The aim of the present study was to investigate whether chronic treatment with the hydrophilic pravastatin induces hepatotoxicity in LDL receptor knockout mice (<i>LDLr</i>^{-/-}), a model for human familial hypercholesterolemia. We evaluated respiration and reactive oxygen production rates, cyclosporine-A sensitive mitochondrial calcium release, antioxidant enzyme activities in liver mitochondria or homogenates obtained from <i>LDLr</i>^{-/-} mice treated with pravastatin for 3 months. We... (truncated)

MESH:D006140 - guaifenesin

Summary:

LLM Prediction Score	0.103 (normalized)
LLM Confidence Score	0.990
Golden Answer (Severity Class)	0.0 (normalized)
Prediction Error	0.103

Retrieved Context:

Title: Important considerations for drugs, nutritional, and herbal supplements in pediatric solid organ transplant recipients.
Pediatric transplant recipients are on multiple prescription and non-prescription drugs. Many patients also use dietary, nutritional, and herbal supplements. This manuscript researched formulations of immunosuppressive drugs currently available and presents information on generic immunosuppressive drugs, commonly used non-prescription medications, dietary supplements, and herbal supplements. Immunosuppressive drugs are available in various formulations. Not all formulations are interchangeable. A number of FDA-approved generic formulations are available commercially in the United States. Generally generic formulations produce similar blood concentration vs time profiles compared to brand name products in adults and are considered to be bioequivalent. NSAID should be avoided in transplant patients due... (truncated)

Title: Facile Electrochemical Sensor for Sensitive and Selective Determination of Guaifenesin, Phenylephrine and Paracetamol on Electrochemically Pretreated Pencil Graphite Electrode.
Guaifenesin (GFS), phenylephrine (PHE) and paracetamol (PAR), drugs used in combination for the relief of cold and flu symptoms, were determined at electrochemically pretreated pencil graphite electrode. Differential pulse voltammetry (DPV) was used for the first time for the concomitant determination of the target compounds based on the electro-oxidation of PAR at 0.43 V, PHE at 0.74 V and GFS at 1.14 V in Britton-Robinson buffer pH 6.0. Under optimized experimental conditions, two linear ranges were obtained for PAR (2.50 × 10⁻⁶ M-1.00 × 10⁻⁵ M and 1.00 × 10⁻⁵ M-1.00 × 10⁻⁴ M) and for PHE and GFS linearity... (truncated)

Title: A case of cholestatic hepatitis associated with histologic features of acute cholangitis.
This report describes a case showing histologic features of acute cholangitis with an over-the-counter drug. A 48-year-old woman was diagnosed with general malaise and progressive jaundice. A thorough review of her medical history revealed that the patient had taken an over-the-counter drug, Pabron Gold(®), which she had used previously, that may have caused liver injury. Laboratory investigations revealed jaundice and liver dysfunction. Endoscopic retrograde cholangiography detected no extrahepatic biliary duct dilatation or stones. Liver biopsy indicated acute cholangitis involving neutrophils and eosinophils. Electron microscopy revealed fragmented nuclei, indicating that the degenerative bile duct-related epithelial cells were in an apoptotic process.

Title: Delayed increase in serum acetaminophen concentration after ingestion of a combination medications: a case report.
Acetaminophen is absorbed rapidly after oral intake, and serum concentration peaks within 4 hours. The Rumack-Matthew (RM) nomogram is widely used to identify the potential risk of liver dysfunction. However, the RM nomogram was intended for use only when a single agent was ingested. We report the case of a patient with overdose ingestion of an over-the-counter combination cold medication that contained acetaminophen, where the patient's serum concentration increased over time. Over-the-counter combination cold medications are designed to relieve cold symptoms. However, the possibility that other agents that were present in the drug may change gastrointestinal kinetics should also be... (truncated)

Title: Drug-induced acute liver failure: results of a U.S. multicenter, prospective study.
Acute liver failure (ALF) due to drug-induced liver injury (DILI), though uncommon, is a concern for both clinicians and patients. The Acute Liver Failure Study Group has prospectively collected cases of all forms of acute liver failure since 1998. We describe here cases of idiosyncratic DILI ALF enrolled during a 10.5-year period. Data were collected prospectively, using detailed case report forms, from 1198 subjects enrolled at 23 sites in the United States, all of which had transplant services. A total of 133 (11.1%) ALF subjects were deemed by expert opinion to have DILI; 81.1% were considered highly likely, 15.0% probable,... (truncated)

MESH:D005665 - furosemide

Summary:

LLM Prediction Score	0.352 (normalized)
LLM Confidence Score	0.990
Golden Answer (Severity Class)	0.25 (normalized)
Prediction Error	0.102

Retrieved Context:

Title: Humanized thymidine kinase-NOG mice can be used to identify drugs that cause animal-specific hepatotoxicity: a case study with furosemide.
Interspecies differences have limited the predictive utility of toxicology studies performed using animal species. A drug that could be a safe and effective treatment in humans could cause toxicity in animals, preventing it from being used in humans. We investigated whether the use of thymidine kinase (TK)-NOG mice with humanized livers could prevent this unfortunate outcome (i.e., "rescue" a drug for use in humans). A high dose of furosemide is known to cause severe liver toxicity in mice, but it is a safe and effective treatment in humans. We demonstrate that administration of a high dose of furosemide (200 mg/kg... (truncated)

Title: A One-Two Punch: Hydralazine-Induced Liver Injury in a Recovering Ischemic Hepatitis.
A 77-year-old woman presented to the emergency department with a 2-day history of nausea and vomiting. Her medical history included diabetes mellitus, hypertension, atrial fibrillation, dilated cardiomyopathy, and coronary artery disease. Her home medications included aspirin, clopidogrel, warfarin, digoxin, metoprolol, losartan, simvastatin, isosorbide dinitrate, furosemide, and spironolactone. Initial physical examination showed blood pressure of 170/80 mm Hg with a heart rate of 69 beats per minute, otherwise unremarkable. Initial laboratory workup was significant for INR of 3.6, with slightly elevated troponin I and creatinine of 0.06 ng/mL and 1.4 mg/dL, respectively. The patient was admitted to the medicine floor. However,... (truncated)

Title: The role of the c-Jun N-terminal kinases 1/2 and receptor-interacting protein kinase 3 in furosemide-induced liver injury.
1. The mechanisms of furosemide (FS) hepatotoxicity were explored in mice. Specifically, C57Bl/6 J mice were treated with 500 mg FS/kg bodyweight, and c-Jun N-terminal kinase (JNK) activation and receptor-interacting protein kinase 3 (RIP3) expression were measured by western blotting. Co-treatment with FS and the JNK inhibitor SP600125 was also performed, and FS-induced liver injury was compared in wild-type and RIP3 knockout (KO) mice. 2. JNK phosphorylation and RIP3 expression were increased in livers from the FS-treated mice as early as 6 h after treatment and persisted until at least 24 h. JNK phosphorylation was also observed in primary mouse... (truncated)

Title: Drug administration in chronic liver disease.
Cirrhosis encompasses a range of pathophysiological changes that may alter drug disposition. Drugs that are dependent primarily on the liver for their systemic clearance are more likely to be subject to reduced elimination and subsequent accumulation. Drug accumulation may lead to excessive plasma drug concentrations and adverse effects, if the adverse effects of the drug are concentration-dependent. The effects of hepatic insufficiency on the pharmacokinetics of drugs are not consistent or predictable. Furthermore, the influence of hepatic disease on the disposition of various drugs can vary, even though those drugs may share the same apparent metabolic pathway. Problems in forecasting... (truncated)

Title: Hypercalcemia worsened by the use of furosemide in a patient suffering from allopurinol-induced granulomatous hepatitis.
No abstract available.

MESH:D008625 - tiopronin

Summary:

LLM Prediction Score	0.273 (normalized)
LLM Confidence Score	0.890
Golden Answer (Severity Class)	0.375 (normalized)
Prediction Error	0.102

Retrieved Context:

Title: The role of tiopronin for the prevention of chemotherapy-related liver toxicity in advanced colorectal cancer patients treated with mFOLFOX7: a prospective analysis.
Chemotherapy-related hepatotoxicity is a limitation for the continuation of chemotherapy in patients with advanced colorectal cancer (CRC). This prospective study determined the efficacy of tiopronin infusion in chemotherapy-induced hepatoxicity.

Title: [Therapy of hepatic diseases with alpha-mercaptopropionylglycine].
Thiopronine has been used to treat 88 patients suffering from chronic hepatitis (65), liver cirrhosis in ascitic phase (14), alcoholic hepatosis(5) and acute hepatitis (4). The drug was employed at the attack stage i.v. at a daily dose of 1-2 grams, and at maintenance phase orally at a dose of 0.750-1 gram. It led to a gradual, significant improvement in clinical symptomatology and hepatofunctional haematochemical indices in all chronic forms, only decompensated cirrhosis showing a slight regression. In hepatic forms, normalisation of subjective and objective parameters was particularly fast, between the 4th and 7th days. Tolerance was very good in... (truncated)

Title: Prospective analysis of tiopronin in prevention of sorafenib and antiviral therapy inducing liver toxicity in advanced hepatitis B virus-related hepatocellular carcinoma.
Hepatotoxicity induced by sorafenib and antiviral therapy is a limitation for its continuation treatment for patients with advanced hepatitis B virus-related hepatocellular carcinoma (HCC). This prospective study determined the efficacy of tiopronin in hepatotoxicity prevention of HBV-related HCC treatment. Eighty-two patients (median age, 50 years; 71 % male) of advanced HCC treated with sorafenib and antiviral therapy were included, of whom 40 were given the supplementation of tiopronin. The primary endpoint was liver function which was checked before the treatment and every week during the therapy. Besides, course discontinuations, dose reductions, HBV DNA levels and treatment efficacy were evaluated. Patient... (truncated)

Title: Minimal change disease induced by tiopronin: a rare case report and a review of the literature.
Tiopronin (TP), a glycine derivative with a free thiol, is extensively used for the treatment of cystinuria. Moreover, TP is usually prescribed as hepatoprotective medicine in China. In the present case, a 36-year-old female who presented with foamy urine and general edema was admitted to the hospital. She had been taking TP for six months to treat drug-induced liver injury due to anti-tuberculosis drugs including isoniazid, rifampicin and pyrazinamide. The urine tests at admission revealed nephritic-range proteinuria with a daily urinary protein level of 8,024 mg. Meanwhile, albumin and cholesterol levels were abnormal. The light microscopy was negative and electron... (truncated)

Title: Prevention and management of idiosyncratic drug-induced liver injury: Systematic review and meta-analysis of randomised clinical trials.
Conducting randomised clinical trials (RCTs) in idiosyncratic drug-induced liver injury (DILI) is challenging. This systematic review aims to summarise the design and findings of RCTs in the prevention and management of idiosyncratic DILI. A systematic literature search up to January 31st, 2020 was performed. Recognised scales were used to assess methodological bias and quality of the studies. Quantitative and qualitative analyses were performed. Heterogeneity was assessed with I² statistic. Overall, 22 RCTs were included: 12 on prevention (n = 2,471 patients) and 10 in management (n = 797) of DILI/non-acetaminophen DILI-related acute liver failure (ALF). Silymarin (eight studies), bicyclol (four),... (truncated)

MESH:D016642 - bupropion

Summary:

LLM Prediction Score	0.524 (normalized)
LLM Confidence Score	0.990
Golden Answer (Severity Class)	0.625 (normalized)
Prediction Error	0.101

Retrieved Context:

Title: Potential Role of Bupropion Sustained Release for Cancer-Related Fatigue: a Double-Blind, Placebo-Controlled Study
Background: Cancer-related fatigue (CRF) is very common and can be experienced at all stages of disease and in survivors. CRF causes patients more distress than pain or nausea and vomiting. Different pharmacologic interventions have been evaluated for the management of CRF. The purpose of this study was to determine the efficacy of bupropion sustained release (SR) as a treatment for fatigue in patients with cancer. Methods: In this randomized, double-blind, placebo-controlled trial, patients with fatigue due to cancer were randomly assigned to either 150mg daily of bupropion SR or matching placebo. The primary endpoint was the changes in average daily... (truncated)

Title: Acute Hepatocellular Drug-Induced Liver Injury From Bupropion and Doxycycline.
The management and diagnosis of drug-induced liver injury (DILI) is often challenging, particularly when patients are taking multiple medications. We present a 29-year-old African American man who presented with jaundice and malaise after starting bupropion and doxycycline 2 weeks prior. He was found to have acute hepatocellular drug-induced liver injury with autoimmune features, and made a complete recovery with prednisone. Although bupropion and doxycycline are both known to cause liver toxicity, a closer inspection of the signature of liver injury and a review of prior related DILI cases assigns causality more to bupropion than doxycycline.

Title: Acute cholestatic hepatitis induced by bupropion prescribed as pharmacological support to stop smoking. A case report.
We report the first case of acute cholestatic hepatitis induced by bupropion. This antidepressant was taken by a 49-year-old female as adjuvant treatment to stop smoking. After 20 days of bupropion, the patient presented a symptomatology characterized by asthenia, nausea and scleral icterus and biochemical analyses showed a dramatic increase in direct bilirubin (up to 28 mg/dl) and transaminases (up to 68-fold normal limits). Antinuclear antibodies were positive (title = 1:80; speckled pattern). Biochemical analyses and antinuclear antibodies were normal two years earlier. The histology showed a pattern of acute hepatitis with involvement of bile ducts and with features of... (truncated)

Title: Rapid bupropion-induced hepatotoxicity: a case report and review of the literature.
Bupropion is an antidepressant that is also used as a non-nicotine method to aid in smoking cessation. Bupropion-induced hepatotoxicity is quoted to affect between 0.1% and 1% of treated patients with either a hepatocellular and/or cholestatic pattern of damage. The mechanism of damage is considered to be predominantly immune-mediated with the presence of a hypersensitivity syndrome (fever, rash, eosinophilia, autoantibodies) and a short latency period (1-6 weeks). We believe our reporting of this case to the already existing small list of only seven cases in the world literature will help practicing physicians to deal with the diagnostic and management dilemmas... (truncated)

Title: Liver injury associated with antidepressants.
Antidepressants are commonly prescribed and used in the management of depression, anxiety disorders, and other psychiatric illnesses. Antidepressants used in therapeutic dosing ranges are associated with causing several adverse drug reactions including hepatotoxicity. Paroxetine, fluoxetine, fluvoxamine, citalopram, mirtazapine and venlafaxine are associated with reversible liver injury upon discontinuation of the agent. Patient cases of hepatotoxicity involving the use of nefazodone, trazodone, duloxetine, bupropion, and sertraline are linked to causing death in its users. Due to the idiosyncratic nature of hepatotoxicity, monitoring of liver function tests and immediate discontinuation upon abnormal lab findings or signs and symptoms of liver dysfunction are... (truncated)

MESH:C060344 - dolasetron

Summary:

LLM Prediction Score	0.274 (normalized)
LLM Confidence Score	0.960
Golden Answer (Severity Class)	0.375 (normalized)
Prediction Error	0.101

Retrieved Context:

Title: Pharmacokinetics of dolasetron after oral and intravenous administration of dolasetron mesylate in healthy volunteers and patients with hepatic dysfunction.
In previous studies, dolasetron was shown to have both renal and hepatic elimination mechanisms. This study was conducted to determine the impact of varying degrees of hepatic dysfunction on the pharmacokinetics and safety of dolasetron and its reduced metabolites. Seventeen adults were studied: six healthy volunteers (group I), seven patients with mild hepatic impairment (Child-Pugh class A; group II), and four patients with moderate to severe hepatic impairment (Child-Pugh class B or C1; group III). Single 150-mg doses of dolasetron mesylate were administered intravenously and orally, with a 7-day washout period separating treatments. After intravenous administration, no differences were observed... (truncated)

Title: Palonosetron versus older 5-HT3 receptor antagonists for nausea prevention in patients receiving chemotherapy: a multistudy analysis.
No clinical standard currently exists for the optimal management of nausea induced by emetogenic chemotherapy, 7particularly delayed nausea.

Title: Effective Dose of Ramosetron for Prophylaxis of Postoperative Nausea and Vomiting in High-Risk Patients.
Postoperative nausea and vomiting (PONV) are common adverse events with an incidence of up to 80% in high-risk patients. Ramosetron, a selective 5-HT3 receptor antagonist, is widely used to prevent PONV. The purpose of this study was to evaluate the effective dose of ramosetron for the prevention of PONV in high-risk patients.

Title: The association of CYP2D6 genotype and postoperative nausea and vomiting in orthopedic trauma patients.
The CYP2D6 gene encodes for an enzyme that is involved in the metabolism of more than 25% of all medications, including many opioids and antiemetics. It may contribute to the risk of postoperative nausea and vomiting (PONV), a common surgical complication. However, little research has been conducted in this area. The purpose of this study was to explore the association of CYP2D6 genotypes with PONV in adult surgical trauma patients. Data from 112 patients (28% female) with single extremity fractures, aged 18-70 years, were analyzed. PONV was defined as present if patients reported nausea, were observed vomiting, or received medication... (truncated)

Title: Inadvertent Provocative Oral Ondansetron use Leading to Toxic Epidermal Necrolysis in an HIV-infected Patient.
Toxic epidermal necrolysis (TEN) is a severe cutaneous adverse reaction to drugs, characterized by extensive detachment of epidermis and mucous membranes with a mortality of 30-40%. An increased occurrence of cutaneous drug reactions is seen in patients with human immunodeficiency virus (HIV) infection. We present this case of TEN caused by ondansetron in an HIV-infected patient. A 24-year-old HIV-1-infected man on antitubercular therapy and cotrimoxazole, presented with extensive and confluent erosions involving the face, trunk, extremities and mucous membranes following the intake of oral ondansetron, ofloxacin and ornidazole. All the drugs were withdrawn and he was treated with intravenous dexamethasone... (truncated)

MESH:D000068717 - glatiramer acetate

Summary:

LLM Prediction Score	0.476 (normalized)
LLM Confidence Score	0.990
Golden Answer (Severity Class)	0.375 (normalized)
Prediction Error	0.101

Retrieved Context:

Title: Drug-Induced Liver Injury by Glatiramer Acetate Used for Treatment of Multiple Sclerosis: A Case Report.
Glatiramer acetate (GA, Copaxone) is an approved drug for the treatment of relapsing-remitting multiple sclerosis. Most common side effects observed with GA are local injection site reactions, which can include pain, swelling, or redness. However, systemic adverse event such as hepatotoxicity related to GA is rarely seen. In this report, we present a case of GA-induced toxic hepatitis associated with cholestatic and hepatocellular damage.

Title: Glatiramer acetate-induced hepatitis in a young female patient with multiple sclerosis.
We report the first Italian case of glatiramer acetate-related acute hepatotoxicity. A 25-years-old woman suffering from multiple sclerosis presented acute hepatitis after eight months of treatment. Neither infective, nor autoimmune markers were detected. Liver biopsy histology was consistent with drug-induced acute injury. Liver function tests became normal after eight weeks of treatment discontinuation. This report points out the importance of monitoring liver function during the first year of treatment with glatiramer acetate.

Title: The Disease-Modifying Therapies of Relapsing-Remitting Multiple Sclerosis and Liver Injury: A Narrative Review.
In this narrative review, we analyze pre-registration and post-marketing data concerning hepatotoxicity of all disease-modifying therapies (DMTs) available for the treatment of relapsing-remitting multiple sclerosis, including beta interferon, glatiramer acetate, fingolimod, teriflunomide, dimethyl fumarate, cladribine, natalizumab, alemtuzumab, and ocrelizumab. We review the proposed causal mechanisms described in the literature and we also address issues like use of DMTs in patients with viral hepatitis or liver cirrhosis. Most data emerged in the post-marketing phase by reports to national pharmacovigilance agencies and published case reports or case series. Serious liver adverse events are rare, but exact incidence is largely unknown, as are... (truncated)

Title: Acute hepatitis induced by glatiramer acetate.
Glatiramer acetate, a mixture of synthetic polypeptides able to prevent autoimmune encephalomyelitis in experimental models, is used as a treatment for patients with active relapsing-remitting multiple sclerosis. We report one case of glatiramer acetate induced hepatitis. Liver biopsy was consistent with a diagnosis of drug induced hepatitis and alanine aminotransferase returned to normal values after treatment discontinuation. The present case should serve as a warning that glatiramer acetate may cause acute liver disease.

Title: Liver injury and glatiramer acetate, an uncommon association: case report and literature review.
We report the case of a 65-year-old woman who presented with a 1-month history of progressive paraparesia associated with a thoracic lesion with irregular ring-like gadolinium enhancement. Biopsy of the lesion confirmed the demyelinating origin and brain magnetic resonance imaging showed additional lesions demonstrative of dissemination in space. Immunomodulatory therapy with glatiramer acetate (GA) was started after having a second relapse 2 months later. Shortly after initiation, the patient developed acute hepatitis. Liver function tests returned to normal values 5 months after discontinuation and the patient was diagnosed with drug-induced liver injury (DILI) associated with GA. A literature review identified... (truncated)

MESH:D008525 - medroxyprogesterone

Summary:

LLM Prediction Score	0.350 (normalized)
LLM Confidence Score	0.990
Golden Answer (Severity Class)	0.25 (normalized)
Prediction Error	0.100

Retrieved Context:

Title: Medroxyprogesterone acetate as a contraceptive for female drug addicts.
A study of 69 female drug addicts is presented who have been given depomedroxyprogesterone acetate (DMPA) as a contraceptive for 1 680 woman-months. No pregnancies were reported during the treatment period with DMPA. After stopping treatment, 60 pregnancies with 43 births were recorded, 12 with low birth weight. In 3 cases pregnancy ended with spontaneous abortion, and in 14 cases legal abortion were performed. During the study, regular liver function tests revealed no hepatotoxic effects from DMPA in this group of patients where one would expect a high frequency of a serum hepatitis. Neither coagulation nor blood pressure were affected.... (truncated)

Title: Evaluation and Management of Chronic Cholestatic Liver Diseases.
Cholestasis is defined as stagnation or a marked reduction in bile secretion and flow. Cholestatic jaundice can thus be classified as intrahepatic or extrahepatic cholestatic, depending on the level of obstruction to bile flow. It is important to recognize the complications of cholestatic in patients with chronic cholestatic liver disease. The two most common complications of cholestasis are pruritus and fatigue, with the former being the most responsive to treatment. Cholestyramine is the first-line treatment for cholestatic pruritus. Rifampicin and oral opioid antagonist naltrexone are extremely effective second-line treatments. To date, there are no specific treatments for chronic cholestatic fatigue... (truncated)

Title: Advances in contraception: vaginal contraceptive rings.
The vaginal contraceptive ring is very effective and user dependent. In this article, we will discuss the different types of vaginal contraceptive rings, namely, the etonogestrel/ethinyl estradiol (ENG/EE) ring (NuvaRing, Merck, Rahway, NJ, USA) and the segesterone acetate (SA)/EE (Annovera, Mayne Pharma, Raleigh, NC, USA) ring. The details of dosing and administration, indications, advantages, disadvantages, and cost-effectiveness are presented. This literature review was conducted using PubMed and Google Scholar. The search terms included 'vaginal contraceptive ring', 'etonogestrel/ethinyl estradiol ring', and 'segesterone acetate/ethinyl estradiol ring'. The search was then sorted by year from 2000 until present, and the most recent articles... (truncated)

Title: Hepatic impairment during simultaneous administration of medroxyprogesterone acetate and tamoxifen in the treatment of endometrial and ovarian carcinoma.
Simultaneous administration of medroxyprogesterone acetate (MPA) and tamoxifen markedly elevated the serum alanine and aspartate aminotransferase activities in 4 out of 30 patients with endometrial or ovarian carcinoma; and also slightly increased the activities of gamma-glutamyl transferase in 2 of the patients. These pathological changes spontaneously returned to normal in 1 patient, and after the cessation of tamoxifen or tamoxifen plus MPA treatment in 3 patients. This kind of hepatic impairment was thought to be caused by reversible damage of liver cells possibly associated with slight intrahepatic cholestasis. It is suggested that special attention be paid to liver function during... (truncated)

Title: Serum phospholipid fractionation after the use of long-acting progestational contraceptives.
The effect of the two long-acting progestational contraceptive injection, depot-medroxyprogesterone acetate (DMPA) and norethisterone onenathate (NET-OEN), on blood phospholipid fractions were studied in eighteen women. The post-injection results showed a significant increase in total serum phospholipids, serum lecithin and serum cephalins. Serum lysolecithin was significantly decreased while serum sphingomyelin showed no significant change. The mechanisms responsible for these phospholipid changes remain somewhat unclear. However deranged liver function may share in its explanation.

MESH:D019793 - fluorescein

Summary:

LLM Prediction Score	0.099 (normalized)
LLM Confidence Score	0.990
Golden Answer (Severity Class)	0.0 (normalized)
Prediction Error	0.099

Retrieved Context:

Title: Quantitative Kinetic Models from Intravital Microscopy: A Case Study Using Hepatic Transport.
The liver performs critical physiological functions, including metabolizing and removing substances, such as toxins and drugs, from the bloodstream. Hepatotoxicity itself is intimately linked to abnormal hepatic transport, and hepatotoxicity remains the primary reason drugs in development fail and approved drugs are withdrawn from the market. For this reason, we propose to analyze, across liver compartments, the transport kinetics of fluorescein-a fluorescent marker used as a proxy for drug molecules-using intravital microscopy data. To resolve the transport kinetics quantitatively from fluorescence data, we account for the effect that different liver compartments (with different chemical properties) have on fluorescein's emission rate.... (truncated)

Title: Hepatocytes maintain greater fluorescent bile acid accumulation and greater sensitivity to drug-induced cell death in three-dimensional matrix culture.
Primary hepatocytes undergo phenotypic dedifferentiation upon isolation from liver that typically includes down regulation of uptake transporters and up regulation of efflux transporters. Culturing cells between layers of collagen in a three-dimensional (3D) "sandwich" is reported to restore hepatic phenotype. This report examines how 3D culturing affects accumulation of fluorophores, the cytotoxic response to bile acids and drugs, and whether cell to cell differences in fluorescent anion accumulation correlate with differences in cytotoxicity. Hepatocytes were found to accumulate fluorescent bile acid (FBA) at significantly higher levels than the related fluorophores, carboxyfluorescein diacetate, (4.4-fold), carboxyfluorescein succinimidyl ester (4.8-fold), and fluorescein (30-fold).... (truncated)

Title: Antimitochondrial antibodies: reagent variables may lead to diagnostic error.
Laboratory-prepared and commercially obtained fluorescein-labeled rabbit antihuman IgG were compared in performing the antimitochondria antibody (AMA) assay. Identical results were obtained using either of the fluorescent antisera at protein concentrations of 1.5 mg/ml and 1:10 dilutions of patients' sera. Positive AMA tests with either antisera were observed in each of 7 patients with primary biliary cirrhosis (PBC), 2 of 83 patients with miscellaneous hepatic diseases, and in 1 of 24 patients with extrahepatic biliary obstruction (EBO) of 2-24 weeks duration. However, when undiluted commercial fluorescent antiserum (15.8 mg protein/ml) was substituted in the assay, sera from 11 of 23 AMA-negative... (truncated)

Title: The localization of Australia antigen by immunofluorescence.
We have studied the localization of Australia antigen, a particulate substance associated with hepatitis, by means of the fluorescent antibody technique. Preparations were made from 61 liver biopsy specimens taken from patients with infectious hepatitis, serum hepatitis, and a variety of other diseases. When tested with fluorescein-conjugated rabbit anti-Au(1) antisera all 26 patients who had Au(1) in their serum had specific fluorescence in their liver cells. The fluorescence appeared in three forms: as discrete particles within the nucleus, diffuse fluorescence of the entire nucleus, and fluorescence of the nuclear rim. Occasionally there were also fluorescent particles in the cytoplasm. Other... (truncated)

Title: Visualization of acute liver damage induced by cycloheximide in rats using PET with [(18)F]FEDAC, a radiotracer for translocator protein (18 kDa).
Liver damage induced by drug toxicity is an important concern for both medical doctors and patients. The aim of this study was to noninvasively visualize acute liver damage using positron emission tomography (PET) with N-benzyl-N-methyl-2-[7,8-dihydro-7-(2-[(18)F]fluoroethyl)-8-oxo-2-phenyl-9H-purin-9-yl]acetamide ([(18)F]FEDAC), a radiotracer specific for translocator protein (18 kDa, TSPO) as a biomarker for inflammation, and to determine cellular sources enriching TSPO expression in the liver. A mild acute liver damage model was prepared by a single intraperitoneal injection of cycloheximide (CHX) into rats. Treatment with CHX induced apoptosis and necrotic changes in hepatocytes with slight neutrophil infiltration. The uptake of radioactivity in the rat... (truncated)

MESH:D009582 - nitrofurantoin

Summary:

LLM Prediction Score	0.901 (normalized)
LLM Confidence Score	0.990
Golden Answer (Severity Class)	1.0 (normalized)
Prediction Error	0.099

Retrieved Context:

Title: An Unusual Case of Drug-Induced Liver Injury Secondary to Nitrofurantoin Use.
Nitrofurantoin is a commonly prescribed antibiotic for uncomplicated urinary tract infections. Despite a number of side effects, it is increasingly prescribed due to its low cost, high efficacy, and minimal antimicrobial resistance. One of the rare, however significant side effects of nitrofurantoin is idiosyncratic drug-induced liver toxicity. It commonly presents with abdominal pain and elevated liver enzymes. Interestingly, it can cause either an acute or a chronic hepatitis-like syndrome that can be severe and lead to liver failure or cirrhosis. We present a case of a healthy 24-year-old female who presented with epigastric abdominal pain, which was found to be... (truncated)

Title: Nitrofurantoin: friend or foe?
Nitrofurantoin remains the gold standard treatment of uncomplicated cystitis as well as prophylactic treatment of recurrent urinary tract infections. Drug-induced hepatotoxicity presents in acute (3 in 1 000 000) and chronic (1 in 1500) forms. We present a patient with acute liver failure after 5 days of treatment. A 69-year-old man admitted for chronic obstructive pulmonary disease exacerbation 5 days into treatment for cystitis with nitrofurantoin. On admission he was noted to be jaundiced with elevated liver enzymes and normal international normalised ratio. Investigation for infectious, autoimmune and cholestatic causes of hepatotoxicity was negative. The patient improved after discontinuation of... (truncated)

Title: [Liver cirrhosis due to chronic use of nitrofurantoin].
A 74-year-old woman was admitted with jaundice. She was suffering from generalised liver failure with a highly prolonged prothrombin time, a low albumin level and ascites. Further anamnesis revealed that she had been taking nitrofurantoin as a prophylactic agent for recurrent urinary tract infections every day for 5 years. Because of the indications for liver damage due to nitrofurantoin, the drug was discontinued immediately on admission. After withdrawal of nitrofurantoin there was a very gradual clinical and biochemical improvement. Liver biopsies to confirm the diagnosis revealed extensive liver damage with cirrhosis such as may be seen following long-term use of... (truncated)

Title: Nitrofurantoin-induced hepatotoxicity: a rare yet serious complication.
Nitrofurantoin is a commonly prescribed antibiotic for the treatment of recurrent uncomplicated urinary tract infections. Its importance has been emphasized by the current international clinical practice guidelines for the management of uncomplicated cystitis. Since its introduction into clinical practice, nitrofurantoin has been associated with various adverse effects, including hepatotoxicity. We searched the English-language literature using PubMed and SCOPUS for the period 1961 through the end of February 2013. Key search terms included "nitrofurantoin AND hepatotoxicity" as well as "nitrofurantoin AND hepatitis." When studies or case reports were found, we assessed articles cited in those publications. A broad spectrum of liver... (truncated)

Title: [Hepatic toxicity of nitrofurantoin. Cases reported to the Center for Monitoring Adverse Drug Reactions 1968-1998].
Forty-four cases of nitrofurantoin-associated hepatic injury were reported to the Danish Center for Monitoring of Adverse Drug reactions from 1968 to 1998. Forty-one were women with a median age of 69 years. They had been treated with nitrofurantoin for median one year (two days-seven years), and all had biochemical and clinical signs of hepatitis. In five cases the injury had been provoked by a rechallenge. Twenty-seven liver biopsies showed an equal amount of acute and chronic histopathological changes. Cirrhosis was diagnosed in seven cases. Four died in liver failure; the others apparently recovered without long-term symptoms. In rare cases, nitrofurantoin... (truncated)

MESH:D009761 - nystatin

Summary:

LLM Prediction Score	0.098 (normalized)
LLM Confidence Score	0.990
Golden Answer (Severity Class)	0.0 (normalized)
Prediction Error	0.098

Retrieved Context:

Title: Oral candidosis: treatment with absorbable and non-absorbable antifungal agents in children.
Oral candidosis in neonates and children is a common infection which occurs often during the first few months after birth, but occasionally also in older children with certain predisposing factors. In neonates, oral candidosis is usually benign, although the symptoms of such an acute infection can be disturbing to both the patient and the parents. In older children developing oral candidosis, specific predisposing factors may be present (e.g. immunodeficiency, chemotherapy, etc.). In such cases, the infection may constitute a source for further dissemination, leading to occasionally fatal Candida sepsis or to widespread chronic mucocutaneous candidosis. Treatment modalities to date include... (truncated)

Title: Be careful, mom and doc: hepatotoxicity associated with prescribed medications in young infants.
Accidental poisonings in young infants are relatively uncommon, and the careless caregiver is usually the culprit. We report two cases of hepatotoxicity due to prescribed medications. An infant was given 15 mL instead of 1.5 mL of paracetamol by his mother because she omitted the decimal point on the label of the drug bottle. The infant became symptomatic, and liver enzyme and clotting profile were abnormal, necessitating treatment with N-acetyl cysteine. Another infant was prescribed oral ketoconazole for thrush, resulting in elevation of liver enzymes. The serum alanine aminotransferase levels were transiently elevated but returned to normal, and both infants... (truncated)

Title: Effect of inhibitors of endocytosis and NF-kB signal pathway on folate-conjugated nanoparticle endocytosis by rat Kupffer cells.
The regular accumulation of nanoparticles in the liver makes them hepatotoxic and decreases the circulation time, thus reducing their therapeutic effect. Resolving this problem will be significant in improving bioavailability and reducing side effects. In this study, we reduced the phagocytosis of epirubicin (EPI)-loaded folic acid-conjugated pullulan acetate (FPA/EPI) nanoparticles by Kupffer cells (KCs) through internalization and nuclear factor kappa B (NF-kB) signal pathway inhibitors, thus allowing development of FPA/EPI nanoparticles as a nanodrug delivery system (NDDS) based on our previous study. FPA/EPI nanoparticles were prepared by the dialysis method. Rat KCs were preincubated with the following individual or compound... (truncated)

Title: Antifungals: From Pharmacokinetics to Clinical Practice.
The use of antifungal drugs started in the 1950s with polyenes nystatin, natamycin and amphotericin B-deoxycholate (AmB). Until the present day, AmB has been considered to be a hallmark in the treatment of invasive systemic fungal infections. Nevertheless, the success and the use of AmB were associated with severe adverse effects which stimulated the development of new antifungal drugs such as azoles, pyrimidine antimetabolite, mitotic inhibitors, allylamines and echinochandins. However, all of these drugs presented one or more limitations associated with adverse reactions, administration route and more recently the development of resistance. To worsen this scenario, there has been an... (truncated)

Title: Signal Detection of Potentially Drug-Induced Liver Injury in Children Using Electronic Health Records.
Background: This study proposes a quantitative 2-stage procedure to detect potential drug-induced liver injury (DILI) signals in pediatric inpatients using an data warehouse of electronic health records (EHRs). Methods: Eight years of medical data from a constructed database were used. A two-stage procedure was adopted: (i) stage 1: the drugs suspected of inducing DILI were selected and (ii) stage 2: the associations between the drugs and DILI were identified in a retrospective cohort study. Results: 1,196 drugs were filtered initially and 12 drugs were further potentially identified as suspect drugs inducing DILI. Eleven drugs (fluconazole, omeprazole, sulfamethoxazole, vancomycin, granulocyte colony-stimulating... (truncated)

MESH:D017693 - sodium bicarbonate

Summary:

LLM Prediction Score	0.098 (normalized)
LLM Confidence Score	0.990
Golden Answer (Severity Class)	0.0 (normalized)
Prediction Error	0.098

Retrieved Context:

Title: The Role of Sodium Bicarbonate in the Management of Some Toxic Ingestions.
Adverse reactions to commonly prescribed medications and to substances of abuse may result in severe toxicity associated with increased morbidity and mortality. According to the Center for Disease Control, in 2013, at least 2113 human fatalities attributed to poisonings occurred in the United States of America. In this article, we review the data regarding the impact of systemic sodium bicarbonate administration in the management of certain poisonings including sodium channel blocker toxicities, salicylate overdose, and ingestion of some toxic alcohols and in various pharmacological toxicities. Based on the available literature and empiric experience, the administration of sodium bicarbonate appears to... (truncated)

Title: Acute renal failure, thrombocytopenia, and elevated liver enzymes after concurrent abuse of alcohol and cocaine.
Cocaine has been associated with known adverse effects on cardiac, cerebrovascular and pulmonary systems. However, the effect of cocaine on other organs has not been extensively reported. A middle age man presented with abdominal pain and nausea after inhalation of crack cocaine. On admission, he was found to be hypertensive and tachycardic. Physical examination revealed mild abdominal tenderness without rebound. Laboratory investigations were significant for acute kidney failure with elevated serum creatinine (3.72 mg/dL), thrombocytopenia (platelet count 74,000/UL), elevated alanine and aspartate transaminases (ALT 331 U/L; AST 462 U/L) and elevated creatine phosphokinase (CPK 5885 U/L). Urine toxicology screening solely... (truncated)

Title: [Acid-base equilibrium and vascular exclusion of the liver: study of 30 extensive hepatectomies].
The acid-base disorders after hepatic vascular exclusion (HVE) were studied in 30 major liver resections. HVE included portal triad clamping and occlusion of the inferior vena cava below and above the liver, without venous shunt nor cooling. Clamping of the supra-coeliac abdominal aorta (AoC) was associated with HVE in 12 patients. HVE lasted 18 to 65 min (mean 37 min). Liver ischemia and splanchnic blood pooling resulted in metabolic acidosis and hyperlactatemia. In order to prevent his acidosis, prophylactic administration of NaHCO23 was used during the first 19 cases. This induced significant metabolic alkalosis during HVE and the early postoperative... (truncated)

Title: Metabolic alkalosis-an adverse effect of baking soda misuse: A case report and literature review.
Baking soda overdose is rarely reported. However, several cases have been previously documented, as baking soda has gained popularity as an over-the-counter remedy. The present study reported the case of a 69-year-old male patient hospitalized with metabolic alkalosis (pH 7.61; bicarbonate levels, 53.2 mEq/l), hypokalemia (K⁺ 2.6 mEq/l), acute kidney injury (serum creatinine level 4.02 mg/dl) and hepatic toxicity (alanine transaminase, 955 U/l; aspartate transaminase, 1,091 U/l) in the context of baking soda misuse as an alternative treatment for gout. The patient's past medical history included chronic uric acid nephropathy, gout, arterial hypertension and permanent atrial fibrillation. Under corrective treatment... (truncated)

Title: Interstitial ion homeostasis and acid-base balance are maintained in oedematous brain of mice with acute toxic liver failure.
Acute toxic liver failure (ATLF) rapidly leads to brain oedema and neurological decline. We evaluated the ability of ATLF-affected brain to control the ionic composition and acid-base balance of the interstitial fluid. ATLF was induced in 10-12 weeks old male C57Bl mice by single intraperitoneal (i.p.) injection of 100 μg/g azoxymethane (AOM). Analyses were carried out in cerebral cortex of precomatous mice 20-24 h after AOM administration. Brain fluid status was evaluated by measuring apparent diffusion coefficient [ADC] using NMR spectroscopy, Evans Blue extravasation, and accumulation of an intracisternally-injected fluorescent tracer. Extracellular pH ([pH]_e) and ([K⁺]_e) were measured in situ with ion-sensitive... (truncated)

MESH:D008614 - meperidine

Summary:

LLM Prediction Score	0.098 (normalized)
LLM Confidence Score	0.990
Golden Answer (Severity Class)	0.0 (normalized)
Prediction Error	0.098

Retrieved Context:

Title: The effects of novel α₂-adrenoreceptor agonist dexmedetomidine on shivering in patients underwent caesarean section.
Objective: Meperidine used to control shivering during perioperative period has associated side effects. The present study compared the safety of selective α2-adrenoreceptor agonist dexmedetomidine and meperidine for anti-shivering in primiparas after caesarean delivery under combined spinal-epidural anesthesia (CSEA).Methods: 100 primiparas scheduled for caesarean delivery were randomly allocated to dexmedetomidine group (Group D, <i>n</i>=50) and meperidine positive control group (Group M, <i>n</i>=50). Primiparas experienced shivering that continued to cord clamping were treated with dexmedetomidine (0.5 μg/kg) or meperidine (0.5 mg/kg) after cord clamping. The primary outcome measures were incidence of nausea, vomiting, and respiratory depression. Secondary outcome measures were shivering score, vital signs... (truncated)

Title: Analgesics in patients with hepatic impairment: pharmacology and clinical implications.
The physiological changes that accompany hepatic impairment alter drug disposition. Porto-systemic shunting might decrease the first-pass metabolism of a drug and lead to increased oral bioavailability of highly extracted drugs. Distribution can also be altered as a result of impaired production of drug-binding proteins or changes in body composition. Furthermore, the activity and capacity of hepatic drug metabolizing enzymes might be affected to various degrees in patients with chronic liver disease. These changes would result in increased concentrations and reduced plasma clearance of drugs, which is often difficult to predict. The pharmacology of analgesics is also altered in liver disease.... (truncated)

Title: Central nervous system toxicity associated with meperidine use in hepatic disease.
Meperidine-associated central nervous system (CNS) excitatory toxicities are believed to be caused by accumulation of the active metabolite normeperidine. Normeperidine is eliminated by the kidneys and accumulates in patients with renal insufficiency, sickle cell disease, and cancer. In patients with cirrhosis, the metabolism of meperidine is decreased, leading to accumulation of the parent drug and possible CNS depressive effects similar to hepatic encephalopathy. Although the elimination of normeperidine is decreased as well in these patients, the ratio of normeperidine to meperidine is generally low, and the narcotic effects of meperidine usually predominate. This is the first reported case of CNS... (truncated)

Title: A randomized prospective controlled study of the metabolism and hepatotoxicity of halothane in humans.
In a randomized prospective controlled study in humans, the metabolism and hepatic effects of a single administration of halothane were compared with enflurane and meperidine. Pre- and postoperative antipyrine pharmacokinetics, intraoperative indocyanine green clearance, liver histology, and postoperative liver function tests were determined in 24 patients undergoing abdominal surgery who were randomly allocated to receive either halothane (0.5%, group I), enflurane (0.8%, group II), or meperidine (group III) as a supplement to a common basal anesthetic regimen consisting of thiopental, nitrous oxide/oxygen/muscle relaxant. In addition, end-tidal concentrations of the volatile reductive metabolites of halothane, chlorodifluoroethylene (CDF), and chlorotrifluoroethane (CTF) were... (truncated)

Title: Acute neurotoxicity of meperidine in an infant.
We report a 6-week-old boy with meperidine neurotoxicity. What distinguished our patient from those previously reported was his minimal exposure to therapeutic doses of meperidine in the setting of normal renal function, and no history of sickle cell anemia, cancer, hepatitis, or cirrhosis. In addition, our patient had no abnormal changes in the electroencephalogram during the event. After only 2 doses of meperidine, he exhibited acute orofacial dyskinesias consisting of tongue thrusting, lip pursing, and facial grimacing combined with prominent flexion of the arms and stiffening of his legs. However, a normal sucking response remained. His symptoms resolved over the... (truncated)

MESH:D000078330 - oxcarbazepine

Summary:

LLM Prediction Score	0.472 (normalized)
LLM Confidence Score	0.980
Golden Answer (Severity Class)	0.375 (normalized)
Prediction Error	0.097

Retrieved Context:

Title: Oxcarbazepine versus carbamazepine in the treatment of paroxysmal kinesigenic dyskinesia.
Paroxysmal kinesigenic dyskinesia (PKD) is an uncommon neurological disorder, consisting of brief attacks of involuntary movements triggered by sudden action. Patients with PKD generally respond positively to antiepileptic drugs. We compared the efficacy and tolerability of oxcarbazepine (OXC) and carbamazepine (CBZ) in the treatment of PKD, in order to find the optimal prescription. This retrospective study reviewed monotherapy use of CBZ or OXC in 28 patients with PKD during 2005-2011, dividing into two groups. The frequency and severity of attacks and adverse events were recorded. Ten patients in the OXC group and 12 in the CBZ group continued the therapy... (truncated)

Title: [New antiepileptic drugs: a review and personal contribution].
In drug-resistant epilepsy the use of VGB, LTG, oxcarbazepine, FBM and GBP resulted in at least a 50% improvement in 20% to 60% of such patients treated and in 7% led to complete seizure control. In the long term, VGB may lose its efficacy and give rise to tolerance phenomena. Another frequent disadvantage of VGB is poor compliance owing to the large number of tablets needed to achieve the necessary dose (2-3 g). VGB may also induce a worsening of myoclonic epilepsies and seems ineffective in absences; whereas it can induce a good response in West's syndrome. LTG and FBM... (truncated)

Title: Acute oxcarbazepine-induced hepatotoxicity in a patient susceptible to developing drug-induced liver injury.
Oxcarbazepine (OXC) is generally accepted as a drug without risk of severe drug-induced hepatotoxicity, but according to recently reported pharmacovigilance data this statement has been challenged. However, in the literature there have been no reports of acute OXC-induced hepatotoxicity without systemic manifestations of Drug Rash with Eosinophilia and Systemic Symptoms (DRESS) syndrome. We present a female with seizures one month after delivery who had borderline elevated liver enzymes prior to the initiation of OXC treatment. Two weeks after introducing OXC, highly elevated liver enzymes were found. After discontinuation of OXC the enzymes continued to rise for another week, and afterward... (truncated)

Title: Use of antiepileptic drugs in hepatic and renal disease.
The use of antiepileptic drugs in patients with renal or hepatic disease is common in clinical practice. Since the liver and kidney are the main organs involved in the elimination of most drugs, their dysfunction can have important effects on the disposition of antiepileptic drugs. Renal or hepatic disease can prolong the elimination of the parent drug or an active metabolite leading to accumulation and clinical toxicity. It can also affect the protein binding, distribution, and metabolism of a drug. The protein binding of anionic acidic drugs, such as phenytoin and valproate, can be reduced significantly by renal failure, causing... (truncated)

Title: Choice and use of newer anticonvulsant drugs in older patients.
Epilepsy is common in the elderly. The incidence of epilepsy is age-dependent, with a peak during the first year of life and higher incidence in those older than 75 years. Cerebrovascular disease is a common cause of epilepsy in the elderly. Drug treatment of the elderly is a challenge because of pharmacokinetic changes with aging, including impaired drug protein binding or displacement of drug from protein binding sites, potentially causing drug toxicity as a result of increased free drug concentrations. With aging, hepatic mass and blood flow decline along with renal function. Established anticonvulsant drugs have adverse effects and drug... (truncated)

MESH:D000077284 - abciximab

Summary:

LLM Prediction Score	0.097 (normalized)
LLM Confidence Score	0.980
Golden Answer (Severity Class)	0.0 (normalized)
Prediction Error	0.097

Retrieved Context:

Title: Targeting integrin pathways: mechanisms and advances in therapy.
Integrins are considered the main cell-adhesion transmembrane receptors that play multifaceted roles as extracellular matrix (ECM)-cytoskeletal linkers and transducers in biochemical and mechanical signals between cells and their environment in a wide range of states in health and diseases. Integrin functions are dependable on a delicate balance between active and inactive status via multiple mechanisms, including protein-protein interactions, conformational changes, and trafficking. Due to their exposure on the cell surface and sensitivity to the molecular blockade, integrins have been investigated as pharmacological targets for nearly 40 years, but given the complexity of integrins and sometimes opposite characteristics, targeting integrin therapeutics... (truncated)

Title: The Contribution of Liver Sinusoidal Endothelial Cells to Clearance of Therapeutic Antibody.
Many chronic inflammatory diseases are treated by administration of "biological" therapies in terms of fully human and humanized monoclonal antibodies or Fc fusion proteins. These tools have widespread efficacy and are favored because they generally exhibit high specificity for target with a low toxicity. However, the design of clinically applicable humanized antibodies is complicated by the need to circumvent normal antibody clearance mechanisms to maintain therapeutic dosing, whilst avoiding development of off target antibody dependent cellular toxicity. Classically, professional phagocytic immune cells are responsible for scavenging and clearance of antibody <i>via</i> interactions with the Fc portion. Immune cells such as... (truncated)

Title: Computational Models Using Multiple Machine Learning Algorithms for Predicting Drug Hepatotoxicity with the DILIrank Dataset.
Drug-induced liver injury (DILI) remains one of the challenges in the safety profile of both authorized and candidate drugs, and predicting hepatotoxicity from the chemical structure of a substance remains a task worth pursuing. Such an approach is coherent with the current tendency for replacing non-clinical tests with in vitro or in silico alternatives. In 2016, a group of researchers from the FDA published an improved annotated list of drugs with respect to their DILI risk, constituting "the largest reference drug list ranked by the risk for developing drug-induced liver injury in humans" (DILIrank). This paper is one of the... (truncated)

Title: Adverse reactions to targeted and non-targeted chemotherapeutic drugs with emphasis on hypersensitivity responses and the invasive metastatic switch.
More than 100 drugs are used to treat the many different cancers. They can be divided into agents with relatively broad, non-targeted specificity and targeted drugs developed on the basis of a more refined understanding of individual cancers and directed at specific molecular targets on different cancer cells. Individual drugs in both groups have been classified on the basis of their mechanism of action in killing cancer cells. The targeted drugs include proteasome inhibitors, toxic chimeric proteins and signal transduction inhibitors such as tyrosine kinase (non-receptor and receptor), serine/threonine kinase, histone deacetylase and mammalian target of rapamycin inhibitors. Increasingly used... (truncated)

Title: Research on Liver Damage Caused by the Treatment of Rheumatoid Arthritis with Novel Biological Agents or Targeted Agents.
Rheumatoid arthritis (RA) is a chronic autoimmune disease characterized by polyarticular, symmetric, and aggressive inflammation of the small joints in the hands and feet, resulting in dysfunction. With progress and development in medicine, treatment of RA is constantly evolving, making several drugs available for the treatment of RA. From the nonsteroidal anti-inflammatory drugs (NSAIDs) at the start of illness to glucocorticoids and then to conventional synthetic DMARDs (csDMARDs), biologic DMARDs (bDMARDs), and targeted synthetic DMARDs (tsDMARDs), therapeutic-use drugs for RA have been keeping pace with scientific research. However, various types of drugs have additional side effects when used over the... (truncated)

MESH:D018967 - risperidone

Summary:

LLM Prediction Score	0.472 (normalized)
LLM Confidence Score	0.990
Golden Answer (Severity Class)	0.375 (normalized)
Prediction Error	0.097

Retrieved Context:

Title: [Hepatic tolerance of atypical antipsychotic drugs].
The strategy in the choice of antipsychotic agent must take into account the hepatic tolerance according to non-negligible incidence of liver disorders among psychiatric population (presence of risk factors like alcoholism, drugs of abuse intake, polymedication including potentially hepatotoxic drugs.). More than 1 000 drugs have been listed as being responsible of hepatic side effects; 16% of these agents were neuropsychiatric drugs. Antidepressive drugs (tricyclic agents or SSRI), mood stabilizing agents and neuroleptic drugs have been implicated in biological or/and clinical hepatotoxicity. For these reasons, some psychotropic agents have been withdrawn of the pharmaceutical market like alpidem or medifoxamine. Atrium*,... (truncated)

Title: Acute cholestatic hepatitis probably associated with risperidone.
Risperidone is an atypical neuroleptic drug widely used due to the lower incidence and severity of hepatic adverse effects in comparison to phenothiazines. Although idiosyncratic reversible hepatotoxicity may occur in association with risperidone, the interaction with fluoxetine might increase the risk of toxic liver injury in a vulnerable patient.

Title: [Disorder of liver functions in a schizophrenic patient after long-term risperidone treatment--case report].
We present a 51-year old man with an 18-year history of chronic paranoid schizophrenia. The last four years he was treated only with risperidone at a dose of 3 mg per day. He was admitted to hospital due to a psychotic decompensation. Jaundice and skin pruritus were also observed at admission. Laboratory tests revealed elevated liver enzymes. Cholestatic and viral hepatitis were excluded. Risperidone was changed to other antipsychotic drugs. After 7 weeks of treatment the patient was discharged with significant improvement of his mental condition. The liver function test results returned to normal values except for the slightly increased... (truncated)

Title: Liver enzyme abnormalities during antipsychotic treatment: a case report of risperidone-associated hepatotoxicity.
Drug-induced liver enzyme abnormalities may indicate hepatic injury. Antipsychotic drugs also may cause increase in the liver enzymes and serum bilirubin levels. The present report evaluates the case of a patient with risperidone-associated hepatocellular damage.

Title: Chronically administered risperidone did not change the number of hepatocytes in rats: a stereological and histopathological study.
Risperidone, an antipsychotic agent, has been used in the therapy of patients with acute and chronic schizophrenia. The strategy in the choice of antipsychotic agent should take into account the hepatic tolerance. The aim of the present study was to analyse whether or not the chronic administration of risperidone has a toxic effect on rat livers. Rats were divided into three groups. Low dose (n = 5) and high dose (n = 5) groups that were given 0.5 and 1 mg/kg intraperitoneal doses of risperidone daily for 6 weeks, respectively. The control group received distilled water as a vehicle. At... (truncated)

MESH:D008468 - meclizine

Summary:

LLM Prediction Score	0.096 (normalized)
LLM Confidence Score	0.980
Golden Answer (Severity Class)	0.0 (normalized)
Prediction Error	0.096

Retrieved Context:

Title: Meclizine is an agonist ligand for mouse constitutive androstane receptor (CAR) and an inverse agonist for human CAR.
The constitutive androstane receptor (CAR, NR1I3) is a key regulator of xenobiotic and endobiotic metabolism. The ligand-binding domains of murine (m) and human (h) CAR are divergent relative to other nuclear hormone receptors, resulting in species-specific differences in xenobiotic responses. Here we identify the widely used antiemetic meclizine (Antivert; Bonine) as both an agonist ligand for mCAR and an inverse agonist for hCAR. Meclizine increases mCAR transactivation in a dose-dependent manner. Like the mCAR agonist 1,4-bis[2-(3,5-dichloropyridyloxy)]benzene, meclizine stimulates binding of steroid receptor coactivator 1 to the murine receptor in vitro. Meclizine administration to mice increases expression of CAR target genes... (truncated)

Title: Meclizine Preconditioning Protects the Kidney Against Ischemia-Reperfusion Injury.
Global or local ischemia contributes to the pathogenesis of acute kidney injury (AKI). Currently there are no specific therapies to prevent AKI. Potentiation of glycolytic metabolism and attenuation of mitochondrial respiration may decrease cell injury and reduce reactive oxygen species generation from the mitochondria. Meclizine, an over-the-counter anti-nausea and -dizziness drug, was identified in a 'nutrient-sensitized' chemical screen. Pretreatment with 100 mg/kg of meclizine, 17 h prior to ischemia protected mice from IRI. Serum creatinine levels at 24 h after IRI were 0.13 ± 0.06 mg/dl (sham, n = 3), 1.59 ± 0.10 mg/dl (vehicle, n = 8) and 0.89... (truncated)

Title: A New Topical Candidate in Acne Treatment: Characterization of the Meclozine Hydrochloride as an Anti-Inflammatory Compound from In Vitro to a Preliminary Clinical Study.
Acne is a chronic inflammatory multifactorial disease involving the anaerobic bacterium Cutibacterium acnes (C. acnes). Current acne treatments are associated with adverse effects, limiting treatment compliance and use. We showed that meclozine, an anti-histaminic H1 compound, has anti-inflammatory properties. In Vitro, meclozine reduced the production of CXCL8/IL-8 and IL-1β mRNA and protein by C. acnes-stimulated human keratinocytes and monocytes. No cell toxicity was observed at the IC50. Meclozine prevented the phosphorylation of ERK and JNK. In Vivo, 1% meclozine gel significantly decreased C. acnes-mouse ear induced inflammation by 26.7% (p = 0.021). Ex vivo experiments on human skin explants showed... (truncated)

Title: The Connection of Azole Fungicides with Xeno-Sensing Nuclear Receptors, Drug Metabolism and Hepatotoxicity.
Azole fungicides, especially triazole compounds, are widely used in agriculture and as pharmaceuticals. For a considerable number of agricultural azole fungicides, the liver has been identified as the main target organ of toxicity. A number of previous studies points towards an important role of nuclear receptors such as the constitutive androstane receptor (CAR), the pregnane-X-receptor (PXR), or the aryl hydrocarbon receptor (AHR), within the molecular pathways leading to hepatotoxicity of these compounds. Nuclear receptor-mediated hepatic effects may comprise rather adaptive changes such as the induction of drug-metabolizing enzymes, to hepatocellular hypertrophy, histopathologically detectable fatty acid changes, proliferation of hepatocytes, and... (truncated)

Title: Dynamic imaging of adaptive stress response pathway activation for prediction of drug induced liver injury.
Drug-induced liver injury remains a concern during drug treatment and development. There is an urgent need for improved mechanistic understanding and prediction of DILI liabilities using in vitro approaches. We have established and characterized a panel of liver cell models containing mechanism-based fluorescent protein toxicity pathway reporters to quantitatively assess the dynamics of cellular stress response pathway activation at the single cell level using automated live cell imaging. We have systematically evaluated the application of four key adaptive stress pathway reporters for the prediction of DILI liability: SRXN1-GFP (oxidative stress), CHOP-GFP (ER stress/UPR response), p21 (p53-mediated DNA damage-related response) and... (truncated)

MESH:D008619 - mepivacaine

Summary:

LLM Prediction Score	0.096 (normalized)
LLM Confidence Score	0.990
Golden Answer (Severity Class)	0.0 (normalized)
Prediction Error	0.096

Retrieved Context:

Title: Influence of obstructive jaundice on pharmacodynamics of rocuronium.
Anesthetics are variable in patients with obstructive jaundice. The minimum alveolar concentration awake of desflurane is reduced in patients with obstructive jaundice, while it has no effect on pharmacodynamics and pharmacokinetics of propofol. In this study, we investigated the influence of obstructive jaundice on the pharmacodynamics and blood concentration of rocuronium.

Title: New formulations of local anaesthetics-part I.
Part 1 comments on the types of local anaesthetics (LAs); it provides a better understanding of the mechanisms of action of LAs, and their pharmacokinetics and toxicity. It reviews the newer LAs such as levobupivacaine, ropivacaine, and articaine, and examines the newer structurally different LAs. The addition of adjuvants such as adrenaline, bicarbonate, clonidine, and corticosteroids is explored. Comment is made on the delivery of topical LAs via bioadhesive plasters and gels and controlled-release local anaesthetic matrices. Encapulation matrices such as liposomes, microemulsions, microspheres and nanospheres, hydrogels and liquid polymers are discussed as well. New innovations pertaining to LA formulations... (truncated)

Title: Local anesthetic systemic toxicity: current perspectives.
Local anesthetic systemic toxicity (LAST) is a life-threatening adverse event that may occur after the administration of local anesthetic drugs through a variety of routes. Increasing use of local anesthetic techniques in various healthcare settings makes contemporary understanding of LAST highly relevant. Recent data have demonstrated that the underlying mechanisms of LAST are multifactorial, with diverse cellular effects in the central nervous system and cardiovascular system. Although neurological presentation is most common, LAST often presents atypically, and one-fifth of the reported cases present with isolated cardiovascular disturbance. There are several risk factors that are associated with the drug used and... (truncated)

Title: Lipid emulsion for local anesthetic systemic toxicity.
The accidental overdose of local anesthetics may prove fatal. The commonly used amide local anesthetics have varying adverse effects on the myocardium, and beyond a certain dose all are capable of causing death. Local anesthetics are the most frequently used drugs amongst anesthetists and although uncommon, local anaesthetic systemic toxicity accounts for a high proportion of mortality, with local anaesthetic-induced cardiac arrest particularly resistant to standard resuscitation methods. Over the last decade, there has been convincing evidence of intravenous lipid emulsions as a rescue in local anesthetic-cardiotoxicity, and anesthetic organisations, over the globe have developed guidelines on the use of... (truncated)

Title: Should we consider the infusion of lipid emulsion in the resuscitation of poisoned patients?
The use of intravenous lipid emulsions (ILEs) as antidote in local anaesthetic systemic toxicity has gained widespread support following convincing data from animal models, and successful case reports in humans. Proposed beneficial mechanisms of action for ILEs include intravascular sequestration of intoxicant and subsequent enhanced redistribution to biologically inert tissues, augmentation of fatty acid utilisation for ATP synthesis in the context of metabolic poisoning, and direct cardiotonic and ion channel effects. The evidence base for use of ILEs in acute drug intoxication is evolving. The present evidence supports use of ILEs only in local anaesthetic systemic toxicity and in lipophilic... (truncated)

MESH:D013792 - thalidomide

Summary:

LLM Prediction Score	0.595 (normalized)
LLM Confidence Score	0.990
Golden Answer (Severity Class)	0.5 (normalized)
Prediction Error	0.095

Retrieved Context:

Title: Thalidomide-associated hepatitis: a case report.
We report a case of hepatitis in a 58-year-old woman being treated with thalidomide for end-stage plasma cell leukemia. The patient had a medical history including chronic stable hepatitis C infection. At diagnosis there was a severe anemia, thrombocytopenia, hypercalcemia, IgG paraproteinemia, peripheral blood myeloma cells, and a marrow plasmacytosis with lytic bony lesion. The disease was refractory to standard chemotherapy, and she was treated with oral thalidomide. Within 1 week she became jaundiced and developed a marked transaminitis. This promptly resolved upon cessation of thalidomide alone. Thalidomide has recently enjoyed renewed interest as a treatment in many disorders, including... (truncated)

Title: [Thalidomide. Clinical trials in cancer].
The breakthrough discovery of the anti-angiogenic effects of thalidomide gave impetus to a series of clinical trials in patients with solid tumors and hematologic malignancies. Thalidomide has been shown to block the activity of angiogenic substances like bFGF, VEGF and interleukin 6. This drug also down-regulates TNF alpha. Thalidomide has shown clinical antitumor activity in single-agent, phase II clinical trials in AIDS-related Kaposi sarcoma, glioma, multiple myeloma refractory to chemotherapy, and hormone-refractory prostate cancer. In contrast, thalidomide was inactive in breast, lung and kidney cancer. The dose-limiting toxicity of thalidomide is sedation. Other adverse effects include skin rash, constipation, dry... (truncated)

Title: Thalidomide-induced severe hepatotoxicity.
Thalidomide is a relatively safe and efficacious form of therapy in the treatment of advanced, refractory multiple myeloma. Hepatotoxicity is listed as an extremely rare adverse effect associated with its use. We describe a 76-year-old woman with multiple myeloma who was treated with dexamethasone and thalidomide. By week 6 of therapy, she had developed acute increases in her aspartate aminotransferase (AST) and alanine aminotransferase (ALT) levels to more than 50 times the upper limit of normal. Her liver function test results had been within the normal ranges before and immediately after the start of therapy, and the patient had no... (truncated)

Title: A Case of Drug-Induced Hepatitis due to Lenalidomide.
Lenalidomide is a recent thalidomide analog used for the treatment of refractory multiple myeloma. The main toxicity of this drug consists in severe neutropenia and thrombocytopenia. Lenalidomide-associated liver injury is rare, manifesting itself as elevated liver enzymes and hyperbilirubinemia reversible upon weeks after drug withdrawal. We report here in detail the clinical course as well as the biological and histological alterations of an acute lenalidomide-induced liver injury. Findings on liver biopsy allowed us to discriminate acute inflammatory changes due to the drug and minor associated lesions of graft-versus-host disease in this patient with recurrent myeloma after allogeneic bone marrow transplantation.

Title: Acute exacerbation of chronic hepatitis B during thalidomide therapy for multiple myeloma: a case report.
We report a case of acute fatal exacerbation of chronic hepatitis B in a 50-year-old man with multiple myeloma being treated with thalidomide. The patient had a medical history of chronic hepatitis B and was diagnosed with stage IIIA multiple myeloma. He suffered two episodes of transient transaminitis of unknown origin after successive autologous stem cell transplantations. Spontaneous resolutions of the transaminitis were observed without special management. At that time, PCR of hepatitis B virus (HBV) were all-negative. After 5-months' administration of thalidomide for the second relapse of the multiple myeloma, he suddenly experienced dizziness and jaundice. The level of... (truncated)

MESH:D020913 - perindopril

Summary:

LLM Prediction Score	0.280 (normalized)
LLM Confidence Score	0.980
Golden Answer (Severity Class)	0.375 (normalized)
Prediction Error	0.095

Retrieved Context:

Title: [Liver damage in a patient treated with a vitamin K antagonist, a statin and an ACE inhibitor].
We report the case of a 71-year-old male patient who presented at the emergency room with episodes of epistaxis and jaundice. The patient was on therapy with phenprocoumon, atorvastatin and perindopril. Findings on admission included prominent elevation of transaminases and bilirubin and a high INR due to impaired liver function and oral anticoagulation. After exclusion of other causes like viral or autoimmune hepatitis and after having obtained a liver biopsy, a diagnosis of drug induced liver damage (DILI) was made. Epidemiology, pathophysiology and clinical signs of DILI are discussed with a special focus on coumarines, statins and ACE-inhibitors.

Title: Angiotensin-I-converting enzyme inhibitors may be an alternative anti-angiogenic strategy in the treatment of liver fibrosis and hepatocellular carcinoma. Possible role of vascular endothelial growth factor.
The renin-angiotensin system (RAS) is frequently activated in patients with chronic liver diseases. Angiotensin-II (AT-II), which is produced by angiotensin-converting enzyme (ACE), has many physiological effects, including strong pro-angiogenic activity. AT-II induces the potent angiogenic factor, vascular endothelial growth factor (VEGF). Recent studies have revealed that angiogenesis is an essential process in many pathological events, such as tumor growth including hepatocellular carcinoma (HCC), and even in liver fibrogenesis. ACE inhibitors are currently widely used as anti-hypertensive agents in clinical practice. Studies have found that the ACE inhibitor, perindopril (PE), which is a potent inhibitor of experimental HCC growth and angiogenesis,... (truncated)

Title: Perindopril sensitizes hepatocellular carcinoma to chemotherapy: A possible role of leptin / Wnt/ β-catenin axis with subsequent inhibition of liver cancer stem cells.
Hepatocellular carcinoma (HCC) is one of the leading causes of cancer-related death. The major challenge in managing HCC is the resistance to chemotherapy. Leptin hormone is associated with different oncogenic pathways implicated in drug resistance. Angiotensin II was found to decrease the production and secretion of leptin.

Title: Salvianolic Acid B Attenuates Rat Hepatic Fibrosis via Downregulating Angiotensin II Signaling.
The renin-angiotensin system (RAS) plays an important role in hepatic fibrosis. Salvianolic acid B (Sal B), one of the water-soluble components from Radix Salviae miltiorrhizae, has been used to treat hepatic fibrosis, but it is still not clear whether the effect of Sal B is related to angiotensin II (Ang II) signaling pathway. In the present study, we studied Sal B effect on rat liver fibrosis and Ang-II related signaling mediators in dimethylnitrosamine-(DMN-) induced rat fibrotic model in vivo and Ang-II stimulated hepatic stellate cells (HSCs) in vitro, with perindopril or losartan as control drug, respectively. The results showed that... (truncated)

Title: Perindopril, fosinopril and losartan inhibited the progression of diethylnitrosamine-induced hepatocellular carcinoma in mice via the inactivation of nuclear transcription factor kappa-B.
Hepatocellular carcinoma (HCC) is a major global health problem. Therapeutic interventions of HCC are still limited because of its complicated molecular pathogenesis. Many reports showed that renin-angiotensin system (RAS) contributes to the development of different types of malignancies. Therefore, the present study aimed to examine the effect of RAS inhibition using perindopril (1 mg/kg), fosinopril (2 mg/kg), or losartan (10 mg/kg) on diethylnitrosamine-induced HCC compared to sorafenib (30 mg/kg). The administration of RAS inhibitors resulted in improved liver function and histologic picture with a reduction in AFP levels. These effects found to be mediated through inactivation of NFκB pathway by... (truncated)

MESH:C051752 - nefazodone

Summary:

LLM Prediction Score	0.906 (normalized)
LLM Confidence Score	0.970
Golden Answer (Severity Class)	1.0 (normalized)
Prediction Error	0.094

Retrieved Context:

Title: Nefazodone-induced liver failure: report of three cases.
Liver failure is a rare but devastating result of drug toxicity.

Title: Endoplasmic Reticulum Stress Induction and ERK1/2 Activation Contribute to Nefazodone-Induced Toxicity in Hepatic Cells.
Nefazodone, an antagonist for the 5-hydroxytryptanine receptor, has been used for the treatment of depression. Acute liver injury has been documented to be associated with the use of nefazodone; however, the mechanisms of nefazodone-induced liver toxicity are not well defined. In this report, using biochemical and molecular analyses, we characterized the molecular mechanisms underlying the hepatotoxicity of nefazodone. We found that nefazodone induced endoplasmic reticulum (ER) stress in HepG2 cells, as the expression of typical ER stress markers, including CHOP, ATF-4, and p-eIF2α, was significantly increased, and splicing of XBP1 was observed. Nefazodone-suppressed protein secretion was evaluated using a Gaussia... (truncated)

Title: Liver injury associated with antidepressants.
Antidepressants are commonly prescribed and used in the management of depression, anxiety disorders, and other psychiatric illnesses. Antidepressants used in therapeutic dosing ranges are associated with causing several adverse drug reactions including hepatotoxicity. Paroxetine, fluoxetine, fluvoxamine, citalopram, mirtazapine and venlafaxine are associated with reversible liver injury upon discontinuation of the agent. Patient cases of hepatotoxicity involving the use of nefazodone, trazodone, duloxetine, bupropion, and sertraline are linked to causing death in its users. Due to the idiosyncratic nature of hepatotoxicity, monitoring of liver function tests and immediate discontinuation upon abnormal lab findings or signs and symptoms of liver dysfunction are... (truncated)

Title: [Acute liver failure ascribed to nefazodone: importance of 'postmarketing surveillance' for recently introduced drugs].
A 50-year-old man developed acute liver failure 7 months after nefazodone treatment was initiated. There was no evidence of any aetiology apart from the exposure to the antidepressant drug nefazodone, while the results of repeated histological examinations of the liver were compatible with serious progressive drug-induced hepatitis. This diagnosis was initially disregarded because in the literature no patients with nefazodone-induced hepatitis were reported. However, the drug had only recently been introduced, which meant that relatively infrequent adverse drug reactions might not have been published. Further analyses of databases of the marketing pharmaceutical industry and of the World Health Organization revealed... (truncated)

Title: Hepatic adverse reactions associated with nefazodone.
Since 1999, international reports of hepatotoxicity associated with the antidepressant nefazodone (Serzone) have increased. In June 2001, a manufacturer's safety advisory notified Canadian physicians of "very rare reports of severe liver injury temporally associated with the use of nefazodone HC1." We undertook this study to determine the prevalence of adverse drug reactions to nefazodone reported in a Canadian database.

MESH:D004113 - succimer

Summary:

LLM Prediction Score	0.156 (normalized)
LLM Confidence Score	0.970
Golden Answer (Severity Class)	0.25 (normalized)
Prediction Error	0.094

Retrieved Context:

Title: [Acute copper poisoning by suicidal attempt].
Voluntary copper poisoning is a rare mode of suicide. We report a case of patient admitted to Metabolic Intensive Care Unit who took fatal dose of copper oxichloride. In his clinical picture dominated gastrointestinal discomfort with the profused diarrhoea leading to the excessive fluids loss exceeding 10 liters over 24 hours. The renal and liver damages typical for copper intoxication were prevented due to the early intensive treatment (2 hours after ingestion of copper oxichlorid) including the application of copper chelator Succicaptal.

Title: Severe Abdominal Pain Caused by Lead Toxicity without Response to Oral Chelators: A Case Report.
A 19-year-old woman was referred to the Emergency Surgery Department with severe abdominal pain, icterus, and anemia. The patient's clinical and paraclinical findings in addition to her occupational and social history, convinced us to assay blood lead level (BLL), which was 41/5 µg/dL. Therefore toxicology consult was performed to treat lead toxicity. Recheck of the BLL showed the level as 53/7 µg/dL. So oral chelator with succimer was started. Despite consumption of oral chelator, there was no response and the pain continued. Because our repeated evaluations were negative, we decided to re-treat lead poisoning by intravenous and intramuscular chelators. Dimercaprol... (truncated)

Title: A comparison of sodium calcium edetate (edetate calcium disodium) and succimer (DMSA) in the treatment of inorganic lead poisoning.
This article reviews the experimental and clinical studies that have compared the efficacy (impact on urine lead excretion, blood and tissue lead concentrations, resolution of features and survival) of sodium calcium edetate (edetate calcium disodium) and succimer (DMSA) in the treatment of inorganic lead poisoning. It also summarizes the pharmacokinetic and pharmacodynamic aspects and the adverse effects of treatment.

Title: N-acetylcysteine and meso-2,3-dimercaptosuccinic acid alleviate oxidative stress and hepatic dysfunction induced by sodium arsenite in male rats.
Environmental exposure to arsenic represents a serious challenge to humans and other animals. The aim of the present study was to test the protective effect of antioxidant N-acetylcysteine (NAC) either individually or in combination with a chelating agent, meso-2,3-dimercaptosuccinic acid (DMSA), against sodium arsenite oral toxicity in male rats. Five groups were used: control; arsenic group (orally administrated in a concentration of 2 mg/kg body weight [b.w.]); the other three groups were orally administrated sodium arsenite in a concentration of 2 mg/kg b.w. followed by either NAC (10 mg/kg b.w., intraperitoneally [i.p.]), DMSA (50 mg/kg b.w., i.p.) or NAC plus... (truncated)

Title: Embracing the Dark Side: Computational Approaches to Unveil the Functionality of Genes Lacking Biological Annotation in Drug-Induced Liver Injury.
In toxicogenomics, functional annotation is an important step to gain additional insights into genes with aberrant expression that drive pathophysiological mechanisms. Nevertheless, there exists a gap on annotation of these genes which often hampers the interpretation of results and limits their applicability in translational medicine. In this study, we evaluated the coverage of functional annotations of differentially expressed genes (DEGs) induced by 10 selected compounds from the TG-GATEs database identified as high- or no-risk in causing drug-induced liver injury (most-DILI or no-DILI, respectively) using <i>in vitro</i> human data. Functional roles of DEGs not present in the most common biological annotation... (truncated)

MESH:D008755 - methylergonovine

Summary:

LLM Prediction Score	0.094 (normalized)
LLM Confidence Score	0.910
Golden Answer (Severity Class)	0.0 (normalized)
Prediction Error	0.094

Retrieved Context:

Title: Acute fatty liver of pregnancy complicated by coagulopathy: A case report.
We present the case of a rare obstetric emergency, which is usually fatal. Our case highlights suspicion of AFLP in patients presenting with jaundice in the third trimester with good maternal and fetal outcomes after a timely intervention.

Title: Era of direct acting anti-viral agents for the treatment of hepatitis C.
Hepatitis C infection is universal and the most common indication of liver transplantation in the United States. The period of less effective interferon therapy with intolerable side effects has gone. Now we have stepped into the era of direct acting anti-viral agents (DAAs) against hepatitis C virus. Treatment of hepatitis C is now extremely effective, tolerable and requires a short duration of intake of oral agents. Less monitoring is required with the current therapy and drug-drug interactions are less than the previous regimen. The current treatment options of chronic hepatitis C with various DAAs are discussed in this article.

Title: Association between forage mycotoxins and liver disease in horses.
Outbreaks of liver disease in horses are common but the etiology of most remains unknown. Forage mycotoxins have been suspected to be a cause.

Title: Medication use and medical comorbidity in patients with chronic hepatitis C from a US commercial claims database: high utilization of drugs with interaction potential.
With the advent of the direct-acting antiviral agents, significant drug-drug interaction (DDI) potential now exists for patients treated for chronic hepatitis C virus (HCV) infection. However, little is known about how often patients with HCV infection use medications that may interact with newer HCV treatments, especially those with cytochrome P450 3A (CYP3A) DDI potential.

Title: Prevalence of Contraindications to Nirmatrelvir-Ritonavir Among Hospitalized Patients With COVID-19 at Risk for Progression to Severe Disease.
No abstract available.

MESH:D005485 - flutamide

Summary:

LLM Prediction Score	0.906 (normalized)
LLM Confidence Score	0.970
Golden Answer (Severity Class)	1.0 (normalized)
Prediction Error	0.094

Retrieved Context:

Title: Flutamide-induced liver injury: a case report.
Flutamide is a nonsteroidal antiandrogen agent. Since it was marketed in February of 1989 in the USA for treatment of prostate cancer, its potential for hepatotoxicity has been reported in Western countries. Here we report the case of a 72-year-old patient who suffered from general malaise, poor appetite, nausea and jaundice after six months of flutamide therapy for the treatment of prostate cancer. He had no past history of liver disease and was not receiving other medications. Liver biochemistries revealed elevated serum alanine aminotransferase and aspartate aminotransferase concentrations of up to 1,035 U/l and 745 U/l, respectively. Serum total bilirubin... (truncated)

Title: Flutamide-induced hepatotoxicity with possible potentiation by simvastatin.
We report a 65 years old male who presented with jaundice in February 2000. He was operated for prostate carcinoma and started on flutamide in May 1999. He developed anorexia in mid January and frank jaundice by end February. LFT showed cholestatic picture. Liver biopsy showed cholestasis. Flutamide was stopped and patient made slow recovery. Possible additional hepatotoxicity of simvastatin is discussed.

Title: Hepatic failure with flutamide.
A 41-year-old woman, who was on flutamide for hair loss for 3 months, presented with deep jaundice. She developed hepatic encephalopathy but gradually recovered after discontinuing flutamide. Flutamide can cause fatal toxic liver injury and hence should be used with close monitoring of liver profile.

Title: Flutamide-induced liver failure.
Two cases of clinically manifest liver failure in patients with metastatic prostatic cancer treated with the non-steroidal antiandrogen flutamide combined with a luteinizing hormone-releasing hormone analogue are described. One patient developed severe hepatic insufficiency with jaundice, ascites and hepatic coma. The condition reversed after discontinuation of flutamide. The other patient only became jaundiced with a biochemical cholestatic pattern. Previous reports of hepatic adverse reactions only include elevated transaminase levels without clinical manifestations. The morphological lesions in the liver biopsy and the clinical condition may be due to a reversible interaction with metabolic processes in the hepatocytes.

Title: A case of flutamide-induced acute cholestatic hepatitis--a case report.
Flutamide, an oral nonsteroidal, antiandrogenic, anilid compound which inhibits the uptake and binding of androgens to nuclear receptors in the prostate, is used with or without LH-RH analogues for treatment of patients with metastatic carcinoma of the prostate. Clinically significant hepatotoxicities such as toxic hepatitis, cholestatic hepatitis, hepatic failure, and even death have rarely been reported in the English literature, but no case has been reported in Korea. A 75-year-old man with metastatic carcinoma of the prostate had taken flutamide (750 mg/day) for 7 months and suddenly developed jaundice and general weakness. The findings of blood chemistries were compatible with... (truncated)

MESH:C417083 - peginterferon alfa-2b

Summary:

LLM Prediction Score	0.406 (normalized)
LLM Confidence Score	0.970
Golden Answer (Severity Class)	0.5 (normalized)
Prediction Error	0.094

Retrieved Context:

Title: [Chronic hepatitis C presenting with hepatic involvement by chronic lymphocytic leukemia responding to polyethylene glycol interferon-α-2b].
Hepatic involvement by chronic lymphocytic leukemia(CLL)is common, but rarely presents with liver injury. We report a case of chronic hepatitis C(CH-C)in a patient who had suffered from liver injury as a result of hepatic involvement by CLL. A 74-year-old man was hospitalized because of an examination confirming him positive for leukocytosis. Computer tomography scan showed mild hepatosplenomegaly, lymph node swelling of the neck and axilla, and abdominal lymphadenopathy. He was diagnosed as CLL by bone marrow examination. His laboratory data revealed hepatis C virus(HCV)antibody-positive, and elevated levels of both aminotransferase and HCV-RNA. Liver biopsy demonstrated significant CLL involvement of portal... (truncated)

Title: Pneumonitis as a consequence of (peg)interferon-ribavirin combination therapy for hepatitis C: a review of the literature.
Combination of peginterferon and ribavirin is the current therapy for chronic hepatitis C infection (HCV). Interstitial pneumonitis is a rare side-effect of HCV therapy and is an important cause of dose reduction or discontinuation, impairing success of antiviral therapy. We performed a review of the literature in order to present diagnostic modalities and possible treatments for pneumonitis and to offer guidelines. We searched for cases where pneumonitis as a side-effect of HCV treatment was documented. First we performed a literature search via PubMed and Web of Science interface and second we searched three drug toxicity databases. We systematically analyzed all... (truncated)

Title: Peginterferon still has a place in the treatment of hepatitis C caused by genotype 3 virus.
Despite recent advances in therapy for chronic hepatitis C (CHC), the disease caused by genotype 3 virus (GEN3) is still considered a treatment challenge in certain patient subgroups. The aim of this retrospective study was to evaluate the effectiveness and safety of the peginterferon (Peg-IFN) and ribavirin (RBV) combination treatment for GEN3/CHC patients, and to evaluate sustained virological response (SVR) indicators and early treatment interruption due to serious adverse events (SAE). This was a retrospective observational study of GEN3/CHC patients, co-infected or not by HIV and treated with Peg-IFN/RBV in nine Brazilian healthcare centers. The study sample included 184 GEN3/CHC... (truncated)

Title: PEG-interferon in acute and chronic hepatitis C: a review.
In patients affected by chronic hepatitis, prevention of hepatitis C virus (HCV) infection complications can be achieved by antiviral therapy based on the use of a combination of pegylated interferon (PEG-IFN) alfa-2b and ribavirin, which yields a sustained eradication of infection in 40% to 50% of cases. The aim of this review is to evaluate the efficacy of PEG-IFN alpha in the treatment of acute and chronic HCV-related hepatitis. In patients affected by acute hepatitis C, the treatment is represented by PEG-IFN alpha-2b monotherapy, and combination therapy with ribavirin produces a similar response to that with interferon monotherapy. Treatment must... (truncated)

Title: [Side-effects of pegylated interferon plus ribavirin therapy with or without protease inhibitor direct acting antiviral agents during treatment of chronic hepatitis C virus infection].
Hepatitis C virus (HCV) infection affects 2-3% of the population, approximately 170 million people worldwide, causing chronic HCV-related hepatitis with subsequent liver cirrhosis, hepatic failure, hepatocellular cancer, and liver-related mortality in a large number of patients. The gold standard therapy, pegylated interferon alpha in combination with ribavirin can eradicate hepatitis C virus infection in approx. 40% of treatment-naïve patients infected with HCV genotype G1, and only 15-20% of patients with previous treatment. Success rate is substantially improved with the development and registration of two direct acting anti-hepatitis C virus protease inhibitors (boceprevir and telaprevir) in the second decade of 21st... (truncated)

MESH:D011342 - procainamide

Summary:

LLM Prediction Score	0.407 (normalized)
LLM Confidence Score	0.980
Golden Answer (Severity Class)	0.5 (normalized)
Prediction Error	0.093

Retrieved Context:

Title: Clinical consequences of polymorphic acetylation of basic drugs.
The clinical consequences (therapeutic and toxic) of drug acetylation polymorphism are reviewed for procainamide, hydralazine, phenelzine, isoniazid, and salicylazosulfapyridine. Genetic slow acetylators are more likely than rapid acetylators to experience the following adverse drug reactions: (1) earlier development of procainamide-induced antinuclear antibody; (2) earlier and more frequent development of procainamide-induced systemic lupus erythematosus (SLE); (3) hydralazine-induced SLE; (4) spontaneous SLE; (5) drowsiness and nausea from phenelzine; (6) cyanosis, hemolysis, and transient reticulocytosis from salicylazosulfapyridine; and (7) polyneuropathy after isoniazid therapy. The incidence of isoniazid hepatitis may, however, be more common in rapid than than in slow acetylators. Genetic slow acetylators... (truncated)

Title: Idiosyncratic NSAID drug induced oxidative stress.
Many idiosyncratic non-steroidal anti-inflammatory drugs (NSAIDs) cause GI, liver and bone marrow toxicity in some patients which results in GI bleeding/ulceration/fulminant hepatic failure/hepatitis or agranulocytosis/aplastic anemia. The toxic mechanisms proposed have been reviewed. Evidence is presented showing that idiosyncratic NSAID drugs form prooxidant radicals when metabolised by peroxidases known to be present in these tissues. Thus GSH, NADH and/or ascorbate were cooxidised by catalytic amounts of NSAIDs and hydrogen peroxide in the presence of peroxidase. During GSH and NADH cooxidation, oxygen uptake and activation occurred. Furthermore the formation of NSAID oxidation products was prevented during the cooxidation indicating that the... (truncated)

Title: Poisoning due to class IA antiarrhythmic drugs. Quinidine, procainamide and disopyramide.
Quinidine, procainamide and disopyramide are antiarrhythmic drugs in the class 1A category. These drugs have a low toxic to therapeutic ratio, and their use is associated with a number of serious adverse effects during long term therapy and life-threatening sequelae following acute overdose. Class 1A agents inhibit the fast inward sodium current and decrease the maximum rate of rise and amplitude of the cardiac action potential. Prolonged Q-T interval and, to a lesser extent, QRS duration may be observed at therapeutic concentrations of quinidine. With increasing plasma concentrations, progressive depression of automaticity and conduction velocity occur. 'Quinidine syncope' (a transient... (truncated)

Title: Reactive metabolites and adverse drug reactions: clinical considerations.
Idiosyncratic reactions can affect many different organ systems, either as -an isolated event (e.g., hepatitis) or as part of a syndrome (e.g., drug hypersensitivity syndrome). Formation of reactive metabolites of drugs in conjunction with a decreased ability for detoxification is believed to be the initiating step in many idiosyncratic reactions. The drug hypersensitivity syndrome, defined by the presence of fever, rash and internal organ involvement, is estimated to occur in approx 1 in 1000 to 1 in 10,000 exposures with drugs such as anticonvulsants sulfonamide antibiotics, allopurinol, and dapsone. Symptoms usually start within 2-8 wk of drug initiation. Serum sicknesslike... (truncated)

Title: Possible case of procainamide-induced intrahepatic cholestatic jaundice.
To describe a possible case of procainamide-induced intrahepatic cholestatic jaundice that was recognized six weeks after the initiation of procainamide therapy and to summarize the five previously reported cases.

MESH:D004111 - dimenhydrinate

Summary:

LLM Prediction Score	0.093 (normalized)
LLM Confidence Score	0.990
Golden Answer (Severity Class)	0.0 (normalized)
Prediction Error	0.093

Retrieved Context:

Title: Focus on Over-the-Counter Drugs' Misuse: A Systematic Review on Antihistamines, Cough Medicines, and Decongestants.
Background: Over the past 20 years or so, the drug misuse scenario has seen the emergence of both prescription-only and over-the-counter (OTC) medications being reported as ingested for recreational purposes. OTC drugs such as antihistamines, cough/cold medications, and decongestants are reportedly the most popular in being diverted and misused. Objective: While the current related knowledge is limited, the aim here was to examine the published clinical data on OTC misuse, focusing on antihistamines (e.g., diphenhydramine, promethazine, chlorpheniramine, and dimenhydrinate), dextromethorphan (DXM)- and codeine-based cough medicines, and the nasal decongestant pseudoephedrine. Methods: A systematic literature review was carried out with the... (truncated)

Title: Jaundice Caused by Hyperemesis Gravidarum.
Background: Hyperemesis gravidarum is characterized by intractable vomiting and associated with weight loss exceeding 5% of prepregnancy body weight, dehydration, and ketosis. Hyperemesis gravidarum occurs during the first trimester and typically resolves by 16 to 20 weeks of gestation. Approximately half of all hospitalized females with hyperemesis gravidarum have a mild elevation in liver enzymes; however, jaundice and hepatic synthetic dysfunction are uncommon. Case Report: A 22-year-old gravida 1 para 0 in her ninth week with a singleton gestation was hospitalized with persistent nausea, vomiting, weight loss of 11% of her prepregnancy body weight, dehydration, hypokalemia, and jaundice. Liver function... (truncated)

Title: A case of diphenhydramine intoxication showing prolonged false positive tricyclic antidepressant in the urine assay.
The urine immunochromatographic assay is a useful screening tool for patients suspected of acute drug intoxication in emergency conditions. Diphenhydramine intoxication shows symptoms similar to those of tricyclic antidepressant (TCA) intoxication.

Title: <i>Zingiber officinale</i> Rosc. in the Treatment of Metabolic Syndrome Disorders-A Review of In Vivo Studies.
Inflammation is a protective reaction of the innate immune system as a response to imbalances caused by a specific stimulus, a disease or a pathogen. A prolonged inflammatory condition may lead to the development of metabolic syndrome, which affects more than one-fourth of the world's population. This condition leads to the development of multi-organ disorders based on disrupted blood lipid and sugar levels, hypertension and oxidative stress. The review aims to present <i>Zingiber officinale</i> Rosc. as a plant that exhibits a variety of healing properties and restores the organism's equilibrium. Ginger (GI) rhizomes have been commonly used in traditional medicine... (truncated)

Title: Silymarin as a Natural Antioxidant: An Overview of the Current Evidence and Perspectives.
Silymarin (SM), an extract from the Silybum marianum (milk thistle) plant containing various flavonolignans (with silybin being the major one), has received a tremendous amount of attention over the last decade as a herbal remedy for liver treatment. In many cases, the antioxidant properties of SM are considered to be responsible for its protective actions. Possible antioxidant mechanisms of SM are evaluated in this review. (1) Direct scavenging free radicals and chelating free Fe and Cu are mainly effective in the gut. (2) Preventing free radical formation by inhibiting specific ROS-producing enzymes, or improving an integrity of mitochondria in stress... (truncated)

MESH:D011736 - pyridoxine

Summary:

LLM Prediction Score	0.093 (normalized)
LLM Confidence Score	0.990
Golden Answer (Severity Class)	0.0 (normalized)
Prediction Error	0.093

Retrieved Context:

Title: Paracetamol toxicity in classic homocystinuria: Effect of <i>N</i>-acetylcysteine on total homocysteine.
Classical homocystinuria (HCU) is caused by cystathionine β-synthase deficiency leading to impaired homocysteine transsulfuration and accumulation of homocysteine and methionine. Patients present with a wide spectrum of manifestations including ocular, skeletal, neuropsychiatric, and vascular manifestations. We report a 48-year-old female with pyridoxine-unresponsive HCU treated with betaine, cyanocobalamin, and folate. Her diet was non-restricted due to intolerance of low-methionine diet. She was admitted to hospital following a fall, with multiple fractures and subsequently developed acute liver failure with encephalopathy. Shock, sepsis, and liver ischaemia/thrombosis were excluded. In the context of glutathione depletion expected in HCU, hepatic dysfunction was presumed to be... (truncated)

Title: [The therapeutic effect of metadoxine on alcoholic and non-alcoholic steatohepatitis].
The therapeutic effect of metadoxine on alcoholic and non-alcoholic steatohepatitis. Alcohol-induced liver disease is one of the main epidemic problems of nowadays. Non-alcoholic steatohepatitis has been described only for some years, but it also needs much more attention in the future. The etiologic factors of both entities are quite different, but the pathologic changes of the liver are nearly the same, therefore there could be certain drugs, that are equally effective in their therapy. Metadoxine is one of them, mainly because its protective effect against the damage done by free radicals. Metadoxine is a pyridoxine-pyrrolidone carboxylate with significant scavenging property.... (truncated)

Title: Vitamin B-6-Induced Neuropathy: Exploring the Mechanisms of Pyridoxine Toxicity.
Vitamin B-6 in the form of pyridoxine (PN) is commonly used by the general population. The use of PN-containing supplements has gained lots of attention over the past years as they have been related to the development of peripheral neuropathy. In light of this, the number of reported cases of adverse health effects due to the use of vitamin B-6 have increased. Despite a long history of study, the pathogenic mechanisms associated with PN toxicity remain elusive. Therefore, the present review is focused on investigating the mechanistic link between PN supplementation and sensory peripheral neuropathy. Excessive PN intake induces neuropathy... (truncated)

Title: Concentration of vitamin B6 and activities of enzymes of B6 metabolism in the blood of alcoholic and nonalcoholic men.
The purpose of this study was to compare concentrations of vitamin B6 compounds and the activities of enzymes that synthesize or catabolize pyridoxal 5'-phosphate in the plasma and erythrocytes of nonalcoholic and alcoholic subjects. Blood was obtained from male nonalcoholics and chronic alcoholics with minimal liver damage and normal hematology. Plasma, erythrocyte, and urinary B6 compounds were analyzed by high performance liquid chromatography, and pyridoxal phosphate was also measured enzymatically. Erythrocyte pyridoxine kinase and pyridoxine phosphate oxidase and erythrocyte and plasma pyridoxine phosphate phosphatases were assayed. Plasma pyridoxal phosphate concentration was significantly lower in the alcoholics (31.3 +/- 3.6 nmol/liter)... (truncated)

Title: The Alleviative Effect of Vitamin B₂ on Potassium Bromate-Induced Hepatotoxicity in Male Rats.
Potassium bromate (PB) is a food enhancer, water disinfection by-product, and a proven carcinogen. It elicits toxicities in the living organism due to exposure and in a dose-dependent manner. The present study discourses the ameliorative efficacy of riboflavin (RF) in PB-administered rodents. The animals were distributed into five treatment groups: control (group I), PB alone (group II, 150 mg/kg), RF alone (group III, 2 mg/kg), PB+RF1 (group IV, 150 mg/kg + 2 mg/kg), and PB+RF2 (group V, 150 mg/kg + 4 mg/kg). After the round of the treatment, the animals were sacrificed to collect their blood and liver samples for... (truncated)

MESH:D014011 - tinidazole

Summary:

LLM Prediction Score	0.468 (normalized)
LLM Confidence Score	0.970
Golden Answer (Severity Class)	0.375 (normalized)
Prediction Error	0.093

Retrieved Context:

Title: Extraintestinal Amoebiasis in Women after 6th Day of Delivery.
Label="BACKGROUND" NlmCategory="BACKGROUND">Extraintestinal amoebiasis is more common in countries with lower socioeconomic status. Complication related to amoebiasis is common in pregnant patients with malnutrition and others. Severe cases can be associated with high fatality rates. We would like to report a patient with a presumptive diagnosis of extraintestinal amoebiasis who was on the 6th postpartum day after intrauterine fetal death (IUFD). <i>Case Presentation</i>. The patient was a 31 year-old female who was on 6th postpartum day after IUFD after the 9th month of amenorrhea. She presented with severe epigastric pain, hiccups, and bloody vomiting of ingested matter for 5 days. She... (truncated)

Title: Flavonoids as a Natural Treatment Against <i>Entamoeba histolytica</i>.
Over the past 20 years, gastrointestinal infections in developing countries have been a serious health problem and are the second leading cause of morbidity among all age groups. Among pathogenic protozoans that cause diarrheal disease, the parasite <i>Entamoeba histolytica</i> produces amebic colitis as well as the most frequent extra-intestinal lesion, an amebic liver abscess (ALA). Usually, intestinal amebiasis and ALA are treated with synthetic chemical compounds (iodoquinol, paromomycin, diloxanide furoate, and nitroimidazoles). Metronidazole is the most common treatment for amebiasis. Although the efficacy of nitroimidazoles in killing amebas is known, the potential resistance of <i>E. histolytica</i> to this treatment is... (truncated)

Title: Curcumin Attenuates the Pathogenicity of <i>Entamoeba histolytica</i> by Regulating the Expression of Virulence Factors in an <i>Ex-Vivo</i> Model Infection.
Infection with the enteric protozoan <i>Entamoeba histolytica</i> is still a serious public health problem, especially in developing countries. Amoebic liver abscess (ALA) is the most common extraintestinal manifestation of the amoebiasis, and it can lead to serious and potentially life-threatening complications in some people. ALA can be cured by metronidazole (MTZ); however, because it has poor activity against luminal trophozoites, 40-60% of treated patients get repeated episodes of invasive disease and require repeated treatments that can induce resistance to MTZ, this may emerge as an important public health problem. Anti-virulence strategies that impair the virulence of pathogens are one of... (truncated)

Title: Ornidazole-Induced Recurrent Encephalopathy in a Chinese Man: A Rare Case Report and Literature Review.
Ornidazole-induced encephalopathy (OIE) is seldom seen in the clinic. In this study, we report a new case of a patient who had taken 1,000 mg ornidazole daily for nearly 4 years because of suspected diarrhea and proctitis and presented with subacute symptoms such as unsteady gait, slurred speech, and psychiatric disorder. These symptoms were significantly relieved 3 days after the patient stopped taking ornidazole. When he took this medicine again, however, similar symptoms occurred 4 months later, which were again reduced after 4 days of drug discontinuation. After the second onset, abnormal signals were identified around the aqueduct of the... (truncated)

Title: Albendazole and Mebendazole as Anti-Parasitic and Anti-Cancer Agents: an Update.
The use of albendazole and mebendazole, i.e., benzimidazole broad-spectrum anthelmintics, in treatment of parasitic infections, as well as cancers, is briefly reviewed. These drugs are known to block the microtubule systems of parasites and mammalian cells leading to inhibition of glucose uptake and transport and finally cell death. Eventually they exhibit ovicidal, larvicidal, and vermicidal effects on parasites, and tumoricidal effects on hosts. Albendazole and mebendazole are most frequently prescribed for treatment of intestinal nematode infections (ascariasis, hookworm infections, trichuriasis, strongyloidiasis, and enterobiasis) and can also be used for intestinal tapeworm infections (taeniases and hymenolepiasis). However, these drugs also exhibit... (truncated)

MESH:D064747 - lansoprazole

Summary:

LLM Prediction Score	0.468 (normalized)
LLM Confidence Score	0.990
Golden Answer (Severity Class)	0.375 (normalized)
Prediction Error	0.093

Retrieved Context:

Title: Voriconazole-Induced Hepatitis via Simvastatin- and Lansoprazole-Mediated Drug Interactions: A Case Report and Review of the Literature.
Therapeutic voriconazole concentrations have a narrow window of effectiveness before causing cholestatic hepatitis. After undergoing 1 year of voriconazole therapy for pulmonary aspergillosis, a 44-year-old man began treatment with 30 mg lansoprazole for gastroesophageal reflux symptoms. Within 5 days of starting treatment with lansoprazole, the patient presented with fatigue, jaundice, and cholestatic hepatitis. The hepatitis promptly resolved after stopping lansoprazole treatment. Sixteen months later, the patient was given simvastatin therapy, as recommended by the American Diabetes Association to prevent cardiovascular disease for patients with diabetes who are aged >40 years and have one additional risk factor. Within 2 weeks of... (truncated)

Title: Lansoprazole protects hepatic cells against cisplatin-induced oxidative stress through the p38 MAPK/ARE/Nrf2 pathway.
Lansoprazole, a proton pump inhibitor, can exert antioxidant effects through the induction of the nuclear factor erythroid 2-related factor 2 (Nrf2) pathway, independently of the inhibition of acid secretion in the gastrointestinal tract. Lansoprazole has been reported to provide hepatoprotection in a drug-induced hepatitis animal model through the Nrf2/heme oxygenase-1 (HO1) pathway. We sought to investigate the molecular mechanism of cytoprotection by lansoprazole. An in vitro experimental model was conducted using cultured rat hepatic cells treated with lansoprazole to analyze the expression levels of Nrf2 and its downstream genes, the activity of Nrf2 using luciferase reporter assays, cisplatin-induced cytotoxicity, and... (truncated)

Title: Lansoprazole-induced acute lung and liver injury: a case report.
A 61-year old woman was admitted with increasing dyspnea and deranged liver function tests. A chest X-ray revealed small volume lungs with reticulo-nodular shadowing. High resolution computed tomography of the chest revealed interlobular septal thickening. The patient subsequently underwent an open lung biopsy and ultrasound-guided liver biopsy, which were consistent with a hypersensitivity pneumonitis and drug-induced liver injury respectively. The patient had previously been commenced on lansoprazole 10 days before the onset of symptoms; this had been stopped at diagnosis. High dose prednisolone was commenced, and the patient went on to make a full recovery. Hypersensitivity pneumonitis is a form... (truncated)

Title: Population Scale Retrospective Analysis Reveals Potential Risk of Cholestasis in Pregnant Women Taking Omeprazole, Lansoprazole, and Amoxicillin.
In nearly 50% of patients with drug-induced liver injury, the bile flow is impaired known as cholestasis. Intrahepatic cholestasis of pregnancy (ICP) is the most common liver disease that happens in pregnancy. Some of the clinical symptoms include pruritus, dark urine, and abnormal liver function tests. A rise of serum bile acids is the most accurate diagnostic evidence. ICP may lead to premature birth, fetal distress, and even postpartum hemorrhage or stillbirth in some severe cases. Higher bile acid levels (> 40 µmol/L) are associated with higher rates of adverse fetal outcomes. Due to the multifactorial nature of ICP, its... (truncated)

Title: Nrf2-inducing anti-oxidation stress response in the rat liver--new beneficial effect of lansoprazole.
Lansoprazole is a potent anti-gastric ulcer drug that inhibits gastric proton pump activity. We identified a novel function for lansoprazole, as an inducer of anti-oxidative stress responses in the liver. Gastric administration of lansoprazole (10-100 mg/kg) to male Wistar rats produced a dose-dependent increase in hepatic mRNA levels of nuclear factor, erythroid-derived 2, -like 2 (Nrf2), a redox-sensitive transcription factor, at 3 h and Nrf2 immunoreactivity (IR) in whole hepatic lysates at 6 h. Conversely, the levels of Kelch-like ECH-associated protein (Keap1), which sequesters Nrf2 in the cytoplasm under un-stimulated conditions, were unchanged. Translocation of Nrf2 into the nuclei of... (truncated)

MESH:D054199 - pseudoephedrine

Summary:

LLM Prediction Score	0.093 (normalized)
LLM Confidence Score	0.990
Golden Answer (Severity Class)	0.0 (normalized)
Prediction Error	0.093

Retrieved Context:

Title: Ephedrine-induced mitophagy via oxidative stress in human hepatic stellate cells.
The herb Ephedra sinica (also known as Chinese ephedra or Ma Huang), used in traditional Chinese medicine, contains alkaloids identical to ephedrine and pseudoephedrine as its principal active constituents. Recent studies have reported that ephedrine has various side effects in the cardiovascular and nervous systems. In addition, herbal Ephedra, a plant containing many pharmacologically active alkaloids, principally ephedrine, has been reported to cause acute hepatitis. Many studies reported clinical cases, however, the cellular mechanism of liver toxicity by ephedrine remains unknown. In this study, we investigated hepatotoxicity and key regulation of mitophagy in ephedrine-treated LX-2 cells. Ephedrine triggered mitochondrial oxidative... (truncated)

Title: Focus on Over-the-Counter Drugs' Misuse: A Systematic Review on Antihistamines, Cough Medicines, and Decongestants.
Background: Over the past 20 years or so, the drug misuse scenario has seen the emergence of both prescription-only and over-the-counter (OTC) medications being reported as ingested for recreational purposes. OTC drugs such as antihistamines, cough/cold medications, and decongestants are reportedly the most popular in being diverted and misused. Objective: While the current related knowledge is limited, the aim here was to examine the published clinical data on OTC misuse, focusing on antihistamines (e.g., diphenhydramine, promethazine, chlorpheniramine, and dimenhydrinate), dextromethorphan (DXM)- and codeine-based cough medicines, and the nasal decongestant pseudoephedrine. Methods: A systematic literature review was carried out with the... (truncated)

Title: Sympathomimetic amine compounds and hepatotoxicity: Not all are alike-Key distinctions noted in a short review.
Sympathomimetic amine compounds are often pooled together and incorrectly assumed to be interchangeable with respect to potential adverse effects. A brief and specific review of sympathomimetic compounds and one instance (i.e., hepatotoxicity) where these compounds have been improperly grouped together is covered. A review of the proposed mechanisms through which known hepatotoxic sympathomimetic agents (e.g., 3,4-methylenedioxymethamphetamine or MDMA, methamphetamine and amphetamine) cause liver injury, along with a corresponding review of in vitro data, interventional data, animal model studies and observational data allow for a comparison/contrast of different agents and reveals a lack of potential toxicity for some agents (e.g., pseudoephedrine,... (truncated)

Title: Emerging drugs of abuse: current perspectives on substituted cathinones.
Substituted cathinones are synthetic analogs of cathinone that can be considered as derivatives of phenethylamines with a beta-keto group on the side chain. They appeared in the recreational drug market in the mid-2000s and now represent a large class of new popular drugs of abuse. Initially considered as legal highs, their legal status is variable by country and is rapidly changing, with government institutions encouraging their control. Some cathinones (such as diethylpropion or pyrovalerone) have been used in a medical setting and bupropion is actually indicated for smoking cessation. Substituted cathinones are widely available from internet websites, retail shops, and... (truncated)

Title: Manganese-Induced Parkinsonism due to Ephedrone Abuse.
During recent years, a syndrome of hypokinesia, dysarthria, dystonia, and postural impairment, related to intravenous use of a "designer" psychostimulant derived from pseudoephedrine using potassium permanganate as the oxidant, has been observed in drug addicts in several countries in Eastern Europe with some cases also in Western countries. A levodopa unresponsive Parkinsonian syndrome occurs within a few months of abusing the homemade drug mixture containing ephedrone (methcathinone) and manganese. The development of this neurological syndrome has been attributed to toxic effects of manganese, but the role of the psychostimulant ephedrone is unclear. This paper describes the clinical syndrome, results of... (truncated)

MESH:C004528 - benzarone

Summary:

LLM Prediction Score	0.908 (normalized)
LLM Confidence Score	0.810
Golden Answer (Severity Class)	1.0 (normalized)
Prediction Error	0.092

Retrieved Context:

Title: [Drug-induced hepatitis due to benzarone (Fragivix): apropos of a clinical case report].
A 46-year-old woman with a previous history of thyroiditis but without clinical evidence of preexisting liver disease presented with severe acute hepatitis while under benzarone therapy (Fragivix, 400 mg per day, p.o.). Because of negative serological work-up, a tentative diagnosis of "sporadic" non-A, non-B hepatitis was considered. Two months after resolution, a relapse of clinical and biochemical features of hepatitis occurring 10 days after the spontaneous resumption of therapy led to the diagnosis of drug-induced liver damage. This case represents the third reported case of severe hepatitis related to benzarone. The mechanism of liver toxicity was likely due to hypersensitivity.

Title: Severe hepatotoxicity related to benzarone: a report of three cases with two fatalities.
We report three cases of severe hepatotoxicity related to benzarone, a benzofuran derivative. Our cases include a 35-year-old woman with (sub)fulminant hepatitis, a 67-year-old woman with macronodular cirrhosis, and a 68-year-old man with severe chronic active hepatitis and cirrhosis, with positivity of anti-smooth muscle antibodies. Two patients died. We stress the potential of benzarone to cause hepatotoxicity, which usually resembles severe chronic active hepatitis. Our cases constitute the most severe cases of benzarone hepatotoxicity reported so far, and comprise the first cases of (sub)fulminant hepatitis and cirrhosis related to benzarone.

Title: [Drug-induced hepatic injury, the challenge for cause investigation].
This report is the story of the long journey to identify the mechanism of fulminant hepatitis by the antigout drug, Benzbromarone. As soon as the 8th gout patient prescribed Benzbromarone (Benz) died of fulminant hepatitis in 20 years, the letter was sent to doctors identifying it as the causative agent in February 2000. At that time, Benz had been prescribed to 350,000 patients/year for 20 years. Is Benz the real cause of fulminant hepatitis? 1. Benz is a PPARa agonist like fenofibrate, and not a PPARgamma like troglitazone. 2. Troglitazone and Allopurinol have shown apoptosis in a human primary hepatocyte... (truncated)

Title: Oxidant stress, mitochondria, and cell death mechanisms in drug-induced liver injury: lessons learned from acetaminophen hepatotoxicity.
Hepatotoxicity is a serious problem during drug development and for the use of many established drugs. For example, acetaminophen overdose is currently the most frequent cause of acute liver failure in the United States and Great Britain. Evaluation of the mechanisms of drug-induced liver injury indicates that mitochondria are critical targets for drug toxicity, either directly or indirectly through the formation of reactive metabolites. The consequence of these modifications is generally a mitochondrial oxidant stress and peroxynitrite formation, which leads to structural alterations of proteins and mitochondrial DNA and, eventually, to the opening of mitochondrial membrane permeability transition (MPT) pores.... (truncated)

Title: Molecular mechanisms involved in drug-induced liver injury caused by urate-lowering Chinese herbs: A network pharmacology study and biology experiments.
As an important part of the comprehensive treatment methods, the urate-lowering Chinese herbs could provide favorable clinical effects on hyperuricemia in its ability to invigorate spleen and remove dampness. Owing to the long-term duration, it brought up the potential adverse reactions (ADRs) and concerns about the drug-induced liver injury from these herbs. To address this problem, the bioinformatics approaches which combined the network pharmacology, computer simulation and molecular biology experiments were undertaken to elucidate the underlying drug-induced liver injury molecular mechanisms of urate-lowering Chinese herbs. Several electronic databases were searched to identify the potential liver injury compounds in published research.... (truncated)

MESH:C084597 - chlorcyclizine

Summary:

LLM Prediction Score	0.091 (normalized)
LLM Confidence Score	0.940
Golden Answer (Severity Class)	0.0 (normalized)
Prediction Error	0.091

Retrieved Context:

Title: Discovery, Optimization, and Characterization of Novel Chlorcyclizine Derivatives for the Treatment of Hepatitis C Virus Infection.
Recently, we reported that chlorcyclizine (CCZ, Rac-2), an over-the-counter antihistamine piperazine drug, possesses in vitro and in vivo activity against hepatitis C virus. Here, we describe structure-activity relationship (SAR) efforts that resulted in the optimization of novel chlorcyclizine derivatives as anti-HCV agents. Several compounds exhibited EC50 values below 10 nM against HCV infection, cytotoxicity selectivity indices above 2000, and showed improved in vivo pharmacokinetic properties. The optimized molecules can serve as lead preclinical candidates for the treatment of hepatitis C virus infection and as probes to study hepatitis C virus pathogenesis and host-virus interaction.

Title: Association of CYP1A1 and CYP1B1 inhibition in in vitro assays with drug-induced liver injury.
Drug-induced liver injury (DILI) is one of the major causes for the discontinuation of drug development and withdrawal of drugs from the market. Since it is known that reactive metabolite formation and being substrates or inhibitors of cytochrome P450s (P450s) are associated with DILI, we systematically investigated the association between human P450 inhibition and DILI. The inhibitory activity of 266 DILI-positive drugs (DILI drugs) and 92 DILI-negative drugs (no-DILI drugs), which were selected from Liver Toxicity Knowledge Base (US Food and Drug Administration), against 8 human P450 forms was assessed using recombinant enzymes and luminescent substrates, and the threshold values... (truncated)

Title: Antimicrobial Properties of Antidepressants and Antipsychotics-Possibilities and Implications.
The spreading of antibiotic resistance is responsible annually for over 700,000 deaths worldwide, and the prevision is that this number will increase exponentially. The identification of new antimicrobial treatments is a challenge that requires scientists all over the world to collaborate. Developing new drugs is an extremely long and costly process, but it could be paralleled by drug repositioning. The latter aims at identifying new clinical targets of an "old" drug that has already been tested, approved, and even marketed. This approach is very intriguing as it could reduce costs and speed up approval timelines, since data from preclinical studies... (truncated)

Title: Identification of Serum Biomarkers to Distinguish Hazardous and Benign Aminotransferase Elevations.
The standard circulating biomarker of liver injury in both clinical settings and drug safety testing is alanine aminotransferase (ALT). However, ALT elevations sometimes lack specificity for tissue damage. To identify novel serum biomarkers with greater specificity for injury, we combined unique animal models with untargeted proteomics, followed by confirmation with immunoblotting. Using proteomics, we identified 109 proteins in serum from mice with acetaminophen (APAP)-induced liver injury that were not detectable in serum from mice with benign ALT elevations due to high-dose dexamethasone (Dex). We selected 4 (alcohol dehydrogenase 1A1 [Aldh1a1], aldehyde dehydrogenase 1 [Adh1], argininosuccinate synthetase 1 [Ass1], and adenosylhomocysteinase... (truncated)

Title: Broad-Spectrum Host-Based Antivirals Targeting the Interferon and Lipogenesis Pathways as Potential Treatment Options for the Pandemic Coronavirus Disease 2019 (COVID-19).
The ongoing Coronavirus Disease 2019 (COVID-19) pandemic caused by severe acute respiratory syndrome coronavirus 2 (SARS-CoV-2) signals an urgent need for an expansion in treatment options. In this study, we investigated the anti-SARS-CoV-2 activities of 22 antiviral agents with known broad-spectrum antiviral activities against coronaviruses and/or other viruses. They were first evaluated in our primary screening in VeroE6 cells and then the most potent anti-SARS-CoV-2 antiviral agents were further evaluated using viral antigen expression, viral load reduction, and plaque reduction assays. In addition to remdesivir, lopinavir, and chloroquine, our primary screening additionally identified types I and II recombinant interferons, 25-hydroxycholesterol,... (truncated)

MESH:C499671 - aplaviroc

Summary:

LLM Prediction Score	0.909 (normalized)
LLM Confidence Score	0.890
Golden Answer (Severity Class)	1.0 (normalized)
Prediction Error	0.091

Retrieved Context:

Title: [Secondary effects of treatment with maraviroc and other CCR5 antagonists. Potential impact of the CCR5 blocker].
Maraviroc is the first inhibitor of CCR5 co-receptors to be marketed as an antiretroviral. The pre-clinical studies and phase III trials have shown that it has a very favourable safety profile. No characteristic adverse effect of maraviroc has been identified. Unlike with aplaviroc, where its clinical development was stopped due to serious hepatoxicity, no increase in liver toxicity has been demonstrated in patients treated with maraviroc even if they are co-infected by hepatotropic virus. Nor was there any evidence of an increase in the incidence of neoplasms or serious infections in patients treated with maraviroc. In a study on naive... (truncated)

Title: [CCR5 antagonists and HIV-1 infection: Bases and consequences of this therapeutic approach].
CCR5 molecule is a chemokine receptor with an important role in infectious diseases; not only is it the main coreceptor for HIV-1, but it has also been involved in the immune defense against various transmissible agents. CCR5 antagonists constitute a new class of antiretrovirals. Three molecules of this class have reached phases 2B and 3 of clinical development: aplaviroc (GlaxoSmithKine), vicriviroc (Schering-Plough) and maraviroc (Pfizer). The development of aplaviroc was stopped because of some cases of drug-induced hepatitis. In ACTG 5211 and Motivate trials, adding vicriviroc (in phase 3 trials) or maraviroc (now approved for clinical use) respectively to an... (truncated)

Title: Effects of CC chemokine receptor 5 (CCR5) inhibitors on the dynamics of CCR5 and CC-chemokine-CCR5 interactions.
This study aimed to examine how CC chemokine receptor 5 (CCR5) inhibitors (aplaviroc [APL], TAK779 and maraviroc [MVC]) interact with CCR5 and affect its dynamics and physiological CC-chemokine-CCR5 interactions.

Title: NNRTI and Liver Damage: Evidence of Their Association and the Mechanisms Involved.
Due to the improved effectiveness and safety of combined antiretroviral therapy, human immunodeficiency virus (HIV) infection has become a manageable, chronic condition rather than a mortal disease. However, HIV patients are at increased risk of experiencing non-AIDS-defining illnesses, with liver-related injury standing out as one of the leading causes of death among these patients. In addition to more HIV-specific processes, such as antiretroviral drug-related toxicity and direct injury to the liver by the virus itself, its pathogenesis is related to conditions that are also common in the general population, such as alcoholic and non-alcoholic fatty liver disease, viral hepatitis, and... (truncated)

Title: Maraviroc, a CCR5 antagonist, prevents development of hepatocellular carcinoma in a mouse model.
Chronic liver disease may result in a sequential progression through fibrosis, cirrhosis and lead, eventually, to hepatocellular carcinoma (HCC). Hepatic stellate cells (HSC) seem to be responsible for the fibrogenic response through the activation of an autocrine loop involving the chemokine receptor, CCR5. However, the role of CCR5 in HCC remains poorly understood. Since this receptor is also one of the main ports of entry for the human immunodeficiency virus (HIV), several CCR5 inhibitors are being used in the clinic to reduce viral load. We used one of these inhibitors, maraviroc (MVC), in a mouse model of diet-induced HCC to... (truncated)

MESH:D000070 - acebutolol

Summary:

LLM Prediction Score	0.285 (normalized)
LLM Confidence Score	0.950
Golden Answer (Severity Class)	0.375 (normalized)
Prediction Error	0.090

Retrieved Context:

Title: LC and LC-MS/MS studies for the identification and characterization of degradation products of acebutolol.
The aim of the present investigation was to demonstrate an approach involving use of liquid chromatography (LC) and liquid chromatography-mass spectrometry (LC-MS) to separate, identify and characterize very small quantities of degradation products (DPs) of acebutolol without their isolation from the reaction mixtures. The drug was subjected to oxidative, hydrolytic, thermal and photolytic stress conditions as per International Conference on Harmonization (ICH) guideline Q1A(R2). Among all the stress conditions the drug was found to be labile in hydrolytic (acidic & basic) and photolytic stress conditions, while it was stable in water-induced hydrolysis, oxidative and thermal stress conditions. A total of... (truncated)

Title: Parenteral Nutrition-Associated Liver Disease: The Role of the Gut Microbiota.
Parenteral nutrition (PN) provides life-saving nutritional support in situations where caloric supply via the enteral route cannot cover the necessary needs of the organism. However, it does have serious adverse effects, including parenteral nutrition-associated liver disease (PNALD). The development of liver injury associated with PN is multifactorial, including non-specific intestine inflammation, compromised intestinal permeability, and barrier function associated with increased bacterial translocation, primary and secondary cholangitis, cholelithiasis, short bowel syndrome, disturbance of hepatobiliary circulation, lack of enteral nutrition, shortage of some nutrients (proteins, essential fatty acids, choline, glycine, taurine, carnitine, etc.), and toxicity of components within the nutrition mixture itself... (truncated)

Title: Cheminformatics analysis of assertions mined from literature that describe drug-induced liver injury in different species.
Drug-induced liver injury is one of the main causes of drug attrition. The ability to predict the liver effects of drug candidates from their chemical structures is critical to help guide experimental drug discovery projects toward safer medicines. In this study, we have compiled a data set of 951 compounds reported to produce a wide range of effects in the liver in different species, comprising humans, rodents, and nonrodents. The liver effects for this data set were obtained as assertional metadata, generated from MEDLINE abstracts using a unique combination of lexical and linguistic methods and ontological rules. We have analyzed... (truncated)

Title: Association of CYP1A1 and CYP1B1 inhibition in in vitro assays with drug-induced liver injury.
Drug-induced liver injury (DILI) is one of the major causes for the discontinuation of drug development and withdrawal of drugs from the market. Since it is known that reactive metabolite formation and being substrates or inhibitors of cytochrome P450s (P450s) are associated with DILI, we systematically investigated the association between human P450 inhibition and DILI. The inhibitory activity of 266 DILI-positive drugs (DILI drugs) and 92 DILI-negative drugs (no-DILI drugs), which were selected from Liver Toxicity Knowledge Base (US Food and Drug Administration), against 8 human P450 forms was assessed using recombinant enzymes and luminescent substrates, and the threshold values... (truncated)

Title: Pharmacokinetics of acebutolol and its main metabolite, diacetolol after oral administration of acebutolol in rabbits with carbon tetrachloride-induced hepatic failure.
Pharmacokinetic characteristics of acebutolol and its main metabolite, diacetolol, following a single 10 mg/kg oral dose, were investigated in rabbits with carbon tetrachloride-induced hepatic failure. Plasma concentrations of acebutolol and diacetolol were determined by a high performance liquid chromatography assay. The area under the plasma concentration-time curves (AUC) and maximum plasma concentration (Cmax) of acebutolol were significantly increased in moderate and severe carbon tetrachloride-induced hepatic failure rabbits. The ratio of the diacetolol to total acebutolol in plasma (i.e., metabolite percentage rate) was significantly decreased in moderate and severe carbon tetrachloride-induced hepatic failure rabbits. Volume of distribution (Vd) and total body... (truncated)

MESH:D000069442 - natalizumab

Summary:

LLM Prediction Score	0.535 (normalized)
LLM Confidence Score	0.970
Golden Answer (Severity Class)	0.625 (normalized)
Prediction Error	0.090

Retrieved Context:

Title: Gynecological adverse effects of natalizumab administration: Case report and review of the literature.
Natalizumab is administered for the treatment of relapsing-remitting multiple sclerosis (RR-MS) with high disease activity.Natalizumab therapy has been associated with adverse effects, such as progressive multifocal leukoencephalopathy, liver damage, nasopharyngitis, urinary tract infection, urticaria, cephalgia, dizziness, fatigue, nausea, fever, rigidity, anxiety and gastroenteritis.

Title: A Case of Drug-Induced Liver Injury Secondary to Natalizumab.
A 33-year-old Caucasian female with relapsing-remitting multiple sclerosis presented with abdominal pain, nausea, and vomiting and was found to have acute liver injury. After thorough investigation, she was diagnosed with drug-induced liver injury (DILI) thought secondary to redosing of Natalizumab therapy.

Title: Natalizumab (TYSABRI) and multiple sclerosis. With longer follow-up: even more toxic than suspected.
The standard disease-modifying treatment for patients with relapsing-remitting multiple sclerosis is interferon beta injection, in the absence of a better alternative. In 2007, natalizumab had an unfavourable harm-benefit balance in patients with severe multiple sclerosis in whom interferon beta was ineffective, due to insufficient evidence of efficacy and a risk of life-threatening progressive multifocal leukoencephalopathy. In 2014, we found no new comparative trials focusing on the efficacy of natalizumab monotherapy in its authorised indications in the EU. Post-marketing data confirm the adverse effects identified in clinical trials, including serious and life-threatening opportunistic infections, particularly progressive multifocal leukoencephalopathy in about two... (truncated)

Title: Acute Hepatitis with Positive Autoantibodies: A Case of Natalizumab-Induced Early-Onset Liver Injury.
BACKGROUND Natalizumab is an anti-integrin monoclonal antibody used as an alternative treatment regimen for patients with autoimmune disorders, especially multiple sclerosis and Crohn's disease. Natalizumab-induced liver injury has been rarely reported and may follow the first dose (with increases in liver enzymes usually after 6 or more days), or after multiple doses. In general, it is non-severe acute hepatitis (with a hepatocellular pattern) and autoantibodies can be positive, mainly anti-nuclear and anti-smooth muscle antibodies. CASE REPORT We are reporting the case of a 60-year-old woman diagnosed with multiple sclerosis previously treated with interferon-beta, dimethyl fumarate, and fingolimod, who presented jaundice... (truncated)

Title: Multiple immune disorders after natalizumab discontinuation: After the CIRIS, the SIRIS?
Natalizumab (NTZ) is an effective treatment for patients with highly active relapsing remitting multiple sclerosis (MS). However, when the therapy must be interrupted, it is important to anticipate the withdrawal to avoid reactivation or disease rebound. Described here is the case of a 35-year-old woman, with a past history of beta thalassemia, bulimia and asthma, who was diagnosed with MS at age 26. She was treated initially with first-line subcutaneous (sc) immunomodulatory treatments. However, due to liver toxicity, interferon beta-1a sc was interrupted and replaced by glatiramer acetate treatment, which was well tolerated and used for several years. Unfortunately, disease... (truncated)

MESH:D001977 - brompheniramine

Summary:

LLM Prediction Score	0.089 (normalized)
LLM Confidence Score	0.960
Golden Answer (Severity Class)	0.0 (normalized)
Prediction Error	0.089

Retrieved Context:

Title: Plasma levels of zimelidine and norzimelidine in endogenous depression.
A new serotonin uptake inhibitor zimelidine was studied in 16 endogenously depressed inpatients, who received 150 mg/kg orally during 3--6 weeks in a phase II-type study. Plasma concentrations of zimelidine and its main metabolite norzimelidine were determined twice a week. Ten patients obtained a well-defined steady-state plasma level within 1--2 weeks, while three patients still had increasing concentrations of both substances or only norzimelidine within the investigation period. In two patients, biochemical affection of the liver could be demonstrated during the treatment; one associated with moderate clinical symptoms (dizziness and fever), the other without clinical symptoms. Both patients recovered upon... (truncated)

Title: Marine Bromophenol Derivative 3,4-Dibromo-5-(2-bromo-3,4-dihydroxy-6-isopropoxymethyl benzyl)benzene-1,2-diol Protects Hepatocytes from Lipid-Induced Cell Damage and Insulin Resistance via PTP1B Inhibition.
3,4-Dibromo-5-(2-bromo-3,4-dihydroxy-6-isopropoxymethyl benzyl)benzene-1,2-diol (HPN) is a bromophenol derivative from the marine red alga Rhodomela confervoides. We have previously found that HPN exerted an anti-hyperglycemic property in db/db mouse model. In the present study, we found that HPN could protect HepG2 cells against palmitate (PA)-induced cell death. Data also showed that HPN inhibited cell death mainly by blocking the cell apoptosis. Further studies demonstrated that HPN (especially at 1.0 μM) significantly restored insulin-stimulated tyrosine phosphorylation of IR and IRS1/2, and inhibited the PTP1B expression level in HepG2 cells. Furthermore, the expression of Akt was activated by HPN, and glucose uptake was significantly... (truncated)

Title: NAFLD as a continuum: from obesity to metabolic syndrome and diabetes.
The prevalence of non-alcoholic fatty liver disease (NAFLD) has been increasing rapidly. It is nowadays recognized as the most frequent liver disease, affecting a quarter of global population and regularly coexisting with metabolic disorders such as type 2 diabetes, hypertension, obesity, and cardiovascular disease. In a more simplistic view, NAFLD could be defined as an increase in liver fat content, in the absence of secondary cause of steatosis. In fact, the clinical onset of the disease is a much more complex process, closely related to insulin resistance, limited expandability and dysfunctionality of adipose tissue. A fatty liver is a main... (truncated)

Title: Prevalence and factors associated with potentially inappropriate medication use in older medicare beneficiaries with cancer.
To assess the factors related to potentially inappropriate medication (PIM) use in elderly patients with cancer, as well as to compare the PIM prevalence in older adults with and without cancer.

Title: Position statement of the Brazilian Academy of Rhinology on the use of antihistamines, antileukotrienes, and oral corticosteroids in the treatment of inflammatory sinonasal diseases.
Inflammatory conditions of the nose and paranasal sinuses are very prevalent in the general population, resulting in marked loss of quality of life in affected patients, as well as significant work, leisure, and social activity losses. These patients require specific and specialized treatment. A wide range of oral medications are available.

MESH:C084834 - cinchophen

Summary:

LLM Prediction Score	0.911 (normalized)
LLM Confidence Score	0.860
Golden Answer (Severity Class)	1.0 (normalized)
Prediction Error	0.089

Retrieved Context:

Title: Glafenine-associated hepatic injury. Analysis of 38 cases and review of the literature.
Glafenine was associated with hepatic injury in 38 cases. The causal relationship was assessed on the basis of the temporal relationship with drug use, course and exclusion of other causes. In 27 cases a causal relationship was considered likely, i.e. 'probable' (12 cases) or 'possible' (15 cases), whereas in 11 cases it was either unlikely or unclassifiable. In both the 'probable' and 'possible' groups 60-70% of individuals were women. Jaundice was present in three-quarters of cases in both groups. Eosinophilia was more frequent in the group of 'probable' cases, and this group had the highest case-fatality rate (42%). Onset varied... (truncated)

Title: [Toxic hepatitis from cinchophen: report of 3 cases].
Three cases of hepatic toxicity due to cincophen (a uricosuric drug used for the treatment of gout) are reported. The three patients were females aged 57, 60 and 67 years, respectively, who were treated with cincophen because of renoureteral urate stones. The mean dose was 300 mg/day for a mean duration of 3-4 months before the hepatotoxic features first developed. Cincophen induces hepatic necrosis through a hypersensitivity or metabolic idiosyncrasy mechanism with histologic abnormalities similar to those due to isoniazid, anticonvulsants, halothane and methyldopa. In two patients the disease presented as acute hepatitis. One had severe hepatic failure and died.... (truncated)

Title: In vitro platforms for evaluating liver toxicity.
The liver is a heterogeneous organ with many vital functions, including metabolism of pharmaceutical drugs and is highly susceptible to injury from these substances. The etiology of drug-induced liver disease is still debated although generally regarded as a continuum between an activated immune response and hepatocyte metabolic dysfunction, most often resulting from an intermediate reactive metabolite. This debate stems from the fact that current animal and in vitro models provide limited physiologically relevant information, and their shortcomings have resulted in "silent" hepatotoxic drugs being introduced into clinical trials, garnering huge financial losses for drug companies through withdrawals and late stage... (truncated)

Title: A correlation between the in vitro drug toxicity of drugs to cell lines that express human P450s and their propensity to cause liver injury in humans.
Drug toxicity to T-antigen-immortalized human liver epithelial (THLE) cells stably transfected with plasmid vectors that encoded human cytochrome P450s 1A2, 2C9, 2C19, 2D6, or 3A4, or an empty plasmid vector (THLE-Null), was investigated. An automated screening platform, which included 1% dimethyl sulfoxide (DMSO) vehicle, 2.7% bovine serum in the culture medium, and assessed 3-(4,5-dimethylthiazol-2-yl)-5-(3-carboxymethoxyphenyl)-2-(4-sulfophenyl)-2H-tetrazolium reduction, was used to evaluate the cytotoxicity of 103 drugs after 24h. Twenty-two drugs caused cytotoxicity to THLE-Null cells, with $EC_{50} \leq 200$ μM; 21 of these drugs (95%) have been reported to cause human liver injury. Eleven drugs exhibited lower EC_{50} values in cells transfected... (truncated)

Title: Drug-induced liver injury: Do we know everything?
Interest in drug-induced liver injury (DILI) has dramatically increased over the past decade, and it has become a hot topic for clinicians, academics, pharmaceutical companies and regulatory bodies. By investigating the current state of the art, the latest scientific findings, controversies, and guidelines, this review will attempt to answer the question: Do we know everything? Since the first descriptions of hepatotoxicity over 70 years ago, more than 1000 drugs have been identified to date, however, much of our knowledge of diagnostic and pathophysiologic principles remains unchanged. Clinically ranging from asymptomatic transaminitis and acute or chronic hepatitis, to acute liver failure,... (truncated)

MESH:D005047 - etoposide

Summary:

LLM Prediction Score	0.464 (normalized)
LLM Confidence Score	0.990
Golden Answer (Severity Class)	0.375 (normalized)
Prediction Error	0.089

Retrieved Context:

Title: Etoposide pharmacokinetics in patients with normal and abnormal organ function.
Precise guidelines for dose modification of etoposide in patients with hepatic dysfunction have not been determined. Etoposide pharmacokinetics were determined in 17 patients. Nine patients had bilirubin less than or equal to 1 mg/dL and eight had bilirubin ranging from 1.9 to 23 mg/dL. Twelve patients received etoposide 100 mg/m2 days 1, 3, and 5, in combination with cisplatin 70 mg/m2 or iproplatin 225 mg/m2 on day 1. Five patients received only one dose of etoposide. Etoposide was measured using a published high pressure liquid chromatography (HPLC) method which also quantitates picro etoposide and its hydroxy acid. Systemic clearance, Vdss... (truncated)

Title: Clinical feasibility of oral low-dose etoposide and sobuzoxane for conventional chemotherapy-intolerant lymphoma patients.
Introduction: In Japan, etoposide or sobuzoxane, a type of topoisomerase II inhibitor, is orally administered in patients with lymphoma who cannot tolerate conventional combination chemotherapy. However, the related clinical data remain to be fully summarized.Areas covered: We evaluate the efficacy and toxicity of etoposide and sobuzoxane.Expert opinion: Previous studies on etoposide or sobuzoxane monotherapy, including those among patients who could not tolerate conventional chemotherapy, suggested a favorable overall response rate (ORR) with moderate gastrointestinal or liver/renal toxicity. As for adult T-cell leukemia/lymphoma, a clinical trial with a limited sample size exhibited an ORR of >70%. Remarkably, the percentage of patients... (truncated)

Title: [Hepatotoxicity with etoposide-ifosfamide combination therapy].
Combination chemotherapy with etoposide and ifosfamide in 2 patients with advanced bronchial carcinomas caused hepatotoxic side effects. Hepatotoxicity was observed during and immediately after the 5-day therapy and was characterised by a massive rise in direct bilirubin, drop in cholinesterase, as well as a rise in the alkaline phosphatase and to a lesser extent in gGT and GOT. Hepatotoxicity was lethal in one case and reversed to normal in the other. Hepatotoxicity was most likely caused by ifosfamide, since this drug was applied for the first time in both cases. Nonetheless, a contribution of etoposide in this regard can not... (truncated)

Title: Toxicity of high-dose chemotherapy with etoposide, thiotepa and CY in treating poor-prognosis Ewing's sarcoma family tumors: the experience of the Bambino Gesù Children's Hospital.
We report the toxicity of high-dose chemotherapy (HDC) based on etoposide, thiotepa and CY (ETC) in children with poor-prognosis Ewing's sarcoma family tumors (ESFTs). A total of 26 patients with high-risk ESFT (metastasis or axis localization or tumor volume >200 ml or necrosis <95%) were reviewed. The conditioning was based on etoposide (600 mg/m(2)), thiotepa (750 mg/m(2)) and CY (120 mg/kg) followed by autologous BM or PBSC rescue. The conditioning regimen was well tolerated, without any toxic deaths. The median time from transplant to a neutrophil count of >0.5 x 10(9)/l was 10 days (range 6-27) and 22.5 days (range... (truncated)

Title: Hepatotoxicity following vincristine therapy.
A patient is reported with small cell lung cancer treated with combination chemotherapy (cyclophosphamide, vincristine and etoposide [VP-16-213] who developed transient liver function abnormalities secondary to vincristine therapy. Serum transaminase (SGOT and SGPT) levels rose by 2 to 6 times, lactic dehydrogenase (LDH) 1.5 to 2 times, and alkaline phosphatase and gamma-glutamyl transpeptidase (GGTP) 1.5 to 2 times normal. Enzyme abnormalities were observed by the 6th day following drug administration and returned to normal between 16 and 48 days, except for the GGTP elevations which persisted longer. Vincristine has been suspected to cause liver damage and to enhance radiation-induced hepatic... (truncated)

MESH:D000069449 - cinacalcet

Summary:

LLM Prediction Score	0.088 (normalized)
LLM Confidence Score	0.980
Golden Answer (Severity Class)	0.0 (normalized)
Prediction Error	0.088

Retrieved Context:

Title: New strategies in treatment of mineral and bone disorders and associated cardiovascular disease in patients with chronic kidney disease.
Mineral and bone disorders in chronic kidney disease (CKD) patients along with the use of calcium-based phosphate binders may result in vascular calcification (VC) development and associated increase in cardiovascular diseases (CVD) mortality. A few treatment modalities to control hyperphosphatemia, VC and CVD over the years have failed. Recently appeared calcium-aluminum free phosphate binders (sevelamer hydrochloride and lanthanum carbonate) have reduced hypercalcemic adverse events compared to calcium-based binders, although beneficial effects on CVD outcome to justify further widespread utilization of these agents in CKD patients are not reported so far. At present long-term safety of lanthanum administration has been challenged... (truncated)

Title: Pharmacokinetics of evocalcet in secondary hyperparathyroidism patients receiving hemodialysis: first-in-patient clinical trial in Japan.
Cinacalcet is a positive allosteric modulator of calcium-sensing receptors in the parathyroid gland and an effective treatment for secondary hyperparathyroidism. However, this agent has considerable side effects and dosage limitations, which impair effective treatment. Therefore, we investigated the pharmacokinetics, pharmacodynamics, and safety of the novel calcimimetic, evocalcet.

Title: Metabolic acidosis-induced hypercalcemia in an azotemic patient with primary hyperparathyroidism.
A 58-year-old man with Stage 3b chronic kidney disease and primary hyperparathyroidism treated with cinacalcet was admitted for acute cholecystitis. A cholecystostomy tube was placed, estimated glomerular filtration rate decreased, metabolic acidosis developed and ionized calcium increased from 1.33 to 1.76 mM despite cinacalcet administration. A sodium bicarbonate infusion corrected the metabolic acidosis restoring ionized calcium to normal despite no improvement in renal function. The correlation between the increase in serum bicarbonate and decrease in ionized calcium was $r = -0.93$, $P < 0.001$. In summary, severe hypercalcemia was attributable to metabolic acidosis increasing calcium efflux from bone while renal... (truncated)

Title: Therapeutic Effect of Calcimimetics on Osteoclast-Osteoblast Crosslink in Chronic Kidney Disease and Mineral Bone Disease.
We have previously demonstrated calcimimetics optimize the balance between osteoclastic bone resorption and osteoblastic mineralization through upregulating Wingless and int-1 (Wnt) signaling pathways in the mouse and cell model. Nonetheless, definitive human data are unavailable concerning therapeutic effects of Cinacalcet on chronic kidney disease and mineral bone disease (CKD-MBD) and osteoclast-osteoblast interaction. We aim to investigate whether Cinacalcet therapy improves bone mineral density (BMD) through optimizing osteocytic homeostasis in a human model. Hemodialysis patients with persistently high intact parathyroid hormone (iPTH) levels > 300 pg/mL for more than 3 months were included and received fixed dose Cinacalcet (25 mg/day, orally)... (truncated)

Title: Pharmacokinetics and pharmacodynamics of cinacalcet in hepatic impairment : phase I, open-label, parallel-group, single-dose, single-centre study.
The calcimimetic cinacalcet lowers blood para-thyroid hormone (PTH), calcium and phosphorus levels and calcium-phosphorus product in patients with chronic kidney disease receiving dialysis. Cinacalcet is metabolized primarily through oxidative and conjugative pathways. Hepatic disease has the potential to alter cinacalcet metabolism. Thus, it is important to establish the potential for altered cinacalcet metabolism according to the level of hepatic function. This study aimed to evaluate the pharmacokinetics and pharmacodynamics of cinacalcet in subjects with different degrees of hepatic function.

MESH:C105254 - pramlintide

Summary:

LLM Prediction Score	0.088 (normalized)
LLM Confidence Score	0.980
Golden Answer (Severity Class)	0.0 (normalized)
Prediction Error	0.088

Retrieved Context:

Title: Sustained weight loss following 12-month pramlintide treatment as an adjunct to lifestyle intervention in obesity.
To assess long-term weight loss efficacy and safety of pramlintide used at different dosing regimens and in conjunction with lifestyle intervention (LSI).

MESH:C053001 - adefovir

Summary:

LLM Prediction Score	0.337 (normalized)
LLM Confidence Score	0.970
Golden Answer (Severity Class)	0.25 (normalized)
Prediction Error	0.087

Retrieved Context:

Title: Adverse effects of oral antiviral therapy in chronic hepatitis B.
Oral nucleoside/nucleotide analogues (NAs) are currently the backbone of chronic hepatitis B (CHB) infection treatment. They are generally well-tolerated by patients and safe to use. To date, a significant number of patients have been treated with NAs. Safety data has accumulated over the years. The aim of this article is to review and update the adverse effects of oral NAs. NAs can cause class adverse effects (<i>i.e</i>., myopathy, neuropathy, lactic acidosis) and dissimilar adverse effects. All NAs carry a "Black Box" warning because of the potential risk for mitochondrial dysfunction. However, these adverse effects are rarely reported. The majority of... (truncated)

Title: Antiviral hepatitis and antiretroviral drug interactions.
More and more HIV-infected patients are treated for viral hepatitis, increasing interactions. HEPATITIS C: The concomitant use of didanosine and ribavirin increases the risk of mitochondrial toxicity, responsible for pancreatitis and/or lactic acidosis. Lactic acidosis is characterized by a high mortality rate. Thus, didanosine, but also stavudine, should not be co-administered with ribavirin. Cases of hepatic decompensation have been reported in cirrhotics concomitantly receiving ribavirin and didanosine. Thus, this co-admininistration should be contraindicated in patients with advanced liver fibrosis. Anemia is a frequent side effect of ribavirin. In patients with zidovudine-related anemia, this drug should be discontinued before prescribing ribavirin.... (truncated)

Title: [Diagnostic, clinical and therapeutic implications of viral hepatitides in HIV-infected patients].
Viral hepatitis B (HBV) and C (HCV) are a significant cause of morbidity and mortality in patients coinfected with human immunodeficiency virus (HIV). In HBV/HIV coinfection, there is a higher frequency of HBV replication, and higher rates of HBV-associated liver disease. The only drugs currently approved for the treatment of HBV infection are lamivudine, adefovir, entecavir, and interferon-a. HIV/HBV coinfection is associated with an increased frequency of hepatotoxicity in patients receiving highly active antiretroviral therapy (HAART), and reactivation of clinical hepatitis is observed upon stopping HBV-active anti-HIV drugs. Liver disease due to HCV infection is currently the leading cause of... (truncated)

Title: Entecavir: new drug. Chronic hepatitis B: a last resort.
(1) For patients with HBeAg-positive chronic hepatitis B, peginterferon alfa-2a is the standard treatment used to prevent clinical complications and death. Lamivudine and adefovir dipivoxil, both taken orally, are second-line options. They can also be used as first-line treatments in patients with HBeAg-negative chronic hepatitis B if the adverse effects of prolonged peginterferon therapy are likely to pose a major problem. (2) Entecavir, a nucleoside analogue, is now marketed for oral treatment of chronic hepatitis B in adults. (3) Entecavir has not been compared with adefovir in clinical trials. Its evaluation is based mainly on three 48-week trials versus lamivudine.... (truncated)

Title: Chronic hepatitis B--treatment with nucleoside analogues.
Nucleot(s)ide analogues are making milestones in the treatment of chronic hepatitis B (CHB) as safe oral therapy. FDA approved lamivudine in adult patients in 1998, adefovir dipivoxil in 2002, and entecavir in March 2005. Lamivudine is effective in viral suppression, ALT normalization, and improvement in histology in both HBeAg positive and HBeAg negative / HBV DNA positive patients. HBeAg seroconversion rates correlate directly with pretreatment ALT levels at 18-30% after one year of therapy. Hepatitis flares may occur if lamivudine is stopped before HBeAg seroconversion. Lamivudine resistant YMDD mutants emerge at a rate of 15-20% per year of therapy; often... (truncated)

MESH:C511194 - ecallantide

Summary:

LLM Prediction Score	0.087 (normalized)
LLM Confidence Score	0.870
Golden Answer (Severity Class)	0.0 (normalized)
Prediction Error	0.087

Retrieved Context:

Title: Long-term Prophylaxis with Androgens in the management of Hereditary Angioedema (HAE) in emerging countries.
Hereditary angioedema (HAE) is a rare autosomal dominant genetic disease characterized by repetitive subcutaneous or submucosal angioedema, activation of the kinin system, and increased vascular permeability. C1-inhibitor (C1-INH) deficiency, the main mechanism of HAE pathogenesis, occurs when abnormal activation of plasma kallikrein, bradykinin, and factor XII, or mutation of genes such as SERPING1 cause quantitative or functional C1-INH defects. Although androgens are not approved for HAE treatment in many countries, they are widely used in China and Brazil to reduce the frequency and severity of HAE attacks. The long-term adverse effects of androgen treatment are concerning for both physicians and... (truncated)

Title: Emerging concepts in the diagnosis and treatment of patients with undifferentiated angioedema.
Angioedema is a sudden, transient swelling of well-demarcated areas of the dermis, subcutaneous tissue, mucosa, and submucosal tissues that can occur with or without urticaria. Up to 25% of people in the US will experience an episode of urticaria or angioedema during their lifetime, and many will present to the emergency department with an acute attack. Most cases of angioedema are attributable to the vasoactive mediators histamine and bradykinin. Histamine-mediated (allergic) angioedema occurs through a type I hypersensitivity reaction, whereas bradykinin-mediated (non-allergic) angioedema is iatrogenic or hereditary in origin.Although their clinical presentations bear similarities, the treatment algorithm for histamine-mediated angioedema... (truncated)

Title: Urticaria and angioedema.
Urticaria (hives) is a common disorder that often presents with angioedema (swelling that occurs beneath the skin). It is generally classified as acute, chronic or physical. Second-generation, non-sedating H1-receptor antihistamines represent the mainstay of therapy for both acute and chronic urticaria. Angioedema can occur in the absence of urticaria, with angiotensin-converting enzyme (ACE) inhibitor-induced angioedema and idiopathic angioedema being the more common causes. Rarer causes are hereditary angioedema (HAE) or acquired angioedema (AAE). Although the angioedema associated with these disorders is often self-limited, laryngeal involvement can lead to fatal asphyxiation in some cases. The management of HAE and AAE involves... (truncated)

Title: Computational Models Using Multiple Machine Learning Algorithms for Predicting Drug Hepatotoxicity with the DILIrank Dataset.
Drug-induced liver injury (DILI) remains one of the challenges in the safety profile of both authorized and candidate drugs, and predicting hepatotoxicity from the chemical structure of a substance remains a task worth pursuing. Such an approach is coherent with the current tendency for replacing non-clinical tests with in vitro or in silico alternatives. In 2016, a group of researchers from the FDA published an improved annotated list of drugs with respect to their DILI risk, constituting "the largest reference drug list ranked by the risk for developing drug-induced liver injury in humans" (DILIrank). This paper is one of the... (truncated)

Title: What would Sérgio Ferreira say to your physician in this war against COVID-19: How about kallikrein/kinin system?
Coronavirus disease 2019 (COVID-19) is an infectious disease with fast spreading all over the world caused by the SARS-CoV-2 virus which can culminate in a severe acute respiratory syndrome by the injury caused in the lungs. However, other organs can be also damaged. SARS-CoV-2 enter into the host cells using the angiotensin-converting enzyme 2 (ACE2) as receptor, like its ancestor SARS-CoV. ACE2 is then downregulated in lung tissues with augmented serum levels of ACE2 in SARS-CoV-2 patients. Interestingly, ACE2⁺ organs reveal the symptomatic repercussions, which are signals of the infection such as dry cough, shortness of breath, heart failure, liver... (truncated)

MESH:D002034 - bumetanide

Summary:

LLM Prediction Score	0.288 (normalized)
LLM Confidence Score	0.980
Golden Answer (Severity Class)	0.375 (normalized)
Prediction Error	0.087

Retrieved Context:

Title: Bumetanide increases manganese accumulation in the brain of rats with liver damage.
Hepatic encephalopathy is a common complication in cases of liver damage; it results from several factors, including the accumulation of toxic substances in the brain, e.g. manganese, ammonia and glutamine. We have previously reported that manganese favors ammonia and glutamine accumulation in the brain of cirrhotic rats, and we suggested that such effect could be mediated by manganese-elicited activation of the NKCC1 (Na(+)/K(+)/2Cl(-) cotransporter 1). To test this hypothesis, we used bumetanide, an NKCC1 blocker prescribed to treat ascites in cirrhotic patients; we expected that if NKCC1 was responsible for manganese-mediated ammonia buildup and the subsequent glutamine accumulation, bumetanide could... (truncated)

Title: Lack of effect of bumetanide on body potassium content in hypertension.
1 Twelve patients with mild hypertension were treated with bumetanide for a six-month period. No evidence was found of hypokalaemia or decreased total exchangeable potassium in subjects with or without additional potassium supplements. 2 Bumetanide was well tolerated by all patients. It caused hyperuricaemia but no episodes of gout occurred. Minor abnormalities of liver function were noted. 3 Bumetanide did not have a sustained antihypertensive action.

Title: Bumetanide. A review of its pharmacodynamic and pharmacokinetic properties and therapeutic use.
Bumetanide is a potent 'loop' diuretic for the treatment of oedema associated with congestive heart failure, hepatic and renal diseases, acute pulmonary congestion and premenstrual syndrome and in forced diuresis during and after surgery. Bumetanide may be given orally, intravenously or intramuscularly and produces a rapid and marked diuresis, and increased urinary excretion of sodium, chloride and other electrolytes (within 30 minutes) which persists for 3 to 6 hours. Its principal site of action is on the ascending limb of the loop of Henle, with a secondary action on the proximal tubule. Pharmacologically, bumetanide is about 40-fold more potent than... (truncated)

Title: Reversible Fulminant Hepatitis Secondary to Cocaine in the Setting of β-Blocker Use.
<i>Background</i>. Fulminant hepatitis is acute hepatic injury with severe decline in hepatic function manifested by encephalopathy, hypercoagulable state, jaundice, renal failure, hypoglycemia, or a constellation of these symptoms in patients without preexisting liver disease. Etiologies include viral infections, hepatotoxic drugs, autoimmune diseases, vaso-occlusive diseases, sepsis, and malignant infiltration. <i>Case Report.</i> A 56-year-old man presented with acute heart failure in the setting of cocaine use. The patient subsequently developed fulminant hepatic failure manifested by acute hypoglycemia, elevated liver enzyme, and worsening liver function, which resolved over 1 week with supportive care. The patient was on β-blocker, which was stopped during the... (truncated)

Title: The pharmacokinetics and pharmacodynamics of the diuretic bumetanide in hepatic and renal disease.
1 Bumetanide (1 mg) was given orally and intravenously to a group of patients with chronic renal failure (n = 6) and to another group with cirrhosis of the liver (n = 8). 2 The pharmacokinetics, using a two-compartment model, and the pharmacodynamics of the drug in these patients were compared with those previously obtained for normal subjects. 3 In the renal group serum bumetanide concentrations were higher than for the normal subjects and the terminal half-lives were significantly prolonged (P less than 0.001). A decreased whole body clearance was attributable to a low renal clearance of drug, the non-renal... (truncated)

MESH:C021139 - anagrelide

Summary:

LLM Prediction Score	0.461 (normalized)
LLM Confidence Score	0.960
Golden Answer (Severity Class)	0.375 (normalized)
Prediction Error	0.086

Retrieved Context:

Title: Platelet Anti-Aggregator Drowns the Heart.
Anagrelide is a drug used for treatment of essential thrombocytosis especially when conventional therapy is insufficient. Adverse effects associated with anagrelide are palpitation, liver toxicity, renal failure and in few cases pericardial effusion. We here report a rare case of anagrelide induced pericardial effusion.

Title: [Unclear liver fibrosis in a 42-year-old patient with polycythemia vera].
A 42-year-old patient was admitted to hospital because of ascites and polyglobulia. Laboratory tests revealed reduced liver function and a significant elevation of all three hematopoietic cell lines. Liver fibrosis and polycythemia vera were diagnosed by histologic examination. The most frequent causes for liver fibrosis were serologically excluded. Ultrasound combined with Doppler imaging revealed an obstruction of the right hepatic vein, which was indicative of Budd-Chiari syndrome. BCS can occur under fulminant and nonfulminant conditions, which can result in progressive damage of the liver. Phlebotomy and combined therapy with low-dose aspirin and anagrelide achieved permanent reduction of the elevated blood... (truncated)

Title: The MDM2 antagonist idasanutlin in patients with polycythemia vera: results from a single-arm phase 2 study.
Idasanutlin, an MDM2 antagonist, showed clinical activity and a rapid reduction in JAK2 V617F allele burden in patients with polycythemia vera (PV) in a phase 1 study. This open-label phase 2 study evaluated idasanutlin in patients with hydroxyurea (HU)-resistant/-intolerant PV, per the European LeukemiaNet criteria, and phlebotomy dependence; prior ruxolitinib exposure was permitted. Idasanutlin was administered once daily on days 1 through 5 of each 28-day cycle. The primary end point was composite response (hematocrit control and spleen volume reduction > 35%) in patients with splenomegaly and hematocrit control in patients without splenomegaly at week 32. Key secondary end points... (truncated)

Title: Hydroxyurea associated ileocecal valve ulcer: evidence for causality.
Hydroxyurea is an antimetabolite drug that is commonly used in many hematological disorders. Ulcer formation in the gastrointestinal tract is a rare phenomenon associated with this drug. We report a case of a 73-year-old woman who was found to have an isolated ileocecal valve ulcer while on hydroxyurea 1 g daily for essential thrombocythemia. A comprehensive evaluation ruled out all other causes. The cytoreductive therapy was switched to anagrelide and the endoscopic evaluation 6 months later showed complete healing of the ulcer. However, the hydroxyurea was resumed due to increasing platelet counts and intolerance to dose increments of the anagrelide.... (truncated)

Title: TJ-M2010-5, A self-developed MyD88 inhibitor, attenuates liver fibrosis by inhibiting the NF-κB pathway.
Liver fibrosis is the result of most chronic inflammatory liver damage and seriously endangers human health. However, no drugs have been approved to treat this disease. Previous studies showed that the Toll-like receptors (TLRs)/myeloid differentiation factor-88 (MyD88)/nuclear factor-κB (NF-κB) pathway plays a key role in liver fibrosis. TJ-M2010-5 is a self-developed small molecule MyD88 inhibitor, which has been proven to have a good protective effect in a variety of inflammatory disease models. In the present study, to investigate the anti-fibrotic effect of TJ-M2010-5, mice were injected with carbon tetrachloride (CCl₄) in vivo and LX2 cells (a human hepatic stellate cell... (truncated)

MESH:D000069603 - sevelamer

Summary:

LLM Prediction Score	0.086 (normalized)
LLM Confidence Score	0.980
Golden Answer (Severity Class)	0.0 (normalized)
Prediction Error	0.086

Retrieved Context:

Title: New strategies in treatment of mineral and bone disorders and associated cardiovascular disease in patients with chronic kidney disease.
Mineral and bone disorders in chronic kidney disease (CKD) patients along with the use of calcium-based phosphate binders may result in vascular calcification (VC) development and associated increase in cardiovascular diseases (CVD) mortality. A few treatment modalities to control hyperphosphatemia, VC and CVD over the years have failed. Recently appeared calcium-aluminum free phosphate binders (sevelamer hydrochloride and lanthanum carbonate) have reduced hypercalcemic adverse events compared to calcium-based binders, although beneficial effects on CVD outcome to justify further widespread utilization of these agents in CKD patients are not reported so far. At present long-term safety of lanthanum administration has been challenged... (truncated)

Title: Phosphate binding therapy in dialysis patients: focus on lanthanum carbonate.
Hyperphosphatemia is an inevitable consequence of end stage chronic kidney disease and is present in the majority of dialysis patients. Recent observational data has associated hyperphosphatemia with increased cardiovascular mortality among dialysis patients. Dietary restriction of phosphate and current dialysis prescription practices are not enough to maintain serum phosphate levels within the recommended range so that the majority of dialysis patients require oral phosphate binders. Unfortunately, conventional phosphate binders are not reliably effective and are associated with a range of limitations and side effects. Aluminium-containing agents are highly efficient but no longer widely used because of well established and proven... (truncated)

Title: Western diet-induced increase in colonic bile acids compromises epithelial barrier in nonalcoholic steatohepatitis.
There is compelling evidence implicating intestinal permeability in the pathogenesis of nonalcoholic steatohepatitis (NASH), but the underlying mechanisms remain poorly understood. Here we examined the role of bile acids (BA) in western diet (WD)-induced loss of colonic epithelial barrier (CEB) function in mice with a genetic impairment in intestinal epithelial barrier function, junctional adhesion molecule A knockout mice, F11r^{-/-} . WD-fed knockout mice developed severe NASH, which was associated with increased BA concentration in the cecum and loss of CEB function. Analysis of cecal BA composition revealed selective increases in primary unconjugated BAs in the WD-fed mice, which correlated with... (truncated)

Title: Bile Acid Sequestrant, Sevelamer Ameliorates Hepatic Fibrosis with Reduced Overload of Endogenous Lipopolysaccharide in Experimental Nonalcoholic Steatohepatitis.
Despite the use of various pharmacotherapeutic strategies, fibrosis due to nonalcoholic steatohepatitis (NASH) remains an unsatisfied clinical issue. We investigated the effect of sevelamer, a hydrophilic bile acid sequestrant, on hepatic fibrosis in a murine NASH model. Male C57BL/6J mice were fed a choline-deficient, L-amino acid-defined, high-fat (CDHF) diet for 12 weeks with or without orally administered sevelamer hydrochloride (2% per diet weight). Histological and biochemical analyses revealed that sevelamer prevented hepatic steatosis, macrophage infiltration, and pericellular fibrosis in CDHF-fed mice. Sevelamer reduced the portal levels of total bile acid and inhibited both hepatic and intestinal farnesoid X receptor activation.... (truncated)

Title: A Case of Accidental Isoniazid Overdose Presenting With Nonspecific Symptoms.
A 68-year-old male with a history of end-stage renal disease and latent tuberculosis on isoniazid (INH), and no psychiatric history presented with a five-day history of anorexia, fatigue, and nausea. Physical exam in the emergency department was notable for somnolence, right upper extremity tremor, and diffuse abdominal pain. Initial workup revealed an anion gap metabolic acidosis with elevated lactate, prompting admission to the general ward for empiric IV antibiotics for suspected bacteremia from his permacath. Within a few hours of admission, he became increasingly encephalopathic and had two episodes of copious hematemesis. Repeat studies revealed a cholestatic pattern of liver... (truncated)

MESH:D000077722 - ceftibuten

Summary:

LLM Prediction Score	0.290 (normalized)
LLM Confidence Score	0.930
Golden Answer (Severity Class)	0.375 (normalized)
Prediction Error	0.085

Retrieved Context:

Title: Antibiotic-Induced Liver Injury in Paediatric Outpatients: A Case-Control Study in Primary Care Databases.
Antibiotics are the most commonly prescribed drug class in children. Real-world data mining on the paediatric population showed potential associations between antibiotic use and acute liver injury.

Title: Pharyngitis.
This article reviews the commonly encountered agents causing acute inflammation of the pharynx and tonsils, with special attention to a practical approach for identifying and dealing with the group A beta-hemolytic streptococcus. Ubiquitous viral agents such as Epstein-Barr virus, rhinovirus, and adenovirus are reviewed. Some agents such as group A beta-hemolytic streptococcus and Epstein-Barr virus are susceptible to treatment. Additionally, unusual infectious agents and noninfectious causes of pharyngitis are enumerated.

Title: Treatment of enteric fever (typhoid and paratyphoid fever) with cephalosporins.
Typhoid and paratyphoid (enteric fever) are febrile bacterial illnesses common in many low- and middle-income countries. The World Health Organization (WHO) currently recommends treatment with azithromycin, ciprofloxacin, or ceftriaxone due to widespread resistance to older, first-line antimicrobials. Resistance patterns vary in different locations and are changing over time. Fluoroquinolone resistance in South Asia often precludes the use of ciprofloxacin. Extensively drug-resistant strains of enteric fever have emerged in Pakistan. In some areas of the world, susceptibility to old first-line antimicrobials, such as chloramphenicol, has re-appeared. A Cochrane Review of the use of fluoroquinolones and azithromycin in the treatment of enteric... (truncated)

Title: Cephalosporin Allergy: Current Understanding and Future Challenges.
Cephalosporins are commonly used antibiotics both in hospitalized patients and in outpatients. Hypersensitivity reactions to cephalosporins are becoming increasingly common with a wide range of immunopathologic mechanisms. Cephalosporins are one of the leading causes for perioperative anaphylaxis and severe cutaneous adverse reactions. Patients allergic to cephalosporins tend to tolerate cephalosporins with disparate R1 side chains but may react to other beta-lactams with common R1 side chains. Skin testing for cephalosporins has not been well validated but appears to have a good negative predictive value for cephalosporins with disparate R1 side chains. In vitro tests including basophil activation tests have lower... (truncated)

Title: Plasma membrane transporters in modern liver pharmacology.
The liver plays a crucial role in the detoxification of drugs used in the treatment of many diseases. The liver itself is the target for drugs aimed to modify its function or to treat infections and tumours affecting this organ. Both detoxification and pharmacological processes occurring in the liver require the uptake of the drug by hepatic cells and, in some cases, the elimination into bile. These steps have been classified as detoxification phase 0 and phase III, respectively. Since most drugs cannot cross the plasma membrane by simple diffusion, the involvement of transporters is mandatory. Several members of the... (truncated)

MESH:D005492 - folic acid

Summary:

LLM Prediction Score	0.083 (normalized)
LLM Confidence Score	0.990
Golden Answer (Severity Class)	0.0 (normalized)
Prediction Error	0.083

Retrieved Context:

Title: Folic acid supplementation reduces oxidative stress and hepatic toxicity in rats treated chronically with ethanol. Folate deficiency and hyperhomocysteinemia are found in most patients with alcoholic liver disease. Oxidative stress is one of the most important mechanisms contributing to homocysteine (Hcy)-induced tissue injury. However it has not been examined whether exogenous administration of folic acid attenuates oxidative stress and hepatic toxicity. The aim of this study was to investigate the in vivo effect of folic acid supplementation on oxidative stress and hepatic toxicity induced by chronic ethanol consumption. Wistar rats (n = 32) were divided into four groups and fed 0%, 12%, 36% ethanol, or 36% ethanol plus folic acid (10 mg folic acid/L) diets.... (truncated)

Title: High folic acid consumption leads to pseudo-MTHFR deficiency, altered lipid metabolism, and liver injury in mice.
Increased consumption of folic acid is prevalent, leading to concerns about negative consequences. The effects of folic acid on the liver, the primary organ for folate metabolism, are largely unknown. Methylenetetrahydrofolate reductase (MTHFR) provides methyl donors for S-adenosylmethionine (SAM) synthesis and methylation reactions.

Title: "Hepatic toxicity by methotrexate with weekly single doses associated with folic acid in rheumatoid and psoriatic arthritis. What is its real frequency?"
Liver injury caused by methotrexate (MTX) has mostly been investigated without applying criteria for the assessment of causality of drug induced liver injury (DILI). Hence, the existence of DILI by MTX in many cases is debatable. This study aimed to describe the frequency and characteristics of liver injury caused by MTX, applying DILI diagnostic criteria.

Title: The use of folates concomitantly with low-dose pulse methotrexate.
Toxicities related to low-dose weekly methotrexate are largely due to its antifolate properties. Preexisting folate deficiency is associated with methotrexate toxicity in some patients. At the onset of methotrexate therapy and throughout therapy, the physician should be vigilant regarding one or more nutrient deficiencies. A multivitamin and, where appropriate, specific daily folic acid supplements should be employed. The only regimen known presently (through controlled trials) to treat side effects is the low-dose folinic acid (leucovorin) protocol outlined herein. Folic acid may be helpful to treat mild gastrointestinal symptoms. Folinic acid supplementation should be considered prophylactically in those requiring methotrexate who... (truncated)

Title: N-acetylcysteine ameliorates methotrexate-induced oxidative liver damage in rats.
Methotrexate (MTX), a folic acid antagonist, is widely used as a cytotoxic chemotherapeutic agent for malignancies as well as in the treatment of various inflammatory diseases. The efficacy of this agent is often limited by severe side effects and toxic conditions. The present study was undertaken to determine whether N-acetylcysteine (NAC), as a potent antioxidant compound, could ameliorate MTX-induced oxidative liver damage.

MESH:C069987 - pamabrom

Summary:

LLM Prediction Score	0.082 (normalized)
LLM Confidence Score	0.900
Golden Answer (Severity Class)	0.0 (normalized)
Prediction Error	0.082

Retrieved Context:

Title: An in vitro coculture system of human peripheral blood mononuclear cells with hepatocellular carcinoma-derived cells for predicting drug-induced liver injury.
Preventing clinical drug-induced liver injury (DILI) remains a major challenge, because DILI develops via multifactorial mechanisms. Immune and inflammatory reactions are considered important mechanisms of DILI; however, biomarkers from in vitro systems using immune cells have not been comprehensively studied. The aims of this study were (1) to identify promising biomarker genes for predicting DILI in an in vitro coculture model of peripheral blood mononuclear cells (PBMCs) with a human liver cell line, and (2) to evaluate these genes as predictors of DILI using a panel of drugs with different clinical DILI risk. Transcriptome-wide analysis of PBMCs cocultured with HepG2... (truncated)

Title: Fixed Dose Versus Loose Dose: Analgesic Combinations.
Combinations of drugs may be fixed (two or more entities in a single product) or loose (two or more agents taken together but as individual agents) to help address multimechanistic pain. The use of opioids plus nonopioids can result in lower opioid consumption without sacrificing analgesic benefits. Drug combinations may offer additive or synergistic benefits. A variety of fixed-dose combination products are available on the market such as diclofenac plus thiocolchicoside, acetaminophen and caffeine, acetaminophen and opioid, ibuprofen and acetaminophen, tramadol and acetaminophen, and others. Fixed-dose combination products offer predictable pharmacokinetics and pharmacodynamics, known adverse events, and can reduce the... (truncated)

MESH:D002433 - cefaclor

Summary:

LLM Prediction Score	0.456 (normalized)
LLM Confidence Score	0.990
Golden Answer (Severity Class)	0.375 (normalized)
Prediction Error	0.081

Retrieved Context:

Title: Second-Generation Cephalosporins-Associated Drug-Induced Liver Disease: A Study in VigiBase with a Focus on the Elderly.
The objective of this study was to characterize individual case safety reports (ICSRs) and adverse drug reactions (ADRs) related to second-generation cephalosporins and resulting in hepatobiliary disorders, in VigiBase, WHO global database.

Title: Herbal products: benefits, limits, and applications in chronic liver disease.
Complementary and alternative medicine soughts and encompasses a wide range of approaches; its use begun in ancient China at the time of Xia dynasty and in India during the Vedic period, but thanks to its long-lasting curative effect, easy availability, natural way of healing, and poor side-effects it is gaining importance throughout the world in clinical practice. We conducted a review describing the effects and the limits of using herbal products in chronic liver disease, focusing our attention on those most known, such as quercetin or curcumin. We tried to describe their pharmacokinetics, biological properties, and their beneficial effects (as... (truncated)

Title: Identification and Characterization of Cefazolin-Induced Liver Injury.
Cephalosporin antibiotics are popular because they have a broad spectrum of activity and are generally well tolerated; however, cephalosporin-induced liver injury is considered rare. We describe a new syndrome associated with a single intravenous dose of cefazolin and the clinical features of cephalosporin-induced liver injury.

Title: The risk of acute liver injury among users of antibiotic medications: a comparison of case-only studies.
The aims of this study were two-fold: (i) to investigate the effect of exposure to antibiotic agents on the risk of acute liver injury using a self-controlled case series and case-crossover study and (ii) to compare the results between the case-only studies.

Title: Hepatoprotective activities of picroliv, curcumin, and ellagic acid compared to silymarin on carbon-tetrachloride-induced liver toxicity in mice.
To evaluate the hepatoprotective activity of active phytochemicals, picroliv, curcumin, and ellagic acid in comparison to silymarin in the mice model of carbon tetrachloride ($CCl_{(4)}$) induced liver toxicity. In addition, attempts were made to elucidate their possible mechanism(s) of action.

MESH:D012633 - secretin

Summary:

LLM Prediction Score	0.080 (normalized)
LLM Confidence Score	0.990
Golden Answer (Severity Class)	0.0 (normalized)
Prediction Error	0.080

Retrieved Context:

Title: Therapeutic effect of secretin in patients with jaundice; double-blind placebo-controlled multicentric trial.
Secretin, a gastrointestinal hormone, has been shown to have a potent choleretic effect. Having already obtained some beneficial effects with secretin in patients with intrahepatic cholestasis, we sought to confirm its effects in a double-blind placebo-controlled study in patients with mild jaundice after acute or during chronic hepatitis, where total bilirubin level was in excess of 4.0 mg/dl for 3 days or more. Patients with primary biliary cirrhosis (PBC), primary sclerosing cholangitis (PSC), and familiar hyperbilirubinemia were excluded from the study. Ninety-three patients were included in this analysis, but the final evaluation covered 69 of them. No statistically significant differences... (truncated)

Title: Knockout of secretin receptor reduces biliary damage and liver fibrosis in Mdr2^{-/-} mice by diminishing senescence of cholangiocytes.
Secretin receptor (SR), only expressed by cholangiocytes, plays a key role in the regulation of biliary damage and liver fibrosis. The aim of this study was to determine the effects of genetic depletion of SR in Mdr2^{-/-} mice on intrahepatic biliary mass, liver fibrosis, senescence, and angiogenesis. 12 wk SR^{-/-}, Mdr2^{-/-}, and SR^{-/-}/Mdr2^{-/-} mice with corresponding wild-type mice were used for the in vivo studies. Immunohistochemistry or immunofluorescence was performed in liver sections for (i) biliary expression of SR; (ii) hematoxylin and eosin; (iii) intrahepatic biliary mass by CK-19; (iv) fibrosis by Col1a1 and α-SMA; (v) senescence by SA-β-gal and... (truncated)

Title: [Changes in pancreatic secretion in alcoholic liver disease].
By means of consecutive pancreatic stimulation, we have investigated the presence of changes of pancreatic function in alcoholic patients, with and without alcoholic liver disease, in order to detect functional alterations and possible association of hepatic and pancreatic disease. The patients were 49 chronic alcoholics (8 patients without liver disease, 11 hepatic steatosis, 9 alcoholic hepatitis and 21 alcoholic cirrhosis) and 15 non alcoholic subjects (8 normal controls and 7 cases of non alcoholic cirrhosis). In all the cases two consecutive stimulations were carried out: first with secretin and cholecystokinin (CCK) and second with CCK alone. The total volume and... (truncated)

Title: [Biliary secretion. Significance of hypercholeresis from the surgical point of view].
In patients carrying a Kehr tube choledochostomy, the surgeon may observe, but rarely, important hypercholeresis, non-bile-acid dependent, in people with severe advanced chronic hepatic diseases or persistent cholestasis. The amount of bile flowing to the outside can reach two liters and more daily. We have seen this in two patients with compensated hepatic disorders and in another suffering from light cholestasis produced by choledocholithiasis. We have also found the same in three other people with liver and the main biliary extrahepatic tract completely normal. This hypercholeresis is continuous and subject to rapid increases relative to the ingestion of food. Such... (truncated)

Title: Secretin/secretin receptor signaling mediates biliary damage and liver fibrosis in early-stage primary biliary cholangitis.
Primary biliary cholangitis (PBC) primarily targets cholangiocytes and is characterized by liver fibrosis and biliary proliferation. Activation of the secretin (Sct)/secretin receptor (SR) axis, expressed only by cholangiocytes, increases biliary proliferation, liver fibrosis, and bicarbonate secretion. We evaluated the effectiveness of SR antagonist treatment for early-stage PBC. Male and female dominant-negative TGF-β receptor II (dnTGF-βRII) (model of PBC) and wild-type mice at 12 wk of age were treated with saline or the SR antagonist, Sec 5-27, for 1 wk. dnTGF-βRII mice expressed features of early-stage PBC along with enhanced Sct/SR axis activation and Sct secretion. dnTGF-βRII mice had increased biliary... (truncated)

MESH:D019379 - teriparatide

Summary:

LLM Prediction Score	0.080 (normalized)
LLM Confidence Score	0.980
Golden Answer (Severity Class)	0.0 (normalized)
Prediction Error	0.080

Retrieved Context:

Title: Safety and effectiveness of daily teriparatide in a prospective observational study in patients with osteoporosis at high risk of fracture in Japan: final report.
This postmarketing surveillance study assessed the safety and effectiveness of teriparatide in patients with osteoporosis at high risk of fracture in Japan. The patients received teriparatide 20 μg daily by subcutaneous injection, for a maximum of 24 months. Safety and effectiveness analyses were based on data from 1,847 patients who were predominantly female (92.6%) with a mean age of 75.4 years. A total of 157 adverse drug reactions (ADRs) were reported in 140 (7.58%) patients; the most common ADRs were hyperuricemia, nausea, and dizziness. Only six (0.32%) patients reported serious ADRs, the most common being nausea (two patients; 0.1%). Persistence... (truncated)

Title: Raised CA19.9 and hepatic space occupying lesion after teriparatide therapy in a case of polyostotic fibrous dysplasia.
No abstract available.

Title: Hypercalcemia due to Primary Hepatic Lymphoma.
A 65-year-old female with a history of mixed connective tissue disease and pulmonary fibrosis on azathioprine, hydroxychloroquine, and prednisone (osteoporosis on teriparatide) presented with a 1-month history of hypercalcemia. After discontinuation of teriparatide, the patient's hypercalcemia persisted. Further evaluation revealed primary hepatic lymphoma as the source of her hypercalcemia.

Title: Osteoporosis in the aging male: treatment options.
In elderly women, loss in bone mass and micro-architectural changes are generally attributed to the onset of menopause. Men do not experience menopause, they do, however, experience age-related acceleration in bone loss and micro-architecture deterioration. The incidence of osteoporotic fractures in elderly men, just as in aged women, increases exponen-tially with age; the rise in men, however, is some 5-10 years later than in women. Up to 50% of male osteoporotics have no identifiable etiology; however elderly males have much higher likelihood of having an identifiable secondary cause than younger men. Therefore, clinical and laboratory evaluation of aged male osteoporotics... (truncated)

Title: Association of Urinary Pentosidine Levels With the Risk of Fractures in Patients With Severe Osteoporosis: The Japanese Osteoporosis Intervention Trial-05 (JOINT-05).
Associations between urinary pentosidine, one of the advanced glycation end products in collagen, and the risk of fracture in patients with severe osteoporosis are unknown. In this study, we investigated whether the urinary pentosidine level is associated with the incidence of morphometric vertebral fracture and nonvertebral fracture using data of a randomized, controlled trial, JOINT-05. JOINT-05 enrolled Japanese women aged 75 years or older with primary osteoporosis. Patients were randomly assigned (1:1) to receive sequential therapy (teriparatide followed by alendronate) or monotherapy with alendronate for 120 weeks. Incidences of vertebral and nonvertebral fractures were assessed morphologically. During treatment, urinary levels of pentosidine... (truncated)

MESH:D010480 - perhexiline

Summary:

LLM Prediction Score	0.921 (normalized)
LLM Confidence Score	0.950
Golden Answer (Severity Class)	1.0 (normalized)
Prediction Error	0.079

Retrieved Context:

Title: Study of the roles of cytochrome P450 (CYPs) in the metabolism and cytotoxicity of perhexiline.
Perhexiline is a prophylactic antianginal agent developed in the 1970s. Although, therapeutically, it remained a success, the concerns of its severe adverse effects including hepatotoxicity caused the restricted use of the drug, and eventually its withdrawal from the market in multiple countries. In the clinical setting, cytochrome P450 (CYP) 2D6 is considered as a possible risk factor for the adverse effects of perhexiline. However, the role of CYP-mediated metabolism in the toxicity of perhexiline, particularly in the intact cells, remains unclear. Using our previously established HepG2 cell lines that individually express 14 CYPs (1A1, 1A2, 1B1, 2A6, 2B6, 2C8, 2C9,... (truncated)

Title: Perhexiline maleate-induced cirrhosis.
The authors report the cases of 2 patients who died from cirrhosis after receiving perhexiline maleate, a drug widely used in Europe for the treatment of angina pectoris. Perhexiline maleate had been ingested for 24 and 28 mo, respectively. Manifestations of cirrhosis included jaundice, hepatic encephalopathy, ascites, and portal hypertension. Associated manifestations of intolerance to perhexiline maleate included peripheral neuropathy in 1 patient and marked weight loss in both. Histologic lesions resembled those observed in patients with alcoholic liver disease. Ultrastructural lesions included numerous enlarged lysosomes containing myeloid figures. Histochemical stains demonstrated increased phospholipid content of the hepatocytes. These findings... (truncated)

Title: Perhexiline.
Perhexiline, 2-(2,2-dicyclohexylethyl)piperidine, was originally developed as an anti-anginal drug in the 1970s. Despite its success, its use diminished due to the occurrence of poorly understood side effects including neurotoxicity and hepatotoxicity in a small proportion of patients. Recently, perhexiline's mechanism of action and the molecular basis of its toxicity have been elucidated. Perhexiline reduces fatty acid metabolism through the inhibition of carnitine palmitoyltransferase, the enzyme responsible for mitochondrial uptake of long-chain fatty acids. The corresponding shift to greater carbohydrate utilization increases myocardial efficiency (work done per unit oxygen consumption) and this oxygen-sparing effect explains its antianginal efficacy. Perhexiline's side effects... (truncated)

Title: Impaired oxidation of debrisoquine in patients with perhexiline neuropathy.
The use of perhexiline maleate as an antianginal agent is occasionally associated with side effects, particularly neuropathy and liver damage. The reason why some individuals develop these toxic reactions is not clear, though some evidence suggests that they may result from impaired oxidative metabolism, due to genetic or hepatic factors, and consequential accumulation of the drug in toxic concentrations. Drug oxidation was measured with an oxidation phenotyping procedure in 34 patients treated with perhexiline, 20 of whom had developed neuropathy and 14 of whom had not. Most of the 20 patients with neuropathy, but not the unaffected patients, showed an... (truncated)

Title: Enantioselectivity in the tissue distribution of perhexiline contributes to different effects on hepatic histology and peripheral neural function in rats.
Perhexiline, a chiral drug, is a potent antiischemic agent whose clinical utility is limited by hepatic and neural toxicities. It inhibits mitochondrial carnitine palmitoyltransferase-1, however, excessive inhibition predisposes toward tissue steatosis. This pilot study investigated the distribution of the two enantiomers and their toxicological potential. Dark Agouti rats (n = 4 per group) were administered vehicle or 200 mg/kg daily of racemic, (+)- or (-)-perhexiline maleate orally for 8 weeks. Plasma biochemical liver function tests and Von Frey assessments of peripheral neural function were performed. Hepatic and neuronal histology, including lipid and glycogen content, was assessed using electron microscopy. Concentrations... (truncated)

MESH:D010424 - pentobarbital

Summary:

LLM Prediction Score	0.296 (normalized)
LLM Confidence Score	0.990
Golden Answer (Severity Class)	0.375 (normalized)
Prediction Error	0.079

Retrieved Context:

Title: Toxicity of alkyldihydrofurans to metabolically active organs in the mouse.
Alkylfurans inflict toxicity in several mammalian species to lung, liver and kidney. Organ specificity of the alkylfurans is a sensitive function of the nature of the alkyl group. To determine if this toxicity requires an aromatic ring in the compound, we synthesized 4-methyl-2,3-dihydrofuran, 4-ethyl-2,3-dihydrofuran and 4-pentyl-2,3-dihydrofuran and determined their toxicity to lung, liver and kidney in mice. Lung damage was evaluated by light microscopy and the incorporation of [14C]thymidine into lung DNA. The results indicated that 4-methyl-2,3-dihydrofuran and 4-ethyl-2,3-dihydrofuran were toxic to the lung whereas 4-pentyl-2,3-dihydrofuran did not produce lung toxicity. Histological examination of liver sections revealed that 4-ethyl-2,3-dihydrofuran induced... (truncated)

Title: Methylene dianiline: acute toxicity and effects on biliary function.
4,4'-Methylene dianiline (4,4'-diaminodiphenylmethane, DAPM), which is used in the polymer industry, causes hepatobiliary damage in exposed humans. Our objectives were to characterize the acute toxicity of DAPM in liver, particularly on secretion of biliary constituents and on biliary epithelial cell gamma-glutamyl transpeptidase (GGT) activity. Biliary cannulas were positioned in Sprague-Dawley male rats under pentobarbital anesthesia. After 1 hr of control bile collection, each rat was given 250 mg DAPM/kg (50 mg/ml) po in 35% ethanol or 35% ethanol only; bile was collected for a further 4 hr. Groups of rats were also examined for liver injury and biliary function at... (truncated)

Title: Hepatoprotective Effects of Crude Stem Bark Extracts and Solvent Fractions of <i>Cordia africana</i> against Acetaminophen-Induced Liver Injury in Rats.
<i>Cordia africana</i> Lam (Boraginaceae) is widely used in Ethiopian folk medicine for the treatment of different types of liver disorders. Thus, this study aimed to investigate the hepatoprotective effects of an aqueous (CAAE), 80% methanol extracts of <i>C. africana</i> stem bark (CAME), and the solvent fractions of the methanol extract against acetaminophen (APAP)-induced liver injury in rats. Acute toxicity test and APAP-induced lethality test were done on mice of either sex, while APAP dose selection test was done on female rats. Male rats were used for hepatoprotective experiments and the liver injury was induced using 2 g/kg APAP given orally. Serum... (truncated)

Title: Hepatoprotective Effect of Low Doses of Caffeine on CCl4-Induced Liver Damage in Rats.
Several studies have shown the hepatoprotective effect of the consumption of coffee and tea, which is mainly attributed to caffeine. Many experimental studies have demonstrated this effect; however, these studies used high caffeine doses that are not related to human consumption. The aim of this study was to evaluate the hepatoprotective effect of low doses of caffeine on carbon tetrachloride (CCl₄)-treated rats. Low doses of caffeine (CAFF) 5 and 10 mg/kg (CAFF5 and CAFF10) were evaluated in chronic liver damage induced by CCl₄ (0.75 mL/kg) in rats. CAFF treatment was administered once a day and CCl₄ administration was twice weekly for 10... (truncated)

Title: Cannabidiol protects against hepatic ischemia/reperfusion injury by attenuating inflammatory signaling and response, oxidative/nitrative stress, and cell death.
Ischemia/reperfusion (I/R) is a pivotal mechanism of liver damage after liver transplantation or hepatic surgery. We have investigated the effects of cannabidiol (CBD), the nonpsychotropic constituent of marijuana, in a mouse model of hepatic I/R injury. I/R triggered time-dependent increases/changes in markers of liver injury (serum transaminases), hepatic oxidative/nitrative stress (4-hydroxy-2-nonenal, nitrotyrosine content/staining, and gp91phox and inducible nitric oxide synthase mRNA), mitochondrial dysfunction (decreased complex I activity), inflammation (tumor necrosis factor α (TNF-α), cyclooxygenase 2, macrophage inflammatory protein-1α/2, intercellular adhesion molecule 1 mRNA levels; tissue neutrophil infiltration; nuclear factor κB (NF-κB) activation), stress signaling (p38MAPK and JNK), and cell death... (truncated)

MESH:D008034 - lincomycin

Summary:

LLM Prediction Score	0.453 (normalized)
LLM Confidence Score	0.970
Golden Answer (Severity Class)	0.375 (normalized)
Prediction Error	0.078

Retrieved Context:

Title: Intestinal flora imbalance promotes alcohol-induced liver fibrosis by the TGFβ/smad signaling pathway in mice.
Intestinal flora performs a crucial role in human health and its imbalance may cause numerous pathological changes. The liver can also affect the intestinal function through bile secretion via the enterohepatic cycle. The pathophysiological association between the gut and the liver is described as the gut-liver axis. The present study investigated the role of intestinal flora in alcohol-induced liver fibrosis. A total of 36 C57 mice were randomly and equally divided into 3 different dietary regimes: Group I (alcohol injury; received alcohol); group II (alcohol injury with flora imbalance; received alcohol plus lincomycin hydrochloride) and group III (alcohol injury with... (truncated)

Title: Baicalin Alleviates Short-Term Lincomycin-Induced Intestinal and Liver Injury and Inflammation in Infant Mice.
The adverse effects of short-term megadose of antibiotics exposure on the gastrointestinal and liver tissue reactions in young children have been reported. Antibiotic-induced intestinal and liver reactions are usually unpredictable and present a poorly understood pathogenesis. It is, therefore, necessary to develop strategies for reducing the adverse effects of antibiotics. Studies on the harm and rescue measures of antibiotics from the perspective of the gut-liver system are lacking. Here, we demonstrate that lincomycin exposure reduced body weight, disrupted the composition of gut microbiota and intestinal morphology, triggered immune-mediated injury and inflammation, caused liver dysfunction, and affected lipid metabolism. However, baicalin... (truncated)

Title: Soil Behaviour of the Veterinary Drugs Lincomycin, Monensin, and Roxarsone and Their Toxicity on Environmental Organisms.
Lincomycin, monensin, and roxarsone are commonly used veterinary drugs. This study investigated their behaviours in different soils and their toxic effects on environmental organisms. Sorption and mobility analyses were performed to detect the migration capacity of drugs in soils. Toxic effects were evaluated by inhibition or acute toxicity tests on six organism species: algae, plants, daphnia, fish, earthworms and quails. The log K_d values (Freundlich model) of drugs were: lincomycin in laterite soil was 1.82; monensin in laterite soil was 2.76; and roxarsone in black soil was 1.29. The R_f value of lincomycin, roxarsone, monensin were 0.4995, 0.4493 and 0.8348... (truncated)

Title: Necrotic enteritis in broiler chickens: disease characteristics and prevention using organic antibiotic alternatives - a comprehensive review.
In line with the substantial increase in the broiler industry worldwide, Clostridium perfringens-induced necrotic enteritis (NE) became a continuous challenge leading to high economic losses, especially after banning antimicrobial growth promoters in feeds by many countries. The disease is distributed worldwide in either clinical or subclinical form, causing a reduction in body weight or body weight gain and the feed conversion ratio, impairing the European Broiler Index or European Production Efficiency Factor. There are several predisposing factors in the development of NE. Clinical signs varied from inapparent signs in case of subclinical infection (clostridiosis) to obvious enteric signs (morbidity), followed... (truncated)

Title: Screening for biomarkers of liver injury induced by Polygonum multiflorum: a targeted metabolomic study.
Heshouwu (HSW), the dry roots of Polygonum multiflorum, a classical traditional Chinese medicine is used as a tonic for a wide range of conditions, particularly those associated with aging. However, it tends to be taken overdose or long term in these years, which has resulted in liver damage reported in many countries. In this study, the indicative roles of nine bile acids (BAs) were evaluated to offer potential biomarkers for HSW induced liver injury. Nine BAs including cholic acid (CA) and chenodeoxycholic acid (CDCA), taurocholic acid (TCA), glycocholic acid (GCA), glycochenodeoxycholic acid (GCDCA), deoxycholic acid (DCA), glycodeoxycholic acid (GDCA), ursodeoxycholic... (truncated)

MESH:C011677 - benoxaprofen

Summary:

LLM Prediction Score	0.924 (normalized)
LLM Confidence Score	0.650
Golden Answer (Severity Class)	1.0 (normalized)
Prediction Error	0.076

Retrieved Context:

Title: Effects of non-narcotic analgesics on the liver.
Serious hepatotoxicity is uncommon with the proper therapeutic use of non-narcotic analgesics but experience with new non-steroidal anti-inflammatory drugs (NSAIDs) is limited. Drugs such as ibufenac, fenclofenac and benoxaprofen were withdrawn from the market because of hepatotoxicity, and liver damage has been reported on occasion with virtually all non-narcotic analgesics. However, a clear pattern of toxicity with characteristic clinical, biochemical and histopathological abnormalities has emerged with relatively few. With the exception of acute hepatic necrosis following overdosage of paracetamol, little is known of the mechanisms of liver injury induced by non-narcotic analgesics. Involvement of the liver in a generalised drug... (truncated)

Title: Liver damage with non-narcotic analgesics.
Non-narcotic analgesics can produce a variety of hepatic lesions but clinically significant liver damage is uncommon with normal therapeutic use. The pattern of hepatotoxicity caused by the salicylates, non-steroidal anti-inflammatory drugs (NSAIDs), paracetamol (acetaminophen) and the pyrazolones differs but many of these drugs can cause generalised reactions which involve the liver. Depending on the drugs in question, the risks of liver injury may be conditioned by factors such as age, sex, dose and duration of treatment. Hepatotoxicity associated with the use of salicylates and most NSAIDs has been reported most often in females with collagen diseases but this may simply... (truncated)

Title: Non-steroidal anti-inflammatory drugs: What is the actual risk of liver damage?
Non-steroidal anti-inflammatory drugs (NSAIDs) constitute a family of drugs, which taken as a group, represents one of the most frequently prescribed around the world. Thus, not surprisingly NSAIDs, along with anti-infectious agents, list on the top for causes of Drug-Induced Liver Injury (DILI). The incidence of liver disease induced by NSAIDs reported in clinical studies is fairly uniform ranging from 0.29/100 000 [95% confidence interval (CI): 0.17-051] to 9/100 000 (95% CI: 6-15). However, compared with these results, a higher risk of liver-related hospitalizations was reported (3-23 per 100 000 patients). NSAIDs exhibit a broad spectrum of liver damage ranging... (truncated)

Title: Drug-induced liver injury in the elderly.
Historically, the elderly have been considered to be at increased risk for drug-induced liver injury (DILI). Animal studies have demonstrated changes in hepatic physiology that affect drug metabolism in the aging liver; however, there is no evidence that this leads to any appreciable deterioration of liver function in healthy older humans. Updated data from international DILI registries give us pause to consider whether the elderly are truly at increased risk to develop hepatic injury. Instead, hepatotoxicity in the elderly appears to be more a function of drug exposure, polypharmacy and drug-drug interactions. Isoniazid and benoxaprofen are the only two agents... (truncated)

Title: Ibuprofen Increases the Hepatotoxicity of Ethanol through Potentiating Oxidative Stress.
Over 30 million prescriptions of NSAIDs (non-steroidal anti-inflammatory drugs) are issued every year. Considering that these drugs are available without a prescription as over the counter (OTC) drugs, their use will be astronomical. With the increasing use of NSAIDs, their adverse effects are drawing attention. Especially, stomach bleeding, kidney toxicity, liver toxicity, and neurological toxicity are reported as common. Ibuprofen, one of the extensively used NSAIDs along with aspirin, can also induce liver toxicity, but few studies are addressing this point. Here we examined the liver toxicity of ibuprofen and investigated whether co-exposure to ethanol can manifest synergistic effects. We... (truncated)

MESH:D000639 - amitriptyline

Summary:

LLM Prediction Score	0.701 (normalized)
LLM Confidence Score	0.990
Golden Answer (Severity Class)	0.625 (normalized)
Prediction Error	0.076

Retrieved Context:

Title: Amitriptyline-induced prolonged cholestasis.
We report the case of a patient in whom amitriptyline administration for 5 wk was followed by prolonged cholestasis. Jaundice and pruritus lasted 19 and 20 mo, respectively. Three liver biopsies were performed at different stages of the disease showing the course of liver lesions. Cholestasis initially located in the region of the hepatic venule came to be associated with the progressive development of portal tract lesions consisting of inflammatory infiltration, fibrosis, and disappearance of interlobular bile ducts. Amitriptyline hydroxylation and dextromethorphan O-demethylation are deficient in subjects with the poor metabolizer phenotype of debrisoquine. Drug oxidation phenotyping with dextromethorphan showed... (truncated)

Title: Amitriptyline-induced fulminant hepatitis.
The authors report the case of a patient who received amitriptyline on 2 occasions. On both occasions, she had fever and jaundice. On the second episode, hepatitis was severe with hepatic encephalopathy, ascites, increased prothrombin time, and massive hepatic necrosis. After interruption of the drug administration, the patient made a slow but complete recovery.

Title: The role of NLRP3 inflammasome in psychotropic drug-induced hepatotoxicity.
Increased medical application of psychotropic drugs raised attention concerning their toxicological effects. In fact, more than 160 psychotropic drugs including antidepressants and antipsychotics, have been shown to cause liver side effects, but the underlying mechanisms are still poorly understood. Here, we discovered that fluoxetine, a common antidepressant, was specifically sensed by NLRP3 inflammasome, whose subsequent activation resulted in the maturation of caspase-1 and IL-1β, as well as gasdermin D (GSDMD) cleavage, which could be completely abrogated by a selective NLRP3 inhibitor MCC950 or Nlrp3 knockout (Nlrp3^{-/-}). Mechanistically, mitochondrial damage and the subsequent mitochondrial reactive oxygen species (mtROS) accumulation were crucial... (truncated)

Title: Amitriptyline induces mitophagy that precedes apoptosis in human HepG2 cells.
Systemic treatments for hepatocellular carcinoma (HCC) have been largely unsuccessful. This study investigated the antitumoral activity of Amitriptyline, a tricyclic antidepressant, in hepatoma cells. Amitriptyline-induced toxicity involved early mitophagy activation that subsequently switched to apoptosis. Amitriptyline induced mitochondria dysfunction and oxidative stress in HepG2 cells. Amitriptyline specifically inhibited mitochondrial complex III activity that is associated with decreased mitochondrial membrane potential ($\Delta\Psi m$) and increased reactive oxygen species (ROS) production. Transmission electron microscopy (TEM) studies revealed structurally abnormal mitochondria that were engulfed by double-membrane structures resembling autophagosomes. Consistent with mitophagy activation, fluorescence microscopy analysis showed mitochondrial Parkin recruitment and colocalization of mitochondria... (truncated)

Title: The combined use of human neural and liver cell lines and mouse hepatocytes improves the predictability of the neurotoxicity of selected drugs.
The cytotoxicity of amitriptyline (0-100microM), selegiline (0-4.5microM), carbamazepine (0-420microM) and paracetamol (0-10mM) was studied in metabolically competent mouse hepatocytes, metabolically incompetent human hepatoblastoma (HepG2) cells, and in neuroblastoma (SH-SY5Y) and astrocytoma (U-373 MG) cells, by using luminescence-based ATP measurement as an endpoint of cell toxicity. The aim was to evaluate the potential of the selected cell cultures to recognize metabolism-induced toxicity of the test compounds, and to predict further hepatic and neural toxicity. In SH-SY5Y cells amitriptyline was severely toxic, while selegiline and paracetamol failed to show any toxic effect, and carbamazepine was only slightly toxic at the highest concentration.... (truncated)

MESH:D004318 - doxycycline

Summary:

LLM Prediction Score	0.450 (normalized)
LLM Confidence Score	0.990
Golden Answer (Severity Class)	0.375 (normalized)
Prediction Error	0.075

Retrieved Context:

Title: Acute Hepatocellular Drug-Induced Liver Injury From Bupropion and Doxycycline.
The management and diagnosis of drug-induced liver injury (DILI) is often challenging, particularly when patients are taking multiple medications. We present a 29-year-old African American man who presented with jaundice and malaise after starting bupropion and doxycycline 2 weeks prior. He was found to have acute hepatocellular drug-induced liver injury with autoimmune features, and made a complete recovery with prednisone. Although bupropion and doxycycline are both known to cause liver toxicity, a closer inspection of the signature of liver injury and a review of prior related DILI cases assigns causality more to bupropion than doxycycline.

Title: Etiological role of brucellosis in autoimmune hepatitis.
To show that brucellosis may trigger autoimmune hepatitis (AIH), in addition to nonspecific liver involvement and toxic hepatitis, due to a class effect of tetracycline family used for treatment. We present a female patient admitted to our hospital due to partially improved fatigue and elevated liver enzymes following doxycycline and streptomycin usage for brucellosis. Brucellosis is endemic in our country, Turkey. It may involve any organ in the body. Liver is frequently involved. Doxycycline used for treatment occasionally may lead to hepatotoxicity. AIH is a necroinflammatory disease of the liver. Certain drugs (e.g. minocycline), toxins, and viruses (hepatitis B, hepatitis... (truncated)

Title: Doxycycline and hepatotoxicity.
The hepatotoxicity of tetracyclines is well known. If microvesicular steatosis due to a high dose of tetracycline has virtually disappeared, it can also be observed with other drugs belonging to the tetracycline family. To our knowledge, hepatotoxicity induced by doxycycline has never been reported. In our patient, the abrupt onset of hepatic failure, five days after the start of doxycycline and the rapid normalization after the drug was stopped, leads to suspect a causal relationship between doxycycline and liver insufficiency. We must however be careful before concluding, because our patient received also acetylsalicylic acid and paracetamol, two other potential hepatotoxic... (truncated)

Title: Association between tetracycline or doxycycline and hepatotoxicity: a population based case-control study.
An FDA Working Group, along with representatives of PhRMA and the American Association for the Study of Liver Diseases, as well as the Institute of Medicine Report 'To Err is Human: Building a Safer Health Care System' have suggested that post-marketing drug surveillance is a important method to decrease adverse drug events. While tetracyclines are known to cause hepatotoxicity, no post-marketing drug surveillance studies have examined the risk of developing hepatotoxicity with tetracyclines. Therefore, the objective of this study is to determine the difference in risk of hepatotoxicity in patients receiving doxycycline or tetracycline using California Medicaid claims.

Title: Liver involvement in patients with brucellosis: results of the Marmara study.
Brucellosis is a zoonotic disease that primarily affects the reticuloendothelial system. But, the extent of liver damage in due course of the disease is unclear. This study included 325 brucellosis patients with significant hepatobiliary involvement identified with microbiological analyses from 30 centers between 2000 and 2013. The patients with ≥5 times of the upper limit of normal for aminotransferases, total bilirubin level ≥2 mg/dl or local liver lesions were enrolled. Clinical hepatitis was detected in 284 patients (87.3 %) and cholestasis was detected in 215 (66.1 %) patients. Fatigue (91 %), fever (86 %), sweating (83 %), arthralgia (79 %),... (truncated)

MESH:D010111 - oxymorphone

Summary:

LLM Prediction Score	0.074 (normalized)
LLM Confidence Score	0.990
Golden Answer (Severity Class)	0.0 (normalized)
Prediction Error	0.074

Retrieved Context:

Title: Opioid Prescription in Patients With Chronic Kidney Disease: A Systematic Review of Comparing Safety and Efficacy of Opioid Use in Chronic Kidney Disease Patients.
Patients with diminished renal function necessitate special care. In patients with chronic kidney disease (CKD), opioid analgesics should be prescribed based on the severity of renal insufficiency; this will determine treatment options at the beginning and throughout the management of pain in CKD patients. The dosage of hydrophilic drugs and drugs with active metabolites should be adjusted according to the severity of CKD, and the process of treatment should be monitored by modifying drug dosages as necessary for background and breakthrough pain. Patients with CKD may benefit from opioid analgesics that are lipophilic, such as methadone, fentanyl, and buprenorphine, as... (truncated)

Title: Opana-induced thrombotic microangiopathy masquerading as thrombotic thrombocytopenic purpura.
Opana (oxymorphone) is a powerful semi-synthetic opioid agonist used for chronic pain management that is ingested orally. However, improper injection of Opana can lead to a rare and fatal blood disorder known as thrombotic microangiopathy. Opana-induced thrombotic microangiopathy can be easily mistaken for thrombotic thrombocytopenic purpura (TTP), leading to the initiation of therapeutic plasma exchange. Current literature has conflicting views on the necessity of therapeutic plasma exchange for the treatment of Opana-induced thrombotic microangiopathy. In our case report, a 47-year-old Caucasian male was admitted with a presentation suspicious for TTP then underwent therapeutic plasma exchange without clinical improvement. With supportive... (truncated)

Title: Review of oral oxymorphone in the management of pain.
Chronic cancer and nonmalignant pain (CNMP) is a common and major health problem afflicting approximately 40 million persons in the US. Most cancer patients, and many patients with CNMP, require opioid analgesics to obtain adequate pain relief. Oral oxymorphone is a new formulation of an existing parenteral opioid that has become available for the treatment of significant pain: acute postoperative, chronic arthritis, chronic low back, and chronic cancer pain. Oxymorphone is a typical mu-opioid agonist that is effective in both immediate- and extended-release (IR and ER) formulations. Oxymorphone is more lipid soluble than morphine, resulting in a rapid onset of... (truncated)

Title: Safe Use of Opioids in Chronic Kidney Disease and Hemodialysis Patients: Tips and Tricks for Non-Pain Specialists.
In patients suffering from moderate-to-severe chronic kidney disease (CKD) or end-stage renal disease (ESRD), subjected to hemodialysis (HD), pain is very common, but often underestimated. Opioids are still the mainstay of severe chronic pain management; however, their prescription in CKD and HD patients is still significantly low and pain is often under-treated. Altered pharmacokinetics and the lack of clinical trials on the use of opioids in patients with renal impairment increase physicians' concerns in this specific population. This narrative review focused on the correct and safe use of opioids in patients with CKD and HD. Morphine and codeine are not... (truncated)

Title: Cholestatic hepatitis as a possible new side-effect of oxycodone: a case report.
Oxycodone is a widely-used semisynthetic opioid analgesic that has been used for over eighty years. Oxycodone is known to cause side effects such as nausea, pruritus, dizziness, constipation and somnolence. As far as we are aware cholestatic hepatitis as a result of oxycodone use has not been reported so far in the world literature.

MESH:D017328 - fosinopril

Summary:

LLM Prediction Score	0.303 (normalized)
LLM Confidence Score	0.930
Golden Answer (Severity Class)	0.375 (normalized)
Prediction Error	0.072

Retrieved Context:

Title: Ramipril-associated cholestasis in the setting of recurrent drug-induced liver injury.
Aim: Angiotensin-converting enzyme inhibitors (ACEIs) are commonly used to treat hypertension. Although generally well tolerated, the adverse effects of ACEIs include hypotension, cough, acute kidney injury and hyperkalemia. Rare reports of ACEI-induced hepatotoxicity have been described, most notably a cholestatic pattern of injury related to captopril. A 67-year-old male presented to the emergency department with a three-week history of jaundice, pruritis and weakness. Eight weeks before, he began taking ramipril and clopidogrel. His past medical history was significant for previous acute cholestatic liver injury approximately 20 years earlier, which was attributed to methimazole. Abnormal blood work demonstrated aspartate aminotransferase (AST)... (truncated)

Title: Fosinopril. A review of its pharmacodynamic and pharmacokinetic properties, and therapeutic potential in essential hypertension.
Fosinopril is a phosphinic acid prodrug which, after oral administration, undergoes rapid hydrolysis to its active diacid, the angiotensin converting enzyme (ACE) inhibitor fosinoprilat. Unlike other ACE inhibitors, fosinoprilat has a compensatory dual route of elimination and is cleared by the liver and kidneys. Thus, in patients with diminished renal function increased hepatic clearance of fosinoprilat is noted and, similarly, in patients with diminished hepatic function increased renal clearance seems to occur. Because of this compensatory elimination, fosinopril therapy for all patients can begin with the same recommended dosage. Fosinopril 10 to 40mg administered once daily is an effective antihypertensive... (truncated)

Title: [Evaluation of the total hepatic function after treatment with fosinopril in hypertensive patients with liver cirrhosis].
Fosinopril is distinguished from other ACE inhibitors by a pharmacokinetic pecularity in the sense that is can be metabolized either by liver or kidney. This was the rationale of the present research the aim of which was to verify if administered to patients with liver cirrhosis the drug was liable to alter global liver function and ability to metabolize drugs. Eight cirrhotic males, mean age 56 years, also suffering from high blood pressure, were studied. In these patients, liver and kidney function tests (BUN, creatinine blood level, serum and urinary electrolytes, creatinine clearance, calcium and phosphor blood level, transaminases, alkaline... (truncated)

Title: Antihypertensive effect of fosinopril, a new angiotensin converting enzyme inhibitor: findings of the Fosinopril Study Group II.
In a double-blind study, after a four- to six-week placebo period, 418 patients with mild to moderate hypertension were randomly assigned to receive 5, 10, 20, or 40 mg of fosinopril or matched placebo orally once daily for four weeks. Seated diastolic blood pressure was determined 24 hours after the most recent dose. For patients with an inadequate blood pressure response after four weeks, doses were doubled during the second four weeks, and hydrochlorothiazide was added during the final four weeks. The patients showed a significant, consistent antihypertensive response in seated and standing systolic and diastolic blood pressures after four... (truncated)

Title: Lisinopril-Induced Liver Injury: An Unusual Presentation and Literature Review.
Lisinopril is an angiotensin converting enzyme inhibitor (ACE-I) that has been on market for more than 25 years. ACE-I are usually well tolerated and rarely have serious or life-threatening side effects. We describe an unusual presentation of fulminant hepatic cholestasis probably secondary to lisinopril. To our knowledge, this is the second case report which shows lisinopril-induced liver injury though a cholestatic mechanism. The patient was a 59-year-old woman with type 2 diabetes, a high body mass index and hypertension, who presented with a 5-week history of jaundice and itching. She had been started on lisinopril for diabetic nephropathy 8 weeks... (truncated)

MESH:D000077236 - topiramate

Summary:

LLM Prediction Score	0.303 (normalized)
LLM Confidence Score	0.990
Golden Answer (Severity Class)	0.375 (normalized)
Prediction Error	0.072

Retrieved Context:

Title: Clinical studies of topiramate.
Topiramate had well-documented efficacy as an adjunctive agent for partial-onset seizures in 5 double-blind, parallel trials, reducing seizure frequency by 35-47% at 400 mg/day, the optimal dose for most patients. It can also be used as monotherapy for partial-onset seizures. A broad spectrum of action is suggested by studies showing efficacy against a variety of generalized-onset seizure types, including primary generalized tonic-clonic seizures and the component seizures of Lennox-Gastaut syndrome. The major adverse effects of topiramate are of CNS origin. Psychomotor slowing is most common, but disappears in most patients with time or dosage adjustments; 11-28% of patients discontinue the... (truncated)

Title: Topiramate-induced acute liver injury: A rare adverse effect.
Idiosyncratic drug-induced liver injury (DILI) is damage to liver occurring at recommended dose of a drug in contrast to toxic or predictable DILI. Although it is common in first-generation antiepileptic drugs (AEDs), it is rare in newer AEDs such as topiramate. Topiramate commonly causes neurological adverse effects such as psychomotor slowing and somnolence. Hepatotoxicity by topiramate is rare and has been previously reported in combination with other drugs such as valproate and carbamazepine. Here, we report a case of topiramate-induced asymptomatic elevation of liver enzymes in an adult man diagnosed with alcohol dependence syndrome and alcohol withdrawal complicated with seizures.

Title: Acute hepatic injury in four children with Dravet syndrome: valproic acid, topiramate or acetaminophen?
We describe four children with Dravet syndrome treated with the combination of valproic acid (VPA) and topiramate (TPM) who developed transient liver toxicity. The time-interval between fever, administration of acetaminophen, epileptic status and liver enzyme disturbances in our four cases suggests that accumulation of toxic acetaminophen-metabolites is possibly responsible for liver toxicity. If acetaminophen and its metabolites cause those liver problems in children treated with the combination of VPA and TPM, the advice to use acetaminophen for treating fever in children using this combination, should be changed. Only future clinical observations and research can solve this clinical dilemma.

Title: Choice and use of newer anticonvulsant drugs in older patients.
Epilepsy is common in the elderly. The incidence of epilepsy is age-dependent, with a peak during the first year of life and higher incidence in those older than 75 years. Cerebrovascular disease is a common cause of epilepsy in the elderly. Drug treatment of the elderly is a challenge because of pharmacokinetic changes with aging, including impaired drug protein binding or displacement of drug from protein binding sites, potentially causing drug toxicity as a result of increased free drug concentrations. With aging, hepatic mass and blood flow decline along with renal function. Established anticonvulsant drugs have adverse effects and drug... (truncated)

Title: Antiepileptic Drugs and Liver Disease.
Acute, symptomatic seizures or epilepsy may complicate the course of hepatic disease. Choosing the most appropriate antiepileptic drug in this setting represents a difficult challenge, as most medications are metabolized by the liver. This article focuses on the acute and chronic treatment of seizures in patients with advanced liver disease and reviews the hepatotoxic potential of specific antiepileptic drugs. Newer antiepileptic drugs without, or with minimal, hepatic metabolism, such as levetiracetam, lacosamide, topiramate, gabapentin, and pregabalin should be used as first-line therapy. Medications undergoing extensive hepatic metabolism, such as valproic acid, phenytoin, and felbamate should be used as drugs of... (truncated)

MESH:D002996 - clomiphene

Summary:

LLM Prediction Score	0.304 (normalized)
LLM Confidence Score	0.990
Golden Answer (Severity Class)	0.375 (normalized)
Prediction Error	0.071

Retrieved Context:

Title: Liver injury induced by clomiphene citrate: A case report and literature reviews.
Clomiphene citrate is used to cause ovulation in females and to increase semen production in males. Clomiphene citrate is well tolerated in most patients and rarely induces liver injury. We report a case of liver injury which is associated with administration of clomiphene citrate in a male patient.

Title: Metformin and/or clomiphene do not adversely affect liver or renal function in women with polycystic ovary syndrome.
Nonalcoholic fatty liver disease is common to insulin-resistant states such as polycystic ovary syndrome (PCOS). Metformin (MET) is often used to treat PCOS but information is limited as to its effects on liver function.

Title: Quercetin and Ferulic Acid Elicit Estrogenic Activities In Vivo and In Silico.
In this study, we examined the sub-acute toxicity of quercetin and ferulic acid and evaluated their effects on protein, cholesterol, and estrogen levels in vivo. Six groups of female Wistar rats were fed by gavage. The first and second groups represent the positive (Clomiphene citrate 10 mg/kg) and negative (NaCl 0.9%) control groups, while the other groups received quercetin and ferulic acid at doses of 5 and 10 mg/kg/day for 28 days. The sub-acute toxicity was monitored by examining the weights, biochemical parameters (AST, ALT, ALP, urea, and CREA), and histological changes in the kidneys and liver of the treated... (truncated)

Title: Assessing the Hepatic Safety of Epigallocatechin Gallate (EGCG) in Reproductive-Aged Women.
A similar abstract of the interim analysis was previously published in Fertility and Sterility. EPIGALLOCATECHIN GALLATE (EGCG) FOR TREATMENT OF UNEXPLAINED INFERTILITY ASSOCIATED WITH UTERINE FIBROIDS (PRE-FRIEND TRIAL): EARLY SAFETY ASSESSMENT. Uterine fibroids are the most common cause of unexplained infertility in reproductive-aged women. Epigallocatechin gallate (EGCG), a green tea catechin, has demonstrated its ability to shrink uterine fibroids in prior preclinical and clinical studies. Hence, we developed an NICHD Confirm-funded trial to evaluate the use of EGCG for treating women with fibroids and unexplained infertility (FRIEND trial). Prior to embarking on that trial, we here conducted the pre-FRIEND study... (truncated)

Title: Herbal and dietary supplement induced liver injury: Highlights from the recent literature.
Herbal-induced liver injury (HILI) is an important and increasingly concerning cause of liver toxicity, and this study presents recent updates to the literature. An extensive literature review was conducted encompassing September 2019 through March 2021. Studies with clinically significant findings were analyzed and included in this review. We emphasized those studies that provided a causality assessment methodology, such as Roussel Uclaf Causality Assessment Method scores. Our review includes reports of individual herbals, including <i>Garcinia cambogia</i>, green tea extract, kratom as well as classes such as performance enhancing supplements, Traditional Chinese medicine, Ayurvedic medicine and herbal contamination. Newly described herbals include... (truncated)

MESH:C526675 - fesoterodine

Summary:

LLM Prediction Score	0.071 (normalized)
LLM Confidence Score	0.940
Golden Answer (Severity Class)	0.0 (normalized)
Prediction Error	0.071

Retrieved Context:

Title: Three-year safety, efficacy and persistence data following the daily use of mirabegron for overactive bladder in the clinical setting: A Japanese post-marketing surveillance study.
The aim of this study was to report the final 3-year results from a surveillance study evaluating the safety, efficacy, and persistence of mirabegron for treating overactive bladder (OAB) symptoms.

Title: Efficacy and Side Effects of Drugs Commonly Used for the Treatment of Lower Urinary Tract Symptoms Associated With Benign Prostatic Hyperplasia.
Benign prostatic hyperplasia (BPH) is the most common benign disease of the prostate gland and is caused by benign hyperplasia of the smooth muscle cells and stromal cells in this important gland. BPH is also the most common disease underlying lower urinary tract symptoms (LUTS). The incidence of BPH increases with age and affects more than half of all men 50 years or older. BPH mainly exerts effects on urinary function and can seriously reduce a patient's quality of life. At present, treatment for BPH aims primarily to improve the quality of life and reduce the risk of BPH-related complications.... (truncated)

Title: Analysis of Drug-Induced Gastrointestinal Obstruction and Perforation Using the Japanese Adverse Drug Event Report Database.
Drug-induced gastrointestinal obstruction (DIGO) and gastrointestinal perforation (DIGP) may be the result of gastrointestinal hypomotility and severe constipation, which may lead to potentially fatal complications of bowel ischemia, sepsis and perforation. We evaluated the onset profile of DIGs (DIGO and DIGP) associated with prescription drugs by analyzing data in the Japanese Adverse Drug Event Report (JADER) database. We selected 161 DIG-related drugs and categorized them into 19 classes based on the Anatomical Therapeutic Chemical (ATC) Classification System. Finally, we focused on 58 drugs and conducted subsequent analyses for the time-to-onset and outcomes. We extracted 79 preferred terms (PTs) with the... (truncated)

Title: Pharmacotherapy of overactive bladder in adults: a review of efficacy, tolerability, and quality of life.
Overactive bladder (OAB) is a prevalent condition that has a significant impact on quality of life. The usual treatment approach is both behavioral and pharmacological. The first-line pharmacological treatment commonly utilizes anticholinergic agents, which may be limited by their tolerability, efficacy, and long-term compliance. Developments in elucidating the pathophysiology of OAB and alternative targets for pharmacological therapy have led to newer agents being developed to manage this condition. These agents include mirabegron and botulinum toxin, which have alternate mechanism of action and avoid the anticholinergic side effects.

Title: Lower urinary tract symptoms in men.
Benign prostatic hyperplasia (BPH) is a highly prevalent and costly condition that affects older men worldwide. Many affected men develop lower urinary tract symptoms, which can have a negative impact on their quality of life. In the past, transurethral resection of the prostate (TURP) was the mainstay of treatment. However, several efficacious drug treatments have been developed, which have transformed BPH from an acute surgical entity to a chronic medical condition. Specifically, multiple clinical trials have shown that α adrenoceptor antagonists can significantly ameliorate lower urinary tract symptoms. Moreover, 5α reductase inhibitors, alone or combined with an α adrenoceptor antagonist,... (truncated)

MESH:D006145 - guanethidine

Summary:

LLM Prediction Score	0.071 (normalized)
LLM Confidence Score	0.960
Golden Answer (Severity Class)	0.0 (normalized)
Prediction Error	0.071

Retrieved Context:

Title: Utility of spherical human liver microtissues for prediction of clinical drug-induced liver injury.
Drug-induced liver injury (DILI) continues to be a major source of clinical attrition, precautionary warnings, and post-market withdrawal of drugs. Accordingly, there is a need for more predictive tools to assess hepatotoxicity risk in drug discovery. Three-dimensional (3D) spheroid hepatic cultures have emerged as promising tools to assess mechanisms of hepatotoxicity, as they demonstrate enhanced liver phenotype, metabolic activity, and stability in culture not attainable with conventional two-dimensional hepatic models. Increased sensitivity of these models to drug-induced cytotoxicity has been demonstrated with relatively small panels of hepatotoxicants. However, a comprehensive evaluation of these models is lacking. Here, the predictive value... (truncated)

Title: Metoprolol-induced Severe Liver Injury and Successful Management with Therapeutic Plasma Exchange.
Liver injury caused by metoprolol is very rare with current reports limited to an isolated elevation in transaminases. We report the first case of severe icteric liver injury leading to hepatic encephalopathy secondary to metoprolol use in a patient diagnosed with coronary heart disease. We also describe the histopathology of metoprolol-related liver injury, discuss mechanisms of injury with new insights on the immunological phenomenon, and shed light on the successful utility of early plasmapheresis as a salvage therapy in metoprolol-induced severe liver damage.

Title: Increased uptake of [^{123}I]meta-iodobenzylguanidine, [^{18}F]fluorodopamine, and [^{3}H]norepinephrine in mouse pheochromocytoma cells and tumors after treatment with the histone deacetylase inhibitors.
[^{131}I]meta-iodobenzylguanidine ([^{131}I]MIBG) is the most commonly used treatment for metastatic pheochromocytoma and paraganglioma. It enters the chromaffin cells via the membrane norepinephrine transporter; however, its success has been modest. We studied the ability of histone deacetylase (HDAC) inhibitors to enhance [^{123}I]MIBG uptake by tumors in a mouse metastatic pheochromocytoma model. HDAC inhibitors are known to arrest growth, induce differentiation and apoptosis in various cancer cells, and further inhibit tumor growth. We report the in vitro and in vivo effects of two HDAC inhibitors, romidepsin and trichostatin A, on the uptake of [(3)H]norepinephrine, [^{123}I]MIBG, and [(18)F]fluorodopamine in a mouse model of... (truncated)

Title: Thyroid and hepatic function after high-dose 131 I-metaiodobenzylguanidine (131 I-MIBG) therapy for neuroblastoma.
(131) I-Metaiodobenzylguanidine ((131) I-MIBG) provides targeted radiotherapy for children with neuroblastoma, a malignancy of the sympathetic nervous system. Dissociated radioactive iodide may concentrate in the thyroid, and (131) I-MIBG is concentrated in the liver after (131) I-MIBG therapy. The aim of our study was to analyze the effects of (131) I-MIBG therapy on thyroid and liver function.

Title: Pathophysiology of acute kidney injury.
Acute kidney injury (AKI) is the leading cause of nephrology consultation and is associated with high mortality rates. The primary causes of AKI include ischemia, hypoxia, or nephrotoxicity. An underlying feature is a rapid decline in glomerular filtration rate (GFR) usually associated with decreases in renal blood flow. Inflammation represents an important additional component of AKI leading to the extension phase of injury, which may be associated with insensitivity to vasodilator therapy. It is suggested that targeting the extension phase represents an area potential of treatment with the greatest possible impact. The underlying basis of renal injury appears to be... (truncated)

MESH:C025130 - quazepam

Summary:

LLM Prediction Score	0.069 (normalized)
LLM Confidence Score	0.910
Golden Answer (Severity Class)	0.0 (normalized)
Prediction Error	0.069

Retrieved Context:

Title: Data-driven identification of structural alerts for mitigating the risk of drug-induced human liver injuries.
The use of structural alerts to de-prioritize compounds with undesirable features as drug candidates has been gaining in popularity. Hundreds of molecular structural moieties have been proposed as structural alerts. An emerging issue is that strict application of these alerts will result in a significant reduction of the chemistry space for new drug discovery, as more than half of the oral drugs on the market match at least one of the alerts. To mitigate this issue, we propose to apply a rigorous statistical analysis to derive/validate structural alerts before use.

Title: The LiverTox Paradox-Gaps between Promised Data and Reality Check.
The LiverTox database compiles cases of idiosyncratic drug-induced liver injury (iDILI) with the promised aims to help identify hepatotoxicants and provide evidence-based information on iDILI. Weaknesses of this approach include case selection merely based on published case number and not on a strong causality assessment method such as the Roussel Uclaf Causality Assessment Method (RUCAM). The aim of this analysis was to find out whether the promised aims have been achieved by comparison of current iDILI case data with those promised in 2012 in LiverTox. First, the LiverTox criteria of likelihood categories applied to iDILI cases were analyzed regarding robustness.... (truncated)

Title: Insomnia drug therapy in COVID-19 patients; a letter to editor.
No abstract available.

Title: Covalent modification of lipids and proteins in rat hepatocytes and in vitro by thioacetamide metabolites.
Thioacetamide (TA) is a well-known hepatotoxin in rats. Acute doses cause centrilobular necrosis and hyperbilirubinemia while chronic administration leads to biliary hyperplasia and cholangiocarcinoma. Its acute toxicity requires its oxidation to a stable S-oxide (TASO) that is oxidized further to a highly reactive S,S-dioxide (TASO(2)). To explore possible parallels among the metabolism, covalent binding, and toxicity of TA and thiobenzamide (TB), we exposed freshly isolated rat hepatocytes to [(14)C]-TASO or [(13)C(2)D(3)]-TASO. TLC analysis of the cellular lipids showed a single major spot of radioactivity that mass spectral analysis showed to consist of N-acetimidoyl PE lipids having the same side chain... (truncated)

Title: Prevalence and factors associated with potentially inappropriate medication use in older medicare beneficiaries with cancer.
To assess the factors related to potentially inappropriate medication (PIM) use in elderly patients with cancer, as well as to compare the PIM prevalence in older adults with and without cancer.

MESH:D004958 - estradiol

Summary:

LLM Prediction Score	0.182 (normalized)
LLM Confidence Score	0.990
Golden Answer (Severity Class)	0.25 (normalized)
Prediction Error	0.068

Retrieved Context:

Title: Experimental Hepatotoxicity Produced by Ethinyl estradiol.
Ethinyl oestradiol (EO) is the most commonly used as a component of oral contraceptive and hormonal replacement therapy (HRT) in women. However, its excessive and prolonged use may cause cytotoxicity, including cancer of many organs. Hence, the present study was performed to produce the experimental hepatotoxicity in female albino rats. EO was administered to different groups of rats, respectively @ 250, 500 and 750 μg/kg body weight, orally, weekly for 16 and 20 weeks. One group of rats was administered with saline alone to serve as control. The rats were sacrificed after their respective experimental periods, and the livers were... (truncated)

Title: Female hepatology: favorable role of estrogen in chronic liver disease with hepatitis B virus infection.
Chronic hepatitis B virus (HBV) infection is the most common cause of hepatic fibrosis and hepatocellular carcinoma (HCC), mainly as a result of chronic necroinflammatory liver disease. A characteristic feature of chronic hepatitis B infection, alcoholic liver disease and nonalcoholic fatty liver disease (NAFLD) is hepatic steatosis. Hepatic steatosis leads to an increase in lipid peroxidation in hepatocytes, which, in turn, activates hepatic stellate cells (HSCs). HSCs are the primary target cells for inflammatory and oxidative stimuli, and these cells produce extracellular matrix components. Chronic hepatitis B appears to progress more rapidly in males than in females, and NAFLD, cirrhosis... (truncated)

Title: Potential Therapeutic Application of Estrogen in Gender Disparity of Nonalcoholic Fatty Liver Disease/Nonalcoholic Steatohepatitis.
Nonalcoholic fatty liver disease (NAFLD) caused by fat accumulation in the liver is globally the most common cause of chronic liver disease. Simple steatosis can progress to nonalcoholic steatohepatitis (NASH), a more severe form of NAFLD. The most potent driver for NASH is hepatocyte death induced by lipotoxicity, which triggers inflammation and fibrosis, leading to cirrhosis and/or liver cancer. Despite the significant burden of NAFLD, there is no therapy for NAFLD/NASH. Accumulating evidence indicates gender-related NAFLD progression. A higher incidence of NAFLD is found in men and postmenopausal women than premenopausal women, and the experimental results, showing protective actions of... (truncated)

Title: Ovarian hormones influence immune response to liver ischemia-reperfusion.
Liver injury occurs after ischemia and reperfusion (IR), as seen in transplant settings. Sex hormones have been implicated in many pathophysiological mechanisms in females and this could lead to liver protection under inflammatory reperfusion conditions where an excessive immune response occurs. Despite such assumptions, this fact needs to be further investigated. To address this, female and male C57BL/6J mice (8-12 weeks old) were studied. Bilateral ovariectomy (OVX) was performed in females to decrease estradiol levels. IR was performed, and after two weeks, all animals underwent a sham control operation or IR with euthanasia at the following time points after reperfusion:... (truncated)

Title: Estradiol and progesterone modulate halothane-induced liver injury in mice.
Drug-induced liver injury (DILI) is one of the major problems in drug development and clinical drug therapy. In general, it is believed that women exhibit worse outcomes from DILI than men. It is known that halothane (HAL), an inhaled anesthetic, rarely induces severe liver injury. The risk factors for severe HAL-induced liver injury (HILI) are female sex, genetics and adult age. To investigate the underlying mechanism by which women are more susceptible to HILI, we focused on two major female sex hormones, estradiol (E2) and progesterone (Prog). In this study, we first found that pretreatment of mice with E2 attenuated... (truncated)

MESH:C088327 - plerixafor

Summary:

LLM Prediction Score	0.067 (normalized)
LLM Confidence Score	0.980
Golden Answer (Severity Class)	0.0 (normalized)
Prediction Error	0.067

Retrieved Context:

Title: Plerixafor for the Treatment of WHIM Syndrome.
WHIM syndrome (warts, hypogammaglobulinemia, infections, and myelokathexis), a primary immunodeficiency disorder involving panleukopenia, is caused by autosomal dominant gain-of-function mutations in CXC chemokine receptor 4 (CXCR4). Myelokathexis is neutropenia caused by neutrophil retention in bone marrow. Patients with WHIM syndrome are often treated with granulocyte colony-stimulating factor (G-CSF), which can increase neutrophil counts but does not affect cytopenias other than neutropenia. In this investigator-initiated, open-label study, three severely affected patients with WHIM syndrome who could not receive G-CSF were treated with low-dose plerixafor, a CXCR4 antagonist, for 19 to 52 months. Myelofibrosis, panleukopenia, anemia, and thrombocytopenia were ameliorated, the wart... (truncated)

Title: Study of Renal Accumulation of Targeted Polycations in Acute Kidney Injury.
Acute kidney injury (AKI) is a global healthcare burden characterized by rapid loss of renal function and high morbidity and mortality. Chemokine receptor CXCR4 participates in the renal infiltration of immune cells following injury and in local inflammatory enhancement. Injured renal tubule cells overexpress CXCR4, which could be used as a target for improved drug delivery in AKI. Plerixafor is a small-molecule CXCR4 antagonist that has shown beneficial effects against AKI and has been previously developed into a polymeric analog (polymeric plerixafor, PP). With the goal of gaining a better understanding of how overall charge and hydrophilicity affect renal accumulation... (truncated)

Title: The role of chemokines in acute liver injury.
Chemokines are small molecular weight proteins primarily known to drive migration of immune cell populations. In both acute and chronic liver injury, hepatic chemokine expression is induced resulting in inflammatory cell infiltration, angiogenesis, and cell activation and survival. During acute injury, massive parenchymal cell death due to apoptosis and/or necrosis leads to chemokine production by hepatocytes, cholangiocytes, Kupffer cells, hepatic stellate cells, and sinusoidal endothelial cells. The specific chemokine profile expressed during injury is dependent on both the type and course of injury. Hepatotoxicity by acetaminophen for example leads to cellular necrosis and activation of Toll-like receptors while the inciting... (truncated)

Title: Polymeric Plerixafor: effect of PEGylation on CXCR4 antagonism, cancer cell invasion, and DNA transfection.
To determine the effect of PEG modification on pharmacologic and gene delivery properties of polymeric CXCR4 antagonist based on Plerixafor.

Title: Cyclam-Modified Polyethyleneimine for Simultaneous TGFβ siRNA Delivery and CXCR4 Inhibition for the Treatment of CCl₄-Induced Liver Fibrosis.
Liver fibrosis is a chronic liver disease with excessive production of extracellular matrix proteins, leading to cirrhosis, hepatocellular carcinoma, and death.

MESH:D003084 - colestipol

Summary:

LLM Prediction Score	0.441 (normalized)
LLM Confidence Score	0.940
Golden Answer (Severity Class)	0.375 (normalized)
Prediction Error	0.066

Retrieved Context:

Title: Adverse effects of hypolipidaemic drugs.
Cholestyramine, colestipol, clofibrate, gemfibrozil, nicotinic acid (niacin), probucol, neomycin, and dextrothyroxine are the most commonly used drugs in the treatment of hyperlipoproteinaemic disorders. While adverse reaction data are available for all of them, definitive data regarding the frequency and severity of potential adverse effects from well-controlled trials using large numbers of patients (greater than 1000) are available only for cholestyramine, clofibrate, nicotinic acid and dextrothyroxine. In adult patients treated with cholestyramine, gastrointestinal complaints, especially constipation, abdominal pain and unpalatability are most frequently observed. Continued administration along with dietary manipulation (e.g. addition of dietary fibre) and/or stool softeners results in diminished... (truncated)

Title: Colestipol-induced hepatotoxicity.
A 65-year-old man with type IIa dyslipidemia who received flavored colestipol granules 2 scoops/day for 3 months developed asymptomatic hepatotoxicity. Several of his liver enzymes were elevated 10 times the upper limit of normal. One week after discontinuing colestipol, serum transaminases fell dramatically, with some returning to normal limits. Four weeks after colestipol was discontinued, all liver function tests were normal. Rechallenge was not attempted. Other potential causes of hepatocellular injury were evaluated. Bile acid-binding resins commonly are administered to treat type IIa dyslipidemia. Despite extensive use of the resins, significant elevations of transaminase levels are rare. Because the exact... (truncated)

Title: Clinical pharmacokinetics of diclofenac. Therapeutic insights and pitfalls.
Diclofenac is a nonsteroidal anti-inflammatory drug (NSAID) of the phenylacetic acid class. When given orally the absorption of diclofenac is rapid and complete. Diclofenac binds extensively to plasma albumin. The area under the plasma concentration-time curve (AUC) of diclofenac is proportional to the dose for oral doses between 25 to 150 mg. Substantial concentrations of drug are attained in synovial fluid, which is the proposed site of action for NSAIDs. Concentration-effect relationships have been established for total bound, unbound and synovial fluid diclofenac concentrations. Diclofenac is eliminated following biotransformation to glucoroconjugated and sulphate metabolites which are excreted in urine, very... (truncated)

Title: Bile Acid Sequestrants Based on Natural and Synthetic Gels.
Bile acid sequestrants (BASs) are non-systemic therapeutic agents used for the management of hypercholesterolemia. They are generally safe and not associated with serious systemic adverse effects. Usually, BASs are cationic polymeric gels that have the ability to bind bile salts in the small intestine and eliminate them by excretion of the non-absorbable polymer-bile salt complex. This review gives a general presentation of bile acids and the characteristics and mechanisms of action of BASs. The chemical structures and methods of synthesis are shown for commercial BASs of first- (cholestyramine, colextran, and colestipol) and second-generation (colesevelam and colestilan) and potential BASs. The... (truncated)

Title: Evaluation and Management of Chronic Cholestatic Liver Diseases.
Cholestasis is defined as stagnation or a marked reduction in bile secretion and flow. Cholestatic jaundice can thus be classified as intrahepatic or extrahepatic cholestatic, depending on the level of obstruction to bile flow. It is important to recognize the complications of cholestatic in patients with chronic cholestatic liver disease. The two most common complications of cholestasis are pruritus and fatigue, with the former being the most responsive to treatment. Cholestyramine is the first-line treatment for cholestatic pruritus. Rifampicin and oral opioid antagonist naltrexone are extremely effective second-line treatments. To date, there are no specific treatments for chronic cholestatic fatigue... (truncated)

MESH:C088769 - ferumoxsil

Summary:

LLM Prediction Score	0.066 (normalized)
LLM Confidence Score	0.890
Golden Answer (Severity Class)	0.0 (normalized)
Prediction Error	0.066

Retrieved Context:

Title: 3-Aminopropylsilane-modified iron oxide nanoparticles for contrast-enhanced magnetic resonance imaging of liver lesions induced by <i>Opisthorchis felineus</i>.
Liver fluke causes severe liver damage in an infected human. However, the infection often remains neglected due to the lack of pathognomonic signs. Nanoparticle-enhanced magnetic resonance imaging (MRI) offers a promising technique for detecting liver lesions induced by parasites.

Title: Nanocarrier-Based Therapeutics and Theranostics Drug Delivery Systems for Next Generation of Liver Cancer Nanodrug Modalities.
The development of therapeutics and theranostic nanodrug delivery systems have posed a challenging task for the current researchers due to the requirement of having various nanocarriers and active agents for better therapy, imaging, and controlled release of drugs efficiently in one platform. The conventional liver cancer chemotherapy has many negative effects such as multiple drug resistance (MDR), high clearance rate, severe side effects, unwanted drug distribution to the specific site of liver cancer and low concentration of drug that finally reaches liver cancer cells. Therefore, it is necessary to develop novel strategies and novel nanocarriers that will carry the drug... (truncated)

Title: Subchronic and chronic toxicity evaluation of inorganic nanoparticles for delivery applications.
Inorganic nanoparticles provide the opportunity to localize bioactive agents to the target sites and protect them from degradation. In many cases, acute toxicities of inorganic nanoparticles used for delivery applications have been investigated. However, little information is available regarding the long-term toxicity of such materials. This review focuses on the importance of subchronic and chronic toxicity assessment of inorganic nanoparticles investigated for delivery applications. We have attempted to provide a comprehensive review of the available literature for chronic toxicity assessment of inorganic nanoparticles. Where possible correlations are made between particle composition, physiochemical properties, duration, frequency and route of administration, as... (truncated)

Title: In vivo delivery, pharmacokinetics, biodistribution and toxicity of iron oxide nanoparticles.
Iron oxide nanoparticles (IONPs) have been extensively used during the last two decades, either as effective bio-imaging contrast agents or as carriers of biomolecules such as drugs, nucleic acids and peptides for controlled delivery to specific organs and tissues. Most of these novel applications require elaborate tuning of the physiochemical and surface properties of the IONPs. As new IONPs designs are envisioned, synergistic consideration of the body's innate biological barriers against the administered nanoparticles and the short and long-term side effects of the IONPs become even more essential. There are several important criteria (e.g. size and size-distribution, charge, coating molecules,... (truncated)

Title: Quercetin attenuates neurotoxicity induced by iron oxide nanoparticles.
Iron oxide nanoparticles (IONPs) have been proposed as targeted carriers to deliver therapeutic molecules in the central nervous system (CNS). However, IONPs may damage neural tissue via free iron accumulation, protein aggregation, and oxidative stress. Neuroprotective effects of quercetin (QC) have been proven due to its antioxidant and anti-inflammatory properties. However, poor solubility and low bioavailability of QC have also led researchers to make various QC-involved nanoparticles to overcome these limitations. We wondered how high doses or prolonged treatment with quercetin conjugated superparamagnetic iron oxide nanoparticles (QCSPIONs) could improve cognitive dysfunction and promote neurogenesis without any toxicity. It can be... (truncated)

MESH:D009268 - nalidixic acid

Summary:

LLM Prediction Score	0.310 (normalized)
LLM Confidence Score	0.950
Golden Answer (Severity Class)	0.375 (normalized)
Prediction Error	0.065

Retrieved Context:

Title: Nalidixic acid potentiates the antitumor activity in sorafenib-resistant hepatocellular carcinoma <i>via</i> the tumor immune microenvironment analysis.
Sorafenib resistance is often developed and impedes the benefits of clinical therapy in hepatocellular carcinoma (HCC) patients. However, the relationship between sorafenib resistance and tumor immune environment and adjuvant drugs for sorafenib-resistant HCC are not systemically identified. This study first analyzed the expression profiles of sorafenib-resistant HCC cells to explore immune cell infiltration levels and differentially expressed immune-related genes (DEIRGs). The prognostic value of DEIRGs was analyzed using Cox regression and Kaplan-Meier analysis based on The Cancer Genome Atlas. The primary immune cells infiltrated in sorafenib-resistant HCC mice were explored using flow cytometry (FCM). Finally, small-molecule drugs for sorafenib-resistant HCC... (truncated)

Title: [Clinical and laboratory features of typhoid fever in Senegal. A 70-case study].
Clinical and laboratory features, complications and treatment were retrospectively studied in 70 patients with bacteriologically documented typhoid fever, treated between January 1995 and June 2002 at Principal Hospital in Dakar, Senegal. Data analysis was done on a global basis as well as comparatively between the 37 children (under 15 years) and 33 adults. Mean age was 16.7 years (range, 1 to 52). The sex ratio was 1.4. Clinical manifestations included fever (97%), headache (50%), vomiting (71%), abdominal pain (54%), diarrhoea (49%), nnd splenomegaly (10%) without statistically significant difference between children and adults. Lyinphopenia was found in 51% of patients and... (truncated)

Title: Successful drug development despite adverse preclinical findings part 2: examples.
To illustrate the process of addressing adverse preclinical findings (APFs) as outlined in the first part of this review, a number of cases with unexpected APF in toxicity studies with drug candidates is discussed in this second part. The emphasis is on risk characterization, especially regarding the mode of action (MoA), and risk evaluation regarding relevance for man. While severe APFs such as retinal toxicity may turn out to be of little human relevance, minor findings particularly in early toxicity studies, such as vasculitis, may later pose a real problem. Rodents are imperfect models for endocrine APFs, non-rodents for human... (truncated)

Title: Current advances on the phytochemical composition, pharmacologic effects, toxicology, and product development of <i>Phyllanthi Fructus</i>.
<i>Phyllanthi Fructus</i> (PF), the edible fruits of <i>Phyllanthus emblica</i> L., serves as an important resource for some health products, foods and drugs due to its high safety and sufficient nutritional value. In recent years, <i>in vivo</i> and <i>in vitro</i> experiments have been conducted to reveal the active components of PF. More than 180 compounds have been isolated and identified from the PF so far, primarily including tannins, phenolic acids, flavonoids, terpenoids, polysaccharides, fatty acids and amino acids. In traditional Chinese medicine (TCM), PF is used to cure several diseases such as bronchitis, asthma, diabetes, peptic ulcer, hepatopathy, leprosy, and jaundice.... (truncated)

Title: Clinical outcomes in typhoid fever: adverse impact of infection with nalidixic acid-resistant Salmonella typhi.
Widespread use of fluoroquinolones has resulted in emergence of Salmonella typhi strains with decreased susceptibility to fluoroquinolones. These strains are identifiable by their nalidixic acid-resistance. We studied the impact of infection with nalidixic acid-resistant S. typhi (NARST) on clinical outcomes in patients with bacteriologically-confirmed typhoid fever.

MESH:D019808 - losartan

Summary:

LLM Prediction Score	0.435 (normalized)
LLM Confidence Score	0.990
Golden Answer (Severity Class)	0.5 (normalized)
Prediction Error	0.065

Retrieved Context:

Title: Drug-Induced Liver Injury Due To Losartan.
Drug-induced liver injury (DILI) is a challenging diagnosis since a wide variety of medicines can cause adverse reactions. Losartan is an angiotensin II receptor antagonist (ARA-II) approved for the treatment of arterial hypertension. The most common adverse effects are fatigue, anaemia, weakness and cough. An increase in transaminases has been reported with less frequency (<2% of cases). Although the mechanism is not fully understood, DILI onset is usually within 1-8 weeks of therapy, and hepatic enzymology usually normalizes 2-4 months after drug suspension. The authors present the case of a 66-year-old male patient with a medical history of arterial hypertension... (truncated)

Title: Losartan-induced Severe Hepatic Injury: A Case Report and Literature Review.
Medications have been known to cause adverse drug reactions that affect various organs; these are mostly reversible reactions that improve with the cessation of the culprit medication. Losartan is an angiotensin-one receptor blocker which has been approved by the Food and Drug Administration (FDA) for the treatment of arterial hypertension. Fatigue, anemia, weakness, and cough are some of the common adverse effects of losartan. Acute hepatic injury has rarely been reported as an adverse effect of losartan. We report a case of a 61-year-old female with severe hepatic injury secondary to losartan use. None of the cases reported so far... (truncated)

Title: Losartan-induced Ischemic Hepatocellular Hepatotoxicity: A Case Report and Literature Review.
With the increasing use of various medications and supplements nowadays, the incidence of abnormal liver function tests and frank hepatic injury is has been increasing. Medications are now considered one of the most common causes of acute hepatic failure in the United States. Losartan was the first angiotensin 1 (AT1) receptor blocker approved by FDA for the treatment of arterial hypertension. It is a well-tolerated medication with few significant adverse effects. However, losartan-related hepatotoxicity has been reported rarely. We report a case of acute hepatic injury in an adult patient treated with losartan as a monotherapy for arterial hypertension.

Title: Renin angiotensin system blockage by losartan neutralize hypercholesterolemia-induced inflammatory and oxidative injuries.
ABSTRACTObjectives: This study explores the protective role of losartan (LT) against oxidative and inflammatory damages in different physiological systems including heart, liver, and kidney tissue in hypercholesterolemic rats.Methods: After induction of hypercholesterolemia by high cholesterol diet for 6 weeks, LT was administered for 4 weeks. In serum, the levels of lipoproteins, aminotransferases, creatine kinases, urea, apoptosis, and inflammatory markers were measured. In cardiac, hepatic, and renal tissues, lipid peroxidation product and GSH as well as antioxidant enzymatic activities were assayed. Finally, histopathological assessment evaluated the structural damage in cardiac, hepatic, and renal tissues.Results: Serum markers of cardiac, hepatic, and renal... (truncated)

Title: Effect of Telmisartan or Losartan for Treatment of Nonalcoholic Fatty Liver Disease: Fatty Liver Protection Trial by Telmisartan or Losartan Study (FANTASY).
This study compared the effects of telmisartan and losartan on nonalcoholic fatty liver disease (NAFLD) and biochemical markers of insulin resistance in hypertensive NAFLD patients with type 2 diabetes mellitus.

MESH:D000097670 - cefprozil

Summary:

LLM Prediction Score	0.435 (normalized)
LLM Confidence Score	0.970
Golden Answer (Severity Class)	0.5 (normalized)
Prediction Error	0.065

Retrieved Context:

Title: Jaundice associated with cephalosporin therapy.
Two cases of cephalosporin-induced hepatotoxicity with associated jaundice are presented. Both patients developed jaundice and liver enzyme abnormalities soon after injectable cephalosporin therapy was started. Other possible causes were ruled out, including TPN and allopurinol hepatoxicity, and additional medical illnesses associated with hepatoxicity. Both patients recovered fully from the episode of jaundice. Review of the literature suggests a possible hypersensitivity reaction similar to liver toxicity of the penicillin antibiotics.

Title: A rare case of hepatitis associated with cefprozil therapy.
Cefprozil is a novel third generation, broad-spectrum oral cephalosporin. Serum sickness-like reaction and rash caused by cefprozil have been previously reported, but liver damage has not been documented. We report on a 15 years-old female with tonsillopharyngitis who developed hepatitis after treatment with cefprozil. Following discontinuation of cefprozil therapy, her liver functions returned to normal limits within four weeks. We suggest that for patients presenting with hepatitis associated with drugs, Cefprozil should be considered in the differential diagnosis of hepatitis.

Title: A rare case of cefepime-induced cholestatic liver injury.
Cefepime is widely used in the hospital setting, and only a few studies have reported neurotoxicity and nephrotoxicity as side effects of this drug. Herein, we present a 93-year-old man who exhibited features of cholestatic hepatitis including elevated blood transaminases and direct-form predominant bilirubin levels after administration of cefepime. Blood liver tests showed total recovery after discontinuing the offending agent. Cefepime was probable to cause drug-induced cholestatic hepatitis in our patient since the Roussel Uclaf Causality Assessment Method score for cefepime was 7. No drug interactions were likely according to the Drug Interaction Probability Scale for this patient. No similar... (truncated)

Title: Cetirizine-induced hepatotoxicity: case series and review of the literature.
Drug-induced liver damage is a frequently encountered clinical table caused by many drugs. Cetirizine is a widely preferred and prescribed antihistaminic agent for allergic disorders due to its non-sedative properties. In view of the literature, we present four cases of hepatotoxicity due to cetirizine use. We conclude that in patients with high levels of liver enzymes of unknown origin, cetirizine as well as other hepatotoxic drugs should be reconsidered.

Title: Hepatic injury following feprazone therapy.
Two cases of feprazone-induced hepatic injury are reported. Both patients developed jaundice within one month of commencing therapy. Histological investigation showed a granulomatous reaction in one case and hepatitic changes in the other. The changes were similar to those seen as a complication of phenylbutazone therapy. When the administration of feprazone was discontinued both the clinical and biochemical changes were resolved.

MESH:D000077712 - telbivudine

Summary:

LLM Prediction Score	0.313 (normalized)
LLM Confidence Score	0.950
Golden Answer (Severity Class)	0.25 (normalized)
Prediction Error	0.063

Retrieved Context:

Title: Adverse effects of oral antiviral therapy in chronic hepatitis B.
Oral nucleoside/nucleotide analogues (NAs) are currently the backbone of chronic hepatitis B (CHB) infection treatment. They are generally well-tolerated by patients and safe to use. To date, a significant number of patients have been treated with NAs. Safety data has accumulated over the years. The aim of this article is to review and update the adverse effects of oral NAs. NAs can cause class adverse effects (<i>i.e</i>., myopathy, neuropathy, lactic acidosis) and dissimilar adverse effects. All NAs carry a "Black Box" warning because of the potential risk for mitochondrial dysfunction. However, these adverse effects are rarely reported. The majority of... (truncated)

Title: A randomized open label trial of tenofovir monotherapy versus tenofovir plus telbivudine in spontaneous reactivation of hepatitis B.
Acute-on-chronic liver failure (ACLF-B) in spontaneous reactivation of chronic hepatitis B (SR-CHB) has high mortality. Tenofovir disoproxil fumarate (TDF) improves survival by ~40% in ACLF-B but is potentially nephrotoxic. Combining telbivudine (LDT) with TDF may negate this risk and could boost rapid viral clearance and improve clinical outcomes.

Title: Baseline prognostic factors and statistic model to predict early virological response in telbivudine-treated patients with chronic hepatitis B.
Hepatitis B virus (HBV) infection is still a worldwide disease, which may cause liver cirrhosis or even hepatocellular carcinoma. Telbivudine is a potent nucleoside analogue used in the treatment of chronic hepatitis B (CHB); however, drug resistance has remained a challenge. As early virological response can predict long-term efficacy of nucleotide analogue treatment, numerous studies have been conducted in this area.

Title: Telbivudine treatment corrects HBV-induced epigenetic alterations in liver cells of patients with chronic hepatitis B.
Hepatitis B virus (HBV) alters the expression of host cellular genes to support its replication and survival and to promote the liver cell injury. However, the underlying mechanism remained incompletely understood. In this study, we investigated HBV-induced epigenetic changes in HepG2 cells by profiling the landscapes of the active histone modification mark H3K4me3 and repressive mark H3K27me3 using chromatin immunoprecipitation-sequencing. HBV caused the altered histone modifications at thousands of genomic loci, which are critically involved in HBV entry, inflammation, fibrosis and carcinogenesis of host cells. Interestingly, treatment of the HBV-transformed HepG2 cells with the anti-HBV drug Telbivudine substantially restored the... (truncated)

Title: Hepatitis B virus genotype is an independent prognostic factor of telbivudine and tenofovir treatment in hepatitis B surface antigen-positive pregnant women.
To investigate whether HBV genotype influences the effect of tenofovir and telbivudine on HBV DNA and RNA levels in HBsAg-positive pregnant women. This was a retrospective study of 74 HBsAg-positive pregnant women in Guizhou of China. All patients were treated with telbivudine or tenofovir from 12 weeks of pregnancy and HBV infection to the date of delivery. Blood samples were collected at 12-24, 28-32, and 36-40 weeks of pregnancy for the measurement of genotype, HBsAg, hepatitis B e antigen (HBeAg), HBV DNA, HBV RNA, and liver function, including alanine transaminase, aspartate transaminase, total bilirubin, total bile acids, cholinesterase, alkaline phosphatase (ALP), and... (truncated)

MESH:D011715 - pyrantel

Summary:

LLM Prediction Score	0.063 (normalized)
LLM Confidence Score	0.980
Golden Answer (Severity Class)	0.0 (normalized)
Prediction Error	0.063

Retrieved Context:

Title: Gastrointestinal Parasites in Shelter Dogs: Occurrence, Pathology, Treatment and Risk to Shelter Workers.
Dogs entering shelters can carry gastrointestinal parasites that may pose serious risks to other animals, shelter staff and visitors. Shelters provide an environment that could facilitate the spread of parasitic infections between animals. Nematodes and protozoa that transmit through ingestion or skin penetration are major enteric parasites of concern in shelter settings. <i>Ancylostoma</i> spp., <i>Uncinaria stenocephala</i>, <i>Toxocara canis</i>, <i>Toxascaris leonina</i>, <i>Trichuris vulpis</i> and <i>Dipylidium caninum</i> are the major helminths while <i>Giardia</i>, <i>Cryptosporidium</i>, <i>Isospora</i> spp. and <i>Sarcocystis</i> spp. are the most prevalent protozoan parasites in shelter dogs. The prevalence of gastrointestinal parasites in shelter dogs is typically higher than in owned... (truncated)

Title: Association of CYP1A1 and CYP1B1 inhibition in in vitro assays with drug-induced liver injury.
Drug-induced liver injury (DILI) is one of the major causes for the discontinuation of drug development and withdrawal of drugs from the market. Since it is known that reactive metabolite formation and being substrates or inhibitors of cytochrome P450s (P450s) are associated with DILI, we systematically investigated the association between human P450 inhibition and DILI. The inhibitory activity of 266 DILI-positive drugs (DILI drugs) and 92 DILI-negative drugs (no-DILI drugs), which were selected from Liver Toxicity Knowledge Base (US Food and Drug Administration), against 8 human P450 forms was assessed using recombinant enzymes and luminescent substrates, and the threshold values... (truncated)

Title: Serious adverse reactions associated with ivermectin: A systematic pharmacovigilance study in sub-Saharan Africa and in the rest of the World.
Ivermectin is known to cause severe encephalopathies in subjects infected with loiasis, an endemic parasite in Sub-Saharan Africa (SSA). In addition, case reports have described ivermectin-related serious adverse drug reactions (sADRs) such as toxidermias, hepatic and renal disorders. The aim of this study was to identify suspected sADRs reported after ivermectin administration in VigiBase, the World Health Organization's global individual case safety reports database and analyze their frequency relative to the frequency of these events after other antinematodal drugs reported in SSA and other areas of the world (ROW).

Title: Who Let the Dogs Out? Unmasking the Neglected: A Semi-Systematic Review on the Enduring Impact of Toxocariasis, a Prevalent Zoonotic Infection.
Toxocariasis remains an important neglected parasitic infection representing one of the most common zoonotic infections caused by the parasite <i>Toxocara canis</i> or, less frequently, by <i>Toxocara cati</i>. The epidemiology of the disease is complex due to its transmission route by accidental ingestion of embryonated <i>Toxocara</i> eggs or larvae from tissues from domestic or wild paratenic hosts. Even though the World Health Organization and Centers for Disease Control classified toxocariasis amongst the top six parasitic infections of priority to public health, global epidemiological data regarding the relationship between seropositivity and toxocariasis is limited. Although the vast majority of the infected individuals... (truncated)

Title: Pharmacologic advances in canine and feline reproduction.
Substantial improvements in therapeutic options for companion animal reproduction and gynecologic emergencies have been made over the last decade. New, alternative drug treatments, with fewer side effects and improved efficacy, are available. This has widened the spectrum of therapeutic possibilities for diseases that were previously treated only by surgical intervention. New drugs are available for estrus induction and pregnancy termination, as well as for the treatment of pyometra. This review summarizes the pharmacology and toxicology of reproductive agents currently in use for contraception, pyometra, dystocia, eclampsia, premature labor, agalactia, mastitis, metritis, and prostatic disorders, and compares their efficacy and safety... (truncated)

MESH:C080163 - trovafloxacin

Summary:

LLM Prediction Score	0.938 (normalized)
LLM Confidence Score	0.890
Golden Answer (Severity Class)	1.0 (normalized)
Prediction Error	0.062

Retrieved Context:

Title: Comparative tolerability of the newer fluoroquinolone antibacterials.
The most common adverse effects of the fluoroquinolones involve the gastrointestinal tract, skin and CNS, and are mainly mild and reversible. Of the gastrointestinal events, nausea and vomiting are the most common. Mild hepatic reactions are a class effect, usually presenting as mild transaminase level increases without clinical symptoms. However, postmarketing surveillance has revealed significant hepatotoxicity with trovafloxacin. It is not currently known whether the severe reactions to trovafloxacin are specific to that agent or simply represent an extreme of an emerging class effect. The enormous worldwide usage of, and extensive published adverse effect data on the other fluoroquinolones and... (truncated)

Title: Acyl-glucuronide as a Possible Cause of Trovafloxacin-Induced Liver Toxicity: Induction of Chemokine (C-X-C Motif) Ligand 2 by Trovafloxacin Acyl-glucuronide.
Trovafloxacin is an antibiotic that was withdrawn from the market relatively soon after its release due to the risk of hepatotoxicity. Trovafloxacin is mainly metabolized to its acyl-glucuronide by uridine 5'-diphosphate (UDP)-glucuronosyltransferase (UGT) 1A1. In this study, we examined whether the acyl-glucuronide is involved in the development of hepatotoxicity. A UGT1A1-induced cell model was developed and the toxicity of trovafloxacin acyl-glucuronide was evaluated. The UGT1A1-induced cell model was developed by treating HepG2 cells with chrysin for 48 h. Chemokine (C-X-C motif) ligand 2, a cytokine involved in drug-induced liver injury, was uniquely induced by trovafloxacin in the UGT1A1-induced HepG2 cells.... (truncated)

Title: Trovafloxacin hepatotoxicity.
A 19-year-old woman was treated for recurrent sinusitis with oral trovafloxacin and developed acute hepatitis and peripheral eosinophilia, with hepatosplenomegaly and ascites. Laparoscopic liver biopsy showed extensive centrilobular hepatocyte necrosis, likely causing venoocclusive disease-like signs and symptoms. Clinical and laboratory abnormalities resolved completely after prolonged treatment with steroids. The temporal relationship between trovafloxacin and the onset of hepatitis favors this drug as a culprit.

Title: Trovafloxacin enhances the inflammatory response to a Gram-negative or a Gram-positive bacterial stimulus, resulting in neutrophil-dependent liver injury in mice.
Trovafloxacin (TVX), a fluoroquinolone antibiotic, has been strongly linked with several cases of idiosyncratic hepatotoxicity in humans. Previous studies showed that a modest inflammatory stress induced by a Gram-negative bacterial stimulus [i.e., lipopolysaccharide (LPS)] rendered nontoxic doses of TVX hepatotoxic in mice. This study compared the interaction of TVX with Gram-negative and Gram-positive stimuli. Mice were given TVX 3 h before LPS (Gram-negative stimulus) or a peptidoglycan-lipoteichoic acid (PGN-LTA) mixture isolated from Staphylococcus aureus (Gram-positive stimulus). Administration of TVX, LPS, or PGN-LTA alone was nonhepatotoxic. However, TVX administration before PGN-LTA or LPS resulted in significant liver injury that occurred with... (truncated)

Title: Microarray analysis in human hepatocytes suggests a mechanism for hepatotoxicity induced by trovafloxacin.
Idiosyncratic drug toxicity, defined as toxicity that is dose independent, host dependent, and usually cannot be predicted during preclinical or early phases of clinical trials, is a particularly confounding complication of drug development. An understanding of the mechanisms that lead to idiosyncratic liver toxicity would be extremely beneficial for the development of new compounds. We used microarray analysis on isolated human hepatocytes to understand the mechanisms underlying the idiosyncratic toxicity induced by trovafloxacin. Our results clearly distinguish trovafloxacin from other marketed quinolone agents and identify unique gene changes induced by trovafloxacin that are involved in mitochondrial damage, RNA processing, transcription,... (truncated)

MESH:D003707 - demeclocycline

Summary:

LLM Prediction Score	0.314 (normalized)
LLM Confidence Score	0.970
Golden Answer (Severity Class)	0.375 (normalized)
Prediction Error	0.061

Retrieved Context:

Title: Demethylchlortetracycline overdosage. A case report of toxic effects in a patient with impaired renal function.
No abstract available.

Title: [Clinical use of tetracyclines in the treatment of periodontal diseases].
There are a number of chemically different tetracycline homologues. The older group of tetracyclines, which was introduced in the 1950-60s, includes tetracycline, oxytetracycline, chlortetracycline and demeclocycline. The newer group of tetracyclines includes doxycycline, methacycline and minocycline.

Title: Pharmacokinetics of conivaptan use in patients with severe hepatic impairment.
Label="PURPOSE" NlmCategory="OBJECTIVE">Conivaptan is an intravenous dual V_{1A}/V₂ vasopressin antagonist approved for the treatment of euvolemic and hypervolemic hyponatremia. Earlier studies showed that patients with moderate liver disease could be safely treated with conivaptan by reducing the dose by 50%, whereas patients with mild hepatic impairment needed no dose adjustment. The objective of this Phase 1, open-label study was to assess the pharmacokinetics, protein binding, and safety of 48 h of conivaptan infusion in individuals with severe hepatic impairment.

Title: Stevens-Johnson syndrome and toxic epidermal necrolysis (SJS/TEN): could retinoids play a causative role?
Stevens-Johnson syndrome and toxic epidermal necrolysis (SJS/TEN) are overlapping manifestations on a spectrum of acute drug-induced conditions associated with severe blistering, skin peeling, and multi-organ damage. TEN is an eruption resembling severe scalding, with ≥30% skin detachment. SJS is a mild form of TEN, characterized histologically by epidermal keratinocyte apoptosis with dermo-epidermal separation and extensive small blisters with <10% body surface skin detachment. The syndrome can be induced by numerous medications and typically occurs 1-4 weeks after the initiation of therapy. Granulysin is found in the lesions of patients with SJS/TEN and plays a significant pathogenic role in the condition,... (truncated)

Title: Effect of trichloroacetaldehyde on the activation of CD4⁺T cells in occupational medicamentosa-like dermatitis: An in vivo and in vitro study.
Occupational medicamentosa-like dermatitis induced by trichloroethylene (OMLDT) is a hypersensitivity disease with autoimmune liver injury, which has increasingly become a serious occupational health problem in China. However, the pathogenesis of OMLDT remained undefined. In this study, 30 TCE-induced OMLDT patients, 58 exposure controls, and 40 non-exposure controls were recruited. We showed that the ratio of activated CD4⁺ T cells (downregulation of CD62 L) was dramatically increased in OMLDT patients compared to exposure and non-exposure control, suggesting that CD4⁺ T cells activation was a key cellular event in the development of OMLDT. In parallel, the expression of cytokine including IL-2, IFN-γ, TNF-α... (truncated)

MESH:D000077269 - lenalidomide

Summary:

LLM Prediction Score	0.436 (normalized)
LLM Confidence Score	0.990
Golden Answer (Severity Class)	0.375 (normalized)
Prediction Error	0.061

Retrieved Context:

Title: A Case of Drug-Induced Hepatitis due to Lenalidomide.
Lenalidomide is a recent thalidomide analog used for the treatment of refractory multiple myeloma. The main toxicity of this drug consists in severe neutropenia and thrombocytopenia. Lenalidomide-associated liver injury is rare, manifesting itself as elevated liver enzymes and hyperbilirubinemia reversible upon weeks after drug withdrawal. We report here in detail the clinical course as well as the biological and histological alterations of an acute lenalidomide-induced liver injury. Findings on liver biopsy allowed us to discriminate acute inflammatory changes due to the drug and minor associated lesions of graft-versus-host disease in this patient with recurrent myeloma after allogeneic bone marrow transplantation.

Title: Lenalidomide-Associated ITP.
Lenalidomide is a potent immunomodulatory agent being used increasingly for treatment of hematologic malignancies including multiple myeloma and myelodysplasia. The common toxicities of lenalidomide, including dose-limiting myelosuppression, are well described. However, the immunomodulatory properties of lenalidomide may give rise to unexpected autoimmune complications. Herein, we describe a case of immune thrombocytopenic purpura (ITP) associated with use of lenalidomide.

Title: Association of pharmacokinetic profiles of lenalidomide in human plasma simulated using pharmacokinetic data in humanized-liver mice with liver toxicity detected by human serum albumin RNA.
Lenalidomide has been shown to be potentially teratogenic in thalidomide-sensitive animal species. Screening for thalidomide analogs devoid of teratogenicity/toxicity-attributable to drug metabolism and disposition, but having immunomodulatory properties-is a strategic pathway towards development of new anticancer drugs. Plasma concentrations of lenalidomide were investigated in immunodeficient control and humanized-liver mice following oral administration of lenalidomide (50 mg/kg). Plasma concentrations of lenalidomide (1-2 hr after administration) were slightly but significantly higher in humanized-liver mice than in control mice ($p < 0.05$). Human albumin mRNA, a liver-specific toxicity marker, was found in the blood of humanized-liver mice 24 hr after lenalidomide administration. Simulations... (truncated)

Title: Successful treatment with lenalidomide of secondary multiple myeloma with extramedullary liver plasmacytoma in a renal transplant recipient: A case report and review of the literature.
Multiple myeloma (MM) represents a rare form of post-transplantation lymphoproliferative disorder, and the presence of plasma cells in the liver is generally associated with aggressive forms of MM. In the present study, an unusual case of extramedullary plasmacytoma, affecting the liver and vertebrae of a recipient of a renal transplant, is reported. The patient had been previously treated with bortezomib for an MM following renal transplantation, as diagnosed by percutaneous needle biopsy of the hepatic lesion. He was then treated with 5 cycles of RCD regimen (lenalidomide, 25 mg, days 1-21; cyclophosphamide. 50-100 mg, days 1-21; and dexamethasone, 20 mg,... (truncated)

Title: Low dose continuous lenalidomide in heavily pretreated patients with relapsed or refractory classical Hodgkin lymphoma: a retrospective case series.
Patients with relapsed or refractory (R/R) classical Hodgkin lymphoma (cHL) following autologous stem cell transplant (ASCT) remain a management challenge with few reliably effective treatments. Lenalidomide, an immunomodulatory drug approved for patients with myelodysplastic syndrome with del(5q), multiple myeloma, and mantle cell lymphoma, has demonstrated some activity in patients with R/R cHL, though the toxicity of traditional doses and schedules has been a barrier to consistent use. Low dose continuous (LDC) schedules have emerged as promising, with a more favorable safety profile. We report herein that LDC schedules are associated with a far more tolerable toxicity profile, and exhibit at... (truncated)

MESH:D003975 - diazepam

Summary:

LLM Prediction Score	0.439 (normalized)
LLM Confidence Score	0.990
Golden Answer (Severity Class)	0.5 (normalized)
Prediction Error	0.061

Retrieved Context:

Title: [Clinical problems in the application of sedatives in patients with chronic liver disease (author's transl)].
In these introductory remarks the application of sedatives in patients with chronic liver diseases is discussed. Sedatives, for example, are used in agitated alcoholics with liver damage, as premedication before endoscopical examinations (emergency endoscopy for hemorrhage from the upper G.i.-tract, peritoneoscopy), and operations necessary for the treatment of typical complications of the liver disease (shunt-op. for portal hypertension). In all these situations the metabolism and pharmaco-kinetics of sedatives may be influenced by the liver disease in a non-predictable way. Generally, an enhanced, enforced and prolonged effect can be assumed. Long acting barbiturates are recommended for a long period treatment, diazepam... (truncated)

Title: Does high-dose benzodiazepine abuse really produce liver toxicity? Results from a series of 201 benzodiazepine monoabusers.
Several side-effects related to prolonged benzodiazepines (BZD) use have been reported. Given the primary role of liver in BZD metabolism, toxicity related to prolonged high-dose BZD use could be conceivable. No data are available on the long-term impact of high-dose BZD use on liver.

Title: Acute Liver Failure due to Trazodone and Diazepam.
Most antidepressant agents have the potential to cause liver injury, even at therapeutic doses. Nevertheless, drug-induced liver injury (DILI) from antidepressant agents is a rare event. There is no way to prevent idiopathic DILI, but the severity of the reaction may be minimized with prompt recognition and early withdrawal of the agent. We describe a rare case of a 63-year-old man presenting with acute liver failure after 3 months of trazodone and diazepam administration at normal therapeutic doses, requiring liver transplantation. This report should increase physicians' awareness of this complication and call attention to the regular monitoring of liver tests... (truncated)

Title: Case Report: Enhanced Diazepam Elimination With the Molecular Adsorbents Recirculating System (MARS) in Severe Autointoxication: A Survival Case Report.
Objective: Due to the extensive use of diazepam worldwide, self-induced intoxication is very common, yet rarely fatal. Nevertheless, the management of intoxication caused by extremely high doses of diazepam is not known, as well as the effectiveness of flumazenil, a specific benzodiazepine (BDZ) antagonist. Here we present the first report on the enhanced elimination (clearance) of diazepam using the Molecular Adsorbents Recirculating System (MARS) following autointoxication with an extremely high dose as part of a suicide attempt. Case: A 44-year-old male patient was admitted to the ICU because of impaired consciousness following the ingestion of 20 g of diazepam. Blood... (truncated)

Title: Psychotropic drugs and liver disease: A critical review of pharmacokinetics and liver toxicity.
The liver is the organ by which the majority of substances are metabolized, including psychotropic drugs. There are several pharmacokinetic changes in end-stage liver disease that can interfere with the metabolization of psychotropic drugs. This fact is particularly true in drugs with extensive first-pass metabolism, highly protein bound drugs and drugs depending on phase I hepatic metabolic reactions. Psychopharmacological agents are also associated with a risk of hepatotoxicity. The evidence is insufficient for definite conclusions regarding the prevalence and severity of psychiatric drug-induced liver injury. High-risk psychotropics are not advised when there is pre-existing liver disease, and after starting a... (truncated)

MESH:D014635 - valproic acid

Summary:

LLM Prediction Score	0.939 (normalized)
LLM Confidence Score	0.990
Golden Answer (Severity Class)	1.0 (normalized)
Prediction Error	0.061

Retrieved Context:

Title: [Acute hepatic failure associated with valproic acid in children. Report of 3 cases].
We report the cases of three epileptic children who developed hepatotoxicity induced by valproic acid. Two patients had developmental delay. Including the one who died, all patients were receiving polytherapy (carbamazepine in two and phenobarbital in one). The patients age ranged from 2 years and 8 months to 5 years and 1 month. The onset of hepatic complications occurred within 6 months of valproate therapy in two patients and 12 months in one. All patients developed the classical clinical signs of hepatotoxicity. Vomiting, edema and jaundice were the initial symptoms. Fever occurred in two patients. The serum levels of glutamic... (truncated)

Title: Severe hepatotoxicity during valproate therapy: an update and report of eight new fatalities.
Since our last report on valproate (VPA)-related hepatotoxicity in 1988, 8 other children have died of VPA-associated liver failure in Germany and Switzerland. We compared the clinical course of these children with that of 6 children with a reversible outcome of severe hepatotoxicity related to VPA. Thirty-five percent of patients with fatal liver failure were normally developed, 23.5% were receiving VPA monotherapy, and 35.3% were aged < or = 2 years. The initial clinical symptoms of VPA-related hepatotoxicity were nausea, vomiting, apathy or coma, and increasing seizures in more than 50% of patients, in combination with febrile infections at onset... (truncated)

Title: Valproic acid hepatic fatalities. III. U.S. experience since 1986.
We report the results of a third retrospective study of the U.S. experience with fatal hepatotoxicity associated with valproic acid (VPA). In the United States, over one million patients received new prescriptions for VPA during the years 1987 to 1993, and 29 patients developed fatal hepatotoxicity. Decreased alertness, jaundice, vomiting, hemorrhage, increased seizures, anorexia, and edema were the most common presenting signs. Risk factors included young age, polytherapy, developmental delay, and coincident metabolic disorders. Patients less than 2 years old receiving VPA as polytherapy were at the greatest risk (1:600) of developing this complication.

Title: A direct hepatotoxic effect of valproic acid.
Nine patients treated with valproic acid had low plasma fibrinogen concentration and other liver function abnormalities. Several patients also had a low platelet count and prolonged bleeding time. The abnormal values returned promptly to normal when the drug therapy was discontinued or the dosage reduced. The liver function abnormalities are consistent with a direct, dose-related hepatotoxicity.

Title: Valproic acid induced acute liver injury resulting in hepatic encephalopathy- a case report and literature review.
Valproic acid (VPA) is a commonly used agent in the management of seizures and psychiatric disorders. Hyperammonemia is a common complication of VPA with 27.8% of patients having elevated levels - that is unrelated to hepatotoxicity and normal transaminases. Common side effects include obesity, insulin resistance, metabolic disorder and severe forms of hepatotoxicity. Other rare and idiosyncratic reactions have been reported, one of which is presented in our case. A 27-year old patient presented with hyperammonemia and encephalopathy as a consequence of idiosyncratic VPA reaction causing drug-induced liver injury (DILI) with severely elevated transaminases. DILI is commonly overlooked when investigating... (truncated)

MESH:C109932 - gadofosveset trisodium

Summary:

LLM Prediction Score	0.061 (normalized)
LLM Confidence Score	0.850
Golden Answer (Severity Class)	0.0 (normalized)
Prediction Error	0.061

Retrieved Context:

Title: Gadofosveset trisodium in the investigation of focal liver lesions in noncirrhotic liver: Early experience.
To investigate enhancement of hepatic lesions with gadofosveset trisodium (Gdfos) (Ablavar) (blood pool agent) compared with the extracellular gadolinium contrast agent (EcGd) gadobutrol (Gadovist).

Title: Compensatory biliary and urinary excretion of gadobenate ion after administration of gadobenate dimeglumine (MultiHance(®)) in cases of impaired hepatic or renal function: a mechanism that may aid in the prevention of nephrogenic systemic fibrosis?
To determine whether increased elimination of gadobenate ion via the hepatobiliary pathway might compensate for reduced/absent elimination via the urinary pathway in the event of compromised renal function, as a possible protective mechanism against nephrogenic systemic fibrosis (NSF).

Title: Magnetic Resonanance Imaging of the Liver (Including Biliary Contrast Agents)-Part 2: Protocols for Liver Magnetic Resonanance Imaging and Characterization of Common Focal Liver Lesions.
No abstract available.

Title: Advances in gadolinium-based MRI contrast agent designs for monitoring biological processes in vivo.
The gadolinium-based contrast agents widely used in diagnostic MRI exams for 30 years are all small molecule agents that distribute into all extracellular spaces in tissues without providing any specific biological information. Although many 'responsive agent' designs have been presented over the past 20 years or so, none have found use in clinical diagnostic medicine at this point. This review summarizes some recent approaches taken to enhance the sensitivity of such gadolinium-based agents, to target them to specific tissue components, and to create new systems for monitoring specific biological processes.

Title: Use of contrast agents in oncological imaging: magnetic resonance imaging.
Magnetic resonance plays a leading role in the management of oncology patients, providing superior contrast resolution and greater sensitivity compared with other techniques, which enables more accurate tumor identification, characterization and staging. Contrast agents are widely used in clinical magnetic resonance imaging; approximately 40-50% of clinical scans are contrast enhanced. Most contrast agents are based on the paramagnetic gadolinium ion Gd^{3+}, which is chelated to avoid the toxic effects of free gadolinium. Multiple factors such as molecule structure, molecule concentration, dose, field strength and temperature determine the longitudinal and transverse relaxation rates (R1 and R2, respectively) and thus the T1-... (truncated)

MESH:D003024 - clozapine

Summary:

LLM Prediction Score	0.685 (normalized)
LLM Confidence Score	0.990
Golden Answer (Severity Class)	0.625 (normalized)
Prediction Error	0.060

Retrieved Context:

Title: [Liver cell necrosis during administration of clozapine].
A 38-year-old woman was treated for mutism with clozapine. After a week liver function disturbances developed, which disappeared when the treatment was discontinued. Histopathological investigation of a liver biopsy specimen revealed extensive liver cell necrosis. So far two patients have been described with cholestatic jaundice induced by clozapine, and one patient with toxic hepatitis due to clozapine.

Title: [Clozapine-induced toxic hepatitis].
A case of clozapine-induced toxic hepatitis in a 49-year old woman with schizophrenia is described. The daily clozapine dose was clinically titrated to 300 mg. Subsequently, the patient experienced lethargy and anorexia, and fever, eosinophilia, leucocytosis and abnormal liver parameters were found. The serum concentration of clozapine was 8595 nmol/l, and treatment was discontinued. After eight days, the condition stabilised, and low-dose clozapine treatment was successfully reinstituted with serum monitoring (TDM).

Title: Eosinophilia, pleural effusion, hepatitis, and jaundice occurring early in clozapine treatment.
Clozapine use is associated with various adverse events, some of which have received little attention, including eosinophilia, pleural effusion, and hepatitis. Because of the fatality of jaundice with hepatitis, it is necessary to understand the course and management of clozapine-induced eosinophilia and hepatitis. We report on a case in which the eosinophil count began to increase shortly after clozapine use, and pleural effusion and fever then developed at the time eosinophilia was at its peak level. Jaundice with hepatitis consecutively developed when all the above symptoms subsided. The liver function recovered rapidly after clozapine was discontinued. We recommend that patients... (truncated)

Title: Evaluation of side effects due to clozapine in long-term treatment of psychosis.
17 patients (13 males and 4 females) were examined for side effects due to long-term treatment with 8-chloro-11-(4-methylpiperazin-1-yl)-5H-dibenzo(b,e)(1,4)diazepine (clozapine) with emphasis on alterations in bone marrow, liver, and kidney function. 16 patients were schizophrenic, one patient was diagnosed psychosis e causa dubia. The age range was 25-68 years, with an average of 38.2 years. The daily clozapine dose varied from 225 to 800 mg with a total intake during the trial period of 149.1-891.6 g yielding an average of 479.8 g. The treatment period varied from 20 to 51 months, with an average of 35.9 months. None of the patients... (truncated)

Title: Hepatotoxicity induced by clozapine: a case report and review of literature.
Clozapine is an effective antipsychotic drug but is associated with serious side effects. Most treatment guidelines give no clear recommendations on regular monitoring of liver function tests, even though up to 60% of patients experience elevations in hepatic transaminases, with 15% to 30% experiencing an elevation two to three times greater than normal. Though elevations in liver function tests are often transient and asymptomatic, there are many reported cases of clozapine-induced hepatotoxicity, with damage to the liver, involvement of multiple organs, and even fulminant liver failure arising with moderate clozapine doses. This case report describes a Chinese woman who developed... (truncated)

MESH:D004221 - disulfiram

Summary:

LLM Prediction Score	0.940 (normalized)
LLM Confidence Score	0.990
Golden Answer (Severity Class)	1.0 (normalized)
Prediction Error	0.060

Retrieved Context:

Title: Disulfiram-induced hepatitis: case report and review of the literature.
A case of hepatitis is reported in a 38-year-old alcoholic woman taking disulfiram to aid in maintaining sobriety. She presented with anorexia, vomiting, fatigue, right upper-quadrant pain, pruritus, darkened urine, and jaundice after about two weeks of disulfiram 500 mg/d. The patient also had been taking enalapril 10 mg/d for one year. Hepatocellular enzymes, total bilirubin, and eosinophils were significantly elevated. Hepatitis B core antibody, hepatitis A antibody, hepatitis B surface antibody, and antinuclear antibody were negative. After discontinuation of disulfiram, the clinical and biochemical manifestations of hepatitis began to resolve and the patient was discharged in a much improved... (truncated)

Title: [Fulminant hepatitis caused by disulfiram].
After undergoing withdrawal treatment for alcoholism as an in-patient for one year a 49-year-old woman was started on disulfiram, 250 mg daily, her liver function tests being normal. Except for vitamin B1 she received no further medication. Jaundice developed 13 days after onset of treatment and acute liver failure was diagnosed on the 18th day after a total disulfiram dose of 4.5 g (Quick value < 10%; bilirubin 460 mumol/l; GPT 5099 U/l; GOT 4142 U/l), as well as early renal failure (creatinine 300 mumol/l). An acute viral infection, autoimmune hepatitis and a metabolic liver disease were excluded by biochemical,... (truncated)

Title: Disulfiram-induced hepatitis.
We reported a case of disulfiram-induced hepatitis with unique clinical features and compared our case with others in the literature. Our patient had headache, mild fever, nausea, vomiting, rash, and eosinophilia after 3 weeks of disulfiram therapy. Subsequent liver biopsy showed low-grade lobular hepatitis. After disulfiram therapy was discontinued, symptoms subsided and results of liver function tests returned to normal.

Title: Safety issues concerning the use of disulfiram in treating alcohol dependence.
Disulfiram is known to cause hepatitis, which is sometimes fatal. The best estimate of the frequency of disulfiram-induced fatal hepatitis is 1 case in 30,000 patients treated/year. Its appears to be more common in patients given disulfiram for the treatment of nickel sensitivity. Frequent blood testing for liver function is probably not necessary, but patients taking disulfiram should be in regular contact with a physician. There are rare reports of psychosis and confusional states in conjunction with disulfiram treatment and peripheral neuropathy and optic neuritis have been reported; these effects are dose-related. Psychiatric complications appear to be more common with... (truncated)

Title: Fatal liver failure due to disulfiram.
We present the case of a 30-year-old man with disulfiram-induced hepatitis complicated by fatal liver failure. The disease followed a protracted course with an interval of 25 days between the onset of jaundice and the onset of encephalopathy. In this patient, the severity of the liver disease might have been due to reingestion of disulfiram shortly after the onset of jaundice.

MESH:D018723 - bethanechol

Summary:

LLM Prediction Score	0.058 (normalized)
LLM Confidence Score	0.970
Golden Answer (Severity Class)	0.0 (normalized)
Prediction Error	0.058

Retrieved Context:

Title: The treatment of pheochromocytoma associated with pseudo-obstruction and perforation of the colon, hepatic failure, and DIC.
The case of a 59-year-old man with paralytic ileus (pseudo-obstruction) associated with pheochromocytoma is reviewed. Paralytic ileus is believed to have been the result of overstimulation of alpha and beta receptors on the intestine by catecholamines. Phentolamine, bunazocin, propranolol, bethanechol and midaglizole in single administrations or in combination were administered. Phentolamine infusion clearly relieved the symptom, but ileus recurred, and the patient died of respiratory failure, liver dysfunction and disseminated intravascular coagulation syndrome. The significant role of catecholamines in causing these symptoms is discussed, and the management of this relatively rare complication is reviewed.

Title: Cholinergic Signaling via Muscarinic Receptors Directly and Indirectly Suppresses Pancreatic Tumorigenesis and Cancer Stemness.
In many solid tumors, parasympathetic input is provided by the vagus nerve, which has been shown to modulate tumor growth. However, whether cholinergic signaling directly regulates progression of pancreatic ductal adenocarcinoma (PDAC) has not been defined. Here, we found that subdiaphragmatic vagotomy in LSL-<i>Kras</i> ^{+/G12D};<i>Pdx1</i>-Cre (KC) mice accelerated PDAC development, whereas treatment with the systemic muscarinic agonist bethanechol restored the normal KC phenotype, thereby suppressing the accelerated tumorigenesis caused by vagotomy. In LSL-<i>Kras</i> ^{+/G12D};LSL-<i>Trp53</i> ^{+/R172H};<i>Pdx1</i>-Cre mice with established PDAC, bethanechol significantly extended survival. These effects were mediated in part through CHRM1, which inhibited downstream MAPK/EGFR and PI3K/AKT pathways in PDAC... (truncated)

Title: Effect of trichloroacetaldehyde on the activation of CD4⁺T cells in occupational medicamentosa-like dermatitis: An in vivo and in vitro study.
Occupational medicamentosa-like dermatitis induced by trichloroethylene (OMLDT) is a hypersensitivity disease with autoimmune liver injury, which has increasingly become a serious occupational health problem in China. However, the pathogenesis of OMLDT remained undefined. In this study, 30 TCE-induced OMLDT patients, 58 exposure controls, and 40 non-exposure controls were recruited. We showed that the ratio of activated CD4⁺ T cells (downregulation of CD62 L) was dramatically increased in OMLDT patients compared to exposure and non-exposure control, suggesting that CD4⁺ T cells activation was a key cellular event in the development of OMLDT. In parallel, the expression of cytokine including IL-2, IFN-γ, TNF-α... (truncated)

Title: Biopolymer Extracted from <i>Anadenanthera colubrina</i> (Red Angico Gum) Exerts Therapeutic Potential in Mice: Antidiarrheal Activity and Safety Assessment.
<i>Anadenanthera colubrina</i> var. cebil (Griseb.) Altschul (Fabaceae family), commonly known as the red angico tree, is a medicinal plant found throughout Brazil's semi-arid area. In this study, a chemical analysis was performed to investigate the antidiarrheal activity and safety profile of red angico gum (RAG), a biopolymer extracted from the trunk exudate of <i>A. colubrina</i>. Upon FT-IR spectroscopy, RAG showed bands in the regions of 1608 cm⁻¹, 1368 cm⁻¹, and 1029 cm⁻¹, which relate to the vibration of O-H water molecules, deformation vibration of C-O bands, and vibration of the polysaccharide C-O band, respectively, all of which are relevant to... (truncated)

Title: Obesity as an Early Symptom of the AMIS Syndrome.
We review evidence that the AMIS (Absence of Meal-induced Insulin Sensitization) syndrome describes a paradigm fundamental to development of obesity. The hypoglycemic response to a pulse of insulin is doubled after a meal as a result of Hepatic Insulin Sensitizing Substance (HISS), released from the liver to act selectively on muscle, heart and kidney. In the absence of HISS action, the hypoglycemic response to insulin is the same as in the fasted state, and only

MESH:D001414 - bacitracin

Summary:

LLM Prediction Score	0.057 (normalized)
LLM Confidence Score	0.980
Golden Answer (Severity Class)	0.0 (normalized)
Prediction Error	0.057

Retrieved Context:

Title: [Prevention of bacterial infections after bone marrow graft by broad-spectrum oral antibiotics, absorbable (pefloxacin, penicillin) and non absorbable (cephalosporin, gentamycin, bacitracin)].
In a consecutive series of 65 patients treated by allogeneic bone marrow transplantation, we have compared the efficacy on prevention of bacterial infection of total gut decontamination with oral non absorbable antibiotics or with oral absorbable broad spectrum antibiotics. On day-8, all patients were randomly allocated to one group: Group I received orally pefloxacin 400 mg/day and penicillin 3 M UI/day. Group II received capsules containing cephalothin 250 mg, gentamicin 20 mg and bacitracin 150 UI. Each patient received 9 to 12 capsules per day according to body weight. Patients less than 5 years old or with severe hepatic abnormalities... (truncated)

Title: Necrotic enteritis in broiler chickens: disease characteristics and prevention using organic antibiotic alternatives - a comprehensive review.
In line with the substantial increase in the broiler industry worldwide, Clostridium perfringens-induced necrotic enteritis (NE) became a continuous challenge leading to high economic losses, especially after banning antimicrobial growth promoters in feeds by many countries. The disease is distributed worldwide in either clinical or subclinical form, causing a reduction in body weight or body weight gain and the feed conversion ratio, impairing the European Broiler Index or European Production Efficiency Factor. There are several predisposing factors in the development of NE. Clinical signs varied from inapparent signs in case of subclinical infection (clostridiosis) to obvious enteric signs (morbidity), followed... (truncated)

Title: Effect of Dietary Supplementation of <i>Bacillus subtilis</i> on Growth Performance, Organ Weight, Digestive Enzyme Activities, and Serum Biochemical Indices in Broiler.
This study was conducted to investigate the effects of supplementing Bacillus subtilis and an antibiotic (Zinc bacitracin) in the diet of broilers on growth performance, organ weight, blood metabolites, and digestive enzymes of broiler chickens. A total of 600 1-d Arbor Acres broilers were randomly allotted to five treatments. Each treatment consisted of six replicates with four pens, and each pen had five birds. The chicks were fed (1) the basal diet (control), (2) the basal diet with 500 mg/kg Zinc bacitracin (APZ), (3) the basal diet with B. subtilis at 1 × 108 CFU/g (B.Sut-1), (4) the basal diet... (truncated)

Title: Hepatic progenitor cell activation is induced by the depletion of the gut microbiome in mice.
The homeostasis of the gut microbiome is crucial for human health and for liver function. However, it has not been established whether the gut microbiome influence hepatic progenitor cells (HPCs). HPCs are capable of self-renewal and differentiate into hepatocytes and cholangiocytes; however, HPCs are normally quiescent and are rare in adults. After sustained liver damage, a ductular reaction occurs, and the number of HPCs is substantially increased. Here, we administered five broad-spectrum antibiotics for 14 days to deplete the gut microbiomes of male C57BL/6 mice, and we measured the plasma aminotransferases and other biochemical indices. The expression levels of two... (truncated)

Title: Novel insights into the organic solute transporter alpha/beta, OSTα/β: From the bench to the bedside.
Organic solute transporter alpha/beta (OSTα/β) is a heteromeric solute carrier protein that transports bile acids, steroid metabolites and drugs into and out of cells. OSTα/β protein is expressed in various tissues, but its expression is highest in the gastrointestinal tract where it facilitates the recirculation of bile acids from the gut to the liver. Previous studies established that OSTα/β is upregulated in liver tissue of patients with extrahepatic cholestasis, obstructive cholestasis, and primary biliary cholangitis (PBC), conditions that are characterized by elevated bile acid concentrations in the liver and/or systemic circulation. The discovery that OSTα/β is highly upregulated in the... (truncated)

MESH:D007556 - isoxsuprine

Summary:

LLM Prediction Score	0.057 (normalized)
LLM Confidence Score	0.880
Golden Answer (Severity Class)	0.0 (normalized)
Prediction Error	0.057

Retrieved Context:

Title: Jatrorrhizine Improves Endothelial Function in Diabetes and Obesity through Suppression of Endoplasmic Reticulum Stress.
Jatrorrhizine (JAT) is one of the major bioactive protoberberine alkaloids found in rhizoma coptidis, which has hypoglycemic and hypolipidemic potential. This study aimed to evaluate the vasoprotective effects of JAT in diabetes and obesity and the underlying mechanism involved. Mouse aortas, carotid arteries and human umbilical cord vein endothelial cells (HUVECs) were treated with risk factors (high glucose or tunicamycin) with and without JAT ex vivo and in vitro. Furthermore, aortas were obtained from mice with chronic treatment: (1) control; (2) diet-induced obese (DIO) mice fed a high-fat diet (45% kcal% fat) for 15 weeks; and (3) DIO mice orally... (truncated)

Title: Equine idiopathic hemorrhagic cystitis: Clinical features and comparison with bladder neoplasia.
A new syndrome of hematuria in horses has been documented.

Title: Collaborative SAR Modeling and Prospective In Vitro Validation of Oxidative Stress Activation in Human HepG2 Cells.
Oxidative stress is the consequence of an abnormal increase of reactive oxygen species (ROS). ROS are generated mainly during the metabolism in both normal and pathological conditions as well as from exposure to xenobiotics. Xenobiotics can, on the one hand, disrupt molecular machinery involved in redox processes and, on the other hand, reduce the effectiveness of the antioxidant activity. Such dysregulation may lead to oxidative damage when combined with oxidative stress overpassing the cell capacity to detoxify ROS. In this work, a green fluorescent protein (GFP)-tagged nuclear factor erythroid 2-related factor 2 (NRF2)-regulated sulfiredoxin reporter (Srxn1-GFP) was used to measure... (truncated)

Title: CSI position statement on management of heart failure in India.
No abstract available.

MESH:C472965 - fospropofol

Summary:

LLM Prediction Score	0.056 (normalized)
LLM Confidence Score	0.910
Golden Answer (Severity Class)	0.0 (normalized)
Prediction Error	0.056

Retrieved Context:

Title: Propofol: a review of its role in pediatric anesthesia and sedation.
Propofol is an intravenous agent used commonly for the induction and maintenance of anesthesia, procedural, and critical care sedation in children. The mechanisms of action on the central nervous system involve interactions at various neurotransmitter receptors, especially the gamma-aminobutyric acid A receptor. Approved for use in the USA by the Food and Drug Administration in 1989, its use for induction of anesthesia in children less than 3 years of age still remains off-label. Despite its wide use in pediatric anesthesia, there is conflicting literature about its safety and serious adverse effects in particular subsets of children. Particularly as children are... (truncated)

Title: Efficacy and Safety of Fospropofol Disodium for Injection in General Anesthesia Induction for Adult Patients: A Phase 3 Trial.
Background: Fospropofol disodium for injection (Fospropofol_{FD}) is a prodrug that is metabolized into propofol to produce a general anesthesia effect when administered intravenously. Objective: This study aimed to assess the efficacy and safety of Fospropofol_{FD} in comparison with propofol medium/long-chain fat emulsion injections (propofol-MCT/LCT) for general anesthesia induction in adult patients undergoing elective surgeries. Setting: Nine academic medical centers in China. Method: This multicenter, randomized, double-blind, double-simulated, controlled, and non-inferiority trial evaluated 540 eligible adult patients randomly assigned (2:1) to the intervention (20 mg/kg Fospropofol_{FD}) or control (2 mg/kg propofol-MCT/LCT) groups. Main Outcome Measure: The primary efficacy endpoint was the success rate,... (truncated)

Title: Perspectives on the role of fospropofol in the monitored anesthesia care setting.
Monitored anesthesia care (MAC) is a safe, effective, and appropriate form of anesthesia for many minor surgical procedures. The proliferation of outpatient procedures has heightened interest in MAC sedation agents. Among the most commonly used MAC sedation agents today are benzodiazepines, including midazolam, and propofol. Recently approved in the United States is fospropofol, a prodrug of propofol which hydrolyzes in the body by alkaline phosphatase to liberate propofol. Propofol liberated from fospropofol has unique pharmacological properties, but recently retracted pharmacokinetic (PK) and pharmacodynamic (PD) evaluations make it difficult to formulate clear conclusions with respect to fospropofol's PK/PD properties. In safety... (truncated)

Title: Infectious Disease Risk Associated with Contaminated Propofol Anesthesia, 1989-2014(1).
Administration of propofol, the most frequently used intravenous anesthetic worldwide, has been associated with several iatrogenic infections despite its relative safety. Little is known regarding the global epidemiology of propofol-related outbreaks and the effectiveness of existing preventive strategies. In this overview of the evidence of propofol as a source of infection and appraisal of preventive strategies, we identified 58 studies through a literature search in PubMed, Embase, and Lilacs for propofol-related infections during 1989-2014. Twenty propofol-related outbreaks have been reported, affecting 144 patients and resulting in 10 deaths. Related factors included reuse of syringes for multiple patients and prolonged exposure... (truncated)

Title: Safety evaluation of fospropofol for sedation during minor surgical procedures.
To evaluate the safety of intravenous (IV) fospropofol when used to provide minimal to moderate sedation in patients undergoing minor surgical procedures.

MESH:D011239 - prednisolone

Summary:

LLM Prediction Score	0.430 (normalized)
LLM Confidence Score	0.990
Golden Answer (Severity Class)	0.375 (normalized)
Prediction Error	0.055

Retrieved Context:

Title: Hepatotoxicity and liver enzyme alteration in patients with immunobullous diseases receiving immunosuppressive therapy.
To avoid complications of high dose corticosteroid, pemphigus patients are usually co-treated with other immunosuppressive agents. Liver enzyme abnormality occurs commonly during treatment and occasionally causes discontinuation of drugs. To assess the rate of therapy-induced hepatotoxicity in patients with immunobullous diseases, we conducted a study of 250 pemphigus patients under immunosuppressive therapy prospectively. Aspartate aminotransferase (AST), alanine aminotransferase (ALT) and alkaline phosphatase (ALP) plasma levels were recorded before the start of treatment and every week under treatment (up to 3 weeks). Hepatotoxicity was defined as the rise in the ALT plasma levels to greater than twice the upper normal limit.... (truncated)

Title: Prednisolone: role in amoxicillin-clavulanate-induced cholestatic liver injury.
A 68-year-old patient presented with symptoms of a urinary tract infection. A deterioration in the patient's liver function tests (LFTs) was noted 1 week following completion of a course of amoxicillin-clavunalate. This progressively worsened, reaching its peak by day 30. Our investigations excluded other possible causes for deranged LFTs and there was no improvement of same despite reduced dosing of potentially hepatotoxic medications.A trial of 30 mg/day prednisolone was commenced, resulting in an immediate and progressive improvement in LFTs to baseline over a period of 22 days and an improvement in constitutional symptoms such as tiredness and poor appetite. Drug-induced... (truncated)

Title: Effect of short-term prednisolone therapy in patients with severe chronic type B hepatitis.
Ten patients with severe chronic type B hepatitis confirmed by liver biopsy were treated with prednisolone for eight weeks and followed up for more than one year. The patients were comprised of 6 males and 4 females, ages 17 to 45 (mean 32) yrs. Serum alanine aminotransferase (ALT) was elevated more than one month before the treatment in all (mean: 379 U/L, range: 87 to 772 U/L). Initial serological tests showed hepatitis B surface antigen (HBsAg) and hepatitis B e antigen (HBeAg) in all and hepatitis B virus DNA (HBV-DNA) in 7/10 (70%). Liver biopsy showed severe chronic active hepatitis... (truncated)

Title: The corticosteroid compounds prednisolone and vamorolone do not alter the nociception phenotype and exacerbate liver injury in sickle cell mice.
Clinicians often hesitate prescribing corticosteroids to treat corticosteroid-responsive conditions in sickle cell disease (SCD) patients because their use can be associated with complications (increased hospital readmission, rebound pain, strokes, avascular necrosis, acute chest syndrome). Consequently, SCD patients may receive suboptimal treatment for corticosteroid-responsive conditions. We conducted a preclinical trial of dissociative (vamorolone) and conventional (prednisolone) corticosteroid compounds to evaluate their effects on nociception phenotype, inflammation, and organ dysfunction in SCD mice. Prednisolone and vamorolone had no significant effects on nociception phenotype or anemia in homozygous mice. Conversely, prednisolone and vamorolone significantly decreased white blood cell counts and hepatic inflammation. Interestingly,... (truncated)

Title: Liver injury induced by high-dose methylprednisolone therapy: a case report and brief review of the literature.
Corticosteroids are used widely to treat many types of disease. In general, these drugs are considered safe for the liver; however, recent reports have demonstrated that high-dose methylprednisolone (MT) may cause severe liver injury. Here, we report a case of a 24-year-old female who was given pulsed MT therapy for multiple sclerosis. MT induced icteric hepatitis and impaired liver synthetic function. Hepatotoxicity developed several weeks after drug exposure, and the causal association with MT was confirmed by unintentional rechallenge test. A brief review of the literature on corticosteroid-induced hepatotoxicity is presented.

MESH:C081732 - iloperidone

Summary:

LLM Prediction Score	0.305 (normalized)
LLM Confidence Score	0.970
Golden Answer (Severity Class)	0.25 (normalized)
Prediction Error	0.055

Retrieved Context:

Title: Safety, tolerability, and risks associated with first- and second-generation antipsychotics: a state-of-the-art clinical review.
Since the discovery of chlorpromazine (CPZ) in 1952, first-generation antipsychotics (FGAs) have revolutionized psychiatric care in terms of facilitating discharge from hospital and enabling large numbers of patients with severe mental illness (SMI) to be treated in the community. Second-generation antipsychotics (SGAs) ushered in a progressive shift from the paternalistic management of SMI symptoms to a patient-centered approach, which emphasized targets important to patients - psychosocial functioning, quality of life, and recovery. These drugs are no longer limited to specific <i>Diagnostic and Statistical Manual of Mental Disorders</i> (<i>DSM</i>) categories. Evidence indicates that SGAs show an improved safety and tolerability profile... (truncated)

Title: Pharmacogenomics of adverse drug reactions.
Considerable progress has been made in identifying genetic risk factors for idiosyncratic adverse drug reactions in the past 30 years. These reactions can affect various tissues and organs, including liver, skin, muscle and heart, in a drug-dependent manner. Using both candidate gene and genome-wide association studies, various genes that make contributions of varying extents to each of these forms of reactions have been identified. Many of the associations identified for reactions affecting the liver and skin involve human leukocyte antigen (HLA) genes and for reactions relating to the drugs abacavir and carbamazepine, HLA genotyping is now in routine use prior... (truncated)

Title: The Importance of Patient-Specific Factors for Hepatic Drug Response and Toxicity.
Responses to drugs and pharmacological treatments differ considerably between individuals. Importantly, only 50%-75% of patients have been shown to react adequately to pharmacological interventions, whereas the others experience either a lack of efficacy or suffer from adverse events. The liver is of central importance in the metabolism of most drugs. Because of this exposed status, hepatotoxicity is amongst the most common adverse drug reactions and hepatic liabilities are the most prevalent reason for the termination of development programs of novel drug candidates. In recent years, more and more factors were unveiled that shape hepatic drug responses and thus underlie the... (truncated)

Title: Critical appraisal of lurasidone in the management of schizophrenia.
Lurasidone is a new atypical antipsychotic in the benzoisothiazoles class of chemicals. Like most second-generation antipsychotics it is a full antagonist at dopamine D(2) and serotonin 5-HT(2A) receptors, and is a partial agonist at 5-HT(1A) receptors, a property shared by some but not all older agents. It has much greater affinity for 5-HT(7) subtype receptors than other atypical antipsychotics. Pharmacokinetic studies showed that lurasidone is reasonably rapidly absorbed, with bioavailability appearing to be increased by food. Lurasidone undergoes extensive metabolism to a number of metabolites, some of which retain pharmacological activities. Metabolism is mainly by CYP3A4, resulting in steady-state concentrations... (truncated)

Title: Atypical antipsychotic metabolism and excretion.
The metabolic/biotransformation pathways of atypical antipsychotics (aripiprazole, clozapine, iloperidone, olanzapine, paliperidone, quetiapine, risperidone, and ziprasidone) have been characterized and reviewed. However, comparisons of excretory pathways remain unexplored.

MESH:D017984 - enoxaparin

Summary:

LLM Prediction Score	0.429 (normalized)
LLM Confidence Score	0.980
Golden Answer (Severity Class)	0.375 (normalized)
Prediction Error	0.054

Retrieved Context:

Title: Enoxaparin-Induced Liver Injury.
Enoxaparin, a form of low-molecular-weight heparin, can cause a rare, underreported, and often reversible form of hepatocellular injury. This report describes a case of enoxaparin-induced hepatotoxicity in a 61-year-old male diagnosed with pulmonary embolism. Elevations of liver enzymes were noted within 1 week of starting the drug, followed by a dramatic improvement upon its discontinuation, with subsequent normalization in the following days.

Title: Safety of fondaparinux versus enoxaparin after TKA in Japanese patients.
Fondaparinux and enoxaparin are useful for preventing venous thromboembolism after total knee arthroplasty (TKA), but both drugs have associated complications. The purpose of this study was to clarify the risks associated with use of these drugs in Japanese patients who underwent TKA.A total of 575 patients (935 knees) underwent TKA and were retrospectively reviewed; 277 patients (454 knees) were treated with fondaparinux and 298 patients (481 knees) were treated with enoxaparin. The authors investigated the incidences of deep venous thrombosis of the lower limbs and pulmonary embolism to evaluate venous thromboembolism, knee enlargement compared with the preoperative size, incidence of... (truncated)

Title: Hepatotoxicity of New Oral Anticoagulants (NOACs).
Case reports and analyses of clinical studies and of pharmacovigilance data suggest that new oral anticoagulants (NOACs) are associated with a small risk for hepatotoxicity. The objective of this publication is to summarize the current data about this subject, with a special emphasis on pharmacovigilance data in the World Health Organization (WHO) Global Individual Case Safety Reports (ICSR) database and on potential mechanisms of hepatotoxicity. For that, all available case reports as well as published analyses of clinical studies were obtained with a detailed search in PubMed. In addition, pharmacovigilance data from VigiBase(®), the WHO Global ICRS database, were extracted... (truncated)

Title: Heparin-binding copolymer reverses effects of unfractionated heparin, enoxaparin, and fondaparinux in rats and mice.
The parenteral anticoagulants may cause uncontrolled and life-threatening bleeding. Protamine, the only registered heparin antidote, is partially effective against low-molecular weight heparins, completely ineffective against fondaparinux and may cause unacceptable toxicity. Therefore, we aimed to develop a synthetic compound for safe and efficient neutralization of all parenteral anticoagulants. We synthesized pegylated PMAPTAC block copolymers, and then, we selected a lead heparin-binding copolymer (HBC). We assessed the effectiveness of HBC in the model of arterial thrombosis electrically induced in the carotid artery of rats by measuring thrombus weight, bleeding time, activated partial thromboplastin time, activated clotting time, and anti-factor Xa activity.... (truncated)

Title: Hepatotoxicity with cholestatic pattern secondary to enoxaparin treatment.
Treatment with low-molecular-weight heparins is very common in clinical practice. Exceptionally, some patients develop hepatitis within a few days of starting treatment, and rapid discontinuation of the drug is decisive to avoid chronification.

MESH:D000077726 - doripenem

Summary:

LLM Prediction Score	0.321 (normalized)
LLM Confidence Score	0.980
Golden Answer (Severity Class)	0.375 (normalized)
Prediction Error	0.054

Retrieved Context:

Title: Efficacy and safety of doripenem for sepsis with neutropenia in Japanese patients with hematologic diseases.
Doripenem (DRPM) is one of the carbapenems which has a broad-spectrum and strong activity against Pseudomonas aeruginosa. This observational study was conducted between April 2006 and March 2007 in Japan to evaluate the efficacy and safety of DRPM 0.5 g three times a day for sepsis with neutropenia in patients with hematologic diseases. One hundred-nineteen patients were enrolled from 34 medical institutes, comprising 117 patients for safety evaluation and 104 for efficacy evaluation. Monotherapy of DRPM 0.5 g three times a day (DRPM monotherapy) was evaluated in 73 patients. The response rates of DRPM monotherapy at 72 hours and at... (truncated)

Title: Gut Microbiota as Well as Metabolomes of Wistar Rats Recover within Two Weeks after Doripenem Antibiotic Treatment.
An understanding of the changes in gut microbiome composition and its associated metabolic functions is important to assess the potential implications thereof on host health. Thus, to elucidate the connection between the gut microbiome and the fecal and plasma metabolomes, two poorly bioavailable carbapenem antibiotics (doripenem and meropenem), were administered in a 28-day oral study to male and female Wistar rats. Additionally, the recovery of the gut microbiome and metabolomes in doripenem-exposed rats were studied one and two weeks after antibiotic treatment (i.e., doripenem-recovery groups). The 16S bacterial community analysis revealed an altered microbial population in all antibiotic treatments and... (truncated)

Title: Risk evaluation of carbapenem-induced liver injury based on machine learning analysis.
Information regarding carbapenem-induced liver injury is limited, and the rate of liver injury caused by meropenem (MEPM) and doripenem (DRPM) remains unknown. Decision tree (DT) analysis, a machine learning method, has a flowchart-like model where users can easily predict the risk of liver injury. Thus, we aimed to compare the rate of liver injury between MEPM and DRPM and construct a flowchart that can be used to predict carbapenem-induced liver injury.

Title: Overuse of antianaerobic drug is associated with poor postchemotherapy prognosis of patients with hepatocellular carcinoma.
Overuse of antibiotic drugs alters the composition of gut microbiota and has detrimental effects on the host. In our study, we investigated association of gut flora and antibiotics in the prognosis of patients with liver cancer who have undergone chemotherapy by analyzing two independent clinical studies. We retrospectively subanalyzed a previously reported randomized controlled trial (RCT) on hepatic arterial infusion chemotherapy in patients with hepatocellular carcinoma (HCC) to investigate the association between use of antibiotics and prognosis. In the other study, we prospectively determined the abundance of specific bacterial genus in patients with HCC by sequencing 16S ribosomal RNA and... (truncated)

Title: Deuterated drugs: where are we now?
Deuterated versions of existing drugs can exhibit improved pharmacokinetic or toxicological properties due the stronger deuterium- carbon bond modifying their metabolism. There is great interest in the current state of development of this approach.

MESH:D000077333 - telmisartan

Summary:

LLM Prediction Score	0.321 (normalized)
LLM Confidence Score	0.990
Golden Answer (Severity Class)	0.375 (normalized)
Prediction Error	0.054

Retrieved Context:

Title: Drug-induced liver injury after switching from tamoxifen to anastrozole in a patient with a history of breast cancer being treated for hypertension and diabetes.
Anastrozole is a selective non-steroidal aromatase inhibitor that blocks the conversion of androgens to estrogens in peripheral tissues. It is used as adjuvant therapy for early-stage hormone-sensitive breast cancer in postmenopausal women. Significant side effects of anastrozole include osteoporosis and increased levels of cholesterol. To date, seven case reports on anastrozole hepatotoxicity have been published. We report the case of an 81-year-old woman with a history of breast cancer, arterial hypertension, type 2 diabetes mellitus, hyperlipidemia, and chronic renal insufficiency. Four days after switching hormone therapy from tamoxifen to anastrozole, icterus developed along with a significant increase in liver enzymes... (truncated)

Title: Effect of Telmisartan or Losartan for Treatment of Nonalcoholic Fatty Liver Disease: Fatty Liver Protection Trial by Telmisartan or Losartan Study (FANTASY).
This study compared the effects of telmisartan and losartan on nonalcoholic fatty liver disease (NAFLD) and biochemical markers of insulin resistance in hypertensive NAFLD patients with type 2 diabetes mellitus.

Title: Effect of captopril and telmisartan on methotrexate-induced hepatotoxicity in rats: impact of oxidative stress, inflammation and apoptosis.
Methotrexate (MTX) is a commonly used antineoplastic and anti-rheumatoid drug whose efficacy is limited by its hepatotoxicity. The aim of this study was to investigate the possible protective role of captopril (100 mg/kg/day, p.o. for seven days), an angiotensin converting enzyme inhibitor, and telmisartan (10 mg/kg/day p.o. for seven days), an angiotensin II receptor blocker with peroxisome proliferative receptor gamma (PPARγ) agonism, in a model of MTX (single dose 20 mg/kg i.p. at the fifth day) induced hepatotoxicity in rats. Results of the present study revealed MTX-induced hepatotoxicity as demonstrated by increased level of liver enzymes and confirmed by histopathology.... (truncated)

Title: Telmisartan/hydrochlorothiazide-induced hepatotoxicity.
No abstract available.

Title: Telmisartan ameliorates fibrocystic liver disease in an orthologous rat model of human autosomal recessive polycystic kidney disease.
Human autosomal recessive polycystic kidney disease (ARPKD) produces kidneys which are massively enlarged due to multiple cysts, hypertension, and congenital hepatic fibrosis characterized by dilated bile ducts and portal hypertension. The PCK rat is an orthologous model of human ARPKD with numerous fluid-filled cysts caused by stimulated cellular proliferation in the renal tubules and hepatic bile duct epithelia, with interstitial fibrosis developed in the liver. We previously reported that a peroxisome proliferator activated receptor (PPAR)-γ full agonist ameliorated kidney and liver disease in PCK rats. Telmisartan is an angiotensin receptor blocker (ARB) used widely as an antihypertensive drug and shows... (truncated)

MESH:D000069059 - atorvastatin

Summary:

LLM Prediction Score	0.678 (normalized)
LLM Confidence Score	0.990
Golden Answer (Severity Class)	0.625 (normalized)
Prediction Error	0.053

Retrieved Context:

Title: Atorvastatin-induced prolonged cholestasis with bile duct damage.
We report a case of acute-onset, long-lasting cholestasis induced by atorvastatin. This antihyperlipidaemic drug was taken for 40 days by a 72-year-old male as a treatment for his mixed dyslipidaemia. At that point, the patient presented with asthenia, nausea, painless icterus, acholic stools and hyperchromic urine with biochemical analyses showing a dramatic increase in bilirubin (total bilirubin 22 mg/dL; direct bilirubin 21 mg/dL) and alkaline phosphatase (up to 4-fold over the normal level) with less marked increases in transaminases. Liver histology showed a pattern of cholestasis with evident signs of cholangiolitis and damage of the interlobular bile ducts. Serum transaminase... (truncated)

Title: Severe acute cholestatic hepatitis with prolonged cholestasis and bile-duct injury following atorvastatin therapy: a case report.
We report the case of a patient who exhibited severe acute hepatitis with symptomatic cholestasis for more than 3 months and bile duct injury following the prescription of atorvastatin. After withdrawal the drug, the patient's wellbeing slowly improves and biological features normalize in 4 months. Therapy aimed at treating severe liver steatosis and hypercholesterolemia. Atorvastatin is a highly effective 3-hydroxy-3 methylglutamyl- coenzyme A reductase (statin) used to lower low-density lipoprotein. Reported frequent adverse events of the medication include nausea, depression, myalgia, abdominal pain and abnormal liver function tests. Although abnormal liver function tests is not an uncommon side effect of... (truncated)

Title: Cholangiolytic Changes in Statin-Induced Liver Injury.
Atorvastatin is a commonly used oral cholesterol-lowering agent. Side effects associated with statin therapy include arthralgia, myalgia, dyspepsia, weakness, and headache. Prospective and retrospective studies of drug-induced liver injury have identified statin-induced hepatotoxicity, with atorvastatin being the most commonly cited. Associated liver function test elevations have varied from hepatocellular to cholestatic/mixed pattern. We report a case of a 58-year-old woman that illustrates unusual histologic findings associated with a mixed pattern of statin-induced liver injury. While being treated with atorvastatin, the patient exhibited repeated bouts of abdominal pain over a year associated with biliary tree dilation, variably attributed to postcholecystectomy dilation... (truncated)

Title: Recurrence and Severe Worsening of Hepatotoxicity After Reintroduction of Atorvastatin in Combination With Ezetimibe.
Severe hepatotoxicity is a rare but well-known adverse reaction to statins. However, despite the widespread use of statins, only a few cases describing statin reexposure or switch to another statin after liver injury have been published. The literature on hepatotoxicity with ezetimibe alone or in combination with statins is also scarce. We report a case where a patient with a history of elevated liver enzymes while using atorvastatin, but prior and subsequent good tolerance to simvastatin and pravastatin, developed drug-induced liver injury on reexposure to a combination of atorvastatin and ezetimibe. The hepatotoxicity in our patient was most likely caused... (truncated)

Title: High Dose Atorvastatin Associated with Increased Risk of Significant Hepatotoxicity in Comparison to Simvastatin in UK GPRD Cohort.
Occasional risk of serious liver dysfunction and autoimmune hepatitis during atorvastatin therapy has been reported. We compared the risk of hepatotoxicity in atorvastatin relative to simvastatin treatment.

MESH:D014810 - vitamin e

Summary:

LLM Prediction Score	0.052 (normalized)
LLM Confidence Score	0.990
Golden Answer (Severity Class)	0.0 (normalized)
Prediction Error	0.052

Retrieved Context:

Title: Effects of vitamin A and vitamin E on attenuation of titanium dioxide nanoparticles-induced toxicity in the liver of male Wistar rats.
The increasing application of titanium dioxide nanoparticles (NTiO₂) in life and the toxicity potential of these nanoparticles have raised concerns about their detrimental effects on human health. This study was conducted to investigate the hepatoprotective effects of vitamin E and vitamin A against hepatotoxicity induced by NTiO₂ in rats. Thirty-six male Wistar rats were randomly divided into six groups of six rats each. Intoxicated group received 300 mg/kg NTiO₂ for two weeks by gavage. Groups treated with vitamin E (100 IU/kg), vitamin A (100 IU/kg) and mixture of these vitamins were orally administered for 3 weeks (started 7 days before NTiO₂ administration). In order to... (truncated)

Title: Effects of dietary vitamin E on clinical course and plasma glutamic oxaloacetic transaminase and glutamic pyruvic transaminase activities in hereditary hepatitis of LEC rats.
Long-Evans Cinnamon (LEC) rats are autosomal recessive mutants that develop hepatitis and hepatocellular carcinoma. Because copper accumulates in the livers of these rats, and some of their clinical and pathological features are similar to those of patients with Wilson's disease, LEC rats are proposed as an animal model of Wilson's disease. It has been thought that unbound copper generates free radicals, which act as hemolytic and hepatocytotoxic agents. To examine the effects of vitamin E as an antioxidant on hereditary hepatitis in LEC rats, we fed 3-week-old rats for 25 weeks either vitamin E-deficient, control, or vitamin E-supplemented diets which... (truncated)

Title: Antioxidative stress effects of vitamins C, E, and B₁₂, and their combination can protect the liver against acetaminophen-induced hepatotoxicity in rats.
Label="BACKGROUND" NlmCategory="BACKGROUND">Several vitamins, including C, E, and B₁₂, have been recognized as antioxidants and have shown hepatoprotective effects against the hepatotoxicity caused by acetaminophen (APAP) overdose. The current investigation aims to study the effect of these vitamins and their combination in protecting the liver from APAP hepatotoxicity in rats.

Title: Inhibition of carbon tetrachloride-induced liver injury by liposomes containing vitamin E.
We tested a variety of antioxidants as possible therapeutic agents in an acute CCl4 mouse model of hepatotoxicity. Liver damage, gauged by the amount of serum aminotransferase released into the blood, morphological changes, lethal dose response, and presence of thiobarbituric acid-reactive substances (TBARS), were significantly inhibited in a dose-dependent manner by liposomes containing vitamin E (LVE) or by Rocavit E, a water-soluble emulsion of alpha-tocopherol. Serum aminotransferase levels in LVE- or Rocavit E-treated animals were always > 10-fold lower than levels in corresponding CCl4 controls. Other liposome-associated antioxidants, butylated hydroxytoluene, vitamin E succinate, catalase, desferoxamine, superoxide dismutase, and ascorbic acid... (truncated)

Title: Evidence for beneficial effects of vitamin E.
Oxidative stress and lipid peroxidation have been implicated in the pathogenesis of various diseases, including atherosclerosis and fatty liver diseases, and consequently the role of antioxidants in the prevention and treatment of such diseases has received much attention. In particular, the effects of vitamin E, the most important lipophilic radical-scavenging antioxidant, have been investigated extensively. Many in vitro, animal, and epidemiological studies have reported positive results, but large-scale randomized controlled intervention studies and meta-analyses have produced inconsistent and often disappointing results. In the present review article, the role and action of vitamin E are discussed, with consideration of the factors... (truncated)

MESH:D017576 - daptomycin

Summary:

LLM Prediction Score	0.676 (normalized)
LLM Confidence Score	0.990
Golden Answer (Severity Class)	0.625 (normalized)
Prediction Error	0.051

Retrieved Context:

Title: Daptomycin-Induced Acute Liver Failure: A Rare Case Report.
Acute liver failure (ALF) is characterized by severe liver injury, encephalopathy, and impaired coagulation/synthetic function. Drug-induced liver injury (DILI) can rarely, in a dose-dependent manner, lead to ALF. This article presents a rare case of daptomycin-induced acute liver failure in a patient with no prior liver disease. A 73-year-old male with multiple comorbidities including heart failure, diabetes, and chronic kidney disease received daptomycin treatment for diabetic left foot osteomyelitis. Five days after starting therapy, he developed weakness, jaundice, and drowsiness, leading to ICU admission. Physical examination and labs revealed hepatomegaly, elevated liver enzymes and abnormal ultrasound findings. Autoimmune and infectious... (truncated)

Title: Possible Hepatotoxicity Associated With Daptomycin: A Case Report and Literature Review.
To report a case of isolated daptomycin-induced acute liver injury without elevations in creatine kinase (CK) levels or kidney dysfunction.

Title: A case report and literature review of daptomycin-induced liver injury.
Daptomycin is a lipopeptide antimicrobial used to treat gram positive organisms including multi-drug resistant infections. It has been shown to occasionally cause abnormalities in liver function but more commonly is associated with elevations in serum creatinine phosphokinase (CK) (Hair and Keam, 2007) [1]. We describe a case where a patient being treated for methicillin-resistant <i>Staphylococcus aureus</i> (MRSA) bacteremia with daptomycin developed asymptomatic elevated transaminases without evidence of multiorgan failure, hyperbilirubinemia or elevation of CK levels. Other etiologies for liver injury were considered and ruled out, and after daptomycin was discontinued, the transaminases returned to normal levels. We also provide a... (truncated)

Title: Daptomycin-Rifampin-Induced Rhabdomyolysis, Acute Renal Failure, and Hepatic Injury: A Case Report and Literature Review.
Daptomycin is a canonical antibiotic used very commonly in practice for its bactericidal activity against Gram-positive bacteria, including vancomycin-resistant enterococci (VRE) and methicillin-resistant Staphylococcus aureus (MRSA) bacteremia, bone infections, skin and soft tissue infections, meningitis, urinary tract infections, and endocarditis. Although daptomycin in conventional doses is usually well tolerated, it is paramount to be aware of the possible adverse effects. Daptomycin is reported to cause an elevation in creatine kinase levels, although frank rhabdomyolysis is rare. An even more infrequent occurrence is the simultaneous development of acute kidney injury and drug-induced liver injury with rhabdomyolysis. Daptomycin and rifampin combination are... (truncated)

Title: Daptomycin-induced acute renal and hepatic toxicity without rhabdomyolysis.
To report a case of a patient who experienced acute renal and hepatic toxicity following administration of daptomycin and review previously published case reports of renal and hepatic dysfunction with daptomycin.

MESH:D015242 - ofloxacin

Summary:

LLM Prediction Score	0.324 (normalized)
LLM Confidence Score	0.990
Golden Answer (Severity Class)	0.375 (normalized)
Prediction Error	0.051

Retrieved Context:

Title: Ofloxacin in miliary tuberculosis.
We report one case of severe miliary tuberculosis with liver failure and respiratory insufficiency in a twenty-seven year old patient. We emphasize the presence of hepatic, occular and vestibular toxicities secondary to the treatment and the usefulness of ofloxacin with cycloserine given for nine months.

Title: Comparison of side effects of levofloxacin versus other fluoroquinolones.
The side-effect profile of levofloxacin was compared with that of other fluoroquinolones based on European and international data from approximately 130 million prescriptions. Levofloxacin was found to be very safe with a low rate of hepatic abnormalities (1/650,000). In contrast, 140 trovafloxacin-treated patients developed hepatic problems, 14 of which were severe, and 8 required transplantation. The main CNS problems associated with fluoroquinolones include dizziness, convulsions, psychosis, and insomnia. Levofloxacin, ofloxacin, and moxifloxacin reportedly have the lowest potential of inducing central nervous system (CNS) adverse events among the fluoroquinolones currently available. Cardiovascular problems were seen in 1/15 million levofloxacin prescriptions compared... (truncated)

Title: Safety of an ofloxacin-based antitubercular regimen for the treatment of tuberculosis in patients with underlying chronic liver disease: a preliminary report.
Hepatotoxicity is a major side-effect of antitubercular drugs (ATD). As these drugs are metabolized in the liver, there is a theoretical risk of increased hepatotoxicity in patients with underlying chronic liver disease (CLD). Ofloxacin has antitubercular activity and has exclusive renal clearance. The aim was to study the efficacy and safety of an ofloxacin-based antitubercular regimen for treating tuberculosis in patients with underlying CLD.

Title: Intravenous ofloxacin in severe infections.
The efficacy and tolerance of intravenous ofloxacin was studied in 70 patients suffering from soft tissue infections (n = 33), intra-abdominal abscesses (n = 14), septicaemia (n = 12), pneumonia (n = 9) and brucellosis (n = 2). The average daily dose was 6 mg/kg divided into two doses. Pathogens treated included Enterobacter cloacae (n = 14), Escherichia coli (n = 12), Staphylococcus aureus (n = 13), Pseudomonas aeruginosa (n = 11), Klebsiella pneumoniae (n = 10), Enterococcus faecalis (n = 8) and Streptococcus spp. (n = 5). Most patients had several underlying diseases. Most of the patients had received... (truncated)

Title: Efficacy and tolerance of oral ofloxacin in treating various infections.
66 patients were given daily doses of ofloxacin between 400 and 800 mg for 10 days to 6 months. They were suffering from exacerbation of chronic bronchitis (15), soft tissue phlegmon (11), complicated urinary tract infections (7), bronchopneumonia (7), chronic osteomyelitis in exacerbation (8), chronic prostatitis in exacerbation (5), lower urinary tract infections (3), chronic otitis media (3), acute otitis (3), acute bronchitis (1), lung abscess (2) or liver abscess (1). Pathogens included Pseudomonas aeruginosa (24), Haemophilus influenzae (16), Proteus mirabilis (6), Escherichia coli (6), Enterobacter cloacae (6), Providencia stuartii (2), Serratia marcescens (2), Citrobacter diversus (1), Salmonella enteritidis (1),... (truncated)

MESH:C419502 - alvimopan

Summary:

LLM Prediction Score	0.051 (normalized)
LLM Confidence Score	0.950
Golden Answer (Severity Class)	0.0 (normalized)
Prediction Error	0.051

Retrieved Context:

Title: Efficacy and Safety of Peripherally Acting μ-Opioid Receptor Antagonist (PAMORAs) for the Management of Patients With Opioid-Induced Constipation: A Systematic Review.
In treating chronic and acute pain, opioids are widely used. Although they do provide analgesia, their usage does come with adverse events (AEs). One of the most burdensome is opioid-induced bowel dysfunction, and more specifically opioid-induced constipation (OIC). The pathogenesis of these AEs is well known as the consequence of the action of opioids on m-receptors in the enteric nervous system. In recent years, medicines counteracting this specific action at the receptors have been registered for clinical use: the peripherally acting μ-opioid receptor antagonists (PAMORAs). The knowledge of their comparative efficacy and tolerability is very important for physicians and patients... (truncated)

Title: The Useage of Opioids and their Adverse Effects in Gastrointestinal Practice: A Review.
Opium is one of the oldest herbal medicines currently used as an analgesic, sedative and antidiarrheal treatment. The effects of opium are principally mediated by the μ-, κ- and δ-opioid receptors. Opioid substances consist of all natural and synthetic alkaloids that are derived from opium. Most of their effects on gastrointestinal motility and secretion result from suppression of neural activity. Inhibition of gastric emptying, increase in sphincter tone, changes in motor patterns, and blockage of peristalsis result from opioid use. Common adverse effects of opioid administration include sedation, dizziness, nausea, vomiting, constipation, dependency and tolerance, and respiratory depression. The most... (truncated)

Title: Improvement of Therapeutic Value of Quercetin with Chitosan Nanoparticle Delivery Systems and Potential Applications.
This paper reviews recent studies investigating chitosan nanoparticles as drug delivery systems for quercetin. The therapeutic properties of quercetin include antioxidant, antibacterial and anti-cancer potential, but its therapeutic value is limited by its hydrophobic nature, low bioavailability and fast metabolism. Quercetin may also act synergistically with other stronger drugs for specific disease states. The encapsulation of quercetin in nanoparticles may increase its therapeutic value. Chitosan nanoparticles are a popular candidate in preliminary research, but the complex nature of chitosan makes standardisation difficult. Recent studies have used in-vitro, and in-vivo experiments to study the delivery of quercetin alone or in combination... (truncated)

Title: Drug Interactions With New Synthetic Opioids.
Fentanyl, fentanyl analogs, and other new synthetic opioids (NSO) have burst onto the illegal drug market as new psychoactive substances (NPS). They are often sold as heroin to unsuspecting users and produce euphoria through their agonist action on μ- opioid receptors. Their high consumption, often combined with other substances, has led to multiple intoxications during recent years. In some countries, such as the United States, the consumption of opioids, whether for medical or recreational purposes, has become epidemic and is considered a public health problem. Fentanyl analogs are more potent than fentanyl which in turn is 50 times more potent... (truncated)

Title: Current Overview on Clinical Management of Chronic Constipation.
Constipation is one of the major gastrointestinal disorders diagnosed in clinical practice in Western countries. Almost 20% of population suffer from this disorder, which means constipation is a substantial utilization of healthcare. Pathophysiology of constipation is complex and multifactorial, where aspects like disturbance in colonic transit, genetic predisposition, lifestyle habits, psychological distress, and many others need to be taken into consideration. Diagnosis of constipation is troublesome and requires thorough accurate examination. A nonpharmacological approach, education of the patient about the importance of lifestyle changes like diet and sport activity state, are the first line of therapy. In case of ineffective... (truncated)

MESH:D014191 - tranylcypromine

Summary:

LLM Prediction Score	0.426 (normalized)
LLM Confidence Score	0.980
Golden Answer (Severity Class)	0.375 (normalized)
Prediction Error	0.051

Retrieved Context:

Title: Transdermal selegiline for the treatment of major depressive disorder.
Non-selective inhibition of monoamine oxidase (MAO) enzymes (ie, isoforms A and B) in the brain are associated with clinically significant antidepressant effects. In the US, the selegiline transdermal system (STS; EMSAM) is the first antidepressant transdermal delivery system to receive Food and Drug Administration (FDA) approved labeling for the treatment of major depressive disorder (MDD). Currently, the use of orally administered MAO inhibitor antidepressants (eg, phenelzine, tranylcypromine) is limited by the risk of tyramine-provoked events (eg, acute hypertension and headache, also known as the "cheese reaction") when combined with dietary tyramine. The selegiline transdermal system is the only MAOI available... (truncated)

Title: Drug-Induced Liver Injury during Antidepressant Treatment: Results of AMSP, a Drug Surveillance Program.
Drug-induced liver injury is a common cause of liver damage and the most frequent reason for withdrawal of a drug in the United States. The symptoms of drug-induced liver damage are extremely diverse, with some patients remaining asymptomatic.

Title: Comparison of in vitro bioactivation of flutamide and its cyano analogue: evidence for reductive activation by human NADPH:cytochrome P450 reductase.
Flutamide (FLU), a nonsteroidal antiandrogen drug widely used in the treatment of prostate cancer, has been associated with idiosyncratic hepatotoxicity in patients. It is proposed that bioactivation of FLU and subsequent binding of reactive metabolite(s) to cellular proteins play a causative role. A toxicogenomic study comparing FLU and its nitro to cyano analogue (CYA) showed that the nitroaromatic group of FLU enhanced cytotoxicity to hepatocytes, indicating that reduction of the nitroaromatic group may represent a potential route of FLU-induced hepatotoxicity [Coe et al. (2007) Chem. Res. Toxicol. 20, 1277-1290]. In the current study, we compared in vitro bioactivation of FLU... (truncated)

Title: Alcohol and medication interactions.
Many medications can interact with alcohol, thereby altering the metabolism or effects of alcohol and/or the medication. Some of these interactions can occur even at moderate drinking levels and result in adverse health effects for the drinker. Two types of alcohol-medication interactions exist: (1) pharmacokinetic interactions, in which alcohol interferes with the metabolism of the medication, and (2) pharmacodynamic interactions, in which alcohol enhances the effects of the medication, particularly in the central nervous system (e.g., sedation). Pharmacokinetic interactions generally occur in the liver, where both alcohol and many medications are metabolized, frequently by the same enzymes. Numerous classes of... (truncated)

Title: [The behavior of gamma-glutamyltranspeptidase and other liver enzymes in the plasma during alcohol withdrawal treatments].
90 chronic alcoholics (55 men and 35 women, aged between 20 and 60 years) were investigated to determine how alcohol withdrawal effects the pattern of enzymes in plasma and if changes in this enzyme pattern could be used as criteria for evaluation of the recovery process. Among the different enzymes tested, gamma-glutamyl-transpeptidase (GGTP) and the transamines seemed the most suitable parameters. At the beginning of the alcohol withdrawal course, 79 out of 90 patients (80%) showed elevated values of one of these enzymes in plasma. GOT was elevated in 31 (34%), GPT in 24 (23%) and GGTP in 79 (88%)... (truncated)

MESH:D009643 - norfloxacin

Summary:

LLM Prediction Score	0.426 (normalized)
LLM Confidence Score	0.970
Golden Answer (Severity Class)	0.375 (normalized)
Prediction Error	0.051

Retrieved Context:

Title: The safety profile of the fluoroquinolones.
Premarketing trials showed the fluoroquinolone agents to have a favorable side-effect profile, with treatment-related adverse events comprising gastrointestinal, central nervous system, and dermatologic effects that were generally mild and reversible on cessation of treatment. However, postmarketing surveillance studies have identified severe adverse events, including severe anaphylaxis, QTc-interval prolongation, and potential cardiotoxicity, associated with 3 quinolone agents that either resulted in the removal of the agent from the market (temafloxacin and grepafloxacin) or significantly restricted its use due to substantial mortality and morbidity associated with liver toxicity (trovafloxacin). To date, there have been no such significant adverse events associated with the... (truncated)

Title: Comparative tolerability of the newer fluoroquinolone antibacterials.
The most common adverse effects of the fluoroquinolones involve the gastrointestinal tract, skin and CNS, and are mainly mild and reversible. Of the gastrointestinal events, nausea and vomiting are the most common. Mild hepatic reactions are a class effect, usually presenting as mild transaminase level increases without clinical symptoms. However, postmarketing surveillance has revealed significant hepatotoxicity with trovafloxacin. It is not currently known whether the severe reactions to trovafloxacin are specific to that agent or simply represent an extreme of an emerging class effect. The enormous worldwide usage of, and extensive published adverse effect data on the other fluoroquinolones and... (truncated)

Title: Adverse reactions to fluoroquinolones in the Nigerian population: an audit of reports submitted to the National Pharmacovigilance Centre from 2004 to 2016.
Adverse drug reactions (ADRs) recorded in national pharmacovigilance databases in developed countries have been analyzed. However, adverse reactions to fluoroquinolones were observed globally despite their wide use and safety concerns. We provided information on the pattern of adverse reactions to fluoroquinolones reported spontaneously to the National Pharmacovigilance Centre (NPC), Nigeria. ADRs to fluoroquinolones reported to the NPC, over a period of 12 years, were analyzed. Evaluation was done for annual reports, age and gender of patients, type of reporter, suspected fluoroquinolones and adverse reactions, onset and outcome of ADRs, and causality. A total of 18527 ADR reports were received by... (truncated)

Title: Norfloxacin-induced acute cholestatic hepatitis in a patient with alcoholic liver cirrhosis.
No abstract available.

Title: Fluoroquinolone-related adverse events resulting in health service use and costs: A systematic review.
Adverse events (AEs) associated with the use of fluoroquinolone antimicrobials include Clostridium difficile associated diarrhea (CDAD), liver injury and seizures. Yet, the economic impact of these AEs is seldom acknowledged. The aim of this review was to identify health service use and subsequent costs associated with ciprofloxacin, levofloxacin, moxifloxacin, norfloxacin and ofloxacin -related AEs.

MESH:D001761 - bleomycin

Summary:

LLM Prediction Score	0.425 (normalized)
LLM Confidence Score	0.980
Golden Answer (Severity Class)	0.375 (normalized)
Prediction Error	0.050

Retrieved Context:

Title: [Hodgkin lymphoma with hepatic involvement treated with dexametasone, gemcitabine and cisplatin as a bridge to standard therapy: report of one case].
The initial presentation of Hodgkin lymphoma with liver involvement is rare. In these patients, the standard first-line therapy with ABVD (Adriamycin, Bleomycine, Vinblastine, Dacarbazine) imply an additional risk for liver toxicity. We report a 64-year-old woman who presented with jaundice, choluria, malaise and weight loss. In the initial evaluation she had jaundice and palpable groin lymph nodes. An obstructive biliary disease was ruled out with magnetic resonance imaging studies. A lymph node biopsy showed a Hodgkins lymphoma, Mixed-cellularity subtype. Considering the liver dysfunction, an alternative scheme of chemotherapy with dexamethasone, gemcitabine and cisplatin (GDP) was administered. After 4 cycles, a... (truncated)

Title: [Complications of antitumor and antileukemic chemotherapy. 1].
The recent development of chemotherapy in the treatment of cancer and leukemia requires that all practitioners involved have a thorough knowledge of the sometimes life-threatening side-effects of chemotherapeutic agents. All these agents, whether used alone or in a combination, carry a risk because of their lack of specificity which make active on normal cells, especially those with a rapid turn-over such as the hematopoietic cells or the cells of the digestive tract. Prior to the prescription of a chemotherapeutic regimen, the acceptable risk must always be clearly defined, according to the seriousness of the disease and to the patient's age,... (truncated)

Title: First reported case of disease: peliosis hepatis as cardinal symptom of Hodgkin's lymphoma.
We present the case of a 25-year-old woman with a history of weakness, weight loss, anemia, and elevated liver enzymes. Outpatient diagnostic evaluation, including abdominal ultrasound and endoscopies, revealed no conclusive explanation for the clinical picture and the patient was admitted to our clinic. Because of the hepatosplenomegaly together with the elevated liver enzymes, one of our differential diagnoses was that of liver disease. To clarify this, we performed a minilaparoscopy, which showed multiple diffuse distributed spots of livid color without clear margins distributed all over both liver lobes. A biopsy taken from these areas revealed the diagnosis of peliosis... (truncated)

Title: Liver damage in bleomycin-induced pulmonary fibrosis in mice.
Pulmonary fibrosis is an emerging disease with a poor prognosis and high mortality rate that is even surpassing some types of cancer. This disease has been linked to the concomitant appearance of liver cirrhosis. Bleomycin-induced pulmonary fibrosis is a widely used mouse model that mimics the histopathological and biochemical features of human systemic sclerosis, an autoimmune disease that is associated with inflammation and expressed in several corporal systems as fibrosis or other alterations. To determine the effects on proliferation, redox and inflammation protein expression markers were analyzed by immunohistochemistry. Analyses showed a significant increase in protein oxidation levels by lipoperoxidation... (truncated)

Title: Protective effect of spin-labeled 1-ethyl-1-nitrosourea against oxidative stress in liver induced by antitumor drugs and radiation.
This study was carried out to investigate possible protection effect of 1-ethyl-3-[4-(2,2,6,6-tetramethylpiperidine-1-oxyl)]-1-nitrosourea (SLENU), synthesized in our laboratory, against oxidative liver injuries induced in mice treated by antitumor drugs: doxorubicin (DOX), bleomycin (BLM), or gamma irradiation (R). Specifically, alterations in some biomarkers of oxidative stress, such as lipid peroxidation products measured as malondialdehyde (MDA) levels and activities of the antioxidant enzymes, superoxide dismutase (SOD) and catalase (CAT), were studied in liver homogenates isolated from tumor bearing C57 black mice after i.p. treatment with solutions of DOX (60 mg/kg), BLM (60 mg/kg), or after total body gamma-irradiation with a single dose of... (truncated)

MESH:D008353 - mannitol

Summary:

LLM Prediction Score	0.050 (normalized)
LLM Confidence Score	0.990
Golden Answer (Severity Class)	0.0 (normalized)
Prediction Error	0.050

Retrieved Context:

Title: Tumor necrosis factor induced oxidative stress in isolated mouse hepatocytes.
Tumor necrosis factor alpha (TNF alpha) is a macrophage-derived cytokine which participates in homeostatic tissue repair. It is also a potentially useful antitumor agent. Liver toxicity, however, limits TNF alpha's clinical utility and suggests that it may play a role in liver toxicity of various etiologies. To determine the direct effects of TNF alpha on hepatocytes, in the absence of infiltrating leukocytes and other inflammatory mediators, an isolated mouse hepatocyte model has been used in the present study. Hepatocytes exposed to recombinant human TNF alpha (1-10 micrograms/ml) exhibited intracellular GSH depletion and GSSG efflux during the first 2 hr of... (truncated)

Title: In vivo evidence for accelerated generation of hydroxyl radicals in liver of Long-Evans Cinnamon (LEC) rats with acute hepatitis.
The Long-Evans Cinnamon (LEC) rats accumulate excess copper (Cu) in the liver in a manner similar to patients with Wilson's disease (WD) and spontaneously develop acute hepatitis with severe jaundice. Although hydroxyl radicals (*OH) have been proposed to be a cause of hepatitis by the accumulation of Cu, it is not clear whether or not *OH can be produced in the liver of hepatitic LEC rats in vivo and also can be involved in the onset of hepatitis. In the present study, *OH production in plasma and liver of hepatitic LEC rats was quantified by trapping *OH with salicylic acid... (truncated)

Title: Nicardipine-induced acute hepatitis in an intensive care unit patient.
Drug-related hepatotoxicity is now the leading cause of acute liver failure in the United States, especially among patients who have no prior liver disease. Nicardipine is the only IV calcium channel blocker available for the short-term treatment of hypertension with a considerably good safety profile. We report a case of nicardipine-induced hepatitis. A patient with history of hypertension was admitted because of right middle cerebral artery infarction. Computed tomography of the brain showed evolving stroke. The patient went for cerebral angiography and stent placement, and during the procedure he had cerebral hemorrhage. He was transferred to neurosurgery. After surgery, he... (truncated)

Title: A 28-days sub-acute toxicity study in swiss albino mice to evaluate toxicity profile of neurotol plus (mannitol and glycerol combination).
Osmotic agents are still the most common treatment options available for controlling intracranial pressure (ICP). Combining Mannitol and Glycerol provides a better alternative and is currently available as best therapy used for increased ICP. The aim of this work was to study the effects of repeated dosing (28 days) of Mannitol 20% and Glycerol 10% combined formulation, Neurotol plus on safety profile. A twenty eight days sub-acute toxicity study was conducted at three different dose levels of 5ml/kg, 10 ml/kg and 20 ml/kg. Mice were randomly divided into four groups of six animals each. Physical parameters, biochemical parameters related to... (truncated)

Title: Amodiaquine-induced oxidative stress in a hepatocyte inflammation model.
Amodiaquine is an antimalarial drug that causes life-threatening agranulocytosis and hepatotoxicity in about 1 in 2000 patients, which is usually associated with an inflammatory response. It was found that the LC(50) (2h) of amodiaquine towards isolated rat hepatocytes was 1mM. The cytotoxic mechanism involved protein carbonylation as well as P450 activation to a reactive metabolite. The cytotoxicity, however, was not reactive oxygen species (ROS)-mediated, as ROS scavengers did not prevent cytotoxicity or protein carbonylation, and it was not accompanied by glutathione (GSH) oxidation or intracellular H(2)O(2) formation. On the other hand, the cytotoxicity could be attributed to a quinoneimine metabolite... (truncated)

MESH:D002998 - clonazepam

Summary:

LLM Prediction Score	0.424 (normalized)
LLM Confidence Score	0.990
Golden Answer (Severity Class)	0.375 (normalized)
Prediction Error	0.049

Retrieved Context:

Title: Clonazepam-induced liver dysfunction, severe hyperlipidaemia, and hyperglycaemic crisis: A case report.
In this study, we report a case of a 50-year-old Japanese man who had chronic whiplash-associated disorder, hyperlipidaemia, hyperuricacidaemia, and mild liver dysfunction due to excessive alcohol intake. Recently, he developed mild tremor in his left hand. Initiation of clonazepam (0.5 mg once daily before bedtime) helped to gradually ameliorate the tremor. However, 13 days after clonazepam initiation, his liver function and lipid profiles aggravated, and his postprandial glucose level increased to 400 mg/dL. Clonazepam was stopped promptly, and at 7 days after discontinuation, the abnormal triglyceride levels, liver dysfunction, and glycometabolism improved without any other medical intervention. This case... (truncated)

Title: Side effects of carbamazepine, valproate and clonazepam during long-term treatment of epilepsy.
Side effects of carbamazepine (CBZ), valproate (VPA) and clonazepam (CZP) are rare during long-term use but rather common and usually transient during the early phases of treatment. The usual side effects of CBZ are drowsiness, dizziness, and diplopia, which are dose dependent in long-term use, but CBZ does not seem to cause cognitive disturbances, as do phenobarbital and phenytoin. Other reactions to CBZ may include leukopenia, hyponatremia, disturbances of vitamin D metabolism and fortunately rarely, agranulocytosis and hepatitis. Use of VPA can lead to gastrointestinal discomfort, weight gain, hair loss, tremor and sedation, but these side effects are rather uncommon,... (truncated)

Title: [Pharmacotherapy of epilepsy--current problems and controversies].
Current trends and controversies in the antiepileptic drug therapy are reviewed from a clinical view. The usefulness of prophylactic therapy of febrile seizures and posttraumatic seizures or posttraumatic epilepsy is compromised by difficulties in the management of the patients especially by noncompliance. Previously untreated epilepsies can be treated successfully in 70--80% of the patients. Phenytoin or carbamazepine are equally effective for generalized tonic-clonic seizures of focal seizures, while absence seizures are controlled by ethosuximide or valproic acid. Only when the epilepsy is uncontrolled despite high plasma concentrations which cannot be raised because of side effects, a second drug should be... (truncated)

Title: Drug-Induced Liver Injury Caused by Kratom Use as an Alternative Pain Treatment Amid an Ongoing Opioid Epidemic.
Kratom (Mitragyna speciosa) is a prevalent medicinal plant used mainly for the stimulant and analgesic properties provided through multiple alkaloid compounds. Over the past decade, use of kratom has increased despite the limited knowledge of toxicities and adverse side effects. With the current opioid epidemic, both patients and providers are seeking alternative methods to treat both addiction and pain control, and kratom as an alternative means of treatment has increasingly entered the mainstream. In this article, we present the clinical course of a 47-year-old male who developed fatigue, pruritus, and abnormal liver tests (with a mixed hepatocellular/cholestatic pattern) approximately... (truncated)

Title: Acute intermittent porphyria and epilepsy: safety of clonazepam.
A young woman with acute intermittent porphyria (AIP) and juvenile myoclonic epilepsy began to have generalized tonic-clonic seizures (GTCs) at age 13. Subsequently, she had myoclonic seizures, which were often precipitated by visual stimulation, tended to occur in the morning, and sometimes evolved into GTCs. Valproate (VPA) resulted in a worsening of latent AIP, and treatment with a combination of phenytoin (PHT), carbamazepine (CBZ), and clonazepam (CZP) led to severe neuropathy of AIP and an electrolyte imbalance. These conditions were improved by water restriction, infusion of high doses of carbohydrates, and discontinuation of all antiepileptic drugs (AEDs) except for CZP.... (truncated)

MESH:D008691 - methadone

Summary:

LLM Prediction Score	0.424 (normalized)
LLM Confidence Score	0.990
Golden Answer (Severity Class)	0.375 (normalized)
Prediction Error	0.049

Retrieved Context:

Title: The spectrum and causes of liver diseases in narcotic addicts.
Fifty charts of patients discharged with a diagnosis of liver disease from the acute care service of a hospital for the treatment of addiction were analyzed. Among 42 active heroin users or former heroin addicts maintained on methadone, 79% were heavy alcohol abusers. Seventeen cases of cirrhosis were encountered. All occurred in those abusing alcohol. Reversible liver disease was seen in both alcohol abusers and nonalcoholics. This study suggests that alcohol is a major factor in the development of irreversible liver disease in active heroin addicts and ex-addicts on methadone maintenance.

Title: Correlates of depressive symptoms among alcohol-using methadone maintained adults.
Alcohol-using clients are considered at great risk for hepatitis and ongoing liver damage. This study explores the correlates of depression among a sample of methadone maintained treatment (MMT) adults in the Los Angeles area, and is part of a larger study on hepatitis health promotion among MMT clients who use alcohol.

Title: Methadone toxicity causing death in ten subjects starting on a methadone maintenance program.
Methadone maintenance therapy is designed to reduce the need for addicts to use heroin or other illegal opiates. Death in patients starting on such a program has not previously been documented. We report the death of 10 persons who died within days of starting a methadone maintenance program administered by general practitioners. Their bodies were subject to a full autopsy by forensic pathologists, with a full toxicological examination. The mean starting dose had been 53 mg, which had been increased to a mean of 57 mg by the final dose. Death occurred after a mean of 3 days. The mean... (truncated)

Title: The comparative toxicology and major organ pathology of fatal methadone and heroin toxicity cases.
In order to determine the comparative toxicology and systemic disease of cases of death due to methadone and heroin toxicity, 1193 coronial cases of opioid overdose that occurred in New South Wales, Australia between 1 January 1998 and 31 December 2007 were inspected. These comprised 193 cases in which cause of death involved methadone toxicity (METH) and 1000 cases in which cause of death involved heroin toxicity in the absence of methadone (HER). METH cases were significantly more likely to have benzodiazepines (63.7% vs. 32.2%), and less likely to have alcohol (23.6% vs. 42.7%) detected. METH cases were significantly more... (truncated)

Title: Buprenorphine/Naloxone and methadone effects on laboratory indices of liver health: a randomized trial.
Buprenorphine/naloxone (BUP) and methadone (MET) are efficacious treatments for opioid dependence, although concerns about a link between BUP and drug-induced hepatitis have been raised. This study compares the effects of BUP and MET on liver health in opioid-dependent participants.

MESH:D000077152 - olanzapine

Summary:

LLM Prediction Score	0.424 (normalized)
LLM Confidence Score	0.990
Golden Answer (Severity Class)	0.375 (normalized)
Prediction Error	0.049

Retrieved Context:

Title: Identifying olanzapine induced liver injury in the setting of acute hepatitis C: A case report.
Olanzapine is linked to asymptomatic, transient elevations of liver aminotransferases but is historically thought to rarely cause significant hepatotoxicity. Underlying liver disease is a risk factor for drug-induced liver injury and may complicate the differential diagnosis of acute transaminitis in patients taking medications associated with hepatotoxicity. Ms L presented with 2 months of new psychotic symptoms resulting in hospitalizations. Although psychosis previously improved with haloperidol, she reported symptoms concerning for akathisia. Restlessness improved and psychotic symptoms resolved after initiation of olanzapine. Concurrently, her alanine aminotransferase (ALT) was elevated, prompting further workup and new diagnosis of acute hepatitis C. Over the... (truncated)

Title: Olanzapine induced biochemical and histopathological changes after its chronic administration in rats.
<i>Objective:</i> Olanzapine is a second generation antipsychotic acting mainly as a dopamine D₂ and serotonine 5-HT₂ receptors antagonist prescribed in the treatment of schizophrenia and various other psychiatric illnesses. Even though olanzapine is widely used in psychiatry, its effects on the architecture of pancreas, liver and kidneys are little known. The histology of pancreas especially has never been studied. For these reasons, the current study was designed to elucidate the toxic effects of chronic administration of olanzapine on pancreas, liver and kidneys and the enzymes released by these tissues in an escalating dose manner. <i>Methods:</i> Fourteen male rats were divided... (truncated)

Title: [Hepatic tolerance of atypical antipsychotic drugs].
The strategy in the choice of antipsychotic agent must take into account the hepatic tolerance according to non-negligible incidence of liver disorders among psychiatric population (presence of risk factors like alcoholism, drugs of abuse intake, polymedication including potentially hepatotoxic drugs.). More than 1 000 drugs have been listed as being responsible of hepatic side effects; 16% of these agents were neuropsychiatric drugs. Antidepressive drugs (tricyclic agents or SSRI), mood stabilizing agents and neuroleptic drugs have been implicated in biological or/and clinical hepatotoxicity. For these reasons, some psychotropic agents have been withdrawn of the pharmaceutical market like alpidem or medifoxamine. Atrium*,... (truncated)

Title: Severe liver enzyme elevations after three years of olanzapine treatment: a case report and review of olanzapine associated hepatotoxicity.
Atypical antipsychotics commonly cause isolated asymptomatic increase in the aminotransferase levels. Among these atypical antipsychotics, mostly transient, asymptomatic increase in hepatic enzymes has been reported with olanzapine, however olanzapine rarely may induce a clinical and/or biological hepatic toxicity. The pathogenesis of olanzapine-associated hepatotoxicity is not well known and is mostly a transient phenomenon. However, substantial and lasting changes may occur and result in symptomatic hepatitis. In the following case report, we report on a 44-year-old female patient diagnosed as Bipolar Disorder Type I, whose liver enzyme levels increased ten fold of normal ranges during the third year of the olanzapine... (truncated)

Title: Hepatotoxicity, leucopenia and neutropenia associated with olanzapine therapy.
Hepatotoxicity, leukopenia and neutropenia are rarely associated with atypical antipsychotic drug therapy. The authors describe a case of a schizophrenic patient, who was treated twice with olanzapine, and who developed an increase of hepatic enzymes, jaundice, reduced WBC, neutropenia, and a high temperature. The patient was diagnosed as suffering from hepatocellular hepatitis, neutropenia and leucopenia. Neuroleptic treatment was discontinued immediately and the patient exhibited consequential clinical improvement. A review of the literature is included.

MESH:D008139 - loperamide

Summary:

LLM Prediction Score	0.048 (normalized)
LLM Confidence Score	0.990
Golden Answer (Severity Class)	0.0 (normalized)
Prediction Error	0.048

Retrieved Context:

Title: In Vivo Effects of Cagaita (Eugenia dysenterica, DC.) Leaf Extracts on Diarrhea Treatment.
Eugenia dysenterica is a plant typically found in the Cerrado biome and commonly used in popular medicine due to its pharmacological properties, which include antidiarrheal, skin healing, and antimicrobial activities. The effects of ethanolic extract, aqueous extract and infusion of E. dysenterica leaves on intestinal motility and antidiarrheal activity were evaluated using ricin oil-induced diarrhea in rats. At doses of 400 and 800 mg·Kg(-1), the ethanolic extract decreased intestinal motility while the other extracts showed no significant effects. Moreover, serum levels of chloride, magnesium, and phosphorus were also measured in rats. Histopathologic and enzymatic analyses were also performed to investigate... (truncated)

Title: <i>Cymbopogon citratus</i> (DC.) Stapf aqueous extract ameliorates loperamide-induced constipation in mice by promoting gastrointestinal motility and regulating the gut microbiota.
Slow transit constipation (STC) is the most common type of functional constipation. Drugs with good effects and few side effects are urgently needed form the treatment of STC. <i>Cymbopogon citratus</i> (DC.) Stapf (CC) is an important medicinal and edible spice plant. The wide range of biological activities suggested that CC may have laxative effects, but thus far, it has not been reported. In this study, the loperamide-induced STC mouse model was used to evaluate the laxative effect of the aqueous extract of CC (CCAE), and the laxative mechanism was systematically explored from the perspectives of the enteric nervous system (ENS),... (truncated)

Title: Pharmacological Aspects of Over-the-Counter Opioid Drugs Misuse.
Several over-the-counter (OTC) drugs are known to be misused. Among them are opioids such as codeine, dihydrocodeine, and loperamide. This work elucidates their pharmacology, interactions, safety profiles, and how pharmacology is being manipulated to misuse these common medications, with the aim to expand on the subject outlined by the authors focusing on abuse prevention and prevalence rates. The reviewed literature was identified in several online databases through searches conducted with phrases created by combining the international non-proprietary names of the drugs with terms related to drug misuse. The results show that OTC opioids are misused as an alternative for illicit... (truncated)

Title: Controlling anal incontinence in women by performing anal exercises with biofeedback or loperamide (CAPABLe) trial: Design and methods.
The goals of this trial are to determine the efficacy and safety of two treatments for women experiencing fecal incontinence. First, we aim to compare the use of loperamide to placebo and second, to compare the use of anal sphincter exercises with biofeedback to usual care. The primary outcome is the change from baseline in the St. Mark's (Vaizey) Score 24weeks after treatment initiation. As a Pelvic Floor Disorders Network (PFDN) trial, subjects are enrolling from eight PFDN clinical centers across the United States. A centralized data coordinating center supervises data collection and analysis. These two first-line treatments for fecal... (truncated)

Title: A Narrative Review of Real-World Data on the Safety of Nintedanib in Patients with Idiopathic Pulmonary Fibrosis.
Nintedanib is a tyrosine kinase inhibitor approved for the treatment of idiopathic pulmonary fibrosis (IPF) and other progressive fibrosing interstitial lung diseases. Placebo-controlled trials showed that the adverse event profile of nintedanib was characterised mainly by gastrointestinal events, particularly diarrhoea. We review the data from all published real-world studies of the safety of nintedanib in patients with IPF. These real-world data were consistent with the safety profile observed in clinical trials and described in the product label. The most common adverse events were diarrhoea, nausea and vomiting, but these infrequently led to permanent treatment discontinuation. Liver enzyme elevations were observed,... (truncated)

MESH:D008535 - megestrol

Summary:

LLM Prediction Score	0.077 (normalized)
LLM Confidence Score	0.990
Golden Answer (Severity Class)	0.125 (normalized)
Prediction Error	0.048

Retrieved Context:

Title: Megestrol acetate dispersible tablets with a 5-HT3 receptor antagonist and dexamethasone <i>vs.</i> 5-HT3 receptor antagonist plus dexamethasone, can better control chemotherapy-induced nausea and vomiting: a randomized controlled study.
A reasonable and effective control of chemotherapy-induced nausea and vomiting (CINV) plays an important role in the comprehensive treatment of cancer. Megestrol belongs to the 17α-hydroxyprogesterone derivative and is a highly effective synthetic progesterone. Recorded in the instructions may improve appetite and cachexia in patients with advanced tumors. In recent years, clinical practice and small sample studies have shown that megestrol combined with chemotherapy can improve CINV. This randomized controlled trial aimed to evaluate the clinical efficacy and safety of megestrol acetate combined with a 5-Hydroxytryptamine (5-HT3) receptor antagonist and dexamethasone in patients with CINV.

Title: Feasibility and Activity of Megestrol Acetate in Addition to Etoposide, Doxorubicin, Cisplatin, and Mitotane as First-Line Therapy in Patients with Metastatic/Unresectable Adrenocortical Carcinoma with Low Performance Status.
(1) Background: The standard first-line therapy for advanced adrenocortical carcinoma (ACC) is represented by EDP-M (etoposide, doxorubicin, cisplatin + mitotane). Progestins have shown cytotoxic activity both in vitro and in vivo on ACC; better EDP-M tolerability and efficacy have been hypnotized due to the association with progestins. (2) Methods: The feasibility and tolerability of EDP-M combined with oral megestrol acetate (EDP-MM) were tested in 24 patients (pts) affected by metastatic ACC with a low performance status (PS); the case group was compared with a 48 pts control group according to the propensity score. The secondary objectives were clinical benefit rate... (truncated)

Title: Levonorgestrel-releasing intrauterine device plus metformin, or megestrol acetate plus metformin for fertility-sparing treatment of atypical endometrial hyperplasia and early endometrial carcinoma: a prospective, randomized, blind-endpoint design trial protocol.
Endometrial adenocarcinoma (EC) is the fifth most common cancer in women worldwide, standard treatment for EC includes hysterectomy, but it results in the loss of reproductive function. Thus, conservative treatment for these patients is strongly demanded, progestin therapy is widely accepted as the main fertility-sparing treatment for young women with endometrial hyperplasia with atypia (EHA) and well-differentiated endometrioid endometrial cancer. This trial will investigate the effectiveness of conservative treatment for obese women with early-stage EC.

Title: A randomized trial of megestrol acetate with or without premarin in the treatment of potentially responsive metastatic breast cancer. A Study of the Eastern Cooperative Oncology Group (E2185).
Human breast cancer cells in vitro exhibit increased levels of progestin receptors (PgR) after brief exposure to physiologic concentrations of estrogens. Prior clinical studies have positively correlated the responsiveness of metastatic breast cancer to progestin therapy with the level of PgR in the tumor cells.

Title: Sex Hormone-Dependent Physiology and Diseases of Liver.
Sexual dimorphism is associated not only with somatic and behavioral differences between men and women, but also with physiological differences reflected in organ metabolism. Genes regulated by sex hormones differ in expression in various tissues, which is especially important in the case of liver metabolism, with the liver being a target organ for sex hormones as its cells express estrogen receptors (ERs: ERα, also known as ESR1 or NR3A; ERβ; GPER (G protein-coupled ER, also known as GPR 30)) and the androgen receptor (AR) in both men and women. Differences in sex hormone levels and sex hormone-specific gene expression are... (truncated)

MESH:D000077235 - vinorelbine

Summary:

LLM Prediction Score	0.327 (normalized)
LLM Confidence Score	0.980
Golden Answer (Severity Class)	0.375 (normalized)
Prediction Error	0.048

Retrieved Context:

Title: [Combination of cisplatin and vinorelbine as adjuvant chemotherapy in non-small cell lung cancer patients--feasibility assessment of 5 consecutively treated patients].
Post-operative adjuvant chemotherapy(POAC)combining cisplatin(CDDP)and vinorelbine(VNR)for non-small cell lung cancer(NSCLC)patients is considered as a standard regimen. However, some Japanese investigators point out the toxic profile of this regimen in the practice settings. Thus, five consecutive patients with mean age of 61.6 years old were treated to evaluate the feasibility of this regimen among Japanese patients. Three male and 2 female patients were enrolled, with post-operative stage of IIB/IIIA/IIIB in 1/2/2 patients, respectively. CDDP was administered on day 1 at 80 mg/m(2), and VNR on days 1 and 8 at 25 mg/m(2) intravenously, every 21 days. The regimen was aimed to complete... (truncated)

Title: Safety and efficacy of vinorelbine in the treatment of non-small cell lung cancer.
Lung cancer remains the most frequently diagnosed cancer in the United States, excluding non-melanoma skin cancer. Non-small cell lung cancer (NSCLC) constitutes the majority (more than 80%) of lung cancer diagnoses. Systemic therapy, with either cytotoxic chemotherapy and/or targeted therapies, has been established to provide benefit to patients with NSCLC in both the adjuvant and advanced disease settings. Vinorelbine, a semi-synthetic vinca-alkaloid has been extensively tested alone and in combination with other cytotoxic or targeted agents in the treatment of NSCLC. Its safety has been well established with neutropenia, anemia, nausea, and vomiting being the most frequently encountered toxicities. The... (truncated)

Title: Vinorelbine in Non-Small Cell Lung Cancer: Real-World Data From a Single-Institution Experience.
The use of vinorelbine as a single agent or in combination regimens in non-small cell lung cancer (NSCLC) is associated with satisfactory clinical activity. However, the role of vinorelbine-based chemotherapy in chemonaive locally advanced unresectable or metastatic NSCLC patients, according to real-world treatment patterns, has still not been widely explored. Eighty-one patients treated at a single institution were retrospectively analyzed. Thirty-seven received standard first-line single-agent vinorelbine, and 44 received vinorelbine plus platinum drugs, based on physician's choice; 61.7% were older than 70 years, and 60.5% were affected by ≥2 comorbidities. Sixty-three patients were evaluable for objective response: 22% achieved partial... (truncated)

Title: A Pilot Study of Vinorelbine Safety and Pharmacokinetics in Patients with Varying Degrees of Liver Dysfunction.
Vinorelbine has demonstrated anticancer activity and is primarily metabolized in the liver. This single-institution, phase I pilot study describes the safety and pharmacokinetics of vinorelbine in patients with varying degrees of hepatic impairment.

Title: Thoracic radiotherapy and daily vinorelbine as radiosensitizer in locally advanced non small cell lung cancer: a phase I study.
Experimental studies have shown that vinorelbine is a powerful radiosensitizer in vitro. To date, no reports on clinical activity of the single agent vinorelbine as radiosensitizer have been published. The aim of the present phase I study was to determine the maximum tolerated dose (MTD) of vinorelbine administered daily concurrently with thoracic radiotherapy, with or without amifostine support, in the treatment of locally advanced non small cell lung cancer. In vitro studies have shown that vinorelbine can potentiate the antitumor effects of radiation therapy. Amifostine is a sulphydril compound that has shown to protect normal tissues from chemotherapy and radiotherapy-induced... (truncated)

MESH:D005480 - flurbiprofen

Summary:

LLM Prediction Score	0.421 (normalized)
LLM Confidence Score	0.950
Golden Answer (Severity Class)	0.375 (normalized)
Prediction Error	0.046

Retrieved Context:

Title: Prolonged Cholestatic Jaundice Associated With Flurbiprofen.
Nonsteroidal anti-inflammatory drugs (NSAIDs) are widely consumed drugs throughout the world for pain relief. Although the adverse effects of NSAIDs to the liver are well known, flurbiprofen-induced liver cholestasis is extremely rare. Herein, we present a patient with prolonged icterus that is associated with the use of flurbiprofen without causing ductopenia.

Title: Nonsteroidal anti-inflammatory drug-induced liver injury: a case-control study in primary care.
Several nonsteroidal anti-inflammatory drugs (NSAIDs) have been withdrawn from the market because of hepatic adverse drug reactions (ADRs). Moreover, some cases of liver diseases have been reported in patients taking NSAIDs (arylcarboxylic NSAIDs, piroxicam, sulindac, nimesulide, etc.). Pharmacoepidemiological studies have shown a risk of hepatic ADRs with NSAIDs used in association with other hepatotoxic drugs. In contrast, other studies performed in hospitalized patients did not found any association. The aim of this study was to assess the hepatic risk associated with the use of NSAID in the setting of primary care. The study design was a case-control study where cases... (truncated)

Title: A correlation between the in vitro drug toxicity of drugs to cell lines that express human P450s and their propensity to cause liver injury in humans.
Drug toxicity to T-antigen-immortalized human liver epithelial (THLE) cells stably transfected with plasmid vectors that encoded human cytochrome P450s 1A2, 2C9, 2C19, 2D6, or 3A4, or an empty plasmid vector (THLE-Null), was investigated. An automated screening platform, which included 1% dimethyl sulfoxide (DMSO) vehicle, 2.7% bovine serum in the culture medium, and assessed 3-(4,5-dimethylthiazol-2-yl)-5-(3-carboxymethoxyphenyl)-2-(4-sulfophenyl)-2H-tetrazolium reduction, was used to evaluate the cytotoxicity of 103 drugs after 24h. Twenty-two drugs caused cytotoxicity to THLE-Null cells, with $EC_{50} \leq 200$ μM; 21 of these drugs (95%) have been reported to cause human liver injury. Eleven drugs exhibited lower EC_{50} values in cells transfected... (truncated)

Title: Risk of acute and serious liver injury associated to nimesulide and other NSAIDs: data from drug-induced liver injury case-control study in Italy.
Drug-induced liver injury is one of the most serious adverse drug reactions and the most frequent reason for restriction of indications or withdrawal of drugs. Some nonsteroidal anti-inflammatory drugs (NSAIDs) were withdrawn from the market because of serious hepatotoxicity. We estimated the risk of acute and serious liver injury associated with the use of nimesulide and other NSAIDs, with a prevalence of use greater than or equal to 5%.

Title: A Systematic Review of Flurbiprofen 8.75 mg Dose and Risk of Haemorrhagic Events.
Oral non-steroidal anti-inflammatory drugs (NSAIDs) are known to be associated with an increased risk of bleeding. The NSAID, flurbiprofen, in the form of 8.75 mg lozenge or oromucosal spray is indicated for the symptomatic relief of sore throat. Despite the low dose as compared to alternative flurbiprofen preparations, concerns have been raised regarding its safety in terms of haemorrhagic events. This systematic review was conducted to identify existing evidence on the risk of haemorrhagic events with flurbiprofen 8.75 mg dose (any formulation), particularly where this may be due to potential interactions with other medicinal products. The systematic review examined studies... (truncated)

MESH:C073590 - gadolinium ethoxybenzyl dtpa

Summary:

LLM Prediction Score	0.045 (normalized)
LLM Confidence Score	0.800
Golden Answer (Severity Class)	0.0 (normalized)
Prediction Error	0.045

Retrieved Context:

Title: Diagnosis of Hepatocellular Carcinoma Using Gd-EOB-DTPA MR Imaging.
Gadolinium ethoxybenzyl diethylenetriamine pentaacetic acid (Gd-EOB-DTPA; Gadoxetic acid; Gadoxetate disodium) is a hepatocyte-specific MR contrast agent. It acts as an extracellular contrast agent in the early phase after intravenous injection, and then is taken up by hepatocytes later. Using this contrast agent, we can evaluate the hemodynamics of the liver and liver tumors, and can therefore improve the detection and characterization of hepatocellular carcinoma (HCC). Gd-EOB-DTPA helps in the more accurate detection of hypervascular HCC than by other agents. In addition, Gd-EOB-DTPA can detect hypovascular HCC, which is an early stage of the multi-stage carcinogenesis, with a low signal in... (truncated)

Title: Gadolinium ethoxybenzyl diethylenetriamine pentaacetic acid-enhanced MR finding of radiation-induced hepatic injury: relationship to absorbed dose and time course after irradiation.
To evaluate if Gd-EOB-DTPA-enhanced MRI could identify liver tissue damage caused by radiation exposure in patients undergoing external beam radiation therapy.

Title: Gadolinium Ethoxybenzyl Diethylenetriamine Pentaacetic Acid (Gd-EOB-DTPA)-Enhanced Magnetic Resonance Imaging and Multidetector-Row Computed Tomography for the Diagnosis of Hepatocellular Carcinoma: A Systematic Review and Meta-analysis.
The purpose of this meta-analysis was to compare the diagnostic accuracy of gadolinium ethoxybenzyl diethylenetriamine pentaacetic acid (Gd-EOB-DTPA)-enhanced magnetic resonance imaging (MRI) and multidetector-row computed tomography (MDCT) for hepatocellular carcinoma (HCC).Medline, Cochrane, EMBASE, and Google Scholar databases were searched until July 4, 2014, using combinations of the following terms: gadoxetic acid disodium, Gd-EOB-DTPA, multidetector CT, contrast-enhanced computed tomography, and magnetic resonance imaging. Inclusion criteria were as follows: confirmed diagnosis of primary HCC by histopathological examination of a biopsy specimen; comparative study of MRI using Gd-EOB-DTPA and MDCT for diagnosis of HCC; and studies that provided quantitative outcome data. The pooled... (truncated)

Title: Quantitative evaluation of liver function with MRI Using Gd-EOB-DTPA.
Gadolinium ethoxybenzyl diethylenetriaminepentaacetic acid (Gd-EOB-DTPA) is a newly developed MR contrast agent. After intravenous injection, Gd-EOB-DTPA is gradually taken up by the hepatocytes and eventually excreted via the biliary pathway without any change to its chemical structure. Because of these characteristics, it can be used as a tracer for quantitative liver function testing. The purpose of this study is to develop a noninvasive method of quantitation of the hepatic function using Gd-EOB-DTPA through the deconvolution analysis.

Title: Focal hepatic lesions in Gd-EOB-DTPA enhanced MRI: the atlas.
This article reviews the different technical aspects and pitfalls of gadolinium (Gd)-ethoxibenzyl (EOB)-diethylenetriamine pentaacetic acid (DTPA) and the advantages of the hepatocellular phase (HCP) and defines its specific imaging features of liver lesions.

MESH:D013747 - tetrabenazine

Summary:

LLM Prediction Score	0.419 (normalized)
LLM Confidence Score	0.980
Golden Answer (Severity Class)	0.375 (normalized)
Prediction Error	0.044

Retrieved Context:

Title: The Importance of Patient-Specific Factors for Hepatic Drug Response and Toxicity.
Responses to drugs and pharmacological treatments differ considerably between individuals. Importantly, only 50%-75% of patients have been shown to react adequately to pharmacological interventions, whereas the others experience either a lack of efficacy or suffer from adverse events. The liver is of central importance in the metabolism of most drugs. Because of this exposed status, hepatotoxicity is amongst the most common adverse drug reactions and hepatic liabilities are the most prevalent reason for the termination of development programs of novel drug candidates. In recent years, more and more factors were unveiled that shape hepatic drug responses and thus underlie the... (truncated)

Title: Treatment of huntington disease.
Many pharmacological agents have been utilized in the treatment of Huntington disease (HD). Several excellent reviews about the treatment of HD are available. Formal treatment guidelines are however lacking. This is mainly the result of limited evidence available in the literature. Further, available treatment studies are frequently hard to compare due to variable outcomes/instruments used, differences in the study population, and confounding effects of complex medication regimens. Generally speaking, the treatment paradigm for an HD patient will depend on the constellation of 3 main clinical domains affected in HD: motor, behavioral/psychiatric, and cognitive. Symptoms within each of these domains remain... (truncated)

Title: Twelfth-Position Deuteration of Nevirapine Reduces 12-Hydroxy-Nevirapine Formation and Nevirapine-Induced Hepatocyte Death.
Cytochrome P450-dependent metabolism of the anti-HIV drug nevirapine (NVP) to 12-hydroxy-NVP (12-OHNVP) has been implicated in NVP toxicities. We investigated the impact of twelfth-position trideuteration (12-D₃NVP) on the hepatic metabolism of and response to NVP. Formation of 12-OHNVP decreased in human (10.6-fold) and mouse (4.6-fold) hepatocytes incubated with 10 μM 12-D₃NVP vs NVP. An observed kinetic isotope effect of 10.1 was measured in human liver microsomes. During mouse hepatocyte treatment (400 μM) with NVP or 12-D₃NVP, cell death was reduced 30% with 12-D₃NVP vs NVP, while glucuronidated and glutathione-conjugated metabolites increased with 12-D₃NVP vs NVP. Using mass spectrometry proteomics, changes... (truncated)

Title: State of the Art and Uses for the Biopharmaceutics Drug Disposition Classification System (BDDCS): New Additions, Revisions, and Citation References.
The Biopharmaceutics Drug Disposition Classification system (BDDCS) is a four-class approach based on water solubility and extent of metabolism/permeability rate. Based on the BDDCS class to which a drug is assigned, it is possible to predict the role of metabolic enzymes and transporters on the drug disposition of a new molecular entity (NME) prior to its administration to animals or humans. Here, we report a total of 1475 drugs and active metabolites to which the BDDCS is applied. Of these, 379 are new entries, and 1096 are revisions of former classification studies with the addition of references for the approved... (truncated)

Title: Adverse drug reactions monitoring: prospects and impending challenges for pharmacovigilance.
Pharmacovigilance plays a consequential role in the surveillance of adverse drug reactions, which is provoked by the drugs used to cure diseases. Adverse drug reactions (ADRs) produce detrimental or undesirable effects to the body after administration of drugs. It has been reported that the number of patients dying because of contrary effects of drugs per year increased upto 2.6-fold. Moreover, rates of hospitalization of patients are increasing owing to adverse effects of drugs. Thus, it becomes challengeable for physician, health care providers, WHO and pharmaceutical industries to resolve the associated problem of ADRs. During the clinical trial of a novel... (truncated)

MESH:D010479 - pergolide

Summary:

LLM Prediction Score	0.419 (normalized)
LLM Confidence Score	0.970
Golden Answer (Severity Class)	0.375 (normalized)
Prediction Error	0.044

Retrieved Context:

Title: Tolcapone: new drug. In Parkinson's disease: unacceptable risk of severe hepatitis.
(1) When patients with Parkinson's disease who are taking levodopa develop motor fluctuations that do not respond to dose adjustments, the standard treatment is the addition of bromocriptine, a dopaminergic agonist. Evaluation of entacapone fails to show whether the risk-benefit balance of this catechol-O-methyltransferase (COMT) inhibitor is at least as favourable as that of bromocriptine. (2) Tolcapone, another COMT inhibitor, is back on the French market after being withdrawn because of serious hepatic effects. The summary of product characteristics (SPC) specifies that tolcapone must only be used when entacapone treatment fails or is poorly tolerated. (3) Renewal of marketing authorisation... (truncated)

Title: Successful drug development despite adverse preclinical findings part 2: examples.
To illustrate the process of addressing adverse preclinical findings (APFs) as outlined in the first part of this review, a number of cases with unexpected APF in toxicity studies with drug candidates is discussed in this second part. The emphasis is on risk characterization, especially regarding the mode of action (MoA), and risk evaluation regarding relevance for man. While severe APFs such as retinal toxicity may turn out to be of little human relevance, minor findings particularly in early toxicity studies, such as vasculitis, may later pose a real problem. Rodents are imperfect models for endocrine APFs, non-rodents for human... (truncated)

Title: Liver-Heart on chip models for drug safety.
Current pre-clinical models to evaluate drug safety during the drug development process (DDP) mainly rely on traditional two-dimensional cell cultures, considered too simplistic and often ineffective, or animal experimentations, which are costly, time-consuming, and not truly representative of human responses. Their clinical translation thus remains limited, eventually causing attrition and leading to high rates of failure during clinical trials. These drawbacks can be overcome by the recently developed Organs-on-Chip (OoC) technology. OoC are sophisticated <i>in vitro</i> systems capable of recapitulating pivotal architecture and functionalities of human organs. OoC are receiving increasing attention from the stakeholders of the DDP, particularly concerning... (truncated)

Title: Effect of trichloroacetaldehyde on the activation of CD4⁺T cells in occupational medicamentosa-like dermatitis: An in vivo and in vitro study.
Occupational medicamentosa-like dermatitis induced by trichloroethylene (OMLDT) is a hypersensitivity disease with autoimmune liver injury, which has increasingly become a serious occupational health problem in China. However, the pathogenesis of OMLDT remained undefined. In this study, 30 TCE-induced OMLDT patients, 58 exposure controls, and 40 non-exposure controls were recruited. We showed that the ratio of activated CD4⁺ T cells (downregulation of CD62 L) was dramatically increased in OMLDT patients compared to exposure and non-exposure control, suggesting that CD4⁺ T cells activation was a key cellular event in the development of OMLDT. In parallel, the expression of cytokine including IL-2, IFN-γ, TNF-α... (truncated)

Title: Proceedings of the 2011 National Toxicology Program Satellite Symposium.
The 2011 annual National Toxicology Program (NTP) Satellite Symposium, entitled "Pathology Potpourri," was held in Denver, Colorado in advance of the Society of Toxicologic Pathology's 30th Annual Meeting. The goal of the NTP Symposium is to present current diagnostic pathology or nomenclature issues to the toxicologic pathology community. This article presents summaries of the speakers' presentations, including diagnostic or nomenclature issues that were presented, along with select images that were used for audience voting or discussion. Some lesions and topics covered during the symposium include: proliferative lesions from various fish species including ameloblastoma, gas gland hyperplasia, nodular regenerative hepatocellular hyperplasia,... (truncated)

MESH:C488777 - romiplostim

Summary:

LLM Prediction Score	0.043 (normalized)
LLM Confidence Score	0.970
Golden Answer (Severity Class)	0.0 (normalized)
Prediction Error	0.043

Retrieved Context:

Title: A Review of Romiplostim Mechanism of Action and Clinical Applicability.
Thrombocytopenia results from a variety of conditions, including radiation, chemotherapy, autoimmune disease, bone marrow disorders, pathologic conditions associated with surgical procedures, hematopoietic stem cell transplant (HSCT), and hematologic disorders associated with severe aplastic anemia. Immune thrombocytopenia (ITP) is caused by immune reactions that accelerate destruction and reduce production of platelets. Thrombopoietin (TPO) is a critical component of platelet production pathways, and TPO receptor agonists (TPO-RAs) are important for the management of ITP by increasing platelet production and reducing the need for other treatments. Romiplostim is a TPO-RA approved for use in patients with ITP in the United States, European Union,... (truncated)

Title: Thrombopoietin Receptor Agonists (TPO-RAs): Drug Class Considerations for Pharmacists.
The thrombopoietin receptor agonists (TPO-RAs) romiplostim, eltrombopag, avatrombopag, and lusutrombopag carry unique US Food and Drug Administration (US FDA)- and European Medicines Agency (EMA)-approved indications and may be used to increase platelet counts in a variety of conditions. Current indications for available TPO-RAs include treatment of chronic immune thrombocytopenia (ITP) in cases of insufficient response to prior treatment (avatrombopag, eltrombopag, romiplostim), management of thrombocytopenia in adult patients with chronic liver disease who are scheduled to undergo a procedure (avatrombopag, lusutrombopag), management of severe aplastic anemia (eltrombopag), and management of thrombocytopenia associated with interferon-based therapy for hepatitis C (eltrombopag). Across current... (truncated)

Title: Adults with immune thrombocytopenia who switched to avatrombopag following prior treatment with eltrombopag or romiplostim: A multicentre US study.
Patients with immune thrombocytopenia (ITP) may respond to one thrombopoietin receptor agonist (TPO-RA) but not another. Limited data are available describing outcomes in patients who switched from romiplostim or eltrombopag to avatrombopag, a newer oral TPO-RA. We performed a retrospective observational study of adults with ITP who switched from eltrombopag or romiplostim to avatrombopag at four US tertiary ITP referral centres. Forty-four patients were included, with a mean ITP duration of 8.3 years and a median (range) of four prior ITP treatments. On avatrombopag, 41/44 patients (93%) achieved a platelet response (≥50 × 10⁹ /l) and 38/44 patients (86%) achieved a complete response... (truncated)

Title: Efficacy and safety of romiplostim in refractory aplastic anaemia: a Phase II/III, multicentre, open-label study.
A previous dose-finding study has suggested that romiplostim is effective in patients with refractory aplastic anaemia (AA) and 10 µg/kg once weekly was recommended as a starting dose. In this Phase II/III, multicentre, open-label study, romiplostim was administered subcutaneously at a fixed dose of 10 µg/kg once weekly for 4 weeks (weeks 1-4) followed by weekly doses (5, 10, 15 and 20 µg/kg) titrated by platelet response for up to 52 weeks (weeks 5-52). A total of 31 patients with AA who were refractory to immunosuppressive therapy (IST) and thrombocytopenia (platelet count of ≤30 × 10⁹ /l) were enrolled. The primary efficacy endpoint of the proportion of... (truncated)

Title: Effectiveness and Safety of Romiplostim Among Patients with Newly Diagnosed, Persistent and Chronic Immune Thrombocytopenia in European Clinical Practice.
Romiplostim has been approved in Europe since 2009 to treat patients with chronic primary immune thrombocytopenia (ITP). Using real-world data from seven European countries, we measured the effectiveness and safety outcomes within 24 weeks following romiplostim initiation by duration of ITP: less than 3 months ("newly diagnosed"), 3-12 months ("persistent"), and more than 12 months ("chronic").

MESH:D053139 - oseltamivir

Summary:

LLM Prediction Score	0.335 (normalized)
LLM Confidence Score	0.990
Golden Answer (Severity Class)	0.375 (normalized)
Prediction Error	0.040

Retrieved Context:

Title: Oseltamivir and acute hepatitis, reality association or coincidence?
Oseltamivir is an orally administered antiviral medication that selectively inhibits the influenza neuraminidase enzymes that are essential for viral replication and it is active against both influenza A and B viruses. Oseltamivir is indicated for therapy or post-exposure prevention of influenza A and B. Side effects are uncommon and include mild nausea, gastrointestinal upset, dizziness, and headache. Despite widespread use, oseltamivir has not been associated with clinically apparent liver injury; however, there is growing evidence of possible toxic liver involvement during oseltamivir therapy. To the best of our knowledge, this is the first reported case in Italy linking the development... (truncated)

Title: First case of acute cholestatic hepatitis attributed to oseltamivir in a young girl with ah1n1 influenza.
Oseltamivir caryboxylase is a potent inhibitor of the enzyme neuramidase of the influenza virus particle and it is active against both influenza A and B viruses. Oseltamivir is indicated for therapy or post-exposure prevention of influenza A and B. Side effects are uncommon and include mild nausea, gastrointestinal upset, dizziness and headache. Despite its widespread use, oseltamivir has not been associated with clinically apparent liver injury. To the best of our knowledge, this is the first case report in the literature linking the development of acute hepatitis to the consumption of oseltamivir in a patient suffering from influenza H1N1 infection.

Title: Adverse Effects of Oseltamivir Phosphate Therapy on the Liver of LDLR-/- Mice Without Any Benefit on Atherosclerosis and Thrombosis.
Desialylation, governed by sialidases or neuraminidases, is strongly implicated in a wide range of human disorders, and accumulative data show that inhibition of neuraminidases, such as neuraminidases 1 sialidase, may be useful for managing atherosclerosis. Several studies have reported promising effects of oseltamivir phosphate, a widely used anti-influenza sialidase inhibitor, on human cancer cells, inflammation, and insulin resistance. In this study, we evaluated the effects of oseltamivir phosphate on atherosclerosis and thrombosis and potential liver toxicity in LDLR-/- mice fed with high-fat diet. Our results showed that oseltamivir phosphate significantly decreased plasma levels of LDL cholesterol and elastin fragmentation in... (truncated)

Title: Alteration of liver function due to H1N1 infection: a case report.
H1N1 virus is known to affect the respiratory tract. The majority of healthcare providers focus on the respiratory complications attributed to H1N1 infection, overlooking possible multi-organ involvement. We present a rare case of abnormal liver function in a child who was admitted for respiratory illness due to the H1N1 virus. There was a marked elevation in liver function tests concurrent with the respiratory disease. Our patient was treated with oseltamavir for the H1N1 infection, and the liver function levels decreased dramatically in 72 hours.

Title: Assessment of adverse events related to anti-influenza neuraminidase inhibitors using the FDA adverse event reporting system and online patient reviews.
The recommended antiviral drugs available for the treatment and prevention of influenza are neuraminidase inhibitors (NAIs). The aim of this study was to evaluate age-related clinical manifestations of adverse events (AEs) related to NAIs. FAERS and WebMD data were downloaded. The available NAIs selected for the analysis were oseltamivir, peramivir, zanamivir, and laninamivir. Disproportionality was analyzed using the proportional reporting ratio (PRR), the reporting odds ratio (ROR), and the information component (IC) methods. In total, 16729 AEs from 4598 patients and 575 AEs from 440 patients in the FAERS and WebMD, respectively, were included in the analysis. In the FAERS,... (truncated)

MESH:C085665 - zaleplon

Summary:

LLM Prediction Score	0.040 (normalized)
LLM Confidence Score	0.980
Golden Answer (Severity Class)	0.0 (normalized)
Prediction Error	0.040

Retrieved Context:

Title: Hulk-Like Urine: A Case of Green Urine Caused by Flupirtine Intoxication.
Acute intoxications are common causes of admission to the Emergency Department (ED). Flupirtine is a non-opioid analgesic, originally used for acute and chronic pain. Because of several reports of severe liver toxicity, its use was limited to acute pain in 2013 by the European Medicines Agency. Although withdrawn from the European market in March 2018, there are still flupirtine tablets in many households, and most people are unaware of the hazards they might be facing. A 58-year-old man was admitted to the ED after a suicide attempt with 1 g of flupirtine. He was lethargic and confused but presented no... (truncated)

Title: Narcolepsy: current treatment options and future approaches.
The management of narcolepsy is presently at a turning point. Three main avenues are considered in this review: 1) Two tendencies characterize the conventional treatment of narcolepsy. Modafinil has replaced methylphenidate and amphetamine as the first-line treatment of excessive daytime sleepiness (EDS) and sleep attacks, based on randomized, double blind, placebo-controlled clinical trials of modafinil, but on no direct comparison of modafinil versus traditional stimulants. For cataplexy, sleep paralysis, and hypnagogic hallucinations, new antidepressants tend to replace tricyclic antidepressants and selective serotonin reuptake inhibitors (SSRIs) in spite of a lack of randomized, double blind, placebo-controlled clinical trials of these compounds;... (truncated)

Title: Management of patients with risk factors.
This review addresses concomitant diseases and risk factors in patients treated for diseases of the ears, nose and throat in outpatient and hospital services. Besides heart disease, lung disease, liver disease and kidney disease, this article also covers disorders of coagulation (including therapy with new oral anticoagulants) and electrolyte imbalance. Special attention is paid to the prophylaxis, diagnosis and treatment of perioperative delirium. It is also intended to help optimise the preparation for surgical procedures and pharmacotherapy during the hospital stay.

Title: Psychological and psychiatric aspects of treatment of obesity and nonalcoholic fatty liver disease.
Chronic illnesses incur a tremendous cost to American lives in dollars and quality of life. Outcomes in these illnesses are often affected by psychological, behavioral, and pharmacologic issues related to mental illness and psychological symptoms. This article focuses on psychological and psychiatric issues related to the treatment of obesity and nonalcoholic fatty liver disease (NAFLD), including available weight-loss interventions, the complex relationship between psychiatric disorders and obesity, and special considerations regarding use of psychiatric drugs in patients with or at risk for NAFLD and obesity. Recommendations for collaborative care of individuals with comorbid NAFLD and psychological disorders/symptoms are discussed.

Title: Use of Prescribed Psychotropics during Pregnancy: A Systematic Review of Pregnancy, Neonatal, and Childhood Outcomes.
This paper reviews the findings from preclinical animal and human clinical research investigating maternal/fetal, neonatal, and child neurodevelopmental outcomes following prenatal exposure to psychotropic drugs. Evidence for the risks associated with prenatal exposure was examined, including teratogenicity, neurodevelopmental effects, neonatal toxicity, and long-term neurobehavioral consequences (i.e., behavioral teratogenicity). We conducted a comprehensive review of the recent results and conclusions of original research and reviews, respectively, which have investigated the short- and long-term impact of drugs commonly prescribed to pregnant women for psychological disorders, including mood, anxiety, and sleep disorders. Because mental illness in the mother is not a benign event,... (truncated)

MESH:D014196 - trazodone

Summary:

LLM Prediction Score	0.664 (normalized)
LLM Confidence Score	0.990
Golden Answer (Severity Class)	0.625 (normalized)
Prediction Error	0.039

Retrieved Context:

Title: Fulminant hepatic failure induced by venlafaxine and trazodone therapy: a case report.
Although acute hepatitis may be a side effect of many medications, most cases are reversible after treatment interruption, and fulminant hepatic failure (FHF) is rare. Venlafaxine and trazodone are 2 popular antidepressant agents. Alteration of liver enzyme levels has been reported as a side effect of these drugs at normal doses. Herein we have reported the case of a 48-year-old woman without any previous history of liver disease, who developed fulminant liver failure after 4 months of venlafaxine and trazodone therapy. She required liver transplantation, a procedure that was successful with full patient recovery. The first 5 years of follow-up... (truncated)

Title: Alleviating the hepatotoxicity of trazodone via supramolecular encapsulation.
In order to develop a novel strategy to alleviate the inherent hepatotoxicity of antidepressant trazodone (TZ), Cucurbit[7]uril (CB[7]) was adopted as pharmaceutical excipients and was studied for its capability to reduce the hepatotoxicity of TZ via supramolecular encapsulation. CB[7] was found to form strong 1:1 host-guest complexes with TZ and its metabolite m-chlorophenyl piperazine (mCPP), with binding constants of 1.50 (±0.13) × 10⁶ M⁻¹ and 6.90 (±0.49) × 10⁵ M⁻¹, respectively. The supramolecular complexations were examined by ¹H NMR and UV-visible spectroscopic titrations, ESI-MS and ITC. In the presence of 0.5 mM CB[7], the IC₅₀ values of TZ and mCPP on a human normal liver cell line... (truncated)

Title: Trazodone-induced hepatotoxicity: a case report with comments on drug-induced hepatotoxicity.
Trazodone (Desyrel) is a second-generation, nontricyclic antidepressant that has been in use in North America since the early 1980s. It has the advantage of being more sedating and having less anticholinergic side effects than other secondary amines in the piperazine class, namely, desipramine and nortriptyline. Five previous cases of trazodone hepatotoxicity have been reported in the literature, one describing chronic damage and the others, more acute cellular and cholestatic injury. We describe a case of acute reversible liver injury with the use of trazodone. This case is unique in that injury occurred after protracted (18 months) drug use and while... (truncated)

Title: Liver injury associated with antidepressants.
Antidepressants are commonly prescribed and used in the management of depression, anxiety disorders, and other psychiatric illnesses. Antidepressants used in therapeutic dosing ranges are associated with causing several adverse drug reactions including hepatotoxicity. Paroxetine, fluoxetine, fluvoxamine, citalopram, mirtazapine and venlafaxine are associated with reversible liver injury upon discontinuation of the agent. Patient cases of hepatotoxicity involving the use of nefazodone, trazodone, duloxetine, bupropion, and sertraline are linked to causing death in its users. Due to the idiosyncratic nature of hepatotoxicity, monitoring of liver function tests and immediate discontinuation upon abnormal lab findings or signs and symptoms of liver dysfunction are... (truncated)

Title: Hepatotoxicity after short-term trazodone therapy.
To report a case of severe hepatotoxicity from a four-day course of trazodone in a patient being treated according to protocol in a detoxification center.

MESH:C426859 - fosamprenavir

Summary:

LLM Prediction Score	0.414 (normalized)
LLM Confidence Score	0.950
Golden Answer (Severity Class)	0.375 (normalized)
Prediction Error	0.039

Retrieved Context:

Title: Low rate of adverse hepatic events associated with fosamprenavir/ritonavir-based antiretroviral regimens.
To appraise the incidence of liver toxicity in a population of patients receiving fosamprenavir/ritonavir (FPV/r) with a high frequency of viral hepatitis co-infection.

Title: Liver function parameters in HIV/HCV co-infected patients treated with amprenavir and ritonavir and correlation with plasma levels.
Acute liver toxicity is a frequent adverse event that occurs during antiretroviral therapy and was observed in 6-30% of the patients on treatment, especially in presence of HCV coinfection (Cooper et al., 2002, Maida et al., 2006, Sulkowski et al., 2000). A correlation between HCV-associated liver-fibrosis severity and the risk of HAART associated hepatoxicity has been demonstrated (Aranzabal et al., 2005, Sulkowski et al., 2004). This high liver toxicity rate might be due to increased drug exposure in patients with liver disease (Veronese et al., 2000). It has been reported that patients with chronic hepatitis C show significantly reduced CPY3A4... (truncated)

Title: [The treatment of HIV infection].
The combination of zidovudine (AZT) + lamivudine (3TC) twice a day + tenofovir once a day, in HIV-infected, antiretroviral-naive patients leads to the rapid and prolonged reduction in viral load, with good immune response and reasonable tolerance profile. According to the results of the boosted FosAmprenavir solo trial, adherence was simplified by the reduced number of daily administration and tablets (once a day) and hence a decline in virological failure rates. Enfuvirtide, an antiretroviral inhibiting the fusion of the virus membrane and cell, combined with optimal antiviral therapy, leads to virological response in 78% of patients and prolonged for more... (truncated)

Title: Liver toxicity of antiretroviral combinations including fosamprenavir plus ritonavir 1400/100 mg once daily in HIV/hepatitis C virus-coinfected patients.
Abstract Our objective was to evaluate the liver toxicity of antiretroviral regimens including fosamprenavir plus ritonavir (FPV/r) 1400/100 mg once daily (QD) in HIV/hepatitis C virus (HCV)-coinfected patients. This was a prospective cohort study that included 117 HIV/HCV-coinfected patients who started FPV/r 1400/100 mg QD-based antiretroviral therapy (ART) and who neither had received a previous antiretroviral regimen containing FPV nor had a past history of virologic failure while receiving protease inhibitors (PI). The primary end point of the study was the occurrence of grade 3-4 liver enzymes elevations (LEE) within 1 year after starting FPV/r QD. Factors potentially associated with... (truncated)

Title: A reduced grade of liver fibro-steatosis after raltegravir, maraviroc and fosamprenavir in an HIV/HCV co-infected patient with chronic hepatitis, cardiomyopathy, intolerance to nelfinavir and a marked increase of serum creatine phosphokinase levels probably related to integrase inhibitor use.
The use of new antiretroviral drugs in HIV infection is particularly important in patients with intolerance or resistance to other antiretroviral agents. Raltegravir and maraviroc represent new, important resources in salvage regimens. A reduced grade of liver fibro-steatosis after a combination of raltegravir and maraviroc (second-line) has not been studied and the mechanism by which these new drug classes induced a marked reduction of grade of liver diseases is currently unknown. In the present case report, nested in an ongoing multicentre observational study on the use of new antiretroviral inhibitors in heavy treatment-experienced HIV patients, we evaluated the correlation between... (truncated)

MESH:C495910 - ramelteon

Summary:

LLM Prediction Score	0.039 (normalized)
LLM Confidence Score	0.990
Golden Answer (Severity Class)	0.0 (normalized)
Prediction Error	0.039

Retrieved Context:

Title: Endogenous and Exogenous Melatonin Exposure Attenuates Hepatic MT₁ Melatonin Receptor Protein Expression in Rat.
Melatonin receptors are highly relevant for the hepatoprotective effects of the pineal hormone melatonin after experimental hemorrhagic shock in rats. In this study, we sought to determine the spatial expression pattern and a putative regulation of two melatonin receptors, membrane bound type 1 and 2 (MT₁ and MT₂), in the liver of rats. In a male rat model (Sprague Dawley) of hemorrhage and resuscitation, we investigated the gene expression and protein of MT₁ and MT₂ in rat liver by utilizing real-time quantitative polymerase chain reaction, a western blot analysis, and immunohistochemistry. Plasma melatonin content was measured by an enzyme-linked immunosorbent... (truncated)

Title: Selective activation of melatonin receptors with ramelteon improves liver function and hepatic perfusion after hemorrhagic shock in rat.
Melatonin may attenuate organ damage via direct antioxidative properties, and was recently demonstrated to reduce cardiac and hepatic injury through receptor-dependent effects. However, the relevance of an isolated activation of melatonin receptors for organ protection, excluding direct antioxidant effects, has not been established yet. This study was designed to investigate whether therapy with melatonin receptor agonist and hypnotic substance ramelteon may improve liver function, hepatic perfusion, and hepatic integrity after hemorrhagic shock in rat.

Title: A review of ramelteon in the treatment of sleep disorders.
Ramelteon is a selective melatonin receptor (MT(1) and MT(2)) agonist that has been approved by the US Food and Drug Administration for the treatment of insomnia characterized by difficulty with sleep onset. It is the only approved sleep-promoting medication that does not have a direct sedating effect, but rather enhances sleep through effects on sleep regulatory mechanisms within the suprachiasmatic nucleus. Ramelteon has been shown to have no abuse liability and therefore is not scheduled by the U.S. Drug Enforcement Agency as a controlled substance. It is available as an 8 mg tablet, which should be taken approximately 30 minutes... (truncated)

Title: Benefits and Risks of Melatonin in Hepatic and Pancreatic Disorders; A Review of Clinical Evidences.
Melatonin is the "clock factor" produced from the pineal gland dominating regular circadian rhythm in mammalians. It is an indoleamine with potent multifunctional pharmacological effects, both receptor dependent and non-receptor dependent effects, including antioxidant and anti-inflammatory activities. The aim of this review is to summarize clinical evidence related to melatonin's effectiveness in the treatment of liver and pancreas diseases. Databases including PubMed, Scopus, and Cochran Library were searched up to November 2020.Finally, this review has summarized up-to-date clinical evidence to investigate the efficacy and safety of melatonin for the management of liver and pancreas diseases. Melatonin has been demonstrated to... (truncated)

Title: Therapeutic effects of melatonin receptor agonists on sleep and comorbid disorders.
Several melatonin receptors agonists (ramelteon, prolonged-release melatonin, agomelatine and tasimelteon) have recently become available for the treatment of insomnia, depression and circadian rhythms sleep-wake disorders. The efficacy and safety profiles of these compounds in the treatment of the indicated disorders are reviewed. Accumulating evidence indicates that sleep-wake disorders and co-existing medical conditions are mutually exacerbating. This understanding has now been incorporated into the new Diagnostic and Statistical Manual of Mental Disorders, 5th Edition (DSM-5). Therefore, when evaluating the risk/benefit ratio of sleep drugs, it is pertinent to also evaluate their effects on wake and comorbid condition. Beneficial effects of melatonin... (truncated)

MESH:D014046 - tolmetin

Summary:

LLM Prediction Score	0.413 (normalized)
LLM Confidence Score	0.910
Golden Answer (Severity Class)	0.375 (normalized)
Prediction Error	0.038

Retrieved Context:

Title: Tolmetin sodium-loaded thermosensitive mucoadhesive liquid suppositories for rectal delivery; strategy to overcome oral delivery drawbacks.
Tolmetin sodium (TS) is a nonsteroidal anti-inflammatory drug (NSAID) indicated for treatment of musculoskeletal issues. As other NSAID, TS displays a marked side effects on the gastro-intestinal (GI) tract after oral administration. Traditional solid suppositories can cause pain and discomfort for patients, may reach the end of the colon; consequently, the drug can undergo the first-pass effect. TS liquid suppository (TS-_{LS}) was developed to enhance patient compliance and rectal mucosal safety in high-risk patients receiving highly NSAID therapy. This work was conducted to optimize and evaluate Poloxamer P407/P188-based thermoresponsive TS-_{LS} by using mucoadhesive polymers such as methylcellulose (MC). TS-_{LS} was... (truncated)

Title: Gastrointestinal and hepatic toxicity of selective and non-selective cyclooxygenase-2 inhibitors in pregnant and non-pregnant rats.
The aim of the study was to evaluate the toxicity of non-selective (tolmetin, ibuprofen and piroxicam) and selective (DFU) cyclooxygenase-2 inhibitors on pregnant and non-pregnant rats. The drugs were administered orally once (DFU, piroxicam) or three times (tolmetin, ibuprofen) a day from days 8 through 21 of gestation experiment in three doses. The initial dose was similar to the human antiinflammatory one and set as 8.5 mg/kg (tolmetin, ibuprofen), 0.3 mg/kg (piroxicam) and 0.2 mg/kg (DFU). The middle dose was increased 10 times and the highest one 100 times the initial dose. The highest dose for ibuprofen was set at... (truncated)

Title: A correlation between the in vitro drug toxicity of drugs to cell lines that express human P450s and their propensity to cause liver injury in humans.
Drug toxicity to T-antigen-immortalized human liver epithelial (THLE) cells stably transfected with plasmid vectors that encoded human cytochrome P450s 1A2, 2C9, 2C19, 2D6, or 3A4, or an empty plasmid vector (THLE-Null), was investigated. An automated screening platform, which included 1% dimethyl sulfoxide (DMSO) vehicle, 2.7% bovine serum in the culture medium, and assessed
3-(4,5-dimethylthiazol-2-yl)-5-(3-carboxymethoxyphenyl)-2-(4-sulfophenyl)-2H-tetrazolium reduction, was used to evaluate the cytotoxicity of 103 drugs after 24h. Twenty-two drugs caused cytotoxicity to THLE-Null cells, with EC_{50} ≤ 200 μM; 21 of these drugs (95%) have been reported to cause human liver injury. Eleven drugs exhibited lower EC_{50} values in cells transfected... (truncated)

Title: Lack of hepatotoxic effects of parenteral ketorolac in the hospital setting.
No large controlled studies to date have examined the hepatic safety of parenteral ketorolac, which is used to treat acutely ill hospitalized patients who may be at greatest risk of liver injury.

Title: Modeling the Bioactivation and Subsequent Reactivity of Drugs.
Electrophilically reactive drug metabolites are implicated in many adverse drug reactions. In this mechanism-termed bioactivation-metabolic enzymes convert drugs into reactive metabolites that often conjugate to nucleophilic sites within biological macromolecules like proteins. Toxic metabolite-product adducts induce severe immune responses that can cause sometimes fatal disorders, most commonly in the form of liver injury, blood dyscrasia, or the dermatologic conditions toxic epidermal necrolysis and Stevens-Johnson syndrome. This study models four of the most common metabolic transformations that result in bioactivation: quinone formation, epoxidation, thiophene sulfur-oxidation, and nitroaromatic reduction, by synthesizing models of metabolism and reactivity. First, the metabolism models predict the... (truncated)

MESH:D000077430 - nabumetone

Summary:

LLM Prediction Score	0.413 (normalized)
LLM Confidence Score	0.930
Golden Answer (Severity Class)	0.375 (normalized)
Prediction Error	0.038

Retrieved Context:

Title: Liver injury from nonsteroidal anti-inflammatory drugs in the United States.
Nonsteroidal anti-inflammatory drugs (NSAIDs) are commonly used and have been associated with hepatotoxicity. Studies of liver injury from NSAIDs have been retrospective and prospective data are lacking. The aim was to report the features and outcomes of the subjects with severe drug-induced liver injury from NSAIDS.

Title: Comparison of the efficacy and safety of oxaprozin and nabumetone in the treatment of patients with osteoarthritis of the knee.
This multicenter, 6-week, double-blind, placebo-controlled, parallel-group study compared the efficacy and safety of oxaprozin 1200 mg once daily with that of nabumetone 1000 mg once daily in patients with moderate-to-severe osteoarthritis (OA) of the knee. To be eligible, patients had to experience a flare of OA within 2 weeks of discontinuing their usual OA medication (nonsteroidal anti-inflammatory drug or analgesic). Eligible patients were assessed at baseline and then randomized to receive oxaprozin (n = 109), nabumetone (n = 110), or placebo (n = 109). Efficacy assessments were performed at weeks 1, 2, 4, and 6. Primary efficacy variables included knee... (truncated)

Title: Association of Aspirin with Hepatocellular Carcinoma and Liver-Related Mortality.
More information is needed about the long-term effects of low-dose aspirin (≤160 mg) on incident hepatocellular carcinoma, liver-related mortality, and gastrointestinal bleeding in persons with chronic hepatitis B or hepatitis C virus infection.

Title: Application of a Rat Liver Drug Bioactivation Transcriptional Response Assay Early in Drug Development That Informs Chemically Reactive Metabolite Formation and Potential for Drug-induced Liver Injury.
Drug-induced liver injury is a major reason for drug candidate attrition from development, denied commercialization, market withdrawal, and restricted prescribing of pharmaceuticals. The metabolic bioactivation of drugs to chemically reactive metabolites (CRMs) contribute to liver-associated adverse drug reactions in humans that often goes undetected in conventional animal toxicology studies. A challenge for pharmaceutical drug discovery has been reliably selecting drug candidates with a low liability of forming CRM and reduced drug-induced liver injury potential, at projected therapeutic doses, without falsely restricting the development of safe drugs. We have developed an in vivo rat liver transcriptional signature biomarker reflecting the cellular... (truncated)

Title: Non-steroidal anti-inflammatory drugs (NSAIDs) and organ damage: A current perspective.
Owing to the efficacy in reducing pain and inflammation, non-steroidal anti-inflammatory drugs (NSAIDs) are amongst the most popularly used medicines confirming their position in the WHO's Model List of Essential Medicines. With escalating musculoskeletal complications, as evident from 2016 Global Burden of Disease data, NSAID usage is evidently unavoidable. Apart from analgesic, anti-inflammatory and antipyretic efficacies, NSAIDs are further documented to offer protection against diverse critical disorders including cancer and heart attacks. However, data from multiple placebo-controlled trials and meta-analyses studies alarmingly signify the adverse effects of NSAIDs in gastrointestinal, cardiovascular, hepatic, renal, cerebral and pulmonary complications. Although extensive research... (truncated)

MESH:D003520 - cyclophosphamide

Summary:

LLM Prediction Score	0.663 (normalized)
LLM Confidence Score	0.990
Golden Answer (Severity Class)	0.625 (normalized)
Prediction Error	0.038

Retrieved Context:

Title: Low-Dose Cyclophosphamide Associated With Hyponatremia and Hepatotoxicity.
Cyclophosphamide (CY) is an alkylating agent often used as a chemotherapeutic agent, with increasing use as an immunosuppressant. Cyclophosphamide has many established adverse effects, including hyponatremia and limited reports of hepatotoxicity, particularly in high-dose treatment. A case of simultaneous hyponatremia and acute liver injury associated with the initiation of cyclophosphamide two weeks prior is discussed here. A 73-year-old male with acquired hemophilia A/factor VIII deficiency presented to the emergency department (ED) with four days of hip pain and was found to have jaundice and confusion. Laboratory evaluation demonstrated hyponatremia and an acute liver injury associated with his recent cyclophosphamide use.... (truncated)

Title: Cyclophosphamide metabolism, liver toxicity, and mortality following hematopoietic stem cell transplantation.
Liver toxicity caused by high-dose myeloablative therapy leads to significant morbidity after hematopoietic cell transplantation. We examined the hypothesis that liver toxicity after cyclophosphamide and total body irradiation is related to cyclophosphamide through its metabolism to toxins. Cyclophosphamide was infused at 60 mg/kg over 1 to 2 hours on each of 2 consecutive days, followed by total body irradiation. Plasma was analyzed for cyclophosphamide and its major metabolites. Liver toxicity was scored by the development of sinusoidal obstruction syndrome (veno-occlusive disease) and by total serum bilirubin levels. The hazards of liver toxicity, nonrelapse mortality, tumor relapse, and survival were calculated... (truncated)

Title: Sinapic acid attenuates cyclophosphamide-induced liver toxicity in mice by modulating oxidative stress, NF-κB, and caspase-3.
Cyclophosphamide (CP) as an antineoplastic drug is widely used in cancer patients, and liver toxicity is one of its complications. Sinapic acid (SA) as a natural phenylpropanoid has anti-oxidant, anti-inflammatory, and anti-cancer properties.

Title: Cyclophosphamide-induced liver necrosis: a possible interaction with azathioprine.
Cyclophosphamide is a valuable drug for the treatment of vasculitis and hepatic toxicity resulting from its use has been reported rarely. We describe four patients in whom cyclophosphamide was associated with liver injury when its administration had been preceded by azathioprine. Microscopy of hepatic biopsy material in three of these cases showed liver cell necrosis. In two of the patients, cyclophosphamide had been given previously without earlier azathioprine and hepatic damage had not been noted. An apparent interaction of cyclophosphamide with azathioprine to cause liver cell necrosis has important implications for the treatment of patients with vasculitis and related disorders.

Title: Panax ginseng is superior to vitamin E as a hepatoprotector against cyclophosphamide-induced liver damage.
Cyclophosphamide (CPh) is a frequently used drug, in human and animals for its immunosuppressive and anticancer potential. However, it is metabolized by the liver yielding damaging toxicants (to the liver itself and other non-target vital organs) via oxidative stress, apoptosis induction and finally necrosis. Since there is no escaping of using such harmful medications, we focused on alleviating its side-effects. Panax ginseng Meyer is a potent candidate, and we still lack adequate information on its hepatoprotective role against cyclophosphamide-induced liver-damage.

MESH:D001379 - azathioprine

Summary:

LLM Prediction Score	0.587 (normalized)
LLM Confidence Score	0.990
Golden Answer (Severity Class)	0.625 (normalized)
Prediction Error	0.038

Retrieved Context:

Title: Late-Onset Acute Liver Injury From Azathioprine.
Azathioprine is a widely prescribed immunosuppressant. Although hepatotoxicity is rare, it commonly presents as mild asymptomatic liver enzyme elevation or acute cholestatic liver injury. We report a case of a 46-year-old woman who presented with jaundice, abdominal pain, fatigue, and elevated aminotransferases in a cholestatic pattern. Endoscopic retrograde cholangiopancreatogram demonstrated no abnormalities, and recently started medications were discontinued without improvement. Liver biopsy was performed, which was consistent with drug-induced liver injury. Despite multiple years of treatment without issue, after azathioprine was discontinued, symptoms and laboratory abnormalities resolved. This case highlights azathioprine's potential for hepatotoxicity even multiple years after initiation.

Title: Azathioprine-induced hepatitis and cholestasis occurring 1 year after treatment.
Azathioprine is an immunosuppressive medication used in the management of many autoimmune conditions. Commonly reported adverse effects from azathioprine therapy are nausea and bone marrow suppression, while less common side effects include hepatotoxicity. We present the case of a 47-year-old man with a history of myasthenia gravis on azathioprine for 1 year, who presented to our institution with painless jaundice. On initial laboratory evaluation, the level of aspartate aminotransferase, alanine aminotransferase and total bilirubin were markedly elevated. Owing to the potential diagnosis of acute liver failure secondary to azathioprine toxicity, this medication was discontinued. A liver biopsy demonstrating drug-induced liver... (truncated)

Title: Cholestatic hepatocellular injury with azathioprine: a case report and review of the mechanisms of hepatotoxicity.
Azathioprine is a drug commonly used for the treatment of inflammatory bowel disease, organ transplantation and various autoimmune diseases. Hepatotoxicity is a rare, but important complication of this drug. The cases reported to date can be grouped into three syndromes: hypersensitivity; idiosyncratic cholestatic reaction; and presumed endothelial cell injury with resultant raised portal pressures, venoocclusive disease or peliosis hepatis. The components of azathioprine, 6-mercaptopurine and the imidazole group, may play different roles in the pathogenesis of hepatotoxicity. The strong association with male sex, and perhaps with human leukocyte antigen type, suggests a genetic predisposition of unknown type. Many of the... (truncated)

Title: Azathioprine induced fever, chills, rash, and hepatotoxicity in rheumatoid arthritis.
Within one year three of 25 patients with rheumatoid arthritis treated with azathioprine 100 mg daily developed the following adverse reactions less than two weeks after starting treatment: patient one showed fever with chills, rash, and severe liver function abnormalities suggestive of cholestasis; the second patient had fever, nausea, diarrhoea, and moderately raised liver enzymes; the third patient showed very high fever and severe chills. In two patients the drug was rechallenged, with more rapidly arising and more severe symptoms. In one case raised liver enzymes persisted until seven months after discontinuation of azathioprine. Hypersensitivity reactions and hepatotoxicity of azathioprine... (truncated)

Title: Severe azathioprine-induced liver injury 22 months after initiation of treatment.
Drug-induced liver injury (DILI) is the leading cause of acute liver failure in high-income countries. Acute cholestasis is one of the most common forms of hepatotoxicity induced by azathioprine. It usually begins during the first year of treatment, with most cases reported during the first month. We describe an uncommon case of DILI that occurred after 22 months of drug administration. A woman in her 50s was hospitalised because of jaundice and asthenia. She had been treated with azathioprine for myasthenia gravis during the last 2 years. Acute cholestatic injury was diagnosed. After ruling out most common causes of cholestasis,... (truncated)

MESH:D010208 - papaverine

Summary:

LLM Prediction Score	0.589 (normalized)
LLM Confidence Score	0.990
Golden Answer (Severity Class)	0.625 (normalized)
Prediction Error	0.036

Retrieved Context:

Title: Papaverine and hepatotoxicity.
Six of 14 patients receiving a sustained release papaverine preparation developed abnormal liver function tests. One patient had jaundice and another abnormal liver histology on a biopsy specimen. This is only the third report of impaired liver function following the administration of papaverine--a drug which has been used in clinical medicine for over 100 years.

Title: Papaverine-induced chronic liver disease.
The case history of a women with jaundice and laboratory evidence of chronic active hepatitis and cirrhosis is reported. The patient had taken papaverine for 6 yr for cerebral arterial disease. Jaundice improved after the drug was discontinued but reappeared when papaverine therapy was reintroduced. Antinuclear antibody and smooth muscle antibody were present. Clinical manifestations disappeared and laboratory results again returned to normal upon withdrawal of papaverine. This case suggests that papaverine should be added to the list of drugs known to produce chronic active hepatitis and cirrhosis.

Title: Quinidine-induced hepatotoxicity revisited.
Although quinidine has been widely used since the beginning of the century, quinidine-induced hepatotoxicity has been recently reported in the literature. We describe a reversible case of quinidine-induced hepatotoxicity. A 62-y-old male with a past medical history of atrial flutter and adult onset diabetes was admitted to the hospital with a 3-d history of diarrhea, nausea, fever, chills and palpitations. Past medications included 7.5 mg glyburide daily for 4 y, 0.25 mg digoxin daily for 3 w, 324 mg quinidine gluconate 3 times daily for 2 w, and 150 mg papaverine daily for 2 y. On admission, liver enzyme levels... (truncated)

Title: Drug-induced liver disease in Denmark. An analysis of 572 cases of hepatotoxicity reported to the Danish Board of Adverse Reactions to Drugs.
During the decade 1968-1978 the Danish Board of Adverse Reactions to Drugs received 572 (6% of the total number) reports on hepatotoxicity. Halothane amounted to one fourth of the reported cases. Among the 94 psychotropic-induced adverse drug reactions 54 cases were attributed to chlorpromazine, 10 to tricyclic antidepressants, and only 2 to benzodiazepines. Considering the drug consumption data, the combination trimethoprim-sulfamethoxazole is nearly five times more frequently associated with hepatotoxicity than administration of sulfamethizole. Almost two thirds of the hepatotoxic reactions were classified as cytotoxic. Halothane, oxyphenisatin, rifampicin, alfa-methyldopa, papaverine, phenytoin, and ajmaline were almost exclusively related to cytotoxic reactions.... (truncated)

Title: Hepatotoxicity related to intracavernous pharmacotherapy with papaverine.
To determine the incidence of hepatotoxicity related to self-administration of intracavernous papaverine or papaverine/phentolamine (bimix).

MESH:D003523 - cycloserine

Summary:

LLM Prediction Score	0.340 (normalized)
LLM Confidence Score	0.990
Golden Answer (Severity Class)	0.375 (normalized)
Prediction Error	0.035

Retrieved Context:

Title: Ofloxacin in miliary tuberculosis.
We report one case of severe miliary tuberculosis with liver failure and respiratory insufficiency in a twenty-seven year old patient. We emphasize the presence of hepatic, occular and vestibular toxicities secondary to the treatment and the usefulness of ofloxacin with cycloserine given for nine months.

Title: Antituberculous therapy-induced fulminant hepatic failure: successful treatment with liver transplantation and nonstandard antituberculous therapy.
Standard antituberculous therapy including isoniazid, rifampin, ethambutol, and pyrazinamide is widely used for the treatment of active tuberculosis. Its most important side effect is hepatotoxicity, ranging from asymptomatic transaminitis to fulminant hepatic failure. A 19-year-old woman was admitted to our unit due to jaundice and unconsciousness. According to her past medical history, she was diagnosed as having extrapulmonary tuberculosis and had been prescribed standard antituberculous therapy. The patient became icteric and unconscious on the fourth day after therapy initiation. She was diagnosed with drug-induced acute fulminant hepatic failure and underwent living-related liver transplantation. Nonhepatotoxic antituberculous therapy (cycloserine, ciprofloxacin, streptomycin, and... (truncated)

Title: Adverse reactions to daily and intermittent rifampicin regimens for pulmonary tuberculosis in Hong Kong.
This paper reports the nature, incidence, and severity of adverse reactions to regimens of rifampicin and ethambutol given once weekly, twice weekly, or daily and to a standard reserve regimen in a total of 330 Chinese failure patients who completed at least six months' chemotherapy in a therapeutic comparison in Hong Kong.The adverse reactions which occurred on the regimens of intermittent rifampicin were termed cutaneous, abdominal, "flu", and respiratory; in addition, purpura and abnormal liver function tests were encountered. There was an association of adverse reactions with the interval between doses and with the dose size of rifampicin, the highest... (truncated)

Title: A Case of Cycloserine-Induced Lichenoid Drug Eruption Supported by the Lymphocyte Transformation Test.
Lichenoid drug eruption (LDE) is a rare form of delayed-type drug eruption. Among anti-tuberculosis (Tb) agents, cycloserine (CS) has been reported as a rare cause of LDE. Positive results on the lymphocyte transformation test (LTT) have not been reported in patients with LDE. In the present case, we performed LTT and a patch test, and successfully proved CS as the offending drug in this patient, who had been treated with multiple anti-Tb drugs. These observations suggest that CS should be considered a possible cause of LDE and that LTT can be an option for the diagnosis of LDE.

Title: Anti-Tuberculosis Treatment: Induced Hepatotoxicity - A Case Report.
Tuberculosis is a potentially communicable disease that can infect any organ in the body such as bones, kidney, intestine but primarily involves lung parenchyma (Pulmonary tuberculosis). The prevalence of TB is 256 per 100,000 population in India. Hepatotoxicity, gastrointestinal and neurological disorders were some the Adverse Drug Reactions (ADR's) reported that significantly increases the mortality rate which leads to decreased efficacy of the treatment. Hepatotoxicity is the most commonly reported ADR in patients treated with anti-tubercular drugs such as isoniazid, rifampicin and pyrazinamide. Clinical manifestations of hepatotoxicity include abdominal pain, nausea, vomiting, and jaundice. We report the case of a... (truncated)

MESH:D015255 - idarubicin

Summary:

LLM Prediction Score	0.341 (normalized)
LLM Confidence Score	0.980
Golden Answer (Severity Class)	0.375 (normalized)
Prediction Error	0.034

Retrieved Context:

Title: A phase II study of idarubicin (4-demethoxydaunorubicin) in advanced myeloma.
Idarubicin (IDA) is an anthracycline analog which differs from the parent compound by the substitution of a C4 methoxyl group with an hydrogen atom in the aglycone moiety. This drug has shown greater potency and activity in experimental and human leukemias and lymphomas by intravenous and oral routes of administration together with less cardiotoxicity than doxorubicin (DX) and daunorubicin (DNR). We have treated 15 patients with advanced multiple myeloma (MM) refractory or relapsed to standard chemotherapy regimens. The treatment schedule consisted of idarubicin 40 mg/m2 orally on day 1 every 3 weeks for 6-8 months. We obtained 8/14 partial response,... (truncated)

Title: 4-demethoxydaunorubicin (idarubicin) in combination with 1-beta-D-arabinofuranosylcytosine in the treatment of relapsed or refractory acute leukemia.
We conducted a Phase I-II trial of 4-demethoxydaunorubicin (idarubicin, IDR) in combination with 1-beta-D-arabinofuranosylcytosine (ara-C) in 51 patients with relapsed or refractory acute nonlymphocytic leukemia, acute lymphocytic leukemia, or chronic myelogenous leukemia in blast crisis. Only 1 of 12 patients treated at the first dose level (idarubicin, 10 mg/m2/day for 3 days and ara-C, 25 mg/m2 i.v. bolus followed by 200 mg/m2 continuous infusion daily for 5 days) achieved aplasia and complete remission. The dose of idarubicin was subsequently increased to 10 mg/m2/day for 4 days with the ara-C dose held constant. Complete remission incidence for this dose schedule was:... (truncated)

Title: Idarubicin for remission induction of acute myeloid leukemia: United Kingdom multicenter experience.
Ninety-eight adult patients with acute myeloid leukemia were given variable remission induction/consolidation regimens containing idarubicin. Sixty-nine (70%) were new cases (median age, 56 years) and 29 (30%) were in relapse (n = 24) or had primary refractory disease (n = 5) (median age, 46 years). Complete remission (CR) rates were 57% (39 of 69 patients) of the newly diagnosed patients, with no difference for those below or above 55 years of age (56% v 59%) or for patients exhibiting white blood cell counts of less or more than 50 x 10(9)/L (52% v 69%; P = .8). Of the 39... (truncated)

Title: Preliminary observations on intravenous idarubicin (4-demethoxydaunorubicin) in the chronic and accelerated phase of chronic myelogenous leukemia.
The study included 13 patients with chronic myelogenous leukemia (8 in the chronic phase with high WBC counts at onset, and 5 in the accelerated phase, poorly responding to conventional drugs for the chronic phase). They were treated with 4-demethoxydaunorubicin (idarubicin), a new anthracycline analog more active than daunorubicin (DNR) and doxorubicin (DX) in experimental tumor models which offers a higher therapeutic index than existing anthracyclines. Idarubicin was administered i.v. at the dose of 8 mg/m2 on days 1, 3 and 5. All patients in the chronic phase (8/8) developed significant leukopenia. Five of these 8 patients showed complete reduction... (truncated)

Title: Idarubicin and cytarabine in combination with gemtuzumab ozogamicin (IAGO) for untreated patients with high-risk MDS or AML evolved from MDS: a phase II study from the EORTC and GIMEMA Leukemia Groups (protocol 06013).
The primary objective of this trial was to assess the feasibility, toxicity profile, and antitumor activity of gemtuzumab ozogamicin (GO) combined with a chemotherapy remission-induction regimen in adults with untreated high-risk myelodysplastic syndrome (HR-MDS) or secondary acute myeloid leukemia (sAML). In this phase II trial, 30 patients with median age of 58 years received 1 day of GO as a 1-h infusion at the dose level of 5 mg/m(2) on day 7 of the remission-induction course further consisting of a continuous infusion of cytarabine 100 mg/m(2)/day for 10 days and idarubicin 12 mg/m(2)/day on days 1, 3, and 5. A... (truncated)

MESH:C430916 - regadenoson

Summary:

LLM Prediction Score	0.034 (normalized)
LLM Confidence Score	0.980
Golden Answer (Severity Class)	0.0 (normalized)
Prediction Error	0.034

Retrieved Context:

Title: Adenosine receptors as drug targets--what are the challenges?
Adenosine signalling has long been a target for drug development, with adenosine itself or its derivatives being used clinically since the 1940s. In addition, methylxanthines such as caffeine have profound biological effects as antagonists at adenosine receptors. Moreover, drugs such as dipyridamole and methotrexate act by enhancing the activation of adenosine receptors. There is strong evidence that adenosine has a functional role in many diseases, and several pharmacological compounds specifically targeting individual adenosine receptors--either directly or indirectly--have now entered the clinic. However, only one adenosine receptor-specific agent--the adenosine A2A receptor agonist regadenoson (Lexiscan; Astellas Pharma)--has so far gained approval from... (truncated)

Title: Emerging adenosine receptor agonists: an update.
Adenosine receptors (ARs), the major targets of caffeine and theophylline, comprise four receptor subtypes designated as A(1), A(2A), A(2B) and A(3). Over a dozen AR agonists are currently in clinical trials for various conditions, including cardiac arrhythmias, neuropathic pain, myocardial perfusion imaging, cardiac ischemia, inflammatory diseases and cancer. Adenosine (nonselective), regadenoson (A(2A)) and dipyridamole (act indirectly via ARs) have received regulatory approval for clinical use. The present editorial will give a brief update on the current status of AR agonists in clinical trials.

Title: Potential for developing purinergic drugs for gastrointestinal diseases.
Treatments for inflammatory bowel disease (IBD), irritable bowel syndrome (IBS), functional dyspepsia, or motility disorders are not adequate, and purinergic drugs offer exciting new possibilities. Gastrointestinal symptoms that could be targeted for therapy include visceral pain, inflammatory pain, dysmotility, constipation, and diarrhea. The focus of this review is on the potential for developing purinergic drugs for clinical trials to treat gastrointestinal symptoms. Purinergic receptors are divided into adenosine P1 (A(1), A(2A), A(2B), A(3)), ionotropic ATP-gated P2X ion channel (P2X(1-7)), or metabotropic P2Y(1,2,4,6,11-14) receptors. There is good experimental evidence for targeting A(2A), A(2B), A(3), P2X(7), and P2X(3) receptors or increasing endogenous... (truncated)

Title: Adenosine A2a Receptor Stimulation Attenuates Ischemia-Reperfusion Injury and Improves Survival in A Porcine Model of DCD Liver Transplantation.
Orthotopic liver transplantation (OLT) using allografts from donation after circulatory death (DCD) is potentially associated with compromised clinical outcomes due to ischemia-reperfusion injury (IRI)-induced organ damage and graft-related complications. The aim of this study was to provide in vivo data on the effects of adenosine A_{2a} receptor stimulation in a clinically relevant large animal model of DCD liver transplantation. Cardiac arrest was induced in German Landrace pigs (<i>n</i> = 10; 20-25 kg). After 30 min of warm ischemia, the donor liver was retrieved following a cold flush with 3 L of histidine-tryptophan-ketoglutarate-HTK solution. Animals of the treatment group (<i>n</i> =... (truncated)

Title: Pharmacological Preconditioning by Adenosine A2a Receptor Stimulation: Features of the Protected Liver Cell Phenotype.
Ischemic preconditioning (IP) of the liver by a brief interruption of the blood flow protects the damage induced by a subsequent ischemia/reperfusion (I/R) preventing parenchymal and nonparenchymal liver cell damage. The discovery of IP has shown the existence of intrinsic systems of cytoprotection whose activation can stave off the progression of irreversible tissue damage. Deciphering the molecular mediators that underlie the cytoprotective effects of preconditioning can pave the way to important therapeutic possibilities. Pharmacological activation of critical mediators of IP would be expected to emulate or even to intensify its salubrious effects. In vitro and in vivo studies have demonstrated... (truncated)

MESH:D015119 - aminocaproic acid

Summary:

LLM Prediction Score	0.034 (normalized)
LLM Confidence Score	0.970
Golden Answer (Severity Class)	0.0 (normalized)
Prediction Error	0.034

Retrieved Context:

Title: Severe acute hepatitis of unknown causes in children - Current findings, questions, opinions, and recommendations, a mini-review.
Since October 2021 in Alabama, the United States, and March 2022 in central Scotland, the United Kingdom, the number of cases of severe acute hepatitis of unknown etiology/causes in children was found to increase, and the total number of cases has reached 920 worldwide by June 22 this year, 45 cases (5%) required liver transplantation, and 18 cases (2%) died according to World Health Organization (WHO). To understand the basic characteristics of this disease/syndrome, a literature search was performed at PubMed, websites of WHO, UK Health Security Agency, and US and European Centers for Disease Control and Prevention, and more... (truncated)

Title: [The efficacy of x-ray endovascular interventions in hepatic lesions and diseases].
Experience in roentgenovascular interventions was summed up for 14 patients aged 17 to 38 (11 men and 3 women). Indications for them were closed liver damages (6), life-threatening hemorrhage resulting from repeated operations for liver echinococcosis (1), primary hepatocellular cancer (4), liver hemangioma (2), metastases of melanoma to the liver (1). Hemostatic sponge, Gianturco's spiral, 60% glucose solution in combination with aminocaproic acid were used as embolizing agents. Complications were observed in one case. Gall bladder necrosis developed after distal-proximal embolization with a hemostatic sponge and spirals. Roentgenovascular occlusion of the hepatic artery can be used to stop dangerous hemorrhage,... (truncated)

Title: The use of epsilon-aminocaproic acid for the management of hemophilia in dental and oral surgery patients.
The use of epsilon-aminocaproic acid has proved to be an efficient and practical method for treating hemophiliacs who require dental work. In the past, patients requiring extractions were admitted to a hospital for approximately ten days and received replacement infusions every 12 hours during their stay. This resulted in a large expense because of the cost of the material and the hospitalization itself, not to mention the trauma sustained by the patient both physically and psychologically. Also, by decreasing the number of factor infusions, the risk of complications such as the transmission of hepatitis, allergic reaction, or inhibitor formation decreased.... (truncated)

Title: hEPCR.hTBM.hCD47.hHO-1 with donor clodronate and DDAVP treatment improves perfusion and function of GalTKO.hCD46 porcine livers perfused with human blood.
Platelet sequestration, inflammation, and inappropriate coagulation cascade activation are prominent in liver xenotransplant models and are associated with poor outcomes. Here, we evaluate a cassette of six additional genetic modifications to reduce anti-pig antibody binding (α-1,3-galactosyl transferase knockout [GalTKO]) and target coagulation dysregulation (human endothelial protein C receptor [hEPRC] and thrombomodulin [hTBM]), complement pathway regulation (human membrane cofactor protein, hCD46), inflammation heme oxygenase 1 [hHO-1]), and a self-recognition receptor (integrin-associated protein [hCD47]), as well as donor pharmacologic treatments designed to blunt these phenomena.

Title: Combination of Intravenous and Intra-Articular Application of Tranexamic Acid and Epsilon-Aminocaproic Acid in Primary Total Knee Arthroplasty: A Prospective Randomized Controlled Trial.
There were limited randomized controlled trials (RCTs) of epsilon-aminocaproic acid (EACA) versus tranexamic acid (TXA) in total knee arthroplasty (TKA). The aim of the study was to compare the efficacy and safety of TXA and EACA in the combination of intravenous (IV) and intra-articular (IA) administration on reducing blood loss in patients following primary TKA.

MESH:D019259 - lamivudine

Summary:

LLM Prediction Score	0.408 (normalized)
LLM Confidence Score	0.990
Golden Answer (Severity Class)	0.375 (normalized)
Prediction Error	0.033

Retrieved Context:

Title: Abacavir/Dolutegravir/Lamivudine (Triumeq)-Induced Liver Toxicity in a Human Immunodeficiency Virus-Infected Patient.
Drug-induced liver injury related to Triumeq (abacavir/lamivudine/dolutegravir) has not been reported in clinical trials. We report a case of hepatotoxicity related to Triumeq exposure in a human immunodeficiency virus-infected patient. Clinicians should remain aware of the risk for acute and late-onset hepatitis with these agents. Close monitoring is recommended.

Title: Hepatotoxicity of antiretroviral therapy.
Hepatotoxicity is a serious complication in patients taking HAART. Coinfection with hepatitis viruses increases the risk of liver toxicity while taking antiretroviral therapy. Baseline transaminases should be checked before beginning antiretorviral therapy and all patients should be screened for pre-existing liver disease, most notably hepatitis B and C infections. Regular monitoring of transaminases is mandatory when commencing antiretroviral therapy. In patients with normal liver function, transaminases may be checked monthly after commencing HAART for the first 3 months. If stable this can be broadened to 3 month intervals. In patients with pre-existing liver disease monitoring should be performed more frequently... (truncated)

Title: Adverse effects of oral antiviral therapy in chronic hepatitis B.
Oral nucleoside/nucleotide analogues (NAs) are currently the backbone of chronic hepatitis B (CHB) infection treatment. They are generally well-tolerated by patients and safe to use. To date, a significant number of patients have been treated with NAs. Safety data has accumulated over the years. The aim of this article is to review and update the adverse effects of oral NAs. NAs can cause class adverse effects (<i>i.e</i>., myopathy, neuropathy, lactic acidosis) and dissimilar adverse effects. All NAs carry a "Black Box" warning because of the potential risk for mitochondrial dysfunction. However, these adverse effects are rarely reported. The majority of... (truncated)

Title: [Hepatotoxicity of chemotherapy].
Patients who will receive chemotherapy require careful assessment of liver function prior to treatment to determine which drugs are not appropriate, and which drug need dose modification. Many medications, alcohol has their more or less hepatotoxic effect, so because of their immunocompromise state, some body are prone to liver infection, including viral hepatitis. Lamivudine is effective in treating hepatitis B reactivation during chemotherapy. Now, the increased use of high-dose regimens with bone marrow or stem-cell support has been shown the drugs toxicities complication more, which does not observed in with conventional doses. Clinical judgment and a high index of suspicion... (truncated)

Title: Lamivudine in the treatment of hepatitis B virus reactivation during cytotoxic chemotherapy.
Hepatitis B virus (HBV) reactivation has been described in cancer patients who received cytotoxic/immunosuppressive therapy and may result in liver damage of varying degrees of severity. There is no known effective treatment. Lamivudine, a nucleoside analogue, has been found to suppress HBV replication and to improve histology in chronic carriers of hepatitis B virus. The outcome of lamivudine therapy (at doses of 100 or 150 mg/day) in eight patients who developed HBV reactivation while receiving cytotoxic chemotherapy is described. Each of the eight patients had >98% suppression of the pretreatment HBV DNA levels. Three of the five patients who were... (truncated)

MESH:C406224 - valdecoxib

Summary:

LLM Prediction Score	0.408 (normalized)
LLM Confidence Score	0.960
Golden Answer (Severity Class)	0.375 (normalized)
Prediction Error	0.033

Retrieved Context:

Title: Tolerability of selective cyclooxygenase 2 inhibitors used for the treatment of rheumatological manifestations of inflammatory bowel disease.
Nonsteroidal anti-inflammatory drugs (NSAIDs) are used to reduce inflammatory pain and swelling in inflammatory bowel disease (IBD) patients with rheumatological manifestations. While these drugs effectively reduce musculoskeletal pain and stiffness, long-term use is limited by gastrointestinal (GI) adverse effects (AEs) and disease exacerbation. As an alternative to NSAIDs, selective cyclooxygenase 2 (COX-2) inhibitors were developed to improve GI safety and tolerability. COX-2 inhibitors include drugs such as celecoxib, rofecoxib, valdecoxib, etoricoxib, and lumiracoxib. Rofecoxib and valdecoxib have been withdrawn from the market worldwide due to safety concerns (most importantly for cardiovascular adverse events) and lumiracoxib has been withdrawn in many... (truncated)

Title: Selective COX-2 Inhibitors: Road from Success to Controversy and the Quest for Repurposing.
The introduction of selective COX-2 inhibitors (so-called 'coxibs') has demonstrated tremendous commercial success due to their claimed lower potential of serious gastrointestinal adverse effects than traditional NSAIDs. However, following the repeated questioning on safety concerns, the coxibs 'controversial me-too' saga increased substantially, inferring to the risk of cardiovascular complications, subsequently leading to the voluntary withdrawal of coxibs (e.g., rofecoxib and valdecoxib) from the market. For instance, the makers (Pfizer and Merck) had to allegedly settle individual claims of cardiovascular hazards from celecoxib and valdecoxib. Undoubtedly, the lessons drawn from this saga revealed the flaws in drug surveillance and regulation, and... (truncated)

Title: A turbulent decade for NSAIDs: update on current concepts of classification, epidemiology, comparative efficacy, and toxicity.
Non-steroidal anti-inflammatory drugs (NSAIDs) represent a diverse class of drugs and are among the most commonly used analgesics for arthritic pain worldwide, though long-term use is associated with a spectrum of adverse effects. The introduction of cyclooxygenase-2-selective NSAIDs early in the last decade offered an alternative to traditional NSAIDs with similar efficacy and improved gastrointestinal tolerability; however, emerging concerns about cardiovascular safety resulted in the withdrawal of two agents (rofecoxib and valdecoxib) in the mid-2000s and, subsequently, in an overall reduction in NSAID use. It is now understood that all NSAIDs are associated with some varying degree of gastrointestinal and... (truncated)

Title: Clinical pharmacology of lumiracoxib, a second-generation cyclooxygenase 2 selective inhibitor.
Although highly selective cyclooxygenase (COX)-2 inhibitors have been shown to be less toxic to the gastrointestinal tract than conventional non-steroidal anti-inflammatory drugs (NSAIDs), their overall safety profile is questioned. Since different selective COX-2 inhibitors were found to be associated with increased cardiovascular thrombotic events, the thrombotic hazard may be a class effect. Furthermore, warnings have been issued regarding serious skin and hypersensitivity reactions associated with valdecoxib. Lumiracoxib is a novel COX-2 selective inhibitor (coxib) with improved biochemical selectivity over that of currently available coxibs. It is structurally distinct from other drugs in the class and has weakly acidic properties. Clinical... (truncated)

Title: The Cyclooxigenase-2 Inhibitor Parecoxib Prevents Epidermal Dysplasia in HPV16-Transgenic Mice: Efficacy and Safety Observations.
Carcinogenesis induced by high-risk human papillomavirus (HPV) involves inflammatory phenomena, partially mediated by cyclooxigenase-2. In pre-clinical models of HPV-induced cancer, cyclooxygenase-2 inhibitors have shown significant efficacy, but also considerable toxicity. This study addresses the chemopreventive effect and hepatic toxicity of a specific cyclooxigensase-2 inhibitor, parecoxib, in HPV16-transgenic mice. Forty-three 20 weeks-old female mice were divided into four groups: I (HPV16^{-/-}, <i>n</i> = 10, parecoxib-treated); II (HPV16^{-/-} <i>n</i> = 11, untreated); III (HPV16^{+/-}, <i>n</i> = 11, parecoxib-treated) and IV (HPV16^{+/-}, <i>n</i> = 11,

MESH:D013827 - thiabendazole

Summary:

LLM Prediction Score	0.907 (normalized)
LLM Confidence Score	0.970
Golden Answer (Severity Class)	0.875 (normalized)
Prediction Error	0.032

Retrieved Context:

Title: [Hepatic transplantation for severe ductopenia related to ingestion of thiabendazole].
We report a case of severe cholestasis and sicca syndrome after thiabendazole administration for Strongyloides stercoralis infection in a 26-year-old patient. Liver biopsy, performed 15 days after the onset of jaundice, revealed a marked paucity of bile ducts, and cholestasis rapidly progressed to biliary cirrhosis. Because of the progression of jaundice and the development of esophageal varices, orthotopic liver transplantation was performed, 18 months after the beginning of disease. The mechanism responsible for thiabendazole-induced biliary injury is unknown. The association between sicca syndrome and biliary disease suggests an immunoallergic mechanism against an antigen which could be common to the biliary,... (truncated)

Title: The fungicide thiabendazole causes apoptosis in rat hepatocytes.
Many pharmaceutical drugs cause hepatotoxicity in humans leading to severe liver diseases, representing a serious public health issue. This study investigates the ability of the anthelmintic and antifungal drug thiabendazole to cause cell death by apoptosis and metabolic changes in primary cultures of rat hepatocytes. Thiabendazole (200-500 µM) induced apoptosis in hepatocytes after 1 to 24h, causing loss of mitochondrial membrane potential, cytochrome c release from mitochondria, Fas-associated death domain (FADD) translocation from the cytosol to membranes, and activation of caspases-3, -8 and -9. Thus, thiabendazole activated both the mitochondrial and death receptor pathways of apoptosis. Under these conditions, cell... (truncated)

Title: Hepatic Capillariasis- Drug Targets.
Zoonotic infections are increasingly becoming public health menaces and are usually transmitted to humans due to unsuitable environmental conditions. One of them is hepatic capillariasis, caused by the parasite Capillaria hepatica, primarily a disease of rodents, with hepatic manifestations in humans. Although its prevalence is very low, it can cause significant morbidity and mortality, with cases reported from all over the world. The main infective form for humans is the embryonated egg of the parasite, which hatches in the intestine and ultimately colonize the liver. The larvae mature and reproduce, and eventually form embryonated eggs, which cause chronic focal inflammation... (truncated)

Title: [Cholestatic hepatitis ascribed to the use of thiabendazole].
Two women, aged 27 and 42 years, both born in Surinam and both suffering from heterozygous thalassemia, developed cholestatic hepatitis three and two weeks respectively after the start of a two-day course of thiabendazol for Strongyloides stercoralis infection. Other causes of cholestasis were unlikely in view of the results of blood tests, echography and the endoscopic retrograde cholangiopancreaticography. The symptoms persisted for several months, and the liver function disorders for 7 years and one year, respectively. The incidence of thiabendazole-induced cholestatic hepatitis is unknown, but probably low.

Title: Hepatic capillariasis: first case report in the Republic of Korea.
We report a case of massive hepatic infection by Capillaria hepatica in a 14-month-old girl who presented with the symptom triad of persistent fever, hepatomegaly, and leukocytosis with eosinophilia. Twenty-five cases of human infection with this parasite, mostly in children, have been reported in the literature. This is the first case of hepatic capillariasis reported in the Republic of Korea. The diagnosis was made by needle biopsy of the liver. Scanning electron microscopic examination of the biopsy specimen was also performed. Thiabendazole therapy was initiated and the patient developed liver disease-related IgA nephropathy during the therapy. The literature dealing with... (truncated)

MESH:D050477 - urofollitropin

Summary:

LLM Prediction Score	0.032 (normalized)
LLM Confidence Score	0.950
Golden Answer (Severity Class)	0.0 (normalized)
Prediction Error	0.032

Retrieved Context:

Title: Design of Infertility Monitoring System: Minimum Data Set Approach.
Reproductive health is vital for human and infertility is also one of the most important challenges in the reproductive system. Infertility is one of the most common chronic health disorders, regardless of age. The Minimum Data Set (MDS) helps to manage infertility by monitoring and evaluating infertility interventions based on collecting data. The development of MDS is an essential objective in order to implement an infertility monitoring system for the creation of standardized and effective data management through the provision of comprehensive and identical data elements for infertility. This is a descriptive cross-sectional study conducted in 2017. The data has... (truncated)

Title: The effect of flexible low-dose GnRH antagonist on pregnancy outcome in the fresh embryo transfer cycle of IVF-ET: a randomized controlled trial.
To explore the practicality and effectiveness of a flexible low-dose protocol in the fresh embryo transfer cycle: reducing the total amount of antagonist by increasing the interval between administrations of Cetrotide.

Title: A Personalized Management Approach of OHSS: Development of a Multiphase Prediction Model and Smartphone-Based App.
This study aimed to develop multiphase big-data-based prediction models of ovarian hyperstimulation syndrome (OHSS) and a smartphone app for risk calculation and patients' self-monitoring.

Title: Molecular Characterization of a Rare Case of Monozygotic Dichorionic Diamniotic Twin Pregnancy after Single Blastocyst Transfer in Preimplantation Genetic Testing (PGT).
Preimplantation genetic testing (PGT) is widely used to select unaffected embryos, increasing the odds of having a healthy baby. During the last few decades, it was accepted that monozygotic dichorionic diamniotic twin pregnancies occurred from the embryo splitting before Day 3 postfertilization according to Corner's dogma. Hence, the occurrence of a dichorionic diamniotic twin pregnancy after a single blastocyst transfer was considered a dizygotic pregnancy resulting from blastocyst transfer and concurrent natural fertilization. In our study, we have provided for the first time molecular proof that a single blastocyst transfer can result in a monozygotic dichorionic diamniotic twin pregnancy, invalidating... (truncated)

MESH:D014311 - triprolidine

Summary:

LLM Prediction Score	0.032 (normalized)
LLM Confidence Score	0.910
Golden Answer (Severity Class)	0.0 (normalized)
Prediction Error	0.032

Retrieved Context:

Title: Potential Small Molecules for Therapy of Lupus Nephritis Based on Genetic Effect and Immune Infiltration.
Lupus nephritis (LN) is the most common and significant complication of systemic lupus erythematosus (SLE) due to its poor prognosis and mortality rates in SLE patients. There is a critical need for new drugs as the pathogenesis of LN remains to be elucidated and immunosuppressive therapy comes with many deficiencies. In this study, 23 hub genes (IFI6, PLSCR1, XAF1, IFI16, IFI44, MX1, IFI44L, IFIT3, IFIT2, IFI27, DDX58, EIF2AK2, IFITM1, RTP4, IFITM3, TRIM22, PARP12, IFIH1, OAS1, HERC6, RSAD2, DDX60, and MX2) were identified through bioinformatics and network analysis and are closely related to interferon production and function. Interestingly, immune cell infiltration... (truncated)

Title: Acute paracetamol poisonings received at the Oran University Hospital.
Paracetamol is the most commonly used drug worldwide for its analgesic/antipyretic effect and especially a non-prescription access in pharmacies. Acute Paracetamol poisoning remains problematic for clinicians because of its insidious progression to fulminant hepatitis and even death. This work proposes to draw up the epidemiological profile of acute Paracetamol poisonings.

Title: An in vitro coculture system of human peripheral blood mononuclear cells with hepatocellular carcinoma-derived cells for predicting drug-induced liver injury.
Preventing clinical drug-induced liver injury (DILI) remains a major challenge, because DILI develops via multifactorial mechanisms. Immune and inflammatory reactions are considered important mechanisms of DILI; however, biomarkers from in vitro systems using immune cells have not been comprehensively studied. The aims of this study were (1) to identify promising biomarker genes for predicting DILI in an in vitro coculture model of peripheral blood mononuclear cells (PBMCs) with a human liver cell line, and (2) to evaluate these genes as predictors of DILI using a panel of drugs with different clinical DILI risk. Transcriptome-wide analysis of PBMCs cocultured with HepG2... (truncated)

Title: Psychotropics and COVID-19: An analysis of safety and prophylaxis.
The use of psychotropics during the COVID-19 pandemic has raised two questions, in order of importance: first, what changes should be made to pharmacological treatments prescribed to mental health patients? Secondly, are there any positive side effects of these substances against SARS-CoV-2? Our aim was to analyze usage safety of psychotropics during COVID-19; therefore, herein, we have studied: (i) the risk of symptomatic complications of COVID-19 associated with the use of these drugs, notably central nervous system activity depression, QTc interval enlargement and infectious and thromboembolic complications; (ii) the risk of mistaking the iatrogenic impact of psychotropics with COVID-19 symptoms,... (truncated)

Title: H1 antihistamines: current status and future directions.
In this review, we compare and contrast the clinical pharmacology, efficacy, and safety of first-generation H1 antihistamines and second-generation H1 antihistamines. First-generation H1 antihistamines cross the blood-brain barrier, and in usual doses, they potentially cause sedation and impair cognitive function and psychomotor performance. These medications, some of which have been in use for more than 6 decades, have never been optimally investigated. Second-generation H1 antihistamines such as cetirizine, desloratadine, fexofenadine, levocetirizine, and loratadine cross the blood-brain barrier to a significantly smaller extent than their predecessors. The clinical pharmacology, efficacy, and safety of these medications have been extensively studied. They are... (truncated)

MESH:D003891 - desipramine

Summary:

LLM Prediction Score	0.407 (normalized)
LLM Confidence Score	0.970
Golden Answer (Severity Class)	0.375 (normalized)
Prediction Error	0.032

Retrieved Context:

Title: Development of a highly sensitive cytotoxicity assay system for CYP3A4-mediated metabolic activation.
Drug-induced hepatotoxicity, which is a rare but serious adverse reaction to a large number of pharmaceutical drugs, is sometimes associated with reactive metabolites produced by drug-metabolizing enzymes. In the present study, we constructed a cell-based system to evaluate the cytotoxicity of reactive metabolites produced by CYP3A4 using human hepatoma cells infected with an adenovirus vector expressing human CYP3A4 (AdCYP3A4). When seven hepatoma cell lines (HepG2, Hep3B, HLE, HLF, Huh6, Huh7, and Fa2N4 cells) were infected with AdCYP3A4, HepG2 cells showed the highest CYP3A4 protein expression and testosterone 6β-hydroxylase activity (670 pmol · min(-1) · mg(-1)). With the use of AdCYP3A4-infected... (truncated)

Title: Trazodone-induced hepatotoxicity: a case report with comments on drug-induced hepatotoxicity.
Trazodone (Desyrel) is a second-generation, nontricyclic antidepressant that has been in use in North America since the early 1980s. It has the advantage of being more sedating and having less anticholinergic side effects than other secondary amines in the piperazine class, namely, desipramine and nortriptyline. Five previous cases of trazodone hepatotoxicity have been reported in the literature, one describing chronic damage and the others, more acute cellular and cholestatic injury. We describe a case of acute reversible liver injury with the use of trazodone. This case is unique in that injury occurred after protracted (18 months) drug use and while... (truncated)

Title: Effects of desipramine on clinical liver function tests.
Results of liver function tests in 46 depressed patients changed little during treatment with desipramine and were uncorrelated with drug plasma levels. The findings suggest that tricyclic-associated hepatitis, rather than being dose dependent, is an uncommon, idiosyncratic phenomenon.

Title: Clearance (née Rowland) concepts: a downdate and an update.
A number of experimental observations in the late 1960s, early 1970s could not be explained by the pharmacokinetic theory available at that time. For example rats receiving phenobarbital as an enzyme inducing agent exhibited increased elimination of phenylbutazone in vitro in liver microsomes and in vivo in whole animals compared to that observed in non-induced animals. However, for desipramine, although phenobarbital increased elimination in microsomes, no change in plasma disappearance was noted in vivo for this drug between rats induced with phenobarbital and control rats. Similar in vitro-in vivo discordancies were seen with changes in protein binding. The introduction of... (truncated)

Title: Hypersensitivity myocarditis and hepatitis associated with imipramine and its metabolite, desipramine.
We present two cases of myocarditis and hepatitis with histologic characteristics of hypersensitivity-mediated drug reactions associated with imipramine and its metabolite, desipramine. In one case, death was directly attributed to myocarditis; in the second case, the patient died of an acute myocardial infarct, but myocarditis may have played a contributory role. One patient was taking imipramine, and therapeutic concentrations of imipramine and desipramine were documented in postmortem blood. The other patient was receiving desipramine documented by in-patient hospital medication records. Both cases had liver lesions associated in the medical literature with adverse drug reaction to imipramine. Although myocarditis has been... (truncated)

MESH:C052036 - alpidem

Summary:

LLM Prediction Score	0.968 (normalized)
LLM Confidence Score	0.740
Golden Answer (Severity Class)	1.0 (normalized)
Prediction Error	0.032

Retrieved Context:

Title: [Hepatitis induced by alpidem (Ananxyl). Four cases, one of them fatal].
Acute hepatitis related to psychotropic drugs has been rarely reported with anxiolytic treatments. Alpidem (Ananxyl) is a new imidazopyridine derivative with anxiolytic properties which was launched in October 1991. Several cases of acute hepatitis, some severe, were described with this drug and its liver toxicity was questioned. Results of a study monitoring drug side-effects caused this drug to be withdrawn from the market for one year. We report four cases of acute hepatitis and discuss the role of alpidem. All but one case were presented as severe acute hepatitis with hepatic failure. One patient ran a fulminant course and died... (truncated)

Title: [Hepatic tolerance of atypical antipsychotic drugs].
The strategy in the choice of antipsychotic agent must take into account the hepatic tolerance according to non-negligible incidence of liver disorders among psychiatric population (presence of risk factors like alcoholism, drugs of abuse intake, polymedication including potentially hepatotoxic drugs.). More than 1 000 drugs have been listed as being responsible of hepatic side effects; 16% of these agents were neuropsychiatric drugs. Antidepressive drugs (tricyclic agents or SSRI), mood stabilizing agents and neuroleptic drugs have been implicated in biological or/and clinical hepatotoxicity. For these reasons, some psychotropic agents have been withdrawn of the pharmaceutical market like alpidem or medifoxamine. Atrium*,... (truncated)

Title: Anxioselective anxiolytics: on a quest for the Holy Grail.
The discovery of benzodiazepine receptors provided the impetus to discover and develop anxioselective anxiolytics ('Valium without the side effects'). The market potential for an anxioselective based on the γ-aminobutyric acid A (GABA(A)) receptor resulted in clinical trials of multiple compounds. In contrast to the anxioselective profile displayed in preclinical models, compounds such as bretazenil, TPA023, and MRK 409 produced benzodiazepine-like side effects (sedation, dizziness) in Phase I studies, whereas alpidem and ocinaplon exhibited many of the characteristics of an anxioselective in the clinic. Alpidem was briefly marketed for the treatment of anxiety, but was withdrawn because of liver toxicity. Reversible... (truncated)

Title: Evaluation of the potential for drug-induced liver injury based on in vitro covalent binding to human liver proteins.
Prediction of idiosyncratic drug-induced liver injury (DILI) is difficult, and the underlying mechanisms are not fully understood. However, many drugs causing DILI are considered to form reactive metabolites and covalently bind to cellular macromolecules in the liver. The objective of this study was to clarify whether the risk of idiosyncratic DILI can be estimated by comparing in vitro covalent binding (CB) levels among 12 positive compounds (acetaminophen, alpidem, bromfenac, carbamazepine, diclofenac, flutamide, imipramine, nefazodone, tacrine, ticlopidine, tienilic acid, and troglitazone) for DILI and 12 negative compounds (acetylsalicylic acid, caffeine, dexamethasone, losartan, ibuprofen, paroxetine, pioglitazone, rosiglitazone, sertraline, theophylline, venlafaxine, and zolpidem).... (truncated)

Title: [A new generation of tranquilizing agents].
The newer anxiolytics include compounds whose the molecular structure, the mechanisms, and the pharmacological properties are heterogeneous. Nevertheless, the most of them have clinical and adverse effects like to the most known: buspirone, after the commercial shrinking for hepatic toxicity of alpidem. These anxiolytics are efficacious against generalized anxiety disorder, like the benzodiazepines. The principal interest consists in minimal adverse effects and the safety of the use. These compounds have not a sedative effect, do not induce rebound, dependence, abuse and withdrawal, do not impair the psychomotor, cognitive and memory performances. The big ratio efficacy/tolerance allows to use them in... (truncated)

MESH:D019354 - sodium lactate

Summary:

LLM Prediction Score	0.031 (normalized)
LLM Confidence Score	0.990
Golden Answer (Severity Class)	0.0 (normalized)
Prediction Error	0.031

Retrieved Context:

Title: Lactate as a myokine and exerkine: drivers and signals of physiology and metabolism.
No longer viewed as a metabolic waste product and cause of muscle fatigue, a contemporary view incorporates the roles of lactate in metabolism, sensing and signaling in normal as well as pathophysiological conditions. Lactate exists in millimolar concentrations in muscle, blood, and other tissues and can rise more than an order of magnitude as the result of increased production and clearance limitations. Lactate exerts its powerful driver-like influence by mass action, redox change, allosteric binding, and other mechanisms described in this article. Depending on the condition, such as during rest and exercise, following carbohydrate nutrition, injury, or pathology, lactate can... (truncated)

Title: Metabolism and Effects on Endogenous Metabolism of Paracetamol (Acetaminophen) in a Porcine Model of Liver Failure.
The metabolic fate, toxicity, and effects on endogenous metabolism of paracetamol (acetaminophen, APAP) in 22 female Landrace cross large white pigs were evaluated in a model of acute liver failure (ALF). Anesthetized pigs were initially dosed at 250 mg/kg via an oroduodenal tube with APAP serum concentrations maintained above 300 mg/l using maintenance doses of 0.5-4 g/h until ALF. Studies were undertaken to determine both the metabolic fate of APAP and its effects on the endogenous metabolic phenotype of ALF in using 1H NMR spectroscopy. Increased concentrations of citrate combined with pre-ALF increases in circulating lactate, pyruvate, and alanine in... (truncated)

Title: Half-molar sodium lactate infusion improves cardiac performance in acute heart failure: a pilot randomised controlled clinical trial.
Acute heart failure (AHF) is characterized by inadequate cardiac output (CO), congestive symptoms, poor peripheral perfusion and end-organ dysfunction. Treatment often includes a combination of diuretics, oxygen, positive pressure ventilation, inotropes and vasodilators or vasopressors. Lactate is a marker of illness severity but is also an important metabolic substrate for the myocardium at rest and during stress. We tested the effects of half-molar sodium lactate infusion on cardiac performance in AHF.

Title: The anti-inflammasome effect of lactate and the lactate GPR81-receptor in pancreatic and liver inflammation.
No abstract available.

Title: Lactate reduces liver and pancreatic injury in Toll-like receptor- and inflammasome-mediated inflammation via GPR81-mediated suppression of innate immunity.
The NACHT, LRR, and pyrin domain-containing protein 3 (NLRP3) inflammasome induces inflammation in response to organ injury, but little is known about its regulation. Toll-like receptors (TLRs) provide the first signal required for activation of the inflammasome and stimulate aerobic glycolysis to generate lactate. We examined whether lactate and the lactate receptor, Gi-protein-coupled receptor 81 (GPR81), regulate TLR induction of signal 1 and limit inflammasome activation and organ injury.

MESH:D016666 - fluvoxamine

Summary:

LLM Prediction Score	0.406 (normalized)
LLM Confidence Score	0.990
Golden Answer (Severity Class)	0.375 (normalized)
Prediction Error	0.031

Retrieved Context:

Title: Liver injury associated with antidepressants.
Antidepressants are commonly prescribed and used in the management of depression, anxiety disorders, and other psychiatric illnesses. Antidepressants used in therapeutic dosing ranges are associated with causing several adverse drug reactions including hepatotoxicity. Paroxetine, fluoxetine, fluvoxamine, citalopram, mirtazapine and venlafaxine are associated with reversible liver injury upon discontinuation of the agent. Patient cases of hepatotoxicity involving the use of nefazodone, trazodone, duloxetine, bupropion, and sertraline are linked to causing death in its users. Due to the idiosyncratic nature of hepatotoxicity, monitoring of liver function tests and immediate discontinuation upon abnormal lab findings or signs and symptoms of liver dysfunction are... (truncated)

Title: The effect of different antidepressant drugs on oxidative stress after lipopolysaccharide administration in mice.
This study investigated the effect of the serotonin selective reuptake inhibitors (SSRIs) fluoxetine, sertraline, fluvoxamine and the tricyclic antidepressant (TCA) impiramine on oxidative stress in brain and liver induced by lipopolysaccharide administration in mice. Each drug was administered subcutaneously at doses of 10 or 20 mg/kg, for two days prior to intraperitoneal (i.p.) administration of lipopolysaccharide E (LPS: 200 µg/kg). Mice were euthanized 4 h after administration of the lipopolysaccharide. Lipid peroxidation (malondialdehyde; MDA), reduced glutathione (GSH) and nitric oxide (nitrite/nitrate) concentrations were measured in brain and liver. <i>Results:</i> The administration of lipopolysaccharide increased oxidative stress in brain and liver;... (truncated)

Title: Tumor necrosis factor-alpha, prostaglandin-E2 and interleukin-1β targeted anti-arthritic potential of fluvoxamine: drug repurposing.
Fluvoxamine, a selective serotonin re uptake inhibitor, is used to treat depression. The aim of present study was to evaluate fluvoxamine in acute (egg albumin-induced inflammation) and chronic inflammatory rat models (formaldehyde and complete Freund's adjuvant (CFA)-induced arthritis). Fluvoxamine showed highly significant (p<0.001) protective effect at dose of 50 mg/kg orally with percentage suppression 21.3% as compared to disease control group in acute model. Likewise, formaldehyde-induced arthritic experiment confirmed the significant (p<0.001) anti-arthritic behavior, showed by fluvoxamine (50 mg/kg orally) throughout the study. Moreover, In CFA-induced model, the higher dose (fluvoxamine 50 mg/kg) exhibited highly significant (p<0.001) decrease in paw... (truncated)

Title: Antidepressants and Hepatotoxicity: A Cohort Study among 5 Million Individuals Registered in the French National Health Insurance Database.
Hepatotoxicity may be a concern when prescribing antidepressants. Nevertheless, this risk remains poorly understood for serotonin and noradrenaline reuptake inhibitors (SNRIs: venlafaxine, milnacipran, duloxetine) and 'other antidepressants' (mianserin, mirtazapine, tianeptine and agomelatine), particularly in comparison with selective serotonin reuptake inhibitors (SSRIs: fluoxetine, citalopram, paroxetine, sertraline, fluvoxamine, escitalopram), which are by far the most commonly prescribed antidepressants.

Title: Fluvoxamine ameliorates oxidative stress and inflammation induced by bile-duct ligation in male rats.
Cholestasis is a disorder that the bile ducts were narrowed and bile acids are not released simply. Bile acids-induced liver damage is exacerbated by inflammation and oxidative stress. The goal of the current study was to investigate the protective impacts of fluvoxamine (Flu) on oxidant-antioxidant balance and inflammatory cytokines in the bile duct ligated (BDL) rats.

MESH:D001058 - apomorphine

Summary:

LLM Prediction Score	0.029 (normalized)
LLM Confidence Score	0.980
Golden Answer (Severity Class)	0.0 (normalized)
Prediction Error	0.029

Retrieved Context:

Title: The effects of apomorphine on paracetamol-induced hepatotoxicity in rats.
It is becoming progressively more understandable that overdose of paracetamol in both humans and animals causes severe hepatotoxicity. Apomorphine is known as a neuroprotective agent. Due to the protective effect, apomorphine had been tested in experimental studies on different models. Findings obtained through series of expriments suggested that apomorphine may also be useful in liver toxicity. The aim of this study is to investigate the relationship among the hepatoprotective mechanism of apomorphine and to determine the possible role of apomorphine on paracetamol-induced hepatotoxicity in rats. 30 Sprague Dawley rats (adult male) were distributed into 5 groups. Group 1 was the... (truncated)

Title: Sub-chronic copper pretreatment reduces oxidative damage in an experimental Huntington's disease model.
Quinolinic acid (QUIN) striatal injection in rat reproduces the main neurochemical features of Huntington's disease (HD), including oxidative damage. In this study, we evaluated the effect of a copper (Cu) supplement in drinking water (90 ppm Cu, 28 days) on the QUIN-induced HD model in the rat. Copper exposure caused no signs of liver toxicity; however, it produced significant Cu accumulation in striatum. It is noteworthy that QUIN also caused increased striatal Cu content; when the supplement was administered to animals with QUIN-injury, an even higher metal striatal accumulation was observed. Cu pre-treatment preserved striatal gamma-aminobutyric acid (GABA) content, which... (truncated)

Title: Off-time Treatment Options for Parkinson's Disease.
Motor fluctuations (MF) are deemed by patients with Parkinson's disease (PD) as the most troublesome disease feature resulting from the increasing impairment in responsiveness to dopaminergic drug treatments. MF are characterized by the loss of a stable response to levodopa over the nychthemeron with the reappearance of motor (and non-motor) parkinsonian clinical signs at various moments during the day and night. They normally appear after a few years of levodopa treatment and with a variable, though overall increasing severity, over the disease course. The armamentarium of first-line treatment options has widened in the last decade with new once-a-daily compounds, including... (truncated)

Title: Methamphetamine-induced toxicity: an updated review on issues related to hyperthermia.
Reports of methamphetamine-related emergency room visits suggest that elevated body temperature is a universal presenting symptom, with lethal overdoses generally associated with extreme hyperthermia. This review summarizes the available information on methamphetamine toxicity as it pertains to elevations in body temperature. First, a brief overview of thermoregulatory mechanisms is presented. Next, central and peripheral targets that have been considered for potential involvement in methamphetamine hyperthermia are discussed. Finally, future areas of investigation are proposed, as further studies are needed to provide greater insight into the mechanisms that mediate the alterations in body temperature elicited by methamphetamine.

Title: Cocaine-Induced Time-Dependent Alterations in Cytochrome P450 and Liver Function.
Cytochrome P450 is responsible for the metabolism of endogenous substrates, drugs and substances of abuse. The brain and nervous system regulate liver cytochrome P450 via neuroendocrine mechanisms, as shown in rodents. Cocaine exerts its addictive effects through the dopaminergic system, the functioning of which undergoes changes during its continuous use. Therefore, it can be hypothesized that the regulation of cytochrome P450 by cocaine may also alter during the addiction process, cessation and relapse. We analyzed preclinical studies on the mechanisms of the pharmacological action of cocaine, the role of the brain's dopaminergic system in the neuroendocrine regulation of cytochrome P450... (truncated)

MESH:D002437 - cefazolin

Summary:

LLM Prediction Score	0.346 (normalized)
LLM Confidence Score	0.980
Golden Answer (Severity Class)	0.375 (normalized)
Prediction Error	0.029

Retrieved Context:

Title: Drug-induced liver injury due to antibiotics.
Drug-induced liver injury (DILI) is an important differential diagnosis in patients with abnormal liver tests and normal hepatobiliary imaging. Of all known liver diseases, the diagnosis of DILI is probably one of the most difficult one to be established. In all major studies on DILI, antibiotics are the most common type of drugs that have been reported. The clinical phenotype of different types of antibiotics associated with liver injury is highly variable. Some widely used antibiotics such as amoxicillin-clavulanate have been shown to have a delayed onset on liver injury and recently cefazolin has been found to lead to liver... (truncated)

Title: Severe hepatitis associated with oxacillin therapy.
A 6-year-old girl had fever, abdominal pain, and severe anicteric hepatitis during intravenous oxacillin therapy for staphylococcal osteomyelitis. She had greatly elevated liver enzymes, prolonged prothrombin time, leukopenia, and eosinophilia. Clinical symptoms resolved and laboratory data returned to normal after withdrawing oxacillin and substituting cefazolin. This hepatotoxicity appears to be specific to oxacillin and not to other beta-lactams. Monitoring liver function tests during oxacillin therapy, especially in patients receiving prolonged treatment, may be warranted.

Title: Antistaphylococcal penicillins vs. cefazolin in the treatment of methicillin-susceptible Staphylococcus aureus infective endocarditis: a quasi-experimental monocentre study.
Whether cefazolin is as effective and safer than antistaphylococcal penicillins (ASPs) for the treatment of methicillin-susceptible Staphylococcus aureus (MSSA) infective endocarditis (IE) is still debated in the absence of a randomized controlled trial. In this quasi-experimental study, we aimed to assess the effectiveness and safety of these two treatments in MSSA-IE, using the ASPs nationwide shortage in April 2016 as a unique opportunity to overcome the indication bias associated with observational studies. In this single-centre study, we compared patients with Duke-Li definite MSSA-IE treated with ASPs from January 2015 to March 2016 versus those treated with cefazolin from April 2016... (truncated)

Title: Drug-Induced Liver Injury: Highlights from a Review of the 2015 Literature.
Numerous publications contributed to the expanding knowledge base about drug-induced liver injury (DILI) in 2015. New findings from the US Drug Induced Liver Injury Network (DILIN) in their most recently updated registry include a 1- to 3-week delay in the appearance of acute DILI from short-course antibiotics such as cefazolin. They corroborated the finding that acute DILI in patients with underlying liver disease was far more severe and potentially fatal than in patients without liver disease. The only drug that seemed to have an increased risk of hepatotoxicity in these patients was azithromycin. While nearly one in six patients with... (truncated)

Title: Severe Drug-Induced Liver Injury as an Adverse Drug Event of Antibiotics: A Case Report and Review of the Literature.
Drug-induced liver injury is one of the main reasons for acute liver failure. We report the case of a young patient who experienced a drug-induced liver injury resulting in life-threatening acute liver failure after treatment with different antibiotics (amoxicillin, ciprofloxacin, cefazolin, clindamycin) and acetaminophen, or a combination of these drugs. Moreover, we provide an overview of the hepatotoxic potential of these drugs.

MESH:D011241 - prednisone

Summary:

LLM Prediction Score	0.402 (normalized)
LLM Confidence Score	0.990
Golden Answer (Severity Class)	0.375 (normalized)
Prediction Error	0.027

Retrieved Context:

Title: [Hepatotoxicity and pancreatitis associated with gold salts: case report].
The case of a patient, 37 years old, born and resident of Lima, suffering rheumatoid arthritis who underwent treatment with prednisone, methotrexate, and chloroquine is reported. This therapy was substituted for gold salts one month before her admission. After the third dose she presented symptoms of abdominal pain and diarrhea, itching, and jaundice, associated with asthenia and a feverish sensation. Liver biochemistry demonstrated elevated transaminase, bilirubin, alkaline phosphatase, eosinophilia, inversion of the rate albumin/globulin, higher titer of immunoglobulin G, as well as an elevation of amylase and lipase. The anatomopathological study showed cholestasis, hepatocyte ballooning, spotty necrosis, predominantly in zone... (truncated)

Title: Deflazacort for type-1 autoimmune hepatitis in a Korean girl.
Prednisone or prednisolone are the mainstay drug treatments for autoimmune hepatitis in children. However, long-term use of corticosteroid is associated with the risk of steroid-induced toxicities, and this situation requires newer immuno-suppressive agents for the treatment of autoimmune hepatitis, especially in growing children. An 11-yr-old Korean girl with type-1 autoimmune hepatitis discontinued prednisolone due to toxicities, i.e., hirsutism, buffalo hump, and skin striae, and remained clinical and biochemical remission under replacement of deflazacort and ursodeoxycholic acid combination therapy. A follow-up liver biopsy after 19 months of deflazacort and ursodeoxycholic acid treatment showed histologic remission.

Title: Alcoholic hepatitis superimposed on cirrhosis. Clinical significance and effect of long-term prednisone treatment.
Of 483 patients with biopsy-verified cirrhosis who entered the controlled trial of prednisone treatment conducted by the Copenhagen Study Group for Liver Diseases, 99 had alcoholic hepatitis as defined by morphological criteria in the initial biopsy. The maximum period of observation varied from 5 to 12 years. The survival of prednisone-treated patients (median survival time 38 months) and placebo-treated patients (median survival time 34 months) with alcoholic hepatitis did not differ significantly. This was also true for each of the two subgroups comprising mild and severe cases respectively. The presence of morphologically defined alcoholic hepatitis was postively correlated with the... (truncated)

Title: Fatal varicella hepatitis in an asthmatic adult after short-term corticosteroid treatment.
A 28-year-old male patient, treated with prednisone for bronchitis with sibilant rales, developed fever with abdominal pain and generalized vesicular rash after coming in contact with varicella-infected children. He was hospitalized after having a seizure. Laboratory values revealed hepatitis and rapidly fulminant hepatic insufficiency with disseminated intravascular coagulation. Despite acyclovir treatment, the patient died 4 days after admission. Clinical presentation could evoke a Reye's syndrome, but liver biopsy showed massive coagulative necrosis. This report demonstrates the increased risk of complicated varicella associated with the use of corticosteroids, even for a short period of time.

Title: Liver Injury Associated With Ezetimibe Monotherapy.
Statin intolerance, primarily myalgia, is not uncommon in patients treated for elevated low-density lipoprotein cholesterol. Nonstatin drugs, such as ezetimibe, can spare patients from statin exposure, while still reducing low-density lipoprotein cholesterol. Ezetimibe is generally very well tolerated, although gastrointestinal and musculoskeletal symptoms have been occasionally reported. We describe an extremely rare case of an ezetimibe-associated liver injury who required protracted treatment with prednisone and azathioprine. Ezetimibe-associated liver injury should be suspected with development of hepatic abnormalities concurrent with the timing of ezetimibe treatment and in the absence of other possible precipitating factors.

MESH:D003915 - dextromethorphan

Summary:

LLM Prediction Score	0.027 (normalized)
LLM Confidence Score	0.990
Golden Answer (Severity Class)	0.0 (normalized)
Prediction Error	0.027

Retrieved Context:

Title: Amitriptyline-induced prolonged cholestasis.
We report the case of a patient in whom amitriptyline administration for 5 wk was followed by prolonged cholestasis. Jaundice and pruritus lasted 19 and 20 mo, respectively. Three liver biopsies were performed at different stages of the disease showing the course of liver lesions. Cholestasis initially located in the region of the hepatic venule came to be associated with the progressive development of portal tract lesions consisting of inflammatory infiltration, fibrosis, and disappearance of interlobular bile ducts. Amitriptyline hydroxylation and dextromethorphan O-demethylation are deficient in subjects with the poor metabolizer phenotype of debrisoquine. Drug oxidation phenotyping with dextromethorphan showed... (truncated)

Title: The MAP2K4/JNK/c-Jun Signaling Pathway Plays A Key Role In Dexmedetomidine Protection Against Acetaminophen-Induced Liver Toxicity.
Label="PURPOSE" NlmCategory="OBJECTIVE">Dexmedetomidine [DEX; (S)-4-[1-(2,3-dimethylphenyl)ethyl]-3H-imidazole] is a selective α₂-adrenergic receptor (α₂-AR) agonist that attenuates the liver damage associated with local or systemic inflammation. However, it remains unclear whether DEX has protective effects against acetaminophen (Paracetamol, PARA)-induced liver toxicity (PILT).

Title: Ultralow doses of dextromethorphan protect mice from endotoxin-induced sepsis-like hepatotoxicity.
Dextromethorphan, a wildly used over-the-counter antitussive drug, is reported to have anti-inflammatory effects. Previously, we and others have demonstrated that dextromethorphan at micromolar doses displays potent hepatoprotective effects and enhances mice survival in a sepsis model. Moreover, we also observed potent anti-inflammatory and neuroprotective effects of subpicomolar concentrations of dextromethorphan in rodent primary neuron-glial cultures. The purpose of this study was to provide a proof of principle that ultralow dose dextromethorphan displays anti-inflammatory and cytoprotective effects in animal studies. Here, we report that subpico- and micromolar doses of dextromethorphan showed comparable efficacy in protecting mice from lipopolysaccharide/d-galactosamine (LPS/GalN)-induced hepatotoxicity and... (truncated)

Title: Dextromethorphan improves locomotor activity and decreases brain oxidative stress and inflammation in an animal model of acute liver failure.
Hepatic encephalopathy (HE) is a serious clinical problem leading to severe neurological disorders and death. No specific treatment is available for the management of HE-associated neurological damage. This study aimed to evaluate the effect of dextromethorphan (DXM) on oxidative stress and disturbed locomotor activity in an animal model of HE.

Title: Dextromethorphan inhibits activations and functions in dendritic cells.
Dendritic cells (DCs) play an important role in connecting innate and adaptive immunity. Thus, DCs have been regarded as a major target for the development of immunomodulators. In this study, we examined the effect of dextromethorphan (DXM), a common cough suppressant with a high safety profile, on the activation and function of DCs. In the presence of DXM, the LPS-induced expression of the costimulatory molecules in murine bone marrow-derived dendritic cells (BMDCs) was significantly suppressed. In addition, DXM treatment reduced the production of reactive oxygen species (ROS), proinflammatory cytokines, and chemokines in maturing BMDCs that were activated by LPS. Therefore,... (truncated)

MESH:D014580 - ursodeoxycholic acid

Summary:

LLM Prediction Score	0.027 (normalized)
LLM Confidence Score	0.980
Golden Answer (Severity Class)	0.0 (normalized)
Prediction Error	0.027

Retrieved Context:

Title: Efficacy and safety of ursodeoxycholic acid composite on fatigued patients with elevated liver function and/or fatty liver: a multi-centre, randomised, double-blinded, placebo-controlled trial.
The aim of this study was to assess the effects of ursodeoxycholic acid composite (URSA-S) on fatigue in patients with elevated liver function tests and/or fatty liver disease.

Title: The effect of ursodeoxycholic acid in liver functional restoration of patients with obstructive jaundice after endoscopic treatment: a prospective, randomized, and controlled study.
In patients with obstructive jaundice, multi-organ dysfunction may develop.

Title: Ursodeoxycholic acid for the treatment of primary sclerosing cholangitis: a 30-month pilot study.
We investigated the effects of once-daily oral administration of 10 mg/kg ursodeoxycholic acid (generic name, ursodiol) on elevated serum enzyme activities, bilirubin, cholesterol, bile acids and symptoms in patients with primary sclerosing cholangitis. A 30-mo, open-label, pilot trial was designed to cover four periods: (a) 3 mo of pretreatment observation (period 1), (b) 6 mo on ursodiol (period 2), (c) 3 mo withdrawal of treatment (period 3) and (d) 18 mo of extended retreatment (period 4). Diagnosis was confirmed by cholangiography and liver biopsy specimens. We enrolled 12 patients with persistently elevated pretreatment alkaline phosphatase and gamma-glutamyltransferase levels (at least... (truncated)

Title: Flutamide-induced toxic hepatitis. Potential utility of ursodeoxycholic acid administration in toxic hepatitis.
Flutamide is a nonsteroidal antiandrogen commonly used in the treatment of prostate cancer. Hepatic toxicity associated with flutamide has been reported with an incidence from less than 1% to about 5%. Ursodeoxycholic acid (UDCA), a hydrophilic bile acid, has been widely used in the treatment of cholesterol gallstones and of several liver diseases, but few data are now available concerning its use in the management of drug-induced hepatitis. The case of a patient who presented severe hepatitis with jaundice following use of flutamide is reported. UDCA treatment was started on admission and, contemporaneously, flutamide was withdrawn. Clinical and biochemical improvement... (truncated)

Title: Ursodeoxycholic acid in chronic liver disease.
The hydrophilic bile acid ursodeoxycholic acid has recently been shown to reduce biochemical markers of both cholestasis and hepatocellular damage in patients with chronic liver diseases. The most compelling evidence available is for chronic cholestatic liver diseases, in particular primary biliary cirrhosis, primary sclerosing cholangitis, and cholestasis associated with cystic fibrosis. The effects may be less beneficial in patients with advanced liver disease from these conditions. Data from placebo controlled trials are now available in support of earlier uncontrolled observations, but it is not yet clear whether short term benefit results in an improvement in longterm prognosis. The mechanism of... (truncated)

MESH:D000077583 - quinapril

Summary:

LLM Prediction Score	0.348 (normalized)
LLM Confidence Score	0.950
Golden Answer (Severity Class)	0.375 (normalized)
Prediction Error	0.027

Retrieved Context:

Title: [Delirium attributed to the use of metoprolol].
A 53-year-old man with a known history of alcohol abuse was admitted to hospital after a minor collapse. He had a laceration to the forehead and three rib fractures. Laboratory blood-analysis showed raised non-cholestatic liver-enzyme levels suggesting alcohol-abuse. On history taking the patient was shown to have been suffering from personality changes and multiple hallucinogenic episodes for the previous two years. He had been seen and evaluated by a neurologist to that effect. The patient's family had accepted the situation and thought of it as dementia, probably caused by alcohol abuse. He had been treated for atrial flutter and was... (truncated)

Title: [Liver cirrhosis and granulomatous hepatitis after prolonged ingestion of lysine acetylsalicylate].
We report the case of a 75-year-old woman who was hospitalized for serum aminotransferase elevation. She had taken 1 g/d of salicylate therapy (Aspegic 1000) for 5 years, clorazepate for 10 years, naftidrofuryl for 8 months and quinapril for 3 months. Liver histology showed cirrhosis and granulomatous hepatitis. Discontinuation of salicylate therapy resulted in normalization of liver tests, although other drug administration was pursued. This observation suggests that salicylate therapy can induce cirrhosis.

Title: Case report of progressive renal dysfunction as a consequence of amiodarone-induced phospholipidosis.
Amiodarone is associated with a range of unwanted effects on pulmonary, thyroid, and liver function. However, the nephrotoxic side effect caused by renal phospholipidosis has hardly received any attention up to now.

Title: Unique case of presumed lisinopril-induced hepatotoxicity.
A case of presumed lisinopril-induced hepatotoxicity is reported.

Title: Postinfantile Giant Cell Hepatitis with Features of Acute Severe Autoimmune Hepatitis Probably Triggered by Diclofenac in a Patient with Primary Myelofibrosis.
Giant cell hepatitis (GCH) is commonly reported in neonatal and infantile liver diseases but rarely in adults where the term postinfantile GCH (PIGCH) is used. PIGCH is associated with many diseases, including drugs toxicity, viruses, and autoimmune liver diseases, with autoimmune hepatitis (AIH) being the most prevalent. We present a case of PIGCH in a 76-year-old female without known history of liver disease who suffered from an acute severe episode of hepatitis. After careful exclusion of other hepatitis causes by imaging, virological, immunological, and microbiological investigations, a diagnosis of acute severe AIH (AS-AIH) was established. The patient was started on... (truncated)

MESH:D000082 - acetaminophen

Summary:

LLM Prediction Score	0.598 (normalized)
LLM Confidence Score	1.000
Golden Answer (Severity Class)	0.625 (normalized)
Prediction Error	0.027

Retrieved Context:

Title: Hepatotoxicity in acetaminophen poisoning.
Evidence of severe liver damage was found in a young women who ingested an overdose of acetaminophen in a suicide attempt. Clinical features included prolonged nausea and vomiting and pain and tenderness over the liver. Biochemical findings included an SGOT value of over 3,000 and a prolonged prothrombin time, with normal alkaline phosphatase and near normal serum bilirubin. Spontaneous recovery was complete.

Title: Hepatotoxicity following the therapeutic use of antipyretic analgesics.
The potential for hepatic injury associated with the therapeutic use of salicylates and acetaminophen has recently attracted considerable attention. About 300 cases have been reported in which elevated transaminase levels or other evidence of hepatic injury developed following treatment with salicylates. Review of the spectrum of abnormalities reveals a group of patients (4 percent) with symptomatic liver damage in whom progressive or chronic liver disease is a possibility with continued use of the drug. In a few patients in this group, jaundice developed; several had abnormal prothrombin times; 11 (70 percent) had transaminase values in excess of 500 units; and... (truncated)

Title: Acetaminophen safety and hepatotoxicity--where do we go from here?
Acetaminophen has been widely used for > 50 years in the treatment of pain and fever and provides for the safe and effective relief of these symptoms. In a small minority of patients, however, acetaminophen is responsible for life-threatening liver injury and accounts for up to 50% of all adult cases of acute liver failure in the US. Although approximately two-thirds of adult overdoses are associated with suicide attempts, many are inadvertent, often due to the use of multiple acetaminophen formulations over many days. Additionally, some individuals appear to experience acetaminophen toxicity at 'therapeutic' doses of < 4 g/day, for... (truncated)

Title: Severe liver damage caused by therapeutic doses of acetaminophen.
Two patients who ingested therapeutic doses of acetaminophen and who developed a severe hepatotoxicity are reported. Both patients had a rather high alcohol consumption, which probably made them susceptible to the toxic effects of acetaminophen.

Title: Liver injury induced by paracetamol and challenges associated with intentional and unintentional use.
Drug induced liver injury (DILI) is a common cause of acute liver injury. Paracetamol, also known as acetaminophen, is a widely used anti-pyretic that has long been established to cause liver toxicity once above therapeutic levels. Hepatotoxicity from paracetamol overdose, whether intentional or non-intentional, is the most common cause of DILI in the United States and remains a global issue. Given the increased prevalence of combination medications in the form of pain relievers and antihistamines, paracetamol can be difficult to identify and remains a significant cause of acute hepatotoxicity, as evidenced by its contribution to over half of all acute... (truncated)

MESH:D000068756 - valsartan

Summary:

LLM Prediction Score	0.402 (normalized)
LLM Confidence Score	0.990
Golden Answer (Severity Class)	0.375 (normalized)
Prediction Error	0.027

Retrieved Context:

Title: Sacubitril/valsartan-induced liver injury: A case report and literature review.
Sacubitril/valsartan (Entresto) is the first drug approved for the treatment of symptomatic chronic heart failure with reduced ejection fraction in adult patients. There have been no reports of hepatotoxicity secondary to sacubitril/valsartan administration. Here, we report the first case of severe liver injury caused by sacubitril/valsartan.

Title: [Biliary stenosis, hepatitis A and valsartan: multifactorial hepatotoxicity in a surgical patient].
We present the case of a male patient with a history of hypertension who developed acute lithiasic cholecystitis. Shortly before the patient's admission, valsartan was prescribed to control his blood pressure. Surgery was performed without complications, but during the immediate postoperative period he developed marked jaundice, light colored stools, dark urine, and malaise. A cholangiogram showed a mild stricture of the common bile duct, and laboratory analyses revealed IgM antibodies against hepatitis A virus. Moreover, the patient reported the he continued to take valsartan. The patient improved with conservative treatment without any further complaints. In patients with jaundice after biliary... (truncated)

Title: Renoprotective effects of stevia (<i>Stevia rebaudiana</i> Bertoni), amlodipine, valsartan, and losartan in gentamycin-induced nephrotoxicity in the rat model: Biochemical, hematological and histological approaches.
The current study investigated the renoprotective effects of stevia, angiotensin-II type 1 receptor (AT₁) blocker and calcium (Ca²⁺) channel blocker in gentamycin-induced nephrotoxicity in rat models. Six groups of male Sprague-Dawley rats of eight weeks old were taken for the experiment: sham control, nephrotoxicity, treatment with amlodipine (4 mg/kg/day); stevia (200 mg/kg/day); losartan (15 mg/kg/day) and valsartan (5 mg/kg/day), accordingly. The blood sample was taken for the assessment of renal and hepatic-functional variables like serum creatinine, blood urea, BUN and SGPT, SGOT, and total serum bilirubin. Hematological parameters were also examined. Histological examination has been done on kidneys and liver. Alterations of the body... (truncated)

Title: Valsartan-induced hepatotoxicity in a HBs-Ag-Positive patient.
No abstract available.

Title: Angiotensin II receptor Neprilysin inhibitor (LCZ696) compared to Valsartan attenuates Hepatotoxicity in STZ-induced hyperglycemic rats.
Background and objectives: Although diabetic-induced hepatotoxicity is less common, it can be included in the list of target organ pathologies associated with diabetes. This study aimed to investigate the potential therapeutic role of sacubitril/valsartan (LCZ696) in modulating oxidative and inflammatory injuries and liver fibrosis in STZ-induced hyperglycemic rats in comparison to valsartan alone. Materials and Methods: Following the induction of diabetes using a single dose of streptozotocin (STZ), STZ-induced hyperglycemic animals were administered LCZ696 or valsartan for 6 weeks. Glucose, transaminases, lipid profile, tumor necrosis factor-alpha (TNF-α), interleukin 1 beta (IL-1β), and interleukin - 6 (IL-6), were estimated using the... (truncated)

MESH:D010404 - penicillin v

Summary:

LLM Prediction Score	0.027 (normalized)
LLM Confidence Score	0.990
Golden Answer (Severity Class)	0.0 (normalized)
Prediction Error	0.027

Retrieved Context:

Title: Identification of a metabolic biomarker panel in rats for prediction of acute and idiosyncratic hepatotoxicity.
It has been estimated that 10% of acute liver failure is due to "idiosyncratic hepatotoxicity". The inability to identify such compounds with classical preclinical markers of hepatotoxicity has driven the need to discover a mechanism-based biomarker panel for hepatotoxicity. Seven compounds were included in this study: two overt hepatotoxicants (acetaminophen and carbon tetrachloride), two idiosyncratic hepatotoxicants (felbamate and dantrolene), and three non-hepatotoxicants (meloxicam, penicillin and metformin). Male Sprague-Dawley rats were orally gavaged with a single dose of vehicle, low dose or high dose of the compounds. At 6 h and 24 h post-dosing, blood was collected for metabolomics and clinical... (truncated)

Title: Hepatic safety of antibiotics used in primary care.
Antibiotics used by general practitioners frequently appear in adverse-event reports of drug-induced hepatotoxicity. Most cases are idiosyncratic (the adverse reaction cannot be predicted from the drug's pharmacological profile or from pre-clinical toxicology tests) and occur via an immunological reaction or in response to the presence of hepatotoxic metabolites. With the exception of trovafloxacin and telithromycin (now severely restricted), hepatotoxicity crude incidence remains globally low but variable. Thus, amoxicillin/clavulanate and co-trimoxazole, as well as flucloxacillin, cause hepatotoxic reactions at rates that make them visible in general practice (cases are often isolated, may have a delayed onset, sometimes appear only after cessation... (truncated)

Title: Scarlet fever: a guide for general practitioners.
There has been an increase in the incidence of scarlet fever with most cases presenting in General Practice and Emergency Departments. Cases present with a distinctive macro-papular rash, usually in children. This article aims to increase awareness of scarlet fever by highlighting key symptoms and stating potential complications if untreated. In patients who have the typical symptoms, a prescription of a suitable antibiotic such as phenoxymethylpenicillin (Penicillin V) should be made immediately to reduce the risk of complications and the spread of infection.

Title: Therapeutic effect and metabolomics mechanism of <i>Patrinia Villosa</i> (Thunb.) juss on liver injury in rats.
Patrinia villosa (Thunb.) Juss (P.V) is widely used in the treatment of chronic diseases, such as appendicitis, enteritis and gynecological inflammation. Modern research indicated that the herb has pharmacological effect on liver injury caused by inflammation, but the metabolomics mechanism is not clear. For the purpose of discovering the therapeutic effect and metabolomic mechanism of P.V on liver injury, 40 Sprague-Dawley (SD) rats were divided into normal group, model group, and P.V groups (0.98, 1.97, and 2.96 g/kg). The model group and P.V groups were injected intraperitoneally with 40% CCl₄ (v/v, olive oil) to establish liver injury model. After administration of... (truncated)

Title: Patients with questionable penicillin (beta-lactam) allergy: Causes and solutions.
In Europe, North America, and Australia, 5% to 10% of the population are now classified as penicillin (β-lactam) allergic. Only ~ 10% of these questionable diagnoses, mostly made in childhood, can be confirmed by allergy diagnostics.

MESH:D005897 - glafenine

Summary:

LLM Prediction Score	0.973 (normalized)
LLM Confidence Score	0.720
Golden Answer (Severity Class)	1.0 (normalized)
Prediction Error	0.027

Retrieved Context:

Title: Bioactivation of glafenine by human liver microsomes and peroxidases: identification of electrophilic iminoquinone species and GSH conjugates.
Glafenine (Privadol; 2,3-dihydroxypropyl 2-[(7-chloro-4-quinolinyl) amino]benzoate) is a non-narcotic analgesic agent widely used for the treatment of pains of various origins. Severe liver toxicity and a high incidence of anaphylaxis were reported in patients treated with glafenine, eventually leading to its withdrawal from the market in most countries. It is proposed that bioactivation of glafenine and subsequent binding of reactive metabolite(s) to critical cellular proteins play a causative role. The study described herein aimed at characterizing pathways of glafenine bioactivation and the metabolic enzymes involved. Two GSH conjugates of glafenine were detected in human liver microsomal incubations using liquid chromatography tandem... (truncated)

Title: Glafenine-associated hepatic injury. Analysis of 38 cases and review of the literature.
Glafenine was associated with hepatic injury in 38 cases. The causal relationship was assessed on the basis of the temporal relationship with drug use, course and exclusion of other causes. In 27 cases a causal relationship was considered likely, i.e. 'probable' (12 cases) or 'possible' (15 cases), whereas in 11 cases it was either unlikely or unclassifiable. In both the 'probable' and 'possible' groups 60-70% of individuals were women. Jaundice was present in three-quarters of cases in both groups. Eosinophilia was more frequent in the group of 'probable' cases, and this group had the highest case-fatality rate (42%). Onset varied... (truncated)

Title: [Fatal liver cell necrosis following administration of glafenine].
The case is described of a patient with fulminant hepatic failure attributed to intake of glafenine 400 mg daily for 15 days. The first symptoms appeared two weeks after discontinuation of glafenine. Other causes of hepatic necrosis could be excluded. Approximately five weeks after the onset of symptoms the patient died of hepatic failure. At autopsy massive hepatic necrosis and massive pancreatic necrosis were demonstrated.

Title: A correlation between the in vitro drug toxicity of drugs to cell lines that express human P450s and their propensity to cause liver injury in humans.
Drug toxicity to T-antigen-immortalized human liver epithelial (THLE) cells stably transfected with plasmid vectors that encoded human cytochrome P450s 1A2, 2C9, 2C19, 2D6, or 3A4, or an empty plasmid vector (THLE-Null), was investigated. An automated screening platform, which included 1% dimethyl sulfoxide (DMSO) vehicle, 2.7% bovine serum in the culture medium, and assessed 3-(4,5-dimethylthiazol-2-yl)-5-(3-carboxymethoxyphenyl)-2-(4-sulfophenyl)-2H-tetrazolium reduction, was used to evaluate the cytotoxicity of 103 drugs after 24h. Twenty-two drugs caused cytotoxicity to THLE-Null cells, with $EC_{50} \leq 200$ μM; 21 of these drugs (95%) have been reported to cause human liver injury. Eleven drugs exhibited lower EC_{50} values in cells transfected... (truncated)

Title: The NSAID glafenine rescues class 2 CFTR mutants via cyclooxygenase 2 inhibition of the arachidonic acid pathway.
Most cases of cystic fibrosis (CF) are caused by class 2 mutations in the cystic fibrosis transmembrane regulator (CFTR). These proteins preserve some channel function but are retained in the endoplasmic reticulum (ER). Partial rescue of the most common CFTR class 2 mutant, F508del-CFTR, has been achieved through the development of pharmacological chaperones (Tezacaftor and Elexacaftor) that bind CFTR directly. However, it is not clear whether these drugs will rescue all class 2 CFTR mutants to a medically relevant level. We have previously shown that the nonsteroidal anti-inflammatory drug (NSAID) ibuprofen can correct F508del-CFTR trafficking. Here, we utilized RNAi and... (truncated)

MESH:D000077544 - panitumumab

Summary:

LLM Prediction Score	0.026 (normalized)
LLM Confidence Score	0.970
Golden Answer (Severity Class)	0.0 (normalized)
Prediction Error	0.026

Retrieved Context:

Title: Acute and long-term gastrointestinal consequences of chemotherapy.
There are many anti-neoplastic agents in cancer treatment. Combination regimens are often the main standard treatment, particularly for gastrointestinal malignancies. The introduction of new regimens that combine fluoropyrimidines with irinotecan or oxaliplatin and recently also with antibodies against the Epidermal Growth Factor Receptor (EGFR) (cetuximab and panitumumab) and against the Vascular Endothelial Growth Factor (VEGF) (bevacizumab) pathways, has dramatically improved the progression free survival and survival of patients with metastatic colorectal cancer. This rapid extension of available anti-neoplastic drugs has, however, also highlighted the urgent need for clinicians to better understand and identify the spectrum of acute and late toxicities... (truncated)

Title: Hepatitis C virus reactivation in cancer patients in the era of targeted therapies.
The purpose of this review is to summarize the evidence of hepatitis C reactivation in cancer patients in the era of targeted therapies. Targeted therapies are novel therapeutics frequently used in cancer patients. During treatment with targeted therapies, viral replication is one of the major problems that can occur. The PubMed database, ASCO, and ASCO Gastrointestinal Cancer Symposium abstracts were searched up until September 15, 2013 using the following search keywords: "targeted therapies, rituximab, alemtuzumab, brentuximab, hepatitis, hepatitis C reactivation, tyrosine kinase inhibitors, imatinib, mammalian target of rapamycin (mTOR) inhibitors, everolimus, anti-HER therapies, trastuzumab, pertuzumab, lapatinib, anti-epidermal growth factor receptor... (truncated)

Title: Co-administered antibody improves penetration of antibody-dye conjugate into human cancers with implications for antibody-drug conjugates.
Poor tissue penetration remains a major challenge for antibody-based therapeutics of solid tumors, but proper dosing can improve the tissue penetration and thus therapeutic efficacy of these biologics. Due to dose-limiting toxicity of the small molecule payload, antibody-drug conjugates (ADCs) are administered at a much lower dose than their parent antibodies, which further reduces tissue penetration. We conducted an early-phase clinical trial (NCT02415881) and previously reported the safety of an antibody-dye conjugate (panitumumab-IRDye800CW) as primary outcome. Here, we report a retrospective exploratory analysis of the trial to evaluate whether co-administration of an unconjugated antibody could improve the intratumoral distribution of... (truncated)

Title: Radioimmunotherapy of PANC-1 human pancreatic cancer xenografts in NOD/SCID or NRG mice with Panitumumab labeled with Auger electron emitting, ¹¹¹In or β-particle emitting, ¹⁷⁷Lu.
Label="BACKGROUND" NlmCategory="BACKGROUND">Epidermal growth factor receptors (EGFR) are overexpressed on > 90% of pancreatic cancers (PnCa) and represent an attractive target for the development of novel therapies, including radioimmunotherapy (RIT). Our aim was to study RIT of subcutaneous (s.c.) PANC-1 human PnCa xenografts in mice using the anti-EGFR monoclonal antibody, panitumumab labeled with Auger electron (AE)-emitting, ¹¹¹In or β-particle emitting, ¹⁷⁷Lu at amounts that were non-toxic to normal tissues.

Title: Hematochezia Due to Panitumumab-induced Colitis with Vitamin K Deficiency.
Panitumumab, a fully human anti-epidermal growth factor receptor (EGFR) monoclonal antibody, has been shown to be useful in treating either advanced or recurrent KRAS/NRAS/BRAF wild-type colorectal cancer. We herein report the case of a 60-year-old man with short bowel syndrome who developed hematochezia due to panitumumab-induced colitis with vitamin K deficiency during third-line chemotherapy. The cause of vitamin K deficiency was the lack of intravenous vitamin K supplementation following a change from central venous nutrition to peripheral venous nutrition. We advise clinicians to carefully check for colitis and manage the infusions of chemotherapy patients with short bowel syndrome.

MESH:C038628 - fipexide

Summary:

LLM Prediction Score	0.975 (normalized)
LLM Confidence Score	0.790
Golden Answer (Severity Class)	1.0 (normalized)
Prediction Error	0.025

Retrieved Context:

Title: Fipexide-induced fulminant hepatitis. Report of three cases with emergency liver transplantation.
Fipexide belongs to a new class of cognition activators and is noted for its lack of amphetamin-like side effects. We describe three patients who developed fulminant hepatic failure less than 2 months after beginning fipexide administration. The mean interval from the onset of jaundice to the onset of encephalopathy was 8 days. Emergency liver transplantation was undertaken when factor V was 20% of normal or less and coma developed. All patients were transplanted less than 1 week after the onset of encephalopathy. Two survived and one died immediately after transplantation. Histologic examination of the livers revealed massive liver cell necrosis,... (truncated)

Title: Palmitate increases the susceptibility of cells to drug-induced toxicity: an in vitro method to identify drugs with potential contraindications in patients with metabolic disease.
Fatty acids are an important source of energy. Excessive energy intake results in elevated levels of free fatty acids that are thought to be the pathogenic factors causing metabolic disorders such as dyslipidemia, obesity, insulin resistance, diabetes, and fatty liver. Underlying metabolic disorders have been suggested to be a predisposing factor for drug-induced liver injury. The steadily expanding population with metabolic disease may pose a higher risk for drug-induced toxicity. In order to understand the interaction of free fatty acids and drug-induced toxicity at the cellular level, we explored whether the saturated free fatty acid palmitate could modulate drug-induced cytotoxicity... (truncated)

Title: Association of CYP1A1 and CYP1B1 inhibition in in vitro assays with drug-induced liver injury.
Drug-induced liver injury (DILI) is one of the major causes for the discontinuation of drug development and withdrawal of drugs from the market. Since it is known that reactive metabolite formation and being substrates or inhibitors of cytochrome P450s (P450s) are associated with DILI, we systematically investigated the association between human P450 inhibition and DILI. The inhibitory activity of 266 DILI-positive drugs (DILI drugs) and 92 DILI-negative drugs (no-DILI drugs), which were selected from Liver Toxicity Knowledge Base (US Food and Drug Administration), against 8 human P450 forms was assessed using recombinant enzymes and luminescent substrates, and the threshold values... (truncated)

Title: Mechanisms of drug toxicity and relevance to pharmaceutical development.
Toxicity has been estimated to be responsible for the attrition of approximately one-third of drug candidates and is a major contributor to the high cost of drug development, particularly when not recognized until late in clinical trials or post-marketing. The causes of drug toxicity can be classified in several ways and include mechanism-based (on-target) toxicity, immune hypersensitivity, off-target toxicity, and bioactivation/covalent modification. In addition, idiosyncratic responses are rare but can be one of the most problematic issues; several hypotheses for these have been advanced. Although covalent binding of drugs to proteins was described almost 40 years ago, the significance to... (truncated)

Title: Bioactivation of sitaxentan in liver microsomes, hepatocytes, and expressed human P450s with characterization of the glutathione conjugate by liquid chromatography tandem mass spectrometry.
Sitaxentan is a selective endothelin-A receptor antagonist that was marketed as Thelin in several European countries and Canada for pulmonary arterial hypertension. Sitaxentan was undergoing further clinical trials in the United States but due to four deaths and one case of liver transplantation from severe liver toxicity that appeared to be idiosyncratic in nature, it was withdrawn worldwide in December, 2010. Sitaxentan contains a 1,3-benzodioxole ring that undergoes enzymatic demethyleneation to an ortho-catechol metabolite that can further oxidize to a reactive ortho-quinone metabolite. Here, we report the detection and mass spectral characterization of a glutathione conjugate of this sitaxentan quinone... (truncated)

MESH:D015742 - propofol

Summary:

LLM Prediction Score	0.399 (normalized)
LLM Confidence Score	0.990
Golden Answer (Severity Class)	0.375 (normalized)
Prediction Error	0.024

Retrieved Context:

Title: Disturbance of hepatocellular integrity associated with propofol anaesthesia in surgical patients.
Propofol anaesthesia has not been associated with any hepatic consequences. We used glutathione transferase Alpha (GSTA), a very sensitive indicator of hepatocellular integrity, to evaluate the effect of propofol on the liver. Total intravenous anaesthesia was induced and maintained with propofol without any supplements in 30 female patients undergoing breast surgery. Ten healthy female volunteers given the lipid vehicle of propofol served as controls. Serum GSTA concentration was measured with a sensitive time-resolved immunofluorometric assay. Total intravenous propofol anaesthesia was stable and postoperative nausea negligible. A significant increase in GSTA from 3.1 micrograms.l-1 (mean baseline) to 10.0 micrograms.l-1 (mean peak)... (truncated)

Title: A Comparison of the Effect of Isoflurane and Propofol on Liver Enzymes.
Isoflurane and propofol are routinely used for the maintenance of general anaesthesia. However, recently, they have been implicated in hepatotoxicity resulting in acute liver failure.

Title: Propofol Attenuates Toxic Oxidative Stress by CCl4 in Liver Mitochondria and Blood in Rat.
Anti-oxidant effects of propofol (2, 6-diisopropylphenol) were evaluated agains carbon tetrachloridet CCl4 -induced oxidative stress in rat liver. 30 male rats were equally divided in to 6 groups (5 rats each). Group I (control), while Group II was given CCl4 (3 mL /Kg/day, IP). Animals of Groups III received only propofol (10 mg/Kg/day, IP). Group IV was given propofol+ CCl4. Group V was administered vitamin E (alpha-tocopherol acetate 15 mg/Kg/day, SC) .Animals of Group VII received alpha-tocopherol acetate + CCl4 once daily for two weeks. After treatment, blood and liver mitochondria were isolated. Anti-oxidant enzymes activity such as glutathione peroxidase... (truncated)

Title: A Rare Case of Propofol-Induced Acute Liver Failure and Literature Review.
The incidence of drug-induced acute liver failure is increasing. A number of drugs can inhibit mitochondrial functions, alter β-oxidation and cause accumulation of free fatty acids within the hepatocytes. This may result in hepatic steatosis, cell death and liver injury. In our case, propofol, an anesthetic drug commonly used in adults and children, is suspected to have induced disturbance of the mitochondrial respiratory chain, which in consequence led to insufficient energy supply and finally liver failure. We report the case of a 35-year-old Caucasian woman with acute liver failure after anesthesia for stripping of varicose veins. Liver histology, imaging and... (truncated)

Title: Propofol inhibits gap junctions by attenuating sevoflurane-induced cytotoxicity against rat liver cells in vitro.
Liver abnormalities are seen in a small proportion of patients following anaesthesia with sevoflurane.

MESH:C056516 - exemestane

Summary:

LLM Prediction Score	0.523 (normalized)
LLM Confidence Score	0.980
Golden Answer (Severity Class)	0.5 (normalized)
Prediction Error	0.023

Retrieved Context:

Title: Exemestane induced cholestatic liver injury - A case report.
Exemestane, a steroidal aromatase inhibitor, is an important therapeutic option in the treatment of post-menopausal hormone receptor positive breast cancer. Adverse effects include hot flashes and bone loss, but rarely is hepatotoxicity reported. We report a case of exemestane induced cholestatic liver injury following exemestane initiation.

Title: [A case of recurrent breast cancer with liver metastasis responding to exemestane].
We report a woman in her 30s who developed a right breast tumor 10 years after undergoing mastectomy for invasive ductal carcinoma of the left breast. She underwent modified radical mastectomy for the right breast cancer, which was diagnosed histologically as invasive ductal carcinoma with metastasis to the axillary lymph nodes. Because of the risk of recurrence, she received postoperative systemic adjunctive chemotherapy using CMF, but this had to be withdrawn because of liver toxicity. The patient therefore received hormonal therapy with goserelin and tamoxifen for 24 months. During this period, however, she became menopausal, necessitating withdrawal of the goserelin.... (truncated)

Title: Exemestane Attenuates Hepatic Fibrosis in Rats by Inhibiting Activation of Hepatic Stellate Cells and Promoting the Secretion of Interleukin 10.
Exemestane (EXE) is an irreversible steroidal aromatase inhibitor mainly used as an adjuvant endocrine therapy for postmenopausal women suffering from breast cancer. Besides inhibiting aromatase activity, EXE has multiple biological functions, such as antiproliferation, anti-inflammatory, and antioxidant activities which are all involved in hepatic fibrosis. Therefore, we investigated the role of EXE during the progress of hepatic fibrosis. The effect of EXE on liver injury and fibrosis were assessed in two hepatic fibrosis rat models, which were induced by either carbon tetrachloride (CCl₄) or bile duct ligation (BDL). The influence of EXE treatment on activation and proliferation of primary rat... (truncated)

Title: [A real-world study of the effects of endocrine therapy on liver function in breast cancer].
Objective: To compare the effect of different endocrine therapy drugs on liver function in patients with early breast cancer. Methods: A retrospective cohort study was conducted to include 4 318 patients with early breast cancer who received adjuvant endocrine therapy in Department of Breast Surgery, Peking Union Medical College Hospital from January 1, 2013 to December 31, 2021. All the patients were female, aged (51.2±11.3) years (range: 20 to 87 years), including 1 182 patients in the anastrozole group, 592 patients in the letrozole group, 332 patients in the exemestane group, and 2 212 patients in the toremifene group. The... (truncated)

Title: Severe prolonged cholestatic hepatitis caused by exemestane.
No abstract available.

MESH:D001590 - benztropine

Summary:

LLM Prediction Score	0.023 (normalized)
LLM Confidence Score	0.970
Golden Answer (Severity Class)	0.0 (normalized)
Prediction Error	0.023

Retrieved Context:

Title: Focus on Over-the-Counter Drugs' Misuse: A Systematic Review on Antihistamines, Cough Medicines, and Decongestants.
Background: Over the past 20 years or so, the drug misuse scenario has seen the emergence of both prescription-only and over-the-counter (OTC) medications being reported as ingested for recreational purposes. OTC drugs such as antihistamines, cough/cold medications, and decongestants are reportedly the most popular in being diverted and misused. Objective: While the current related knowledge is limited, the aim here was to examine the published clinical data on OTC misuse, focusing on antihistamines (e.g., diphenhydramine, promethazine, chlorpheniramine, and dimenhydrinate), dextromethorphan (DXM)- and codeine-based cough medicines, and the nasal decongestant pseudoephedrine. Methods: A systematic literature review was carried out with the... (truncated)

Title: Utility of spherical human liver microtissues for prediction of clinical drug-induced liver injury.
Drug-induced liver injury (DILI) continues to be a major source of clinical attrition, precautionary warnings, and post-market withdrawal of drugs. Accordingly, there is a need for more predictive tools to assess hepatotoxicity risk in drug discovery. Three-dimensional (3D) spheroid hepatic cultures have emerged as promising tools to assess mechanisms of hepatotoxicity, as they demonstrate enhanced liver phenotype, metabolic activity, and stability in culture not attainable with conventional two-dimensional hepatic models. Increased sensitivity of these models to drug-induced cytotoxicity has been demonstrated with relatively small panels of hepatotoxicants. However, a comprehensive evaluation of these models is lacking. Here, the predictive value... (truncated)

Title: Identifying olanzapine induced liver injury in the setting of acute hepatitis C: A case report.
Olanzapine is linked to asymptomatic, transient elevations of liver aminotransferases but is historically thought to rarely cause significant hepatotoxicity. Underlying liver disease is a risk factor for drug-induced liver injury and may complicate the differential diagnosis of acute transaminitis in patients taking medications associated with hepatotoxicity. Ms L presented with 2 months of new psychotic symptoms resulting in hospitalizations. Although psychosis previously improved with haloperidol, she reported symptoms concerning for akathisia. Restlessness improved and psychotic symptoms resolved after initiation of olanzapine. Concurrently, her alanine aminotransferase (ALT) was elevated, prompting further workup and new diagnosis of acute hepatitis C. Over the... (truncated)

Title: Drug-Induced Liver Injury: An Institutional Case Series and Review of Literature.
Drug-induced liver injury (DILI) is the most common cause of acute liver failure in the USA. DILI can be broadly classified as Intrinsic and Idiosyncratic. Identifying predictors and at-risk patients are challenging but can have a substantial clinical implication. This case report series demonstrates the importance of valproic acid, fluconazole, and amiodarone as potential hepatoxic agents of drug-induced liver injury leading to acute hepatic failure. The causality in all cases was established by Roussel Uclaf Causality Assessment Method/Council for International Organizations of Medical Sciences score and Naranjo Algorithm. Obesity, hypo-perfusion state, and concurrent hepatotoxic agent might identify at-risk patients. Further... (truncated)

Title: Addressing the Side Effects of Contemporary Antidepressant Drugs: A Comprehensive Review.
Randomized trials have shown that selective serotonin reuptake inhibitors (SSRIs) and serotonin-norepinephrine reuptake inhibitors (SNRIs) have better safety profiles than classical tricyclic antidepressants (TCAs). However, an increasing number of studies, including meta-analyses, naturalistic studies, and longer-term studies suggested that SSRIs and SNRIs are no less safe than TCAs. We focused on comparing the common side effects of TCAs with those of newer generation antidepressants including SSRIs, SNRIs, mirtazapine, and bupropion. The main purpose was to investigate safety profile differences among drug classes rather than the individual antidepressants, so studies containing comparison data on drug groups were prioritized. In terms of... (truncated)

MESH:C063533 - dofetilide

Summary:

LLM Prediction Score	0.022 (normalized)
LLM Confidence Score	0.950
Golden Answer (Severity Class)	0.0 (normalized)
Prediction Error	0.022

Retrieved Context:

Title: TMP-SMX induced type 4 hypersensitivity with multi-organ involvement.
Trimethoprim-sulfamethoxazole (TMP-SMX), also referred to as co-trimazole, is a common antibiotic used to treat a wide range of infections ranging from simple skin and soft tissue infections to opportunistic infections such as Pneumocystis jirovecii. Generally, this medication is well-tolerated, but severe adverse reactions, such as myelosuppression and hepatitis, can occur, albeit rarely. In this case report, we describe a patient who presented to the hospital with symptoms of rash, elevated liver enzymes, thrombocytopenia, and acute kidney injury 2 weeks after completing a course of TMP-SMX for a skin infection. We highlight the difficulties in diagnosing adverse events associated with this... (truncated)

Title: The role of dolutegravir in the management of HIV infection.
Dolutegravir is the most recent integrase strand transfer inhibitor approved for HIV-1 infection in both treatment-naïve and experienced patients. As a tricyclic carbamoyl pyridone analog, dolutegravir is rapidly absorbed and distributes through the cerebrospinal fluid. It is hepatically metabolized by uridine diphosphate glucuronosyl transferase 1A1; no inhibition or induction of cytochrome P450 enzymes is noted. As a substrate of CYP 3A4, dolutegravir is affected by rifampin, efavirenz, tipranavir/ritonavir, fosamprenavir/ritonavir, and dose increase is required. Dolutegravir inhibits the organic cation transporter 2, resulting in decreased creatinine clearance with no apparent decrease in renal function. Other adverse effects are minimal but include... (truncated)

Title: Voriconazole in the management of nosocomial invasive fungal infections.
Voriconazole is a new triazole developed for the treatment of life-threatening fungal infections. The drug is available for both oral and intravenous administration; the oral formulation has excellent bioavailability. The side-effect profile of voriconazole presents an acceptable safety and tolerability spectrum: transient visual disturbances, liver enzyme abnormalities, and skin rashes are the most frequently reported side effects but rarely lead to discontinuation. The potential for drug-drug interactions is high, because of its extensive hepatic metabolism. Careful attention to dosage is required, and serum levels and the effects of interacting drugs should be monitored. Review of 25 470 isolates of yeasts... (truncated)

Title: Fungal infections: their diagnosis and treatment in transplant recipients.
Systemic fungal infections typically occur in individuals who are seriously ill with recognized risk factors such as those frequently found in transplant recipients. Unfortunately, they are often diagnosed late, when the efficacy of the available treatments is low, often less than 50%, and the cost in terms of lives lost, hospital length of stay, and total hospital costs is substantially increased. The application of antifungal therapies associated with reported efficacy rates greater than 50% are those used prophylactically. When used prophylactically, these infections are reduced in greater than 95% of the expected cases. The choice of a prophylactic agent should... (truncated)

Title: Role of Pharmacogenetics in Adverse Drug Reactions: An Update towards Personalized Medicine.
Adverse drug reactions (ADRs) are an important and frequent cause of morbidity and mortality. ADR can be related to a variety of drugs, including anticonvulsants, anaesthetics, antibiotics, antiretroviral, anticancer, and antiarrhythmics, and can involve every organ or apparatus. The causes of ADRs are still poorly understood due to their clinical heterogeneity and complexity. In this scenario, genetic predisposition toward ADRs is an emerging issue, not only in anticancer chemotherapy, but also in many other fields of medicine, including hemolytic anemia due to glucose-6-phosphate dehydrogenase (G6PD) deficiency, aplastic anemia, porphyria, malignant hyperthermia, epidermal tissue necrosis (Lyell's Syndrome and Stevens-Johnson Syndrome), epilepsy,... (truncated)

MESH:D002442 - ceftazidime

Summary:

LLM Prediction Score	0.353 (normalized)
LLM Confidence Score	0.990
Golden Answer (Severity Class)	0.375 (normalized)
Prediction Error	0.022

Retrieved Context:

Title: Ceftazidime induced liver injury.
A 65-year-old woman with type II diabetes mellitus complicated by non-healing ulcers with recurrent osteomyelitis was admitted for progression of cellulitis after treatment failure with an outpatient course of amoxicillin-clavulanate. She was found to have persistent osteomyelitis and started on ceftazidime for a culture documented <i>Pseudomonas aeruginosa</i> infection. After two parenteral doses, she had a rapid rise in liver function tests (LFTs) in a hepatocellular pattern. Due to rapid identification, all medications with potential hepatotoxicity, including ceftazidime, were discontinued and the LFTs promptly returned to baseline over 3 days. Of note, the patient did not experience any symptoms of liver injury.... (truncated)

Title: Use of ceftazidime in the therapy of serious infections, including those due to multiresistant organisms.
Ceftazidime was administered intravenously or intramuscularly or both in doses of 1 to 6 g per day to 33 patients with serious gram-negative bacillary infections (12 pulmonary, 10 urinary tract, 4 soft tissue, 4 intraabdominal, and 3 miscellaneous infections). Twenty-one patients were septicemic. We identified 20 isolates of members of the family Enterobacteriaceae and 13 isolates of Pseudomonas aeruginosa. Seventeen patients had failed to respond to previous antimicrobial therapy. A total of 23 patients were clinically cured, 7 patients improved, and 3 patients failed to respond to therapy. The selection or emergence of resistant organisms during treatment (mostly Candida spp.,... (truncated)

Title: Ceftazidime as a single agent in the treatment of severe Pseudomonas aeruginosa infections.
Ceftazidime was administered intravenously in doses of 1 to 6 g/day to 21 patients with serious Pseudomonas aeruginosa infections (12 pulmonary, 6 septicaemias, 3 urinary tract infections). Mean MIC was 1.0 mg/l. Eighteen (86%) of the 21 patients responded satisfactorily (cured or improved). The selection or emergence of resistant organisms during treatment (mostly Candida, Staphylococcus aureus, and enterococci) was noted in 6 patients. Toxicity was minimal (eosinophilia and reversible mild liver function abnormalities).

Title: A rare case of cefepime-induced cholestatic liver injury.
Cefepime is widely used in the hospital setting, and only a few studies have reported neurotoxicity and nephrotoxicity as side effects of this drug. Herein, we present a 93-year-old man who exhibited features of cholestatic hepatitis including elevated blood transaminases and direct-form predominant bilirubin levels after administration of cefepime. Blood liver tests showed total recovery after discontinuing the offending agent. Cefepime was probable to cause drug-induced cholestatic hepatitis in our patient since the Roussel Uclaf Causality Assessment Method score for cefepime was 7. No drug interactions were likely according to the Drug Interaction Probability Scale for this patient. No similar... (truncated)

Title: Hypersensitivity Pneumonitis Caused by Cephalosporins With Identical R1 Side Chains.
Drug-induced hypersensitivity pneumonitis results from interactions between pharmacologic agents and the human immune system. We describe a 54-year-old man with hypersensitivity pneumonitis caused by cephalosporins with identical R1 side chains. The patient, who complained of cough with sputum, was prescribed ceftriaxone and clarithromycin at a local clinic. The following day, he complained of dyspnea, and chest X-ray revealed worsening of inflammation. Upon admission to our hospital, antibiotics were changed to cefepime with levofloxacin, but his pneumonia appeared to progress. Changing antibiotics to meropenem with ciprofloxacin improved his symptoms and radiologic findings. Antibiotics were de-escalated to ceftazidime with levofloxacin, and his... (truncated)

MESH:D004492 - edetic acid

Summary:

LLM Prediction Score	0.397 (normalized)
LLM Confidence Score	0.980
Golden Answer (Severity Class)	0.375 (normalized)
Prediction Error	0.022

Retrieved Context:

Title: Toxicity of bean cooking media containing EDTA in mice.
The possible renal and hepatic toxicities of ethylenediaminetetraacetic acid (EDTA) in bean cooking media were studied using 100 male albino mice. Two sublethal doses of EDTA were used to explore their toxic effects; 20 mg/kg and 200 mg/kg, which corresponded to 1/100th and 1/10th of LD₅₀, respectively. Accordingly, the toxicity study was performed using 50 mice, divided into five groups (<i>n</i> = 10/group) as follows: group 1 (Gp1) served as a negative control and was orally administered normal saline; group 2 (Gp2) was administered the bean cooking medium; group 3 (Gp3) was administered EDTA (200 mg/kg); group 4 (Gp4) was... (truncated)

Title: Salvia officinalis flowers extract ameliorates liver and kidney injuries induced by simultaneous intoxication with ethanol/castor oil.
The current study investigated the possible mechanisms of aqueous extract Salvia officinalis flowers (SF-AE) and its protective effects against hepatorenal toxicities produced by simultaneous acute administration of ethanol (EtOH)/castor oil (CO). Healthy male rats (N = 50) were separated into five equal groups: control, Ethanol (EtOH) + Castor oil (CO), doses of increasing orders of SF-AE (50, 100, and 200 mg/kg, b.w., p.o.) during 15 days. Liver and kidney injuries were induced by EtOH (4 g/kg, b.w., p.o.) combined with CO (5 mL/kg, b.w., p.o.). Compared to the control group, SF-AE pretreatment protected against simultaneous administration of EtOH and CO-caused serious histological alterations in liver and kidney tissues.... (truncated)

Title: Acute hepatitis in a woman following excessive ingestion of an energy drink: a case report.
The consumption of energy drinks has increased significantly. We report the case of a patient who presented to our hospital with jaundice, abdominal pain, and markedly increased liver transaminases likely due to the increased consumption of an energy drink. To the best of our knowledge, this is the first case report in the literature linking the development of acute hepatitis to the consumption of an energy drink.

Title: <i>Fat-1</i> Transgenic Mice With Augmented n3-Polyunsaturated Fatty Acids Are Protected From Liver Injury Caused by Acute-On-Chronic Ethanol Administration.
Alcohol-associated liver disease (ALD) is the leading cause of liver disease worldwide, and alcohol-associated hepatitis (AH), a severe form of ALD, is a major contributor to the mortality and morbidity due to ALD. Many factors modulate susceptibility to ALD development and progression, including nutritional factors such as dietary fatty acids. Recent work from our group and others showed that modulation of dietary or endogenous levels of n6-and n3-polyunsaturated fatty acids (PUFAs) can exacerbate or attenuate experimental ALD, respectively. In the current study, we interrogated the effects of endogenous n3-PUFA enrichment in a mouse model which recapitulates features of early human... (truncated)

Title: Transition between acute and chronic hepatotoxicity in mice is associated with impaired energy metabolism and induction of mitochondrial heme oxygenase-1.
The formation of protein inclusions is frequently associated with chronic metabolic diseases. In mice, short-term intoxication with 3,5-diethoxycarbonyl-1,4-dihydrocollidine (DDC) leads to hepatocellular damage indicated by elevated serum liver enzyme activities, whereas only minor morphological changes are observed. Conversely, chronic administration of DDC for several weeks results in severe morphological damage, characterized by hepatocellular ballooning, disruption of the intermediate filament cytoskeleton, and formation of Mallory-Denk bodies consisting predominantly of misfolded keratins, Sqstm1/p62, and heat shock proteins. To evaluate the mechanistic underpinnings for this dichotomy we dissected the time-course of DDC intoxication for up to 10 weeks. We determined body weight change,... (truncated)

MESH:D010110 - oxymetholone

Summary:

LLM Prediction Score	0.895 (normalized)
LLM Confidence Score	0.980
Golden Answer (Severity Class)	0.875 (normalized)
Prediction Error	0.020

Retrieved Context:

Title: [Peliosis hepatis, complicating treatment with anabolic steroids (author's transl)].
The clinical and pathological findings of peliosis hepatis are reported, complicating a long-term treatment with an anabolic steroid in a 19 year old male patient suffering from chronic aplastic anemia. Following a 15 months course of oxymetholone (Anapolon) a cholestatic jaundice developed without regression despite discontinuing the drug. 4 months later the patient expired as a consequence of his hematologic disease. At autopsy the liver showed a parenchymal type of peliosis hepatis with multiple blood filled cavities and focal liver cell necroses. The pathogenesis of this rare condition is discussed by means of literature demonstrating the hepatotoxic effect of C17-alkylated... (truncated)

Title: Effects of Platelet-Rich Plasma on the Oxymetholone-Induced Testicular Toxicity.
Oxymetholone is one of the anabolic steroids that has widely been used among teenagers and athletes to increase their muscle bulk. It has undesirable effects on male health and fertility. In this study, the therapeutic effects of platelet-rich plasma (PRP) on oxymetholone-induced testicular toxicity were investigated in adult albino rats. During the experiments, 49 adult male albino rats were divided into 4 main groups: Group 0 (donor group) included 10 rats for the donation of PRP, Group I (control group) included 15 rats, Group II included 8 rats that received 10 mg/kg of oxymetholone orally, once daily, for 30 days,... (truncated)

Title: Peliosis hepatis. Twelve cases associated with oral androgen therapy.
Peliosis hepatis was encountered in 12 patients treated with high-dose oxymetholone or fluoxymesterone therapy. In three cases liver failure was the primary cause of death. In one case, the diagnosis was established by biopsy, the androgen therapy was discontinued, and the lesion was absent at autopsy two years later. In eight cases peliosis hepatis was an incidental finding at postmortem examination. The clinical and anatomic features of these cases are described, and previously reported cases of peliosis are briefly reviewed. A hypothesis is offered to explain the association of this peculiar lesion with anabolic androgen therapy.

Title: Oxymetholone treatment for sickle cell anemia.
Seven patients with sickle cell anemia were treated with oxymetholone for at least 2 mo. Markedly increased basal rates of hemolysis and erythropoiesis were confirmed. The urinary erythropoietin excretion was either normal or lower than expected for the red cell mass, and an expanded blood volume was due primarily to an increased plasma volume. After androgen therapy, six patients demonstrated more than a fivefold increase in urinary erythropoietin, with an increase in red cell mass ranging from 17%-75% above the control value. All showed a decline in serum iron level to the 25-75 mug/100 ml range within 4 wk after... (truncated)

Title: Oxymetholone hepatotoxicity enhanced by concomitant use of cyclosporin A in a bone marrow transplant patient.
No abstract available.

MESH:C078058 - exametazime

Summary:

LLM Prediction Score	0.019 (normalized)
LLM Confidence Score	0.870
Golden Answer (Severity Class)	0.0 (normalized)
Prediction Error	0.019

Retrieved Context:

Title: A humanized mouse model of liver fibrosis following expansion of transplanted hepatic stellate cells.
Hepatic stellate cells (HSCs) are major contributors to liver fibrosis, as hepatic injuries may cause their transdifferentiation into myofibroblast-like cells capable of producing excessive extracellular matrix proteins. Also, HSCs can modulate engraftment of transplanted hepatocytes and contribute to liver regeneration. Therefore, understanding the biology of human HSCs (hHSCs) is important, but effective methods have not been available to address their fate in vivo. To investigate whether HSCs could engraft and repopulate the liver, we transplanted GFP-transduced immortalized hHSCs into immunodeficient NOD/SCID mice. Biodistribution analysis with radiolabeled hHSCs showed that after intrasplenic injection, the majority of transplanted cells rapidly translocated to... (truncated)

Title: Optical fluorescence imaging with shortwave infrared light emitter nanomaterials for in vivo cell tracking in regenerative medicine.
In vivo tracking and monitoring of adoptive cell transfer has a distinct importance in cell-based therapy. There are many imaging modalities for in vivo monitoring of biodistribution, viability and effectiveness of transferred cells. Some of these procedures are not applicable in the human body because of low sensitivity and high possibility of tissue damages. Shortwave infrared region (SWIR) imaging is a relatively new technique by which deep biological tissues can be potentially visualized with high resolution at cellular level. Indeed, scanning of the electromagnetic spectrum (beyond 1000 nm) of SWIR has a great potential to increase sensitivity and resolution of... (truncated)

Title: Current and Future Theranostic Applications of the Lipid-Calcium-Phosphate Nanoparticle Platform.
Over the last four years, the Lipid-Calcium-Phosphate (LCP) nanoparticle platform has shown success in a wide range of treatment strategies, recently including theranostics. The high specific drug loading of radiometals into LCP, coupled with its ability to efficiently encapsulate many types of cytotoxic agents, allows a broad range of theranostic applications, many of which are yet unexplored. In addition to providing an overview of current medical imaging modalities, this review highlights the current theranostic applications for LCP using SPECT and PET, and discusses potential future uses of the platform by comparing it with both systemically and locally delivered clinical radiotherapy... (truncated)

MESH:D013989 - ticrynafen

Summary:

LLM Prediction Score	0.981 (normalized)
LLM Confidence Score	0.770
Golden Answer (Severity Class)	1.0 (normalized)
Prediction Error	0.019

Retrieved Context:

Title: Ticrynafen-associated hepatic injury: analysis of 340 cases.
Ticrynafen, a uricosuric diuretic, was withdrawn from clinical use in the United States in 1980 after having been implicated as the cause of a number of instances of serious hepatic injury. In this report, we analyze 340 cases of ticrynafen-associated hepatic disease reported to the manufacturer from the time of initial marketing until shortly after the drug had been recalled. Jaundice was recorded in 246 of 287 patients with sufficient clinical information, and 25 (10.2%) of these icteric patients died. The high levels of serum aminotransferase and the case fatality rate are consistent with the hepatocellular injury that was evident... (truncated)

Title: Immune mechanisms in tienilic acid associated hepatotoxicity.
In order to investigate the mechanisms underlying the hepatotoxicity associated with tienilic acid (Ticrynafen) ingestion we have looked for evidence of sensitisation to drug altered liver cell determinants using an indirect antibody dependent, cell mediated cytotoxicity assay (ADCC). As targets, hepatocytes were isolated from rabbits pretreated with either tienilic acid or its isomer with or without previous enzyme induction with either phenobarbitone or B-naphthoflavone (BNF). Sera from 16 of 36 patients with presumed tienilic acid hepatotoxicity induced significant cytotoxicity to hepatocytes isolated from rabbits pretreated with BNF and subsequently tienilic acid. Three of 10 sera from patients receiving tienilic acid... (truncated)

Title: Drug-induced hepatitis associated with anticytoplasmic organelle autoantibodies.
A study from five hepatology units documenting 157 cases of drug-induced hepatitis and a second study from a laboratory of immunology which tested more than 100,000 sera permitted us to establish the frequency of antiorganelle antibodies and their diagnostic value in drug-induced hepatitis. In drug-induced hepatitis caused by a heterogenous group of drugs consisting of ajmaline, aminopterine, isaxonine, isoniazid, perhexiline, phenylbutazone and troleandromycine, antiorganelle antibodies were absent or rare. In drug-induced hepatitis caused by another heterogenous group of drugs, including clometacin, fenofibrate, oxyphenisatin and papaverine, antismooth muscle, antinucleus and antimitochondria antibodies were found in isolation or in different combinations in... (truncated)

Title: Evaluation of the potential for drug-induced liver injury based on in vitro covalent binding to human liver proteins.
Prediction of idiosyncratic drug-induced liver injury (DILI) is difficult, and the underlying mechanisms are not fully understood. However, many drugs causing DILI are considered to form reactive metabolites and covalently bind to cellular macromolecules in the liver. The objective of this study was to clarify whether the risk of idiosyncratic DILI can be estimated by comparing in vitro covalent binding (CB) levels among 12 positive compounds (acetaminophen, alpidem, bromfenac, carbamazepine, diclofenac, flutamide, imipramine, nefazodone, tacrine, ticlopidine, tienilic acid, and troglitazone) for DILI and 12 negative compounds (acetylsalicylic acid, caffeine, dexamethasone, losartan, ibuprofen, paroxetine, pioglitazone, rosiglitazone, sertraline, theophylline, venlafaxine, and zolpidem).... (truncated)

Title: Hepatotoxicity associated with ticrynafen--a uricosuric diuretic.
Herein, we describe a patient who had ticrynafen-associated acute hepatitis. This complication has been reported in 57 patients (52 to the manufacturer, four to the Food and Drug Administration, and the current patient) in this country. The mechanism of hepatocellular injury is not known, but from the data to date it seems most likely to result from a hypersensitivity reaction rather than a direct hepatotoxic effect. The histopathological findings are difficult to differentiate from acute viral hepatitis. Removal of this drug from the market resulted from the prompt action of physicians in reporting the complications to the manufacturer and the... (truncated)

MESH:D003907 - dexamethasone

Summary:

LLM Prediction Score	0.357 (normalized)
LLM Confidence Score	0.990
Golden Answer (Severity Class)	0.375 (normalized)
Prediction Error	0.018

Retrieved Context:

Title: Dexamethasone Pretreatment Alleviates Isoniazid/Lipopolysaccharide Hepatotoxicity: Inhibition of Inflammatory and Oxidative Stress.
Isoniazid (INH) remains a cornerstone key constitute of the current tuberculosis management strategy, but its hepatotoxic potentiality remains a significant clinical problem. Our previous findings succeed to establish a rat model of INH hepatotoxicity employing the inflammatory stress theory in which non-injurious doses of inflammatory-mediating agent bacterial lipopolysaccharides (LPS) augmented the toxicity of INH that assist to uncover the mechanisms behind INH hepatotoxicity. Following LPS exposure, several inflammatory cells are activated and it is likely that the consequences of this activation rather than direct hepatocellular effects of LPS underlie the ability of LPS to augment toxic responses. In this study,... (truncated)

Title: Dexamethasone reduces methotrexate biliary elimination and potentiates its hepatotoxicity in rats.
Increased hepatotoxicity of methotrexate has been reported during dexamethasone therapy in humans. Despite the observed inducing effect of dexamethasone on some methotrexate transporting proteins in the liver, the kinetic aspects of this interaction have not been studied yet. Thus, the aim of the present study was to evaluate the influence of dexamethasone on the hepatic and overall pharmacokinetics of methotrexate. Pharmacokinetics of methotrexate was evaluated in rats during an in vivo steady-state clearance study after either single intravenous dose of dexamethasone or its four-day oral administration in a dose optimized for transport proteins induction. Dexamethasone oral pretreatment reduced biliary clearance... (truncated)

Title: Chronic dexamethasone exposure markedly decreased the hepatic triglyceride accumulation in growing goats.
Chronic stress seriously threatens welfare and health in animals and humans. Consecutive dexamethasone (Dex) injection was used to mimic chronic stress previously. In order to investigate the effect of chronic stress on hepatic lipids metabolism, in this study, 10 healthy male goats were randomly allocated into two groups, one received a consecutive injection of Dex via intramuscularly for 3 weeks (Dex group), the other received the same volume of saline as the control group (Con group). Hepatic health and triglyceride (TG) metabolism were analyzed and compared between two groups. The data showed that a significant decrease of TG in plasma... (truncated)

Title: Dexamethasone Transiently Enhances Transgene Expression in the Liver When Administered at Late-Phase Post Long-Term Adeno-Associated Virus Transduction.
Glucocorticoids have anti-inflammatory and immunosuppressive functions and have commonly been used for preventing liver toxicity after the systemic application of a high dose of adeno-associated virus (AAV) vector for gene therapy. Clinical studies have reported that glucocorticoids have rescued factor IX (FIX) expression in patients with hemophilia B who showed a reduced FIX expression at 6 to 10 weeks post-AAV vector administration. In this study, we explored whether glucocorticoids could affect transgene expression in AAV targeted livers in animal models. When dexamethasone was applied before AAV9/FIX vector administration in the wild-type C57BL/6 mice, FIX expression was much higher than that... (truncated)

Title: Complete protection by high-dose dexamethasone against the hepatotoxicity of the novel antitumor drug yondelis (ET-743) in the rat.
Yondelis (ET-743) is a promising antitumor drug with hepatotoxic properties in animals and humans. Here the hypothesis was tested that dexamethasone can ameliorate manifestations of yondelis-induced hepatotoxicity in the female Wistar rat, which is the animal species with the highest sensitivity toward the adverse hepatic effect of yondelis. Hepatotoxicity was adjudged by measurement of plasma levels of alkaline phosphatase, aspartate aminotransferase, and bilirubin, and by liver histopathology. Yondelis (40 micro g/kg i.v.) alone caused a dramatic elevation of plasma alkaline phosphatase, aspartate aminotransferase, and bilirubin levels, and degeneration and patchy focal necrosis of bile duct epithelial cells. Pretreatment of rats... (truncated)

MESH:D000077466 - tirofiban

Summary:

LLM Prediction Score	0.017 (normalized)
LLM Confidence Score	0.980
Golden Answer (Severity Class)	0.0 (normalized)
Prediction Error	0.017

Retrieved Context:

Title: Effects of tirofiban on stent thrombosis, Hs-CRP, IL-6 and sICAM-1 after PCI of acute myocardial infarction.
Effects of tirofiban on stent thrombosis, high-sensitivity C-reactive protein (Hs-CRP), interleukin-6 (IL-6) and soluble intercellular adhesion molecule-1 (sICAM-1) after percutaneous coronary intervention (PCI) of acute myocardial infarction (AMI) were investigated. A total of 94 AMI patients receiving PCI in Shouguang City People's Hospital from January 2016 to September 2016 were selected and randomly divided into control (n=47) and observation group (n=47). The control group was treated with aspirin + clopidogrel before and after operation, while the observation group was treated with tirofiban based on the treatment of control group. The postoperative stent thrombosis was compared between the two groups, and... (truncated)

Title: How to Solve the Conundrum of Heparin-Induced Thrombocytopenia during Cardiopulmonary Bypass.
Heparin-induced thrombocytopenia (HIT) is a major issue in cardiac surgery requiring cardiopulmonary bypass (CPB). HIT represents a severe adverse drug reaction after heparin administration. It consists of immune-mediated thrombocytopenia paradoxically leading to thrombotic events. Detection of antibodies against platelets factor 4/heparin (anti-PF4/H) and aggregation of platelets in the presence of heparin in functional in vitro tests confirm the diagnosis. Patients suffering from HIT and requiring cardiac surgery are at high risk of lethal complications and present specific challenges. Four distinct phases are described in the usual HIT timeline, and the anticoagulation strategy chosen for CPB depends on the phase in... (truncated)

Title: Efficacy and safety of fondaparinux versus enoxaparin in patients undergoing percutaneous coronary intervention treated with the glycoprotein IIb/IIIa inhibitor tirofiban.
In worldwide, the mortality rate of acute myocardial infarction (AMI) raises year by year. Although the applications of percutaneous coronary intervention (PCI) and anticoagulants effectively reduce the mortality of patients with acute coronary syndrome (ACS), but also increase the incidence of bleeding. Therefore, drugs with stable anticoagulant effects are urgently required.

Title: Efficacy and safety of quadruple therapy including tirofiban in the treatment of Chinese NSTE-ACS patients failing to receive timely percutaneous coronary intervention.
Although it has been shown to be superior to simple antithrombotic drug therapy, most patients are unable to receive timely percutaneous coronary intervention (PCI) and are treated with conventional triple antithrombotic therapy (aspirin, clopidogrel, low-molecular-weight heparin). Here, we evaluate the efficacy and safety of adding low-dose tirofiban to this regimen.

Title: Clinical efficacy of tirofiban combined with a Solitaire stent in treating acute ischemic stroke.
This study explores the safety and effect of acute cerebral infarction treatment by microcatheter injection of tirofiban combined with a Solitaire AB stent and/or stent implantation. Emergency cerebral angiograms showing the responsible vascular occlusion of 120 acute cerebral infarction patients who underwent emergency endovascular thrombectomy were included in the study. These patients were randomly divided into two groups using the random number table method: treatment group (n=60) that received thrombectomy (with cerebral artery stents) combined with intracerebral injection of tirofiban and control group (n=60) that only received thrombectomy (with cerebral artery stents alone). The baseline data, cerebral angiography before and... (truncated)

MESH:D020888 - vigabatrin

Summary:

LLM Prediction Score	0.233 (normalized)
LLM Confidence Score	0.980
Golden Answer (Severity Class)	0.25 (normalized)
Prediction Error	0.017

Retrieved Context:

Title: [Newer antiepileptic drugs].
Ten newer antiepileptic drugs have been developed since 1990s. These drugs have wider therapeutic spectra, fewer side-effects, and lesser drug-to-drug interactions compared with the older typical antiepileptic drugs. Among them, zonisamide was developed in Japan and has been used from 1989. Gabapentin was at length approved in 2006. The other newer antiepileptic drugs are not approved yet in Japan. Felbamate can not be used in Europe because it may induce lethal hepatic toxicity and aplastic anemia. Vigabatrin is not approved in USA because it may induce permanent visual field deficit. The USA guideline for epilepsy treatment recommends that patients with... (truncated)

Title: [Newer antiepileptic drugs].
Ten newer antiepileptic drugs have been developed since 1990s. These drugs have wider therapeutic spectra, fewer side-effects, and lesser drug-to-drug interactions compared with the older typical antiepileptic drugs. Among them, zonisamide was developed in Japan and has been used from 1989. Gabapentin was at length approved in 2006. The other newer antiepileptic drugs are not approved yet in Japan. Felbamate can not be used in Europe because it may induce lethal hepatic toxicity and aplastic anemia. Vigabatrin is not approved in USA because it may induce permanent visual field deficit. The USA guideline for epilepsy treatment recommends that patients with... (truncated)

Title: Antiepileptic Drugs and Liver Disease.
Acute, symptomatic seizures or epilepsy may complicate the course of hepatic disease. Choosing the most appropriate antiepileptic drug in this setting represents a difficult challenge, as most medications are metabolized by the liver. This article focuses on the acute and chronic treatment of seizures in patients with advanced liver disease and reviews the hepatotoxic potential of specific antiepileptic drugs. Newer antiepileptic drugs without, or with minimal, hepatic metabolism, such as levetiracetam, lacosamide, topiramate, gabapentin, and pregabalin should be used as first-line therapy. Medications undergoing extensive hepatic metabolism, such as valproic acid, phenytoin, and felbamate should be used as drugs of... (truncated)

Title: Use of antiepileptic drugs in hepatic and renal disease.
The use of antiepileptic drugs in patients with renal or hepatic disease is common in clinical practice. Since the liver and kidney are the main organs involved in the elimination of most drugs, their dysfunction can have important effects on the disposition of antiepileptic drugs. Renal or hepatic disease can prolong the elimination of the parent drug or an active metabolite leading to accumulation and clinical toxicity. It can also affect the protein binding, distribution, and metabolism of a drug. The protein binding of anionic acidic drugs, such as phenytoin and valproate, can be reduced significantly by renal failure, causing... (truncated)

Title: Evidence for both in vivo and in vitro interaction between vigabatrin and alanine transaminase.
Decreases in plasma alanine transaminase (ALA-T) activity of 20-100% have been reported following the use of vigabatrin (Sabril) in patients with uncontrolled epilepsy. This effect has a potential clinical significance as it may mask signs of early, underlying hepatic disease. It is particularly significant in a patient population known to have a higher than average risk of hepatotoxicity due to treatment with other anti-epilepsy drugs or to an independent, but concomitant, disease process. Vigabatrin is a highly specific enzyme antagonist. There is an almost 1000-fold difference between its activity against gamma-amino butyric acid aminotransaminase and ALA-T. However, some activity against... (truncated)

MESH:D002207 - capreomycin

Summary:

LLM Prediction Score	0.391 (normalized)
LLM Confidence Score	0.980
Golden Answer (Severity Class)	0.375 (normalized)
Prediction Error	0.016

Retrieved Context:

Title: Anti-Tuberculosis Treatment: Induced Hepatotoxicity - A Case Report.
Tuberculosis is a potentially communicable disease that can infect any organ in the body such as bones, kidney, intestine but primarily involves lung parenchyma (Pulmonary tuberculosis). The prevalence of TB is 256 per 100,000 population in India. Hepatotoxicity, gastrointestinal and neurological disorders were some the Adverse Drug Reactions (ADR's) reported that significantly increases the mortality rate which leads to decreased efficacy of the treatment. Hepatotoxicity is the most commonly reported ADR in patients treated with anti-tubercular drugs such as isoniazid, rifampicin and pyrazinamide. Clinical manifestations of hepatotoxicity include abdominal pain, nausea, vomiting, and jaundice. We report the case of a... (truncated)

Title: Hepatotoxicity associated with acetaminophen usage in patients receiving multiple drug therapy for tuberculosis.
We report three patients who experienced hepatotoxic reactions in association with acetaminophen ingestion while undergoing treatment for active tuberculosis with isoniazid, rifampin, and other agents. All were young adult women. One patient intentionally took a large amount of acetaminophen and had typical signs and symptoms of acetaminophen overdosage; another took acetaminophen in combination form for a minor upper respiratory illness. She experienced no symptoms. The remaining patient took acetaminophen to ameliorate the symptoms of fever and malaise that were subsequently attributed to tuberculosis. She had the rapid onset of signs and symptoms of isoniazid hepatotoxicity. The patterns of liver function... (truncated)

Title: Long-Term Effects of Multi-Drug-Resistant Tuberculosis Treatment on Gut Microbiota and Its Health Consequences.
Gut microbiota dysbiosis has adverse health effects on human body. Multi-drug-resistant tuberculosis (MDR-TB) treatment uses a variety of antibiotics typically for more than 20 months, which may induce gut microbiota dysbiosis. The aim of this study is to investigate the long-term effects of MDR-TB treatment on human gut microbiota and its related health consequences. A total of 76 participants were recruited at a hospital in Linyi, China. The study included one active MDR-TB treatment group, one recovered group from MDR-TB and two treatment-naive tuberculosis groups as control. The two treatment-naïve tuberculosis groups were constructed to match the sex and the... (truncated)

Title: Adverse events in healthy individuals and MDR-TB contacts treated with anti-tuberculosis drugs potentially effective for preventing development of MDR-TB: a systematic review.
A recent systematic review concluded that there is insufficient evidence on the effectiveness to support or reject preventive therapy for treatment of contacts of patients with multidrug resistant tuberculosis (MDR-TB). Whether preventive therapy is favorable depends both on the effectiveness and the adverse events of the drugs used. We performed a systematic review to assess adverse events in healthy individuals and MDR-TB contacts treated with anti-tuberculosis drugs potentially effective for preventing development of MDR-TB. We searched MEDLINE, EMBASE, and other databases (August 2011). Record selection, data extraction, and study quality assessment were done in duplicate. The quality of evidence was... (truncated)

Title: Review on Usage of Vancomycin in Livestock and Humans: Maintaining Its Efficacy, Prevention of Resistance and Alternative Therapy.
Vancomycin is one of the "last-line" classes of antibiotics used in the treatment of life-threatening infections caused by Gram-positive bacteria. Even though vancomycin was discovered in the 1950s, it was widely used after the 1980s for the treatment of infections caused by methicillin-resistant <i>Staphylococci</i>, as the prevalence of these strains were increased. However, it is currently evident that vancomycin-resistant <i>Staphylococcus aureus</i> and vancomycin-resistant <i>Enterococci</i> have developed for various reasons, including the use of avaparcin-an analog of vancomycin-as a feed additive in livestock. Therefore, prophylactic and empiric use of antibiotics and their analogues need to be minimized. Herein we discuss the... (truncated)

MESH:D000077542 - eptifibatide

Summary:

LLM Prediction Score	0.015 (normalized)
LLM Confidence Score	0.910
Golden Answer (Severity Class)	0.0 (normalized)
Prediction Error	0.015

Retrieved Context:

Title: Targeting integrin pathways: mechanisms and advances in therapy.
Integrins are considered the main cell-adhesion transmembrane receptors that play multifaceted roles as extracellular matrix (ECM)-cytoskeletal linkers and transducers in biochemical and mechanical signals between cells and their environment in a wide range of states in health and diseases. Integrin functions are dependable on a delicate balance between active and inactive status via multiple mechanisms, including protein-protein interactions, conformational changes, and trafficking. Due to their exposure on the cell surface and sensitivity to the molecular blockade, integrins have been investigated as pharmacological targets for nearly 40 years, but given the complexity of integrins and sometimes opposite characteristics, targeting integrin therapeutics... (truncated)

Title: Drug-induced hematologic syndromes.
Objective. Drugs can induce almost the entire spectrum of hematologic disorders, affecting white cells, red cells, platelets, and the coagulation system. This paper aims to emphasize the broad range of drug-induced hematological syndromes and to highlight some of the newer drugs and syndromes. Methods. Medline literature on drug-induced hematologic syndromes was reviewed. Most reports and reviews focus on individual drugs or cytopenias. Results. Drug-induced syndromes include hemolytic anemias, methemoglobinemia, red cell aplasia, sideroblastic anemia, megaloblastic anemia, polycythemia, aplastic anemia, leukocytosis, neutropenia, eosinophilia, immune thrombocytopenia, microangiopathic syndromes, hypercoagulability, hypoprothrombinemia, circulating anticoagulants, myelodysplasia, and acute leukemia. Some of the classic drugs known... (truncated)

Title: Drug-drug interactions between antithrombotics and direct-acting antivirals in hepatitis C virus (HCV) patients: A brief, updated report.
Hepatitis C virus (HCV) is one of the leading causes of chronic liver disease affecting over 71 million people worldwide. An increased incidence of atherothrombotic events [e.g. coronary artery disease (CAD), atrial fibrillation (AF)] has been observed in HCV seropositive patients. On the other hand, an increased bleeding risk is another clinical issue, particularly in subjects with liver cirrhosis, gastroesophageal varices, portal hypertension, thrombocytopenia and alcohol consumption. The introduction and progressively greater use of direct-acting antivirals (DAAs) (instead of protease and polymerase inhibitors) during the last decade has enabled a sustained virological response to be achieved in a significant percentage... (truncated)

Title: Hypersensitivity reactions to modern antiplatelet and anticoagulant drugs.
Anticoagulation and antiplatelet drugs are among the most commonly used medical drugs. In addition to the long-established heparins, hirudins, coumarins and antiplatelet drugs such as acetylsalicylic acid, numerous novel and predominantly synthetic pharmacologic agents have come onto the market in recent years. These new agents act at various sites in coagulation and have significantly broadened treatment options. Whilst immunological hypersensitivity reactions are on the whole rare, they have a considerable impact on patient management when they do occur. The present overview discusses the currently known hypersensitivity reactions to anticoagulant and antiplatelet agents, with particular attention to the newer substance classes... (truncated)

Title: The Role of Renal Replacement Therapy in the Management of Pharmacologic Poisonings.
Pharmacologic toxicities are common and range from mild to life-threatening. The aim of this study is to review and update the data on the role of renal replacement therapy (RRT) in the management of various pharmacologic poisonings. We aim to provide a focused review on the role of RRT in the management of pharmacological toxicities. Relevant publications were searched in MEDLINE with the following search terms alone or in combination: pharmacologic toxicity, hemodialysis, hemofiltration, renal replacement therapy, toxicology, poisonings, critical illness, and intensive care. The studies showed that a pharmacologic substance should meet several prerequisites to be deemed dialyzable. These... (truncated)

MESH:D002228 - carbenicillin

Summary:

LLM Prediction Score	0.390 (normalized)
LLM Confidence Score	0.960
Golden Answer (Severity Class)	0.375 (normalized)
Prediction Error	0.015

Retrieved Context:

Title: Anicteric carbenicillin hepatitis. Eight episodes in four patients.
Carbenicillin disodium was temporally associated with eight episodes of a mild reversible anicteric hepatitis characterized by nausea, vomiting, and a tender, somewhat enlarged liver. Serum glutamic and oxaloacetic transaminase as well as alkaline phosphatase levels rose, but serum bilirubin values remained normal. There usually were no signs of concomitant allergy to penicillin, and other penicillins could be given subsequently without ill effects. Biopsy specimens of the liver showed spotty liver cell necrosis with no cholestasis.

Title: Updating the side effects of the penicillins.
Use of the penicillins in patients may result in a variety of side effects in addition to the well known occasional allergic reactions. Some of these side effects may be quite serious. Treatment with the various penicillins, expecially in the large amounts presently prescribed, may be attended by immunologic reactions, such as nephritis, and by direct toxic effects which may involve the blood platelets, the neurones of the central nervous system, and perhaps the parenchymal cells of the liver.

Title: Use of cassette dosing in sandwich-cultured rat and human hepatocytes to identify drugs that inhibit bile acid transport.
Hepatocellular accumulation of bile acids due to inhibition of the canalicular bile salt export pump (BSEP/ABCB11) is one proposed mechanism of drug-induced liver injury (DILI). Some hepatotoxic compounds also are potent inhibitors of bile acid uptake by Na(+)-dependent taurocholate cotransporting polypeptide (NTCP/SLC10A1). This study used a cassette dosing approach in rat and human sandwich-cultured hepatocytes (SCH) to determine whether known or suspected hepatotoxic drugs inhibit bile acid transport individually or in combination. [(3)H]-Taurocholate served as the NTCP/BSEP probe substrate. Individually, cyclosporin A and rifampin decreased taurocholate in vitro biliary clearance (Cl(biliary)) and biliary excretion index (BEI) by more than 20%... (truncated)

Title: Adverse reactions to prolonged treatment with high doses of carbenicillin and ureidopenicillins.
Charts were reviewed for 63 patients whose chronic pseudomonas osteomyelitis was treated with high doses of extended-spectrum penicillins for prolonged periods. The incidence of untoward drug reactions was significantly higher than expected. Carbenicillin evoked adverse reactions in 22.8% of patients. However, most of these reactions were mild, and a change of drug was required in only 5.7% of cases. No adverse drug reactions were observed with cumulative doses of less than 750 g. In contrast to carbenicillin, the ureidopenicillins were associated with adverse reactions in 67.7% of patients; most reactions were moderate to severe in intensity; a cumulative dose of... (truncated)

Title: Hepatic safety of antibiotics used in primary care.
Antibiotics used by general practitioners frequently appear in adverse-event reports of drug-induced hepatotoxicity. Most cases are idiosyncratic (the adverse reaction cannot be predicted from the drug's pharmacological profile or from pre-clinical toxicology tests) and occur via an immunological reaction or in response to the presence of hepatotoxic metabolites. With the exception of trovafloxacin and telithromycin (now severely restricted), hepatotoxicity crude incidence remains globally low but variable. Thus, amoxicillin/clavulanate and co-trimoxazole, as well as flucloxacillin, cause hepatotoxic reactions at rates that make them visible in general practice (cases are often isolated, may have a delayed onset, sometimes appear only after cessation... (truncated)

MESH:D004077 - digoxin

Summary:

LLM Prediction Score	0.015 (normalized)
LLM Confidence Score	0.990
Golden Answer (Severity Class)	0.0 (normalized)
Prediction Error	0.015

Retrieved Context:

Title: Quinidine-induced hepatotoxicity revisited.
Although quinidine has been widely used since the beginning of the century, quinidine-induced hepatotoxicity has been recently reported in the literature. We describe a reversible case of quinidine-induced hepatotoxicity. A 62-y-old male with a past medical history of atrial flutter and adult onset diabetes was admitted to the hospital with a 3-d history of diarrhea, nausea, fever, chills and palpitations. Past medications included 7.5 mg glyburide daily for 4 y, 0.25 mg digoxin daily for 3 w, 324 mg quinidine gluconate 3 times daily for 2 w, and 150 mg papaverine daily for 2 y. On admission, liver enzyme levels... (truncated)

Title: A One-Two Punch: Hydralazine-Induced Liver Injury in a Recovering Ischemic Hepatitis.
A 77-year-old woman presented to the emergency department with a 2-day history of nausea and vomiting. Her medical history included diabetes mellitus, hypertension, atrial fibrillation, dilated cardiomyopathy, and coronary artery disease. Her home medications included aspirin, clopidogrel, warfarin, digoxin, metoprolol, losartan, simvastatin, isosorbide dinitrate, furosemide, and spironolactone. Initial physical examination showed blood pressure of 170/80 mm Hg with a heart rate of 69 beats per minute, otherwise unremarkable. Initial laboratory workup was significant for INR of 3.6, with slightly elevated troponin I and creatinine of 0.06 ng/mL and 1.4 mg/dL, respectively. The patient was admitted to the medicine floor. However,... (truncated)

Title: Spectrum of digoxin-induced ocular toxicity: a case report and literature review.
Digoxin intoxication results in predominantly digestive, cardiac and neurological symptoms. This case is outstanding in that the intoxication occurred in a nonagenarian and induced severe, extensively documented visual symptoms as well as dysphagia and proprioceptive illusions. Moreover, it went undiagnosed for a whole month despite close medical follow-up, illustrating the difficulty in recognizing drug-induced effects in a polymorbid patient.

Title: Digoxin mitigates diethylnitrosamine-induced acute liver injury in mice via limiting production of inflammatory mediators.
The cardiotonic digoxin has been recently shown to possess an anti-inflammatory potential in numerous metabolic and inflammatory disorders. However, data about digoxin's impact in the setting of acute liver injury and sterile inflammation are still limited. Here, we investigated the potential effect of digoxin pretreatments (0.25 and 0.5 mg/kg, oral) on the severity of acute hepatotoxicity in mice challenged with a single dose of diethylnitrosamine (DN; 150 mg/kg, intraperitoneal) for 24 h. Our results indicated that digoxin pretreatments dose-dependently mitigated DN-induced rise of hepatocellular injury parameters and necroinflammation scores. Digoxin, particularly at dose of 0.5 mg/kg, boosted the number of... (truncated)

Title: [HEPATOCELLULAR TOXICITY IN THE BACKGROUND OF AMIODARON-INDUCED THYROID DYSFUNCTION IN PATIENTS WITH ATRIAL FIBRILLATION].
The purpose of the work was to establish the frequency and conditions in which structural and functional changes of the liver might occur in case of long-term amiodarone use, depending on thyroid dysfunction. The study included 80 patients with cardiosclerosis with atrial fibrillation (AF). The patients were assigned to: group I (n=60) - received amiodarone at a maintenance dose for one year (on background of basic therapy); control group (CG) - patients (n=20) who received on the background of basic therapy digoxin and bisoprolol. Biochemical tests were conducted: fT3, thyroid-stimulating hormone (TSH), fT4, anti-TPO Ab, transaminases (ALT, AST), alkaline phosphatase... (truncated)

MESH:D009853 - omeprazole

Summary:

LLM Prediction Score	0.514 (normalized)
LLM Confidence Score	0.990
Golden Answer (Severity Class)	0.5 (normalized)
Prediction Error	0.014

Retrieved Context:

Title: [Evaluation of unexpected and toxic effects of omeprazole (Mopral) reported to the regional centers of pharmacovigilance during the first 22 postmarketing months].
Omeprazole has been marketed in France since 1989, for the healing of peptic ulcers, erosive reflux esophagitis and the Zollinger Ellison syndrome. It is a proton pump inhibitor which inhibits the acid secretion in the stomach. In the majority of the clinical trials, omeprazole has been found to be well tolerated: headache, dizziness, skin rash, constipation have just been noted. Since September 1989, 143 adverse reactions have been reported to pharmacovigilance centres and Astra France: 37 neurological and psychiatric side effects, especially confusion in patients with hepatic diseases and/or advanced age; 35 cutaneous reactions, generally rash and urticaria; 22 hematological... (truncated)

Title: A case of probable esomeprazole-induced transient liver injury in a pregnant woman with hyperemesis.
We report a case of 22-year-old primigravida presented to Women's Hospital - Hamad Medical Corporation emergency with severe epigastric pain, nausea, and vomiting. On admission, she was dehydrated with remarkably worsening symptoms. Laboratory findings revealed significantly elevated liver enzymes with unknown etiology. Her past medical history showed an admission for nausea and vomiting 3 weeks previously and she was discharged on antiemetics, and esomeprazole for the first time. Due to the predominantly elevated liver enzymes, the clinical pharmacist discussed the possibility of esomeprazole-induced adverse effects and suggested to suspend esomeprazole based on the evidence from literature review. The liver enzymes... (truncated)

Title: Hepatic effects of drugs used in the treatment of peptic ulcer disease.
Although one member of the H2 blocker family (oxmetidine) proved to be intrinsically hepatotoxic, overt hepatic injury attributable to cimetidine and ranitidine is decidedly rare, given the tens of millions of patients who have received these medications. Even considering the fact that serious adverse reactions may be underreported by as much as a factor of 100, the number of reports submitted to the FDA-DDE of possible hepatic toxicity associated with these agents supports this low incidence. Whether or not a similarly low incidence will be seen with famotidine and the newer H2 receptor antagonists still under investigation must await additional... (truncated)

Title: Omeprazole-induced hepatotoxicity? A case report.
We observed a serious symptomatic hepatocellular liver injury in an 85-year-old man treated with omeprazole for many years. Peak values for AST, ALT and AP were 1542 U/l (normal range 14-50), 1236 U/l (11-60) and 154 U/l (30-125) respectively. Abdominal CT scan was normal and viral serologic testing was negative. Omeprazole was discontinued and liver enzymes normalized in 12 days. The patient was known to suffer from ischemic heart disease and had had a myocardial infarction 6 months previously. He was reexposed to omeprazole and the level of liver enzymes rose again and normalized after stopping omeprazole. Despite the improvement... (truncated)

Title: Immune-mediated drug-induced liver injury secondary to Omeprazole: A case report.
Omeprazole is a rare cause of DILI with autoimmune hepatitis features and should be considered when seeing patients with acute liver injury. The causative drug should be promptly identified and discontinued to avoid any permanent liver damage.

MESH:C081643 - candesartan

Summary:

LLM Prediction Score	0.389 (normalized)
LLM Confidence Score	0.970
Golden Answer (Severity Class)	0.375 (normalized)
Prediction Error	0.014

Retrieved Context:

Title: Candesartan-induced cholestatic hepatitis: a case report.
We present the case of a 55-year-old male that was admitted to our hospital for cholestatic hepatitis. Other causes of hepatitis were excluded with imaging, laboratory and serologic tests. A liver biopsy was performed, which was compatible with toxicity-induced hepatitis. Re-assessing the patient, he mentioned the start of treatment with candesartan cilexetil 3 weeks prior to the onset of the symptoms. Candesartan was withdrawn, with a progressive improvement in cholestasis and complete normalization of liver biochemistry at 6 months. The patient was diagnosed with candesartan-induced cholestatic hepatitis, probable according to CIOMS/RUCAM scale.

Title: Candesartan Protects Against Cadmium-Induced Hepatorenal Syndrome by Affecting Nrf2, NF-κB, Bax/Bcl-2/Cyt-C, and Ang II/Ang 1-7 Signals.
Cadmium (Cd) is a serious pollutant in the environment. Candesartan is an angiotensin II (Ang II) receptor antagonist with promising diverse health benefits. The current study is planned to investigate the hepatorenal protective effects of candesartan against Cd-induced hepatic and renal intoxication. Our results demonstrated that candesartan effectively attenuated Cd-induced hepatorenal intoxication, as evidenced by improving hepatic and renal function biomarkers. Besides, candesartan reversed hepatic and renal histopathological abrasions induced by Cd toxicity. Candesartan antioxidant effect was mediated by Nrf2 activation. Also, candesartan suppressed hepatorenal inflammation by modulating NF-κB/IκB. Moreover, candesartan attenuated Cd hepatorenal apoptosis by upregulating Bcl-2 and downregulating... (truncated)

Title: Losartan-induced Severe Hepatic Injury: A Case Report and Literature Review.
Medications have been known to cause adverse drug reactions that affect various organs; these are mostly reversible reactions that improve with the cessation of the culprit medication. Losartan is an angiotensin-one receptor blocker which has been approved by the Food and Drug Administration (FDA) for the treatment of arterial hypertension. Fatigue, anemia, weakness, and cough are some of the common adverse effects of losartan. Acute hepatic injury has rarely been reported as an adverse effect of losartan. We report a case of a 61-year-old female with severe hepatic injury secondary to losartan use. None of the cases reported so far... (truncated)

Title: Beneficial effects of candesartan, an angiotensin-blocking agent, on compensated alcoholic liver fibrosis - a randomized open-label controlled study.
Recent studies have shown that the renin-angiotensin system is implicated in hepatic fibrogenesis in vitro and in vivo. However, no study was done in humans with alcoholic liver disease.

Title: Candesartan, rather than losartan, improves motor dysfunction in thioacetamide-induced chronic liver failure in rats.
Minimal hepatic encephalopathy is more common than the acute syndrome. Losartan, the first angiotensin-II receptor blocker (ARB), and candesartan, another widely-used ARB, have protected against developing fibrogenesis, but there is no clear data about their curative antifibrotic effects. The current study was designed to examine their effects in an already-established model of hepatic fibrosis and also their effects on the associated motor dysfunction. Low-grade chronic liver failure (CLF) was induced in 3-month old Sprague-Dawley male rats using thioacetamide (TAA, 50 mg·kg-1·day-1) intraperitoneally for 2 weeks. The TAA-CLF rats were randomly divided into five groups (n=8) treated orally for 14 days... (truncated)

MESH:D008466 - mechlorethamine

Summary:

LLM Prediction Score	0.361 (normalized)
LLM Confidence Score	0.970
Golden Answer (Severity Class)	0.375 (normalized)
Prediction Error	0.014

Retrieved Context:

Title: Hypersensitivity reactions to procarbazine with mechlorethamine, vincristine, and procarbazine chemotherapy in the treatment of glioma.
The authors report the clinical features of hypersensitivity reactions believed to result from procarbazine in eight patients treated with mechlorethamine, vincristine, and procarbazine (MOP) for high-grade glioma. There was one instance of hypersensitivity in 7 patients treated for recurrent disease and seven instances in 16 patients treated with an adjuvant protocol using MOP directly after surgery. Maculopapular rash was seen in seven of eight, fever was seen in four of eight, and reversible abnormal liver function test results were seen in three of four patients. Pulmonary toxic effects were seen in five of eight patients and consisted of isolated interstitial... (truncated)

Title: Low-Dose Cyclophosphamide Associated With Hyponatremia and Hepatotoxicity.
Cyclophosphamide (CY) is an alkylating agent often used as a chemotherapeutic agent, with increasing use as an immunosuppressant. Cyclophosphamide has many established adverse effects, including hyponatremia and limited reports of hepatotoxicity, particularly in high-dose treatment. A case of simultaneous hyponatremia and acute liver injury associated with the initiation of cyclophosphamide two weeks prior is discussed here. A 73-year-old male with acquired hemophilia A/factor VIII deficiency presented to the emergency department (ED) with four days of hip pain and was found to have jaundice and confusion. Laboratory evaluation demonstrated hyponatremia and an acute liver injury associated with his recent cyclophosphamide use.... (truncated)

Title: Chemotherapy induced liver abnormalities: an imaging perspective.
Treating patients undergoing chemotherapy who display findings of liver toxicity, requires a solid understanding of these medications. It is important for any clinician to have an index of suspicion for liver toxicity and be able to recognize it, even on imaging. Cancer chemotherapy has evolved, and newer medications that target cell biology have a different pattern of liver toxicity and may differ from the more traditional cytotoxic agents. There are several hepatic conditions that can result and keen clinical as well as radiographic recognition are paramount. Conditions such as sinusoidal obstructive syndrome, steatosis, and pseudocirrhosis are more commonly associated with... (truncated)

Title: Particulate and drug-induced toxicity assessed in novel quadruple cell human primary hepatic disease models of steatosis and pre-fibrotic NASH.
In an effort to replace, reduce and refine animal experimentation, there is an unmet need to advance current in vitro models that offer features with physiological relevance and enhanced predictivity of in vivo toxicological output. Hepatic toxicology is key following chemical, drug and nanomaterials (NMs) exposure, as the liver is vital in metabolic detoxification of chemicals as well as being a major site of xenobiotic accumulation (i.e., low solubility particulates). With the ever-increasing production of NMs, there is a necessity to evaluate the probability of consequential adverse effects, not only in health but also in clinically asymptomatic liver, as part... (truncated)

Title: Implications of Breast Cancer Chemotherapy-Induced Inflammation on the Gut, Liver, and Central Nervous System.
Breast Cancer is still one of the most common cancers today; however, with advancements in diagnostic and treatment methods, the mortality and survivorship of patients continues to decrease and increase, respectively. Commonly used treatments today consist of drug combinations, such as doxorubicin and cyclophosphamide; docetaxel, doxorubicin, and cyclophosphamide; or doxorubicin, cyclophosphamide, and paclitaxel. Although these combinations are effective at destroying cancer cells, there is still much to be understood about the effects that chemotherapy can have on normal organ systems such as the nervous system, gastrointestinal tract, and the liver. Patients can experience symptoms of cognitive impairments or "chemobrain", such... (truncated)

MESH:C115647 - eletriptan

Summary:

LLM Prediction Score	0.388 (normalized)
LLM Confidence Score	0.980
Golden Answer (Severity Class)	0.375 (normalized)
Prediction Error	0.013

Retrieved Context:

Title: Targeted CGRP Small Molecule Antagonists for Acute Migraine Therapy.
Migraine is a highly prevalent, severe, and disabling neurological condition with a significant unmet need for effective acute therapies. Patients (~50%) are dissatisfied with their currently available therapies. Calcitonin gene-related peptide (CGRP) has emerged as a key neuropeptide involved in the pathophysiology of migraines. As reviewed in this manuscript, a number of small molecule antagonists of the CGRP receptor have been developed for migraine therapy. Incredibly, the majority of the clinical trials conducted have proven positive, demonstrating the importance of this signalling pathway in migraine. Unfortunately, a number of these molecules raised liver toxicity concerns when used daily for as... (truncated)

Title: Interactions between Food and Drugs, and Nutritional Status in Renal Patients: A Narrative Review.
Drugs and food interact mutually: drugs may affect the nutritional status of the body, acting on senses, appetite, resting energy expenditure, and food intake; conversely, food or one of its components may affect bioavailability and half-life, circulating plasma concentrations of drugs resulting in an increased risk of toxicity and its adverse effects, or therapeutic failure. Therefore, the knowledge of these possible interactions is fundamental for the implementation of a nutritional treatment in the presence of a pharmacological therapy. This is the case of chronic kidney disease (CKD), for which the medication burden could be a problem, and nutritional therapy plays... (truncated)

Title: Fungal infections: their diagnosis and treatment in transplant recipients.
Systemic fungal infections typically occur in individuals who are seriously ill with recognized risk factors such as those frequently found in transplant recipients. Unfortunately, they are often diagnosed late, when the efficacy of the available treatments is low, often less than 50%, and the cost in terms of lives lost, hospital length of stay, and total hospital costs is substantially increased. The application of antifungal therapies associated with reported efficacy rates greater than 50% are those used prophylactically. When used prophylactically, these infections are reduced in greater than 95% of the expected cases. The choice of a prophylactic agent should... (truncated)

Title: Pharmacokinetics and pharmacodynamics of antifungals in children and their clinical implications.
Invasive fungal infections are a significant cause of morbidity and mortality in children. Successful management of these systemic infections requires identification of the causative pathogen, appropriate antifungal selection, and optimisation of its pharmacokinetic and pharmacodynamic properties to maximise its antifungal activity and minimise toxicity and the emergence of resistance. This review highlights salient scientific advancements in paediatric antifungal pharmacotherapies and focuses on pharmacokinetic and pharmacodynamic studies that underpin current clinical decision making. Four classes of drugs are widely used in the treatment of invasive fungal infections in children, including the polyenes, triazoles, pyrimidine analogues and echinocandins. Several lipidic formulations of... (truncated)

Title: Pharmacology.
Headache treatment has been based primarily on experiences with non-specific drugs such as analgesics, non-steroidal anti-inflammatory drugs, or drugs that were originally developed to treat other diseases, such as beta-blockers and anticonvulsant medications. A better understanding of the basic pathophysiological mechanisms of migraine and other types of headache has led to the development over the past two decades of more target-specific drugs. Since activation of the trigeminovascular system and neurogenic inflammation are thought to play important roles in migraine pathophysiology, experimental studies modeling those events successfully predicted targets for selective development of pharmacological agents to treat migraine. Basically, there are... (truncated)

MESH:C008924 - pirprofen

Summary:

LLM Prediction Score	0.988 (normalized)
LLM Confidence Score	0.680
Golden Answer (Severity Class)	1.0 (normalized)
Prediction Error	0.012

Retrieved Context:

Title: Fatal acute liver failure associated with pirprofen. Report of a case and a review of the literature.
This report is about a 71-yr-old woman who suffered from acute liver failure, induced by the nonsteroidal antiinflammatory drug, pirprofen. She presented with jaundice 6 weeks after starting treatment with 800 mg pirprofen daily. She is the fifth patient described in the literature to die from pirprofen-induced hepatotoxicity.

Title: Pirprofen-induced fulminant hepatitis.
We report the cases of 2 female patients aged 69 and 61 yr, suffering from fulminant hepatitis induced by pirprofen, a new nonsteroidal antiinflammatory drug. The duration of pirprofen administration before the onset of hepatitis was long, 7 and 9 mo, respectively. Hepatitis was not preceded or accompanied by hypersensitivity manifestations. The liver lesion consisted of massive, predominantly centrilobular hepatic cell necrosis and microvesicular steatosis. One patient died of liver failure. Although the risk of fulminant hepatitis is very low, we recommend that, in patients taking pirprofen for more than 2 mo and complaining of asthenia, nausea, or vomiting, serum... (truncated)

Title: [Hepatitis due to pirprofen; 3 cases, 1 fatality].
We report three cases of jaundice in women taking 800 mg pirprofen.day-1 during 6 weeks to 4 months and a half. Two of them presented cutaneous side effects: one case was rapidly better with corticosteroids. Fatal case possibility incite to transaminase surveillance.

Title: [Hepatitis due to nonsteroidal anti-inflammatory agents].
The extended prescription of non-steroidal anti-inflammatory drugs in medical practice involve numerous adverse effects. Among them, hepatic injuries, rather uncommon, are very diverse with regard to clinical type and evolution scheme, according to the derivatives used. Salicylates, when taken at high doses, increase serum transaminases, mostly without overt clinical symptoms. Phenylbutazone is obviously hepatotoxic: it induces cytolytic hepatitis, in some cases with fatal issue. Among the indole derivatives, indometacine was involved, especially in children; mixed hepatitis have been noted during sulindac therapy, mostly with favourable outcome. In the group of propionic acid derivatives, ibuprofen, pirprofen and naproxen have been implicated... (truncated)

Title: [Hepatitis secondary to current non-steroidal anti-inflammatory agents].
The authors report 83 cases of acute hepatitis secondary to non steroid anti-inflammatory drugs (NSAID), published in the literature. The NSAID in question are: niflumic acid, tolfenamic acid, diclofenac, fenoprofen, ibuprofen, indomethacin, naproxen, piroxicam, pirprofen and sulindac. Six deaths are directly ascribed to NSAID: although rare, these forms of hepatitis deserve therefore to be reported. They are usually mixed immuno-allergic forms of hepatitis (cytolytic as well as cholestatic). They often affect elderly women taking multiple medications. Monitoring of the liver function tests is necessary, during prolonged treatment with NSAID, especially during the first six months.

MESH:D000069582 - eszopiclone

Summary:

LLM Prediction Score	0.386 (normalized)
LLM Confidence Score	0.990
Golden Answer (Severity Class)	0.375 (normalized)
Prediction Error	0.011

Retrieved Context:

Title: Case report of acute liver injury caused by the eszopiclone in a patient with chronic liver disease.
Eszopiclone, sold under the brand name Lunesta, is a new type of non-benzodiazepine hypnotic. Eszopiclone is a zopiclone dextrorotation, which is classified as a cyclopyrrolone. It functions by binding gamma-aminobutyric acid (GABA) receptors. Compared with benzodiazepines hypnotics, eszopiclone has higher selectivity for certain subunits of the GABA(A) receptor. So far, there are no reports about the elevation of serum enzymes or severe liver injury caused by eszopiclone. Here, we present a case report of acute liver injury following eszopiclone treatment in a patient with chronic hepatitis B virus (HBV).

Title: Eszopiclone.
The pharmacology, pharmacokinetics, indications, clinical efficacy, adverse effects, drug interactions, dosing, and administration of eszopiclone are discussed.

Title: Eszopiclone for late-life insomnia.
Insomnia, the most common sleep disturbance in later life, affects 20%-50% of older adults. Eszopiclone, a short-acting nonbenzodiazepine hypnotic agent developed for the treatment of insomnia, has been available in Europe since 1992 and in the US since 2005. Although not yet evaluated for transient insomnia in older adults, eszopiclone has been shown to be safe and efficacious for short-term treatment (2 weeks) of chronic, primary insomnia in older adults (64-91 years). Clinical studies in younger adults (mean = 44 years) have shown eszopiclone can be used for 6-12 months without evidence of problems. Because the oldest participant in these... (truncated)

Title: Effects of eszopiclone on safety, subjective measures of efficacy, and quality of life in elderly and nonelderly Japanese patients with chronic insomnia, both with and without comorbid psychiatric disorders: a 24-week, randomized, double-blind study.
The primary objective of this study was to evaluate long-term (24-week) safety of eszopiclone in elderly and nonelderly Japanese patients with chronic insomnia. The secondary objectives were to evaluate short-term (4-week) efficacy and to assess for rebound insomnia or dependence after long-term treatment.

Title: Psychological and psychiatric aspects of treatment of obesity and nonalcoholic fatty liver disease.
Chronic illnesses incur a tremendous cost to American lives in dollars and quality of life. Outcomes in these illnesses are often affected by psychological, behavioral, and pharmacologic issues related to mental illness and psychological symptoms. This article focuses on psychological and psychiatric issues related to the treatment of obesity and nonalcoholic fatty liver disease (NAFLD), including available weight-loss interventions, the complex relationship between psychiatric disorders and obesity, and special considerations regarding use of psychiatric drugs in patients with or at risk for NAFLD and obesity. Recommendations for collaborative care of individuals with comorbid NAFLD and psychological disorders/symptoms are discussed.

MESH:C106276 - sitaxsentan

Summary:

LLM Prediction Score	0.989 (normalized)
LLM Confidence Score	0.900
Golden Answer (Severity Class)	1.0 (normalized)
Prediction Error	0.011

Retrieved Context:

Title: Bioactivation of sitaxentan in liver microsomes, hepatocytes, and expressed human P450s with characterization of the glutathione conjugate by liquid chromatography tandem mass spectrometry.
Sitaxentan is a selective endothelin-A receptor antagonist that was marketed as Thelin in several European countries and Canada for pulmonary arterial hypertension. Sitaxentan was undergoing further clinical trials in the United States but due to four deaths and one case of liver transplantation from severe liver toxicity that appeared to be idiosyncratic in nature, it was withdrawn worldwide in December, 2010. Sitaxentan contains a 1,3-benzodioxole ring that undergoes enzymatic demethyleneation to an ortho-catechol metabolite that can further oxidize to a reactive ortho-quinone metabolite. Here, we report the detection and mass spectral characterization of a glutathione conjugate of this sitaxentan quinone... (truncated)

Title: A review of sitaxsentan sodium in patients with pulmonary arterial hypertension.
Pulmonary arterial hypertension (PAH) is a life threatening, progressive condition which eventually leads to fatal right heart failure. Endothelin-1 (ET-1), a potent vasoconstrictor peptide, is increased in the pulmonary arteries of patients with pulmonary hypertension. Endothelin-1 acts through the stimulation of 2 subtypes of receptors (endothelin receptor subtypes A [ET(A)] and B [ET(B)]). In PAH patients, ETRAs block the deleterious vasoconstrictor effects of ET-1, and ETRA treatment in PAH patients has been shown to be safe and efficacious. Sitaxsentan is an orally active, highly ET(A) selective ETRA that, in clinical trials, has demonstrated improvements in exercise capacity, functional class and... (truncated)

Title: An overview of the preclinical toxicity and potential carcinogenicity of sitaxentan (Thelin®), a potent endothelin receptor antagonist developed for pulmonary arterial hypertension.
Sitaxentan (Thelin®), an endothelin receptor antagonist with a long duration of action and high specificity for the endothelin receptor A subtype, was used to treat pulmonary arterial hypertension. It was withdrawn from the market due to an idiosyncratic risk of drug-induced liver injury identified from emerging clinical trial data and clinical case reports. The preclinical safety profile of sitaxentan is presented, including single- and repeat-dose toxicity in mice, rats, and dogs and carcinogenicity in mice and rats. Sitaxentan-related adverse effects included coagulopathy in rats and dogs, increased serum alkaline phosphatase activity in mice and dogs, and hepatic hypertrophy in all... (truncated)

Title: Iontophoresis of endothelin receptor antagonists in rats and men.
The treatment of scleroderma-related digital ulcers is challenging. The oral endothelin receptor antagonist (ERA) bosentan has been approved but it may induce liver toxicity. The objective of this study was to test whether ERAs bosentan and sitaxentan could be locally delivered using iontophoresis.

Title: Multiple compound-related adverse properties contribute to liver injury caused by endothelin receptor antagonists.
Drug-induced liver injury has been observed in patients treated with the endothelin receptor antagonists sitaxentan and bosentan, but not following treatment with ambrisentan. The aim of our studies was to assess the possible role of multiple contributory mechanisms in this clinically relevant toxicity. Inhibition of the bile salt export pump (BSEP) and multidrug resistance-associated protein 2 was quantified using membrane vesicle assays. Inhibition of mitochondrial respiration in human liver-derived HuH-7 cells was determined using a Seahorse XF(e96) analyzer. Cytochrome P450 (P450)-independent and P450-mediated cell toxicity was assessed using transfected SV40-T-antigen-immortalized human liver epithelial (THLE) cell lines. Exposure-adjusted assay ratios were... (truncated)

MESH:D002438 - cefoperazone

Summary:

LLM Prediction Score	0.364 (normalized)
LLM Confidence Score	0.970
Golden Answer (Severity Class)	0.375 (normalized)
Prediction Error	0.011

Retrieved Context:

Title: Cefoperazone for empiric therapy in patients with impaired renal function.
Thirty-five patients with serious infections and impaired renal function were treated empirically with 2 to 8 g of cefoperazone per day. Infections included sepsis in 14, nonbacteremic urinary infections in nine, pneumonia in five, intra-abdominal infection in five, fasciitis in one, and malignant otitis externa in one. The average age of this group was 64.3 years, 25 had ultimately fatal underlying diseases, and their average serum creatinine level was 5.2 mg/dl. Infections were caused by Enterobacteriaceae in 23 patients, Streptococcus faecalis in five, Pseudomonas aeruginosa in four, Staphylococcus aureus in four, Hemophilus influenzae in three, and Staphylococcus epidermidis, Streptococcus pneumoniae,... (truncated)

Title: Cefoperazone in the treatment of severe or complicated infections.
We report the use of cefoperazone in 62 cases of serious infection, most of which occurred in patients with renal impairment. 43 severe or complicated urinary tract infections, 11 cases of pneumonia and 8 with other severe sepsis were treated with cefoperazone 1 to 2 g twice daily usually for 5 to 10 days. Of the patients with urinary tract infection, all who were symptomatic showed a rapid clinical response; 26 (61%) were cured including 11 of 16 with chronic renal failure; 12 relapsed and 5 were reinfected with a different pathogen. All of these patients were infected by organisms... (truncated)

Title: Cefoperazone versus cefotaxime, plus amikacin or sisomicin, in fever and infection in hematologic granulocytopenic patients.
Forty patients with leukemia or aplastic anemia were randomized to receive one of the following antibiotic regimens at the onset of fever during granulocytopenia: cefoperazone + amikacin (regimen A), cefoperazone + sisomicin (regimen B), cefotaxime + amikacin (regimen C), cefotaxime + sisomicin (regimen D). All patients were receiving gut decontamination at the time of randomization. Patients were monitored twice weekly with swabs and cultures for bacteria and fungi. Overall, there were 56 febrile episodes: 31 were proven bacterial, 3 were probable, and 16 were of unknown origin. Response rates were comparable in all 4 treatment regimens: 90%, 91%, 92% and... (truncated)

Title: The influence of chronic lobular hepatitis on pharmacokinetics of cefoperazone--a novel galactose single-point method as a measure of residual liver function.
Cefoperazone is a semisynthetic cephalosporin antibiotic containing a piperazine side chain, which results in antipseudomonal activity. Unlike the other cephalosporins, it is mainly cleared by the liver (60-80%) and it may be more sensitive to changes in the liver function and/or plasma protein binding than other cephalosporins, which are not primarily cleared by the liver. In order to study the influence of chronic lobular hepatitis on the pharmacokinetics of cefoperazone, a dose of 1 g of cefoperazone was administered to 11 normal, healthy volunteers and 16 subjects with chronic lobular hepatitis. In each volunteer or patient, a novel galactose single-point... (truncated)

Title: Cefoperazone pharmacokinetics in normal subjects and patients with cirrhosis.
The pharmacokinetics of cefoperazone were studied and compared in six normal subjects and six patients with severe liver disease. All subjects received a 2-g intravenous infusion of cefoperazone over 15 min. Significantly different results were noted between normal subjects and patients with cirrhosis (range [mean]) for the following: peak serum concentrations (203 to 345 [239] versus 82 to 206 [141] micrograms/ml; P less than 0.01); serum beta half-lives (1.0 to 1.8 [1.5] versus 2.3 to 9.9 [4.5] h; P less than 0.05); renal excretion (17 to 27 [21] versus 32 to 60 [50]%; P less than 0.01); and apparent volumes... (truncated)

MESH:D008558 - melphalan

Summary:

LLM Prediction Score	0.636 (normalized)
LLM Confidence Score	0.980
Golden Answer (Severity Class)	0.625 (normalized)
Prediction Error	0.011

Retrieved Context:

Title: Veno-occlusive disease of the liver after busulfan, melphalan, and thiotepa conditioning therapy: incidence, risk factors, and outcome.
The purpose of this study was to determine the incidence of veno-occlusive disease (VOD) after a high-dose regimen of busulfan, melphalan, and thiotepa and the risk factors for a more severe outcome. We followed 253 consecutive patients with malignant disorders who received autologous transplants after stem cell harvest followed by 12 mg/kg busulfan, 100 mg/m2 melphalan, and 500 mg/m2 thiotepa. Diagnosis of VOD was based on weight gain, hepatomegaly, and jaundice. Risk factors for moderate or severe VOD were identified using logistic regression models. VOD occurred in 70 of 253 patients (28%), of whom 31 (12%) had moderate and 11... (truncated)

Title: Safety of Percutaneous Hepatic Perfusion with Melphalan in Patients with Unresectable Liver Metastases from Ocular Melanoma Using the Delcath Systems' Second-Generation Hemofiltration System: A Prospective Non-randomized Phase II Trial.
To investigate the safety and toxicity of percutaneous hepatic perfusion with melphalan (M-PHP) with the Delcath Systems' second-generation (GEN 2) filter and compare the outcomes with historical data from studies using the first-generation filter.

Title: Correlation between melphalan pharmacokinetics and hepatic toxicity following hyperthermic isolated liver perfusion for unresectable metastatic disease.
In the present work, we report on the results of our pilot study of hyperthermic isolated hepatic perfusion (IHP) with melphalan alone for patients with unresectable metastatic liver tumors refractory to conventional treatments, with particular regard to the correlation between pharmacokinetic findings and hepatic toxicity.

Title: Role of tumor necrosis factor on toxicity and cytokine production after isolated hepatic perfusion.
Isolated limb or liver perfusion with tumor necrosis factor (TNF) and melphalan results in regression of advanced cancers in the majority of treated patients. However, the contribution of TNF to the efficacy of isolation perfusion with melphalan has not been demonstrated conclusively in random assignment trials. Furthermore, TNF is an inflammatory cytokine and may be associated with significant systemic and regional toxicity. This study was conducted to characterize the toxicity and secondary cytokine production attributable to TNF by comparing these parameters in patients undergoing isolated hepatic perfusion (IHP) using melphalan with or without TNF.

Title: Reduced liver uptake of arterially infused melphalan during retrograde rat liver perfusion with unaffected liver tumor uptake.
Isolated hepatic perfusion (IHP) with melphalan is used for patients with nonresectable metastases confined to the liver. To improve the efficacy of IHP and to reduce the toxicity to the liver, reversion (retrograde perfusion) of the bloodstream through the liver in a rat model was studied. For liver tumor induction male WAG/Rij rats were inoculated with CC531 cells, a colorectal tumor cell line. After 11 to 12 days the tumor-bearing rat livers were perfused by single-pass perfusion through either the portal (orthograde) or caval vein (retrograde) for different time periods. During perfusion melphalan (160 Schultze) was infused in the hepatic... (truncated)

MESH:D008797 - metyrapone

Summary:

LLM Prediction Score	0.009 (normalized)
LLM Confidence Score	0.940
Golden Answer (Severity Class)	0.0 (normalized)
Prediction Error	0.009

Retrieved Context:

Title: Current approaches to the pharmacological management of Cushing's disease.
If treatment of Cushing's disease (CD) by surgery is not successful, medical therapy is often required. Long-term use of metyrapone is limited by hirsutism and hypertension and escape because of increased ACTH levels. Although ketoconazole can normalize cortisol levels in 50%, liver toxicity limits its use. Mitotane, an adrenolytic agent, has had minimal use for benign disease. Etomidate is useful when rapid reduction in cortisol levels is needed. Cabergoline can normalize cortisol levels in CD in about one-third of patients and is well tolerated. Pasireotide can normalize cortisol levels in CD in about 25% but causes worsening of glucose tolerance... (truncated)

Title: Steroid biosynthesis inhibitors in the therapy of hypercortisolism: theory and practice.
Cushing's syndrome is a rare disease with significant morbidity and mortality. Surgical intervention represents the most effective treatment option in both adrenocorticotropin-dependent and -independent forms of hypercortisolism. It is not uncommon, however, that surgery fails to cure or control the disease. Pharmacotherapy with drugs inhibiting steroid biosynthesis can be effectively used in these cases in order to alleviate symptoms or even to induce chemical adrenalectomy. A few drugs inhibiting single or multiple steps in adrenal steroid biosynthesis can be used in clinical practice. Drugs predominantly inhibiting single enzymatic steps include the 11beta-hydroxylase inhibitor metyrapone and the 3beta-hydroxysteroid dehydrogenase inhibitor trilostane,... (truncated)

Title: Hormone secretion in alcohol-induced pseudo-Cushing's syndrome. Differential diagnosis with Cushing disease.
We describe the clinical and biochemical characteristics of alcohol-induced pseudo-Cushing's syndrome in two patients with signs and symptoms of Cushing's syndrome and slight disturbances in liver function. An insufficient suppression of plasma cortisol to the overnight administration of 1 mg of dexamethasone was accompanied by an absent diurnal rhythm of plasma cortisol and an increased cortisol secretion rate. The plasma cortisol levels at 8 AM normalized during hospital admission (ie, alcohol withdrawal) at the same rate or parallel with the serum gamma-glutamyl transferase concentration. A normal increase of plasma cortisol, adrenocorticotropic hormone, and growth hormone in response to an insulin... (truncated)

Title: Hepatotoxicity of piperazine designer drugs: Comparison of different in vitro models.
Piperazine derived drugs emerged on the drug market in the last decade. The aim of this study was to investigate in vitro the potential hepatotoxicity of the designer drugs N-benzylpiperazine (BZP), 1-(3-trifluoromethylphenyl)piperazine (TFMPP), 1-(4-methoxyphenyl)piperazine (MeOPP) and 1-(3,4-methylenedioxybenzyl)piperazine (MDBP) in two human hepatic cell lines (HepaRG and HepG2) and in primary rat hepatocytes. Cell death was evaluated by the MTT assay, after 24 h-incubations. Among the tested drugs, TFMPP was the most cytotoxic. HepaRG cells and primary hepatocytes revealed to be the most and the least resistant cellular models, respectively. To ascertain whether the CYP450 metabolism could explain their higher susceptibility,... (truncated)

Title: Fatal liver failure due to ketoconazole treatment of a girl with Cushing's syndrome.
A patient is reported who developed fatal liver failure on ketoconazole treatment for Cushing's syndrome. It is recommended that metyrapone be used when hypercortisolism has to be controlled as a temporary measure in childhood and adolescence.

MESH:D000077288 - troglitazone

Summary:

LLM Prediction Score	0.992 (normalized)
LLM Confidence Score	0.970
Golden Answer (Severity Class)	1.0 (normalized)
Prediction Error	0.008

Retrieved Context:

Title: Troglitazone induces a rapid drop of mitochondrial membrane potential in liver HepG2 cells.
Troglitazone, a thiazolidinedione containing compound, was widely used to treat non-insulin dependent-diabetes. Unfortunately, troglitazone was associated with a sporadic liver toxicity that led to a cessation of its use clinically. Here we show that troglitazone induces a rapid and dose-dependent drop of mitochondrial membrane potential in liver HepG2 cells. The decrease in mitochondrial membrane potential induced by 100 microM troglitazone was completed after 5 min and similar in magnitude to that caused by carbonyl cyanide m-chloro phenylhydrazone. The troglitazone-induced loss of mitochondrial membrane potential preceded changes in cell permeability and cell count. In addition, troglitazone-induced a rise of intracellular calcium,... (truncated)

Title: Metabolic and non-metabolic factors determining troglitazone hepatotoxicity: a review.
Troglitazone (TGZ), a thiazolidinedione class of antidiabetic agent, causes serious idiosyncratic hepatotoxicity. TGZ is metabolized into reactive metabolites that covalently bind to cellular macromolecules, one of which is oxidation at the chromane ring, a unique structure of TGZ, and another involves oxidative cleavage of the thiazolidinedione ring, a structure common to less hepatotoxic antidiabetics, rosiglitazone and pioglitazone. TGZ is cytotoxic to HepG2 cells and rat and human hepatocytes. However, the role of the reactive metabolite on the TGZ toxicity is controversial, because there was no correlation of the generation of the reactive metabolites with susceptibility to the TGZ cytotoxicity, and... (truncated)

Title: Thiazolidinediones and liver toxicity.
Thiazolidinediones or glitazones specifically target insulin resistance. They have proven efficacy for reducing plasma glucose levels of type 2 diabetic patients treated with diet alone, sulphonylureas, metformin or insulin. In addition, they may be associated to some improvement of cardiovascular risk profile. However, troglitazone, the first compound approved by the FDA in the US, proved to be hepatotoxic and was withdrawn from the market after the report of several dozens of deaths or cases of severe hepatic failure requiring liver transplantation. It remains unclear whether or not hepatotoxicity is a class effect or is related to the unique tocopherol side... (truncated)

Title: Cytotoxicity and apoptosis produced by troglitazone in human hepatoma cells.
Troglitazone is an antidiabetic agent that increases the insulin sensitivity of target tissues in non-insulin-dependent diabetes mellitus. It has been reported that troglitazone causes severe hepatic injury in certain individuals. In the present study, the mechanism for the hepatic injury by troglitazone was investigated with human hepatoma cell lines. HepG2 cells were incubated with troglitazone, its metabolites M-1 (sulfate), M-2 (gulucronide), M-3 (quinone), and other thiazolidinediones (pioglitazone and rosiglitazone). Troglitazone exhibited time- and concentration-dependent cytotoxicity and M-3 also exhibited weak cytotoxicity. Troglitazone induced apoptotic cell death characterized by internucleosomal DNA fragmentation and nuclear condensation. As other thiazolidinediones, pioglitazone and rosiglitazone,... (truncated)

Title: Mitochondrial dysfunction and delayed hepatotoxicity: another lesson from troglitazone.
Troglitazone was approved for treatment of type 2 diabetes mellitus, but by 2000 it had been removed from all world markets due to severe drug-induced liver injury. Even today, we still do not know how many patients sustained a long-term liver injury. No system is in place to acquire that knowledge. Regarding toxicity mechanisms, controversy persists as to which ones are class effects of thiazolidinediones (TZDs) and which are unique to troglitazone. This study aims to provide long-term outcome data and new insights on mechanisms of troglitazone-induced liver injury.

MESH:D011433 - propranolol

Summary:

LLM Prediction Score	0.383 (normalized)
LLM Confidence Score	0.990
Golden Answer (Severity Class)	0.375 (normalized)
Prediction Error	0.008

Retrieved Context:

Title: [Drug-induced liver damage from the clinical viewpoint].
Extensive drug-induced hepatic injury leading up to jaundice occurs relatively rarely. Generally it is not predictable, independent on dosage and irreproductible in animals. As a rule you find it in less than 0.1% of the patients taking the drug, whereas the degree may range up to heavy or lethal (e.g. for isoniazid, methyldopa, halothane). Pathogenetically reactive metabolites capable of producing cytotoxic and/or immune reactions may play a role. Obviously higher age, female sex and endogenous or exogenous alterations in hepatocellular drug metabolism are disposing factors. Drugs, to which our patients most frequently reacted, were dihydralazine alone or in combination with... (truncated)

Title: Fulminant hepatitis after carbimazole and propranolol administration.
We report the case of a patient who developed jaundice, encephalopathy, a marked increase in serum aminotransferase activity and a decrease in prothrombin and proaccelerin levels, after 6 weeks' treatment with carbimazole and propranolol for hyperthyroidism. The patient ultimately underwent orthotopic liver transplantation. This case strongly suggests that carbimazole may occasionally induce fulminant hepatitis and that careful monitoring of liver enzymes may be useful during the treatment of hyperthyroidism with this drug.

Title: The novel effect of propranolol in glibenclamide induced hepatotoxicity.
Glibenclamide (GBC) has been associated with hepatotoxicity in humans. This study conducted on rabbits to evaluate the hepatotoxicity of GBC alone and in combination therapy with propranolol (PPL). Liver enzymes like alanine transaminase (ALT), alkaline phosphatase (ALP) and gamma-glutamyltransferase (γ-GT) and bilirubin (BRB) are used to evaluate hepatotoxicity associated with GBC. Histological findings, micrometry and scanning electron microscopy (SEM) used to find hepatotoxicity by GBC and with PPL. GBC caused significant elevation of liver functions as compared to control ($p<0.005$). PPL reduced the level of serum ALT, ALP, γGT and BRB when administered with GBC ($p<0.005$). The results prevailed that... (truncated)

Title: [The so-called dihydralazine hepatitis. A contribution to the pathogenesis].
Liver injuries following application of dihydralazine in 14 patients, ten of them with the typical pattern of perivenous liver cell necrosis, are reported. The other 4 patients, all having been exposed to additional hepatotoxic substances, histologically showed mixed patterns of tissue injury in liver biopsy. Moreover, in 6 of 14 patients there were diseases in the upper abdomen and/or metabolic disorders as well as an exposition to chemical hepatotoxic substances, or an additional application of potential hepatotoxic drugs. 9 of 14 patients had been treated with propranolol and dihydralazine simultaneously. One female patient showed the typical morphologic pattern of 'dihydralazine-damage'... (truncated)

Title: Propranolol prevents liver cirrhosis by inhibiting hepatic stellate cell activation mediated by the PDGFR/Akt pathway.
Propranolol is known to reduce portal pressure by decreasing blood flow to the splanchnic circulation and the liver. However, it is unknown if propranolol improves fibrogenesis and sinusoidal remodeling in the cirrhotic liver. The aim of this study was to investigate the therapeutic effects of propranolol on carbon tetrachloride (CCl₄)-induced liver fibrosis in a mouse model and the intrinsic mechanisms underlying those effects. In this study, a hepatic cirrhosis mouse model was induced by CCl₄ administration for 6 weeks. Propranolol was simultaneously administered orally in the experimental group. Liver tissue and blood samples were collected for histological and molecular analyses. LX-2... (truncated)

MESH:C042852 - perflutren

Summary:

LLM Prediction Score	0.008 (normalized)
LLM Confidence Score	0.940
Golden Answer (Severity Class)	0.0 (normalized)
Prediction Error	0.008

Retrieved Context:

Title: Subchronic oral toxicity and metabolite profiling of the p53 stabilizing agent, CP-31398, in rats and dogs.
CP-31398 (N'-[2-[2-(4-methoxyphenyl)ethenyl]-4-quinazolinyl]-N,N-dimethyl-1,3-propanediamine dihydrochloride) is a styrylquinazoline that stabilizes the DNA binding conformation of p53, thereby maintaining the activity of p53 as a transcription factor and tumor suppressor. In consideration of the potential use of p53 stabilizers for cancer prevention and therapy, 28-day studies (with recovery) were performed to characterize the toxicity of CP-31398 in rats and dogs. In the rat study, groups of 15 CD rats/sex received daily gavage exposure to CP-31398 at 0, 40, 80, or 160mg/kg/day (0, 240, 480, or 960mg/m(2)/day). In the dog study, groups of five beagle dogs received daily gavage exposure to CP-31398 at 0,... (truncated)

Title: Hepatoprotective and antidiabetic effects of Pistacia lentiscus leaf and fruit extracts.
Pistacia lentiscus (Anacardiaceae) is commonly used in folk medicine to treat various diseases. The aim of the present study was to evaluate the hepatoprotective and antioxidant activities of extracts of P. lentiscus leaves (PL) and fruits (PF) against experimentally induced liver damage. Furthermore, characterization of extracts was attempted by a spectroscopic methodology (Fourier transform infrared spectroscopy) and high-performance liquid chromatography with diode array detection analysis. A hepatoprotective potential against paracetamol [165 mg/kg body weight (b.w.)] toxicity was noticed in mice pretreated with the same dose of PL or PF extract (125 mg/kg b.w.) or a combination of both (PL/PF 63/63... (truncated)

Title: Circulating extracellular vesicles with specific proteome and liver microRNAs are potential biomarkers for liver injury in experimental fatty liver disease.
Nonalcoholic fatty liver disease (NAFLD) is the most common chronic liver disease in both adult and children. Currently there are no reliable methods to determine disease severity, monitor disease progression, or efficacy of therapy, other than an invasive liver biopsy.

Title: Polyphenols in Liubao Tea Can Prevent CCl_4-Induced Hepatic Damage in Mice through Its Antioxidant Capacities.
The present study investigated the preventive effect of polyphenols in Liubao tea (PLT) on carbon tetrachloride (CCl_4)-induced liver injury in mice. The mice were initially treated with PLT, followed by induction of liver injury using 10 mL/kg CCl_4. Then liver and serum indices, as well as the expression levels of related messenger RNAs (mRNAs) and proteins in liver tissues were measured. The results showed that PLT reduces the liver quality and indices of mice with liver injury. PLT also downregulates aspartate aminotransferase (AST), alanine aminotransferase (ALT), triglycerides (TGs), and malondialdehyde (MDA), and upregulates superoxide dismutase (SOD) and glutathione peroxidase (GSH-Px)... (truncated)

Title: Synergistic delivery of resveratrol and ultrasmall copper-based nanoparticles by aptamer-functionalized ultrasound nanobubbles for the treatment of nonalcoholic fatty liver disease.
Nonalcoholic fatty liver disease (NAFLD) is related to the production of reactive oxygen species (ROS) and oxidative stress, so antioxidant treatment can prevent its further development. Ultrasmall copper-based nanoparticles (CuNPs) have shown multiple enzyme-like activities for scavenging oxygen species, providing a new strategy for the treatment of inflammatory diseases. Resveratrol (Res), a natural polyphenol compound, has attracted much attention due to its ability to inhibit oxidative stress. We therefore aimed to first combine these two agents for the treatment of NAFLD. However, due to the poor water solubility and stability of Res, which is easily metabolized in the intestine, the... (truncated)

MESH:D012601 - scopolamine

Summary:

LLM Prediction Score	0.008 (normalized)
LLM Confidence Score	0.990
Golden Answer (Severity Class)	0.0 (normalized)
Prediction Error	0.008

Retrieved Context:

Title: Cimetidine protects against acetaminophen toxicity.
Generally, acetaminophen (APAP) overdoses with elimination half-lives over 4 hr. sustain liver damage. In the following cases, cimetidine (C) seems to have protected against APAP toxicity. An 18 yr. old, 64 kg female smoker presented 6 hr. after taking 10Rg APAP, 1200+ mg C, and small amounts of flurazepam and Sleepeze (methaprilene + scopolamine). Three plasma APAP levels (by HPLC) revealed an elimination half-life of 4.4 hr. C did not interfere with the APAP assay. Despite the long half-life in a patient with microsomal enzymes induced by smoking, no evidence of hepatotoxicity developed. A month later, the same patient overdosed... (truncated)

Title: Enhanced performance of spatial and visual recognition memory tasks by the selective acetylcholinesterase inhibitor E2020 in rhesus monkeys.
Hepatotoxicity limits the clinical utility of the cholinesterase inhibitor tacrine as a palliative therapy for Alzheimer's disease. The present studies examined the effects of E2020, a selective acetylcholinesterase inhibitor not associated with liver toxicity in man, on cognitive performance in rhesus monkeys using tasks employed previously to evaluate tacrine and other cholinomimetic agents. The ability of E2020 to prevent the induction of a cognitive impairment by the muscarinic receptor antagonist scopolamine was assessed using an automated spatial delayed response task. Coadministration of E2020 (0.5-1.75 mg/kg) caused a dose-dependent reversal of the scopolamine (0.03 mg/kg) induced impairment observed after retention intervals... (truncated)

Title: <i>Origanum vulgare</i> ssp. <i>hirtum</i> (Lamiaceae) Essential Oil Prevents Behavioral and Oxidative Stress Changes in the Scopolamine Zebrafish Model.
<i>Origanum vulgare</i> ssp. <i>hirtum</i> has been used as medicinal herbs promoting antioxidant, anti-inflammatory, antimicrobial, and neuroprotective activities. We investigated the protective effects and the mechanism of <i>O. vulgare</i> ssp. <i>hirtum</i> essential oil (OEO) on cognitive impairment and brain oxidative stress in a scopolamine (Sco)-induced zebrafish (<i>Danio rerio</i>) model of cognitive impairment. Our results show that exposure to Sco (100 µM) leads to anxiety, spatial memory, and response to novelty dysfunctions, whereas the administration of OEO (25, 150, and 300 µL/L, once daily for 13 days) reduced anxiety-like behavior and improved cognitive ability, which was confirmed by behavioral tests, such as... (truncated)

Title: DNA damage by Withanone as a potential cause of liver toxicity observed for herbal products of <i>Withania somnifera</i> (Ashwagandha).
<i>Withania somnifera,</i> commonly known as Ashwagandha, is a medicinal plant used for thousands of years for various remedies. Extracts of Ashwagandha contain more than 200 metabolites, with withanone (win) being one of the major ones responsible for many of its medicinal properties. Recently, several cases of liver toxicity resulting from commercially available Ashwagandha products have been reported. The first report of Ashwagandha-related liver damage was from Japan, which was quickly resolved after drug-withdrawal. Later, similar cases of liver toxicity due to Ashwagandha consumption were reported from the USA and Iceland. Towards understanding the liver toxicity of Ashwagandha extracts, we studied... (truncated)

Title: Poisoned after Dinner: Dolma with Datura Stramonium.
Datura stramonium, which is also known as Thorn Apple or Jimson Weed, is an alkaloid containing plant that is entirely toxic. The active toxic constituents of the plant are atropine, scopolamine and hyoscyamine. It has been abused worldwide for hundreds of years because of its hallucinogenic properties. Previous reports have shown that herbal medication overdose and accidental food contamination are ways it can cause poisoning. Herein we present a family that had three of its members poisoned after eating a traditional meal "dolma" made of datura flowers. None had fatal complications and all were discharged healthy. Datura stromonium may be... (truncated)

MESH:D000077206 - gabapentin

Summary:

LLM Prediction Score	0.368 (normalized)
LLM Confidence Score	0.990
Golden Answer (Severity Class)	0.375 (normalized)
Prediction Error	0.007

Retrieved Context:

Title: [Hepatotoxicity probably associated with gabapentin].
Gabapentin is an anticonvulsant medication used as an adjuvant in the treatment of neuropathic pain; few cases have been reported in which it causes acute liver injury.

Title: Hold the Gaba: A Case of Gabapentin-induced Hepatotoxicity.
A drug-induced liver injury is one of the most common causes of acute liver failure. While acetaminophen is the most common etiology, other offending medications include amoxicillin-clavulanic acid, amiodarone, isoniazid, and fluoroquinolones to name a few. Gabapentin, a gamma-aminobutyric acid (GABA) analogue, has infrequently been reported to cause liver injury; however, the causality in the previous reports is contested. Herein, we report a gabapentin-induced hepatocellular injury in a patient without another identifiable cause for acute liver injury. Discontinuing gabapentin resulted in rapid reversal improvement in hepatocellular injury.

Title: Antiepileptic Drugs and Liver Disease.
Acute, symptomatic seizures or epilepsy may complicate the course of hepatic disease. Choosing the most appropriate antiepileptic drug in this setting represents a difficult challenge, as most medications are metabolized by the liver. This article focuses on the acute and chronic treatment of seizures in patients with advanced liver disease and reviews the hepatotoxic potential of specific antiepileptic drugs. Newer antiepileptic drugs without, or with minimal, hepatic metabolism, such as levetiracetam, lacosamide, topiramate, gabapentin, and pregabalin should be used as first-line therapy. Medications undergoing extensive hepatic metabolism, such as valproic acid, phenytoin, and felbamate should be used as drugs of... (truncated)

Title: Review about gabapentin misuse, interactions, contraindications and side effects.
The current work is targeted to review the risks of gabapentin misuse, its potential interactions with other drugs, side effects and use contraindications. This review consists of a total of 99 biographical references (from the year 1983 to 2016). A publication search of PubMed was performed from January 1983 to December 2016. It included animal studies, clinical studies, case studies and reviews related to gabapentin misuse, potential interactions, side effects and use contraindications. The search terms were gabapentin, anticonvulsant and antiepileptic. In general, it seems that gabapentin has risks of being misused based on the increased level of prescriptions, related... (truncated)

Title: Chronic effect of gabapentin on liver function in adult male rats.
Gabapentin (GPN) is a new antiepileptic agent currently in used as add-on therapy in adult patients suffering from partial seizures. The extent of liver damage at different dosage and long term treatment with GPN is not yet clear. Therefore this study was undertaken to find out the possibility of liver damage by this drug. Adult male (Wistar) rats of 180-220 g were administered intraperitoneally with GPN (20 or 100 mg/kg) for 45 days. After the experimental period, the liver function tests were carried out in control and experimental groups. The activity of liver enzymes, with 20 mg/kg of GPN were... (truncated)

MESH:D000078334 - lacosamide

Summary:

LLM Prediction Score	0.382 (normalized)
LLM Confidence Score	0.980
Golden Answer (Severity Class)	0.375 (normalized)
Prediction Error	0.007

Retrieved Context:

Title: Antiepileptic Drugs and Liver Disease.
Acute, symptomatic seizures or epilepsy may complicate the course of hepatic disease. Choosing the most appropriate antiepileptic drug in this setting represents a difficult challenge, as most medications are metabolized by the liver. This article focuses on the acute and chronic treatment of seizures in patients with advanced liver disease and reviews the hepatotoxic potential of specific antiepileptic drugs. Newer antiepileptic drugs without, or with minimal, hepatic metabolism, such as levetiracetam, lacosamide, topiramate, gabapentin, and pregabalin should be used as first-line therapy. Medications undergoing extensive hepatic metabolism, such as valproic acid, phenytoin, and felbamate should be used as drugs of... (truncated)

Title: A case of lacosamide-induced hepatotoxicity.
Lacosamide is a novel antiepileptic drug that acts mainly via the selective enhancement of slow inactivation of voltage-gated sodium channels. It has been reported that lacosamide is effective and generally tolerable as an adjuvant treatment in patients with partial seizures. There are few reports regarding liver damage caused by lacosamide. We describe a case of a patient with drug-resistant epilepsy who developed symptomatic hepatotoxicity after lacosamide administration.

Title: Lacosamide as treatment for partial epilepsy: mechanisms of action, pharmacology, effects, and safety.
Lacosamide (LCM) is a novel agent that has been developed as an antiepileptic drug. In vitro studies suggest that LCM modulates voltage-gated sodium channels by enhancing their slow inactivation. In addition, LCM seems to interact with collapsin-response mediator protein 2 and thus may mediate neuronal plasticity. LCM has an elimination half-life of 13 hours, no relevant protein binding, and does not induce or inhibit enzymes of the cytochrome P450 system. No clinically significant drug-drug interactions have been discovered as yet. Experimental data suggest anticonvulsant as well as analgesic effects. Large clinical studies have demonstrated its efficacy for treatment of patients... (truncated)

Title: Use of antiepileptic drugs in hepatic and renal disease.
The use of antiepileptic drugs in patients with renal or hepatic disease is common in clinical practice. Since the liver and kidney are the main organs involved in the elimination of most drugs, their dysfunction can have important effects on the disposition of antiepileptic drugs. Renal or hepatic disease can prolong the elimination of the parent drug or an active metabolite leading to accumulation and clinical toxicity. It can also affect the protein binding, distribution, and metabolism of a drug. The protein binding of anionic acidic drugs, such as phenytoin and valproate, can be reduced significantly by renal failure, causing... (truncated)

Title: Lacosamide poisoning improved by hemodialysis.
Lacosamide (LCM) is a third-generation antiepileptic drug that has been proven to be effective and safe, with few side-effects.

MESH:C012655 - nimesulide

Summary:

LLM Prediction Score	0.994 (normalized)
LLM Confidence Score	0.950
Golden Answer (Severity Class)	1.0 (normalized)
Prediction Error	0.006

Retrieved Context:

Title: [Bleeding gastric ulcers and acute hepatitis: 2 simultaneous adverse reactions due to nimesulide in a case].
A 66 year-old obese woman with arthrosis, self-medicated with oral nimesulide, 200 mg daily. After 6 weeks she developed nausea, jaundice and dark urine. Two weeks later she had recurrent hematemesis and was hospitalized. Besides obesity and anemia her physical examination was unremarkable. An upper GI endoscopy revealed 3 acute gastric ulcers and a 4th one in the pyloric channel. Abdominal ultrasonogram showed a slightly enlarged liver with diffuse reduction in ecogenicity; the gallbladder and biliary tract were normal. Blood tests demonstrated a conjugated hyperbilirubinemia (maximal total value: 18.4 mg/dl), ALAT 960 U/l, ASAT 850 U/l, GGT 420 U/l, alkaline... (truncated)

Title: Nimesulide-induced fulminant hepatitis.
An 18-year-old boy was brought to the hospital with jaundice, confusion, abdominal discomfort and distension. He had a history of oral intake of nimesulide for three days. Clinical and laboratory findings were compatible with fulminant hepatitis. Exclusion of other causes of liver injury strongly favored drug-induced toxicity. All of the signs, symptoms and laboratory abnormalities returned to normal after cessation of the nimesulide and supportive treatment, and he was discharged on the 15th day after admission. This case differs from the other cases in the literature regarding the time of onset, and indicates that nimesulide may induce fulminant hepatitis in... (truncated)

Title: Fatal hepatitis and renal failure during treatment with nimesulide.
A healthy 70-year-old woman who took nimesulide for 5 days, presented 2 weeks later with jaundice for which no other cause was found. Laboratory evidence of coagulopathy, hypoalbuminaemia and hypoglycaemia were present on admission, and liver biopsy showed massive necrosis of hepatocytes and severe inflammatory infiltrate. Despite supportive and corticosteroid treatment, her jaundice deepened and progressive acute renal failure developed, characterized by a 'prerenal' profile changing into irreversible acute tubular necrosis pattern, coma, occult Gram-negative sepsis and death. Although rare, nimesulide-associated hepatotoxicity and nephrotoxicity may occur and should be recognized as early as possible, to ensure immediate drug withdrawal and... (truncated)

Title: Nimesulide-induced severe hemolytic anemia and acute liver failure leading to liver transplantation.
We present the case of a 63-year-old woman who had undergone 7 months of treatment with Nimesulide (100 mg/b.i.d.) for symptomatic osteoarthritis. The patient was admitted to our unit with a clinical picture of progressive jaundice over 3 weeks. Clinical and analytical studies revealed acute liver failure, this being confirmed by liver biopsy, which showed submassive necrosis. Serological tests for different viral agents causing hepatitis were all negative. In addition, she presented a picture of severe haemolytic anaemia resistant to several treatments and needed multiple transfusions. Twenty-three days after admission, the patient presented hepatic encephalopathy and received an orthotopic liver... (truncated)

Title: Fatal hepatotoxicity secondary to nimesulide.
This report describes a 57-year-old female patient with chronic lumbago, who died from the sequelae of acute liver failure induced by nimesulide medication. Nimesulide is a non-steroidal anti-inflammatory drug (NSAID) which preferentially inhibits cyclo-oxygenase 2 and has been associated with a total of 13 reported cases of severe liver injury including our case. On the basis of the literature reports, the following features of nimesulide-associated hepatotoxicity were identified: female sex (84% of cases), age (mean age 62 years), jaundice as a primary manifestation (90%) and the absence of peripheral blood eosinophilia. The average duration of therapy of the published cases... (truncated)

MESH:D000069455 - palivizumab

Summary:

LLM Prediction Score	0.006 (normalized)
LLM Confidence Score	0.980
Golden Answer (Severity Class)	0.0 (normalized)
Prediction Error	0.006

Retrieved Context:

Title: Antibody development for preventing the human respiratory syncytial virus pathology.
Human respiratory syncytial virus (hRSV) is the most important etiological agent causing hospitalizations associated with respiratory diseases in children under 5 years of age as well as the elderly, newborns and premature children are the most affected populations. This viral infection can be associated with various symptoms, such as fever, coughing, wheezing, and even pneumonia and bronchiolitis. Due to its severe symptoms, the need for mechanical ventilation is not uncommon in clinical practice. Additionally, alterations in the central nervous system -such as seizures, encephalopathy and encephalitis- have been associated with cases of hRSV-infections. Furthermore, the absence of effective vaccines or... (truncated)

Title: Respiratory Syncytial Virus: A Comprehensive Review of Transmission, Pathophysiology, and Manifestation.
With an increasing global incidence in children younger than the age of five, respiratory syncytial virus (RSV) is one of the most common viral respiratory infections worldwide. Despite the increasing number of cases among infants and young children, RSV can infect any age group; however, some individuals are more high risk than others. Premature infants, young children, elderly, and immunocompromised individuals are the most likely to suffer a more severe presentation of RSV in comparison to healthy adults. RSV is transmitted through respiratory droplets via direct contact with an infected individual or with contaminated surfaces. The viral genome of RSV... (truncated)

Title: Safety, Tolerability, and Pharmacokinetics of MEDI8897, the Respiratory Syncytial Virus Prefusion F-Targeting Monoclonal Antibody with an Extended Half-Life, in Healthy Adults.
Prevention of respiratory syncytial virus (RSV) illness in infants is a major public health priority, but there is no approved vaccine. Palivizumab is a monoclonal antibody that provides RSV prophylaxis but requires 5 monthly injections and is approved only for infants who experience the greatest morbidity and mortality from RSV. Thus, there remains a significant unmet medical need for prevention of RSV disease in healthy infants. MEDI8897 is a recombinant human RSV monoclonal antibody with a modified Fc region that extends its half-life and is being developed as RSV prophylaxis for all infants. In this phase 1, first-in-human, placebo-controlled study,... (truncated)

Title: Immunological Features of Respiratory Syncytial Virus-Caused Pneumonia-Implications for Vaccine Design.
The human respiratory syncytial virus (hRSV) is the causative agent for high rates of hospitalizations due to viral bronchiolitis and pneumonia worldwide. Such a disease is characterized by an infection of epithelial cells of the distal airways that leads to inflammation and subsequently to respiratory failure. Upon infection, different pattern recognition receptors recognize the virus and trigger the innate immune response against the hRSV. Further, T cell immunity plays an important role for virus clearance. Based on animal studies, it is thought that the host immune response to hRSV is based on a biased T helper (Th)-2 and Th17 T... (truncated)

Title: Trends in the development and approval of monoclonal antibodies for viral infections.
Monoclonal antibodies (mAbs) developed for either the prevention or treatment of viral diseases represent a small, but valuable, class of products. Since 1985, commercial firms have initiated clinical studies involving a total of 28 mAbs. To date, one product (palivizumab) has been approved and eight candidates are currently in clinical study. Most commercial mAbs studied as antiviral agents in the clinic have either directly or indirectly targeted human immunodeficiency virus, respiratory syncytial virus, or hepatitis C virus infections. However, the ability of mAbs to bind to specific targets and utilize various anti-infective modes of action would seem to make them... (truncated)

MESH:D006024 - glycopyrrolate

Summary:

LLM Prediction Score	0.006 (normalized)
LLM Confidence Score	0.980
Golden Answer (Severity Class)	0.0 (normalized)
Prediction Error	0.006

Retrieved Context:

Title: The Movement Disorder Society Evidence-Based Medicine Review Update: Treatments for the non-motor symptoms of Parkinson's disease.
The Movement Disorder Society (MDS) Task Force on Evidence-Based Medicine (EBM) Review of Treatments for Parkinson's Disease (PD) was first published in 2002 and was updated in 2005 to cover clinical trial data up to January 2004 with the focus on motor symptoms of PD. In this revised version the MDS task force decided it was necessary to extend the review to non-motor symptoms. The objective of this work was to update previous EBM reviews on treatments for PD with a focus on non-motor symptoms. Level-I (randomized controlled trial, RCT) reports of pharmacological and nonpharmacological interventions for the non-motor symptoms... (truncated)

Title: Red-fleshed apple flavonoid extract alleviates CCl₄-induced liver injury in mice.
In recent years, the global incidence of liver damage has increased. Despite the many known health benefits of red-fleshed apple flavonoids, their potential liver-protective effects have not yet been investigated. In this study, we analyzed the composition of red-fleshed apple flavonoid extract (RAFE) by high-performance liquid chromatography (HPLC). We then induced liver damage in mice with carbon tetrachloride (CCl₄) and performed interventions with RAFE to analyze its effect on liver damage, using bifendate as a positive control. The results showed that catechin was the most abundant flavonoid in 'XJ4' RAFE (49.346 mg/100 g). In liver-injured mice, the liver coefficients converged... (truncated)

Title: A phase IIb randomized, chronic-dosing, incomplete block, cross-over study of glycopyrronium, delivered via metered dose inhaler, compared with a placebo and an active control in patients with moderate-to-severe COPD.
Label="BACKGROUND">Long-acting muscarinic antagonist (LAMA) and long-acting β₂-agonist (LABA) bronchodilators are key to the pharmacologic treatment of chronic obstructive pulmonary disease (COPD). This Phase IIb study investigated the safety and efficacy of four doses of the LAMA glycopyrronium (GP) delivered using co-suspension delivery technology via metered dose inhaler (MDI). The study was part of a wider clinical trial program performed to determine the optimal dose of GP MDI, the LABA formoterol fumarate dihydrate (FF) MDI, and glycopyrronium/formoterol fumarate dihydrate (GFF) MDI fixed-dose combination to be taken forward into Phase III studies.

Title: In Vivo Magnetic Resonance Spectroscopy of Hyperpolarized [1-¹³ C]Pyruvate and Proton Density Fat Fraction in a Guinea Pig Model of Non-Alcoholic Fatty Liver Disease Development After Life-Long Western Diet Consumption.
Alterations in glycolysis are central to the increasing incidence of non-alcoholic fatty liver disease (NAFLD), highlighting a need for in vivo, non-invasive technologies to understand the development of hepatic metabolic aberrations.

Title: Case Report: Ursodeoxycholic acid treatment in Niemann-Pick disease type C; clinical experience in four cases.
In this case series, we demonstrate that Ursodeoxycholic acid (UDCA) improves liver dysfunction in Niemann-Pick type C (NPC) and may restore a suppressed cytochrome p450 system. NPC disease is a progressive neurodegenerative lysosomal storage disease caused by mutations in either the <i>NPC1</i> or <i>NPC2</i> genes. Liver disease is a common feature presenting either acutely as cholestatic jaundice in the neonatal period, or in later life as elevated liver enzymes indicative of liver dysfunction. Recently, an imbalance in bile acid synthesis in a mouse model of NPC disease was linked to suppression of the P450 detoxification system and was corrected by... (truncated)

MESH:D011374 - progesterone

Summary:

LLM Prediction Score	0.255 (normalized)
LLM Confidence Score	0.990
Golden Answer (Severity Class)	0.25 (normalized)
Prediction Error	0.005

Retrieved Context:

Title: Hepatobiliary complications of oral contraceptives.
Complications secondary to the use of oral contraceptive agents are rare. Hepatobiliary complications, while often dramatic in presentation, occur infrequently. In a patient without predisposing conditions to complications, the benefits achieved with estrogen/progesterone products outweigh the risks. Those conditions that would absolutely and relatively contraindicate the use of oral contraceptives are listed in Table 4. Patients with a past history of liver disease in whom liver function tests have returned to normal may tolerate the introduction of oral contraceptives. They need to be monitored closely for adverse reactions. Patients who have experienced cholestatic jaundice of pregnancy should avoid all contraceptives... (truncated)

Title: The Hepatoprotective and Hepatotoxic Roles of Sex and Sex-Related Hormones.
Most liver diseases, including acute liver injury, drug-induced liver injury, viral hepatitis, metabolic liver diseases, and end-stage liver diseases, are strongly linked with hormonal influences. Thus, delineating the clinical manifestation and underlying mechanisms of the "sexual dimorphism" is critical for providing hints for the prevention, management, and treatment of those diseases. Whether the sex hormones (androgen, estrogen, and progesterone) and sex-related hormones (gonadotrophin-releasing hormone, luteinizing hormone, follicle-stimulating hormone, and prolactin) play protective or toxic roles in the liver depends on the biological sex, disease stage, precipitating factor, and even the psychiatric status. Lifestyle factors, such as obesity, alcohol drinking, and... (truncated)

Title: Effect of pregnancy on pre-existing liver disease physiological changes during pregnancy.
The pregnant woman experiences physiological changes to support fetal growth and development. Particularly the physiological changes of the liver are the results of the increment of estrogens and progesterone during the pregnancy, and also the hemodynamics changes. (hemodilution). Telangiectasia may appear in up to 60% of normal pregnancies. Liver function test (LFT) abnormalities occurs in 3% of the pregnancies, and the Preeclampsia is the most frequent cause. Most of the articles agree that in normal pregnancy the LFT are either normal or slightly increase o decrease but within normal range. Thus, an increase in serum ALT, AST and GGT activities... (truncated)

Title: Involvement of calcium channels in the sexual dimorphism of cadmium-induced hepatotoxicity.
Cadmium toxicity has been evaluated in a number of in vivo and in vitro toxicological studies. In vivo Cd toxicity exhibits sexual dimorphism with females being more susceptible to Cd uptake, accumulation, and toxicity in the liver. Research to date does not explain why females are more sensitive to Cd-induced hepatotoxicity. Recent studies demonstrate that progesterone sensitizes female F(344) rats and TRL-1215 cells to Cd toxicity, however the mode of action is still unclear. Approximately one half of the Cd entering the cytoplasm does so through receptor operated Ca(2+) channels. Progesterone treatment of human spermatozoa and Xenopus laevis oocytes causes... (truncated)

Title: Uses of progesterone in clinical practice.
Progesterone is the natural progestagen produced by the corpus luteum during the luteal phase. It is absorbed when administered orally, but is greater than 90% metabolized during the first hepatic pass. This greatly limits the efficacy of once-daily administration and also results in unphysiologically high levels of progesterone metabolites, particularly those reduced at the 5-a position. These metabolites can cause dizziness and drowsiness to the point of preventing the operation of a motor vehicle. Synthetic progestins, such as medroxyprogesterone acetate and norethindrone acetate (NETA), have been specifically designed to resist enzymatic degradation and remain active after oral administration. However, these... (truncated)

MESH:D005913 - glipizide

Summary:

LLM Prediction Score	0.380 (normalized)
LLM Confidence Score	0.980
Golden Answer (Severity Class)	0.375 (normalized)
Prediction Error	0.005

Retrieved Context:

Title: Idiosyncratic toxicity associated with potentiated sulfonamides in the dog.
Idiosyncratic toxicity to potentiated sulfonamides occurs in both humans and dogs, with considerable clinical similarities. The syndrome in dogs can consist of fever, arthropathy, blood dyscrasias (neutropenia, thrombocytopenia, or hemolytic anemia), hepatopathy consisting of cholestasis or necrosis, skin eruptions, uveitis, or keratoconjunctivitis sicca. Other manifestations seen less commonly include protein-losing nephropathy, meningitis, pancreatitis, pneumonitis, or facial nerve palsy. The pathogenesis of these reactions is not completely understood, but may be due to a T-cell-mediated response to proteins haptenated by oxidative sulfonamide metabolites. Our laboratory is working on tests to characterize dogs with possible idiosyncratic sulfonamide reactions, to include ELISA for... (truncated)

Title: Sulfonylurea-Induced Hypoglycemia in a Patient With Cirrhosis.
Type 2 diabetes mellitus is highly prevalent among patients with cirrhosis. The pharmacological management of this disease in patients with chronic liver disease remains controversial, however. Insulin secretagogues such as sulfonylureas are associated with a high risk of hypoglycemia among diabetics. In patients with cirrhosis, this risk is more pronounced due to decreased hepatic clearance, concurrent alcoholism, hypoalbuminemia, and acute liver decompensation. In this case report, we present a case of severe refractory hypoglycemia secondary to glipizide in a patient with alcoholic cirrhosis. We believe that the use of sulfonylureas in this patient population should be contraindicated to avoid debilitating... (truncated)

Title: Comparison of twelve single-drug regimens for the treatment of type 2 diabetes mellitus.
We performed a network meta-analysis to compare the efficacy of 12 single-drug regimens (Glibenclamide, Glimepiride, Pioglitazone, Rosiglitazone, Repaglinide, Metformin, Sitaglitin, Exenatide, Liraglutide, Acarbose, Benfluorex, and Glipizide) in the treatment of type 2 diabetes mellitus (T2DM). Fifteen relevant randomized controlled trials (RCTs) were included; direct and indirect evidence from these studies was combined, and weighted mean difference (WMD) and surface under the cumulative ranking curves (SUCRAs) were examined to evaluate the monotherapies. Liraglutide was more effective than Glimepiride, Pioglitazone, Sitaglitin, Exenatide, and Glipizide at reducing glycated hemoglobin (HbA1c) levels. In contrast, Acarbose was less effective than Glibenclamide, Glimepiride, Pioglitazone, Rosiglitazone, Repaglinide,... (truncated)

Title: A Post Hoc Analysis of HbA1c, Hypoglycemia, and Weight Change Outcomes with Alogliptin vs Glipizide in Older Patients with Type 2 Diabetes.
Adverse events and complications limit the long-term use of current antidiabetic treatment options for patients with type 2 diabetes mellitus (T2DM), particularly for older adults who are often receiving therapy for other comorbid conditions. The aim of this study was to evaluate the benefits of the dipeptidyl peptidase-4 inhibitor, alogliptin, versus glipizide, a sulfonylurea, in achieving glycemic control without the risk of hypoglycemia, weight gain, or both in older patients with T2DM.

Title: Clinical characteristics of drug-induced liver injury and related risk factors.
Drug-induced liver injury (DILI) is often undiagnosed or misdiagnosed clinically because of diagnostic difficulties caused by lack of laboratory-specific serological markers. In this study, we comprehensively assessed the clinical characteristics, laboratory indices, hepatotoxic drugs, risk factors and outcomes concerning DILI, and explored the similarities in mechanisms between Chinese and Western drug-induced DILI. Patients with a first diagnosis of DILI and a Roussel Uclaf Causality Assessment Method (RUCAM) score >3 points were enrolled for systematic retrospective study. Their clinical characteristics, clinical classification, risk factors, laboratory indices, hepatotoxic drugs and outcomes were analyzed. Cholestatic patients had the highest alkaline phosphatase (ALP) and... (truncated)

MESH:C071192 - entacapone

Summary:

LLM Prediction Score	0.004 (normalized)
LLM Confidence Score	0.970
Golden Answer (Severity Class)	0.0 (normalized)
Prediction Error	0.004

Retrieved Context:

Title: Safety and tolerability of COMT inhibitors.
Combining levodopa with the catechol-O-methyltransferase (COMT) inhibitor entacapone has been shown to be an effective strategy in the management of Parkinson's disease (PD) patients experiencing motor fluctuations. Safety and tolerability information has come from postmarketing surveillance studies as well as several randomized, placebo-controlled trials with long-term open-label extension phases specifically investigating the safety and tolerability of levodopa plus entacapone. Results show the most common dopaminergic side effects to be dyskinesia and nausea, which result from the increased bioavailability of levodopa and can be readily managed. Non-dopaminergic side effects include diarrhea and harmless urine discoloration. There is no convincing evidence of... (truncated)

Title: Levodopa + carbidopa + entacapone. Entacapone: a second look: new preparations. Parkinson's disease: a modest effect.
(1) If patients with Parkinson's disease treated with levodopa develop end-of-dose motor fluctuations, the standard therapy is to add bromocriptine, a dopamine receptor agonist, to their ongoing treatment. (2) Evaluation data available in 1999 on entacapone, a catechol-o-methyltransferase (COMT) inhibitor, failed to show whether the balance of benefits versus harm was at least equivalent to that of bromocriptine. (3) Entacapone is now also available as a triple fixed-dose combination with levodopa + carbidopa. (4) Three double-blind trials have compared triple combinations of levodopa + carbidopa (or benserazide) + entacapone with levodopa + carbidopa (or benserazide) + placebo. Two of these... (truncated)

Title: Hepatotoxic profile of catechol-O-methyltransferase inhibitors in Parkinson's disease.
Entacapone and tolcapone are selective catechol-O-methyltransferase (COMT) inhibitors developed recently as adjuncts to levodopa for the treatment of Parkinson's disease (PD). They extend the duration of action of levodopa. As a result, they increase 'on' time, decrease 'off' time and improve motor scores in patients with motor fluctuations. Both benefits and main side effects are related to increased dopaminergic activity. This paper reviews the use of those COMT inhibitors in PD with particular focus on the issue of hepatotoxicity. Neither tolcapone nor entacapone caused hepatotoxicity in preclinical studies. However, in 1998, four patients who were using tolcapone presented with serious... (truncated)

Title: In vitro metabolism of tolcapone to reactive intermediates: relevance to tolcapone liver toxicity.
Tolcapone is a catechol-O-methyltransferase (COMT) inhibitor used for control of motor fluctuations in Parkinson's disease (PD). Since its entry onto the market in 1998, tolcapone has been associated with numerous cases of hepatotoxicity, including three cases of fatal fulminant hepatic failure. The cause of this toxicity is not known; however, it does not occur with the use of the structurally similar drug entacapone. It is known that tolcapone is metabolized to amine (M1) and acetylamine (M2) metabolites in humans, but that the analogous metabolites were not detected in a limited human study of entacapone metabolism. We hypothesized that one or... (truncated)

Title: Toxicology and safety of COMT inhibitors.
The development of catechol-O-methyltransferase (COMT) inhibitors for the adjunct treatment to levodopa and aromatic L-amino acid decarboxylase (AADC) inhibitors in Parkinson's disease started in the late 1950s. The first-generation inhibitors were associated with toxic properties: they induced convulsions, or they were toxic to the liver. None of them was taken into clinical use. The second-generation inhibitors entacapone and tolcapone have now been in clinical use for over a decade, and some new inhibitors are under development. The main adverse events in the use of entacapone and tolcapone are dopaminergic and dependent of the concomitant use of levodopa, but the symptoms... (truncated)

MESH:C569381 - levomefolate calcium

Summary:

LLM Prediction Score	0.004 (normalized)
LLM Confidence Score	0.950
Golden Answer (Severity Class)	0.0 (normalized)
Prediction Error	0.004

Retrieved Context:

Title: Thromboembolic safety of norethisterone vs levonorgestrel in combined oral contraceptive users: a pooled analysis of 4 large prospective cohort studies.
Norethisterone (acetate) and levonorgestrel are marketed globally as components of combined oral contraceptives. Although guidelines recommend both as first-line combined oral contraceptives, no direct, comparative safety studies are available.

Title: Conversion of calcium-l-methylfolate and (6S)-5-methyltetrahydrofolic acid glucosamine salt into dietary folate equivalents.
Following a request from the European Commission, the EFSA Panel on Nutrition, Novel Foods and Food Allergens (NDA) was asked to deliver a scientific opinion on the conversion of calcium-l-methylfolate and (<i>6S</i>)-5-methyltetrahydrofolic acid glucosamine salt (collectively called 5-MTHF hereafter) into dietary folate equivalents (DFE). Following a systematic review, the conclusions of the opinion are based on one intervention study in adults for intakes < 400 μg/day and three intervention studies in adults for intakes ≥ 400 μg/day. At intakes below 400 μg/day, folic acid (FA) is assumed to be linearly related to responses of biomarkers of intake and status and is an appropriate comparator for deriving... (truncated)

Title: Case report: COVID-19 infection in a pregnant 33-year-old kidney transplant recipient.
Patients facing severe acute respiratory syndrome-coronavirus 2 (SARS-CoV-2) infections with comorbidities, especially patients whose immune system is weakened have higher chances to face severe outcomes. One of the main reasons behind the suppression of the immune system is iatrogenic, in patients who have autoimmune diseases and/or had an organ transplant. Although there are studies that are examining immunocompromised and/or transplanted patients with COVID-19 infection, furthermore there is a limited number of studies available which are dealing with COVID-19 in pregnant women; however, it is unique and is worth reporting when these factors are coexisting. In this study, we present the... (truncated)

MESH:D002110 - caffeine

Summary:

LLM Prediction Score	0.004 (normalized)
LLM Confidence Score	1.000
Golden Answer (Severity Class)	0.0 (normalized)
Prediction Error	0.004

Retrieved Context:

Title: Caffeine intake decreases oxidative stress and inflammatory biomarkers in experimental liver diseases induced by thioacetamide: Biochemical and histological study.
Liver disease remains a significant global health problem. Increased caffeine consumption has been associated with a lower prevalence of chronic liver disease. This study aimed to investigate the modifying effects of caffeine on liver injury induced by thioacetamide (TAA) administration in male rats and the possible underlying mechanisms. Forty adult male rats were equally classified into four groups: control group, received only tap water; caffeine-treated group, received caffeine (37.5 mg/kg per day); TAA-treated group, received intraperitoneal (i.p.) TAA (200 mg/kg b.w.) twice a week; and caffeine + TAA-treated group, received combined TAA and caffeine in the same previous doses. After... (truncated)

Title: [Influence of caffeine on toxicity and pharmacokinetics of paracetamol].
The aim of this study was an experimental assessment of the influence of caffeine on the symptoms of the toxic action of paracentamol in mice as well as a detailed analysis if paracetamol pharmacokinetics in men receiving caffeine at the same time. The toxicologic investigations were performed in 620 Swiss mice. The LD50 and LD100 were determined after an administration of paracetamol intraperitoneally. The effects of two doses of caffeine on the survival time and number of animal deaths were investigated. The degree of hepatic damage was assessed on the basis of biochemical serum criteria, i.e. alanine aminotransferase, aspartate aminotransferase,... (truncated)

Title: Caffeine in liver diseases: Pharmacology and toxicology.
We have previously shown that adenosine A1AR antagonists, adenosine A2aAR antagonists, and caffeine have significant inhibitory effects on the activation and proliferation of hepatic stellate cells in alcoholic liver fibrosis. Many recent studies have found that moderate coffee consumption is beneficial for various liver diseases. The main active ingredient of coffee is caffeine, which is a natural non-selective adenosine receptor antagonist. Moreover, numerous preclinical epidemiological studies and clinical trials have examined the association between frequent coffee consumption and the risk of developing different liver diseases. In this review, we summarize and analyze the prophylactic and therapeutic effects of caffeine on... (truncated)

Title: Coffee and caffeine protect against liver injury induced by thioacetamide in male Wistar rats.
Coffee intake has been inversely related to the incidence of liver diseases, although there are controversies on whether these beneficial effects on human health are because of caffeine or other specific components in this popular beverage. Thus, this study evaluated the protective effects of coffee or caffeine intake on liver injury induced by repeated thioacetamide (TAA) administration in male Wistar rats. Rats were randomized into five groups: one untreated group (G1) and four groups (G2-G5) treated with the hepatotoxicant TAA (200 mg/kg b.w., i.p.) twice a week for 8 weeks. Concomitantly, rats received tap water (G1 and G2), conventional coffee... (truncated)

Title: Caffeine reduces the hepatotoxicity of paracetamol in mice.
Paracetamol causes extensive liver damage when taken in overdose quantities; however, it is less hepatotoxic when administered in combination with caffeine. The present work in mice was undertaken to study the effect of caffeine on mortality rates and biochemical and histological parameters of liver damage after administration of toxic doses of paracetamol. It was found that caffeine markedly increased the survival rate after administration of a dose of paracetamol that was lethal to 50% and 100% of mice, reduced liver damage as assessed by serum glutamic pyruvic and glutamic oxaloacetic transaminase activities, partially prevented the depletion of reduced glutathione and... (truncated)

MESH:D017239 - paclitaxel

Summary:

LLM Prediction Score	0.496 (normalized)
LLM Confidence Score	0.990
Golden Answer (Severity Class)	0.5 (normalized)
Prediction Error	0.004

Retrieved Context:

Title: Organic effects of associating paclitaxel with a lipid-based nanoparticle system on a nonhuman primate, <i>Cebus apella</i>.
Lipid-based nanoparticle systems have been used as vehicles for chemotherapeutic agents in experimental cancer treatments. Those systems have generally been credited with attenuating the severe toxicity of chemotherapeutic agents. This study aimed to investigate the effects of associating paclitaxel (PTX) with a lipid-based nanoparticle system on a nonhuman primate, <i>Cebus apella</i>, documenting the toxicity as measured by serum biochemistry, which is a detailed analysis of blood and tissue. Eighteen <i>C. apella</i> were studied: three animals were treated with cholesterol-rich nanoemulsion (LDE) only, without PTX, administered intravenously every 3 weeks, during six treatment cycles; six animals were treated with PTX associated... (truncated)

Title: Hepatotoxicity induced by paclitaxel interaction with turmeric in association with a microcystin from a contaminated dietary supplement.
A 67-year-old Caucasian male with lung cancer was presented to the Emergency Department with asthenia, anorexia, jaundice and choluria. The patient's lung cancer was being treated medically by a combination of paclitaxel/carboplatin with bi-monthly frequency. The patient was also self-medicating with several natural products, including Chlorella (520 mg/day), Silybum marianum (total of 13.5 mg silymarin/day), zinc sulphate (5.5 mg), selenium (50 µg) and 15 g/day of Curcuma longa. In first chemotherapy cycle no toxicity was observed even he was taking other medications as budesonide and sitagliptin. The toxic events started only after the introduction of the dietary products. Chlorella had... (truncated)

Title: Toxicity of Nab-Paclitaxel Compared to Paclitaxel in a Tertiary Hospital in Jeddah, Saudi Arabia: A Retrospective Cohort Study.
Background Nanoparticle albumin-bound paclitaxel has been developed to avoid the toxicities associated with Cremophor-solved paclitaxel. Although many studies confirm this hypothesis, there is recent evidence showing no difference between paclitaxel and nab-paclitaxel in their efficacy and safety profile. This study further assesses the toxicity of both paclitaxel and nab-paclitaxel in adult patients with breast and pancreatic cancer in a tertiary hospital in Jeddah, Saudi Arabia. These toxicities include neutropenia, anaemia, and effects on kidney and liver functions. Methods The study is a retrospective cohort study done at King Abdulaziz University Hospital, Jeddah, Saudi Arabia, from January 2018 to December 2021,... (truncated)

Title: Rutin and Hesperidin Revoke the Hepatotoxicity Induced by Paclitaxel in Male Wistar Rats <i>via</i> Their Antioxidant, Anti-Inflammatory, and Antiapoptotic Activities.
Paclitaxel, one of the most effective chemotherapeutic drugs, is used to treat various cancers but it is exceedingly toxic when used long-term and can harm the liver. This study aimed to see if rutin, hesperidin, and their combination could protect male Wistar rats against paclitaxel (Taxol)-induced hepatotoxicity. Adult male Wistar rats were subdivided into 5 groups (each of six rats). The normal group was orally given the equivalent volume of vehicles for 6 weeks. The paclitaxel-administered control group received intraperitoneal injection of paclitaxel at a dose of 2 mg/Kg body weight twice a week for 6 weeks. Treated paclitaxel-administered groups were... (truncated)

Title: Drug-Induced Liver Injury Caused by Capecitabine: A Case Report and a Literature Review.
Chemotherapy is widely used in cancer treatment, and the drug Capecitabine is often used in treatment of breast cancer and usually well-tolerated. Toxicity from Capecitabine typically involves hand-foot syndrome, fatigue, nausea, reduced appetite, and diarrhea, while severe liver toxicity is rarely seen. We present a case of a 63-year-old female with metastatic breast cancer, without liver metastasis, who developed a severe drug-induced liver injury (DILI) with critically elevated liver enzyme levels as reaction to Capecitabine treatment with seemingly no evident explanation as to why. The patient had a RUCAM score of 7 and a Naranjo score of 6 implying that... (truncated)

MESH:D007792 - lactulose

Summary:

LLM Prediction Score	0.003 (normalized)
LLM Confidence Score	0.990
Golden Answer (Severity Class)	0.0 (normalized)
Prediction Error	0.003

Retrieved Context:

Title: Antifibrotic Effect of Lactulose on a Methotrexate-Induced Liver Injury Model.
The most severe side effect of prolonged MTX treatment is hepatotoxicity. The aim of this study is to investigate the effect of lactulose treatment on MTX-induced hepatotoxicity in a rat model. Twenty-four male rats were included in the study. Sixteen rats were given a single dose of 20 mg/kg MTX to induce liver injury. Eight rats were given no drugs. 16 MTX-given rats were divided into two equal groups. Group 1 subjects were given lactulose 5 g/kg/day, and group 2 subjects were given saline 1 ml/kg/day for 10 days. The rats were then sacrificed to harvest blood and liver tissue samples in order... (truncated)

Title: The effects of concurrent treatment of silymarin and lactulose on memory changes in cirrhotic male rats.
<i>Introduction:</i> Chronic liver disease frequently accompanied by hepatic encephalopathy (HE). Changes in the permeability of the blood-brain barrier in HE, make an easier entrance of ammonia among other substances to the brain, which leads to neurotransmitter disturbances. Lactulose (LAC), causes better defecation and makes ammonia outreach of blood. Silymarin (SM) is a known standard drug for liver illnesses. The purpose of this research was to determine the results of LAC and SM combined treatment, on the changes in memory of cirrhotic male rats. <i>Methods:</i> The cirrhotic model established by treatment with thioacetamide (TAA) for 18 weeks. Cirrhotic rats randomized to... (truncated)

Title: Acute Liver Failure Following One Year of Daily Consumption of a Sugar-Free Energy Drink.
We report a 36-year-old man who presented with 1 week of right upper quadrant abdominal pain, jaundice, and fatigue. He consumed 3 sugar-free energy drinks daily for the past year with binge alcohol use. His liver function progressively deteriorated, requiring orthotopic liver transplantation. Submassive hepatic necrosis with eosinophilic infiltrate was seen on pathology, consistent with drug-induced liver injury. Further investigation is warranted into identifying which individuals are susceptible to liver failure from energy drink consumption.

Title: METH-Induced Neurotoxicity Is Alleviated by Lactulose Pretreatment Through Suppressing Oxidative Stress and Neuroinflammation in Rat Striatum.
Abuse of methamphetamine (METH) results in neurological and psychiatric abnormalities. Lactulose is a poorly absorbed derivative of lactose and can effectively alleviate METH-induced neurotoxicity in rats. The present study was designed to investigate the effects of lactulose on METH-induced neurotoxicity. Rats received METH (15 mg/kg, 8 intraperitoneal injections, 12-h interval) or saline and received lactulose (5.3 g/kg, oral gavage, 12-h interval) or vehicle 2 days prior to the METH administration. Reactive oxygen species (ROS) and malondialdehyde (MDA) were measured. Protein levels of toll-like receptor 4 (TLR4), myeloid differentiation factor 88 (MyD88), tumor necrosis factor receptor associated factor 6 (TRAF6), nuclear... (truncated)

Title: Active Clinical Trials in Hepatic Encephalopathy: Something Old, Something New and Something Borrowed.
Hepatic encephalopathy (HE) is a potentially reversible neurocognitive syndrome that occurs in patients with acute or chronic liver disease. Currently, most of the therapies for HE aim to reduce ammonia production or increase its elimination. To date, only two agents have been approved as treatments for HE: lactulose and rifaximin. Many other drugs have also been used, but data to support their use are limited, preliminary or lacking. The aim of this review is to provide an overview and discussion of the current development of treatments for HE. Data from ongoing clinical trials in HE were obtained from the ClinicalTrials.gov... (truncated)

MESH:C007288 - dexpanthenol

Summary:

LLM Prediction Score	0.003 (normalized)
LLM Confidence Score	0.970
Golden Answer (Severity Class)	0.0 (normalized)
Prediction Error	0.003

Retrieved Context:

Title: Protective effect of dexpanthenol against cisplatin-induced hepatotoxicity.
The aim of the present study was to investigate the protective effect of dexpanthenol (Dexp) against cisplatin (Cis)-induced hepatotoxicity. Thirty-two Sprague Dawley rats were divided into four groups: Control group (n=8), Dexp group (n=8, 500 mg/kg/ip/daily single dose/3 days Dexp), Cis group (n=8, 7 mg/kg/ip/single dose Cis) and Cis+Dexp group (n=8, 500 mg/kg/ip/daily single dose/3 days Dexp +7 mg/kg/ip/single dose Cis). MDA, CAT, GSH, GSH-Px, TOS, TAS, OSI, Total Nitrit, IL-1β, IL-6 and TNF-α levels were analyzed in liver tissue samples. After paraffinization of liver tissue samples, histopathological (congestion, loss of glycogen, number of Kupffer cells) and immunohistochemical (caspase-3 expression)... (truncated)

Title: Apetamin Hepatotoxicity: Potential Consequences of Purchasing a Body Enhancement Drug Off the Internet.
We report Apetamin (cyproheptadine lysine and vitamin syrup), a non-US Food and Drug Administration-approved weight gain supplement, causing drug-induced autoimmune hepatitis. A 40-year-old previously healthy woman presented with fatigue, right-sided abdominal discomfort, and jaundice 6 weeks after starting Apetamin, which she learned from social media for figure augmentation. Labs were significant for elevated transaminases, positive smooth muscle antibody, and increased immunoglobulins. Biopsy indicated drug-induced autoimmune hepatitis. Symptoms improved with prednisone, azathioprine, and stopping Apetamin which contains cyproheptadine, a known hepatotoxin. The case reveals the influence of social media and its impact on health and the importance of a complete drug... (truncated)

Title: Targeting ferroptosis, a novel programmed cell death, for the potential of alcohol-related liver disease therapy.
Ferroptosis is a new iron-dependent cell death mode, which is different from the other types of programmed cell death, such as apoptosis, necrosis, and autophagy. Ferroptosis is characterized by a process in which fatal lipids from lipid peroxidation accumulate in cells and eventually lead to cell death. Alcohol-related liver disease (ALD) is a type of liver injury caused by excessive alcohol intake. Alcohol-related liver disease is a broad-spectrum disease category, which includes fatty liver, steatohepatitis, hepatitis, cirrhosis, and hepatocellular tumors. Recent studies have found that ferroptosis is involved in the pathological development of non-viral liver diseases. Therefore, ferroptosis may be... (truncated)

Title: Impact of age on liver damage, inflammation, and molecular signaling pathways in response to femoral fracture and hemorrhage.
Trauma causes disability and mortality globally, leading to fractures and hemorrhagic shock. This can trigger an irregular inflammatory response that damages remote organs, including liver. Aging increases the susceptibility to dysregulated immune responses following trauma, raising the risk of organ damage, infections, and higher morbidity and mortality in elderly patients. This study investigates how aging affects liver inflammation and damage post-trauma.

Title: Effects of Gut Metabolites and Microbiota in Healthy and Marginal Livers Submitted to Surgery.
Microbiota is defined as the collection of microorganisms within the gastrointestinal ecosystem. These microbes are strongly implicated in the stimulation of immune responses. An unbalanced microbiota, termed dysbiosis, is related to the development of several liver diseases. The bidirectional relationship between the gut, its microbiota and the liver is referred to as the gut-liver axis. The translocation of bacterial products from the intestine to the liver induces inflammation in different cell types such as Kupffer cells, and a fibrotic response in hepatic stellate cells, resulting in deleterious effects on hepatocytes. Moreover, ischemia-reperfusion injury, a consequence of liver surgery, alters the... (truncated)

MESH:C043457 - fialuridine

Summary:

LLM Prediction Score	0.998 (normalized)
LLM Confidence Score	0.840
Golden Answer (Severity Class)	1.0 (normalized)
Prediction Error	0.002

Retrieved Context:

Title: The utility of a differentiated preclinical liver model, HepaRG cells, in investigating delayed toxicity via inhibition of mitochondrial-replication induced by fialuridine.
During its clinical development fialuridine caused liver toxicity and the death of five patients. This case remains relevant due to the continued development of mechanistically-related compounds against a back-drop of simple in vitro models which remain limited for the preclinical detection of such delayed toxicity. Here, proteomic investigation of a differentiated, HepaRG, and proliferating, HepG2 cell model was utilised to confirm the presence of the hENT1 transporter, thymidine kinase-1 and -2 (TK1, TK2) and thymidylate kinase, all essential in order to reproduce the cellular activation and disposition of fialuridine in the clinic. Acute metabolic modification assays could only identify mitochondrial... (truncated)

Title: Histopathologic changes associated with fialuridine hepatotoxicity.
Chronic hepatitis B is a widespread viral illness with the serious sequelae of cirrhosis and hepatocellular carcinoma. Current therapy with interferon is not universally efficacious, and this has led to the evaluation of other antiviral agents. A recent Phase II trial of the nucleoside analogue, fluoroiodoarabinofuranosyluracil (fialuridine, FIAU) was halted because of the sudden development of severe multisystem toxicity characterized by hepatic failure, lactic acidosis, and pancreatitis, which resulted in the deaths of five patients. We systematically evaluated pre- and post-therapy biopsy, explant, and autopsy specimens from the 15 patients involved in this trial to define the hepatic changes of... (truncated)

Title: Fialuridine induces acute liver failure in chimeric TK-NOG mice: a model for detecting hepatic drug toxicity prior to human testing.
Seven of 15 clinical trial participants treated with a nucleoside analogue (fialuridine [FIAU]) developed acute liver failure. Five treated participants died, and two required a liver transplant. Preclinical toxicology studies in mice, rats, dogs, and primates did not provide any indication that FIAU would be hepatotoxic in humans. Therefore, we investigated whether FIAU-induced liver toxicity could be detected in chimeric TK-NOG mice with humanized livers.

Title: Mechanisms of drug-induced liver injury: from bedside to bench.
The low incidence of idiosyncratic drug-induced liver injury (DILI), together with the lack of a reliable diagnostic biomarker and robust preclinical and in vitro toxicology test systems for the condition have limited our ability to define the mechanisms of DILI. A notable exception is acetaminophen hepatotoxicity, which is associated with the formation of a well-characterized and highly reactive intermediate metabolite, N-acetyl-p-benzoquinone imine. However, studies have also suggested a role for the host immune response and variation in the expression of the lymphocyte CD44 gene in the pathogenesis of acetaminophen hepatotoxicity. A careful review of the laboratory, clinical and histological phenotype... (truncated)

Title: Integrated in vitro models for hepatic safety and metabolism: evaluation of a human Liver-Chip and liver spheroid.
Drug-induced liver injury remains a frequent reason for drug withdrawal. Accordingly, more predictive and translational models are required to assess human hepatotoxicity risk. This study presents a comprehensive evaluation of two promising models to assess mechanistic hepatotoxicity, microengineered Organ-Chips and 3D hepatic spheroids, which have enhanced liver phenotype, metabolic activity and stability in culture not attainable with conventional 2D models. Sensitivity of the models to two hepatotoxins, acetaminophen (APAP) and fialuridine (FIAU), was assessed across a range of cytotoxicity biomarkers (ATP, albumin, miR-122, α-GST) as well as their metabolic functionality by quantifying APAP, FIAU and CYP probe substrate metabolites. APAP... (truncated)

MESH:D000097623 - cefpodoxime

Summary:

LLM Prediction Score	0.377 (normalized)
LLM Confidence Score	0.970
Golden Answer (Severity Class)	0.375 (normalized)
Prediction Error	0.002

Retrieved Context:

Title: Worsening cholestasis and possible cefuroxime-induced liver injury following "successful" therapeutic endoscopic retrograde cholangiopancreatography for a distal common bile duct stone: a case report.
Cefuroxime very rarely causes drug-induced liver injury. We present a case of a patient with paradoxical worsening of jaundice caused by cefuroxime-induced cholestasis following therapeutic endoscopic retrograde cholangiopancreatography for a distal common bile duct stone.

Title: Ceftazidime induced liver injury.
A 65-year-old woman with type II diabetes mellitus complicated by non-healing ulcers with recurrent osteomyelitis was admitted for progression of cellulitis after treatment failure with an outpatient course of amoxicillin-clavulanate. She was found to have persistent osteomyelitis and started on ceftazidime for a culture documented <i>Pseudomonas aeruginosa</i> infection. After two parenteral doses, she had a rapid rise in liver function tests (LFTs) in a hepatocellular pattern. Due to rapid identification, all medications with potential hepatotoxicity, including ceftazidime, were discontinued and the LFTs promptly returned to baseline over 3 days. Of note, the patient did not experience any symptoms of liver injury.... (truncated)

Title: Jaundice associated with cephalosporin therapy.
Two cases of cephalosporin-induced hepatotoxicity with associated jaundice are presented. Both patients developed jaundice and liver enzyme abnormalities soon after injectable cephalosporin therapy was started. Other possible causes were ruled out, including TPN and allopurinol hepatoxicity, and additional medical illnesses associated with hepatoxicity. Both patients recovered fully from the episode of jaundice. Review of the literature suggests a possible hypersensitivity reaction similar to liver toxicity of the penicillin antibiotics.

Title: A rare case of cefepime-induced cholestatic liver injury.
Cefepime is widely used in the hospital setting, and only a few studies have reported neurotoxicity and nephrotoxicity as side effects of this drug. Herein, we present a 93-year-old man who exhibited features of cholestatic hepatitis including elevated blood transaminases and direct-form predominant bilirubin levels after administration of cefepime. Blood liver tests showed total recovery after discontinuing the offending agent. Cefepime was probable to cause drug-induced cholestatic hepatitis in our patient since the Roussel Uclaf Causality Assessment Method score for cefepime was 7. No drug interactions were likely according to the Drug Interaction Probability Scale for this patient. No similar... (truncated)

Title: Comparative toxicities of third-generation cephalosporins.
Data on the adverse effects experienced by 2,539 patients who received ceftazidime were compared with adverse effects reported with cefoperazone, cefotaxime, ceftizoxime, and moxalactam. There were 216 such reactions among the ceftazidime-treated patients; 158 patients (6.2 percent) had reactions that were possibly or probably drug-related. The clinical and laboratory safety profile of ceftazidime in regard to renal, hepatic, hematopoietic, and hemostatic parameters compared favorably with that of other third-generation cephalosporins. An increased serum creatinine level was observed in 0.8 percent of ceftazidime-treated patients, an increased blood urea nitrogen level in 1.6 percent, hepatic abnormalities in approximately 6 percent, diarrhea in... (truncated)

MESH:D012256 - riboflavin

Summary:

LLM Prediction Score	0.002 (normalized)
LLM Confidence Score	0.990
Golden Answer (Severity Class)	0.0 (normalized)
Prediction Error	0.002

Retrieved Context:

Title: The Alleviative Effect of Vitamin B₂ on Potassium Bromate-Induced Hepatotoxicity in Male Rats.
Potassium bromate (PB) is a food enhancer, water disinfection by-product, and a proven carcinogen. It elicits toxicities in the living organism due to exposure and in a dose-dependent manner. The present study discourses the ameliorative efficacy of riboflavin (RF) in PB-administered rodents. The animals were distributed into five treatment groups: control (group I), PB alone (group II, 150 mg/kg), RF alone (group III, 2 mg/kg), PB+RF1 (group IV, 150 mg/kg + 2 mg/kg), and PB+RF2 (group V, 150 mg/kg + 4 mg/kg). After the round of the treatment, the animals were sacrificed to collect their blood and liver samples for... (truncated)

Title: Riboflavin ameliorates mitochondrial dysfunction via the AMPK/PGC1α/HO-1 signaling pathway and attenuates carbon tetrachloride-induced liver fibrosis in rats.
Hepatic fibrosis is a global health problem, with increasing evidence demonstrating that oxidative stress serves a pivotal role in fibrogenesis. Riboflavin is a vital nutrient in the human and animal diet, which enhances the activity of antioxidant enzymes and ameliorates oxidative stress. The present study evaluated the effect of riboflavin on liver fibrosis and the mechanisms underlying this process. Rats were subcutaneously injected with carbon tetrachloride (CCl₄) dissolved in sterile olive oil twice per week to induce hepatic fibrosis. The effect of riboflavin on CCl₄-induced liver fibrosis was then assessed. Blood samples and liver tissues were collected and analyzed. The... (truncated)

Title: Acute Liver Failure Following One Year of Daily Consumption of a Sugar-Free Energy Drink.
We report a 36-year-old man who presented with 1 week of right upper quadrant abdominal pain, jaundice, and fatigue. He consumed 3 sugar-free energy drinks daily for the past year with binge alcohol use. His liver function progressively deteriorated, requiring orthotopic liver transplantation. Submassive hepatic necrosis with eosinophilic infiltrate was seen on pathology, consistent with drug-induced liver injury. Further investigation is warranted into identifying which individuals are susceptible to liver failure from energy drink consumption.

Title: Ameliorative effect of riboflavin on the cisplatin induced nephrotoxicity and hepatotoxicity under photoillumination.
Cisplatin is a widely used anticancer drug. It is documented that it elicits major side effects like nephrotoxicity and hepatotoxicity due to oxidative stress forcing the patients to limit its clinical use in long term treatment. Riboflavin (vitamin B(2)) is a strong photosensitizer because it generates reactive oxygen species (ROS) upon photoillumination. We have tried to trap its photosensitizing property to ameliorate the cisplatin induced nephrotoxicity and hepatotoxicity in mice. They were treated with riboflavin and cisplatin separately as well as with their combination under photoilluminated condition. The status of major antioxidant enzymes, antioxidant proteins, functional markers, lipid peroxidation and... (truncated)

Title: Riboflavin: The Health Benefits of a Forgotten Natural Vitamin.
Riboflavin (RF) is a water-soluble member of the B-vitamin family. Sufficient dietary and supplemental RF intake appears to have a protective effect on various medical conditions such as sepsis, ischemia etc., while it also contributes to the reduction in the risk of some forms of cancer in humans. These biological effects of RF have been widely studied for their anti-oxidant, anti-aging, anti-inflammatory, anti-nociceptive and anti-cancer properties. Moreover, the combination of RF and other compounds or drugs can have a wide variety of effects and protective properties, and diminish the toxic effect of drugs in several treatments. Research has been done... (truncated)

MESH:D004921 - erythropoietin

Summary:

LLM Prediction Score	0.002 (normalized)
LLM Confidence Score	0.990
Golden Answer (Severity Class)	0.0 (normalized)
Prediction Error	0.002

Retrieved Context:

Title: Recombinant human erythropoietin prevents etoposide- and methotrexate-induced toxicity in kidney and liver tissues via the regulation of oxidative damage and genotoxicity in Wistar rats.
Etoposide (ETO) and methotrexate (MTX) are two effective chemotherapeutic drugs. However, the clinical use of these drugs is limited by its toxicity in normal tissues, especially in kidney and in liver tissues. Recombinant human erythropoietin (rhEPO), erythropoietin hormone, has also been shown to exert tissue protective effects. The purpose of this study was to explore the protective effect of rhEPO against oxidative stress and genotoxicity induced by ETO and MTX in vivo. Adult male Wistar rats were divided into 10 groups (6 animals each): control group, rhEPO alone group, ETO alone group, MTX alone group and rhEPO + ETO/MTX groups.... (truncated)

Title: The protective effect of recombinant human erythropoietin against cisplatin-induced renal and hepatic dysfunctions in Wistar rats.
Cisplatin (Cisp) is one of the most effective chemotherapeutic drugs. However, the dose of Cisp is greatly limited by its toxicity. Recombinant human erythropoietin (rhEPO), a hormone that regulates hematopoiesis, has also been shown to exert tissue-protective effects. The purpose of this study was to explore the protective effect of rhEPO against Cisp-induced renal and liver dysfunctions. Adult male Wistar rats were divided into six groups of six each: control, rhEPO-alone group, Cisp-alone group and rhEPO + Cisp group (pretreatment, cotreatment and posttreatment conditions). Our results showed that Cisp-induced a marked renal and liver failure characterized by a significant decrease... (truncated)

Title: Erythropoietin inhibits gluconeogenesis and inflammation in the liver and improves glucose intolerance in high-fat diet-fed mice.
Erythropoietin (EPO) has multiple biological functions, including the modulation of glucose metabolism. However, the mechanisms underlying the action of EPO are still obscure. This study is aimed at investigating the potential mechanisms by which EPO improves glucose tolerance in an animal model of type 2 diabetes. Male C57BL/6 mice were fed with high-fat diet (HFD) for 12 weeks and then treated with EPO (HFD-EPO) or vehicle saline (HFD-Con) for two week. The levels of fasting blood glucose, serum insulin and glucose tolerance were measured and the relative levels of insulin-related phosphatidylinositol 3-kinase (PI3K)/Akt, insulin receptor (IR) and IR substrate 1... (truncated)

Title: Role of recombinant human erythropoietin against mitomycin C-induced cardiac, hepatic and renal dysfunction in Wistar rats.
Mitomycin C (MMC) is one of the most effective chemotherapeutic drugs. However, the dose of MMC is greatly limited by its toxicity in normal tissues. Recombinant human erythropoietin (rhEPO), an erythropoietic hormone, has also been shown to exert tissue protective effects. The purpose of this study was to explore the protective effect of rhEPO against MMC-induced heart, liver, and renal dysfunction. Adult male Wistar rats were divided into six groups (with six animals each), namely control, rhEPO alone group, MMC alone group, and rhEPO + MMC group (pre-, co-, and posttreatment conditions). The results showed that MMC induced a marked... (truncated)

Title: Erythropoietin suppresses hepatic steatosis and obesity by inhibiting endoplasmic reticulum stress and upregulating fibroblast growth factor 21.
Erythropoietin (EPO), known primarily for its role in erythropoiesis, was recently reported to play a beneficial role in regulating lipid metabolism; however, the underlying mechanism through which EPO decreases hepatic lipid accumulation requires further investigation. Endoplasmic reticulum (ER) stress may contribute to the progression of hepatic steatosis. The present study investigated the effects of EPO on regulating ER stress in fatty liver. It was demonstrated that EPO inhibited hepatic ER stress and steatosis in vivo and in vitro. Interestingly, these beneficial effects were abrogated in liver-specific sirtuin 1 (SIRT1)-knockout mice compared with wild-type littermates. In addition, in palmitate-treated hepatocytes, small... (truncated)

MESH:D003561 - cytarabine

Summary:

LLM Prediction Score	0.374 (normalized)
LLM Confidence Score	0.990
Golden Answer (Severity Class)	0.375 (normalized)
Prediction Error	0.001

Retrieved Context:

Title: Combination chemotherapy utilizing continuous infusion of intermediate-dose cytarabine for refractory or recurrent acute myeloid leukemia.
Between October 1991 and December 1998, 19 patients (12 males and 7 females) with refractory (six patients) or recurrent (13 patients) AML were treated with a combination chemotherapy of cytarabine given by continuous infusion over 24-h at a rate of 1 upward arrow g/m2 per day for 5 days along with idarubicin (12 upward arrow mg/m2 per day x 3) and etoposide (150 mg /m(2) per day x 3). Median age of the patients was 28 years (range, 15--61). Seven (37%) of 19 patients achieved complete remission (CR) with median CR duration of 6.7 months (range, 2.5--61.4+). Two patients are... (truncated)

Title: The toxicity of cytarabine.
The principal toxicity of standard induction regimens for acute non-lymphocytic leukemia (ANLL) [including cytarabine (ARA-C) 100 mg/m2 for 7 days plus an anthracycline] is myelotoxicity, leading to death in at least 25% of cases during induction in non-selected patients. The complete remission rate is less than 35% in patients over 65 years of age, due in part to an age-related increase of myelotoxicity. The other important adverse effect of standard-dose cytarabine is gastrointestinal toxicity, especially oral mucositis, diarrhoea, intestinal ulceration, ileus and subsequent Gram-negative septicaemia. Idiosyncratic reactions like exanthema, fever and elevation of hepatic enzymes are relatively frequent, but do... (truncated)

Title: Pharmacology and toxicity of high-dose cytarabine by 72-hour continuous infusion.
Most studies using high-dose cytarabine (ara-c) for the therapy of acute leukemia have employed intermittent short infusions. In this study, we have evaluated the pharmacology and toxicity of high-dose ara-c by 72-hour continuous infusion. Plasma ara-c concentrations varied from 3.6 microM at the starting dose of 4 g/m2/72 hours to 22.6 microM at 18 g. Plasma clearance appeared to decrease progressively at doses greater than 10 g, suggesting that the route of elimination was saturable. CSF ara-c concentrations ranged from 1.2 microM at 4 g to 4.1 microM at 18 g; the ratio of CSF to plasma ara-c decreased progressively... (truncated)

Title: Cytarabine and clofarabine after high-dose cytarabine in relapsed or refractory AML patients.
Clofarabine has been shown to be effective in AML patients, either as single agent or, mainly, in association with intermediate dose cytarabine. Based on these reports, we conducted a preliminary study combining clofarabine and intermediate dose cytarabine in AML patients who relapsed or failed to respond to at least two induction therapies. We treated 47 patients affected by relapsed/refractory AML with a regimen including clofarabine at 22.5 mg/m(2) daily on days 1-5, followed after 3 hr by cytarabine at 1 g/m(2) daily on days 1-5. Ten patients received a further consolidation cycle with clofarabine at 22.5 mg/m(2) and cytarabine at... (truncated)

Title: Veno-occlusive disease of the liver associated with chronic myelomonocytic leukemia treated with vincristine and standard doses of cytarabine.
A unique case of a 72-year-old man with chronic myelomonocytic leukemia (CMML) who developed hepatic veno-occlusive disease (VOD) after treatment with a single dose of vincristine and standard doses of cytarabine is described. Unexpected peroneal nerve palsy suggestive of vincristine neurotoxicity occurred concurrently and pointed to vincristine as the most likely cause of the VOD. To the best of our knowledge, association between vincristine and hepatic VOD has not been previously described in chemotherapy-naive patients with CMML.

MESH:D001622 - betaine

Summary:

LLM Prediction Score	0.001 (normalized)
LLM Confidence Score	0.980
Golden Answer (Severity Class)	0.0 (normalized)
Prediction Error	0.001

Retrieved Context:

Title: Betaine transport in kidney and liver: use of betaine in liver injury.
Betaine, also known as trimethylglycine, is an important human nutrient obtained from a variety of foods and also can be synthesized from choline. Betaine is much more abundant in kidney and liver compared to other mammalian organs. The principal role of betaine in the kidney is osmoprotection in cells of the medulla and it enters these cells via the betaine/γ-aminobutyric acid (GABA) transporter protein (BGT1), which is upregulated by hyperosmotic stress. This process has been studied in great detail. In liver, the main role of betaine is a methyl donor in the methionine cycle. However, recent studies showed that BGT1... (truncated)

Title: Beneficial Effects of Betaine: A Comprehensive Review.
Medicinal herbs and many food ingredients possess favorable biological properties that contribute to their therapeutic activities. One such natural product is betaine, a stable, nontoxic natural substance that is present in animals, plants, and microorganisms. Betaine is also endogenously synthesized through the metabolism of choline or exogenously consumed through dietary intake. Betaine mainly functions as (i) an osmolyte and (ii) a methyl-group donor. This review describes the major physiological effects of betaine in whole-body health and its ability to protect against both liver- as well as non-liver-related diseases and conditions. Betaine's role in preventing/attenuating both alcohol-induced and metabolic-associated liver diseases... (truncated)

Title: Hepatoprotective effects of betaine on liver damages followed by myocardial infarction.
Myocardial infarction is commonly considered as a leading cause of cardiovascular disease taking the lives of seven million people annually. Liver dysfunction is associated with cardiac diseases. The profile of abnormal liver functions in heart failure is not clearly defined. This study was designed to investigate the protective effects of betaine on liver injury after myocardial infarction induced by isoprenaline in rats. Forty-eight male rats were divided into four groups: the control group received normal diet and the experimental groups received 50, 150, and 250 mg kg⁻¹ body weight of betaine daily through gastric gavages for 60 days. All of... (truncated)

Title: Protective Effects of S-Adenosylmethionine and Its Combinations With Taurine and/or Betaine Against Lipopolysaccharide or Polyinosinic-polycytidylic Acid-induced Acute Hepatotoxicity.
Several mechanisms for the pathogenesis of many liver diseases are related with oxidative stress, endotoxins, and infections by many microorganisms. These can lead to chronic hepatitis, cirrhosis, and even liver cancer. The aim of this study was to evaluate the effects of S-adenosylmethionine (SAMe) and its combinations with taurine and/or betaine against hepatotoxicites induced by lipopolysaccharide (LPS) or polyinosinic-polycytidylic acid (polyI:C).

Title: Alleviation of Carbon-Tetrachloride-Induced Liver Injury and Fibrosis by Betaine Supplementation in Chickens.
Betaine is a food component with well-reported hepatoprotection effects. However, the effects and mechanisms of betaine on liver fibrosis development are still insufficient. Because metabolic functions of chicken and human liver is similar, we established a chicken model with carbon Tetrachloride- (CCl4-) induced fibrosis for studying antifibrotic effect of betaine in vivo and in vitro. Two-week-old male chicks were supplemented with betaine (1%, w/v) in drinking water for 2 weeks prior to the initiation of CCl4 treatment (i.p.) until sacrifice. Primary chicken hepatocytes were treated with CCl4 and betaine to mimic the in vivo supplementation. The supplementation of betaine significantly... (truncated)

MESH:D013831 - thiamine

Summary:

LLM Prediction Score	0.000 (normalized)
LLM Confidence Score	0.990
Golden Answer (Severity Class)	0.0 (normalized)
Prediction Error	0.000

Retrieved Context:

Title: High-dose vitamin B1 therapy prevents the development of experimental fatty liver driven by overnutrition.
Fatty liver is an abnormal metabolic condition of excess intrahepatic fat. This condition, referred to as hepatic steatosis, is tightly associated with chronic liver disease and systemic metabolic morbidity. The most prevalent form in humans, i.e. non-alcoholic fatty liver, generally develops due to overnutrition and sedentary lifestyle, and has as yet no approved drug therapy. Previously, we have developed a relevant large-animal model in which overnourished sheep raised on a high-calorie carbohydrate-rich diet develop hyperglycemia, hyperinsulinemia, insulin resistance, and hepatic steatosis. Here, we tested the hypothesis that treatment with thiamine (vitamin B1) can counter the development of hepatic steatosis driven... (truncated)

Title: The genesis of alcoholic brain tissue injury.
1. Acetaldehyde has been implicated in the pathogenesis of alcohol-related liver damage by two mechanisms. Adduct formation with many tissue constituents, especially proteins, makes them immunologically foreign or reduces enzyme activity and formation of cytotoxic free radicals from acetaldehyde metabolism. Adduct formation damage to microtubule associated proteins and to hepatocyte membranes impedes protein movement into, out of and around the cell. 2. Evidence that these mechanisms also have a role in alcoholic brain damage includes raised blood acetaldehyde in alcoholics, especially in those chemically dependent, or in other abnormal states; effects of extra-hepatic free radical toxicity, including induction of superoxide... (truncated)

Title: [Vitamin B 1 deficiency in chronic alcoholics and its clinical correlation].
50 chronic alcoholics reporting to the medical emergency ward of Basle University Hospital with alcohol-related illness were examined with respect to thiamine nutritional status by means of the transketolase activation test of erythrocytes (ETK). 46% of the chronic alcoholics, compared to only 2% of the control population (1152 healthy adults), had transketolase activation quotients indicating a strong probability of thiamine deficiency (alphaETK greater than 1.25). The most important symptoms associated with the biochemical parameters of thiamine deficiency were: anemia, pathologic liver functions (bilirubin, gamma-globulins), low diastolic blood pressure and Wernicke's encephalopathy. There was a statistically significant correlation (p less than... (truncated)

Title: Concomitants of alcoholism: differential effects of thiamine deficiency, liver damage, and food deprivation on the rat brain in vivo.
Serious neurological concomitants of alcoholism include Wernicke's encephalopathy (WE), Korsakoff's syndrome (KS), and hepatic encephalopathy (HE).

Title: In vivo evidence for alcohol-induced neurochemical changes in rat brain without protracted withdrawal, pronounced thiamine deficiency, or severe liver damage.
Magnetic resonance spectroscopy (MRS) studies in human alcoholics report decreases in N-acetylaspartate (NAA) and choline-containing (Cho) compounds. Whether alterations in brain metabolite levels are attributable to alcohol per se or to physiological effects of protracted withdrawal or impaired nutritional or liver status remains unclear. Longitudinal effects of alcohol on brain metabolites measured in basal ganglia with single-voxel MRS were investigated in sibling pairs of wild-type Wistar rats, with one rat per pair exposed to escalating doses of vaporized alcohol, the other to vapor chamber air. MRS was conducted before alcohol exposure and twice during exposure. After 16 weeks of alcohol... (truncated)

MESH:D012841 - simethicone

Summary:

LLM Prediction Score	0.000 (normalized)
LLM Confidence Score	0.990
Golden Answer (Severity Class)	0.0 (normalized)
Prediction Error	0.000

Retrieved Context:

Title: Epidemiology, Clinical Features, and Prescribing Patterns of Irritable Bowel Syndrome in Taiwan.
Background: Understanding the prescribing patterns could better inform irritable bowel syndrome (IBS) management and health policy. However, there is no study on prescribing patterns of IBS in Taiwan. This study was conducted to evaluate the epidemiology, clinical features, and prescribing patterns of IBS in Taiwan. Methods: This population-based cross-sectional study was performed by retrieving claim data from National Health Insurance Research Database (NHIRD) between 2011 and 2018 in Taiwan. Patients who were diagnosed with IBS during 2012-2018 and more than 20 years old were included. The annual incidence and prevalence of IBS were estimated. The characteristics and prescribing pattern were evaluated... (truncated)

Title: Carbamazepine overdose after exposure to simethicone: a case report.
Carbamazepine is an anticonvulsant drug and is also used as a treatment for patients with manic-depressive illness, post-herpetic neuralgia or phantom limb pain. The drug itself has many drug interactions. Simethicone is an antifoaming agent and is reported to be an inert material with no known drug interaction with carbamazepine.

Title: Safety assessment of a highly bioavailable curcumin-galactomannoside complex (CurQfen) in healthy volunteers, with a special reference to the recent hepatotoxic reports of curcumin supplements: A 90-days prospective study.
Recently, there is a growing concern about the use of curcumin supplements owing to a few reported hepatotoxicity related adverse events among some of the long-term consumers. Even though no clear evidence was elucidated for the suspected toxicity, the addition of adjuvants that inhibits body's essential detoxification pathways, adulteration with synthetic curcumin, and presence of contaminants including heavy metals, chromate, illegal dyes, non-steroidal anti-inflammatory agents, and pyrrole alkaloids were suggested as plausible reasons. Considering these incidences and speculations, there is a need to critically evaluate the safety of curcumin supplements for prolonged intake. The present study is an evaluation of... (truncated)

Title: Ultrasound Prevalence and Clinical Features of Nonalcoholic Fatty Liver Disease in Patients with Inflammatory Bowel Diseases: A Real-Life Cross-Sectional Study.
<i>Background and Objectives</i>: Inflammatory bowel disease (IBD) is a condition characterized by chronic intestinal inflammation. We can identify two major forms: Crohn's disease (CD) and ulcerative colitis (UC). One of the extraintestinal manifestations of IBD is nonalcoholic fatty liver disease (NAFLD). IBD and NAFLD share common pathogenetic mechanisms. Ultrasound (US) examination is the most commonly used imaging method for the diagnosis of NAFLD. This cross-sectional observational retrospective study aimed to evaluate the US prevalence of NAFLD in IBD patients and their clinical features. <i>Materials and Methods</i>: A total of 143 patients with IBD underwent hepatic US and were divided into... (truncated)

Title: Recent advances in management of the HIV/HCV coinfected patient.
Chronic hepatitis C virus (HCV) is a global epidemic, affecting approximately 150 million individuals throughout the world. The implications of HCV infection have been magnified in those who are infected with both HCV and the HIV as liver disease progression, liver failure and liver-related death are increased, particularly in those without well-controlled HIV disease. The development of direct-acting antiviral agents for HCV that allow shorter treatment periods with increased efficacy and decreased adverse events have greatly changed the outlook for HCV-infected individuals. With these advancements, growing treatment options for the coinfected population have also come. This review will address pharmacotherapy... (truncated)

MESH:D008464 - mecamylamine

Summary:

LLM Prediction Score	0.000 (normalized)
LLM Confidence Score	0.920
Golden Answer (Severity Class)	0.0 (normalized)
Prediction Error	0.000

Retrieved Context:

Title: Huperzine-A Improved Animal Behavior in Cuprizone-Induced Mouse Model by Alleviating Demyelination and Neuroinflammation.
Huperzine A (HupA) is a natural acetylcholinesterase inhibitor (AChEI) with the advantages of high efficiency, selectivity as well as reversibility and can exhibit significant therapeutic effects against certain neurodegenerative diseases. It is also beneficial in reducing the neurological impairment and neuroinflammation of experimental autoimmune encephalomyelitis (EAE), a classic model for multiple sclerosis (MS). However, whether HupA can directly regulate oligodendrocyte differentiation and maturation and promote remyelination has not been investigated previously. In this study, we have analyzed the potential protective effects of HupA on the demylination model of MS induced by cuprizone (CPZ). It was found that HupA significantly attenuated... (truncated)

Title: Cholinergic Dysfunction Involvement in Chronic Cerebral Hypoperfusion-Induced Impairment of Medial Septum-dCA1 Neurocircuit in Rats.
Chronic cerebral hypoperfusion (CCH) is considered a preclinical condition of mild cognitive impairment and thought to precede dementia. However, as the principal cholinergic source of hippocampus, whether the septo-hippocampal neurocircuit was impaired after CCH is still unknown. In this study, we established the CCH rat model by bilateral common carotid artery occlusion (2VO). Under anesthesia, the medial septum (MS) of rats was stimulated to evoke the field excitatory post-synaptic potential (fEPSP) in the pyramidal cell layer of dCA1. Consequently, we observed decreased amplitude of fEPSP and increased paired-pulse ratio (PPR) after 8-week CCH. After tail pinch, we also found decreased... (truncated)

Title: Utility of spherical human liver microtissues for prediction of clinical drug-induced liver injury.
Drug-induced liver injury (DILI) continues to be a major source of clinical attrition, precautionary warnings, and post-market withdrawal of drugs. Accordingly, there is a need for more predictive tools to assess hepatotoxicity risk in drug discovery. Three-dimensional (3D) spheroid hepatic cultures have emerged as promising tools to assess mechanisms of hepatotoxicity, as they demonstrate enhanced liver phenotype, metabolic activity, and stability in culture not attainable with conventional two-dimensional hepatic models. Increased sensitivity of these models to drug-induced cytotoxicity has been demonstrated with relatively small panels of hepatotoxicants. However, a comprehensive evaluation of these models is lacking. Here, the predictive value... (truncated)

Title: Chronic nicotine exposure stimulates biliary growth and fibrosis in normal rats.
Epidemiological studies have indicated smoking to be a risk factor for the progression of liver diseases. Nicotine is the chief addictive substance in cigarette smoke and has powerful biological properties throughout the body. Nicotine has been implicated in a number of disease processes, including increased cell proliferation and fibrosis in several organ systems.

Title: The evolution of strategies to minimise the risk of human drug-induced liver injury (DILI) in drug discovery and development.
Early identification of toxicity associated with new chemical entities (NCEs) is critical in preventing late-stage drug development attrition. Liver injury remains a leading cause of drug failures in clinical trials and post-approval withdrawals reflecting the poor translation between traditional preclinical animal models and human clinical outcomes. For this reason, preclinical strategies have evolved over recent years to incorporate more sophisticated human in vitro cell-based models with multi-parametric endpoints. This review aims to highlight the evolution of the strategies adopted to improve human hepatotoxicity prediction in drug discovery and compares/contrasts these with recent activities in our lab. The key role of... (truncated)

MESH:D000077270 - exenatide

Summary:

LLM Prediction Score	0.000 (normalized)
LLM Confidence Score	0.980
Golden Answer (Severity Class)	0.0 (normalized)
Prediction Error	0.000

Retrieved Context:

Title: Evolution of exenatide as a diabetes therapeutic.
Type 2 diabetes (T2DM) is a disease of epidemic proportion associated with significant morbidity and excess mortality. Optimal glucose control reduces the risk of microvascular and possibly macrovascular complications due to diabetes. However, glycemic control is rarely optimal and several therapeutic interventions for the treatment of diabetes cause hypoglycemia and weight gain; some may exacerbate cardiovascular risk. Exenatide (synthetic exendin-4) is a glucagon-like peptide-1 receptor (GLP-1R) agonist developed as a first-in-class diabetes therapy. This review presents an overview of the evolution of exenatide as a T2DM treatment, beginning with the seminal preclinical discoveries and continuing through to clinical pharmacology... (truncated)

Title: Exenatide Delays the Progression of Nonalcoholic Fatty Liver Disease in C57BL/6 Mice, Which May Involve Inhibition of the NLRP3 Inflammasome through the Mitophagy Pathway.
This study is aimed at investigating whether exenatide (Exe) delays the progression of nonalcoholic fatty liver disease (NAFLD) in C57BL/6 mice by targeting the NLRP3 inflammasome through the autophagy/mitophagy pathway.

Title: Role of Glucagon-Like Peptide-1 Receptor Agonists in the Management of Non-Alcoholic Steatohepatitis: A Clinical Review Article.
Non-alcoholic fatty liver disease (NAFLD) has emerged as one of the lethal causes of chronic liver disease globally. NAFLD can ultimately progress to non-alcoholic steatohepatitis (NASH) given persistent cellular insult. The crux of the problem lies in fat accumulation in the liver, such as increased fatty acid substrates owing to consumption of a high-fat diet, altered gut physiology, and excess adipose tissue. Being the hepatic manifestation of metabolic syndrome, insulin resistance is also among one of the many stimuli. Therefore, drugs, such as glucagon-like peptide-1 receptor agonist (GLP-1 RA) can play a significant role in reducing inflammation, in addition to... (truncated)

Title: Exenatide Prevents Diet-induced Hepatocellular Injury in A CEACAM1-Dependent Mechanism.
The Carcinoembryonic Antigen-Related Cell Adhesion Molecule 1 (CEACAM1) promotes insulin sensitivity by inducing insulin clearance and reducing de novo lipogenesis in the liver. Consistently, <i>Cc1</i>^{-/-} mice with null deletion of Ceacam1 gene exhibit hyperinsulinemia and insulin resistance, in addition to steatohepatitis. They also exhibit early pericellular fibrosis. Redelivering Ceacam1 to the liver reverses the altered metabolism and histopathology of <i>Cc1</i>^{-/-} mice. Exenatide, a long-acting glucagon-like peptide-1 receptor agonist, induces Ceacam1 transcription and consequently, reverses impaired insulin clearance and insulin resistance caused by high-fat intake. Additionally, it reverses fat accumulation in the liver. The current studies show that exenatide also restored... (truncated)

Title: Exaggerated liver injury induced by renal ischemia reperfusion in diabetes: effect of exenatide.
This study was designed to investigate the possible effect of exenatide (Glucagon like Peptide-1 receptor agonist) on liver injury (distant organ) induced by renal ischemia reperfusion (IR) in diabetic rats.

MESH:C053083 - bromfenac

Summary:

LLM Prediction Score	1.000 (normalized)
LLM Confidence Score	0.940
Golden Answer (Severity Class)	1.0 (normalized)
Prediction Error	0.000

Retrieved Context:

Title: Severe hepatotoxicity associated with bromfenac sodium.
Subacute hepatitis and liver failure occurred in a 40-yr-old woman following a 1-month course of treatment with the nonsteroidal anti-inflammatory drug bromfenac. Serologies for hepatitis A, B, and C were negative, as were antinuclear antibodies and ceruloplasmin. A transjugular liver biopsy demonstrated submassive hepatic necrosis. The clinical course was complicated by encephalopathy, fluid retention, and spontaneous bacterial peritonitis, prompting consideration for liver transplantation. With supportive measures, jaundice and fluid retention resolved over a 3-month period. We conclude that prolonged use of bromfenac was the etiological agent in this case, and that this drug can cause severe hepatotoxicity resulting in liver... (truncated)

Title: Acute liver failure associated with prolonged use of bromfenac leading to liver transplantation. The Acute Liver Failure Study Group.
Bromfenac, a nonnarcotic analgesic nonsteroidal anti-inflammatory drug, was associated with reversible, minor elevations in serum aminotransferase levels during clinical trials. The aim of this study is to describe the clinical, laboratory, and histological features of 4 patients with severe bromfenac hepatotoxicity identified at 3 tertiary care centers participating in the US Acute Liver Failure Study Group. Bromfenac was administered for chronic musculoskeletal disorders to 4 women in therapeutic doses of 25 to 100 mg/d for a minimum of 90 days. All patients reported a prodrome of malaise and fatigue and presented with severe, symptomatic hepatocellular injury with associated hypoprothrombinemia. None... (truncated)

Title: Hepatotoxicity of commonly used drugs: nonsteroidal anti-inflammatory drugs, antihypertensives, antidiabetic agents, anticonvulsants, lipid-lowering agents, psychotropic drugs.
Hepatotoxic adverse drug reactions have contributed to the decline of many promising therapies, even among mainstream medication classes (bromfenac and troglitazone are recent examples). The spectrum of nonsteroidal anti-inflammatory drug-related liver toxicity continues to expand, with reports in children, interactive toxicity in persons with hepatitis C, and recognition of the toxicity of both the preferential and selective cyclooxygenase-2 inhibitors. Of the antihypertensive agents, methyldopa is now rarely prescribed and adverse effects are reported infrequently, whereas cases of liver injury associated with the angiotensin receptor and converting enzyme inhibitors are increasingly reported. Of the antidiabetic agents, acarbose, gliclazide, metformin, and human... (truncated)

Title: Evolution of the Food and Drug Administration approach to liver safety assessment for new drugs: current status and challenges.
Prompted by approval in 1997 of troglitazone and bromfenac, two drugs that promptly began to show serious and sometimes fatal liver toxicity, we began at the Food and Drug Administration (FDA) a series of annual conferences in 1999 to consider issues of drug-induced liver injury (DILI). First inviting reviewers of new drug applications we opened the audiences in 2001 to pharmaceutical industry and academic consultants to industry and FDA, and slides shown at the meetings were posted on the internet to be available at the website of the American Association for the Study of Liver Diseases (AASLD)-go to (http://www.aasld.org/dili/Pages/default.aspx... (truncated)

Title: Non-steroidal anti-inflammatory drugs: What is the actual risk of liver damage?
Non-steroidal anti-inflammatory drugs (NSAIDs) constitute a family of drugs, which taken as a group, represents one of the most frequently prescribed around the world. Thus, not surprisingly NSAIDs, along with anti-infectious agents, list on the top for causes of Drug-Induced Liver Injury (DILI). The incidence of liver disease induced by NSAIDs reported in clinical studies is fairly uniform ranging from 0.29/100 000 [95% confidence interval (CI): 0.17-051] to 9/100 000 (95% CI: 6-15). However, compared with these results, a higher risk of liver-related hospitalizations was reported (3-23 per 100 000 patients). NSAIDs exhibit a broad spectrum of liver damage ranging... (truncated)

MESH:C029755 - benzonatate

Summary:

LLM Prediction Score	0.000 (normalized)
LLM Confidence Score	0.970
Golden Answer (Severity Class)	0.0 (normalized)
Prediction Error	0.000

Retrieved Context:

Title: Ocular manifestations of drug and alcohol abuse.
The purpose of this study is to review commonly encountered adverse ocular effects of illicit drug use.

Title: Risk factors for idiosyncratic drug-induced liver injury.
Idiosyncratic drug-induced liver injury (DILI) is a rare disorder that is not related directly to dosage and little is known about individuals who are at increased risk. There are no suitable preclinical models for the study of idiosyncratic DILI and its pathogenesis is poorly understood. It is likely to arise from complex interactions among genetic, nongenetic host susceptibility, and environmental factors. Nongenetic risk factors include age, sex, and other diseases (eg, chronic liver disease or human immunodeficiency virus infection). Compound-specific risk factors include daily dose, metabolism characteristics, and propensity for drug interactions. Alcohol consumption has been proposed as a risk... (truncated)

Title: A young man with myelosuppression caused by clindamycin: a case report.
Clindamycin is used to treat various bacterial infections, but its administration can cause anaphylaxis, liver reactions, pseudomembranous colitis, and peripheral blood cytopenias (anemia, neutropenia, and thrombocytopenia), alone or in combination. We report the case of a patient with a recurrent infection of the tonsils who received clindamycin. Pancytopenia, a previously unreported hematological disorder related to clindamycin use, was observed in conjunction with the infection and clindamycin treatment.

Title: Antitussives and substance abuse.
Abuse of antitussive preparations is a continuing problem in the United States and throughout the world. Illicit, exploratory, or recreational use of dextromethorphan and codeine/promethazine cough syrups is widely described. This review describes the pharmacology, clinical effects, and management of toxicity from commonly abused antitussive formulations.

Title: Relationship between characteristics of medications and drug-induced liver disease phenotype and outcome.
It is not known whether specific characteristics of medication are associated with type of drug-induced liver injury (DILI) or outcome. We examined the relationships among select characteristics of medications and DILI phenotype and outcome.

MESH:C015121 - dexbrompheniramine

Summary:

LLM Prediction Score	0.000 (normalized)
LLM Confidence Score	0.830
Golden Answer (Severity Class)	0.0 (normalized)
Prediction Error	0.000

Retrieved Context:

Title: Hepatitis C virus infection influences the S-methadone metabolite plasma concentration.
Heroin-dependent patients typically contract hepatitis C virus (HCV) at a disproportionately high level due to needle exchange. The liver is the primary target organ of HCV infection and also the main organ responsible for drug metabolism. Methadone maintenance treatment (MMT) is a major treatment regimen for opioid dependence. HCV infection may affect methadone metabolism but this has rarely been studied. In our current study, we aimed to test the hypothesis that HCV may influence the methadone dosage and its plasma metabolite concentrations in a MMT cohort from Taiwan.

Title: State of the Art and Uses for the Biopharmaceutics Drug Disposition Classification System (BDDCS): New Additions, Revisions, and Citation References.
The Biopharmaceutics Drug Disposition Classification system (BDDCS) is a four-class approach based on water solubility and extent of metabolism/permeability rate. Based on the BDDCS class to which a drug is assigned, it is possible to predict the role of metabolic enzymes and transporters on the drug disposition of a new molecular entity (NME) prior to its administration to animals or humans. Here, we report a total of 1475 drugs and active metabolites to which the BDDCS is applied. Of these, 379 are new entries, and 1096 are revisions of former classification studies with the addition of references for the approved... (truncated)

Title: Prevalence and factors associated with potentially inappropriate medication use in older medicare beneficiaries with cancer.
To assess the factors related to potentially inappropriate medication (PIM) use in elderly patients with cancer, as well as to compare the PIM prevalence in older adults with and without cancer.

Title: Delivery of therapeutic oligonucleotides with cell penetrating peptides.
Oligonucleotide-based drugs have received considerable attention for their capacity to modulate gene expression very specifically and as a consequence they have found applications in the treatment of many human acquired or genetic diseases. Clinical translation has been often hampered by poor biodistribution, however. Cell-penetrating peptides (CPPs) appear as a possibility to increase the cellular delivery of non-permeant biomolecules such as nucleic acids. This review focuses on CPP-delivery of several classes of oligonucleotides (ONs), namely antisense oligonucleotides, splice switching oligonucleotides (SSOs) and siRNAs. Two main strategies have been used to transport ONs with CPPs: covalent conjugation (which is more appropriate for... (truncated)

Title: Effects and Safety of the Tripterygium Glycoside Adjuvant Methotrexate Therapy in Rheumatoid Arthritis: A Systematic Review and Meta-Analysis.
This study aimed to systematically review the efficacy and clinical safety of different courses and doses of tripterygium glycoside (TG) adjuvant methotrexate (MTX) therapy in the treatment of rheumatoid arthritis (RA).

MESH:D011413 - propantheline

Summary:

LLM Prediction Score	0.000 (normalized)
LLM Confidence Score	0.950
Golden Answer (Severity Class)	0.0 (normalized)
Prediction Error	0.000

Retrieved Context:

Title: Utility of spherical human liver microtissues for prediction of clinical drug-induced liver injury.
Drug-induced liver injury (DILI) continues to be a major source of clinical attrition, precautionary warnings, and post-market withdrawal of drugs. Accordingly, there is a need for more predictive tools to assess hepatotoxicity risk in drug discovery. Three-dimensional (3D) spheroid hepatic cultures have emerged as promising tools to assess mechanisms of hepatotoxicity, as they demonstrate enhanced liver phenotype, metabolic activity, and stability in culture not attainable with conventional two-dimensional hepatic models. Increased sensitivity of these models to drug-induced cytotoxicity has been demonstrated with relatively small panels of hepatotoxicants. However, a comprehensive evaluation of these models is lacking. Here, the predictive value... (truncated)

Title: Pharmacological interventions for clozapine-induced hypersalivation.
Clozapine is widely used for people with schizophrenia. Although agranulocytosis, weight gain, and cardiac problems are serious problems associated with its use, hypersalivation, sometimes of a gross and socially unacceptable quantity, is also common (30-80%).

Title: Prevalence and factors associated with potentially inappropriate medication use in older medicare beneficiaries with cancer.
To assess the factors related to potentially inappropriate medication (PIM) use in elderly patients with cancer, as well as to compare the PIM prevalence in older adults with and without cancer.

Title: Detecting Novel Ototoxins and Potentiation of Ototoxicity by Disease Settings.
Over 100 drugs and chemicals are associated with permanent hearing loss, tinnitus, and vestibular deficits, collectively known as ototoxicity. The ototoxic potential of drugs is rarely assessed in pre-clinical drug development or during clinical trials, so this debilitating side-effect is often discovered as patients begin to report hearing loss. Furthermore, drug-induced ototoxicity in adults, and particularly in elderly patients, may go unrecognized due to hearing loss from a variety of etiologies because of a lack of baseline assessments immediately prior to novel therapeutic treatment. During the current pandemic, there is an intense effort to identify new drugs or repurpose FDA-approved... (truncated)

Title: Dynamic carbon 13 breath tests for the study of liver function and gastric emptying.
In gastroenterological practice, breath tests (BTs) are diagnostic tools used for indirect, non-invasive assessment of several pathophysiological metabolic processes, by monitoring the appearance in breath of a metabolite of a specific substrate. Labelled substrates originally employed radioactive carbon 14 ((14)C) and, more recently, the stable carbon 13 isotope ((13)C) has been introduced to label specific substrates. The ingested (13)C-substrate is metabolized, and exhaled (13)CO2 is measured by mass spectrometry or infrared spectroscopy. Some (13)C-BTs evaluate specific (microsomal, cytosolic, and mitochondrial) hepatic metabolic pathways and can be employed in liver diseases (i.e. simple liver steatosis, non-alcoholic steato-hepatitis, liver fibrosis, cirrhosis, hepatocellular... (truncated)

MESH:C054871 - ioversol

Summary:

LLM Prediction Score	0.000 (normalized)
LLM Confidence Score	0.960
Golden Answer (Severity Class)	0.0 (normalized)
Prediction Error	0.000

Retrieved Context:

Title: Contrast-Induced Encephalopathy after Cerebral Angiogram: A Case Series and Review of Literature.
Contrast-induced encephalopathy (CIE) is a rare complication that arises from exposure to iodinated contrast medium and can result in a range of symptoms, including cortical blindness, aphasia, focal neurological deficits, and altered mental status. We present 4 individual cases of CIE who presented with stroke-mimic symptoms following surgery with localized iodixanol or ioversol injection. We outline a clinical timeline of all patients, showing that CIE follows a general pattern of delayed onset, worsening symptomology, and ultimately full recovery. All patients received IV hydration, corticosteroids, or both as part of their treatment protocol.

Title: The Image Quality and Diagnostic Performance of CT with Low-Concentration Iodine Contrast (240 mg Iodine/mL) for the Abdominal Organs.
Purpose: To evaluate the difference between CT examinations using 240 mgI/mL contrast material (CM) and 320 mgI/mL CM in the contrast enhancement of the abdominal organs and the diagnostic performance for focal hepatic lesions. Materials and methods: This retrospective study included 422 CT examinations, using 240 mgI/mL iohexol (Group A, 206 examinations) and 320 mgI/mL ioversol (Group B, 216 examinations), performed between April 2019 and May 2020. Two CT scanners (single-source CT (machine A) and dual-source CT (machine B)) were used to obtain CT images. Two radiologists independently drew regions of interest (ROIs) in the liver, pancreas, spleen, kidney, aorta,... (truncated)

Title: Sulforaphane Attenuates Contrast-Induced Nephropathy in Rats via Nrf2/HO-1 Pathway.
Background. Oxidative stress plays an important role in the pathogenesis of contrast-induced nephropathy (CIN). The aim of this study was to investigate the antioxidant effects of sulforaphane (SFN) in a rat model of CIN and a cell model of oxidative stress in HK2 cells. Methods. Rats were randomized into four groups (n = 6 per group): control group, Ioversol group (Ioversol-induced CIN), Ioversol + SFN group (CIN rats pretreated with SFN), and SFN group (rats treated with SFN). Renal function tests, malondialdehyde (MDA), and reactive oxygen species (ROS) were measured. Western blot, real-time polymerase chain reaction analysis, and immunohistochemical analysis... (truncated)

Title: Double-low protocol for hepatic dynamic CT scan: Effect of low tube voltage and low-dose iodine contrast agent on image quality.
The radiation-induced carcinogenesis from computed tomography (CT) and iodine contrast agent induced nephropathy has attracted international attention. The reduction of the radiation dose and iodine intake in CT scan is always a direction for researchers to strive. The purpose of this study was to evaluate the feasibility of a "double-low" (i.e., low tube voltage and low-dose iodine contrast agent) scanning protocol for dynamic hepatic CT with the adaptive statistical iterative reconstruction (ASIR) in patients with a body mass index (BMI) of 18.5 to 27.9 kg/m.A total of 128 consecutive patients with a BMI between 18.5 and 27.9 kg/m were randomly... (truncated)

Title: Effect of varying rates of low-osmolarity contrast media injection for hepatic CT: correlation with indocyanine green transit time.
Contrast enhancement in hepatic computed tomography (CT) is related to multiple factors, including the amount of iodine injected, the rate of injection, and body weight. Fifty patients were randomized into two groups: 19 patients (group 1) received a 160-mL dose of Optiray 320 (ioversol) at 3.0 mL/sec, and 31 (group 2) received the same dose at 4.5 mL/sec. Indocyanine green dye transit time, peak enhancement, delayed enhancement, time to peak enhancement, age, and weight were statistically analyzed. Time to peak enhancement was significantly shorter in group 2 than in group 1 (62 seconds vs 73 seconds, respectively; P less than... (truncated)

MESH:D007069 - ifosfamide

Summary:

LLM Prediction Score	0.500 (normalized)
LLM Confidence Score	0.990
Golden Answer (Severity Class)	0.5 (normalized)
Prediction Error	0.000

Retrieved Context:

Title: Acute liver toxicity with ifosfamide in the treatment of sarcoma: a case report.
Ifosfamide is a chemotherapy agent infrequently associated with liver toxicity. To the best of our knowledge, this report is the first to describe serious liver toxicity associated with ifosfamide used in combination with doxorubicin that caused acute but fully reversible liver failure and encephalopathy. This report reviews the possible mechanisms by which ifosfamide causes this adverse effect.

Title: Toxicity of high-dose ifosfamide in children.
Ifosfamide has been shown to be an active agent in the treatment of several childhood cancers. However, the optimal dose and method of administration remains to be established. The dose/response relationship of ifosfamide suggests that a maximum tolerable, fractionated dose be given, and to reduce hospitalisation this dose should be given in the shortest possible time. A total of 20 patients aged 1-23 years received 124 courses (mean, 6 courses/patient; range, 1-16); 9 subjects had either relapsed or resistant disease, and all of these had previously received cyclophosphamide. A dose of 3 g/m2 ifosfamide was given for 2 (five patients)... (truncated)

Title: High-dose chemotherapy with carboplatin, etoposide and ifosfamide followed by autologous stem cell rescue in patients with relapsed or refractory malignant lymphomas: a phase I/II study.
Patients with relapsed or refractory non-Hodgkin's lymphomas (NHL) and Hodgkin's disease (HD) with recurrences after an anthracyclin-containing regimen only have a chance of cure of below 10% with conventional chemotherapy. In order to improve their prognosis, we started a phase I/II trial using high-dose therapy comprising carboplatin, together with etoposide and ifosfamide (CEI), followed by autologous stem cell rescue (ASCR) as consolidation after salvage treatment. Since September 1990, 40 patients with intensively pretreated advanced NHL (n = 24) or HD (n = 16) received one cycle of high-dose therapy (HDT) consisting of carboplatin 1500 mg/m2, ifosfamide 10 g/m2 and etoposide... (truncated)

Title: Investigation of Ifosfamide Toxicity Induces Common Upstream Regulator in Liver and Kidney.
Ifosfamide is an alkylating agent, a synthetic analogue of cyclophosphamide, used to treat various solid cancers. In this study, the toxicity of ifosfamide was evaluated using single-and multiple-dose intraperitoneal administration in rats under Good Laboratory Practice guidelines, and an additional microarray experiment was followed to support toxicological findings. A single dose of ifosfamide (50 mg/kg) did not induce any pathological changes. Meanwhile, severe renal toxicity was observed in the 7 and 28 days consecutively administered groups, with significant increases in blood urea nitrogen and creatinine levels. In the tox-list analysis, cholesterol synthesis-related genes were mostly affected in the liver and... (truncated)

Title: [Hepatotoxicity with etoposide-ifosfamide combination therapy].
Combination chemotherapy with etoposide and ifosfamide in 2 patients with advanced bronchial carcinomas caused hepatotoxic side effects. Hepatotoxicity was observed during and immediately after the 5-day therapy and was characterised by a massive rise in direct bilirubin, drop in cholinesterase, as well as a rise in the alkaline phosphatase and to a lesser extent in gGT and GOT. Hepatotoxicity was lethal in one case and reversed to normal in the other. Hepatotoxicity was most likely caused by ifosfamide, since this drug was applied for the first time in both cases. Nonetheless, a contribution of etoposide in this regard can not... (truncated)

MESH:D057073 - thyrotropin alfa

Summary:

LLM Prediction Score	0.000 (normalized)
LLM Confidence Score	0.870
Golden Answer (Severity Class)	0.0 (normalized)
Prediction Error	0.000

Retrieved Context:

Title: Propylthiouracil hepatitis: report of a case and extensive review of the literature.
Antithyroid drugs (ATDs) have been widely and effectively used for the treatment of pediatric and adult thyrotoxicosis for more than a half century. Since the very beginning of ATD use, reports of hepatic dysfunction related to propylthiouracil (PTU) therapy have been published. We describe a case of a 12-year-old girl, who, after 4 weeks of therapy for Graves disease (GD) with PTU (300 mg/day at 100 mg given three times a day), developed fatigue, fever, diarrhea, nausea, and vomiting. The initial diagnosis was "viral gastrointestinal infection". Few days after the initiation of her symptoms, the patient developed jaundice, hepatic tenderness,... (truncated)

Title: A Case of Propylthiouracil-Induced Hepatitis during Pregnancy.
A 32-year-old with no pre-existing liver disease was diagnosed with Graves' disease at week 4 of pregnancy. Thyroid-stimulating hormone was undetectable with elevated free thyroxine levels and positive thyroid receptor antibodies. She was started on a reducing regime of propylthiouracil (PTU). At week 20 in pregnancy, she became jaundiced. Initial bloods revealed: bilirubin 91 µmol/l, alanine aminotransferase 1,796 IU/l, alkaline phosphatase 200 IU/l, international normalized ratio 1.2, and albumin 33 g/l. A presumptive diagnosis of PTU-induced hepatitis was made. PTU was immediately discontinued and best supportive care instigated. Serum markers for autoimmune and viral hepatitis were negative, abdomen ultrasound, ferritin... (truncated)

Title: [Two cases of serious hepatic injury caused by antithyroid drugs].
We present two cases of severe hepatotoxicity caused by antithyroid drugs in women with Graves disease. The first medication in both cases was methimazole replaced by propylhiouracil due to intolerance. The main symptom was jaundice lasting about 2 months. As a first step in the treatment we terminated the antithyroid drug administration and introduced prednisone and ursodesoksycholic acid therapy. The termination of thyrotoxicosis by means of 131 I administration had also positive influence on the course of disease. In summary we emphasise the significance of prompt discontinuation of antithyroid drug once toxic symptoms appeared and avoid replacement one thyrostatic drug... (truncated)

Title: Rescue of Graves Thyrotoxicosis-Induced Cholestatic Liver Disease Without Antithyroid Drugs: A Case Report.
Graves thyrotoxicosis rarely presents with painless jaundice resulting from hyperthyroidism-associated hepatotoxicity, without preexisting liver disease. Management in patients with this presentation is challenging, given that the thionamides, methimazole and propylthiouracil, have both been associated with drug-induced liver injury. Radioactive iodine ablation and thyroidectomy are well-established alternatives, but each have their associated risks and contraindications. We present an unusual case of severe hyperthyroidism-associated hepatotoxicity, in which adjuvant therapies, including glucocorticoids, saturated solution of potassium iodide, and cholestyramine, were used as a bridge to definitive therapy with thyroidectomy.

Title: Propylthiouracil-induced liver failure and artificial liver support systems: a case report and review of the literature.
Antithyroid drugs carry a potential risk of hepatotoxicity. Propylthiouracil (PTU) is commonly prescribed for patients with hyperthyroidism. PTU, however, can induce liver injury, ranging from mild asymptomatic elevation of aminotransferases to acute liver failure (ALF).

MESH:C118563 - clevidipine

Summary:

LLM Prediction Score	0.000 (normalized)
LLM Confidence Score	0.930
Golden Answer (Severity Class)	0.0 (normalized)
Prediction Error	0.000

Retrieved Context:

Title: Role of clevidipine butyrate in the treatment of acute hypertension in the critical care setting: a review.
Acutely elevated blood pressure in the critical care setting is associated with a higher risk of acute end-organ damage (eg, myocardial ischemia, stroke, and renal failure) and perioperative bleeding. Urgent treatment and careful blood pressure control are crucial to prevent significant morbidity. Clevidipine butyrate (Cleviprex) is an ultrashort-acting, third-generation intravenous calcium channel blocker. It is an arterial-selective vasodilator with no venodilatory or myocardial depressive effects. Clevidipine has an extremely short half-life of approximately 1 minute as it is rapidly metabolized by blood and tissue esterases. These metabolites are then primarily eliminated through urine and fecal pathways. The rapid onset and... (truncated)

Title: Perioperative blood pressure management with clevidipine during coiling of cerebral artery aneurysms.
The tight control of blood pressure (BP) is mandatory during cerebral aneurysm coiling to minimize abrupt changes in the transmural pressure across the aneurysm and thereby decrease the risk of rupture. Critical times during these procedures when significant BP changes may occur include anesthetic induction, endotracheal intubation, and emergence. Clevidipine is a recently introduced, rapidly acting dihydropyridine calcium channel antagonist. Its rapid metabolism by tissue and plasma esterases results in an effective half-life of 1 to 3 minutes. We present our preliminary experience with the use of clevidipine to control BP during the anesthetic care of three patients undergoing coiling... (truncated)

Title: Intravenous nicardipine and labetalol use in hypertensive patients with signs or symptoms suggestive of end-organ damage in the emergency department: a subgroup analysis of the CLUE trial.
To compare the efficacy of Food and Drug Administration recommended dosing of nicardipine versus labetalol for the management of hypertensive patients with signs and/or symptoms (S/S) suggestive of end-organ damage (EOD).

Title: Association of CYP1A1 and CYP1B1 inhibition in in vitro assays with drug-induced liver injury.
Drug-induced liver injury (DILI) is one of the major causes for the discontinuation of drug development and withdrawal of drugs from the market. Since it is known that reactive metabolite formation and being substrates or inhibitors of cytochrome P450s (P450s) are associated with DILI, we systematically investigated the association between human P450 inhibition and DILI. The inhibitory activity of 266 DILI-positive drugs (DILI drugs) and 92 DILI-negative drugs (no-DILI drugs), which were selected from Liver Toxicity Knowledge Base (US Food and Drug Administration), against 8 human P450 forms was assessed using recombinant enzymes and luminescent substrates, and the threshold values... (truncated)

Title: Control of hypertension in the critically ill: a pathophysiological approach.
Severe acute arterial hypertension can be associated with significant morbidity and mortality. After excluding a reversible etiology, choice of therapeutic intervention should be based on evaluation of a number of factors, such as age, comorbidities, and other ongoing therapies. A rational pathophysiological approach should then be applied that integrates the effects of the drug on blood volume, vascular tone, and other determinants of cardiac output. Vasodilators, calcium channel blockers, and beta-blocking agents can all decrease arterial pressure but by totally different modes of action, which may be appropriate or contraindicated in individual patients. There is no preferred agent for all... (truncated)

MESH:D004400 - dyphylline

Summary:

LLM Prediction Score	0.000 (normalized)
LLM Confidence Score	0.920
Golden Answer (Severity Class)	0.0 (normalized)
Prediction Error	0.000

Retrieved Context:

Title: Clinical characteristics of antiepileptic-induced liver injury in patients from the DILIN prospective study.
Antiepileptic drugs (AEDs) are a common cause of drug-induced liver injury (DILI). Over the last few decades, several newer AEDs were approved for marketing in the United States, and they are increasingly prescribed for indications other than seizures. Contemporaneous data related to trends and characteristics of AED-related liver injury are sparse.

Title: Leflunomide-induced liver injury: Differences in characteristics and outcomes in Indian and US registries.
Leflunomide, a disease-modifying anti-rheumatic drug, has been associated with elevations of serum aminotransferases. Herein, we describe the clinical, laboratory features and outcomes of 17 patients with leflunomide/teriflunomide hepatotoxicity from two large drug-induced liver injury (DILI) registries.

Title: Adverse Effects of Kampo Medicines.
This review summarizes the adverse effects of Kampo medicines. These adverse effects in terms of immunoallergic reactions include interstitial pneumonia, liver injury, allergic cystitis, and drug eruption. Many cases of interstitial pneumonia, liver injury, and allergic cystitis associated with Kampo formulas have been reported to be caused by formulas containing Scutellariae Radix (Scutellaria root, ogon). The known adverse effects linked to overdose of Kampo formulas include pseudoaldosteronism [caused by Glycyrrhizae Radix (licorice, kanzo)], sympathomimetic symptoms [caused by Ephedrae Herba (ephedra, mao)], aconite poisoning [caused by Aconiti Tuber (processed aconite root, bushi and uzu)], and diarrhea [caused by Rhei Rhizoma (rhubarb,... (truncated)

Title: Incidence of Hepatitis E Infection in American Patients With Suspected Drug-Induced Liver Injury Is Low and Declining: The DILIN Prospective Study.
Hepatitis E virus (HEV) infection rarely causes icteric hepatitis, yet 10%-40% of adult Americans have serological evidence of previous infection. The aim of this study was to investigate the incidence, presentation, and outcome of acute and previous HEV infection in a large cohort of patients with suspected drug-induced liver injury (DILI).

Title: A revised electronic version of RUCAM for the diagnosis of DILI.
Roussel Uclaf Causality Assessment Method (RUCAM) for DILI has been hindered by subjectivity and poor reliability. We sought to improve the RUCAM using data from the Drug-Induced Liver Injury Network (DILIN) and the Spanish DILI Registry, published literature, and iterative computer modeling.

MESH:D015248 - gemfibrozil

Summary:

LLM Prediction Score	0.500 (normalized)
LLM Confidence Score	0.970
Golden Answer (Severity Class)	0.5 (normalized)
Prediction Error	0.000

Retrieved Context:

Title: Gemfibrozil disrupts lysophosphatidylcholine and bile acid homeostasis via PPARα and its relevance to hepatotoxicity.
Gemfibrozil, a ligand of peroxisome proliferator-activated receptor α (PPARα), is one of the most widely prescribed anti-dyslipidemia fibrate drugs. Among the adverse reactions observed with gemfibrozil are alterations in liver function, cholestatic jaundice, and cholelithiasis. However, the mechanisms underlying these toxicities are poorly understood. In this study, wild-type and Ppara-null mice were dosed with a gemfibrozil-containing diet for 14 days. Ultra-performance chromatography electrospray ionization quadrupole time-of-flight mass spectrometry-based metabolomics and traditional approaches were used to assess the mechanism of gemfibrozil-induced hepatotoxicity. Unsupervised multivariate data analysis revealed four lysophosphatidylcholine components in wild-type mice that varied more dramatically than those in Ppara-null... (truncated)

Title: Enhanced antihyperlipidemic potential of gemfibrozil under co-administration with piperine.
Gemfibrozil is a well-known potent antihyperlipidemic drug with the capacity to lower triglyceride and cholesterol levels, which are responsible for most cardiovascular and cerebrovascular diseases. In addition, gemfibrozil has a potent activity at elevating the high density lipoprotein levels. However, this drug has a very short half-life of about 2 h and toxicity is observed in the liver as the dose increases. The drug piperine has the capacity to enhance the bioavailability of other drugs without altering their basic properties as well as improving their activity. In this study, we aimed to enhance the bioavailability of gemfibrozil as well as making... (truncated)

Title: Gemfibrozil hepatotoxicity: a case report.
A 55-year-old woman was admitted to our hospital for management of thoracic trauma and bone fractures. One month after admission she started to receive gemfibrozil for hypertriglyceridemia. In the second month of admission, the patient complained of nausea and malaise. Laboratory value showed an acute hepatitis with raised AST, ALT. The abdominal ultrasound scan was normal, and viral serologic tests were negative. Gemfibrozil was discontinued and in a few days AST and ALT levels returned to normal.

Title: [Side effects of fibrates (except liver and muscle)].
If the iatrogenic acute muscular syndromes (rhabdomyolysis) and hepatic diseases following hypolipidemic drugs therapy are very well known, the other unwanted effects associated with fibrate therapy are not well established. It is the reason why we have selected, in the computerised data base of side effects from the French Network of "Centres de Pharmacovigilance" organisation, the pathological events associated with fibrate therapy during five years (1985 to 1989) (with exception for the acute muscular and hepatic diseases). The 277 side effects represent 67% of the side effects in which the product is regarded as "suspect" (S). These effects were observed... (truncated)

Title: Lipid-lowering drugs and serum liver enzymes: the effects of body weight and baseline enzyme levels.
The most important side effects of fibrate and 3-hydroxy-3-methylglutaryl-coenzyme A (HMG-CoA) reductase inhibitor (statin) treatment are hepatic toxicity and myopathy. Obese individuals may have higher levels of serum transaminases than their lean counterparts. The main purpose of this study was to examine the effects of statins and fibrates on liver enzymes in obese patients and to compare them with their effects on patients with various body mass indexes (BMI). Two hundred and sixty-three hyperlipidemic patients of both sexes aged 31-74 years were studied for 24 weeks. One hundred and three patients received fluvastatin (40 mg/day), 62 atorvastatin (10-20 mg/day), 45... (truncated)

MESH:D011352 - procyclidine

Summary:

LLM Prediction Score	0.000 (normalized)
LLM Confidence Score	0.930
Golden Answer (Severity Class)	0.0 (normalized)
Prediction Error	0.000

Retrieved Context:

Title: Utility of spherical human liver microtissues for prediction of clinical drug-induced liver injury.
Drug-induced liver injury (DILI) continues to be a major source of clinical attrition, precautionary warnings, and post-market withdrawal of drugs. Accordingly, there is a need for more predictive tools to assess hepatotoxicity risk in drug discovery. Three-dimensional (3D) spheroid hepatic cultures have emerged as promising tools to assess mechanisms of hepatotoxicity, as they demonstrate enhanced liver phenotype, metabolic activity, and stability in culture not attainable with conventional two-dimensional hepatic models. Increased sensitivity of these models to drug-induced cytotoxicity has been demonstrated with relatively small panels of hepatotoxicants. However, a comprehensive evaluation of these models is lacking. Here, the predictive value... (truncated)

Title: An in vitro coculture system of human peripheral blood mononuclear cells with hepatocellular carcinoma-derived cells for predicting drug-induced liver injury.
Preventing clinical drug-induced liver injury (DILI) remains a major challenge, because DILI develops via multifactorial mechanisms. Immune and inflammatory reactions are considered important mechanisms of DILI; however, biomarkers from in vitro systems using immune cells have not been comprehensively studied. The aims of this study were (1) to identify promising biomarker genes for predicting DILI in an in vitro coculture model of peripheral blood mononuclear cells (PBMCs) with a human liver cell line, and (2) to evaluate these genes as predictors of DILI using a panel of drugs with different clinical DILI risk. Transcriptome-wide analysis of PBMCs cocultured with HepG2... (truncated)

Title: Human hepatic in vitro models reveal distinct anti-NASH potencies of PPAR agonists.
Non-alcoholic steatohepatitis (NASH) is a highly prevalent, chronic liver disease characterized by hepatic lipid accumulation, inflammation, and concomitant fibrosis. Up to date, no anti-NASH drugs have been approved. In this study, we reproduced key NASH characteristics in vitro by exposing primary human hepatocytes (PHH), human skin stem cell-derived hepatic cells (hSKP-HPC), HepaRG and HepG2 cell lines, as well as LX-2 cells to multiple factors that play a role in the onset of NASH. The obtained in vitro disease models showed intracellular lipid accumulation, secretion of inflammatory chemokines, induced ATP content, apoptosis, and increased pro-fibrotic gene expression. These cell systems were... (truncated)

Title: Fluphenazine (oral) versus placebo for schizophrenia.
Fluphenazine is one of the first drugs to be classed as an 'antipsychotic' and has been widely available for five decades.

Title: Inflammatory response to clozapine in the absence of myocarditis: case report.
A case is presented of a 25-year-old man with treatment-resistant paranoid schizophrenia whose only previous trial of clozapine had been stopped following a suspected clozapine-induced myocarditis. Due to the failure of his psychosis to respond to a number of antipsychotic treatments and augmentation strategies, clozapine was restarted on admission. His rechallenge was marked by intermittent pyrexia, tachycardia and elevated C-reactive protein (CRP), but eosinophilia was absent. Clozapine was started and then stopped twice following extensive investigation and with specialist cardiology consultation. Physical symptoms and CRP elevation resolved shortly after clozapine cessation. We believe this constituted an idiosyncratic systemic inflammatory response... (truncated)

MESH:D001712 - biperiden

Summary:

LLM Prediction Score	0.000 (normalized)
LLM Confidence Score	0.960
Golden Answer (Severity Class)	0.0 (normalized)
Prediction Error	0.000

Retrieved Context:

Title: Selected Molecular Targets for Antiepileptogenesis.
The term epileptogenesis defines the usually durable process of converting normal brain into an epileptic one. The resistance of a significant proportion of patients with epilepsy to the available pharmacotherapy prompted the concept of a causative treatment option consisting in stopping or modifying the progress of epileptogenesis. Most antiepileptic drugs possess only a weak or no antiepileptogenic potential at all, but a few of them appear promising in this regard; these include, for example, eslicarbazepine (a sodium and T-type channel blocker), lamotrigine (a sodium channel blocker and glutamate antagonist) or levetiracetam (a ligand of synaptic vehicle protein SV2A). Among the... (truncated)

Title: Trifluoperazine-induced cholestatic jaundice.
Liver injury occurs with many drugs; therefore, a thorough work up is important for establishing the diagnosis. We report a case of trifluoperazine-induced cholestatic jaundice. A 44-year old male with schizoaffective disorder developed an increase in liver enzymes and jaundice after starting treatment with trifluoperazine. Workup for other potential etiologies was negative.

Title: Critical appraisal of lurasidone in the management of schizophrenia.
Lurasidone is a new atypical antipsychotic in the benzoisothiazoles class of chemicals. Like most second-generation antipsychotics it is a full antagonist at dopamine D(2) and serotonin 5-HT(2A) receptors, and is a partial agonist at 5-HT(1A) receptors, a property shared by some but not all older agents. It has much greater affinity for 5-HT(7) subtype receptors than other atypical antipsychotics. Pharmacokinetic studies showed that lurasidone is reasonably rapidly absorbed, with bioavailability appearing to be increased by food. Lurasidone undergoes extensive metabolism to a number of metabolites, some of which retain pharmacological activities. Metabolism is mainly by CYP3A4, resulting in steady-state concentrations... (truncated)

Title: Drug-induced liver injury: results from the hospital-based Berlin Case-Control Surveillance Study.
Drug-induced liver injury (DILI) is often responsible for acute liver failure, drug withdrawal, boxed warnings or drug non-approval. Therefore, we conducted a case-control study to determine the hepatotoxic risk of a wide range of drugs.

Title: Management of patients with risk factors.
This review addresses concomitant diseases and risk factors in patients treated for diseases of the ears, nose and throat in outpatient and hospital services. Besides heart disease, lung disease, liver disease and kidney disease, this article also covers disorders of coagulation (including therapy with new oral anticoagulants) and electrolyte imbalance. Special attention is paid to the prophylaxis, diagnosis and treatment of perioperative delirium. It is also intended to help optimise the preparation for surgical procedures and pharmacotherapy during the hospital stay.

MESH:D001726 - bisacodyl

Summary:

LLM Prediction Score	0.000 (normalized)
LLM Confidence Score	0.980
Golden Answer (Severity Class)	0.0 (normalized)
Prediction Error	0.000

Retrieved Context:

Title: Efficacy and Patient Tolerability Profiles of Probiotic Solution with Bisacodyl Versus Conventional Cleansing Solution for Bowel Preparation: A Prospective, Randomized, Controlled Trial.
Although adequate bowel preparation is essential in screening colonoscopy, patient intolerability to bowel cleansing agents is problematic. Recently, a probiotic mixture solution with bisacodyl emerged to improve patient tolerability. We investigated the efficacy, safety, and patient tolerability profiles of probiotics with bisacodyl versus conventional polyethylene glycol (PEG) solution for bowel preparation for screening colonoscopies in healthy patients in this prospective, randomized, case-control study. In total, 385 volunteers were randomly assigned to receive 2 L of water + 200 mL of probiotic solution (case group, <i>n</i> = 195) or 4 L of PEG solution (control group, <i>n</i> = 190). The efficacy... (truncated)

Title: A Randomized, Endoscopist-Blinded, Prospective Trial to Compare the Efficacy and Patient Tolerability between Bowel Preparation Protocols Using Sodium Picosulfate Magnesium Citrate and Polyethylene-Glycol (1 L and 2 L) for Colonoscopy.
Patient compliance during bowel preparation is important for successful colonoscopy. Bowel preparation with polyethylene glycol (PEG), the most commonly used solution for cleansing, involves the unpleasant ingestion of a large amount of liquid. Sodium picosulfate magnesium citrate (SP-MC) solution is an alternative option with better palatability than PEG. Therefore, in this study, we compared the efficacy and patient tolerability among the following three bowel preparation protocols: 2 L PEG-ascorbic acid (ASc), 1 L PEG-ASc plus bisacodyl, and SP-MC 340 mL plus bisacodyl. We conducted a randomized prospective endoscopist-blinded study between August 2018 and January 2019. A total of 311 patients were randomly classified... (truncated)

Title: Preventive effect of resistant starch on activated carbon-induced constipation in mice.
The aim of this study was to investigate the effects of resistant starch (RS) on activated carbon-induced constipation in ICR mice. ICR mice were fed on diet containing 15% RS of type RS2, RS3 or RS4 for 9 days. Gastrointestinal transit, defecation time and intestinal tissue histopathological sections, as well as motilin (MTL), gastrin (Gas), endothelin (ET), somatostatin (SS), acetylcholinesterase (AChE), substance P (SP) and vasoactive intestinal peptide (VIP) levels in serum were used to evaluate the preventive effects of RS on constipation. Bisacodyl, a laxative drug, was used as a positive control. The time to the first black stool... (truncated)

Title: Gastrointestinal diseases during pregnancy: what does the gastroenterologist need to know?
Pregnancy is characterized by numerous physiological changes that may lead to a diversity of symptoms and frequently to gastrointestinal complaints, such as heartburn, nausea and vomiting, or constipation. Chronic gastrointestinal diseases require treatment maintenance during this period, raising the challenging question whether outcomes beneficial to the mother may be harmful for the fetus. In addition, certain diseases, such as acute fatty liver of pregnancy, only develop during pregnancy and may require urgent procedures, such as fetus delivery. Even though they are not present in our day-to-day practice, knowledge of pregnancy-related diseases is fundamental and collaboration between gastroenterologists and obstetricians is... (truncated)

Title: Paracetamol in therapeutic dosages and acute liver injury: causality assessment in a prospective case series.
Acute liver injury (ALI) induced by paracetamol overdose is a well known cause of emergency hospital admission and death. However, there is debate regarding the risk of ALI after therapeutic dosages of the drug.The aim is to describe the characteristics of patients admitted to hospital with jaundice who had previous exposure to therapeutic doses of paracetamol. An assessment of the causality role of paracetamol was performed in each case.

MESH:C018904 - dexchlorpheniramine

Summary:

LLM Prediction Score	0.000 (normalized)
LLM Confidence Score	0.930
Golden Answer (Severity Class)	0.0 (normalized)
Prediction Error	0.000

Retrieved Context:

Title: Focus on Over-the-Counter Drugs' Misuse: A Systematic Review on Antihistamines, Cough Medicines, and Decongestants.
Background: Over the past 20 years or so, the drug misuse scenario has seen the emergence of both prescription-only and over-the-counter (OTC) medications being reported as ingested for recreational purposes. OTC drugs such as antihistamines, cough/cold medications, and decongestants are reportedly the most popular in being diverted and misused. Objective: While the current related knowledge is limited, the aim here was to examine the published clinical data on OTC misuse, focusing on antihistamines (e.g., diphenhydramine, promethazine, chlorpheniramine, and dimenhydrinate), dextromethorphan (DXM)- and codeine-based cough medicines, and the nasal decongestant pseudoephedrine. Methods: A systematic literature review was carried out with the... (truncated)

Title: Retrospective Study of Reported Adverse Events Due to Complementary Health Products in Singapore From 2010 to 2016.
The objective of this study is to collate and analyse adverse event reports associated with the use of complementary health products (CHP) submitted to the Health Sciences Authority (HSA) of Singapore for the period 2010-2016 to identify various trends and signals for pharmacovigilance purposes. A total of 147,215 adverse event reports suspected to be associated with pharmaceutical products and CHP were received by HSA between 2010 and 2016. Of these, 143,191 (97.3%) were associated with chemical drugs, 1,807 (1.2%) with vaccines, 1,324 (0.9%) with biological drugs (biologics), and 893 (0.6%) with CHP. The number of adverse event reports associated with... (truncated)

Title: Paracetamol in therapeutic dosages and acute liver injury: causality assessment in a prospective case series.
Acute liver injury (ALI) induced by paracetamol overdose is a well known cause of emergency hospital admission and death. However, there is debate regarding the risk of ALI after therapeutic dosages of the drug.The aim is to describe the characteristics of patients admitted to hospital with jaundice who had previous exposure to therapeutic doses of paracetamol. An assessment of the causality role of paracetamol was performed in each case.

Title: Severe and prolonged cholestasis caused by trimethoprim-sulfamethoxazole: a case report.
No abstract available.

Title: Assessment of liver stiffness in patients with HCV and mixed cryoglobulinemia undergoing rituximab treatment.
Mixed cryoglobulinemia (MC) is a HCV-related lymphoproliferative disorder generally associated with advanced liver disease. Liver stiffness has been significantly correlated with histopathological stage of fibrosis. Moreover, it was influenced by necroinflammatory activity. Rituximab (RTX) is a chimeric anti-CD20 monoclonal antibody inducing transient B lymphocytes depletion that was shown to be useful and safe in the majority of HCV MC patients, leading also to improvement of cirrhotic syndrome. Aim of this study was to evaluate the modifications of liver stiffness following RTX treatment in HCV-related MC patients.

MESH:D004491 - edrophonium

Summary:

LLM Prediction Score	0.000 (normalized)
LLM Confidence Score	0.940
Golden Answer (Severity Class)	0.0 (normalized)
Prediction Error	0.000

Retrieved Context:

Title: Myasthenia gravis.
Myasthenia gravis (MG) is a rare, autoimmune neuromuscular junction disorder. Contemporary prevalence rates approach 1/5,000. MG presents with painless, fluctuating, fatigable weakness involving specific muscle groups. Ocular weakness with asymmetric ptosis and binocular diplopia is the most typical initial presentation, while early or isolated oropharyngeal or limb weakness is less common. The course is variable, and most patients with initial ocular weakness develop bulbar or limb weakness within three years of initial symptom onset. MG results from antibody-mediated, T cell-dependent immunologic attack on the endplate region of the postsynaptic membrane. In patients with fatigable muscle weakness, the diagnosis of MG... (truncated)

Title: D-Penicillamine-Induced Myasthenia Gravis-A Probable Complication of Wilson's Disease Treatment-A Case Report and Systematic Review of the Literature.
Wilson's disease (WD) is a genetic disorder with copper accumulation in various tissues leading to related clinical symptoms (mainly hepatic and neuropsychiatric) which can be in 85% of patients successfully treated with anti-copper agents. However, during WD treatment neurological deterioration may occur in several patients. D-penicillamine (DPA) is one of the most frequently used drugs in WD treatment. Despite its efficacy, DPA can produce many adverse drug reactions, which should be recognized early. We present the case of a 51-year-old man diagnosed with the hepatic form of WD and initially treated with DPA in whom after 15 months of treatment,... (truncated)

Title: Computational Models Using Multiple Machine Learning Algorithms for Predicting Drug Hepatotoxicity with the DILIrank Dataset.
Drug-induced liver injury (DILI) remains one of the challenges in the safety profile of both authorized and candidate drugs, and predicting hepatotoxicity from the chemical structure of a substance remains a task worth pursuing. Such an approach is coherent with the current tendency for replacing non-clinical tests with in vitro or in silico alternatives. In 2016, a group of researchers from the FDA published an improved annotated list of drugs with respect to their DILI risk, constituting "the largest reference drug list ranked by the risk for developing drug-induced liver injury in humans" (DILIrank). This paper is one of the... (truncated)

Title: Dynamic imaging of adaptive stress response pathway activation for prediction of drug induced liver injury.
Drug-induced liver injury remains a concern during drug treatment and development. There is an urgent need for improved mechanistic understanding and prediction of DILI liabilities using in vitro approaches. We have established and characterized a panel of liver cell models containing mechanism-based fluorescent protein toxicity pathway reporters to quantitatively assess the dynamics of cellular stress response pathway activation at the single cell level using automated live cell imaging. We have systematically evaluated the application of four key adaptive stress pathway reporters for the prediction of DILI liability: SRXN1-GFP (oxidative stress), CHOP-GFP (ER stress/UPR response), p21 (p53-mediated DNA damage-related response) and... (truncated)

Title: Role of Pharmacogenetics in Adverse Drug Reactions: An Update towards Personalized Medicine.
Adverse drug reactions (ADRs) are an important and frequent cause of morbidity and mortality. ADR can be related to a variety of drugs, including anticonvulsants, anaesthetics, antibiotics, antiretroviral, anticancer, and antiarrhythmics, and can involve every organ or apparatus. The causes of ADRs are still poorly understood due to their clinical heterogeneity and complexity. In this scenario, genetic predisposition toward ADRs is an emerging issue, not only in anticancer chemotherapy, but also in many other fields of medicine, including hemolytic anemia due to glucose-6-phosphate dehydrogenase (G6PD) deficiency, aplastic anemia, porphyria, malignant hyperthermia, epidermal tissue necrosis (Lyell's Syndrome and Stevens-Johnson Syndrome), epilepsy,... (truncated)

MESH:D008874 - midazolam

Summary:

LLM Prediction Score	0.000 (normalized)
LLM Confidence Score	0.990
Golden Answer (Severity Class)	0.0 (normalized)
Prediction Error	0.000

Retrieved Context:

Title: Effect of Adding Midazolam to Dual Prophylaxis for Preventing Postoperative Nausea and Vomiting.
Multimodal prophylaxis for postoperative nausea and vomiting (PONV) has been recommended, even in low-risk patients. Midazolam is known to have antiemetic properties. We researched the effects of adding midazolam to the dual prophylaxis of ondansetron and dexamethasone on PONV after gynecologic laparoscopy. In this prospective, randomized, double-blinded trial, 144 patients undergoing gynecological laparoscopic surgery under sevoflurane anesthesia were randomized to receive either normal saline (control group, <i>n</i> = 72) or midazolam 0.05 mg/kg (midazolam group, <i>n</i> = 72) intravenously at pre-induction. All patients were administered dexamethasone 4 mg at induction and ondansetron 4 mg at the completion of the laparoscopy,... (truncated)

Title: Causality Evaluation of Drug-Induced Liver Injury in Newborns and Children in the Intensive Care Unit Using the Updated Roussel Uclaf Causality Assessment Method.
Purpose: Drug-induced liver injury (DILI) is a common adverse reaction in the clinic; however, there are relatively few reports of DILI in critically ill newborns and children. Making use of the Pediatric Intensive Care database (PIC), this study identifies which drugs are related to DILI in neonates and children in China. Methods: Using the PIC, we screened for patients whose liver was suspected of being injured by drugs during hospitalization. The medicine they used was then assessed by the Roussel Uclaf Causality Assessment Method (RUCAM). At the same time, we also collated drug combinations that may affect CYP (Cytochrome P)... (truncated)

Title: Midazolam Ameliorates Acute Liver Injury Induced by Carbon Tetrachloride <i>via</i> Enhancing Nrf2 Signaling Pathway.
Oxidative stress contributes greatly to initiation and progression of liver injury. Activation of nuclear-factor erythroid 2-related factor 2 (Nrf2) has been considered as an attractive strategy for preventing and treating the oxidative damage related to liver injury. This study aimed to find an efficacious agent to activate Nrf2/HO-1 signaling pathway from clinically used therapeutic agents and to characterize the usefulness for preventing and treating CCl₄-induced acute liver injury. For this purpose, a series of clinically used therapeutic agents were collected and their activation potentials on Nrf2 were assayed by using 293T-Nrf2-luc cell line. Among all tested therapeutic agents, midazolam was... (truncated)

Title: Anesthesiological management during isolated liver perfusion.
The treatment of irresectable hepatic metastases is limited by the systemic toxicity of anticancer agents. Isolated hyperthermic liver perfusion (IHLP) with anticancer agents is a new therapy for irresectable liver tumors. The risks of this therapy lie in the extended operation, the anhepatic phase and the possibility of liver damage due to the anticancer drug and hyperthermia. Experience of this method is rare, and the side effects are not well known. To estimate the individual risk of patients before isolated liver perfusion an extended evaluation of the preoperative conditions is usual. Titration of all anesthetic agents is advisable to prevent... (truncated)

Title: The Protection of Midazolam Against Immune Mediated Liver Injury Induced by Lipopolysaccharide and Galactosamine in Mice.
Objectives: Liver macrophages agitated by Lipopolysaccharide (LPS) can enhance immuno-inflammatory responses in the liver which mediate liver injury and result in dysfunction. Midazolam has been reported to have inhibitory effects on activated immunity and escalated inflammation, however, what the effects of midazolam on the liver injury caused by excessive immuno-inflammatory response in sepsis, and what influence it will exert on inflamed liver macrophages need to be elucidated. Methods: In the present study, LPS and galactosamine-induced acute liver injury mice were used to observe the effect of midazolam <i>in vivo</i>. LPS-stimulated bone marrow cells were used to evaluate the influence of... (truncated)

MESH:D010121 - oxytocin

Summary:

LLM Prediction Score	0.000 (normalized)
LLM Confidence Score	0.990
Golden Answer (Severity Class)	0.0 (normalized)
Prediction Error	0.000

Retrieved Context:

Title: Ameliorative Effect of Oxytocin on <i>FBN1</i> and <i>PEPCK</i> Gene Expression, and Behavioral Patterns in Rats' Obesity-Induced Diabetes.
Amelioration of hyperinsulinemia and insulin resistance associated with obesity is a cardinal target for therapeutics. Therefore, we investigated the relation of <i>Fibrilln-1</i> (<i>FBN1) mRNA</i> expression and hepatic <i>phosphoenolpyruvate caboxykinase</i> (<i>PEPCK</i>) enzyme to the ameliorative impact of oxytocin on obesity-induced diabetes, suggesting glycogenolysis markers in diabetic models. Four groups of forty male Wistar rats were formed (<i>n</i> = 10): a control group fed basal diet and <i>intra</i>peritoneal injections of saline; an oxytocin-injected group; a diet-induced obese group fed a high-fat/high-sugar diet and injected with saline; a diet-induced obese group injected with oxytocin. Depending on blood glucose levels, obese groups were further... (truncated)

Title: Oxytocin ameliorates remote liver injury induced by renal ischemia-reperfusion in rats.
Renal ischemia-reperfusion (IR) causes remote liver damage. Oxytocin has anti-inflammatory and antioxidant effects. The main purpose of this study was to evaluate the protective function of oxytocin (OT) in remote liver damage triggered by renal IR in rats. Twenty four rats were randomly divided into four different groups, each containing 8 rats. The groups were as follows: (1) Sham operated group; (2) Sham operated+OT group (3) Renal IR group; (4) Renal IR+OT group. OT (500µg/kg) was administered subcutaneously 12 and 24 hours before and immediately after ischemia. At the end of experimental procedure, the rats were sacrificed, and liver specimens... (truncated)

Title: Oxytocin and oxygen: the evolution of a solution to the 'stress of life'.
Oxytocin (OT) and the OT receptor occupy essential roles in our current understanding of mammalian evolution, survival, sociality and reproduction. This narrative review examines the hypothesis that many functions attributed to OT can be traced back to conditions on early Earth, including challenges associated with managing life in the presence of oxygen and other basic elements, including sulfur. OT regulates oxidative stress and inflammation especially through effects on the mitochondria. A related nonapeptide, vasopressin, as well as molecules in the hypothalamic-pituitary-adrenal axis, including the corticotropin-releasing hormone family of molecules, have a broad set of functions that interact with OT. Interactions... (truncated)

Title: Sex Differences in the Regulation of Vasopressin and Oxytocin Secretion in Bile Duct-Ligated Rats.
Hyponatremia due to elevated arginine vasopressin (AVP) secretion increases mortality in liver failure patients. No previous studies have addressed sex differences in hyponatremia in liver failure animal models.

Title: Oxytocin promotes hepatic regeneration in elderly mice.
Liver aging impairs the ability of hepatocyte regeneration. Recent studies have found that oxytocin (OT) plays an important role in promoting tissue repair and maintaining differentiation and regeneration of stem cells. Here, we reported that OT receptors, which are specifically located in hepatocytes, decrease with aging in human and mice. Interestingly, the level of serum OT also decline with age. Notably, OT promotes hepatocyte regeneration only in aged mice but not in young mice <i>in vitro</i> and <i>in vivo</i>. Further studies reveal that OT promotes autophagy in either AML12 mouse hepatocytes or aged mice after partial hepatectomy or with CCl4-induced acute liver... (truncated)

MESH:C105308 - oprelvekin

Summary:

LLM Prediction Score	0.000 (normalized)
LLM Confidence Score	0.920
Golden Answer (Severity Class)	0.0 (normalized)
Prediction Error	0.000

Retrieved Context:

Title: Protective effect of rhIL-11 in a murine model of acetaminophen-induced hepatotoxicity.
Acetaminophen intoxication results in hepatotoxicity associated with increased serum concentrations of hepatocellular leakage enzymes such as aspartate aminotransferase, lactate dehydrogenase, and alanine aminotransferase, centrilobular degeneration and necrosis, and activation of Kupffer cells. Recombinant human Interleukin-11 (rhIL-11) downregulates the production of proinflammatory mediators from activated macrophages and has direct effects on hepatocyte gene expression. Based on these biological activities of rhIL-11, the effect of pretreatment with rhIL-11 in a murine model of acetaminophen-induced hepatotoxicity was examined. Administration of 500 microg/kg acetaminophen to B6C3F1 mice resulted in progressive hepatotoxicity as demonstrated by elevated serum concentrations of hepatocellular leakage enzymes and TNFalpha and... (truncated)

MESH:D000583 - amikacin

Summary:

LLM Prediction Score	0.000 (normalized)
LLM Confidence Score	0.990
Golden Answer (Severity Class)	0.0 (normalized)
Prediction Error	0.000

Retrieved Context:

Title: [Comparative study of cefpiramide + amikacin versus piperacillin + amikacin in granulocytopenic patients: a randomized, prospective study].
Cefpiramide + amikacin (CPM + AMK) was compared in a prospective randomized trial with our standard regimen of piperacillin plus amikacin (PIPC + AMK) as an empiric therapy for fever in 252 episodes of 141 patients with granulocytopenia. Initial profound granulocytopenia (fewer than 100/mm3 mature granulocytes) was present in approximately 69% of the patient trials in both treatment groups. Both groups were equally distributed in regard to age, disease, pretreatment WBC count and duration of antibiotic treatment, although septic shock was more frequently seen in CPM + AMK. Responses to PIPC + AMK and CPM + AMK were similar for... (truncated)

Title: Cefoperazone versus cefotaxime, plus amikacin or sisomicin, in fever and infection in hematologic granulocytopenic patients.
Forty patients with leukemia or aplastic anemia were randomized to receive one of the following antibiotic regimens at the onset of fever during granulocytopenia: cefoperazone + amikacin (regimen A), cefoperazone + sisomicin (regimen B), cefotaxime + amikacin (regimen C), cefotaxime + sisomicin (regimen D). All patients were receiving gut decontamination at the time of randomization. Patients were monitored twice weekly with swabs and cultures for bacteria and fungi. Overall, there were 56 febrile episodes: 31 were proven bacterial, 3 were probable, and 16 were of unknown origin. Response rates were comparable in all 4 treatment regimens: 90%, 91%, 92% and... (truncated)

Title: Clinical experience with tobramycin in the treatment of infections due to gram-negative bacilli.
Tobramycin, a new aminocyclitol antimicrobial agent, was administered parenterally to 28 patients infected with gram-negative bacilli. The dosage was 1.5 mg/kg every 8 hours, except in patients with renal insufficiency in whom the dosage interval was 10 times the serum creatinine in hours. Serum levels after intramuscular administration were 3.9 +/- 1.6 mug/ml and 1.0 +/- 0.7 mug/ml at 2 and 8 hours, respectively. Serum levels after intravenous administration were 4.6 +/- 0.5 mug/ml, 2.3 +/- 0.6 mug/ml, and 0.6 +/- 0.8 mug/ml at 15 minutes, 1 hour, and 8 hours, respectively. Clinical isolates included 21 of Pseudomonas aeruginosa, 6... (truncated)

Title: A Critical Review of the Pharmacokinetics, Pharmacodynamics, and Safety Data of Antibiotics in Avian Species.
In avian medicine, the use of antibiotic dosing regimens based on species-specific pharmacological studies is ideal. However, due to a lack of such studies, dose extrapolation, which may cause inefficacy and toxicity, is common practice. Multiple searches were performed using the PubMed and Web of Science databases to extract relevant pharmacological studies performed in exotic avian species. The pharmacokinetics (PK), pharmacodynamics (PD), and safety data of the selected antibiotics (enrofloxacin, marbofloxacin, gentamicin, amikacin, ceftiofur, doxycycline, and amoxicillin/clavulanate) from these studies were reviewed. This review aimed to identify trends amenable for safe inter-species dose extrapolation and provide updated findings on dosing... (truncated)

Title: Pirprofen-induced fulminant hepatitis.
We report the cases of 2 female patients aged 69 and 61 yr, suffering from fulminant hepatitis induced by pirprofen, a new nonsteroidal antiinflammatory drug. The duration of pirprofen administration before the onset of hepatitis was long, 7 and 9 mo, respectively. Hepatitis was not preceded or accompanied by hypersensitivity manifestations. The liver lesion consisted of massive, predominantly centrilobular hepatic cell necrosis and microvesicular steatosis. One patient died of liver failure. Although the risk of fulminant hepatitis is very low, we recommend that, in patients taking pirprofen for more than 2 mo and complaining of asthenia, nausea, or vomiting, serum... (truncated)

MESH:C090840 - alosetron

Summary:

LLM Prediction Score	0.500 (normalized)
LLM Confidence Score	0.980
Golden Answer (Severity Class)	0.5 (normalized)
Prediction Error	0.000

Retrieved Context:

Title: Acute hepatitis associated with alosetron (Lotronex).
Alosetron hydrochloride (Lotronex, GlaxoSmithKline, Inc) is a safe and effective agent for selective patients with severe irritable bowel syndrome when prescribed as recommended. We describe the first reported case of acute liver injury in a 39-year-old white woman who developed symptomatic hepatitis 28 days after starting alosetron. All other competing causes of acute hepatitis were excluded by radiologic and laboratory studies and the liver injury resolved after drug discontinuation. Although the mechanism of alosetron hepatotoxicity is unknown, metabolic idiosyncrasy is suspected since the drug is known to be extensively metabolized by cytochrome-P450 enzymes. Clinicians prescribing alosetron should be aware of... (truncated)

Title: The safety of drugs used in acid-related disorders and functional gastrointestinal disorders.
Medicines are frequently used in the management of acid-related disorders and functional gastrointestinal disorders. With the exception of complicated peptic ulcer disease, these disorders are not associated with appreciable mortality. Drug treatments have consequently been held to the highest standards of safety. Some medicines have been withdrawn or restricted based on assessments and perceptions of risk. However, the risk of serious toxicity is low for most of the agents discussed in this article. Assessments are made of the safety and adverse-event profiles of certain drug classes and, where appropriate, individual medicines. For conditions with a low risk of mortality or... (truncated)

Title: Association of CYP1A1 and CYP1B1 inhibition in in vitro assays with drug-induced liver injury.
Drug-induced liver injury (DILI) is one of the major causes for the discontinuation of drug development and withdrawal of drugs from the market. Since it is known that reactive metabolite formation and being substrates or inhibitors of cytochrome P450s (P450s) are associated with DILI, we systematically investigated the association between human P450 inhibition and DILI. The inhibitory activity of 266 DILI-positive drugs (DILI drugs) and 92 DILI-negative drugs (no-DILI drugs), which were selected from Liver Toxicity Knowledge Base (US Food and Drug Administration), against 8 human P450 forms was assessed using recombinant enzymes and luminescent substrates, and the threshold values... (truncated)

Title: Effect of Hepatic Impairment on Eluxadoline Pharmacokinetics.
No abstract available.

Title: Effect of total intravenous anaesthesia and prophylactic ramosetron on postoperative nausea and vomiting after thyroidectomy: A prospective, randomized controlled study.
To investigate the effect of combined prophylactic ramosetron and total intravenous anaesthesia (TIVA) on postoperative nausea and vomiting (PONV), compared with sevoflurane anaesthesia without prophylactic antiemetics, in female patients undergoing thyroidectomy.

MESH:D000077465 - cabergoline

Summary:

LLM Prediction Score	0.000 (normalized)
LLM Confidence Score	0.990
Golden Answer (Severity Class)	0.0 (normalized)
Prediction Error	0.000

Retrieved Context:

Title: Current approaches to the pharmacological management of Cushing's disease.
If treatment of Cushing's disease (CD) by surgery is not successful, medical therapy is often required. Long-term use of metyrapone is limited by hirsutism and hypertension and escape because of increased ACTH levels. Although ketoconazole can normalize cortisol levels in 50%, liver toxicity limits its use. Mitotane, an adrenolytic agent, has had minimal use for benign disease. Etomidate is useful when rapid reduction in cortisol levels is needed. Cabergoline can normalize cortisol levels in CD in about one-third of patients and is well tolerated. Pasireotide can normalize cortisol levels in CD in about 25% but causes worsening of glucose tolerance... (truncated)

Title: Successful Management of Cushing Syndrome From Ectopic ACTH Secretion in an Adolescent With Osilodrostat.
A previously healthy 11-year-old male was found to have a mass in the pancreatic head after several months of abdominal pain and jaundice. Pathology was consistent with a World Health Organization grade 2 pancreatic neuroendocrine tumor. He developed refractory hypertension and was found to have Cushing syndrome from ectopic ACTH secretion, with oligometastatic liver disease. He underwent surgical resection of the pancreatic tumor and metastases. Postoperatively, his Cushing syndrome resolved, but it reemerged 1 year later in the setting of disease recurrence. He was not a candidate for bilateral adrenalectomy. Ketoconazole therapy was inadequate and he was started on metyrapone,... (truncated)

Title: Pegvisomant-induced cholestatic hepatitis with jaundice in a patient with Gilbert's syndrome.
We report on a patient with active acromegaly and Gilbert's syndrome who developed severe hepatic dysfunction during pegvisomant (PEGv) monotherapy. She was partially resistant to all previous therapies, including long-acting somatostatin analogs and cabergoline. Five months after starting PEGv therapy, with an already normalized IGF1, she developed cholestatic liver dysfunction with jaundice. Liver or biliary diseases including biliary sludge, cholelithiasis or liver steatosis were excluded. A liver biopsy was in keeping with drug-induced liver injury. The discontinuation of PEGv was followed by full clinical and biochemical recovery in 6 weeks. PEGv therapy was not resumed. Apart from a minimal increase... (truncated)

Title: Medical management of functioning pituitary adenoma: an update.
The treatment of functioning pituitary adenoma (FPA) must achieve endocrinological remission as well as tumor size reduction. The first-line treatment of FPA except prolactinoma is transsphenoidal surgery (TSS). Medical treatments and/or radiation will be applied as adjuvant therapies succeeding to TSS. In patients with prolactinoma, dopamine agonists, especially cabergoline, are quite efficient. Dopamine agonists decrease plasma prolactin levels and induce shrinkage in most patients and can be ceased in some of them. In patients with acromegaly, dopamine agonists, somatostatin analogues, and growth hormone receptor antagonist have been used as a monotherapy or the combination, and the high remission rate can... (truncated)

Title: The Role of Isotretinoin Therapy for Cushing's Disease: Results of a Prospective Study.
Objective. This prospective open trial aimed to evaluate the efficacy and safety of isotretinoin (13-cis-retinoic acid) in patients with Cushing's disease (CD). Methods. Sixteen patients with CD and persistent or recurrent hypercortisolism after transsphenoidal surgery were given isotretinoin orally for 6-12 months. The drug was started on 20 mg daily and the dosage was increased up to 80 mg daily if needed and tolerated. Clinical, biochemical, and hormonal parameters were evaluated at baseline and monthly for 6-12 months. Results. Of the 16 subjects, 4% (25%) persisted with normal urinary free cortisol (UFC) levels at the end of the study. UFC... (truncated)

MESH:D002506 - cephalexin

Summary:

LLM Prediction Score	0.500 (normalized)
LLM Confidence Score	0.990
Golden Answer (Severity Class)	0.5 (normalized)
Prediction Error	0.000

Retrieved Context:

Title: Cephalexin induced cholestatic jaundice.
Cephalexin is a very commonly prescribed orally administered antibiotic which has many potential side effects. Amongst these cholestatic jaundice has been infrequently reported as an adverse reaction. We present a case of a 57-year-old male who exhibited features of cholestatic jaundice including elevated liver function tests (LFTs) after taking cephalexin and showed improvement on removal of the offending agent. During this time he was symptomatically treated with cholestyramine. Complete resolution of LFTs was seen in four weeks. Cephalexin induced cholestasis is rare and hence requires a high degree of clinical suspicion for prompt diagnosis and treatment.

Title: Uncommon Cause of Acute Drug-induced Liver Injury Following Mammoplasty.
Cephalexin is a well tolerated antimicrobial and hepatic injury is an infrequent occurrence with its use. We here describe a 21-year-old female who presented with jaundice and elevated liver enzymes after 4 weeks completion of 10 day course of cephalexin, prescribed prophylactically after mammoplasty. Extensive work up including all causes of hepatitis was within normal limits and she improved with conservative management. This case highlights the need to suspect drug induced liver injury in cases of jaundice and cephalexin use.

Title: A large population-based follow-up study of trimethoprim-sulfamethoxazole, trimethoprim, and cephalexin for uncommon serious drug toxicity.
We conducted a population-based 45-day follow-up study of 232,390 people who were prescribed trimethoprim-sulfamethoxazole (TMP-SMZ), 266,951 prescribed trimethoprim alone, and 196,397 prescribed cephalexin, to estimate the risk of serious liver, blood, skin, and renal disorders resulting in referral or hospitalization associated with these drugs. The results were based on information recorded on office computers by selected general practitioners in the United Kingdom, together with a review of clinical records. The risk of clinically important idiopathic liver disease was similar for persons prescribed TMP-SMZ (5.2/100,000) and those prescribed trimethoprim alone (3.8/100,000). The risk for those prescribed cephalexin was somewhat lower (2.0/100,000).... (truncated)

Title: Drug-Induced Liver Injury with Commonly Used Antibiotics in the All of Us Research Program.
Antibiotics are a known cause of idiosyncratic drug-induced liver injury (DILI). According to the Centers for Disease Control and Prevention, the five most commonly prescribed antibiotics in the United States are azithromycin, ciprofloxacin, cephalexin, amoxicillin, and amoxicillin-clavulanate. We quantified the frequency of acute DILI for these common antibiotics in the All of Us Research Program, one of the largest electronic health record (EHR)-linked research cohorts in the United States. Retrospective analyses were conducted applying a standardized phenotyping algorithm to de-identified clinical data available in the All of Us database for 318,598 study participants. Between February 1984 and December 2022, more... (truncated)

Title: Grayken lessons: between a rock and a hard place? A 37-year-old man with acute liver injury while enrolled in a managed alcohol program for severe alcohol use disorder.
Managed alcohol programs aim to reduce health and social harms associated with severe alcohol use disorder. Here, we describe a young man with severe alcohol use disorder enrolled in a managed alcohol program, who was admitted to hospital with acute liver injury. Fearing that alcohol was contributing, the inpatient care team discontinued the managed alcohol dose in hospital. He was ultimately diagnosed with cephalexin-induced liver injury. After consideration of risks, benefits, and alternative options, the patient and care team jointly decided to restart managed alcohol after hospital discharge. With this case, we describe managed alcohol programs and summarize the emerging... (truncated)

MESH:D002720 - chlormezanone

Summary:

LLM Prediction Score	1.000 (normalized)
LLM Confidence Score	0.850
Golden Answer (Severity Class)	1.0 (normalized)
Prediction Error	0.000

Retrieved Context:

Title: Drug-induced severe skin reactions. Incidence, management and prevention.
Severe skin adverse drug reactions can result in death, but the rate of such events is fortunately low. The incidences of Stevens-Johnson syndrome and toxic epidermal necrolysis range from 1.2 to 6 per million per year and 0.4 to 1.2 per million per year, respectively. Stevens-Johnson syndrome is fatal in about 5% and toxic epidermal necrolysis in 30% of cases. Drugs implicated in these diseases are the sulphonamides, anticonvulsants, allopurinol, pyrazolone derivatives, oxicams and chlormezanone. The principles of symptomatic treatment are the same as for burns, and patients with extensive skin detachment should be transferred to an intensive care unit... (truncated)

Title: [Drug-induced hepatitis with cholestasis following therapy with chlormezanone].
The history of a 46 years old female patient is reported who suffered from rheumatoid arthritis. During therapy with chlormezanon and paracetamol symptoms of cholestatic liver disease developed. We believe that this was a side-effect of chlormezanon.

Title: Severe hepatocellular damage induced by chlormezanone overdose.
Chlormezanone is a widely prescribed muscle relaxant (1-3) whose pharmacology is well known (4-6), but the clinical effect of overdose still remains relatively obscure (1, 7, 8). We here report a recent case of a patient who had severe liver function impairment and other associated findings typical of chlormezanone overdose. The ingested dose (at least 12 g) is much larger than those of previous record (1, 2, 7, 8); thus, her critical presentation deserves to be reviewed.

Title: [Undesired drug effects after taking chlormezanone (Muscle Trancopal) with lethal results].
A 34-year-old woman was admitted for treatment of toxic epidermolysis of the skin and mucosa. 16 days previously she had started to take chlormezanone (Muskel Trancopal) and some other medications for pain in the shoulder and neck. On admission she had a fever of 39 degrees C and, in addition to the epidermolysis, diffuse abdominal pain on pressure and blood-streaked stool.

Title: Chlormezanone-induced fulminant hepatitis in a pregnant woman: successful delivery and liver transplantation.
The case of a 29 year old woman affected by fulminant hepatitis during the third trimester of pregnancy, after a 3 week administration of chlormezanone is reported. Following induced Caesarean delivery, the patient underwent an orthotopic liver transplantation. The mother and her baby were in good condition 26 months after liver transplantation. In this case, chlormezanone was probably responsible for the fulminant hepatitis.

MESH:D000068238 - lubiprostone

Summary:

LLM Prediction Score	0.000 (normalized)
LLM Confidence Score	0.970
Golden Answer (Severity Class)	0.0 (normalized)
Prediction Error	0.000

Retrieved Context:

Title: Analysis of Nausea in Clinical Studies of Lubiprostone for the Treatment of Constipation Disorders.
Lubiprostone is a ClC-2 chloride channel activator approved for the treatment of chronic idiopathic constipation (CIC) and opioid-induced constipation (OIC) in adults and irritable bowel syndrome with constipation (IBS-C) in women. Lubiprostone is generally well tolerated, with nausea being the most common adverse event.

Title: Lubiprostone improves intestinal permeability in humans, a novel therapy for the leaky gut: A prospective randomized pilot study in healthy volunteers.
The barrier function of the small intestinal mucosa prevents the introduction of undesired pathogens into the body. Breakdown of this barrier function increases intestinal permeability. This has been proposed to induce not only gastrointestinal diseases, including inflammatory bowel disease and irritable bowel syndrome, but also various other diseases, including allergies, diabetes mellitus, liver diseases, and collagen diseases, which are associated with this so called "leaky gut syndrome." As such, a method to prevent leaky gut syndrome would have substantial clinical value. However, no drugs have been demonstrated to improve disturbed intestinal permeability in humans to date. Therefore, we investigated whether... (truncated)

Title: The Role of Leaky Gut in Nonalcoholic Fatty Liver Disease: A Novel Therapeutic Target.
The liver directly accepts blood from the gut and is, therefore, exposed to intestinal bacteria. Recent studies have demonstrated a relationship between gut bacteria and nonalcoholic fatty liver disease (NAFLD). Approximately 10-20% of NAFLD patients develop nonalcoholic steatohepatitis (NASH), and endotoxins produced by Gram-negative bacilli may be involved in NAFLD pathogenesis. NAFLD hyperendotoxicemia has intestinal and hepatic factors. The intestinal factors include impaired intestinal barrier function (leaky gut syndrome) and dysbiosis due to increased abundance of ethanol-producing bacteria, which can change endogenous alcohol concentrations. The hepatic factors include hyperleptinemia, which is associated with an excessive response to endotoxins, leading to... (truncated)

Title: Current developments in pharmacological therapeutics for chronic constipation.
Chronic constipation is a common gastrointestinal disease severely affecting the patient's quality of life. The traditional treatment of constipation is the use of laxatives. Recently, several new drugs including lubiprostone, linaclotide and prucalopride have been approved for treatment of chronic constipation. However, a significant unmet medical need still remains, particularly among those patients achieving poor results by current therapies. The 5-HT4 receptor modulators velusetrag and naronapride, the guanylate cyclase C agonist plecanatide and the ileal bile acid transporter inhibitor elobixibat are recognized as the most promising drugs under investigation. Herein, we give a comprehensive review on the pharmacological therapeutics for... (truncated)

Title: Lubiprostone stimulates duodenal bicarbonate secretion in rats.
Lubiprostone, a bicyclic fatty acid, is used for the treatment of chronic constipation. No published study has addressed the effect of lubiprostone on intestinal ion secretion in vivo.

MESH:D000069439 - dasatinib

Summary:

LLM Prediction Score	0.500 (normalized)
LLM Confidence Score	0.990
Golden Answer (Severity Class)	0.5 (normalized)
Prediction Error	0.000

Retrieved Context:

Title: Dasatinib-Induced CMV Hepatitis in an Immunocompetent Patient: A Rare Complication of a Common Drug.
Dasatinib is a common anticancer drug used in the treatment of leukemia. Several side effects have been reported, the most common being myelosuppression, diarrhea, edema, and nausea. Three papers have been published reporting hepatic side effects of dasatinib treatment. A rare side effect of dasatinib treatment is reactivation of latent cytomegalovirus (CMV) infection. Never before has dasatinib therapy shown to be the cause of CMV hepatitis in an immunocompetent patient. We present a case of an immunocompetent patient who was treated with the standard dose of dasatinib therapy and subsequently developed CMV hepatitis. Well-known side effects of dasatinib therapy are... (truncated)

Title: The Potential Protective Role of Naringenin against Dasatinib-Induced Hepatotoxicity.
Dasatinib (DASA) is a novel tyrosine kinase inhibitor, approved for leukemia treatment. However, the long-term use of DASA induces several complications, especially liver damage. On the other hand, Naringenin (NGN) is a potent antioxidant and anti-inflammatory agent which is known to exert protective effects in several liver disease animal models. Yet, the effect of NGN on DASA-induced hepatotoxicity has not been examined. This study investigated the hepatoprotective effects of NGN against DASA-induced acute liver injury, using a mouse model. The mice were given NGN (50, 100, and 200 mg/kg po) or saline for 7 days, followed by DASA on the... (truncated)

Title: Mechanisms of toxicity associated with six tyrosine kinase inhibitors in human hepatocyte cell lines.
Tyrosine kinase inhibitors have revolutionized the treatment of certain cancers. They are usually well tolerated, but can cause adverse reactions including liver injury. Currently, mechanisms of hepatotoxicity associated with tyrosine kinase inhibitors are only partially clarified. We therefore aimed at investigating the toxicity of regorafenib, sorafenib, ponatinib, crizotinib, dasatinib and pazopanib on HepG2 and partially on HepaRG cells. Regorafenib and sorafenib strongly inhibited oxidative metabolism (measured by the Seahorse-XF24 analyzer) and glycolysis, decreased the mitochondrial membrane potential and induced apoptosis and/or necrosis of HepG2 cells at concentrations similar to steady-state plasma concentrations in humans. In HepaRG cells, pretreatment with rifampicin... (truncated)

Title: Treatment with dasatinib for chronic myeloid leukemia following imatinib-induced hepatotoxicity.
Treatment with the tyrosine kinase inhibitor (TKI) imatinib for chronic myeloid leukemia in chronic phase (CML-CP) of disease may cause severe hepatotoxicity in rare cases. However, it remains unclear (1) how imatinib-induced hepatotoxicity should be treated; and (2) if and how TKI treatment can be resumed after recovery. We report a 32-year-old woman with CML-CP and histology confirmed imatinib-induced liver toxicity after 6 months of treatment. In this case, the early administration of corticosteroid therapy resulted in rapid and complete hepatic recovery, and treatment for CML-CP with the second generation TKI dasatinib was continued safely after recovery. Thus, dasatinib represents... (truncated)

Title: Autophagy protects against dasatinib-induced hepatotoxicity via p38 signaling.
Liver dysfunction is a common side effect associated with the treatment of dasatinib and its mechanism is poorly understood. Autophagy has been thought to be a potent survival or death factor for liver dysfunction, which may shed the light on a novel strategy for the intervention of hepatotoxicity caused by dasatinib. In this study, we show for the first time that autophagy is induced, which is consistent with the formation of liver damage. Autophagy inhibition exacerbated dasatinib-induced liver failure, suggesting that autophagy acted as a self-defense mechanism to promote survival. Oxidative stress has been shown to be an important stimulus... (truncated)

MESH:D004316 - doxepin

Summary:

LLM Prediction Score	0.500 (normalized)
LLM Confidence Score	0.990
Golden Answer (Severity Class)	0.5 (normalized)
Prediction Error	0.000

Retrieved Context:

Title: Neuropathy and fatal hepatitis in a patient receiving amiodarone.
Muscle weakness, neuropathy, and transient rises in hepatic enzyme activity have been reported with the use of the antiarrhythmic agent amiodarone. A 68 year old teetotaller with normal liver function was given amiodarone for resistant supraventricular arrhythmias. He presented 19 months later with vomiting, muscle weakness and wasting, sensory neuropathy, and hepatomegaly. Liver biopsy showed fibrosis and the presence of hyaline. The amiodarone was withdrawn. Three months later he developed ascites. Oesophageal varices were found and he later died. The liver showed micronodular cirrhosis. The large volume of distribution and long half life of amiodarone may explain the persistence of... (truncated)

Title: Doxepin Exacerbates Renal Damage, Glucose Intolerance, Nonalcoholic Fatty Liver Disease, and Urinary Chromium Loss in Obese Mice.
Doxepin is commonly prescribed for depression and anxiety treatment. Doxepin-related disruptions to metabolism and renal/hepatic adverse effects remain unclear; thus, the underlying mechanism of action warrants further research. Here, we investigated how doxepin affects lipid change, glucose homeostasis, chromium (Cr) distribution, renal impairment, liver damage, and fatty liver scores in C57BL6/J mice subjected to a high-fat diet and 5 mg/kg/day doxepin treatment for eight weeks. We noted that the treated mice had higher body, kidney, liver, retroperitoneal, and epididymal white adipose tissue weights; serum and liver triglyceride, alanine aminotransferase, aspartate aminotransferase, blood urea nitrogen, and creatinine levels; daily food efficiency;... (truncated)

Title: Drug-Induced Liver Injury during Antidepressant Treatment: Results of AMSP, a Drug Surveillance Program.
Drug-induced liver injury is a common cause of liver damage and the most frequent reason for withdrawal of a drug in the United States. The symptoms of drug-induced liver damage are extremely diverse, with some patients remaining asymptomatic.

Title: The Importance of Patient-Specific Factors for Hepatic Drug Response and Toxicity.
Responses to drugs and pharmacological treatments differ considerably between individuals. Importantly, only 50%-75% of patients have been shown to react adequately to pharmacological interventions, whereas the others experience either a lack of efficacy or suffer from adverse events. The liver is of central importance in the metabolism of most drugs. Because of this exposed status, hepatotoxicity is amongst the most common adverse drug reactions and hepatic liabilities are the most prevalent reason for the termination of development programs of novel drug candidates. In recent years, more and more factors were unveiled that shape hepatic drug responses and thus underlie the... (truncated)

Title: Addressing the Side Effects of Contemporary Antidepressant Drugs: A Comprehensive Review.
Randomized trials have shown that selective serotonin reuptake inhibitors (SSRIs) and serotonin-norepinephrine reuptake inhibitors (SNRIs) have better safety profiles than classical tricyclic antidepressants (TCAs). However, an increasing number of studies, including meta-analyses, naturalistic studies, and longer-term studies suggested that SSRIs and SNRIs are no less safe than TCAs. We focused on comparing the common side effects of TCAs with those of newer generation antidepressants including SSRIs, SNRIs, mirtazapine, and bupropion. The main purpose was to investigate safety profile differences among drug classes rather than the individual antidepressants, so studies containing comparison data on drug groups were prioritized. In terms of... (truncated)

MESH:C036604 - esmolol

Summary:

LLM Prediction Score	0.000 (normalized)
LLM Confidence Score	0.980
Golden Answer (Severity Class)	0.0 (normalized)
Prediction Error	0.000

Retrieved Context:

Title: Management of atrial fibrillation in critically ill patients.
Atrial fibrillation (AF) is common in ICU patients and is associated with a two- to fivefold increase in mortality. This paper provides a reappraisal of the management of AF with a special focus on critically ill patients with haemodynamic instability. AF can cause hypotension and heart failure with subsequent organ dysfunction. The underlying mechanisms are the loss of atrial contraction and the high ventricular rate. In unstable patients, sinus rhythm must be rapidly restored by synchronised electrical cardioversion (ECV). If pharmacological treatment is indicated, clinicians can choose between the rate control and the rhythm control strategy. The optimal substance should... (truncated)

Title: Acute rate control in atrial fibrillation: an urgent need for the clinician.
Rate and rhythm control are still considered equivalent strategies for symptom control using the Atrial Fibrillation Better Care algorithm recommended by the recent atrial fibrillation guideline. In acute situations or critically ill patients, a personalized approach should be used for rapid rhythm or rate control. Even though electrical cardioversion is generally indicated in haemodynamically unstable patients or for rapid effective rhythm control in critically ill patients, this is not always possible due to the high percentage of failure or relapses in such patients. Rate control remains the background therapy for all these patients, and often rapid rate control is mandatory.... (truncated)

Title: Pharmacokinetics of esmolol in hepatic disease.
Esmolol is an intravenous beta blocker with a short duration of action. The pharmacokinetics of esmolol and its acid metabolite, ASL-8123, were studied in nine patients who had stable, biopsy-proved Laennec's cirrhosis and in three normal volunteer controls. Kinetics were determined after a four-hour continuous infusion of esmolol at a rate of 200 micrograms/kg/min. Blood samples were collected during the infusions and at frequent intervals thereafter. The parameters studied were the steady state concentration, the total body clearance, the elimination half-life, the area under the curve, and the volume of distribution. No significant differences in any of these parameters were... (truncated)

Title: Comparison of effects of intraoperative esmolol and ketamine infusion on acute postoperative pain after remifentanil-based anesthesia in patients undergoing laparoscopic cholecystectomy.
Remifentanil is a short-acting drug with a rapid onset that is useful in general anesthesia. Recently, however, it has been suggested that the use of opioids during surgery may cause opioid-induced hyperalgesia (OIH). Researchers have recently reported that esmolol, an ultra-short-acing β1 receptor antagonist, reduces the postoperative requirement for morphine and provides more effective analgesia than the administration of remifentanil and ketamine. Hence, this study was conducted to determine whether esmolol reduces early postoperative pain in patients who are continuously infused with remifentanil for anesthesia during laparoscopic cholecystectomy.

Title: Effects of Amiodarone and Esmolol for Heart Rate and Cardiovascular Changes.
To probe into the effects of amiodarone and esmolol for heart rate disorders and myocardial infarction.

MESH:D007490 - iproniazid

Summary:

LLM Prediction Score	1.000 (normalized)
LLM Confidence Score	0.970
Golden Answer (Severity Class)	1.0 (normalized)
Prediction Error	0.000

Retrieved Context:

Title: Iproclozide fulminant hepatitis. Possible role of enzyme induction.
The authors report the cases of 3 patients who died from fulminant hepatitis after receiving iproclozide, a hydrazine-containing monoamine oxidase inhibitor. Fulminant hepatitis in these patients resembled that reported in patients receiving other hydrazine-containing monoamine oxidase inhibitors: (1) the 3 patients were women; (2) the monoamine oxidase inhibitor has been ingested for 1 month or more; (3) the main clinical manifestations were jaundice and disorders of consciousness; (4) hypersensitivity manifestations were absent; (5) the predominant liver lesion was necrosis; (6) all 3 patients died. In our 3 patients, jaundice occurred 7 to 10 days after the adjunction to iproclozide of... (truncated)

Title: [Auxiliary hepatic transplantation in iproniazid-induced subfulminant hepatitis. Should iproniazid still be sold in France?].
We report a new case of subfulminant hepatitis due to iproniazid, a MAO-inhibitor antidepressant, in a 27-year-old man. An auxiliary liver transplantation was performed. Liver function returned to normal and the patient was discharged from the hospital. However, the patient's native liver did not regenerate, and immunosuppressive therapy had to be maintained. Iproniazid hepatotoxicity is characterized by jaundice in 1% of cases, with a fulminant or subfulminant course in 20% of icteric patients. Although iproniazid is no longer sold in most countries, it is still commercialized in France. Because of the frequency and severity of hepatic injury, commercialization of iproniazid... (truncated)

Title: [Hepatotoxicity of isaxonine phosphate: 4 cases of severe subacute hepatitis].
Isaxonine phosphate is known to induce acute hepatitis which in most cases is reversible after withdrawal of the drug. The authors describe 4 new cases of hepatitis which differ from those previously reported by their evolutive and pathological features. In 3 cases, the outcome was fatal within a delay of 15-40 days despite discontinuation of the drug, and was associated with hepatic encephalopathy and ascites. In the 4 cases plasma concentration of alanine- and aspartate-aminotransferases were initially increased (up to 33 X upper normal range) and decreased to normal values in 2 cases. Plasma bilirubin levels were also elevated at... (truncated)

Title: Isoniazid and iproniazid: activation of metabolites to toxic intermediates in man and rat.
Acetylhydrazine, a metabolite of isoniazid, a widely used antituberculosis drug, and isopropylhydrazine, a metabolite of iproniazid, an antidepressant removed from clinical use because of high incidence of liver injury, were oxidized by cytochrome P-450 enzymes in human and rat liver microsomes to highly reactive acylating and alkylating agents. Covalent binding of these metabolites to liver macromolecules paralleled hepatic cellular necrosis. The metabolites formed from these and probably other monosubstituted hydrazines are reactive electrophiles.

Title: [Iproniazid-induced acute hepatitis].
Many drugs can cause an acute liver damage. The patient history is the guideline for diagnosis. Iproniazid, a monoamine-oxidase inhibitor not for sale in Italy, can frequently cause severe acute hepatitis. A case of acute iproniazid-induced, hepatitis in which the course was favourable, is reported.

MESH:D013392 - sucralfate

Summary:

LLM Prediction Score	0.000 (normalized)
LLM Confidence Score	0.980
Golden Answer (Severity Class)	0.0 (normalized)
Prediction Error	0.000

Retrieved Context:

Title: Hepatic effects of drugs used in the treatment of peptic ulcer disease.
Although one member of the H2 blocker family (oxmetidine) proved to be intrinsically hepatotoxic, overt hepatic injury attributable to cimetidine and ranitidine is decidedly rare, given the tens of millions of patients who have received these medications. Even considering the fact that serious adverse reactions may be underreported by as much as a factor of 100, the number of reports submitted to the FDA-DDE of possible hepatic toxicity associated with these agents supports this low incidence. Whether or not a similarly low incidence will be seen with famotidine and the newer H2 receptor antagonists still under investigation must await additional... (truncated)

Title: [The effect of cimetidine and gastrozepin on liver function and the pharmacotherapy of "hepatogenous" gastroduodenal ulcers and erosions].
Toxic hepatitis developed but in one out of 127 peptic ulcer patients treated with cimetidine. In patients (n-142) treated with gastrozepine, no cases of toxic hepatitis were recorded. These anti-ulcer agents did not influence absorptive capacities of the liver or hepatic blood flow. Meanwhile microsomal exidase (antitoxic) function of hepatocytes noticeably declined as a result of cimetidine treatment in every 8th patient with peptic ulcer subjected to the continuous 5-week treatment with the drug and in every 5th patient given the treatment (maintenance included) for a longer time. In patients suffering from liver cirrhosis with secondary gastroduodenal ulcers or multiple... (truncated)

Title: ACVIM consensus statement: Support for rational administration of gastrointestinal protectants to dogs and cats.
The gastrointestinal (GI) mucosal barrier is continuously exposed to noxious toxins, reactive oxygen species, microbes, and drugs, leading to the development of inflammatory, erosive, and ultimately ulcerative lesions. This report offers a consensus opinion on the rational administration of GI protectants to dogs and cats, with an emphasis on proton pump inhibitors (PPIs), histamine type-2 receptor antagonists (H₂ RAs), misoprostol, and sucralfate. These medications decrease gastric acidity or promote mucosal protective mechanisms, transforming the management of dyspepsia, peptic ulceration, and gastroesophageal reflux disease. In contrast to guidelines that have been established in people for the optimal treatment of gastroduodenal ulcers... (truncated)

Title: Effects of anti-ulcer agents on ethanol-induced gastric mucosal lesions in D-galactosamine-induced hepatitis rats.
Patients with hepatic injury have an increased incidence of gastric ulcers and erosions. In this study, the effect of D-galactosamine(GalN)-induced hepatitis on ethanol-induced gastric mucosal lesions and the protective effect of anti-ulcer agents in rats were examined. Subcutaneous injection of GalN (1 g/kg) remarkably increased serum alanine aminotransferase (ALT) and aspartate aminotransferase (AST) activities suggesting induction of hepatic injury. Gastric mucosal lesions induced by ethanol were significantly aggravated in GalN-induced hepatitis rats. Orally administered ecabet (CAS 86408-72-2; 20-200 mg/kg) dose dependently inhibited ethanol-induced gastric mucosal lesions in GalN-induced hepatitis rats. Sucralfate (CAS 54182-58-0) tended to inhibit the gastric mucosal lesions... (truncated)

Title: Gastrointestinal diseases during pregnancy: what does the gastroenterologist need to know?
Pregnancy is characterized by numerous physiological changes that may lead to a diversity of symptoms and frequently to gastrointestinal complaints, such as heartburn, nausea and vomiting, or constipation. Chronic gastrointestinal diseases require treatment maintenance during this period, raising the challenging question whether outcomes beneficial to the mother may be harmful for the fetus. In addition, certain diseases, such as acute fatty liver of pregnancy, only develop during pregnancy and may require urgent procedures, such as fetus delivery. Even though they are not present in our day-to-day practice, knowledge of pregnancy-related diseases is fundamental and collaboration between gastroenterologists and obstetricians is... (truncated)

MESH:D010394 - penbutolol

Summary:

LLM Prediction Score	0.000 (normalized)
LLM Confidence Score	0.880
Golden Answer (Severity Class)	0.0 (normalized)
Prediction Error	0.000

Retrieved Context:

Title: Utility of spherical human liver microtissues for prediction of clinical drug-induced liver injury.
Drug-induced liver injury (DILI) continues to be a major source of clinical attrition, precautionary warnings, and post-market withdrawal of drugs. Accordingly, there is a need for more predictive tools to assess hepatotoxicity risk in drug discovery. Three-dimensional (3D) spheroid hepatic cultures have emerged as promising tools to assess mechanisms of hepatotoxicity, as they demonstrate enhanced liver phenotype, metabolic activity, and stability in culture not attainable with conventional two-dimensional hepatic models. Increased sensitivity of these models to drug-induced cytotoxicity has been demonstrated with relatively small panels of hepatotoxicants. However, a comprehensive evaluation of these models is lacking. Here, the predictive value... (truncated)

Title: Drug disposition in pathophysiological conditions.
Expression and activity of several key drug metabolizing enzymes (DMEs) and transporters are altered in various pathophysiological conditions, leading to altered drug metabolism and disposition. This can have profound impact on the pharmacotherapy of widely used clinically relevant medications in terms of safety and efficacy by causing inter-individual variabilities in drug responses. This review article highlights altered drug disposition in inflammation and infectious diseases, and commonly encountered disorders such as cancer, obesity/diabetes, fatty liver diseases, cardiovascular diseases and rheumatoid arthritis. Many of the clinically relevant drugs have a narrow therapeutic index. Thus any changes in the disposition of these drugs... (truncated)

Title: An in vitro coculture system of human peripheral blood mononuclear cells with hepatocellular carcinoma-derived cells for predicting drug-induced liver injury.
Preventing clinical drug-induced liver injury (DILI) remains a major challenge, because DILI develops via multifactorial mechanisms. Immune and inflammatory reactions are considered important mechanisms of DILI; however, biomarkers from in vitro systems using immune cells have not been comprehensively studied. The aims of this study were (1) to identify promising biomarker genes for predicting DILI in an in vitro coculture model of peripheral blood mononuclear cells (PBMCs) with a human liver cell line, and (2) to evaluate these genes as predictors of DILI using a panel of drugs with different clinical DILI risk. Transcriptome-wide analysis of PBMCs cocultured with HepG2... (truncated)

Title: Human hepatic in vitro models reveal distinct anti-NASH potencies of PPAR agonists.
Non-alcoholic steatohepatitis (NASH) is a highly prevalent, chronic liver disease characterized by hepatic lipid accumulation, inflammation, and concomitant fibrosis. Up to date, no anti-NASH drugs have been approved. In this study, we reproduced key NASH characteristics in vitro by exposing primary human hepatocytes (PHH), human skin stem cell-derived hepatic cells (hSKP-HPC), HepaRG and HepG2 cell lines, as well as LX-2 cells to multiple factors that play a role in the onset of NASH. The obtained in vitro disease models showed intracellular lipid accumulation, secretion of inflammatory chemokines, induced ATP content, apoptosis, and increased pro-fibrotic gene expression. These cell systems were... (truncated)

Title: Understanding the hysteresis loop conundrum in pharmacokinetic/pharmacodynamic relationships.
Hysteresis loops are phenomena that sometimes are encountered in the analysis of pharmacokinetic and pharmacodynamic relationships spanning from pre-clinical to clinical studies. When hysteresis occurs it provides insight into the complexity of drug action and disposition that can be encountered. Hysteresis loops suggest that the relationship between drug concentration and the effect being measured is not a simple direct relationship, but may have an inherent time delay and disequilibrium, which may be the result of metabolites, the consequence of changes in pharmacodynamics or the use of a non-specific assay or may involve an indirect relationship. Counter-clockwise hysteresis has been generally... (truncated)

MESH:C087896 - reteplase

Summary:

LLM Prediction Score	0.000 (normalized)
LLM Confidence Score	0.970
Golden Answer (Severity Class)	0.0 (normalized)
Prediction Error	0.000

Retrieved Context:

Title: Augmentation of hepatic uridine-diphosphate glucuronyl transferase activity by antituberculous drugs in hamsters in vivo.
Changes in uridine-diphosphate glucuronyl transferase activity (UDP-GT) in liver homogenates of hamsters treated with different doses of isoniazid (INH), rifampicin (RMP), para-aminosalicylic acid (PAS) and hydrocortisone for several periods of time were studied and expressed as mg of bilirubin conjugated per g of protein per h. INH, RMP, PAS and hydrocortisone induced UDP-GT activity to a statistically significant degree. The optimum dose for high induction was 20 mg for INH, RMP and hydrocortisone, and 200 mg for PAS per kg of body weight. The optimum time of treatment for high induction was 10 consecutive days of intraperitoneal administration for all... (truncated)

Title: Tacrine. A cause of fatal hepatotoxicity?
Tacrine, an acetyl cholinesterase inhibitor used in the treatment of Alzheimer's disease, often causes reversible abnormalities in liver enzymes, but significant hepatotoxicity is uncommon. We describe fatal hepatic failure associated with tacrine administration. A 75-year-old woman with Alzheimer's disease, taking tacrine for 14 months, developed progressive jaundice. Liver function abnormalities developed during tacrine treatment and led to hepatic failure and death. An extensive evaluation for other etiologies of liver disease was negative. Other potentially hepatotoxic medicines had been administered for at least 2 years before beginning tacrine, and postmortem examination of the liver was consistent with drug-induced hepatotoxicity. Approximately half... (truncated)

Title: The current state of serum biomarkers of hepatotoxicity.
The level of serum alanine aminotransferase (ALT) activity reflects damage to hepatocytes and is considered to be a highly sensitive and fairly specific preclinical and clinical biomarker of hepatotoxicity. However, an increase in serum ALT activity level has also been associated with other organ toxicities, thus, indicating that the enzyme has specificity beyond liver in the absence of correlative histomorphologic alteration in liver. Thus, unidentified non-hepatic sources of serum ALT activity may inadvertently influence the decision of whether to continue development of a novel pharmaceutical compound. To assess the risk of false positives due to extraneous sources of serum ALT... (truncated)

Title: [Hepatic involvement associated with ingestion of metapramine].
The authors report the cases of 3 women who developed hepatic injury during administration of metapramine, a tricyclic antidepressant introduced in France in 1984. One patient had jaundice and pruritus; the 2 others had loss of weight. Serum alkaline phosphatase and serum transaminase activities were increased in 3 and 2 patients, respectively. Blood hypereosinophilia was found in one patient; erythrocyte sedimentation rate was elevated in 2 patients. The outcome was favorable after drug withdrawal in the 3 patients. Liver biopsy showed centrolobular cholestasis in the 3 patients. There was no rechallenge; in 2 patients, other drugs than metapramine might be... (truncated)

Title: Liver damage due to free radicals.
The involvement of free radical reactions in the pathogenesis of liver injury has been investigated for many years in a few defined experimental systems using carbon tetrachloride, excess iron or ethanol as prooxidant agents. More recently, the hepatotoxicity of several other free radical-generating compounds has been characterised mainly in the rat hepatocyte model. In particular, the mechanisms by which drugs like paracetamol, halothane, paraquat or conditions such as ischemia-reperfusion exert their damaging activity to the liver have mostly been clarified. Since we are not trying to cure diseases occurring only in rats, the likely relevance of free radical reactions also... (truncated)

MESH:D000077867 - tolcapone

Summary:

LLM Prediction Score	1.000 (normalized)
LLM Confidence Score	0.970
Golden Answer (Severity Class)	1.0 (normalized)
Prediction Error	0.000

Retrieved Context:

Title: Tolcapone: review of its pharmacology and use as adjunctive therapy in patients with Parkinson's disease.
Levodopa has been the gold standard therapy for the motor symptoms of Parkinson's disease for more than three decades. Although it remains the most effective treatment, its long-term use is associated with motor fluctuations and dyskinesias that can be disabling for patients and difficult for physicians to manage medically. In the last 10 years, the catechol-O-methyltransferase (COMT) inhibitor tolcapone has been studied for its efficacy as an adjunctive treatment to levodopa plus a dopa decarboxylase inhibitor. Adjunctive therapy with tolcapone can significantly reduce the dose of levodopa required. Moreover, treatment with tolcapone significantly reduces wearing off and on-off periods in... (truncated)

Title: Tolcapone-related liver dysfunction: implications for use in Parkinson's disease therapy.
Levodopa is the cornerstone of idiopathic Parkinson's disease (PD) treatment. However, after long-term use of levodopa, a significant percentage of patients experience motor fluctuations, which worsen their quality of life. Catechol-O-methyltransferase (COMT) inhibitors reduce levodopa metabolism and enhance the respective plasma levels, resulting in improvements in symptoms and overall quality of life. Tolcapone was the first drug of this class to be marketed, but was withdrawn in the European Union due to its implication in the deaths of three PD patients due to hepatic failure. Three deaths from fulminant hepatic failure in 40000 patient-years is a number that is 10-100... (truncated)

Title: In vitro metabolism of tolcapone to reactive intermediates: relevance to tolcapone liver toxicity.
Tolcapone is a catechol-O-methyltransferase (COMT) inhibitor used for control of motor fluctuations in Parkinson's disease (PD). Since its entry onto the market in 1998, tolcapone has been associated with numerous cases of hepatotoxicity, including three cases of fatal fulminant hepatic failure. The cause of this toxicity is not known; however, it does not occur with the use of the structurally similar drug entacapone. It is known that tolcapone is metabolized to amine (M1) and acetylamine (M2) metabolites in humans, but that the analogous metabolites were not detected in a limited human study of entacapone metabolism. We hypothesized that one or... (truncated)

Title: Tolcapone in Parkinson's disease: liver toxicity and clinical efficacy.
Parkinson's disease patients treated with a combination of levodopa and an aromatic L-amino acid decarboxylase inhibitor usually develop motor complications after some years. To minimise this problem, selective catechol-O-methyltransferase (COMT) inhibitors were developed in order to improve the poor pharmacokinetic profile of levodopa. Tolcapone and entacapone are the two marketed drugs in this class, and both increase the half-life of levodopa and improve clinical parameters, such as the increase in the duration of 'on' and decrease of 'off' time. Soon after its release, tolcapone was suspended in the EU due to it's implication in the deaths of three Parkinsonian patients.... (truncated)

Title: Toxicology and safety of COMT inhibitors.
The development of catechol-O-methyltransferase (COMT) inhibitors for the adjunct treatment to levodopa and aromatic L-amino acid decarboxylase (AADC) inhibitors in Parkinson's disease started in the late 1950s. The first-generation inhibitors were associated with toxic properties: they induced convulsions, or they were toxic to the liver. None of them was taken into clinical use. The second-generation inhibitors entacapone and tolcapone have now been in clinical use for over a decade, and some new inhibitors are under development. The main adverse events in the use of entacapone and tolcapone are dopaminergic and dependent of the concomitant use of levodopa, but the symptoms... (truncated)

MESH:C093230 - fexofenadine

Summary:

LLM Prediction Score	0.000 (normalized)
LLM Confidence Score	0.980
Golden Answer (Severity Class)	0.0 (normalized)
Prediction Error	0.000

Retrieved Context:

Title: Investigation of the effect of hepatic metabolism on off-target cardiotoxicity in a multi-organ human-on-a-chip system.
Regulation of cosmetic testing and poor predictivity of preclinical drug studies has spurred efforts to develop new methods for systemic toxicity. Current in vitro assays do not fully represent physiology, often lacking xenobiotic metabolism. Functional human multi-organ systems containing iPSC derived cardiomyocytes and primary hepatocytes were maintained under flow using a low-volume pumpless system in a serum-free medium. The functional readouts for contractile force and electrical conductivity enabled the non-invasive study of cardiac function. The presence of the hepatocytes in the system induced cardiotoxic effects from cyclophosphamide and reduced them for terfenadine due to drug metabolism, as expected from each... (truncated)

Title: Drug-Induced Liver Injury Caused by Kratom Use as an Alternative Pain Treatment Amid an Ongoing Opioid Epidemic.
Kratom (Mitragyna speciosa) is a prevalent medicinal plant used mainly for the stimulant and analgesic properties provided through multiple alkaloid compounds. Over the past decade, use of kratom has increased despite the limited knowledge of toxicities and adverse side effects. With the current opioid epidemic, both patients and providers are seeking alternative methods to treat both addiction and pain control, and kratom as an alternative means of treatment has increasingly entered the mainstream. In this article, we present the clinical course of a 47-year-old male who developed fatigue, pruritus, and abnormal liver tests (with a mixed hepatocellular/cholestatic pattern) approximately... (truncated)

Title: Mechanisms of Hepatic Cholestatic Drug Injury.
Drug-induced cholestasis represents a form of drug-induced liver disease that can lead to severe impairment of liver function. Numerous drugs have been shown to cause cholestasis and consequently bile duct toxicity. However, there is still lack of therapeutic tools that can prevent progression to advanced stages of liver injury. This review focuses on the various pathological mechanisms by which drugs express their hepatotoxic effects, as well as consequences of increased bile acid and toxin accumulation in the hepatocytes.

Title: Drug interaction study of flavonoids toward OATP1B1 and their 3D structure activity relationship analysis for predicting hepatoprotective effects.
Organic anion transporting polypeptide 1B1 (OATP1B1), a liver-specific uptake transporter, was associated with drug induced liver injury (DILI). Screening and identifying potent OATP1B1 inhibitors with little toxicity is of great value in reducing OATP1B1-mediated DILI. Flavonoids are a group of polyphenols ubiquitously present in vegetables, fruits and herbal products, some of them were reported to produce transporter-mediated DDI. Our objective was to investigate potential inhibitors of OATP1B1 from 99 flavonoids, and to assess the hepatoprotective effects on bosentan induced liver injury. Eight flavonoids, including biochanin A, hispidulin, isoliquiritigenin, isosinensetin, kaempferol, licochalcone A, luteolin and sinensetin exhibited significant inhibition (>50 %)... (truncated)

Title: The Combination of a Human Biomimetic Liver Microphysiology System with BIOLOGXsym, a Quantitative Systems Toxicology (QST) Modeling Platform for Macromolecules, Provides Mechanistic Understanding of Tocilizumab- and GGF2-Induced Liver Injury.
Biologics address a range of unmet clinical needs, but the occurrence of biologics-induced liver injury remains a major challenge. Development of cimaglermin alfa (GGF2) was terminated due to transient elevations in serum aminotransferases and total bilirubin. Tocilizumab has been reported to induce transient aminotransferase elevations, requiring frequent monitoring. To evaluate the clinical risk of biologics-induced liver injury, a novel quantitative systems toxicology modeling platform, BIOLOGXsym™, representing relevant liver biochemistry and the mechanistic effects of biologics on liver pathophysiology, was developed in conjunction with clinically relevant data from a human biomimetic liver microphysiology system. Phenotypic and mechanistic toxicity data and metabolomics... (truncated)

MESH:C455861 - pegfilgrastim

Summary:

LLM Prediction Score	0.000 (normalized)
LLM Confidence Score	0.990
Golden Answer (Severity Class)	0.0 (normalized)
Prediction Error	0.000

Retrieved Context:

Title: Avoiding Peg-Filgrastim Prophylaxis During the Paclitaxel Portion of the Dose-Dense Doxorubicin-Cyclophosphamide and Paclitaxel Regimen: A Prospective Study.
The use of growth factors adds considerable expense and some toxicity to adjuvant breast cancer chemotherapy. We tested the feasibility and safety of omitting routine peg-filgrastim use during the paclitaxel portion of the dose-dense doxorubicin-cyclophosphamide-paclitaxel regimen.

Title: Intensive consolidation with G-CSF support: Tolerability, safety, reduced hospitalization, and efficacy in acute myeloid leukemia patients ≥60 years.
The aim of this study was to evaluate the efficacy and feasibility of intensified consolidation therapy employing fludarabine and ARA-C in cycle 1 and intermediate-dose ARA-C (IDAC) in cycles 2 through 4, in elderly acute myeloid leukemia (AML) patients and to analyze the effects of pegfilgrastim on the duration of neutropenia, overall toxicity, and hospitalization-time during consolidation in these patients. Thirty nine elderly patients with de novo AML (median age 69.9 years) who achieved complete remission (CR) after induction-chemotherapy were analyzed. To examine the effect of pegfilgrastim on neutropenia and hospitalization, we compared cycles 2 and 4 where pegfilgrastim was... (truncated)

Title: A phase I trial of dose-dense (biweekly) carboplatin combined with paclitaxel and pegfilgrastim: a feasibility study in patients with untreated Stage III and IV ovarian, tubal or primary peritoneal cancer: a Gynecologic Oncology Group study.
Dose-dense regimens have been shown to improve outcome when given as adjuvant therapy to patients with breast cancer compared with their three weekly counterparts. We investigated the feasibility of a dose-dense regimen with carboplatin/paclitaxel followed by pegfilgrastim in patients with advanced ovarian cancer. We also investigated the toxicities including the percentage of patients with grade 2 or greater peripheral neurotoxicity and the clinical response of this regimen.

Title: Confirmation of Biosimilarity in a Pharmacokinetic/Pharmacodynamic Study in Healthy Volunteers for an Analytically Highly Similar Pegfilgrastim.
A phase 1 pharmacokinetic (PK) and pharmacodynamic (PD) study was conducted to demonstrate similarity of a proposed pegfilgrastim biosimilar to its reference product. In a single-dose, randomized, assessor-blinded, 2-way crossover, active-controlled PK/PD study, 66 healthy adults received the proposed pegfilgrastim biosimilar and US-licensed pegfilgrastim reference product. Primary end points were pegfilgrastim AUCt and Cmax (PK), and absolute neutrophil count AUECt and Emax (PD). Safety and immunogenicity were also measured. Fifty-six subjects completed both arms of the study. Mean pegfilgrastim concentration-time profile for both products was similar, with the 90% confidence intervals (CI) of the relative mean ratio for the primary... (truncated)

Title: A Phase II trial of docetaxel and carboplatin administered every 2 weeks as preoperative therapy for stage II or III breast cancer: NCCTG study N0338.
We conducted a multicenter phase II trial to assess the efficacy and toxicity of docetaxel and carboplatin combination as neoadjuvant therapy for stage II or III breast cancer (BC).

MESH:D005442 - flumazenil

Summary:

LLM Prediction Score	0.000 (normalized)
LLM Confidence Score	0.980
Golden Answer (Severity Class)	0.0 (normalized)
Prediction Error	0.000

Retrieved Context:

Title: [Acute pharmacologic poisoning (I). Psychotropic and antiarrhythmic drugs].
In the evaluation of Acute Drug Poisoning (ADP) in patients seriously ill with a potentially non-toxic dose of the drug that is theoretically responsible, it is important to insist on anamnesis in the coingestion of other drugs or toxics. Initially attention is given to life support measures, oxygenation, protection of the airway and expanding the volemia. The ECG is a diagnostic tool of the first order in ADPs, above all for tricyclic antidepressants (TAD) and cardio-vascular drugs. In the majority of cases continuous monitoring is usually necessary during the first 12-24 hours. The benzodiazepines do not usually give rise to... (truncated)

Title: Inhibition of canalicular and sinusoidal taurocholate efflux by cholestatic drugs in human hepatoma HepaRG cells.
HepaRG cells are highly-differentiated human hepatoma cells, which are increasingly recognized as a convenient cellular model for in vitro evaluation of hepatic metabolism, transport, and/or toxicity of drugs. The present study was designed to evaluate whether HepaRG cells can also be useful for studying drug-mediated inhibition of canalicular and/or sinusoidal hepatic efflux of bile acids, which constitutes a major mechanism of drug-induced liver toxicity. For this purpose, HepaRG cells, initially loaded with the bile acid taurocholate (TC), were reincubated in TC-free transport assay medium, in the presence or absence of calcium or drugs, before analysis of TC retention. This method... (truncated)

Title: The use of flumazenil in the management of acute drug poisoning--a review.
The popularity and widespread availability of benzodiazepines (BZD) has led to their frequent abuse in intentional drug poisoning. Although mortality from pure BZD overdose is usually small, in elderly, debilitated patients, or when BZD are combined with other CNS depressant drugs, morbidity increases significantly and outcome may be fatal. Drug overdose is therefore a medical emergency necessitating close observation and support of vital functions. Recently, the specific BZD antagonist flumazenil (Anexate) has become clinically available and much experience in its usefulness has accumulated. The present review summarizes a total of 30 studies and reports published to date, involving approximately 760... (truncated)

Title: Case Report: Enhanced Diazepam Elimination With the Molecular Adsorbents Recirculating System (MARS) in Severe Autointoxication: A Survival Case Report.
Objective: Due to the extensive use of diazepam worldwide, self-induced intoxication is very common, yet rarely fatal. Nevertheless, the management of intoxication caused by extremely high doses of diazepam is not known, as well as the effectiveness of flumazenil, a specific benzodiazepine (BDZ) antagonist. Here we present the first report on the enhanced elimination (clearance) of diazepam using the Molecular Adsorbents Recirculating System (MARS) following autointoxication with an extremely high dose as part of a suicide attempt. Case: A 44-year-old male patient was admitted to the ICU because of impaired consciousness following the ingestion of 20 g of diazepam. Blood... (truncated)

Title: Etifoxine for pain patients with anxiety.
Etifoxine (etafenoxine, Stresam®) is a non-benzodiazepine anxiolytic with an anticonvulsant effect. It was developed in the 1960s for anxiety disorders and is currently being studied for its ability to promote peripheral nerve healing and to treat chemotherapy-induced pain. In addition to being mediated by GABAAα2 receptors like benzodiazepines, etifoxine appears to produce anxiolytic effects directly by binding to β2 or β3 subunits of the GABAA receptor complex. It also modulates GABAA receptors indirectly via stimulation of neurosteroid production after etifoxine binds to the 18 kDa translocator protein (TSPO) of the outer mitochondrial membrane in the central and peripheral nervous systems,... (truncated)

MESH:D009266 - nalbuphine

Summary:

LLM Prediction Score	0.000 (normalized)
LLM Confidence Score	0.980
Golden Answer (Severity Class)	0.0 (normalized)
Prediction Error	0.000

Retrieved Context:

Title: [Conventional techniques for analgesia: opioids and non-opioids. Indications, adverse effects and monitoring]. Morphine dosage must be carefully adapted in patients with renal failure or severe liver failure. The i.v. route is used for morphine titration in the post anaesthesia care unit (PACU), or for analgesia in children. Systematic (not on demand) intramuscular or subcutaneous morphine must be administered at intervals not longer than 4 hours. Dosage is best determined after i.v. titration in the PACU. Codeine, administered orally, is metabolised into morphine. Codeine has almost no effect in 7% of Caucasians and at least 15% of Asians. Nalbuphine, which has a sedative effect and a short half-life, is mainly used in children.... (truncated)

Title: Pharmacokinetic Study of Nalbuphine in Surgical Patients Undergoing General Anesthesia with Varying Degrees of Liver Dysfunction.
This study aimed to characterize the pharmacokinetics of nalbuphine in patients undergoing general anesthesia with varying degrees of liver dysfunction.

Title: <i>Syzygium aqueum</i>: A Polyphenol- Rich Leaf Extract Exhibits Antioxidant, Hepatoprotective, Pain-Killing and Anti-inflammatory Activities in Animal Models.
<i>Syzygium aqueum</i> is widely used in folk medicine. A polyphenol-rich extract from its leaves demonstrated a plethora of substantial pharmacological properties. The extract showed solid antioxidant properties <i>in vitro</i> and protected human keratinocytes (HaCaT cells) against UVA damage. The extract also reduced the elevated levels of ALT, AST, total bilirubin (TB), total cholesterol (TC) and triglycerides (TG) in rats with acute CCl₄ intoxication. In addition to reducing the high MDA level, the extract noticeably restored GSH and SOD to the normal control levels in liver tissue homogenates and counteracted the deleterious histopathologic changes in liver after CCl₄ injection. Additionally, the... (truncated)

Title: Impact of intravenous administration of nalbuphine at different time points for postoperative analgesia and sedation in adenotonsillectomized children: a prospective, randomized controlled trial.
To compare the efficacy of intravenous administration of nalbuphine at different time points for postoperative analgesia and sedation in adenotonsillectomized children.

Title: Functional shift with maintained regenerative potential following portal vein ligation.
Selective portal vein ligation (PVL) allows the two-stage surgical resection of primarily unresectable liver tumours by generating the atrophy and hypertrophy of portally ligated (LL) and non-ligated lobes (NLL), respectively. To evaluate critically important underlying functional alterations, present study characterised in vitro and vivo liver function in male Wistar rats (n = 106; 210-250 g) before, and 24/48/72/168/336 h after PVL. Lobe weights and volumes by magnetic resonance imaging confirmed the atrophy-hypertrophy complex. Proper expression and localization of key liver transporters (Ntcp, Bsep) and tight junction protein ZO-1 in isolated hepatocytes demonstrated constantly present viable and well-polarised cells in both lobes. In vitro taurocholate... (truncated)

MESH:D004999 - amifostine

Summary:

LLM Prediction Score	0.000 (normalized)
LLM Confidence Score	0.970
Golden Answer (Severity Class)	0.0 (normalized)
Prediction Error	0.000

Retrieved Context:

Title: A randomized trial of amifostine as a cytoprotectant for patients receiving myeloablative therapy for allogeneic hematopoietic stem cell transplantation.
We initiated a randomized study of amifostine (the organic thiophosphate formerly known as WR-2721) given to patients during myeloablative conditioning therapy for allogeneic bone marrow transplantation. Amifostine was given at a dose of 1000 mg/day of conditioning and was well tolerated if attention was given to serum calcium levels, blood pressure and antiemetics. Since August 1998, 60 patients (30 on each arm) have completed the study. There was no significant difference in the days to neutrophil or platelet engraftment in either arm of the study. Significantly, the duration of grade I-IV mucositis was decreased in the group that received amifostine... (truncated)

Title: Amifostine (Ethyol) as modulator of hepatic and biliary toxicity from intraarterial hepatic chemoembolization: results of a phase I study.
Hepatic and biliary toxicity are still significant problems after intraarterial hepatic chemoembolization for liver metastases from large bowel cancers. In about 30-60% of the patients hepatic and biliary toxicity are the limiting aspects of intraarterial hepatic chemoembolization and exclude a lot of patients from a repeated beneficial treatment. Amifostine (Ethyol) is a prodrug that must be dephosphorylated to the free thiol in which form it can detoxify free oxygen radicals generated by radiation, hypoxia and by drugs such anthracyclines, platinum analogues and alkylating agents. Amifostine as inactive prodrug is primarily metabolized at the tissue site by membrane alkaline phosphatase, which... (truncated)

Title: Use of amifostine, a novel cytoprotective, in alpha-amanitin poisoning.
Mortality from ingestions of the mushroom Amanita phalloides remains as high as 20-40% with many surviving patients requiring liver transplantation. A variety of treatments for Amanita ingestion have been evaluated, yet other than supportive measures, no effective therapy has been identified. In addition, an antidote for Amanita toxicity may not be practical due to delayed patient presentation. The drug amifostine was proposed to potentially improve survival from alpha-amanitin toxicity by conferring cytoprotective effects on hepatocytes at risk for cell death. Amifostine is used as a radio--and chemo-protective agent. It protects against lipoperoxidation, interferes with the cross-linking of DNA, and may... (truncated)

Title: The Efficacy of Amifostine against Multiple-Dose Doxorubicin-Induced Toxicity in Rats.
Amifostine is well known cytoprotector which is efficient when administered before a wide range of antineoplastic agents. The aim of our study was to investigate amifostine effects on doxorubicin-induced toxic changes in rats. Amifostine (75 mg/kg ip) was given 30 min before each dose of doxorubicin (cumulatively 20 mg/kg ip, for 28 days). The animals' whole-body, liver, and kidney weight, serum biochemical examination, as well as microscopic examination of bone marrow, peripheral blood, liver, and kidney, were done on day 56 of the study. Hepatic and renal alterations were carefully quantified by semiquantitative grading scales-hepatic and renal damage score, respectively.... (truncated)

Title: Amifostine inhibits acrylamide-induced hepatotoxicity by inhibiting oxidative stress and apoptosis.
Acrylamide (ACR) is a toxic chemical agent that can induce hepatotoxicity through different mechanisms including oxidative stress and apoptosis. Amifostine is an important hepatoprotective and anti-oxidant compound. In this research, the hepatoprotective effect of amifostine on ACR-induced hepatotoxicity in rats has been investigated.

MESH:C018421 - rifapentine

Summary:

LLM Prediction Score	0.500 (normalized)
LLM Confidence Score	0.980
Golden Answer (Severity Class)	0.5 (normalized)
Prediction Error	0.000

Retrieved Context:

Title: Antituberculosis drugs and hepatotoxicity.
Isoniazid, pyrazinamide and rifampicin have hepatotoxic potential, and can lead to such reactions during antituberculosis chemotherapy. Most of the hepatotoxic reactions are dose-related; some are, however, caused by drug hypersensitivity. The immunogenetics of antituberculosis drug-induced hepatotoxicity, especially inclusive of acetylaor phenotype polymorphism, have been increasingly unravelled. Other principal clinical risk factors for hepatotoxicity are old age, malnutrition, alcoholism, HIV infection, as well as chronic hepatitis B and C infections. Drug-induced hepatic dysfunction usually occurs within the initial few weeks of the intensive phase of antituberculosis chemotherapy. Vigilant clinical (including patient education on symptoms of hepatitis) and biochemical monitoring are mandatory... (truncated)

Title: Anti-Tuberculosis Treatment: Induced Hepatotoxicity - A Case Report.
Tuberculosis is a potentially communicable disease that can infect any organ in the body such as bones, kidney, intestine but primarily involves lung parenchyma (Pulmonary tuberculosis). The prevalence of TB is 256 per 100,000 population in India. Hepatotoxicity, gastrointestinal and neurological disorders were some the Adverse Drug Reactions (ADR's) reported that significantly increases the mortality rate which leads to decreased efficacy of the treatment. Hepatotoxicity is the most commonly reported ADR in patients treated with anti-tubercular drugs such as isoniazid, rifampicin and pyrazinamide. Clinical manifestations of hepatotoxicity include abdominal pain, nausea, vomiting, and jaundice. We report the case of a... (truncated)

Title: Rifapentine-proliposomes for inhalation: in vitro and in vivo toxicity.
Oral therapy for pulmonary tuberculosis (TB) treatment suffers from the limitation of hepatic metabolism leading insufficient concentration of antitubercular (anti-TB) drugs in alveolar macrophage which harbors Mycobacterium tuberculosis (MTB). Targeted aerosol delivery of antituberculous drug to lung is efficient for treating local lung TB infection.

Title: Treatment of latent tuberculosis infection: An update.
Isoniazid (INH) has been the mainstay of treatment of latent tuberculosis infection for almost 50 years. The currently recommended preferred regimen is 9 months daily self-administered INH (9H); this has efficacy of more than 90% if completed properly. Unfortunately, INH is associated with serious adverse events, including hepatotoxicity. Although risk factors for this complication are well established, allowing for better selection of candidates for therapy, this complication still occurs, and is occasionally fatal. Hence close follow up of patients is necessary, increasing the cost and complexity of treatment. This problem, plus the lengthy duration, results in poor acceptance by patients... (truncated)

Title: Clinical and pharmacological hallmarks of rifapentine's use in diabetes patients with active and latent tuberculosis: do we know enough?
Rifapentine is a rifamycin derivate approved by the US Food and Drug Administration in 1998 for the treatment of active, drug-susceptible tuberculosis (TB). In 2014, rifapentine was approved for the treatment of latent TB infection in patients at high risk of progression to active disease and is currently under evaluation by the European Medicines Agency. Expanding indications of rifapentine largely affect diabetes patients, since about one-third of them harbor latent TB. Clinical consequences of rifapentine use in this population and potentially harmful interactions with hypoglycemic agents are widely underexplored and generally considered similar to the ones of rifampicin. Indeed, rifapentine... (truncated)

MESH:C112666 - gadoversetamide

Summary:

LLM Prediction Score	0.000 (normalized)
LLM Confidence Score	0.860
Golden Answer (Severity Class)	0.0 (normalized)
Prediction Error	0.000

Retrieved Context:

Title: Cumulative administrations of gadolinium-based contrast agents: risks of accumulation and toxicity of linear vs macrocyclic agents.
Ever since gadolinium was found to deposit in the brain of patients with normal kidney function by Kanda et al. in 2014, several studies have been conducted to evaluate its effect on the patients' health. However, conflicting results were obtained regarding imaging in gadolinium retention. These finding were attributed to the chelating structure of the administered gadolinium-based contrast agent (GBCA): linear agents were found to accumulate in the dentate nucleus (DN) and the globus pallidus (GP) of subjects even after one dose. There are some contradictory results when assessing macrocyclic agents. In the following article, we review the basis of... (truncated)

Title: Nephrogenic systemic fibrosis risk after liver magnetic resonance imaging with gadoxetate disodium in patients with moderate to severe renal impairment: results of a prospective, open-label, multicenter study.
The objective of this study was to assess the risk of gadoxetate disodium in liver imaging for the development of nephrogenic systemic fibrosis (NSF) in patients with moderate to severe renal impairment.

Title: Risk factors for developing gadolinium-induced nephrogenic systemic fibrosis.
To identify medications and other potential risk factors, in addition to renal dysfunction, for developing gadolinium-induced nephrogenic systemic fibrosis (NSF).

Title: Compensatory biliary and urinary excretion of gadobenate ion after administration of gadobenate dimeglumine (MultiHance(®)) in cases of impaired hepatic or renal function: a mechanism that may aid in the prevention of nephrogenic systemic fibrosis?
To determine whether increased elimination of gadobenate ion via the hepatobiliary pathway might compensate for reduced/absent elimination via the urinary pathway in the event of compromised renal function, as a possible protective mechanism against nephrogenic systemic fibrosis (NSF).

Title: Understanding nephrogenic systemic fibrosis.
Nephrogenic systemic fibrosis (NSF) is a rare and a debilitating disease noted uncommonly in patients with impaired renal function when exposed to low-stability gadolinium-based contrast agents (Gd-CAs). According to experimental studies, cytokines released by the stimulation of effector cells such as skin macrophages and peripheral blood monocytes activate circulating fibroblasts which play a major role in the development of NSF lesions. The presence of permissive factors, presumably, provides an environment conducive to facilitate the process of fibrosis. Multiple treatment modalities have been tried with variable success rates. More research is necessary to elucidate the underlying pathophysiological mechanisms which could potentially... (truncated)

MESH:D000111 - acetylcysteine

Summary:

LLM Prediction Score	0.000 (normalized)
LLM Confidence Score	0.990
Golden Answer (Severity Class)	0.0 (normalized)
Prediction Error	0.000

Retrieved Context:

Title: N-acetylcysteine improves liver function in patients with non-alcoholic Fatty liver disease.
Non-alcoholic fatty liver change is a common disease of the liver in which oxidative stress plays a basic role. Studies are largely focused on protecting the liver by means of anti-oxidative material. The aim of this study is to evaluate the role of N- acetylcysteine in the process of liver injury.

Title: A Randomized Controlled Trial of Intravenous N-Acetylcysteine in the Management of Anti-tuberculosis Drug-Induced Liver Injury.
Liver injury is a common complication of anti-tuberculosis therapy. N-acetylcysteine (NAC) used in patients with paracetamol toxicity with limited evidence of benefit in liver injury due to other causes.

Title: Drug-Induced Liver Injury: Clinical Evidence of N-Acetyl Cysteine Protective Effects.
Oxidative stress is a key pathological feature implicated in both acute and chronic liver diseases, including drug-induced liver injury (DILI). The latter describes hepatic injury arising as a direct toxic effect of administered drugs or their metabolites. Although still underreported, DILI remains a significant cause of liver failure, especially in developed nations. Currently, it is understood that mitochondrial-generated oxidative stress and abnormalities in phase I/II metabolism, leading to glutathione (GSH) suppression, drive the onset of DILI. N-Acetyl cysteine (NAC) has attracted a lot of interest as a therapeutic agent against DILI because of its strong antioxidant properties, especially in relation... (truncated)

Title: Intravenous acetylcysteine for the treatment of acetaminophen overdose.
Acetaminophen is a leading cause of overdose-related hepatotoxicity. Although acetylcysteine prevents or minimizes acetaminophen-induced hepatotoxicity and reduces mortality, some patients presenting with complicated overdose scenarios (massive ingestions or combination or modified-release formulations) may develop toxicity despite administration of recommended dosage regimen.

Title: Protective effects of <i>N</i>-acetylcysteine on cisplatin-induced oxidative stress and DNA damage in HepG2 cells.
Hepatocyte injury is a common pathological effect of cisplatin (CDDP) in various solid tumor therapies. Thus, strategies for minimizing CDDP toxicity are of great clinical interest. <i>N</i>-acetylcysteine (NAC), a known antioxidant, is often used as an antidote for acetaminophen overdose in the clinic due to its ability to increase the levels of glutathione (GSH). In the present study, the aim was to investigate the protective effects of NAC against CDDP-induced apoptosis in human-derived HepG2 cells. The results showed that upon exposure of the cells to CDDP, oxidative stress was significantly induced. DNA damage caused by CDDP was associated with cell... (truncated)

MESH:D003366 - cosyntropin

Summary:

LLM Prediction Score	0.000 (normalized)
LLM Confidence Score	0.930
Golden Answer (Severity Class)	0.0 (normalized)
Prediction Error	0.000

Retrieved Context:

Title: Cortisol values during the standard-dose cosyntropin stimulation test: Personal experience with Elecsys cortisol II assay.
Label="Purpose">There has been debate regarding the appropriate cortisol cutoff during the cosyntropin stimulation test (CST) when newer cortisol assays are used. We aimed to evaluate the proper cortisol values during the standard dose CST in patients with normal hypothalamic-pituitary-adrenal (HPA) axis when the Elecsys® Cortisol II assay from Roche Diagnostics is used.

MESH:D012844 - sincalide

Summary:

LLM Prediction Score	0.000 (normalized)
LLM Confidence Score	0.840
Golden Answer (Severity Class)	0.0 (normalized)
Prediction Error	0.000

Retrieved Context:

Title: A New Perspective on Liver Injury by Traditional Chinese Herbs Such As <i>Polygonum multiflorum</i>: The Geographical Area of Harvest As an Important Contributory Factor.
Herbal medicine has been widely used in the treatment of various diseases; however, the adverse reactions cannot be ignored. Most previous studies have ignored the relationship between the factors of geographical areas/batches and toxicity. This study used <i>Polygonum multiflorum</i> (PM) as an example to analyze the relationship between the geographical areas/batches and toxicity and speculated on the hepatotoxicity-inducing components in PM based on high content screening, UHPLC-Q-TOF/MS and Progenesis QI software analysis. The results of the study show that the toxicity of PM was obviously different among the different geographical areas, and the most toxic PM was from the Sichuan... (truncated)

Title: Integrating systematic pharmacology-based strategy and experimental validation to explore mechanism of <i>Tripterygium</i> glycoside on cholangiocyte-related liver injury.
Label="Objective" NlmCategory="UNASSIGNED"> <i>Tripterygium</i> glycoside (TG) is widely used in clinical practice for its multiple bioactivities including anti-inflammatory and immunosuppressive effects. However, emerging studies have frequently reported TG-induced adverse reactions to multiple organs, especially liver. Here, this study aimed to investigate the mechanism of liver damage induced by TG and explore representative components to reflect TG hepatotoxicity.

Title: Inhibition of Oxidative Stress and ALOX12 and NF-κB Pathways Contribute to the Protective Effect of Baicalein on Carbon Tetrachloride-Induced Acute Liver Injury.
This study investigates the protective effect of baicalein on carbon tetrachloride (CCl₄)-induced acute liver injury and the underlying molecular mechanisms. Mice were orally administrated baicalein at 25 and 100 mg/kg/day for 7 consecutive days or ferrostatin-1 (Fer-1) at 10 mg/kg was i.p. injected in mice at 2 and 24 h prior to CCl₄ injection or the vehicle. Our results showed that baicalein or Fer-1 supplementation significantly attenuated CCl₄ exposure-induced elevations of serum alanine aminotransferase and aspartate aminotransferase, and malondialdehyde levels in the liver tissues and unregulated glutathione levels. Baicalein treatment inhibited the nuclear factor kappa-B (NF-κB) pathway, activated the erythroid... (truncated)

Title: Mitochondrial toxicants in Xian-Ling-Gu-Bao induce liver injury by regulating the PI3K/mTOR signaling pathway: an in vitro study.
Drug-induced mitochondrial toxicity is thought to be a common mechanism of drug hepatotoxicity. Xian-Ling-Gu-Bao (XLGB) oral preparation is a commonly used drug for osteoporosis in China. Classical safety evaluation studies have shown that the entire preparation and six Chinese herbal medicines have high safety, but the incidence of drug-induced liver damage due to XLGB remains high, the mechanism and toxic substances causing liver injury are still unclear. The purpose of this study is to identify compounds with potential mitochondrial liabilities in XLGB, and to clarify their underlying mechanisms and related pathways.

Title: Subchronic Toxicity Studies of Cortex Dictamni Extracts in Mice and Its Potential Hepatotoxicity Mechanisms in Vitro.
Cortex Dictamni is a commonly-used traditional Chinese herbal medicine for the treatment of skin inflammation, tinea, and eczema. Recently, some studies reported that Cortex Dictamni might induce liver injury, suggesting more attention to its safety. The current study was designed to investigate subchronic toxicity of Cortex Dictamni aqueous extract (CDAE) and ethanol extract (CDEE) in mice and the potential hepatotoxicity mechanisms in vitro. Firstly, CDAE or CDEE groups were administrated with varying dosages (2.3, 4.6, or 9.2 g/kg/day, p.o.) in mice for 28 days in subchronic toxicity studies. General clinical signs and biochemical parameters were examined, and morphological analyses were... (truncated)

MESH:D010197 - pancuronium

Summary:

LLM Prediction Score	0.000 (normalized)
LLM Confidence Score	0.960
Golden Answer (Severity Class)	0.0 (normalized)
Prediction Error	0.000

Retrieved Context:

Title: [Death after delayed recovery and respiratory failure following injection sclerotherapy in a small infant under general anesthesia].
A 12 month old boy weighing 6.4kg with esophageal varices caused by congenital biliary hypoplasia was scheduled for emergency sclerotherapy under general anesthesia. Anesthesia was induced with thiamylal sodium 3mg.kg-1 i.v. and then maintained with nitrous oxide, oxygen and a low concentration of enflurane, paralysed with pancuronium bromide. As soon as a small dose of sclerosant (5% ethanolamine oleate) was injected, transient moderate bradycardia and hypotension occurred. As his spontaneous breathing was very weak and the movements of extremities convulsive and his consciousness drowsy, prophylactic respiratory care was carried out. He had pneumonia and manifestation of DIC 4 days after... (truncated)

Title: [Anesthesia-relevant changes in metabolic parameters with different circulatory and liver functions].
The relationship between oxygen consumption (VO2) and oxygen delivery (DO2) is of interest in critically ill patients. Various studies of these parameters have resulted in different concepts for optimizing DO2 and VO2. During liver transplantation without anhepatic veno-venous bypass, caval cross-clamping initiates a series of haemodynamic and metabolic alterations including the rapid change from hyperdynamic to hypodynamic conditions. In addition, simultaneous changes in DO2 and VO2 occur in these patients. The goal of our present study was to test the clinical relevance of therapeutic interventions based on metabolic monitoring in patients with terminal liver disease undergoing orthotopic liver transplantation. PATIENTS... (truncated)

Title: Anesthesiological management during isolated liver perfusion.
The treatment of irresectable hepatic metastases is limited by the systemic toxicity of anticancer agents. Isolated hyperthermic liver perfusion (IHLP) with anticancer agents is a new therapy for irresectable liver tumors. The risks of this therapy lie in the extended operation, the anhepatic phase and the possibility of liver damage due to the anticancer drug and hyperthermia. Experience of this method is rare, and the side effects are not well known. To estimate the individual risk of patients before isolated liver perfusion an extended evaluation of the preoperative conditions is usual. Titration of all anesthetic agents is advisable to prevent... (truncated)

Title: Enflurane in cardiac surgery.
Due to its advantageous properties the volatile anesthetic enflurane is used as the main agent for all routine open heart cases in our department. The technique consists of the induction of the heavely premedicated patient with fentanyl (10 micrograms/kg i.v.) followed by pancuroniumbromide (100 micrograms/kg i.v.). For the maintenance of anesthesia, i.e. before, during, and after cardio-pulmonary bypass, enflurane together with nitrous-oxide (up to 66%) is used, in a concentration between 1.0-2.0 vol%. Compared to other volatile anesthetics we foster a technique with enflurane because it has following advantages: 1. Less negative inotropic effect on the myocardium. 2. Less tendency... (truncated)

Title: Atracurium and vecuronium: two unique neuromuscular blocking agents.
Atracurium and vecuronium are two new nondepolarizing skeletal muscle relaxants that were developed to overcome the deficiencies seen with currently available agents (tubocurarine, metocurine, pancuronium, and gallamine). Both compounds have unique metabolic profiles, separating them from other nondepolarizing agents. Neither drug depends on normal renal function for excretion and each can safely be given to patients with renal failure. Atracurium also does not depend on hepatic function for metabolism; however, vecuronium may require dosing adjustments in hepatic disease. Atracurium and vecuronium have similar onset times for muscle relaxation but shorter durations of action than other nondepolarizing muscle relaxants. Both agents... (truncated)

MESH:D010418 - pentagastrin

Summary:

LLM Prediction Score	0.000 (normalized)
LLM Confidence Score	0.950
Golden Answer (Severity Class)	0.0 (normalized)
Prediction Error	0.000

Retrieved Context:

Title: The pentagastrin test in the diagnosis of the carcinoid syndrome. Blockade of gastrointestinal symptoms by ketanserin.
The levels of 5-hydroxytryptamine (serotonin, 5-HT) and substance P (SP) were assayed (using high performance liquid chromatography-electron capture and radioimmunoassay methods) in the peripheral blood of 17 patients with known mid-gut carcinoids, 16 of whom had hepatic metastases. All patients had supranormal basal levels of 5-HT and SP. The clinical and hormonal changes induced by two provocation tests, intravenous pentagastrin (PG) and calcium infusion, were compared. Pentagastrin caused flushing in all the patients, induced gastrointestinal symptoms in all but one of the patients with hepatic involvement, and universally elevated circulating 5-HT levels. Pretreatment with a 5-HT2-receptor blocking agent, ketanserin, abolished... (truncated)

Title: Bioconjugation of calcium phosphosilicate composite nanoparticles for selective targeting of human breast and pancreatic cancers in vivo.
The early diagnosis of cancer is the critical element in successful treatment and long-term favorable patient prognoses. The high rate of mortality is mainly attributed to the tendency for late diagnoses as symptoms may not occur until the disease has metastasized, as well as the lack of effective systemic therapies. Late diagnosis is often associated with the lack of timely sensitive imaging modalities. The promise of nanotechnology is presently limited by the inability to simultaneously seek, treat, and image cancerous lesions. This study describes the design and synthesis of fluorescent calcium phosphosilicate nanocomposite particles (CPNPs) that can be systemically targeted... (truncated)

Title: Current pharmacological management of gastroesophageal reflux disease.
Gastroesophageal reflux disease (GERD), a common disorder with troublesome symptoms caused by reflux of gastric contents into the esophagus, has adverse impact on quality of life. A variety of medications have been used in GERD treatment, and acid suppression therapy is the mainstay of treatment for GERD. Although proton pump inhibitor is the most potent acid suppressant and provides good efficacy in esophagitis healing and symptom relief, about one-third of patients with GERD still have persistent symptoms with poor response to standard dose PPI. Antacids, alginate, histamine type-2 receptor antagonists, and prokinetic agents are usually used as add-on therapy to... (truncated)

Title: ACVIM consensus statement: Support for rational administration of gastrointestinal protectants to dogs and cats.
The gastrointestinal (GI) mucosal barrier is continuously exposed to noxious toxins, reactive oxygen species, microbes, and drugs, leading to the development of inflammatory, erosive, and ultimately ulcerative lesions. This report offers a consensus opinion on the rational administration of GI protectants to dogs and cats, with an emphasis on proton pump inhibitors (PPIs), histamine type-2 receptor antagonists (H₂ RAs), misoprostol, and sucralfate. These medications decrease gastric acidity or promote mucosal protective mechanisms, transforming the management of dyspepsia, peptic ulceration, and gastroesophageal reflux disease. In contrast to guidelines that have been established in people for the optimal treatment of gastroduodenal ulcers... (truncated)

Title: Human hepatic in vitro models reveal distinct anti-NASH potencies of PPAR agonists.
Non-alcoholic steatohepatitis (NASH) is a highly prevalent, chronic liver disease characterized by hepatic lipid accumulation, inflammation, and concomitant fibrosis. Up to date, no anti-NASH drugs have been approved. In this study, we reproduced key NASH characteristics in vitro by exposing primary human hepatocytes (PHH), human skin stem cell-derived hepatic cells (hSKP-HPC), HepaRG and HepG2 cell lines, as well as LX-2 cells to multiple factors that play a role in the onset of NASH. The obtained in vitro disease models showed intracellular lipid accumulation, secretion of inflammatory chemokines, induced ATP content, apoptosis, and increased pro-fibrotic gene expression. These cell systems were... (truncated)

MESH:D004369 - pentetic acid

Summary:

LLM Prediction Score	0.000 (normalized)
LLM Confidence Score	0.940
Golden Answer (Severity Class)	0.0 (normalized)
Prediction Error	0.000

Retrieved Context:

Title: A Mixture of Endocrine Disruptors and the Pesticide Roundup® Induce Oxidative Stress in Rabbit Liver When Administered under the Long-Term Low-Dose Regimen: Reinforcing the Notion of Real-Life Risk Simulation.
Humans are exposed to xenobiotic mixtures daily through the long-term, low-dose regimen. Investigations designed to simulate this exposure profile approach the real-life risk simulation (RLRS) idea of modern toxicology. The purpose of the present study was to investigate the effects of 12-month exposure of New Zealand rabbits to a xenobiotic mixture comprising seven endocrine disruptors (EDs), which are chemical substances raising great concerns for human health, as well as the herbicide glyphosate, and its commercial formulation Roundup®, on blood and tissues redox status. It is reported herein that at the systemic level, the administration of the EDs mixture induced perturbations... (truncated)

Title: Liver proteome alterations in psychologically distressed rats and a nootropic drug.
Chronic psychological distress is considered today a pandemic due to the modern lifestyle and has been associated with various neurodegenerative, autoimmune, or systemic inflammation-related diseases. Stress is closely related to liver disease exacerbation through the high activity of the endocrine and autonomic nervous systems, and the connection between the development of these pathologies and the physiological effects induced by oxidative stress is not yet completely understood. The use of nootropics, as the cognitive enhancer and antioxidant piracetam, is attractive to repair the oxidative damage. A proteomic approach provides the possibility to obtain an in-depth comprehension of the affected cellular processes... (truncated)

Title: Antioxidant and hepatoprotective effects of novel heteropolysaccharide isolated from <i>Lobularia maritima</i> on CCl4-induced liver injury in rats.
The aim of the present study was to investigate the extraction and the characterization of a novel heteropolysaccharide from Tunisian halophyte <i>Lobularia maritima</i> (LmPS). We were also interested in its antioxidant, anti-inflammatory, and hepatoprotective effects on carbon tetrachloride (CCl₄)-induced liver injury in rats. LmPS physicochemical properties were evaluated by thin-layer chromatography (TLC), high-performance liquid chromatography (HPLC), thermogravimetric analysis (TGA), and UV absorption. According to TLC and HPLC results, LmPS was a heteropolysaccharide composed of glucose, galactose, and xylose. Its molecular weight was 130.62 kDa. This heteropolysaccharide was characterized by a significant antioxidant potential and was efficient against oxidative stress and CCL₄-induced... (truncated)

Title: Low-Level Saturated Fatty Acid Palmitate Benefits Liver Cells by Boosting Mitochondrial Metabolism via CDK1-SIRT3-CPT2 Cascade.
Saturated fatty acids (SFAs) (the "bad" fat), especially palmitate (PA), in the human diet are blamed for potential health risks such as obesity and cancer because of SFA-induced lipotoxicity. However, epidemiological results demonstrate a latent benefit of SFAs, and it remains elusive whether a certain low level of SFAs is physiologically essential for maintaining cell metabolic hemostasis. Here, we demonstrate that although high-level PA (HPA) indeed induces lipotoxic effects in liver cells, low-level PA (LPA) increases mitochondrial functions and alleviates the injuries induced by HPA or hepatoxic agent carbon tetrachloride (CCl₄). LPA treatment in mice enhanced liver mitochondrial activity and... (truncated)

Title: Formation of gamma-ketoaldehyde-protein adducts during ethanol-induced liver injury in mice.
Ethanol metabolism promotes the formation of a variety of reactive aldehydes in the liver. These aldehydes can rapidly form covalent protein adducts. Accumulating evidence indicates that these protein adducts may contribute to ethanol-mediated liver injury. Overproduction of gamma-ketoaldehydes, levuglandins (LGs) and isolevuglandins, is implicated in the pathogenesis of several chronic inflammatory diseases. gamma-Ketoaldehydes can form protein adducts orders of magnitude more quickly than 4-hydroxynonenal (4-HNE) or malondialdehyde. We hypothesized that

MESH:C062843 - carboprost tromethamine

Summary:

LLM Prediction Score	0.000 (normalized)
LLM Confidence Score	0.950
Golden Answer (Severity Class)	0.0 (normalized)
Prediction Error	0.000

Retrieved Context:

Title: A Comparison of Dexmedetomidine and Midazolam for the Prevention of Postoperative Nausea and Vomiting Caused by Hemabate in Cesarean Delivery: A Randomized Controlled Trial.
To compare the efficacy of dexmedetomidine and midazolam in the prevention of postoperative nausea and vomiting (PONV) caused by hemabate in postpartum hemorrhage during cesarean delivery.

Title: Retained Placenta and Postpartum Hemorrhage: A Case Report and Review of Literature.
The third stage of labor (delivery of the placenta), per current definition, takes place within 30 minutes of fetal delivery in a nulliparous or multiparous woman. According to the American Pregnancy Association, a retained placenta is diagnosed if the placenta is not delivered within 30 minutes following delivery of the fetus. Retained placenta can be caused by placenta accreta, increta, or percreta. There are several complications of a retained placenta, including postpartum hemorrhage, which can lead to maternal death if not treated promptly. We report the case of a 32-year-old female, gravida 4 para 3, who was diagnosed with a... (truncated)

Title: Diagnosis and management of pre-eclampsia: an update.
Pre-eclampsia is a significant, multifactorial, multiorgan disease affecting 5%-8% of all pregnancies in the US where it is the third leading cause of maternal mortality. Despite improvements in the diagnosis and management of pre-eclampsia, severe complications can occur in both the mother and the fetus, and there is no effective method of prevention. Early detection and identification of pregnant women most at risk of developing the disease have proven challenging, but recent efforts combining biochemical and biophysical markers are promising. Efforts at prevention of pre-eclampsia with aspirin and calcium have had limited success, but research on modifiable risk factors, such... (truncated)

Title: Maternal health: a case study of Rajasthan.
This case study has used the results of a review of literature to understand the persistence of poor maternal health in Rajasthan, a large state of north India, and to make some conclusions on reasons for the same. The rate of reduction in Rajasthan's maternal mortality ratio (MMR) has been slow, and it has remained at 445 per 1000 livebirths in 2003. The government system provides the bulk of maternal health services. Although the service infrastructure has improved in stages, the availability of maternal health services in rural areas remains poor because of low availability of human resources, especially midwives... (truncated)

Title: The Advantage of Implementation of Enhanced Recovery After Surgery (ERAS) in Acute Pain Management During Elective Cesarean Delivery: A Prospective Randomized Controlled Trial.
The aim of this study was to test whether the implementation of an enhanced recovery after surgery (ERAS) protocol for patients undergoing elective cesarean delivery has a positive impact on the postoperative status of the patients in terms of pain management, hospital stay, hospitalization costs, and adverse reactions.

MESH:D002117 - calcitriol

Summary:

LLM Prediction Score	0.000 (normalized)
LLM Confidence Score	0.990
Golden Answer (Severity Class)	0.0 (normalized)
Prediction Error	0.000

Retrieved Context:

Title: Acute hepatologic and nephrologic effects of calcitriol in Syrian golden hamster (Mesocricetus auratus).
Although vitamin D is included in the group of fat-soluble vitamins, it must be considered as a prohormone. Its active forms, including calcitriol, have pleiotropic effects and play an important role in the regulation of cell proliferation, differentiation and apoptosis, as well as in hormone secretion, and they demonstrate anti-cancer properties. Since calcitriol delivery can be beneficial for the organism, and Syrian golden hamsters represent a unique experimental model, we decided to investigate its toxicity in this species. In this study, we injected calcitriol intraperitoneally at doses 0 (control), 0.180±0.009 µg/kg and 0.717±0.032 µg/kg. Animal behavior was observed for 72... (truncated)

Title: Calcitriol Protects against Acetaminophen-Induced Hepatotoxicity in Mice.
Acetaminophen (APAP) overdose is one of the major causes of acute liver failure. Severe liver inflammation and the production of oxidative stress occur due to toxic APAP metabolites and glutathione depletion. Growing evidence has proved that vitamin D (VD) exerts anti-inflammatory and antioxidative functions. Our objective was to explore the protective role of calcitriol (VD3) in acute APAP-induced liver injury. Methods: Adult male mice were randomized into three groups; control (n = 8), APAP (n = 8), and VD3 group (n = 8). All mice, except controls, received oral administration of APAP (400 mg/kg) and were sacrificed 24 h later.... (truncated)

Title: Calcipotriol attenuates liver fibrosis through the inhibition of vitamin D receptor-mediated NF-κB signaling pathway.
Liver fibrosis is an inevitable stage in the development of chronic liver disease to cirrhosis. Nonetheless, the interventional treatment and achieving control over the disease at this stage can substantially reduce the incidence of liver cirrhosis. To demonstrate these aspects, liver pathological sections of 18 patients with chronic liver disease are collected for research according to the degree of fibrosis. Further, the expressions of related proteins in each group are studied by the Western blot method. The cell proliferation and apoptosis are detected by CKK-8 and flow cytometry analyses. Further, a rat model with carbon tetrachloride (CCl₄)-induced liver fibrosis is... (truncated)

Title: The role of vitamin d in primary biliary cirrhosis: possible genetic and cell signaling mechanisms.
Primary biliary cirrhosis (PBC) is an immune-mediated chronic inflammatory disease of the liver of unknown etiology. Vitamin D deficiency is highly prevalent in patients with PBC, and many studies have demonstrated the significant effect of calcitriol on liver cell physiology. Vitamin D has antiproliferative and antifibrotic effects on liver fibrosis. Genetic studies have provided an opportunity to determine which proteins link vitamin D to PBC pathology (e.g., the major histocompatibility complex class II molecules, the vitamin D receptor, toll-like receptors, apolipoprotein E, Nramp1, and cytotoxic T lymphocyte antigen-4). Vitamin D also exerts its effect on PBC through cell signaling mechanisms,... (truncated)

Title: Protective Effect of Calcitriol on Organ Damage Induced by 5-Fluorouracil Treatment.
Chemotherapy is a major therapeutic strategy for patients with cancer. Owing to the severe inflammatory response of chemotherapy, patients experience extreme discomfort during treatment, and this may interrupt treatment completion. The vitamin D3 has a role in anti-inflammation, but no study has explored whether vitamin D3 has beneficial effects on patients undergoing chemotherapy. In this study, we investigated the effect of calcitriol (Vit-D) on inflammatory responses during 5-fluorouracil (5-FU) treatment. Rats were divided into five groups and treated with 1:1 dilution of 5-FU with equal amount of 0.9% saline, 1:3 dilution of 5-FU with 0.9% saline threefold dilution, 5-FU, Vit-D,... (truncated)

MESH:D017409 - sufentanil

Summary:

LLM Prediction Score	0.000 (normalized)
LLM Confidence Score	0.980
Golden Answer (Severity Class)	0.0 (normalized)
Prediction Error	0.000

Retrieved Context:

Title: Effects of dexmedetomidine on perioperative stress response, inflammation and immune function in patients with different degrees of liver cirrhosis.
Effects of dexmedetomidine (DEX) on perioperative stress response, inflammation and immune function in patients with different degrees of liver cirrhosis were investigated. A total of 94 patients with liver cirrhosis who were admitted to the Affiliated Hospital of Shandong University of Traditional Chinese Medicine from December 2016 to November 2017 were included, and randomly divided into control and observation group (n=47). Patients in control group were given remifentanil for anesthesia, while patients in observation group were treated with remifentanil and for DEX anesthesia. Venous blood was collected immediately before induction of anesthesia (T1), 10 min (T2) after the beginning of... (truncated)

Title: Sufentanil Preconditioning Protects Against Hepatic Ischemia-Reperfusion Injury by Suppressing Inflammation.
BACKGROUND Inflammation is one of the most significant mechanisms of hepatic ischemia-reperfusion injury (IRI). Sufentanil has a protective effect against liver injury by reducing inflammatory response. In this study, we used a cellular hepatic ischemic/reoxygenated (IR) model to determine whether sufentanil preconditioning protects against hepatic IRI. MATERIAL AND METHODS The human normal liver cells line L-O2 was studied. The levels of glutamic oxaloacetic transaminase (AST), lactate dehydrogenase (LDH), malonaldehyde (MDA), and superoxide dismutase (SOD) were measured using corresponding assay kits. The protein levels of total and phosphorylated ERK1/2, JNK, and p38, and the expression of p65 and COX2 genes, were... (truncated)

Title: Sufentanil postoperative analgesia reduce the increase of T helper 17 (Th17) cells and FoxP3⁺ regulatory T (Treg) cells in rat hepatocellular carcinoma surgical model: A randomised animal study.
Surgery-related pain and opioids might exacerbate immune defenses in immunocompromised cancer patients which might affect postoperativd overall survival. Sufentanil is a good postoperative pain control drug,the present study aimed to figure out whether it effect T cell immunity in rat hepatocellular carcinoma surgical model.

Title: Factors affecting sufentanil consumption for intravenous controlled analgesia after hepatectomy: retrospective analysis.
Pain control after hepatectomy is usually achieved by opioids. There are significant individual differences in the amount of opioids used after hepatectomy, and the metabolism of opioids is liver-dependent. The purpose of our study was to explore the possible risk factors for opioid consumption during the first 48 h after surgery.

Title: Analgesics in patients with hepatic impairment: pharmacology and clinical implications.
The physiological changes that accompany hepatic impairment alter drug disposition. Porto-systemic shunting might decrease the first-pass metabolism of a drug and lead to increased oral bioavailability of highly extracted drugs. Distribution can also be altered as a result of impaired production of drug-binding proteins or changes in body composition. Furthermore, the activity and capacity of hepatic drug metabolizing enzymes might be affected to various degrees in patients with chronic liver disease. These changes would result in increased concentrations and reduced plasma clearance of drugs, which is often difficult to predict. The pharmacology of analgesics is also altered in liver disease.... (truncated)

MESH:D014148 - tranexamic acid

Summary:

LLM Prediction Score	0.000 (normalized)
LLM Confidence Score	0.990
Golden Answer (Severity Class)	0.0 (normalized)
Prediction Error	0.000

Retrieved Context:

Title: TRANEXAMIC ACID ACTION ON LIVER REGENERATION AFTER PARTIAL HEPATECTOMY: EXPERIMENTAL MODEL IN RATS.
Different lesions may affect the liver resulting in harmful stimuli. Some therapeutic procedures to treat those injuries depend on liver regeneration to increase functional capacity of this organ.

Title: Major liver resection, systemic fibrinolytic activity, and the impact of tranexamic acid.
Hyperfibrinolysis may occur due to systemic inflammation or hepatic injury that occurs during liver resection. Tranexamic acid (TXA) is an antifibrinolytic agent that decreases bleeding in various settings, but has not been well studied in patients undergoing liver resection.

Title: The Role of Tranexamic Acid in the Prevention and Management of Blood Loss in Total Joint Arthroplasty.
Objective To collect data on the role played by tranexamic acid in the prevention and management of blood loss in patients undergoing total hip arthroplasty and total knee arthroplasty. Methods In the present prospective, comparative study, 30 patients undergoing total hip arthroplasty (THA) or total knee arthroplasty (TKA) were randomly allocated into 1 of 2 groups with 15 patients each. Tranexamic acid was administered by intravenous and topical routes in the study group, but it was not administered in the control group. Preoperative blood parameters, intraoperative and postoperative blood loss, and need for blood transfusion were noted. Statistical analysis was... (truncated)

Title: The antifibrinolytic drug tranexamic acid reduces liver injury and fibrosis in a mouse model of chronic bile duct injury.
Hepatic fibrin deposition has been shown to inhibit hepatocellular injury in mice exposed to the bile duct toxicant α-naphthylisothiocyanate (ANIT). Degradation of fibrin clots by fibrinolysis controls the duration and extent of tissue fibrin deposition. Thus, we sought to determine the effect of treatment with the antifibrinolytic drug tranexamic acid (TA) and plasminogen activator inhibitor-1 (PAI-1) deficiency on ANIT-induced liver injury and fibrosis in mice. Plasmin-dependent lysis of fibrin clots was impaired in plasma from mice treated with TA (1200 mg/kg i.p., administered twice daily). Prophylactic TA administration reduced hepatic inflammation and hepatocellular necrosis in mice fed a diet containing... (truncated)

Title: Tranexamic acid for acute gastrointestinal bleeding (the HALT-IT trial): statistical analysis plan for an international, randomised, double-blind, placebo-controlled trial.
Acute gastrointestinal (GI) bleeding is an important cause of mortality worldwide. Bleeding can occur from the upper or lower GI tract, with upper GI bleeding accounting for most cases. The main causes include peptic ulcer/erosive mucosal disease, oesophageal varices and malignancy. The case fatality rate is around 10% for upper GI bleeding and 3% for lower GI bleeding. Rebleeding affects 5-40% of patients and is associated with a four-fold increased risk of death. Tranexamic acid (TXA) decreases bleeding and the need for blood transfusion in surgery and reduces death due to bleeding in patients with trauma and postpartum haemorrhage. It... (truncated)

MESH:D018818 - fenoldopam

Summary:

LLM Prediction Score	0.000 (normalized)
LLM Confidence Score	0.970
Golden Answer (Severity Class)	0.0 (normalized)
Prediction Error	0.000

Retrieved Context:

Title: Future insights of pharmacological prevention for AKI post cardiopulmonary bypass surgery (based on PK/PD approach).
The incidence of acute kidney injury (AKI) post-cardiopulmonary bypass (CPB) can cause an increase in the rate of renal replacement therapy (RRT) and mortality rate. Compared to brain and liver damage post-CPB, AKI has the highest incidence of 83%. Based on this phenomenon, various efforts have been made to reduce the incidence of AKI post-CPB, both pharmacologically and non-pharmacologically interventions. The purpose of this review is to emphasize several renal protector agents which under optimal conditions can provide significant benefits in reducing the incidence of AKI post-CPB. This article was obtained by conducting a study on several kinds of literature,... (truncated)

Title: Exercise activates vagal induction of dopamine and attenuates systemic inflammation.
Physical exercise is one of the most important factors improving quality of life, but it is not feasible for patients with morbidity or limited mobility. Most previous studies focused on high-intensity or long-term exercise that causes metabolic stress or physiological adaption, respectively. Here, we studied how moderate-intensity swimming affects systemic inflammation in 6-8 week old C57BL/6J male mice during endotoxemia. One-hour swimming prevented hypokalemia, hypocalcemia, attenuated serum levels of inflammatory cytokines, increased anti-inflammatory cytokines but affected neither IL6 nor glycemia before or after the endotoxic challenge. Exercise attenuated serum TNF levels by inhibiting its production in the spleen through a... (truncated)

Title: Dopamine, Immunity, and Disease.
The neurotransmitter dopamine is a key factor in central nervous system (CNS) function, regulating many processes including reward, movement, and cognition. Dopamine also regulates critical functions in peripheral organs, such as blood pressure, renal activity, and intestinal motility. Beyond these functions, a growing body of evidence indicates that dopamine is an important immunoregulatory factor. Most types of immune cells express dopamine receptors and other dopaminergic proteins, and many immune cells take up, produce, store, and/or release dopamine, suggesting that dopaminergic immunomodulation is important for immune function. Targeting these pathways could be a promising avenue for the treatment of inflammation and... (truncated)

Title: Evaluation of von Willebrand factor and von Willebrand factor propeptide in models of vascular endothelial cell activation, perturbation, and/or injury.
Pharmacologically, vasoactive agents targeting endothelial and/or smooth muscle cells (SMC) are known to cause acute drug-induced vascular injury (DIVI) and the resulting pathology is due to endothelial cell (EC) perturbation, activation, and/or injury. Alteration in EC structure and/or function may be a critical event in vascular injury and, therefore, evaluation of the circulatory kinetic profile and secretory pattern of EC-specific proteins such as VWF and VWFpp could serve as acute vascular injury biomarkers. In rat and dog models of DIVI, this profile was determined using pharmacologically diverse agents associated with functional stimulation/perturbation (DDAVP), pathological activation (lipopolysaccharide [LPS]/endotoxin), and structural damage... (truncated)

Title: Anti-Inflammatory Effects of Peripheral Dopamine.
Dopamine is synthesized in the nervous system where it acts as a neurotransmitter. Dopamine is also synthesized in a number of peripheral organs as well as in several types of cells and has organ-specific functions and, as demonstrated more recently, is involved in the regulation of the immune response and inflammatory reaction. In particular, the renal dopaminergic system is very important in the regulation of sodium transport and blood pressure and is particularly sensitive to stimuli that cause oxidative stress and inflammation. This review is focused on how dopamine is synthesized in organs and tissues and the mechanisms by which... (truncated)

MESH:C120662 - calcium acetate

Summary:

LLM Prediction Score	0.000 (normalized)
LLM Confidence Score	0.990
Golden Answer (Severity Class)	0.0 (normalized)
Prediction Error	0.000

Retrieved Context:

Title: Diet-induced oxalate nephropathy.
Oxalate nephropathy is a rare condition and may be overlooked due to lack of recognition and understanding of triggers. An 81-year-old man was sent to nephrologist because of significantly increased creatinine (1.5-1.9 mg/dL) noted for 3 months. He had well-controlled diabetes but no history of kidney disease. He had no chronic diarrhoea or intestinal surgery. He was a health-minded individual who had read extensively about benefit of antioxidants. Initial work-up was unrevealing. Within a few weeks after first visit, he developed acute symptomatic worsening kidney injury with nausea, vomiting and creatinine up to 6.8 mg/dL. Repeat examination of the urine... (truncated)

Title: Computational Models Using Multiple Machine Learning Algorithms for Predicting Drug Hepatotoxicity with the DILIrank Dataset.
Drug-induced liver injury (DILI) remains one of the challenges in the safety profile of both authorized and candidate drugs, and predicting hepatotoxicity from the chemical structure of a substance remains a task worth pursuing. Such an approach is coherent with the current tendency for replacing non-clinical tests with in vitro or in silico alternatives. In 2016, a group of researchers from the FDA published an improved annotated list of drugs with respect to their DILI risk, constituting "the largest reference drug list ranked by the risk for developing drug-induced liver injury in humans" (DILIrank). This paper is one of the... (truncated)

Title: The Mutual Contribution of 3-NT, IL-18, Albumin, and Phosphate Foreshadows Death of Hemodialyzed Patients in a 2-Year Follow-Up.
Patients with chronic kidney disease (CKD), especially those who are hemodialyzed (HD), are at significantly high risk of contracting cardiovascular disease and having increased mortality. This study aimed to find potential death predictors, the measurement of which may reflect increased mortality in HD patients, and then combine the most promising ones in frames of a simple death risk assessment model. For this purpose, HD patients (n=71) with acute myocardial infarction in the last year (HD group) and healthy people (control group) as a comparative group (n=32) were included in the study. Various laboratory determinations and non-invasive cardiovascular tests were performed.... (truncated)

Title: Alox12/15 Deficiency Exacerbates, While Lipoxin A₄ Ameliorates Hepatic Inflammation in Murine Alcoholic Hepatitis.
Alcoholism is one of the leading and increasingly prevalent reasons of liver associated morbidity and mortality worldwide. Alcoholic hepatitis (AH) constitutes a severe disease with currently no satisfying treatment options. Lipoxin A₄ (LXA₄), a 15-lipoxygenase (ALOX15)-dependent lipid mediator involved in resolution of inflammation, showed promising pre-clinical results in the therapy of several inflammatory diseases. Since inflammation is a main driver of disease progression in alcoholic hepatitis, we investigated the impact of endogenous ALOX15-dependent lipid mediators and exogenously applied LXA₄ on AH development. A mouse model for alcoholic steatohepatitis (NIAAA model) was tested in Alox12/15^{+/+} and Alox12/15^{-/-} mice, with or without... (truncated)

Title: Clinical Evaluation of the Safety, Efficacy and Tolerability of Lanthanum Carbonate in the Management of Hyperphosphatemia in Patients with End-Stage Renal Disease.
Patients with progressive chronic kidney disease (CKD) commonly develop mineral and bone abnormalities and extraskeletal calcifications with following increased cardiovascular risk. A key pathophysiological role is played by hyperphosphatemia. Since diet and dialysis are often insufficient to control serum phosphorus levels, many patients require treatment with phosphate binders. Among them is lanthanum carbonate, an aluminum-free non-calcium-based compound. The present review summarizes the most recent literature data concerning the safety, efficacy and tolerability of lanthanum carbonate in patients with end-stage renal disease and hyperphosphatemia. The drug is taken orally as chewable tablets or powder with only minimal gastrointestinal absorption and resulting... (truncated)

MESH:D000077590 - mivacurium

Summary:

LLM Prediction Score	0.000 (normalized)
LLM Confidence Score	0.930
Golden Answer (Severity Class)	0.0 (normalized)
Prediction Error	0.000

Retrieved Context:

Title: Recent advances in neuromuscular blocking agents.
Factors driving the development of neuromuscular blocking agents are discussed. The goal of recent development of neuromuscular blocking agents is to develop agents with fewer adverse effects than succinylcholine and greater control. Greater control can be achieved through a short duration of action and a fast onset, similar to that found with succinylcholine. Duration control can be achieved through rapid, reliable metabolism that is organ independent, as with cisatracurium, or that occurs in the liver, because this will fail only in patients with severe hepatic disease. Rapid onset can be achieved by giving higher doses of drugs that have a... (truncated)

Title: [Neuromuscular and cardiovascular effect of mivacurium in anesthesia induction in patients with renal failure].
Mivacurium produces a prolonged neuromuscular block (NMB) in anuric patients (13), in spite of its rapid hydrolysis by pseudocholinesterase (PChE) which is independent of renal function (17). In the present study the pharmacodynamics and the cardiovascular effects of a bolus dose of mivacurium (0.15 mg/kg) in relation to impairment of renal function were evaluated.

Title: Management of patients with risk factors.
This review addresses concomitant diseases and risk factors in patients treated for diseases of the ears, nose and throat in outpatient and hospital services. Besides heart disease, lung disease, liver disease and kidney disease, this article also covers disorders of coagulation (including therapy with new oral anticoagulants) and electrolyte imbalance. Special attention is paid to the prophylaxis, diagnosis and treatment of perioperative delirium. It is also intended to help optimise the preparation for surgical procedures and pharmacotherapy during the hospital stay.

Title: Suspected anaphylactic reactions associated with anaesthesia.
No abstract available.

Title: Overview of extracellular microvesicles in drug metabolism.
Liver is the major body reservoir for enzymes involved in the metabolism of endogenous and xenobiotic compounds. Recently, it has been shown that hepatocytes release exosome-like vesicles to the extracellular medium, and the proteomic characterization of these hepatocyte-secreted exosomes has revealed the presence of several of these enzymes on them.

MESH:C067192 - ibutilide

Summary:

LLM Prediction Score	0.000 (normalized)
LLM Confidence Score	0.960
Golden Answer (Severity Class)	0.0 (normalized)
Prediction Error	0.000

Retrieved Context:

Title: Role of Pharmacogenetics in Adverse Drug Reactions: An Update towards Personalized Medicine.
Adverse drug reactions (ADRs) are an important and frequent cause of morbidity and mortality. ADR can be related to a variety of drugs, including anticonvulsants, anaesthetics, antibiotics, antiretroviral, anticancer, and antiarrhythmics, and can involve every organ or apparatus. The causes of ADRs are still poorly understood due to their clinical heterogeneity and complexity. In this scenario, genetic predisposition toward ADRs is an emerging issue, not only in anticancer chemotherapy, but also in many other fields of medicine, including hemolytic anemia due to glucose-6-phosphate dehydrogenase (G6PD) deficiency, aplastic anemia, porphyria, malignant hyperthermia, epidermal tissue necrosis (Lyell's Syndrome and Stevens-Johnson Syndrome), epilepsy,... (truncated)

Title: Suitability, efficacy, and safety of vernakalant for new onset atrial fibrillation in critically ill patients.
Objectives. This study investigates the suitability, safety, and efficacy of vernakalant in critically ill patients with new onset atrial fibrillation (AF) after cardiac surgery. Methods. Patients were screened for inclusion and exclusion criteria according to the manufacturers' recommendations. Included patients were treated with 3 mg/kg of vernakalant over 10 min and, if unsuccessful, a second dose of 2 mg/kg. Blood pressure was measured continuously for 2 hours after treatment. Results. Of the 191 patients screened, 159 (83%) were excluded, most importantly due to hemodynamic instability (59%). Vernakalant was administered to 32 (17% of the screened) patients. Within 6 hours, 17... (truncated)

Title: Guide to selection of fluoroquinolones in patients with lower respiratory tract infections.
Newer fluoroquinolones such as levofloxacin, moxifloxacin, gatifloxacin and gemifloxacin have several attributes that make them excellent choices for the therapy of lower respiratory tract infections. In particular, they have excellent intrinsic activity against Streptococcus pneumoniae, Haemophilus influenzae, Moraxella catarrhalis and the atypical respiratory pathogens. Fluoroquinolones may be used as monotherapy to treat high-risk patients with acute exacerbation of chronic bronchitis, and for patients with community-acquired pneumonia requiring hospitalisation, but not admission to intensive care. Overall, the newer fluoroquinolones often achieve clinical cure rates in > or =90% of these patients. However, rates may be lower in hospital-acquired pneumonia, and this... (truncated)

Title: Pharmacological management of atrial fibrillation: one, none, one hundred thousand.
atrial fibrillation (AF) is associated with a significant burden of morbidity and increased risk of mortality. Antiarrhythmic drug therapy remains a cornerstone to restore and maintain sinus rhythm for patients with paroxysmal and persistent AF based on current guidelines. However, conventional drugs have limited efficacy, present problematic risks of proarrhythmia and cause significant noncardiac organ toxicity. Thus, inadequacies in current therapies for atrial fibrillation have made new drug development crucial. New antiarrhythmic drugs and new anticoagulant agents have changed the current management of AF. This paper summarizes the available evidence regarding the efficacy of medications used for acute management of... (truncated)

Title: Amiodarone and thyroid physiology, pathophysiology, diagnosis and management.
Although amiodarone is considered the most effective antiarrhythmic agent, its use is limited by a wide variety of potential toxicities. The purpose of this review is to provide a comprehensive "bench to bedside" overview of the ways amiodarone influences thyroid function. We performed a systematic search of MEDLINE to identify peer-reviewed clinical trials, randomized controlled trials, meta-analyses, and other clinically relevant studies. The search was limited to English-language reports published between 1950 and 2017. Amiodarone was searched using the terms adverse effects, hypothyroidism, myxedema, hyperthyroidism, thyroid storm, atrial fibrillation, ventricular arrhythmia, and electrical storm. Google and Google scholar as well... (truncated)

MESH:D000077409 - tamsulosin

Summary:

LLM Prediction Score	0.000 (normalized)
LLM Confidence Score	0.980
Golden Answer (Severity Class)	0.0 (normalized)
Prediction Error	0.000

Retrieved Context:

Title: A Severe Case of Drug-Induced Liver Injury after Gemcitabine Administration: A Highly Probable Causality Grading as Assessed by the Updated RUCAM Diagnostic Scoring System.
Gemcitabine is an antineoplastic drug used in several forms of advanced pancreatic, lung, breast, ovarian, and bladder cancer. Common side effects include bone marrow suppression, fatigue, diarrhea, nausea, gastrointestinal upset, rash, alopecia, and stomatitis. Transient serum enzyme elevations could be observed during therapy, but clinically significant acute liver injury has been rarely associated with its use. Few cases of acute liver injury have been reported in the literature. We reported the clinical case of a 73--year-old man who developed clinically significant acute hepatic injury after using gemcitabine. Possible causes, clinical presentation, and treatments are discussed. According to the updated RUCAM... (truncated)

Title: Tamsulosin Associated with Interstitial Lung Damage in CYP2D6 Variant Alleles Carriers.
Drugs are serious but underestimated causative agents of interstitial lung disease (ILD). Both cytotoxic and immune mechanisms may be involved in drug-induced ILD (DI-ILD). We aimed to investigate whether polymorphisms of relevant CYP enzymes involved in the metabolization of tamsulosin might explain the pathologic mechanism of the DI-ILD in the cases with suspected tamsulosin DI-ILD. We collected 22 tamsulosin-associated DI-ILD cases at two ILD Expertise Centers in the Netherlands between 2009 and 2020. CYP2D6, CYP2C9, CYP2C19, CYP3A4, and CYP3A5 single nucleotide polymorphisms were genotyped and compared with a control group of 78 healthy Caucasian male volunteers. Nine cases were phenotyped... (truncated)

Title: Acute hepatocellular drug induced liver injury probably by alfuzosin.
Alpha blockers are the drugs that exert their effects by binding to alpha receptors and relaxing smooth muscles and are currently used for treatment of benign prostate hyperplasia (BPH). These drugs are often tolerated well by the patients. However, they also possess some common side effects. Hepatotoxicity, on the other hand, is quite rare. We report herein a case with the rare complication of acute hepatocellular drug induced liver injury (DILI) by administration of Alfuzosin.

Title: Levomilnacipran for the treatment of major depressive disorder: a review.
Levomilnacipran (LVM, Fetzima(®)) was recently approved by the US Food and Drug Administration for the treatment of major depressive disorder. It is a unique dual neurotransmitter reuptake inhibitor. In contrast with other selective serotonin norepinephrine reuptake inhibitors, including duloxetine, venlafaxine, and desvenlafaxine, it has greater selectivity for inhibiting norepinephrine reuptake than serotonin reuptake. Our review focuses on the efficacy, safety, and tolerability data for five double-blind, placebo-controlled, short-term studies and two long-term studies. In the short-term studies, LVM was found to be more effective than placebo in reducing depression (Montgomery-Åsberg Depression Rating Scale) scores as well as improving functional impairment... (truncated)

Title: The Role of α1-Adrenoceptor Antagonists in the Treatment of Prostate and Other Cancers.
This review evaluates the role of α-adrenoceptor antagonists as a potential treatment of prostate cancer (PCa). Cochrane, Google Scholar and Pubmed were accessed to retrieve sixty-two articles for analysis. In vitro studies demonstrate that doxazosin, prazosin and terazosin (quinazoline α-antagonists) induce apoptosis, decrease cell growth, and proliferation in PC-3, LNCaP and DU-145 cell lines. Similarly, the piperazine based naftopidil induced cell cycle arrest and death in LNCaP-E9 cell lines. In contrast, sulphonamide based tamsulosin did not exhibit these effects. In vivo data was consistent with in vitro findings as the quinazoline based α-antagonists prevented angiogenesis and decreased tumour mass in... (truncated)

MESH:C058254 - sibutramine

Summary:

LLM Prediction Score	0.500 (normalized)
LLM Confidence Score	0.980
Golden Answer (Severity Class)	0.5 (normalized)
Prediction Error	0.000

Retrieved Context:

Title: A Systematic Review of the European Rapid Alert System for Food and Feed: Tendencies in Illegal Food Supplements for Weight Loss.
Background: Slimming products represent a dynamically growing group of food supplements worldwide. The efficacy of safely usable natural ingredients is usually below consumers' expectations. Certain manufacturers add unauthorized or prohibited ingredients to weight loss supplements in order to increase their efficacy. Hence, many of these products are adulterated and may pose a risk to the consumers' health. Aims: The aim of our work was to give an overview on natural ingredients used in slimming products, to summarize the frequently used synthetic adulterants and also to assess the trends of adulterated and illegal food supplements in the European Union based on... (truncated)

Title: Evolution of the Food and Drug Administration approach to liver safety assessment for new drugs: current status and challenges.
Prompted by approval in 1997 of troglitazone and bromfenac, two drugs that promptly began to show serious and sometimes fatal liver toxicity, we began at the Food and Drug Administration (FDA) a series of annual conferences in 1999 to consider issues of drug-induced liver injury (DILI). First inviting reviewers of new drug applications we opened the audiences in 2001 to pharmaceutical industry and academic consultants to industry and FDA, and slides shown at the meetings were posted on the internet to be available at the website of the American Association for the Study of Liver Diseases (AASLD)-go to (http://www.aasld.org/dili/Pages/default.aspx... (truncated)

Title: Adulteration of Weight Loss Supplements by the Illegal Addition of Synthetic Pharmaceuticals.
Weight loss supplements that have illegal additives of pharmaceutical drugs or analogues have additional health risks, and customers may not be aware of what they are taking. This research is an essential investigation and quantification of illegally added pharmaceuticals or prescription medications, specifically fluoxetine, phenolphthalein, and sibutramine, in herbal weight loss supplements offered for sale in the United Arab Emirates (UAE). In this case, 137 weight loss supplements were collected and analyzed in this study. Reversed-phase high-performance liquid chromatography with UV absorption detection coupled to tandem mass spectrometry (RP-HPLC-MS/MS) analyses were used to determine the presence of the pharmaceutical chemicals.... (truncated)

Title: Sympathomimetic amine compounds and hepatotoxicity: Not all are alike-Key distinctions noted in a short review.
Sympathomimetic amine compounds are often pooled together and incorrectly assumed to be interchangeable with respect to potential adverse effects. A brief and specific review of sympathomimetic compounds and one instance (i.e., hepatotoxicity) where these compounds have been improperly grouped together is covered. A review of the proposed mechanisms through which known hepatotoxic sympathomimetic agents (e.g., 3,4-methylenedioxymethamphetamine or MDMA, methamphetamine and amphetamine) cause liver injury, along with a corresponding review of in vitro data, interventional data, animal model studies and observational data allow for a comparison/contrast of different agents and reveals a lack of potential toxicity for some agents (e.g., pseudoephedrine,... (truncated)

Title: Behavioral and pharmacologic therapies for obesity.
This article reviews novel developments in the behavioral and pharmacologic treatment of obesity and explores the potential contribution of genomics research to weight control. A comprehensive program of lifestyle modification, comprised of diet, physical activity and behavior therapy, induces a mean loss of 7-10% of initial weight in individuals with obesity. Two trials demonstrated that weight loss of this magnitude, combined with increased physical activity, substantially reduced the risk of developing type 2 diabetes mellitus in individuals with impaired glucose tolerance. A third trial is now investigating whether lifestyle intervention will reduce cardiovascular morbidity and mortality in overweight individuals who... (truncated)

MESH:C045621 - miglitol

Summary:

LLM Prediction Score	0.000 (normalized)
LLM Confidence Score	0.960
Golden Answer (Severity Class)	0.0 (normalized)
Prediction Error	0.000

Retrieved Context:

Title: A review of alpha-glucosidase inhibitors from plants as potential candidates for the treatment of type-2 diabetes. Diabetes mellitus is a multifactorial global health disorder that is rising at an alarming rate. Cardiovascular diseases, kidney damage and neuropathy are the main cause of high mortality rates among individuals with diabetes. One effective therapeutic approach for controlling hyperglycemia associated with type-2 diabetes is to target alpha-amylase and alpha-glucosidase, enzymes that catalyzes starch hydrolysis in the intestine. At present, approved inhibitors for these enzymes are restricted to acarbose, miglitol and voglibose. Although these inhibitors retard glucose absorption, undesirable gastrointestinal side effects impede their application. Therefore, research efforts continue to seek novel inhibitors with improved efficacy and minimal side effects.... (truncated)

Title: Miglitol improves postprandial endothelial dysfunction in patients with acute coronary syndrome and new-onset postprandial hyperglycemia.
Hyperglycemia, a risk factor for development of cardiovascular disease, causes endothelial dysfunction.
Alpha-glucosidase inhibitors (α-GIs) improve postprandial hyperglycemia (PPHG) and may have favorable effects on associated cardiovascular disease. Effects of α-GIs in patients with acute coronary syndrome (ACS) and PPHG remain unclear; thus, we assessed the effect of α-GI miglitol on endothelial function in such patients by digital reactive hyperemia peripheral arterial tonometry (RH-PAT).

Title: Pharmacological Efficacy of <i>Tamarix aphylla</i>: A Comprehensive Review.
<i>Tamarix aphylla</i> is a well-known species of the genus <i>Tamarix</i>. <i>T. aphylla</i> (Tamaricaceae) is a perennial tree in Asia, the Middle East, and Central Africa. It is used as a carminative diuretic in tuberculosis, leprosy, and hepatitis. Various pharmacological properties have been shown by <i>T. aphylla,</i> such as antidiabetic, anti-inflammatory, antibacterial, antifungal, anticholinesterase, and wound-healing activity. However, <i>T. aphylla</i> has not received much attention for its secondary metabolites and bioactive constituents. Research has shown that this plant has hidden potential that needs to be explored. This review aims to cover botanical classification, geographical distribution, taxonomy, ethnobotanical uses, and the phytochemical... (truncated)

Title: α-Glucosidase inhibitors and their use in clinical practice.
Post-prandial hyperglycemia still remains a problem in the management of type 2 diabetes mellitus. Of all available anti-diabetic drugs, α-glucosidase inhibitors seem to be the most effective in reducing post-prandial hyperglycemia. We conducted a review analyzing the clinical efficacy and safety of α-glucosidase inhibitors, both alone and in combination with other anti-diabetic drugs, with respect to glycemic control, inflammation and atherosclerosis. α-Glucosidase inhibitors proved to be effective and safe both in monotherapy and as an add-on to other anti-diabetic drugs. Compared to miglitol and voglibose, acarbose seems to have some additive effects such as stabling carotid plaques, and reducing inflammation.... (truncated)

Title: Consensus Statement on Dose Modifications of Antidiabetic Agents in Patients with Hepatic Impairment.
Liver disease is an important cause of mortality in type 2 diabetes mellitus (T2DM). It is estimated that diabetes is the most common cause of liver disease in the United States. Virtually, entire spectrum of liver disease is seen in T2DM including abnormal liver enzymes, nonalcoholic fatty liver disease, cirrhosis, hepatocellular carcinoma, and acute liver failure. The treatment of diabetes mellitus (DM) in cirrhotic patients has particular challenges as follows: (1) about half the patients have malnutrition; (2) patients already have advanced liver disease when clinical DM is diagnosed; (3) most of the oral antidiabetic agents (ADAs) are metabolized in... (truncated)

MESH:C000595212 - polyethylene glycol 3350

Summary:

LLM Prediction Score	0.000 (normalized)
LLM Confidence Score	0.970
Golden Answer (Severity Class)	0.0 (normalized)
Prediction Error	0.000

Retrieved Context:

Title: Polyethylene Glycol 3350 Crystal Nephropathy in Association With Glomerular Mesangial Immunoglobin A Deposition.
Polyethylene glycol (PEG) 3350, an active ingredient of over-the-counter MiraLAX, is a commonly used laxative in children and is produced by polymerization of ethylene glycol (EG). Masked EG toxicity secondary to contamination of PEG 3350 could occur. We present a 7-year-old child with developmental delay who presented with altered mental status and acute kidney injury (AKI) following intake of generic PEG 3350 for few days prior to presentation. There was high anion gap metabolic acidosis, hypernatremia, elevated osmolar gap, lactic acidosis, and AKI. Urinalysis showed tubular proteinuria, microscopic hematuria, and calcium oxalate crystals. Prior urinalyses were normal without hematuria or... (truncated)

Title: Antifibrotic preventive effect of polyethylene glycol (PEG) 3350 in methotrexateinduced hepatoxicity model.
Liver damage caused by drugs and other chemicals accounts for about 5% of all cases. Methotrexate (MTX), a folic acid analogue, is a first-line synthetic antimetabolite agent routinely used in the treatment of rheumatoid arthritis and other autoimmune and chronic inflammatory diseases. Polyethylene glycol (PEG) has antioxidant activity. In this study, we evaluated biochemically and histopathologically the antifibrotic effect of PEG 3350 administered intraperitoneally to prevent methotrexate-induced liver damage in rats.

Title: Lactulose vs polyethylene glycol 3350--electrolyte solution for treatment of overt hepatic encephalopathy: the HELP randomized clinical trial.
Hepatic encephalopathy (HE) is a common cause of hospitalization in patients with cirrhosis. Pharmacologic treatment for acute (overt) HE has remained the same for decades.

Title: Crystallographic analysis of human serum albumin complexed with 4Z,15E-bilirubin-IXalpha.
Bilirubin, an insoluble yellow-orange pigment derived from heme catabolism, accumulates to toxic levels in individuals with impaired or immature liver function. The resulting jaundice may be managed with phototherapy to isomerize the biosynthetic 4Z,15Z-bilirubin-IXalpha to more soluble and excretable isomers, such as 4Z,15E-bilirubin. Bilirubin and its configurational isomers are transported to the liver by human serum albumin (HSA) but their precise binding location(s) on the protein have yet to be determined. To investigate the molecular details of their interaction, we co-crystallised bilirubin with HSA. Strikingly, the crystal structure--determined to 2.42 A resolution--revealed the 4Z,15E-bilirubin-IXalpha isomer bound to an L-shaped pocket... (truncated)

Title: Polyethylene Glycol Exposure with Antihemophilic Factor (Recombinant), PEGylated (rurioctocog alfa pegol) and Other Therapies Indicated for the Pediatric Population: History and Safety.
Polyethylene glycol (PEG) is an inert, water soluble polymer, used for decades in pharmaceuticals. Although PEG is considered safe, concerns persist about the potential adverse effects of long-term exposure to PEG-containing therapies, specifically in children, following the introduction of PEGylated recombinant factor products used for the treatment of hemophilia. Given the absence of long-term surveillance data, and to evaluate the potential risk, we estimated PEG exposure in the pediatric population receiving PEGylated therapies with pediatric indications administered intravenously or intramuscularly. We used a range of pediatric weights and doses based on prescribing information (PI) or treatment guidelines. PIs and reporting... (truncated)

MESH:C074619 - bivalirudin

Summary:

LLM Prediction Score	0.000 (normalized)
LLM Confidence Score	0.960
Golden Answer (Severity Class)	0.0 (normalized)
Prediction Error	0.000

Retrieved Context:

Title: [Direct inhibitors of thrombin, hirudin, bivalirudin, and dabigatran etexilate].
Thrombin inhibition is an important objective in the prevention and treatment of thrombosis. A new molecule, dabigatran etexilate or Pradaxa(®), has been recently licensed for thromboprophylaxis in major orthopedic surgery in several countries but not in the USA. In contrast, the FDA has approved it for prevention in patients with non-valvular atrial fibrillation. This new orally active anticoagulant is being developed for the treatment of venous thromboembolism and acute coronary syndromes in patients with non-valvular atrial fibrillation. Dabigatran is a reversible inhibitor of free thrombin and clot-bound thrombin. An oral thrombin inhibitor melagatran is no longer available due to hepatic... (truncated)

Title: Bivalirudin in combination with heparin to control mesenchymal cell procoagulant activity.
Islet and hepatocyte transplantation are associated with tissue factor-dependent activation of coagulation which elicits instant blood mediated inflammatory reaction, thereby contributing to a low rate of engraftment. The aim of this study was i) to evaluate the procoagulant activity of human adult liver-derived mesenchymal progenitor cells (hALPCs), ii) to compare it to other mesenchymal cells of extra-hepatic (bone marrow mesenchymal stem cells and skin fibroblasts) or liver origin (liver myofibroblasts), and iii) to determine the ways this activity could be modulated. Using a whole blood coagulation test (thromboelastometry), we demonstrated that all analyzed cell types exhibit procoagulant activity. The hALPCs... (truncated)

Title: Selection of an aptamer antidote to the anticoagulant drug bivalirudin.
Adverse drug reactions, including severe patient bleeding, may occur following the administration of anticoagulant drugs. Bivalirudin is a synthetic anticoagulant drug sometimes employed as a substitute for heparin, a commonly used anticoagulant that can cause a condition called heparin-induced thrombocytopenia (HIT). Although bivalrudin has the advantage of not causing HIT, a major concern is lack of an antidote for this drug. In contrast, medical professionals can quickly reverse the effects of heparin using protamine. This report details the selection of an aptamer to bivalirudin that functions as an antidote in buffer. This was accomplished by immobilizing the drug on a... (truncated)

Title: Prolonged infusion of bivalirudin after elective percutaneous coronary intervention protects against procedural myocardial injury (a COBER study)-a randomized trial.
Procedural myocardial injury (PMI), which is the most common complication of elective percutaneous coronary intervention (ePCI), is associated with future adverse cardiac events. In this randomized pilot trial, we assessed the effects of prolonged use of the anti-coagulant bivalirudin on PMI after ePCI. Patients undergoing ePCI were randomized into the following two groups: the bivalirudin use during operation group (BUDO, 0.75 mg/kg bolus plus 1.75 mg/kg/h) and the bivalirudin use during and after operation for 4 h (BUDAO, 0.75 mg/kg bolus plus 1.75 mg/kg/h). Blood samples were collected before and 24 h after ePCI (per 8 h). The primary outcome,... (truncated)

Title: Safety profile of bivalirudin in Chinese female patients undergoing percutaneous coronary intervention: a multi-center study.
The present study aimed to comprehensively investigate the occurrence and risk factors of adverse events (AEs) or adverse drug reactions (ADRs) (especially for thrombocytopenia and bleeding) in Chinese female patients receiving bivalirudin during percutaneous coronary intervention (PCI).

MESH:D014295 - trimethoprim

Summary:

LLM Prediction Score	0.500 (normalized)
LLM Confidence Score	0.990
Golden Answer (Severity Class)	0.5 (normalized)
Prediction Error	0.000

Retrieved Context:

Title: Sulfamethoxazole/Trimethoprim induced liver failure: a case report.
Sulfamethoxazole/Trimethoprim (SMX/TMP) is a commonly used antibiotic, its' known adverse reaction of hepatotoxicity leading to acute liver failure is considered rare. We present a case of a 22 year old female who developed jaundice and acute liver failure one week after taking SMX/TMP for a UTI. After an extensive work up, a clinical diagnosis of SMX/TMP induced liver failure was reached. Over the course of several weeks she made a good clinical and biochemical recovery with supportive care. In this case report we describe her clinical presentation and course, and present a brief review of the literature.

Title: [Trimethoprim-sulfamethoxazole-induced cholestatic hepatitis. Clinico-immunological demonstration of its allergic origin].
A 22-year-old woman was given trimethoprim plus sulphamethoxazole for a urinary infection (160 and 800 mg, respectively, daily), drugs she had not previously taken. After 2 weeks she noticed a rash of small spots on her trunk. In addition she had nausea and vomiting. The rash faded within 2 days of stopping the drug, but progressive jaundice developed.

Title: Metabolism of trimethoprim to a reactive iminoquinone methide by activated human neutrophils and hepatic microsomes.
The antibacterial agent, trimethoprim, is normally used synergistically with sulfonamides. Its use is associated with idiosyncratic reactions including liver toxicity and agranulocytosis. In this study, we demonstrated that trimethoprim was oxidized by activated human neutrophils, as well as a combination of myeloperoxidase/hydrogen peroxide/chloride or hypochlorous acid, to a reactive pyrimidine iminoquinone methide intermediate with a protonated molecular ion of m/z 289 as detected by mass spectrometry. In the presence of N-acetyl-L-cysteine (NAC), the pyrimidine iminoquinone methide could be trapped as three NAC adducts. The three NAC adducts were separable on HPLC, but showed the same protonated molecular ion of m/z... (truncated)

Title: Adverse reactions to trimethoprim-sulfamethoxazole.
A comprehensive review is presented of the world literature on adverse reactions to trimethoprim-sulfamethoxazole (TMP-SMZ) since its first use in 1968, when concern was expressed about possible hematologic toxicity. Serious toxicity is a rare event, but when it occurs severe skin lesions and thrombocytopenia/leukopenia are the most likely complications. Like other antibacterial agents, TMP-SMZ is associated with gastrointestinal upset, but adverse reactions affecting the kidney, liver, and fetus are rare. If the use of TMP-SMZ in pregnant women and in those known to be sensitive to the drug is avoided and if there is an awareness of possible drug interactions,... (truncated)

Title: A large population-based follow-up study of trimethoprim-sulfamethoxazole, trimethoprim, and cephalexin for uncommon serious drug toxicity.
We conducted a population-based 45-day follow-up study of 232,390 people who were prescribed trimethoprim-sulfamethoxazole (TMP-SMZ), 266,951 prescribed trimethoprim alone, and 196,397 prescribed cephalexin, to estimate the risk of serious liver, blood, skin, and renal disorders resulting in referral or hospitalization associated with these drugs. The results were based on information recorded on office computers by selected general practitioners in the United Kingdom, together with a review of clinical records. The risk of clinically important idiopathic liver disease was similar for persons prescribed TMP-SMZ (5.2/100,000) and those prescribed trimethoprim alone (3.8/100,000). The risk for those prescribed cephalexin was somewhat lower (2.0/100,000).... (truncated)

MESH:D001285 - atropine

Summary:

LLM Prediction Score	0.000 (normalized)
LLM Confidence Score	0.990
Golden Answer (Severity Class)	0.0 (normalized)
Prediction Error	0.000

Retrieved Context:

Title: Investigation into the role of the cholinergic system in radiation-induced damage in the rat liver and ileum.
It has been previously shown that acetylcholine (ACh) may affect pro-inflammatory and anti-inflammatory cytokines. The role of the cholinergic system in radiation-induced inflammatory responses and tissue damage remains unclear. Therefore, the present study was designed to determine the radio-protective properties of the cholinergic system in the ileum and the liver of rats. Rats were exposed to 8-Gy single-fraction whole-abdominal irradiation and were then decapitated at either 36 h or 10 d post-irradiation. The rats were treated either with intraperitoneal physiological saline (1 ml/kg), physostigmine (80 µg/kg) or atropine (50 µg/kg) twice daily for 36 h or 10 d. Cardiac blood... (truncated)

Title: Pesticide poisoning.
Acute poisoning with pesticides is a global public health problem and accounts for as many as 300,000 deaths worldwide every year. The majority of deaths occur due to exposure to organophosphates, organochlorines and aluminium phosphide. Organophosphate compounds inhibit acetylcholinesterase resulting in acute toxicity. Intermediate syndrome can develop in a number of patients and may lead to respiratory paralysis and death. Management consists of proper oxygenation, atropine in escalating doses and pralidoxime in high doses. It is Important to decontaminate the skin while taking precautions to avoid secondary contamination of health personnel. Organochlorine pesticides are toxic to the central nervous system... (truncated)

Title: Effects of dexmedetomidine on perioperative stress response, inflammation and immune function in patients with different degrees of liver cirrhosis.
Effects of dexmedetomidine (DEX) on perioperative stress response, inflammation and immune function in patients with different degrees of liver cirrhosis were investigated. A total of 94 patients with liver cirrhosis who were admitted to the Affiliated Hospital of Shandong University of Traditional Chinese Medicine from December 2016 to November 2017 were included, and randomly divided into control and observation group (n=47). Patients in control group were given remifentanil for anesthesia, while patients in observation group were treated with remifentanil and for DEX anesthesia. Venous blood was collected immediately before induction of anesthesia (T1), 10 min (T2) after the beginning of... (truncated)

Title: Atropa belladonna intoxication: a case report.
Atropa belladonna is a poisonous plant also called deadly nightshade. Its roots, leaves and fruits contain alkaloids: atropine, hyocyamine and scopolamine. The risk of poisoning in children is important because of possible confusion with other berries. Atropa belladonna acute intoxication is a severe condition, it's should be considered in the presence of anti-cholinergic toxidrome, the differential diagnosis include other plants or psychoactive drugs containing atropine. The treatment is mainly symptomatic including gastrointestinal decontamination with activated charcoal. In severe cases, physostigmine can be used as an antidote. We report the case of 11 year old girl with Atropa belladonna poisoning which... (truncated)

Title: Successful management of a kitten with chlorpyrifos and cypermethrin toxicosis with pralidoxime and atropine.
Organophosphates and pyrethroids have been widely used as agricultural and domestic insecticides. This case report describes a 3-month-old free-roaming female kitten, weighing 930 g, that developed hypersalivation, hypothermia, dyspnoea due to increased bronchial secretion, bradycardia, miosis and neurological signs, including restlessness, ataxia, disorientation, apparent hallucination, muscle twitching and seizures within 6 h of accidental ingestion of an insecticide containing chlorpyrifos (500 g/l) and cypermethrin (50 g/l). The kitten was treated empirically with intramuscular atropine and dexamethasone, and rectal diazepam. The history of insecticide exposure was obtained after 6 h of treatment and intramuscular 2-pyridine aldoxime methochloride (pralidoxime [2-PAM]) and atropine... (truncated)
