# Supplementary figures and images for: Systematic analysis of hepatotoxicity: combining literature mining and AI language models
Source: Front Artif Intell. 2025 Jul 21;8:1561292. doi: 10.3389/frai.2025.1561292 (PMC12338115; doi:10.3389/frai.2025.1561292)

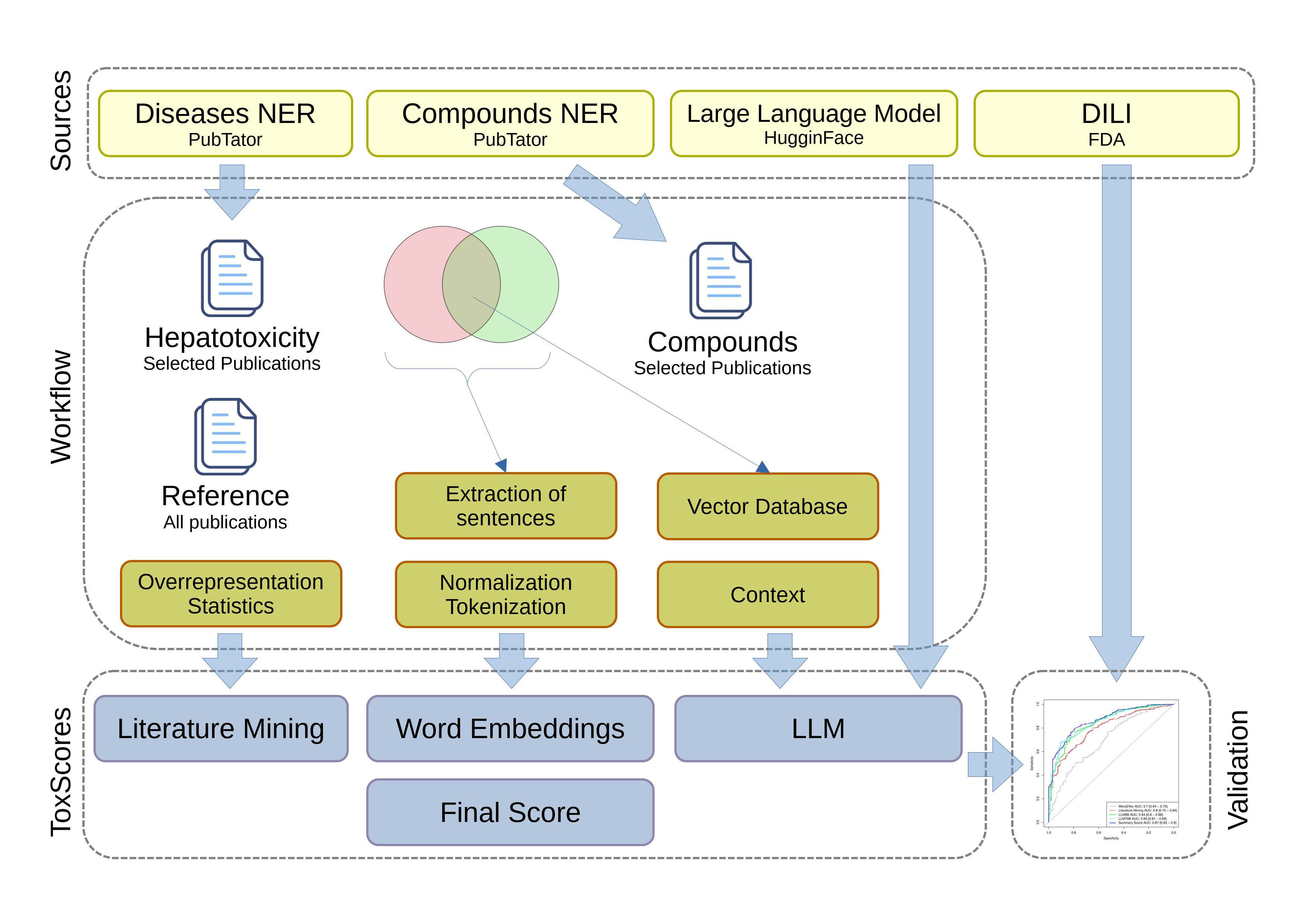

Supplement: Supplementary file 3 [file Image_1.tif]
